# The Cellular Transcriptome in the Maternal Circulation During Normal Pregnancy: A Longitudinal Study

**DOI:** 10.3389/fimmu.2019.02863

**Published:** 2019-12-17

**Authors:** Nardhy Gomez-Lopez, Roberto Romero, Sonia S. Hassan, Gaurav Bhatti, Stanley M. Berry, Juan Pedro Kusanovic, Percy Pacora, Adi L. Tarca

**Affiliations:** ^1^Perinatology Research Branch, Division of Obstetrics and Maternal-Fetal Medicine, Division of Intramural Research, Eunice Kennedy Shriver National Institute of Child Health and Human Development, National Institutes of Health, U.S. Department of Health and Human Services, Bethesda, MD and Detroit, MI, United States; ^2^Department of Obstetrics and Gynecology, Wayne State University School of Medicine, Detroit, MI, United States; ^3^Department of Biochemistry, Microbiology and Immunology, Wayne State University School of Medicine, Detroit, MI, United States; ^4^Department of Obstetrics and Gynecology, University of Michigan, Ann Arbor, MI, United States; ^5^Department of Epidemiology and Biostatistics, Michigan State University, East Lansing, MI, United States; ^6^Center for Molecular Medicine and Genetics, Wayne State University, Detroit, MI, United States; ^7^Detroit Medical Center, Detroit, MI, United States; ^8^Department of Obstetrics & Gynecology, Florida International University, Miami, FL, United States; ^9^Department of Physiology, Wayne State University School of Medicine, Detroit, MI, United States; ^10^Division of Obstetrics and Gynecology, Faculty of Medicine, Pontificia Universidad Católica de Chile, Santiago, Chile; ^11^Center for Research and Innovation in Maternal-Fetal Medicine (CIMAF), Department of Obstetrics and Gynecology, Sótero del Río Hospital, Santiago, Chile; ^12^Department of Computer Science, Wayne State University College of Engineering, Detroit, MI, United States

**Keywords:** B cells, biomarker, cytokines, erythroid cells, immunity, pregnancy, T cells

## Abstract

Pregnancy represents a unique immunological state in which the mother adapts to tolerate the semi-allogenic conceptus; yet, the cellular dynamics in the maternal circulation are poorly understood. Using exon-level expression profiling of up to six longitudinal whole blood samples from 49 pregnant women, we undertook a systems biology analysis of the cellular transcriptome dynamics and its correlation with the plasma proteome. We found that: (1) chromosome 14 was the most enriched in transcripts differentially expressed throughout normal pregnancy; (2) the strongest expression changes followed three distinct longitudinal patterns, with genes related to host immune response (e.g., *MMP8, DEFA1B, DEFA4*, and *LTF*) showing a steady increase in expression from 10 to 40 weeks of gestation; (3) multiple biological processes and pathways related to immunity and inflammation were modulated during gestation; (4) genes changing with gestation were among those specific to T cells, B cells, CD71+ erythroid cells, natural killer cells, and endothelial cells, as defined based on the GNF Gene Expression Atlas; (5) the average expression of mRNA signatures of T cells, B cells, and erythroid cells followed unique patterns during gestation; (6) the correlation between mRNA and protein abundance was higher for mRNAs that were differentially expressed throughout gestation than for those that were not, and significant mRNA-protein correlations were observed for genes part of the T-cell signature. In summary, unique changes in immune-related genes were discovered by longitudinally assessing the cellular transcriptome in the maternal circulation throughout normal pregnancy, and positive correlations were noted between the cellular transcriptome and plasma proteome for specific genes/proteins. These findings provide insights into the immunobiology of normal pregnancy.

## Introduction

Pregnancy represents a unique immunological state in which the immune system of the mother undergoes adaptations that allow her to tolerate the semi-allogenic conceptus (1–3). Indeed, pregnancy is divided into three different immunological stages based on cytokine profiles (4). Pioneer studies indicated that, while the innate immune system is upregulated to protect the mother against infection and the fetus from rejection (5, 6), the adaptive immune response toward paternal/fetal antigens seems to be selectively suppressed [i.e., driven toward a T-helper (Th)2-like phenotype] (7–11). Specifically, the cellular components of the innate immune system in the maternal systemic circulation are activated as evidenced by increased numbers of monocytes and granulocytes (12, 13). Such innate immune cells display an activated phenotype, comparable to that observed in women with sepsis (13), and exhibit enhanced functionality (phagocytosis, respiratory burst activity, and cytokine production) (14–18). The humoral components of the innate immune system are also boosted during pregnancy (5). For example, complement components and acute phase proteins are increased in the circulation of pregnant women (19–24). In contrast to the innate immune system, the cellular (e.g., T cells and B cells) and humoral (e.g., antibodies) components of the adaptive immune system in the maternal circulation during normal pregnancy have received less attention.

The systemic intravascular inflammatory response during normal pregnancy is especially activated in women who experience the physiological process of labor at term (18, 25) and in those who undergo pregnancy complications such as preterm labor (18, 26, 27), preterm premature rupture of membranes (28), and preeclampsia (13, 17, 29–32). Therefore, the systemic immune response reflects both physiological and pathological processes, and the early detection of these changes may lead to the discovery of non-invasive biomarkers for obstetrical disease.

Herein, for the first time, we aimed to provide a roadmap of the modulations in the cellular transcriptome in maternal circulation during normal pregnancy. In addition, we assessed whether gestational mRNA changes of the cellular transcriptome correlate to those of the plasma proteome during normal pregnancy.

## Materials and Methods

### Study Design

We conducted a prospective longitudinal study that enrolled women attending the Center for Advanced Obstetrical Care and Research of the Perinatology Research Branch, NICHD/NIH/DHHS; the Detroit Medical Center, and Wayne State University School of Medicine. Based on this cohort, we designed a retrospective study that included 49 women with normal pregnancy who delivered at term and had 4–6 blood samples collected throughout gestation [median number of samples = 5, interquartile range (IQR) = 5–6] (*n* = 282). Blood samples were collected at the time of a prenatal visit, scheduled at 4-week intervals from the first or early second trimester until delivery in the following gestational age intervals: 8- <16, 16- <24, 24- <28, 28- <32, 32- <37, and >37 weeks. All patients provided written informed consent and the use of biological specimens, as well as clinical and ultrasound data, for research purposes were approved by the Institutional Review Boards of Wayne State University and NICHD. All experiments were performed in accordance with relevant guidelines and regulations.

### RNA Extraction

RNA was isolated from PAXgene® Blood RNA collection tubes (BD Biosciences, San Jose, CA; Catalog #762165), as described in the PAXgene® Blood miRNA Kit Handbook. Purified RNA was quantified by UV spectrophotometry using the DropSense96® Microplate Spectrophotometer (Trinean, Gentbrugge, Belgium), and quality was assessed by microfluidics using the RNA ScreenTape on the Agilent 2200 TapeStation (Agilent Technologies, Wilmington, DE, USA).

### Microarray Analysis

RNA was processed and hybridized to GeneChip™ Human Transcriptome Arrays 2.0 (P/N 902162) according to the Affymetrix GeneChip™ WT Pico Reagent Kit Users Guide (P/N 703262 Rev. 1) as follows: Biotinylated cDNA were prepared from 20–50 ng total RNA. Labeled cDNA were hybridized to the arrays in a GeneChip™ Hybridization Oven 640 by rotating at 60 rpm, 45°C for 16 h. Arrays were then washed and stained in the Affymetrix Fluidics Station 450 and scanned using the Affymetrix 3000 7G GeneChip™ Scanner with Autoloader. Raw intensity data were generated from array images using the Affymetrix AGCC software.

### Data Analysis

#### Preprocessing

Affymetrix Human Transcriptome Arrays CEL files were preprocessed using Robust Multi-array Average (RMA) (33) implemented in the *oligo* package (34) and annotation from the *hta20sttranscriptcluster.db* package of Bioconductor (35). Since samples were profiled in several batches as a part of a larger study, correction for potential batch effects was performed using the *removeBatchEffect* function of the *limma* package in *Bioconductor*. After batch effect correction, data from the sample collected at the time of labor from the 21 women who had spontaneous term labor were removed to avoid confounding gestational age-related changes with those due to the onset of labor at term. The final analysis set of 261 transcriptomes was used in downstream analyses described below.

#### Expression Calling

Transcript clusters (typically one or two per unique gene) were deemed present in a given sample if one of its probesets (targeting a specific exon) was expressed above background (*p*-value for expression above background p_DBAG_ <0.05) determined using the Transcriptome Analysis Console (version 4.0) (ThermoFisher Scientific). Genes were retained if deemed present in >25% of the 261 samples.

#### Differential Expression

Expression profiles were visually inspected to determine consistency of the data in sequential samples collected from the same woman. One of 261 samples consistently had the lowest value for a large fraction of the genes and was deemed as outlier and removed from further analysis. Linear mixed-effects models (36) were then used to fit log_2_ gene expression data as a function of gestational age (continuous) and included cubic polynomial terms of gestational age as fixed effects and a random intercept term for each woman. Significance *p*-values for the association of gene expression and gestational age were determined using likelihood ratio tests between a model with and without gestational age terms. A False Discovery Rate adjusted *p*-value (*q*-value) <0.1 and a fold change (FC) of >1.25 were required for significance. Fold change was determined as the ratio of the highest vs. lowest average expression from 10 to 40 weeks of gestation. Linear mixed-effects models were fit using the *lmer* function, while the likelihood ratio tests were performed using the *anova* function available in the *lme4* R package (36).

#### Gene Ontology and Pathway Analysis

Gene ontology and pathway analysis was conducted using a hypergeometric test on Gene Ontology (GO) (37) and Developmental FunctionaL Annotation at Tufts (DFLAT) databases (38), as well as on Curated Gene Sets (C2) collection from the Molecular Signatures Database (MSigDB) database (39). In addition, enrichment tests were performed for tissue specificity and chromosomal locations of genes. Tissue-specific genes were defined as those with median expression 30 times higher in a given tissue than the median expression of all other tissues documented in the Gene Atlas (40) as previously described (41).

Unless otherwise stated, all enrichment analyses were based on a hypergeometric test and accounted for multiple testing with *q* < 0.05 being considered a significant result. In all enrichment analyses, the background gene list was defined as the compendium of genes deemed present in >25% of the samples.

#### Changes in Cell-Type Specific mRNA Signatures With Gestational Age

In this analysis, we tested whether previously reported cell-type specific mRNA signatures derived by single-cell RNA-Seq studies of placenta tissues (42) were modulated with advancing gestation in normal pregnancy. The 13 cell types identified by Tsang et al. (42) were: B cells, T cells, monocytes, cytotrophoblasts, syncytiotrophoblast, decidual cells, dendritic cells, endothelial cells, erythrocytes, Hofbauer cells, stromal cells, vascular smooth muscle cell, and extravillous trophoblasts. The mRNA signatures for these cell types were first quantified in each patient sample by averaging expression data over genes part of each signature. Before averaging, the data for each gene was first standardized by subtracting the mean and dividing by standard deviation of expression across term samples (>37 weeks). Cell-type specific expression averages were then fit as a function of gestational age using linear mixed-effects models, as described above for the analysis of data of individual genes.

#### Assessment of mRNA Protein Correlations

Maternal plasma abundance of 1,125 proteins in 71 samples collected from 16 of the women included in the current study were obtained from the S1 File of Erez et al. (43). The correlation between each mRNA and corresponding protein pair was assessed by fitting linear mixed-effects models with the response being the protein abundance and the predictor being the mRNA expression. These models included a random intercept term to account for the repeated observations from the same subject. The meaning of the mRNA coefficient in this model is change in log_2_ protein abundance for one unit change in log_2_ gene expression. The significance of the protein—mRNA correlation was assessed by the t-score for the regression line slope, and false discovery rate adjustment of resulting *p*-values was performed across all mRNA-protein pairs that were tested. A q-value <0.1 was considered a significant result.

## Results

### Longitudinal Patterns of the Cellular Transcriptome Throughout Normal Pregnancy

**Figure 1:**
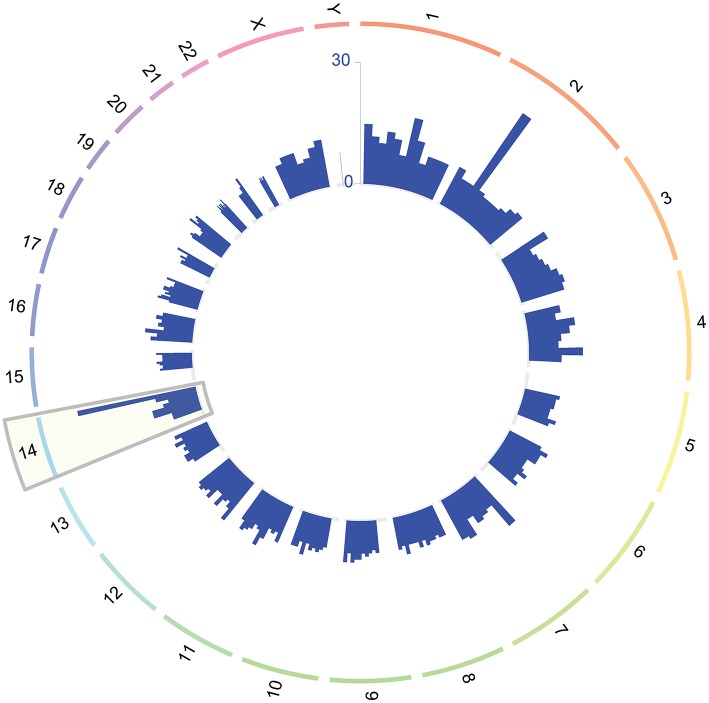
Chromosomal location of genes modulated throughout normal pregnancy. The outer circle represents the different chromosomes while the inner histograms show the number of differentially expressed genes binned by the genomic location within each chromosome. Chromosome 14 was the most enriched in differentially expressed genes throughout normal pregnancy (gray rectangle).

The mRNA profiles of longitudinal maternal blood samples were determined at exon level resolution by microarrays. The characteristics of the study population are shown in Table 1. A total of 26,458 protein-coding mRNA transcript clusters were expressed above background levels in at least 25% of the samples, as were 5,706 non-coding RNA transcript clusters. Analysis of longitudinal expression patterns identified 614 transcript clusters (510 coding and 104 non-coding) with significant expression modulation during gestation (*q* < 0.1 and minimum fold change of 1.25) (Supplementary File 1, Supplementary Figure 1). Significant transcripts were found on all chromosomes; yet, more differentially expressed transcripts than expected were observed on chromosome 14 (51/614 transcript clusters, odds ratio = 3.5, *p* < 0.0001; Figure 1), with 28/51 differentially expressed genes on this chromosome being annotated to immune processes. Chromosome 14 includes genes of critical importance for immunity (44); therefore, these data show that pregnancy has a strong effect on the maternal immune system.

**Table 1:** Demographic characteristics of the women included in the study.

**Characteristics**	**Median (IQR) or % (*n*)**
Age (years)	25 (21–28)
Prepregnant BMI (kg/m^2^)	25.8 (22.5–30.9)
Nulliparity (%)	32.7% (16)
Race (%)	
African American	91.8% (45)
White	4.1% (2)
Other	4.1% (2)
Gestational age at delivery (weeks)	39.3 (38.6–39.9)
Route of delivery	
Vaginal delivery	53.1%(26)
Cesarean delivery	46.9% (23)
Birth weight (grams)	3,285 (3,050–3,495)

*Continuous data are presented as median [Interquartile Range (IQR)] and categorical data as percentage (number). BMI, body mass index*.

**Figure 2:**
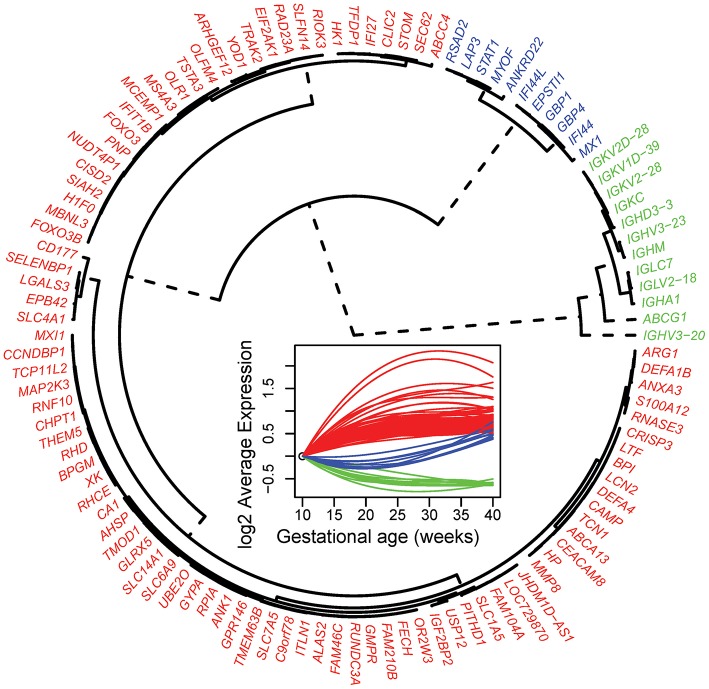
Clustering of average gene expression profiles throughout normal pregnancy. Average profiles of genes that change throughout normal pregnancy and have a fold change >1.5 were clustered using hierarchical clustering. The distance metric used in the clustering was 1-Pearson correlation. Three clusters were identified: Cluster 1 (red, 89 genes), Cluster 2 (green, 12 genes), and Cluster 3 (blue, 11 genes). Note that, in this figure, the average gene expression profiles vs. gestational age were reset so that their value is 0 at 10 weeks of gestation.

To define clusters of expression trajectories during gestation, we focused on 112 of the 614 significant transcript clusters that changed more than 1.5-fold from 10 to 40 weeks of gestation. Three distinct clusters of expression modulation emerged: genes that (1) steadily increased throughout gestation (89 genes; Figure 2, red cluster), (2) steadily decreased throughout gestation (12 genes; Figure 2, green cluster), and (3) decreased prior to mid-gestation followed by an increase (11 genes; Figure 2, blue cluster). These results indicate that the expression of the most highly modulated genes increases with advancing gestational age.

**Figure 3:**
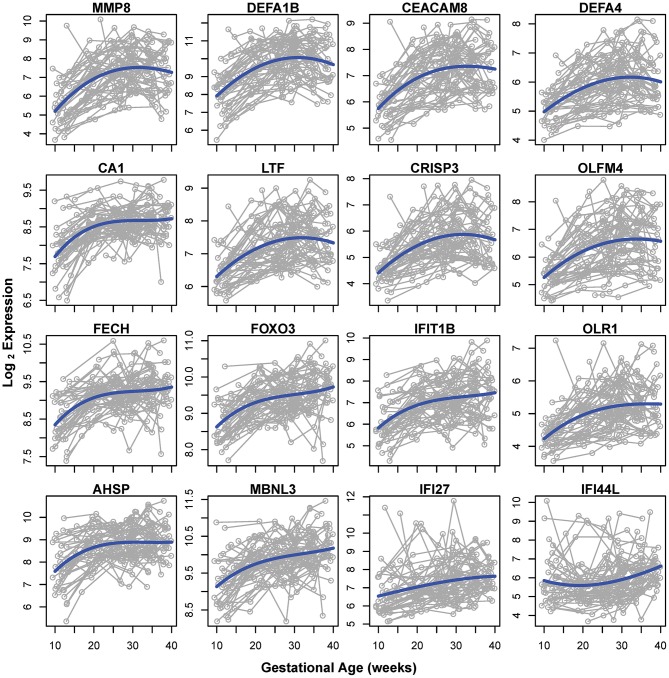
Genes changing in expression >2-fold from 10 to 40 weeks of gestation. Gray lines represent log_2_ normalized gene expression in 4–6 samples for each of the 49 women. Blue lines correspond to the average expression determined by a polynomial fit by linear mixed-effects models.

Of note, the 19 mRNA transcript clusters (corresponding to 16 unique genes) that changed more than 2-fold in expression during pregnancy all increased from 10 to 40 weeks of gestation (Figure 3, gray lines correspond to individual pregnancies and blue lines show the average expression). The expression of these 16 genes increased from early to late pregnancy and tended to plateau near term, with the exception of 2 genes (interferon-induced protein 27 and 44-like) (Figure 3). Several of these most highly modulated genes are related to host immune response (e.g., *MMP8, DEFA1B*, and *DEFA4*) (45, 46), again emphasizing the immune response adaptations during normal pregnancy.

### Biological Processes, Pathways, and Immune Cell Signatures Associated With Advancing Gestation in Normal Pregnancy

We performed gene ontology enrichment analysis to interpret the changes in gene expression occurring throughout gestation. We identified 157 biological processes modulated during gestation, which included *cellular and humoral immunity, defense response, response to external biotic stimulus* (e.g., bacteria and viruses), *regulation of lipid storage, interleukin-1beta production and secretion*, and *erythrocyte development*, among others (Table 2). An additional 134 biological processes altered during gestation were found when querying the Developmental FunctionaL Annotation at Tufts (DFLAT) database, such as *stress response, immune system development, cytokine response*, and *regulation of angiogenesis* (Table 3).

**Table 2:** Gene ontology biological processes enriched in genes differentially expressed with gestational age.

**Biological process**	**Count**	**Size**	**Odds ratio**	***q***
Immune system process	176	2,520	4.4	0.000
Immune response	128	1,580	4.6	0.000
Defense response	125	1,736	3.9	0.000
Regulation of immune system process	103	1,470	3.6	0.000
Innate immune response	85	1,063	4	0.000
Regulation of immune response	74	956	3.8	0.000
Immune effector process	61	740	3.9	0.000
Positive regulation of immune system process	65	886	3.5	0.000
Response to external biotic stimulus	63	845	3.5	0.000
Response to other organism	63	845	3.5	0.000
Defense response to other organism	47	515	4.3	0.000
Response to biotic stimulus	63	879	3.4	0.000
Positive regulation of immune response	49	639	3.5	0.000
Humoral immune response	24	178	6.4	0.000
Immune response-regulating signaling pathway	47	631	3.4	0.000
Immune response-activating signal transduction	38	459	3.8	0.000
Activation of immune response	40	510	3.6	0.000
Lymphocyte mediated immunity	25	217	5.3	0.000
Immune response-regulating cell surface receptor signaling pathway	39	499	3.5	0.000
Hemopoiesis	48	708	3.1	0.000
Immune response-activating cell surface receptor signaling pathway	30	315	4.3	0.000
Leukocyte mediated immunity	28	285	4.5	0.000
Adaptive immune response	30	332	4.1	0.000
Response to bacterium	37	481	3.5	0.000
Defense response to bacterium	23	211	5	0.000
Complement activation, classical pathway	10	43	12	0.000
Hemoglobin metabolic process	7	17	27.6	0.000
Defense response to virus	26	313	3.7	0.000
Regulation of viral genome replication	12	69	8.4	0.000
Response to virus	29	391	3.3	0.000
Complement activation	11	61	8.7	0.000
Phagocytosis	20	209	4.3	0.000
Fc-gamma receptor signaling pathway	13	90	6.7	0.000
Adaptive immune response based on somatic recombination of immune receptors built from immunoglobulin superfamily domains	20	215	4.1	0.000
Immunoglobulin mediated immune response	14	112	5.7	0.000
B cell mediated immunity	14	115	5.5	0.000
Defense response to fungus	8	34	12.2	0.000
Immune response-regulating cell surface receptor signaling pathway involved in phagocytosis	12	86	6.5	0.000
Fc-gamma receptor signaling pathway involved in phagocytosis	12	86	6.5	0.000
Humoral immune response mediated by circulating immunoglobulin	10	58	8.3	0.000
Cell killing	13	103	5.8	0.000
Fc receptor signaling pathway	26	364	3.1	0.000
Cellular defense response	10	59	8.1	0.000
Protoporphyrinogen IX metabolic process	5	10	39.3	0.000
Viral genome replication	12	89	6.2	0.000
Porphyrin-containing compound metabolic process	8	36	11.3	0.000
Modification of morphology or physiology of other organism	12	90	6.1	0.000
Antibacterial humoral response	9	48	9.1	0.000
Receptor-mediated endocytosis	21	260	3.5	0.000
Fc receptor mediated stimulatory signaling pathway	12	92	6	0.000
Killing of cells of other organism	7	27	13.8	0.000
Disruption of cells of other organism	7	27	13.8	0.000
Type I interferon signaling pathway	11	78	6.5	0.000
Cellular response to type I interferon	11	78	6.5	0.000
Response to type I interferon	11	79	6.4	0.000
Antimicrobial humoral response	9	53	8.1	0.000
Regulation of symbiosis, encompassing mutualism through parasitism	18	213	3.7	0.000
Protein activation cascade	11	84	6	0.000
Tetrapyrrole metabolic process	9	56	7.6	0.000
Response to fungus	8	47	8.1	0.000
Extrinsic apoptotic signaling pathway	18	229	3.4	0.000
Antigen receptor-mediated signaling pathway	14	147	4.2	0.000
Iron ion homeostasis	11	95	5.2	0.001
Porphyrin-containing compound biosynthetic process	6	25	12.4	0.001
Cytokine secretion	14	152	4	0.001
Positive regulation of leukocyte activation	19	265	3.1	0.001
Positive regulation of viral genome replication	6	27	11.2	0.001
Regulation of macrophage derived foam cell differentiation	6	28	10.7	0.001
Tetrapyrrole biosynthetic process	6	28	10.7	0.001
Regulation of response to reactive oxygen species	6	28	10.7	0.001
Regulation of response to oxidative stress	8	55	6.7	0.001
Regulation of viral process	15	188	3.5	0.001
Cytolysis	6	30	9.8	0.001
Negative regulation of multi-organism process	13	148	3.8	0.001
Natural killer cell mediated immunity	8	58	6.3	0.001
Negative regulation of extrinsic apoptotic signaling pathway	10	92	4.8	0.002
Macrophage derived foam cell differentiation	6	31	9.4	0.002
Regulation of bone resorption	6	31	9.4	0.002
Foam cell differentiation	6	31	9.4	0.002
T cell receptor signaling pathway	11	114	4.2	0.002
Regulation of viral life cycle	14	176	3.4	0.002
Transition metal ion homeostasis	12	135	3.9	0.002
Erythrocyte differentiation	10	99	4.4	0.003
Hydrogen peroxide catabolic process	5	23	10.9	0.003
Positive regulation of leukocyte mediated immunity	9	83	4.8	0.003
Positive regulation of lymphocyte mediated immunity	8	67	5.3	0.003
Regulation of cellular response to oxidative stress	7	51	6.3	0.003
Negative regulation of viral process	9	85	4.7	0.004
Regulation of bone remodeling	6	37	7.6	0.004
Myeloid cell development	7	52	6.1	0.004
Negative regulation of cysteine-type endopeptidase activity involved in apoptotic process	9	87	4.6	0.004
Erythrocyte homeostasis	10	106	4.1	0.004
Erythrocyte development	5	25	9.8	0.004
Regulation of lipid storage	6	39	7.1	0.005
Negative regulation of cysteine-type endopeptidase activity	9	90	4.4	0.005
Response to interferon-gamma	12	154	3.3	0.006
Antigen processing and presentation of exogenous peptide antigen via MHC class I, TAP-dependent	8	74	4.8	0.006
Interaction with host	12	155	3.3	0.006
Heme metabolic process	5	28	8.5	0.006
Respiratory burst	5	28	8.5	0.006
Regulation of extrinsic apoptotic signaling pathway	12	157	3.3	0.006
Interleukin-1 beta secretion	5	29	8.2	0.007
Antigen processing and presentation of exogenous peptide antigen via MHC class I	8	78	4.5	0.008
Hydrogen peroxide metabolic process	6	44	6.2	0.008
Negative regulation of viral genome replication	6	45	6	0.008
Leukocyte mediated cytotoxicity	8	81	4.3	0.009
Negative regulation of viral life cycle	8	82	4.3	0.010
Response to transition metal nanoparticle	10	123	3.5	0.010
Interaction with symbiont	6	47	5.7	0.010
Myeloid cell homeostasis	10	125	3.4	0.011
Interleukin-1 secretion	5	33	7	0.012
Bone resorption	6	49	5.5	0.012
Positive regulation of immune effector process	11	150	3.1	0.013
Negative regulation of signal transduction in absence of ligand	5	34	6.8	0.013
Negative regulation of extrinsic apoptotic signaling pathway in absence of ligand	5	34	6.8	0.013
Interferon-gamma-mediated signaling pathway	8	87	4	0.013
Interleukin-1 beta production	6	51	5.2	0.014
T cell costimulation	7	71	4.3	0.016
Nucleotide-binding domain, leucine rich repeat containing receptor signaling pathway	6	53	5	0.016
Negative regulation of epithelial cell proliferation	9	112	3.4	0.016
Lymphocyte costimulation	7	72	4.2	0.017
Negative regulation of I-kappaB kinase/NF-kappaB signaling	6	54	4.9	0.018
Defense response to Gram-positive bacterium	7	73	4.2	0.018
Modification of morphology or physiology of other organism involved in symbiotic interaction	7	74	4.1	0.019
Cofactor biosynthetic process	10	139	3.1	0.020
Regulation of antigen receptor-mediated signaling pathway	5	39	5.8	0.021
Regulation of tissue remodeling	6	57	4.6	0.022
Lipid storage	6	60	4.4	0.026
Interleukin-1 production	6	60	4.4	0.026
Positive regulation of NF-kappaB transcription factor activity	9	123	3.1	0.026
Antigen processing and presentation of peptide antigen via MHC class I	8	101	3.4	0.026
Regulation of transforming growth factor beta receptor signaling pathway	8	102	3.3	0.028
Regulation of cellular response to transforming growth factor beta stimulus	8	102	3.3	0.028
Signal transduction in absence of ligand	7	81	3.7	0.028
Extrinsic apoptotic signaling pathway in absence of ligand	7	81	3.7	0.028
Regulation of lymphocyte mediated immunity	8	105	3.2	0.031
Positive regulation of cytokine secretion	7	84	3.6	0.032
Alpha-beta T cell activation	8	107	3.2	0.033
Positive regulation of adaptive immune response based on somatic recombination of immune receptors built from immunoglobulin superfamily domains	6	66	3.9	0.036
Macrophage activation	5	48	4.6	0.039
Regulation of extrinsic apoptotic signaling pathway in absence of ligand	5	48	4.6	0.039
Natural killer cell activation	6	68	3.8	0.039
Protein K48-linked ubiquitination	5	49	4.5	0.040
Positive regulation of adaptive immune response	6	69	3.7	0.041
Cellular iron ion homeostasis	6	71	3.6	0.045
Cholesterol transport	6	71	3.6	0.045
Negative regulation of transforming growth factor beta receptor signaling pathway	6	71	3.6	0.045
Negative regulation of cellular response to transforming growth factor beta stimulus	6	71	3.6	0.045
Regulation of cofactor metabolic process	5	51	4.3	0.045
Regulation of coenzyme metabolic process	5	51	4.3	0.045
Regulation of transcription factor import into nucleus	7	93	3.2	0.045
Sterol transport	6	72	3.6	0.046
Bone remodeling	6	72	3.6	0.046
Regulation of cytokine biosynthetic process	7	94	3.2	0.046
Transcription factor import into nucleus	7	94	3.2	0.046
Positive regulation of inflammatory response	7	94	3.2	0.046
Response to zinc ion	5	52	4.2	0.047

*Count, number of differentially expressed genes annotated to the category; Size, Number of expressed genes assigned to the category. Odds ratio, Enrichment odds ratio by Fisher's exact (hypergeometric) test; q, false discovery rate adjusted p-value. Values displayed as 0.000 should be interpreted as <0.0005*.

**Table 3:** DFLAT biological processes enriched in genes differentially expressed with gestational age.

**DFLAT biological process**	**Count**	**Size**	**Odds ratio**	***q***
Response to stress	138	2,632	3.4	0.000
Homeostatic process	44	785	3.2	0.000
Response to cytokine	35	542	3.7	0.000
Immune system development	36	618	3.4	0.000
Cellular response to cytokine stimulus	31	492	3.6	0.000
Regulation of defense response	35	609	3.3	0.000
Hematopoietic or lymphoid organ development	34	590	3.3	0.000
Cytokine-mediated signaling pathway	27	404	3.8	0.000
Regulation of multi-organism process	25	403	3.5	0.000
Regulation of cytokine production	25	415	3.4	0.000
Myeloid cell differentiation	17	206	4.8	0.000
Endocytosis	20	297	3.8	0.000
Regulation of innate immune response	22	358	3.5	0.000
Homeostasis of number of cells	11	99	6.6	0.000
Cytokine production	9	64	8.6	0.000
Positive regulation of defense response	19	321	3.3	0.000
Fc-epsilon receptor signaling pathway	18	298	3.4	0.000
Regulation of defense response to virus	13	169	4.4	0.000
Regulation of response to biotic stimulus	14	201	4	0.000
Regulation of immune effector process	18	315	3.2	0.000
Apoptotic signaling pathway	16	261	3.5	0.000
Regulation of apoptotic signaling pathway	15	240	3.5	0.001
Response to transforming growth factor beta	11	137	4.6	0.001
Leukocyte migration	13	188	3.9	0.001
Positive regulation of innate immune response	16	271	3.3	0.001
Regulation of peptidase activity	16	273	3.3	0.001
Cell cycle arrest	10	117	4.9	0.001
Blood circulation	13	194	3.8	0.001
Circulatory system process	13	195	3.8	0.001
Negative regulation of apoptotic signaling pathway	10	122	4.7	0.001
Organic anion transport	15	260	3.2	0.001
Regulation of endopeptidase activity	15	263	3.2	0.001
Protein maturation	12	183	3.7	0.001
Secretion by cell	15	272	3.1	0.002
Cellular response to transforming growth factor beta stimulus	10	135	4.2	0.002
Regulation of hemopoiesis	11	162	3.8	0.002
Antigen processing and presentation of exogenous peptide antigen	11	165	3.8	0.002
Antigen processing and presentation of exogenous antigen	11	165	3.8	0.002
Transforming growth factor beta receptor signaling pathway	9	115	4.5	0.002
Pigment metabolic process	5	33	9.3	0.002
Inflammatory response	10	142	4	0.002
Protein polyubiquitination	11	169	3.7	0.002
Regulation of leukocyte activation	14	256	3.1	0.002
Positive regulation of lymphocyte activation	11	170	3.6	0.002
Regulation of carbohydrate metabolic process	8	94	4.9	0.002
Regulation of lymphocyte activation	13	227	3.2	0.002
Cellular response to interferon-gamma	8	96	4.8	0.003
Antigen processing and presentation of peptide antigen	11	175	3.5	0.003
Regulation of cysteine-type endopeptidase activity	11	175	3.5	0.003
Negative regulation of endopeptidase activity	10	150	3.8	0.003
Cellular transition metal ion homeostasis	7	78	5.2	0.004
Protein homooligomerization	9	127	4	0.004
Negative regulation of cytokine production	10	154	3.7	0.004
Negative regulation of peptidase activity	10	155	3.6	0.004
Protein secretion	6	58	6	0.004
Tumor necrosis factor-mediated signaling pathway	9	131	3.9	0.004
Response to tumor necrosis factor	11	187	3.3	0.004
Positive regulation of sequence-specific DNA binding transcription factor activity	11	188	3.3	0.005
Antigen processing and presentation	11	190	3.2	0.005
Regulation of lymphocyte proliferation	8	109	4.2	0.005
Regulation of cysteine-type endopeptidase activity involved in apoptotic process	10	163	3.4	0.005
Cell redox homeostasis	5	42	7.1	0.005
Regulation of mononuclear cell proliferation	8	110	4.1	0.005
Positive regulation of cell activation	11	193	3.2	0.005
Positive regulation of protein serine/threonine kinase activity	11	195	3.1	0.006
Regulation of cellular carbohydrate metabolic process	7	87	4.6	0.006
Positive regulation of T cell activation	9	140	3.6	0.006
Regulation of leukocyte proliferation	8	114	4	0.006
Positive regulation of hemopoiesis	7	89	4.5	0.007
Regulation of leukocyte differentiation	8	115	3.9	0.007
Protein processing	10	171	3.3	0.007
Positive regulation of leukocyte cell-cell adhesion	9	143	3.5	0.007
Cellular response to tumor necrosis factor	10	172	3.2	0.007
Leukocyte activation involved in immune response	7	91	4.4	0.007
Positive regulation of homotypic cell-cell adhesion	9	144	3.5	0.007
Cell activation involved in immune response	7	93	4.3	0.008
Regulation of intrinsic apoptotic signaling pathway	7	96	4.1	0.009
Regulation of T cell activation	10	180	3.1	0.009
positive regulation of lymphocyte proliferation	6	74	4.6	0.010
Reactive oxygen species metabolic process	6	74	4.6	0.010
Positive regulation of peptidyl-serine phosphorylation	5	52	5.6	0.011
Positive regulation of mononuclear cell proliferation	6	75	4.6	0.011
Regulation of epithelial cell proliferation	9	157	3.2	0.011
Male gonad development	7	101	3.9	0.011
Development of primary male sexual characteristics	7	101	3.9	0.011
Positive regulation of leukocyte proliferation	6	77	4.4	0.012
Retina homeostasis	5	54	5.3	0.012
Transmembrane receptor protein serine/threonine kinase signaling pathway	9	162	3.1	0.013
Positive regulation of cell-cell adhesion	9	163	3.1	0.014
Myotube differentiation	5	56	5.1	0.014
Regulation of interleukin-8 production	5	56	5.1	0.014
Positive regulation of cell cycle arrest	6	83	4.1	0.016
Negative regulation of intrinsic apoptotic signaling pathway	5	59	4.8	0.017
Positive regulation of transmembrane transport	6	84	4	0.017
Positive regulation of defense response to virus by host	7	112	3.5	0.017
Plasma membrane organization	8	142	3.1	0.018
Regulation of nucleocytoplasmic transport	8	143	3.1	0.019
Response to molecule of bacterial origin	7	114	3.4	0.019
Positive regulation of stress-activated MAPK cascade	6	87	3.9	0.019
Positive regulation of MAP kinase activity	8	144	3.1	0.019
Regulation of angiogenesis	8	145	3.1	0.019
Negative regulation of establishment of protein localization	8	145	3.1	0.019
Positive regulation of stress-activated protein kinase signaling cascade	6	88	3.8	0.020
Stimulatory C-type lectin receptor signaling pathway	7	116	3.4	0.020
Regulation of lymphocyte differentiation	5	63	4.5	0.020
Innate immune response activating cell surface receptor signaling pathway	7	117	3.3	0.020
Intrinsic apoptotic signaling pathway	7	117	3.3	0.020
Positive regulation of apoptotic signaling pathway	7	117	3.3	0.020
Organic hydroxy compound transport	6	90	3.7	0.021
Notch signaling pathway	6	91	3.7	0.022
Regulation of defense response to virus by host	7	120	3.2	0.022
Response to reactive oxygen species	6	94	3.6	0.025
Xenophagy	6	95	3.5	0.026
Male sex differentiation	7	124	3.1	0.026
Establishment of protein localization to plasma membrane	5	69	4.1	0.027
Positive regulation of leukocyte differentiation	5	69	4.1	0.027
Lymphocyte activation involved in immune response	5	71	4	0.029
Mitochondrial membrane organization	5	71	4	0.029
Regulation of peptidyl-serine phosphorylation	5	71	4	0.029
Response to UV	6	99	3.4	0.030
Negative regulation of cellular protein localization	6	102	3.3	0.033
Organic acid transmembrane transport	5	75	3.7	0.035
Regulation of cell cycle arrest	6	104	3.2	0.035
Activation of cysteine-type endopeptidase activity involved in apoptotic process	5	76	3.7	0.036
Positive regulation of binding	5	77	3.6	0.038
Regulation of T cell proliferation	5	78	3.6	0.040
Positive regulation of ion transmembrane transport	5	79	3.5	0.041
Negative regulation of transmembrane receptor protein serine/threonine kinase signaling pathway	5	80	3.5	0.043
Platelet degranulation	5	81	3.4	0.044
Activation of cysteine-type endopeptidase activity	5	82	3.4	0.046
Negative regulation of cytoplasmic transport	5	82	3.4	0.046
Tissue homeostasis	5	83	3.4	0.048
Amino acid transport	5	83	3.4	0.048
Localization within membrane	5	83	3.4	0.048

*DFLAT, Developmental FunctionaL Annotation at Tufts (DFLAT) database. Table are the same columns as in the legend of Table 2*.

Enrichment analyses were then expanded to canonical pathways and gene sets from the Molecular Signatures Database (MSigDB), and 53 such pathways were found to be associated with advancing gestation. These included the Reactome database (47) pathways: *immune system, adaptive immune system, cytokine signaling in immune system*, and *immunoregulatory interactions between a lymphoid cell and a non-lymphoid cell*, as well as the KEGG database (48) pathways: *natural killer cell-mediated cytotoxicity, antigen processing and presentation*, and *graft vs. host disease* (Table 4).

**Table 4:** MSigDB canonical pathways enriched in genes differentially expressed with gestational age.

**MSIGDB gene set name**	**Count**	**Size**	**Odds ratio**	***q***
Reactome immune system	61	877	4.2	0.000
Reactome adaptive immune system	38	510	4.4	0.000
Reactome immunoregulatory interactions between a lymphoid and a non-lymphoid cell	12	63	12.5	0.000
KEGG graft vs. host disease	10	39	18.2	0.000
KEGG natural killer cell mediated cytotoxicity	16	127	7.7	0.000
KEGG antigen processing and presentation	13	79	10.4	0.000
Reactome interferon signaling	16	149	6.4	0.000
BIOCARTA AHSP pathway	6	13	45	0.000
KEGG hematopoietic cell lineage	12	87	8.5	0.000
Reactome interferon gamma signaling	10	61	10.3	0.000
Reactome interferon alpha beta signaling	9	57	9.9	0.000
Reactome cytokine signaling in immune system	18	257	4	0.000
Reactome metabolism of porphyrins	5	14	29.1	0.000
Biocarta TOB1 pathway	5	19	18.7	0.000
PID HIF1 TF Pathway	8	66	7.3	0.001
Reactome Class I MHC mediated antigen processing presentation	15	234	3.6	0.001
KEGG allograft rejection	6	37	10.1	0.001
PID HDAC CLASS III pathway	5	25	13.1	0.001
PID SMAD2 3NUCLEAR Pathway	8	81	5.8	0.002
KEGG ABC transporters	6	44	8.3	0.002
PID IL4 2 pathway	7	63	6.6	0.002
PID IL12 2 pathway	7	63	6.6	0.002
Reactome L1CAM interactions	8	84	5.5	0.002
PID TNF pathway	6	46	7.9	0.002
KEGG asthma	5	30	10.5	0.002
KEGG autoimmune thyroid disease	6	47	7.7	0.002
KEGG porphyrin and chlorophyll metabolism	5	32	9.7	0.003
PID IL12 STAT4 pathway	5	33	9.3	0.003
Reactome antigen processing cross presentation	7	73	5.6	0.003
Reactome transcriptional activity of SMAD2 SMAD3 SMAD4 heterotrimer	5	36	8.4	0.004
Reactome signaling by TGF beta receptor complex	6	59	5.9	0.006
PID CD8 TCR downstream pathway	6	60	5.8	0.006
PID FCER1 pathway	6	61	5.7	0.006
KEGG T cell receptor signaling pathway	8	108	4.2	0.006
Reactome costimulation by the CD28 family	6	62	5.6	0.007
KEGG type I diabetes mellitus	5	43	6.9	0.007
Reactome nucleotide binding domain leucine rich repeat containing receptor NLR signaling pathways	5	44	6.7	0.008
Reactome apoptosis	9	144	3.5	0.009
Reactome signaling by the B cell receptor BCR	8	121	3.7	0.010
KEGG B cell receptor signaling pathway	6	75	4.6	0.013
KEGG amyotrophic lateral sclerosis ALS	5	53	5.5	0.013
PID MYC ACTIV pathway	6	77	4.4	0.014
KEGG ubiquitin mediated proteolysis	8	133	3.4	0.015
KEGG apoptosis	6	86	3.9	0.019
KEGG pancreatic cancer	5	70	4	0.031
KEGG viral myocarditis	5	70	4	0.031
Reactome metabolism of nucleotides	5	72	3.9	0.033
KEGG chronic myeloid leukemia	5	73	3.8	0.034
Reactome signaling by EGFR in cancer	6	103	3.2	0.036
Reactome signaling by SCF kit	5	75	3.7	0.036
PID p73 pathway	5	79	3.5	0.040
Reactome response to elevated platelet cytosolic Ca2	5	80	3.5	0.040
Reactome cell surface interactions at the vascular wall	5	83	3.4	0.044

*MSigDB, Molecular Signatures Database. Table columns as in legend of Table 2*.

We then aimed at determining the origin of observed transcriptional activity by using the GNF Gene Expression Atlas to define genes predominantly expressed in specific human tissue or cell types, as previously described (41). This analysis revealed that gene sets specific to CD4+ and CD8+ T cells, CD71+ erythroid cells, CD105+ endothelial cells, and CD56+ NK cells, among others, were over-represented among the mRNAs that were modulated during gestation (*q* < 0.05) (Table 5). In addition, genes reported to be specific to fetal organs (liver, lung, and brain) and the placenta were also enriched among significant genes (*q* < 0.05) (Table 5). These data show that maternal and fetal cell-specific transcripts found in the maternal circulation are being modulated with advancing gestation.

**Table 5:** Tissue or cell type-specific gene sets enriched in genes differentially expressed with gestational age.

**Tissue/cell type**	**Count**	**Size**	**Odds ratio**	***q***
CD71+ early erythroid cells	80	198	40.4	0.000
Bone marrow	45	102	44.2	0.000
CD105+ endothelial cells	34	142	17.3	0.000
CD56+ NK cells	33	279	7.3	0.000
CD8+ T cells	28	215	8.1	0.000
CD4+ T cells	27	207	8.1	0.000
Fetal liver	22	129	11.1	0.000
Tonsil	17	91	12.3	0.000
CD19+ B cells (neg. sel.)	23	217	6.4	0.000
BDCA4+ dentritic cells	25	269	5.5	0.000
Trachea	12	74	10.2	0.000
Whole blood	24	322	4.3	0.000
Burkitt's lymphoma cells (Daudi)	13	103	7.7	0.000
CD34+ cells	14	157	5.2	0.000
Salivary gland	8	51	9.8	0.000
HL-60 promyelocytic leukemia cells	8	58	8.4	0.000
721-B-lymphoblasts	18	299	3.4	0.000
CD33+ myeloid cells	19	335	3.2	0.000
Lymph node	8	64	7.5	0.000
Thymus	9	85	6.2	0.000
Bronchial epithelial cells	11	141	4.5	0.000
Colorectal adenocarcinoma	8	83	5.6	0.000
Burkitt's lymphoma cells (Raji)	9	110	4.7	0.001
Colon	10	138	4.1	0.001
CD14+ monocytes	15	285	2.9	0.001
Pancreatic islet	8	117	3.9	0.004
Fetal lung	7	111	3.5	0.011
Prostate	7	114	3.4	0.013
K-562 chronic myelogenous leukemia cells	4	42	5.5	0.016
Fetal brain	9	186	2.7	0.018
Placenta	10	233	2.4	0.026
Ovary	3	29	6	0.032
Small intestine	7	142	2.7	0.034

*Gene sets are defined based on average expression in a given tissue/cell type >30 time the median expression across all other biotypes cataloged in the GNF Gene Expression Atlas (see Methods). Table columns are the same as in the legend of Table 2*.

**Figure 4:**
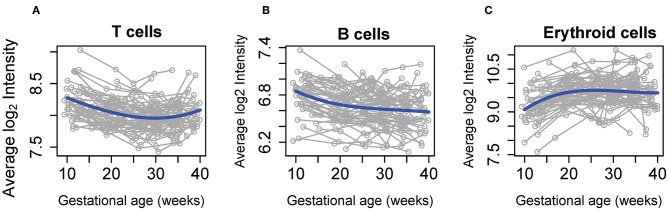
Meta-gene expression of specific cell types differentially regulated throughout normal pregnancy. The average expression of genes defined as specific for **(A)** T cell, **(B)** B cell, and **(C)** erythroid cell populations by Tsang et al. (42) are shown as a function of gestation. Blue lines correspond to the average expression estimated by linear mixed-effects models. The fold change in expression from 10 to 40 weeks was 1.2 for T cells and B cells and 1.6 for erythroid cells (all, *p* < 0.001).

The average abundance of cell type-specific gene sets recently defined using single-cell transcriptomics (42) were also analyzed for systematic changes with gestational age at blood draw in our cohort. This analysis revealed that expression of mRNAs specific to three cell subtypes were dynamically altered throughout normal pregnancy: (1) the T-cell-specific mRNA signature decreased from the first to second trimester, followed by a subsequent increase during the third trimester (Figure 4A); (2) the B-cell-specific mRNA signature decreased steadily throughout gestation (Figure 4B); and (3) the expression of genes specific to nucleated erythroid cells (*HBZ, ALAS2*, and *AHSP*) significantly increased as gestation progressed (Figure 4C). These findings demonstrate, for the first time, that single-cell RNA-Seq-derived signatures of erythroid cells change throughout normal gestation in maternal whole blood, while trends found for T cells and B cells were similar to those reported in whole blood (49) and by using cell-free RNA analysis (42).

### Correlation Between the Cellular Transcriptome and Plasma Proteome Throughout Normal Pregnancy

**Figure 5:**
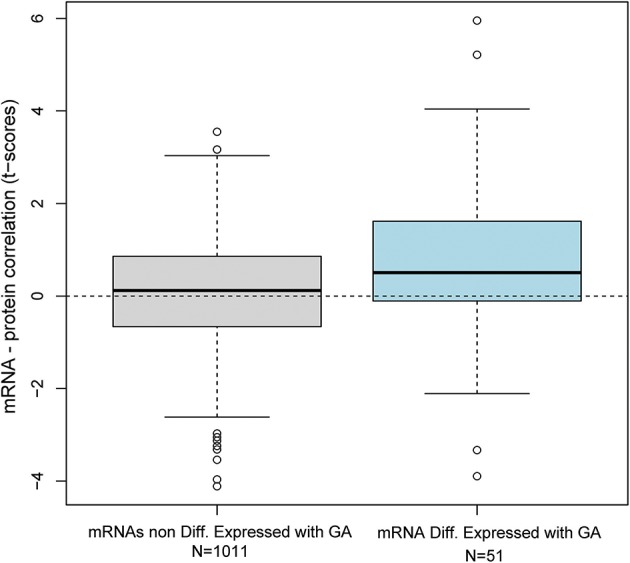
Distribution of mRNA-protein correlation *t*-scores. The correlation between mRNA and protein abundance was assessed by linear mixed-effects models using data collected from 71 samples provided by 16 women. The distribution of t-scores for the linear correlation slope is shown for 51 mRNAs differentially expressed with gestation and 1011 mRNAs that were not differentially expressed.

**Figure 6:**
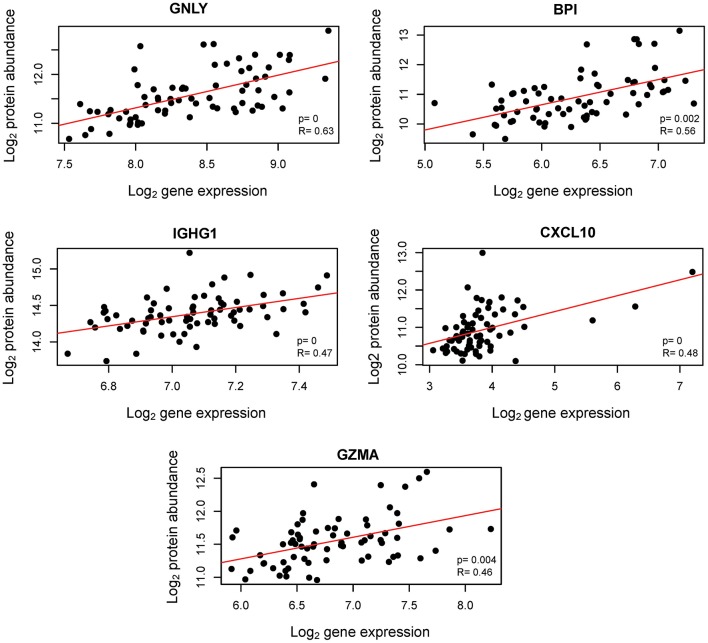
Correlation between cellular transcriptome and maternal plasma proteome throughout normal pregnancy. Aptamer-based protein abundance measurements are plotted against mRNA expression. R: naïve Spearman correlation coefficient; p: likelihood ratio test *p*-value from linear mixed-effects models assessing the linear correlation accounting for repeated measurements from the same subjects.

Transcription does not always correlate with protein translation (50, 51). Therefore, we investigated the correlation between the mRNAs that were modulated throughout gestation and their corresponding protein abundance. Maternal plasma abundance of 1,125 proteins in 71 samples collected from 16 of the women included in the current study was previously reported (43, 52). First, we assessed the mRNA-protein correlation for 53 of the 614 transcript clusters that changed throughout gestation and for which abundance data for the corresponding protein were available. These correlations were compared to those of 1,011 mRNA-protein pairs that did not change with gestation. The mRNA-protein correlations were significantly higher for transcripts that changed throughout gestation compared to those that did not (Wilcoxon test for comparing t-scores of the linear regression slope obtained by linear mixed-effects models for each mRNA-protein pair, *p* = 0.01) (Figure 5). Among the 53 transcripts that changed throughout gestation, *BPI, IGHG1, CXCL10, GNLY*, and *GZMA* had a significant mRNA-protein correlation as assessed by both linear mixed-effects models and a naïve Spearman correlation test (*q* < 0.05 for both analyses) (Figure 6). Notably, two of these genes (*GNLY* and *GZMA*) were included in the T-cell-specific mRNA signature that was modulated overall throughout gestation.

## Discussion

### Principal Findings of the Study

(1) Chromosome 14 was the most enriched in transcripts differentially expressed throughout normal pregnancy (51/613 mRNA clusters). (2) Among the most differentially expressed genes (*q* < 0.1, and fold change > 1.5), three distinct longitudinal patterns were observed: (i) steady increase throughout gestation (89 genes), (ii) steady decrease throughout gestation (12 genes), or (iii) decrease prior to mid-gestation followed by an increase (11 genes). (3) Sixteen genes, most of them related to host immune response mediators (e.g., *MMP8, DEFA1B, DEFA4, LTF*), displayed >2-fold change in expression and steadily increased from 10 to 40 weeks of gestation. (4) Approximately 300 biological processes and 53 pathways, many of which were related to immunity and inflammation, were enriched among the differentially expressed genes (*q* < 0.05). (5) Genes changing with gestation were among those specific to T cells, B cells, CD71+ erythroid cells, natural killer cells, and endothelial cells, as defined based on the GNF Gene Expression Atlas. (6) The meta-gene expression of mRNA signatures for T cells, B cells, and erythrocyte cells were significantly modulated throughout gestation, each following a unique pattern (*p* < 0.05). (7) The correlation between mRNA and protein abundance was higher for mRNAs that were differentially expressed throughout gestation than for those that were not (*p* = 0.01). (8) Significant and positive mRNA-protein correlations (*q* < 0.05) were observed for *BPI, IGHG1, CXCL10*, and two members of the T-cell mRNA signature (*GNLY, GZMA*). The expression trends and variability in expression of individual genes and meta-genes in normal pregnancy (nomograms) derived herein will be the basis for future studies aiming at developing biomarkers for obstetrical disease.

### Transcriptomic Changes During Pregnancy

Previous studies have investigated the cellular (53, 54) and cell-free (55) transcriptome in the maternal circulation at different time points during normal pregnancy using either 3-prime-end biased microarrays or targeted approaches. The current study, however, is the first to quantify at exon-level resolution the cellular transcriptome during normal pregnancy in up to six samples per pregnancy. More than one-half (54%, 277/514) of the unique differentially expressed genes identified herein were also among the 2,321 genes (*q* < 0.1) reported by Heng et al. (54) to change from 17–23 to 27–33 weeks of gestation. Similarly, 47% (242/514) of the genes found in this study were among the 3,830 genes reported by Al-Garawi et al. (53) as changing from 10–18 to 30–38 weeks. The overlap between the genes reported as differentially expressed in these two studies and those identified herein is significant (Fisher's exact test *p* < 0.0001 for both). However, unlike in the two previous studies involving a pair of samples from each woman, the availability of four to six longitudinal samples per patient in this study enabled us to capture more complex expression trajectories in the window of 10–40 weeks of gestation, and to identify distinct clusters of such gene expression trajectories.

Compared to another recent study by Ngo et al. (55) that involved more frequent sampling than used herein, our study has the advantage of an unbiased assessment of the whole cellular transcriptome as opposed to a targeted assessment of genes that are placenta-, immune-, and fetal liver-specific. Of note, among the 14 immune-specific cell-free mRNAs selected by Ngo et al. (55) as best predictors of gestational age at blood draw, 11 were also identified in our study, with *CEACAM8, DEFA4, LTF*, and *MMP8* being among those with highest fold change. Although our results are somewhat consistent with those reported by Ngo et al. (55), it should be noted that cellular and cell-free transcripts can follow different patterns in similar physiological and pathological processes (56).

### Correlations Between the Cellular Transcriptome and the Plasma Proteome Throughout Normal Pregnancy

The finding that the maternal transcriptome features inflammation-related processes and pathways that are being activated in preparation for labor at term is in agreement with our previous studies in gestational tissues [cervix (57), myometrium (58), membranes (59)] and a similar longitudinal study of the maternal plasma proteome (52). In addition to finding several common biological processes that are modulated in both the maternal plasma proteome and cellular transcriptome (such as *defense response, defense response to bacterium, defense response to fungus, regulation of bone resorption, leukocyte migration*) we assessed, for the first time, the extent of the agreement in whole blood mRNA and protein changes with gestation in the same set of samples. Although the correlations between mRNA and protein expression reported in the literature are notoriously poor, recent studies showed that mRNA-protein correlation is higher for mRNAs that are differentially expressed in a given condition than for those that are not (51). Our finding that the correlation of mRNA-protein pairs is higher for transcripts changing with gestation than those who do not is therefore consistent with previous observations (51).

### T Cells in the Maternal Circulation During Normal Pregnancy

Maternal T cells are implicated in the physiological processes occurring throughout gestation (60–63). Effector and activated T cells are found at the maternal-fetal interface before (64–70) and during (71–73) spontaneous labor at term, and these cells are associated with the timing of term parturition (74). Effector T cells are also found in the maternal blood prior to Shah et al. (75) and during (76) labor at term. In the current study, we demonstrated that the T-cell-specific mRNA expression in the maternal circulation was decreased prior to mid-gestation but upregulated from mid-gestation until term. Moreover, for two of 19 genes of this signature, there was a significant correlation between cellular mRNA and plasma proteomic profiles; this is consistent with recent cytomic and proteomic studies in the maternal circulation (77, 78). In addition, we recently showed the same u-shaped pattern of expression for the T-cell mRNA signature during gestation in a smaller set of patients profiled with RNA-Seq and qRT-PCR platforms (49). Together, these findings illustrate the importance of maternal T cells during normal pregnancy.

Alteration of systemic T-cell populations has also been implicated in preterm parturition (79–81), especially since aberrant activation of these cells can induce the onset of preterm labor (82, 83). On the other hand, the absence of T cells in a mouse model caused an increased susceptibility to endotoxin-induced preterm birth, which was reversed by adoptive transfer of CD4+ T cells (84). From a histopathological standpoint, T cells are detected in placental lesions related to maternal anti-fetal rejection such as villitis of unknown etiology (85–87), chronic chorioamnionitis (88), and chronic deciduitis (89), which have also been linked to the onset of term and preterm labor (88, 90–94). The chronic nature (95, 96) of these lesions has led our group to propose them as indicators of maternal anti-fetal rejection, which can lead to preterm labor or even fetal death (86, 90, 93, 94, 97–100). Future studies are needed to establish whether the early detection of T-cell alterations in the maternal circulation may identify pregnancies at risk for obstetrical disease such as preterm labor/birth and fetal death.

### B Cells in the Maternal Circulation During Normal Pregnancy

Several studies have suggested a role for B cells in the maintenance and success of pregnancy (101–106). Circulating CD5+ (B1) B cells were shown to decrease during pregnancy, only returning to normal levels after parturition (107). This finding was later shown to occur in mice, where a decreased influx of newly generated B cells to the blood and spleen was observed while mature B cells were increased in uterine-draining lymph nodes (108). An expansion of marginal zone B cells also ensued (108, 109), which was proposed to participate in the production of protective antibodies during pregnancy (109, 110). Accordingly, maternal serum antibody concentrations increased concomitantly with B-cell population changes (109), possibly as a result of the anti-inflammatory microenvironment maintained at the maternal-fetal interface throughout most of pregnancy (111). Such antibody production has been considered the primary contribution of B cells to maternal-fetal tolerance during pregnancy (101).

Interleukin-10-producing regulatory B cells (Bregs) have also been described as important players during pregnancy (112, 113). Such adaptive immune cells increased in normal pregnancy in an hCG-dependent manner (113, 114) and suppressed effector T-cell cytokine production (113). Trophoblast cells facilitated the conversion of IL10-deficient B cells into IL10-expressing B cells (114), which is in line with a previous report showing that the adoptive transfer of Bregs restored maternal-fetal tolerance (112). Indeed, pregnant women treated with the B-cell-depleting treatment rituximab had a higher occurrence of pregnancy loss (115), although further investigation of this phenomenon is warranted (116).

In the current study, we showed that the B-cell-specific mRNA signature moderately decreased throughout pregnancy. Our observations correspond to a previous report indicating that the majority of maternal peripheral B-cell subsets are reduced in late gestation compared to the non-pregnant state (117), whereas Bregs are upregulated (117). The combined effects of such dynamic changes on the overall circulating B-cell mRNA signature are therefore minimal, as we have demonstrated here. Taken together, these findings suggest that, while total maternal peripheral B cells are mostly maintained, subset-specific changes occur throughout pregnancy.

### Erythroid Cells in the Maternal Circulation During Normal Pregnancy

A constant bi-directional trafficking of maternal and fetal cells occurs during normal pregnancy (118–124). Indeed, cell-free fetal DNA is present in the maternal circulation throughout normal pregnancy (125–132) and its levels increased from mid to late gestation (128, 133–140). Increased concentrations of cell-free fetal DNA or numbers of fetal cells in the maternal circulation (fetal microchimerism) have been linked to pregnancy complications such as preterm labor (141–145), preeclampsia (146–150), and intrauterine growth restriction (149, 151, 152). In addition, sequencing cell-free fetal DNA in the maternal circulation may serve for non-invasive prenatal testing (153). Therefore, determining the impact that fetal cells and their released products (e.g., cell-free fetal DNA) may have on maternal health is critical, given that such cells can remain in the maternal circulation for decades (153, 154).

Fetal nucleated erythroid cells have been detected in the maternal blood (121, 155, 156) where they may be a source of cell-free fetal DNA (155). Previous reports showed that neonatal CD71+ erythroid cells have immunomodulatory functions on cord blood leukocytes (157–159), and that their direct contact with maternal peripheral immune cells increases the secretion of pro-inflammatory mediators by such cells (159). Therefore, it is likely that the trafficking of CD71+ erythroid cells from the fetus into the mother directly affects maternal immune responses (159). Nucleated erythroid cells have also been described in the placenta, where their presence is correlated with the number of such cells in the cord blood (160), and these cells also display immunomodulatory properties *in vitro* (161).

Herein, we showed that the erythroid cell-specific mRNA signature was upregulated throughout gestation in the maternal circulation. This finding is in line with previous reports showing that fetal microchimerism increases during pregnancy (162, 163). In addition, a recent study showed that CD71+ erythroid cells are increased in the maternal circulation throughout gestation, peaking during the third trimester and falling to baseline levels after delivery (164). Together, these findings illustrate that erythroid cells, most likely of fetal origin, are present in the maternal circulation and their transcriptome is modulated as gestation progresses. These data provide a possible mechanism whereby the developing fetus can modulate maternal immunity.

## Conclusion

We have reported herein a detailed characterization of the longitudinal maternal whole blood transcriptomic changes in normal pregnancy. We have shown that these changes are genome-wide, yet we found that chromosome 14 was particularly enriched in genes modulated with advancing gestation. There was a significant overlap in expression changes described herein with those previously reported in whole blood analyses based on only two time points, while some of the most strongly modulated mRNAs identified herein were also previously reported as the best predictors of gestational age in cell-free RNA analyses of maternal blood. Our systems biology approach to the interpretation of these expression changes in the maternal cellular transcriptome during pregnancy revealed significant longitudinal patterns of expression for immune-related gene sets, such as those specific to T cells, B cells, and erythroid cells. Moreover, for the first time, we demonstrated positive correlations between the cellular transcriptome and plasma proteome for specific genes, including those expressed by T cells. The expression trajectories of protein coding and non-coding transcripts in normal pregnancy described herein may serve as references and hence enable the discovery of biomarkers for obstetrical disease.

## Data Availability Statement

The raw and summarized microarrays gene expression data are available as a Gene Expression Omnibus series (https://www.ncbi.nlm.nih.gov/geo/query/acc.cgi?acc=GSE121974).

## Ethics Statement

The studies involving human participants were reviewed and approved by Institutional Review Boards of Wayne State University and NICHD. The patients/participants provided their written informed consent to participate in this study.

## Author Contributions

AT, RR, SH, and NG-L conceived the research. SH and RR supervised the enrollment of the patients and collection of samples. AT and NG-L carried out research and drafted the manuscript. GB contributed to data visualization and to the preparation of data submission to the Gene Expression Omnibus. AT analyzed data. AT, RR, and NG-L interpreted the data. SH, RR, PP, JK, and SB provided feedback on the manuscript. All authors read and approved the final manuscript.

### Conflict of Interest

The authors declare that the research was conducted in the absence of any commercial or financial relationships that could be construed as a potential conflict of interest. The handling editor is currently co-organizing a Research Topic with one of the authors, NG-L, and confirms the absence of any other collaboration.

## Supplementary Material

Supplementary File 1 Differential gene expression with gestational age. Each row corresponds to one of the 614 transcript clusters associated with gestational age. ID, Affymetrix transcript cluster identifier; SYMBOL, gene symbol; ENTREZ, Entrez gene database identifier; Name, Gene name; mRNA, mRNA identifier; Source, mRNA identifier source; locus.type, coding vs. non-protein coding assignment; Chromosome, chromosome number; strand, chromosome strand; start/stop, genomic coordinate start and stop for the transcript cluster; *p*-value, significance value for the polynomial relation between log gene expression and gestational age; FC, log_2_ fold change representing the log_2_ ratio of the highest and lowest fitted value from 10 to 40 weeks of gestation (see Figure 3). The sign is positive if value at 40 weeks is higher than the value at 10 weeks, and negative otherwise. adj.P.Value, False discovery adjusted *p*-value.

Supplementary Figure 1 Longitudinal gene expression profiles of genes associated with gestational age. Each figure shows data for one of the 614 transcript clusters associated with gestational age. The y-axis represents the log_2_ normalized gene expression, while the x-axis represents gestational age (weeks). Each line corresponds to one patient and each dot to one sample. The thick blue line represents the linear mixed effects model fit. The title in each plot, represents the transcript cluster identifier, gene symbol, gene name, *p*-value, and fold change. The meanings of these annotations are as in the legend of Supplementary File 1.
