# Supplementary material for: The Cellular Transcriptome in the Maternal Circulation During Normal Pregnancy: A Longitudinal Study
Source: Front Immunol. 2019 Dec 17;10:2863. doi: 10.3389/fimmu.2019.02863 (PMC6928201; doi:10.3389/fimmu.2019.02863)
Supplement: Supplementary Figure 1 — Longitudinal gene expression profiles of genes associated with gestational age. Each figure shows data for one of the 614 transcript clusters associated with gestational age. The y-axis represents the log2 normalized gene expression, while the x-axis represents gestational age (weeks). Each line corresponds to one patient and each dot to one sample. The thick blue line represents the linear mixed effects model fit. The title in each plot, represents the transcript cluster identifier, gene symbol, gene name, p-value, and fold change. The meanings of these annotations are as in the legend of Supplementary File 1. [file Image_1.PDF]

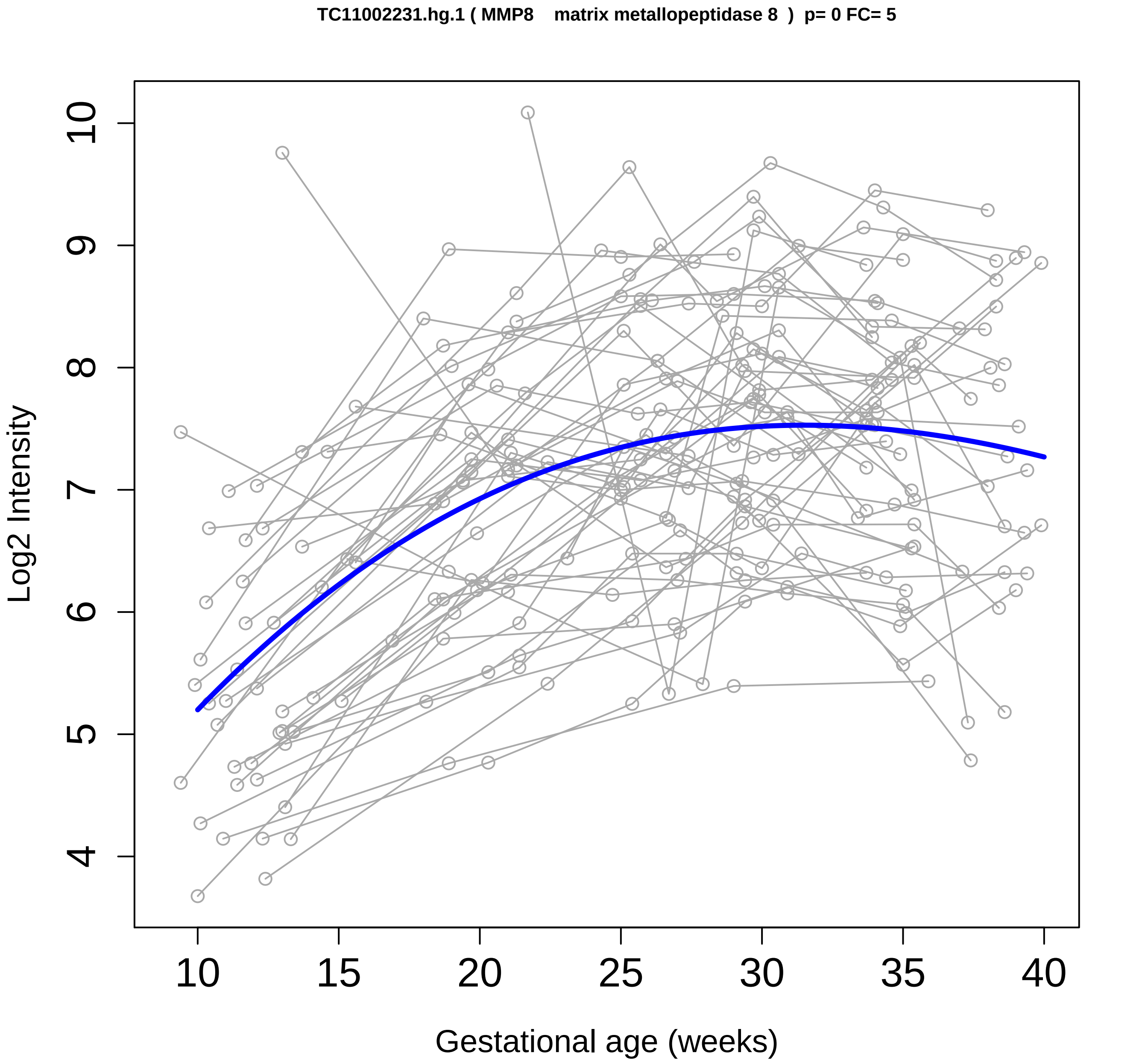
TC11002231.hg.1 (MMP8 matrix metallopeptidase 8) p= 0 FC= 5
Log2 Intensity
Gestational age (weeks)
10
9
8
7
6
5
4
10
15
20
25
30
35
40

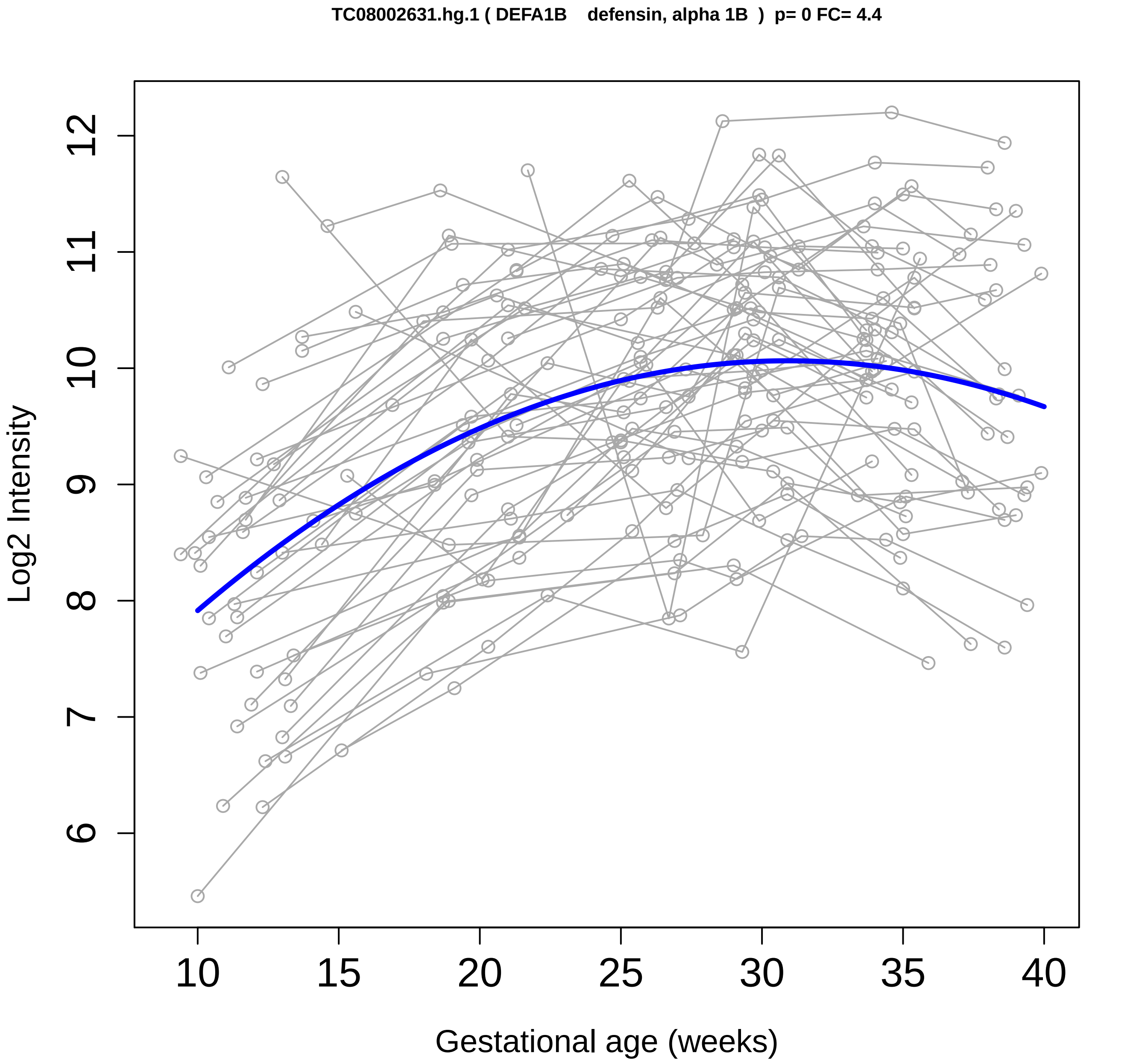

TC08002631.hg.1 (DEFA1B defensin, alpha 1B) p= 0 FC= 4.4
Log2 Intensity
6
7
8
9
10
11
12
10
15
20
25
30
35
40
Gestational age (weeks)

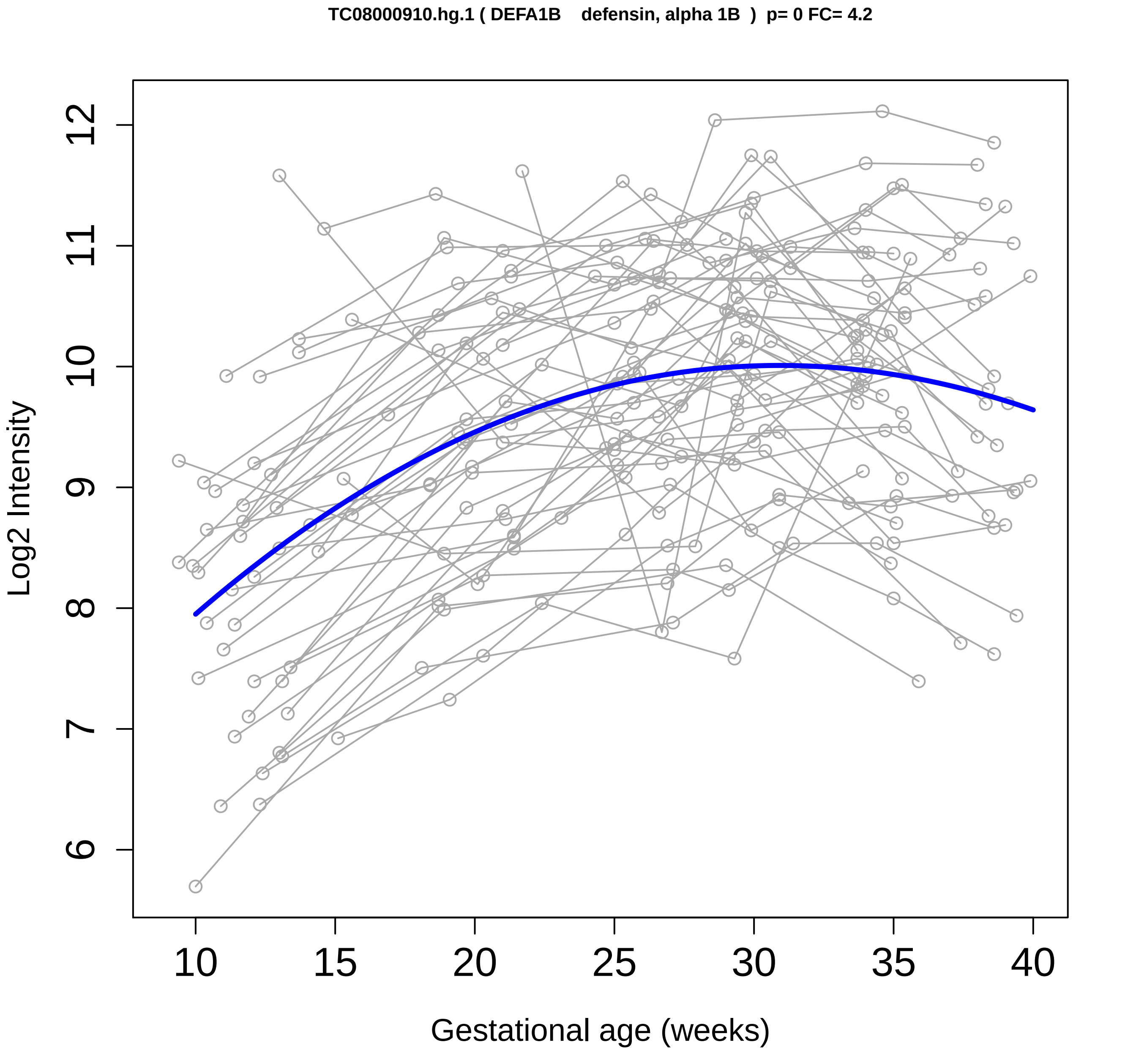
TC08000910.hg.1 (DEFA1B defensin, alpha 1B) p= 0 FC= 4.2
Log2 Intensity
6
7
8
9
10
11
12
10
15
20
25
30
35
40
Gestational age (weeks)

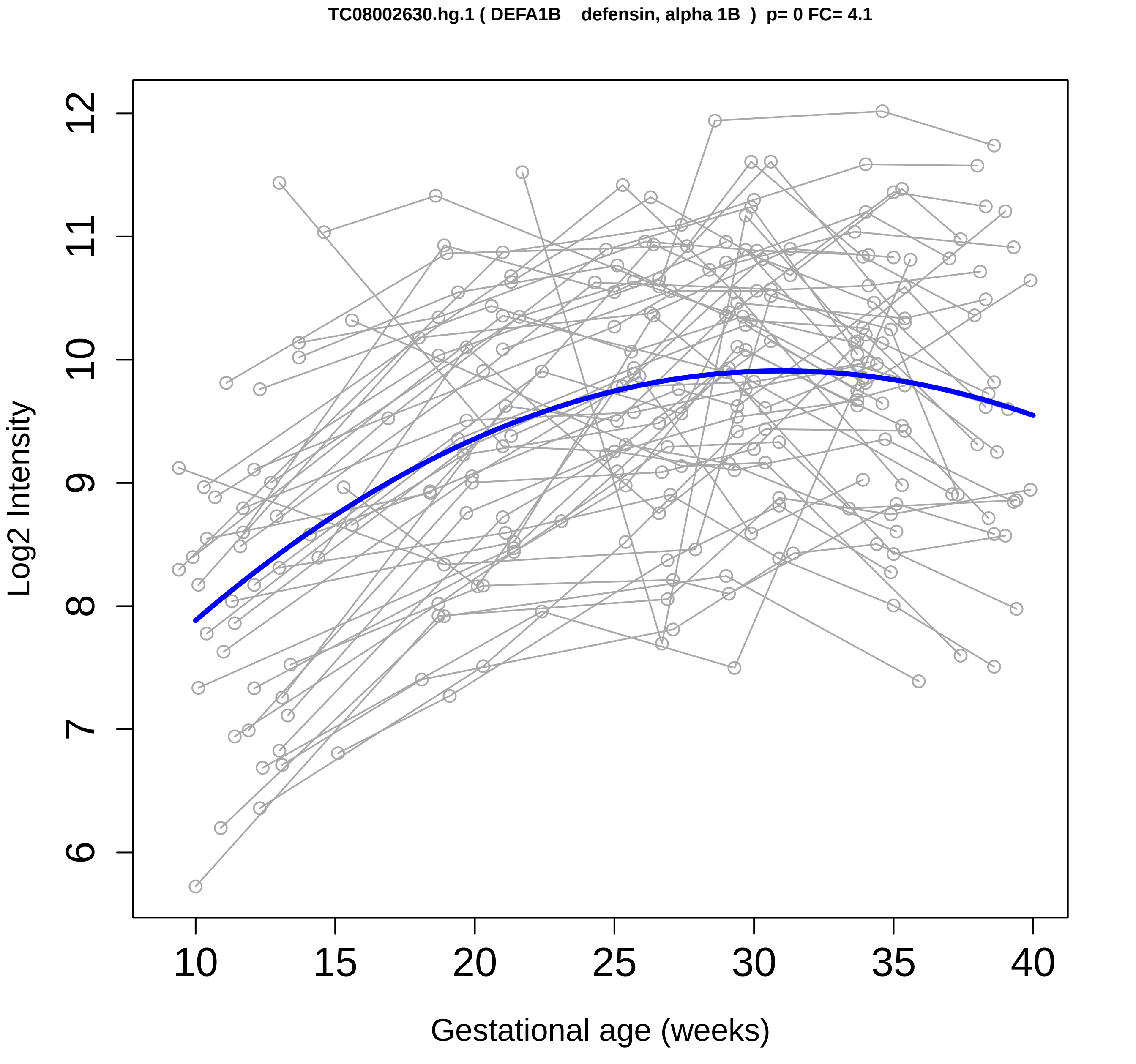
TC08002630.hg.1 (DEFA1B defensin, alpha 1B) p= 0 FC= 4.1
Log2 Intensity
6
7
8
9
10
11
12
10
15
20
25
30
35
40
Gestational age (weeks)

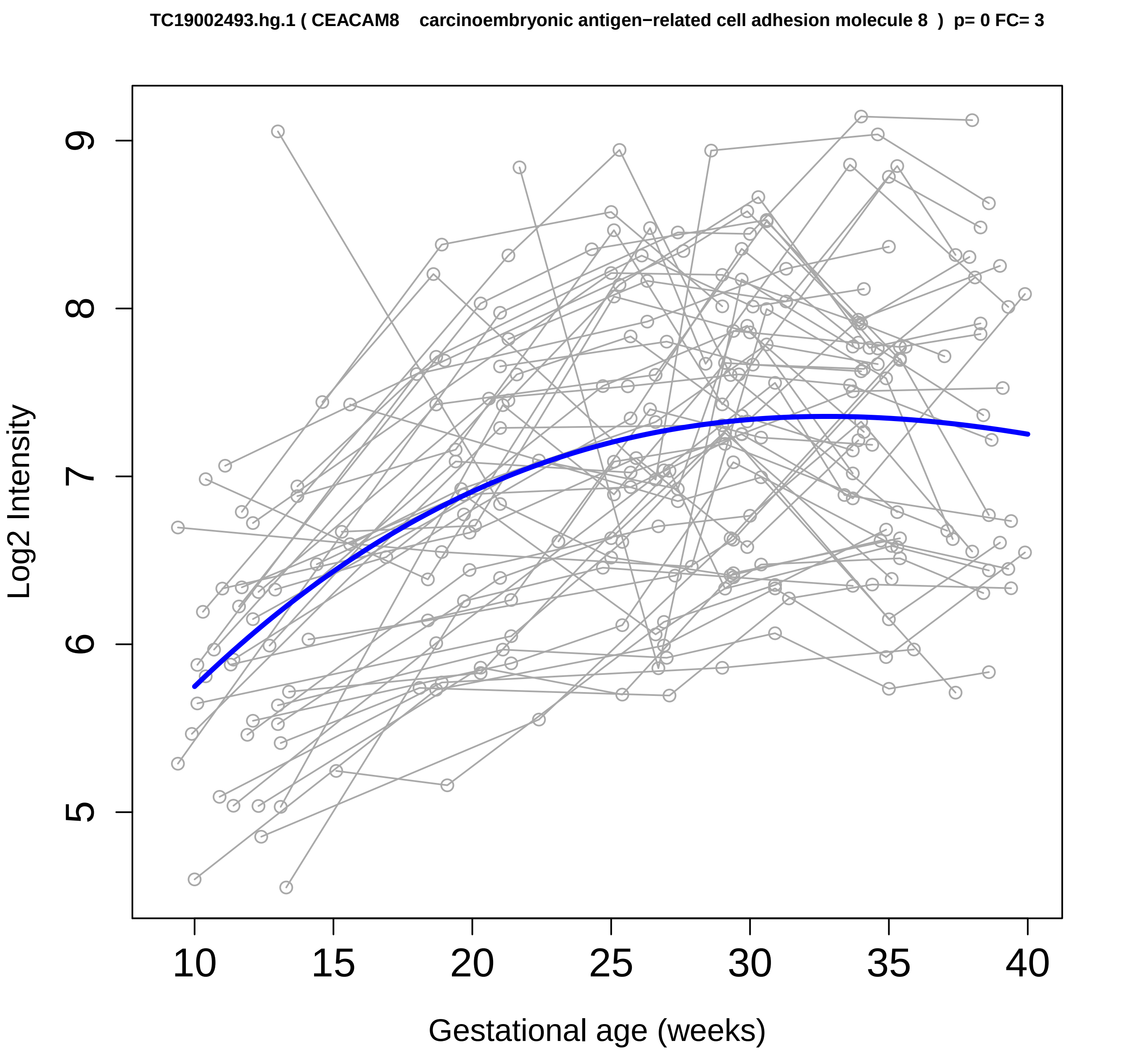

TC19002493.hg.1 (CEACAM8 carcinoembryonic antigen−related cell adhesion molecule 8) p= 0 FC= 3
Log2 Intensity
5
6
7
8
9
10
15
20
25
30
35
40
Gestational age (weeks)

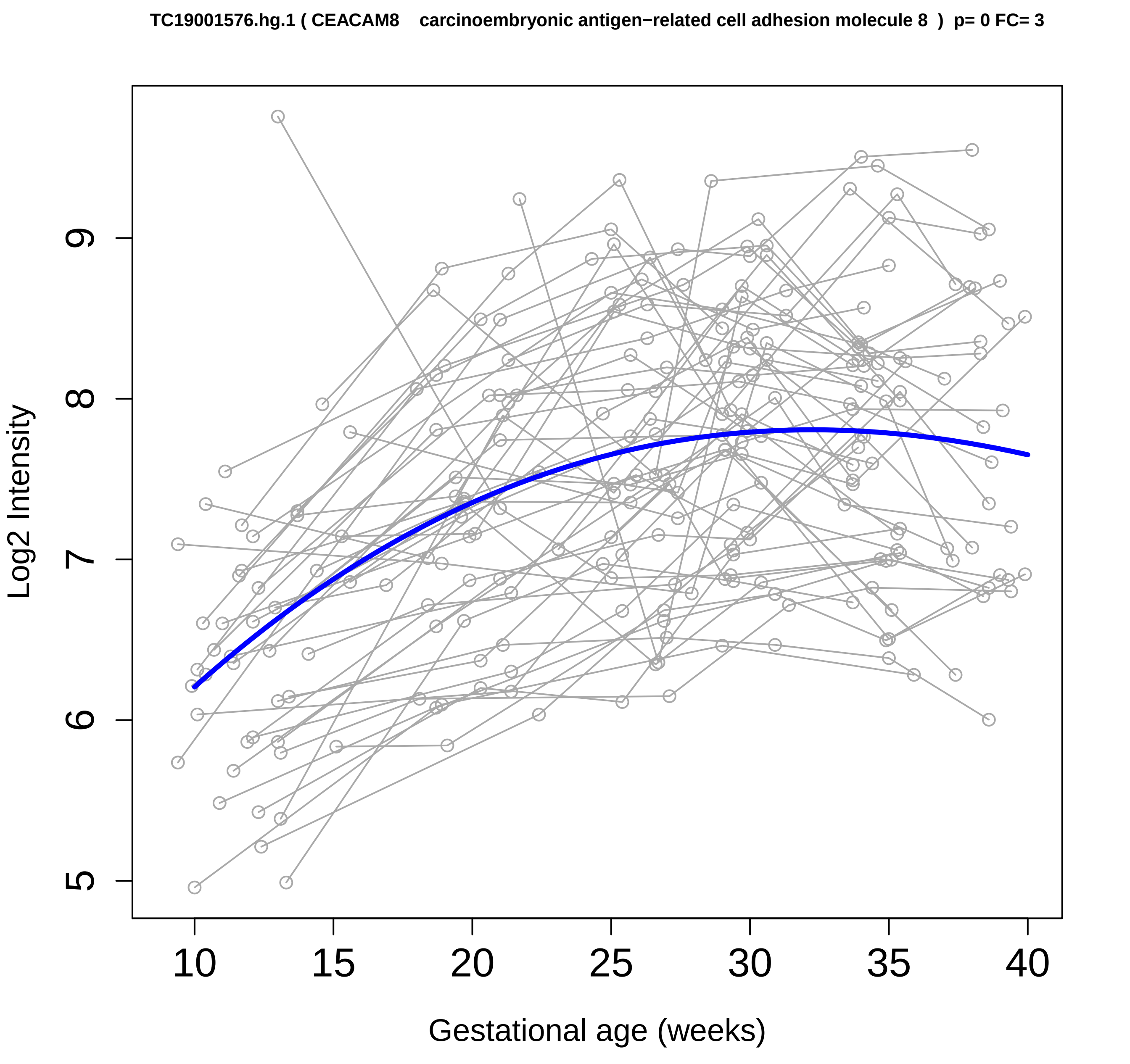

TC19001576.hg.1 (CEACAM8 carcinoembryonic antigen−related cell adhesion molecule 8) p= 0 FC= 3
Log2 Intensity
5
6
7
8
9
10
15
20
25
30
35
40
Gestational age (weeks)

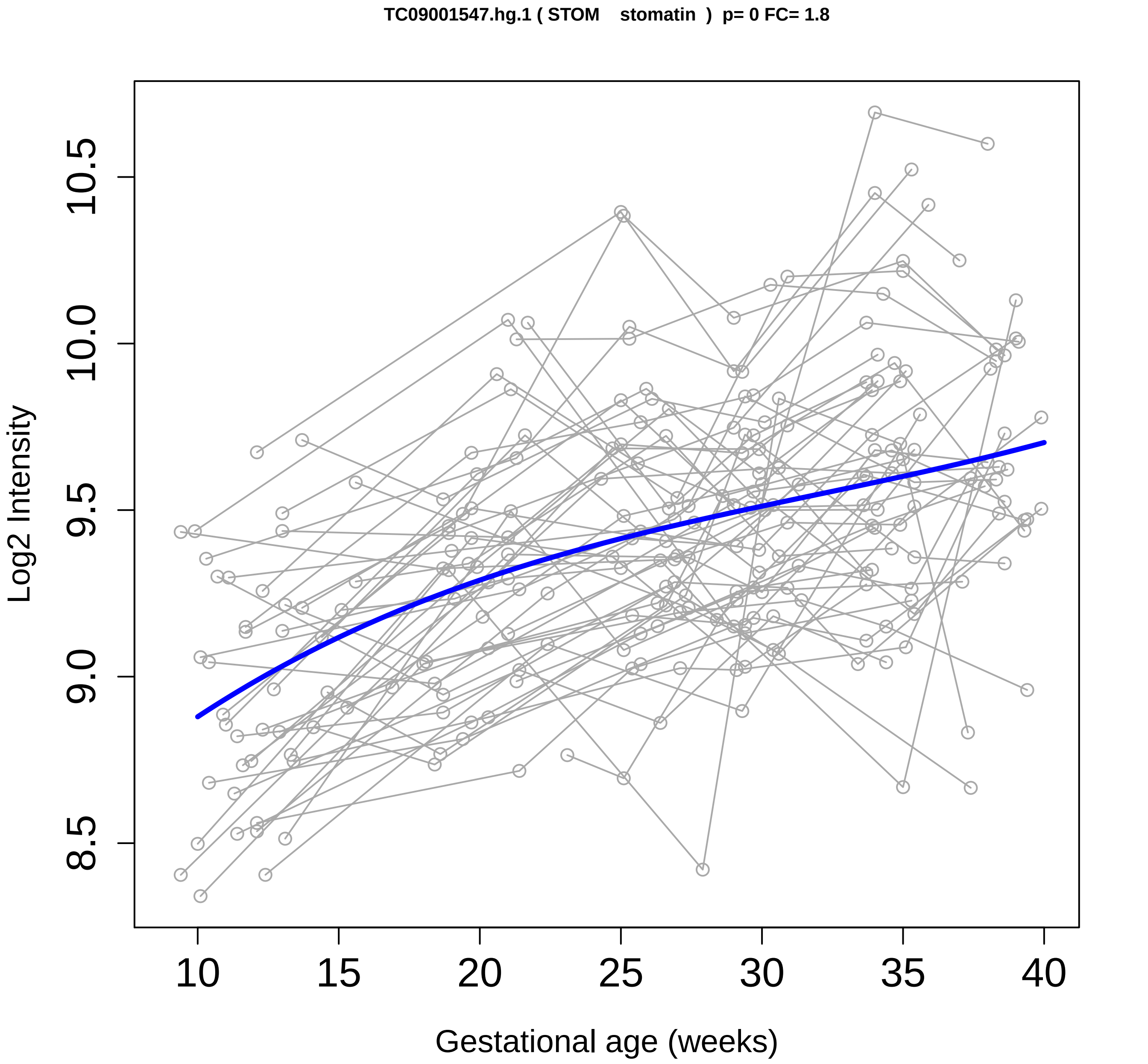

TC09001547.hg.1 (STOM stomatin) p= 0 FC= 1.8
Log2 Intensity
8.5
9.0
9.5
10.0
10.5
10
15
20
25
30
35
40
Gestational age (weeks)

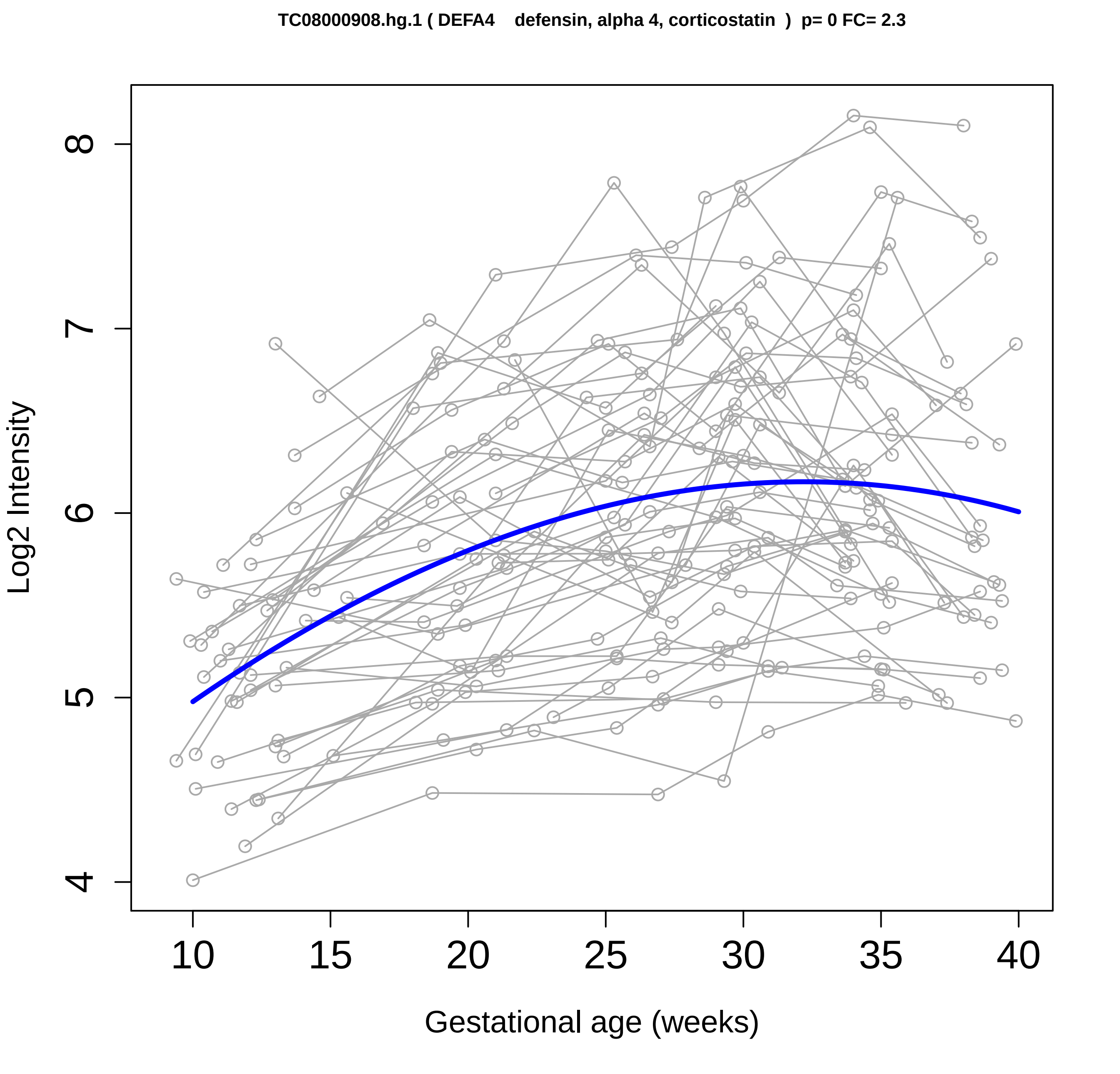
TC08000908.hg.1 (DEFA4 defensin, alpha 4, corticostatin) p= 0 FC= 2.3
Log2 Intensity
4
5
6
7
8
10
15
20
25
30
35
40
Gestational age (weeks)

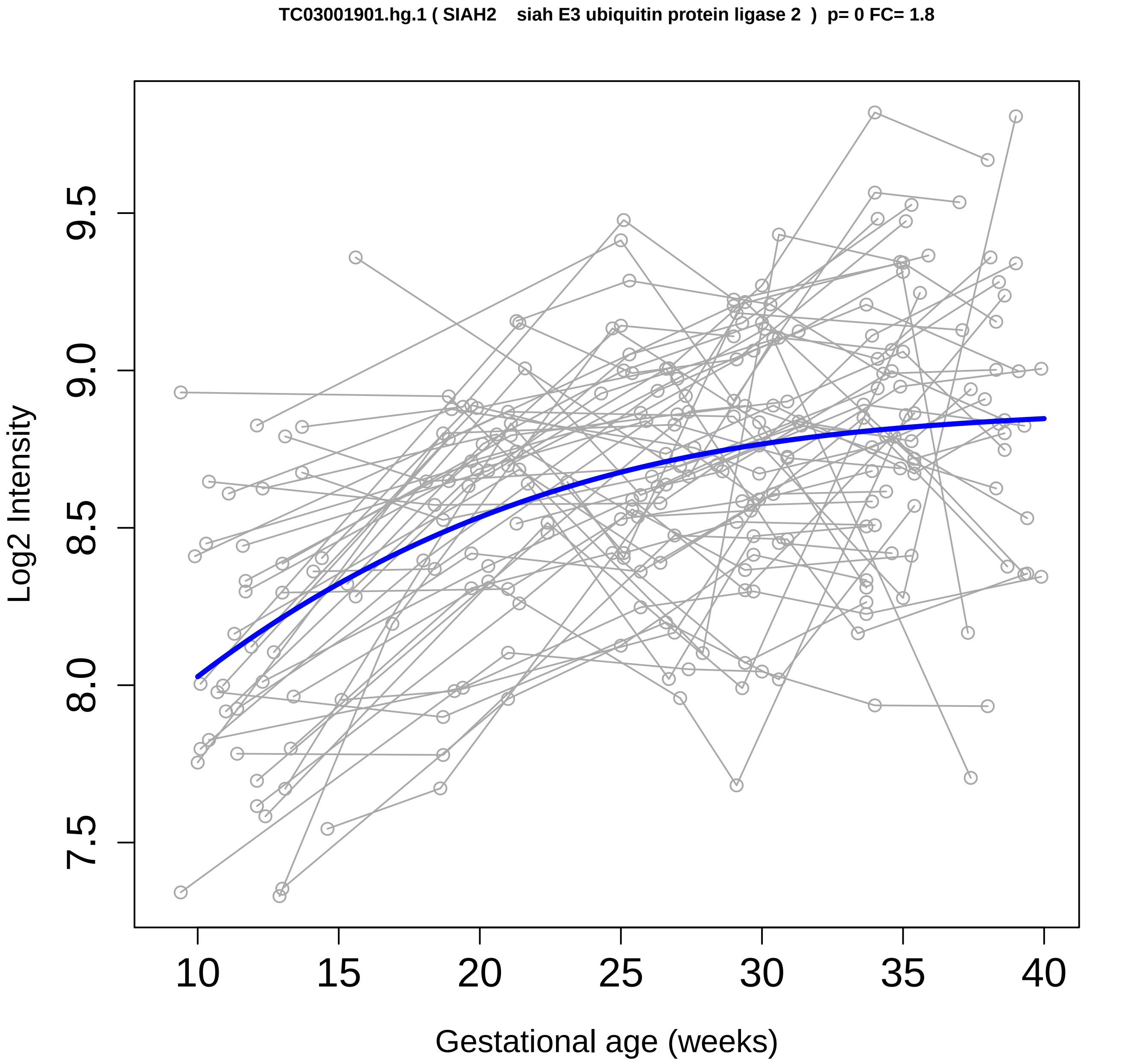
TC03001901.hg.1 (SIAH2 siah E3 ubiquitin protein ligase 2) p= 0 FC= 1.8
Log2 Intensity
7.5
8.0
8.5
9.0
9.5
10
15
20
25
30
35
40
Gestational age (weeks)

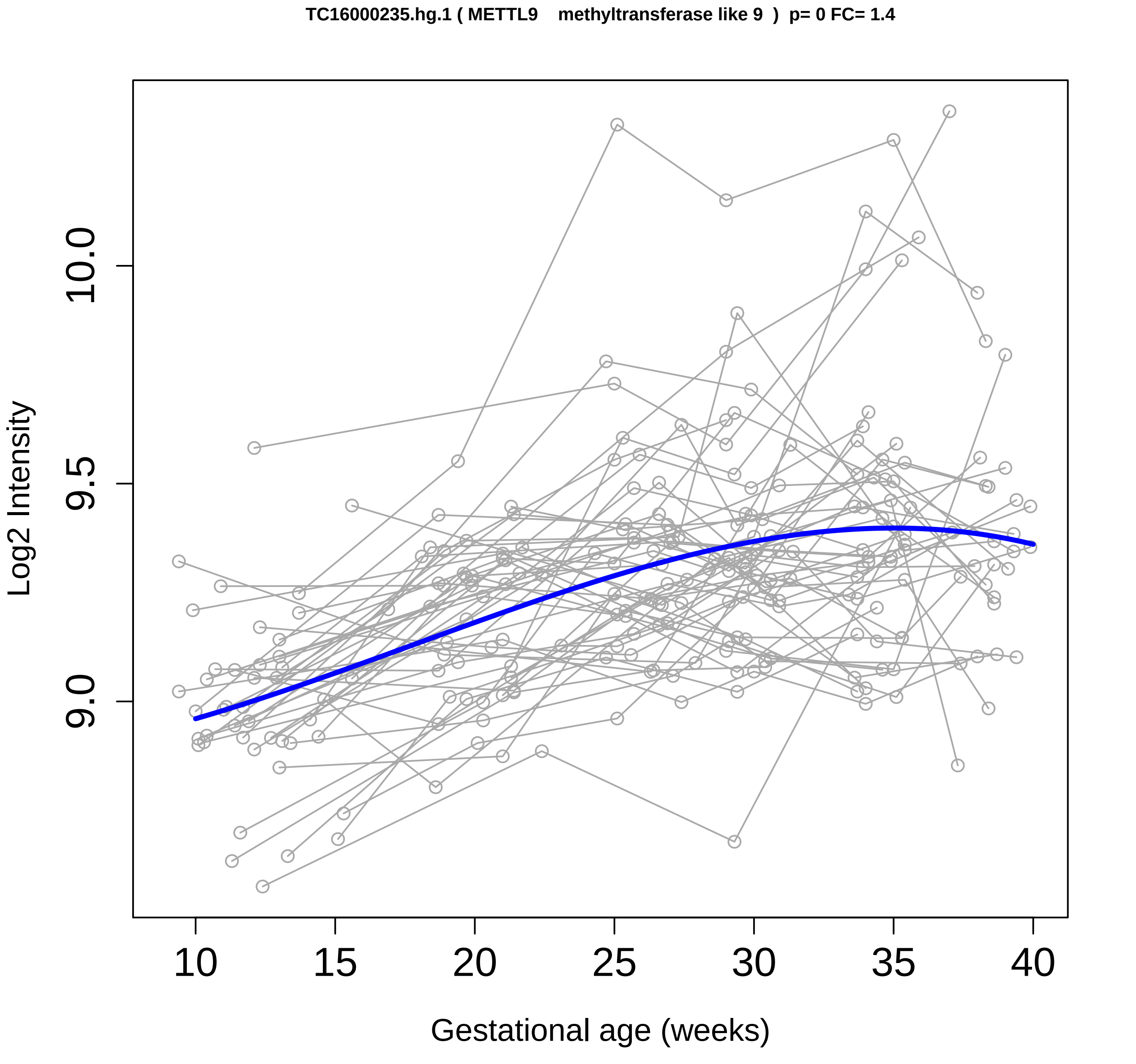
TC16000235.hg.1 (METTL9 methyltransferase like 9) p= 0 FC= 1.4
Log2 Intensity
9.0
9.5
10.0
10
15
20
25
30
35
40
Gestational age (weeks)

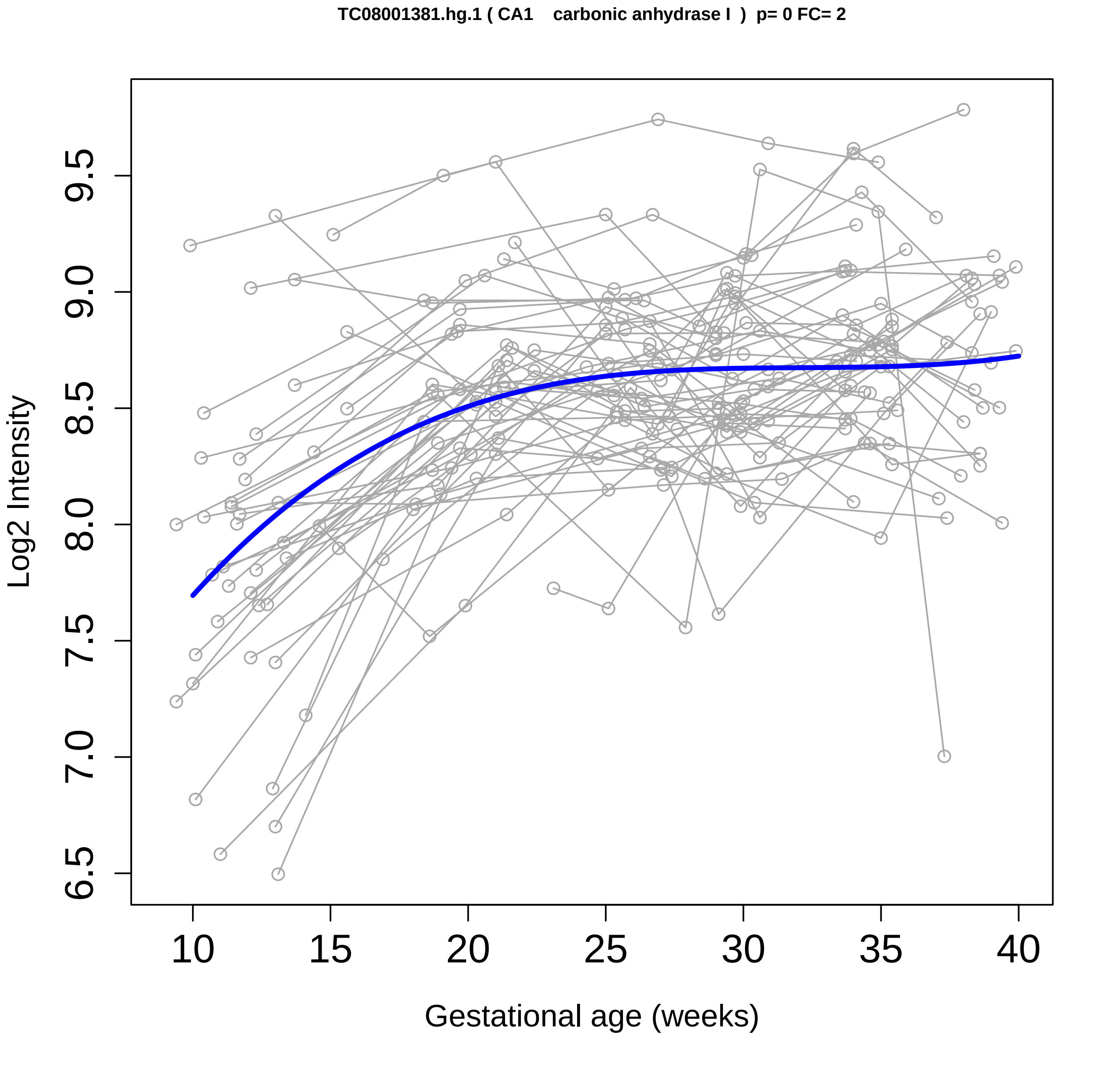

TC08001381.hg.1 (CA1 carbonic anhydrase I) p= 0 FC= 2
Log2 Intensity
6.5
7.0
7.5
8.0
8.5
9.0
9.5
10
15
20
25
30
35
40
Gestational age (weeks)

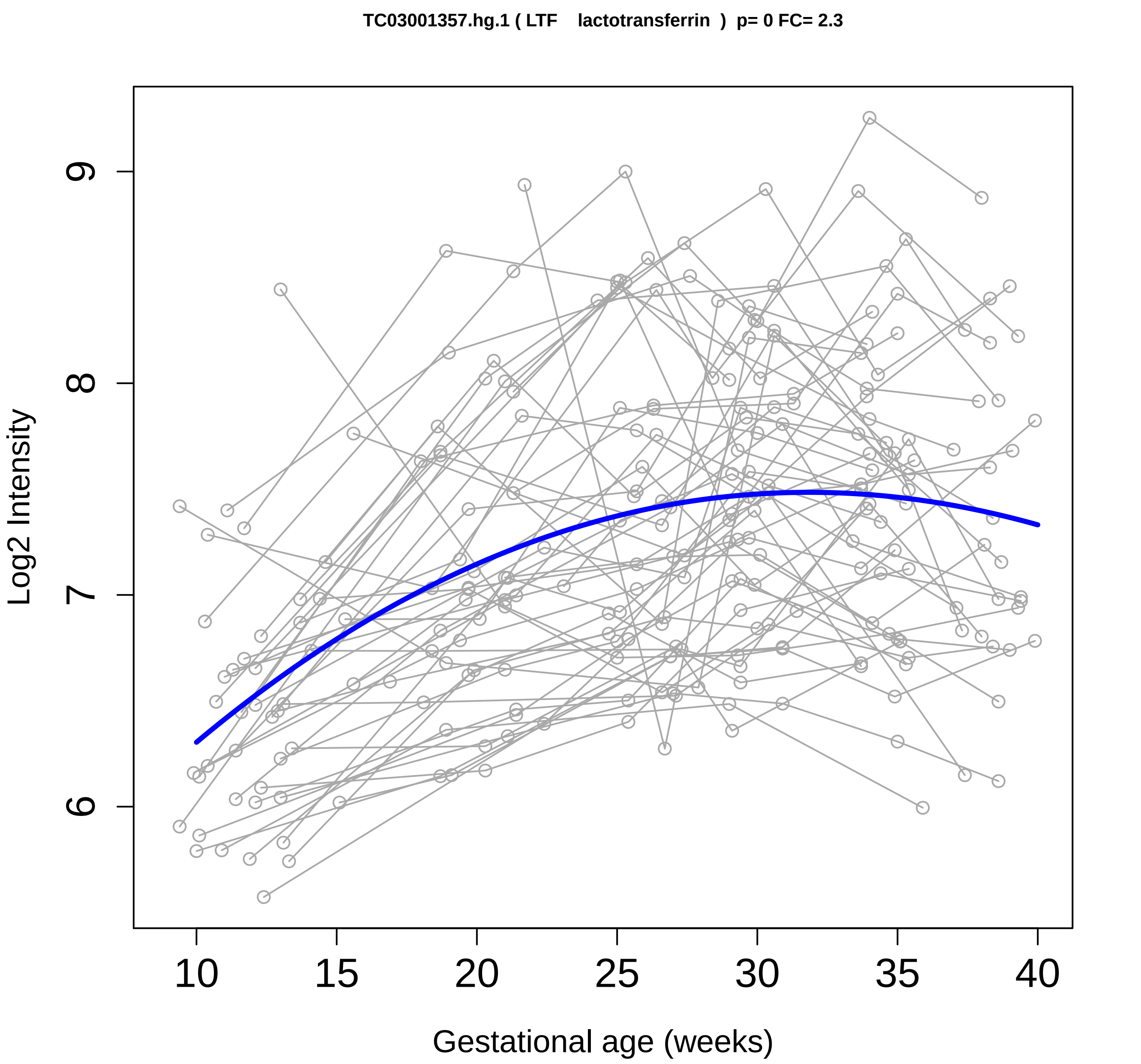

TC03001357.hg.1 (LTF   lactotransferrin) p= 0 FC= 2.3
Log2 Intensity
6
7
8
9
10
15
20
25
30
35
40
Gestational age (weeks)

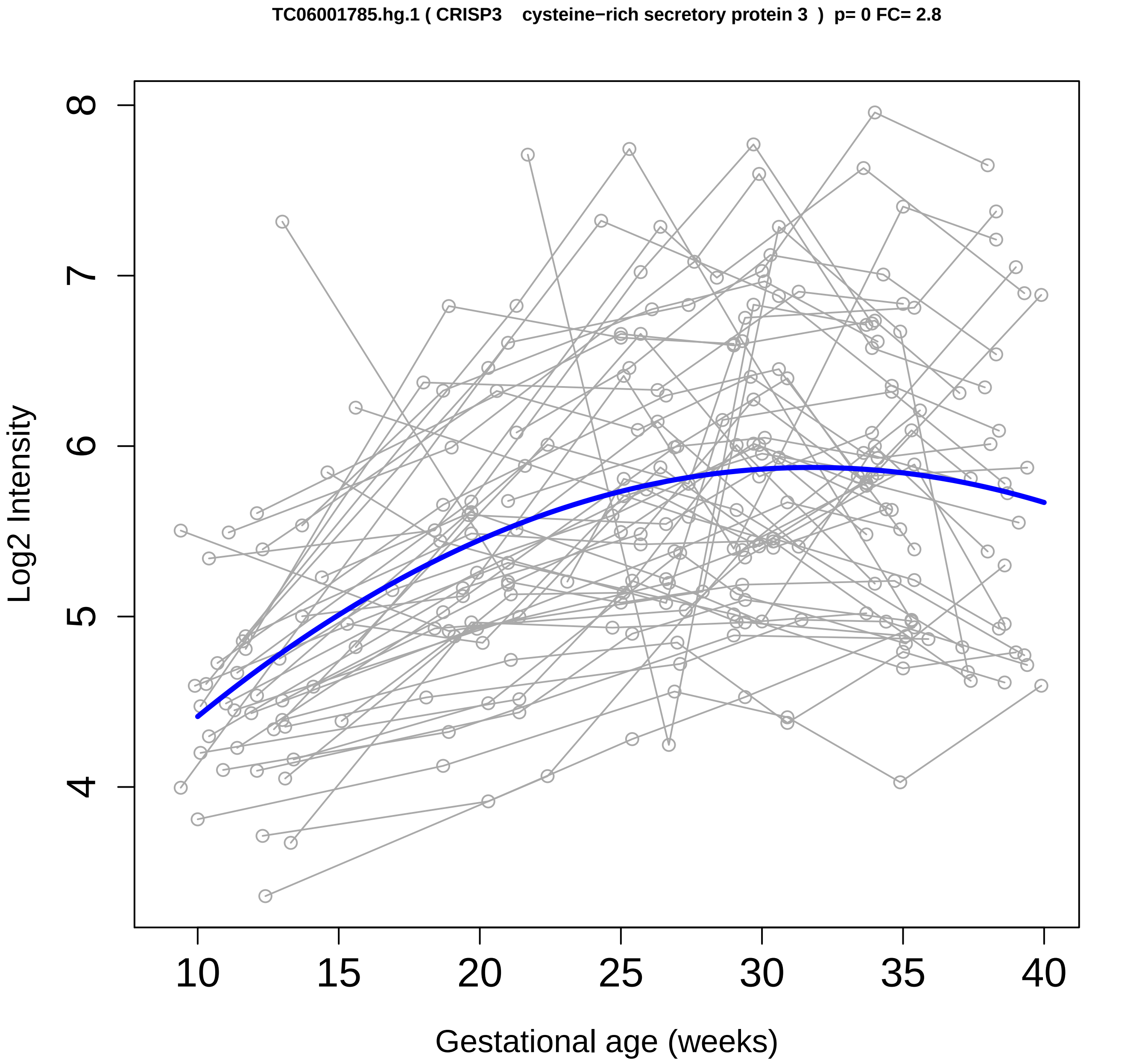

TC06001785.hg.1 (CRISP3 cysteine−rich secretory protein 3) p= 0 FC= 2.8
Log2 Intensity
8
7
6
5
4
10
15
20
25
30
35
40
Gestational age (weeks)

TC08002237.hg.1 (MSR1 macrophage scavenger receptor 1) p= 0 FC= 1.5

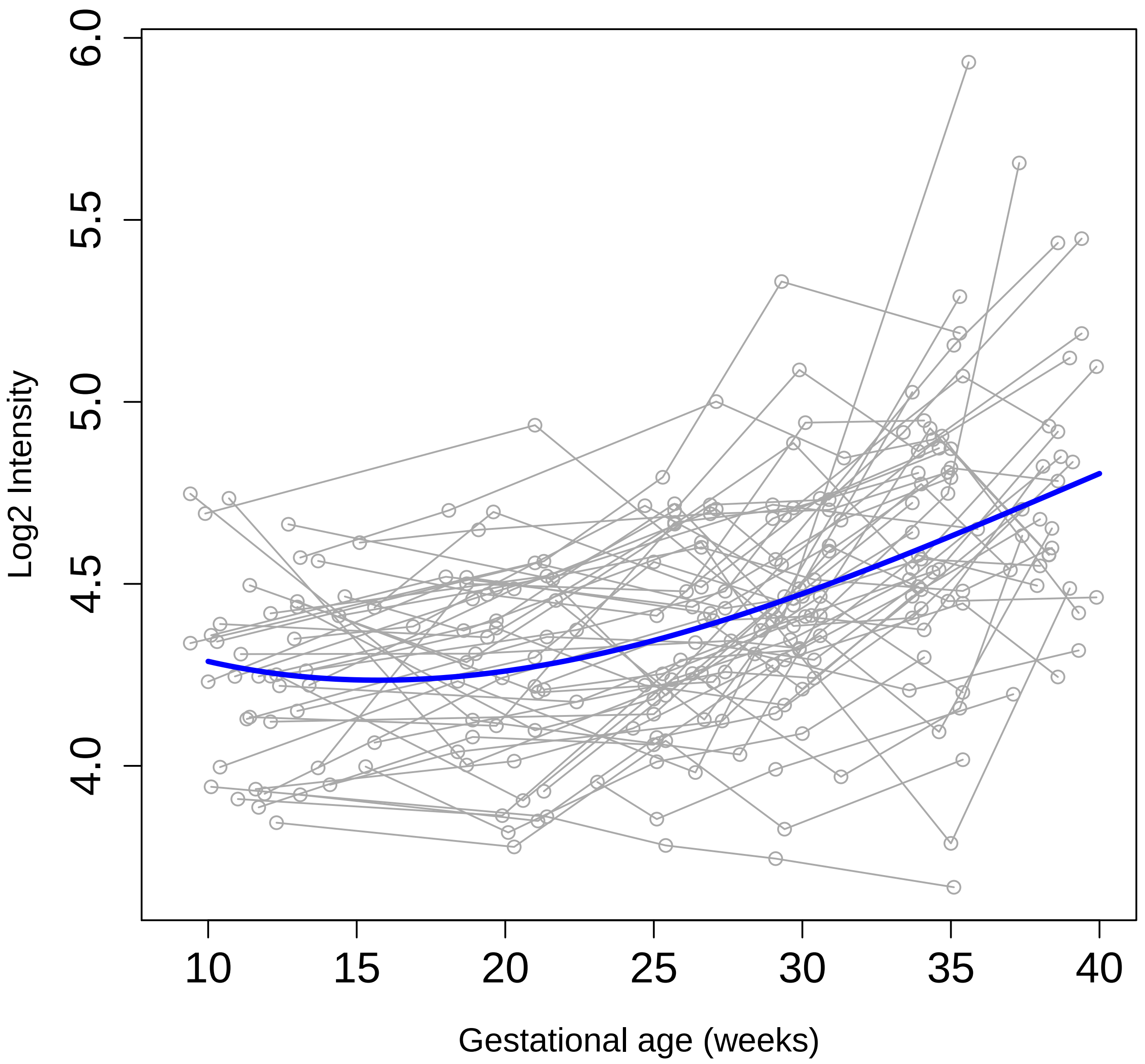

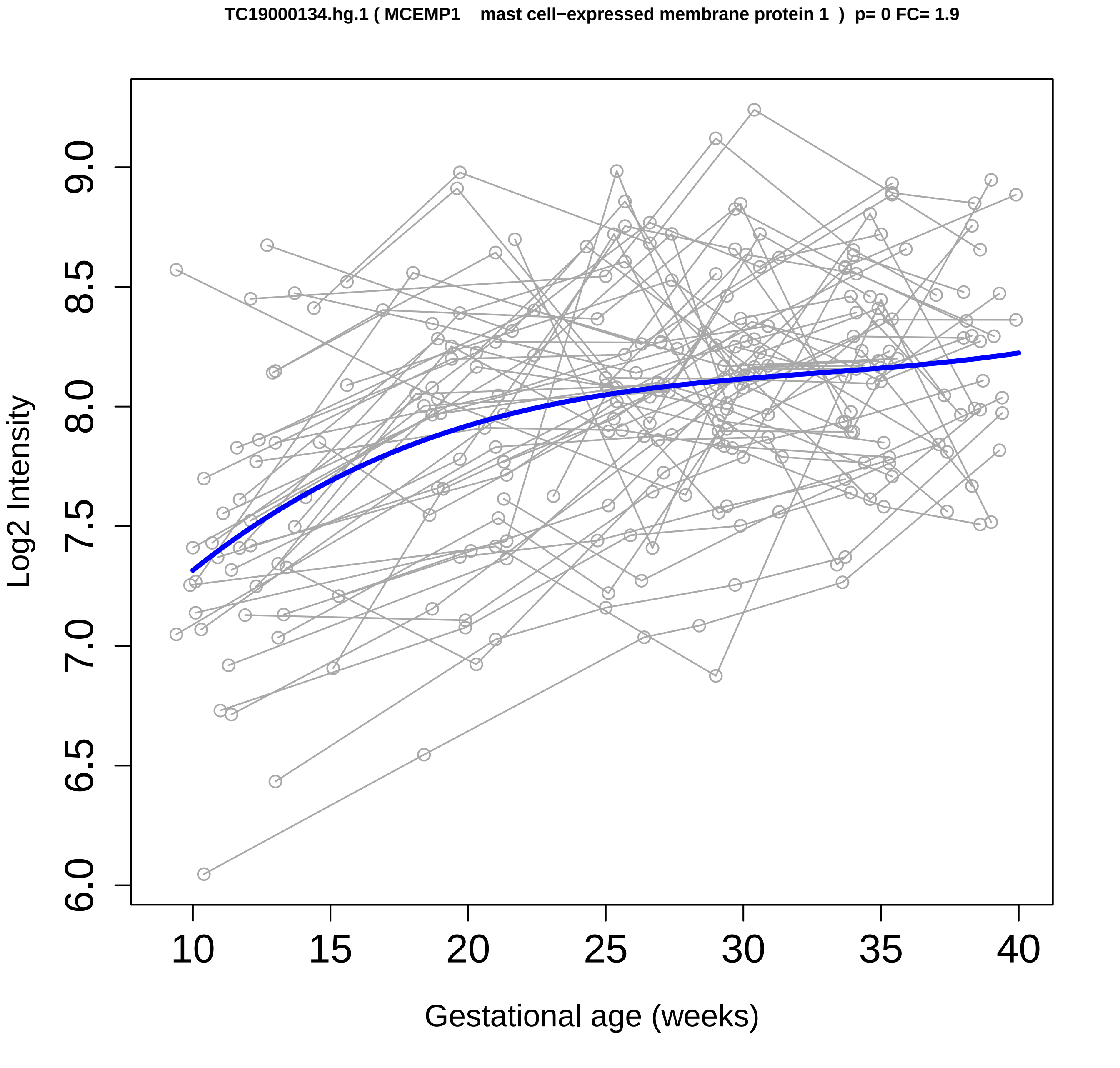

TC19000134.hg.1 (MCEMP1   mast cell−expressed membrane protein 1) p= 0 FC= 1.9
Log2 Intensity
6.0
6.5
7.0
7.5
8.0
8.5
9.0
10
15
20
25
30
35
40
Gestational age (weeks)

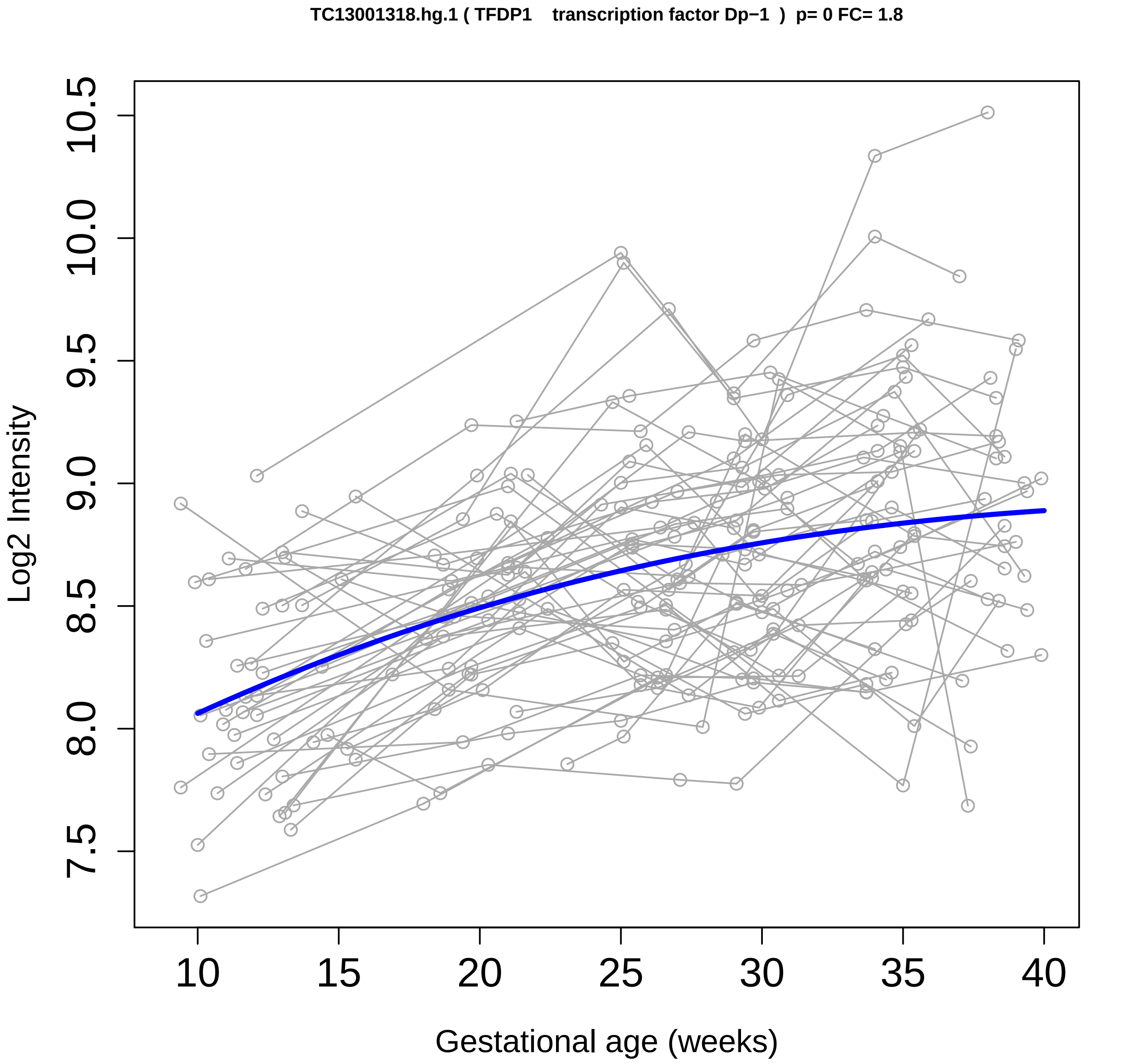
TC13001318.hg.1 (TFDP1 transcription factor Dp−1) p= 0 FC= 1.8
Log2 Intensity
7.5
8.0
8.5
9.0
9.5
10.0
10.5
10
15
20
25
30
35
40
Gestational age (weeks)

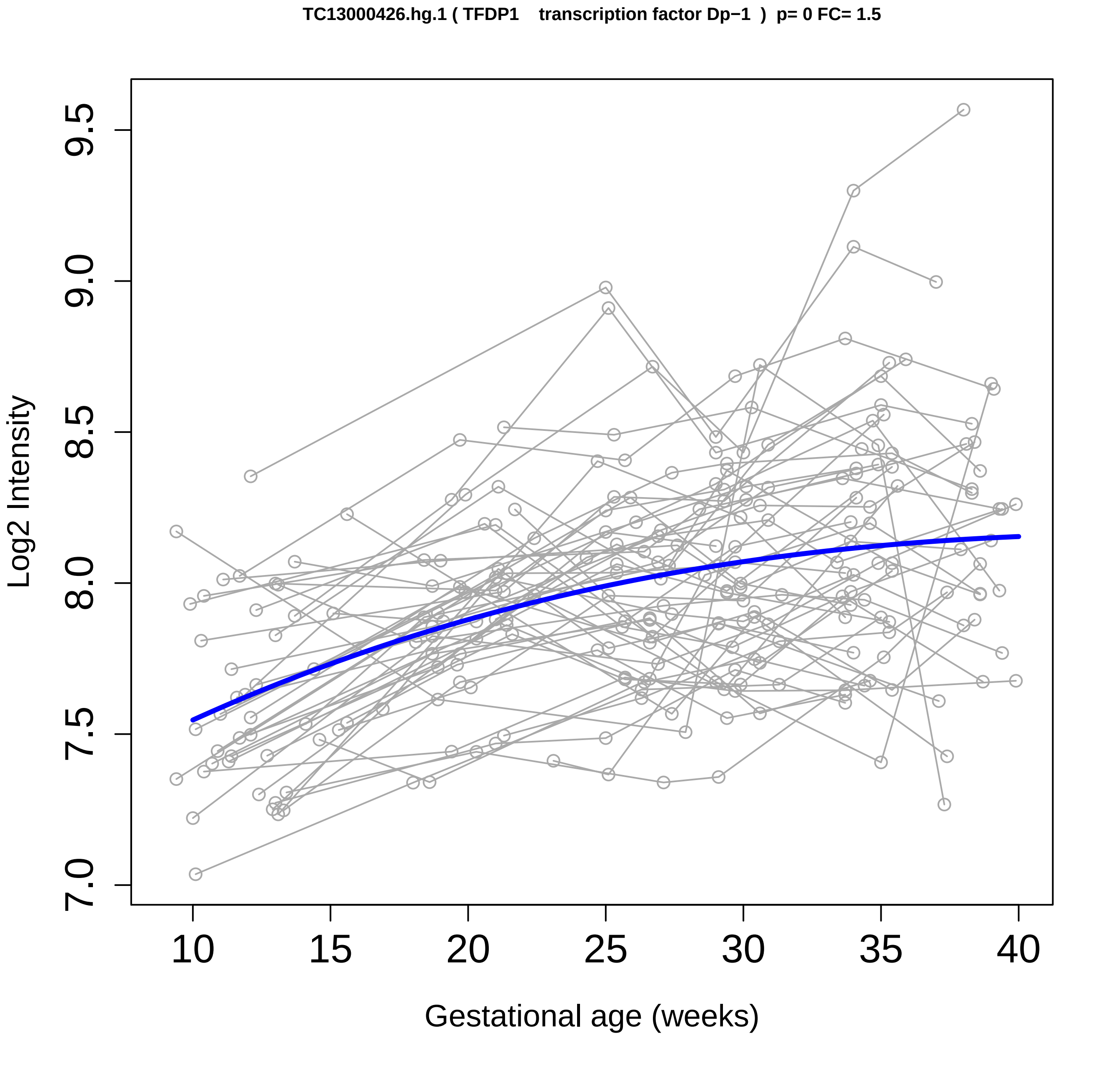
TC13000426.hg.1 (TFDP1 transcription factor Dp−1) p= 0 FC= 1.5
Log2 Intensity
7.0
7.5
8.0
8.5
9.0
9.5
10
15
20
25
30
35
40
Gestational age (weeks)

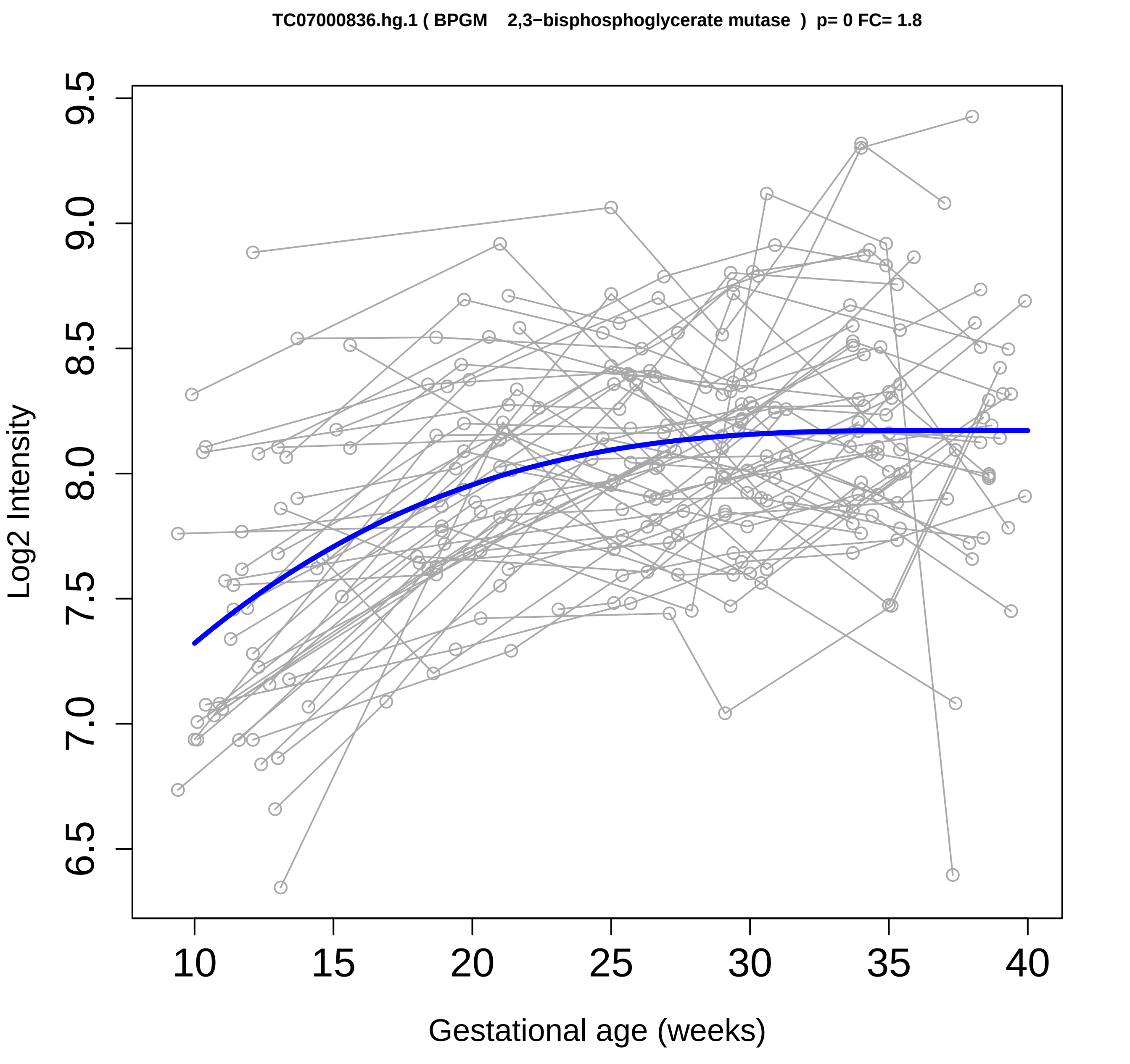
TC07000836.hg.1 (BPGM 2,3−bisphosphoglycerate mutase) p= 0 FC= 1.8
Log2 Intensity
6.5
7.0
7.5
8.0
8.5
9.0
9.5
10
15
20
25
30
35
40
Gestational age (weeks)

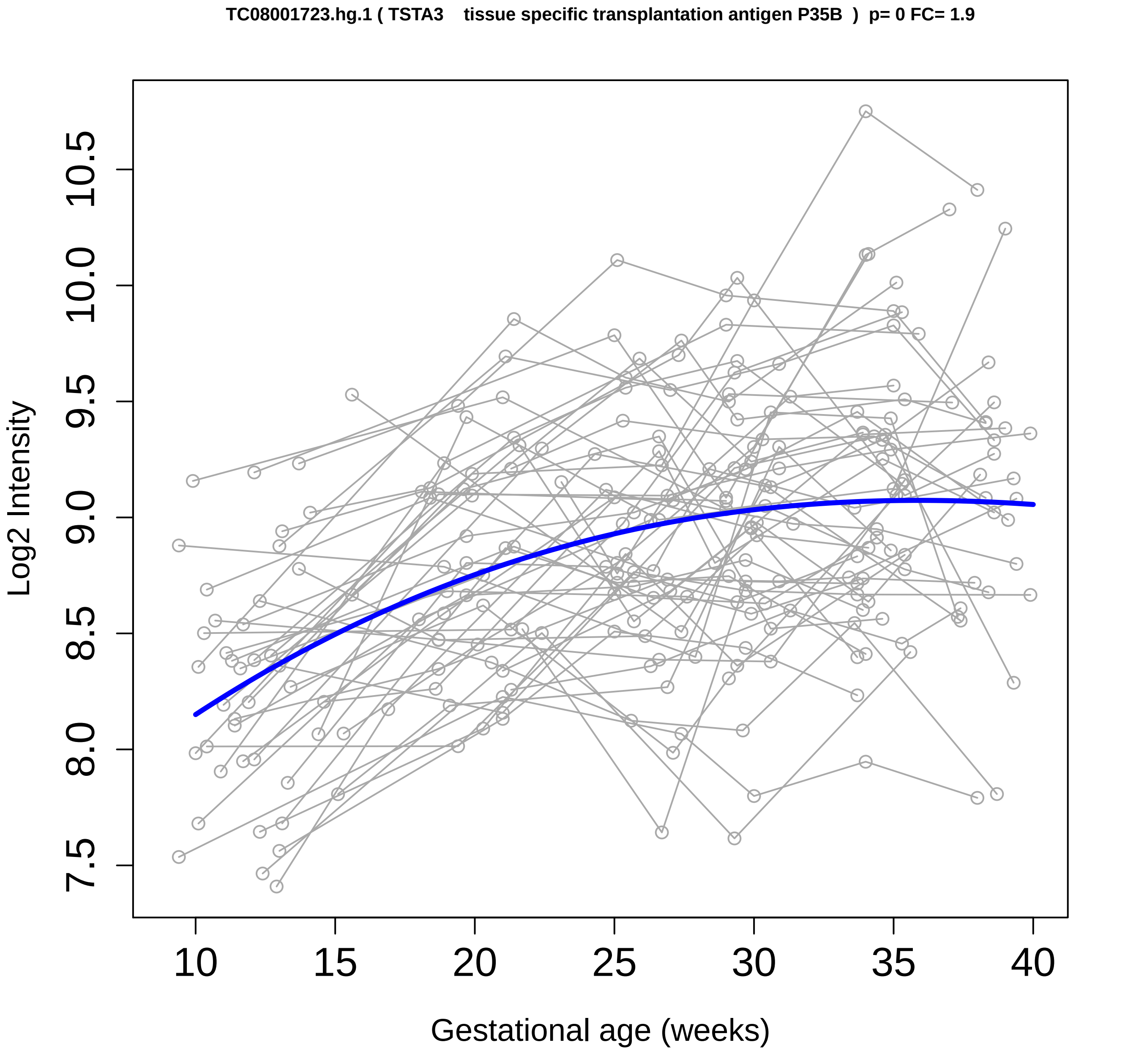
TC08001723.hg.1 (TSTA3 tissue specific transplantation antigen P35B) p= 0 FC= 1.9
Log2 Intensity
7.5
8.0
8.5
9.0
9.5
10.0
10.5
10
15
20
25
30
35
40
Gestational age (weeks)

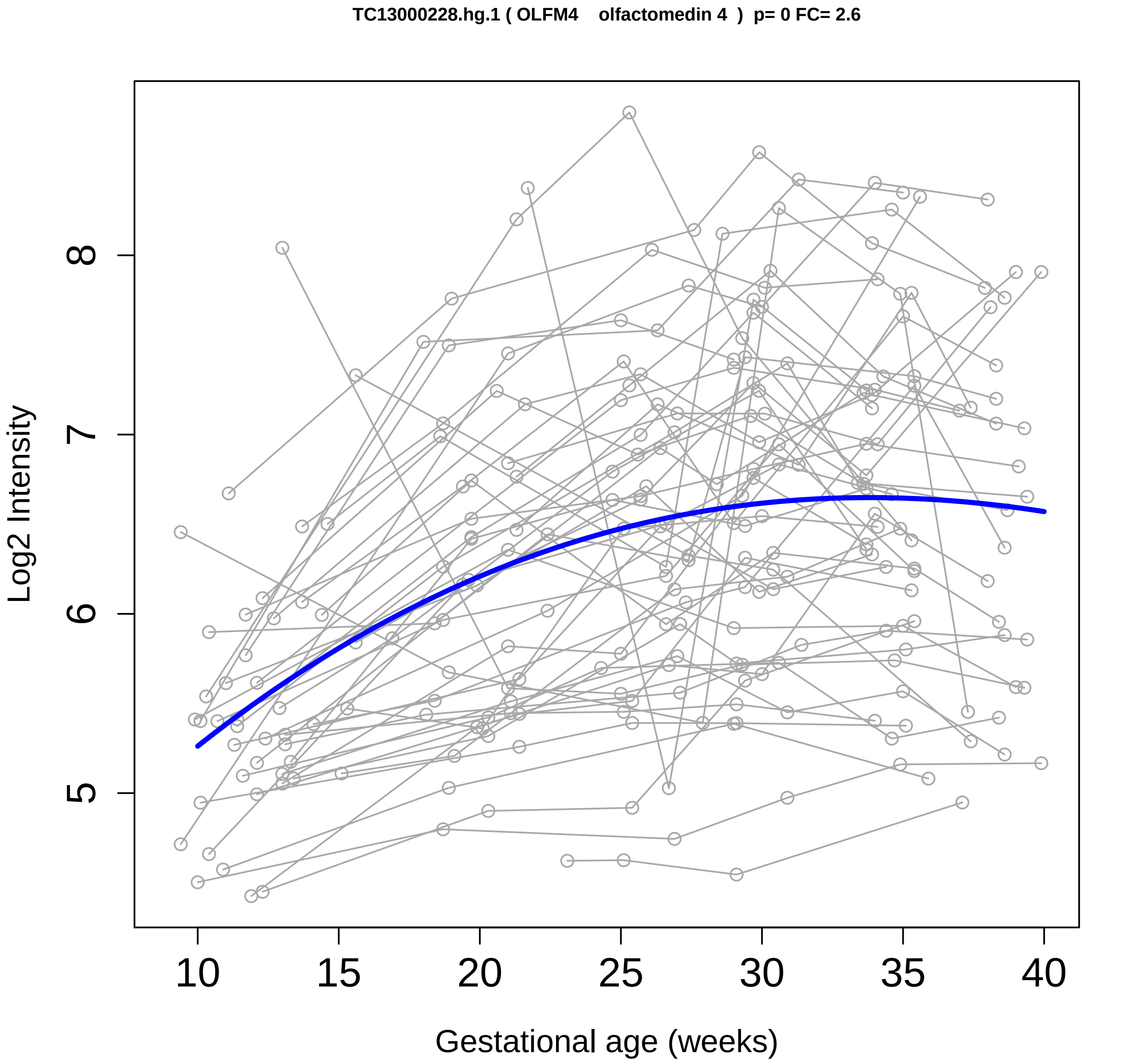

TC13000228.hg.1 (OLFM4 olfactomedin 4) p= 0 FC= 2.6
Log2 Intensity
5
6
7
8
10
15
20
25
30
35
40
Gestational age (weeks)

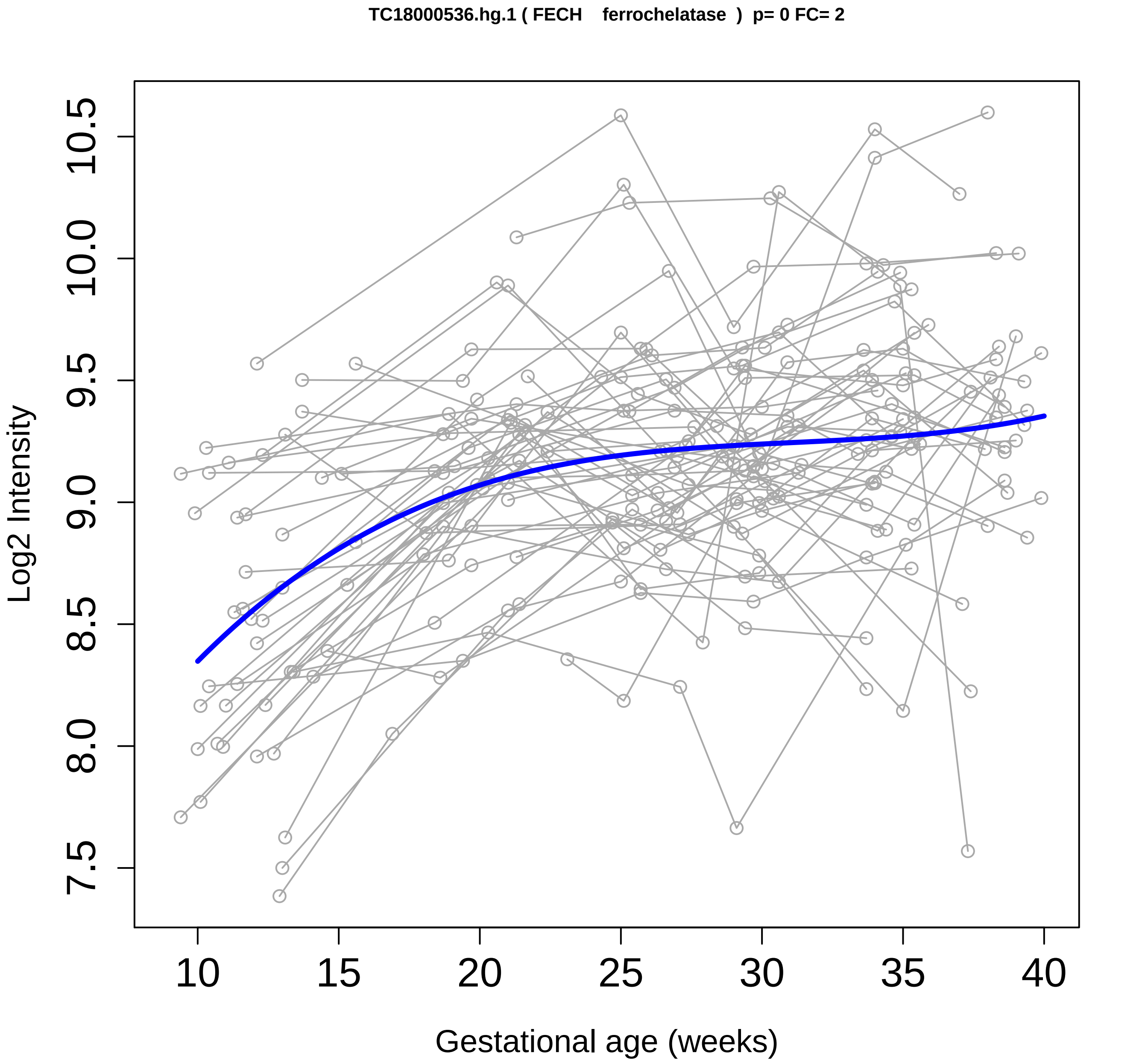
TC18000536.hg.1 (FECH ferrochelatase) p= 0 FC= 2
Log2 Intensity
7.5
8.0
8.5
9.0
9.5
10.0
10.5
10
15
20
25
30
35
40
Gestational age (weeks)

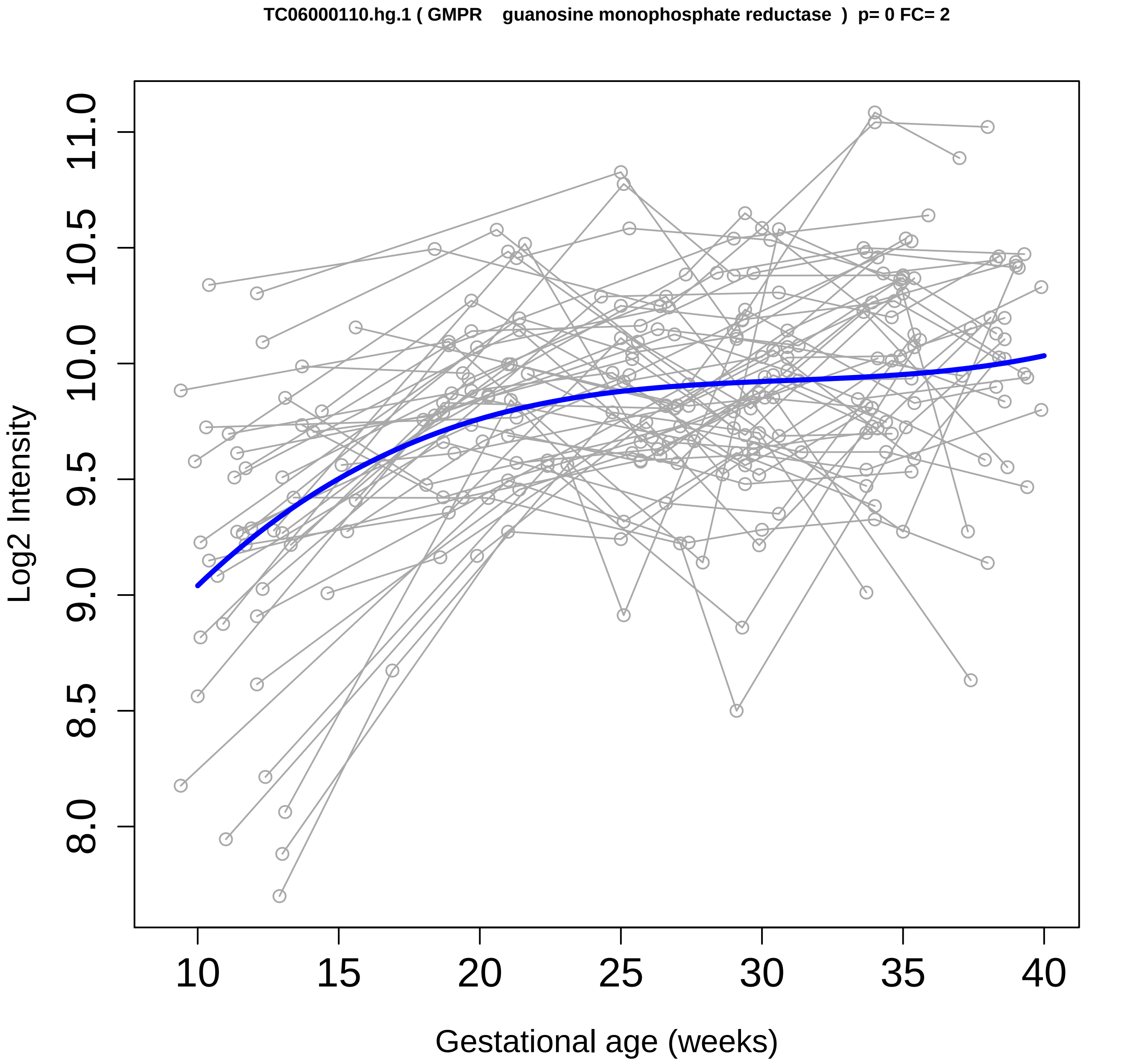

TC06000110.hg.1 (GMPR guanosine monophosphate reductase) p= 0 FC= 2
Log2 Intensity
8.0
8.5
9.0
9.5
10.0
10.5
11.0
10
15
20
25
30
35
40
Gestational age (weeks)

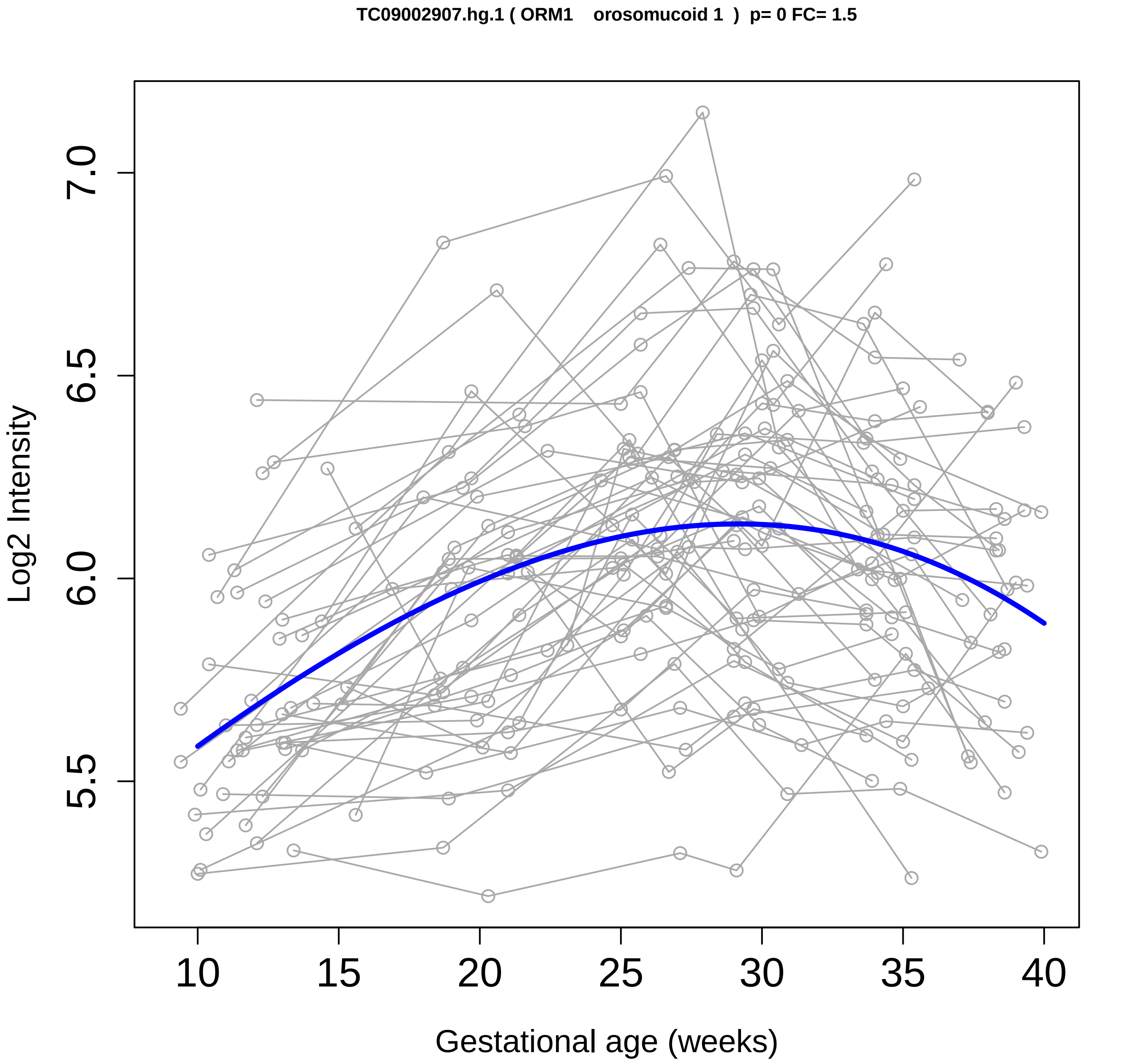
TC09002907.hg.1 (ORM1 orosomucoid 1) p= 0 FC= 1.5
Log2 Intensity
5.5
6.0
6.5
7.0
10
15
20
25
30
35
40
Gestational age (weeks)

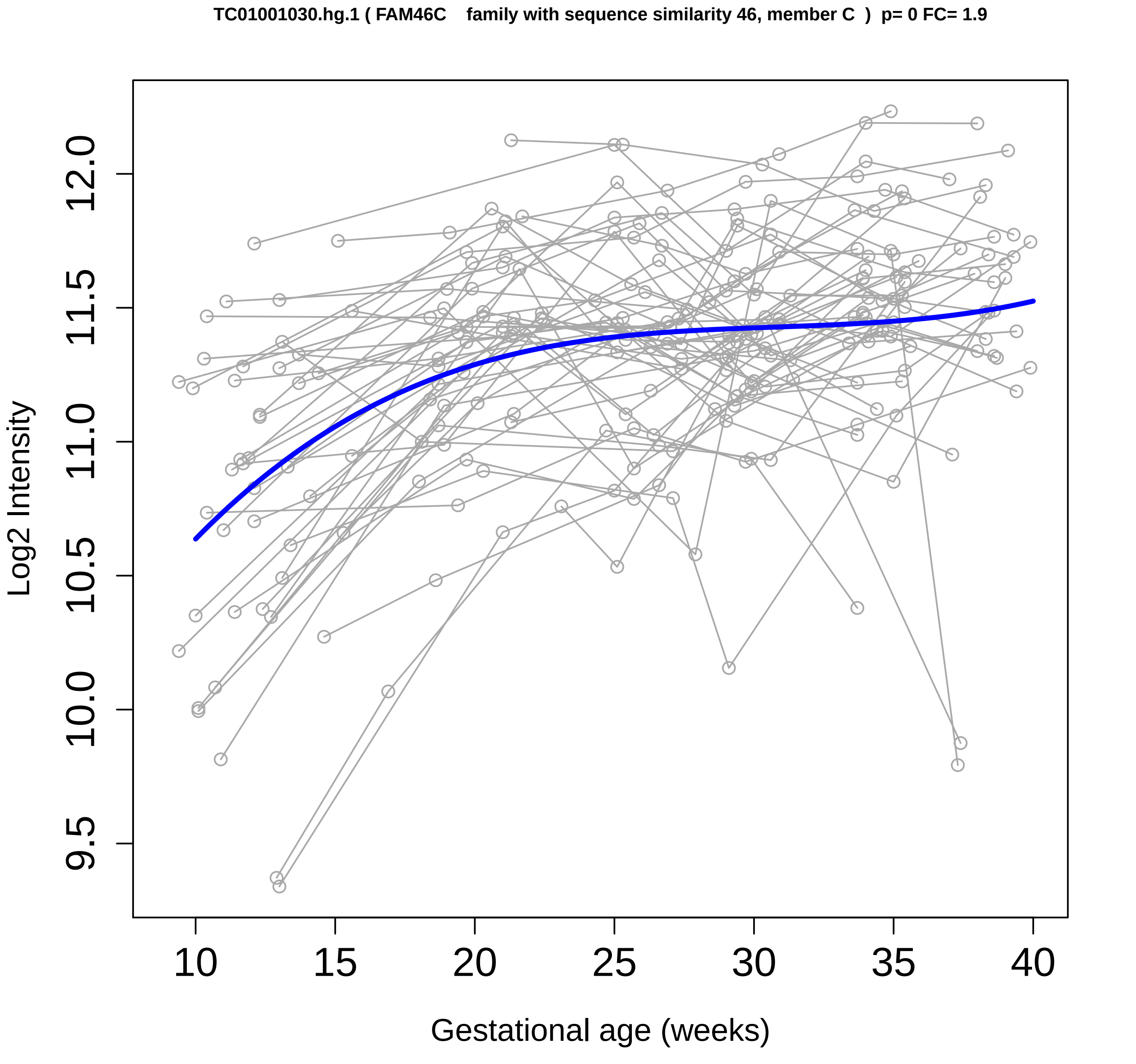

TC01001030.hg.1 (FAM46C family with sequence similarity 46, member C) p= 0 FC= 1.9
Log2 Intensity
9.5
10.0
10.5
11.0
11.5
12.0
10
15
20
25
30
35
40
Gestational age (weeks)

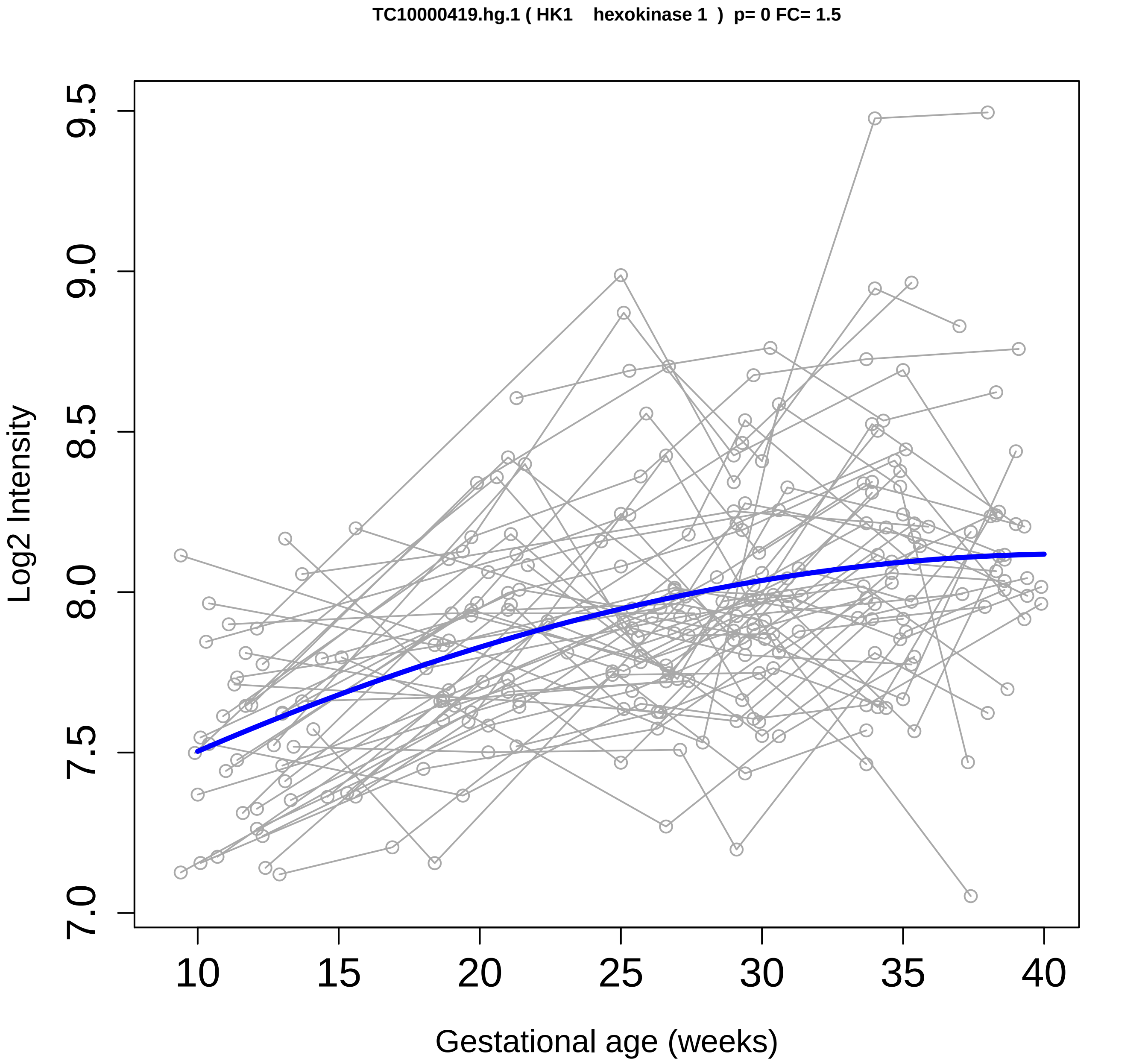
TC10000419.hg.1 (HK1 hexokinase 1) p= 0 FC= 1.5
Log2 Intensity
7.0
7.5
8.0
8.5
9.0
9.5
10
15
20
25
30
35
40
Gestational age (weeks)

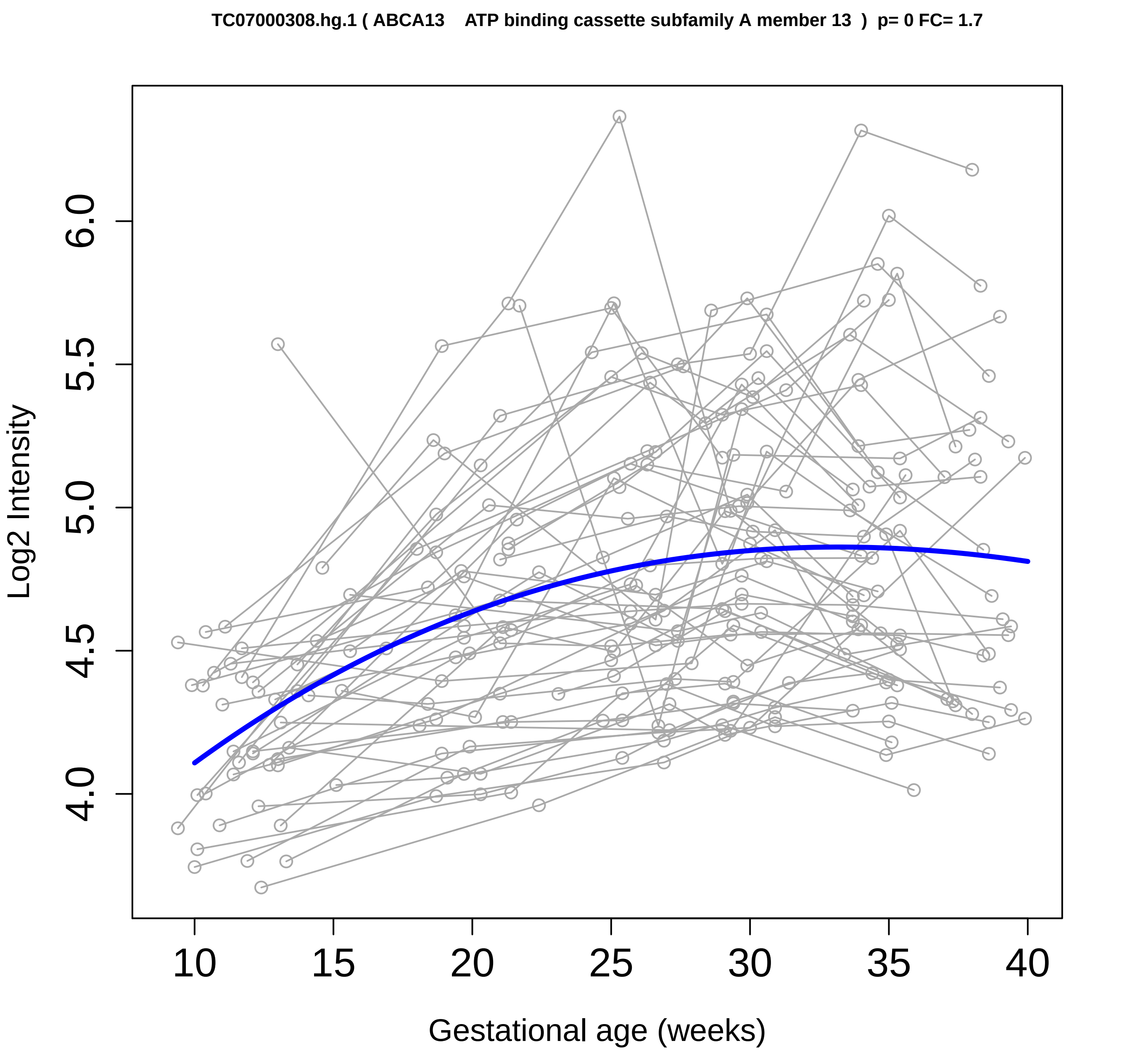
TC07000308.hg.1 (ABCA13   ATP binding cassette subfamily A member 13) p= 0 FC= 1.7
Log2 Intensity
4.0
4.5
5.0
5.5
6.0
10
15
20
25
30
35
40
Gestational age (weeks)

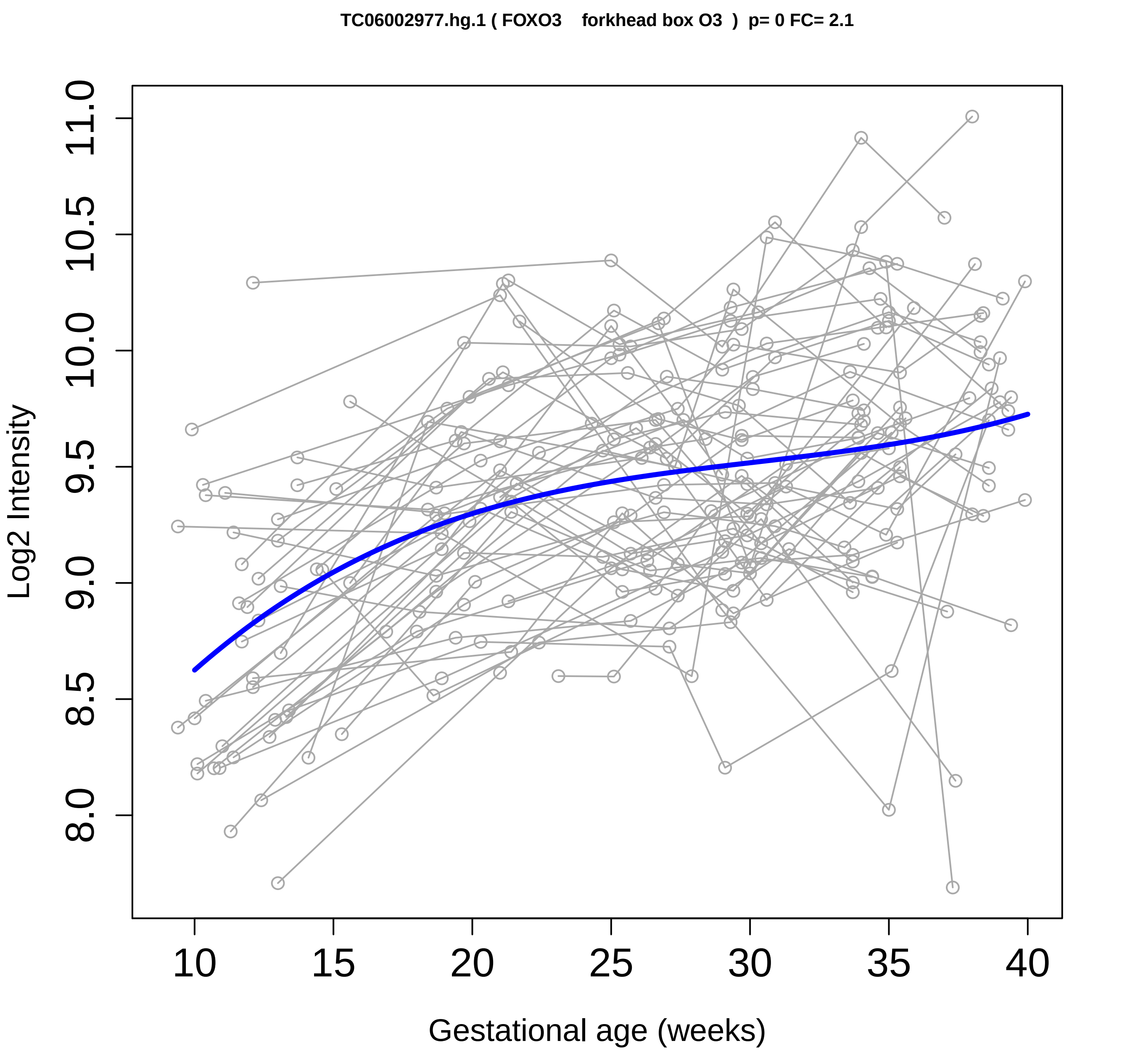
TC06002977.hg.1 (FOXO3   forkhead box O3) p= 0 FC= 2.1
Log2 Intensity
8.0
8.5
9.0
9.5
10.0
10.5
11.0
10
15
20
25
30
35
40
Gestational age (weeks)

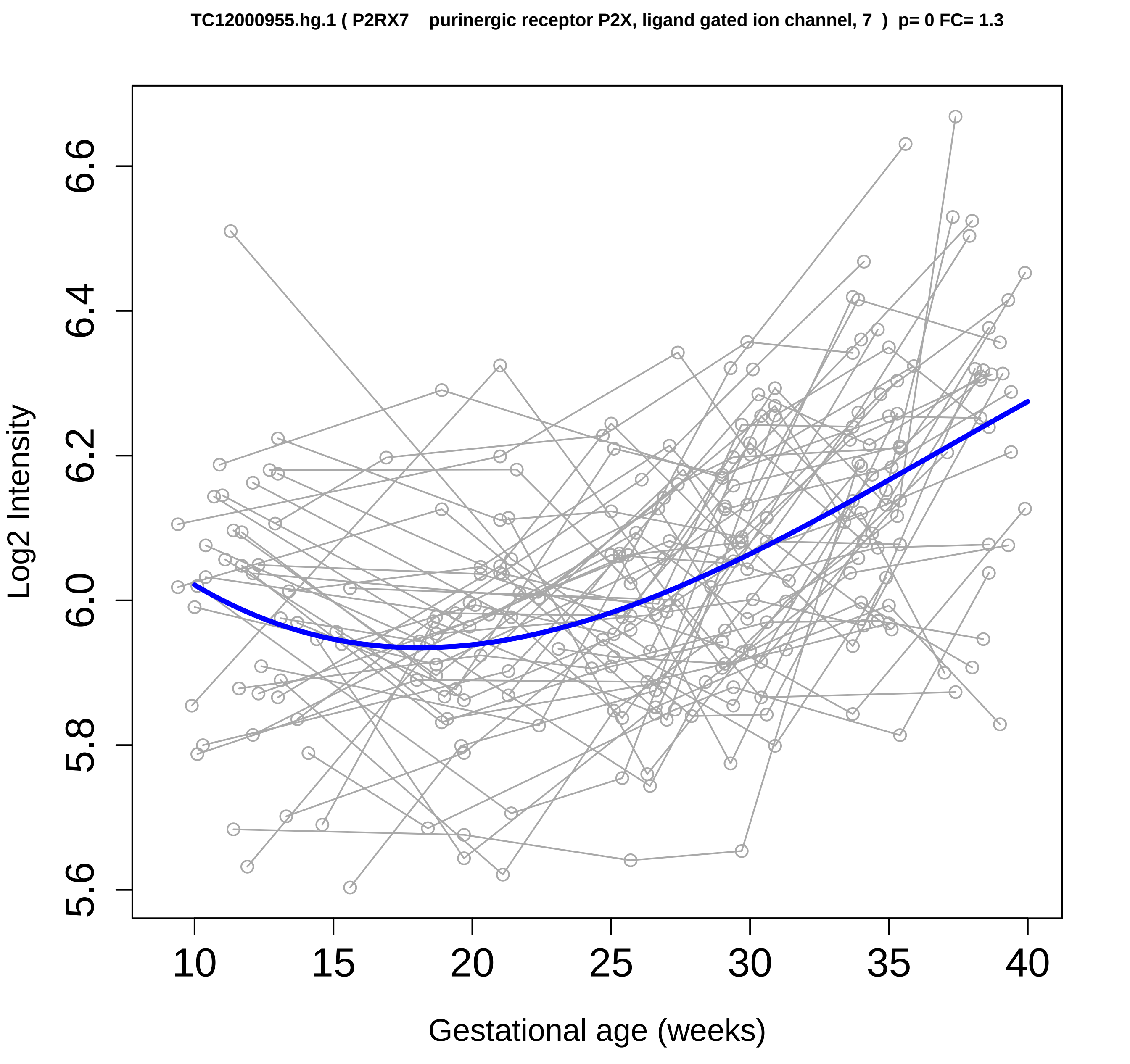

TC12000955.hg.1 (P2RX7 purinergic receptor P2X, ligand gated ion channel, 7) p= 0 FC= 1.3
Log2 Intensity
5.6
5.8
6.0
6.2
6.4
6.6
10
15
20
25
30
35
40
Gestational age (weeks)

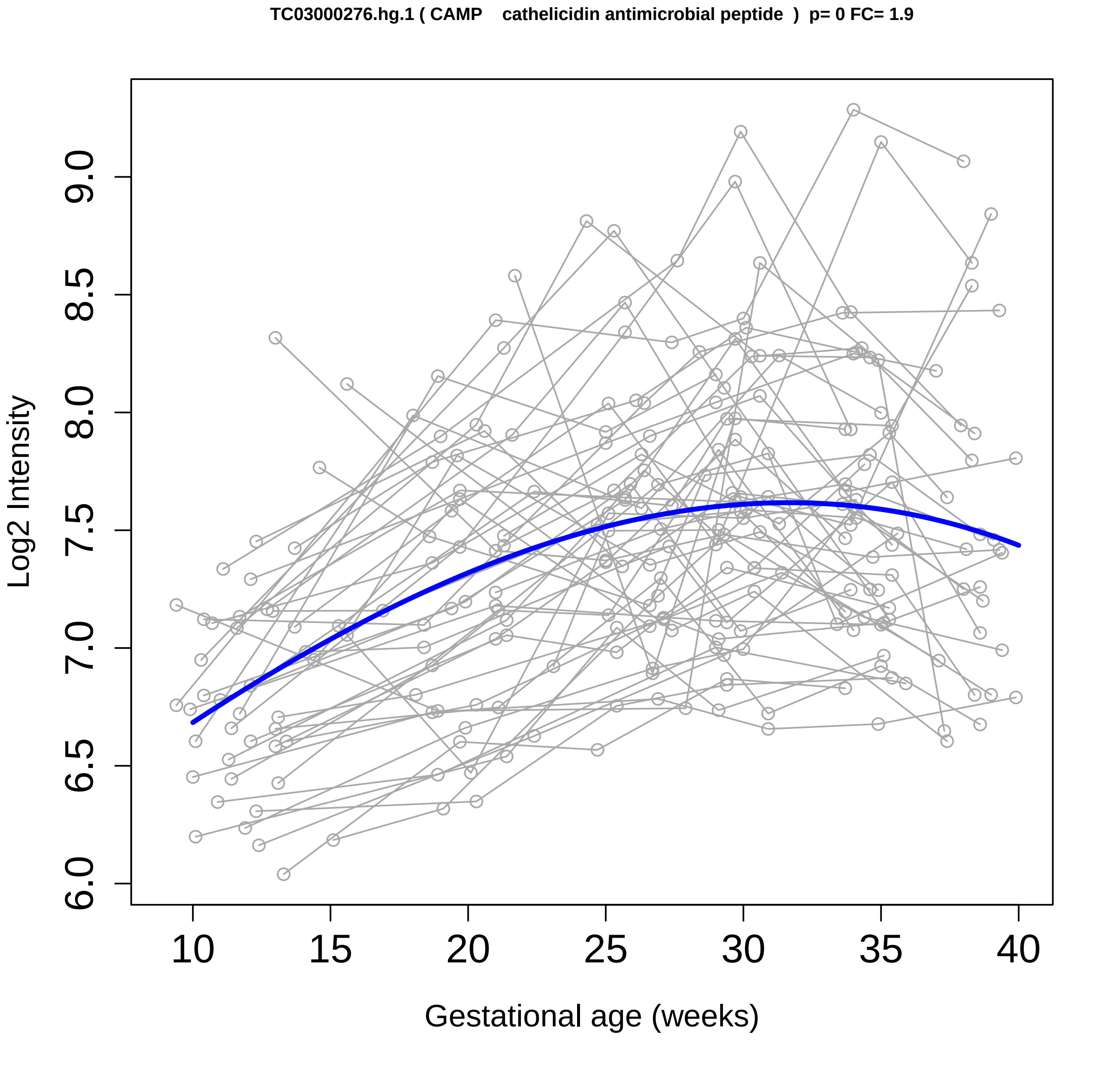
TC03000276.hg.1 (CAMP cathelicidin antimicrobial peptide) p= 0 FC= 1.9
Log2 Intensity
6.0
6.5
7.0
7.5
8.0
8.5
9.0
10
15
20
25
30
35
40
Gestational age (weeks)

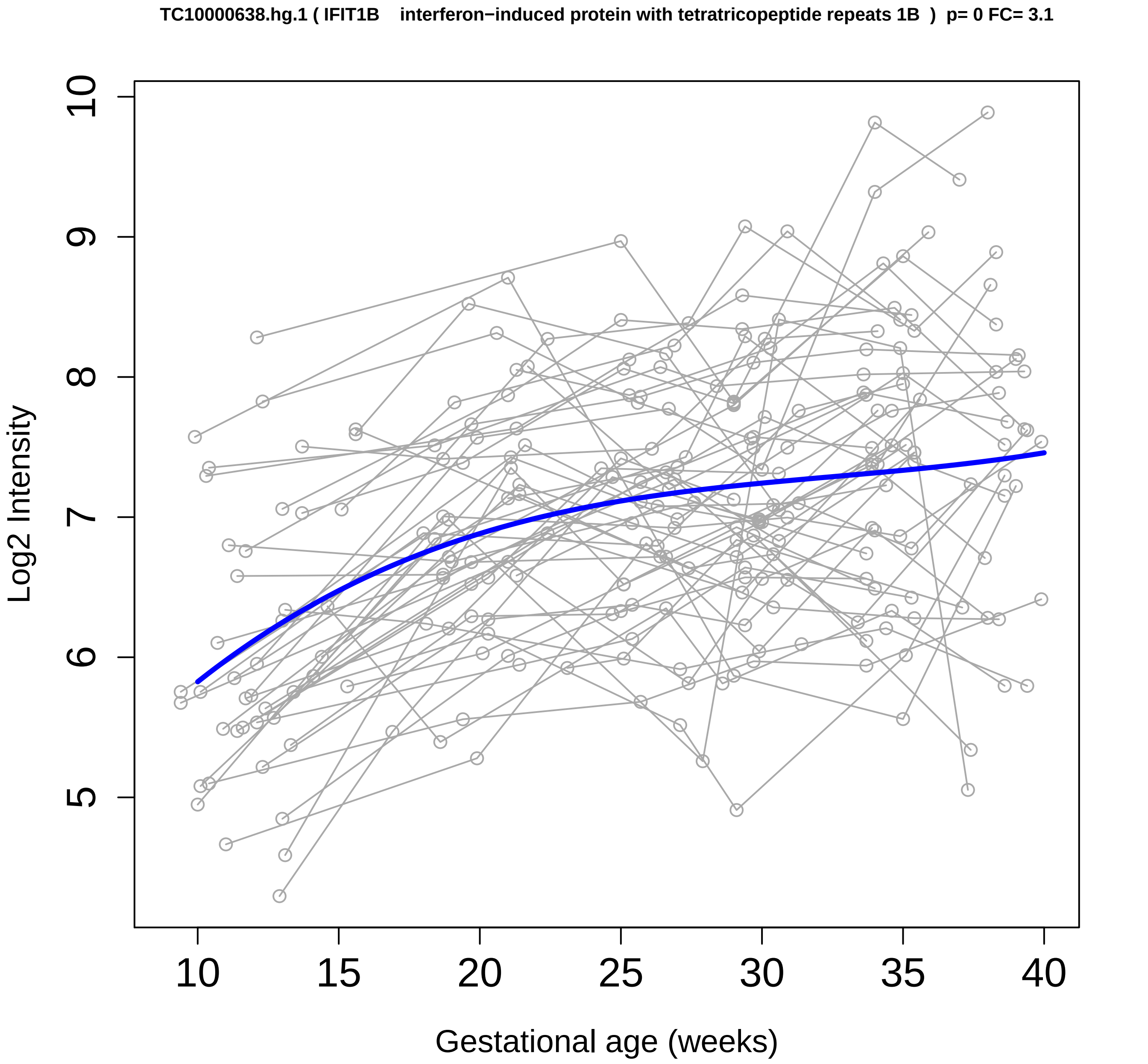
TC10000638.hg.1 (IFIT1B interferon−induced protein with tetratricopeptide repeats 1B) p= 0 FC= 3.1
Log2 Intensity
5
6
7
8
9
10
10
15
20
25
30
35
40
Gestational age (weeks)

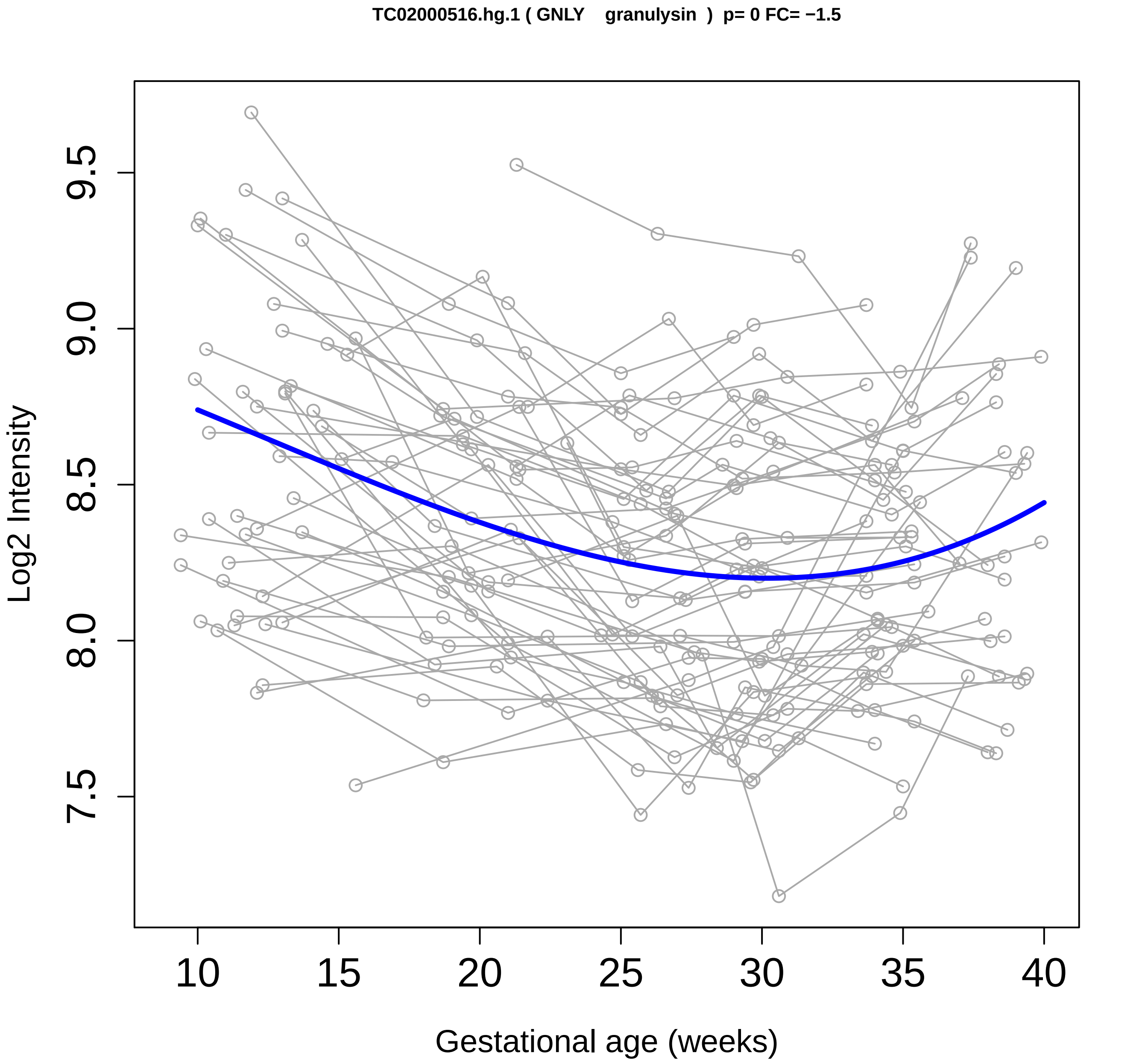

TC02000516.hg.1 (GNLY granulysin) p= 0 FC= −1.5
Log2 Intensity
9.5
9.0
8.5
8.0
7.5
10
15
20
25
30
35
40
Gestational age (weeks)

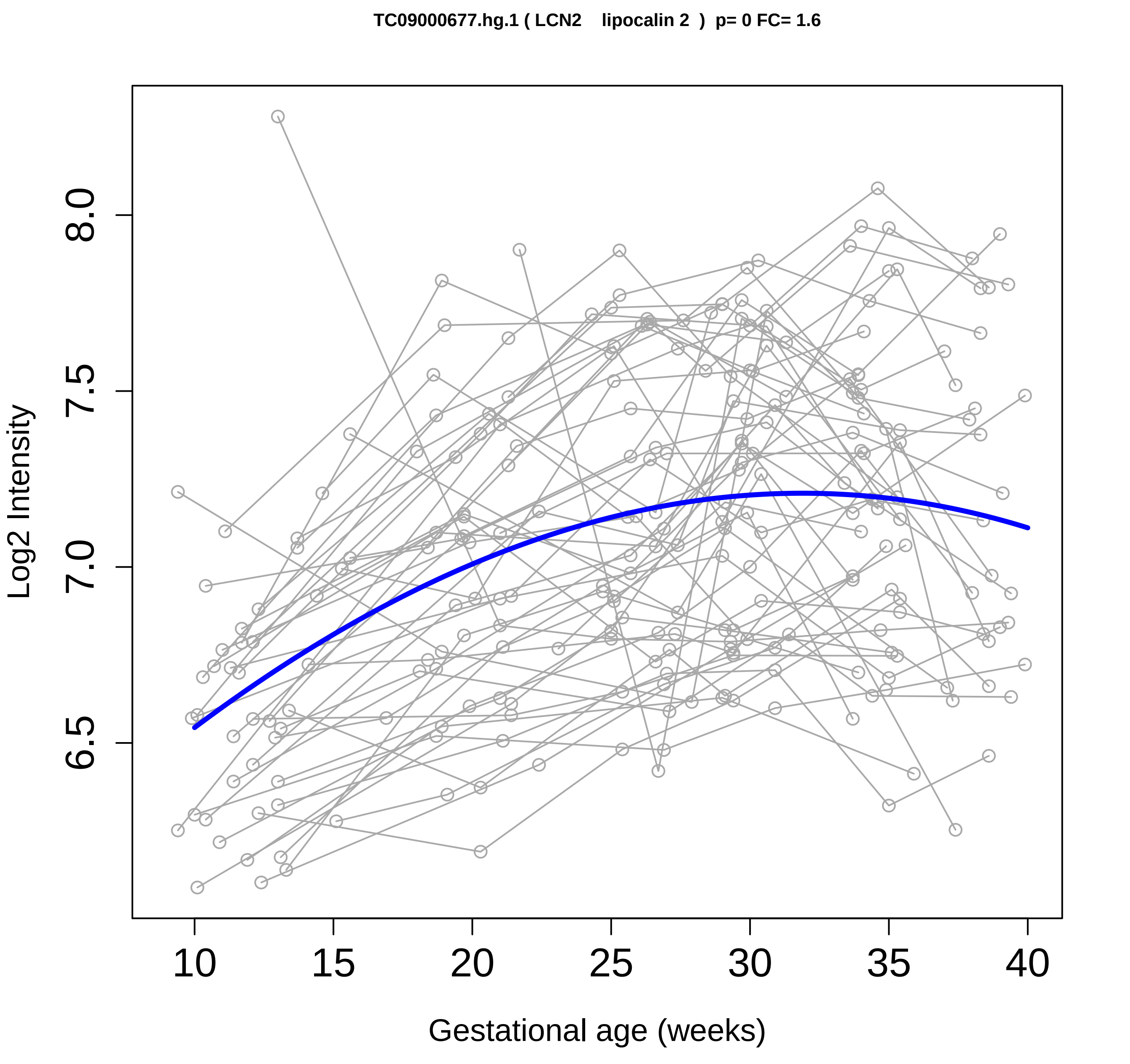

TC09000677.hg.1 (LCN2 lipocalin 2) p= 0 FC= 1.6
Log2 Intensity
6.5
7.0
7.5
8.0
10
15
20
25
30
35
40
Gestational age (weeks)

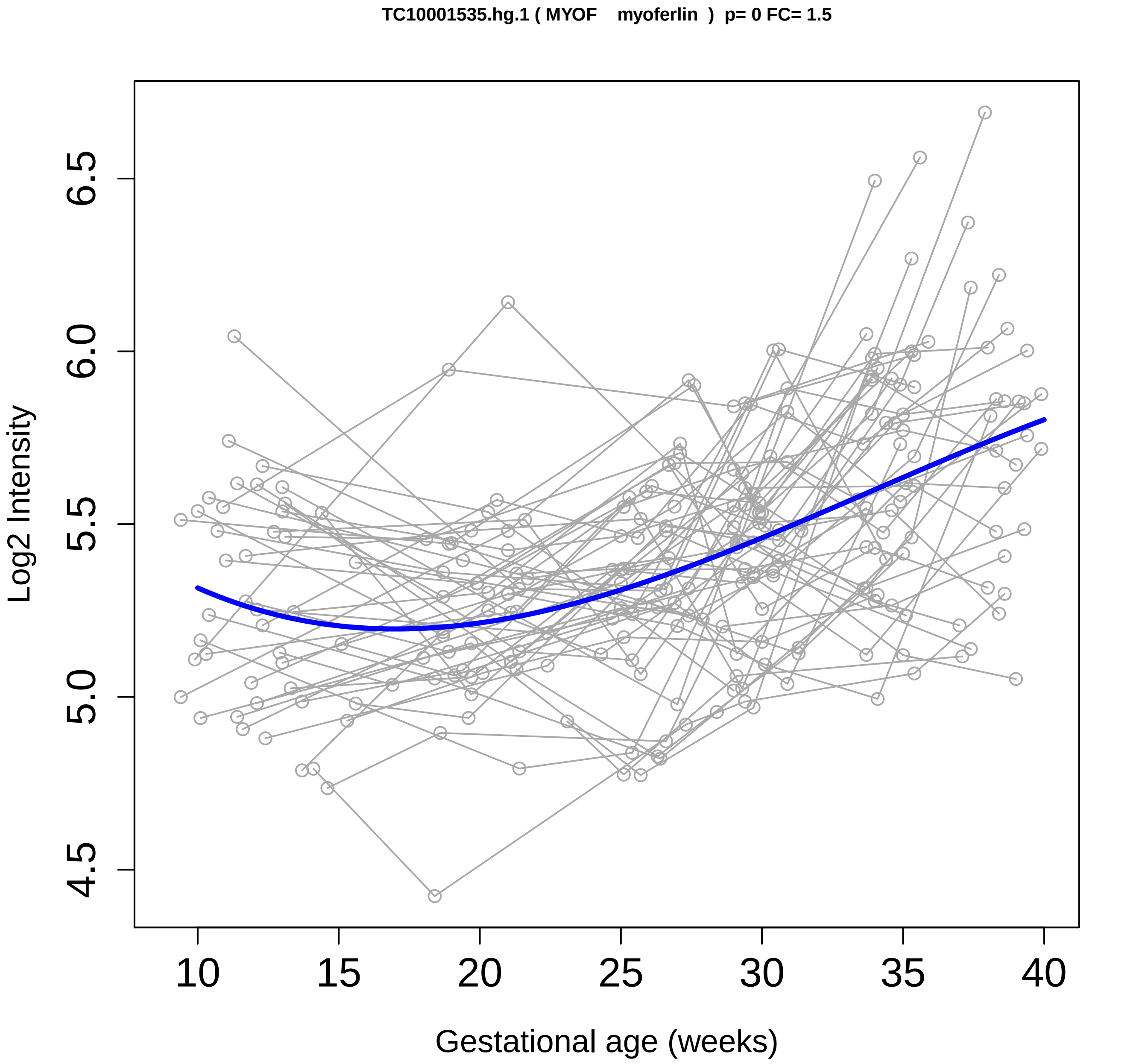

TC10001535.hg.1 (MYOF myoferlin) p= 0 FC= 1.5
Log2 Intensity
4.5
5.0
5.5
6.0
6.5
10
15
20
25
30
35
40
Gestational age (weeks)

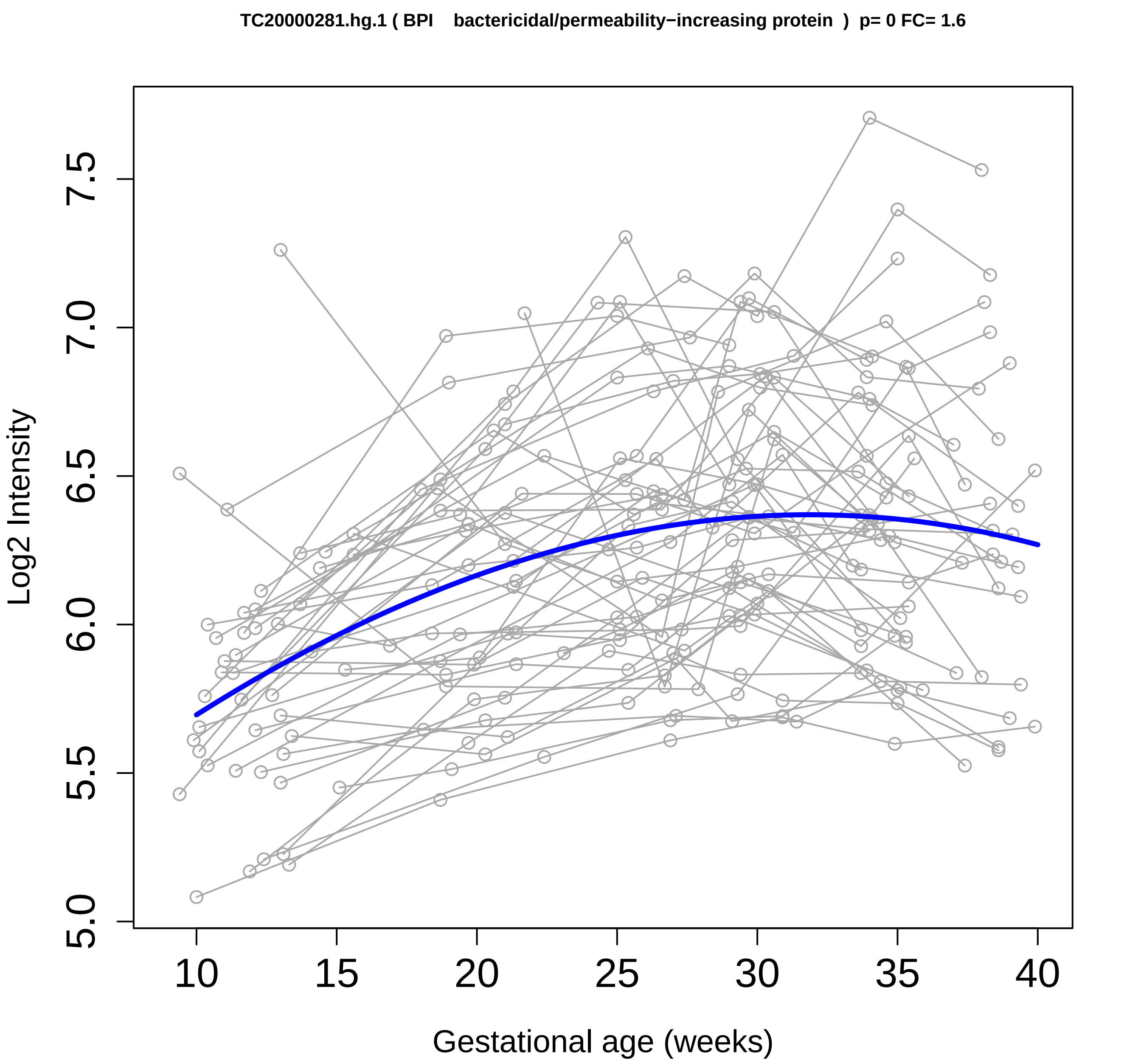

TC20000281.hg.1 (BPI bactericidal/permeability−increasing protein) p= 0 FC= 1.6
Log2 Intensity
5.0
5.5
6.0
6.5
7.0
7.5
10
15
20
25
30
35
40
Gestational age (weeks)

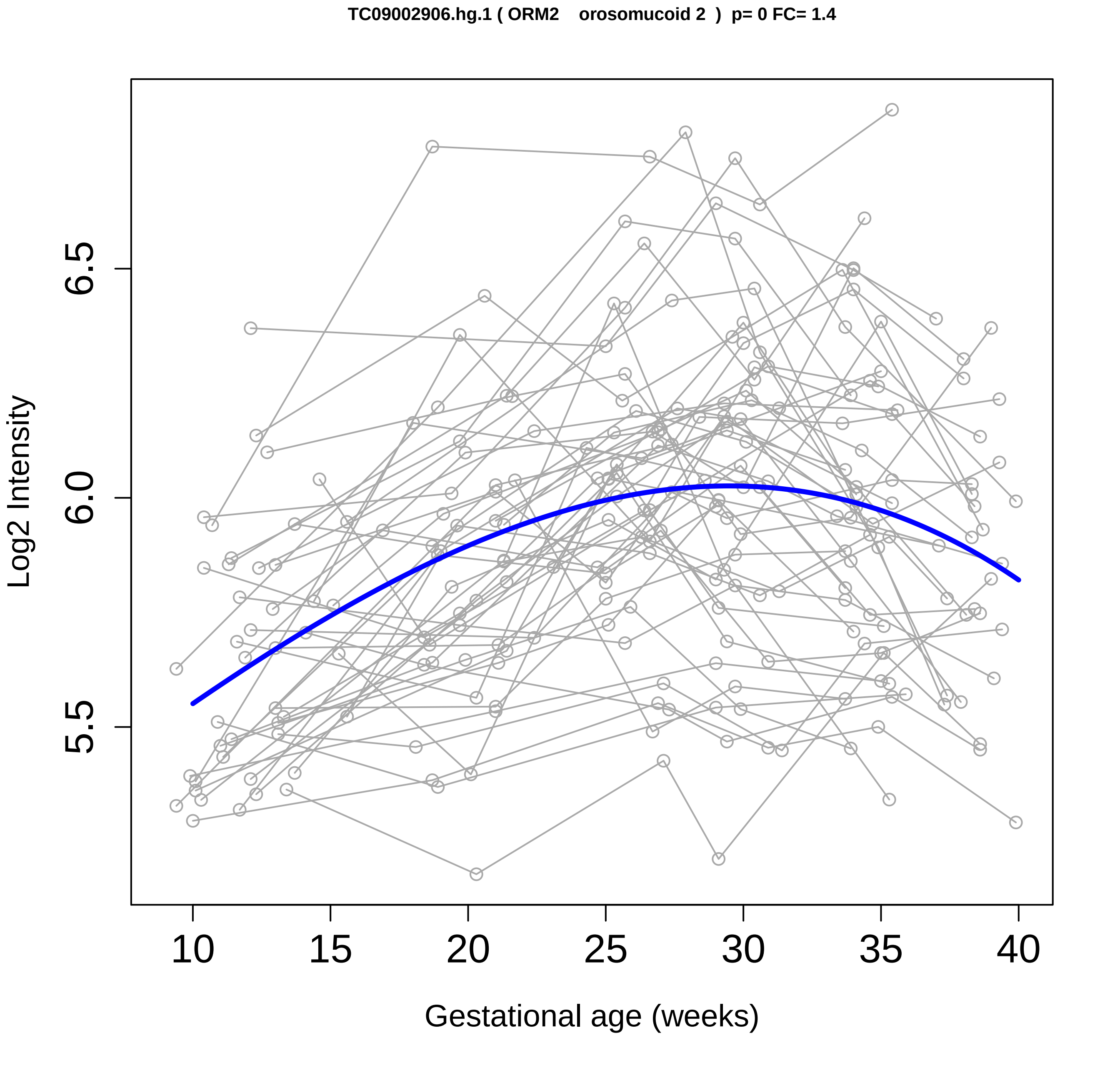
TC09002906.hg.1 (ORM2 orosomucoid 2) p= 0 FC= 1.4
Log2 Intensity
5.5
6.0
6.5
10
15
20
25
30
35
40
Gestational age (weeks)

TC07001858.hg.1 (UBE2H ubiquitin conjugating enzyme E2H) p= 0 FC= 1.5

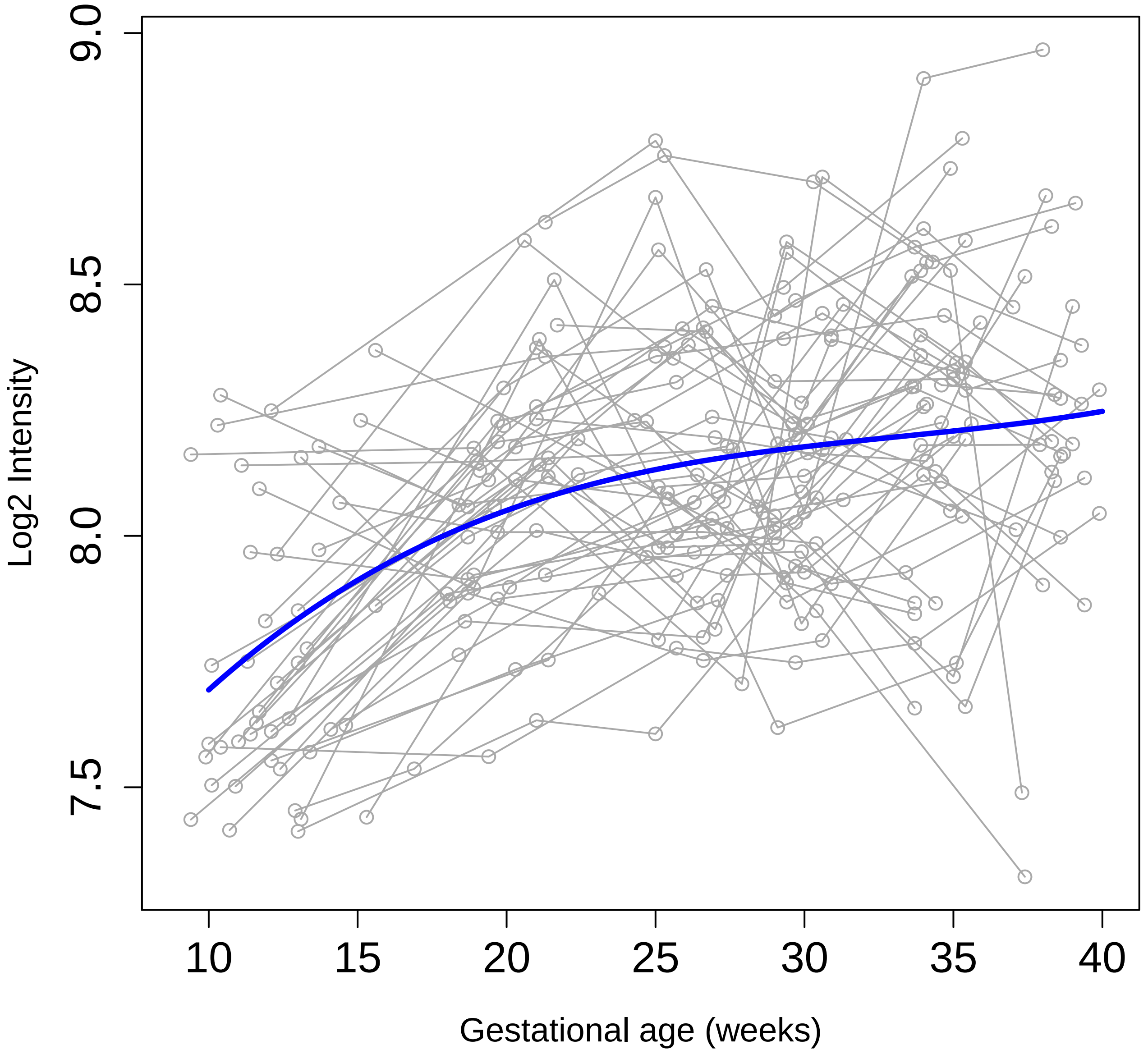

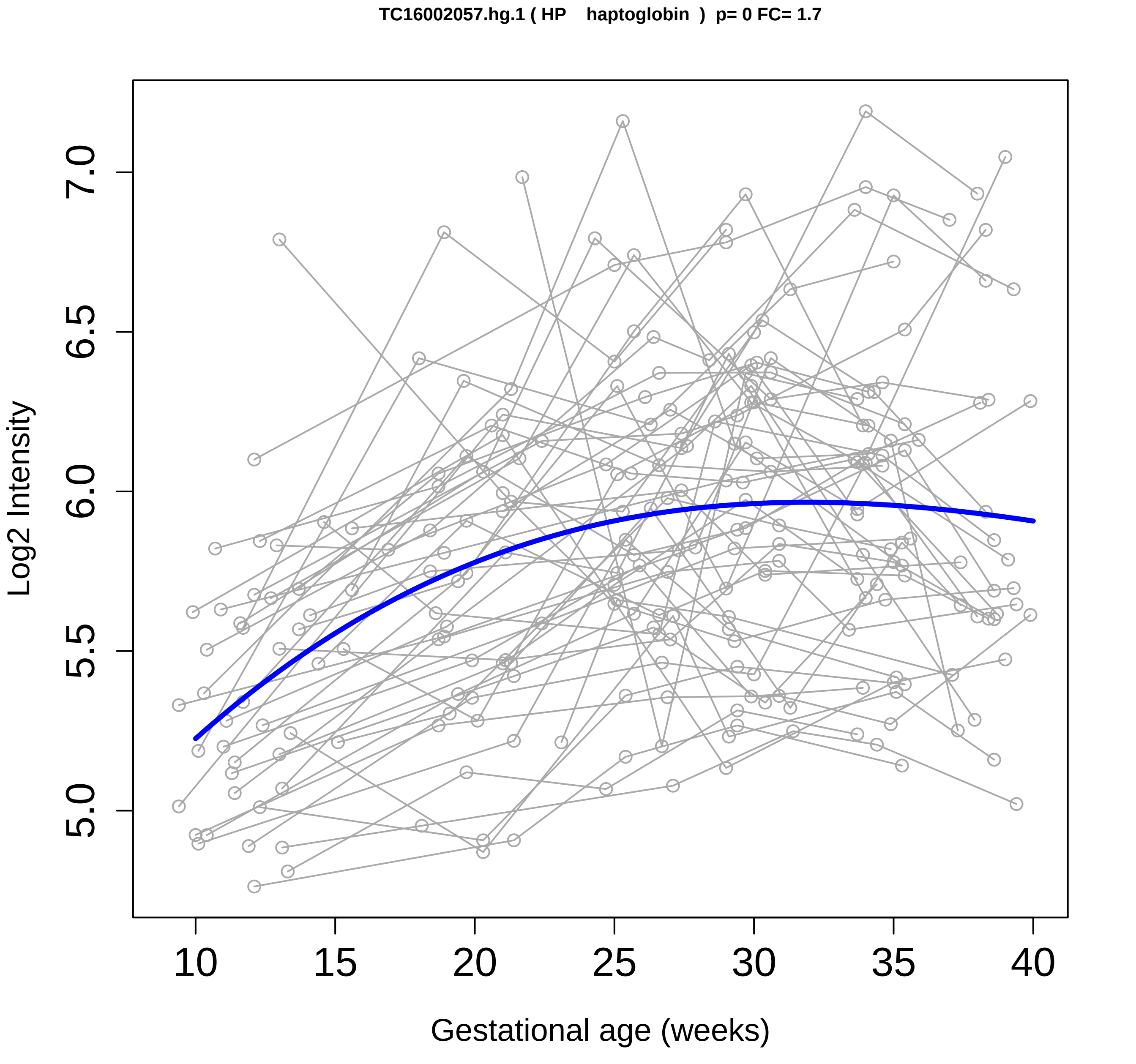

TC16002057.hg.1 (HP haptoglobin) p= 0 FC= 1.7
Log2 Intensity
5.0
5.5
6.0
6.5
7.0
10
15
20
25
30
35
40
Gestational age (weeks)

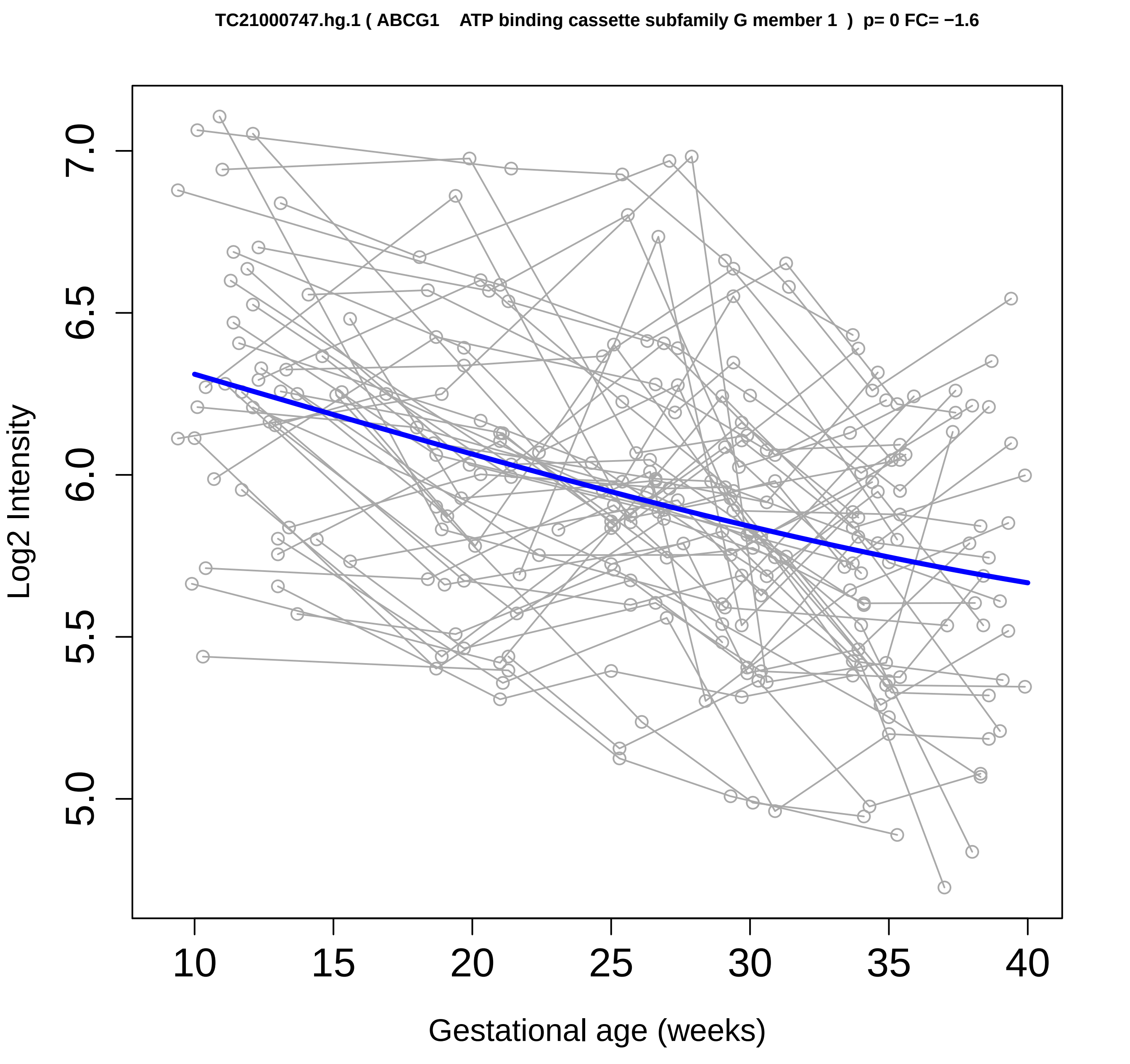
TC21000747.hg.1 (ABCG1 ATP binding cassette subfamily G member 1) p= 0 FC= −1.6
Log2 Intensity
7.0
6.5
6.0
5.5
5.0
10
15
20
25
30
35
40
Gestational age (weeks)

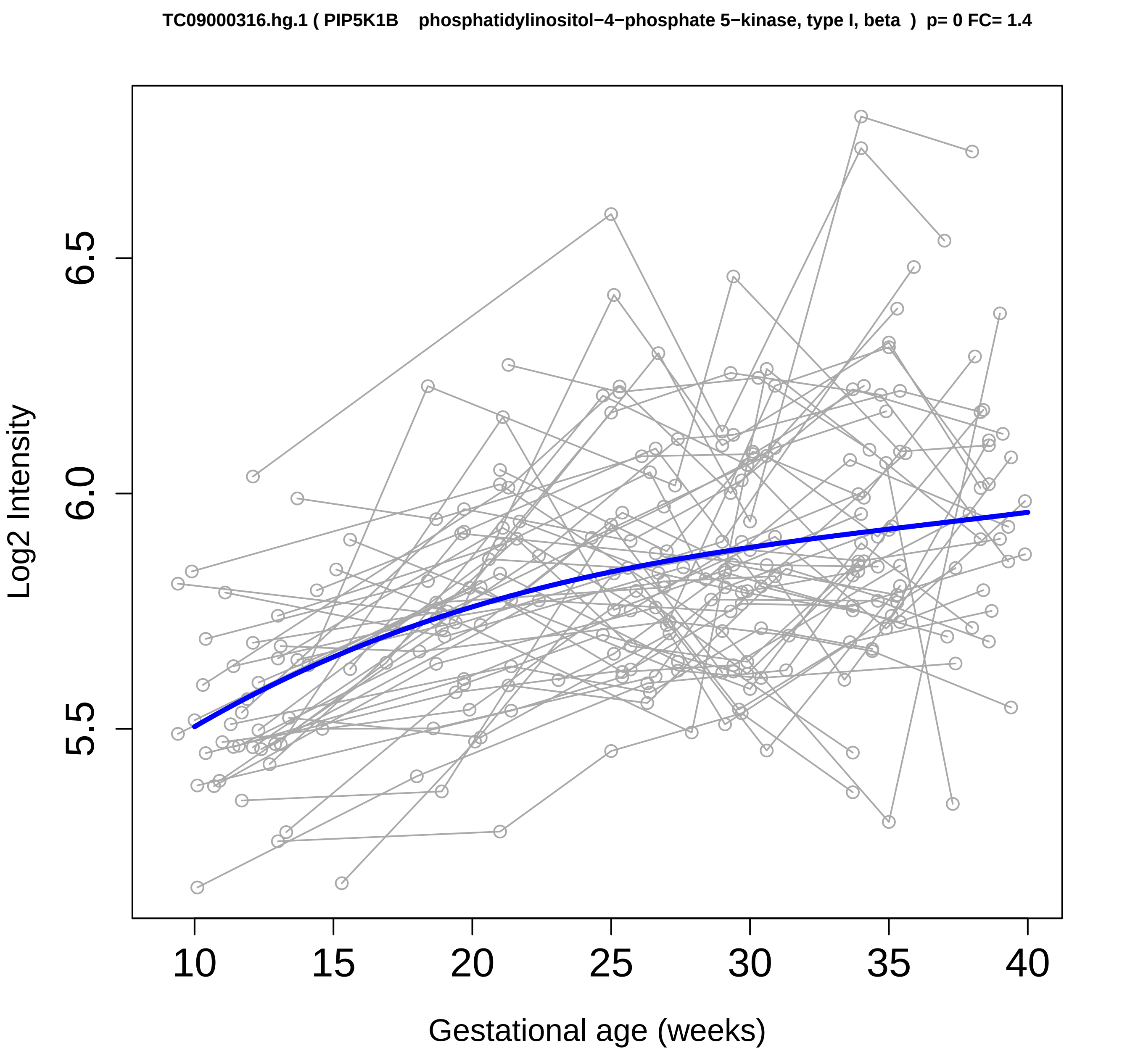
TC09000316.hg.1 (PIP5K1B phosphatidylinositol−4−phosphate 5−kinase, type I, beta) p= 0 FC= 1.4
Log2 Intensity
6.5
6.0
5.5
10
15
20
25
30
35
40
Gestational age (weeks)

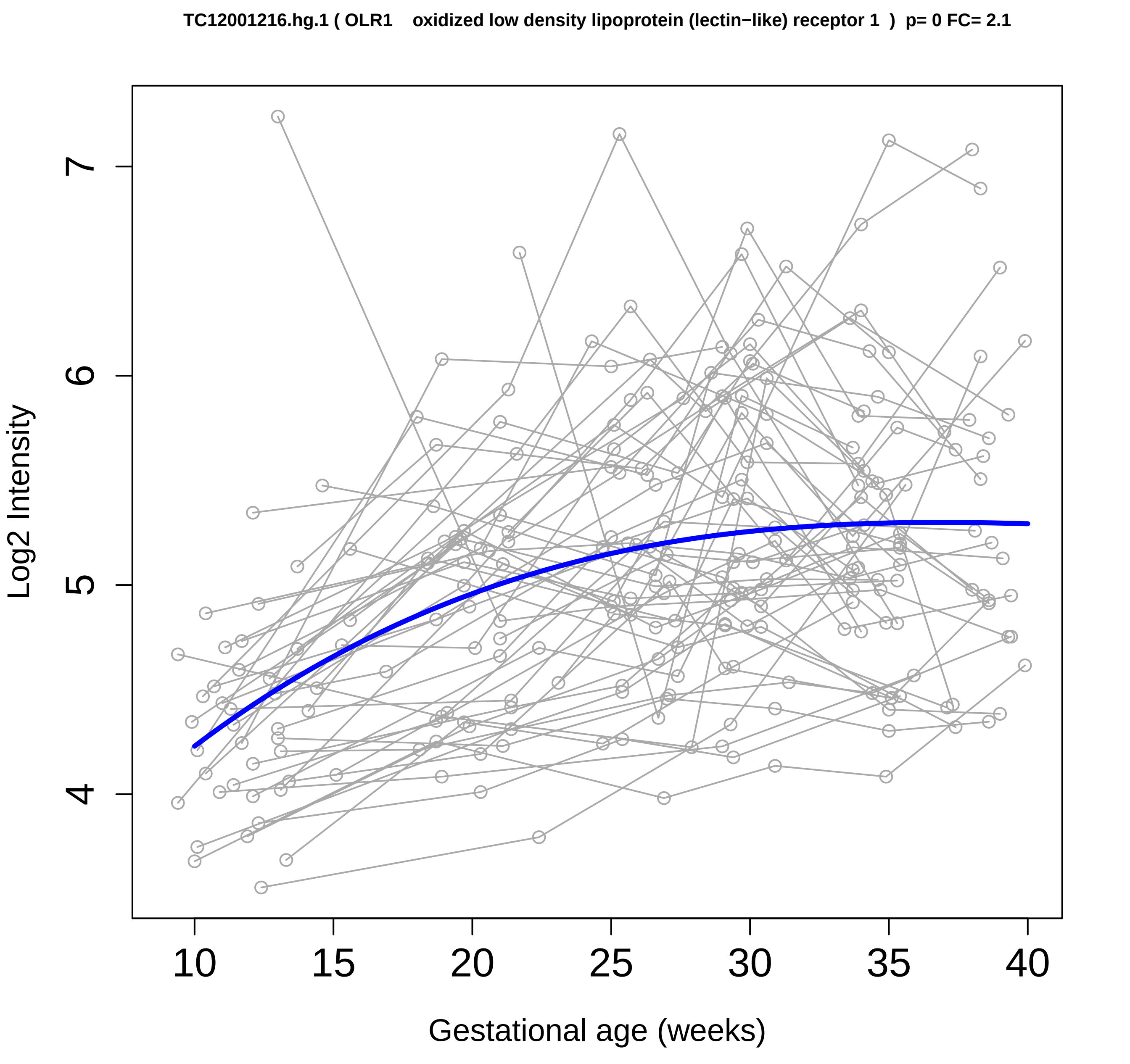

TC12001216.hg.1 (OLR1 oxidized low density lipoprotein (lectin−like) receptor 1) p= 0 FC= 2.1
Log2 Intensity
4
5
6
7
10
15
20
25
30
35
40
Gestational age (weeks)

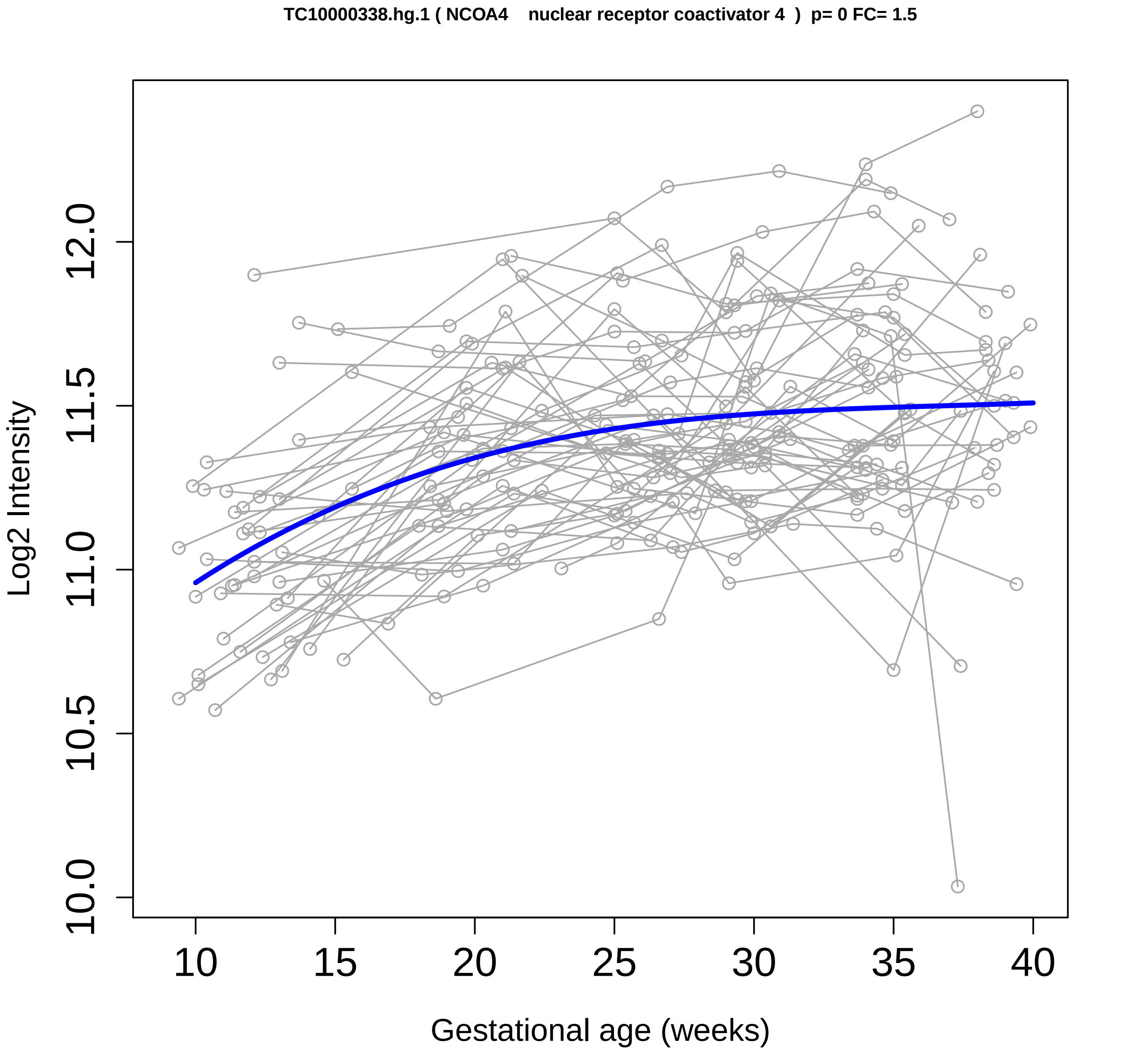
TC10000338.hg.1 (NCOA4 nuclear receptor coactivator 4) p= 0 FC= 1.5
Log2 Intensity
10.0
10.5
11.0
11.5
12.0
10
15
20
25
30
35
40
Gestational age (weeks)

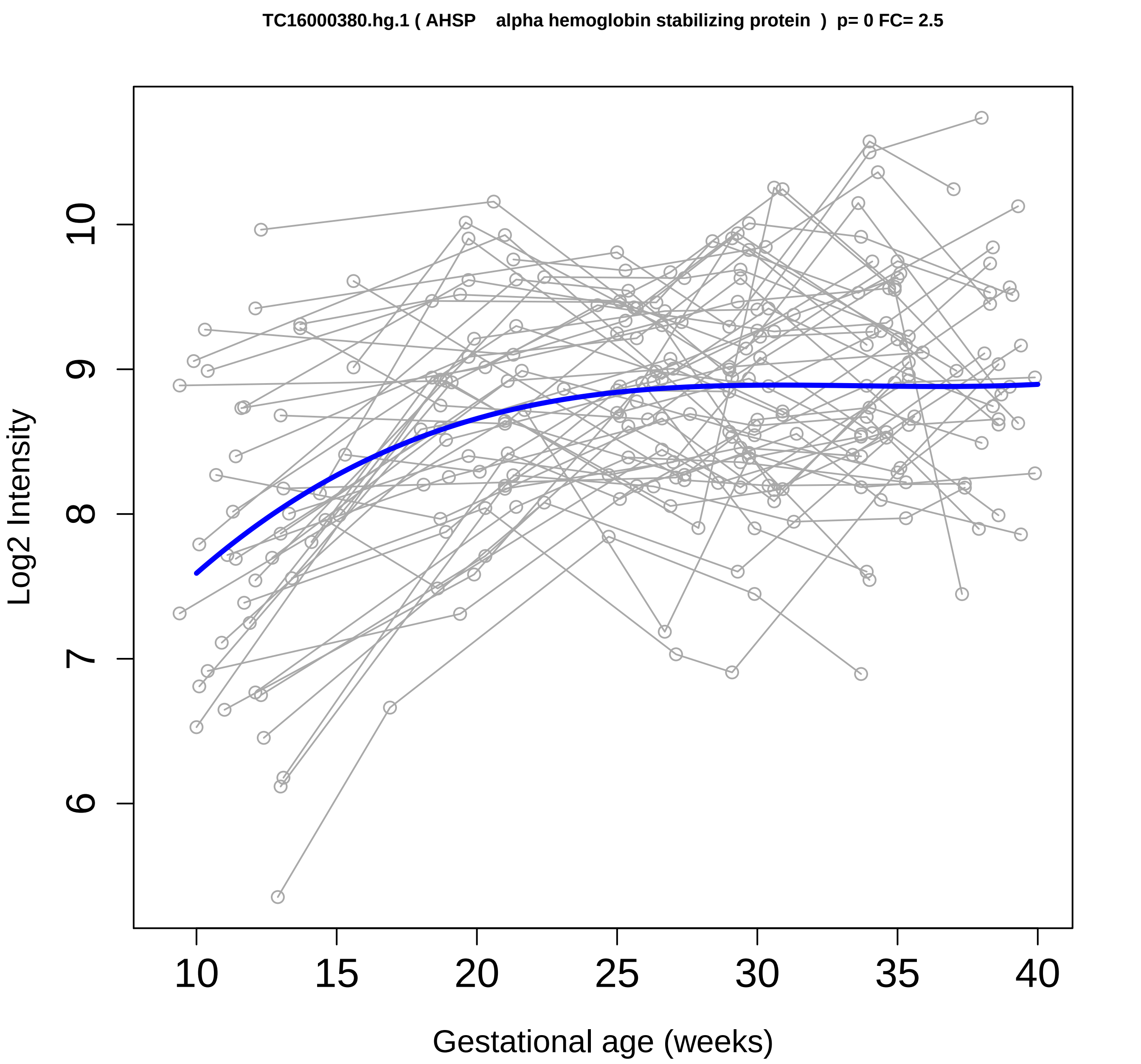
TC16000380.hg.1 (AHSP alpha hemoglobin stabilizing protein) p= 0 FC= 2.5
Log2 Intensity
6
7
8
9
10
Gestational age (weeks)
10
15
20
25
30
35
40

TC21000200.hg.1 (ABCG1   ATP binding cassette subfamily G member 1) p= 0 FC= −1.6

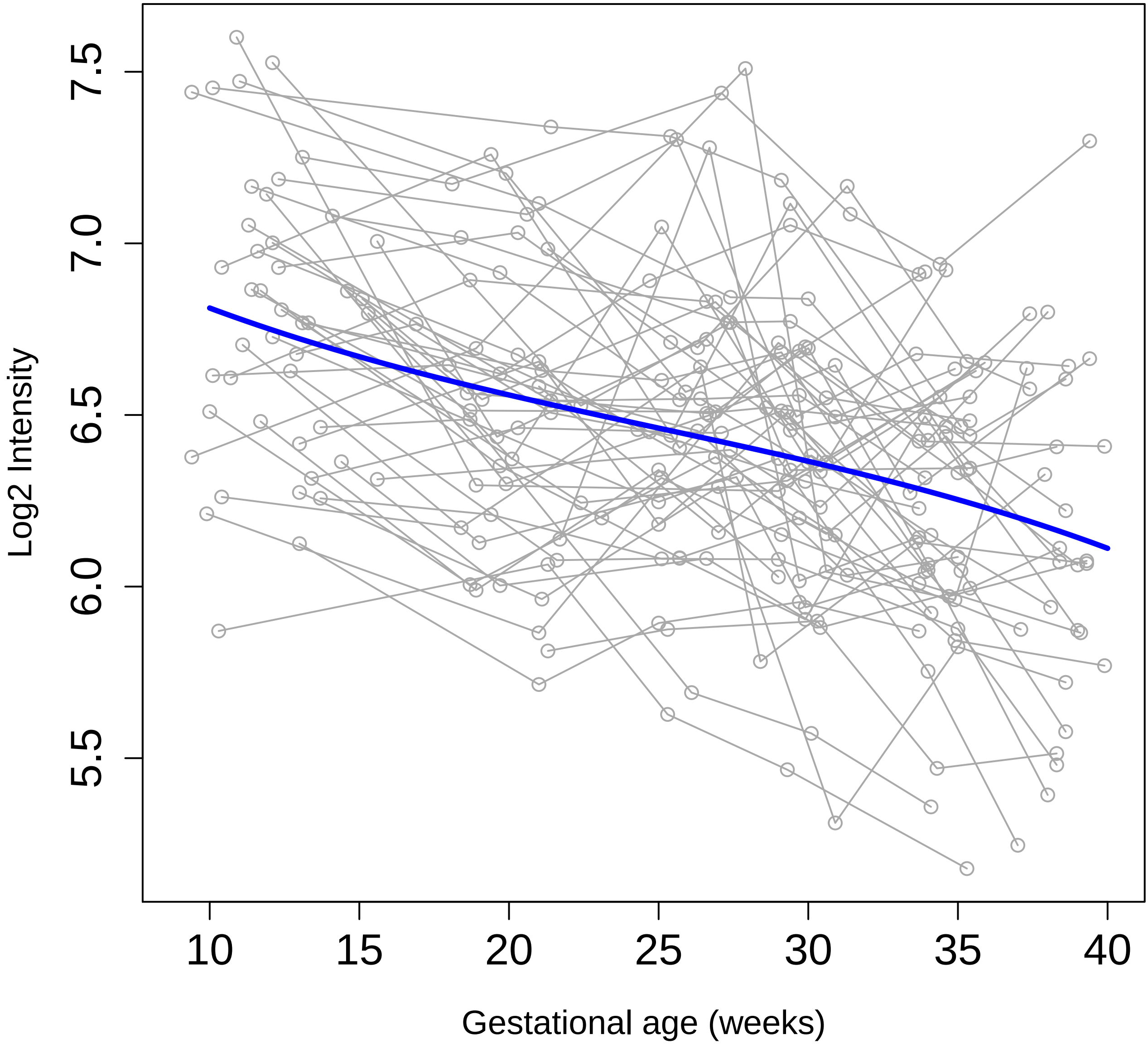

TC01000301.hg.1 (PITHD1 PITH (C−terminal proteasome−interacting domain of thioredoxin−like) domain containing 1) p= 0 FC

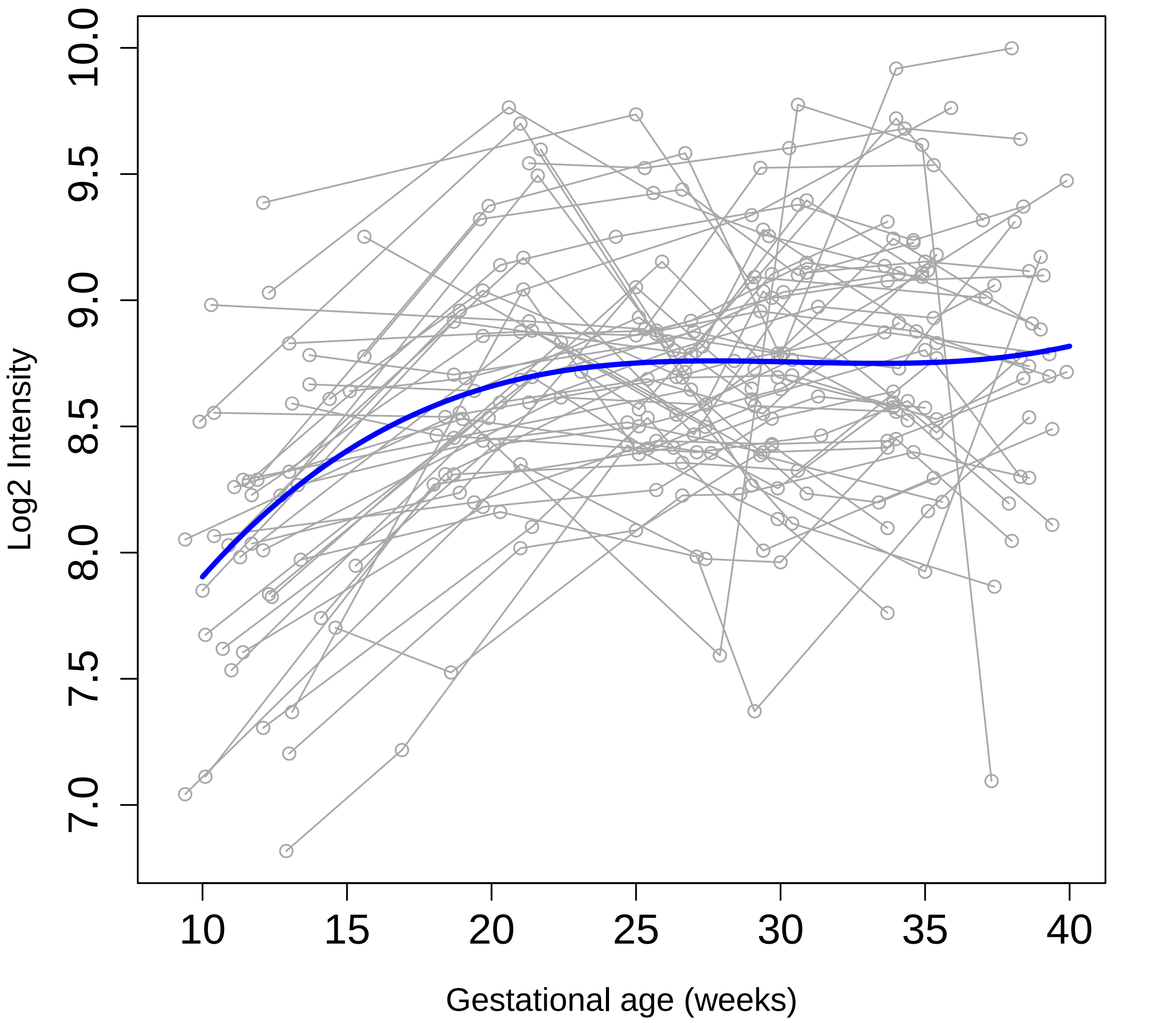

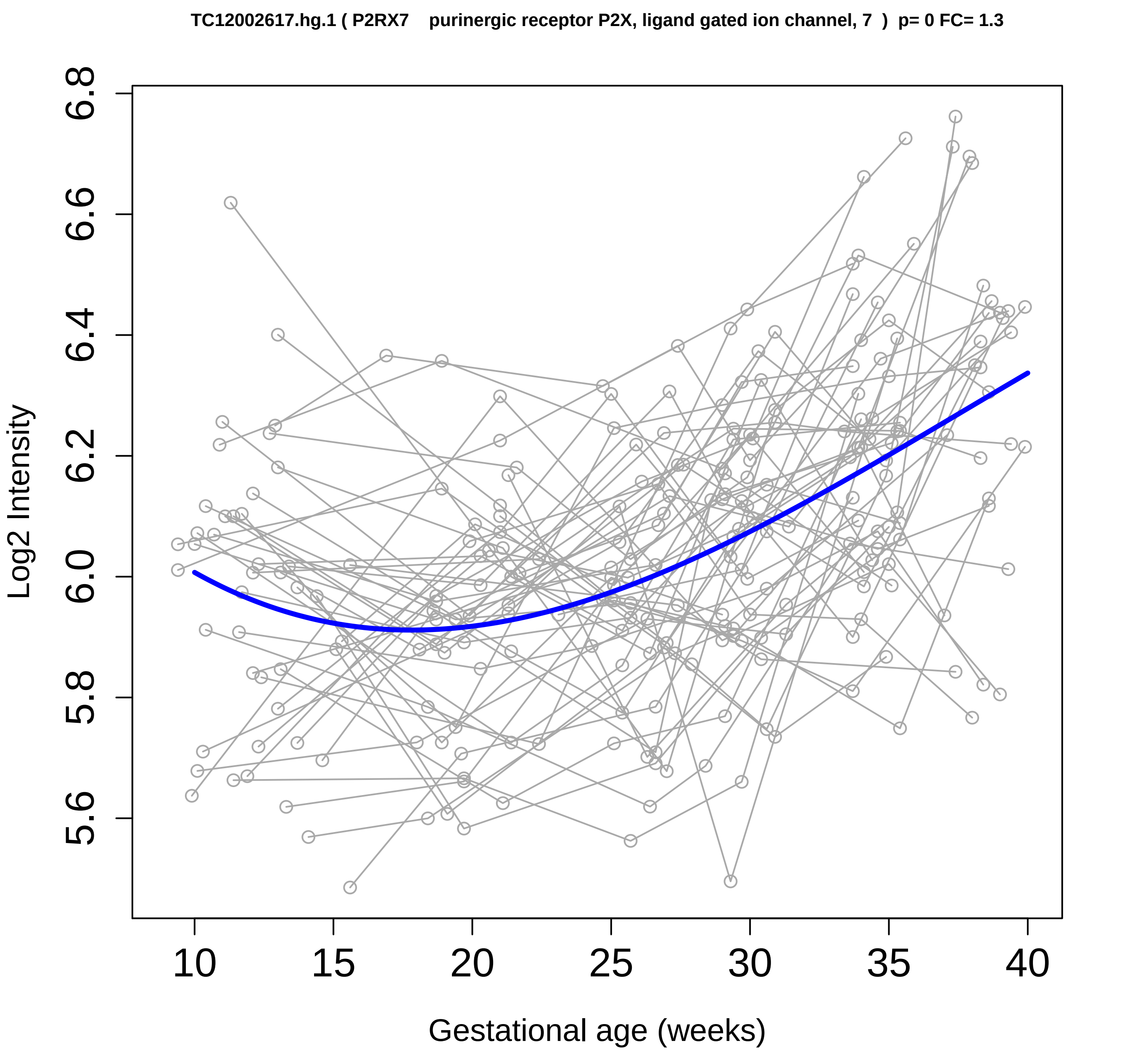
TC12002617.hg.1 (P2RX7 purinergic receptor P2X, ligand gated ion channel, 7) p= 0 FC= 1.3
Log2 Intensity
5.6
5.8
6.0
6.2
6.4
6.6
6.8
10
15
20
25
30
35
40
Gestational age (weeks)

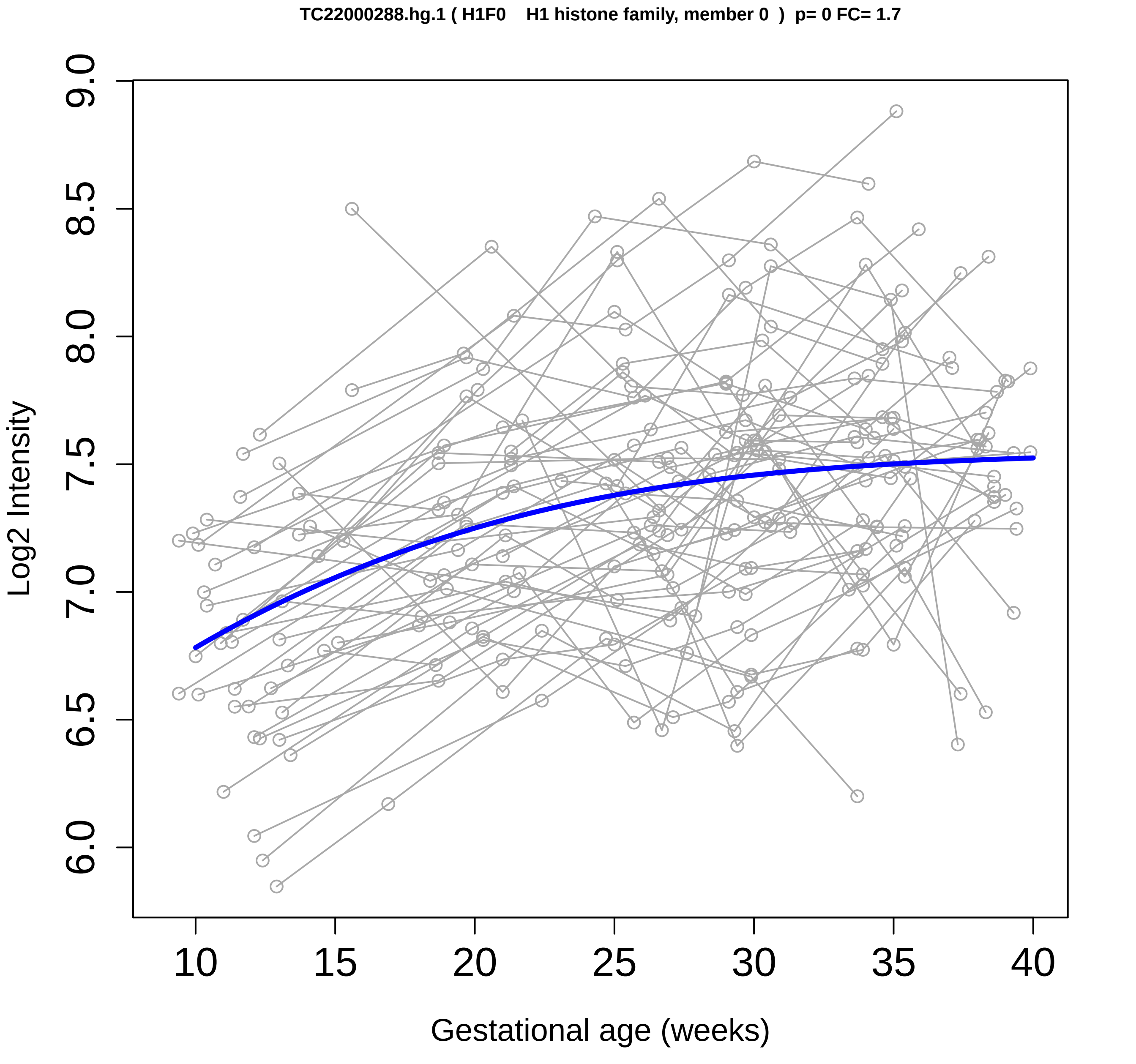
TC22000288.hg.1 (H1F0 H1 histone family, member 0) p= 0 FC= 1.7
9.0
8.5
8.0
7.5
7.0
6.5
6.0
Log2 Intensity
10
15
20
25
30
35
40
Gestational age (weeks)

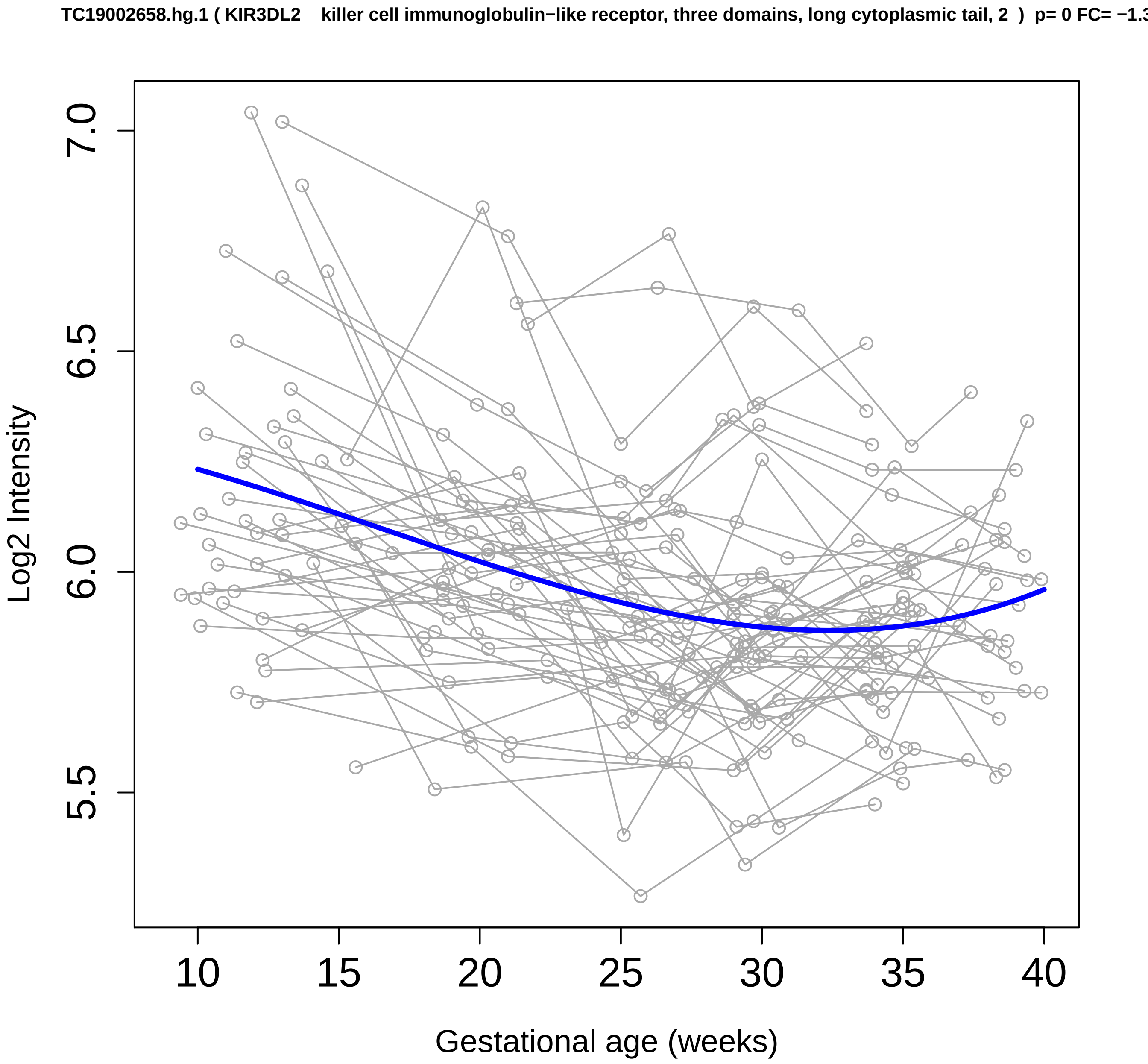
TC19002658.hg.1 (KIR3DL2 killer cell immunoglobulin−like receptor, three domains, long cytoplasmic tail, 2) p= 0 FC= −1.3
Log2 Intensity
7.0
6.5
6.0
5.5
10
15
20
25
30
35
40
Gestational age (weeks)

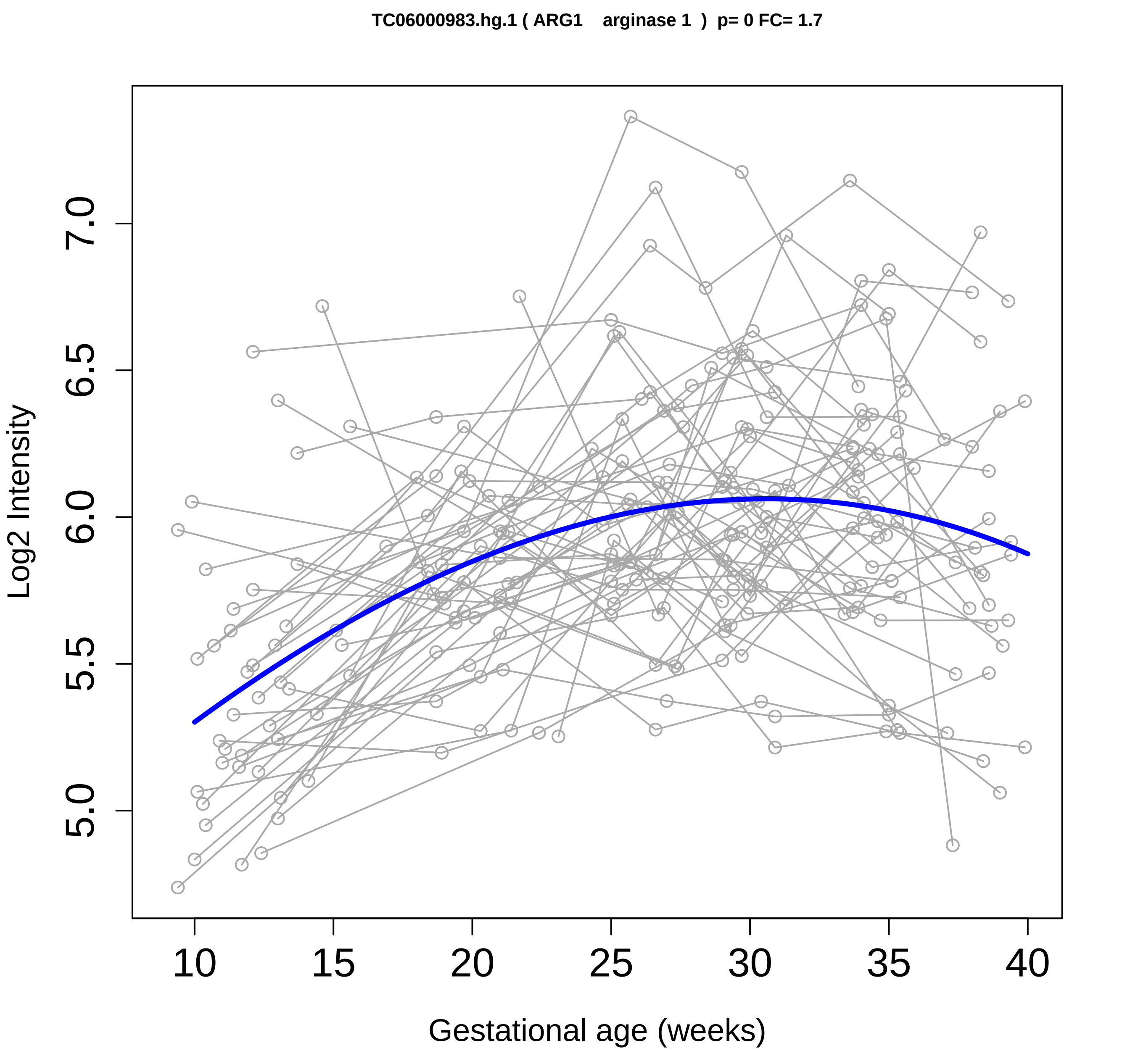

TC06000983.hg.1 (ARG1 arginase 1) p= 0 FC= 1.7
Log2 Intensity
5.0
5.5
6.0
6.5
7.0
10
15
20
25
30
35
40
Gestational age (weeks)

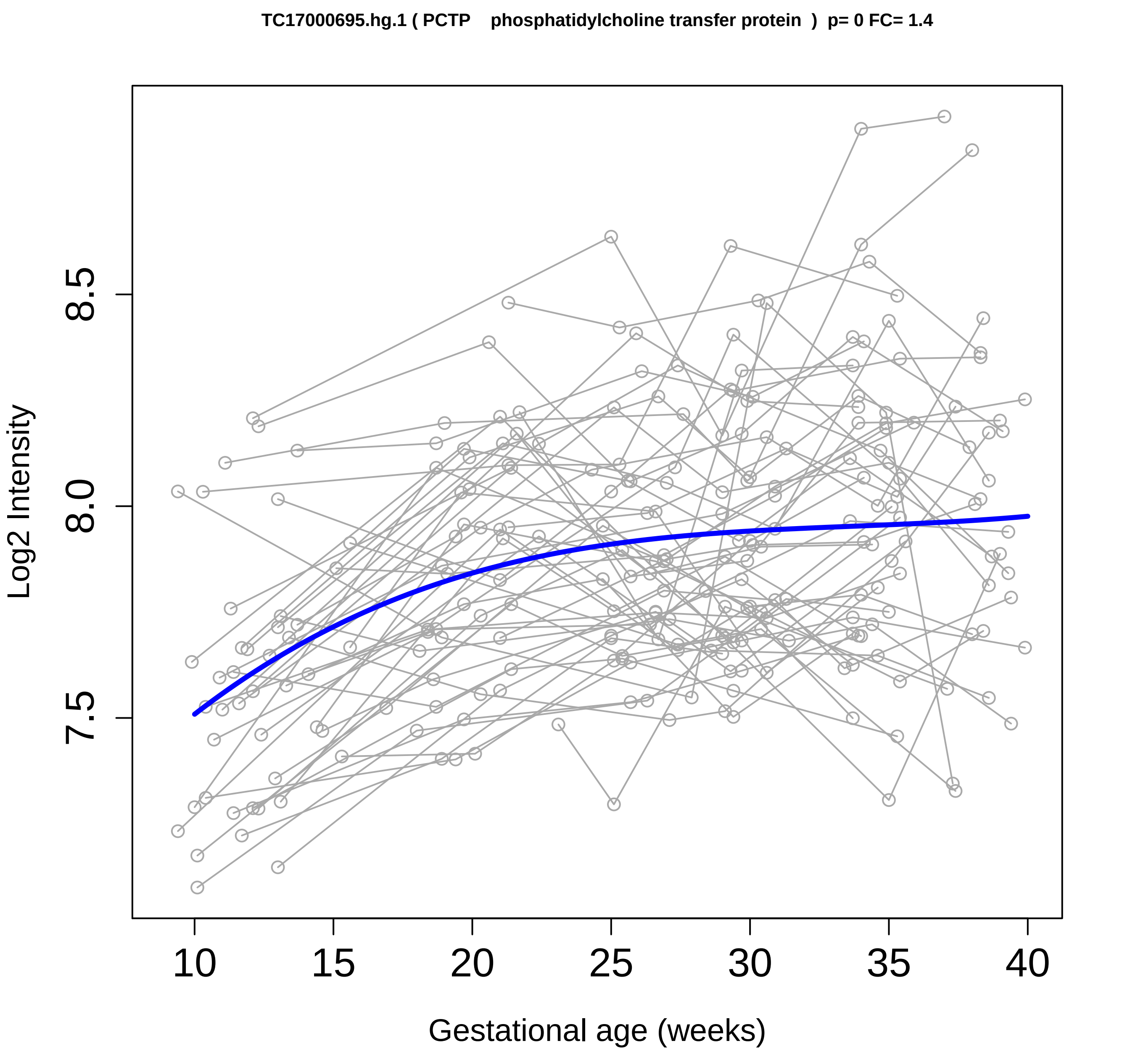

TC17000695.hg.1 (PCTP   phosphatidylcholine transfer protein) p= 0 FC= 1.4
Log2 Intensity
8.5
8.0
7.5
10
15
20
25
30
35
40
Gestational age (weeks)

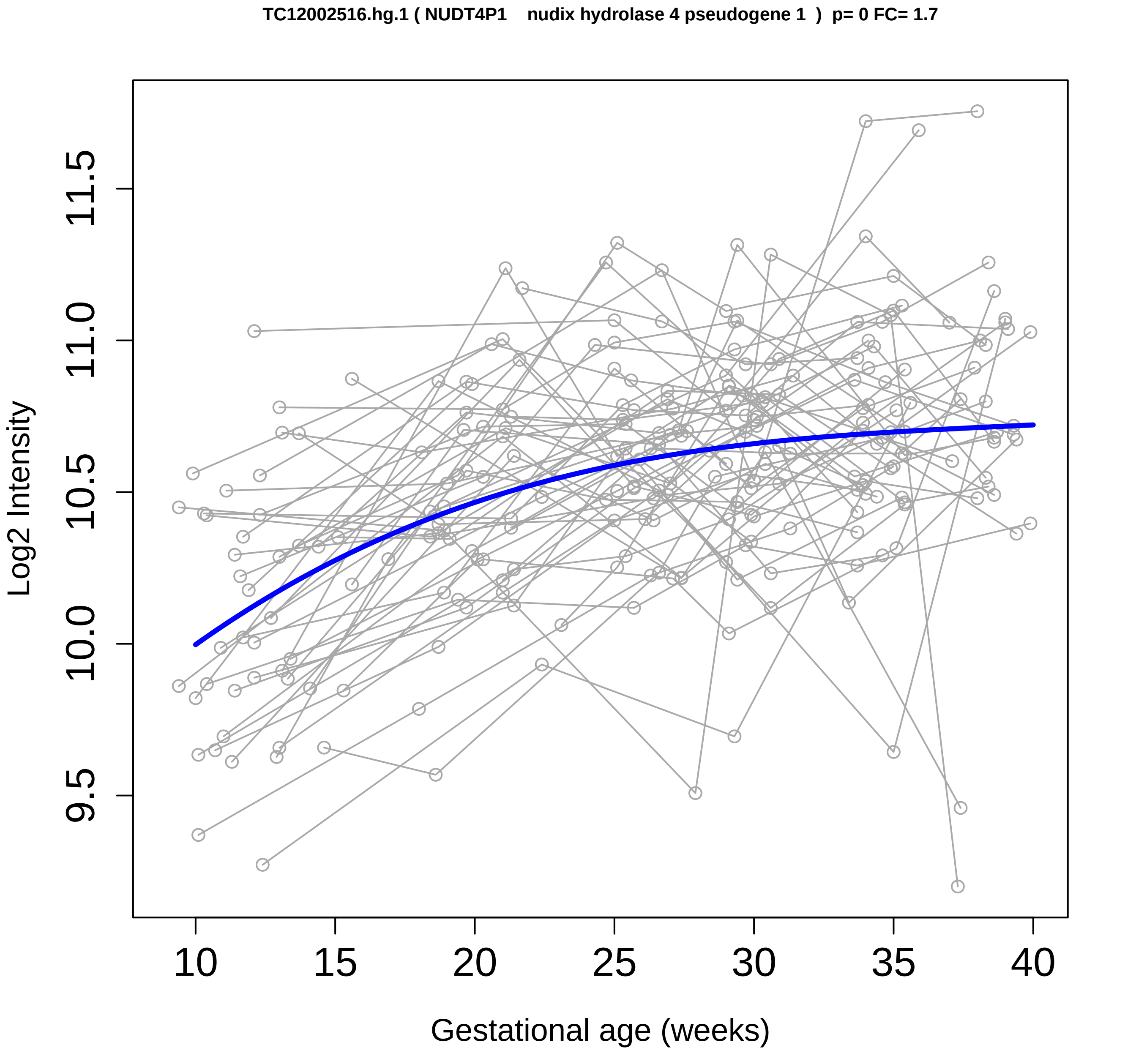

TC12002516.hg.1 (NUDT4P1 nudix hydrolase 4 pseudogene 1) p= 0 FC= 1.7
Log2 Intensity
9.5
10.0
10.5
11.0
11.5
10
15
20
25
30
35
40
Gestational age (weeks)

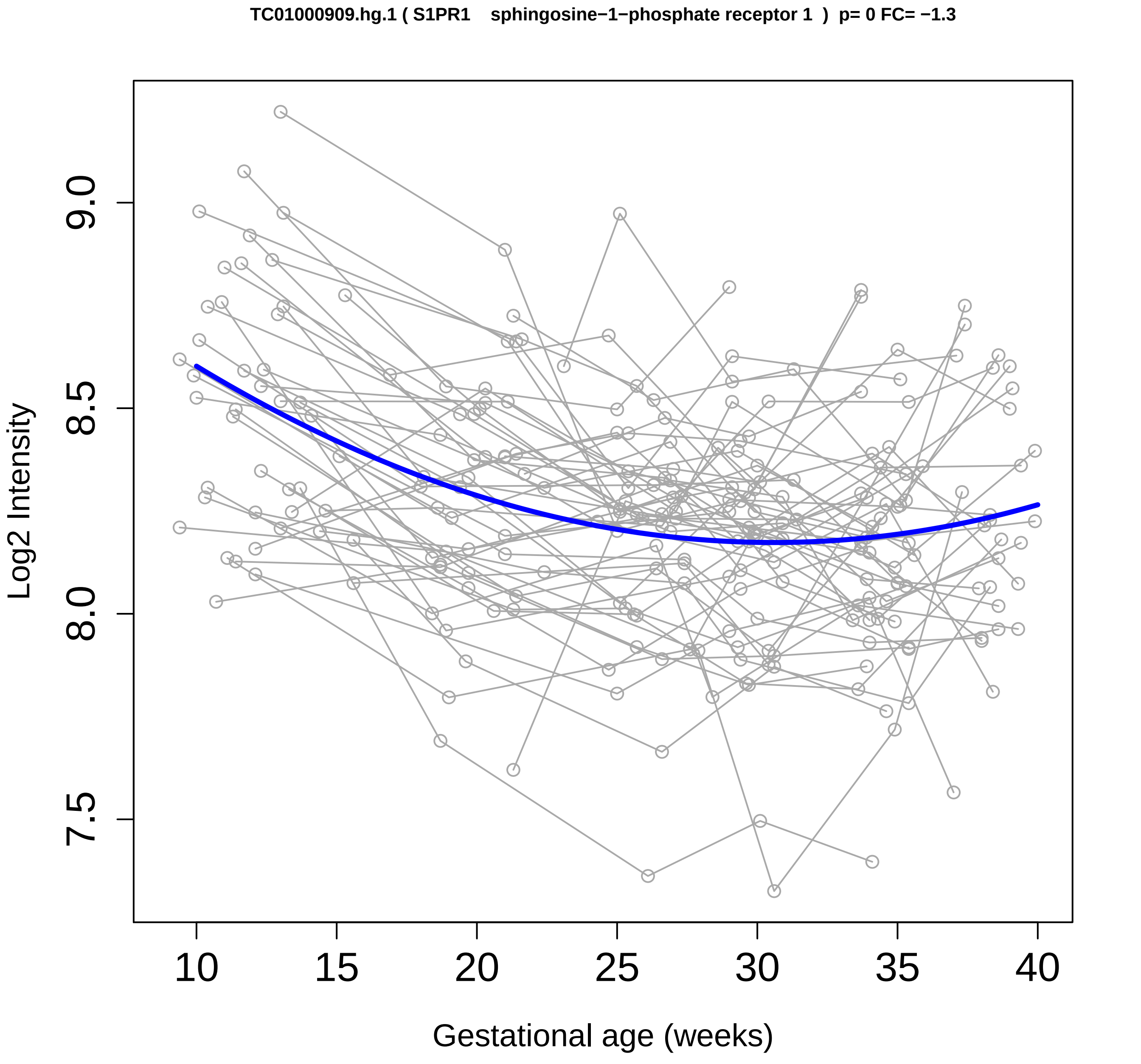
TC01000909.hg.1 (S1PR1 sphingosine−1−phosphate receptor 1) p= 0 FC= −1.3
Log2 Intensity
9.0
8.5
8.0
7.5
10
15
20
25
30
35
40
Gestational age (weeks)

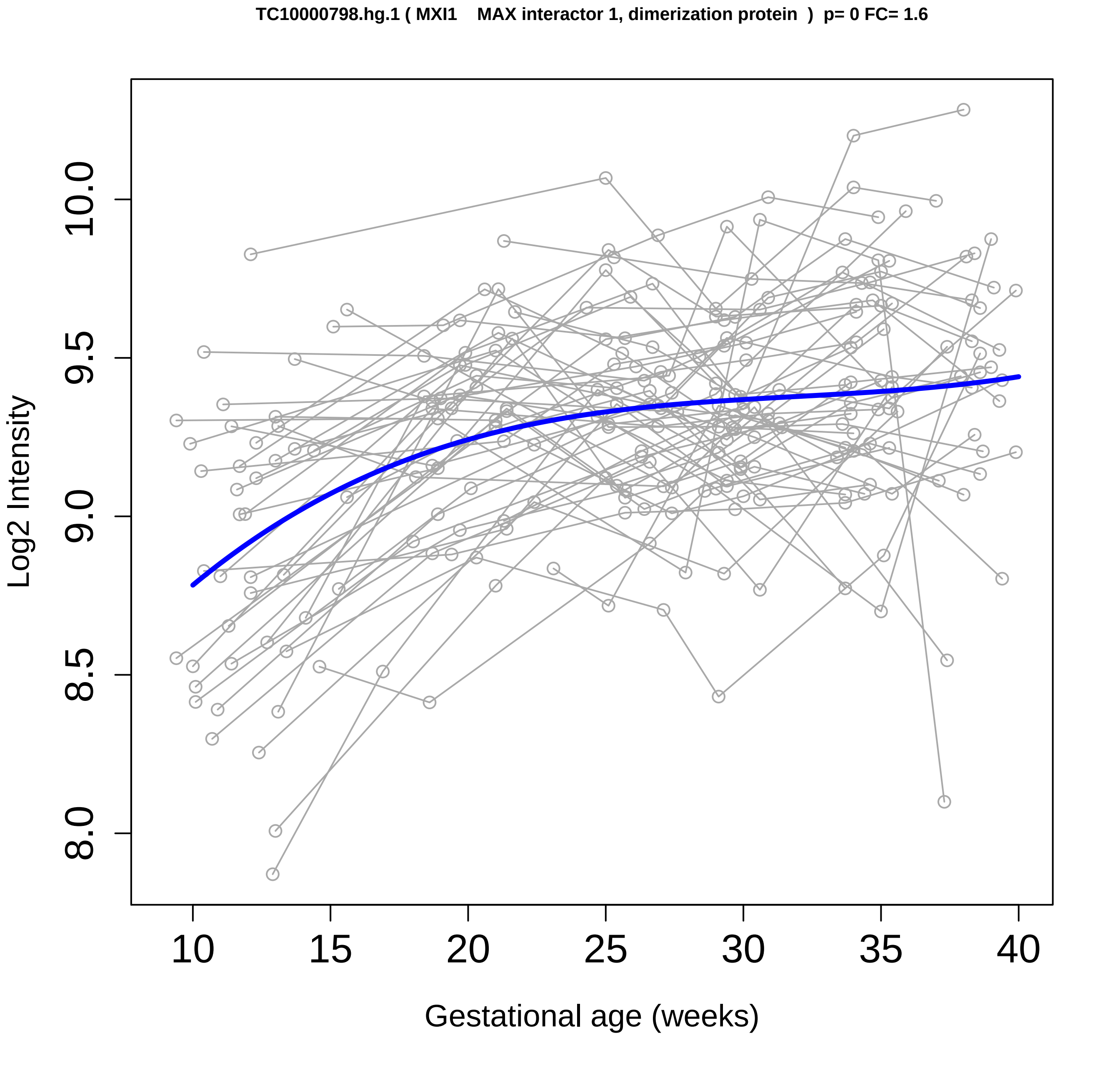
TC10000798.hg.1 (MXI1   MAX interactor 1, dimerization protein) p= 0 FC= 1.6
Log2 Intensity
8.0
8.5
9.0
9.5
10.0
10
15
20
25
30
35
40
Gestational age (weeks)

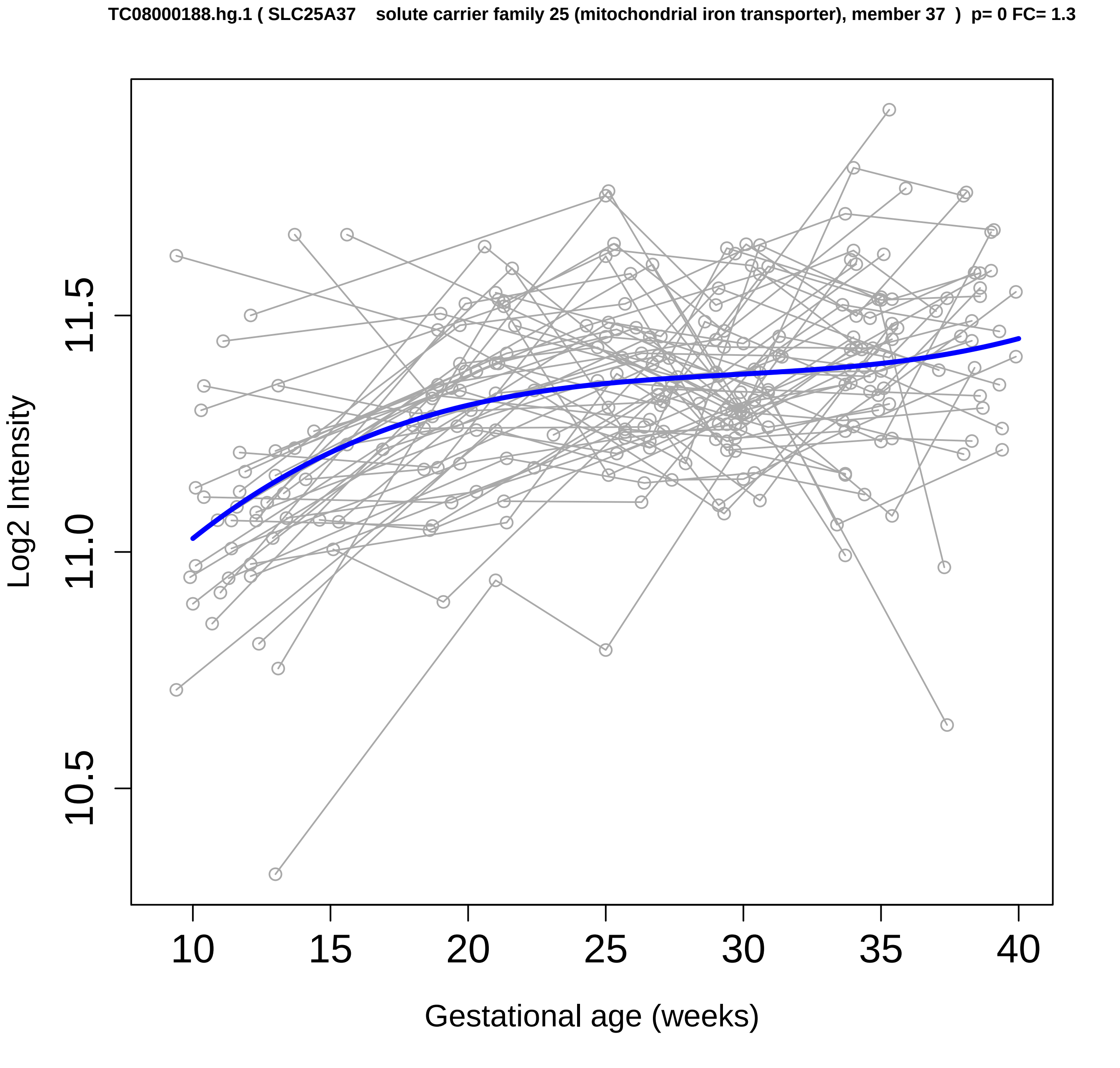
TC08000188.hg.1 (SLC25A37 solute carrier family 25 (mitochondrial iron transporter), member 37) p= 0 FC= 1.3
Log2 Intensity
11.5
11.0
10.5
10
15
20
25
30
35
40
Gestational age (weeks)

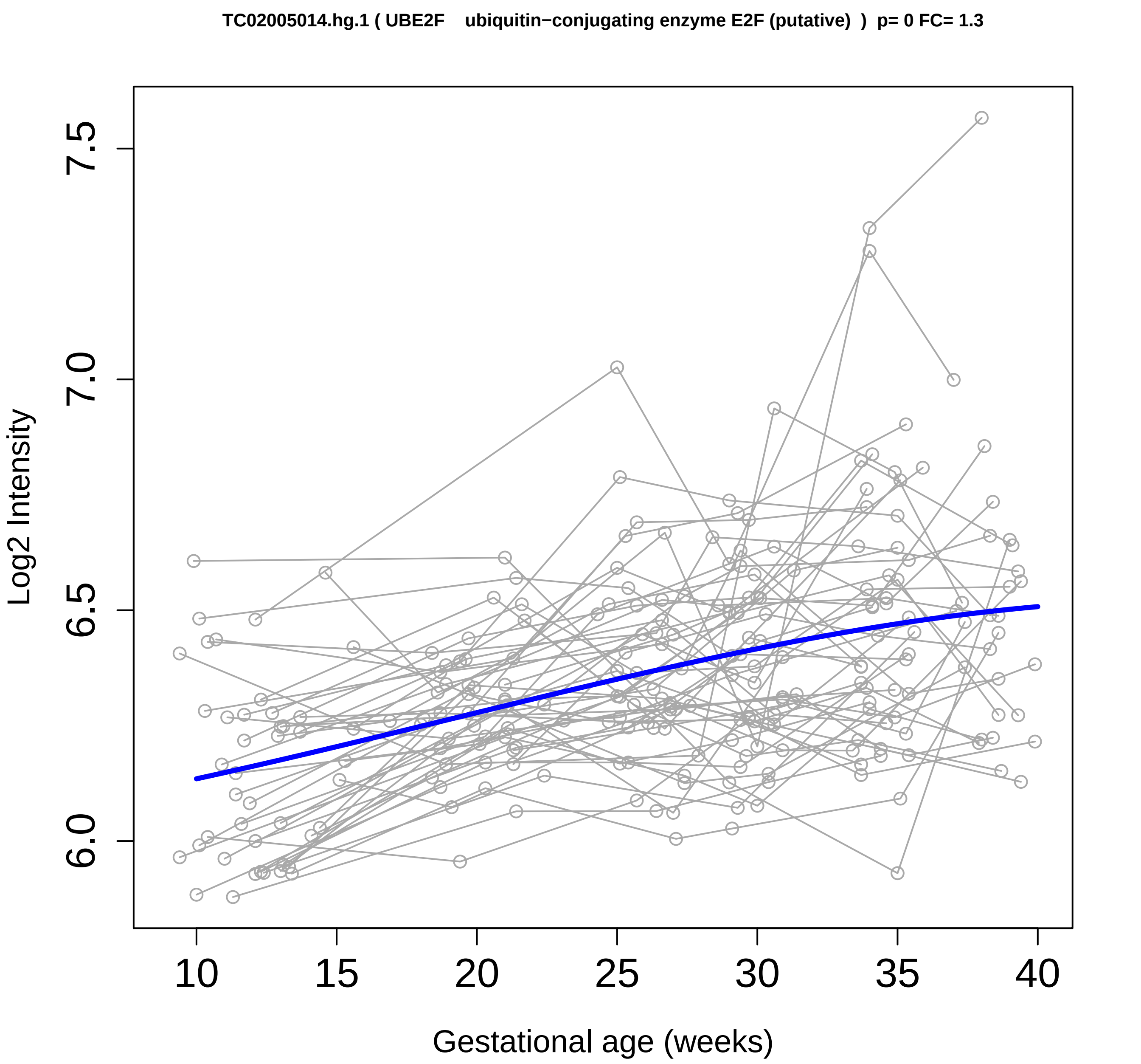

TC02005014.hg.1 (UBE2F ubiquitin−conjugating enzyme E2F (putative)) p= 0 FC= 1.3
Log2 Intensity
7.5
7.0
6.5
6.0
10
15
20
25
30
35
40
Gestational age (weeks)

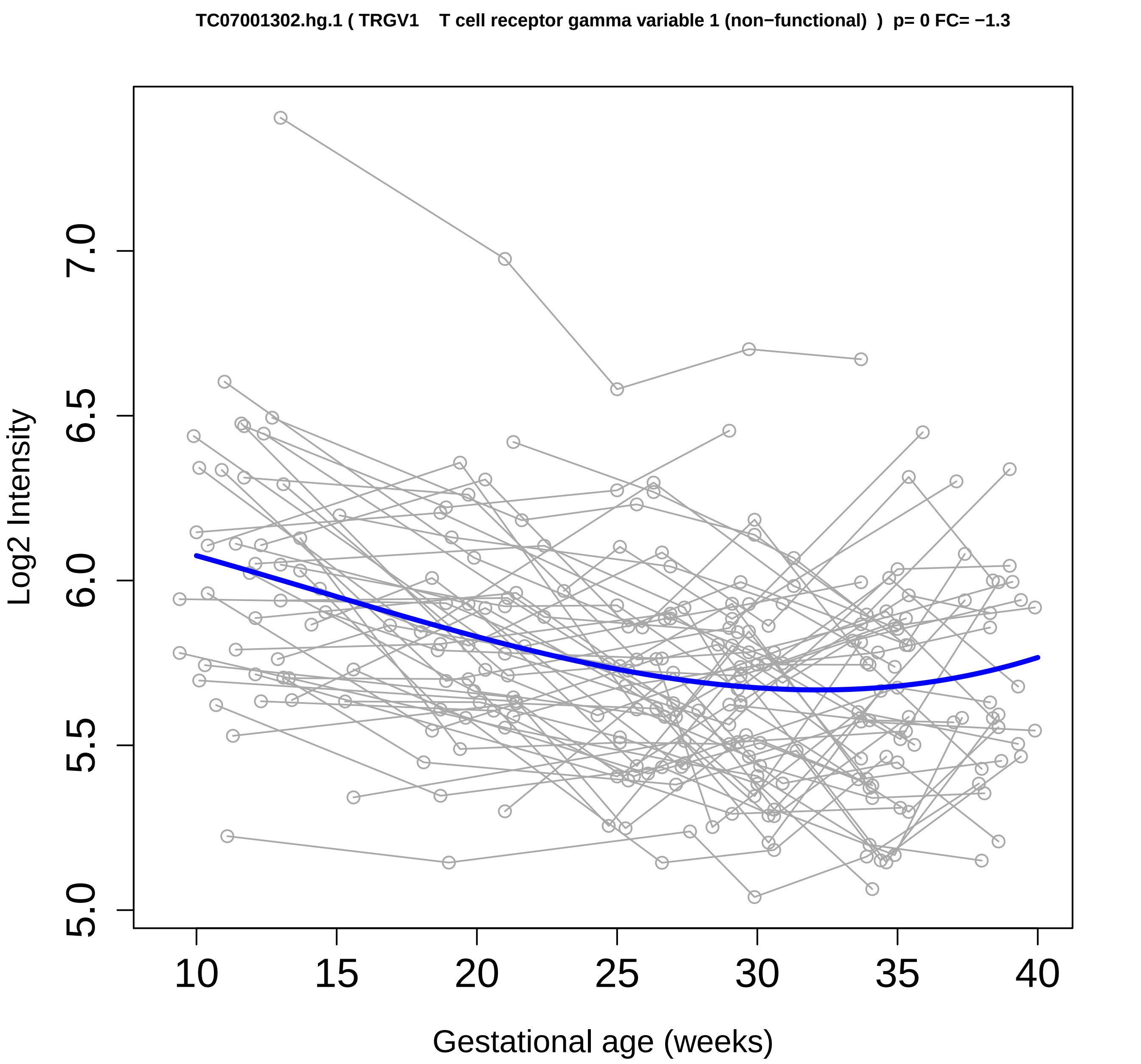
TC07001302.hg.1 (TRGV1   T cell receptor gamma variable 1 (non−functional)) p= 0 FC= −1.3
Log2 Intensity
5.0
5.5
6.0
6.5
7.0
10
15
20
25
30
35
40
Gestational age (weeks)

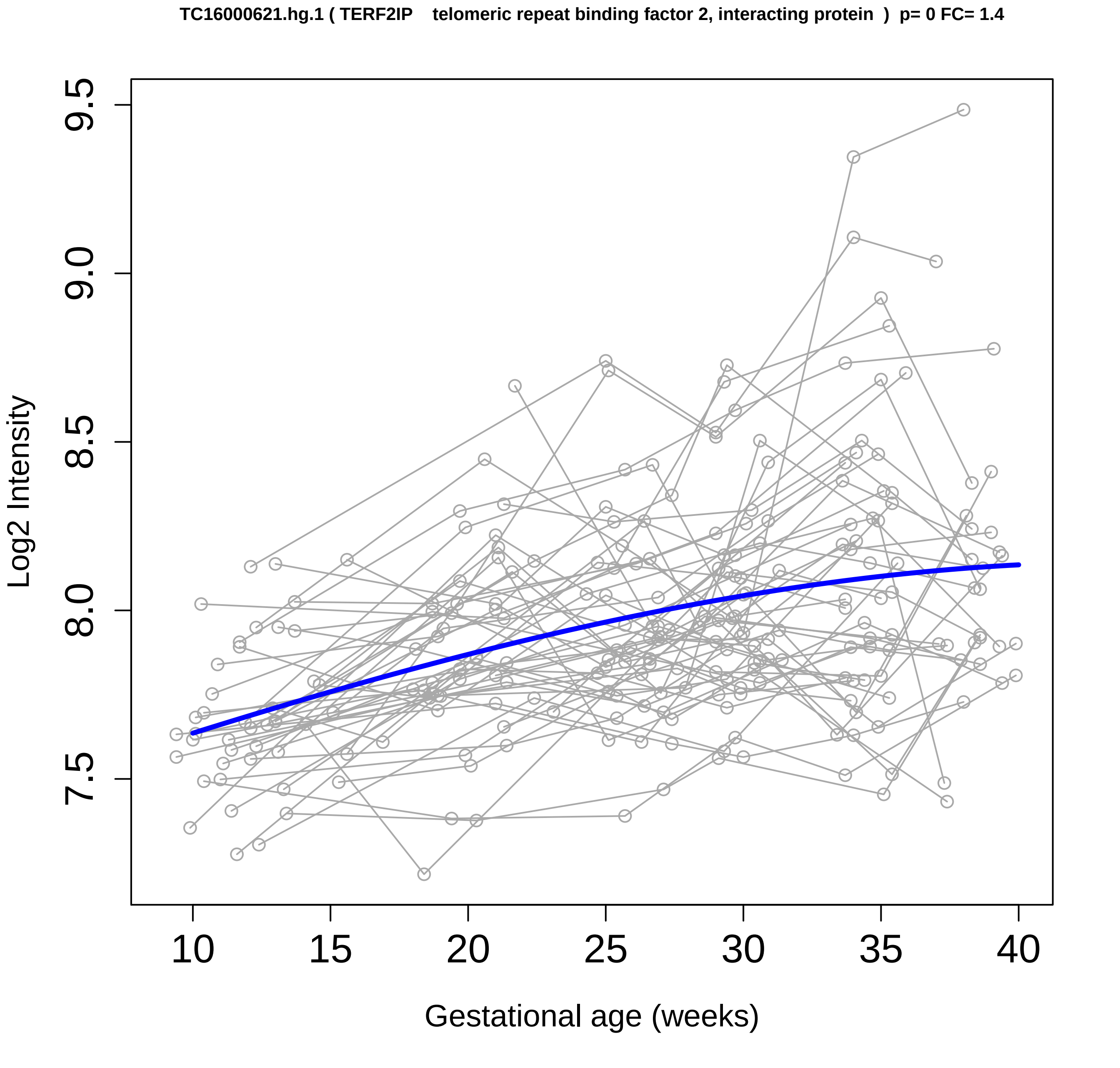

TC16000621.hg.1 (TERF2IP telomeric repeat binding factor 2, interacting protein) p= 0 FC= 1.4
Log2 Intensity
7.5
8.0
8.5
9.0
9.5
10
15
20
25
30
35
40
Gestational age (weeks)

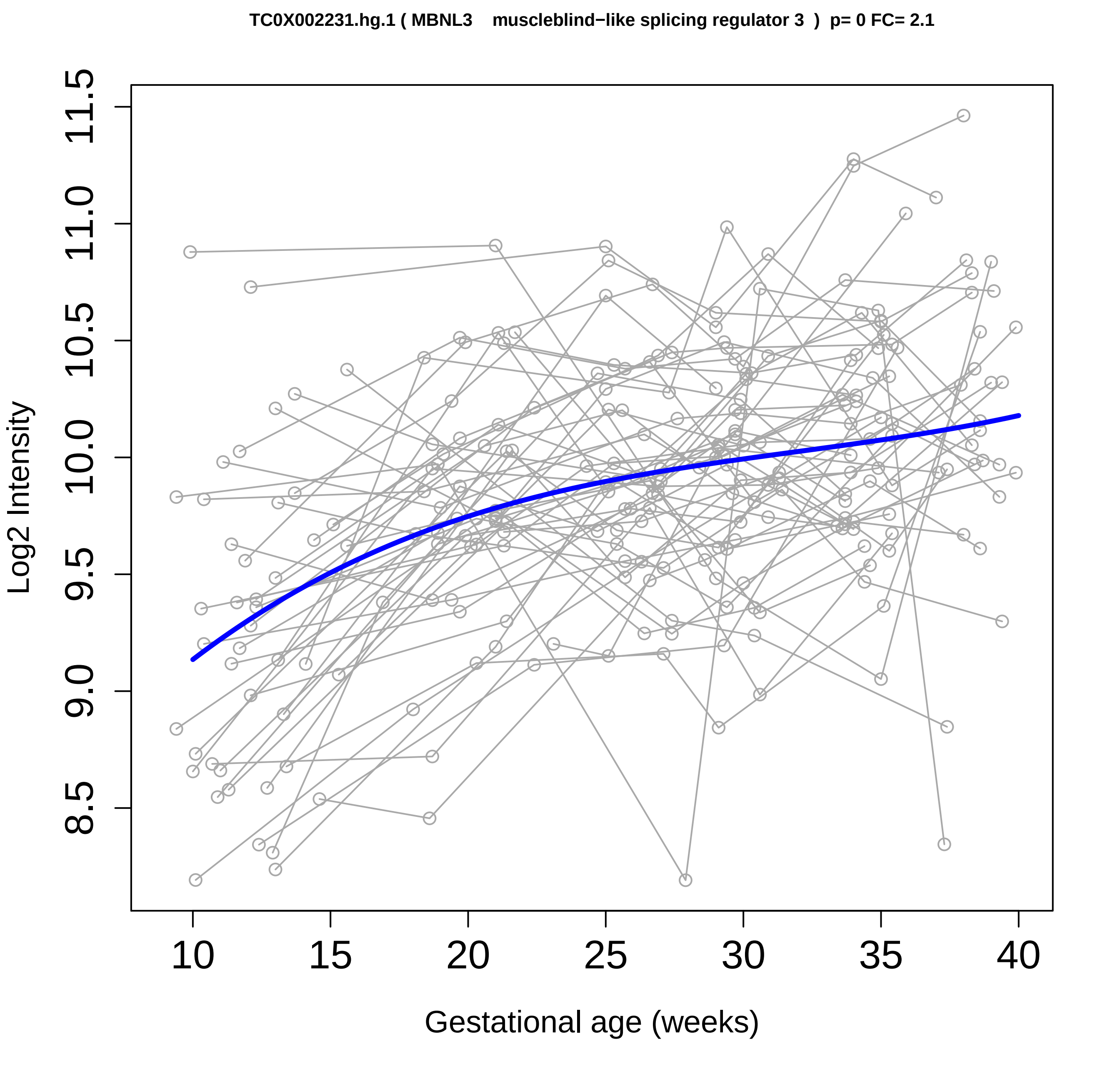

TC0X002231.hg.1 (MBNL3   muscleblind−like splicing regulator 3) p= 0 FC= 2.1
Log2 Intensity
8.5
9.0
9.5
10.0
10.5
11.0
11.5
10
15
20
25
30
35
40
Gestational age (weeks)

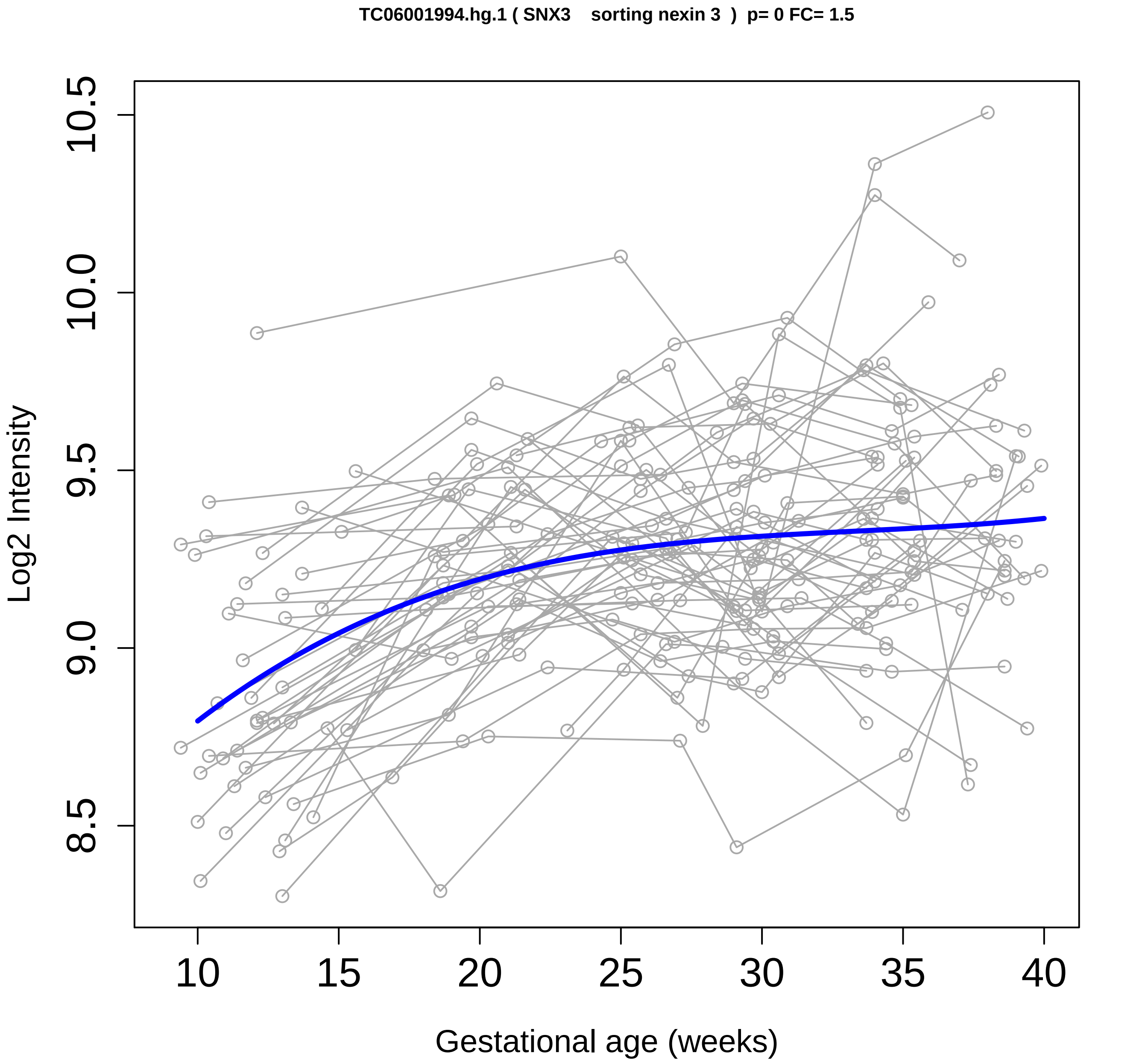

TC06001994.hg.1 (SNX3 sorting nexin 3) p= 0 FC= 1.5
Log2 Intensity
8.5
9.0
9.5
10.0
10.5
10
15
20
25
30
35
40
Gestational age (weeks)

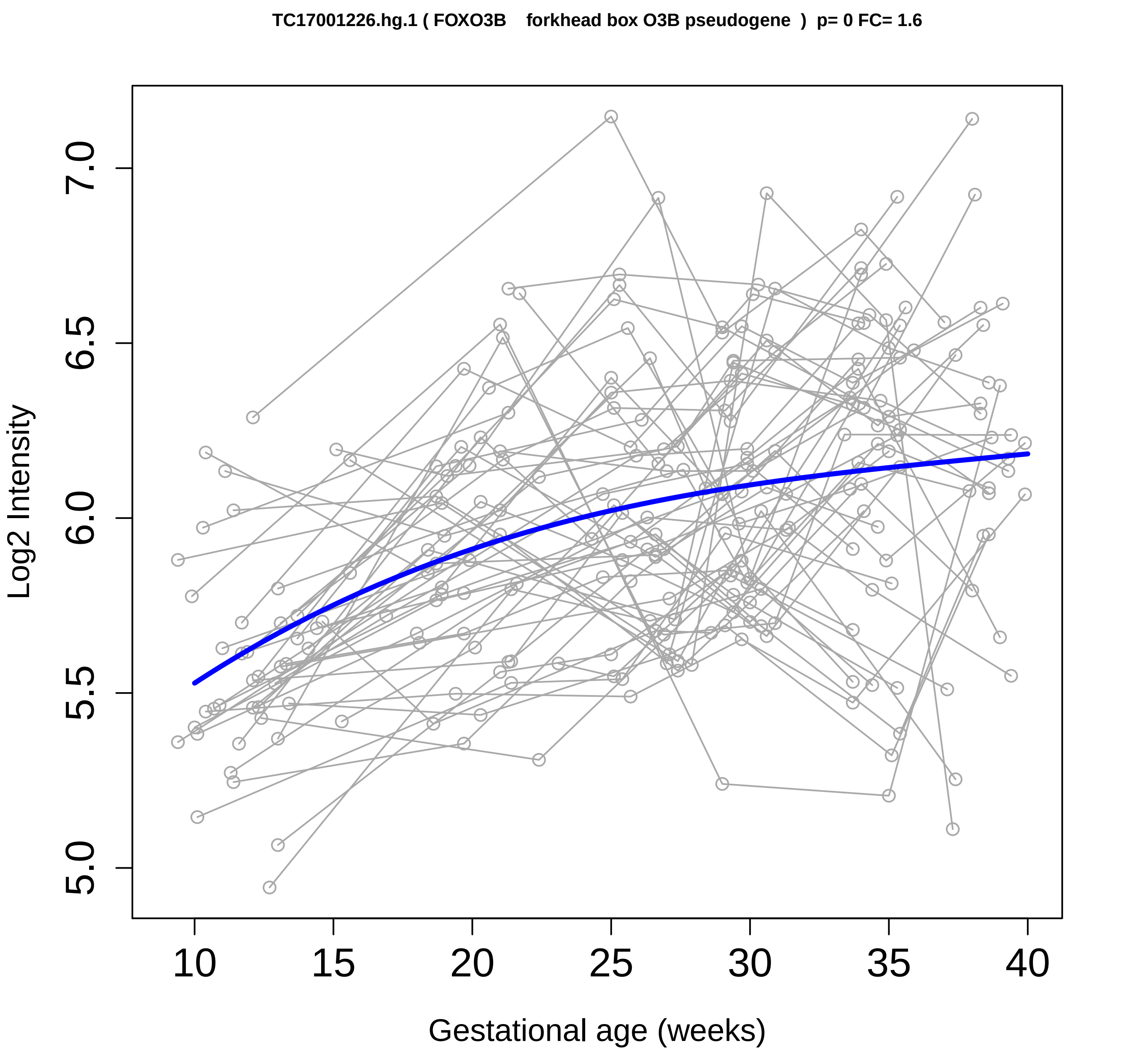

TC17001226.hg.1 (FOXO3B forkhead box O3B pseudogene) p= 0 FC= 1.6
Log2 Intensity
5.0
5.5
6.0
6.5
7.0
10
15
20
25
30
35
40
Gestational age (weeks)

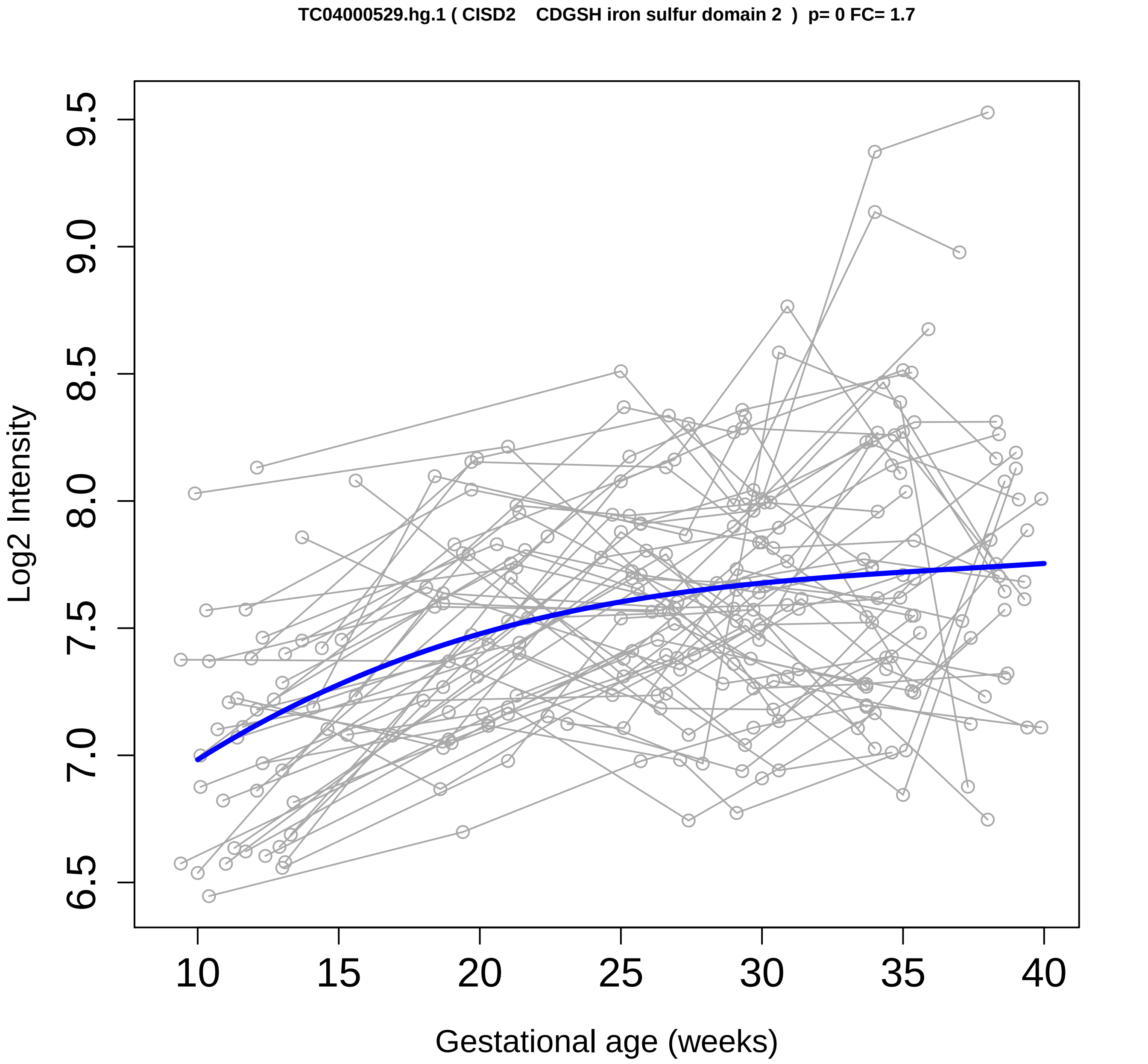
TC04000529.hg.1 (CISD2 CDGSH iron sulfur domain 2) p= 0 FC= 1.7
Log2 Intensity
6.5
7.0
7.5
8.0
8.5
9.0
9.5
10
15
20
25
30
35
40
Gestational age (weeks)

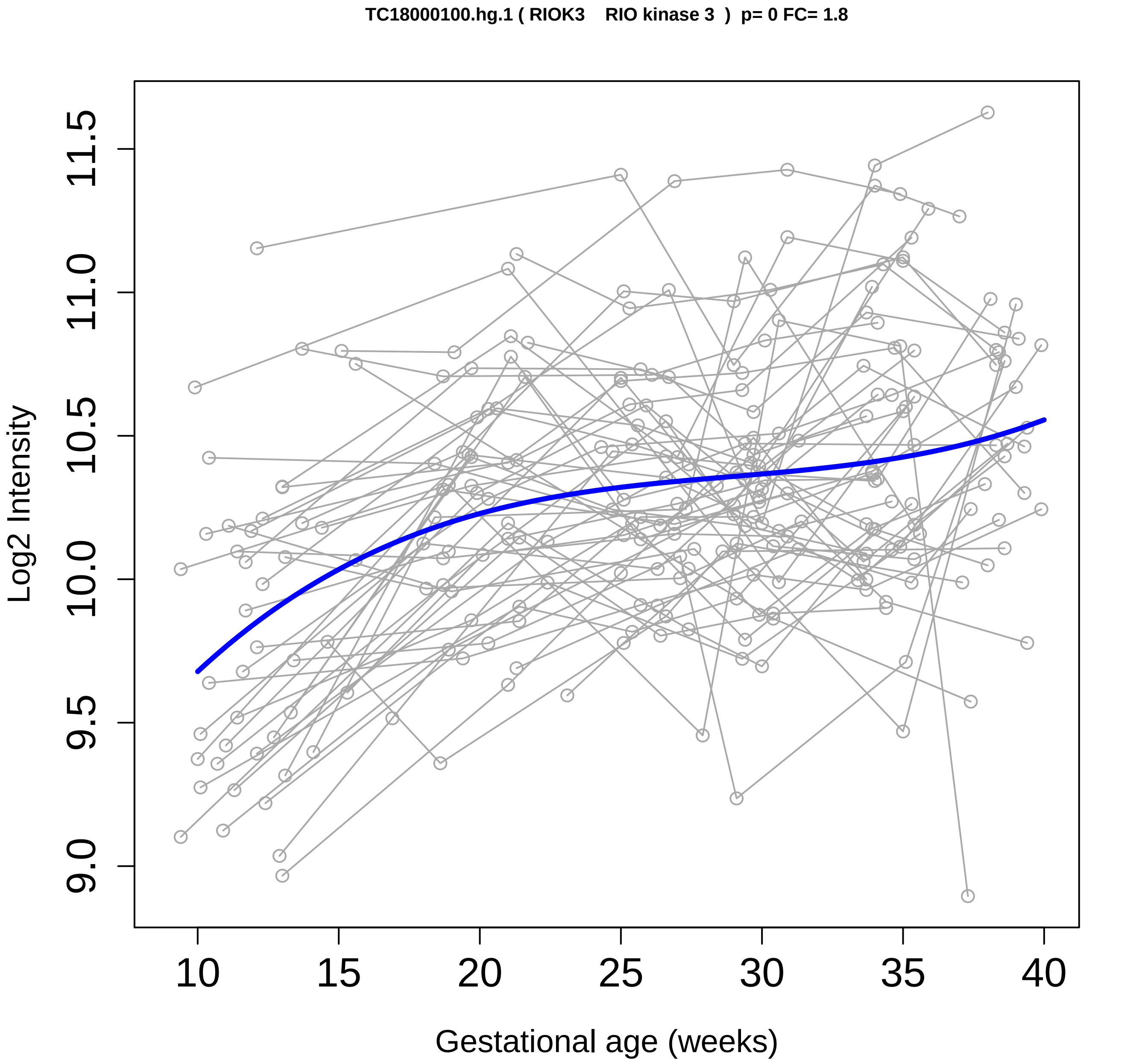
TC18000100.hg.1 (RIOK3 RIO kinase 3) p= 0 FC= 1.8
Log2 Intensity
9.0
9.5
10.0
10.5
11.0
11.5
10
15
20
25
30
35
40
Gestational age (weeks)

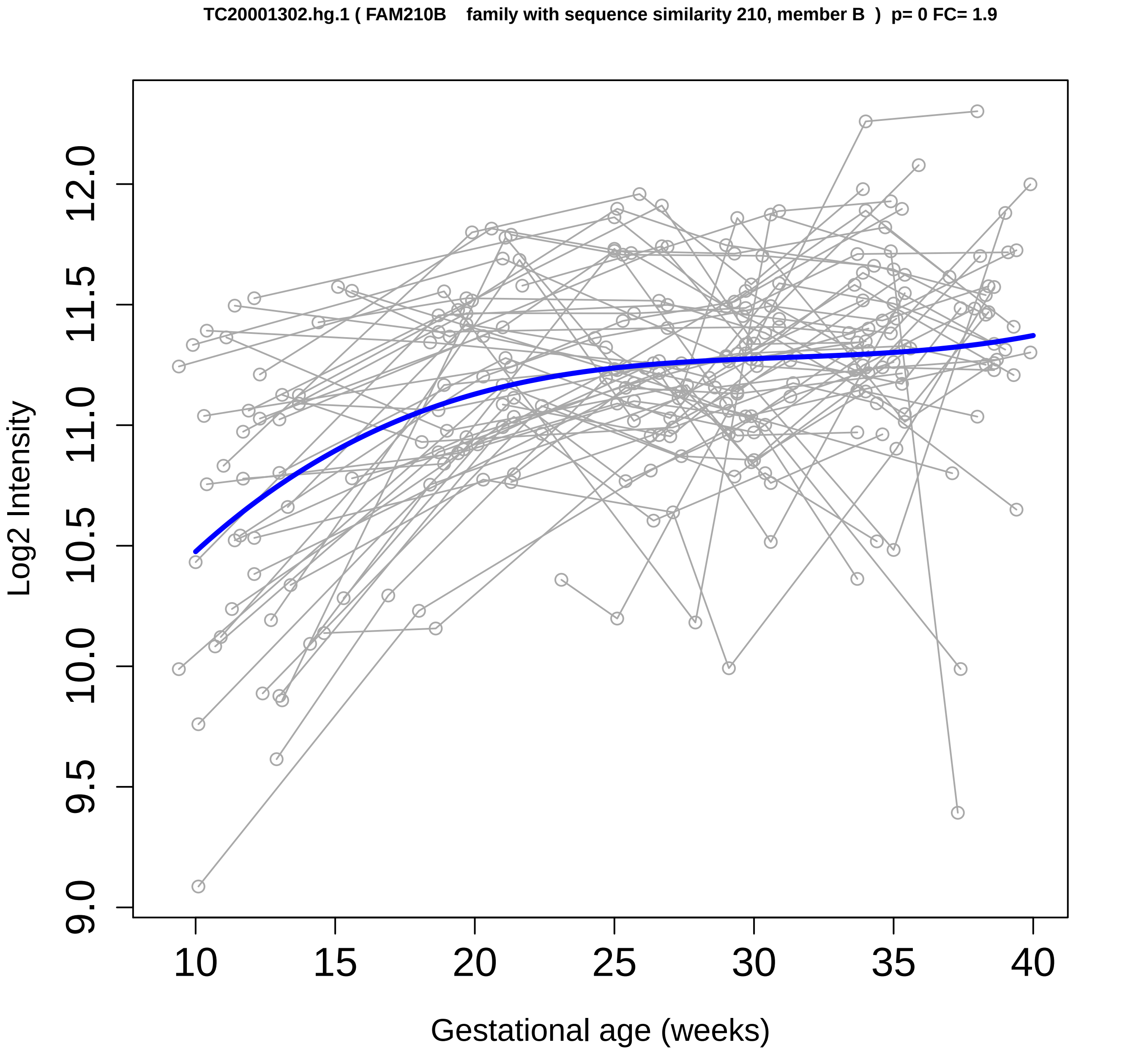

TC20001302.hg.1 (FAM210B family with sequence similarity 210, member B) p= 0 FC= 1.9
Log2 Intensity
9.0
9.5
10.0
10.5
11.0
11.5
12.0
10
15
20
25
30
35
40
Gestational age (weeks)

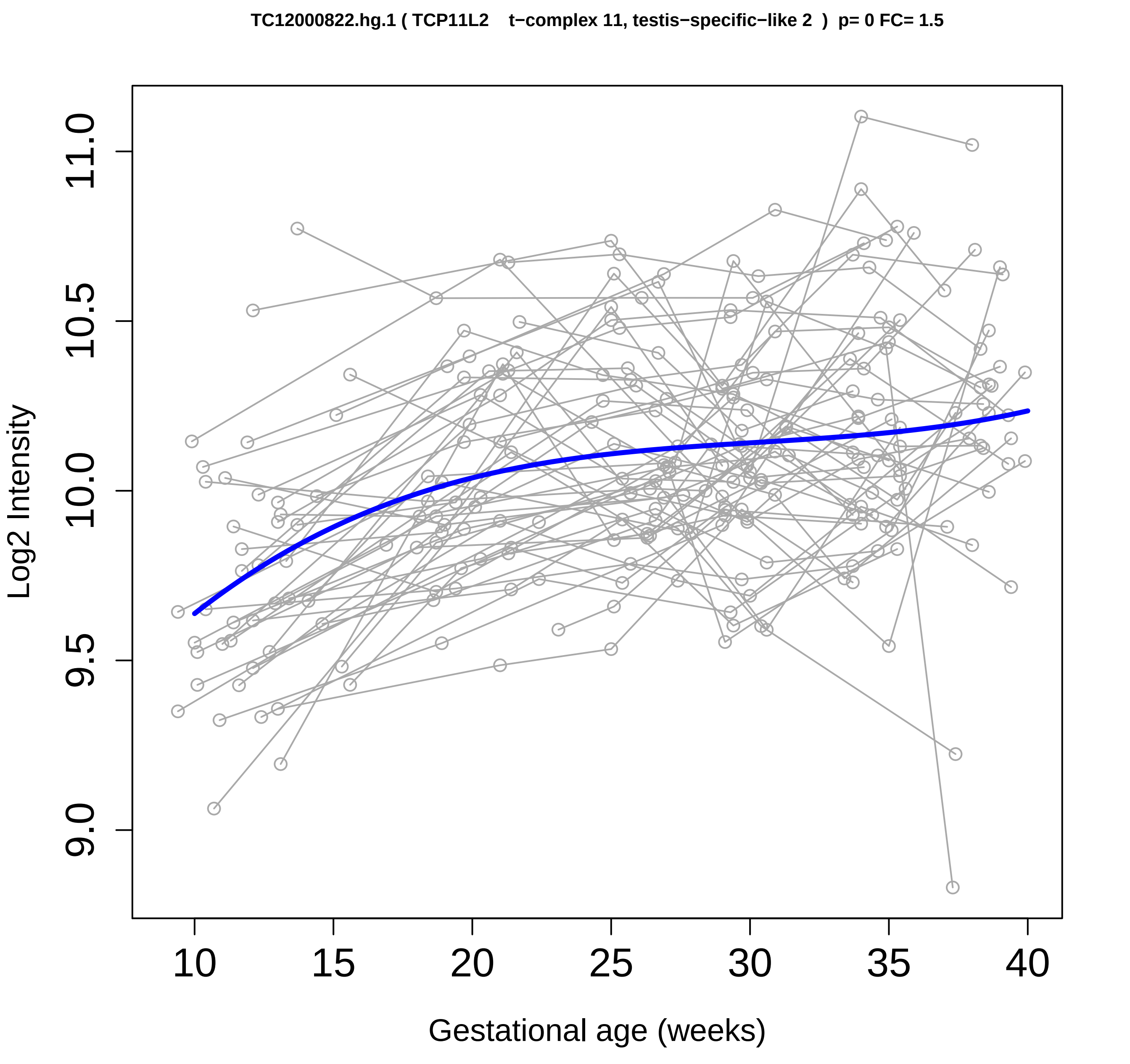
TC12000822.hg.1 (TCP11L2 t−complex 11, testis−specific−like 2) p= 0 FC= 1.5
Log2 Intensity
9.0
9.5
10.0
10.5
11.0
10
15
20
25
30
35
40
Gestational age (weeks)

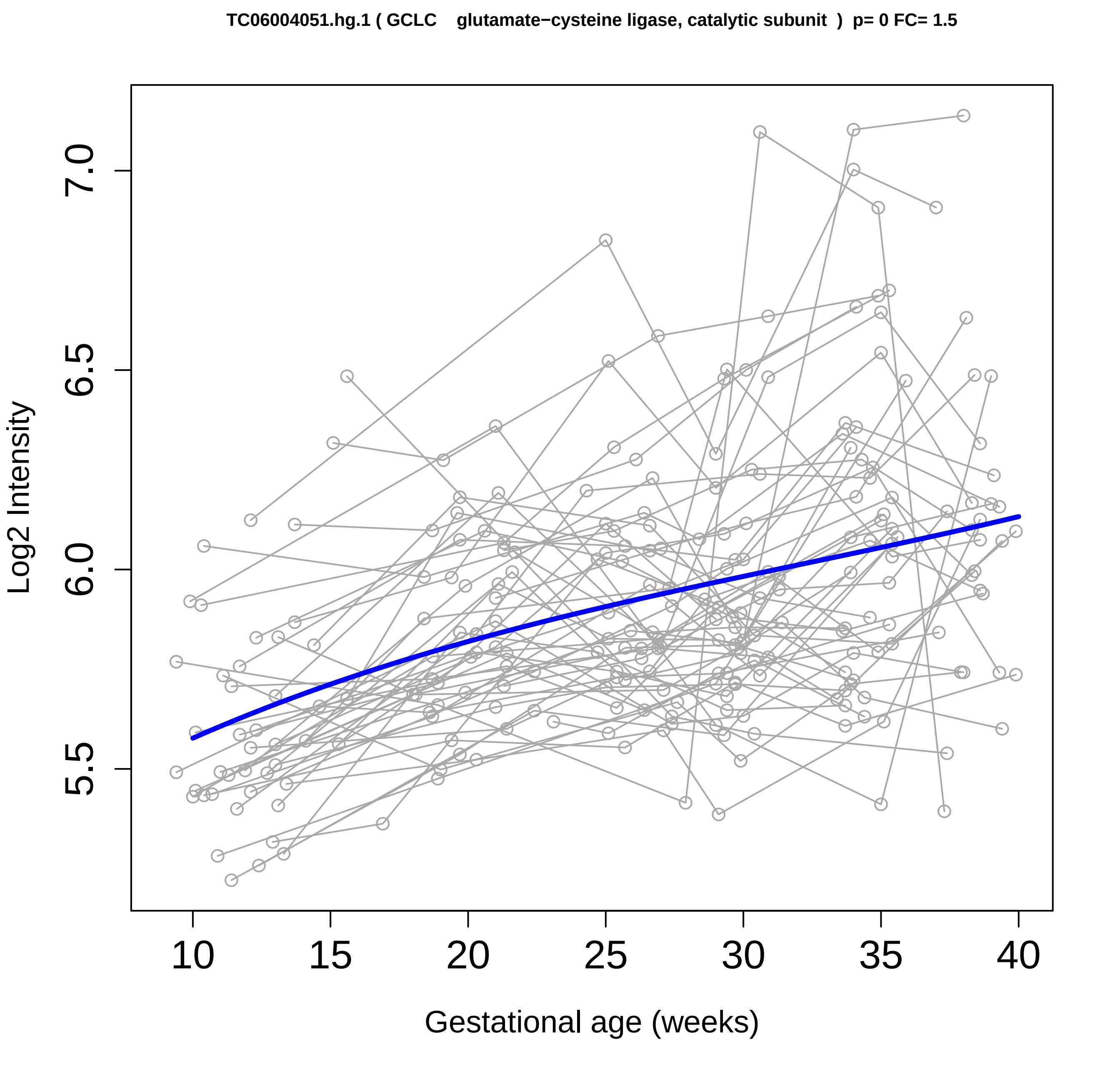
TC06004051.hg.1 (GCLC glutamate−cysteine ligase, catalytic subunit) p= 0 FC= 1.5
Log2 Intensity
5.5
6.0
6.5
7.0
10
15
20
25
30
35
40
Gestational age (weeks)

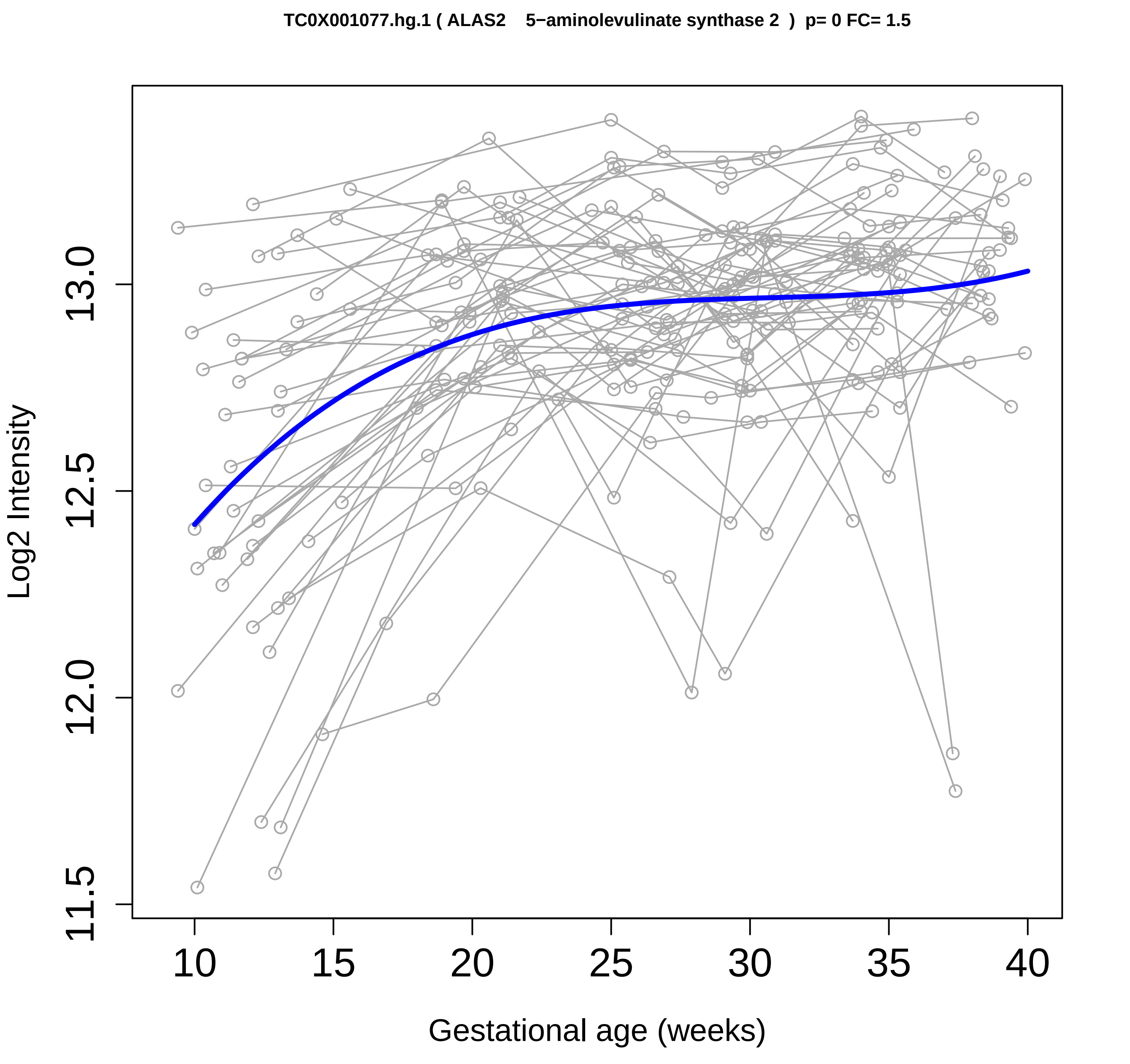
TC0X001077.hg.1 (ALAS2 5−aminolevulinate synthase 2) p= 0 FC= 1.5
Log2 Intensity
11.5
12.0
12.5
13.0
10
15
20
25
30
35
40
Gestational age (weeks)

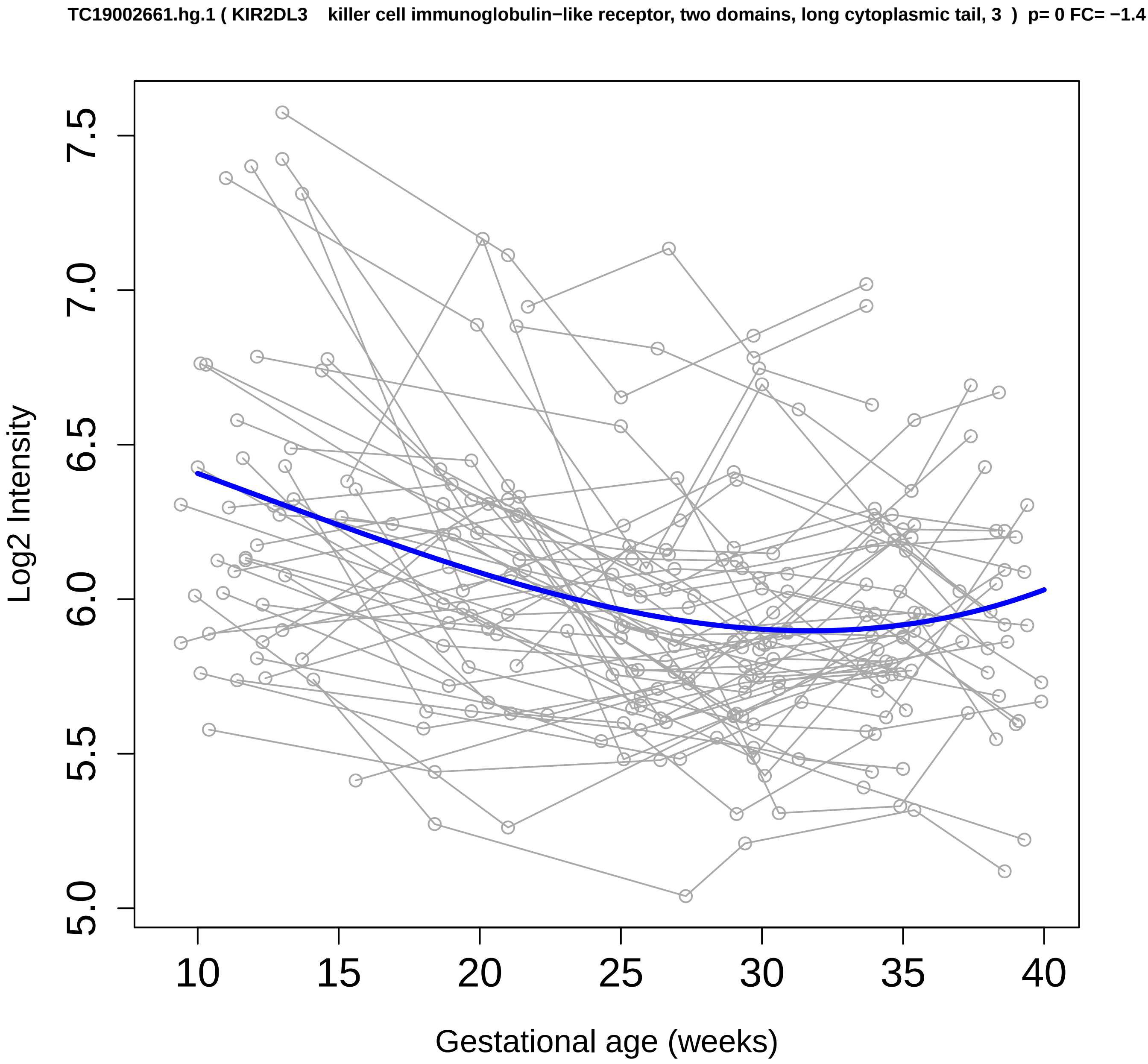
TC19002661.hg.1 (KIR2DL3 killer cell immunoglobulin−like receptor, two domains, long cytoplasmic tail, 3) p= 0 FC= −1.4
Log2 Intensity
7.5
7.0
6.5
6.0
5.5
5.0
10
15
20
25
30
35
40
Gestational age (weeks)

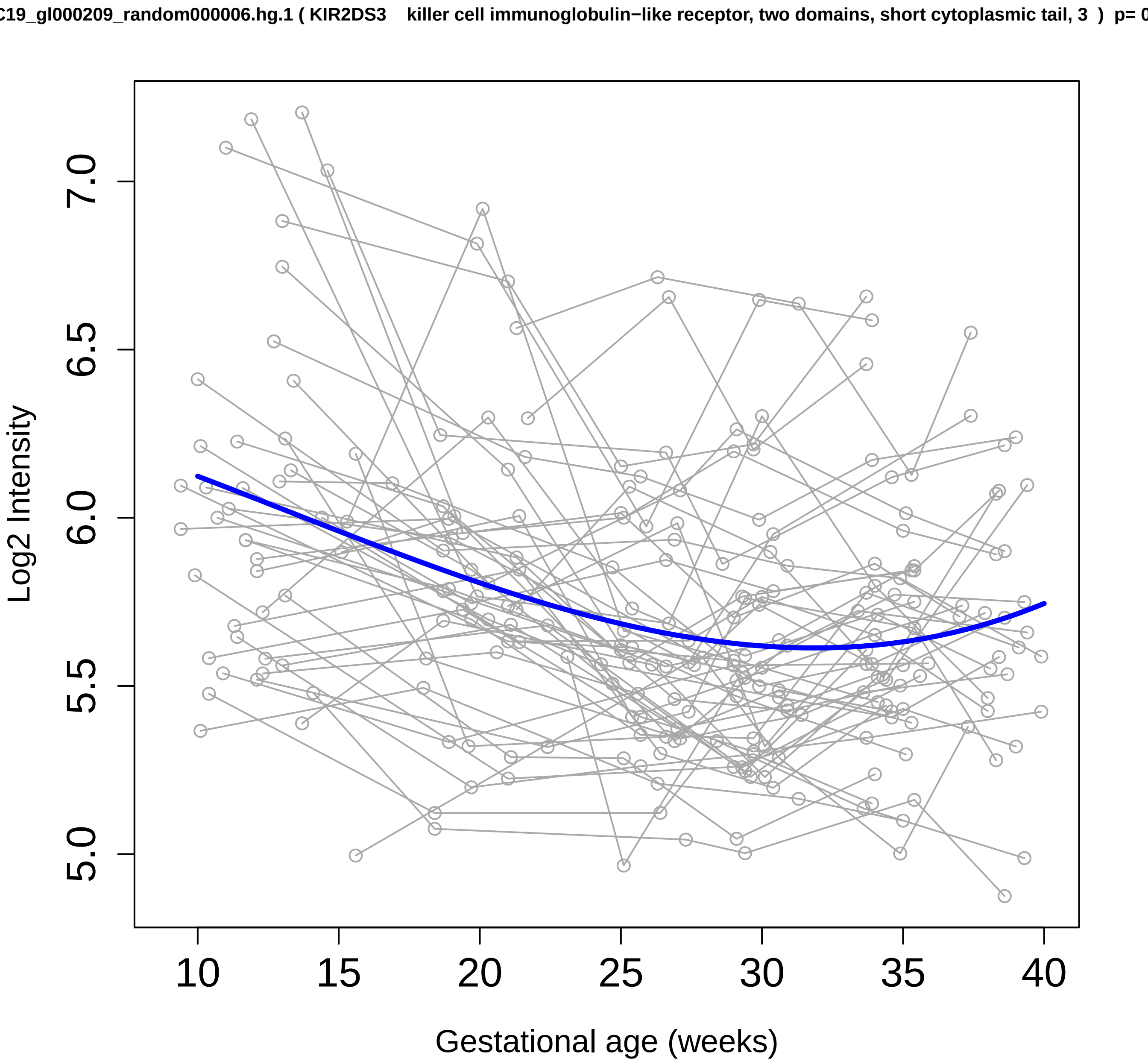

C19_gl000209_random000006.hg.1 (KIR2DS3 killer cell immunoglobulin−like receptor, two domains, short cytoplasmic tail, 3) p= 0
Log2 Intensity
7.0
6.5
6.0
5.5
5.0
10
15
20
25
30
35
40
Gestational age (weeks)

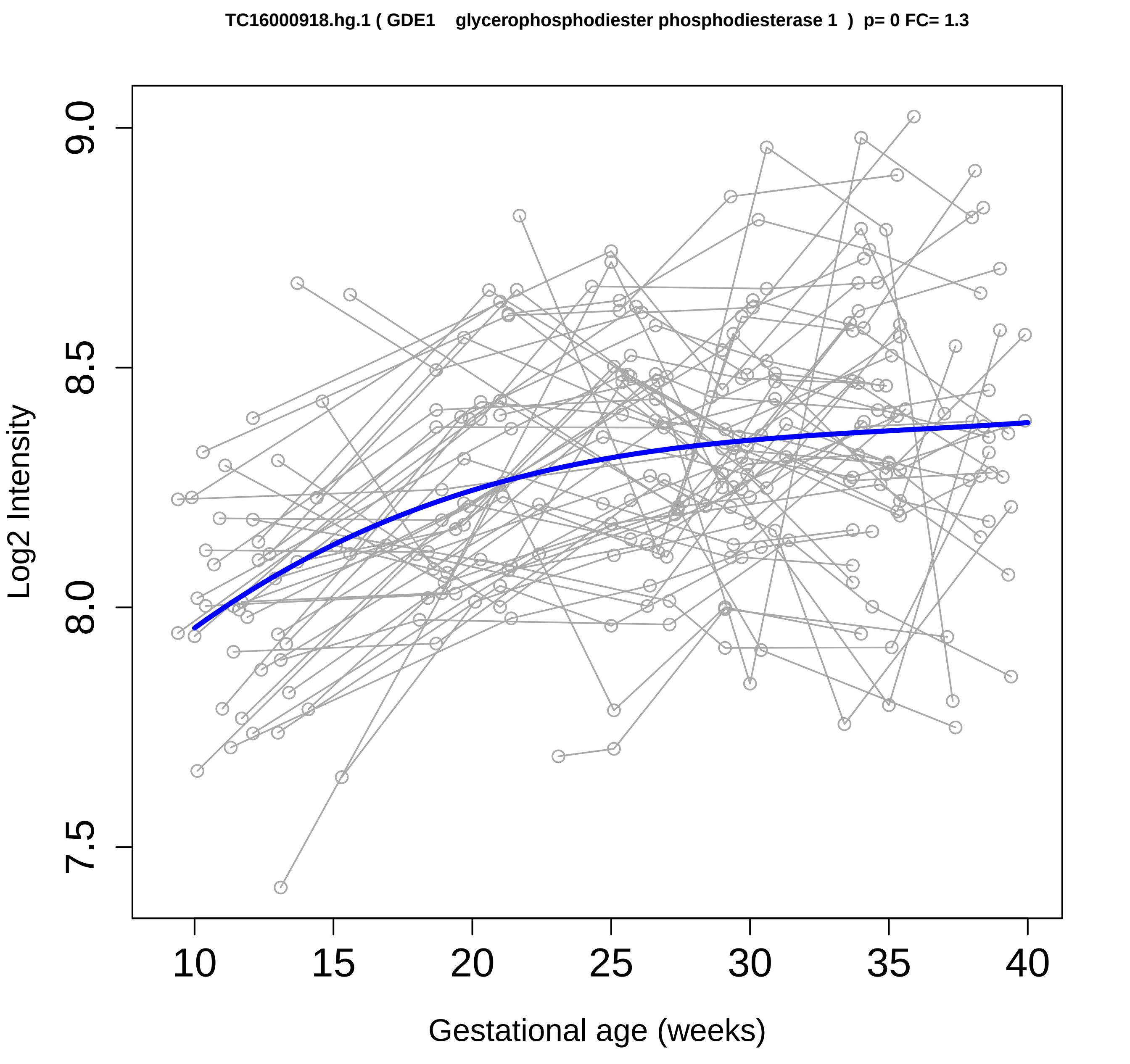
TC16000918.hg.1 (GDE1 glycerophosphodiester phosphodiesterase 1) p= 0 FC= 1.3
Log2 Intensity
9.0
8.5
8.0
7.5
10
15
20
25
30
35
40
Gestational age (weeks)

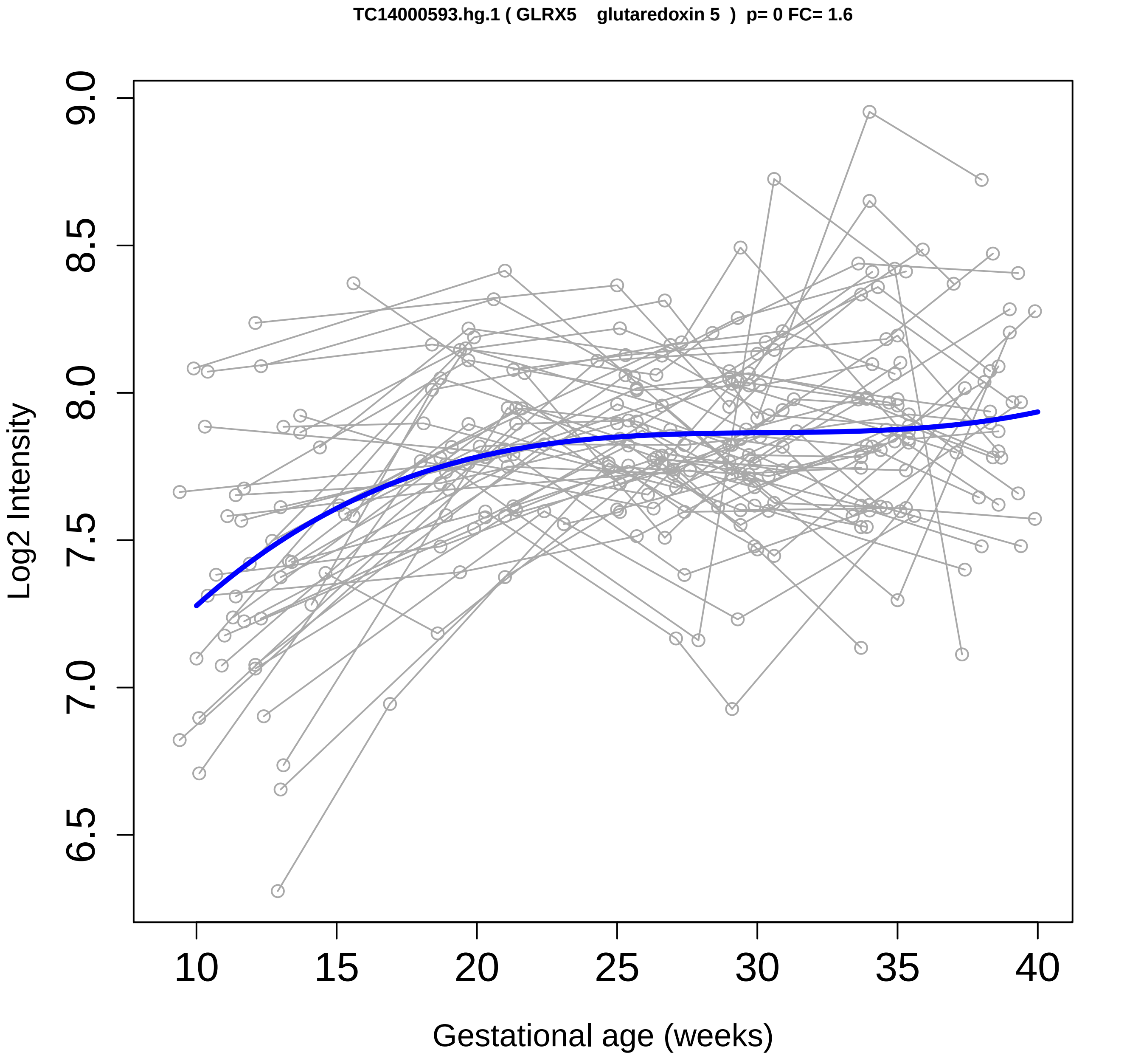

TC14000593.hg.1 (GLRX5 glutaredoxin 5) p= 0 FC= 1.6
Log2 Intensity
6.5
7.0
7.5
8.0
8.5
9.0
10
15
20
25
30
35
40
Gestational age (weeks)

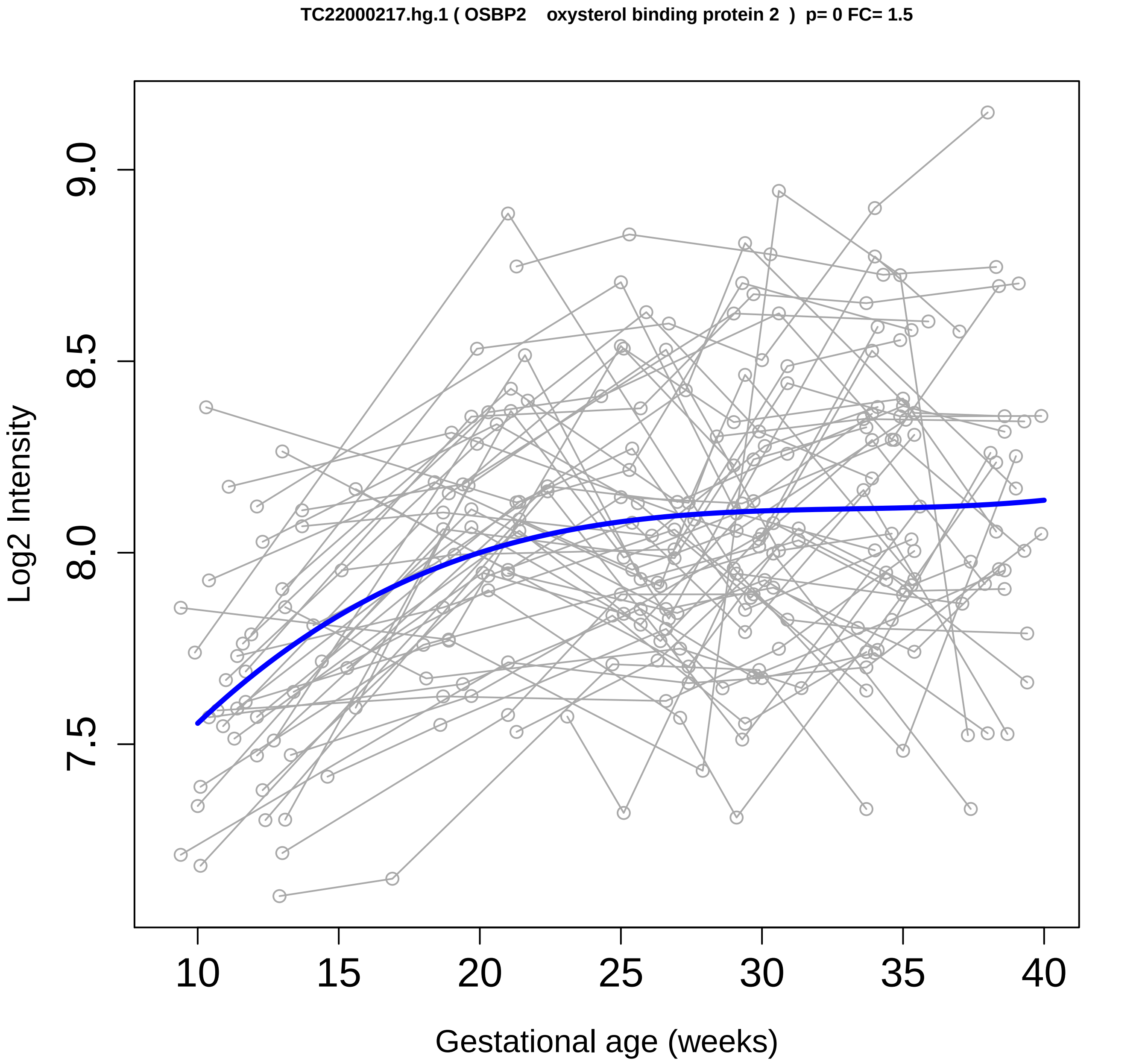

TC22000217.hg.1 (OSBP2   oxysterol binding protein 2) p= 0 FC= 1.5
Log2 Intensity
9.0
8.5
8.0
7.5
10
15
20
25
30
35
40
Gestational age (weeks)

TC12000724.hg.1 (NUDT4 nudix hydrolase 4) p= 0 FC= 1.5

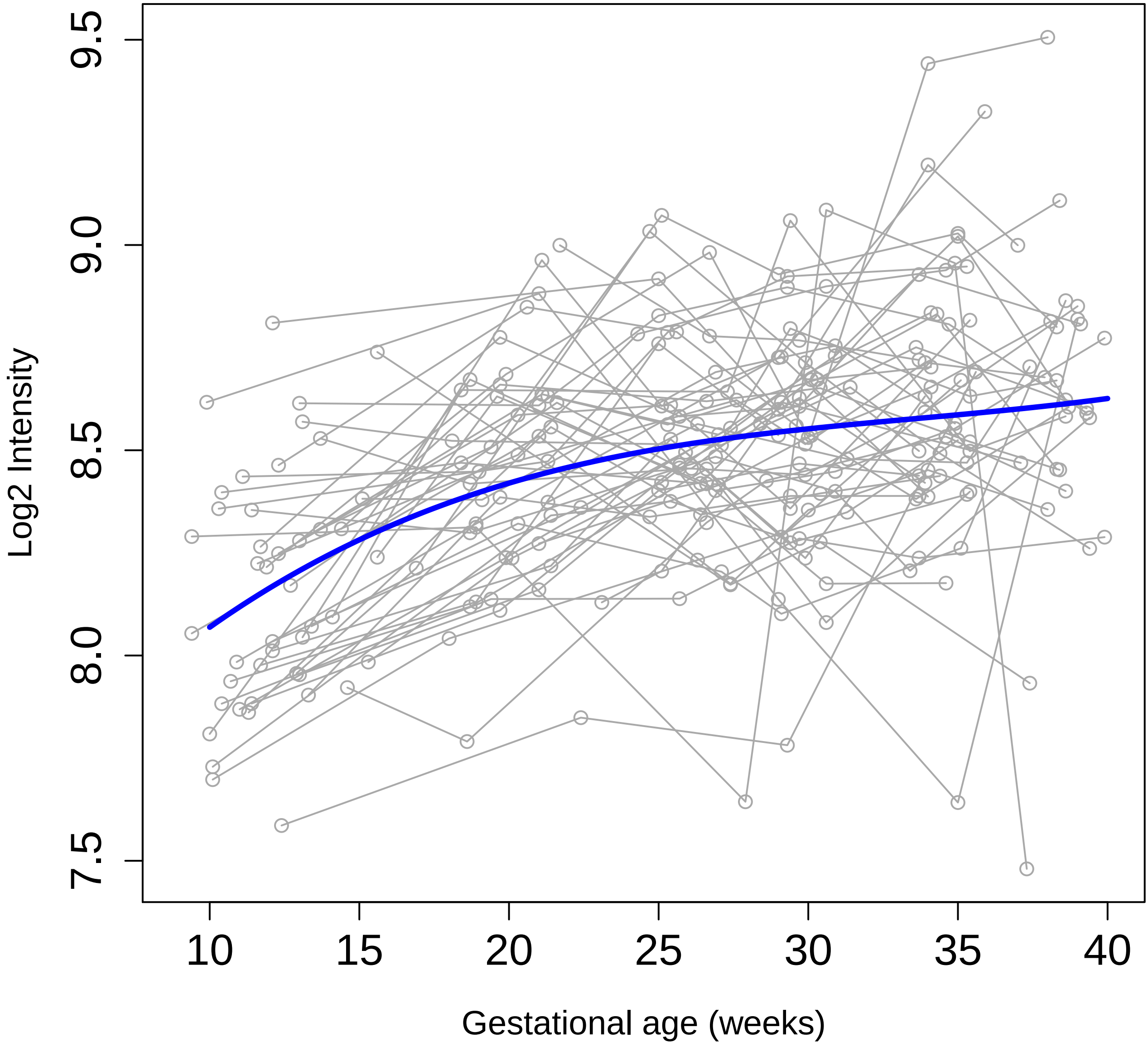

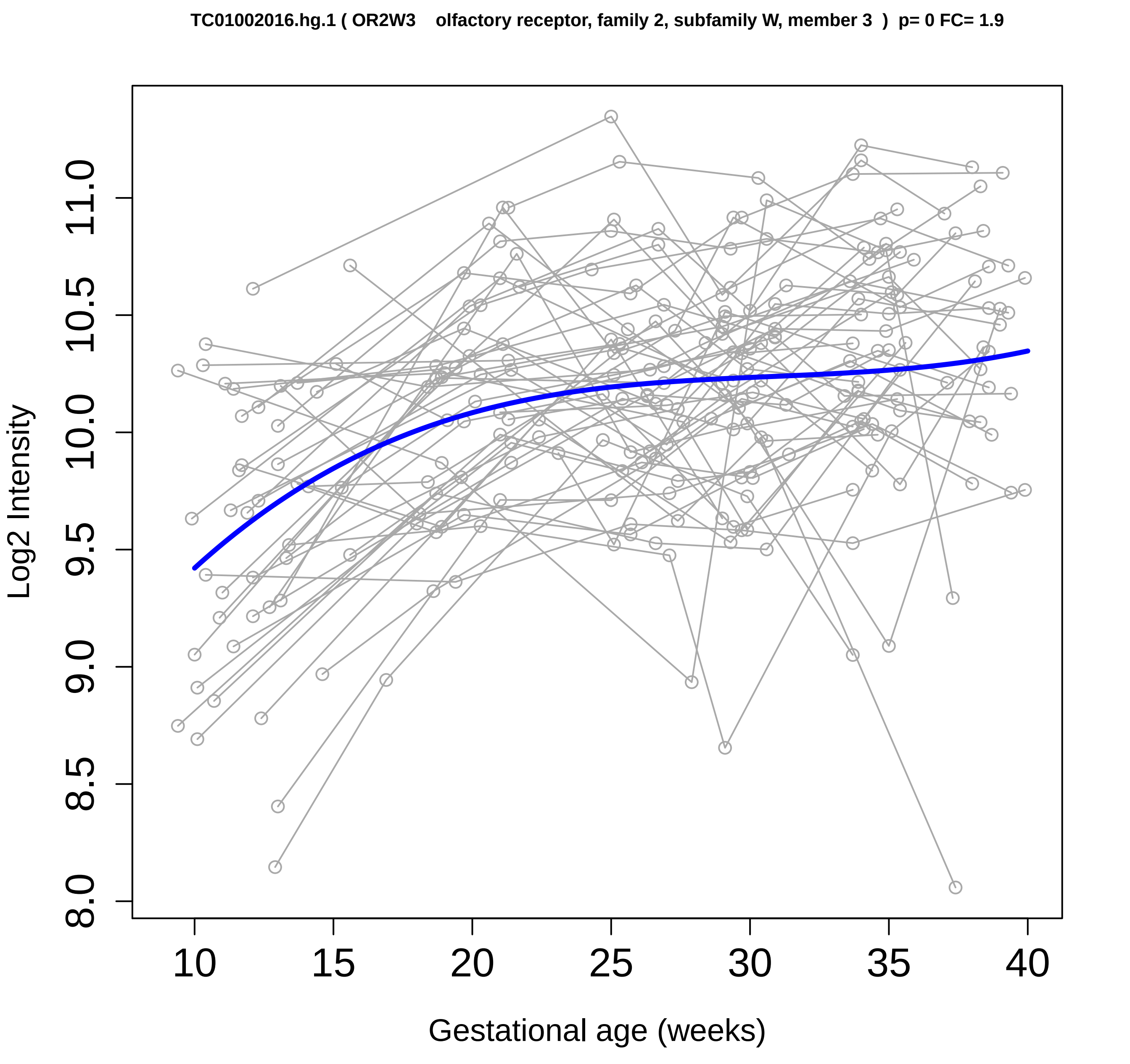

TC01002016.hg.1 (OR2W3 olfactory receptor, family 2, subfamily W, member 3) p= 0 FC= 1.9
Log2 Intensity
8.0
8.5
9.0
9.5
10.0
10.5
11.0
10
15
20
25
30
35
40
Gestational age (weeks)

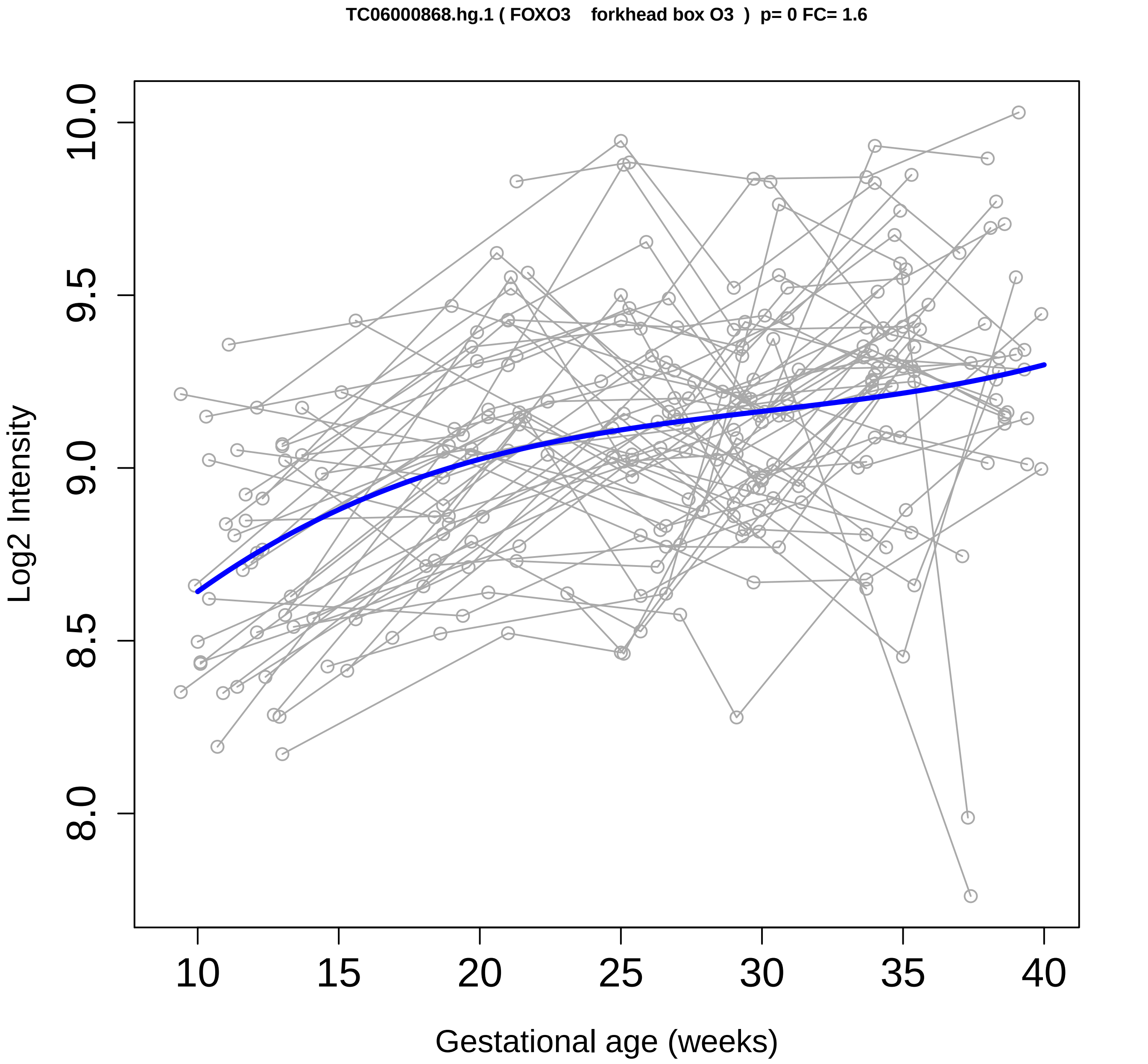
TC06000868.hg.1 (FOXO3   forkhead box O3) p= 0 FC= 1.6
Log2 Intensity
8.0
8.5
9.0
9.5
10.0
10
15
20
25
30
35
40
Gestational age (weeks)

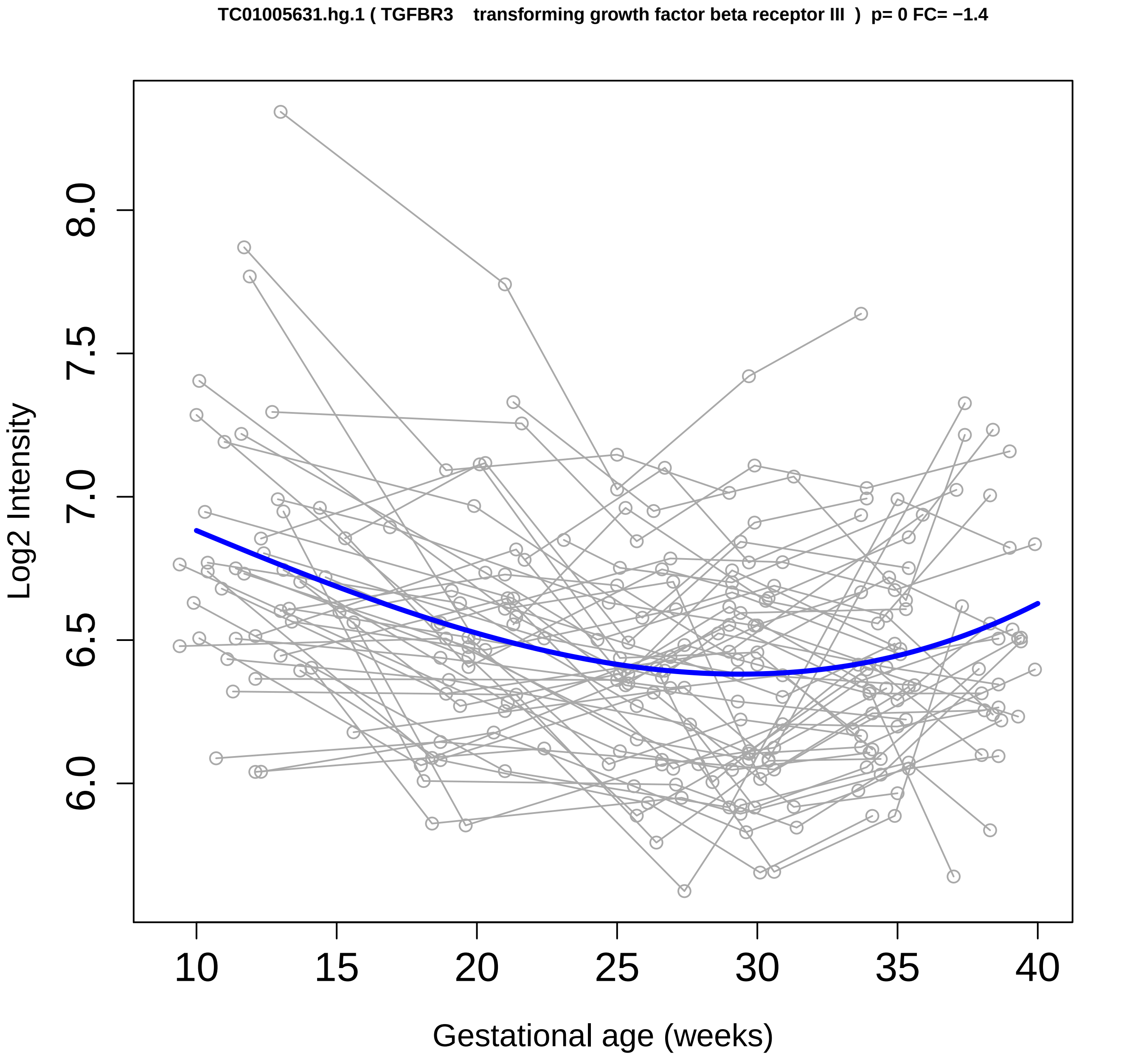

TC01005631.hg.1 (TGFBR3   transforming growth factor beta receptor III) p= 0 FC= −1.4
Log2 Intensity
6.0
6.5
7.0
7.5
8.0
10
15
20
25
30
35
40
Gestational age (weeks)

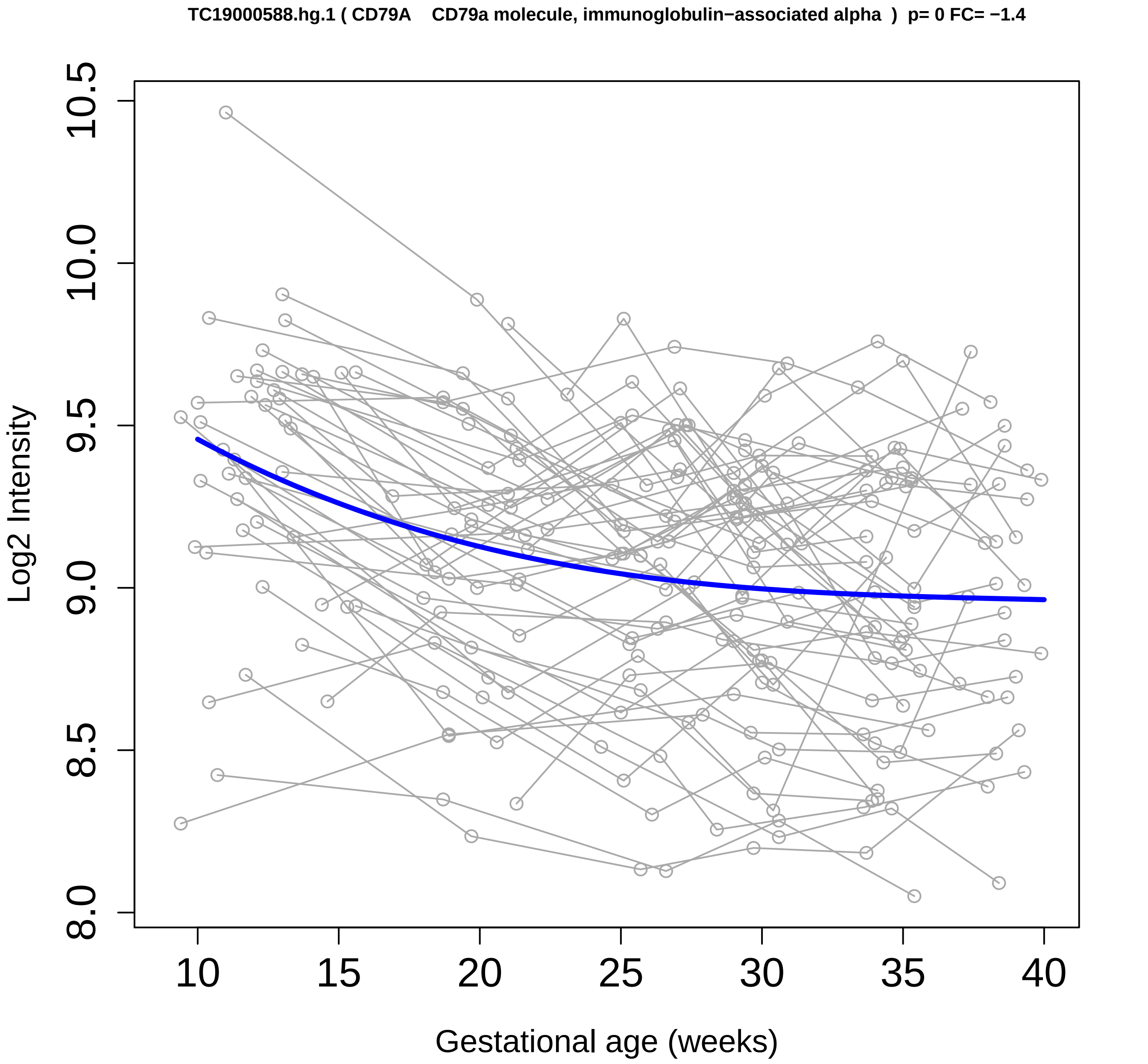
TC19000588.hg.1 (CD79A CD79a molecule, immunoglobulin−associated alpha) p= 0 FC= −1.4
Log2 Intensity
10.5
10.0
9.5
9.0
8.5
8.0
10
15
20
25
30
35
40
Gestational age (weeks)

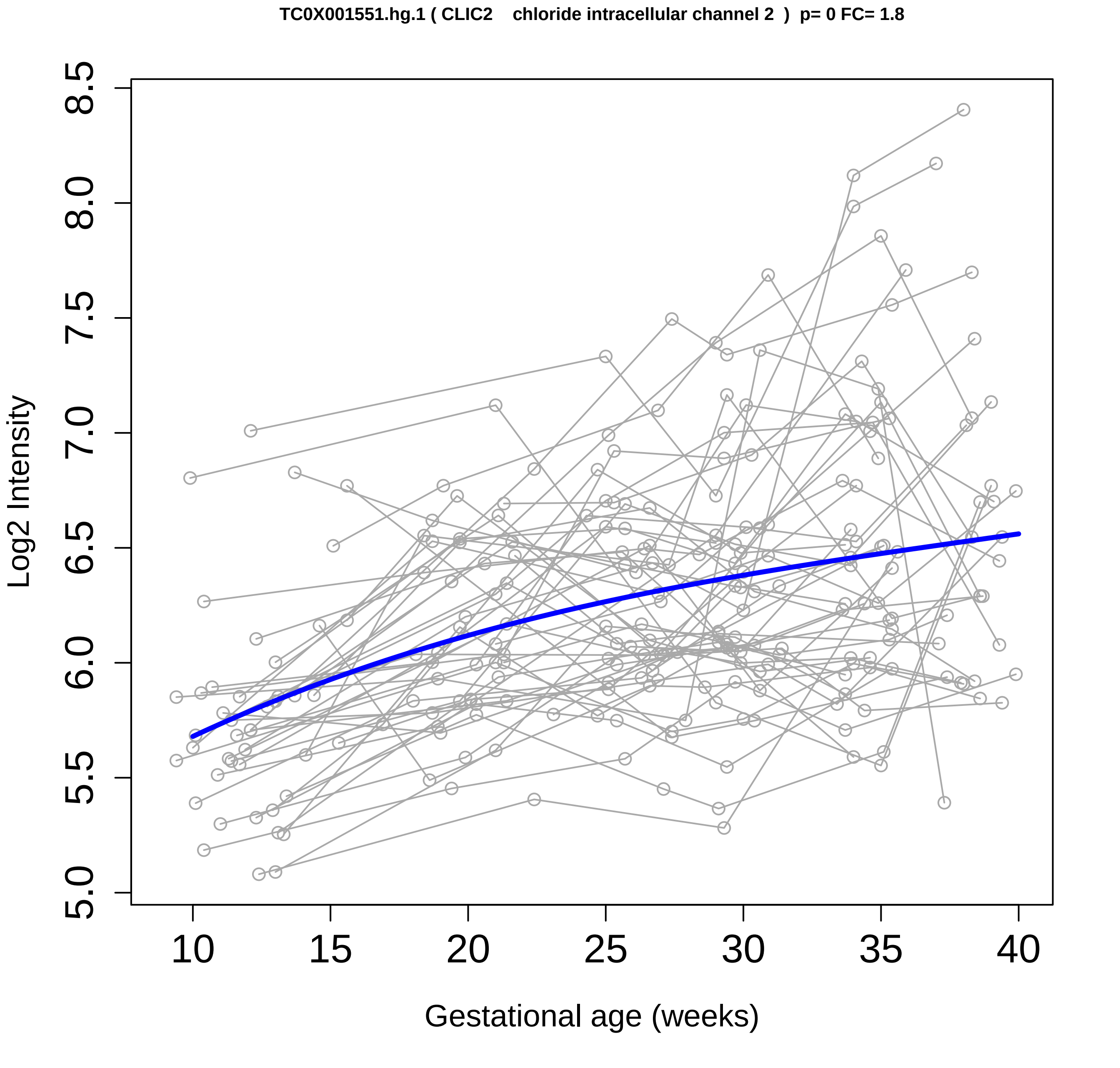
TC0X001551.hg.1 (CLIC2 chloride intracellular channel 2) p= 0 FC= 1.8
Log2 Intensity
5.0
5.5
6.0
6.5
7.0
7.5
8.0
8.5
10
15
20
25
30
35
40
Gestational age (weeks)

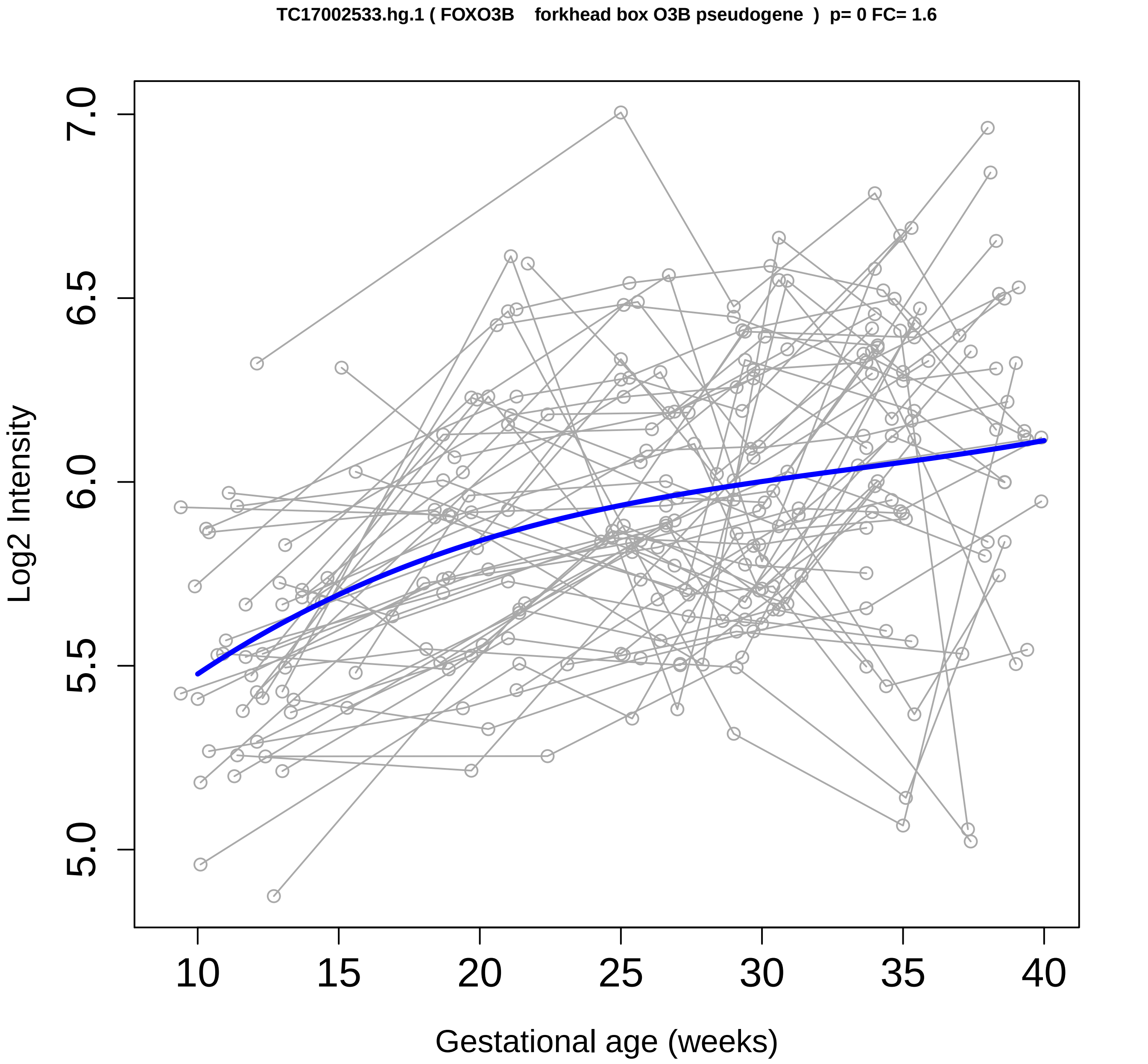

TC17002533.hg.1 (FOXO3B forkhead box O3B pseudogene) p= 0 FC= 1.6
Log2 Intensity
5.0
5.5
6.0
6.5
7.0
10
15
20
25
30
35
40
Gestational age (weeks)

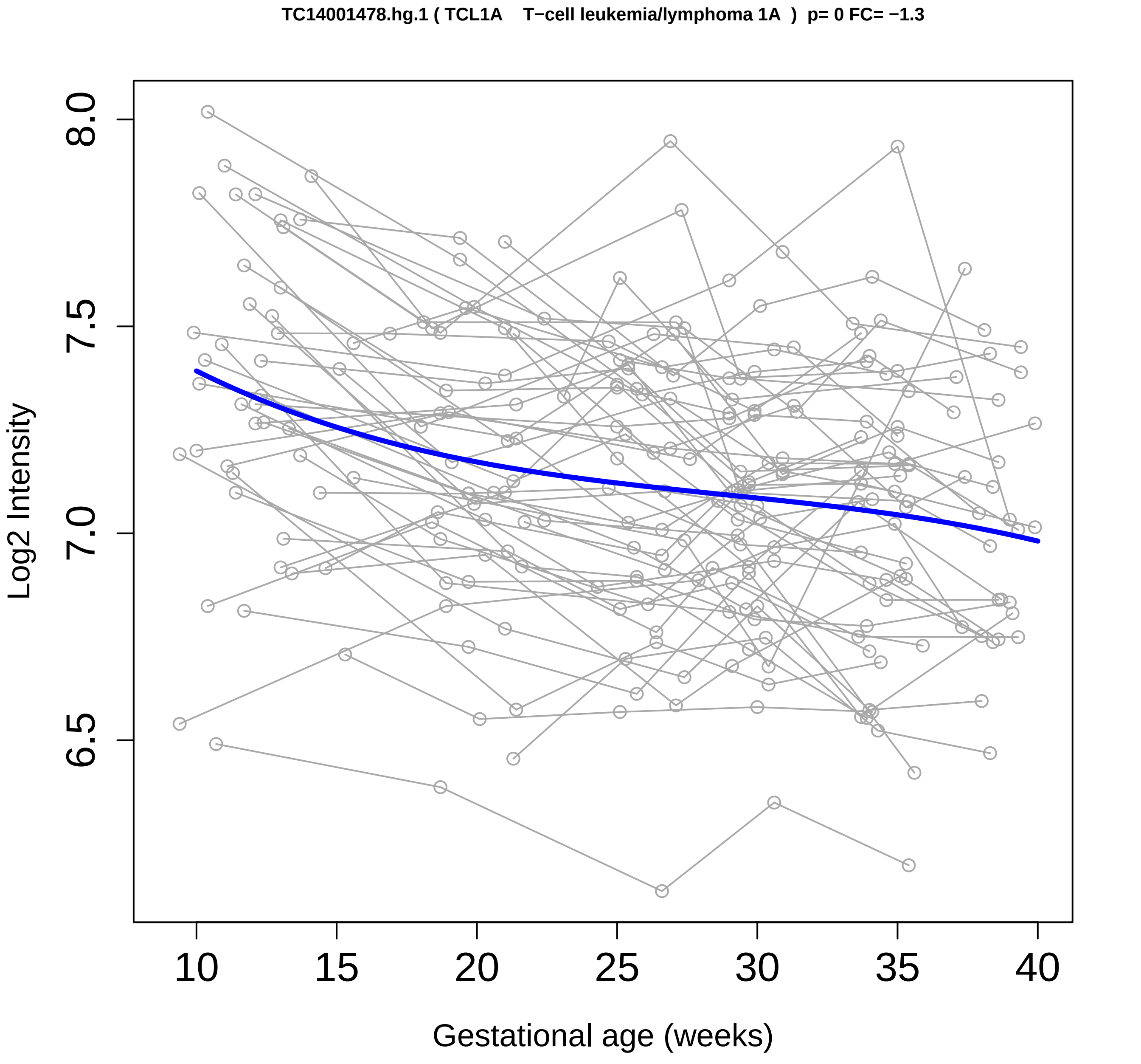
TC14001478.hg.1 (TCL1A T−cell leukemia/lymphoma 1A) p= 0 FC= −1.3
Log2 Intensity
8.0
7.5
7.0
6.5
10
15
20
25
30
35
40
Gestational age (weeks)

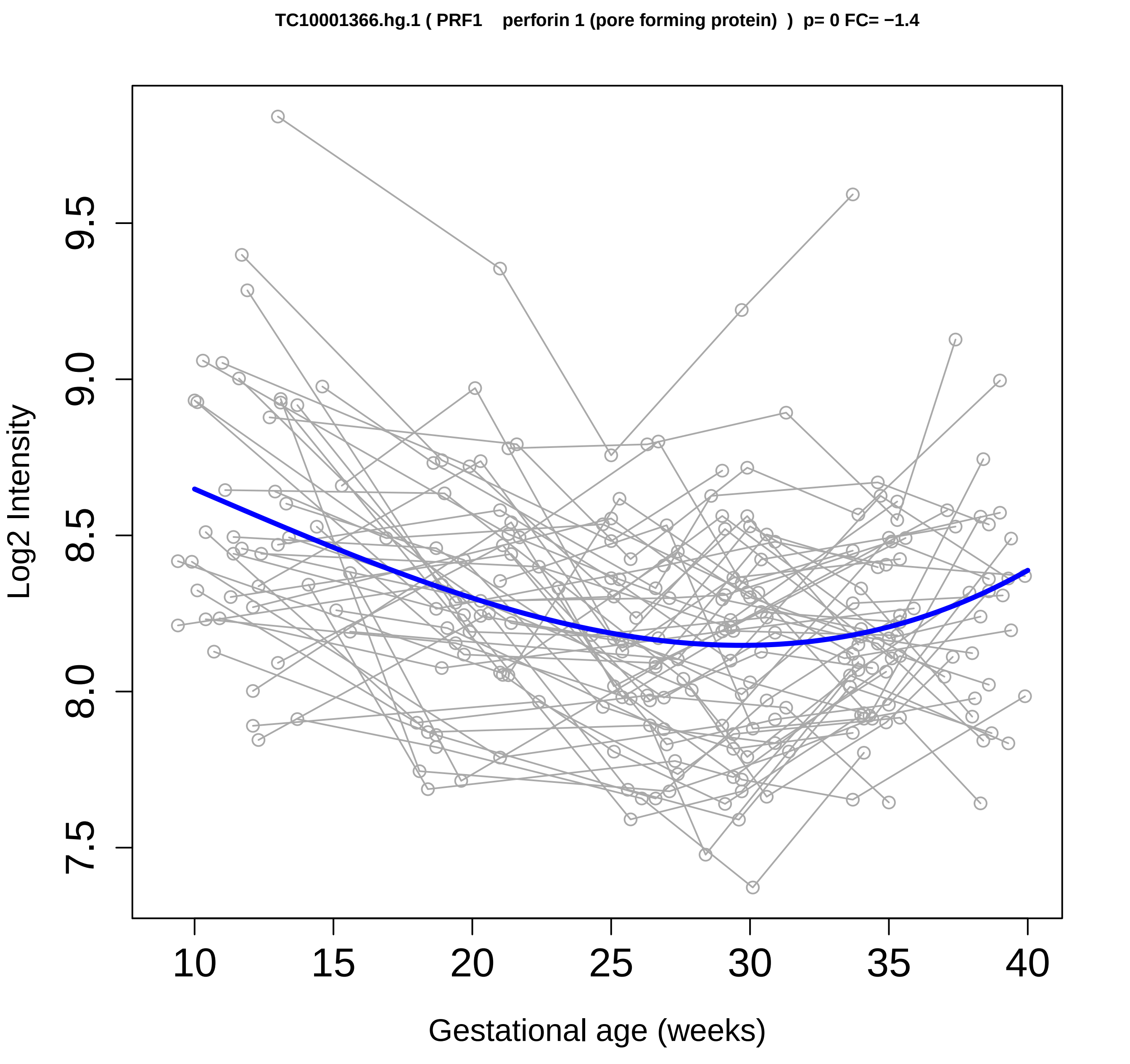

TC10001366.hg.1 (PRF1 perforin 1 (pore forming protein)) p= 0 FC= −1.4
Log2 Intensity
9.5
9.0
8.5
8.0
7.5
10
15
20
25
30
35
40
Gestational age (weeks)

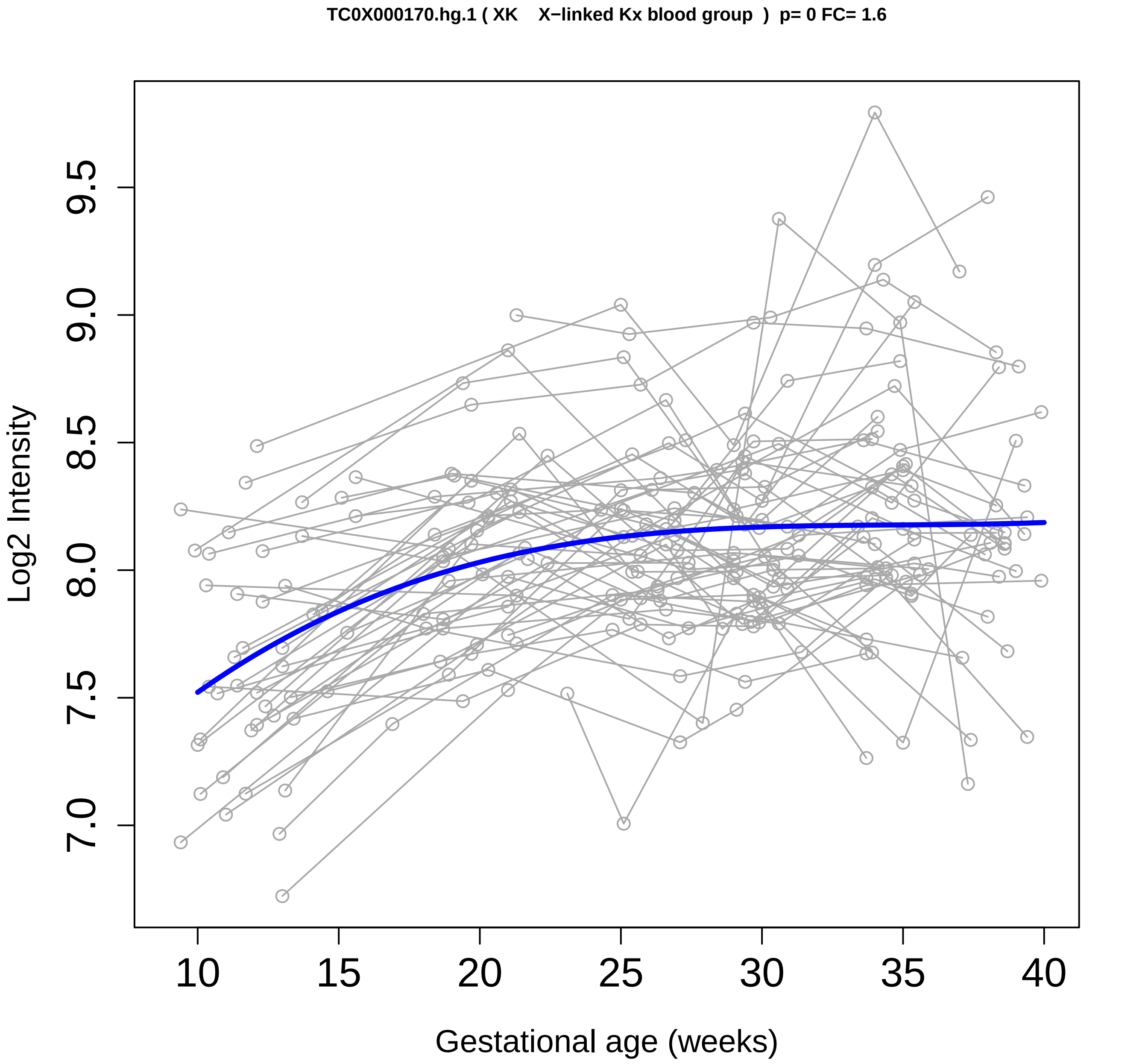

TC0X000170.hg.1 (XK X−linked Kx blood group) p= 0 FC= 1.6
Log2 Intensity
9.5
9.0
8.5
8.0
7.5
7.0
10
15
20
25
30
35
40
Gestational age (weeks)

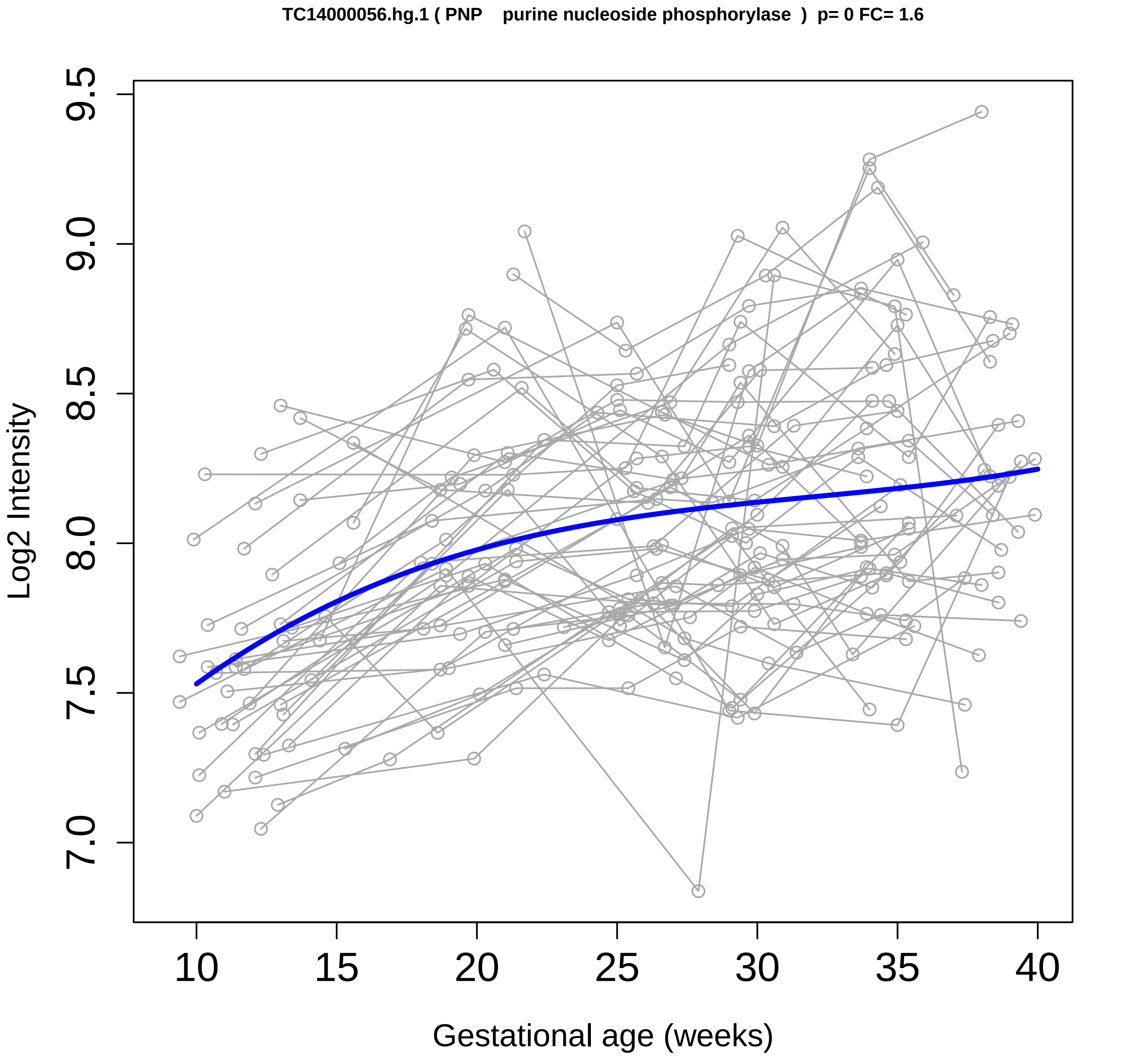

TC14000056.hg.1 (PNP   purine nucleoside phosphorylase) p= 0 FC= 1.6
Log2 Intensity
7.0
7.5
8.0
8.5
9.0
9.5
10
15
20
25
30
35
40
Gestational age (weeks)

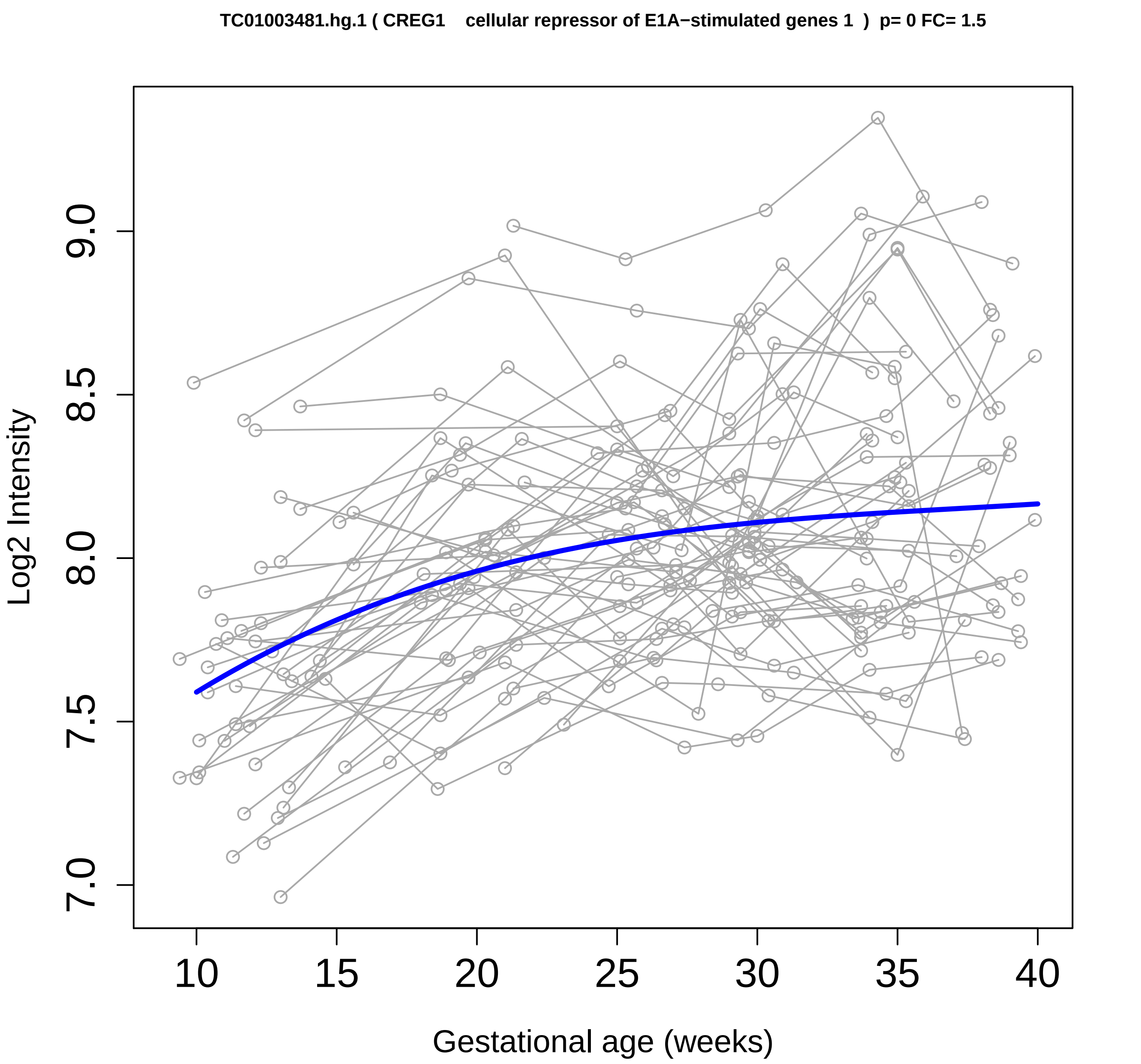
TC01003481.hg.1 (CREG1 cellular repressor of E1A−stimulated genes 1) p= 0 FC= 1.5
Log2 Intensity
7.0
7.5
8.0
8.5
9.0
10
15
20
25
30
35
40
Gestational age (weeks)

TC19002659.hg.1 (KIR2DL1 killer cell immunoglobulin−like receptor, two domains, long cytoplasmic tail, 1) p= 0 FC= −1.4

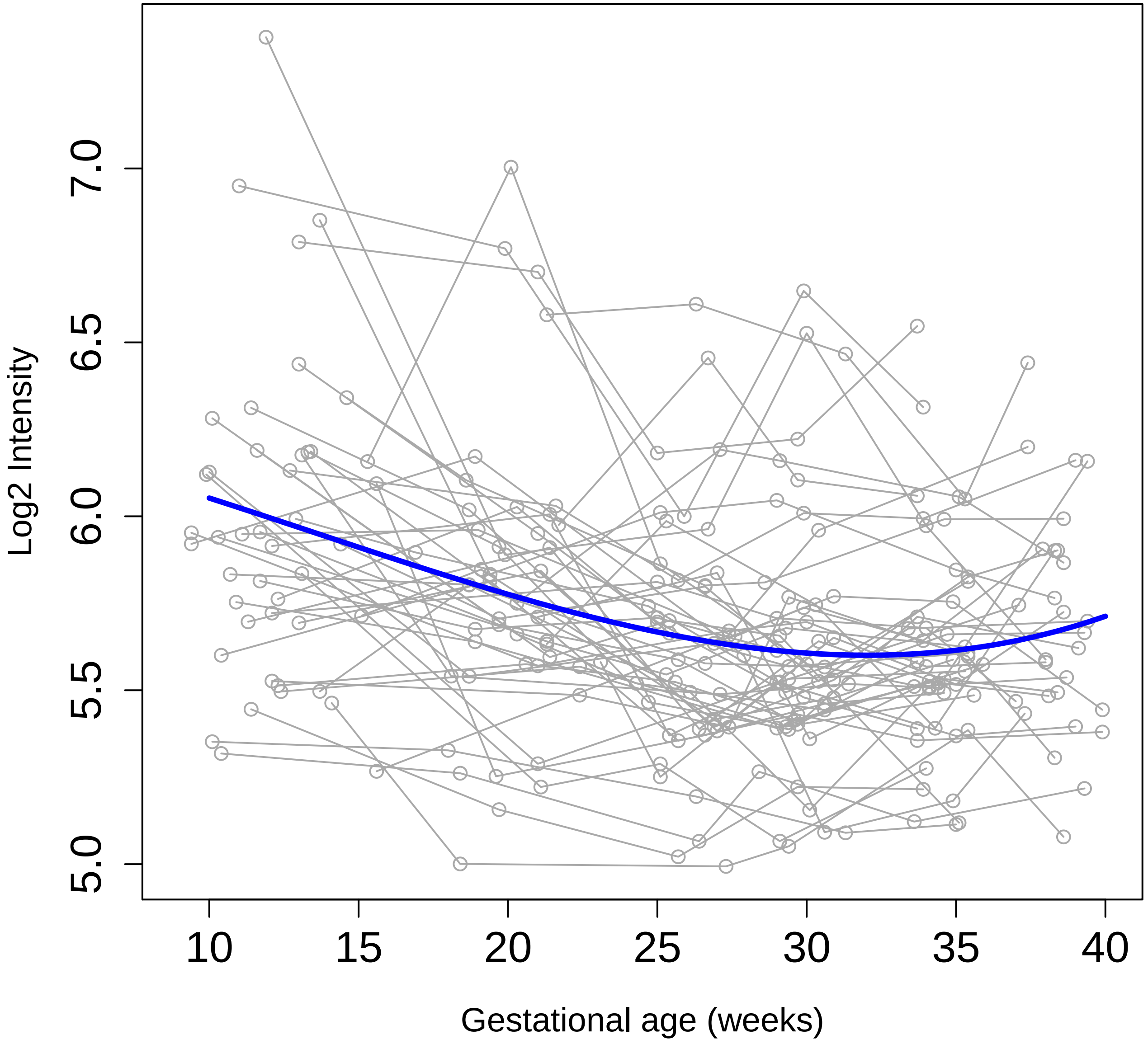

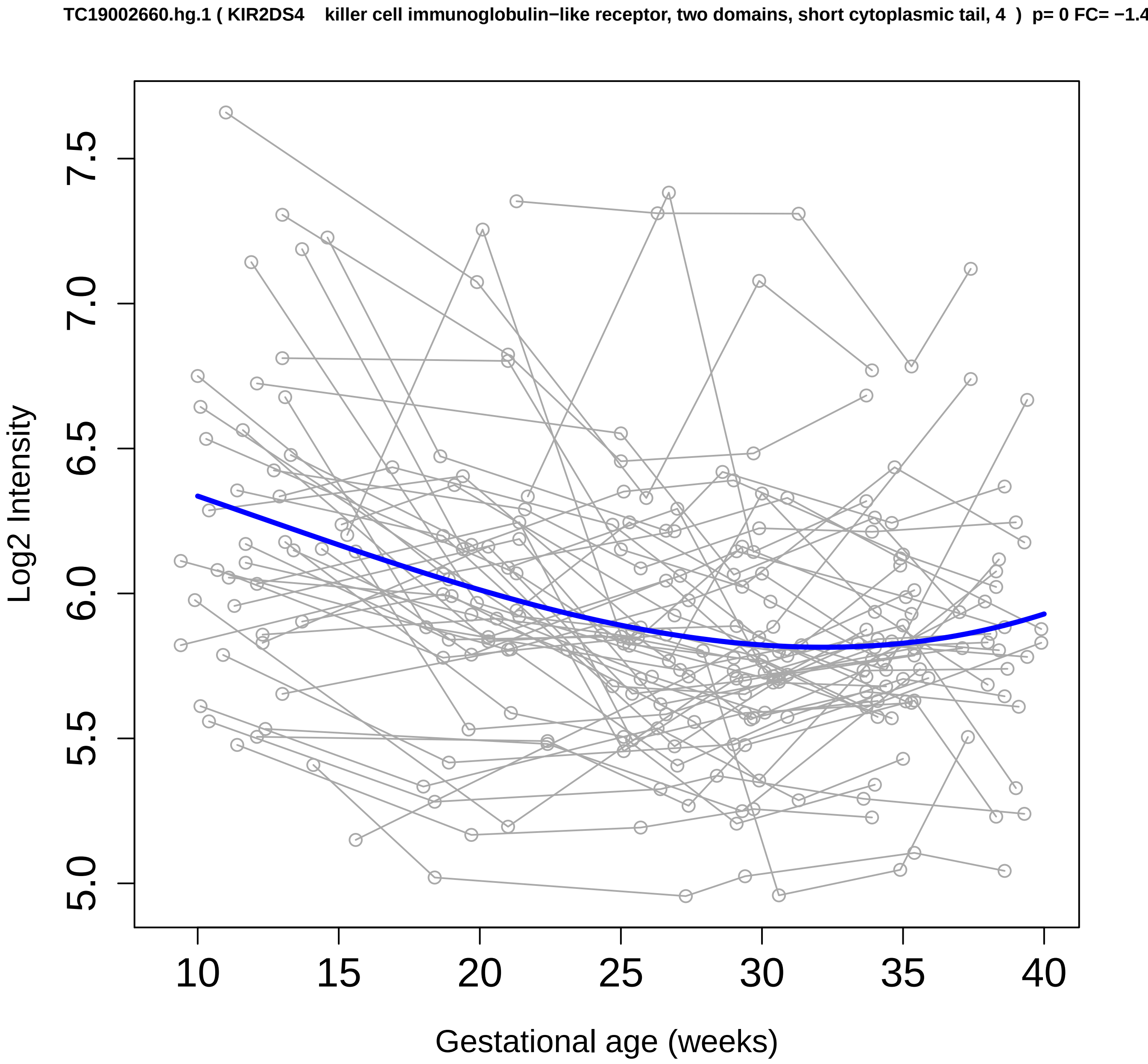
TC19002660.hg.1 (KIR2DS4 killer cell immunoglobulin−like receptor, two domains, short cytoplasmic tail, 4) p= 0 FC= −1.4
Log2 Intensity
5.0
5.5
6.0
6.5
7.0
7.5
10
15
20
25
30
35
40
Gestational age (weeks)

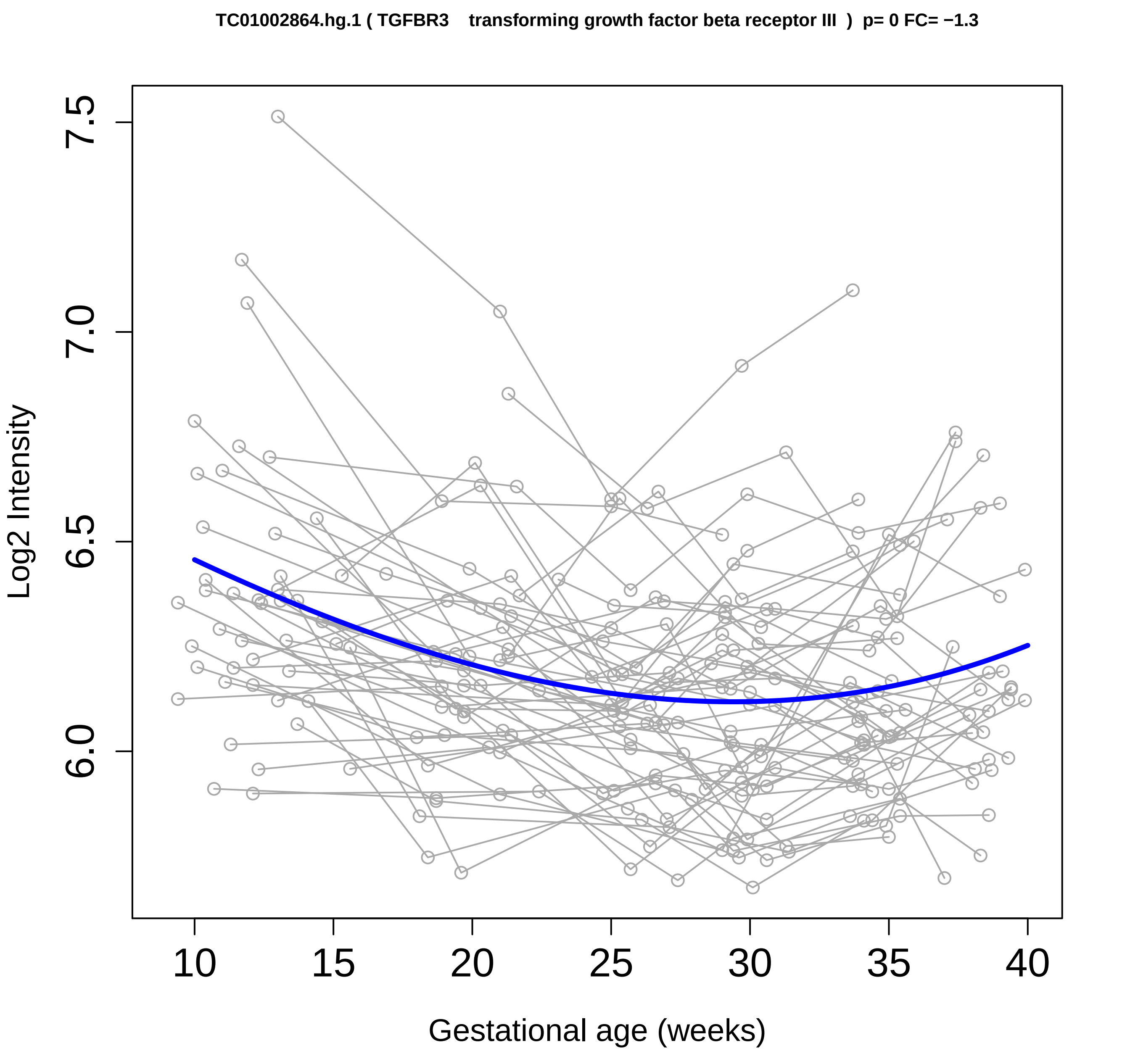

TC01002864.hg.1 (TGFBR3   transforming growth factor beta receptor III) p= 0 FC= −1.3
Log2 Intensity
7.5
7.0
6.5
6.0
10
15
20
25
30
35
40
Gestational age (weeks)

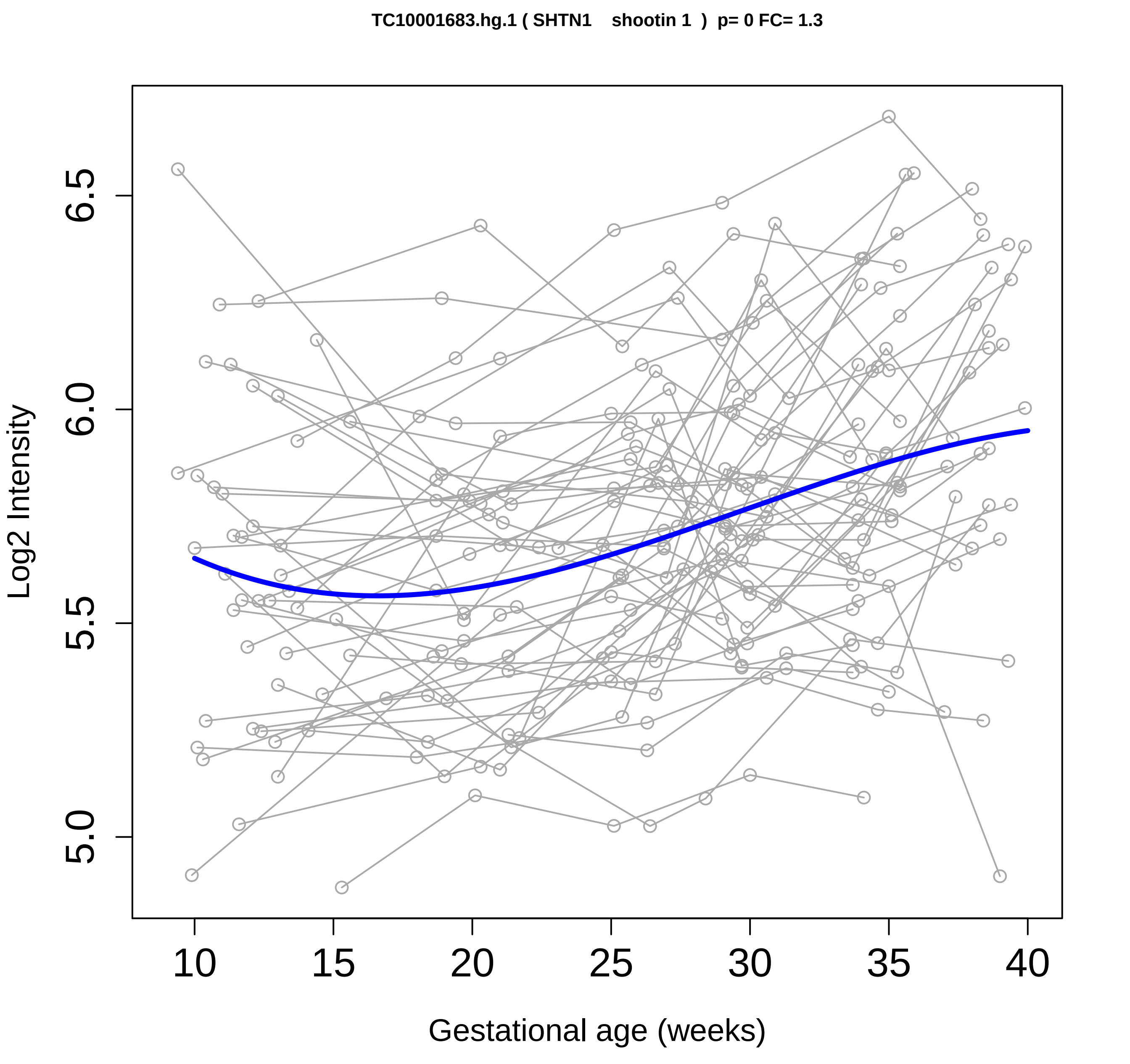
TC10001683.hg.1 (SHTN1 shootin 1) p= 0 FC= 1.3
Log2 Intensity
5.0
5.5
6.0
6.5
10
15
20
25
30
35
40
Gestational age (weeks)

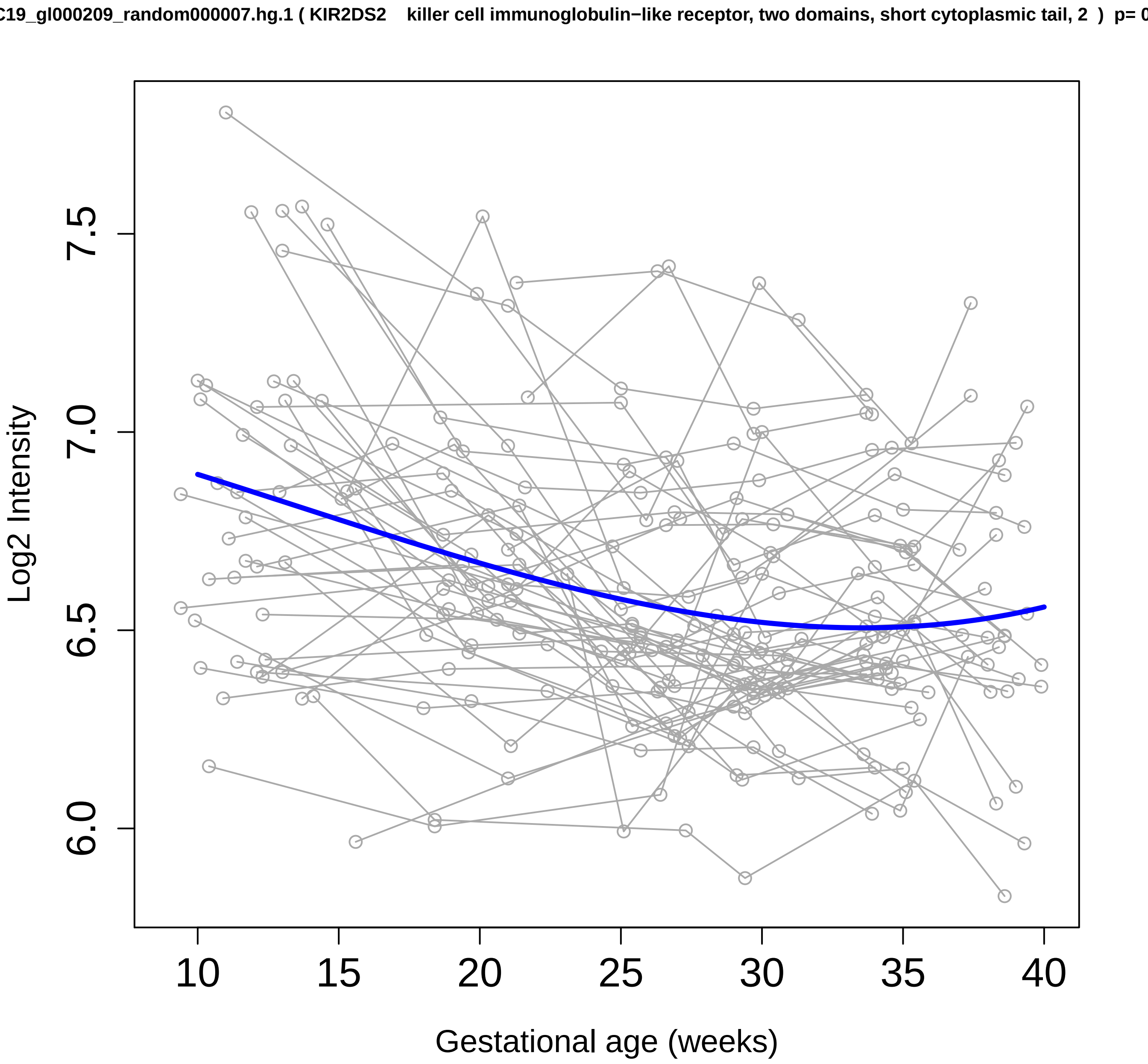

C19_gl000209_random000007.hg.1 (KIR2DS2 killer cell immunoglobulin−like receptor, two domains, short cytoplasmic tail, 2) p= 0
Log2 Intensity
6.0
6.5
7.0
7.5
10
15
20
25
30
35
40
Gestational age (weeks)

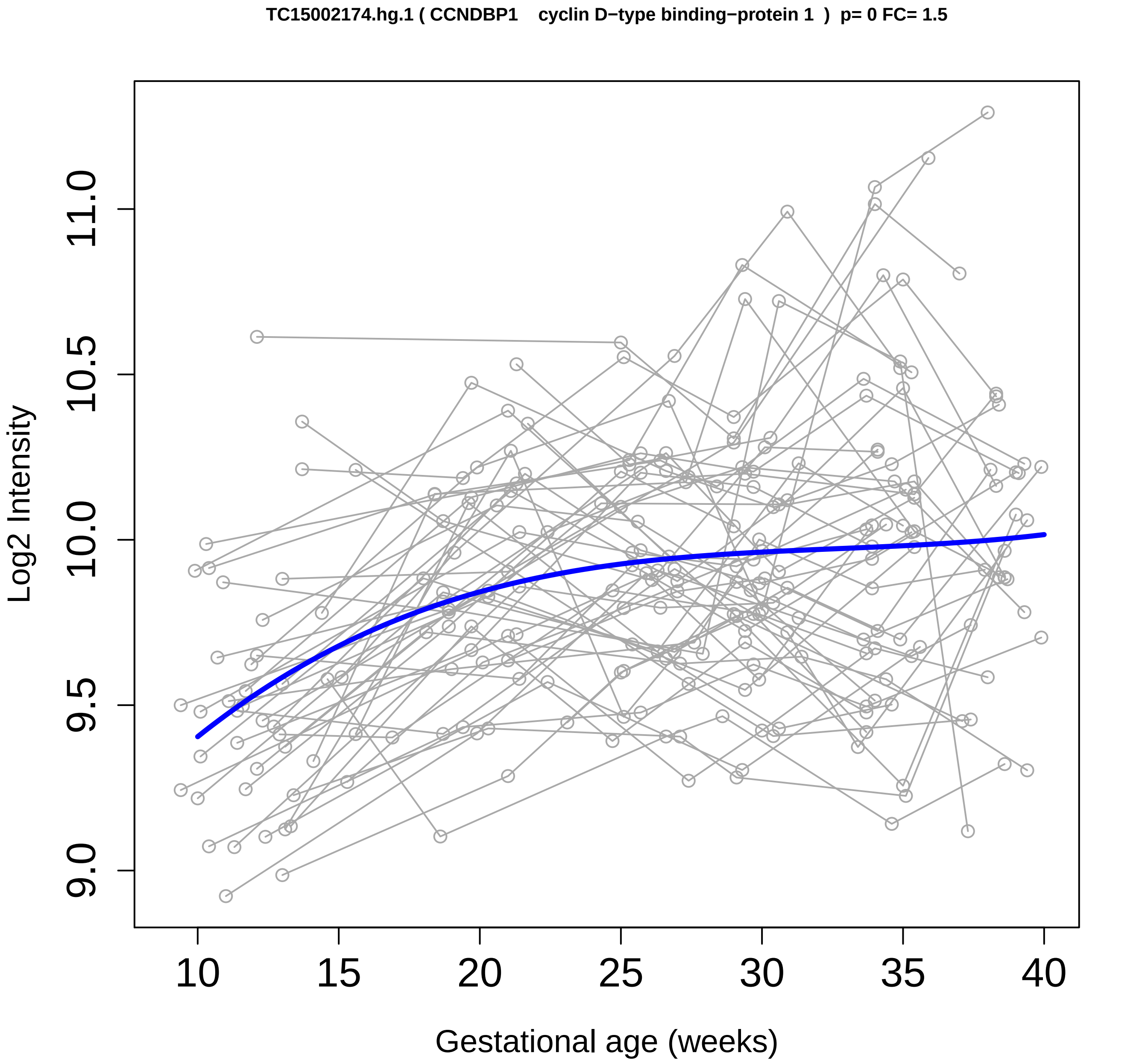

TC15002174.hg.1 (CCNDBP1 cyclin D−type binding−protein 1) p= 0 FC= 1.5
Log2 Intensity
9.0
9.5
10.0
10.5
11.0
10
15
20
25
30
35
40
Gestational age (weeks)

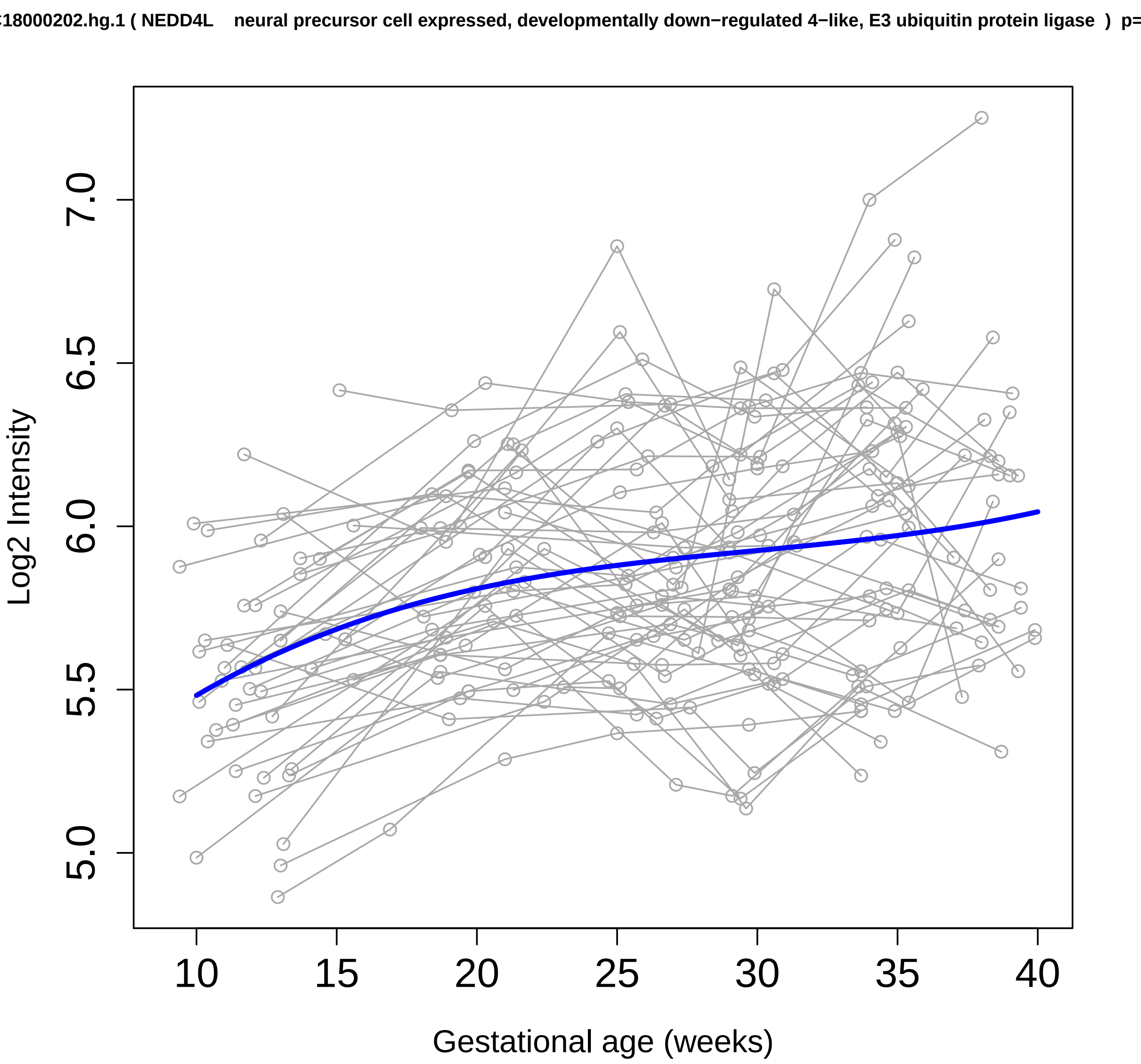

:18000202.hg.1 (NEDD4L neural precursor cell expressed, developmentally down−regulated 4−like, E3 ubiquitin protein ligase) p=
Log2 Intensity
5.0
5.5
6.0
6.5
7.0
10
15
20
25
30
35
40
Gestational age (weeks)

TC18000164.hg.1 (SLC14A1 solute carrier family 14 (urea transporter), member 1 (Kidd blood group)) p= 0 FC= 1.8

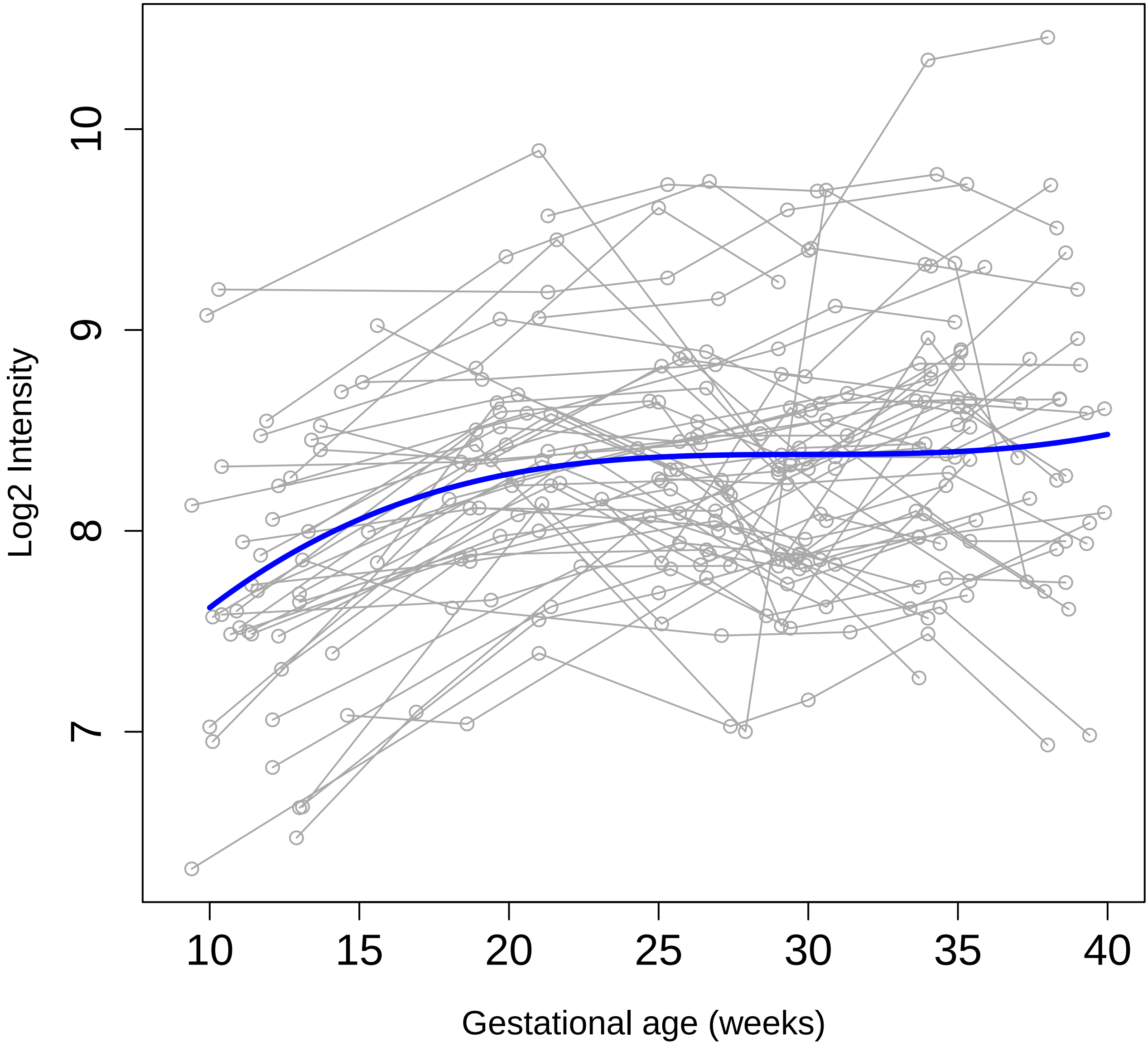

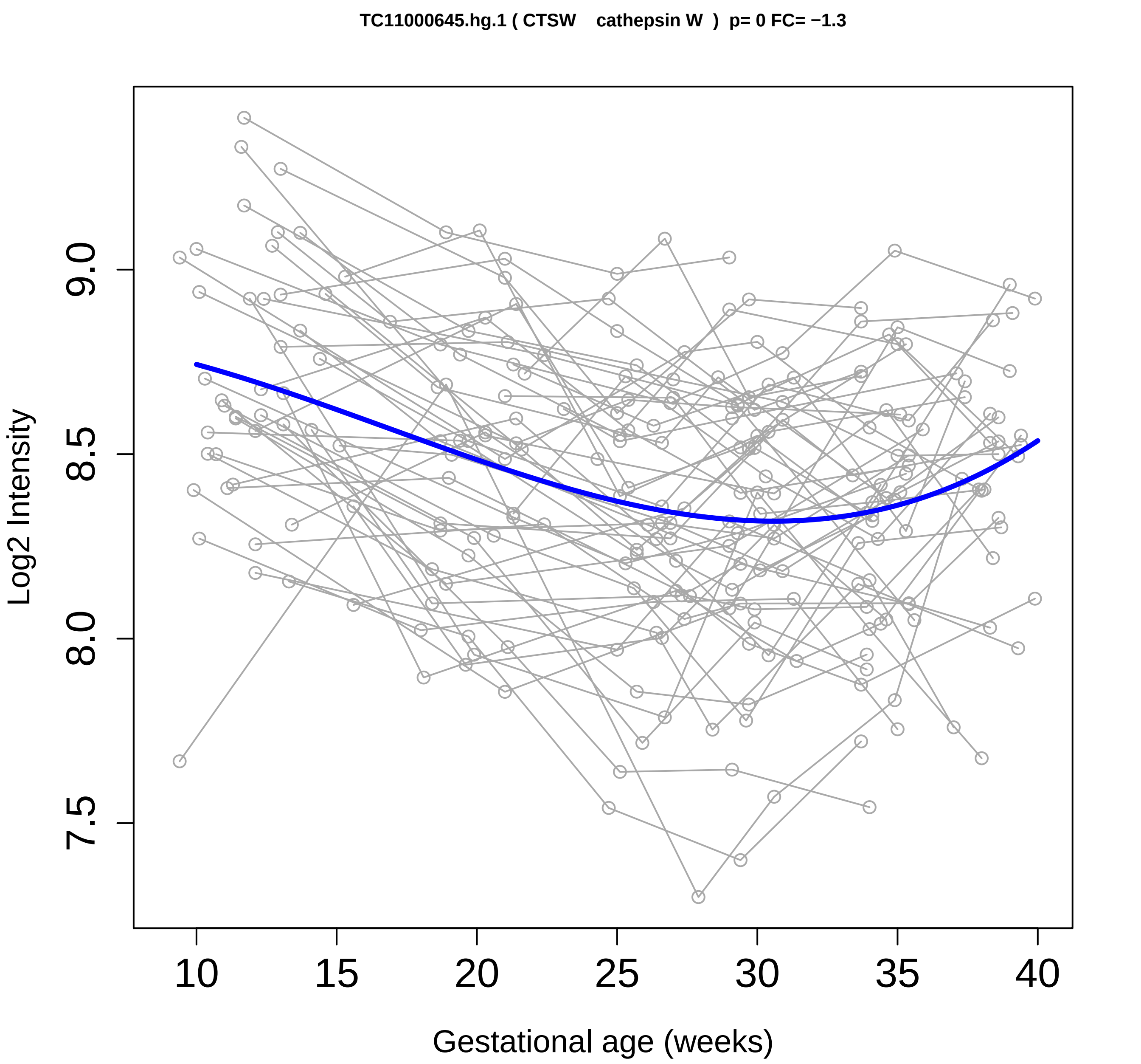

TC11000645.hg.1 (CTSW cathepsin W) p= 0 FC= −1.3
Log2 Intensity
7.5
8.0
8.5
9.0
10
15
20
25
30
35
40
Gestational age (weeks)

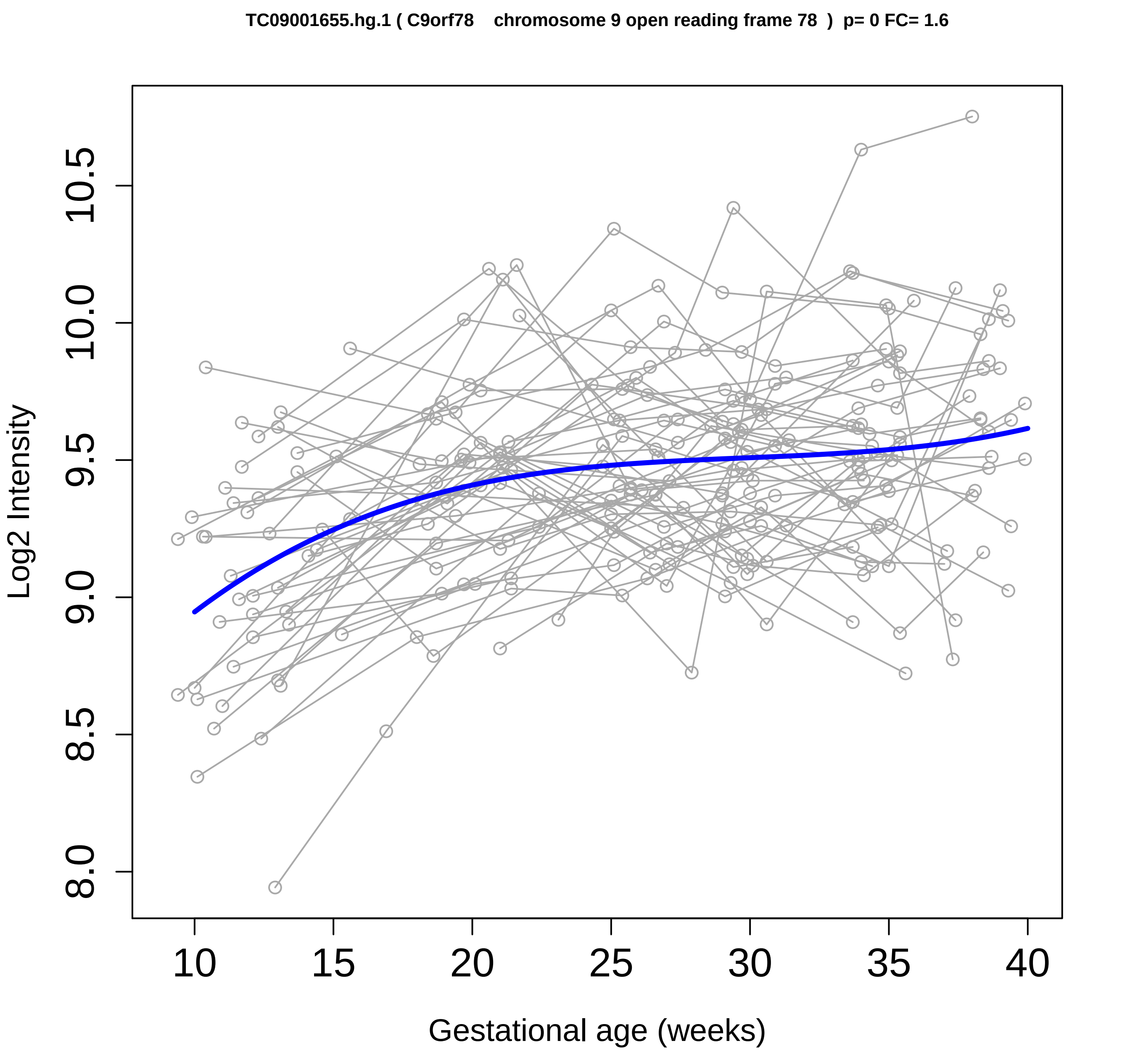

TC09001655.hg.1 (C9orf78 chromosome 9 open reading frame 78) p= 0 FC= 1.6
Log2 Intensity
8.0
8.5
9.0
9.5
10.0
10.5
10
15
20
25
30
35
40
Gestational age (weeks)

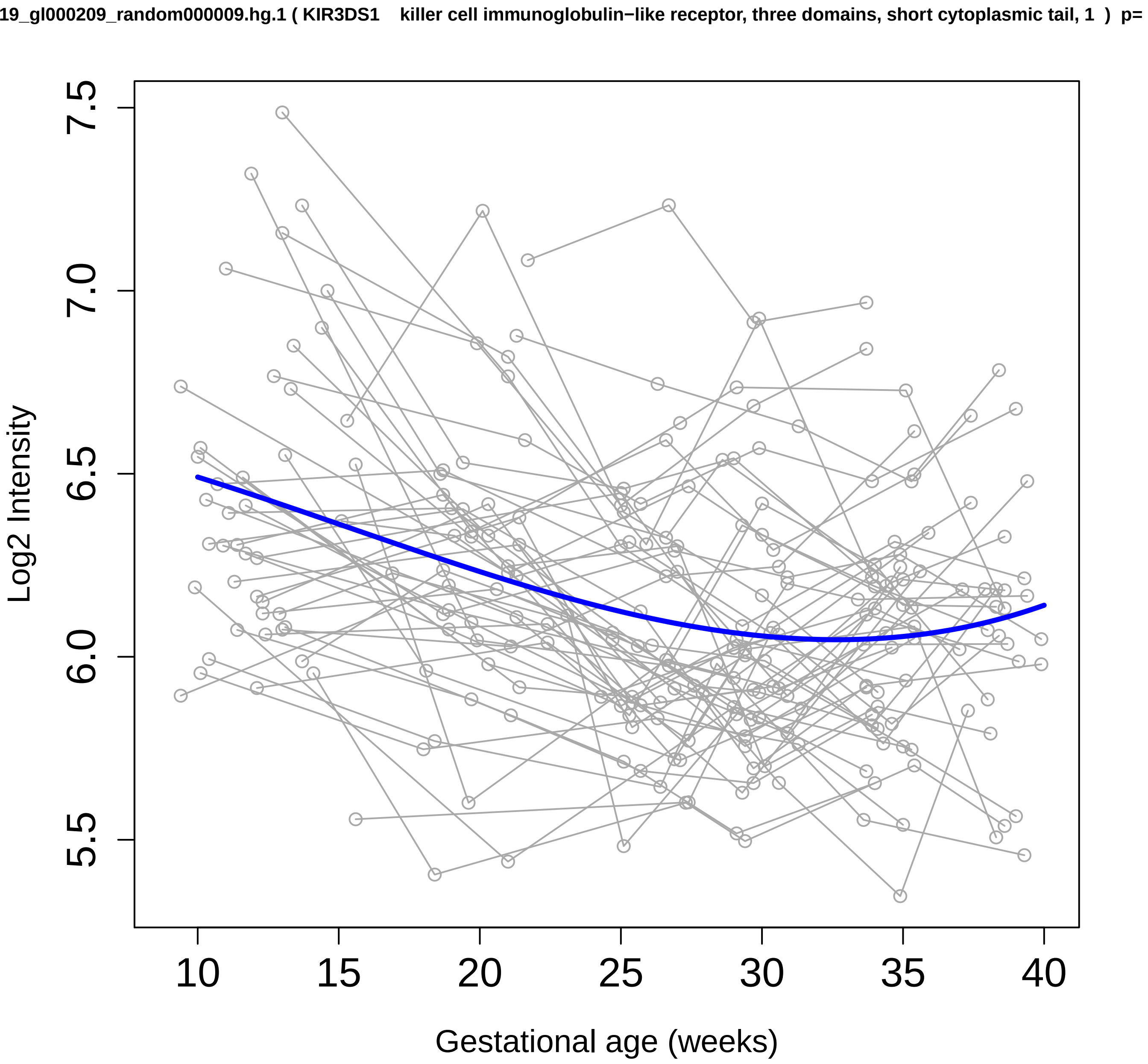
19_gl000209_random000009.hg.1 (KIR3DS1 killer cell immunoglobulin−like receptor, three domains, short cytoplasmic tail, 1) p=
Log2 Intensity
7.5
7.0
6.5
6.0
5.5
10
15
20
25
30
35
40
Gestational age (weeks)

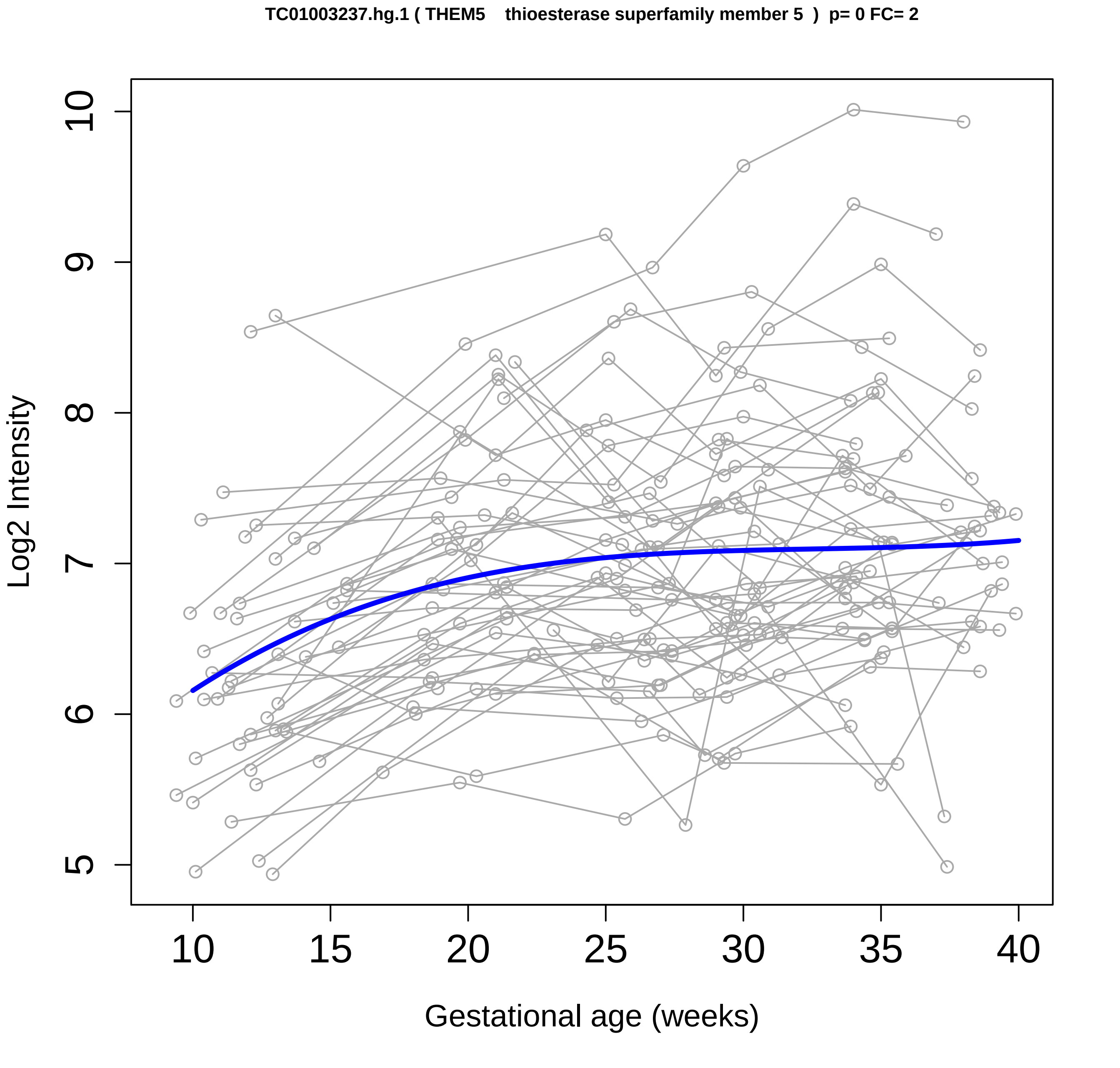

TC01003237.hg.1 (THEM5 thioesterase superfamily member 5) p= 0 FC= 2
Log2 Intensity
10
9
8
7
6
5
10
15
20
25
30
35
40
Gestational age (weeks)

C19000584.hg.1 (CEACAM6   carcinoembryonic antigen−related cell adhesion molecule 6 (non−specific cross reacting antigen)) p=

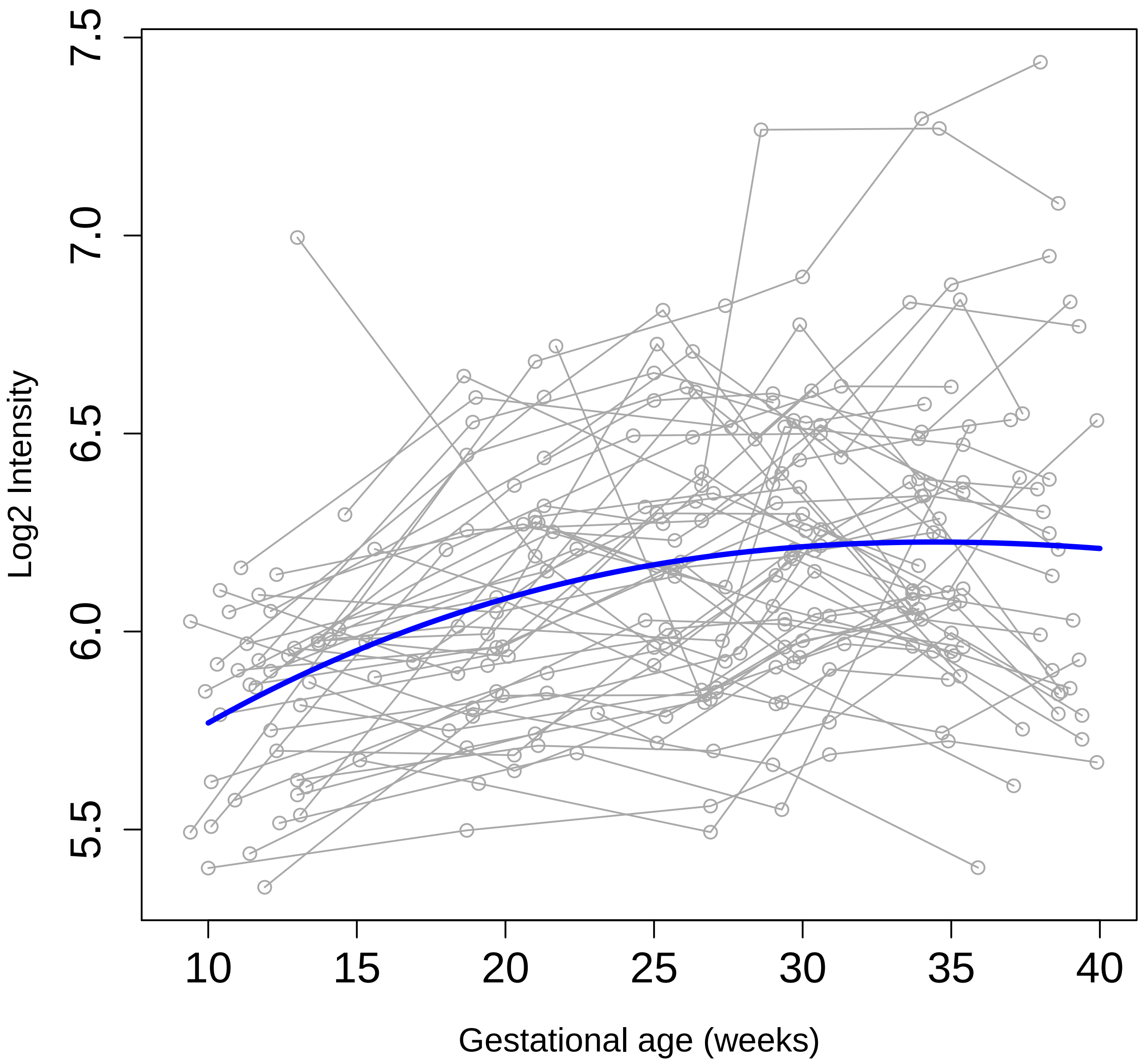

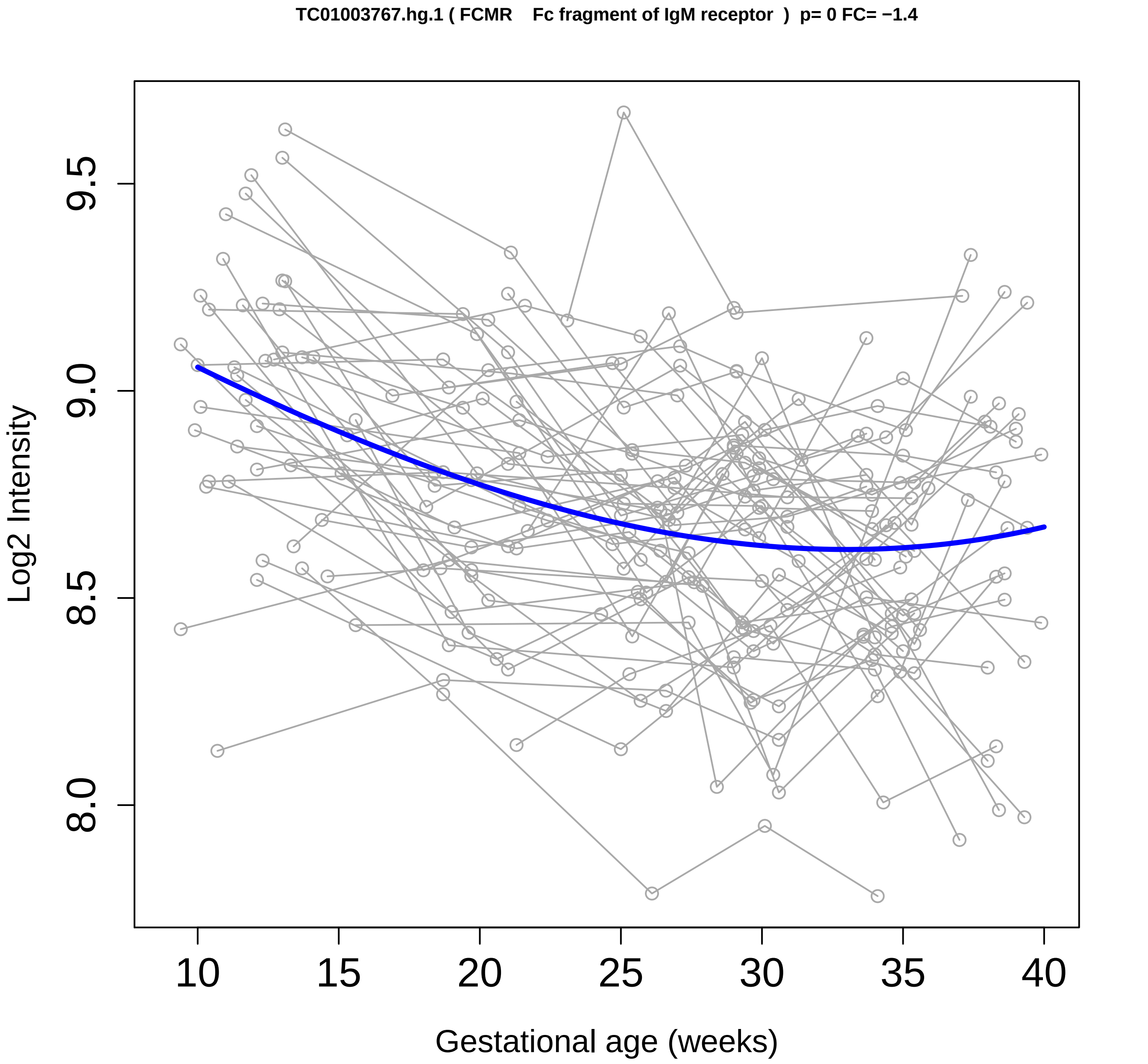
TC01003767.hg.1 (FCMR Fc fragment of IgM receptor) p= 0 FC= −1.4
Log2 Intensity
9.5
9.0
8.5
8.0
10
15
20
25
30
35
40
Gestational age (weeks)

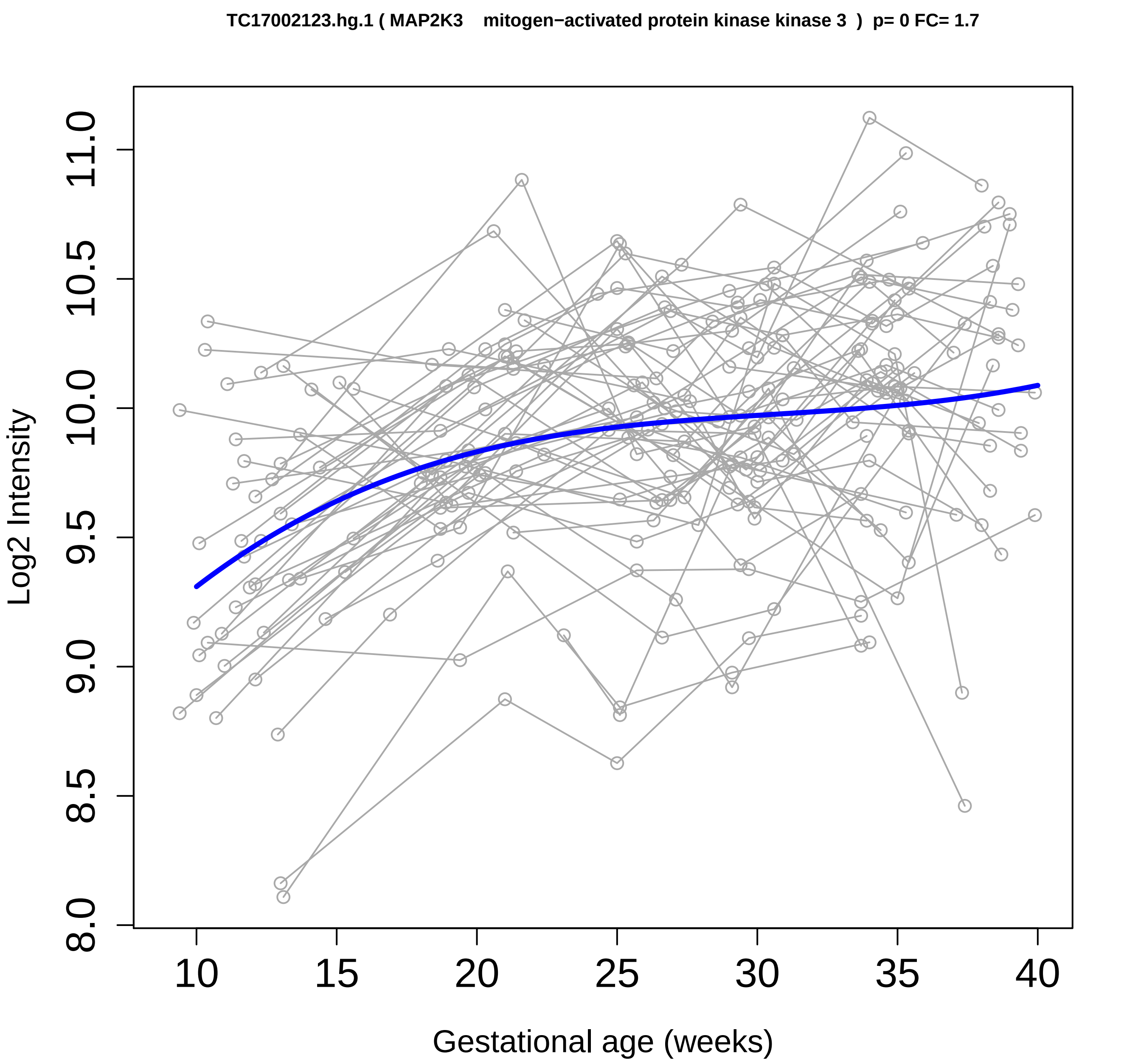
TC17002123.hg.1 (MAP2K3 mitogen−activated protein kinase kinase 3) p= 0 FC= 1.7
Log2 Intensity
Gestational age (weeks)
8.0
8.5
9.0
9.5
10.0
10.5
11.0
10
15
20
25
30
35
40

TC14002213.hg.1 (IGHM immunoglobulin heavy constant mu) p= 0 FC= −1.5

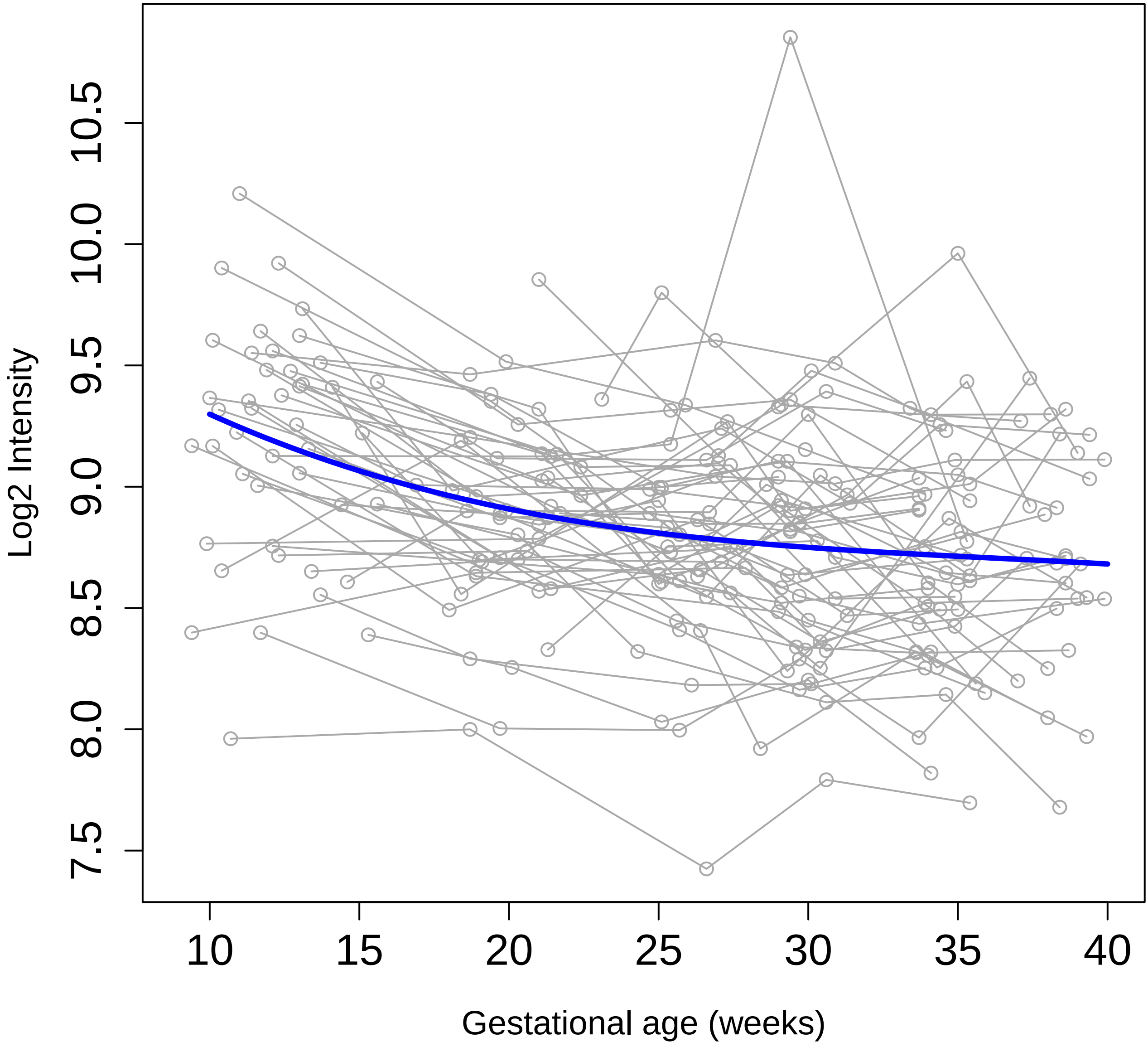

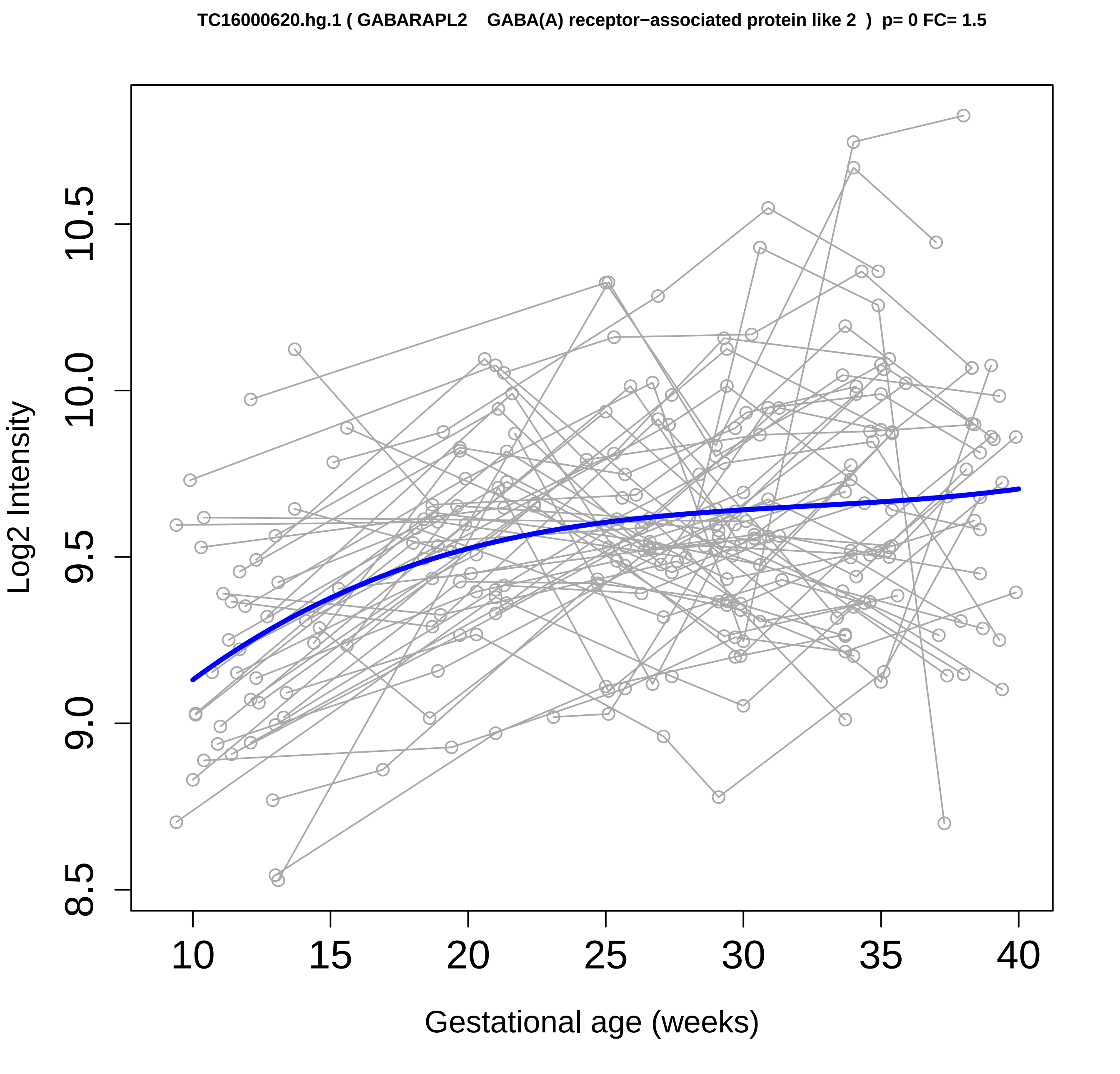
TC16000620.hg.1 (GABARAPL2   GABA(A) receptor−associated protein like 2) p= 0 FC= 1.5
Log2 Intensity
8.5
9.0
9.5
10.0
10.5
10
15
20
25
30
35
40
Gestational age (weeks)

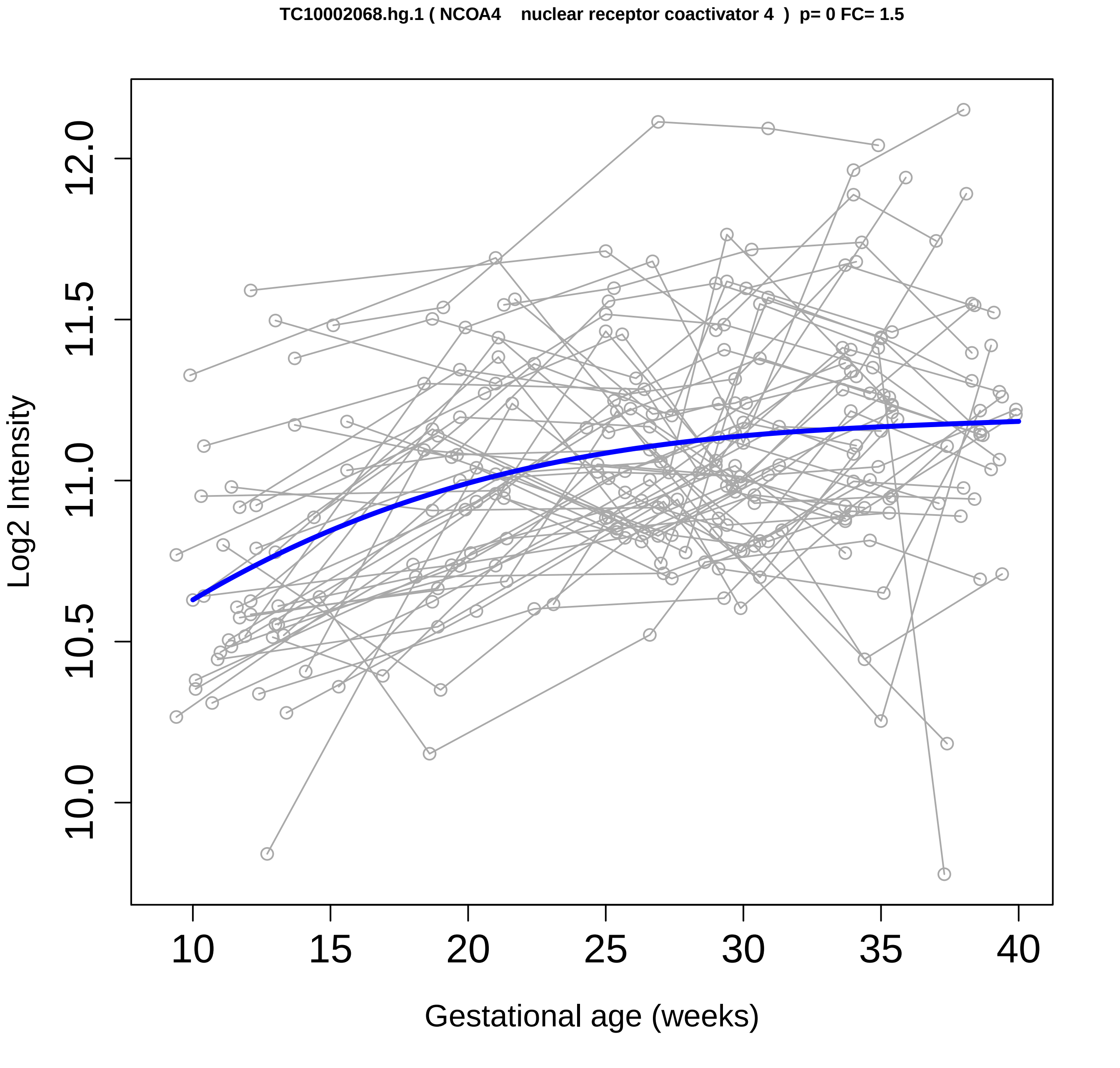

TC10002068.hg.1 (NCOA4 nuclear receptor coactivator 4) p= 0 FC= 1.5
Log2 Intensity
10.0
10.5
11.0
11.5
12.0
10
15
20
25
30
35
40
Gestational age (weeks)

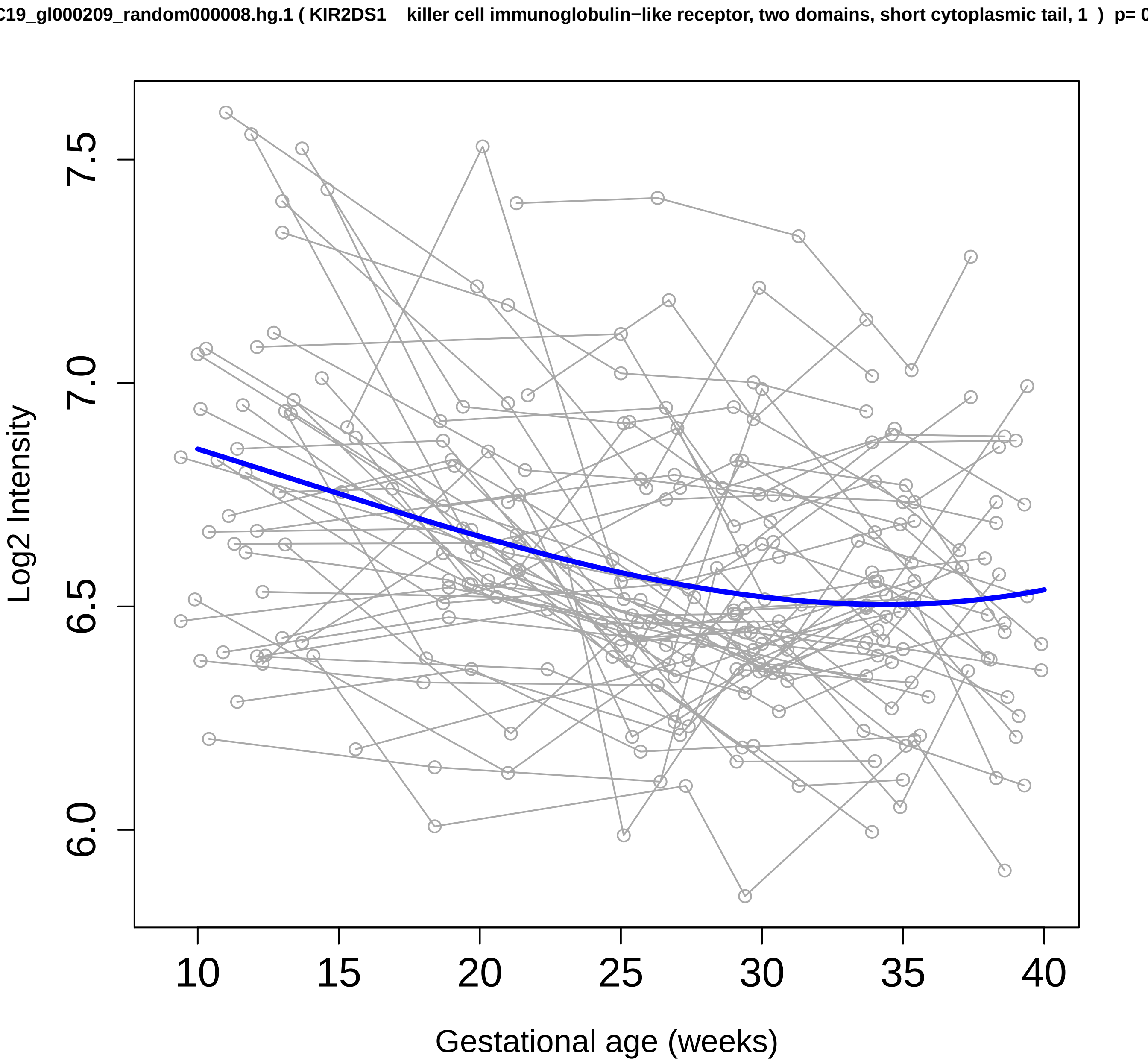

C19_gl000209_random000008.hg.1 (KIR2DS1 killer cell immunoglobulin−like receptor, two domains, short cytoplasmic tail, 1) p= 0
Log2 Intensity
7.5
7.0
6.5
6.0
10
15
20
25
30
35
40
Gestational age (weeks)

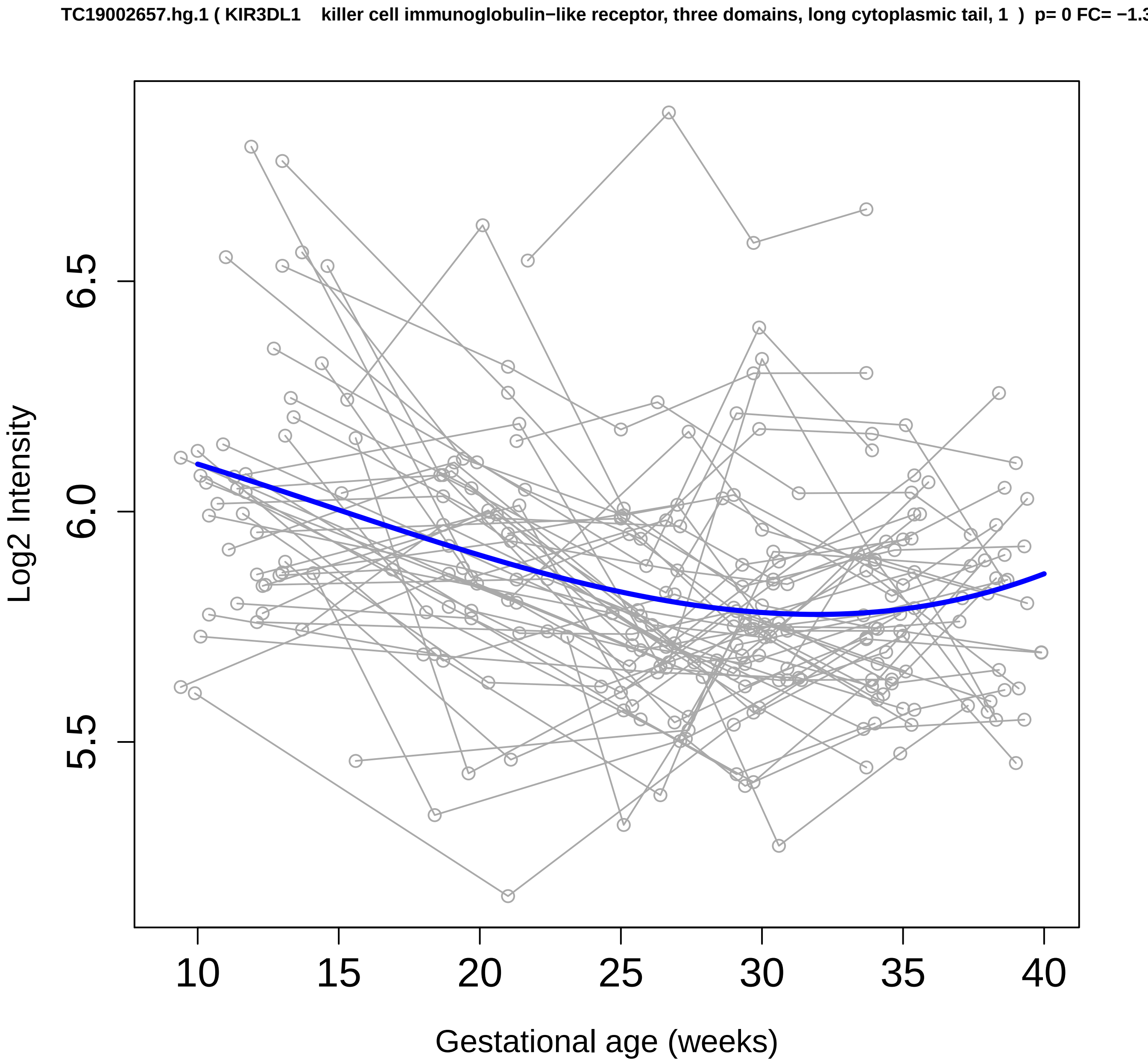

TC19002657.hg.1 (KIR3DL1 killer cell immunoglobulin−like receptor, three domains, long cytoplasmic tail, 1) p= 0 FC= −1.3
Log2 Intensity
6.5
6.0
5.5
10
15
20
25
30
35
40
Gestational age (weeks)

TC11001837.hg.1 (TCN1   transcobalamin I (vitamin B12 binding protein, R binder family)) p= 0 FC= 1.7

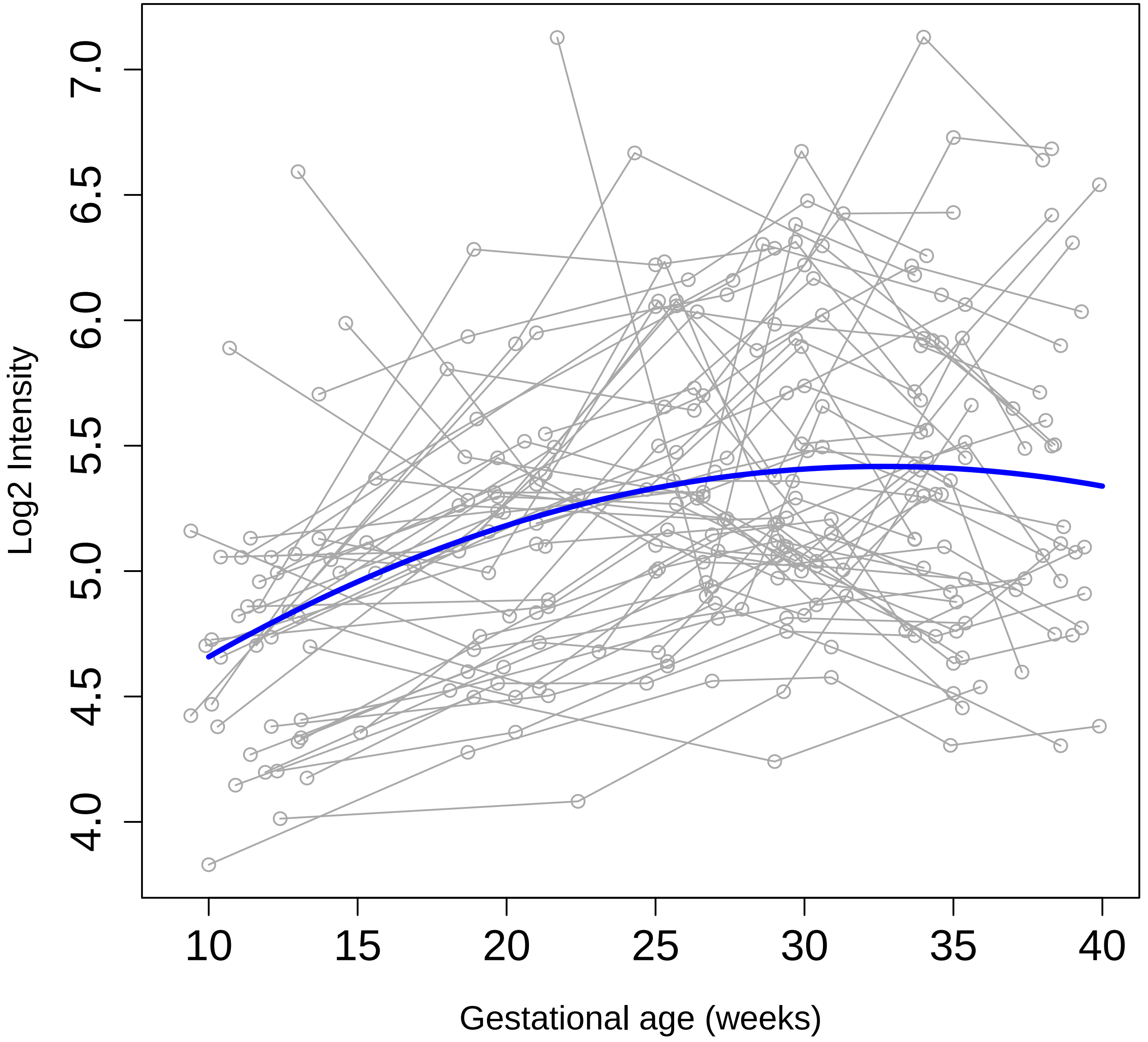

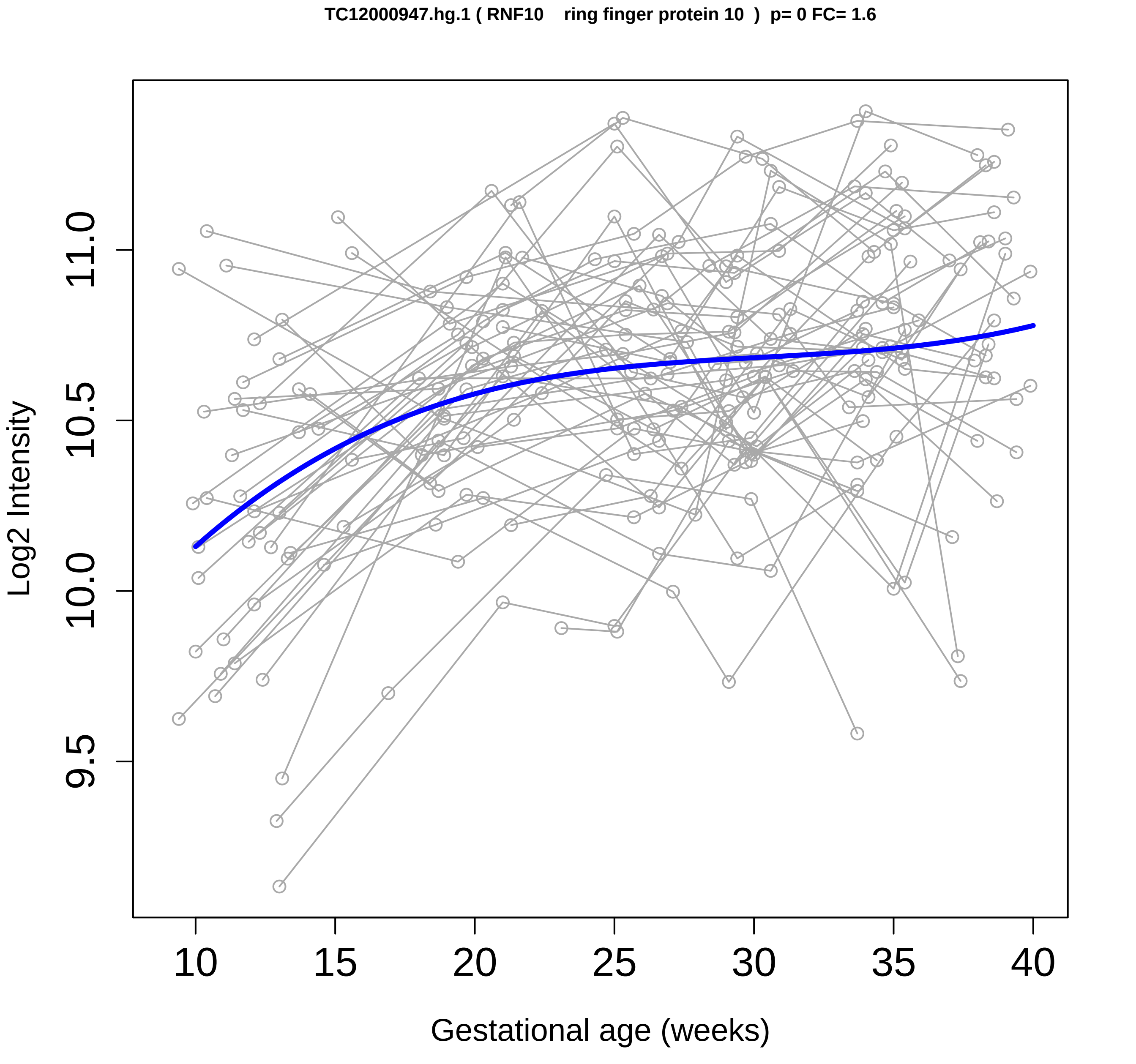

TC12000947.hg.1 (RNF10 ring finger protein 10) p= 0 FC= 1.6
Log2 Intensity
9.5
10.0
10.5
11.0
10
15
20
25
30
35
40
Gestational age (weeks)

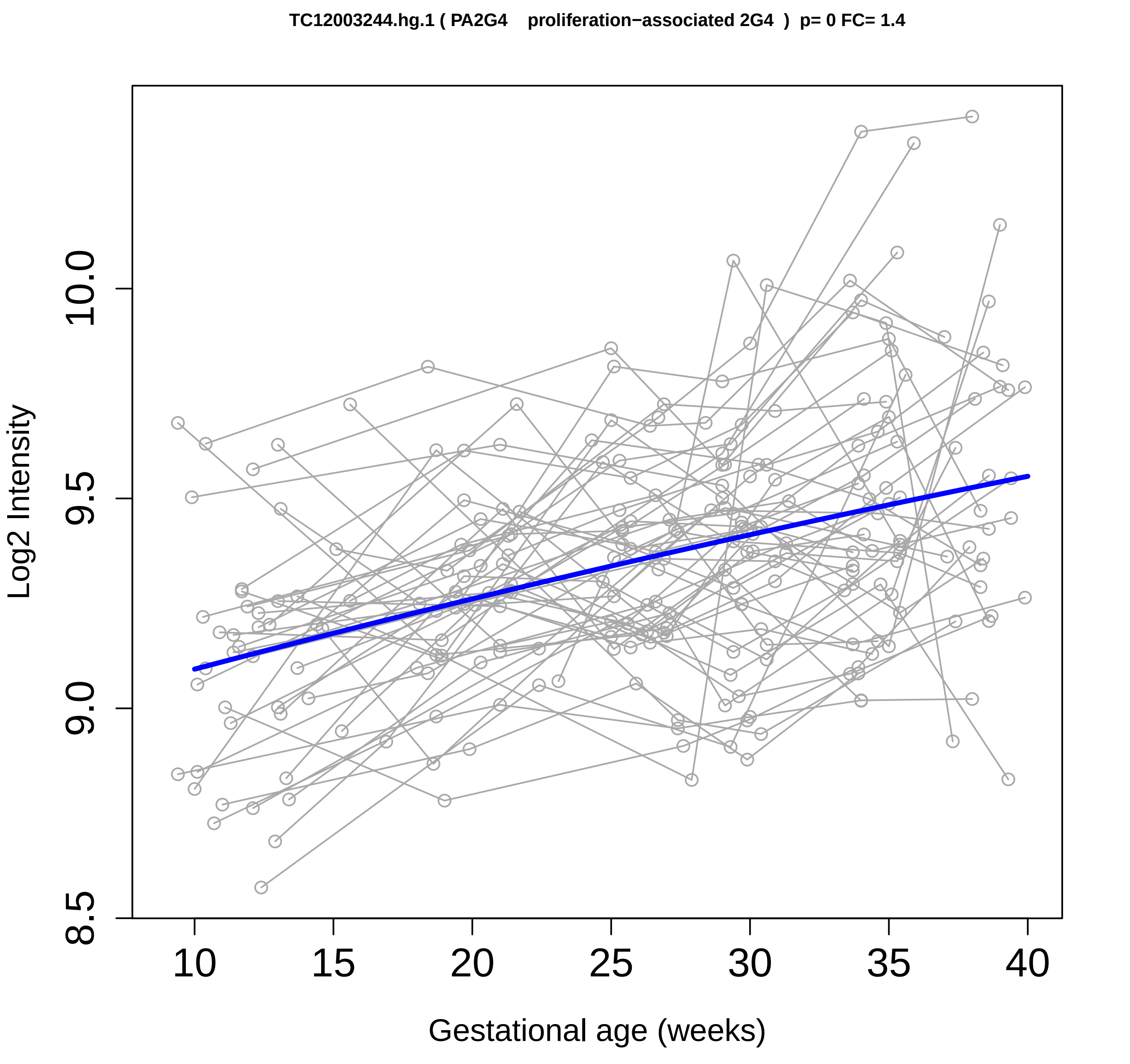
TC12003244.hg.1 (PA2G4   proliferation−associated 2G4) p= 0 FC= 1.4
Log2 Intensity
8.5
9.0
9.5
10.0
10
15
20
25
30
35
40
Gestational age (weeks)

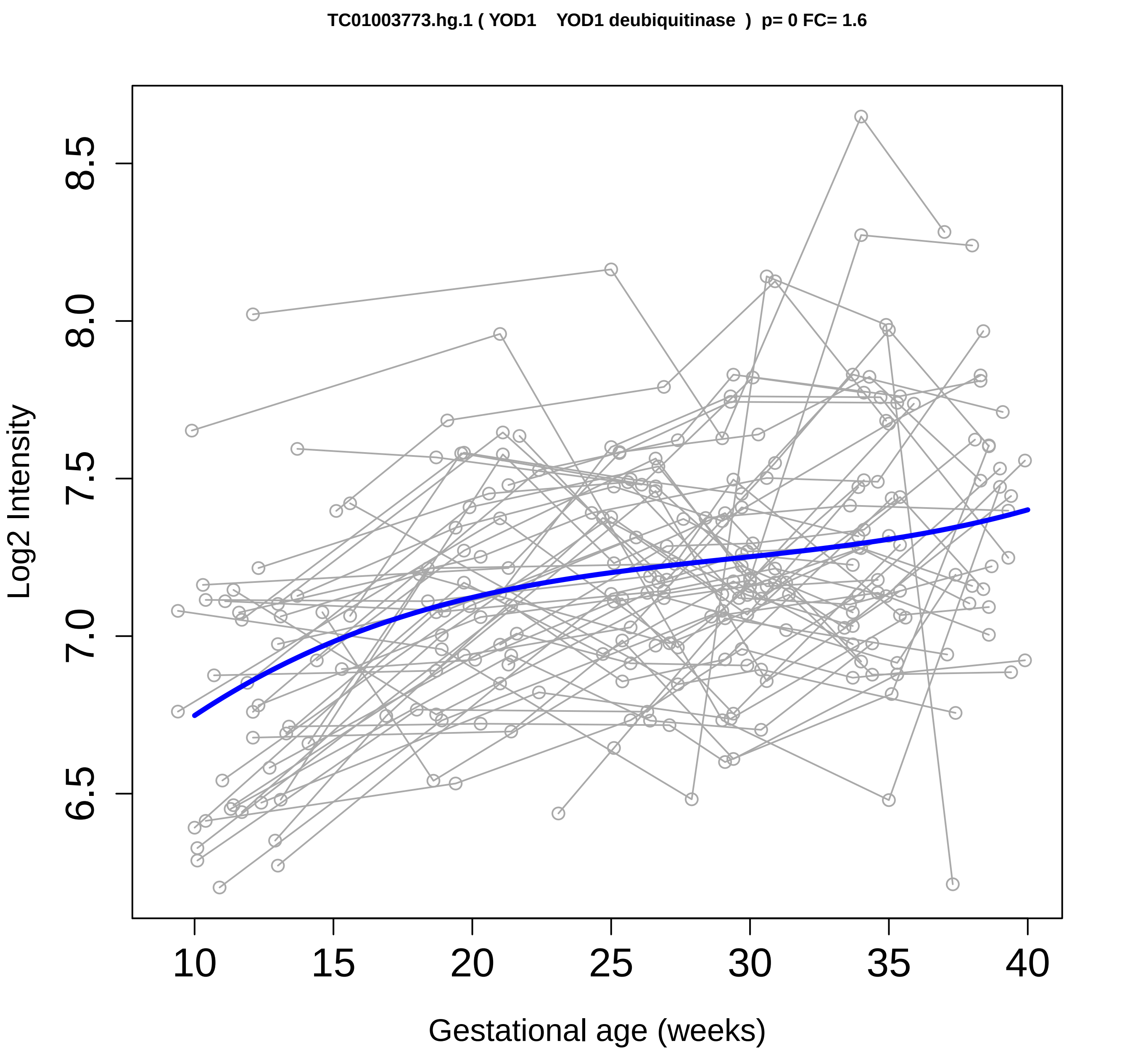
TC01003773.hg.1 (YOD1 YOD1 deubiquitinase) p= 0 FC= 1.6
Log2 Intensity
8.5
8.0
7.5
7.0
6.5
10
15
20
25
30
35
40
Gestational age (weeks)

TC03000164.hg.1 (CCR4 chemokine (C−C motif) receptor 4) p= 0 FC= −1.3

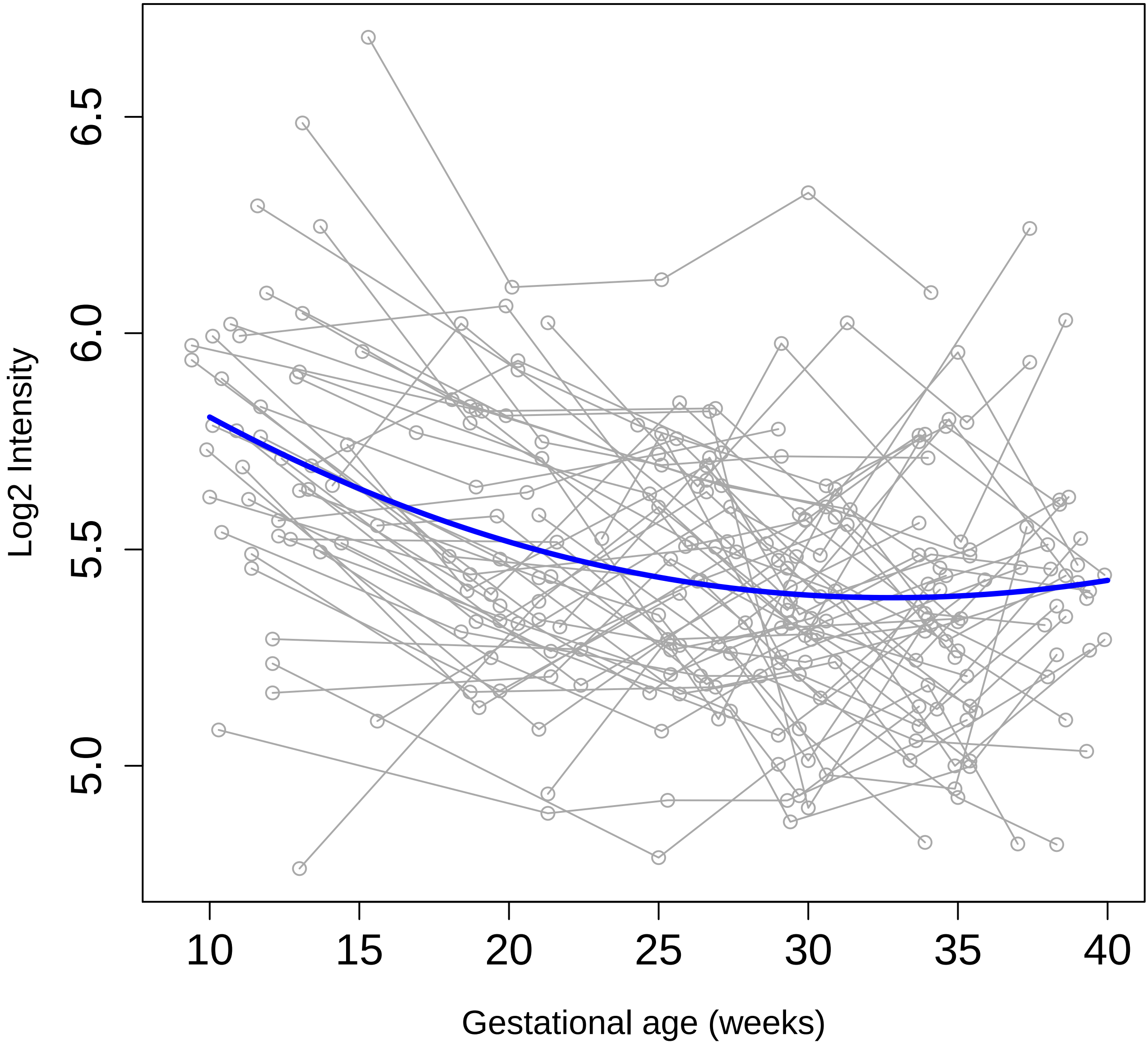

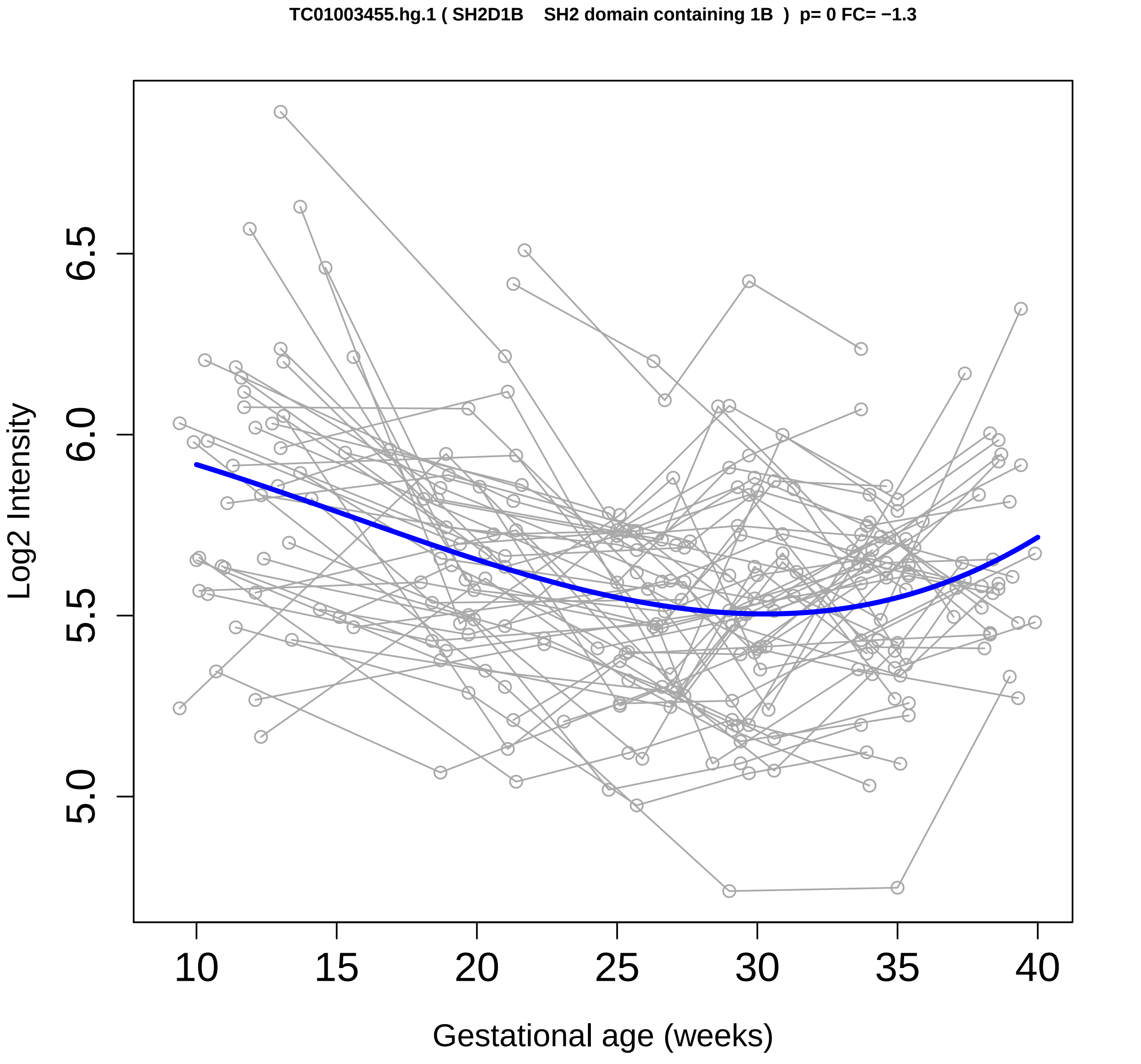

TC01003455.hg.1 (SH2D1B SH2 domain containing 1B) p= 0 FC= −1.3
Log2 Intensity
5.0
5.5
6.0
6.5
10
15
20
25
30
35
40
Gestational age (weeks)

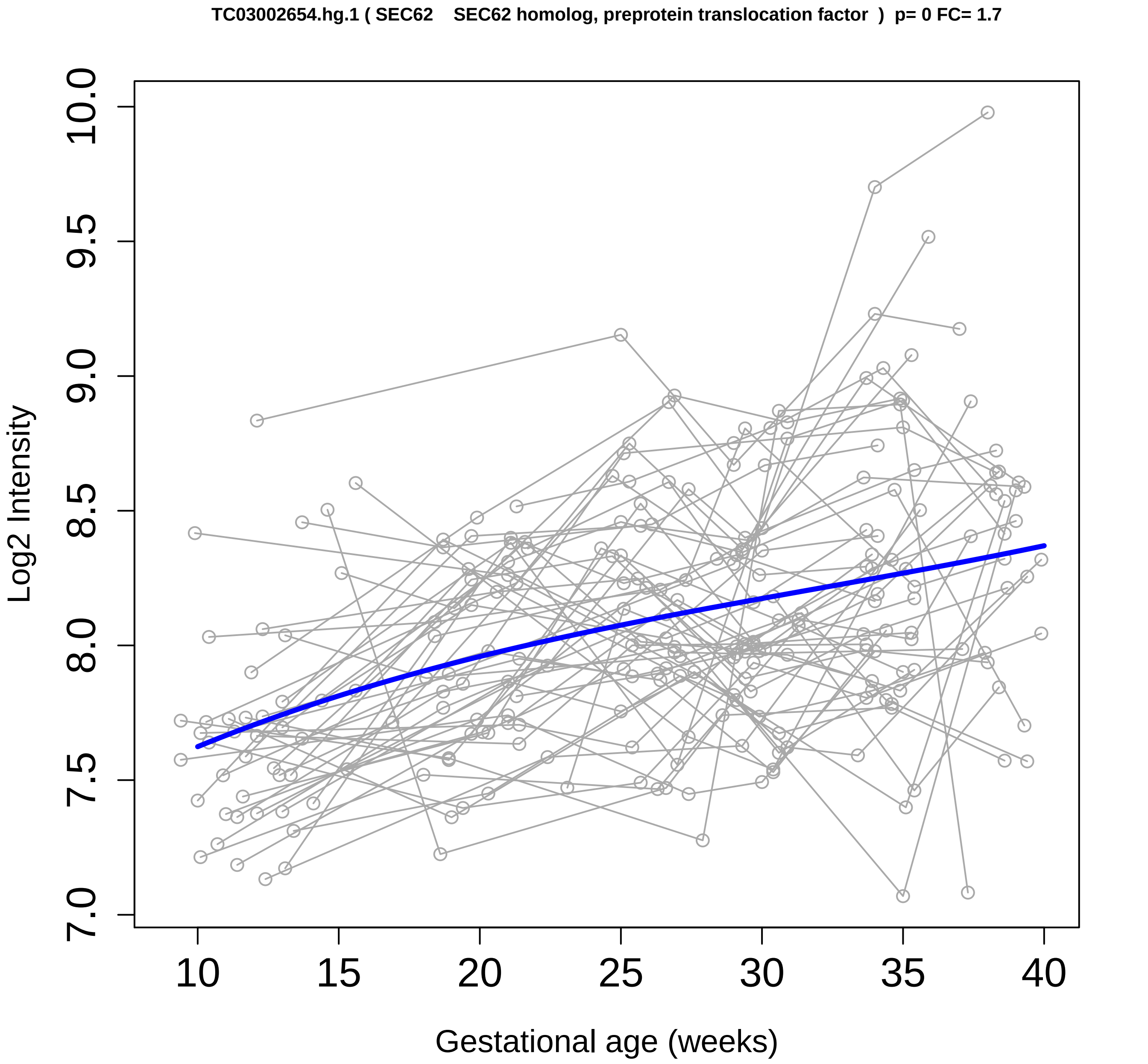
TC03002654.hg.1 (SEC62   SEC62 homolog, preprotein translocation factor) p= 0 FC= 1.7
Log2 Intensity
7.0
7.5
8.0
8.5
9.0
9.5
10.0
10
15
20
25
30
35
40
Gestational age (weeks)

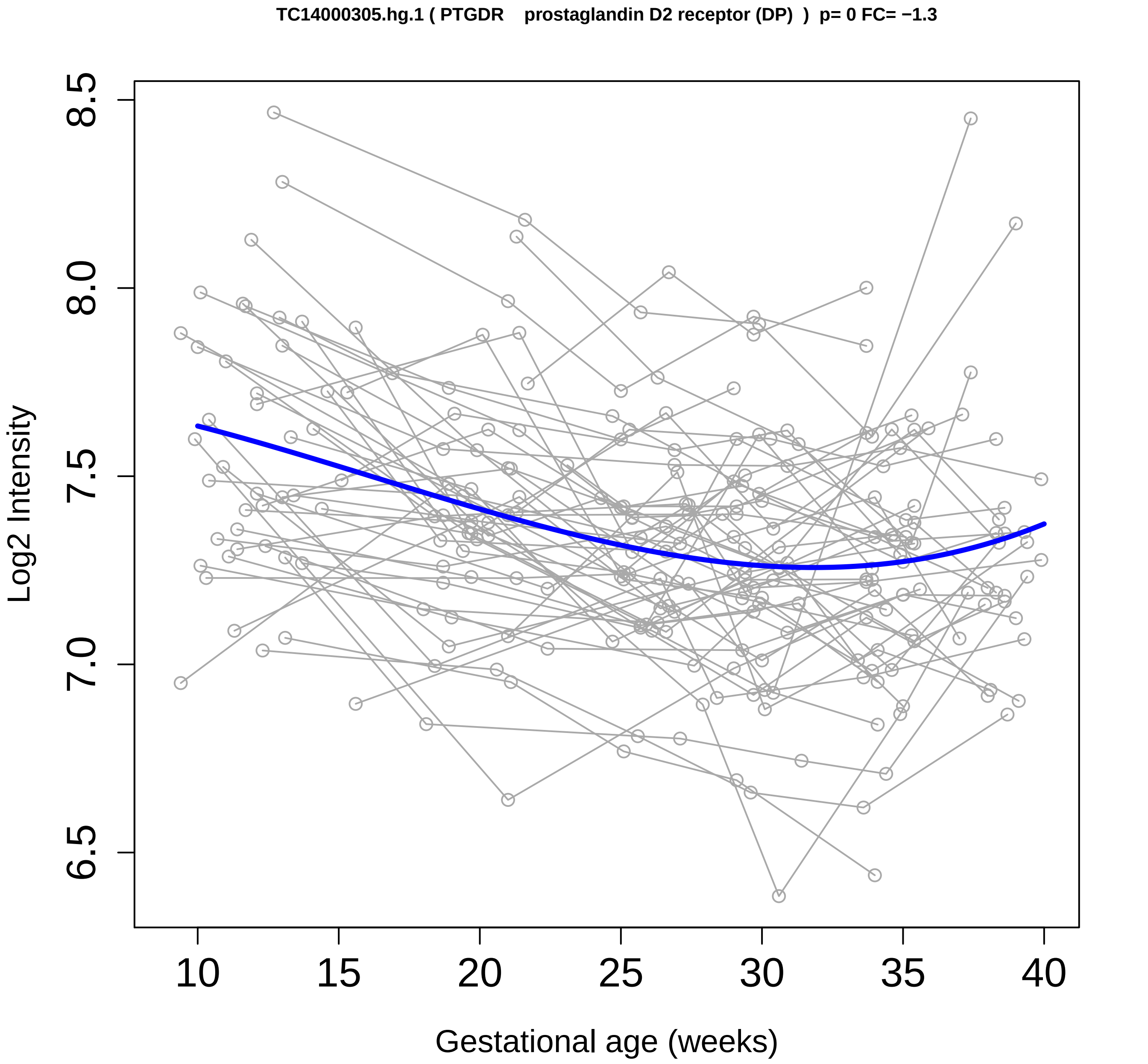

TC14000305.hg.1 (PTGDR prostaglandin D2 receptor (DP)) p= 0 FC= −1.3
Log2 Intensity
6.5
7.0
7.5
8.0
8.5
10
15
20
25
30
35
40
Gestational age (weeks)

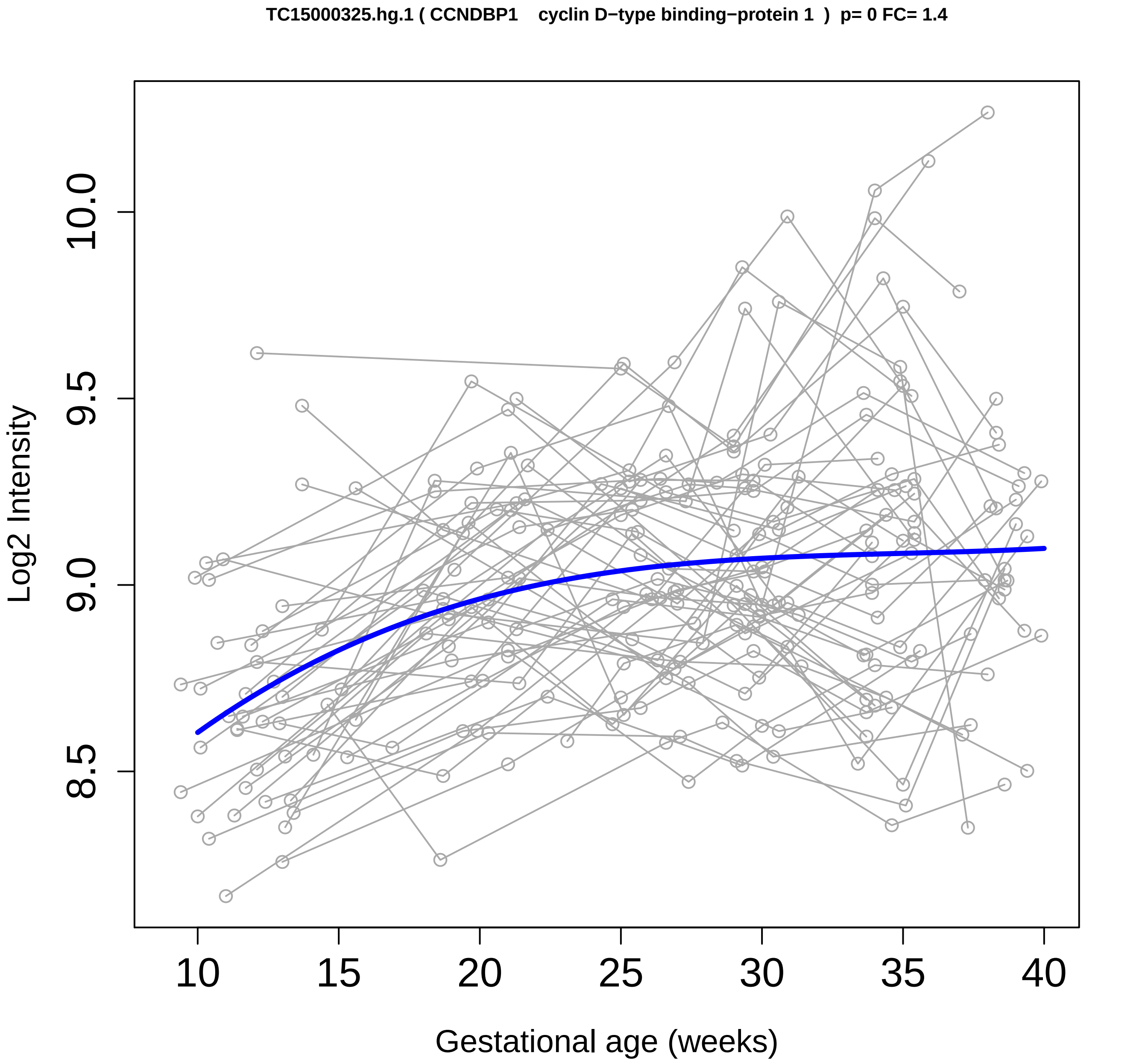

TC15000325.hg.1 (CCNDBP1 cyclin D−type binding−protein 1) p= 0 FC= 1.4
Log2 Intensity
10.0
9.5
9.0
8.5
10
15
20
25
30
35
40
Gestational age (weeks)

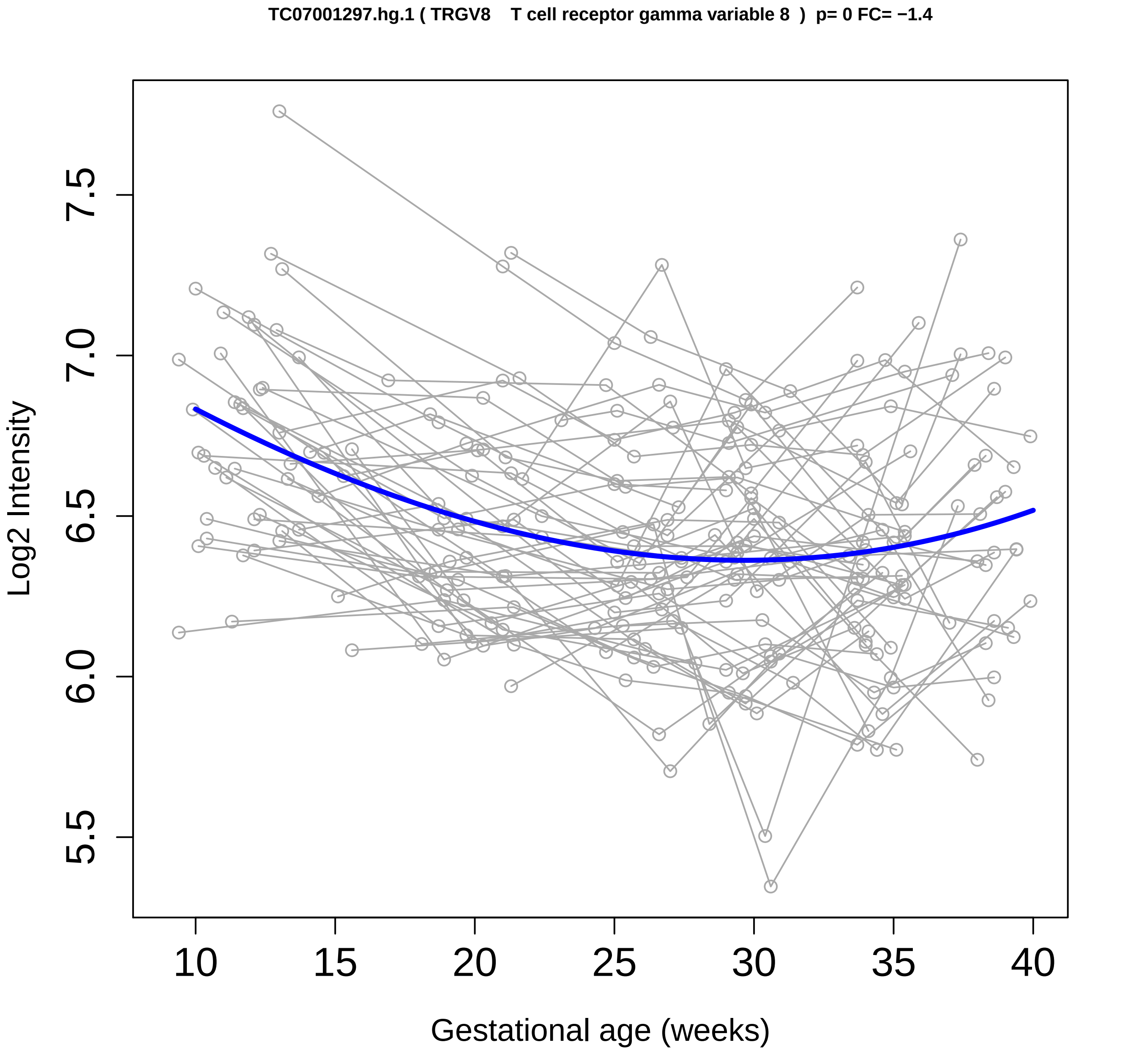

TC07001297.hg.1 (TRGV8   T cell receptor gamma variable 8) p= 0 FC= −1.4
Log2 Intensity
7.5
7.0
6.5
6.0
5.5
10
15
20
25
30
35
40
Gestational age (weeks)

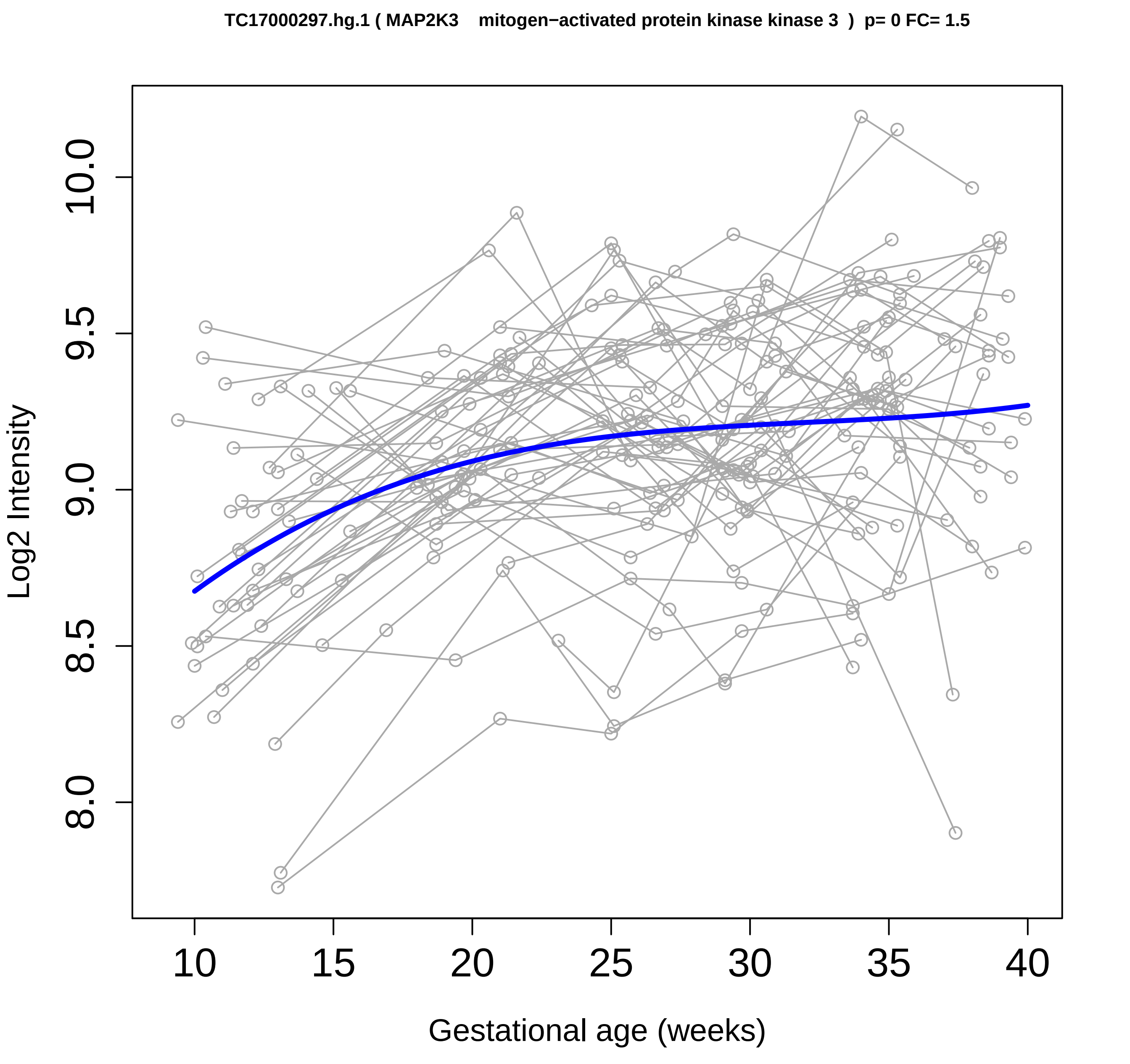
TC17000297.hg.1 (MAP2K3 mitogen−activated protein kinase kinase 3) p= 0 FC= 1.5
Log2 Intensity
8.0
8.5
9.0
9.5
10.0
10
15
20
25
30
35
40
Gestational age (weeks)

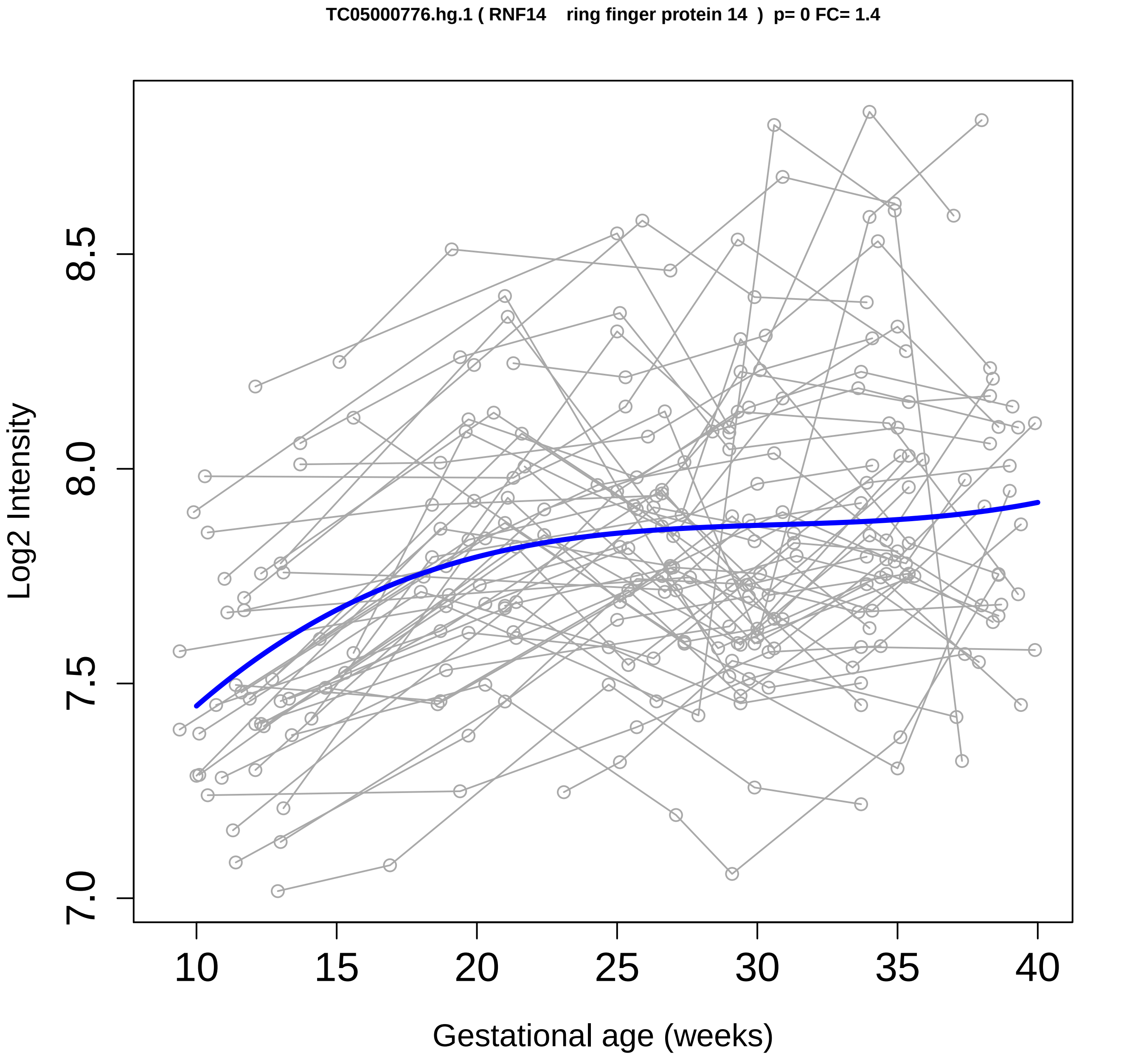

TC05000776.hg.1 (RNF14 ring finger protein 14) p= 0 FC= 1.4
Log2 Intensity
7.0
7.5
8.0
8.5
10
15
20
25
30
35
40
Gestational age (weeks)

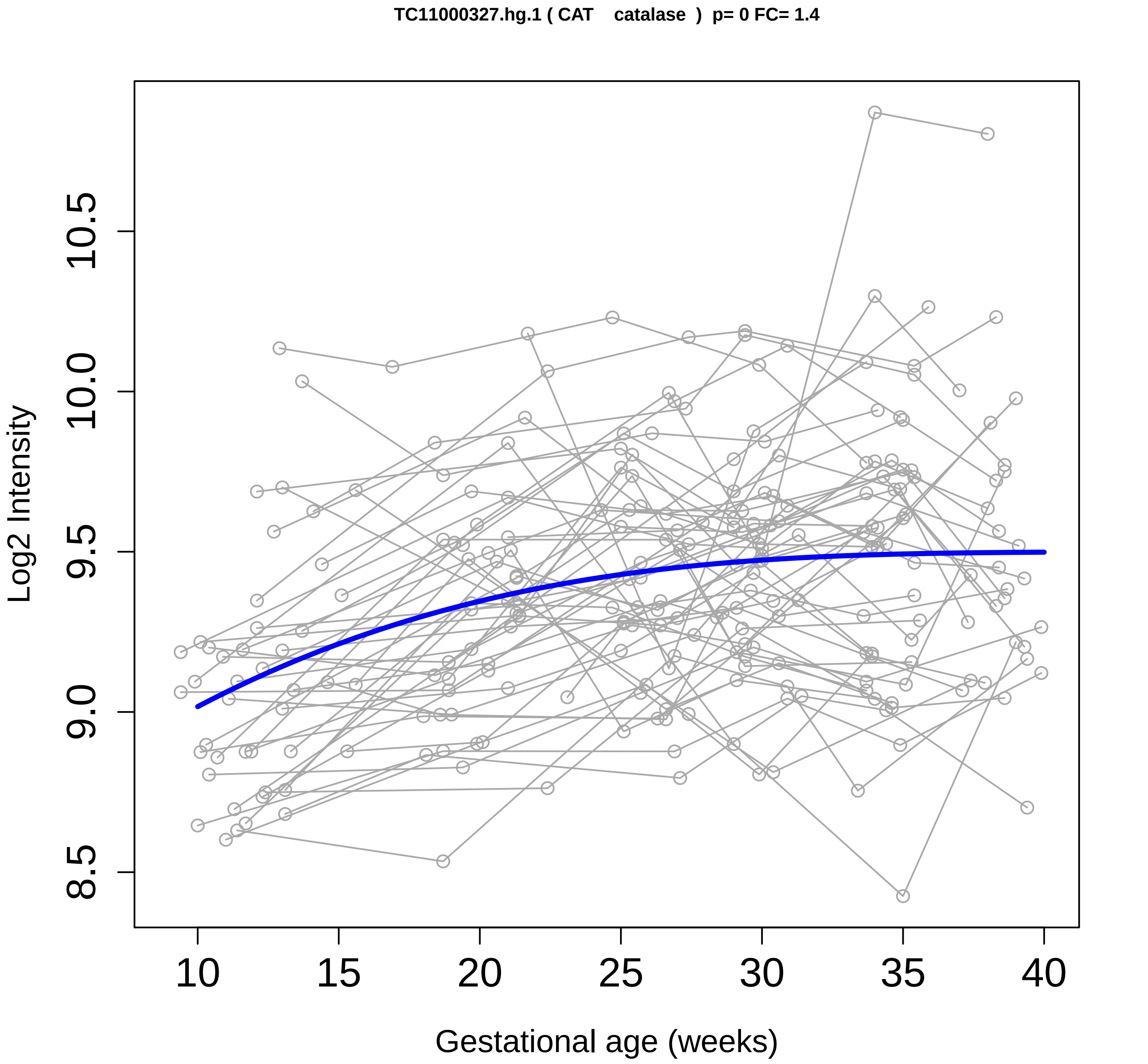
TC11000327.hg.1 (CAT catalase) p= 0 FC= 1.4
Log2 Intensity
8.5
9.0
9.5
10.0
10.5
10
15
20
25
30
35
40
Gestational age (weeks)

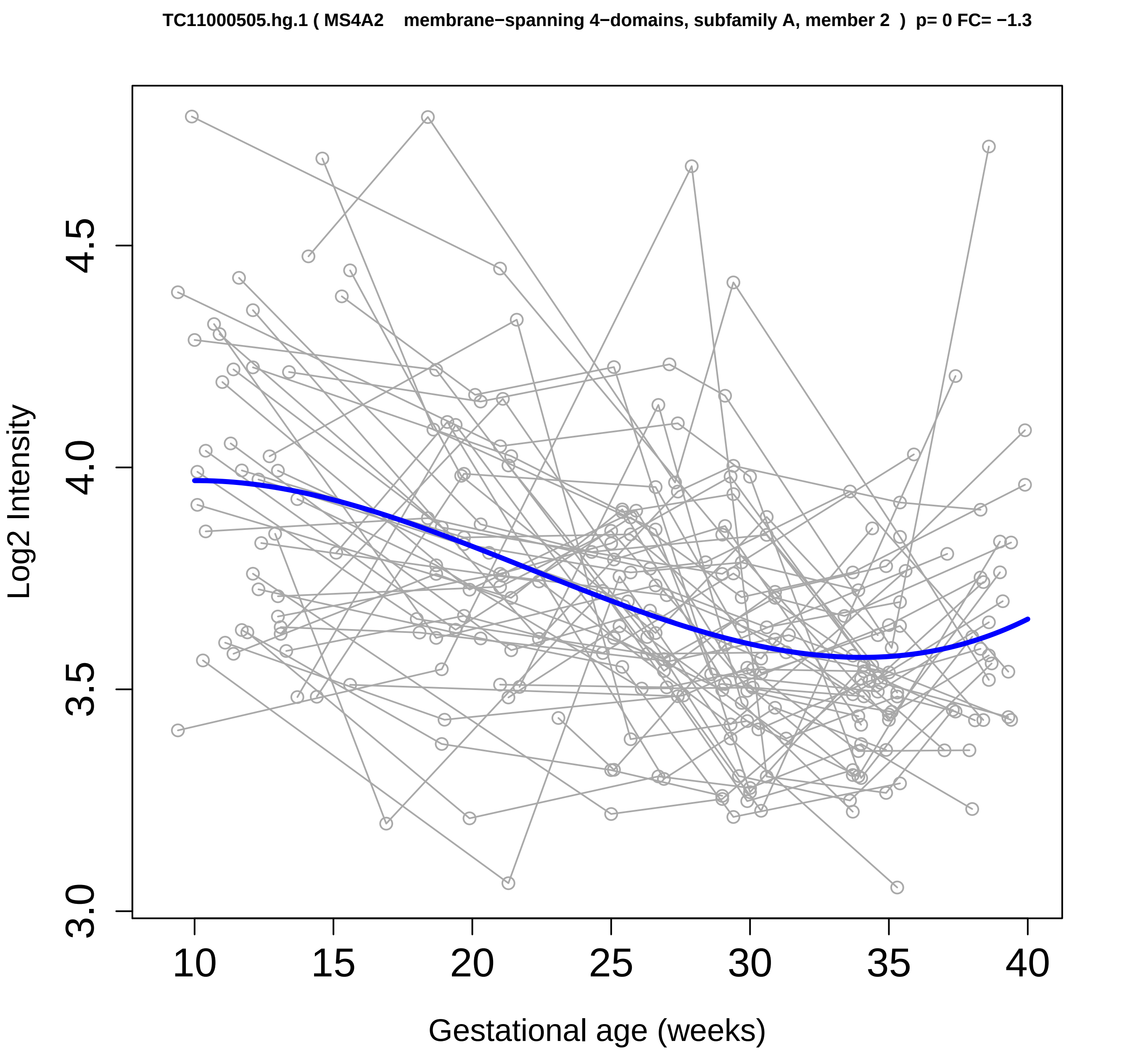

TC11000505.hg.1 (MS4A2 membrane−spanning 4−domains, subfamily A, member 2) p= 0 FC= −1.3
Log2 Intensity
3.0
3.5
4.0
4.5
10
15
20
25
30
35
40
Gestational age (weeks)

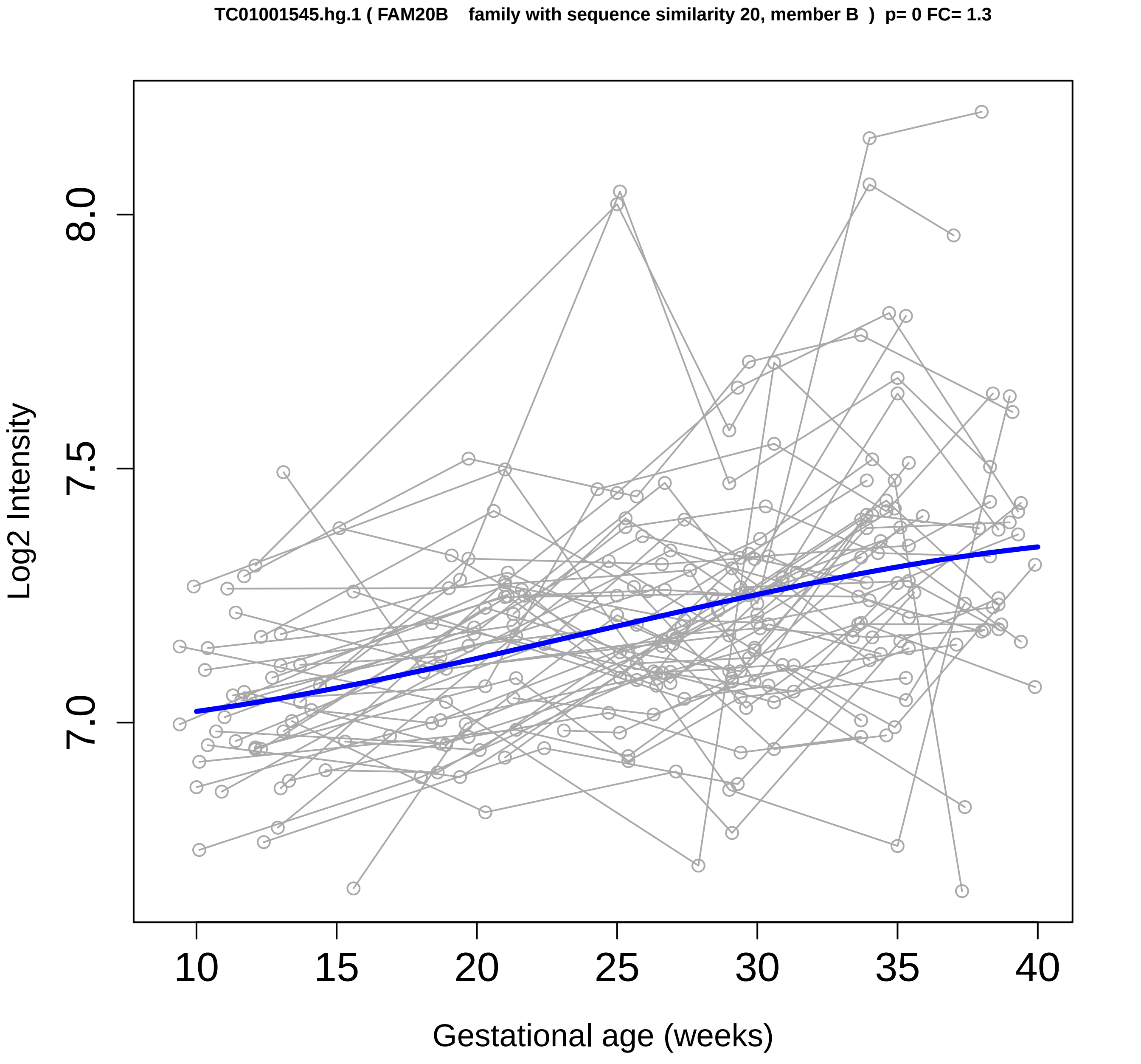

TC01001545.hg.1 (FAM20B family with sequence similarity 20, member B) p= 0 FC= 1.3
Log2 Intensity
7.0
7.5
8.0
10
15
20
25
30
35
40
Gestational age (weeks)

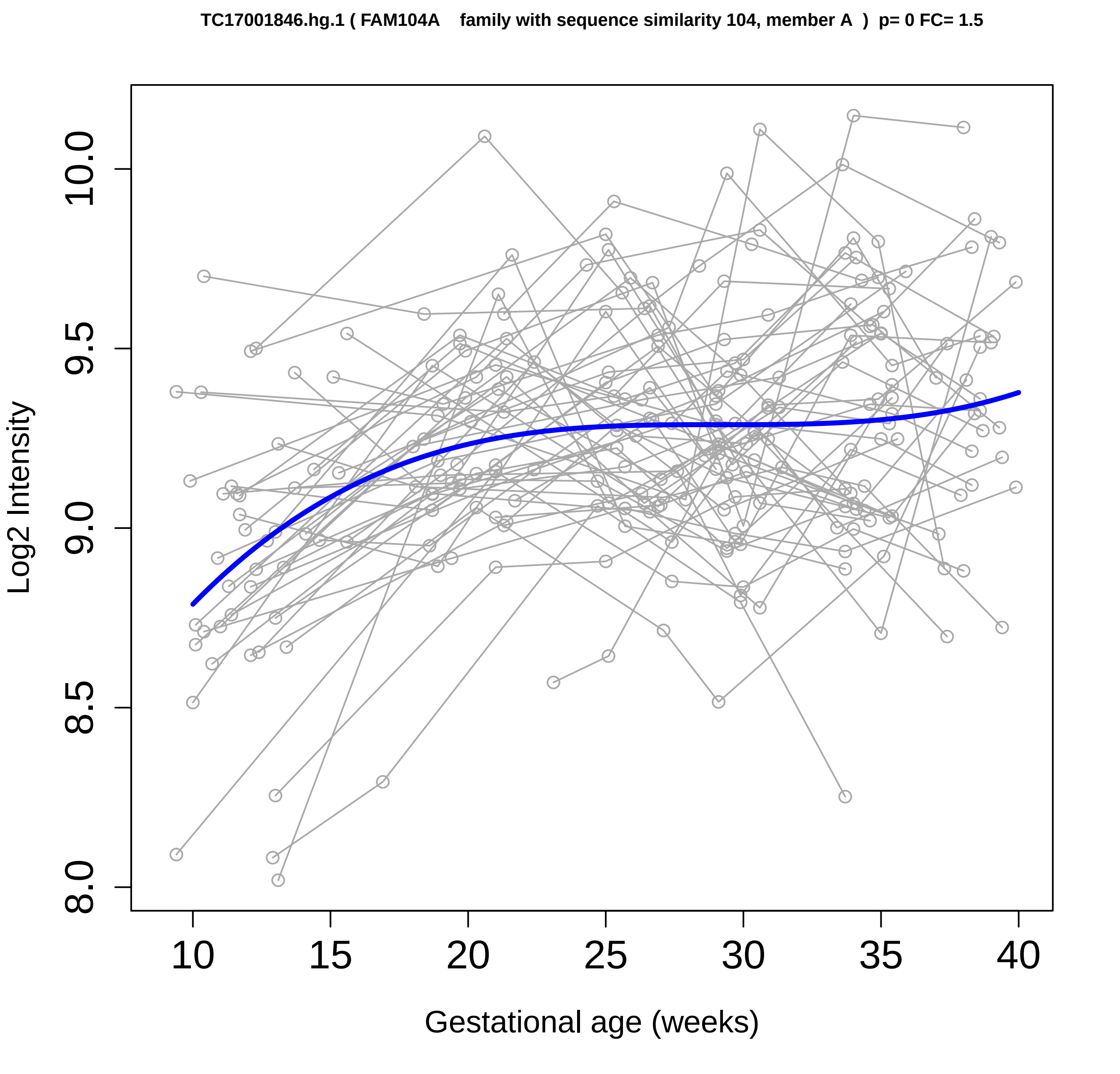

TC17001846.hg.1 (FAM104A family with sequence similarity 104, member A) p= 0 FC= 1.5
Log2 Intensity
8.0
8.5
9.0
9.5
10.0
10
15
20
25
30
35
40
Gestational age (weeks)

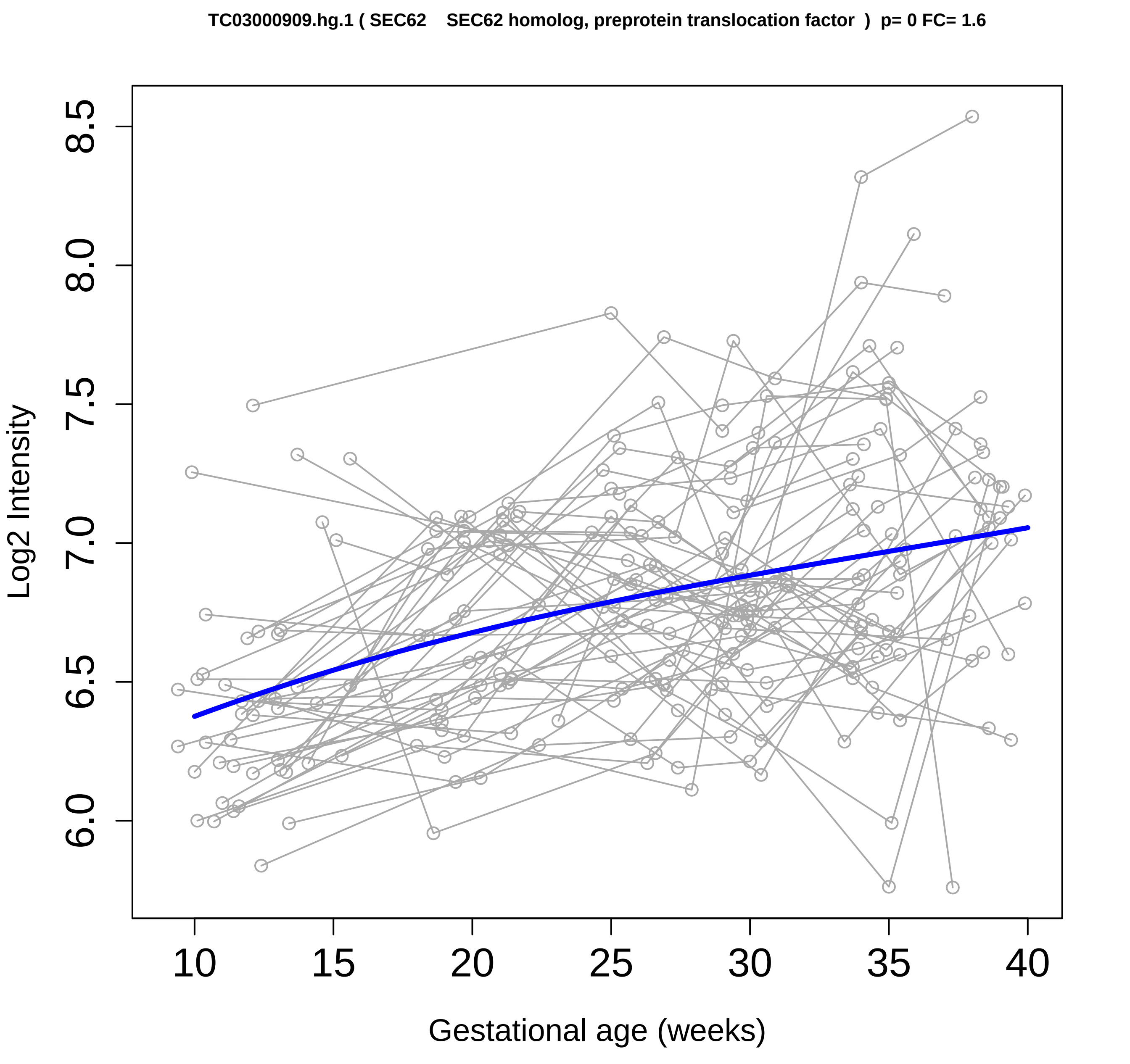
TC03000909.hg.1 (SEC62   SEC62 homolog, preprotein translocation factor) p= 0 FC= 1.6
Log2 Intensity
6.0
6.5
7.0
7.5
8.0
8.5
10
15
20
25
30
35
40
Gestational age (weeks)

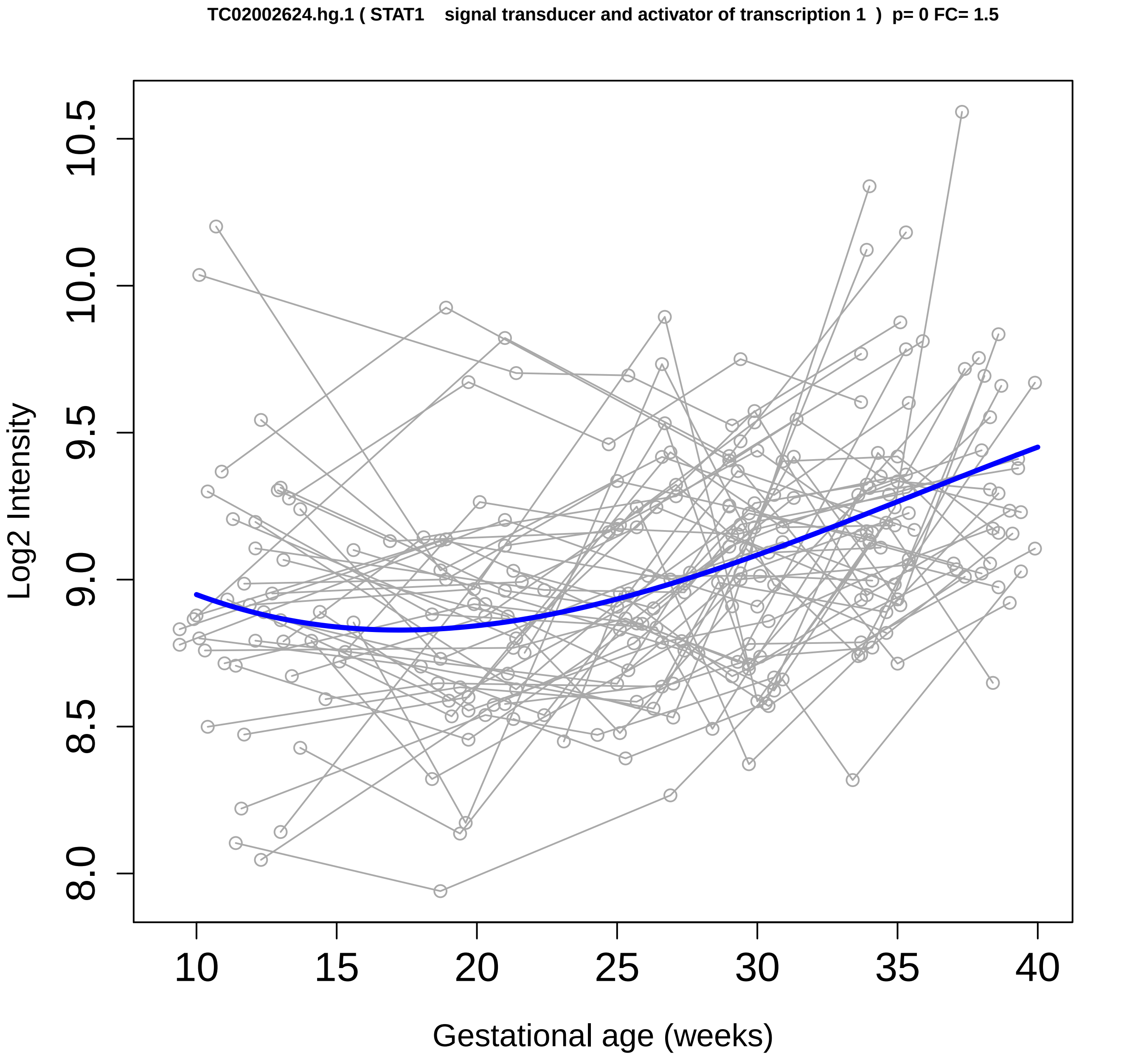

TC02002624.hg.1 (STAT1   signal transducer and activator of transcription 1) p= 0 FC= 1.5
Log2 Intensity
10.5
10.0
9.5
9.0
8.5
8.0
10
15
20
25
30
35
40
Gestational age (weeks)

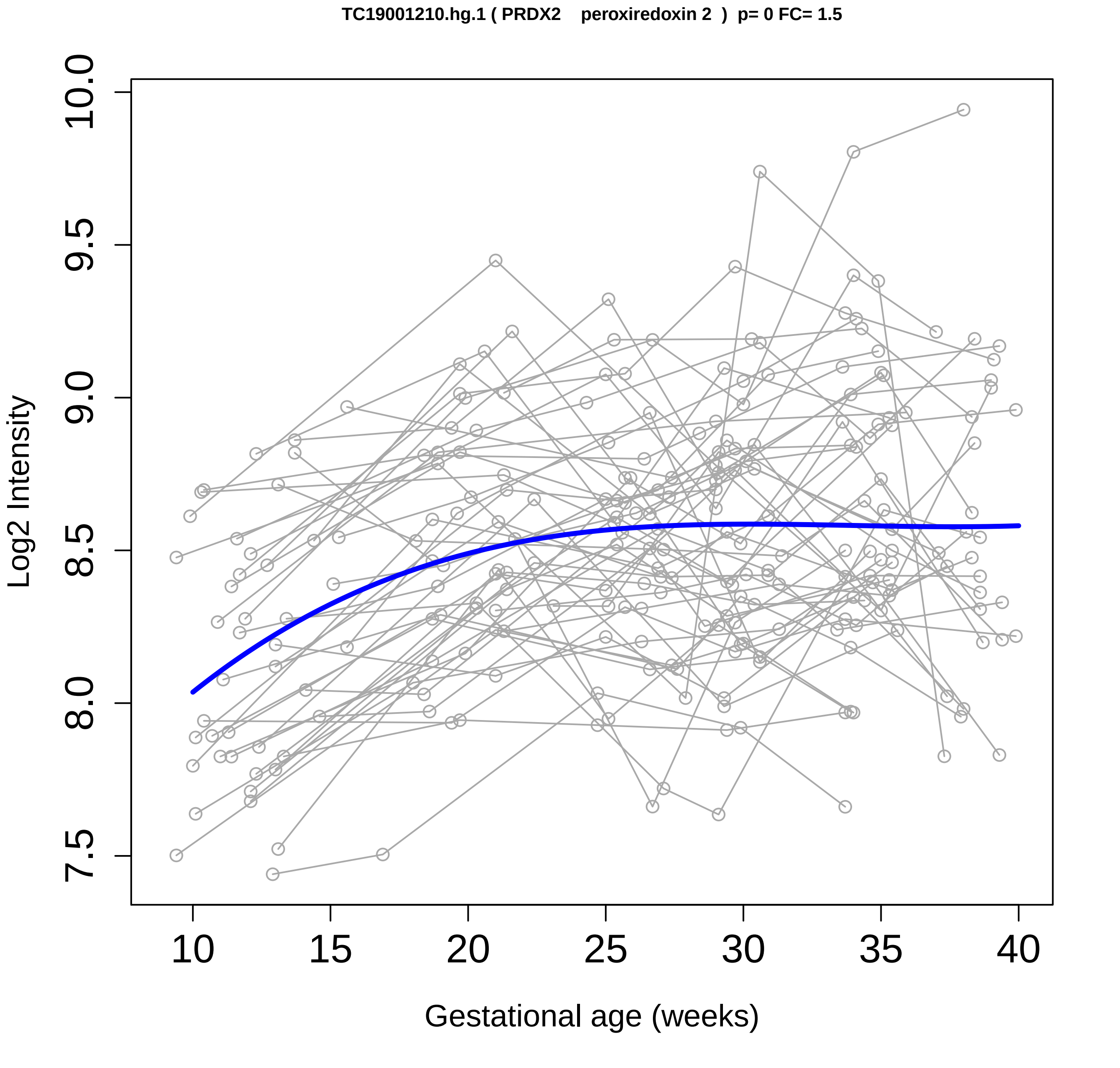
TC19001210.hg.1 (PRDX2 peroxiredoxin 2) p= 0 FC= 1.5
Log2 Intensity
10.0
9.5
9.0
8.5
8.0
7.5
10
15
20
25
30
35
40
Gestational age (weeks)

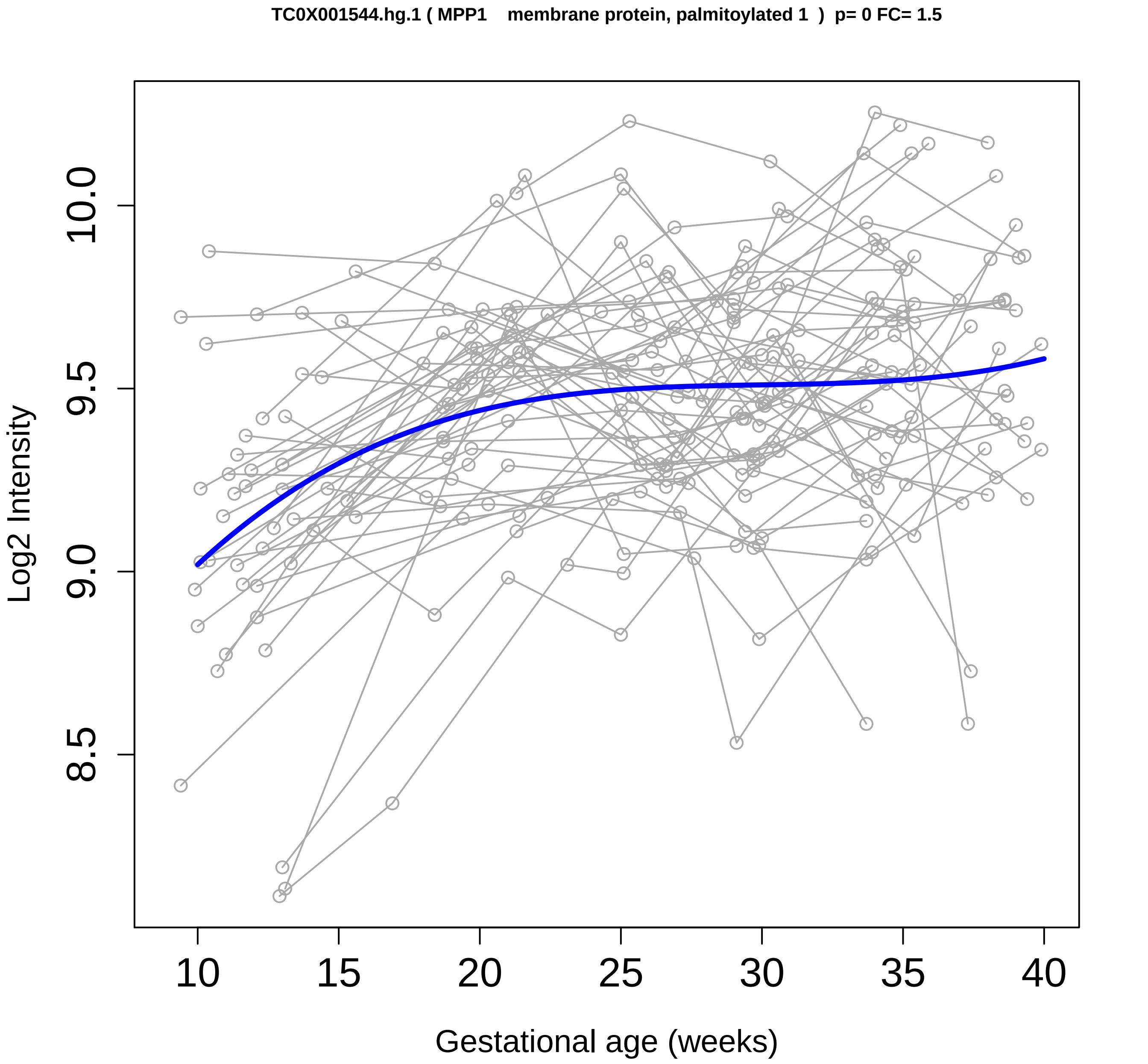

TC0X001544.hg.1 (MPP1   membrane protein, palmitoylated 1) p= 0 FC= 1.5
Log2 Intensity
8.5
9.0
9.5
10.0
10
15
20
25
30
35
40
Gestational age (weeks)

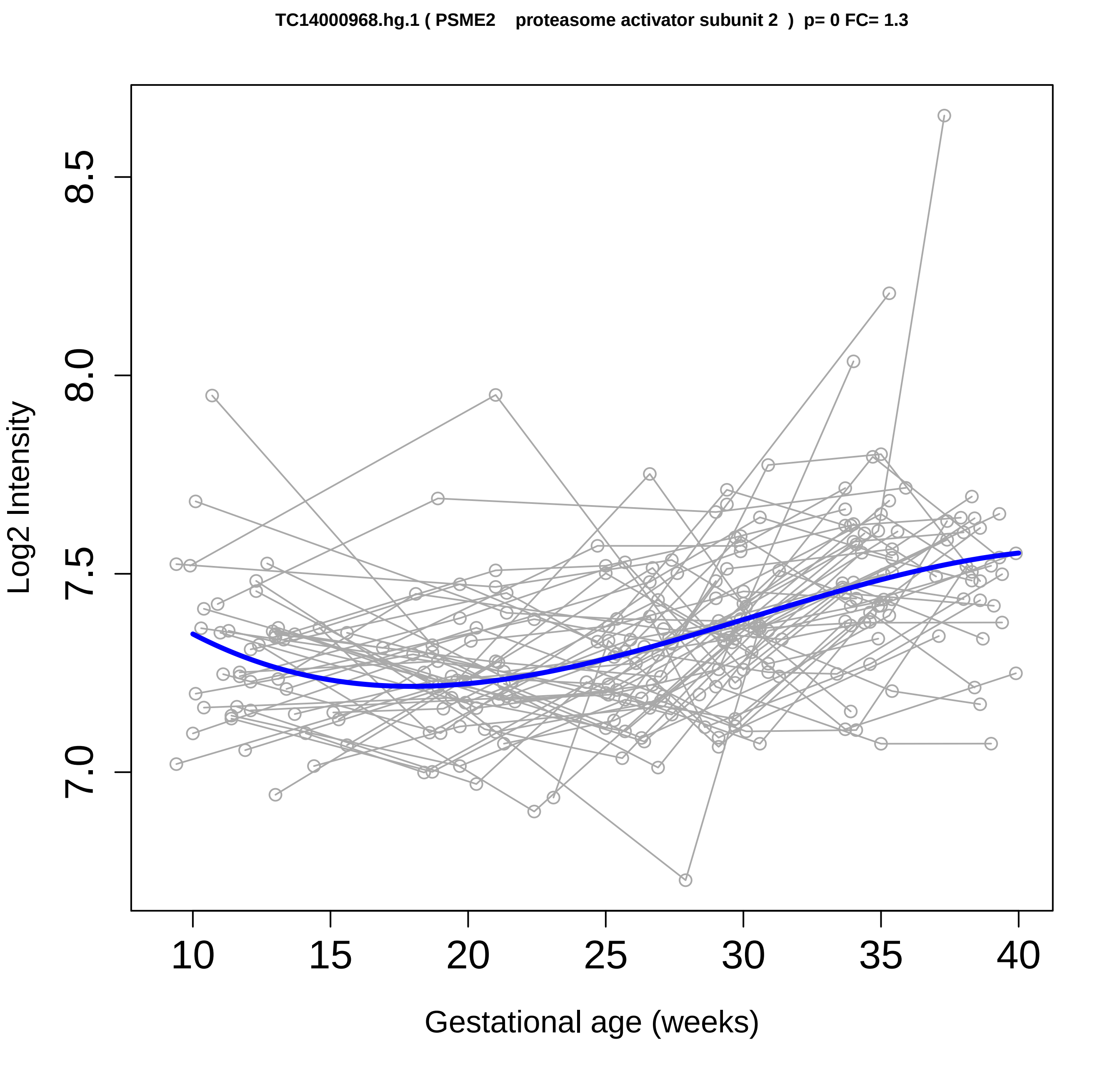

TC14000968.hg.1 (PSME2 proteasome activator subunit 2) p= 0 FC= 1.3
Log2 Intensity
7.0
7.5
8.0
8.5
10
15
20
25
30
35
40
Gestational age (weeks)

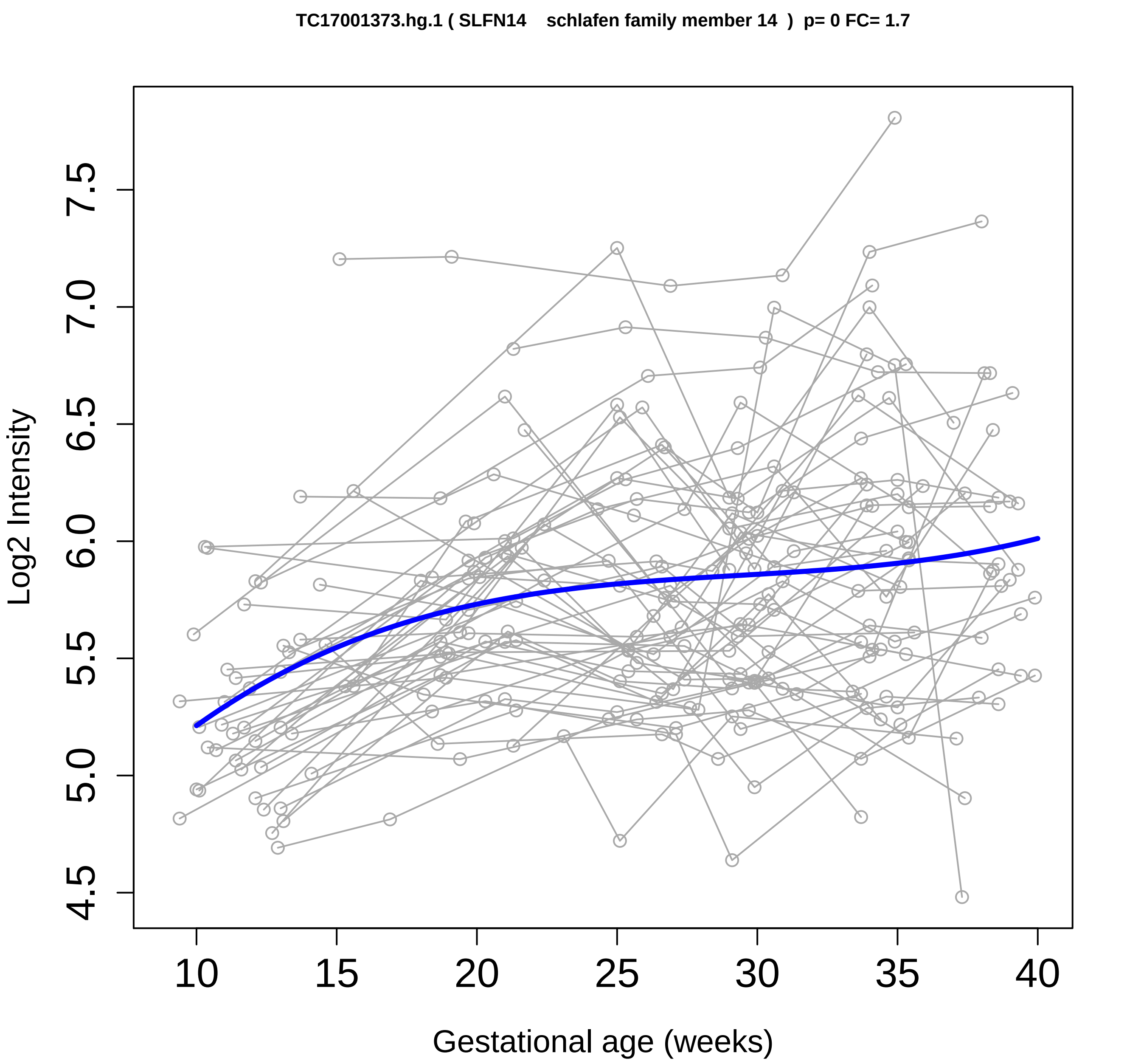

TC17001373.hg.1 (SLFN14 schlafen family member 14) p= 0 FC= 1.7
Log2 Intensity
4.5
5.0
5.5
6.0
6.5
7.0
7.5
10
15
20
25
30
35
40
Gestational age (weeks)

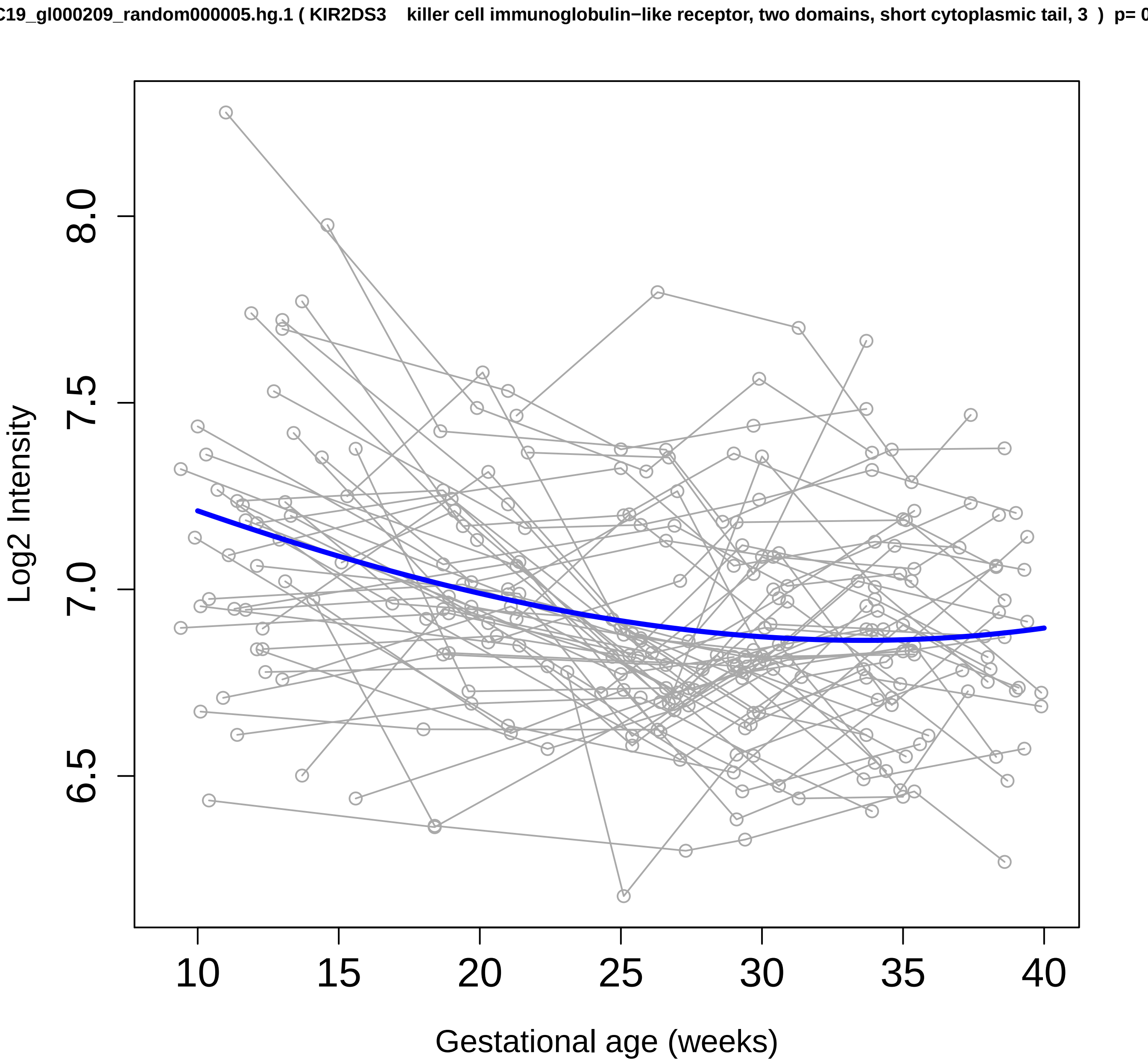

C19_gl000209_random000005.hg.1 (KIR2DS3 killer cell immunoglobulin−like receptor, two domains, short cytoplasmic tail, 3) p= 0
Log2 Intensity
8.0
7.5
7.0
6.5
10
15
20
25
30
35
40
Gestational age (weeks)

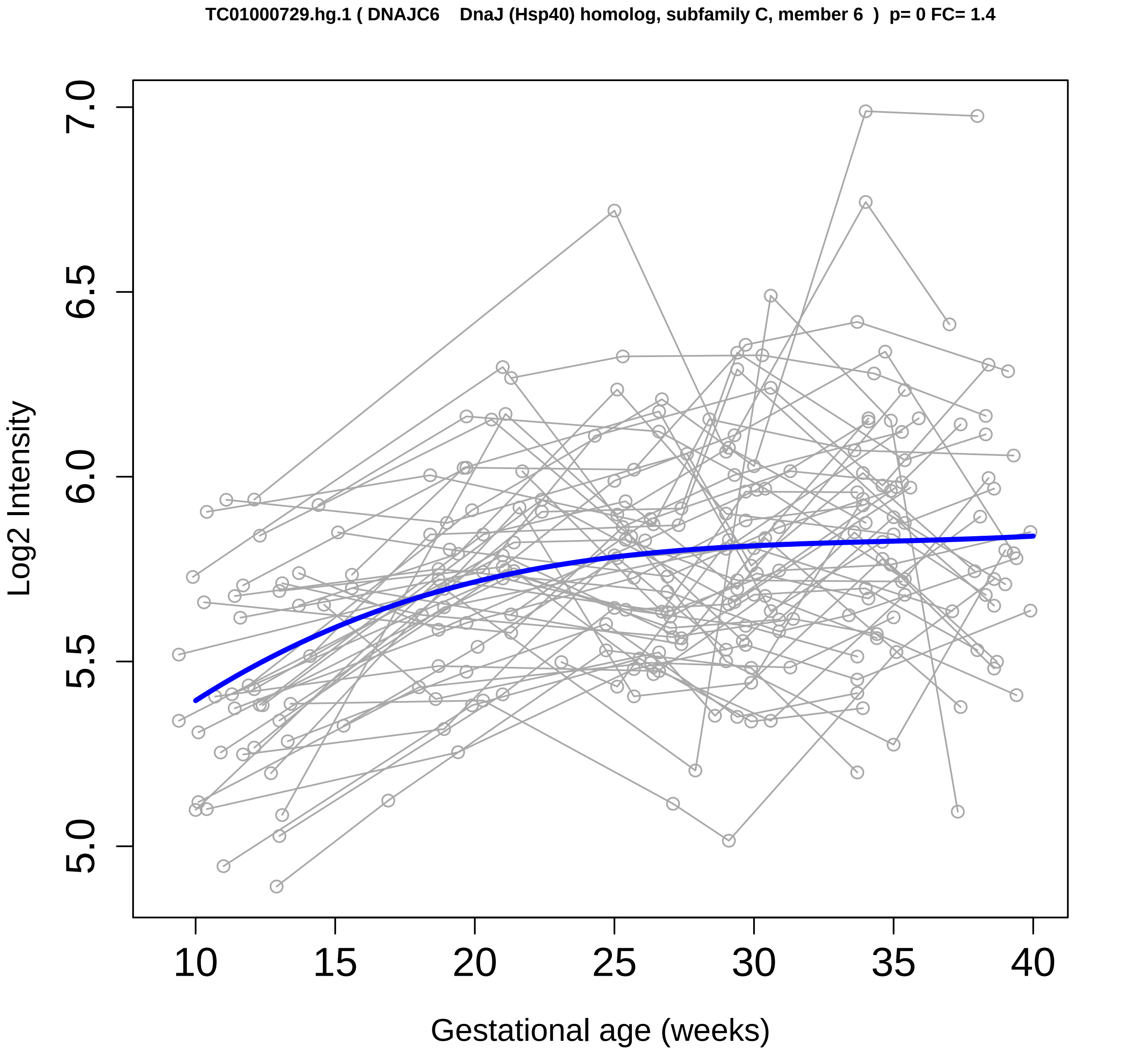
TC01000729.hg.1 (DNAJC6   DnaJ (Hsp40) homolog, subfamily C, member 6) p= 0 FC= 1.4
Log2 Intensity
5.0
5.5
6.0
6.5
7.0
10
15
20
25
30
35
40
Gestational age (weeks)

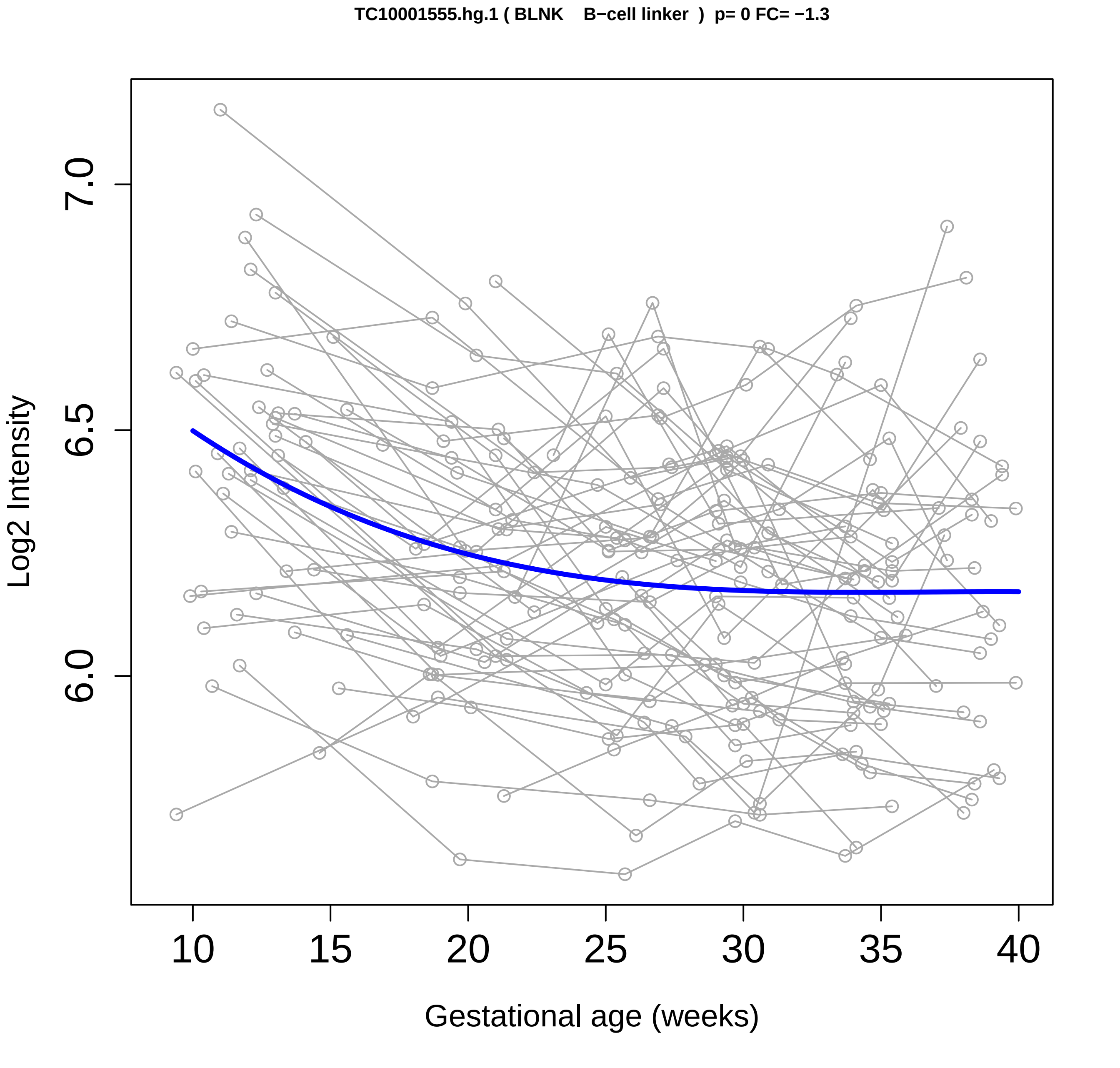
TC10001555.hg.1 (BLNK B−cell linker) p= 0 FC= −1.3
Log2 Intensity
7.0
6.5
6.0
10
15
20
25
30
35
40
Gestational age (weeks)

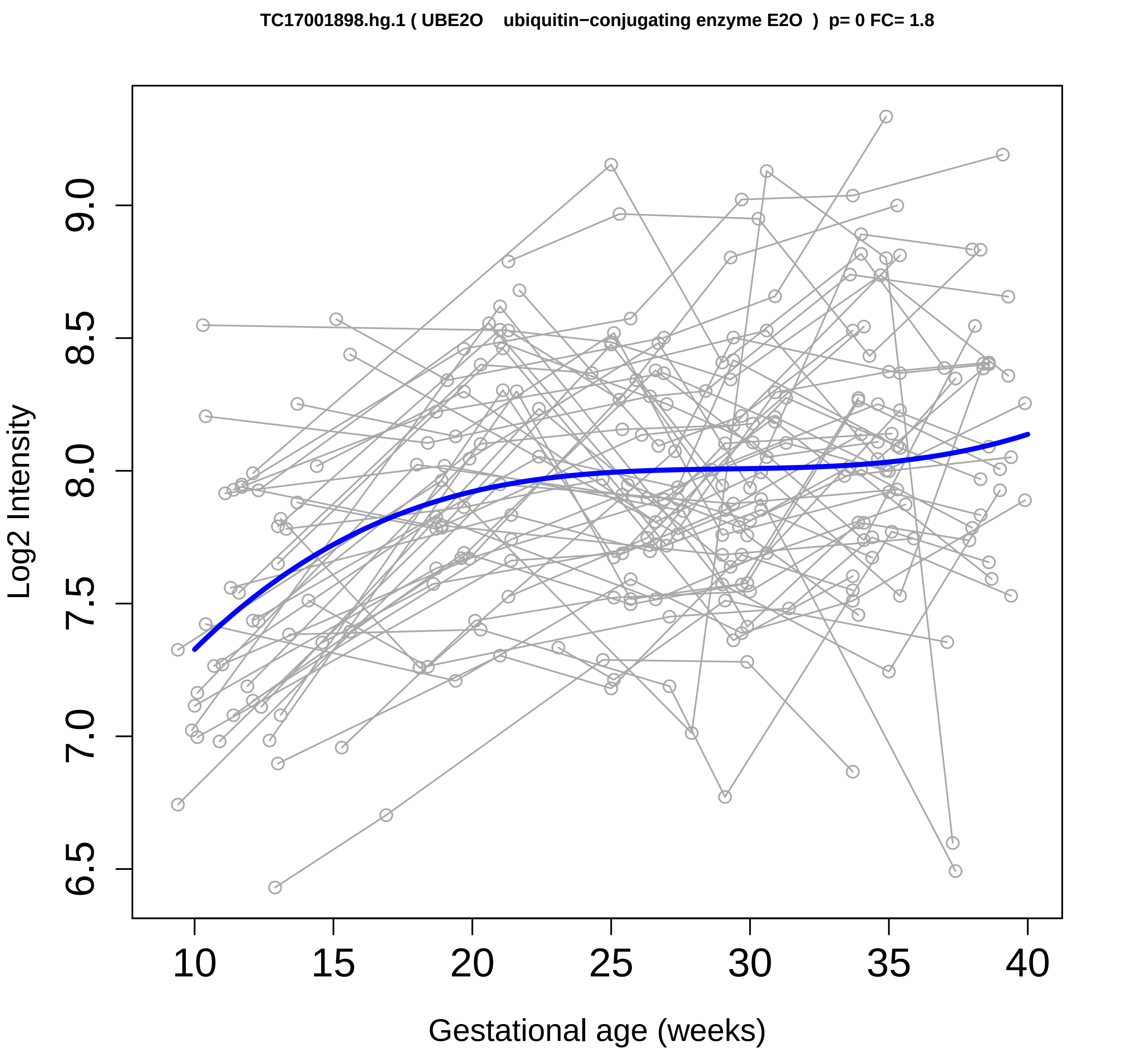

TC17001898.hg.1 (UBE2O ubiquitin−conjugating enzyme E2O) p= 0 FC= 1.8
Log2 Intensity
6.5
7.0
7.5
8.0
8.5
9.0
10
15
20
25
30
35
40
Gestational age (weeks)

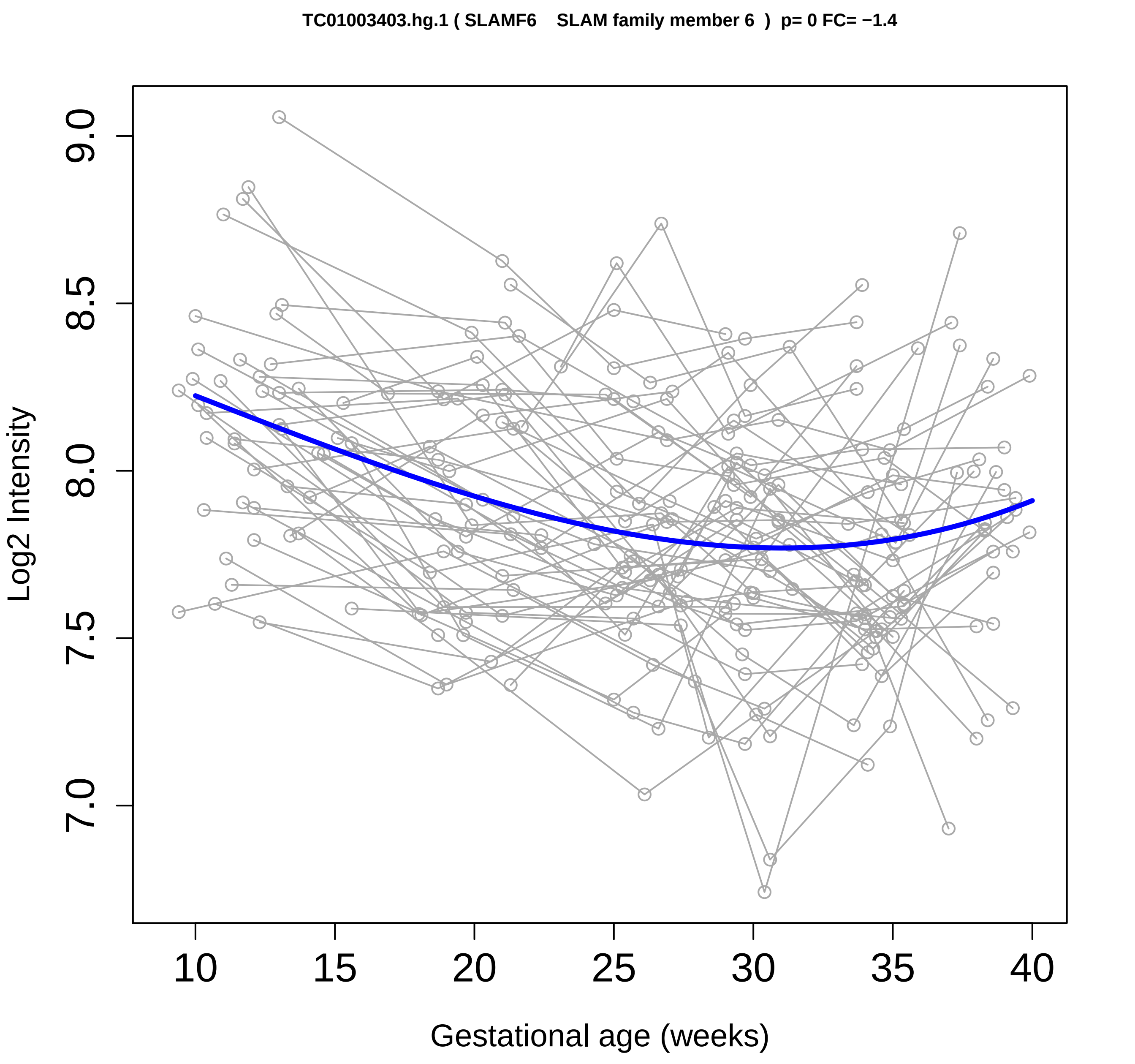

TC01003403.hg.1 (SLAMF6   SLAM family member 6) p= 0 FC= −1.4
Log2 Intensity
9.0
8.5
8.0
7.5
7.0
10
15
20
25
30
35
40
Gestational age (weeks)

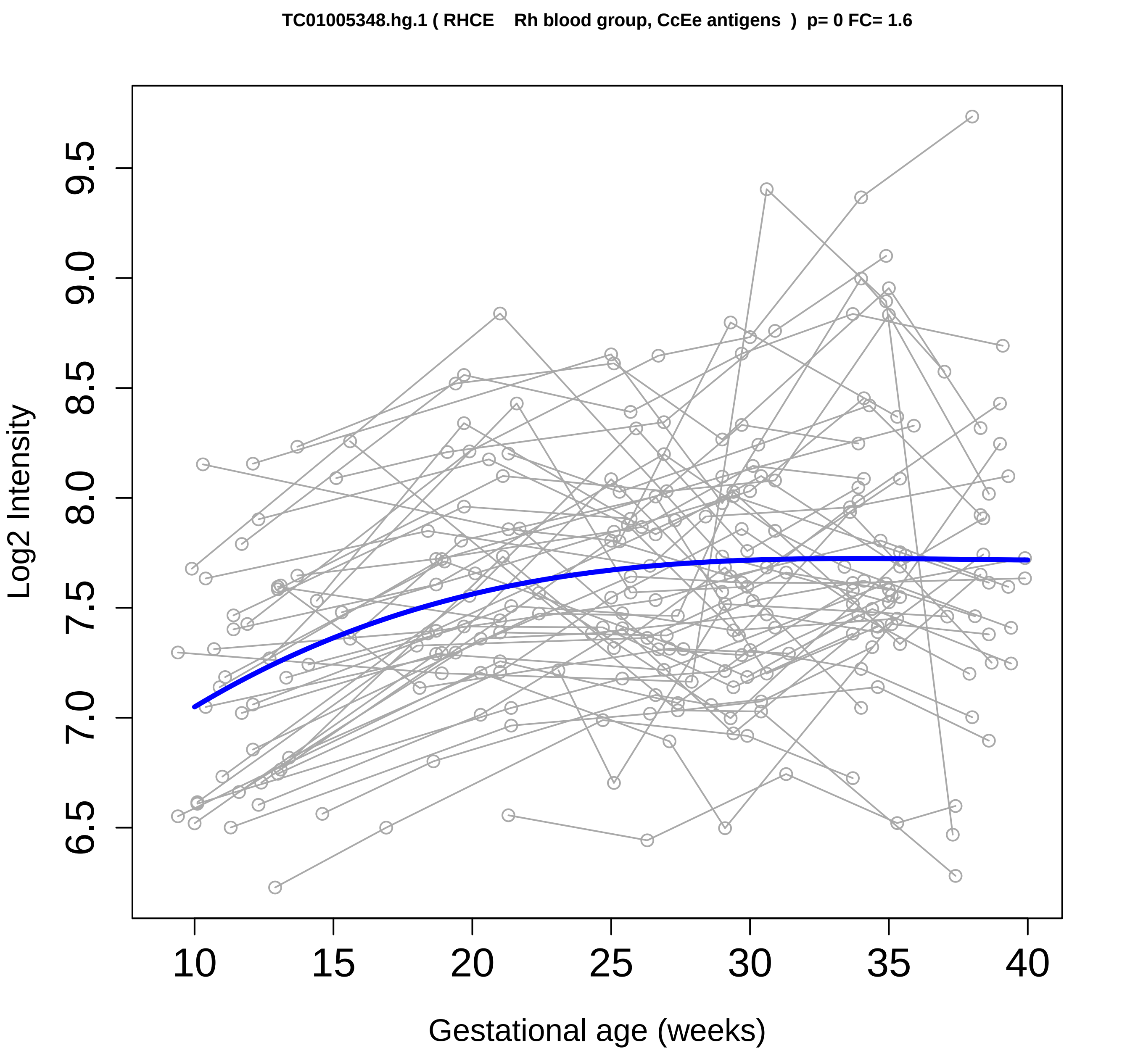

TC01005348.hg.1 (RHCE   Rh blood group, CcEe antigens) p= 0 FC= 1.6
Log2 Intensity
6.5
7.0
7.5
8.0
8.5
9.0
9.5
10
15
20
25
30
35
40
Gestational age (weeks)

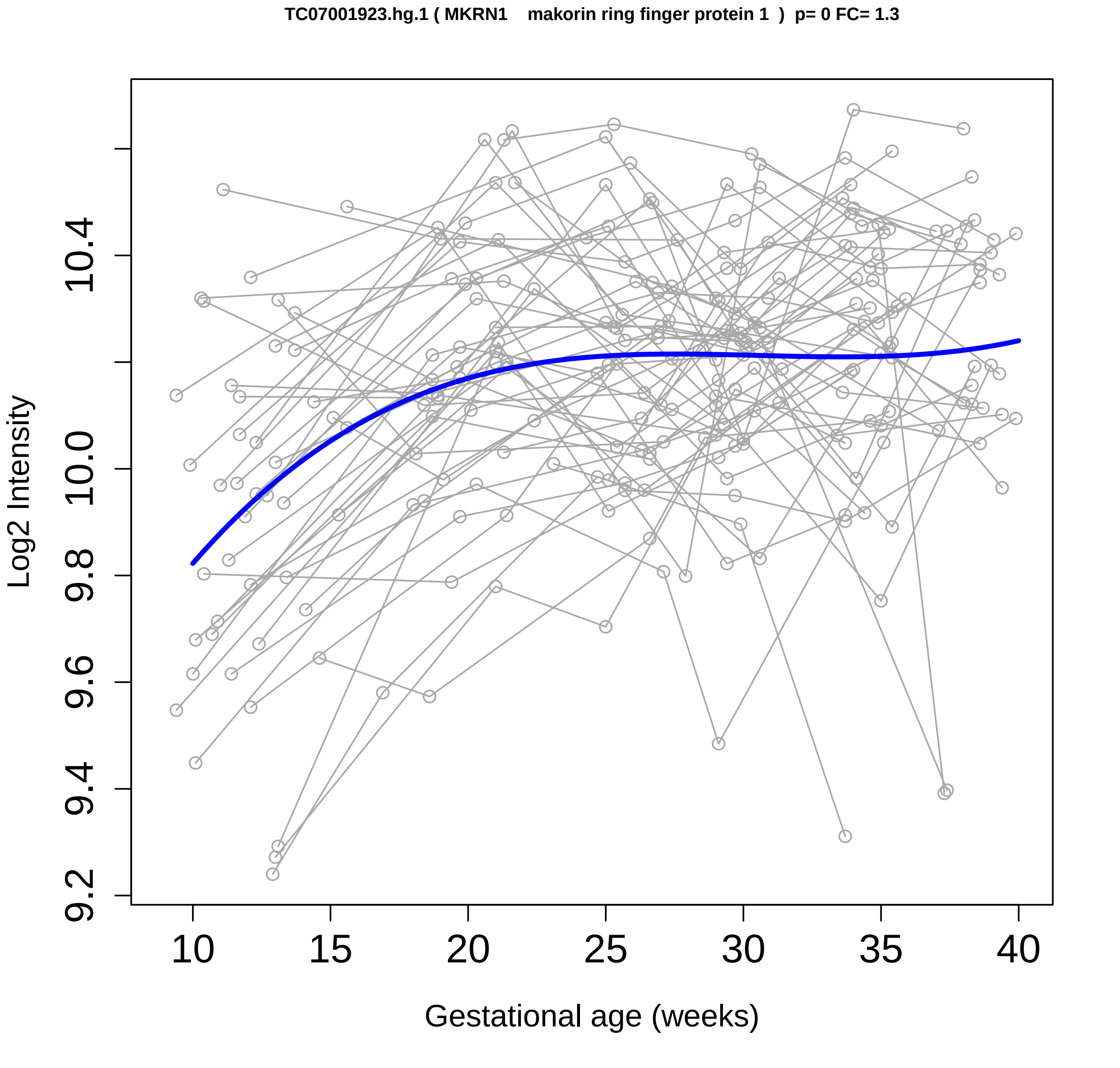

TC07001923.hg.1 (MKRN1 makorin ring finger protein 1) p= 0 FC= 1.3
Log2 Intensity
9.2
9.4
9.6
9.8
10.0
10.4
10
15
20
25
30
35
40
Gestational age (weeks)

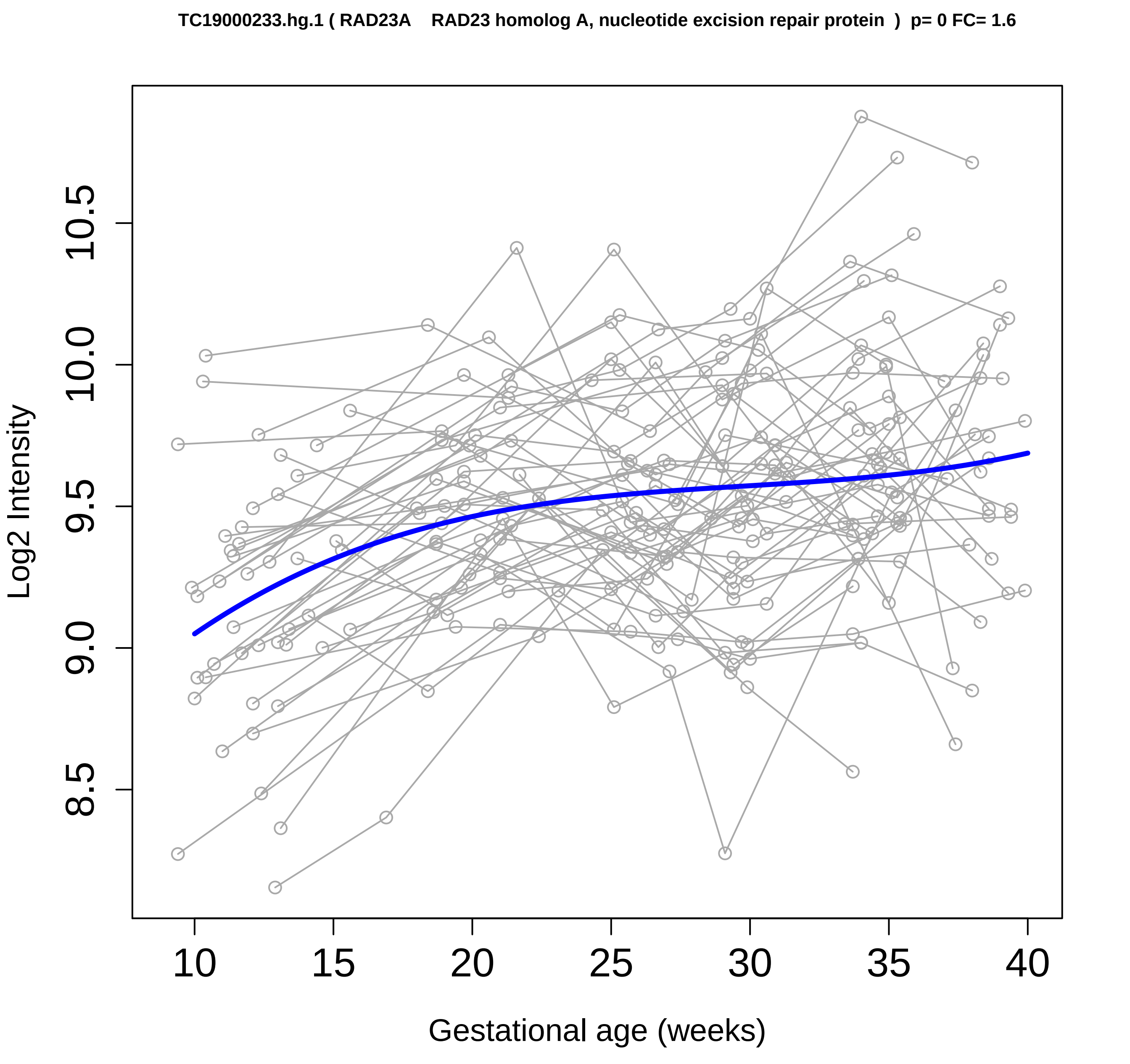

TC19000233.hg.1 (RAD23A RAD23 homolog A, nucleotide excision repair protein) p= 0 FC= 1.6
Log2 Intensity
8.5
9.0
9.5
10.0
10.5
10
15
20
25
30
35
40
Gestational age (weeks)

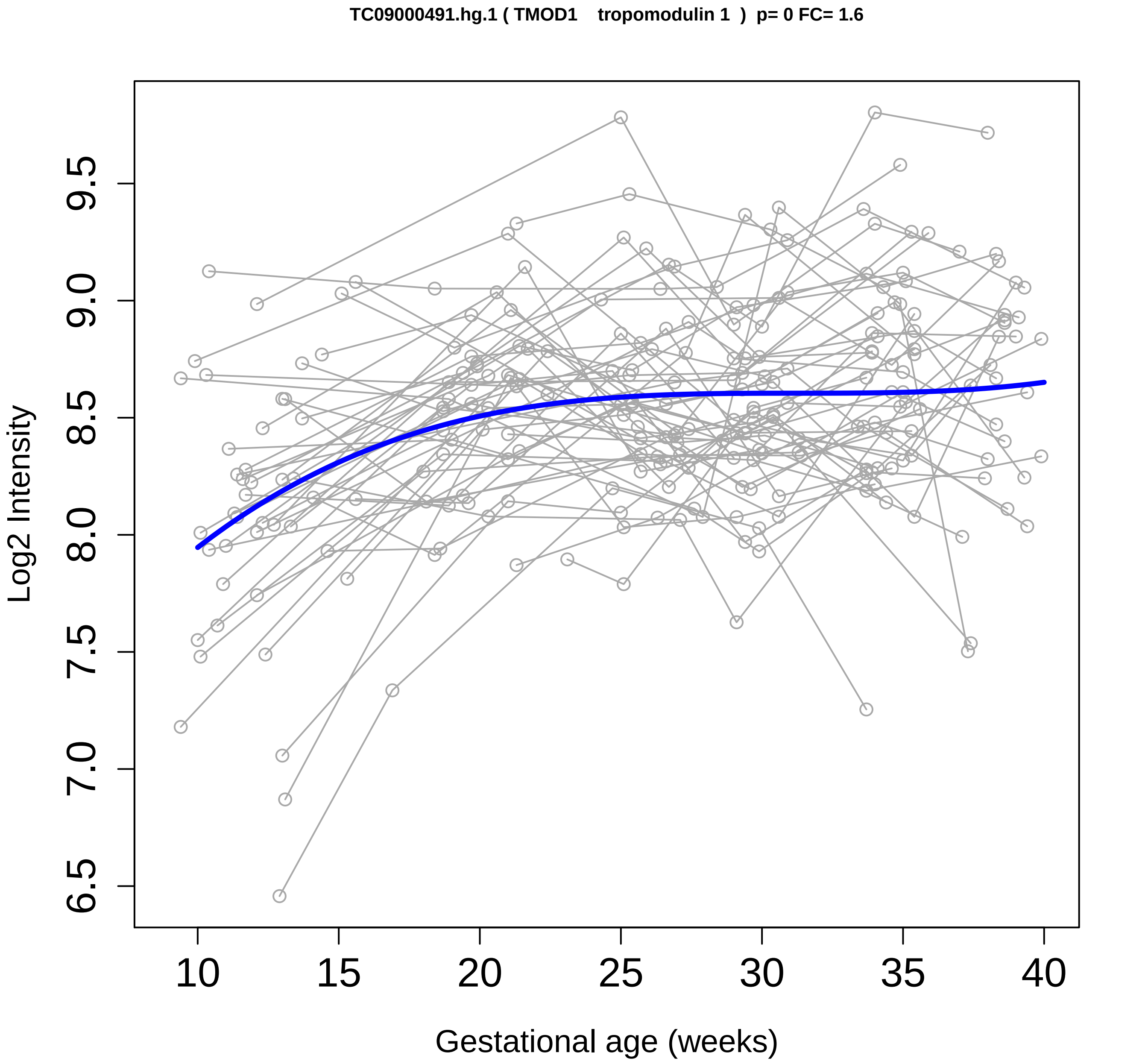

TC09000491.hg.1 (TMOD1 tropomodulin 1) p= 0 FC= 1.6
Log2 Intensity
6.5
7.0
7.5
8.0
8.5
9.0
9.5
10
15
20
25
30
35
40
Gestational age (weeks)

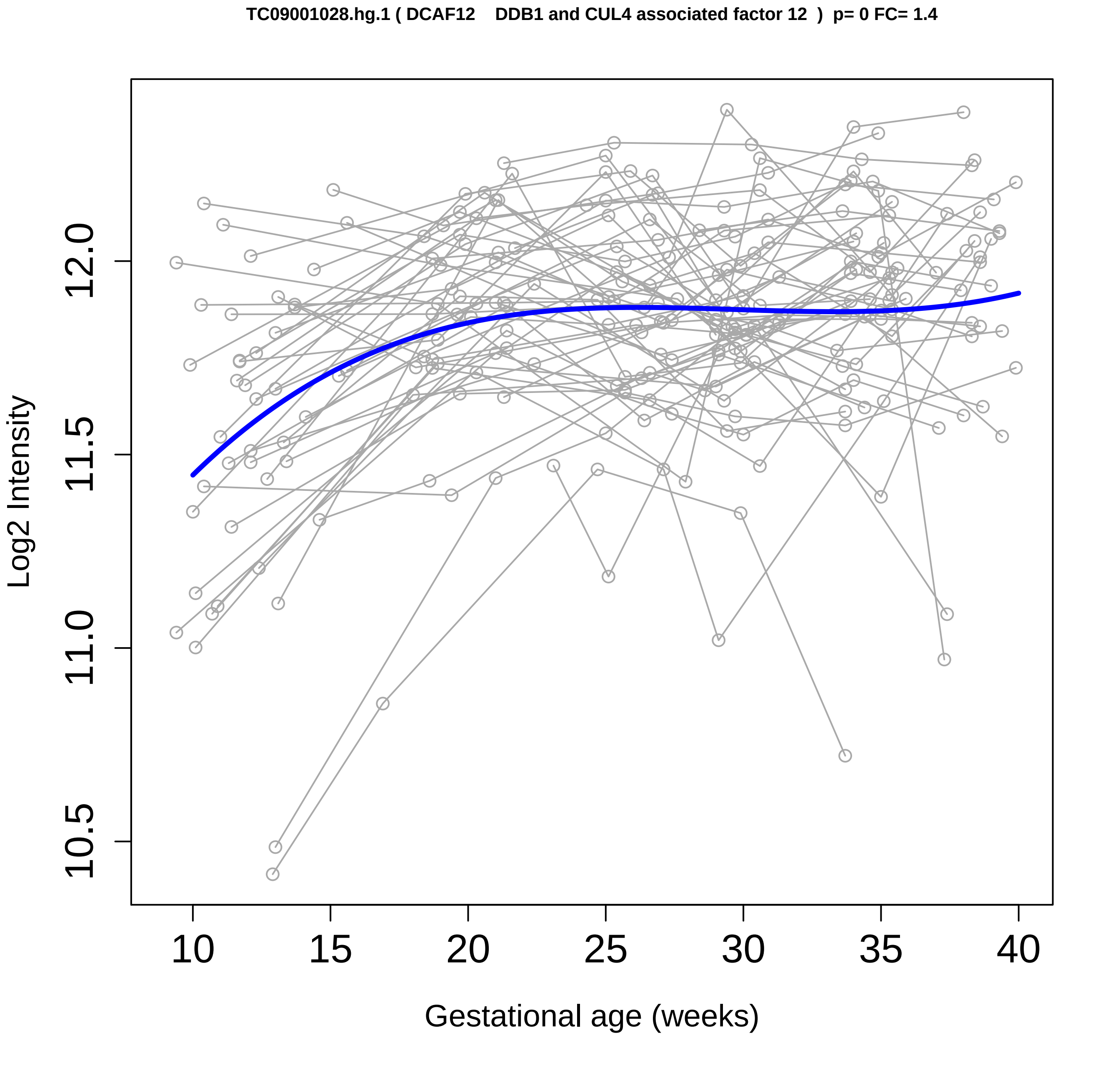

TC09001028.hg.1 (DCAF12 DDB1 and CUL4 associated factor 12) p= 0 FC= 1.4
Log2 Intensity
10.5
11.0
11.5
12.0
10
15
20
25
30
35
40
Gestational age (weeks)

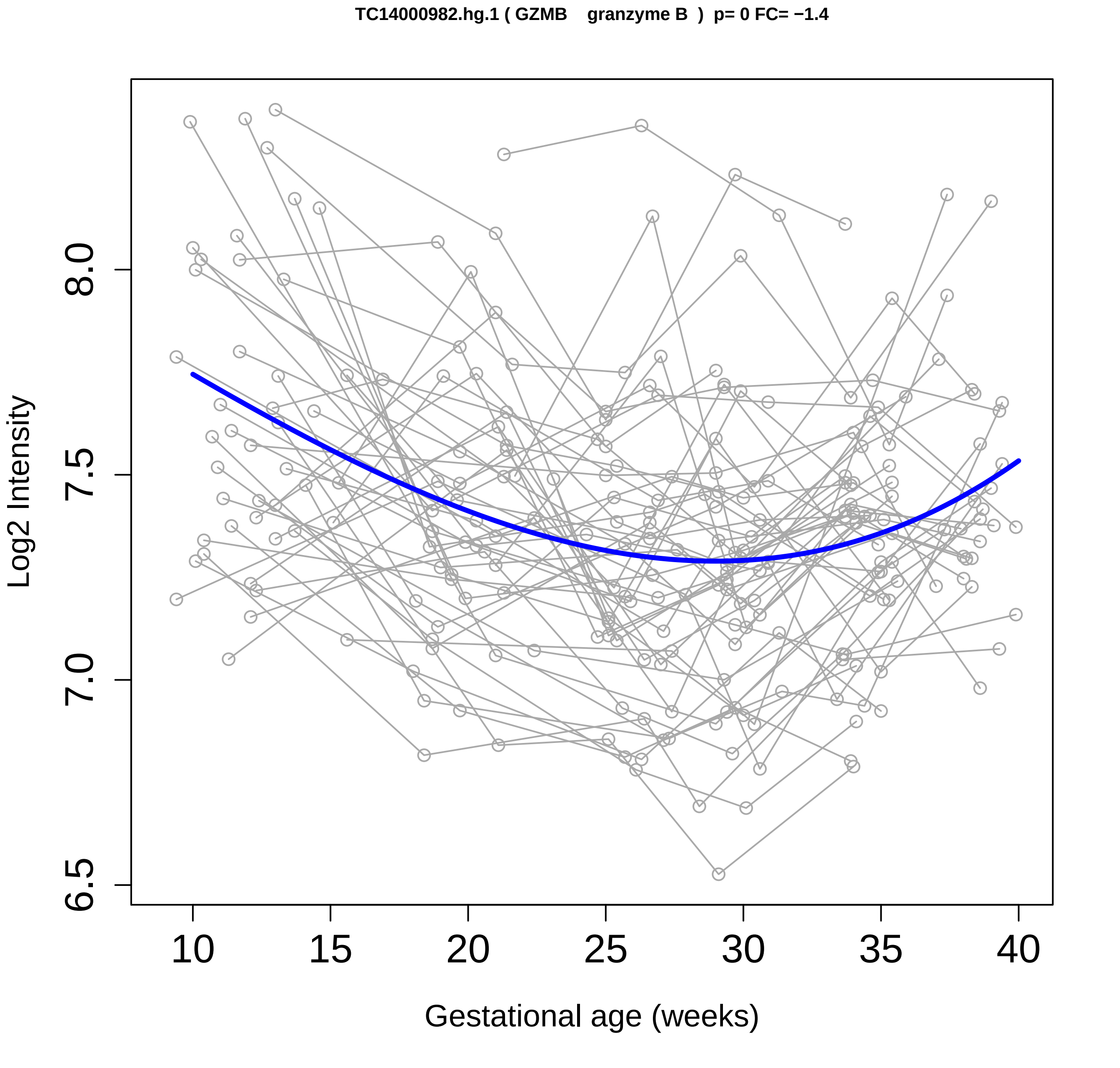

TC14000982.hg.1 (GZMB granzyme B) p= 0 FC= −1.4
Log2 Intensity
6.5
7.0
7.5
8.0
10
15
20
25
30
35
40
Gestational age (weeks)

TC07000246.hg.1 (TRG−AS1   T cell receptor gamma locus antisense RNA 1) p= 0 FC= −1.3

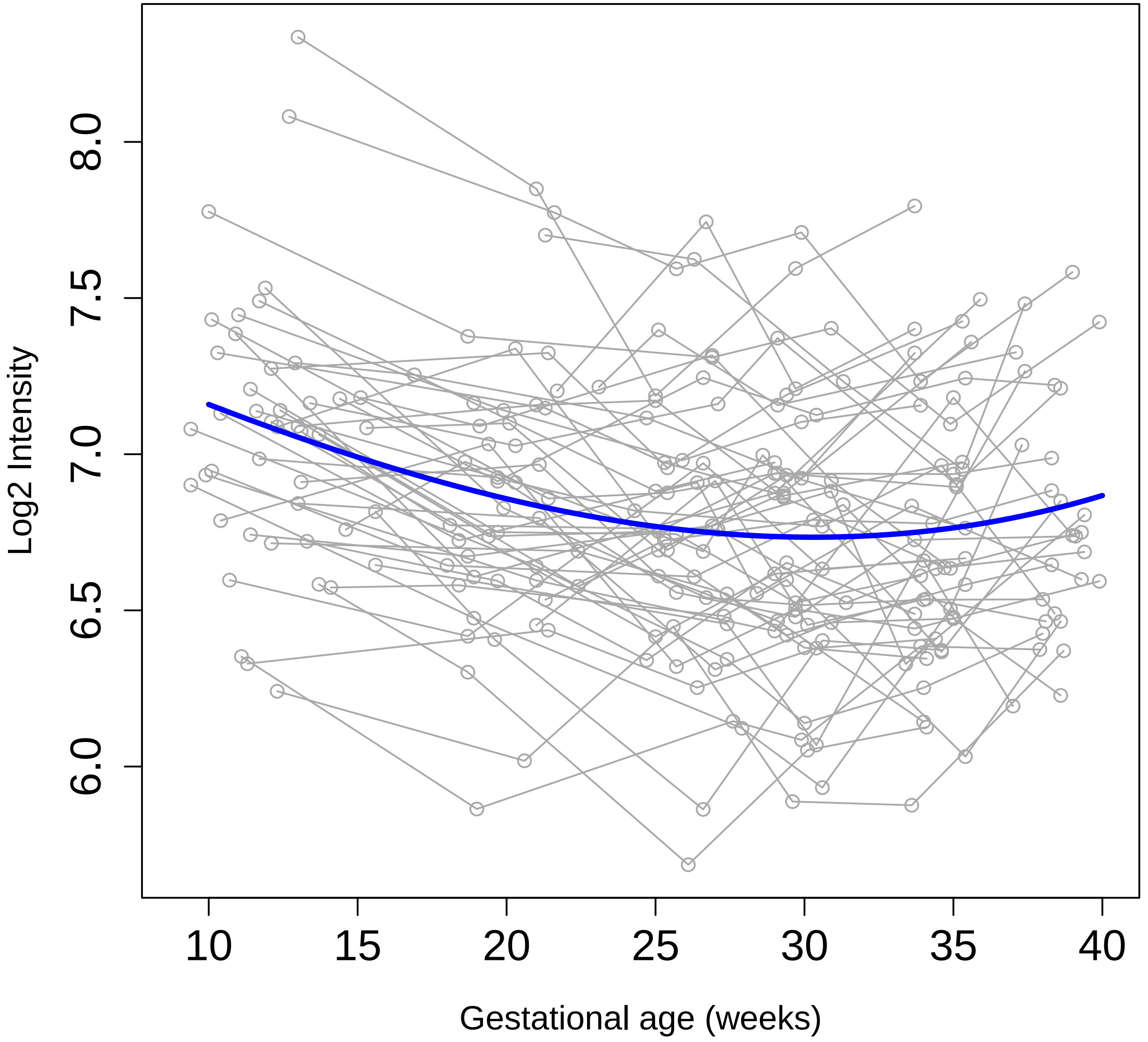

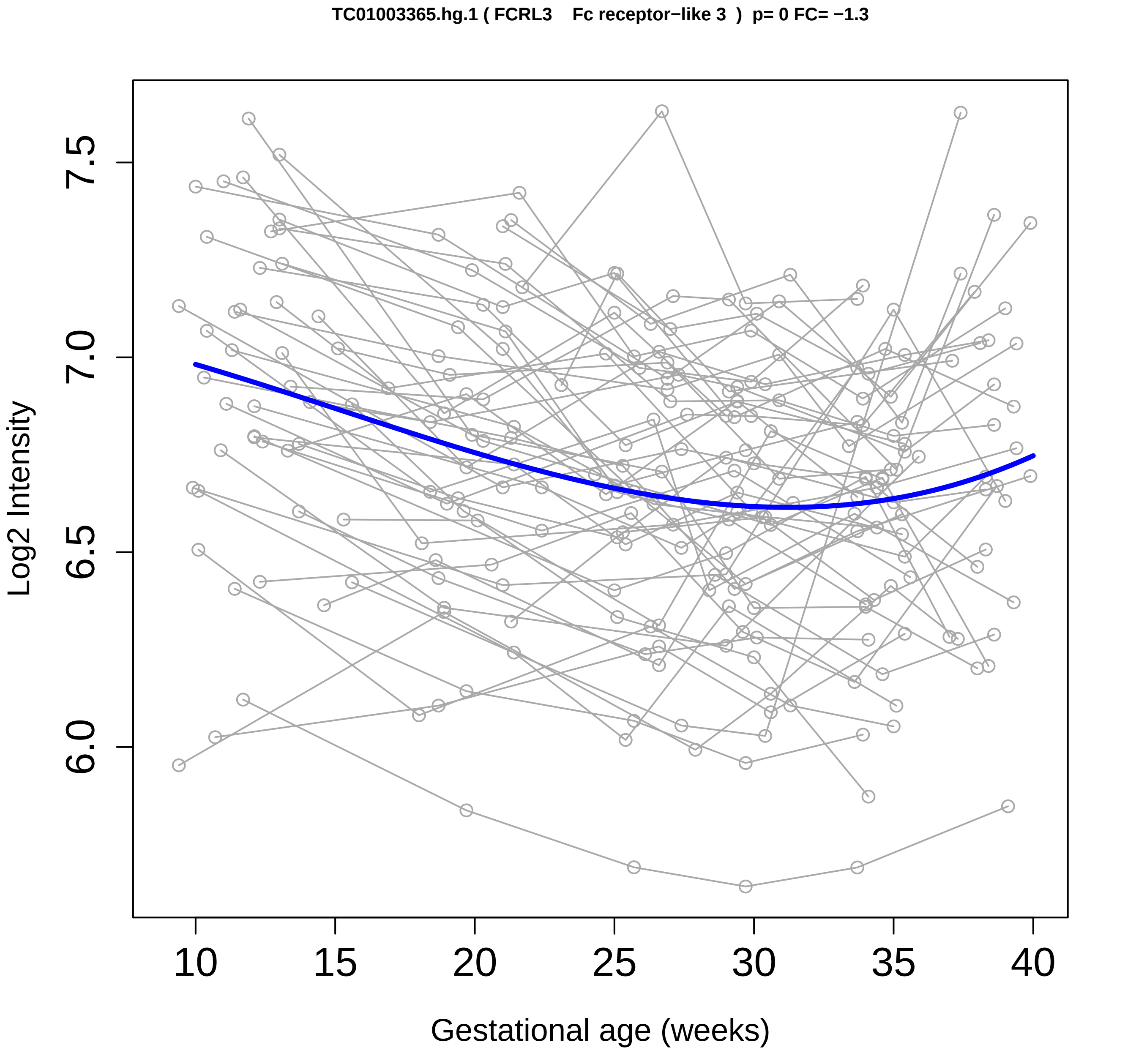
TC01003365.hg.1 (FCRL3   Fc receptor−like 3) p= 0 FC= −1.3
Log2 Intensity
6.0
6.5
7.0
7.5
10
15
20
25
30
35
40
Gestational age (weeks)

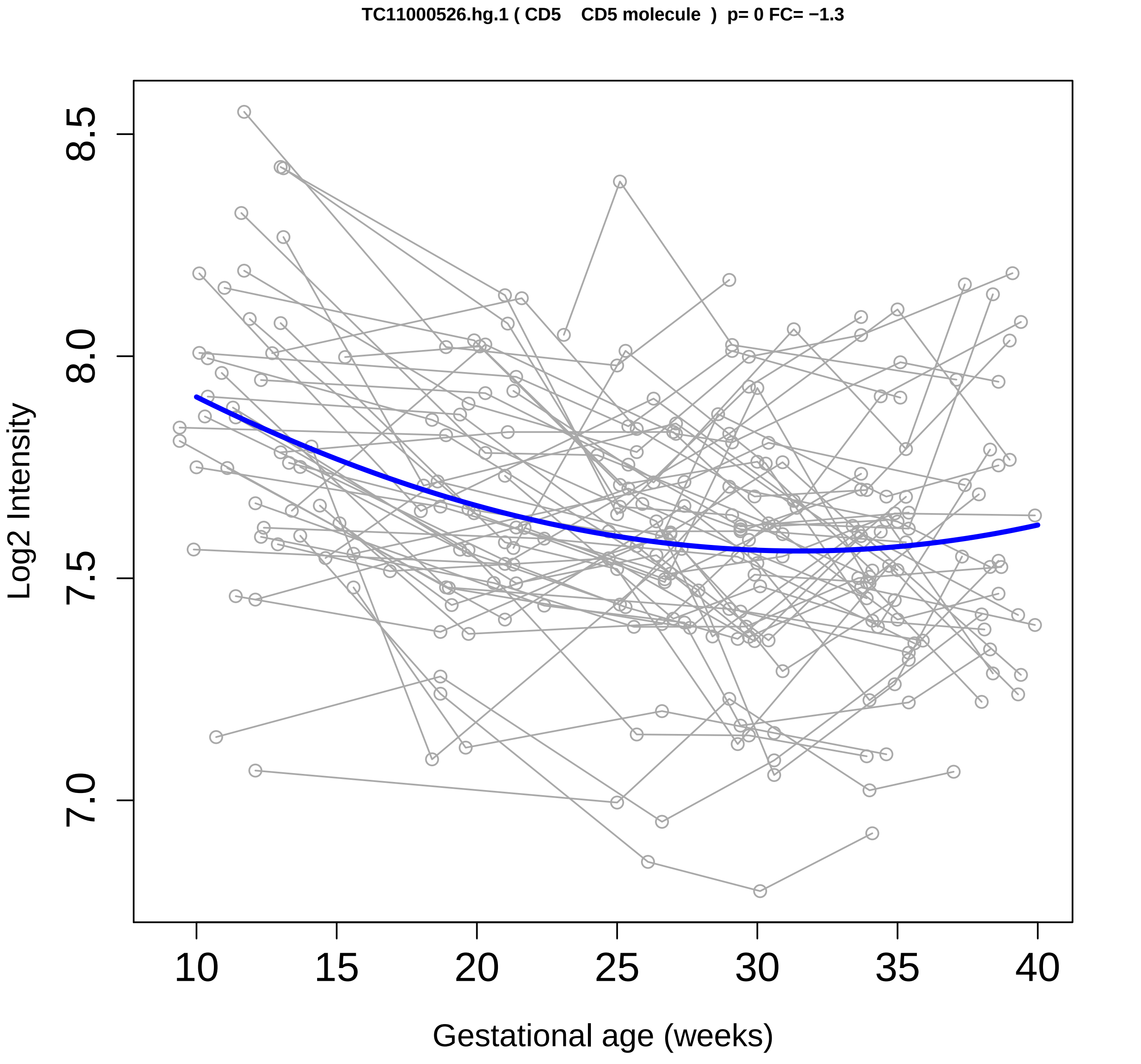

TC11000526.hg.1 (CD5 CD5 molecule) p= 0 FC= −1.3
Log2 Intensity
7.0
7.5
8.0
8.5
10
15
20
25
30
35
40
Gestational age (weeks)

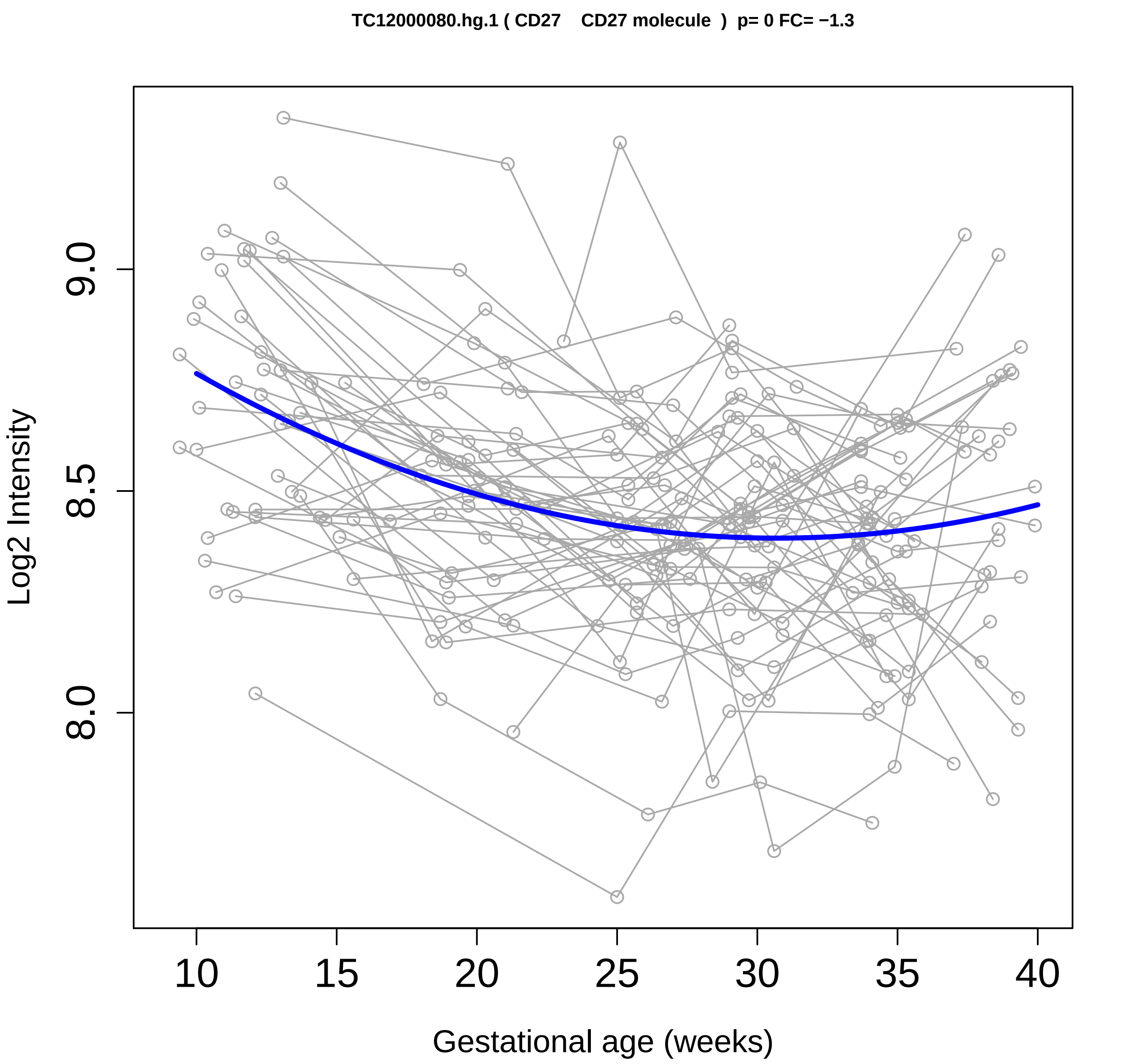
TC12000080.hg.1 (CD27 CD27 molecule) p= 0 FC= −1.3
Log2 Intensity
8.0
8.5
9.0
10
15
20
25
30
35
40
Gestational age (weeks)

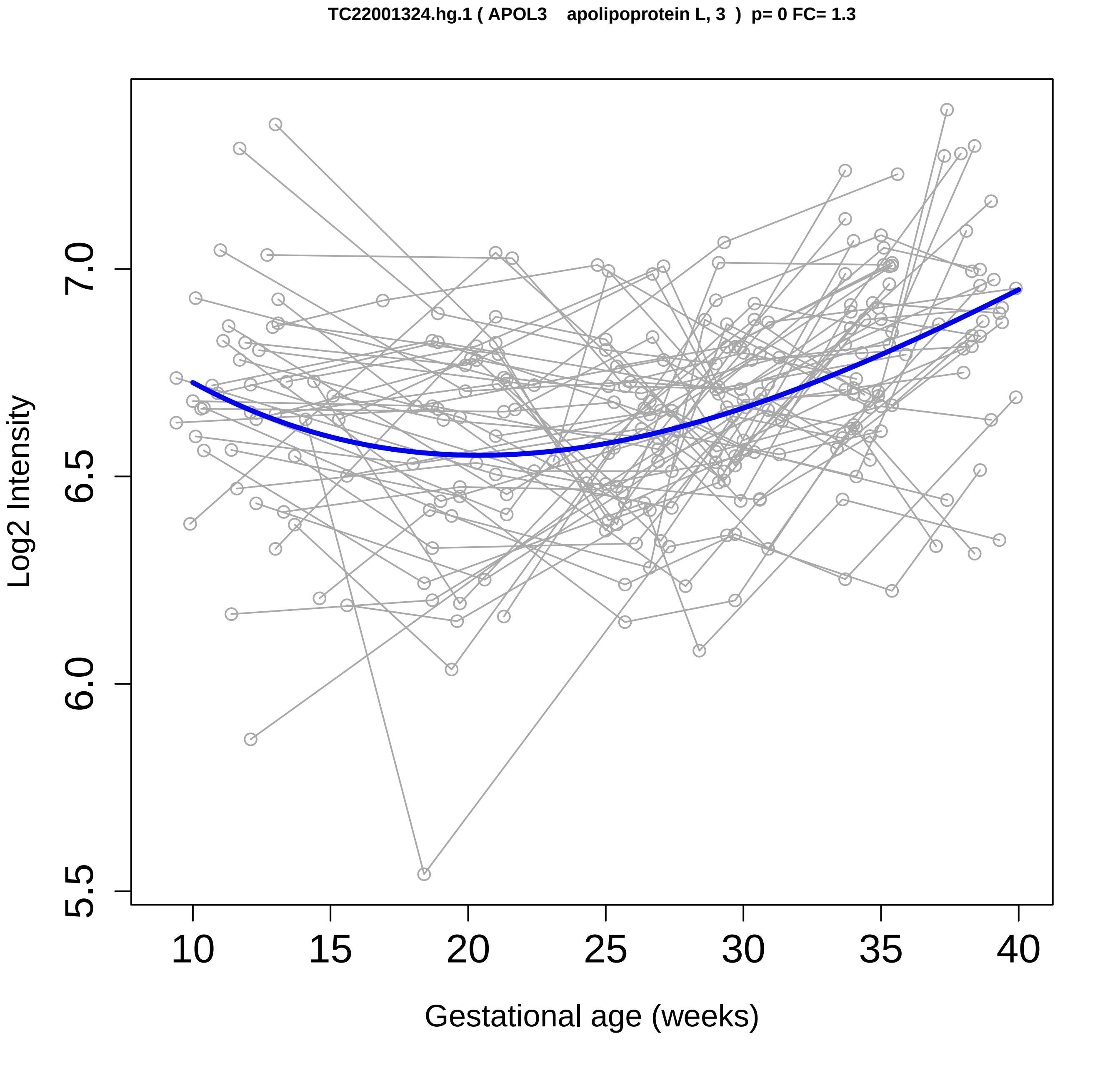

TC22001324.hg.1 (APOL3 apolipoprotein L, 3) p= 0 FC= 1.3
Log2 Intensity
5.5
6.0
6.5
7.0
10
15
20
25
30
35
40
Gestational age (weeks)

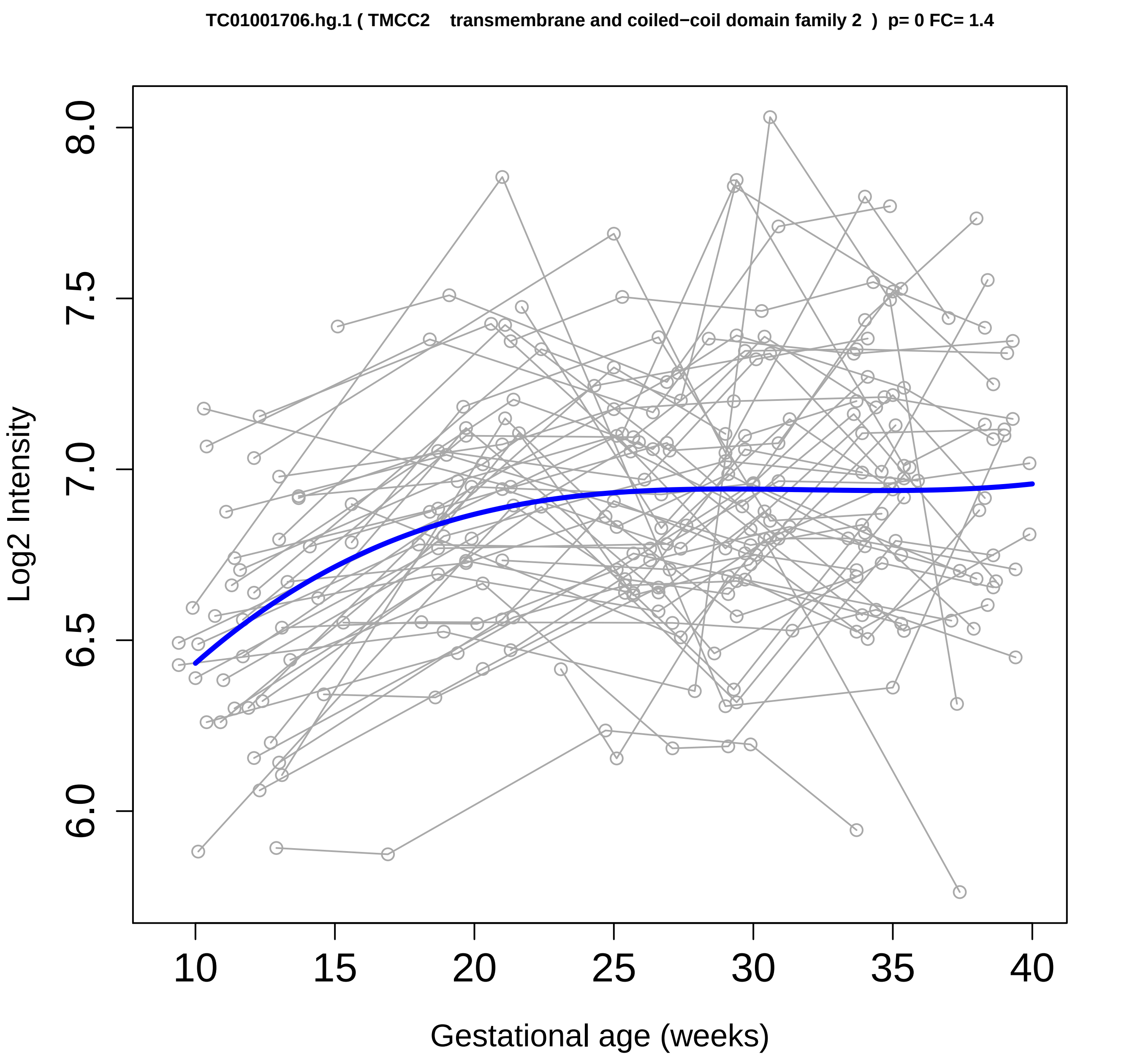
TC01001706.hg.1 (TMCC2   transmembrane and coiled−coil domain family 2) p= 0 FC= 1.4
Log2 Intensity
8.0
7.5
7.0
6.5
6.0
10
15
20
25
30
35
40
Gestational age (weeks)

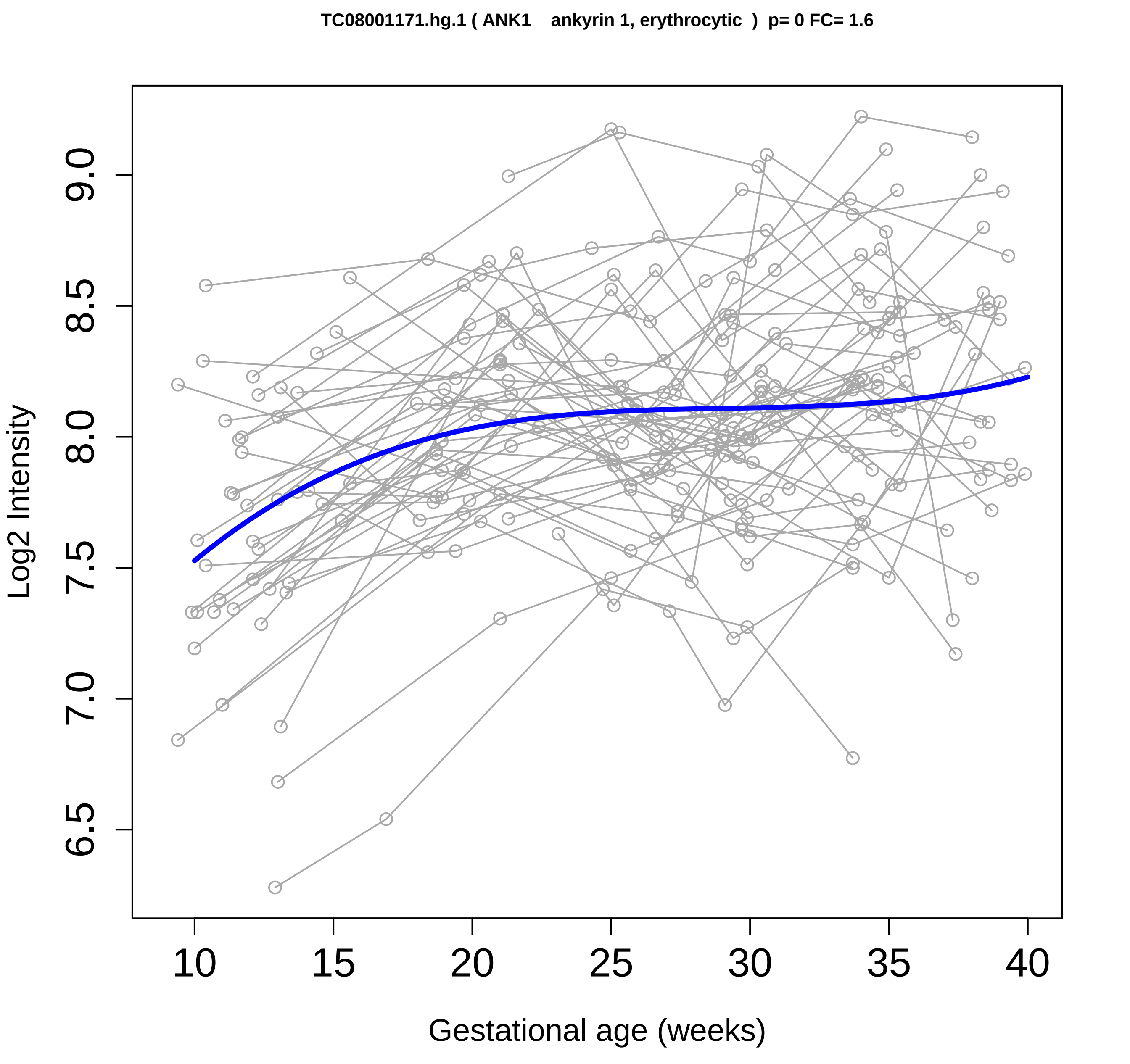

TC08001171.hg.1 (ANK1 ankyrin 1, erythrocytic) p= 0 FC= 1.6
Log2 Intensity
9.0
8.5
8.0
7.5
7.0
6.5
10
15
20
25
30
35
40
Gestational age (weeks)

TC08001248.hg.1 (TOX   thymocyte selection−associated high mobility group box) p= 0 FC= −1.3

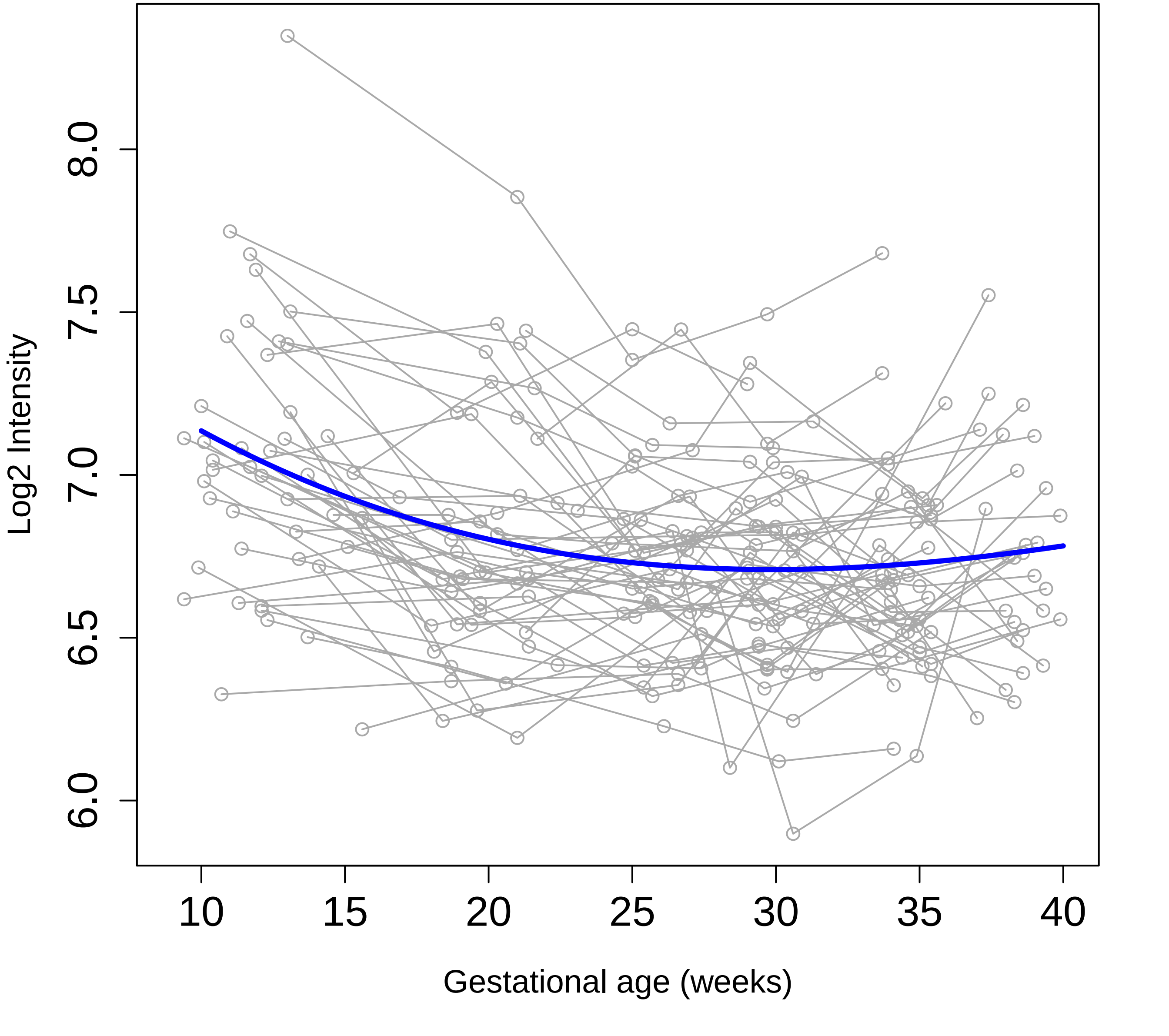

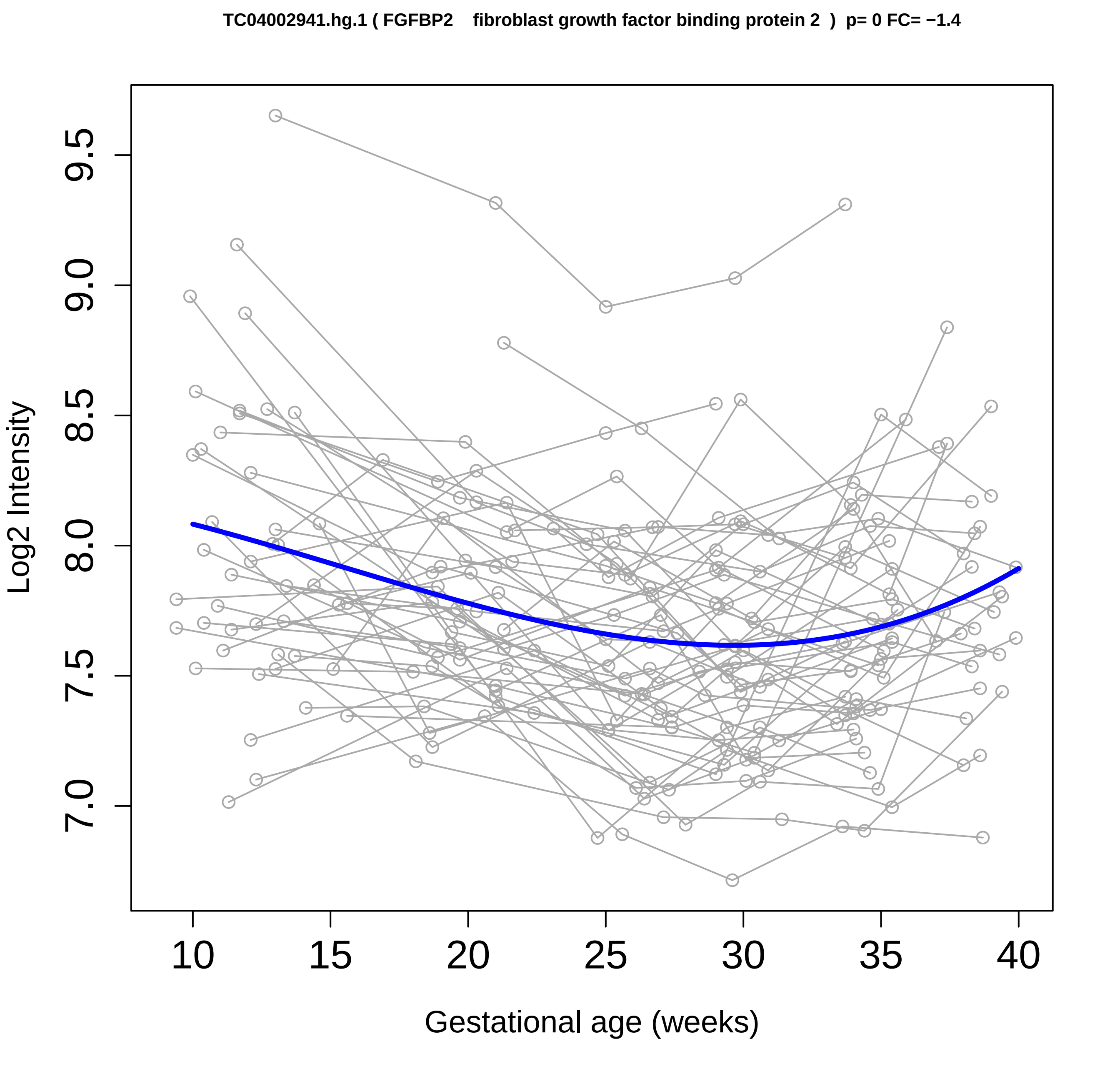
TC04002941.hg.1 (FGFBP2 fibroblast growth factor binding protein 2) p= 0 FC= −1.4
Log2 Intensity
9.5
9.0
8.5
8.0
7.5
7.0
10
15
20
25
30
35
40
Gestational age (weeks)

TC14001588.hg.1 (YME1L1 YME1−like 1 ATPase) p= 0 FC= −1.3

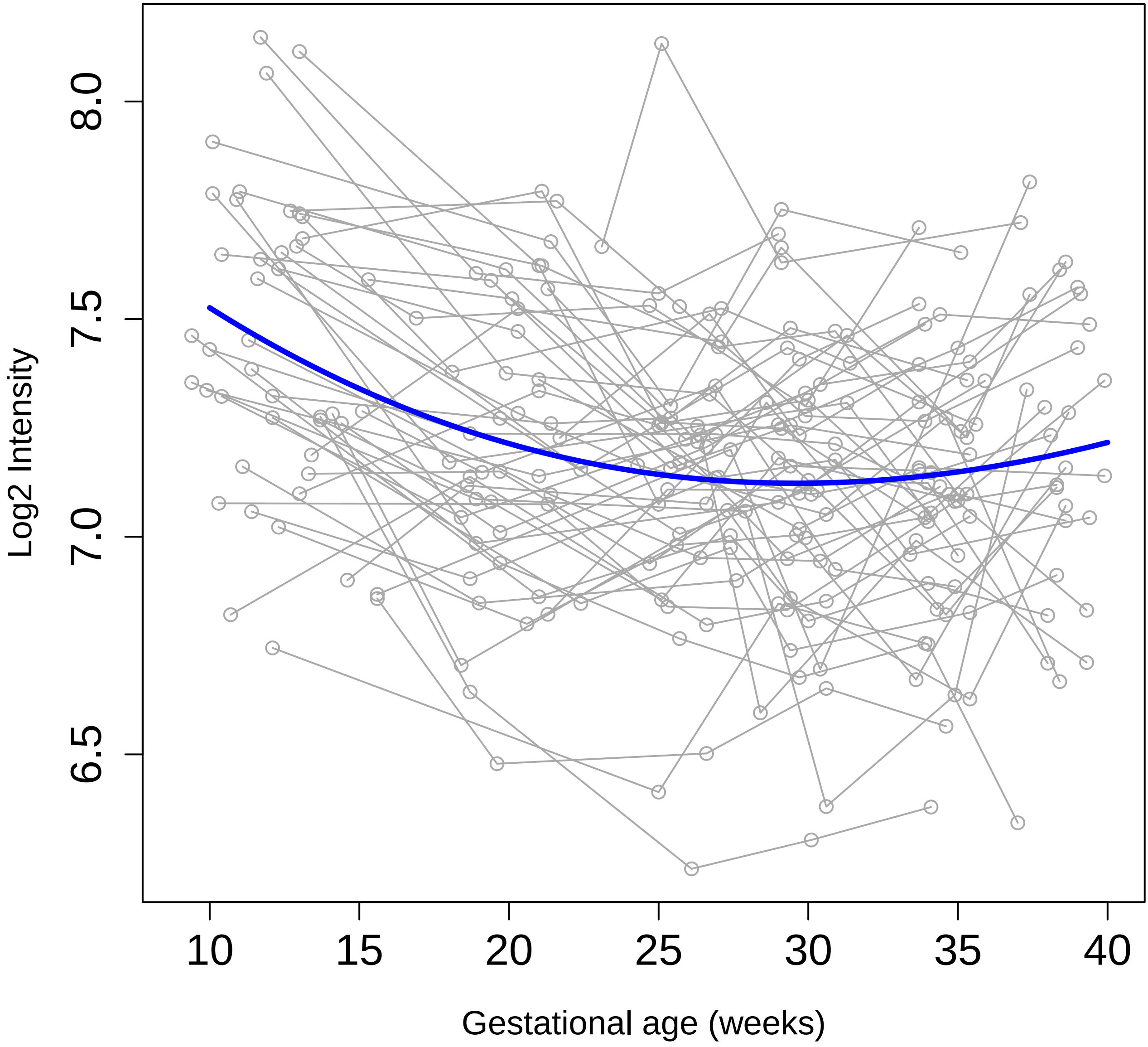

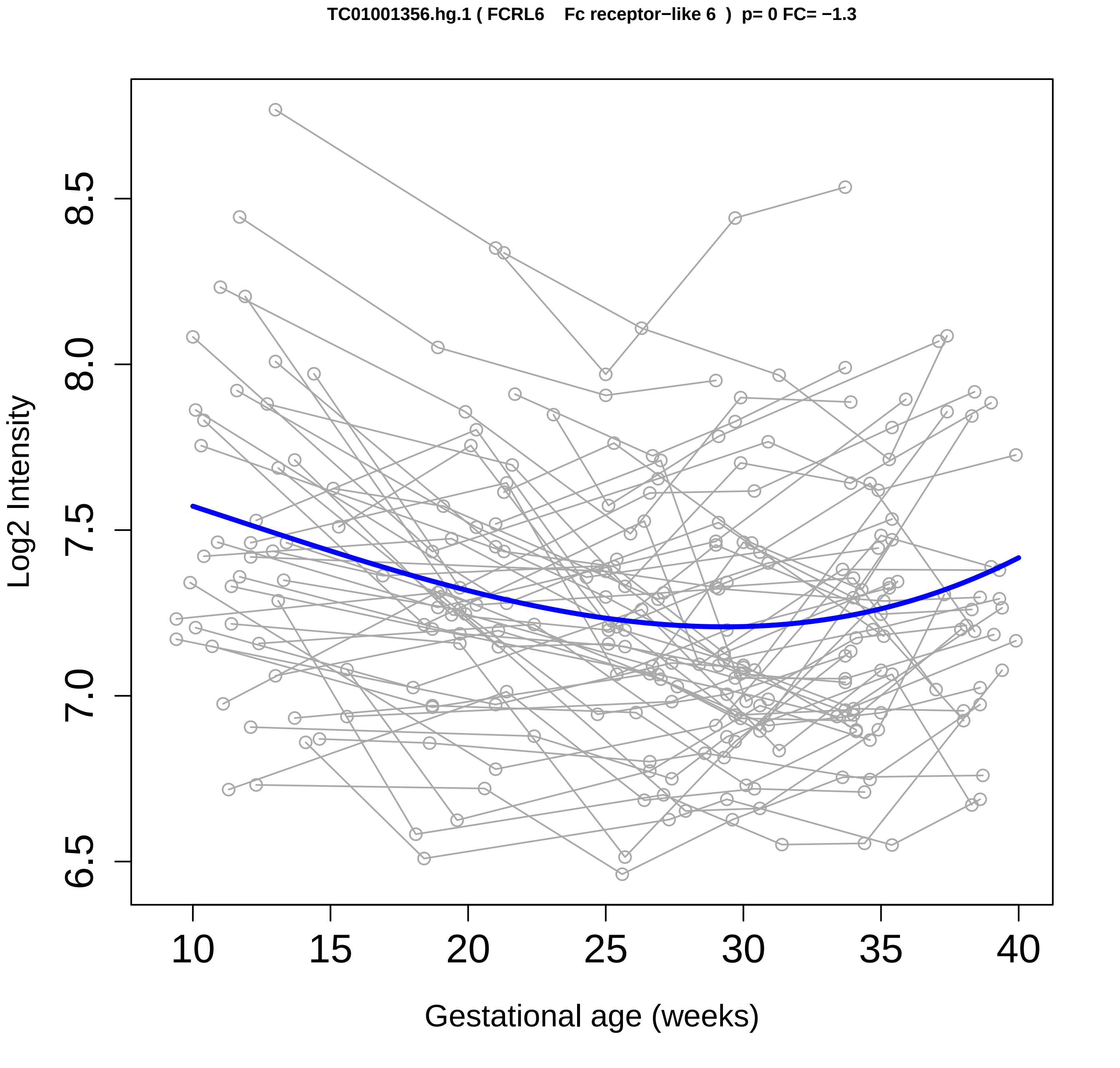
TC01001356.hg.1 (FCRL6 Fc receptor−like 6) p= 0 FC= −1.3
Log2 Intensity
6.5
7.0
7.5
8.0
8.5
10
15
20
25
30
35
40
Gestational age (weeks)

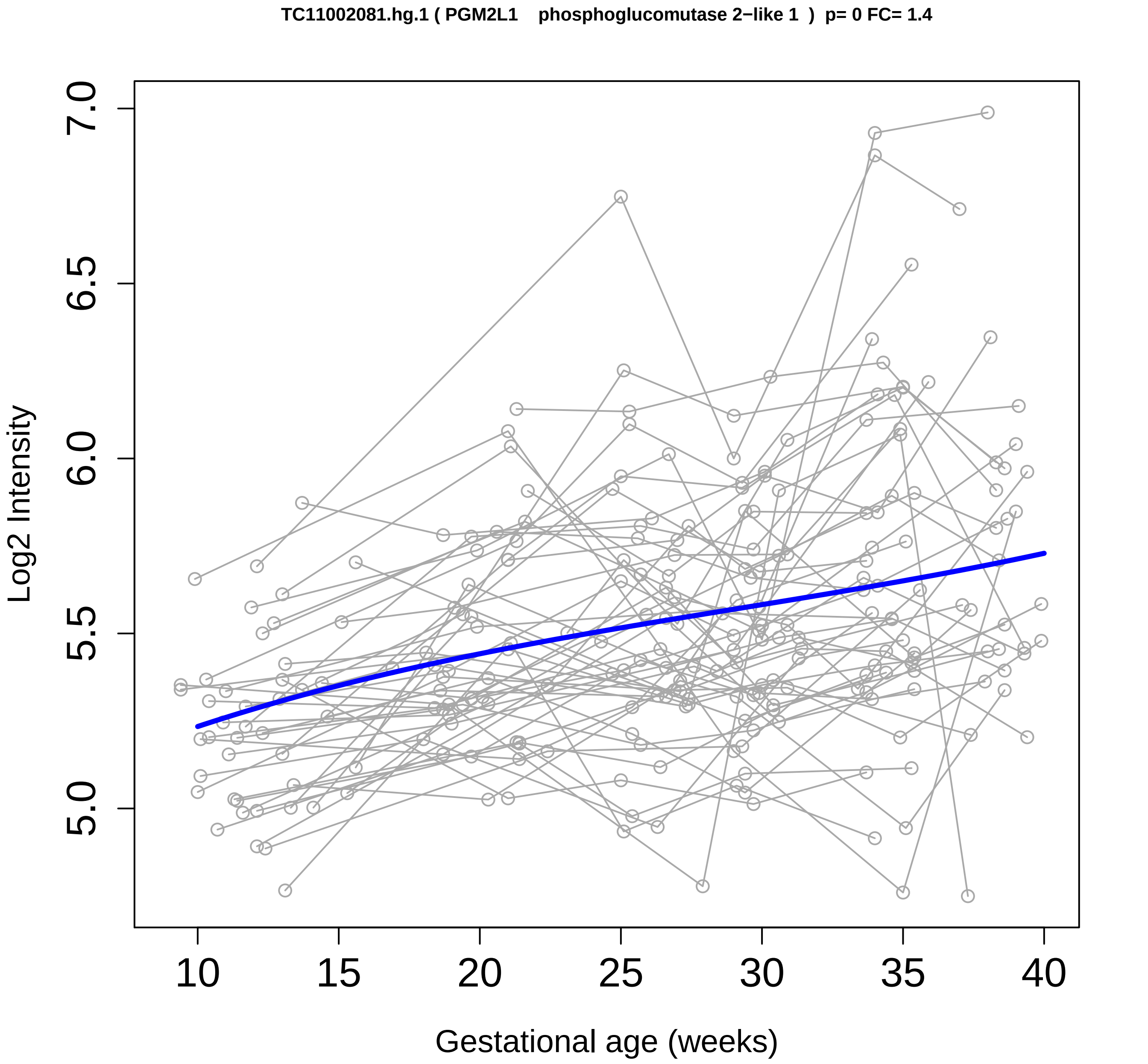
TC11002081.hg.1 (PGM2L1 phosphoglucomutase 2−like 1) p= 0 FC= 1.4
Log2 Intensity
5.0
5.5
6.0
6.5
7.0
10
15
20
25
30
35
40
Gestational age (weeks)

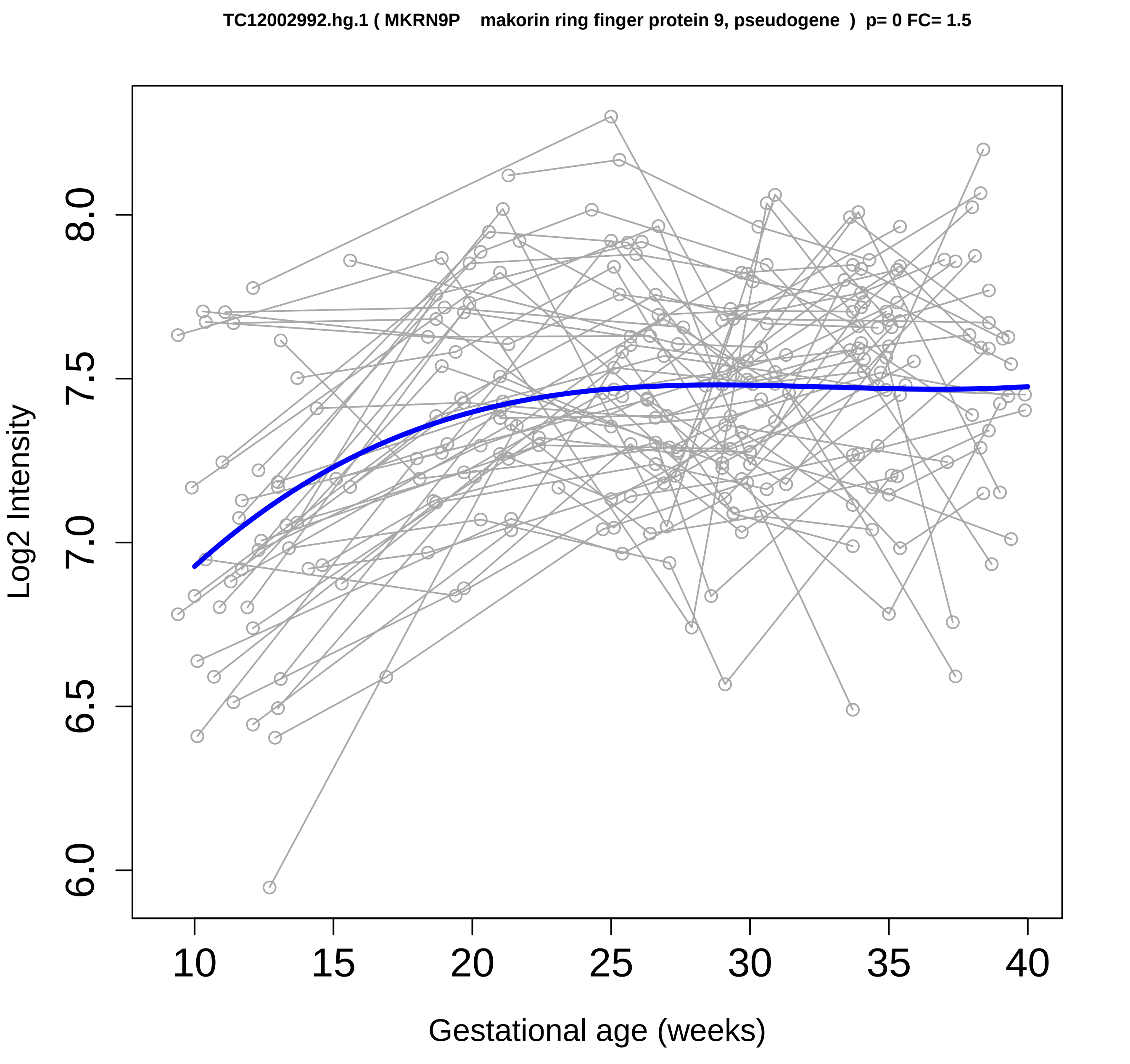
TC12002992.hg.1 (MKRN9P makorin ring finger protein 9, pseudogene) p= 0 FC= 1.5
Log2 Intensity
6.0
6.5
7.0
7.5
8.0
10
15
20
25
30
35
40
Gestational age (weeks)

TC07002778.hg.1 (EIF2AK1 eukaryotic translation initiation factor 2−alpha kinase 1) p= 0 FC= 1.6

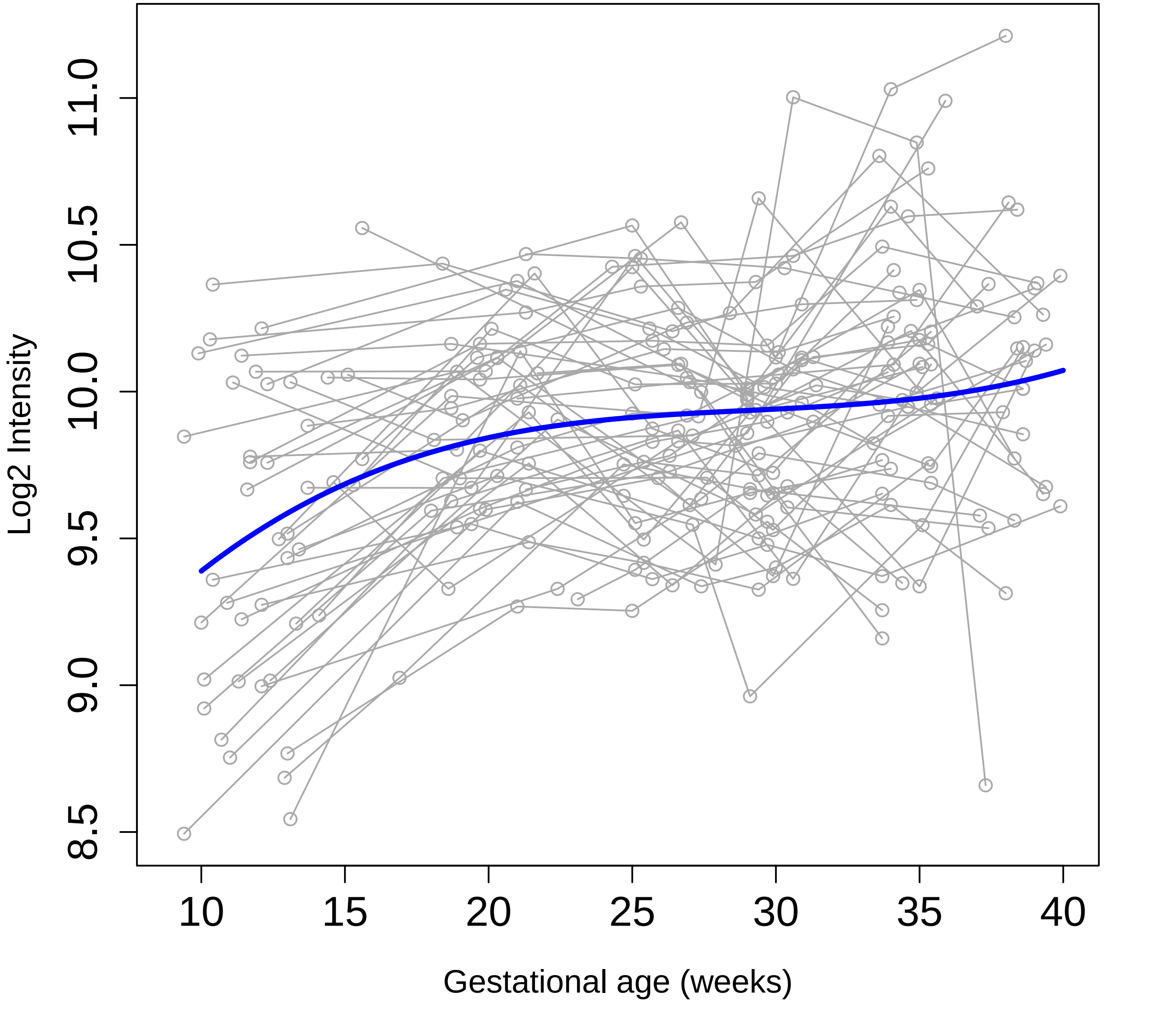

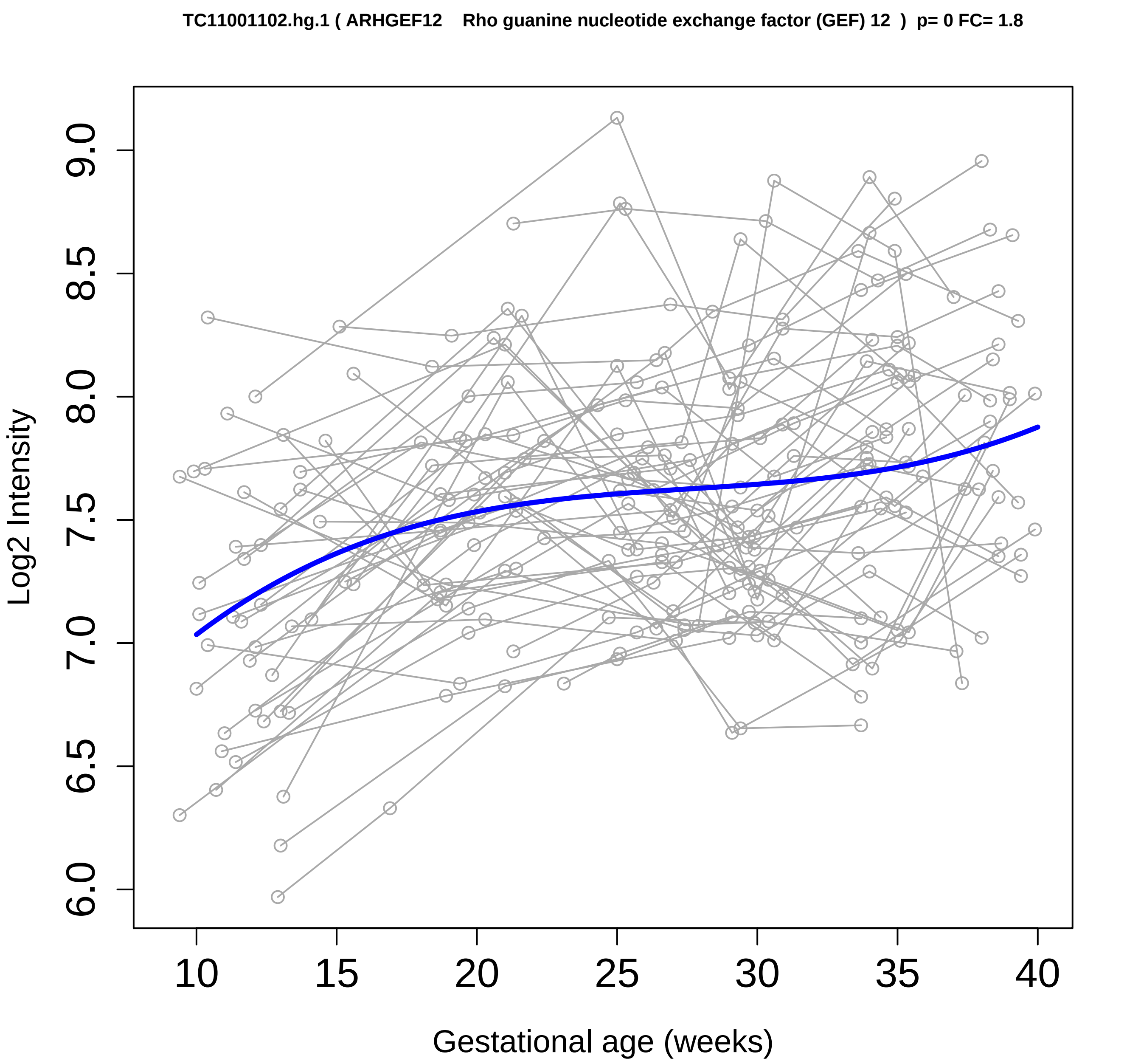

TC11001102.hg.1 (ARHGEF12 Rho guanine nucleotide exchange factor (GEF) 12) p= 0 FC= 1.8
Log2 Intensity
6.0
6.5
7.0
7.5
8.0
8.5
9.0
10
15
20
25
30
35
40
Gestational age (weeks)

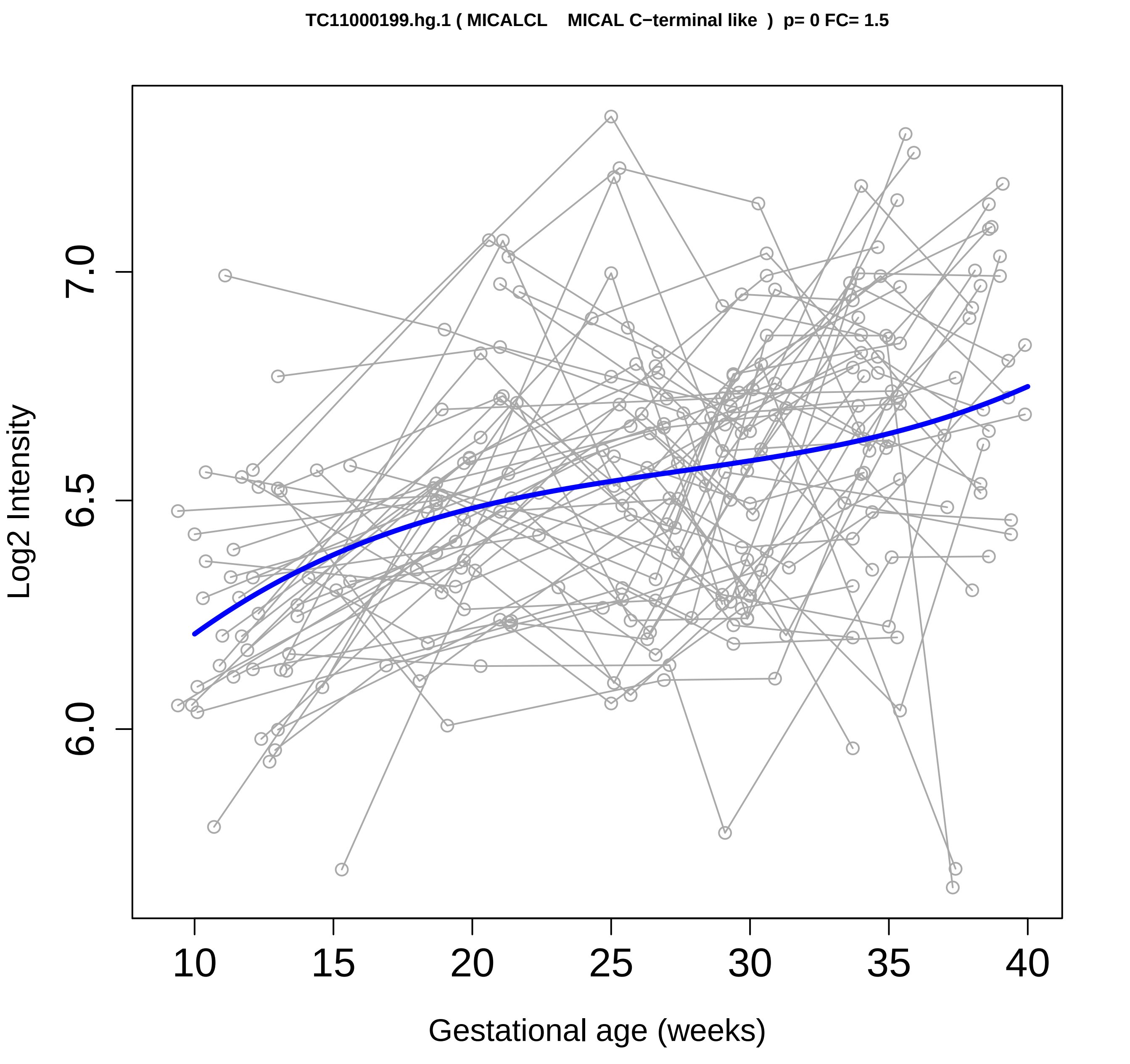

TC11000199.hg.1 (MICALCL MICAL C−terminal like) p= 0 FC= 1.5
Log2 Intensity
Gestational age (weeks)
6.0
6.5
7.0
10
15
20
25
30
35
40

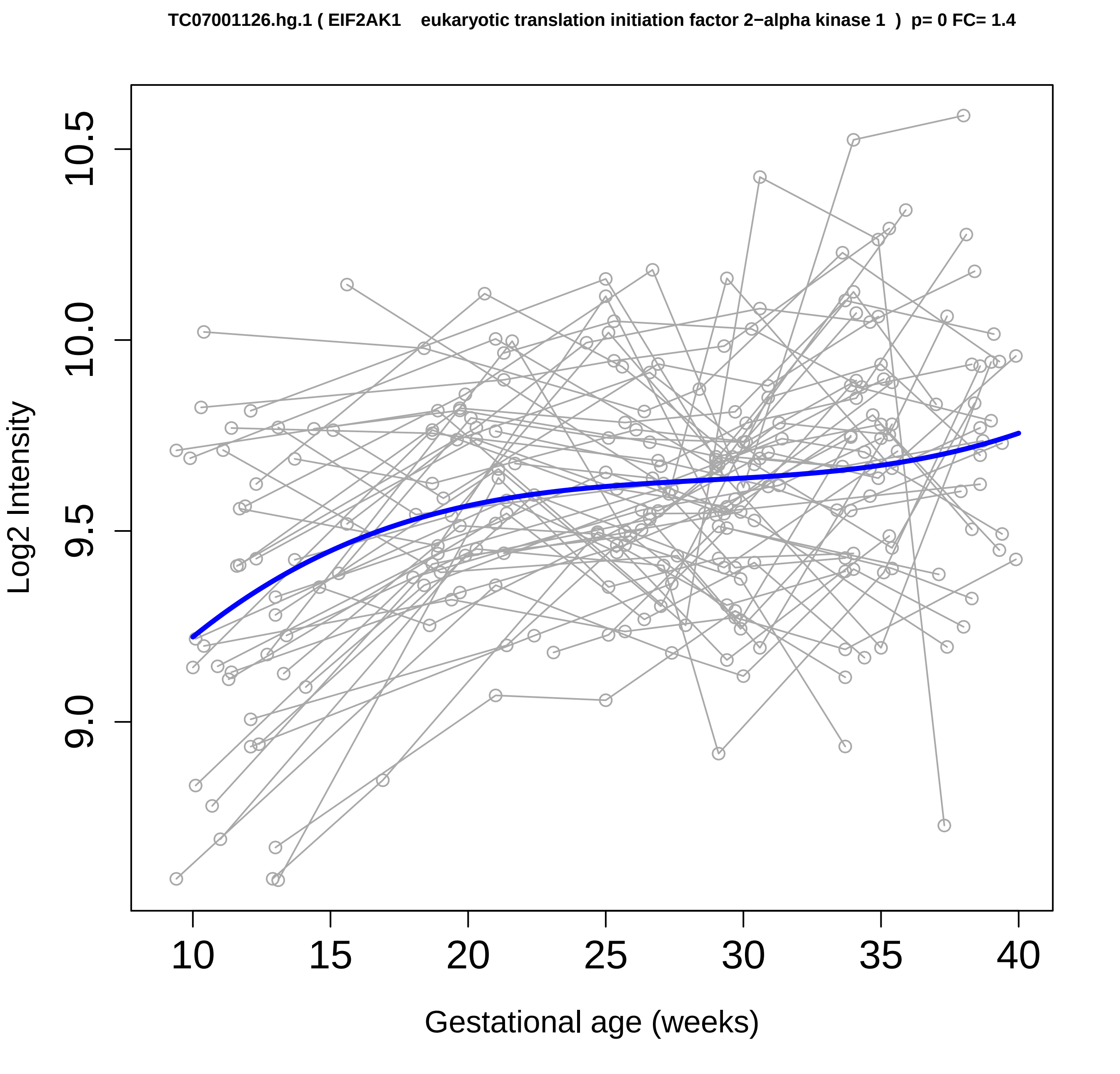

TC07001126.hg.1 (EIF2AK1 eukaryotic translation initiation factor 2−alpha kinase 1) p= 0 FC= 1.4
Log2 Intensity
10.5
10.0
9.5
9.0
10
15
20
25
30
35
40
Gestational age (weeks)

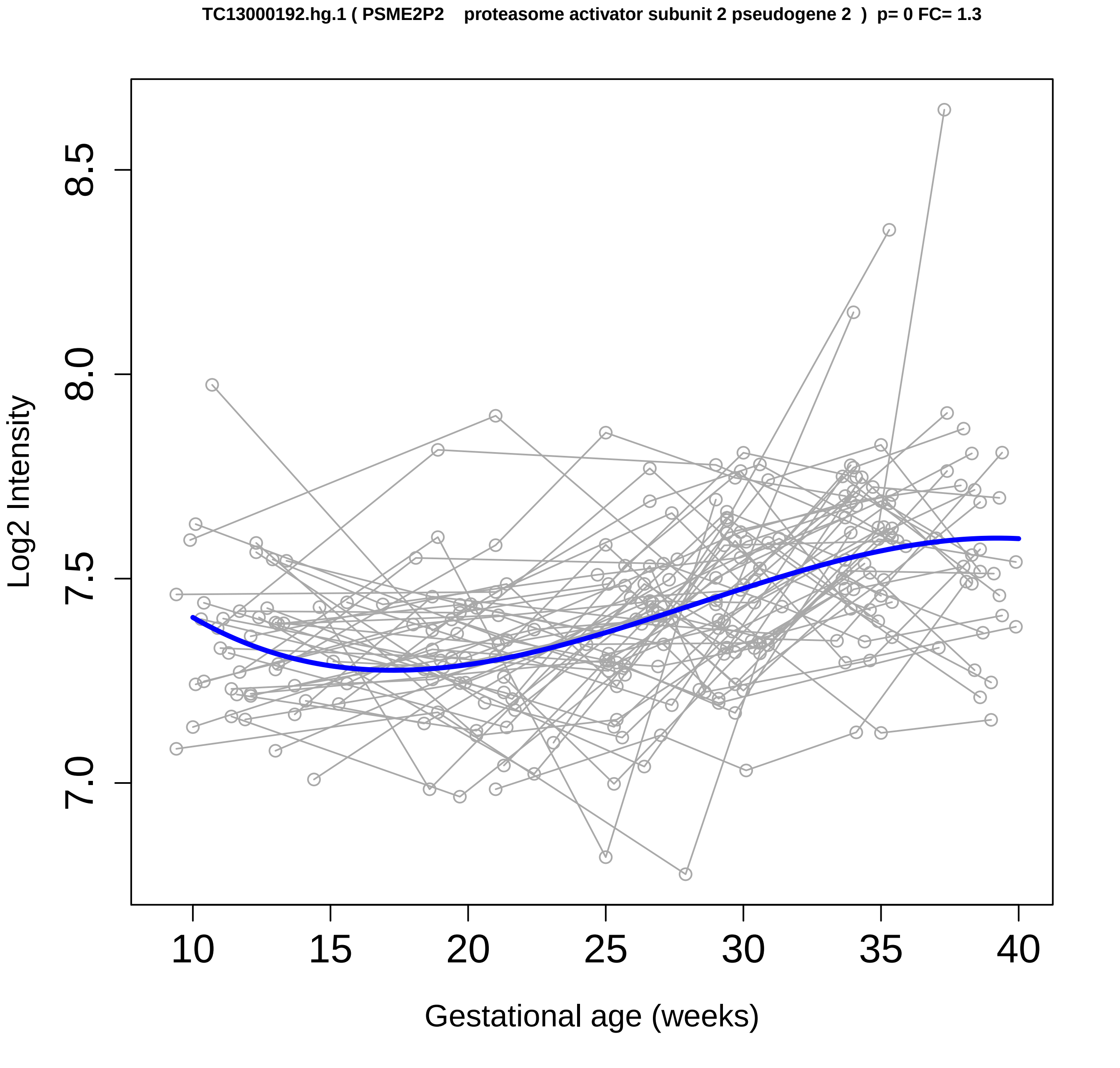

TC13000192.hg.1 (PSME2P2 proteasome activator subunit 2 pseudogene 2) p= 0 FC= 1.3
Log2 Intensity
7.0
7.5
8.0
8.5
10
15
20
25
30
35
40
Gestational age (weeks)

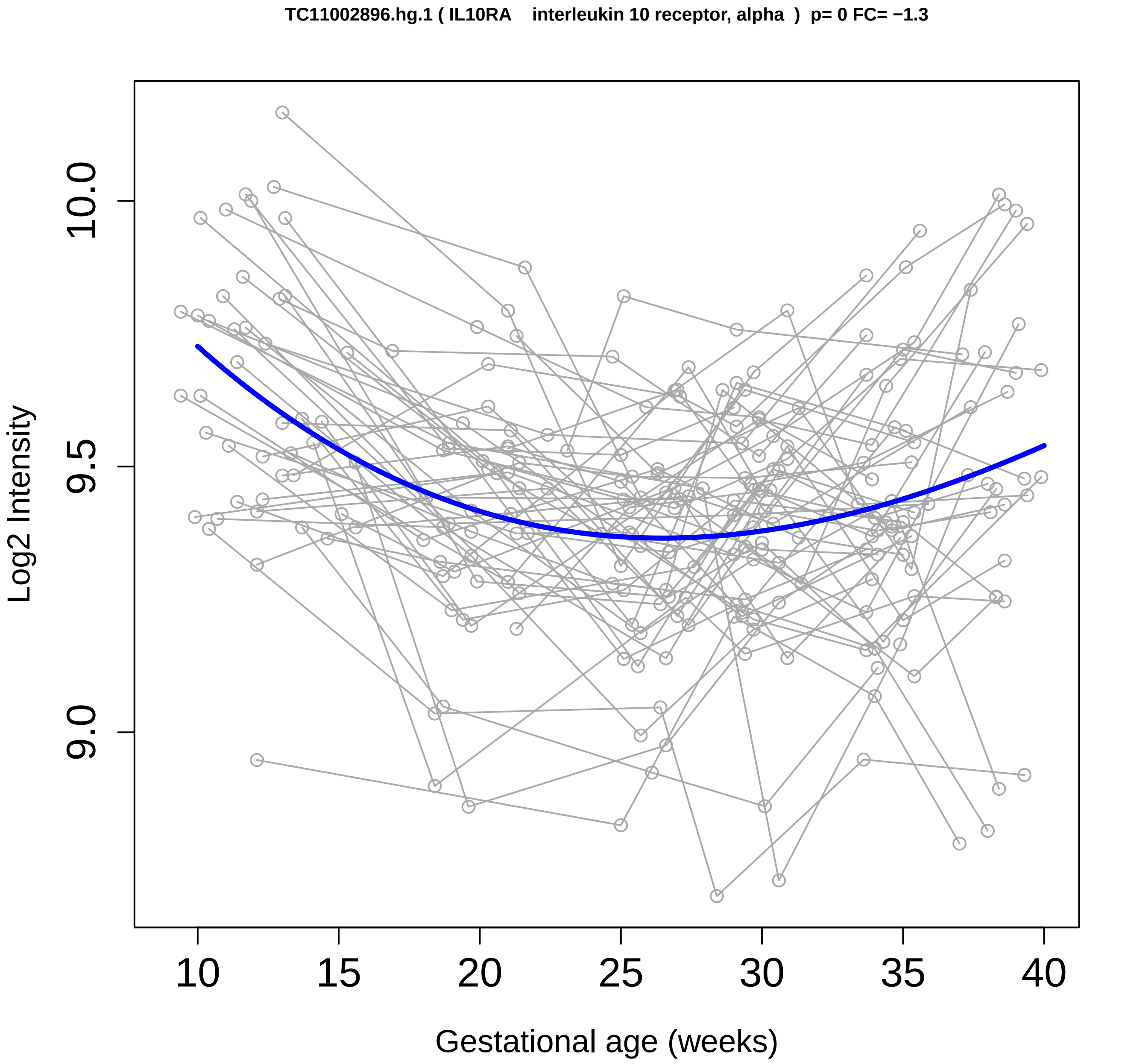
TC11002896.hg.1 (IL10RA interleukin 10 receptor, alpha) p= 0 FC= −1.3
Log2 Intensity
9.0
9.5
10.0
10
15
20
25
30
35
40
Gestational age (weeks)

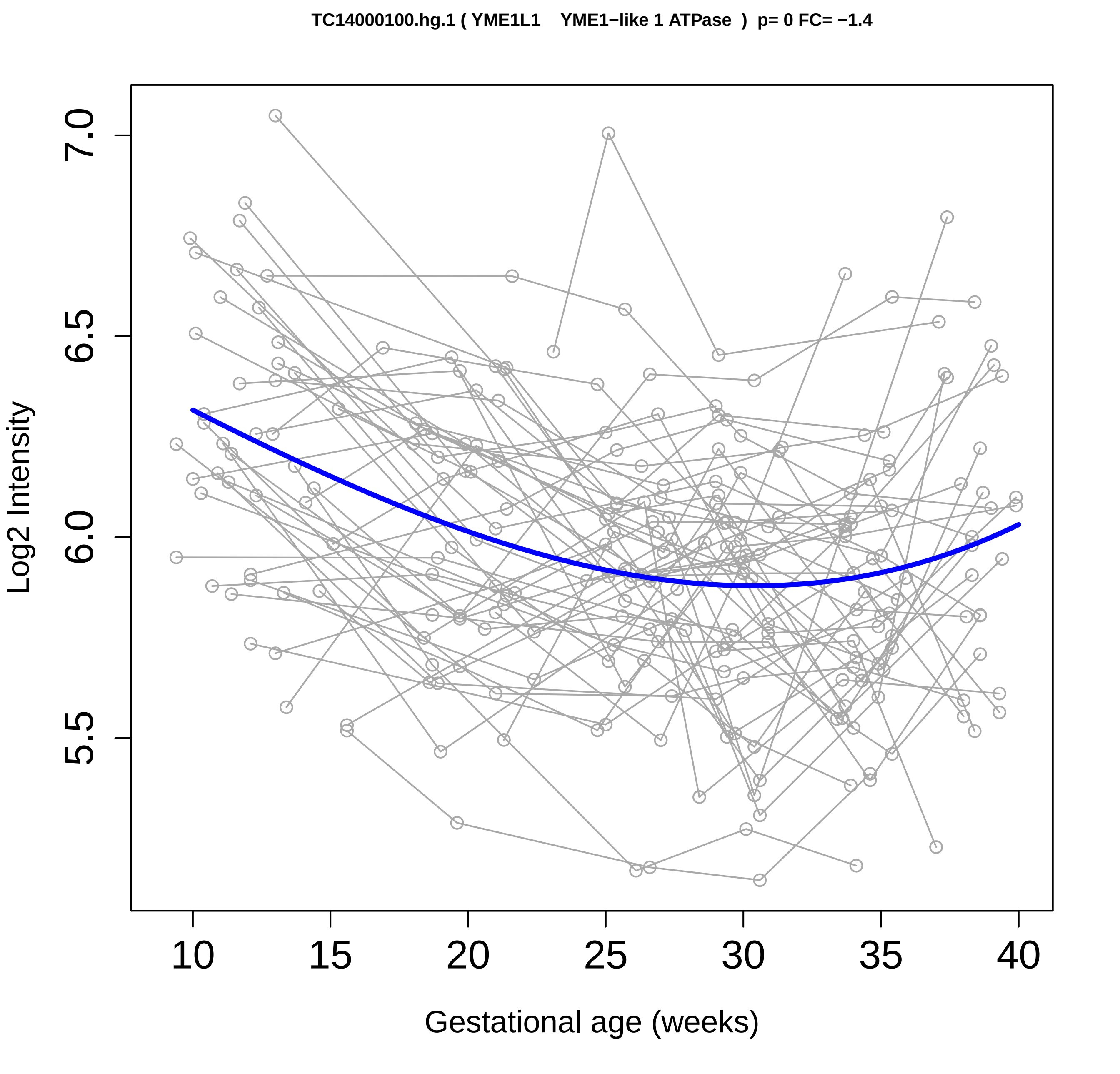

TC14000100.hg.1 (YME1L1 YME1−like 1 ATPase) p= 0 FC= −1.4
Log2 Intensity
7.0
6.5
6.0
5.5
10
15
20
25
30
35
40
Gestational age (weeks)

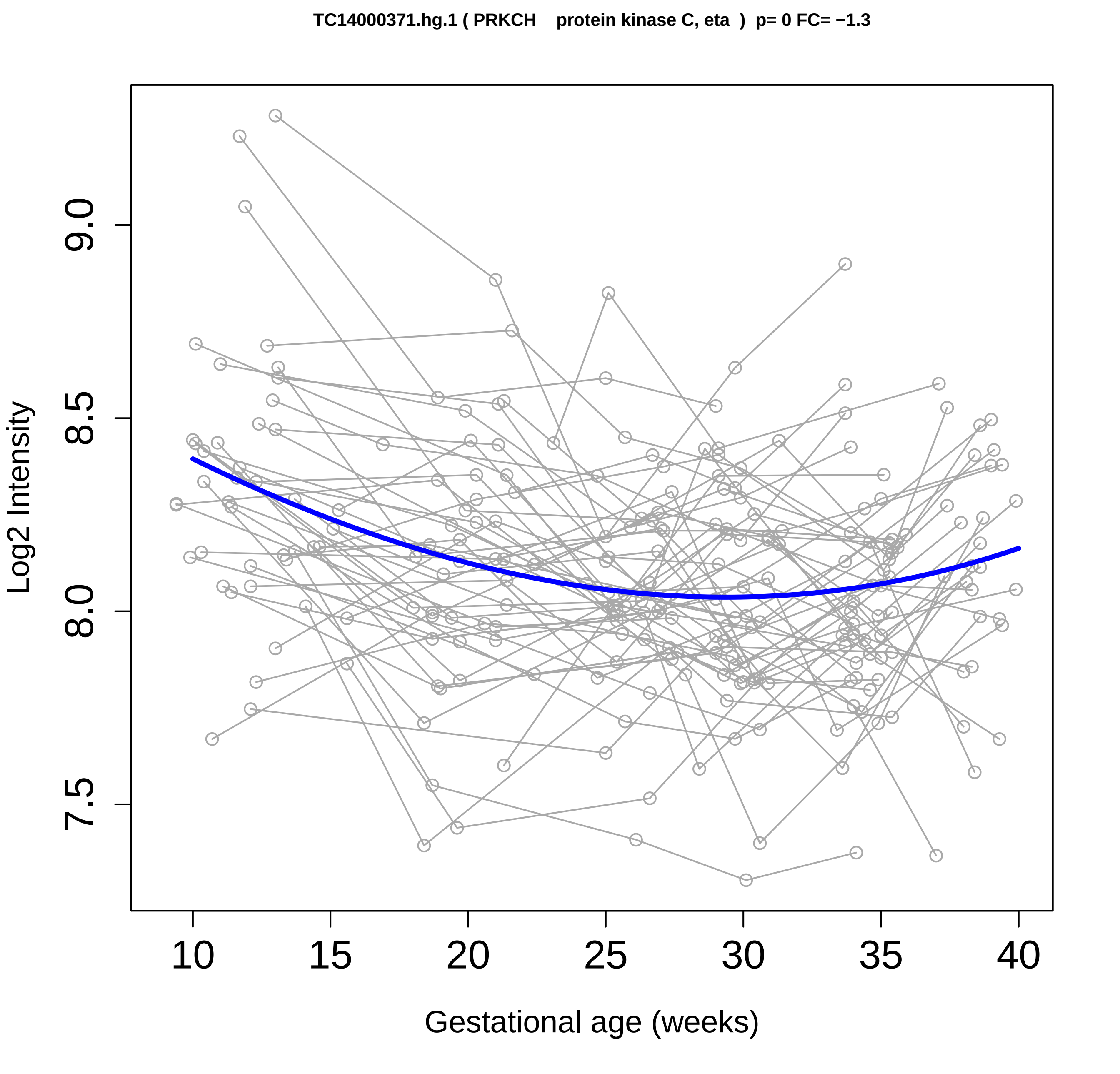

TC14000371.hg.1 (PRKCH protein kinase C, eta) p= 0 FC= −1.3
Log2 Intensity
9.0
8.5
8.0
7.5
10
15
20
25
30
35
40
Gestational age (weeks)

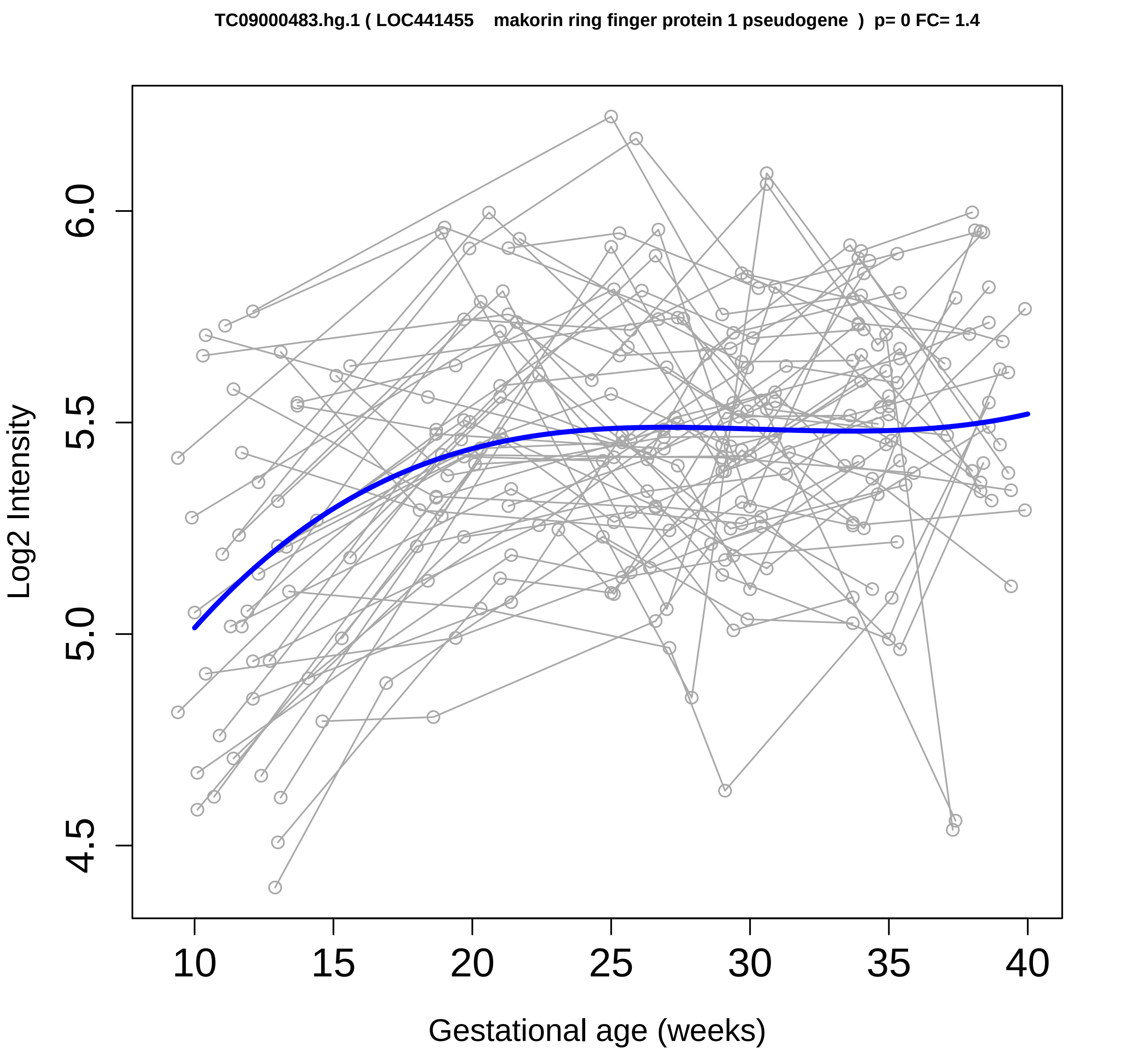
TC09000483.hg.1 (LOC441455 makorin ring finger protein 1 pseudogene) p= 0 FC= 1.4
Log2 Intensity
4.5
5.0
5.5
6.0
10
15
20
25
30
35
40
Gestational age (weeks)

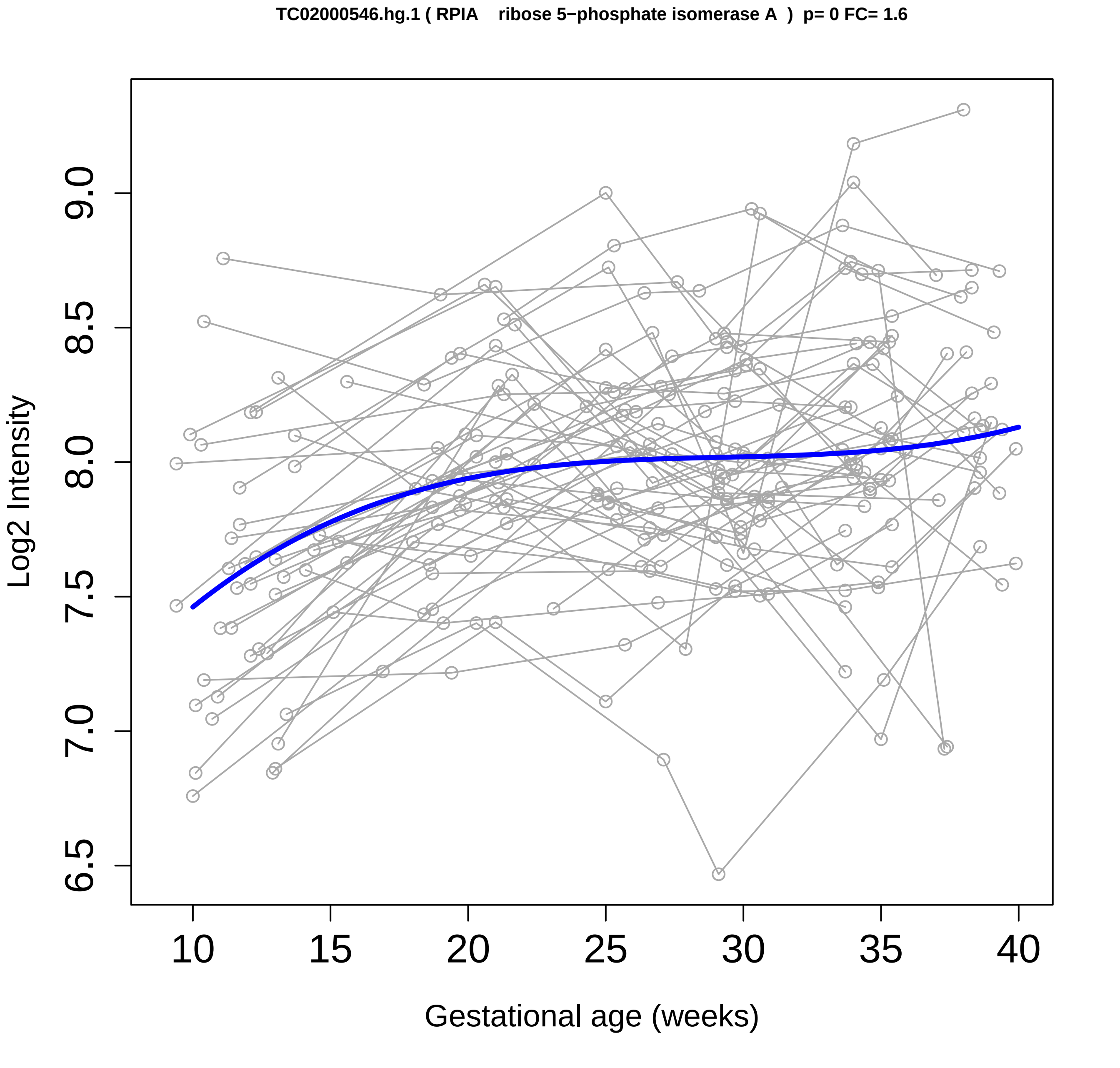

TC02000546.hg.1 (RPIA ribose 5−phosphate isomerase A) p= 0 FC= 1.6
Log2 Intensity
6.5
7.0
7.5
8.0
8.5
9.0
10
15
20
25
30
35
40
Gestational age (weeks)

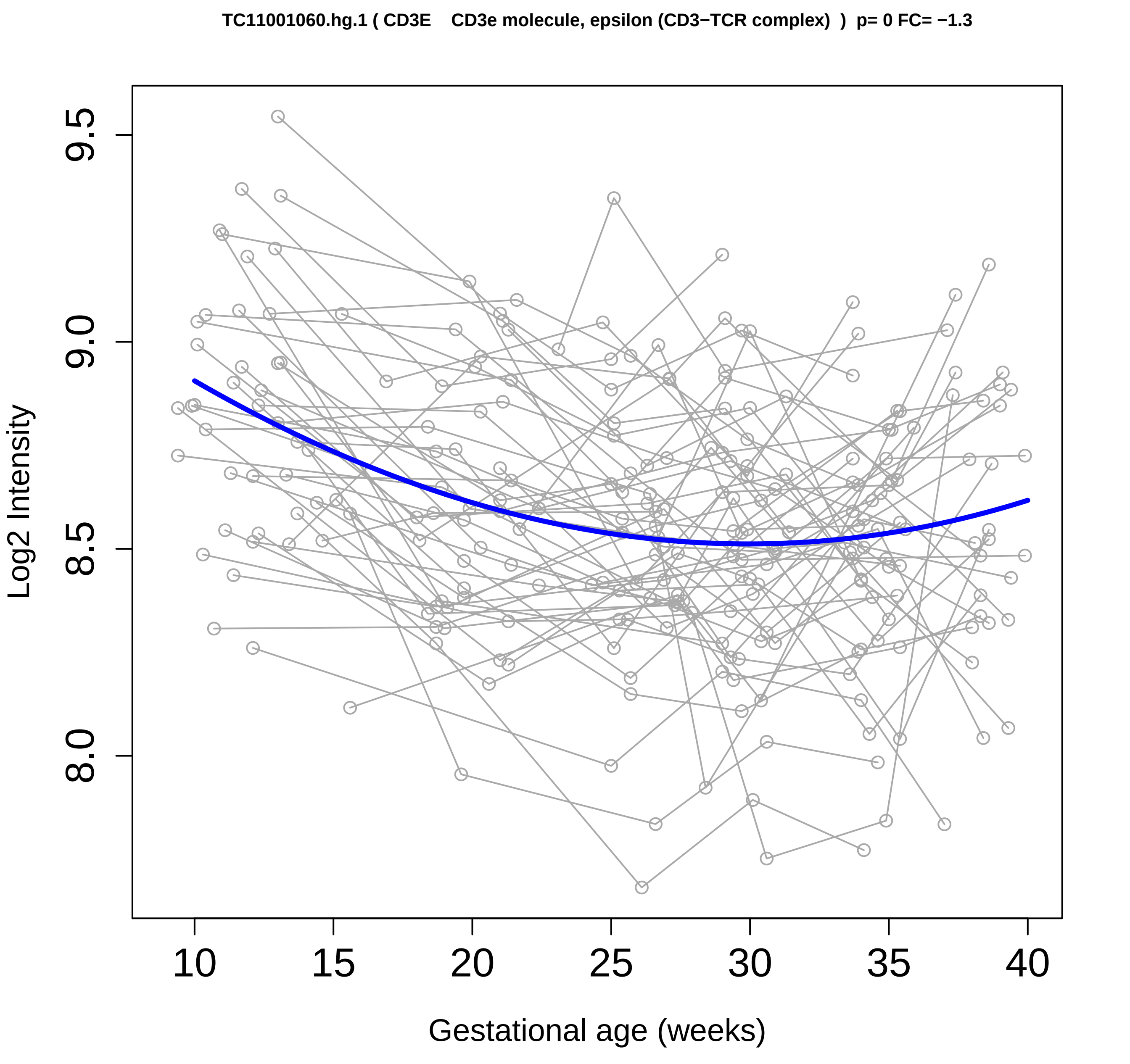

TC11001060.hg.1 (CD3E   CD3e molecule, epsilon (CD3−TCR complex)) p= 0 FC= −1.3
Log2 Intensity
9.5
9.0
8.5
8.0
10
15
20
25
30
35
40
Gestational age (weeks)

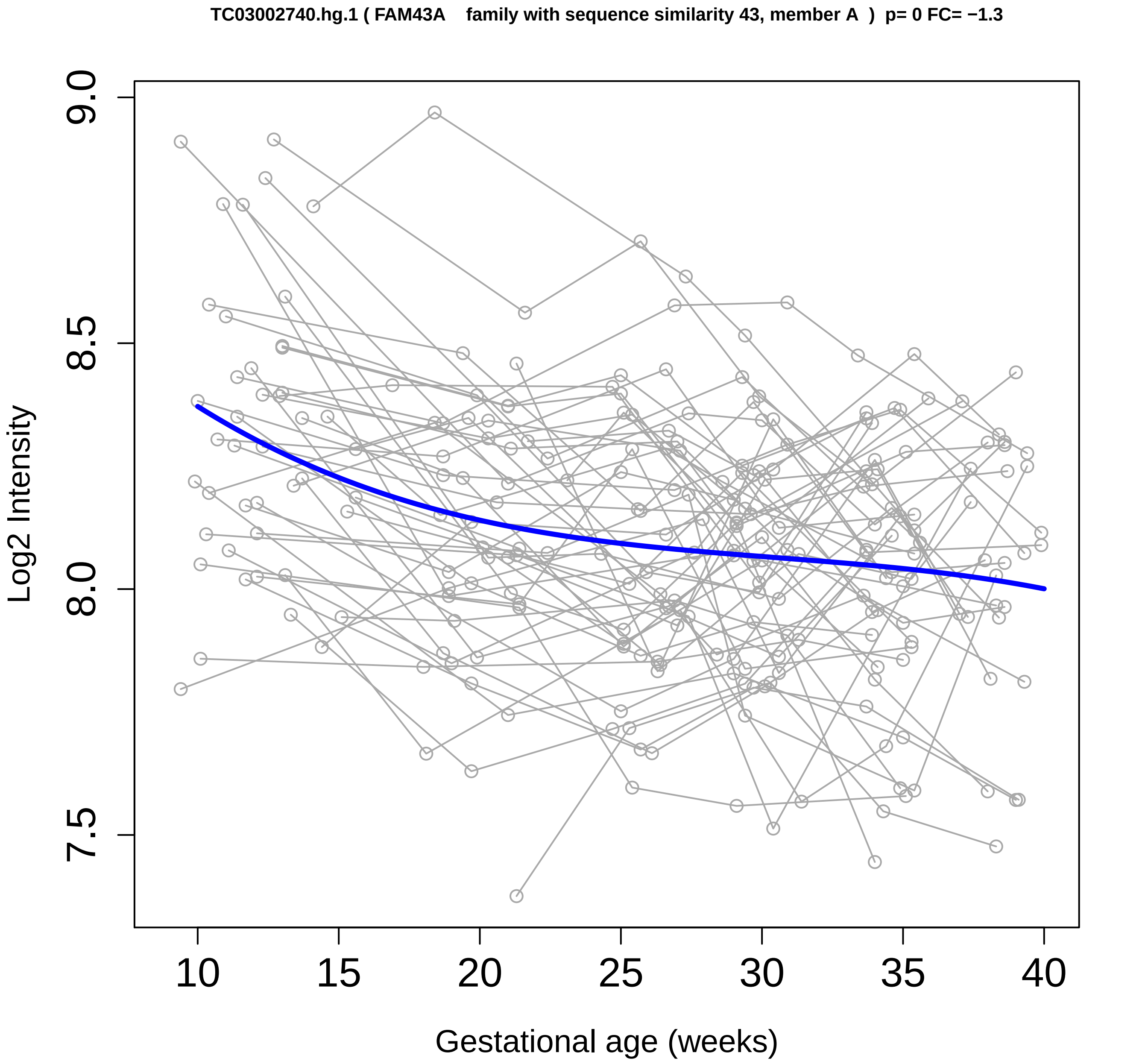
TC03002740.hg.1 (FAM43A family with sequence similarity 43, member A) p= 0 FC= −1.3
Log2 Intensity
9.0
8.5
8.0
7.5
10
15
20
25
30
35
40
Gestational age (weeks)

TC05000633.hg.1 (SLC22A4   solute carrier family 22 (organic cation/zwitterion transporter), member 4) p= 0 FC= 1.3

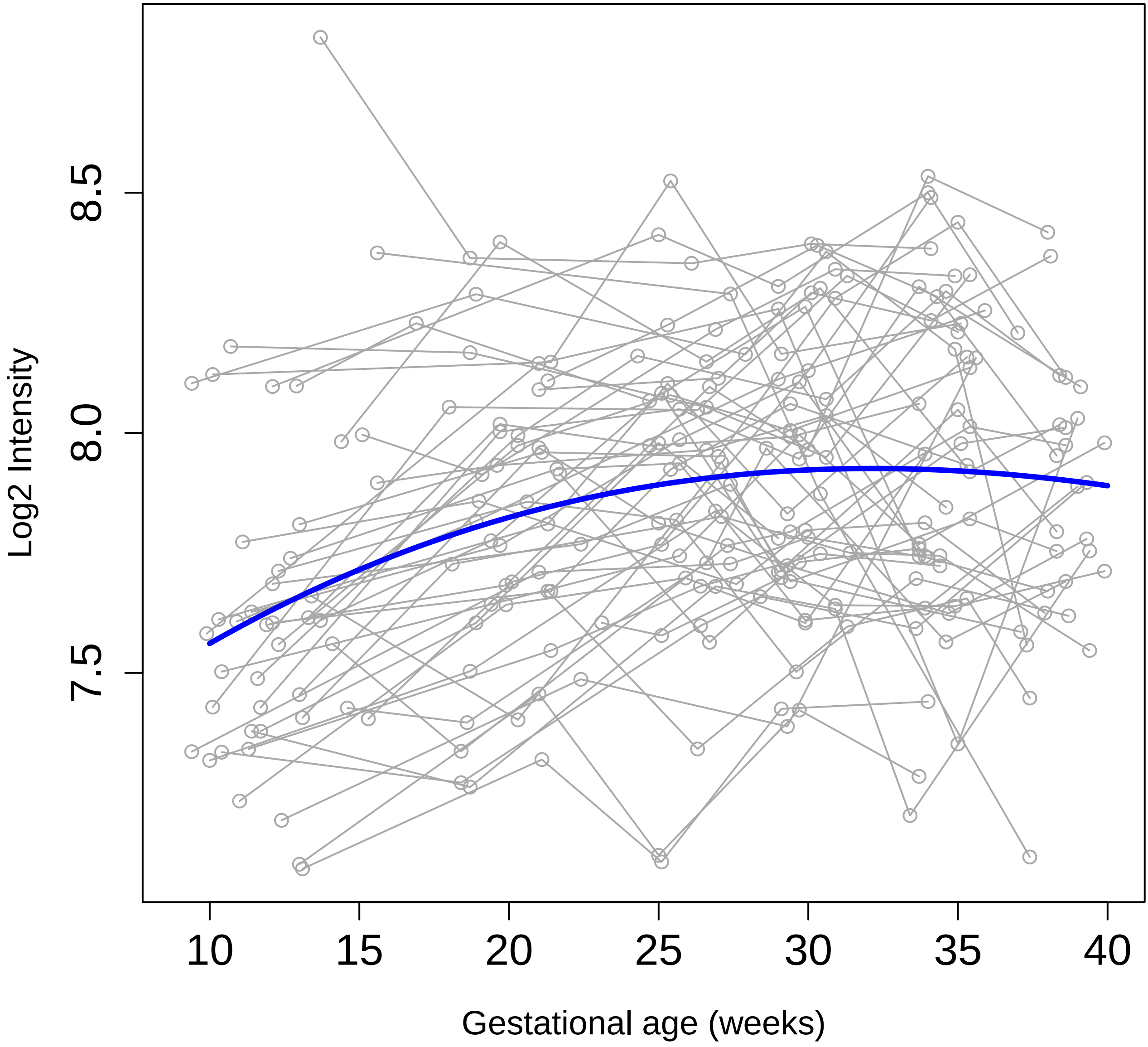

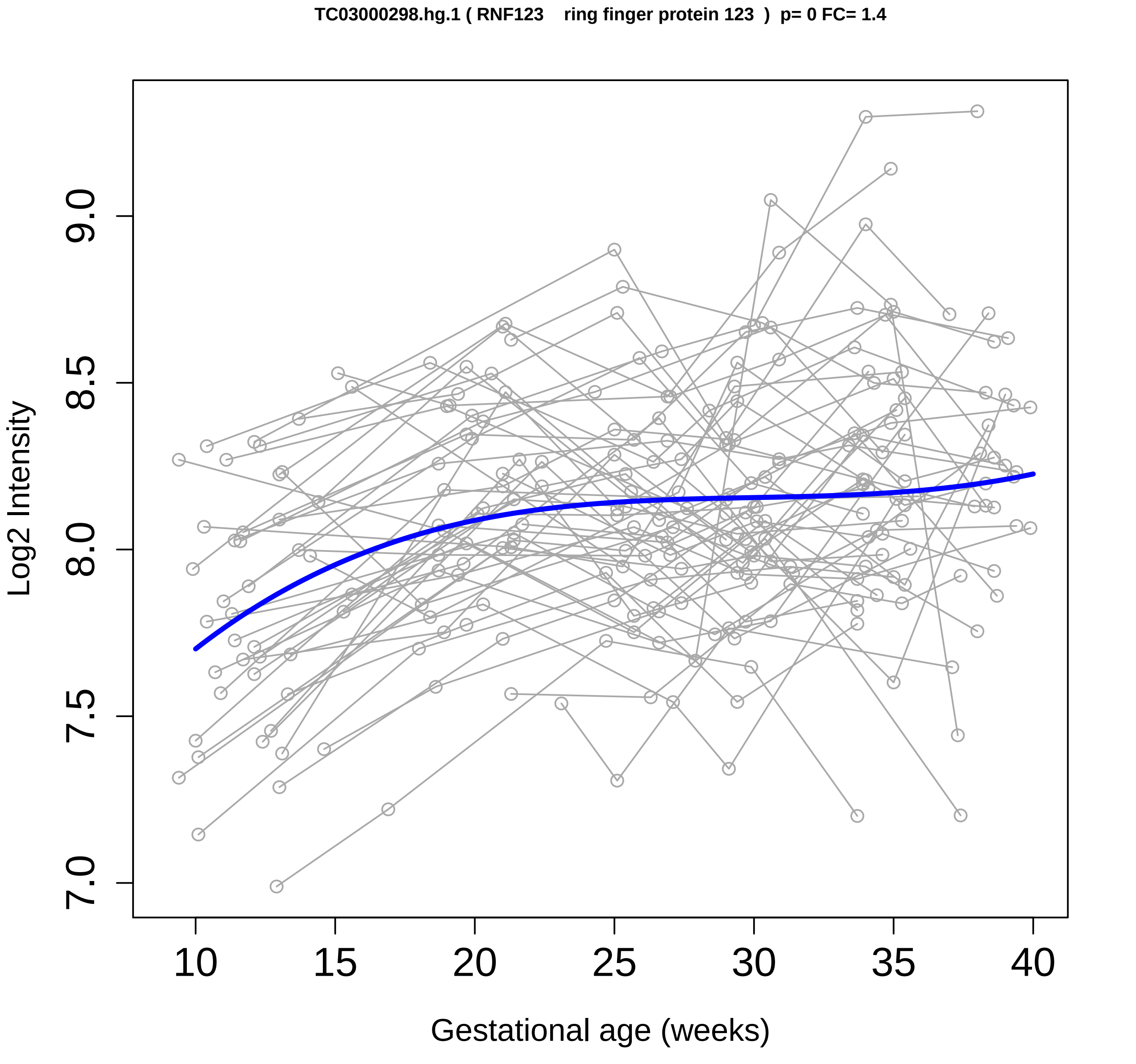

TC03000298.hg.1 (RNF123 ring finger protein 123) p= 0 FC= 1.4
Log2 Intensity
7.0
7.5
8.0
8.5
9.0
10
15
20
25
30
35
40
Gestational age (weeks)

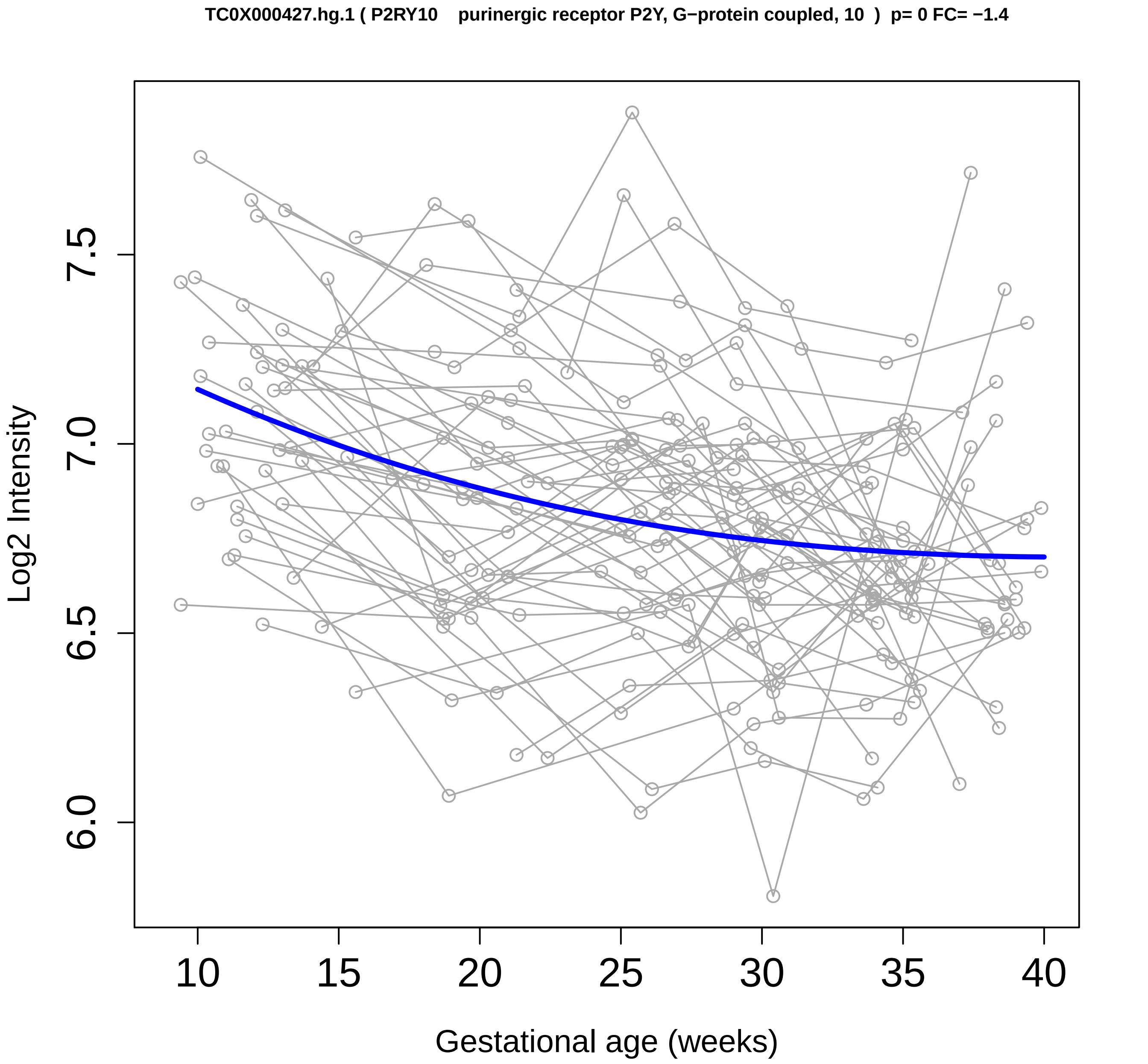
TC0X000427.hg.1 (P2RY10 purinergic receptor P2Y, G−protein coupled, 10) p= 0 FC= −1.4
Log2 Intensity
Gestational age (weeks)
6.0
6.5
7.0
7.5
10
15
20
25
30
35
40

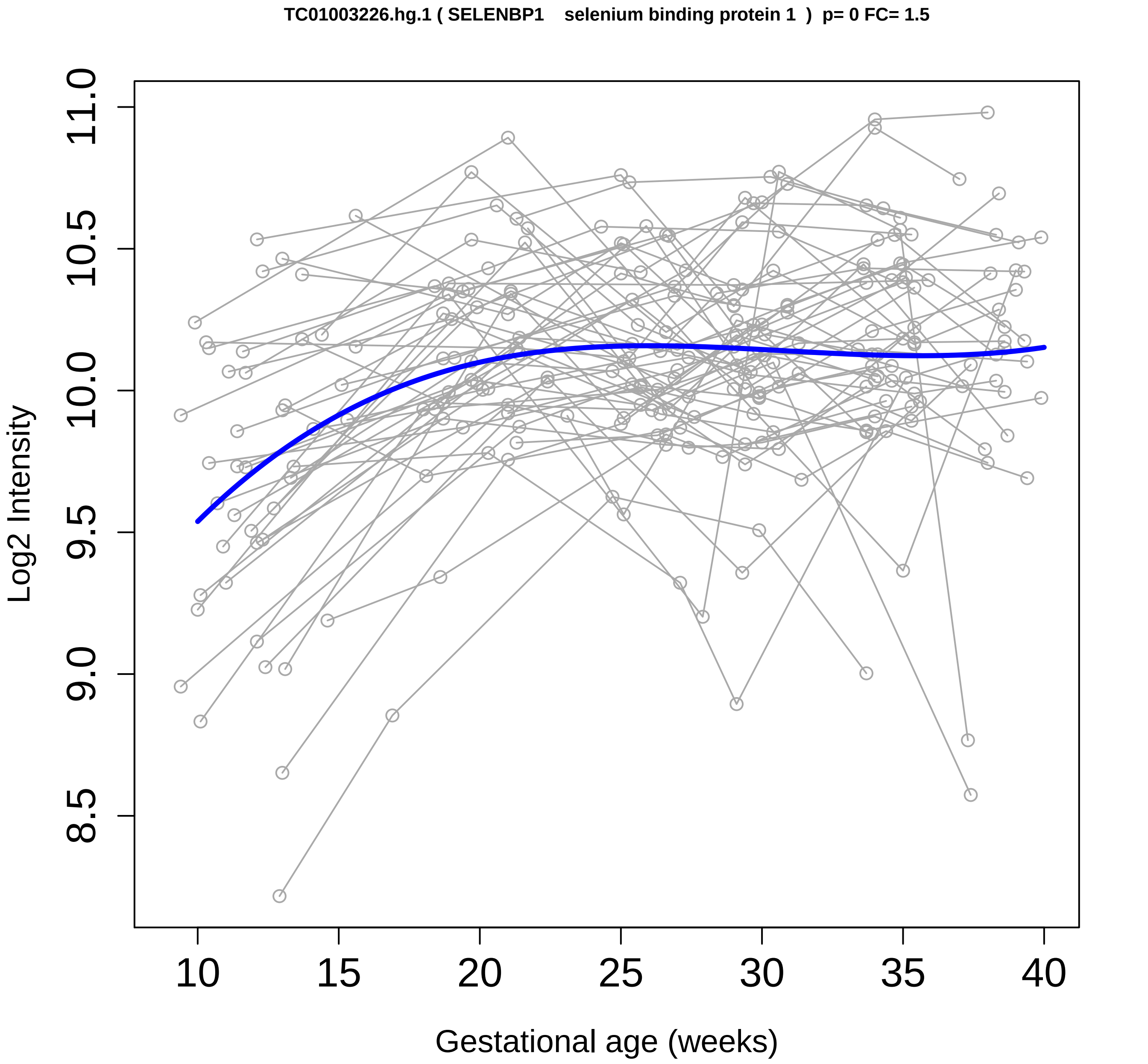
TC01003226.hg.1 (SELENBP1 selenium binding protein 1) p= 0 FC= 1.5
Log2 Intensity
11.0
10.5
10.0
9.5
9.0
8.5
10
15
20
25
30
35
40
Gestational age (weeks)

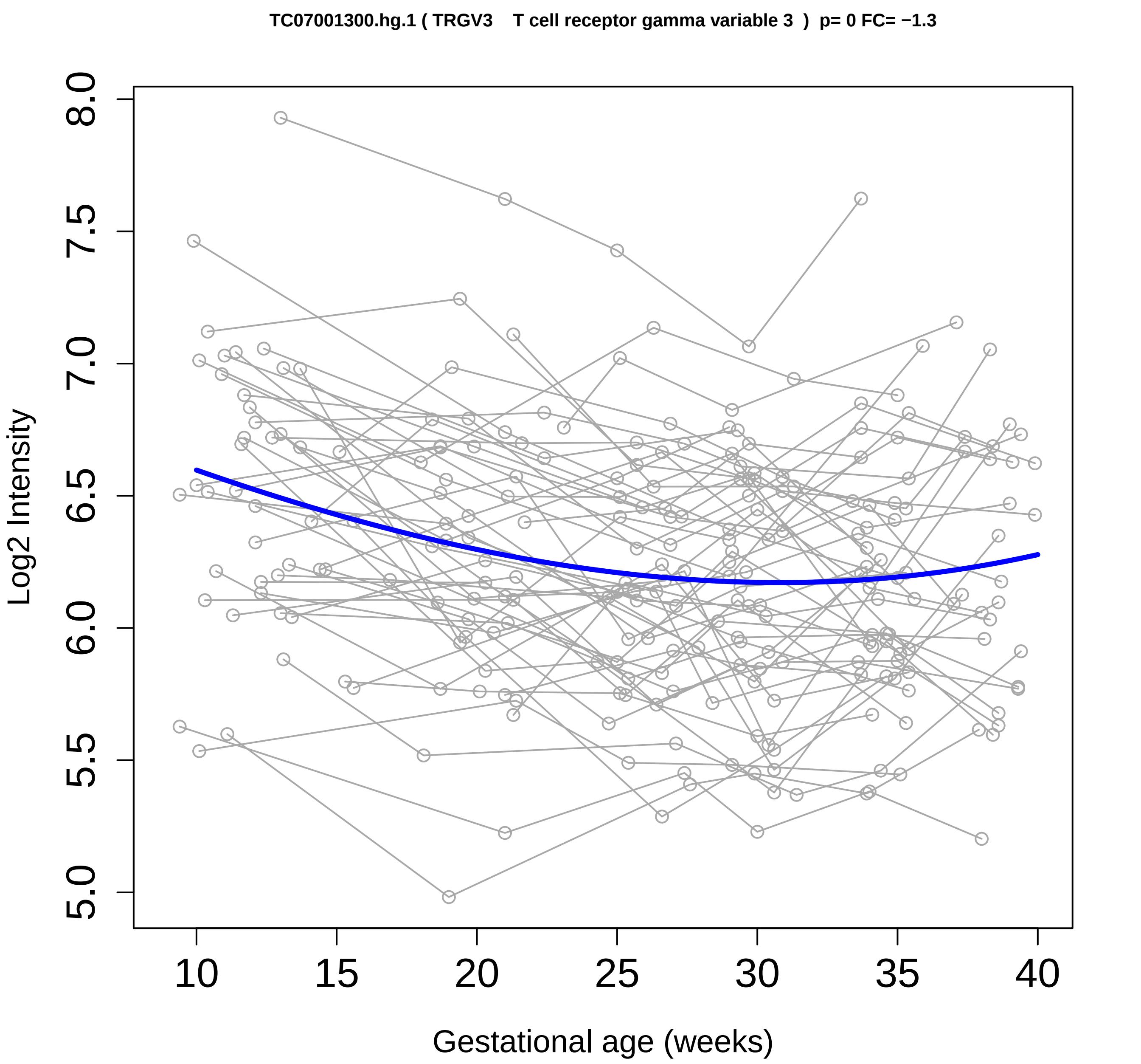
TC07001300.hg.1 (TRGV3 T cell receptor gamma variable 3) p= 0 FC= −1.3
Log2 Intensity
8.0
7.5
7.0
6.5
6.0
5.5
5.0
10
15
20
25
30
35
40
Gestational age (weeks)

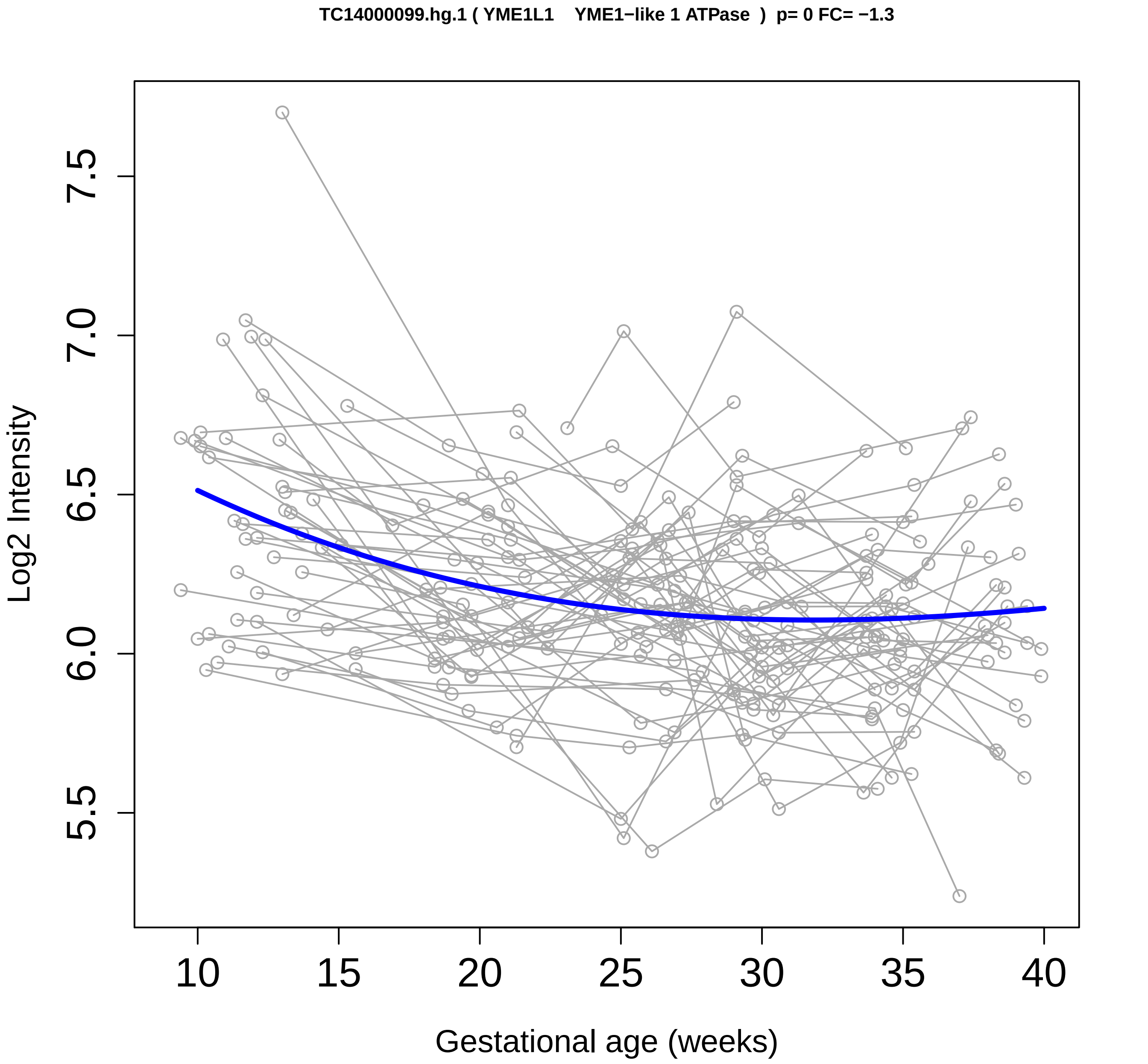

TC14000099.hg.1 (YME1L1   YME1−like 1 ATPase) p= 0 FC= −1.3
Log2 Intensity
7.5
7.0
6.5
6.0
5.5
10
15
20
25
30
35
40
Gestational age (weeks)

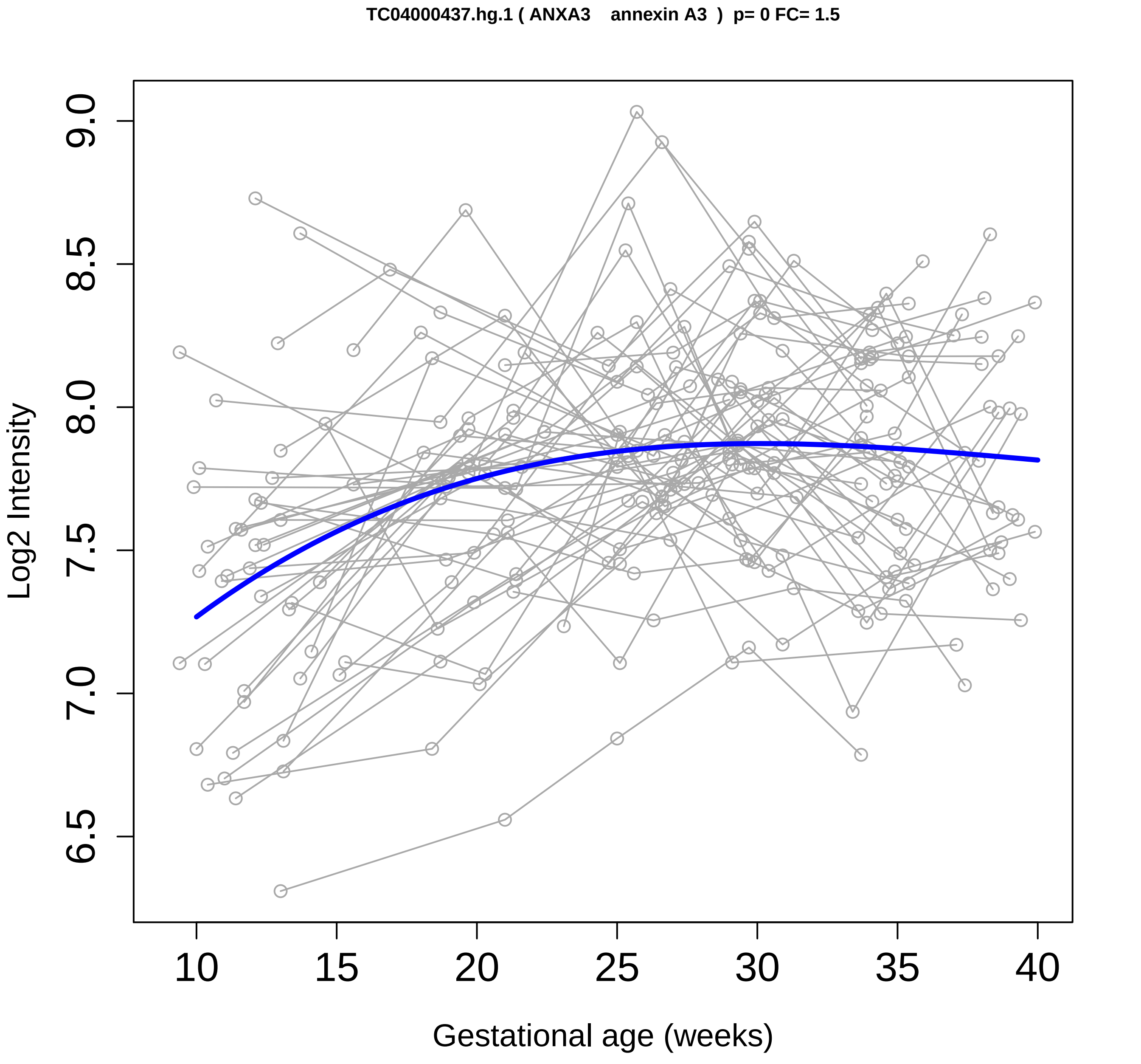
TC04000437.hg.1 (ANXA3 annexin A3) p= 0 FC= 1.5
Log2 Intensity
9.0
8.5
8.0
7.5
7.0
6.5
10
15
20
25
30
35
40
Gestational age (weeks)

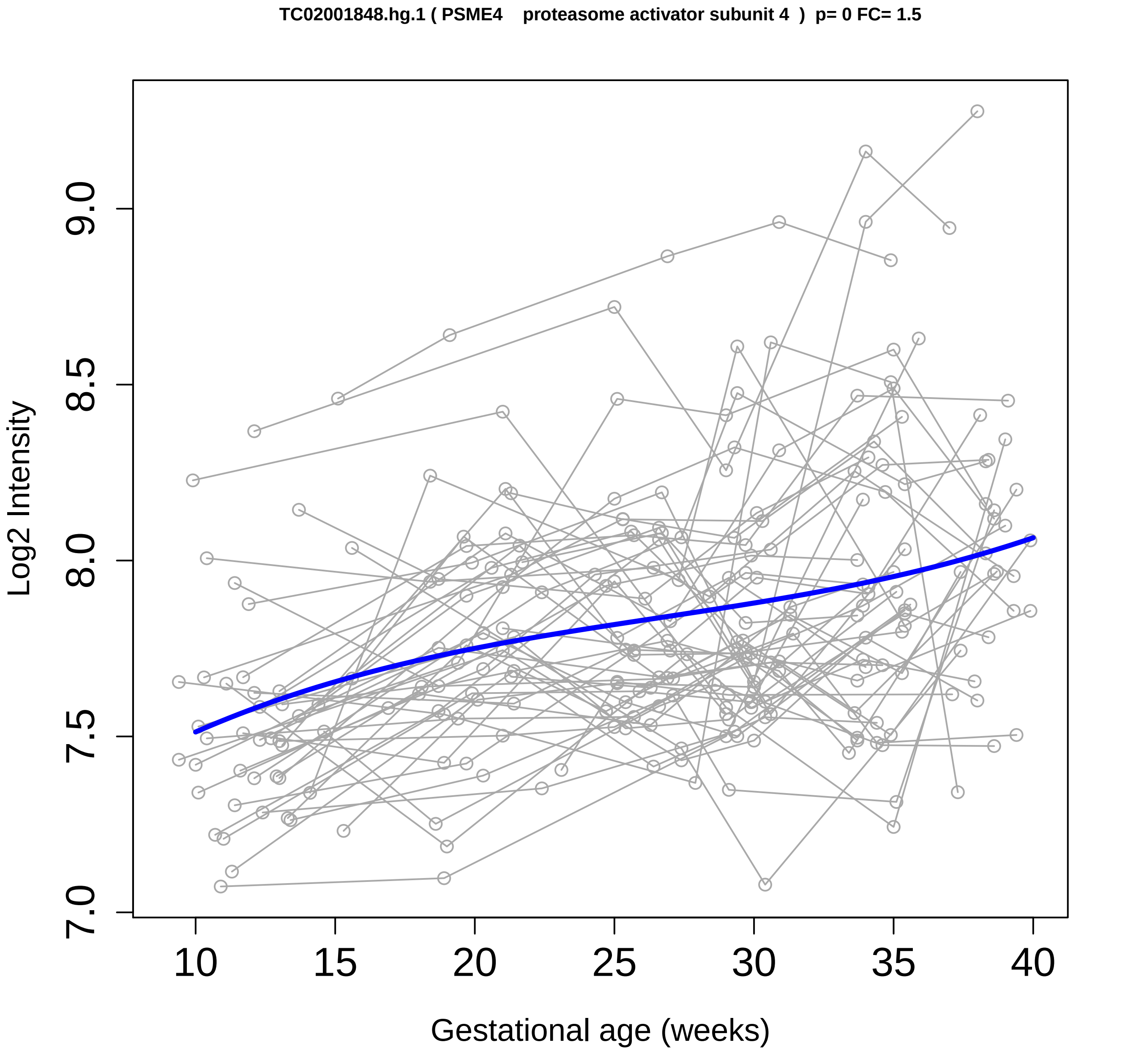
TC02001848.hg.1 (PSME4 proteasome activator subunit 4) p= 0 FC= 1.5
Log2 Intensity
7.0
7.5
8.0
8.5
9.0
10
15
20
25
30
35
40
Gestational age (weeks)

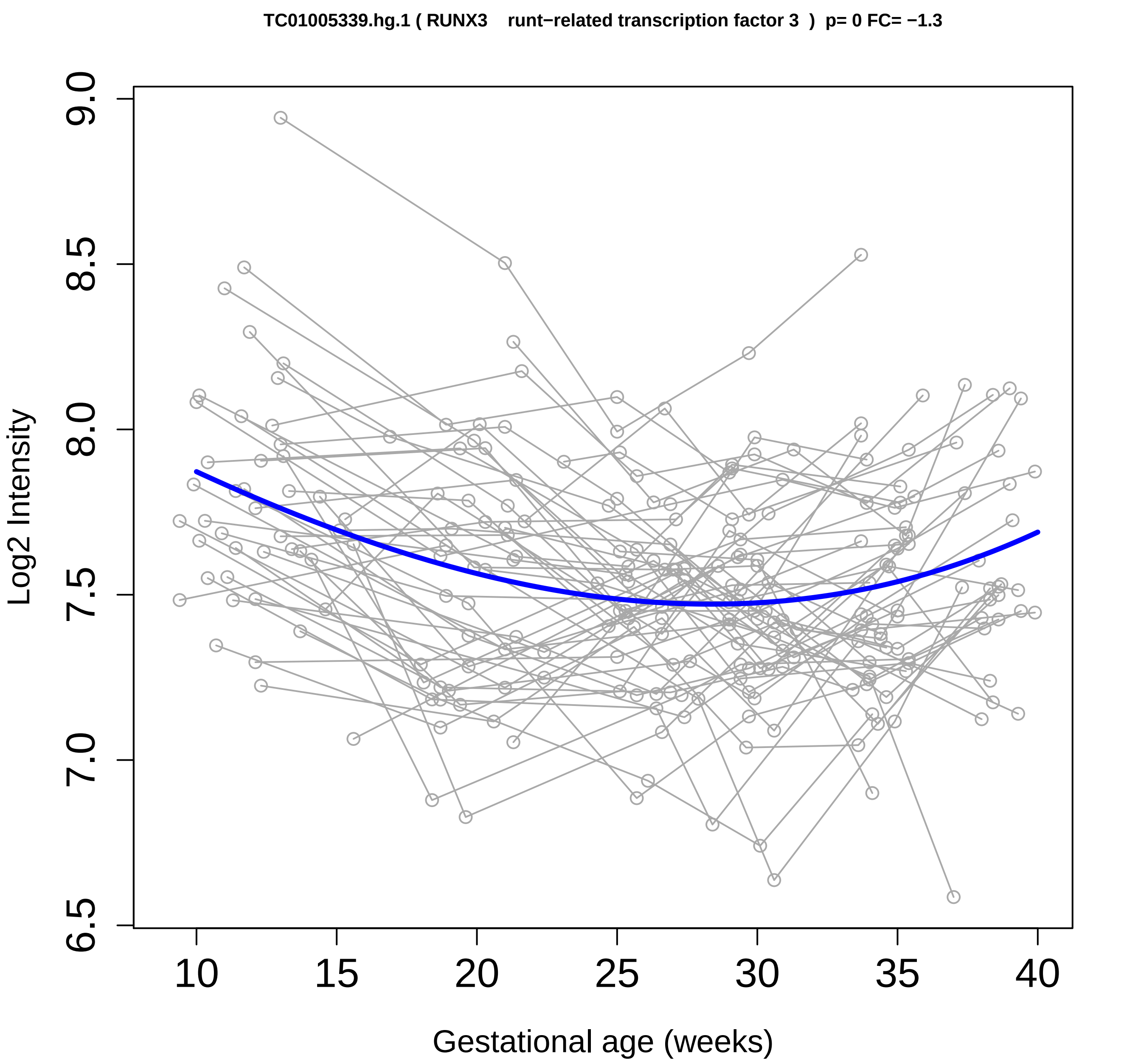

TC01005339.hg.1 (RUNX3 runt−related transcription factor 3) p= 0 FC= −1.3
Log2 Intensity
9.0
8.5
8.0
7.5
7.0
6.5
10
15
20
25
30
35
40
Gestational age (weeks)

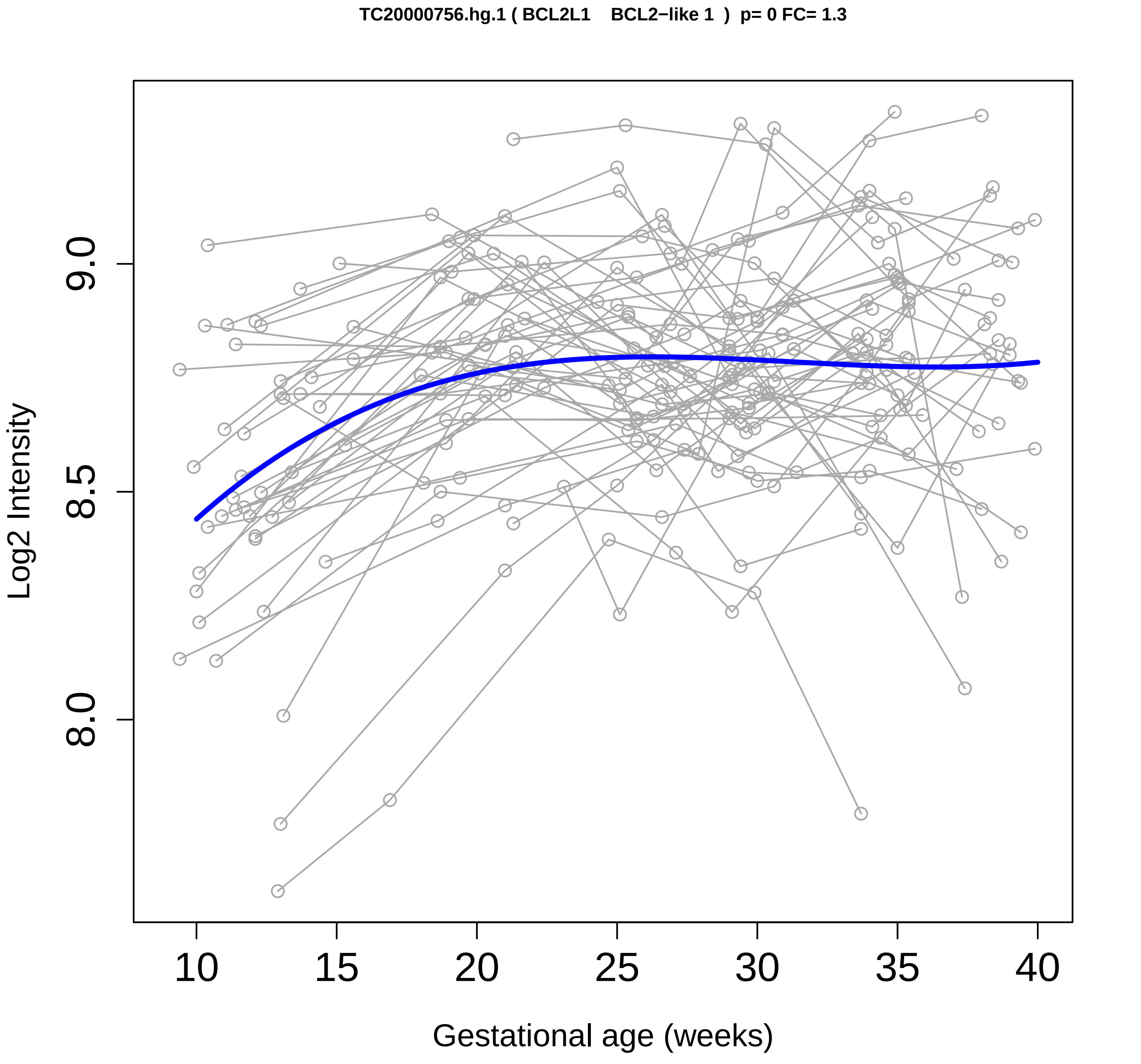
TC20000756.hg.1 (BCL2L1 BCL2−like 1) p= 0 FC= 1.3
Log2 Intensity
8.0
8.5
9.0
10
15
20
25
30
35
40
Gestational age (weeks)

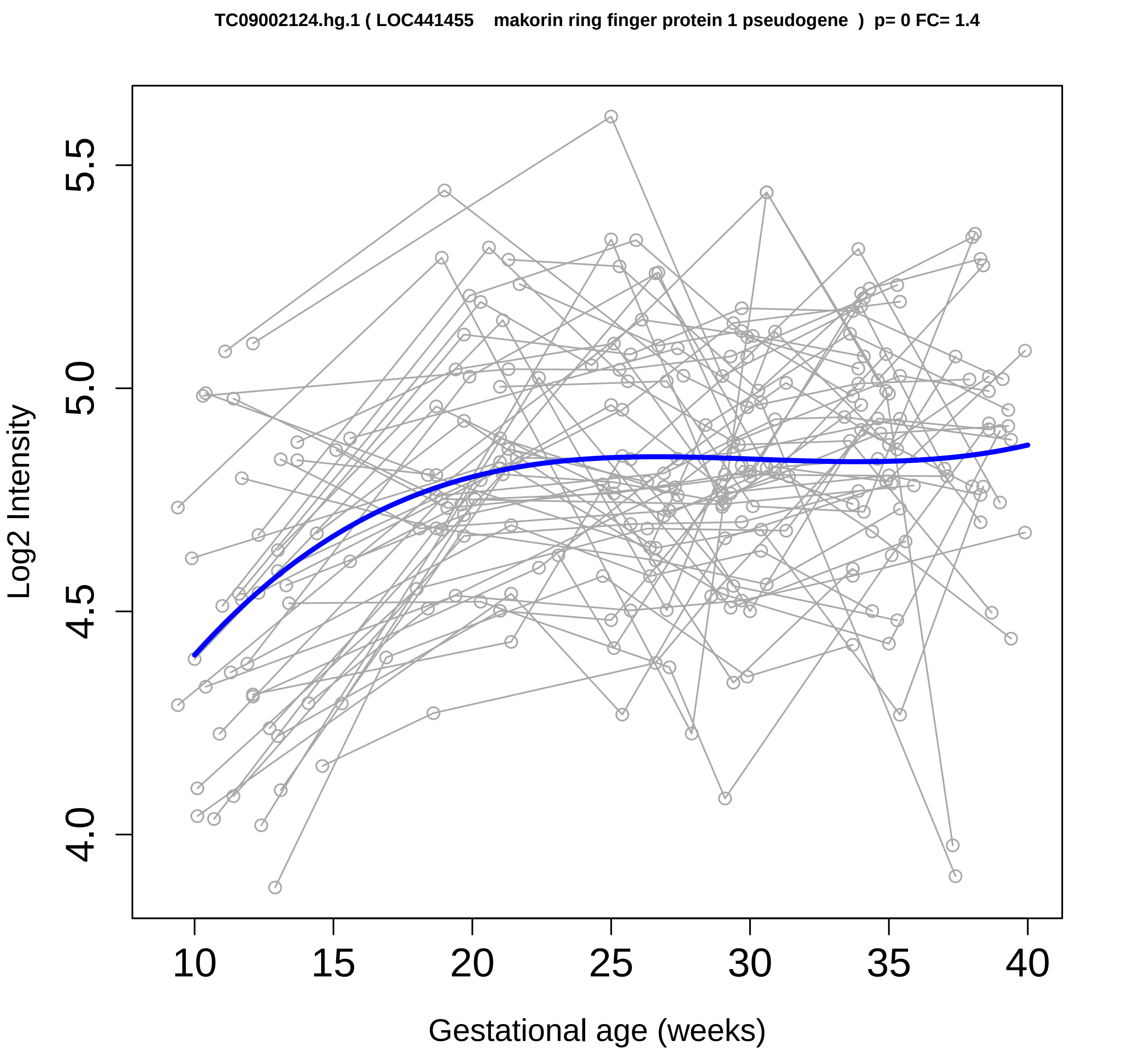

TC09002124.hg.1 (LOC441455 makorin ring finger protein 1 pseudogene) p= 0 FC= 1.4
Log2 Intensity
4.0
4.5
5.0
5.5
10
15
20
25
30
35
40
Gestational age (weeks)

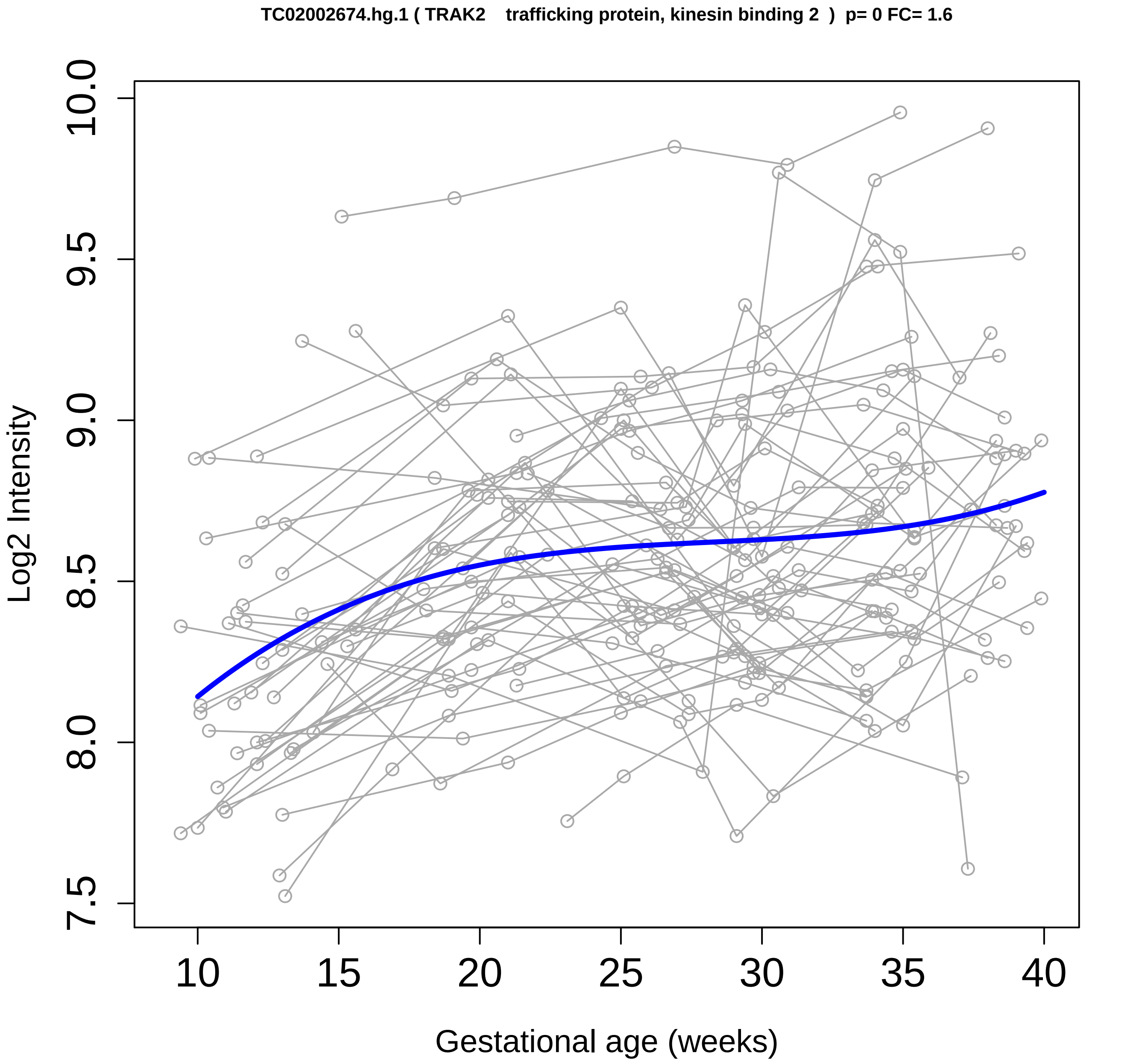

TC02002674.hg.1 (TRAK2 trafficking protein, kinesin binding 2) p= 0 FC= 1.6
Log2 Intensity
7.5
8.0
8.5
9.0
9.5
10.0
10
15
20
25
30
35
40
Gestational age (weeks)

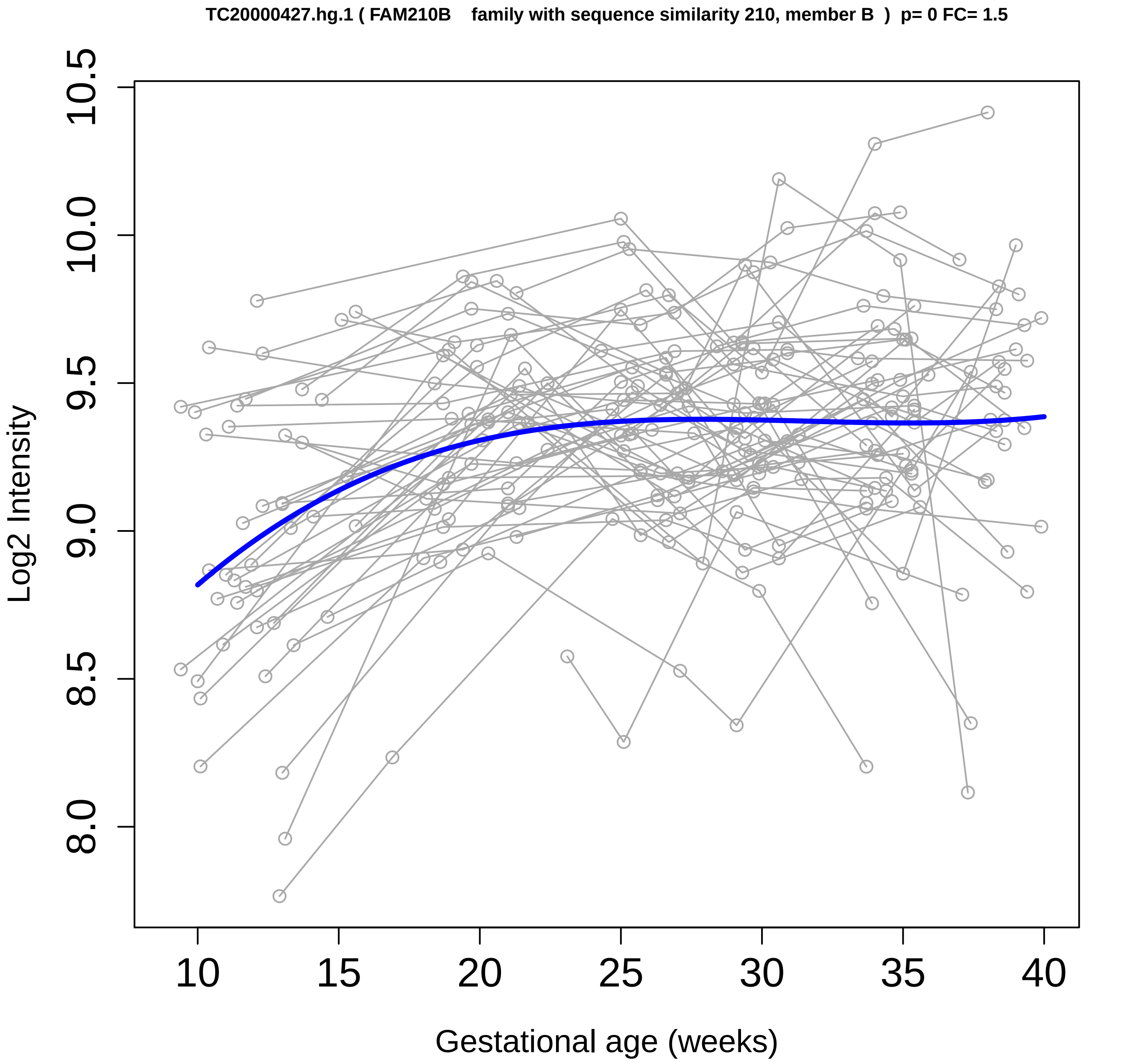

TC20000427.hg.1 (FAM210B family with sequence similarity 210, member B) p= 0 FC= 1.5
Log2 Intensity
10.5
10.0
9.5
9.0
8.5
8.0
10
15
20
25
30
35
40
Gestational age (weeks)

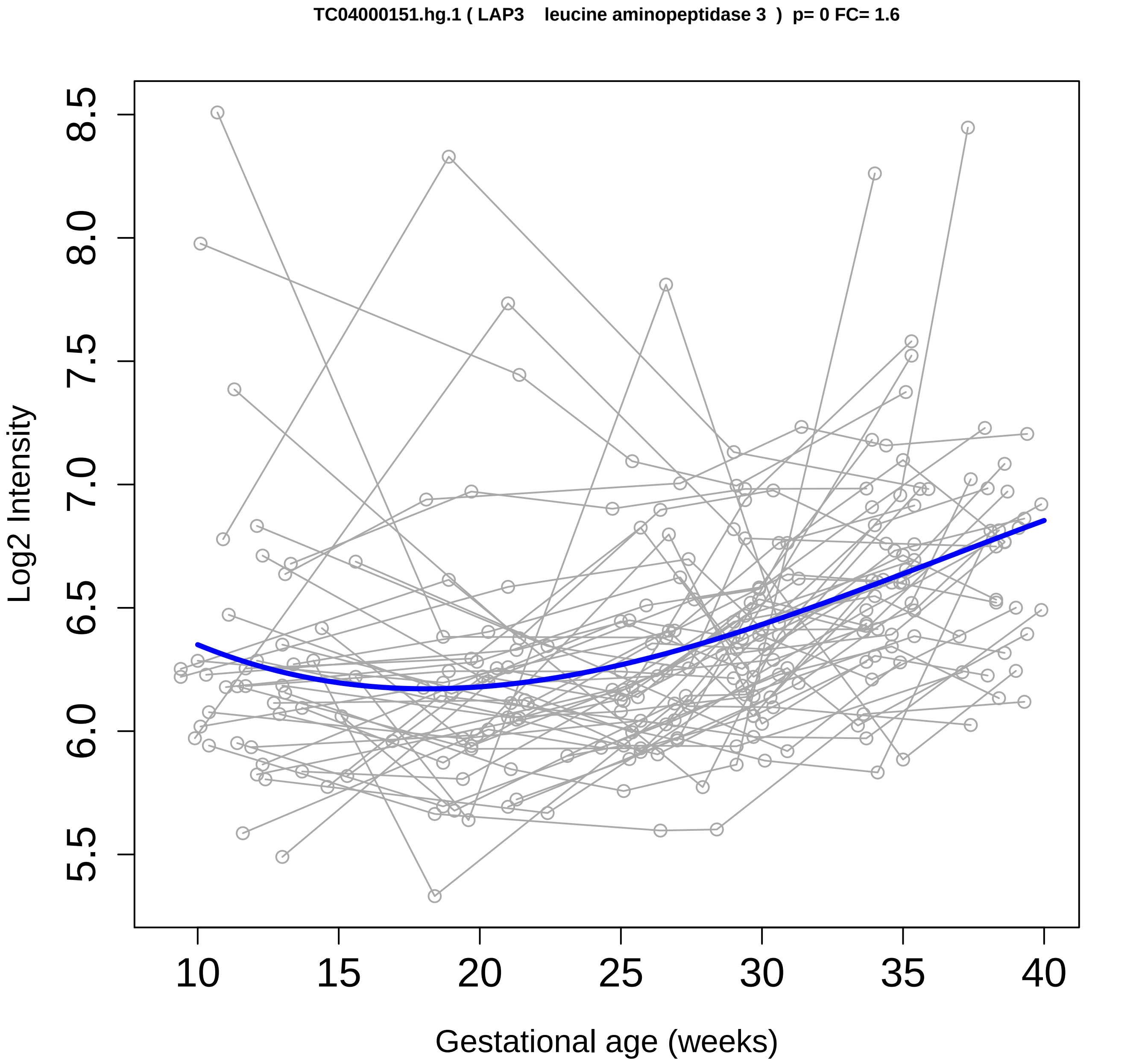
TC04000151.hg.1 (LAP3   leucine aminopeptidase 3) p= 0 FC= 1.6
Log2 Intensity
8.5
8.0
7.5
7.0
6.5
6.0
5.5
10
15
20
25
30
35
40
Gestational age (weeks)

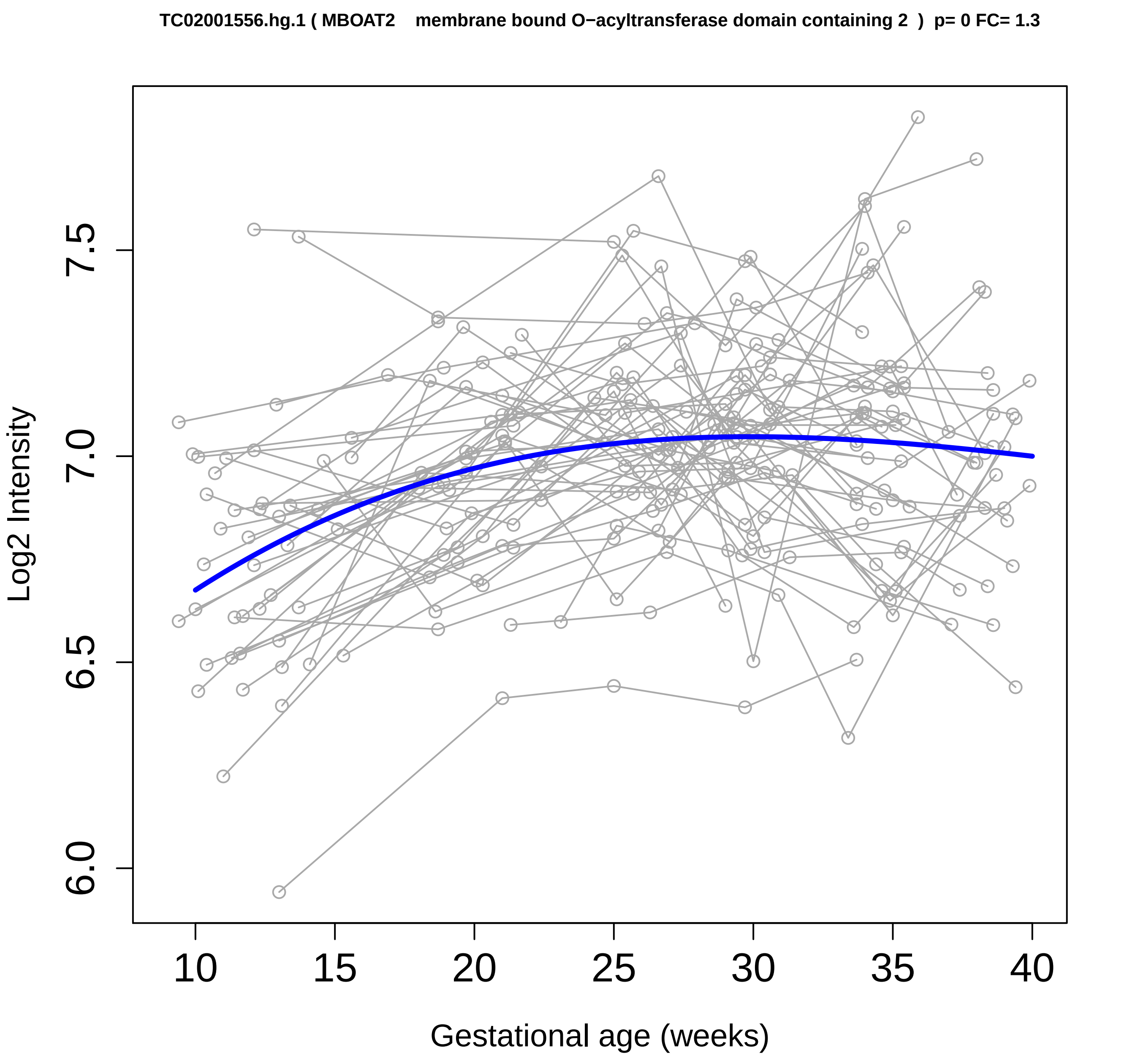
TC02001556.hg.1 (MBOAT2 membrane bound O−acyltransferase domain containing 2) p= 0 FC= 1.3
Log2 Intensity
6.0
6.5
7.0
7.5
10
15
20
25
30
35
40
Gestational age (weeks)

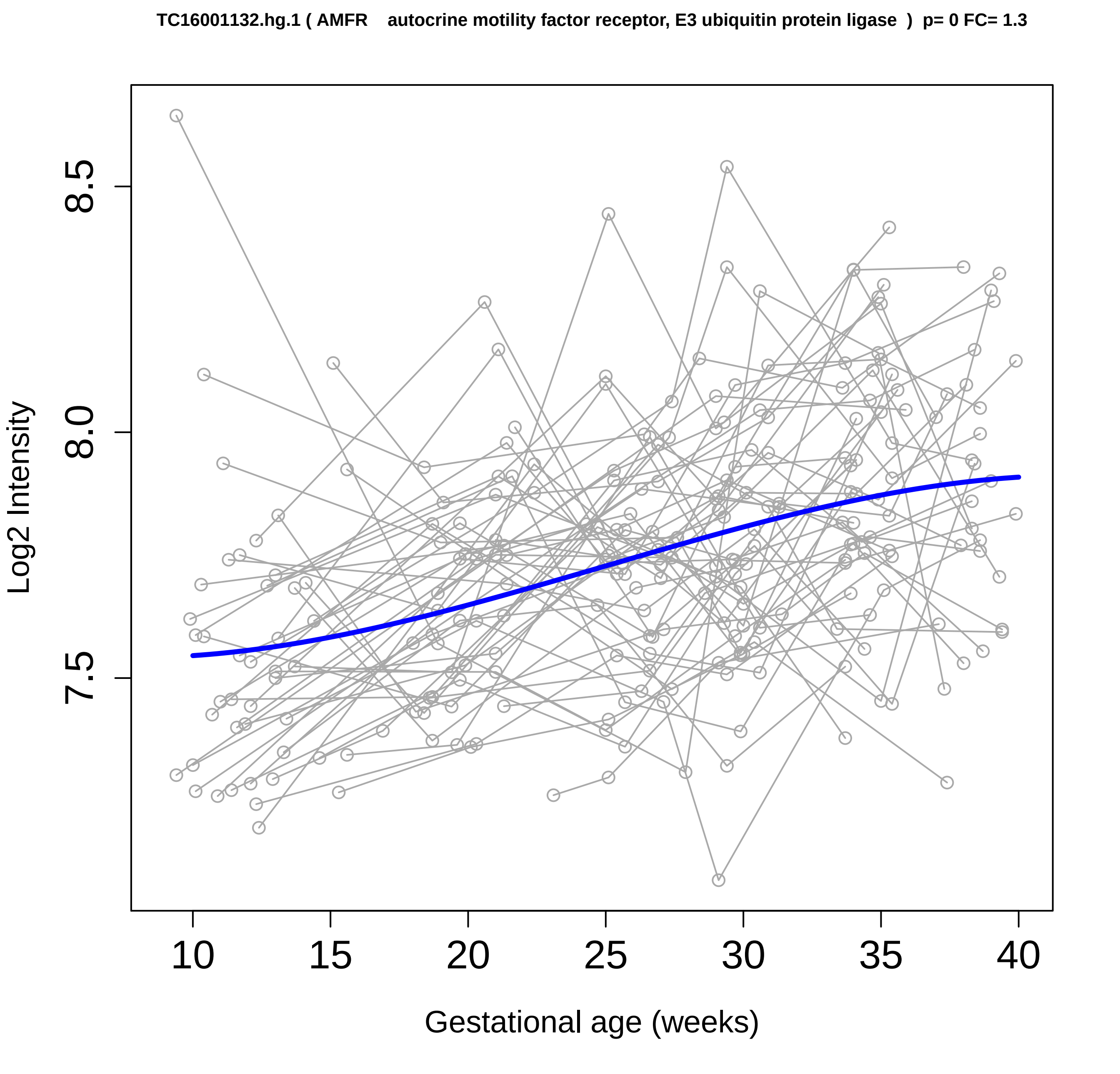

TC16001132.hg.1 (AMFR autocrine motility factor receptor, E3 ubiquitin protein ligase) p= 0 FC= 1.3
Log2 Intensity
8.5
8.0
7.5
10
15
20
25
30
35
40
Gestational age (weeks)

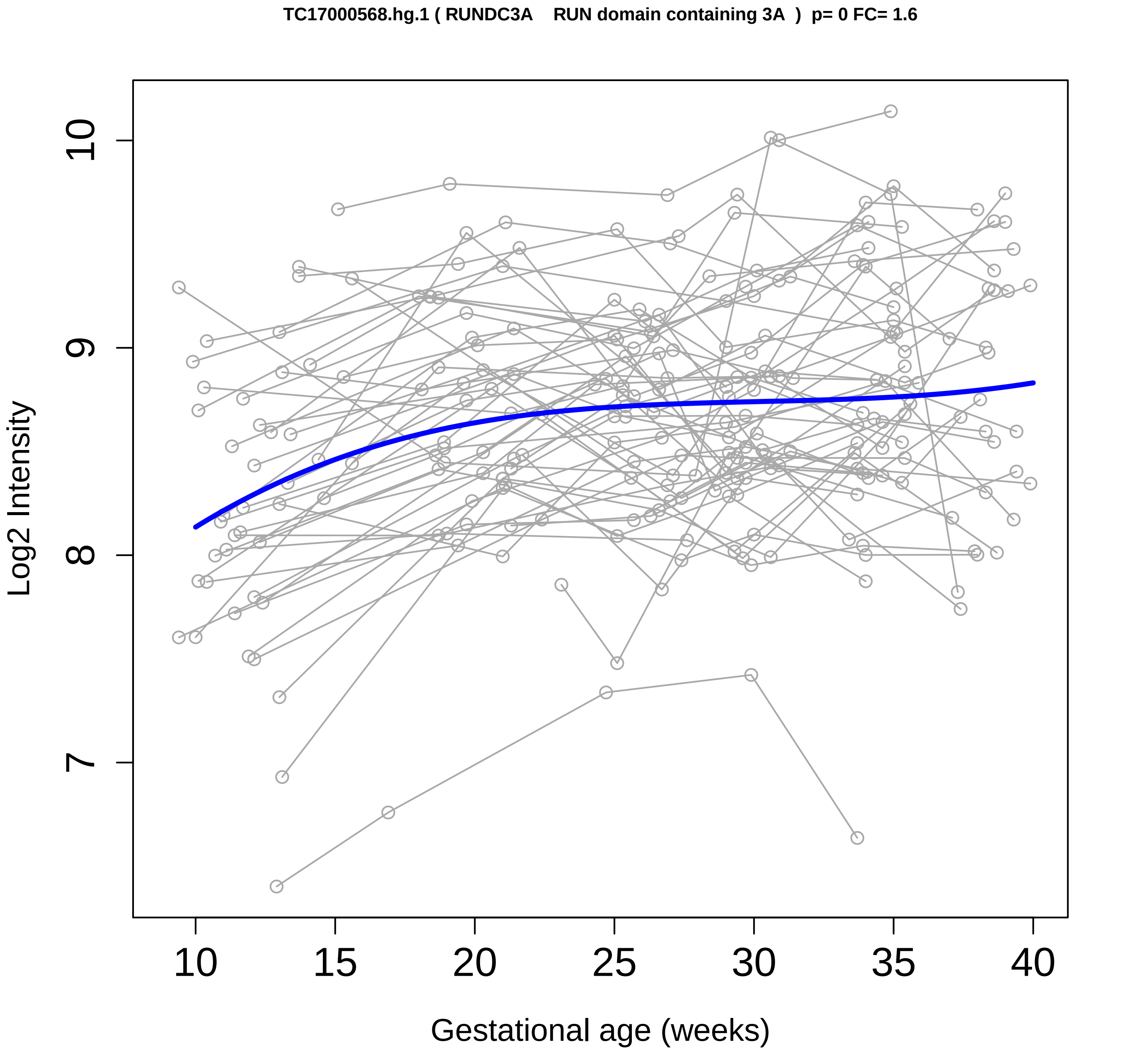
TC17000568.hg.1 (RUNDC3A RUN domain containing 3A) p= 0 FC= 1.6
Log2 Intensity
7
8
9
10
10
15
20
25
30
35
40
Gestational age (weeks)

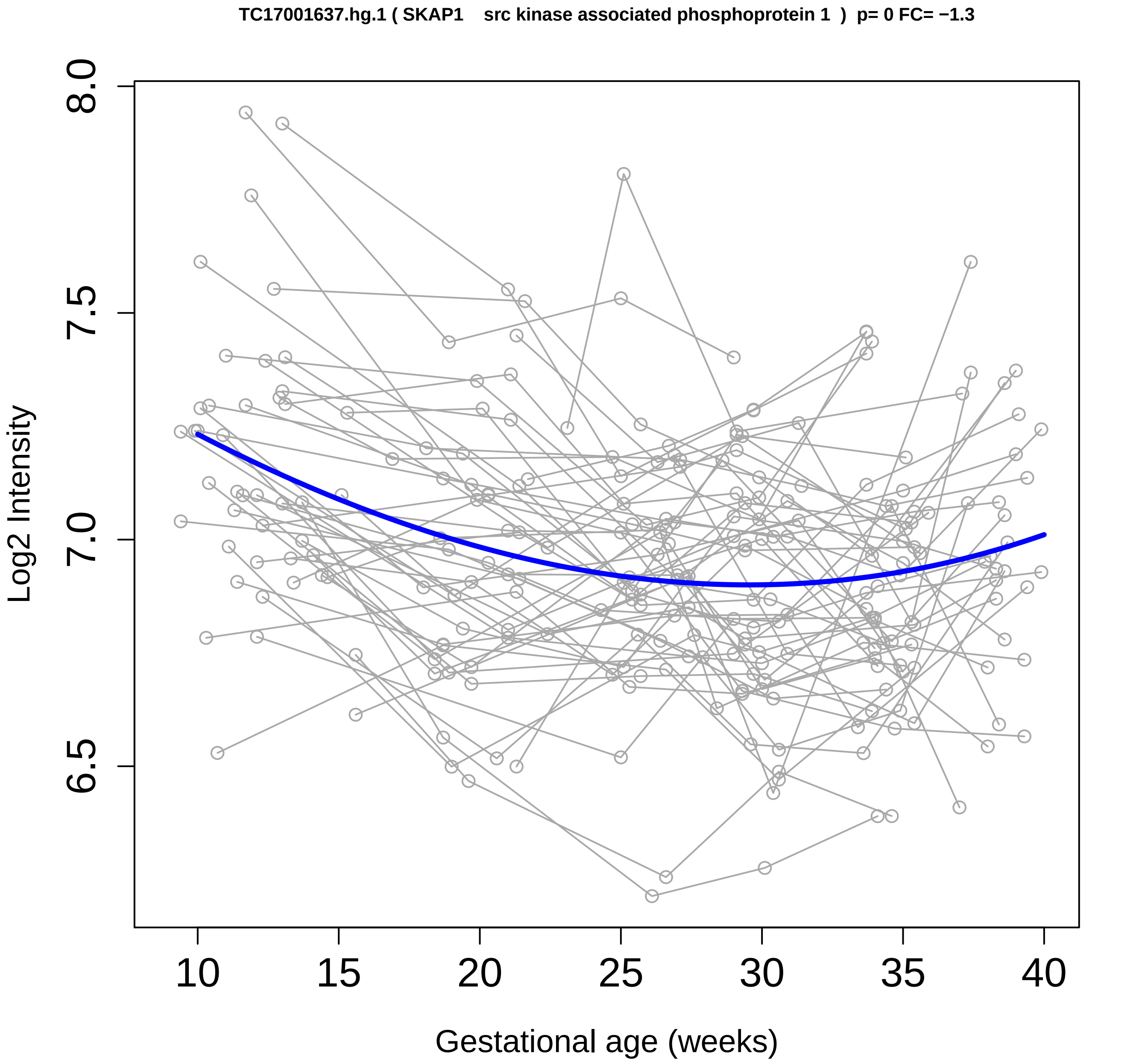

TC17001637.hg.1 (SKAP1 src kinase associated phosphoprotein 1) p= 0 FC= −1.3
Log2 Intensity
8.0
7.5
7.0
6.5
10
15
20
25
30
35
40
Gestational age (weeks)

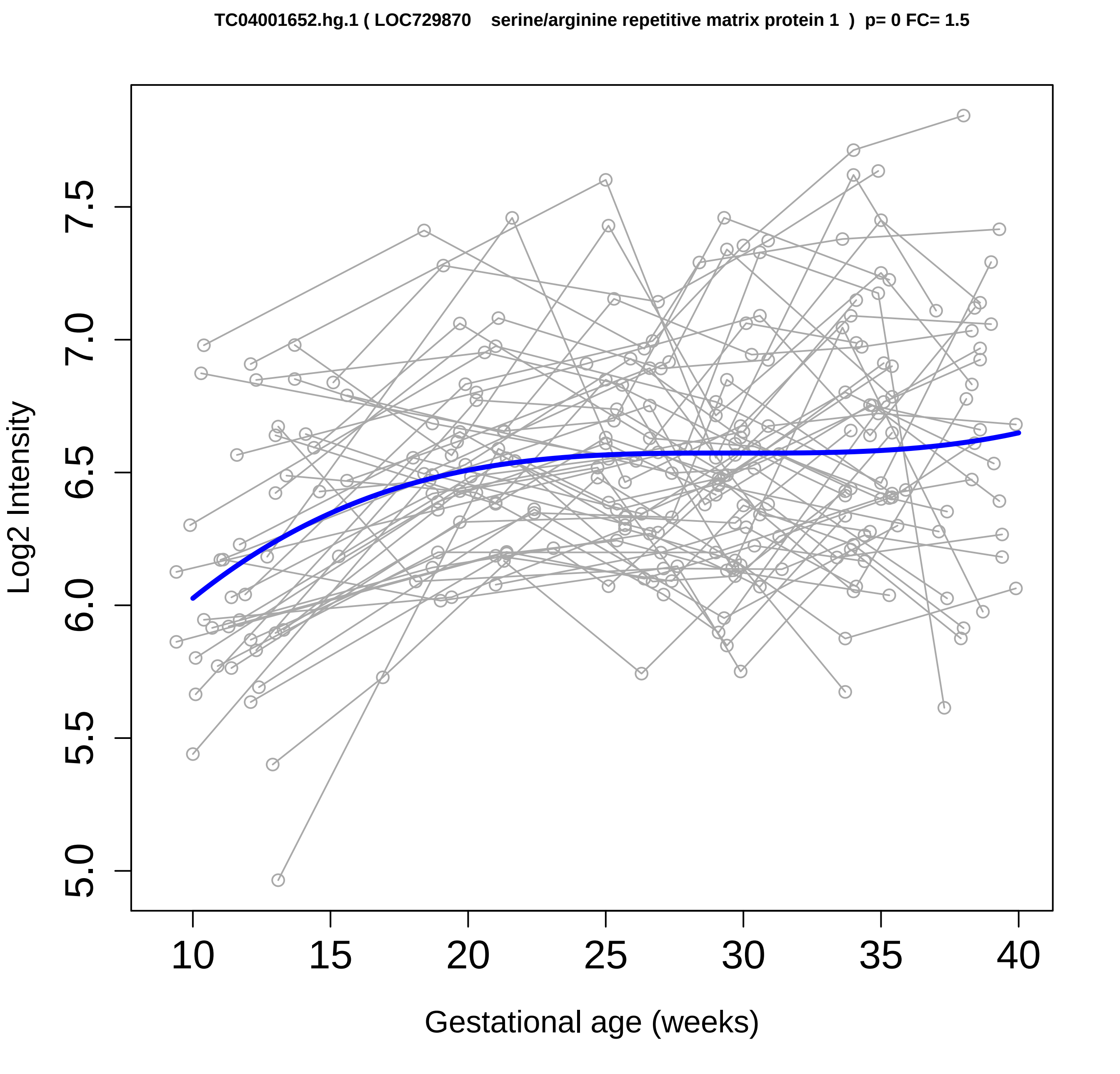
TC04001652.hg.1 (LOC729870 serine/arginine repetitive matrix protein 1) p= 0 FC= 1.5
Log2 Intensity
5.0
5.5
6.0
6.5
7.0
7.5
10
15
20
25
30
35
40
Gestational age (weeks)

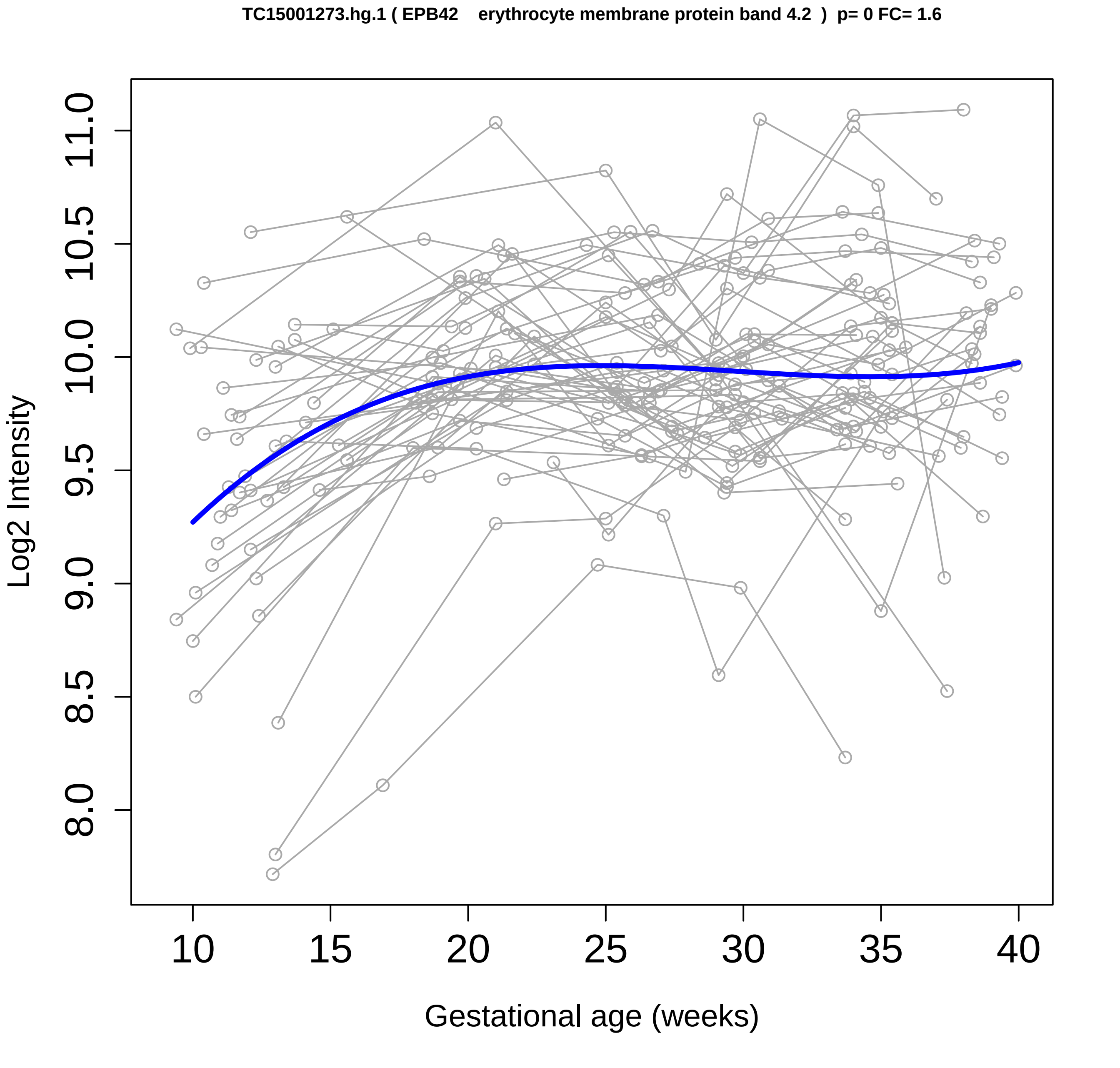
TC15001273.hg.1 (EPB42 erythrocyte membrane protein band 4.2) p= 0 FC= 1.6
Log2 Intensity
8.0
8.5
9.0
9.5
10.0
10.5
11.0
10
15
20
25
30
35
40
Gestational age (weeks)

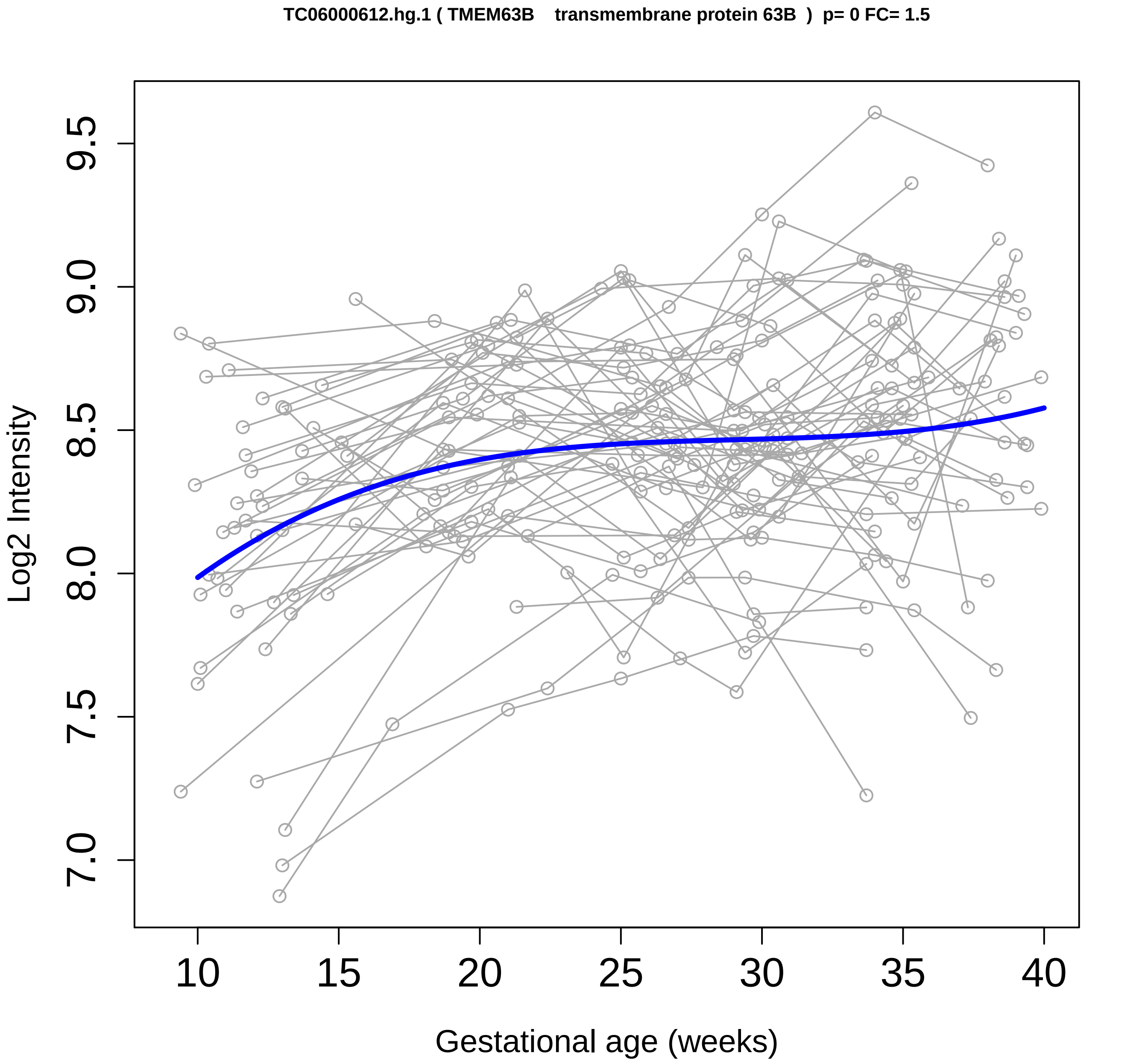

TC06000612.hg.1 (TMEM63B transmembrane protein 63B) p= 0 FC= 1.5
Log2 Intensity
7.0
7.5
8.0
8.5
9.0
9.5
10
15
20
25
30
35
40
Gestational age (weeks)

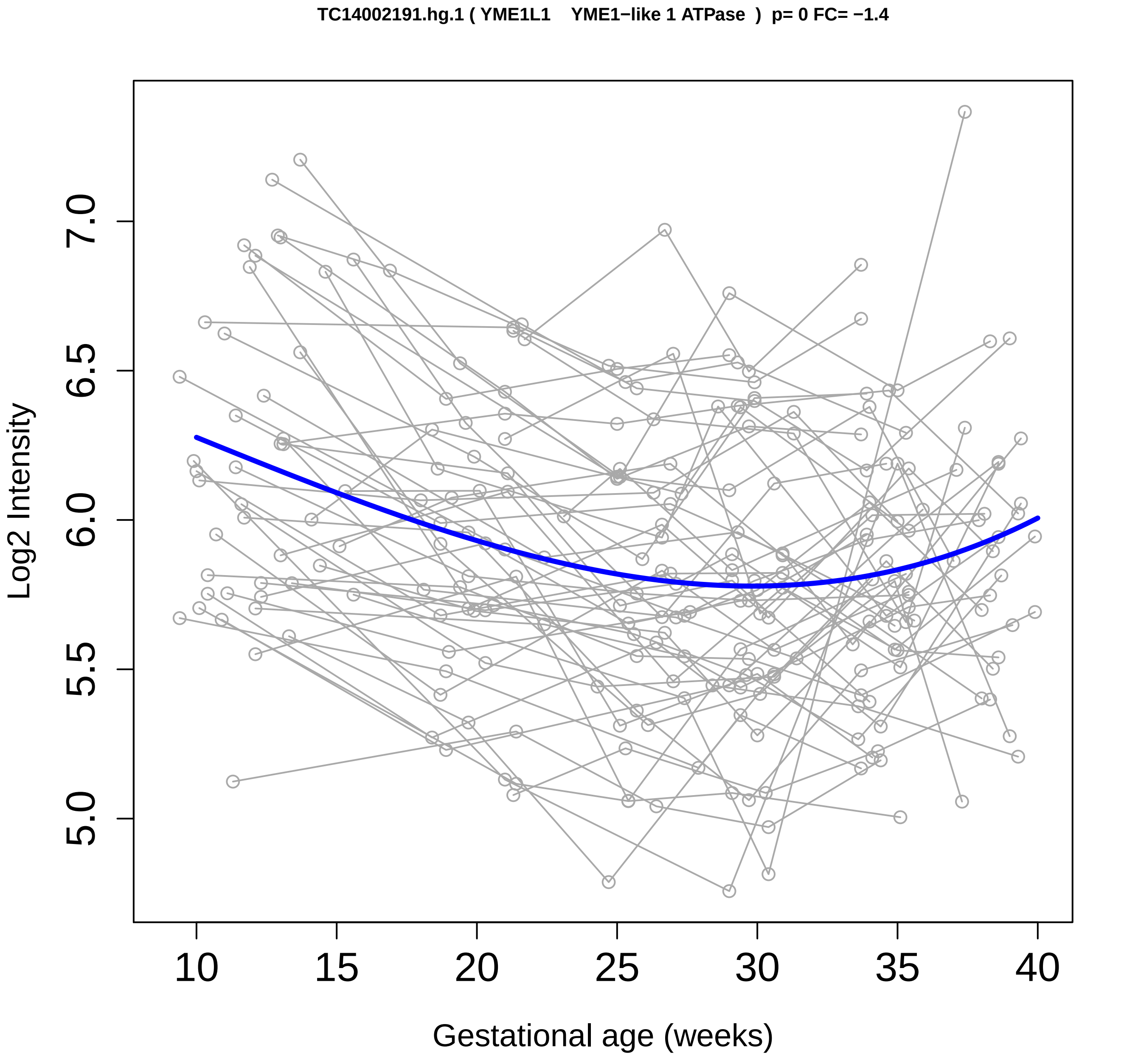
TC14002191.hg.1 (YME1L1 YME1−like 1 ATPase) p= 0 FC= −1.4
Log2 Intensity
7.0
6.5
6.0
5.5
5.0
10
15
20
25
30
35
40
Gestational age (weeks)

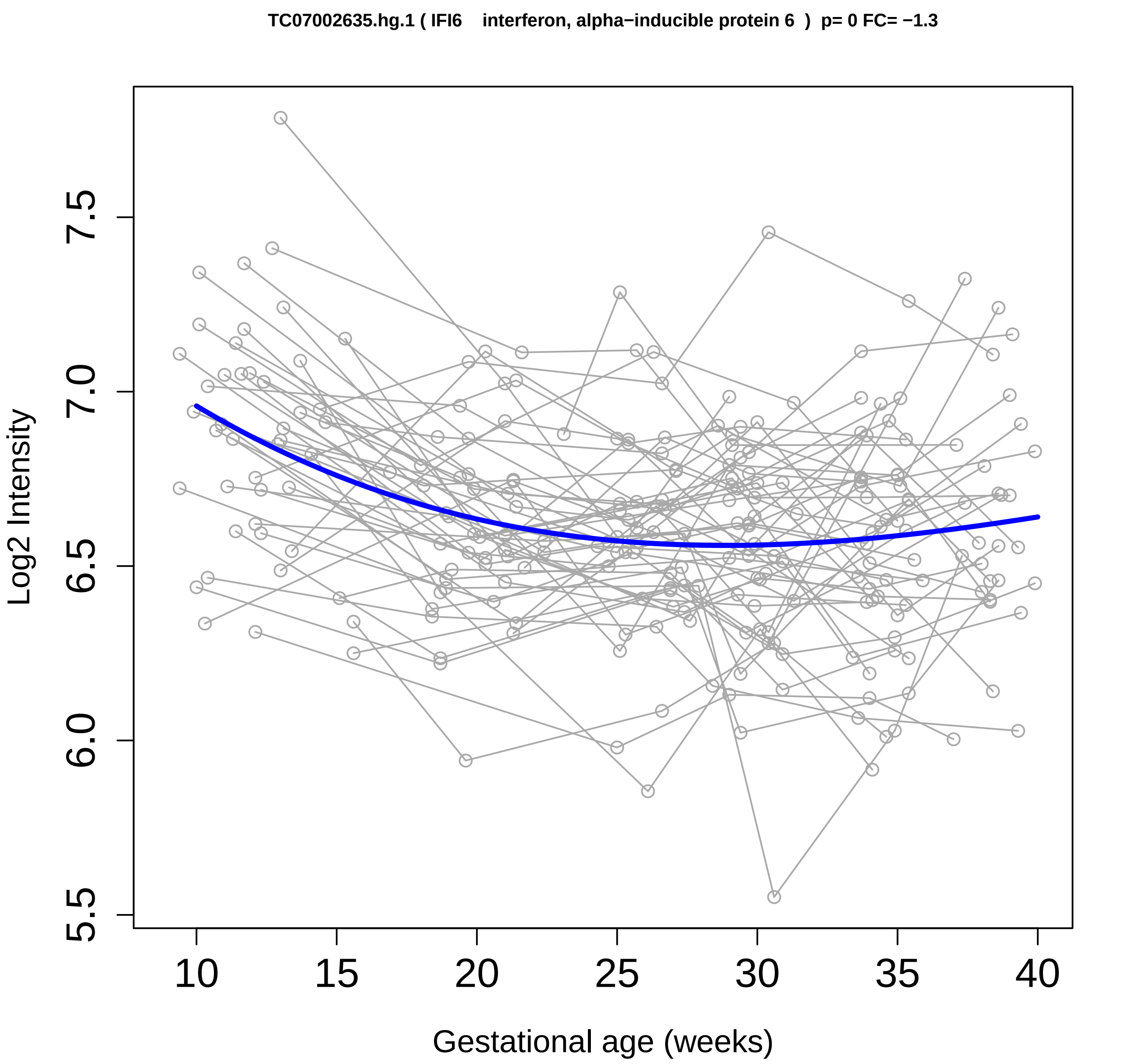

TC07002635.hg.1 (IFI6   interferon, alpha−inducible protein 6) p= 0 FC= −1.3
Log2 Intensity
5.5
6.0
6.5
7.0
7.5
10
15
20
25
30
35
40
Gestational age (weeks)

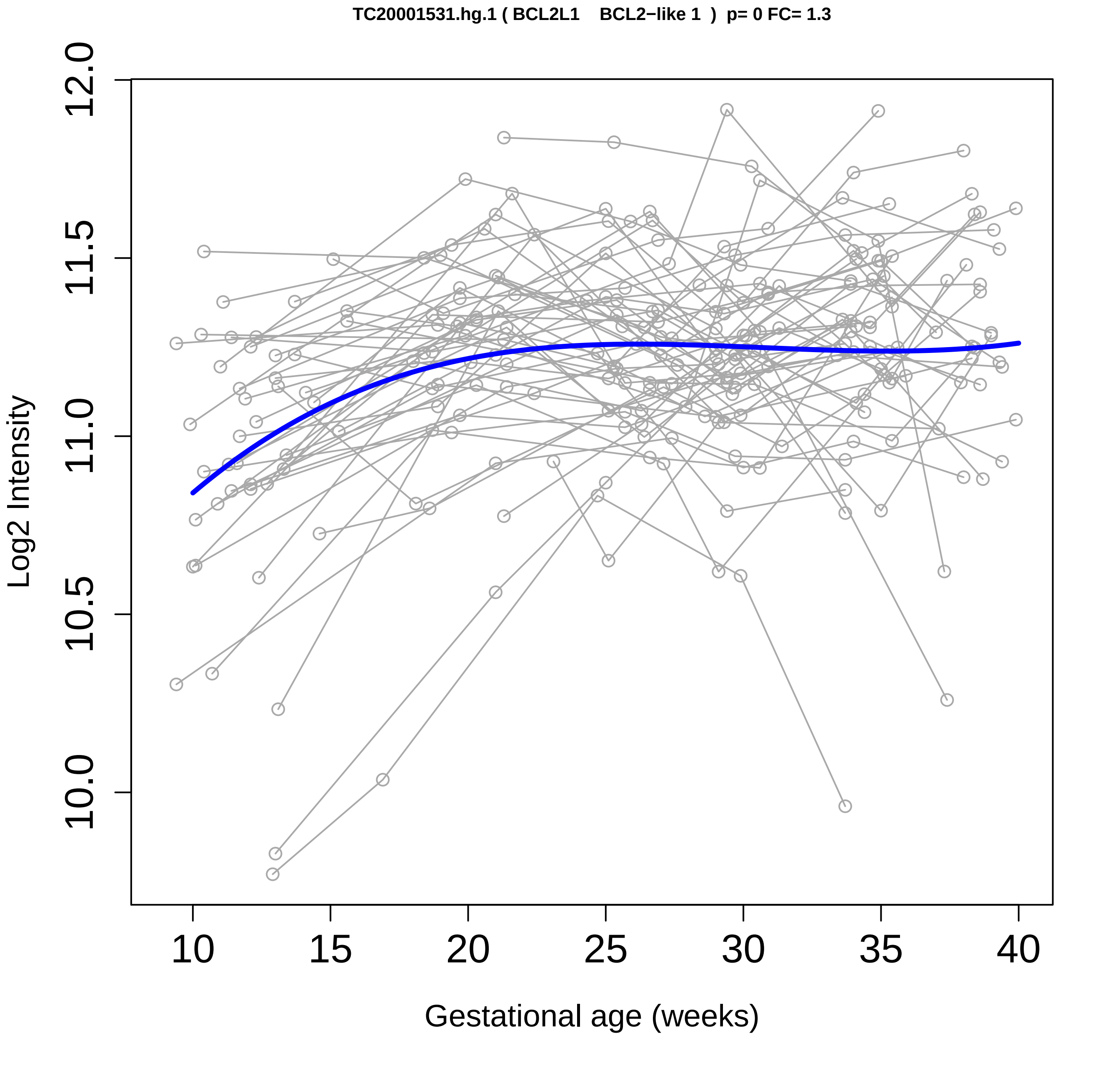

TC20001531.hg.1 (BCL2L1 BCL2−like 1) p= 0 FC= 1.3
Log2 Intensity
12.0
11.5
11.0
10.5
10.0
10
15
20
25
30
35
40
Gestational age (weeks)

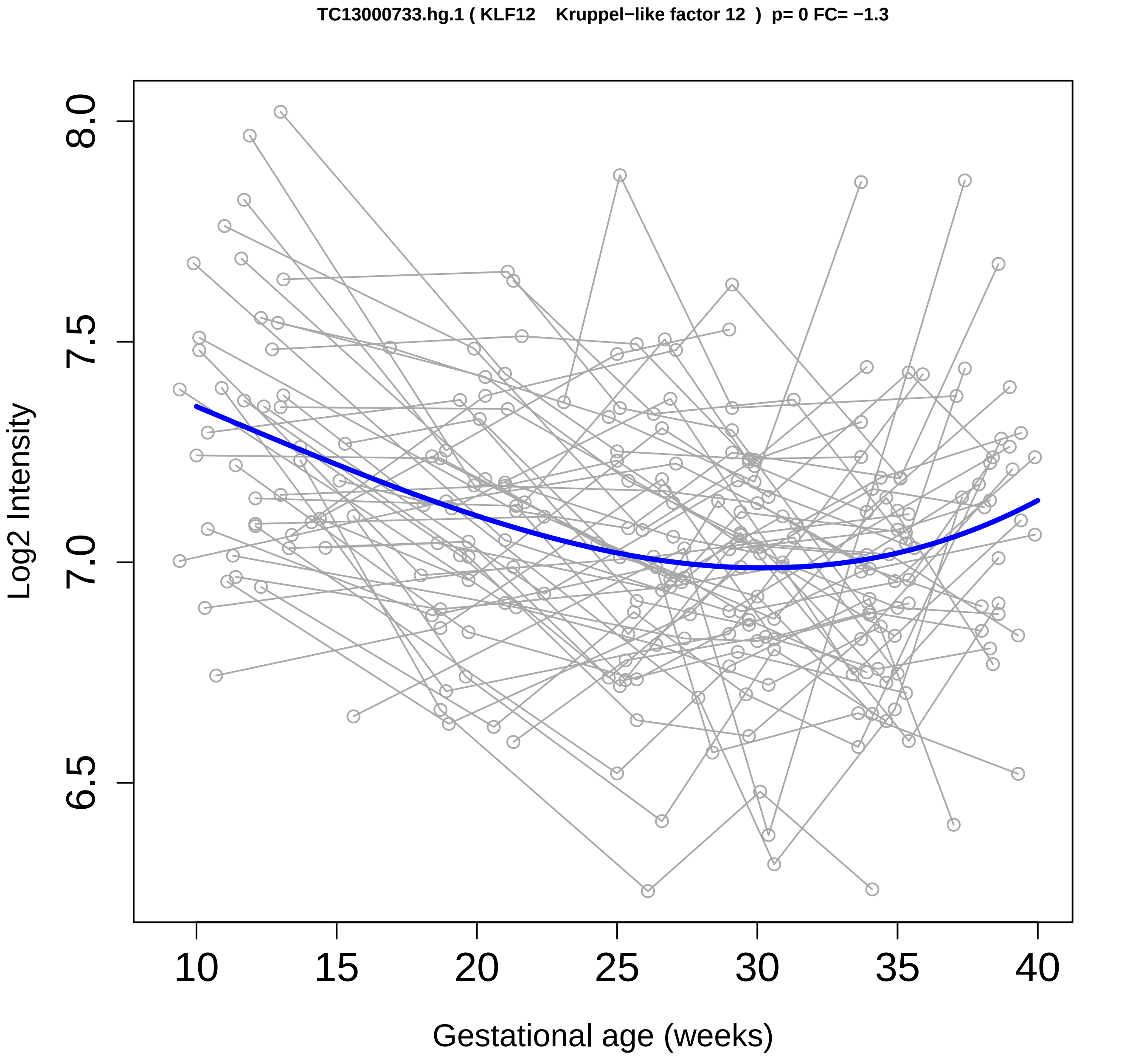

TC13000733.hg.1 (KLF12 Kruppel−like factor 12) p= 0 FC= −1.3
Log2 Intensity
8.0
7.5
7.0
6.5
10
15
20
25
30
35
40
Gestational age (weeks)

TC09001211.hg.1 (ALDH1A1 aldehyde dehydrogenase 1 family, member A1) p= 0 FC= 1.3

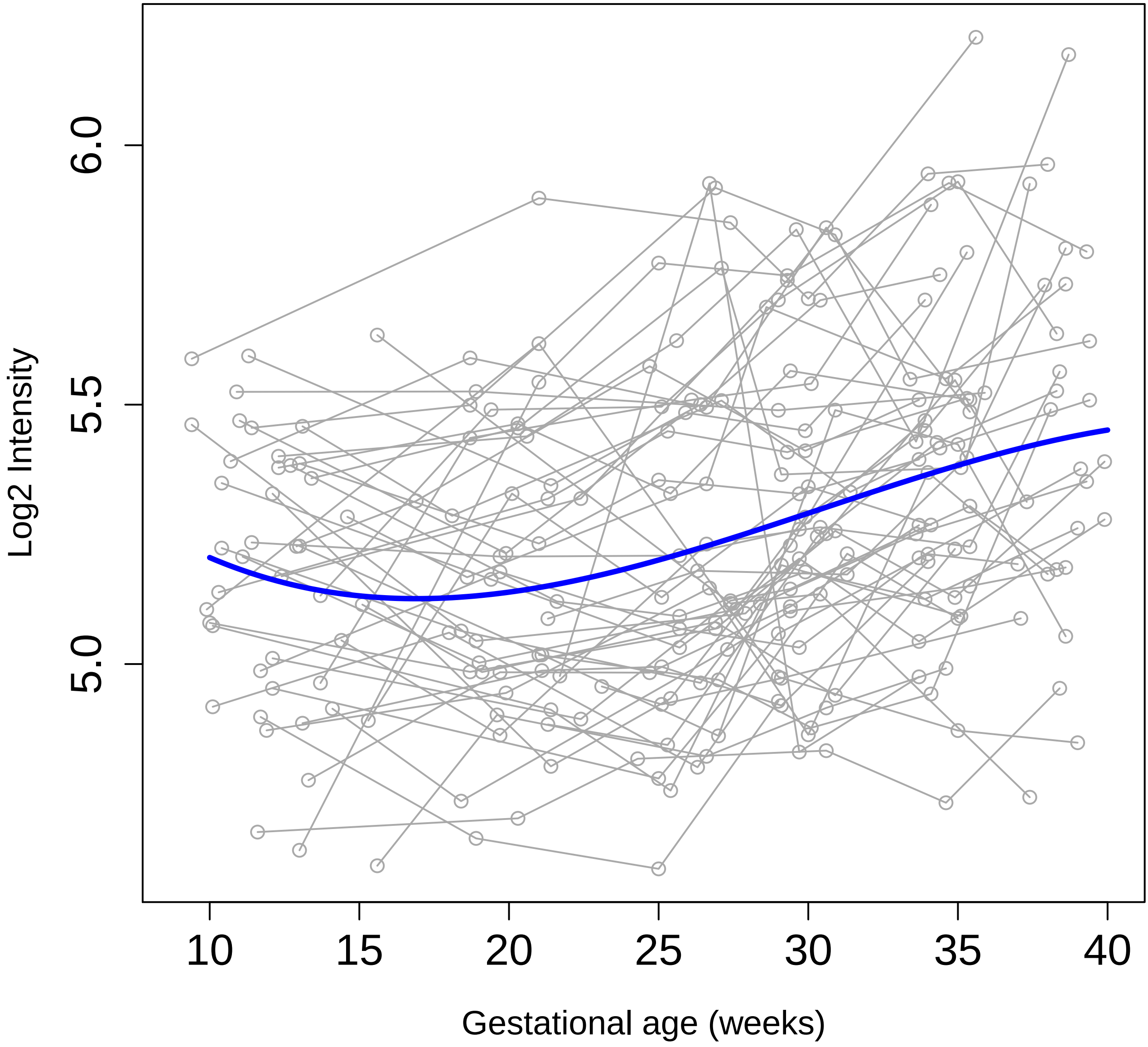

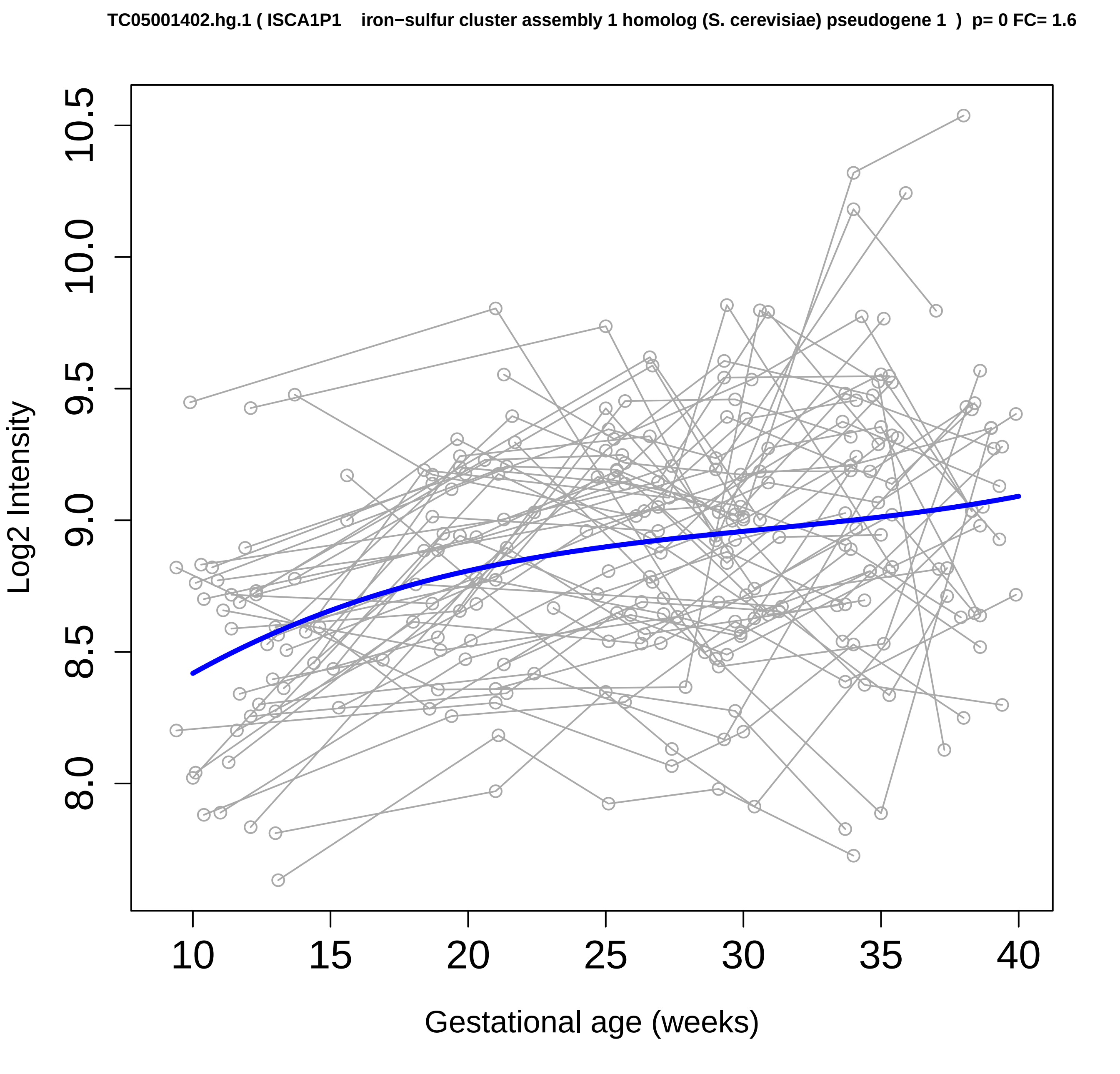
TC05001402.hg.1 (ISCA1P1 iron−sulfur cluster assembly 1 homolog (S. cerevisiae) pseudogene 1) p= 0 FC= 1.6
Log2 Intensity
8.0
8.5
9.0
9.5
10.0
10.5
10
15
20
25
30
35
40
Gestational age (weeks)

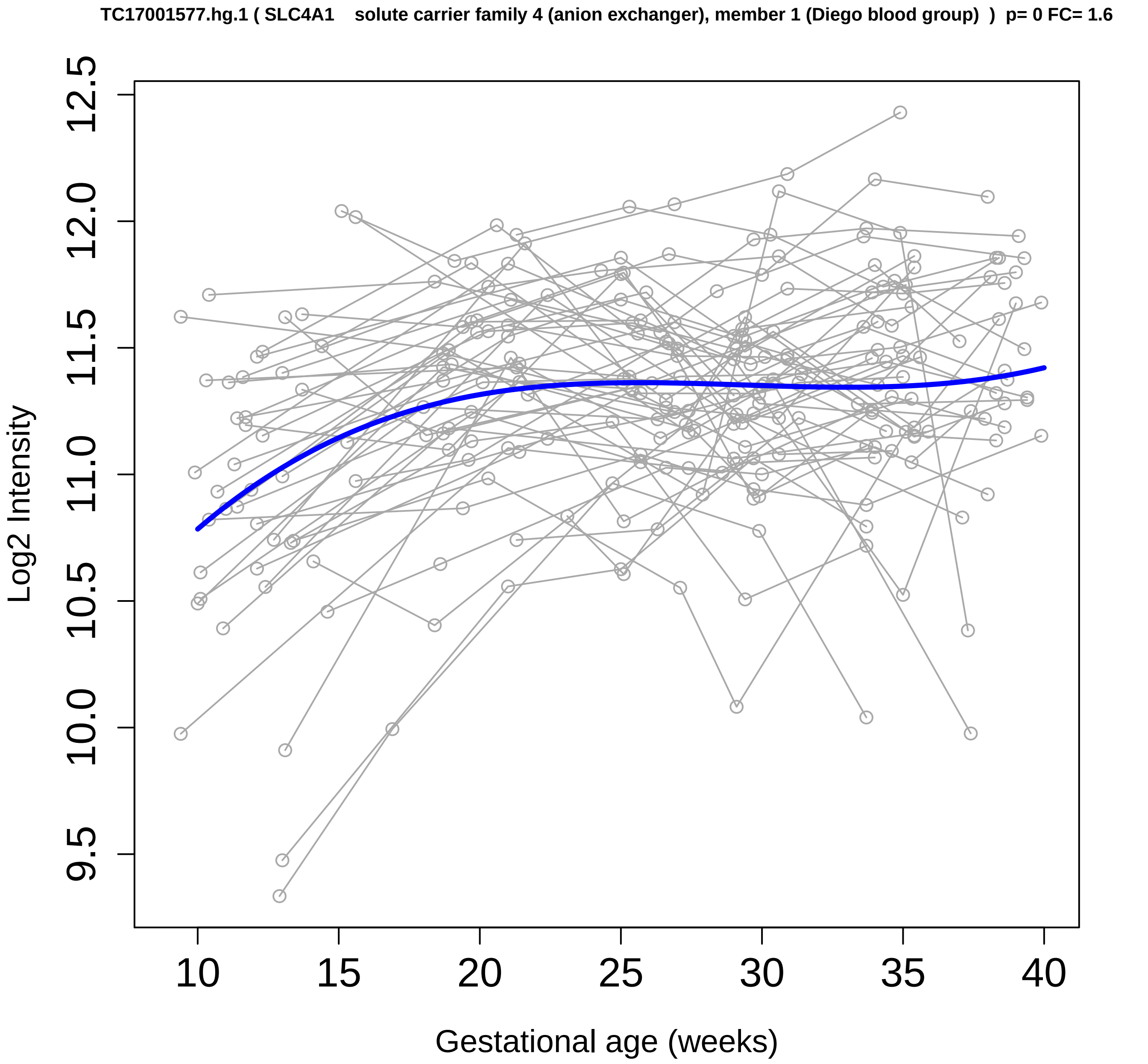

TC17001577.hg.1 (SLC4A1 solute carrier family 4 (anion exchanger), member 1 (Diego blood group)) p= 0 FC= 1.6
Log2 Intensity
9.5
10.0
10.5
11.0
11.5
12.0
12.5
10
15
20
25
30
35
40
Gestational age (weeks)

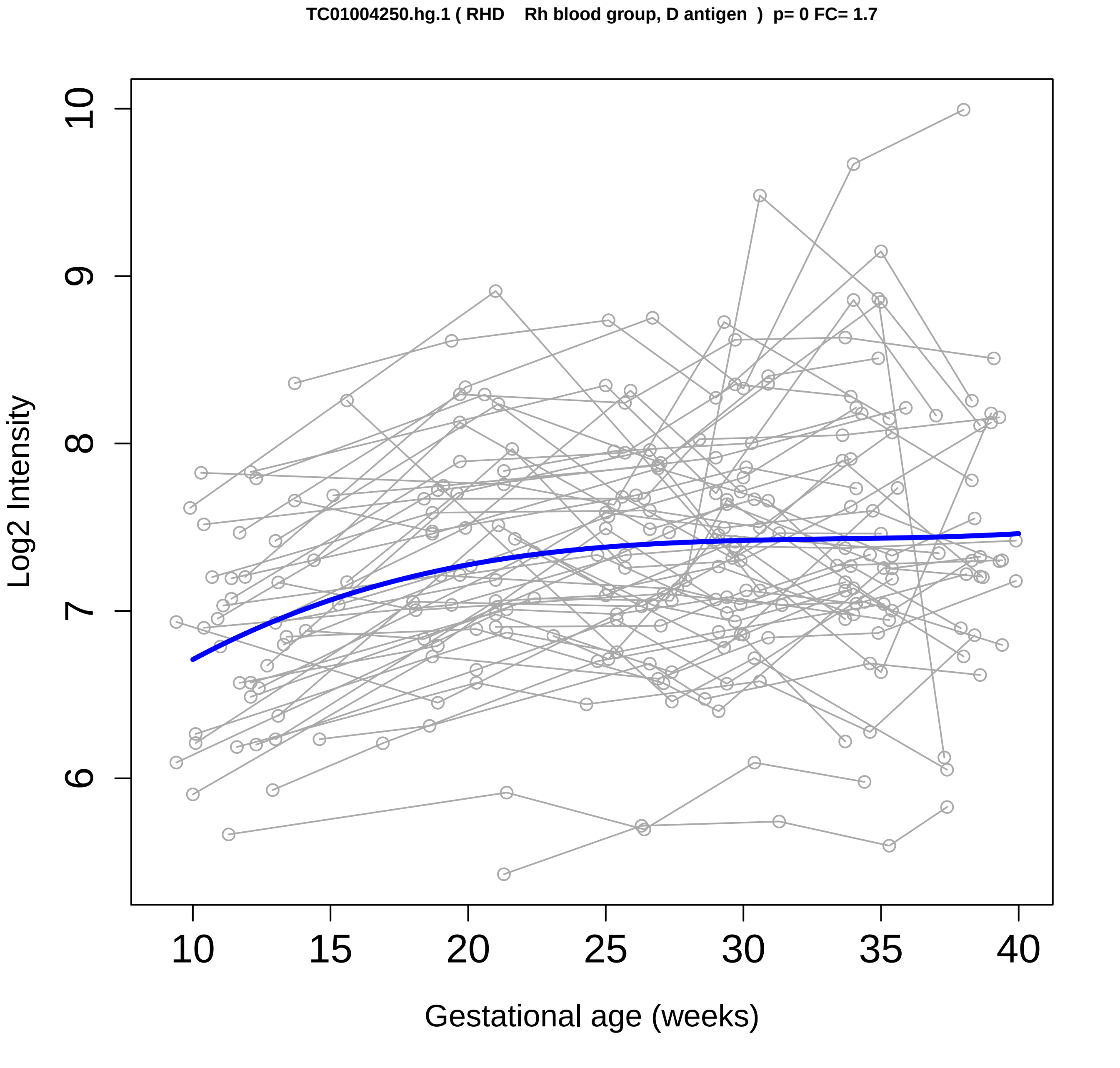

TC01004250.hg.1 (RHD Rh blood group, D antigen) p= 0 FC= 1.7
Log2 Intensity
6
7
8
9
10
10
15
20
25
30
35
40
Gestational age (weeks)

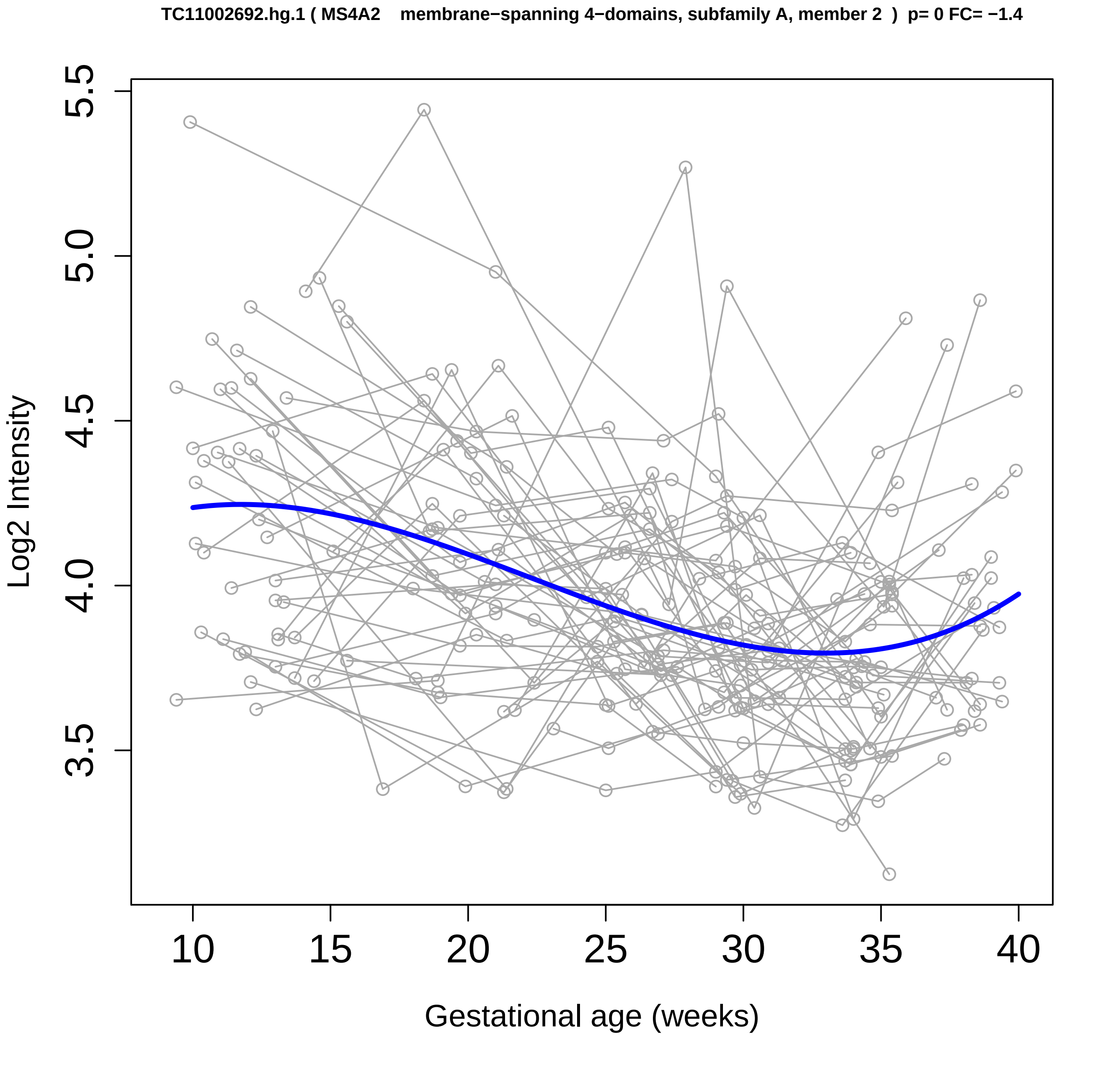
TC11002692.hg.1 (MS4A2 membrane−spanning 4−domains, subfamily A, member 2) p= 0 FC= −1.4
Log2 Intensity
5.5
5.0
4.5
4.0
3.5
10
15
20
25
30
35
40
Gestational age (weeks)

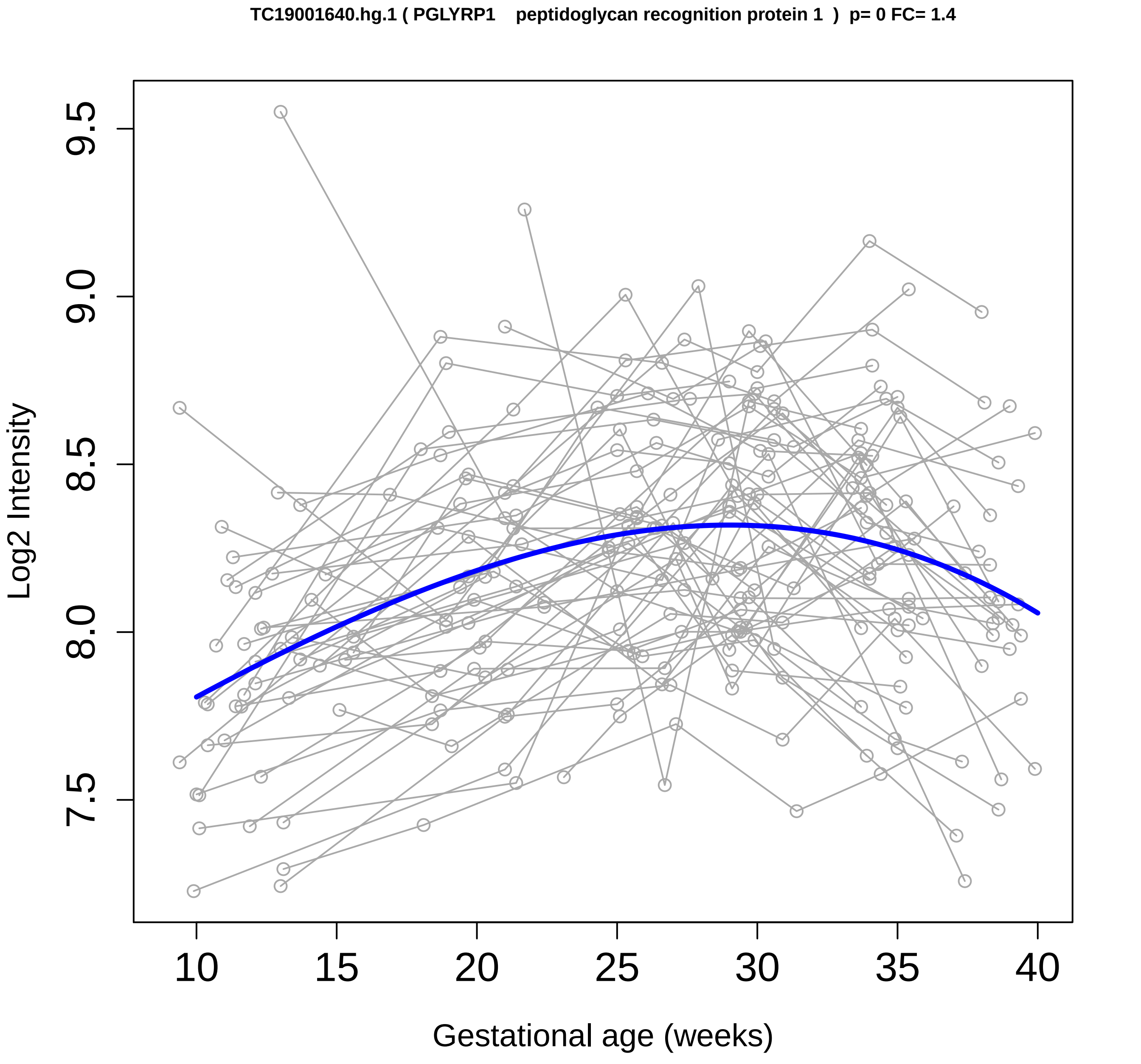

TC19001640.hg.1 (PGLYRP1 peptidoglycan recognition protein 1) p= 0 FC= 1.4
Log2 Intensity
7.5
8.0
8.5
9.0
9.5
10
15
20
25
30
35
40
Gestational age (weeks)

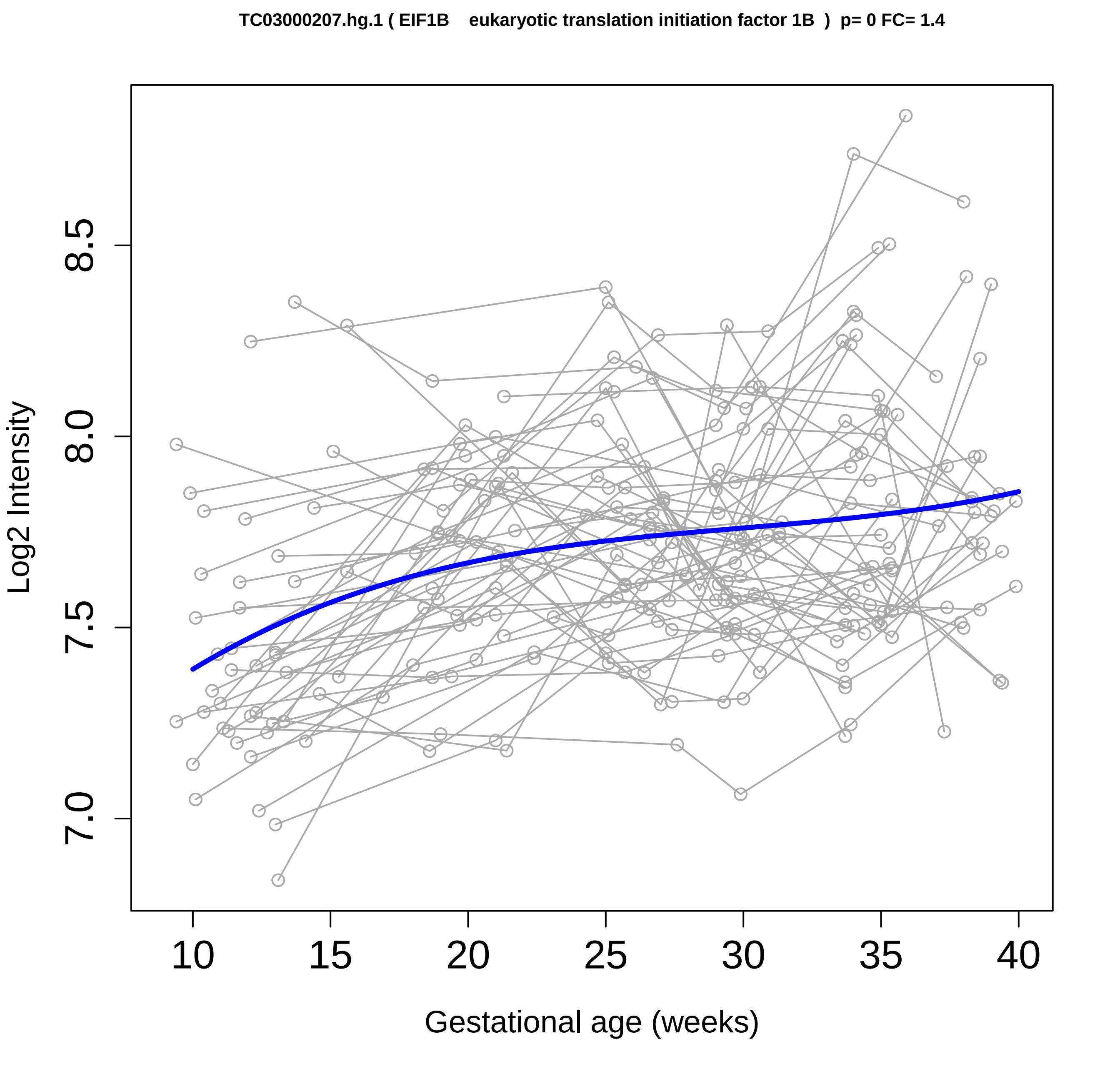

TC03000207.hg.1 (EIF1B eukaryotic translation initiation factor 1B) p= 0 FC= 1.4
Log2 Intensity
7.0
7.5
8.0
8.5
10
15
20
25
30
35
40
Gestational age (weeks)

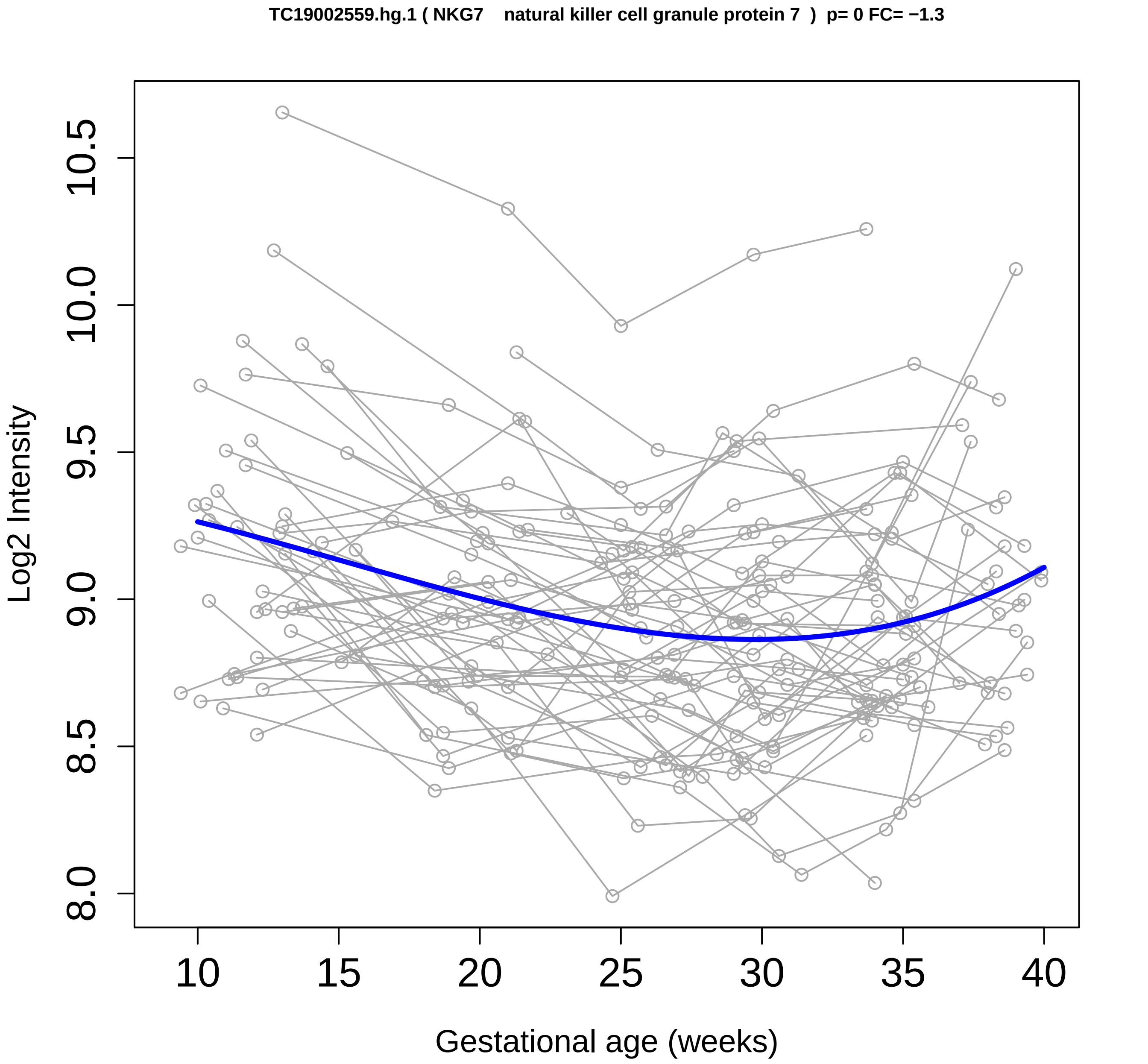

TC19002559.hg.1 (NKG7 natural killer cell granule protein 7) p= 0 FC= −1.3
Log2 Intensity
8.0
8.5
9.0
9.5
10.0
10.5
10
15
20
25
30
35
40
Gestational age (weeks)

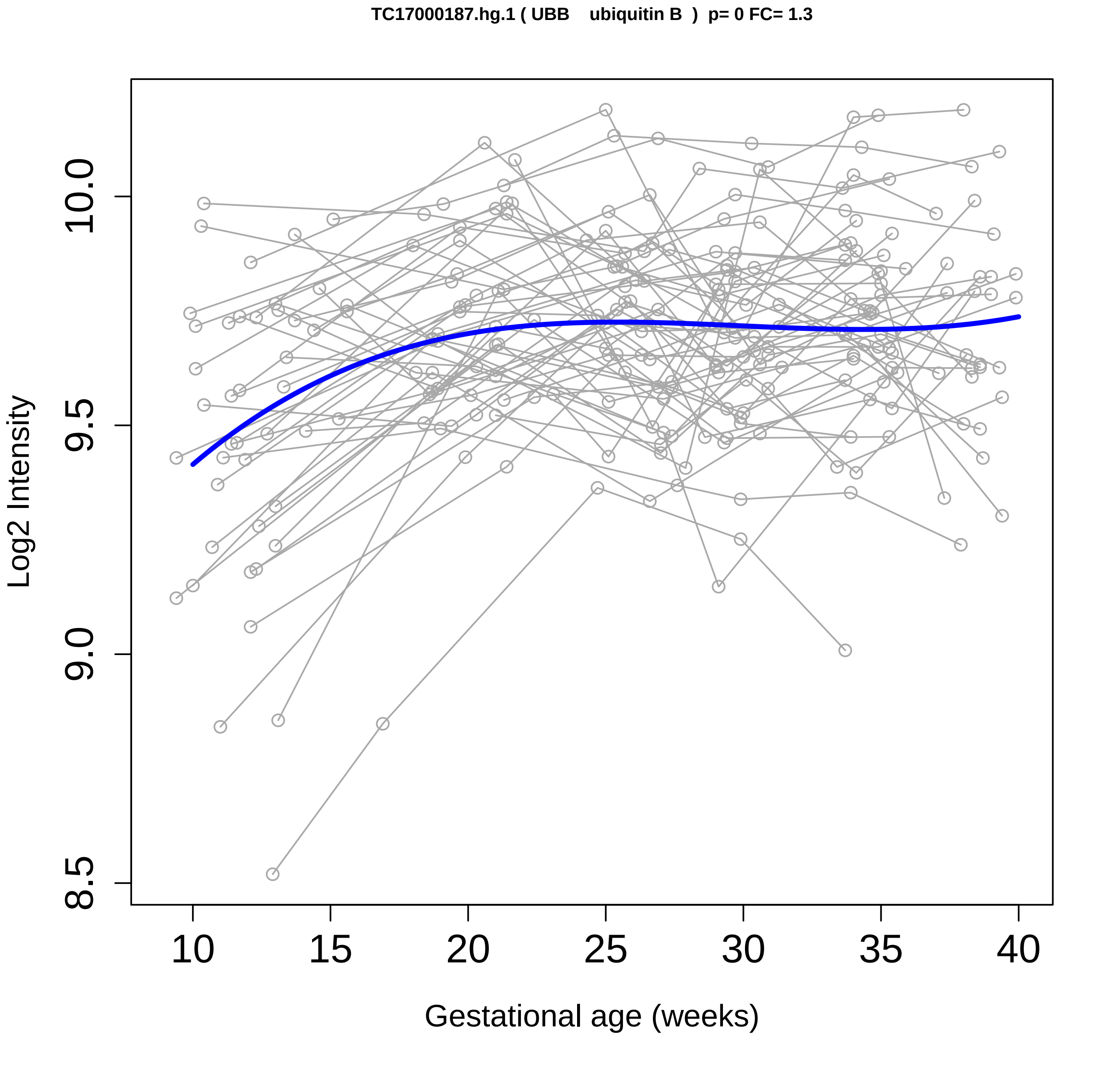
TC17000187.hg.1 (UBB ubiquitin B) p= 0 FC= 1.3
Log2 Intensity
10.0
9.5
9.0
8.5
10
15
20
25
30
35
40
Gestational age (weeks)

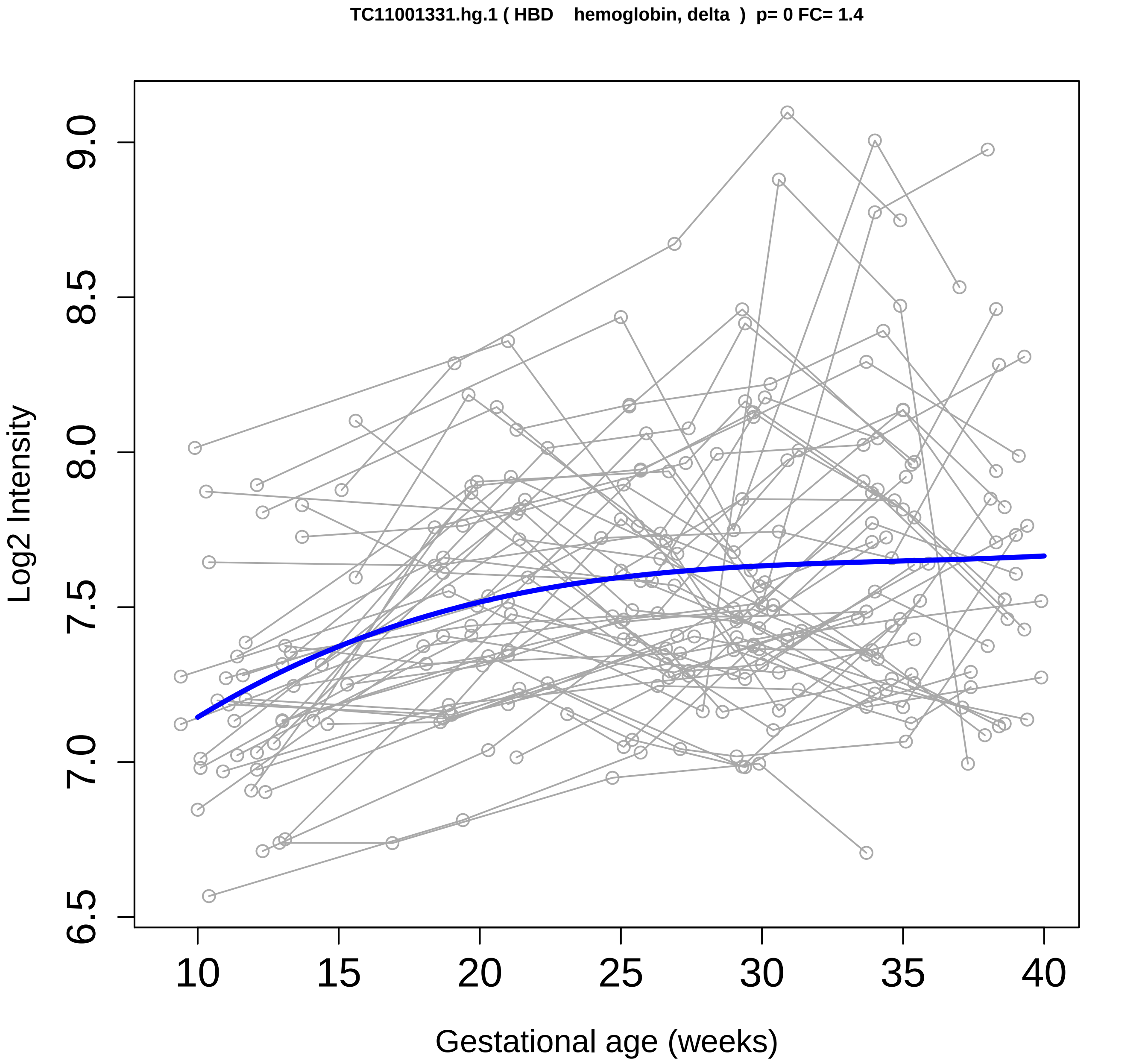
TC11001331.hg.1 (HBD hemoglobin, delta) p= 0 FC= 1.4
Log2 Intensity
6.5
7.0
7.5
8.0
8.5
9.0
10
15
20
25
30
35
40
Gestational age (weeks)

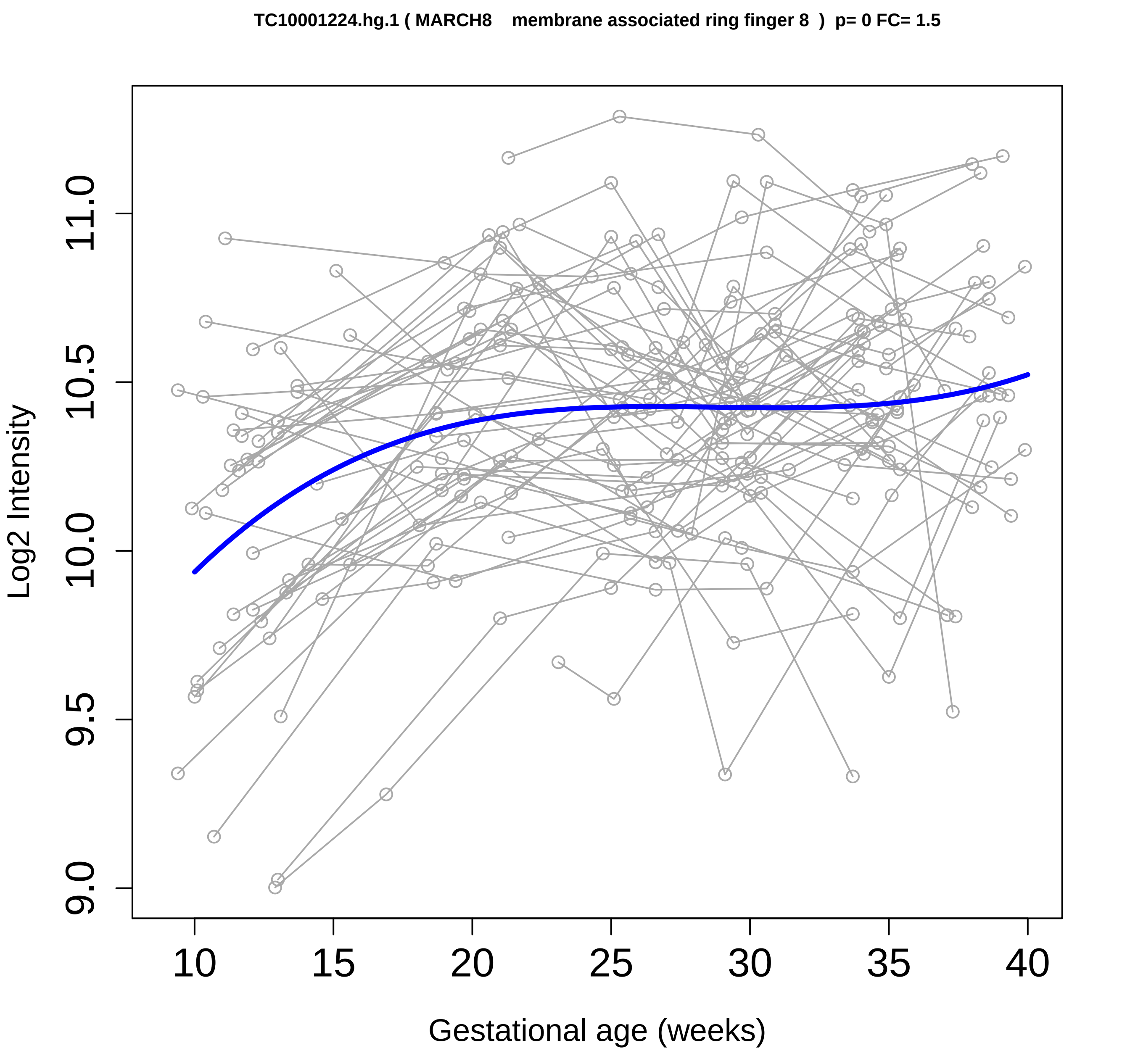
TC10001224.hg.1 (MARCH8 membrane associated ring finger 8) p= 0 FC= 1.5
Log2 Intensity
9.0
9.5
10.0
10.5
11.0
10
15
20
25
30
35
40
Gestational age (weeks)

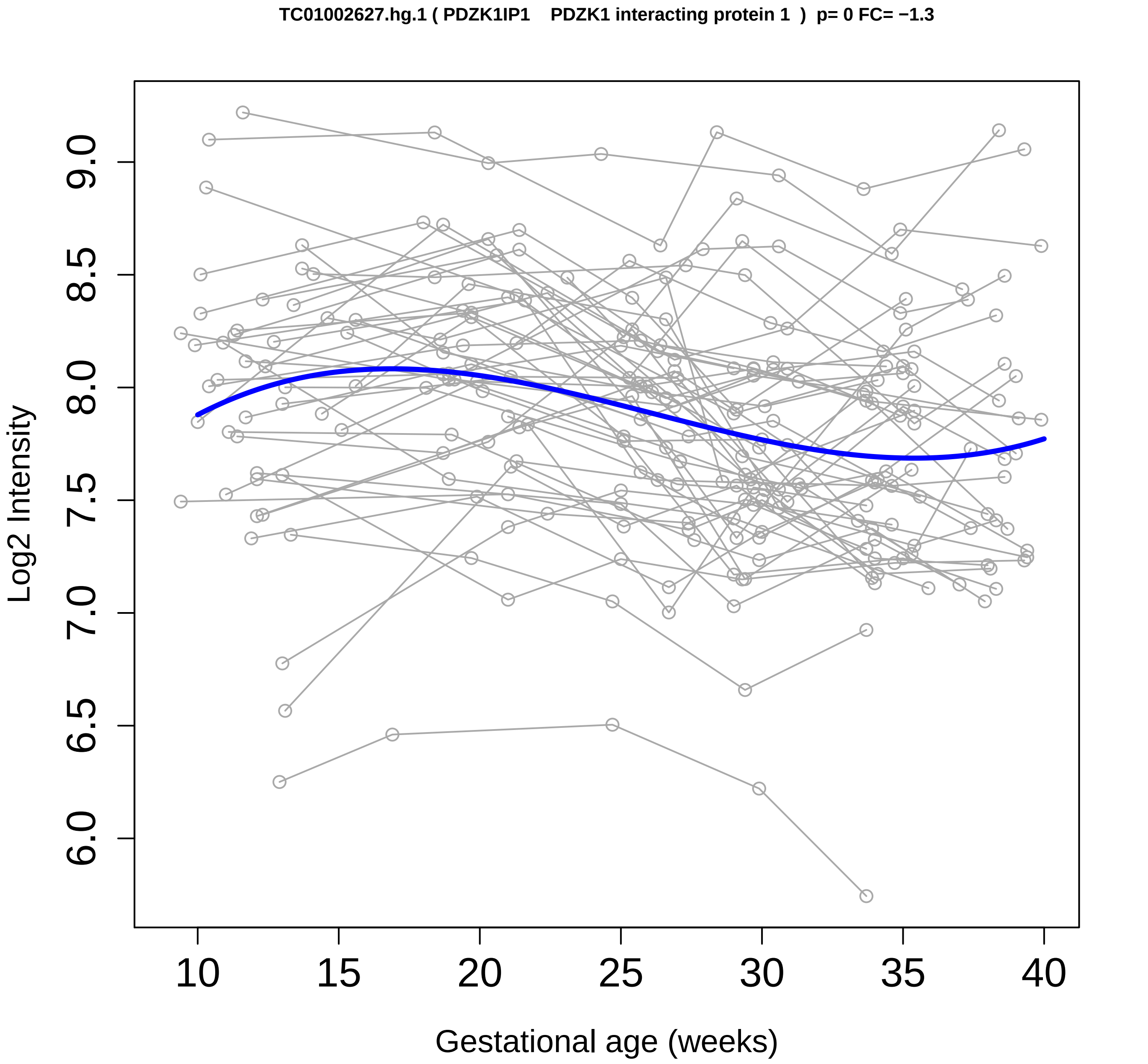

TC01002627.hg.1 (PDZK1IP1 PDZK1 interacting protein 1) p= 0 FC= −1.3
Log2 Intensity
9.0
8.5
8.0
7.5
7.0
6.5
6.0
10
15
20
25
30
35
40
Gestational age (weeks)

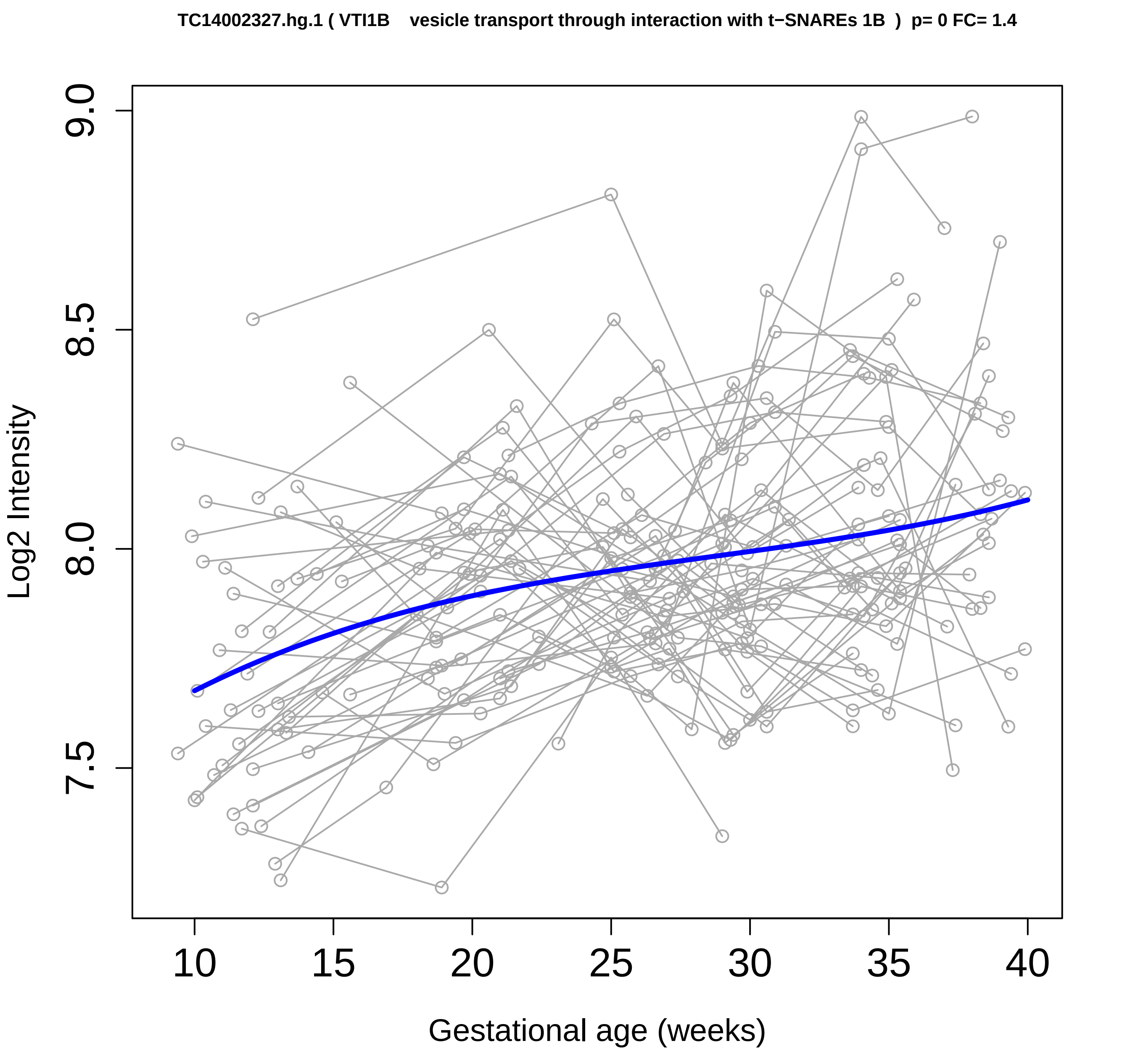

TC14002327.hg.1 (VTI1B vesicle transport through interaction with t−SNAREs 1B) p= 0 FC= 1.4
Log2 Intensity
9.0
8.5
8.0
7.5
10
15
20
25
30
35
40
Gestational age (weeks)

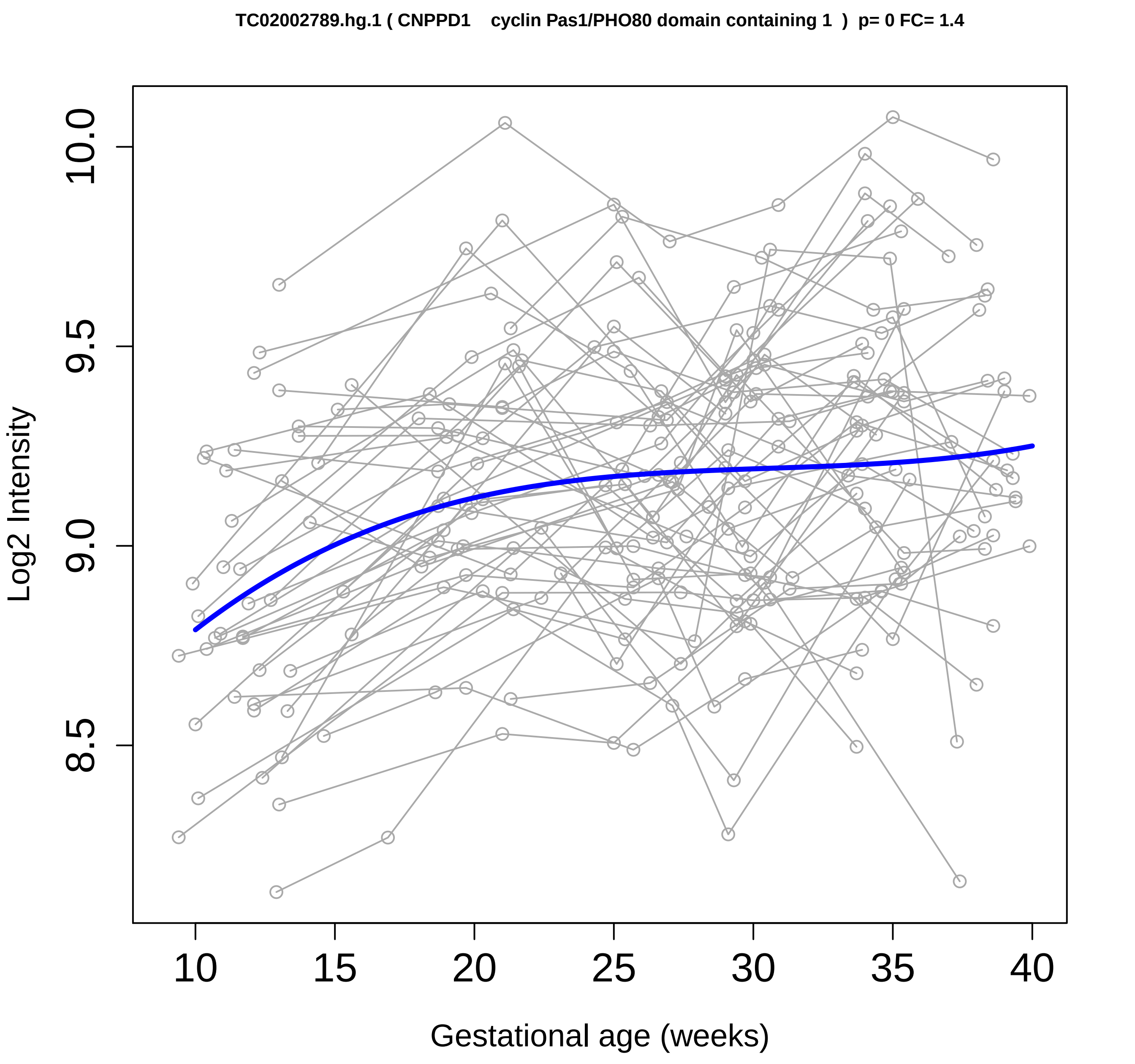

TC02002789.hg.1 (CNPPD1 cyclin Pas1/PHO80 domain containing 1) p= 0 FC= 1.4
Log2 Intensity
10.0
9.5
9.0
8.5
10
15
20
25
30
35
40
Gestational age (weeks)

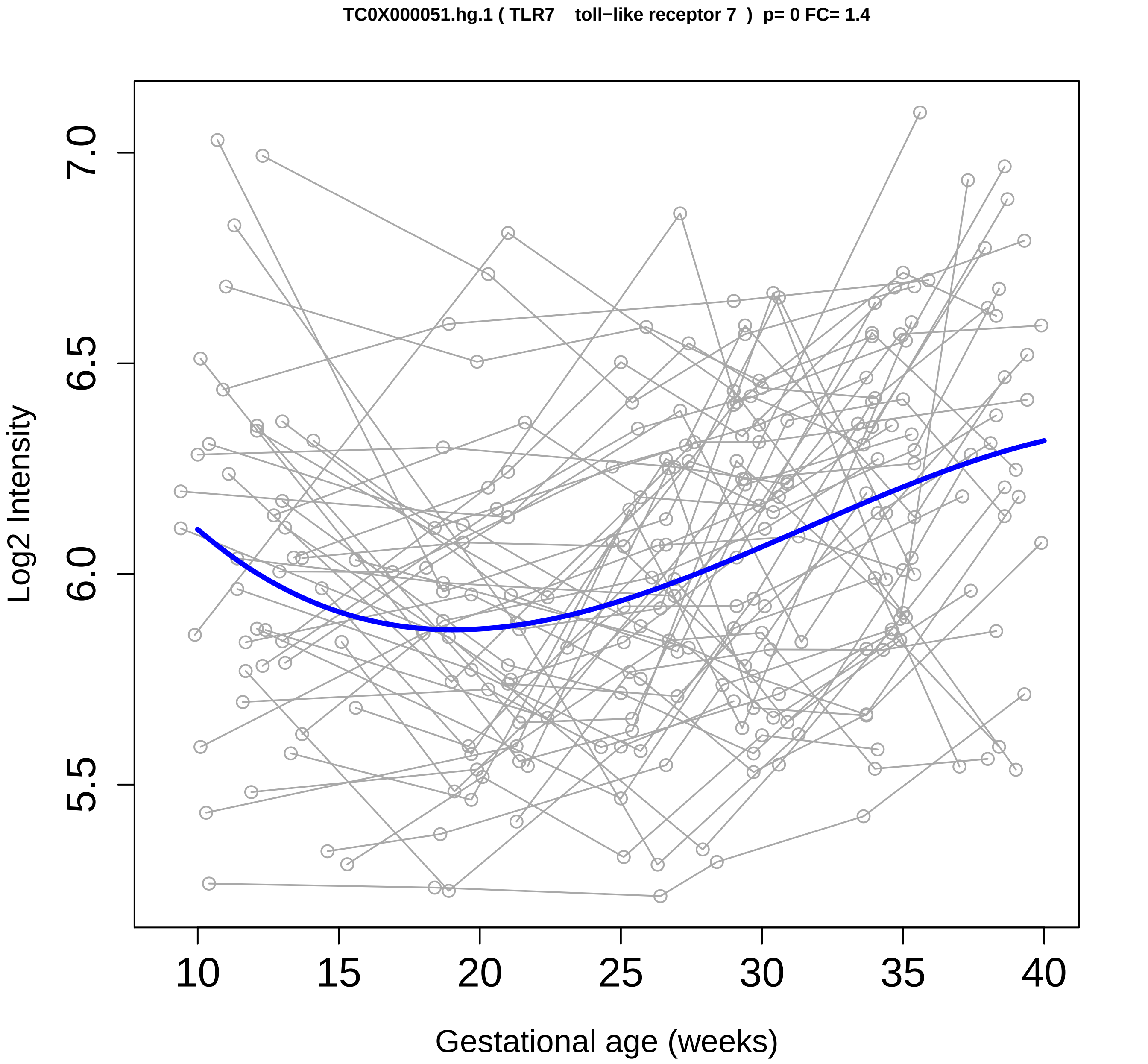

TC0X000051.hg.1 (TLR7 toll−like receptor 7) p= 0 FC= 1.4
Log2 Intensity
7.0
6.5
6.0
5.5
10
15
20
25
30
35
40
Gestational age (weeks)

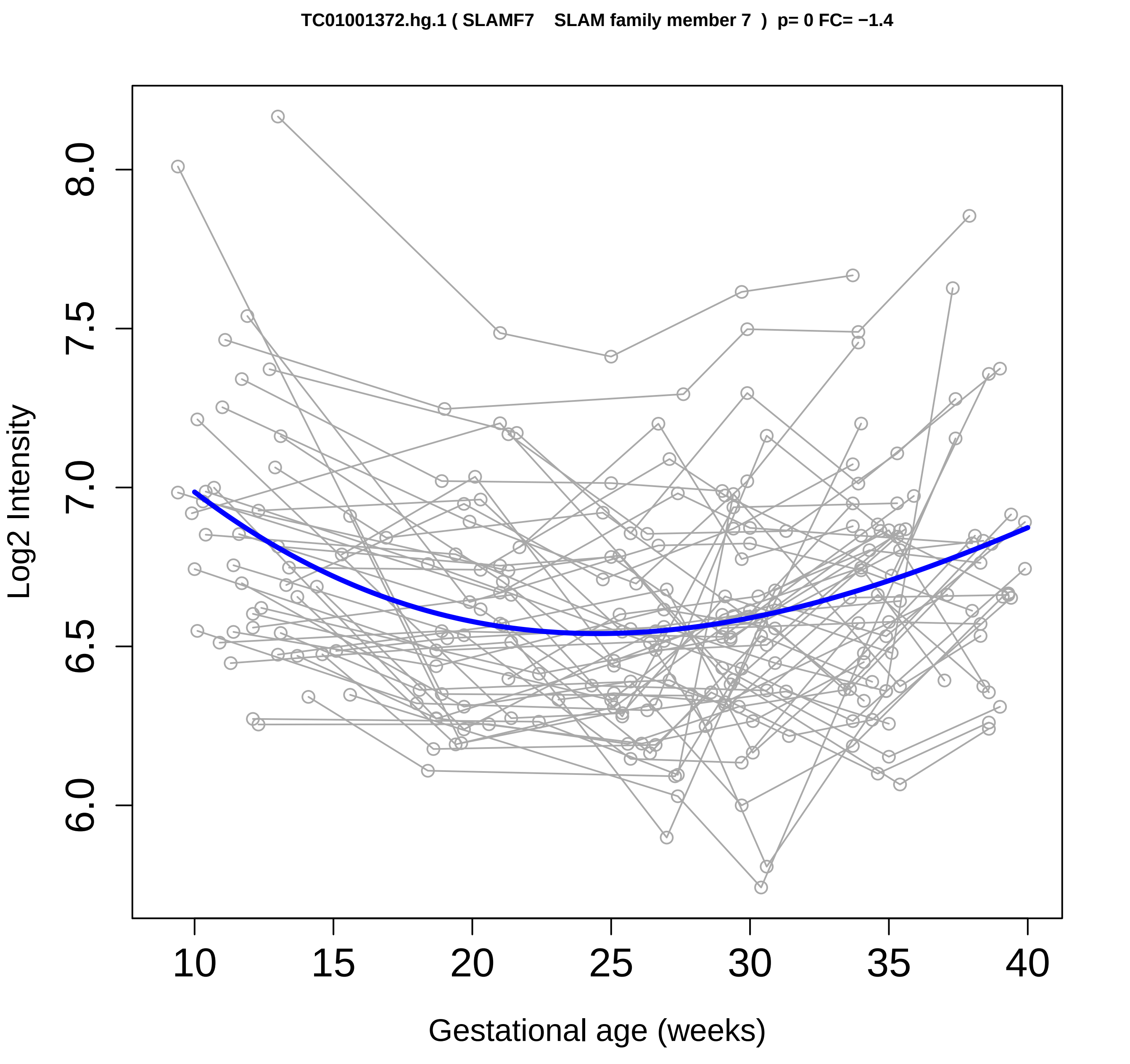

TC01001372.hg.1 (SLAMF7 SLAM family member 7) p= 0 FC= −1.4
Log2 Intensity
6.0
6.5
7.0
7.5
8.0
10
15
20
25
30
35
40
Gestational age (weeks)

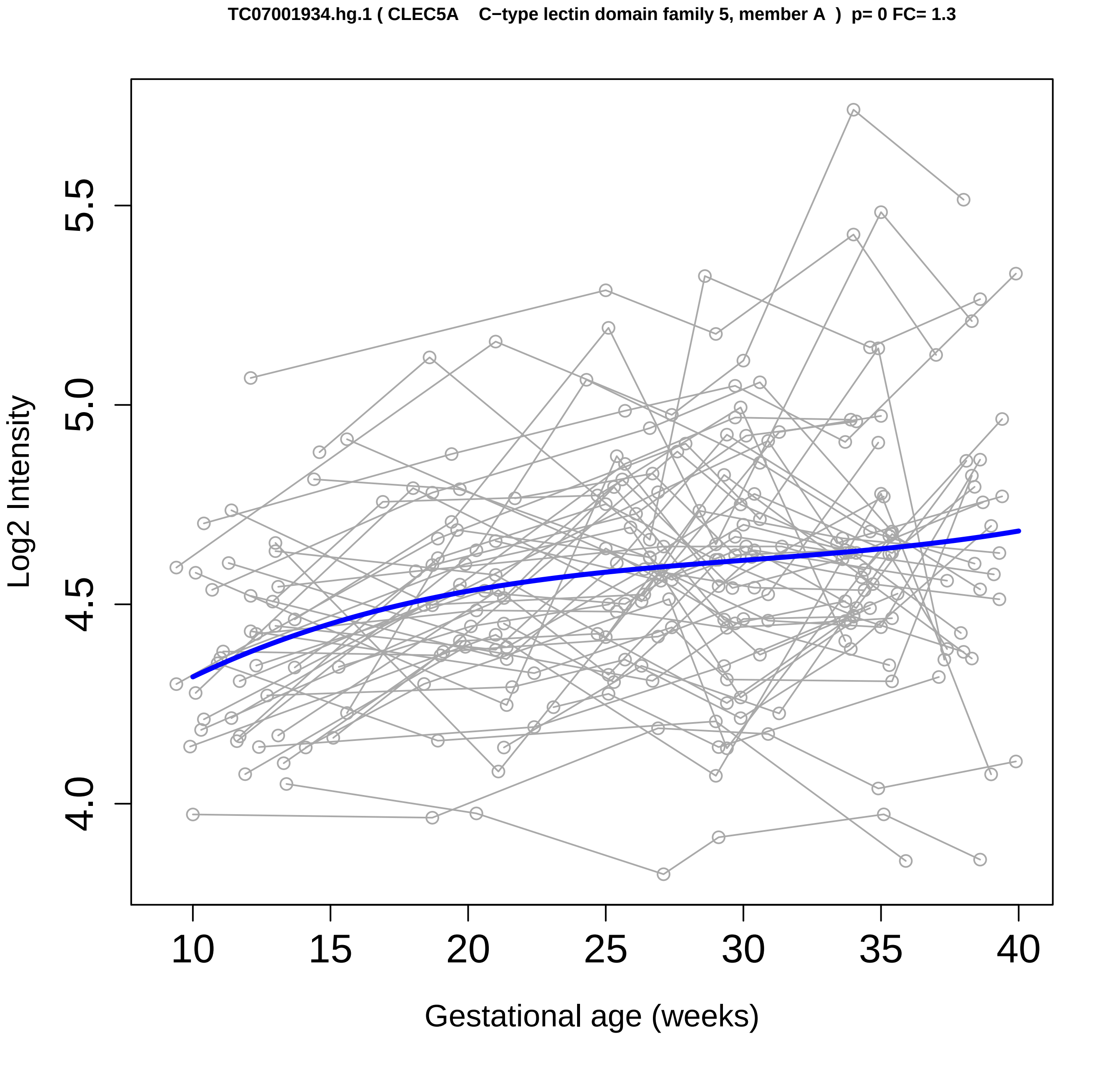

TC07001934.hg.1 (CLEC5A   C−type lectin domain family 5, member A) p= 0 FC= 1.3
Log2 Intensity
4.0
4.5
5.0
5.5
10
15
20
25
30
35
40
Gestational age (weeks)

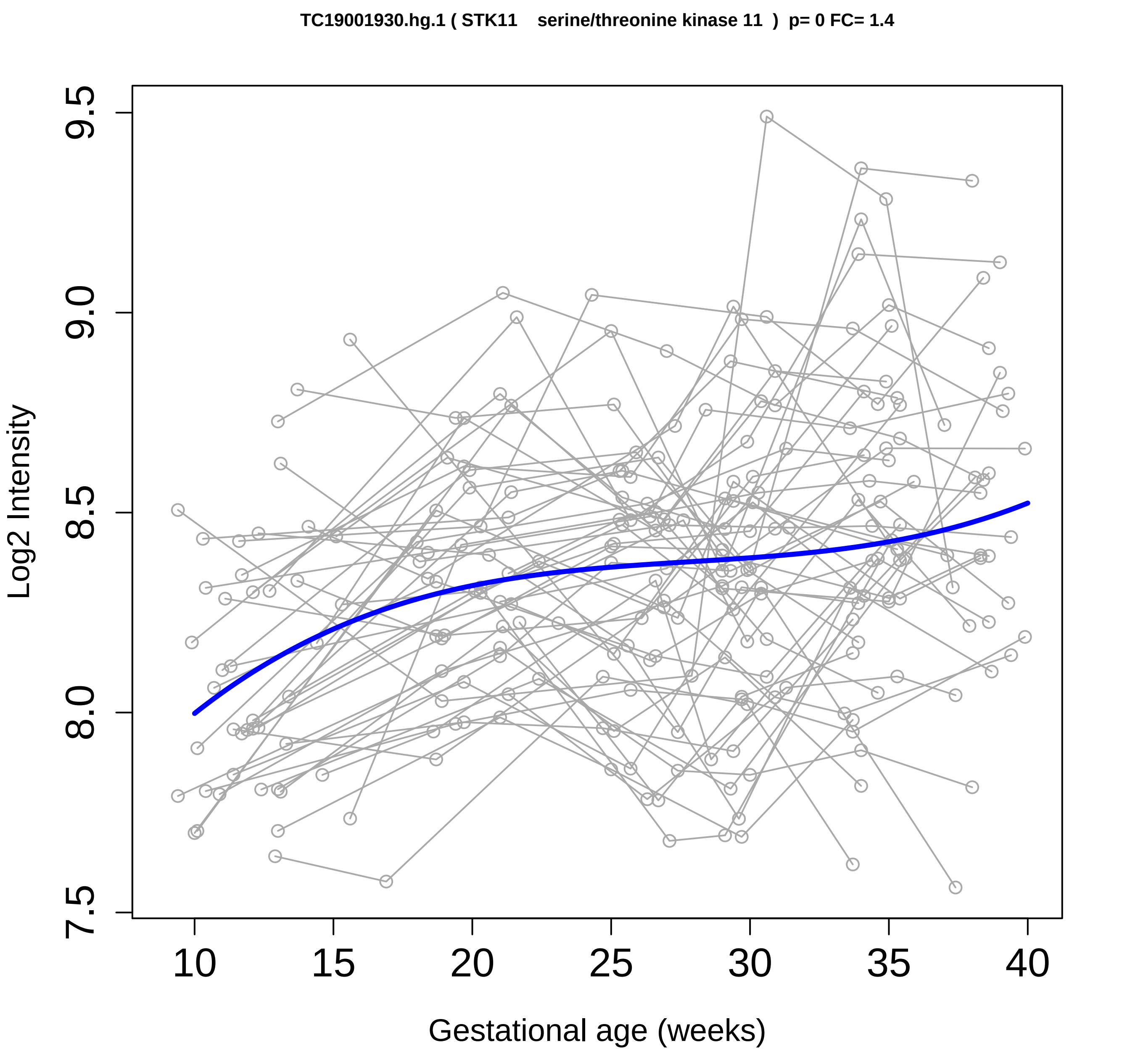

TC19001930.hg.1 (STK11 serine/threonine kinase 11) p= 0 FC= 1.4
Log2 Intensity
9.5
9.0
8.5
8.0
7.5
10
15
20
25
30
35
40
Gestational age (weeks)

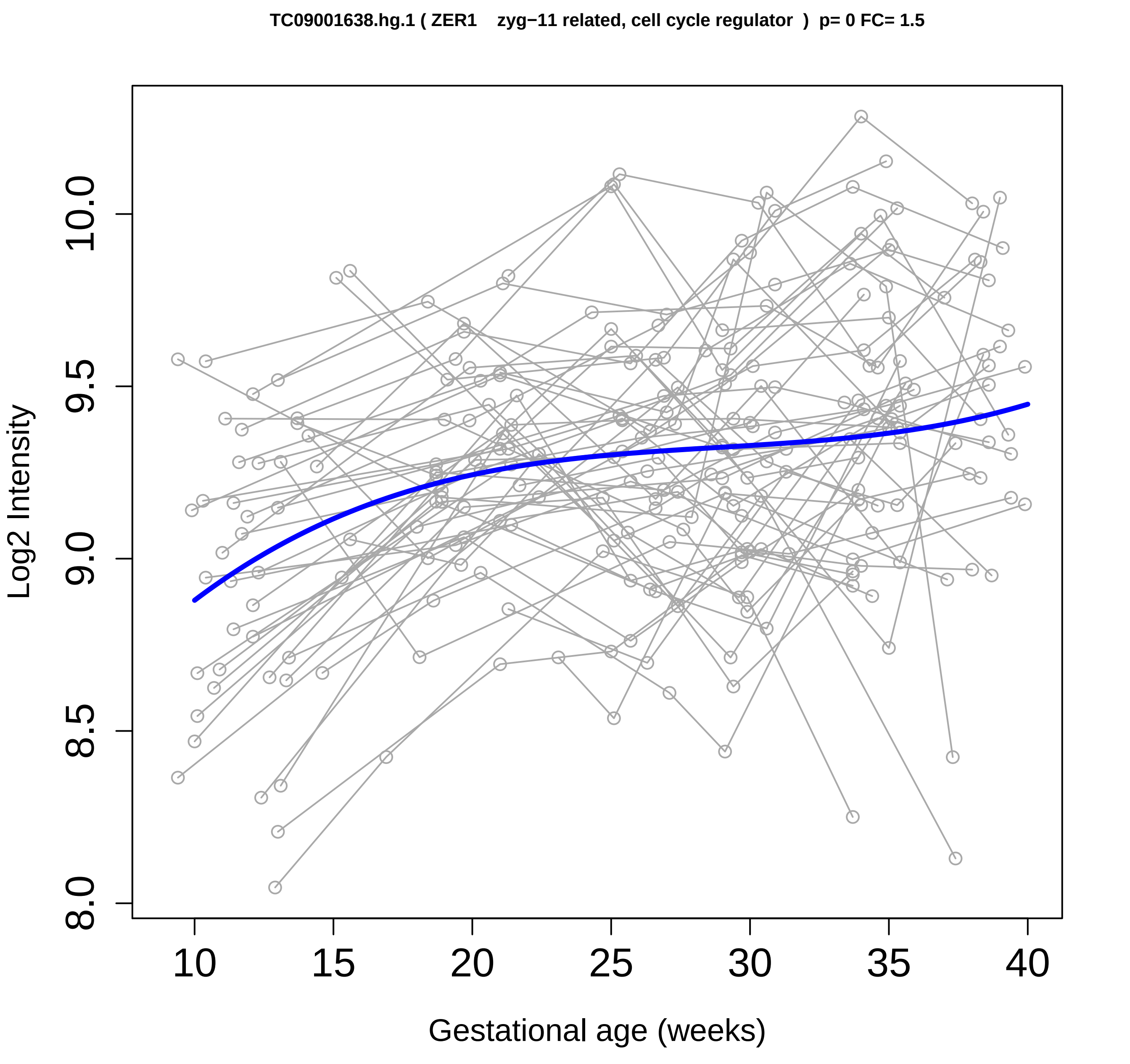

TC09001638.hg.1 (ZER1 zyg−11 related, cell cycle regulator) p= 0 FC= 1.5
Log2 Intensity
10.0
9.5
9.0
8.5
8.0
10
15
20
25
30
35
40
Gestational age (weeks)

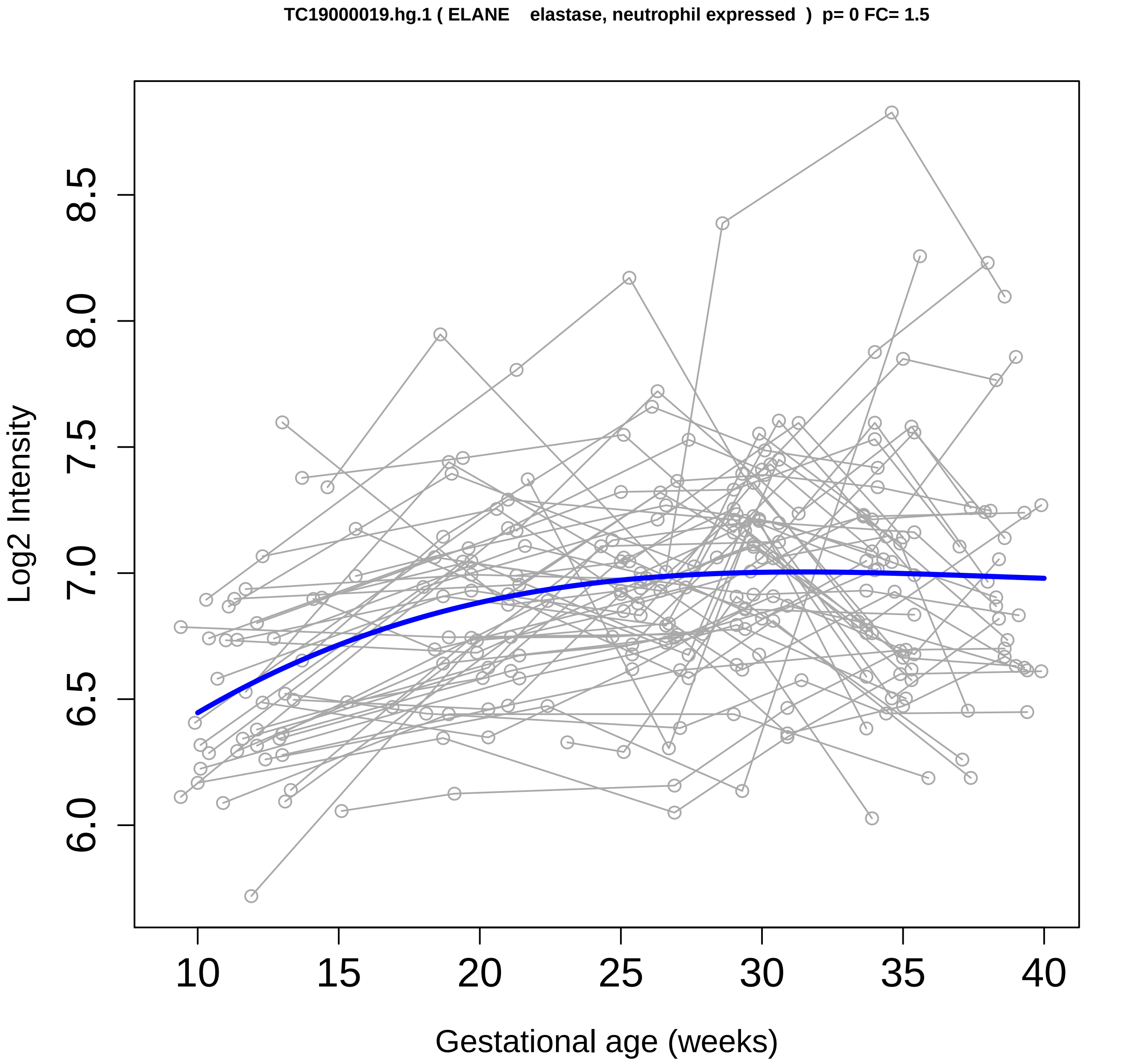

TC19000019.hg.1 (ELANE elastase, neutrophil expressed) p= 0 FC= 1.5
Log2 Intensity
6.0
6.5
7.0
7.5
8.0
8.5
10
15
20
25
30
35
40
Gestational age (weeks)

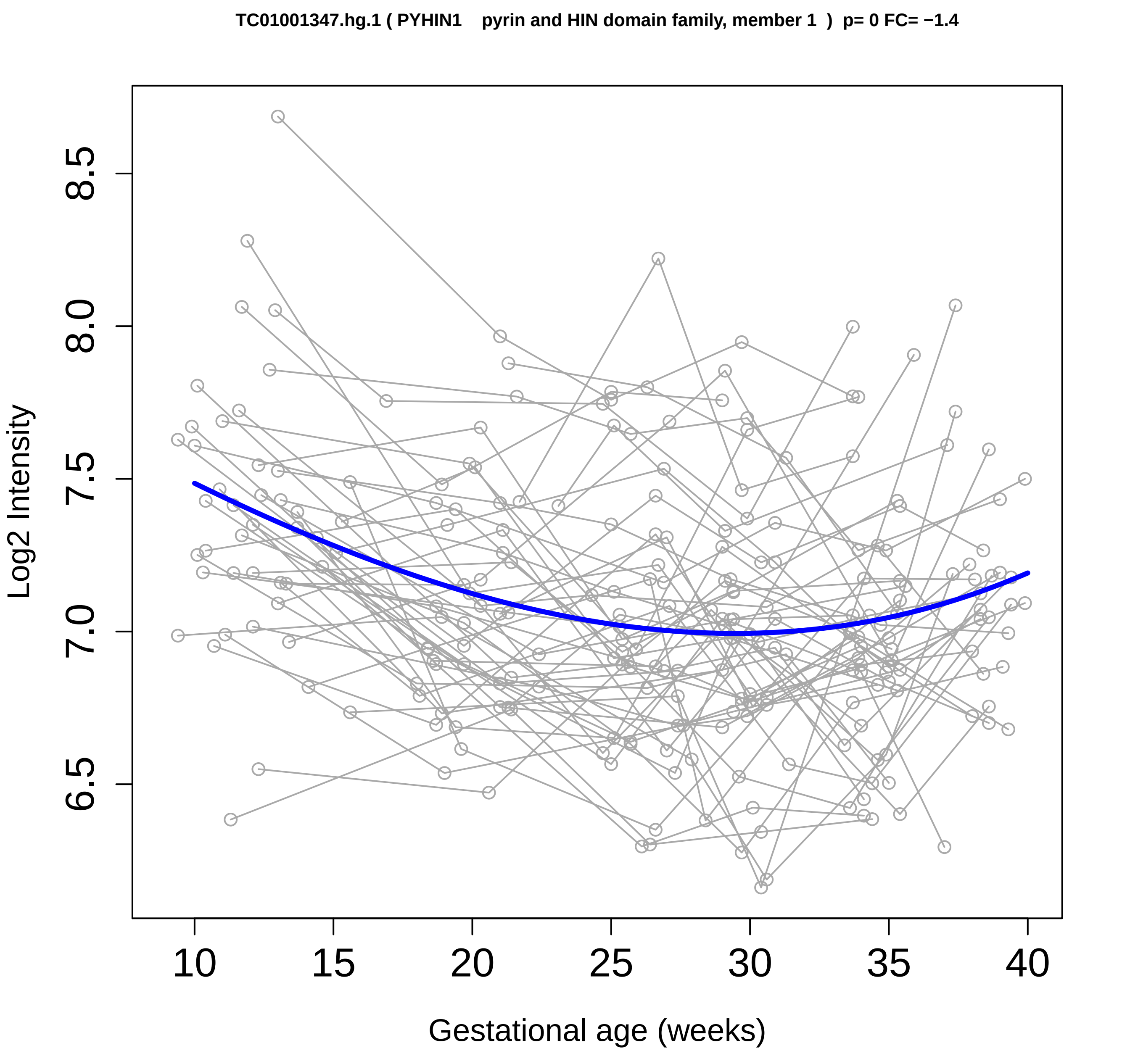
TC01001347.hg.1 (PYHIN1 pyrin and HIN domain family, member 1) p= 0 FC= −1.4
Log2 Intensity
6.5
7.0
7.5
8.0
8.5
10
15
20
25
30
35
40
Gestational age (weeks)

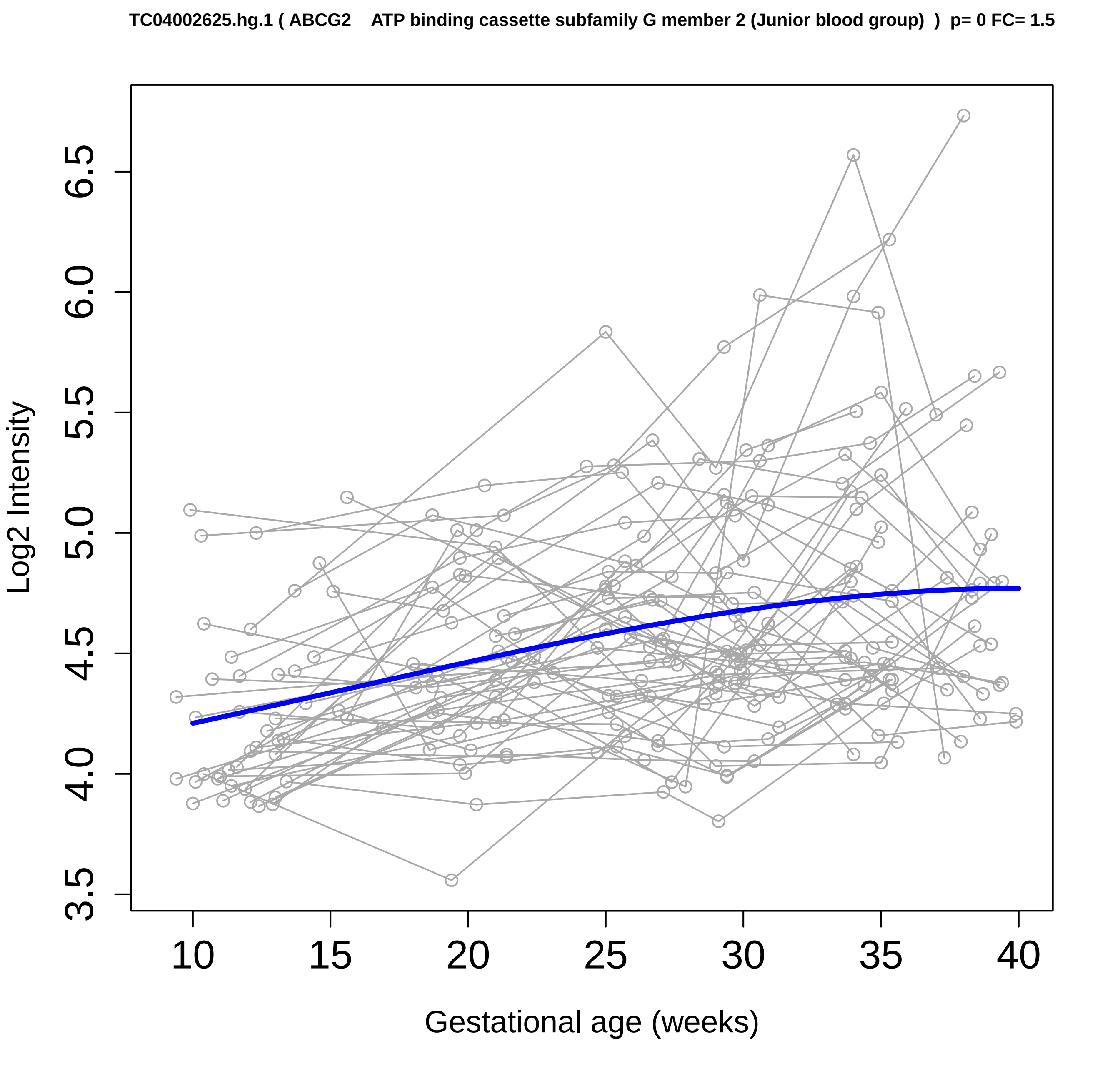

TC04002625.hg.1 (ABCG2 ATP binding cassette subfamily G member 2 (Junior blood group)) p= 0 FC= 1.5
Log2 Intensity
3.5
4.0
4.5
5.0
5.5
6.0
6.5
10
15
20
25
30
35
40
Gestational age (weeks)

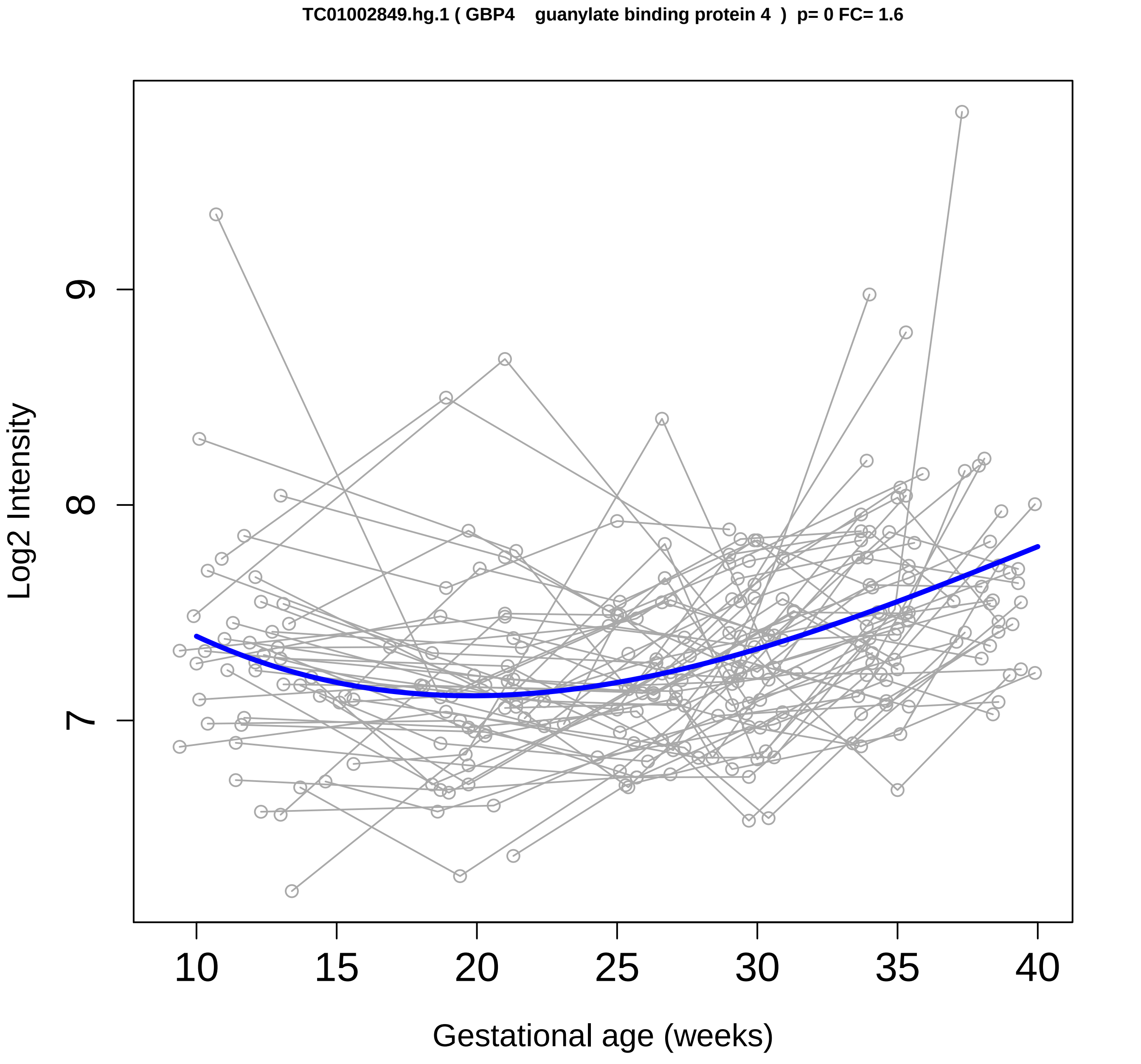

TC01002849.hg.1 (GBP4 guanylate binding protein 4) p= 0 FC= 1.6
Log2 Intensity
7
8
9
10
15
20
25
30
35
40
Gestational age (weeks)

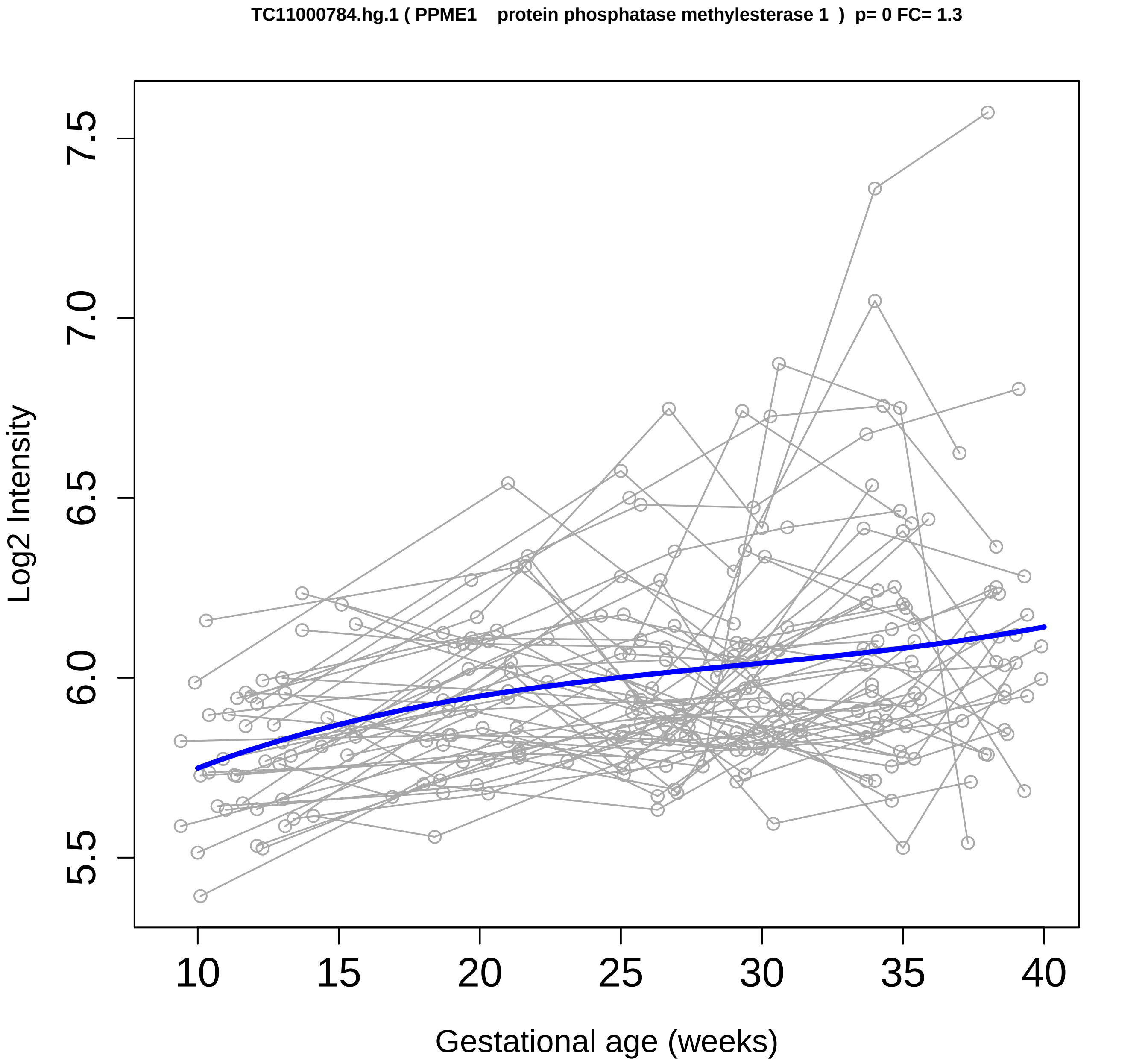

TC11000784.hg.1 (PPME1 protein phosphatase methylesterase 1) p= 0 FC= 1.3
Log2 Intensity
5.5
6.0
6.5
7.0
7.5
10
15
20
25
30
35
40
Gestational age (weeks)

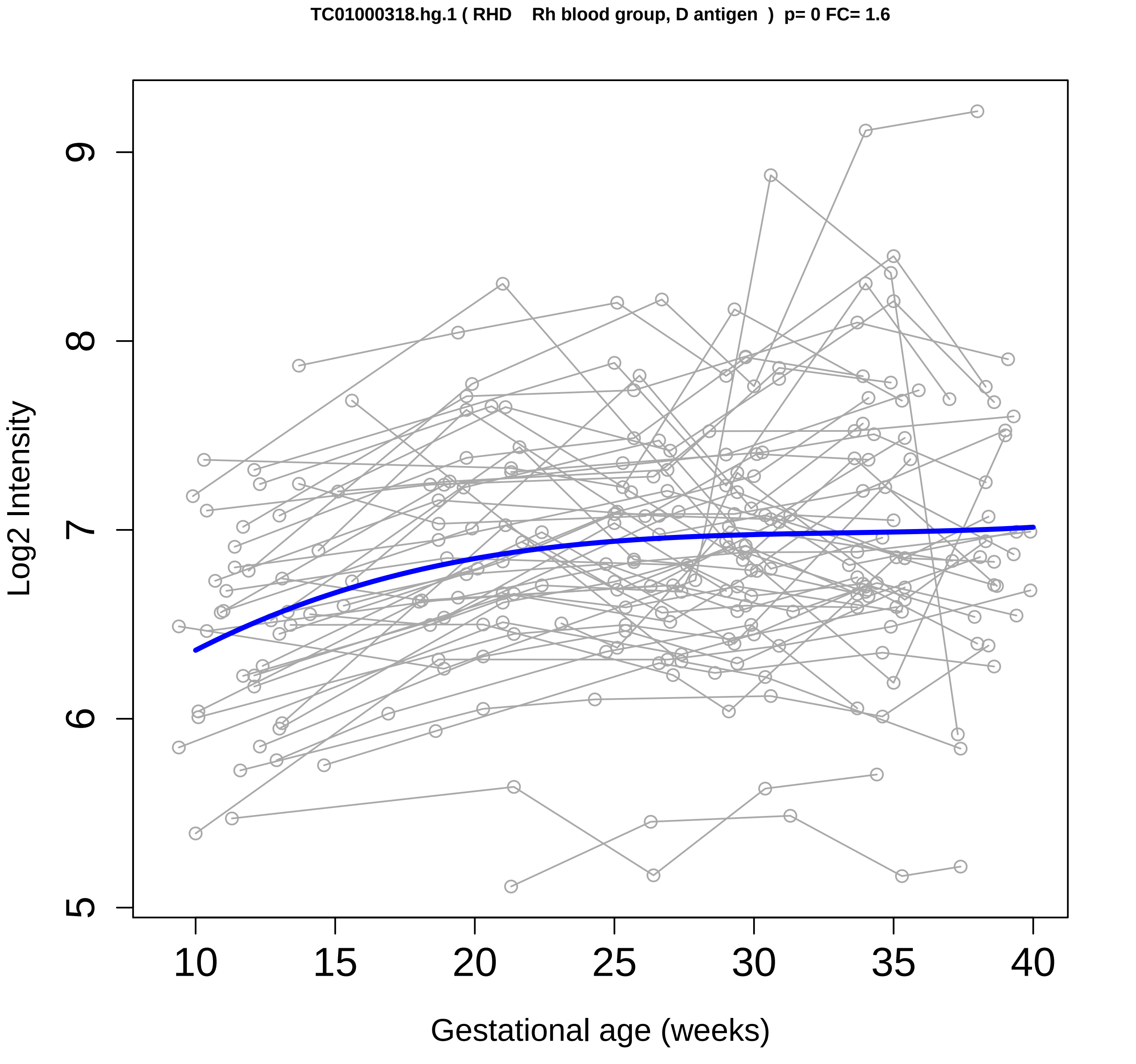

TC01000318.hg.1 (RHD Rh blood group, D antigen) p= 0 FC= 1.6
Log2 Intensity
5
6
7
8
9
10
15
20
25
30
35
40
Gestational age (weeks)

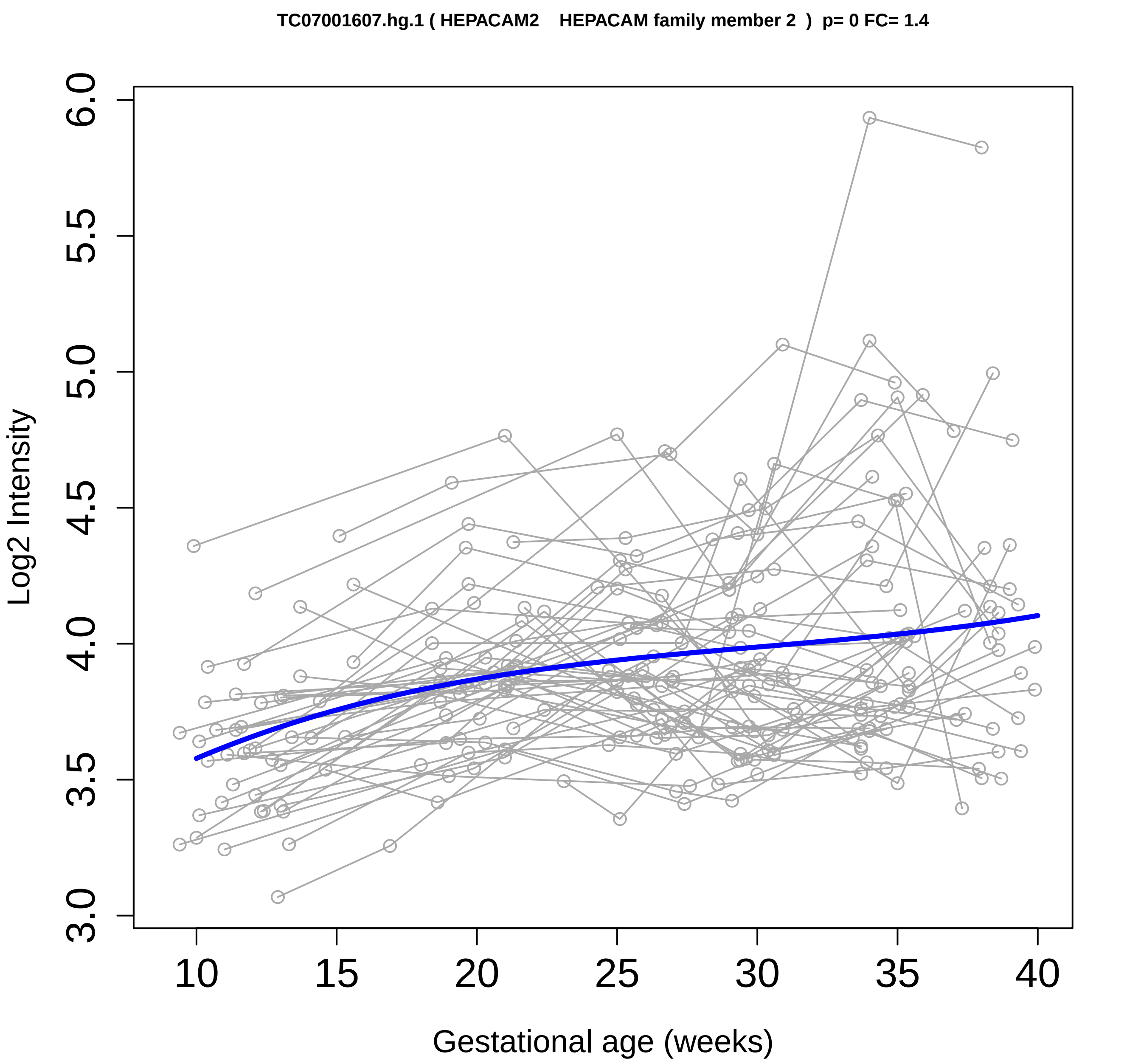
TC07001607.hg.1 (HEPACAM2   HEPACAM family member 2) p= 0 FC= 1.4
Log2 Intensity
6.0
5.5
5.0
4.5
4.0
3.5
3.0
10
15
20
25
30
35
40
Gestational age (weeks)

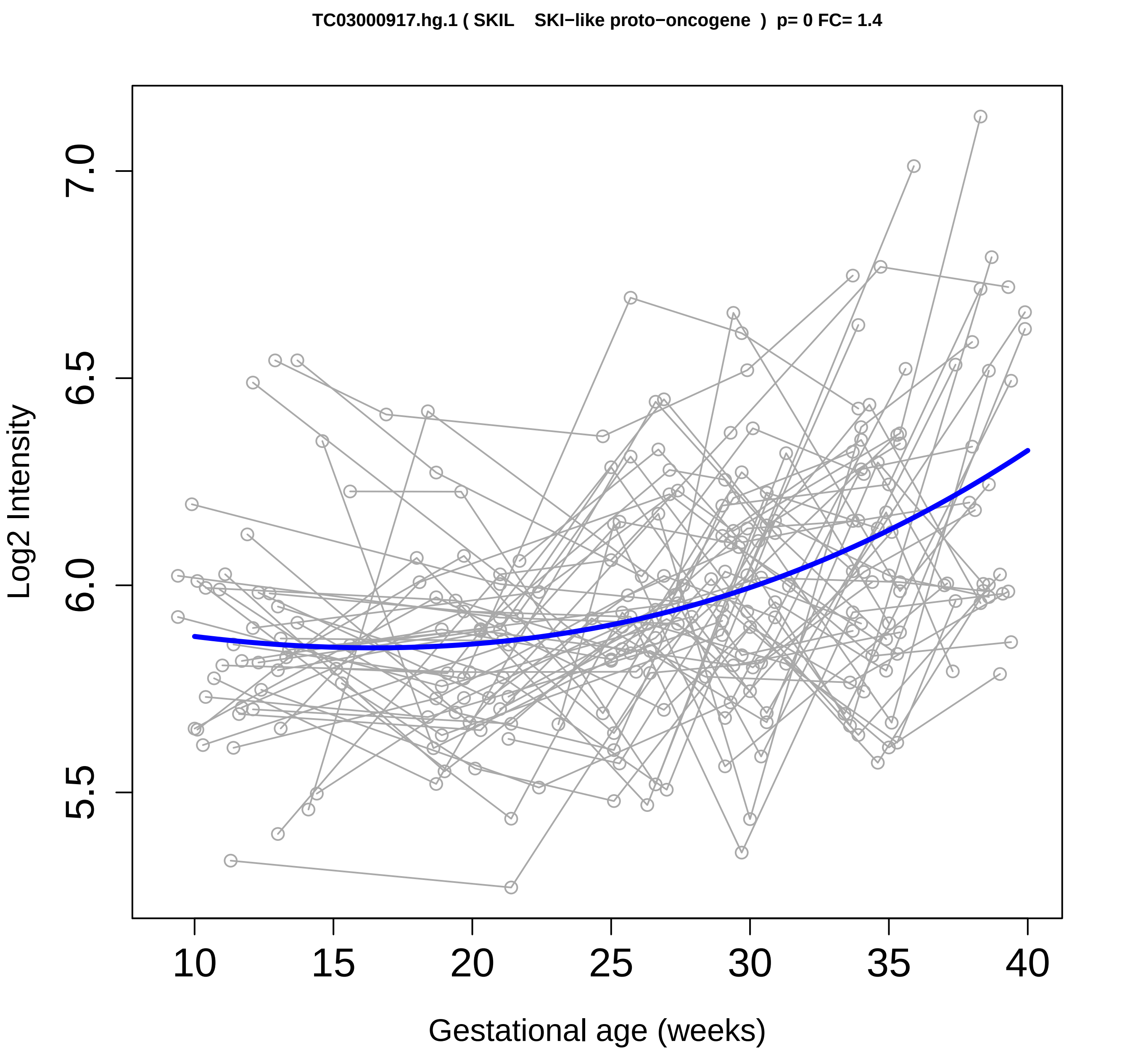
TC03000917.hg.1 (SKIL SKI−like proto−oncogene) p= 0 FC= 1.4
Log2 Intensity
7.0
6.5
6.0
5.5
10
15
20
25
30
35
40
Gestational age (weeks)

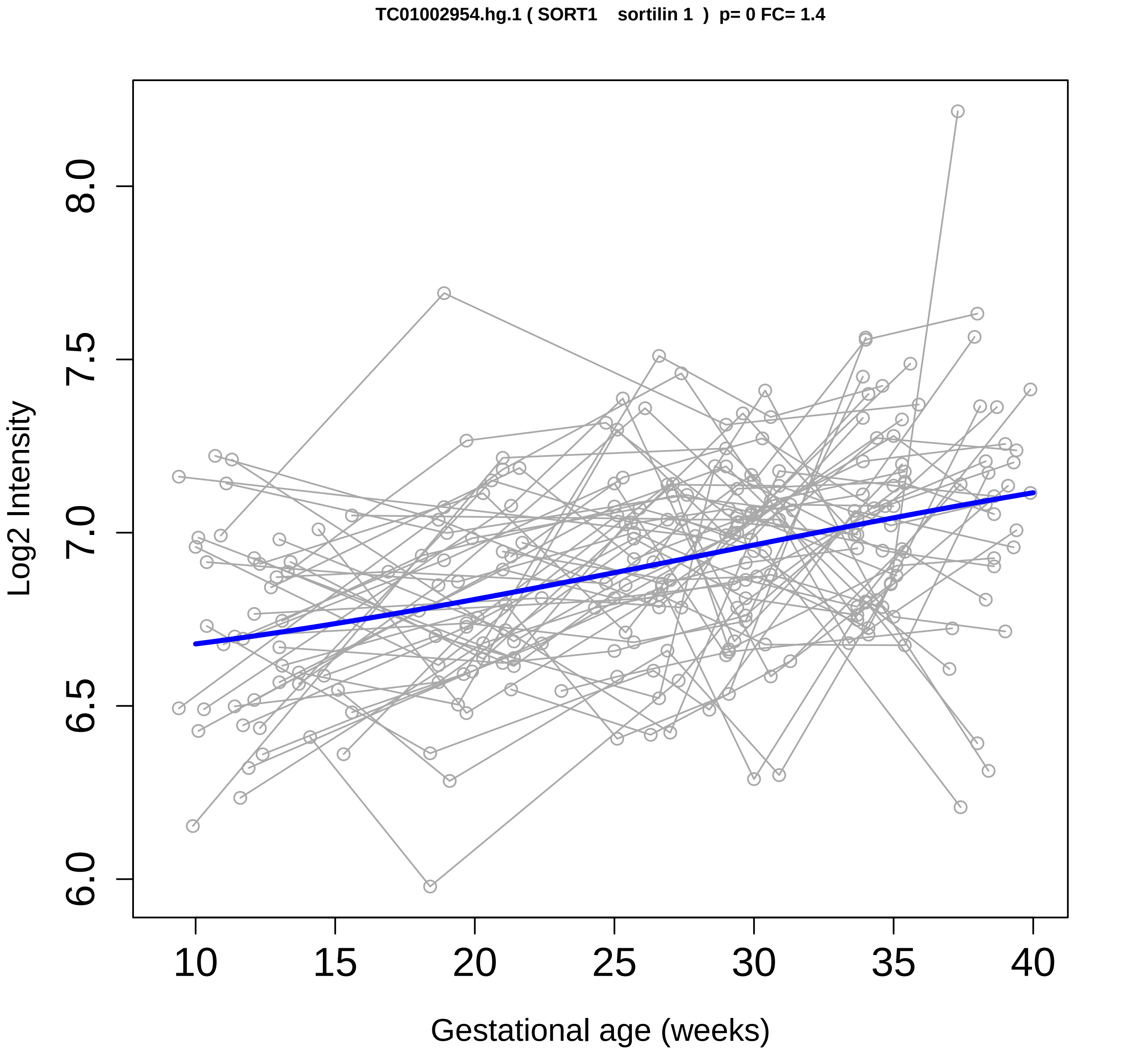

TC01002954.hg.1 (SORT1 sortilin 1) p= 0 FC= 1.4
Log2 Intensity
6.0
6.5
7.0
7.5
8.0
10
15
20
25
30
35
40
Gestational age (weeks)

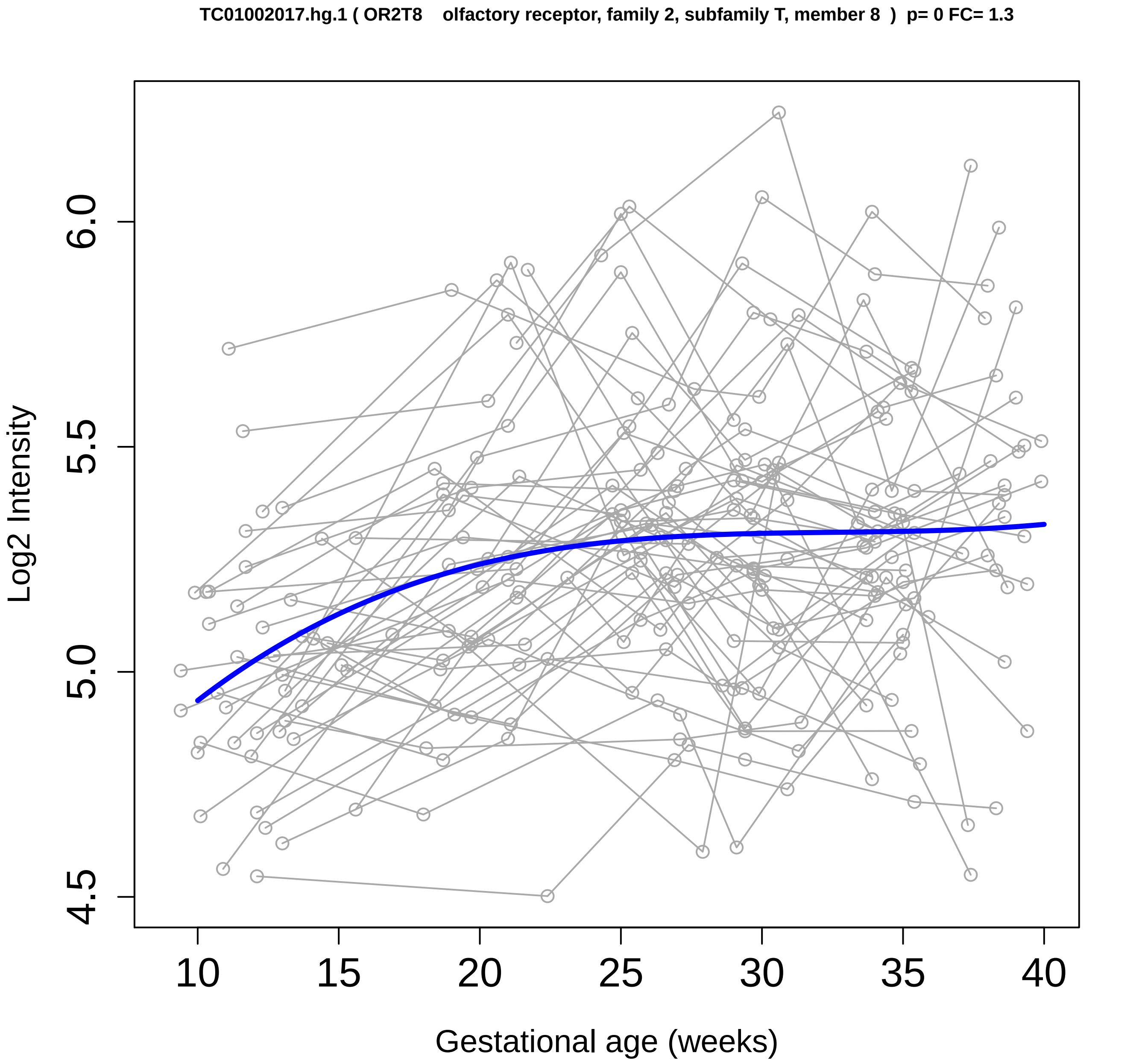

TC01002017.hg.1 (OR2T8 olfactory receptor, family 2, subfamily T, member 8) p= 0 FC= 1.3
Log2 Intensity
6.0
5.5
5.0
4.5
10
15
20
25
30
35
40
Gestational age (weeks)

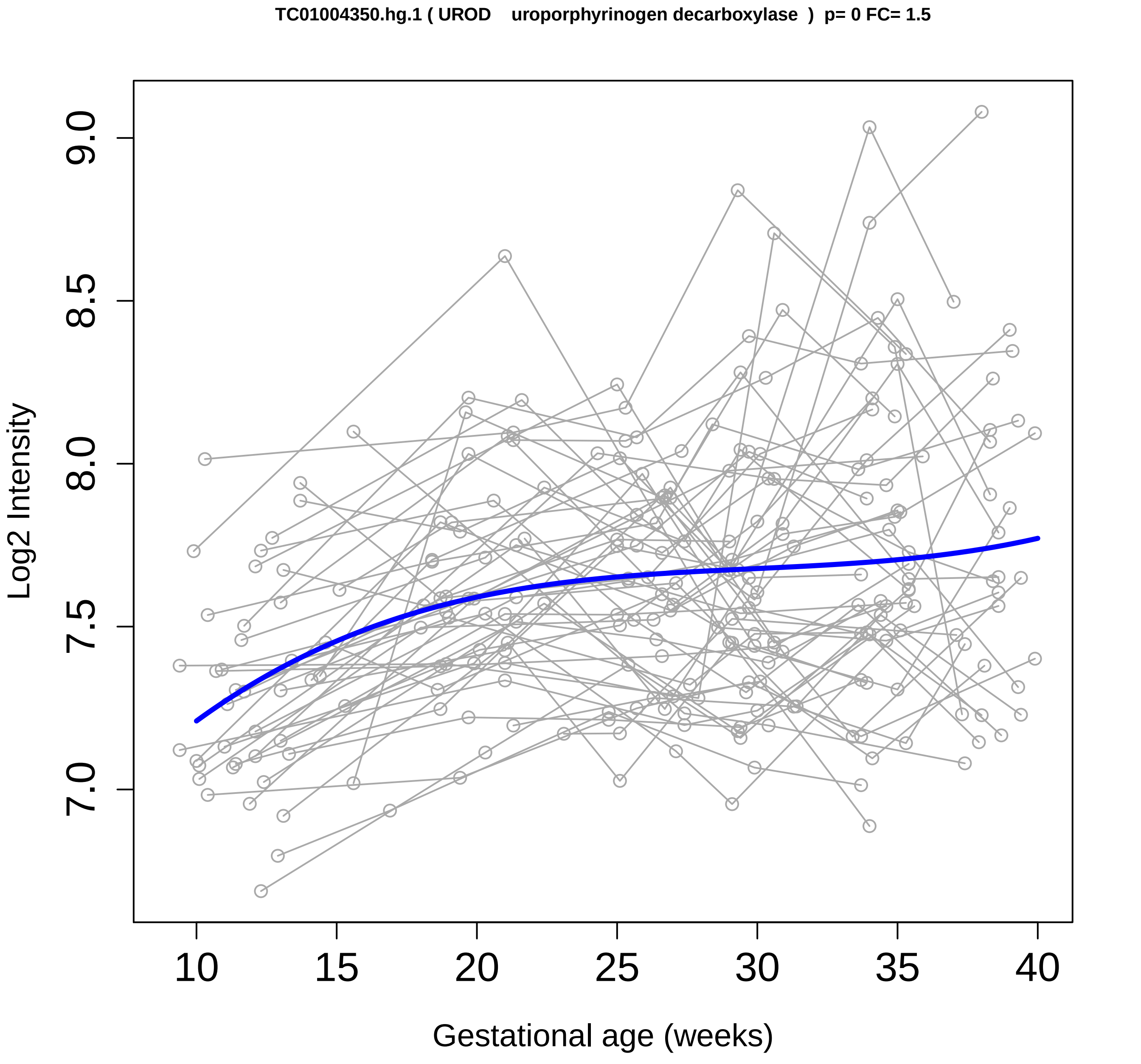

TC01004350.hg.1 (UROD uroporphyrinogen decarboxylase) p= 0 FC= 1.5
Log2 Intensity
9.0
8.5
8.0
7.5
7.0
10
15
20
25
30
35
40
Gestational age (weeks)

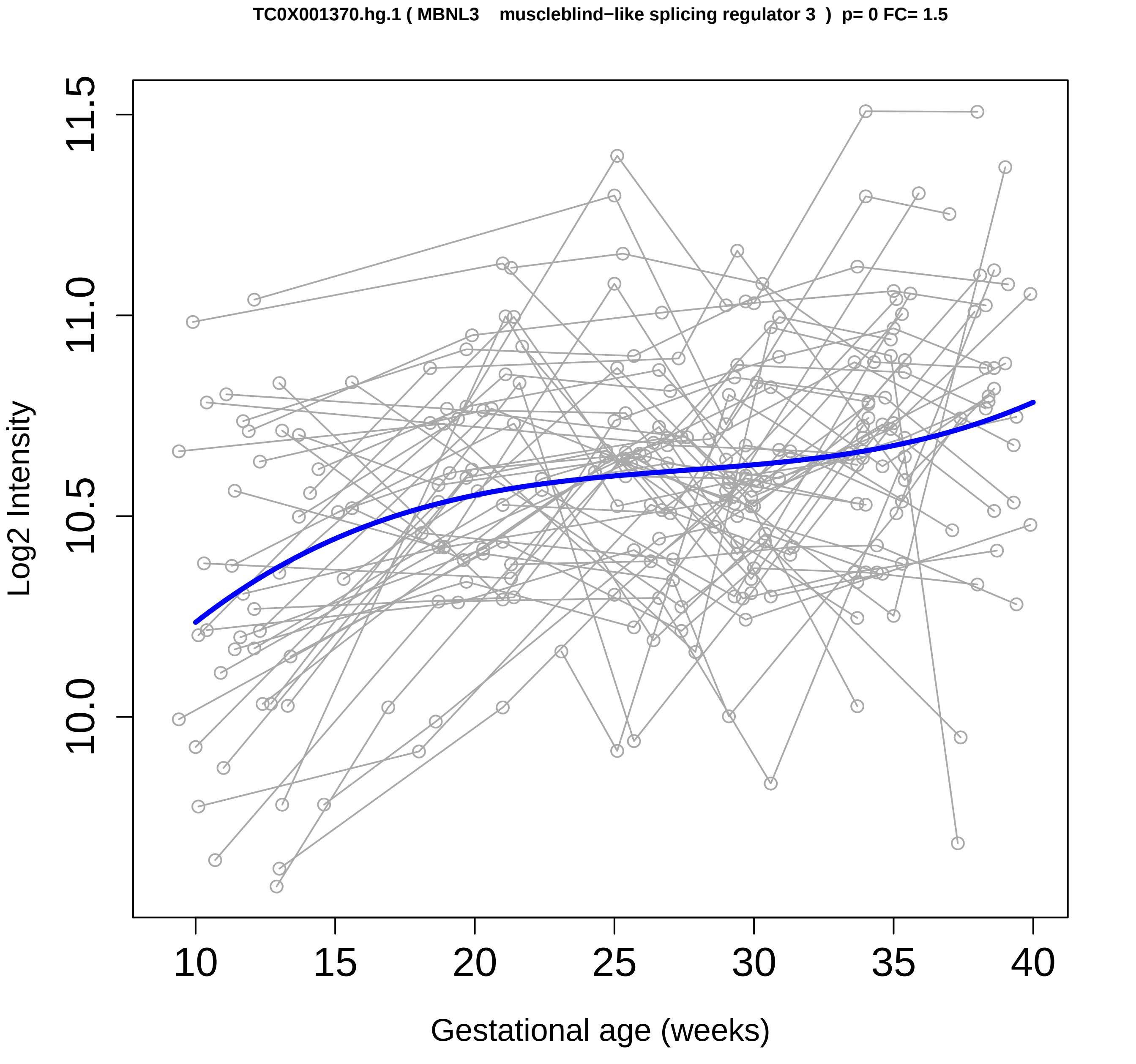

TC0X001370.hg.1 (MBNL3 muscleblind−like splicing regulator 3) p= 0 FC= 1.5
Log2 Intensity
10.0
10.5
11.0
11.5
10
15
20
25
30
35
40
Gestational age (weeks)

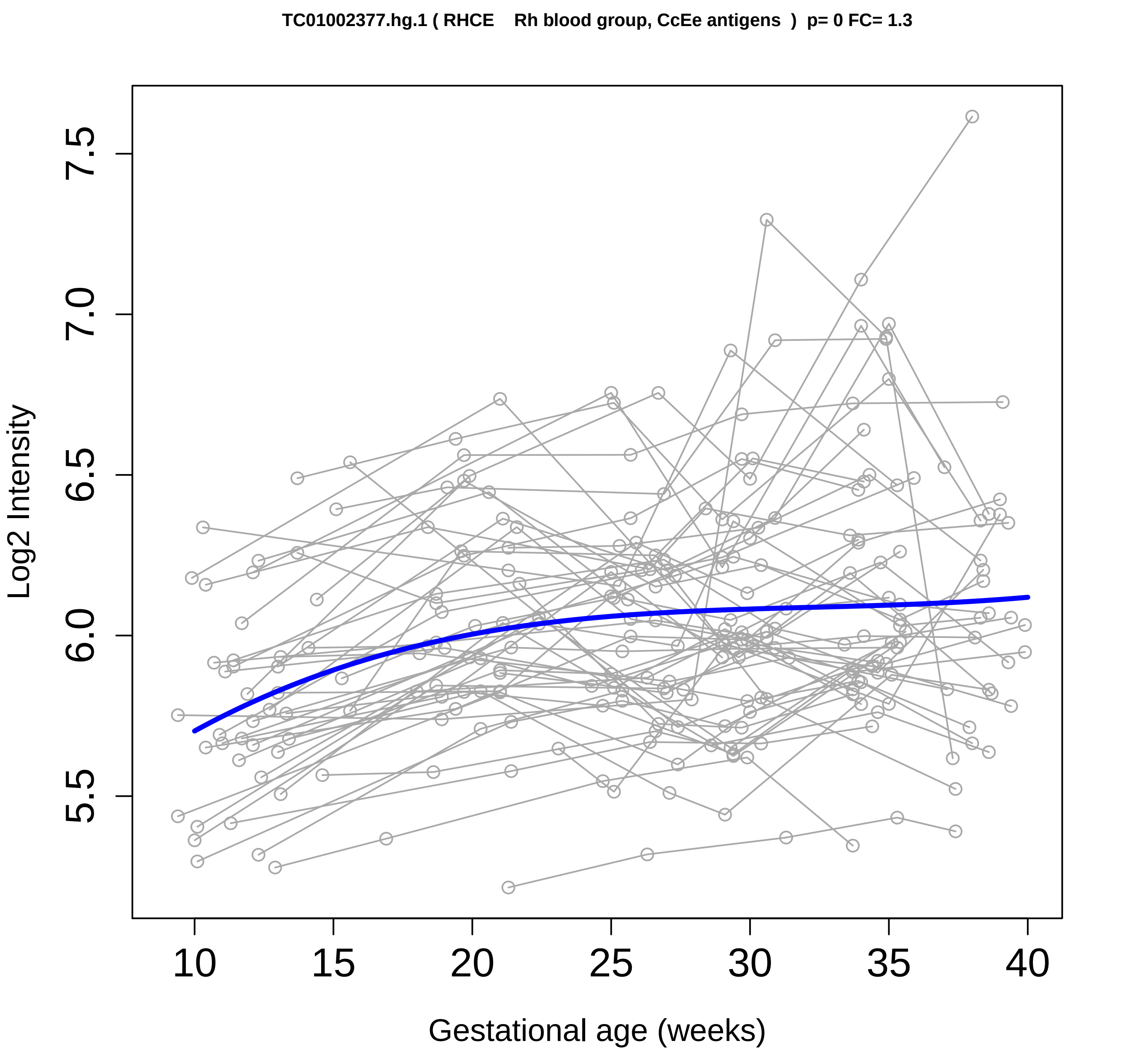
TC01002377.hg.1 (RHCE Rh blood group, CcEe antigens) p= 0 FC= 1.3
Log2 Intensity
5.5
6.0
6.5
7.0
7.5
10
15
20
25
30
35
40
Gestational age (weeks)

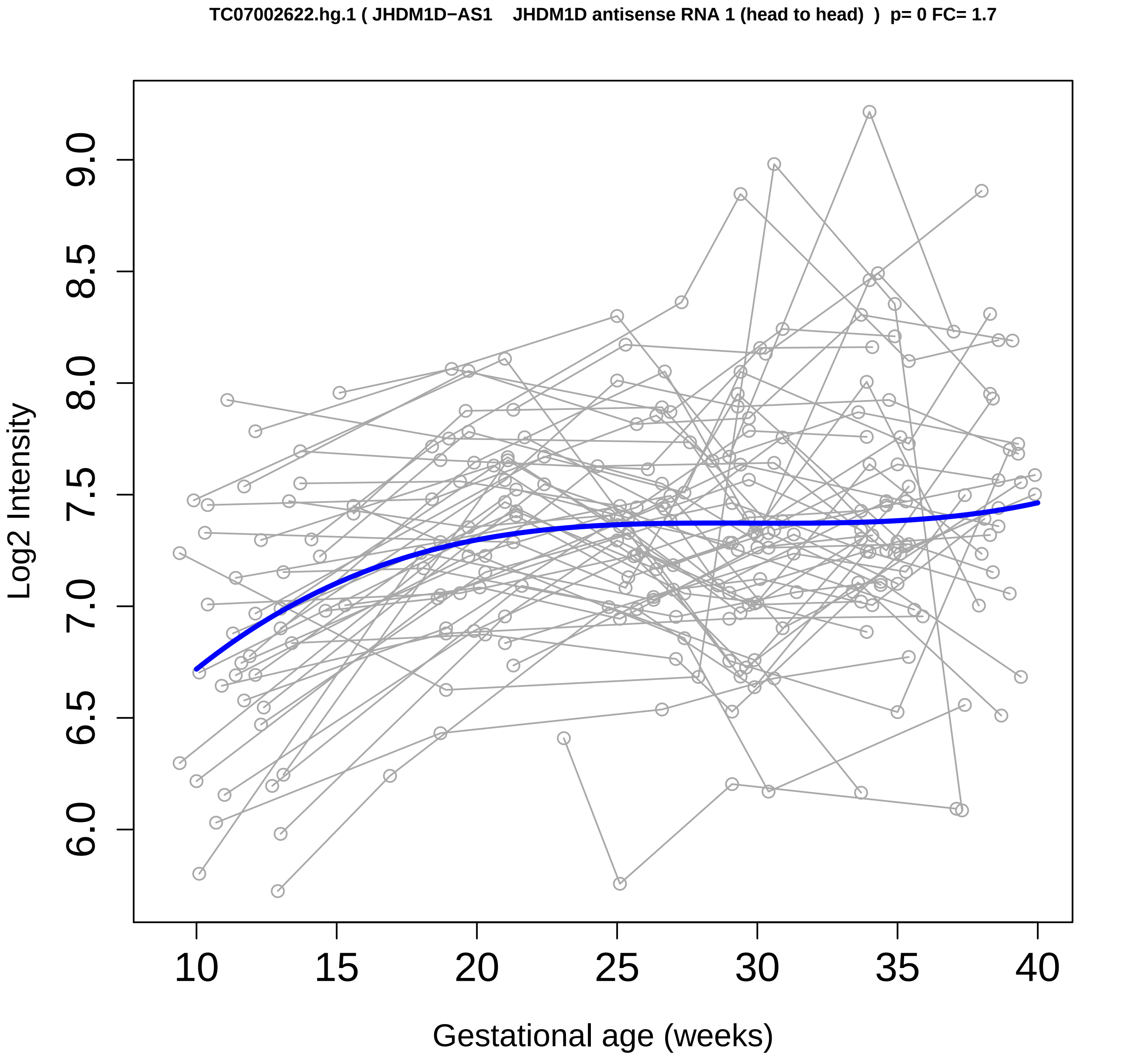
TC07002622.hg.1 (JHDM1D−AS1   JHDM1D antisense RNA 1 (head to head)) p= 0 FC= 1.7
Log2 Intensity
6.0
6.5
7.0
7.5
8.0
8.5
9.0
10
15
20
25
30
35
40
Gestational age (weeks)

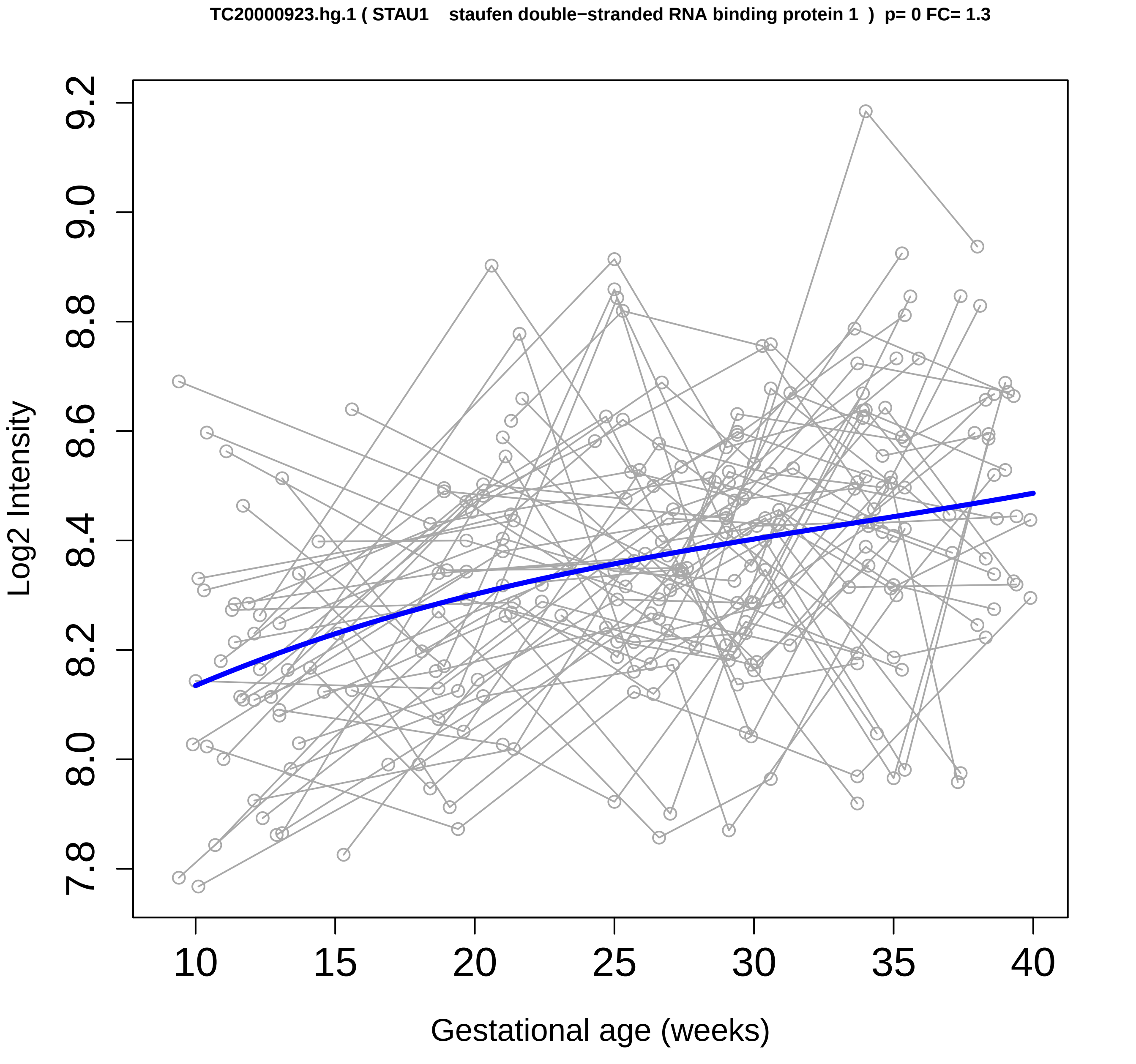

TC20000923.hg.1 (STAU1 staufen double−stranded RNA binding protein 1) p= 0 FC= 1.3
Log2 Intensity
7.8
8.0
8.2
8.4
8.6
8.8
9.0
9.2
10
15
20
25
30
35
40
Gestational age (weeks)

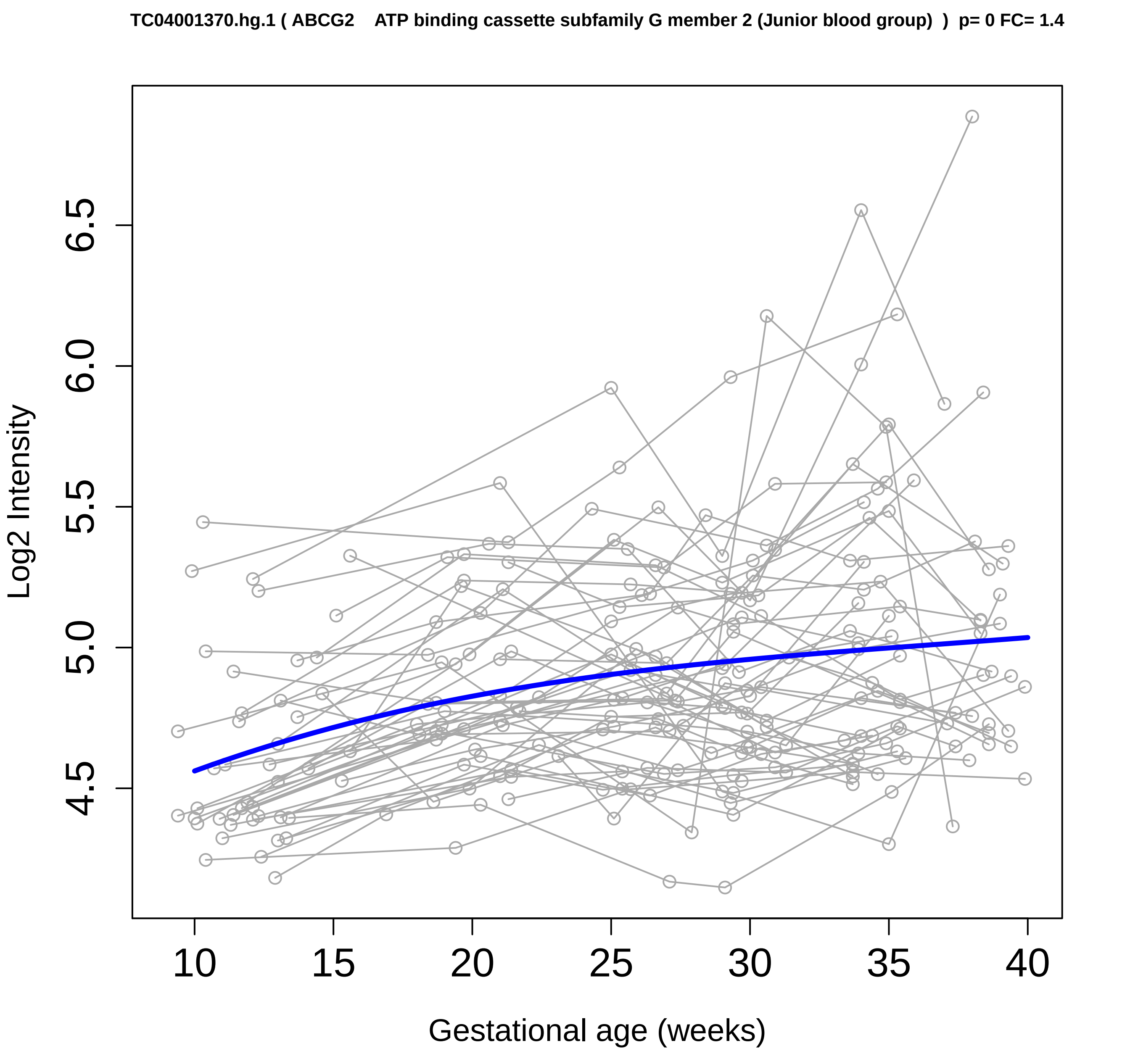
TC04001370.hg.1 (ABCG2   ATP binding cassette subfamily G member 2 (Junior blood group)) p= 0 FC= 1.4
Log2 Intensity
4.5
5.0
5.5
6.0
6.5
10
15
20
25
30
35
40
Gestational age (weeks)

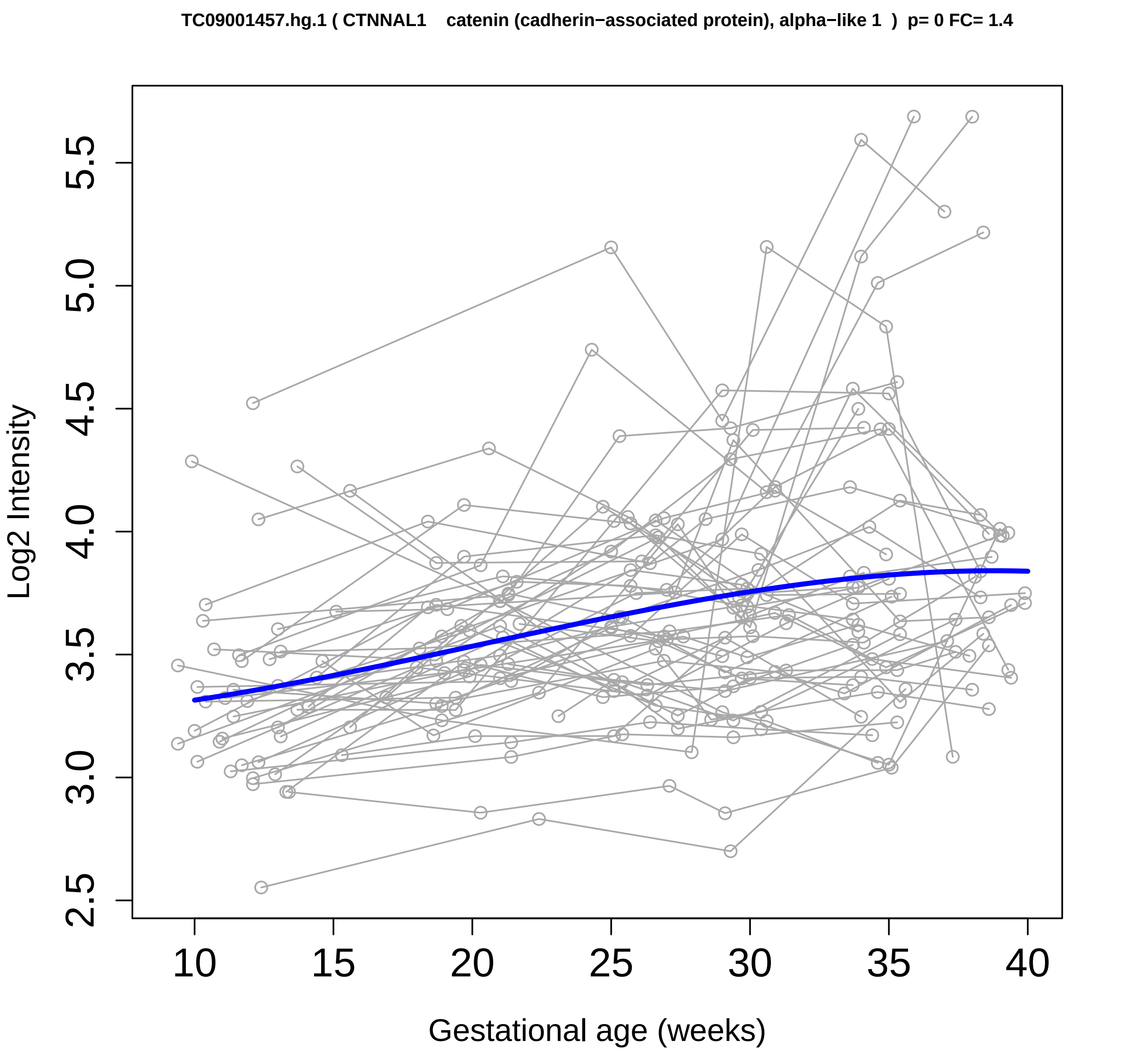
TC09001457.hg.1 (CTNNAL1   catenin (cadherin−associated protein), alpha−like 1) p= 0 FC= 1.4
Log2 Intensity
2.5
3.0
3.5
4.0
4.5
5.0
5.5
10
15
20
25
30
35
40
Gestational age (weeks)

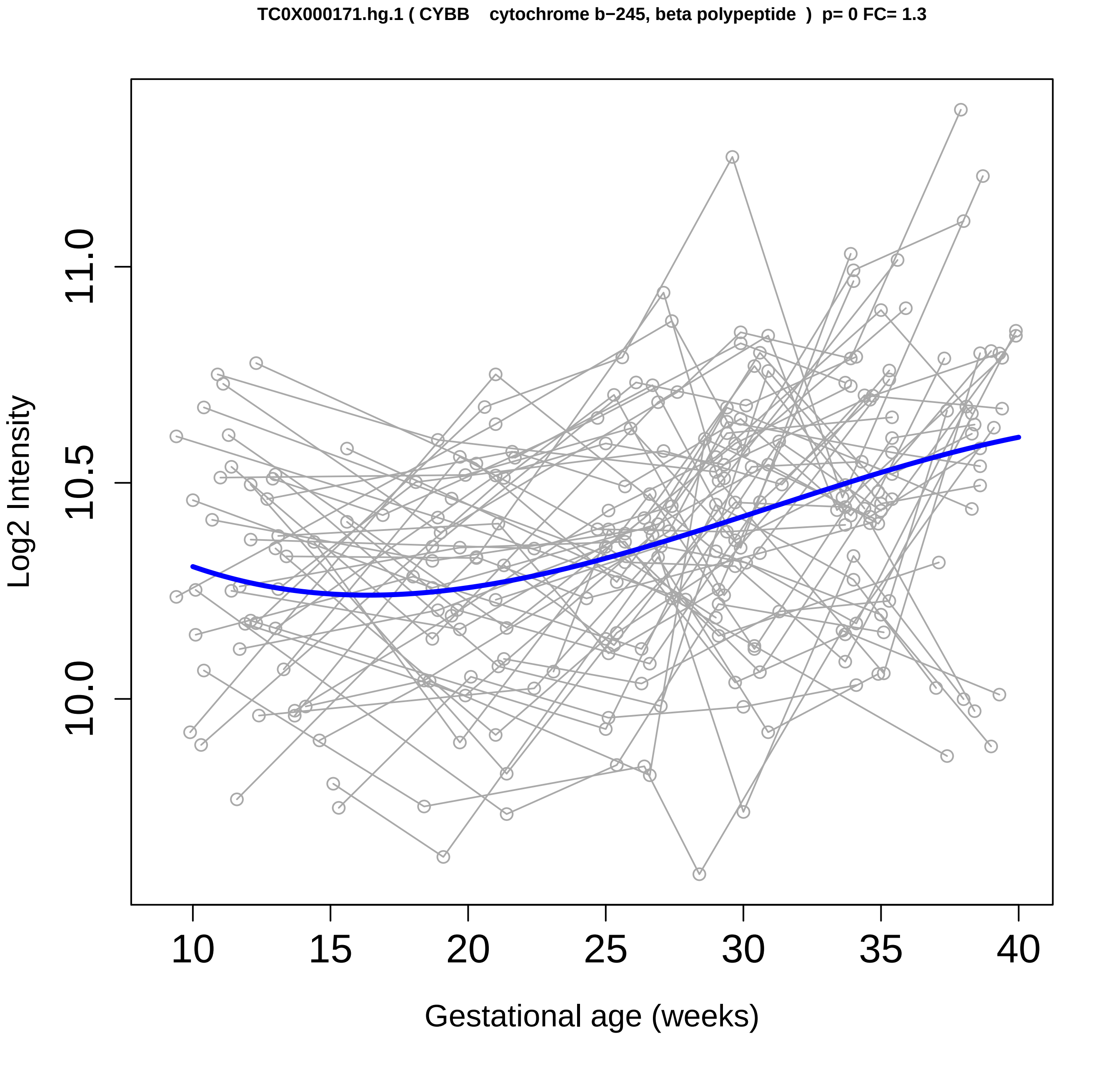

TC0X000171.hg.1 (CYBB cytochrome b−245, beta polypeptide) p= 0 FC= 1.3
Log2 Intensity
10.0
10.5
11.0
10
15
20
25
30
35
40
Gestational age (weeks)

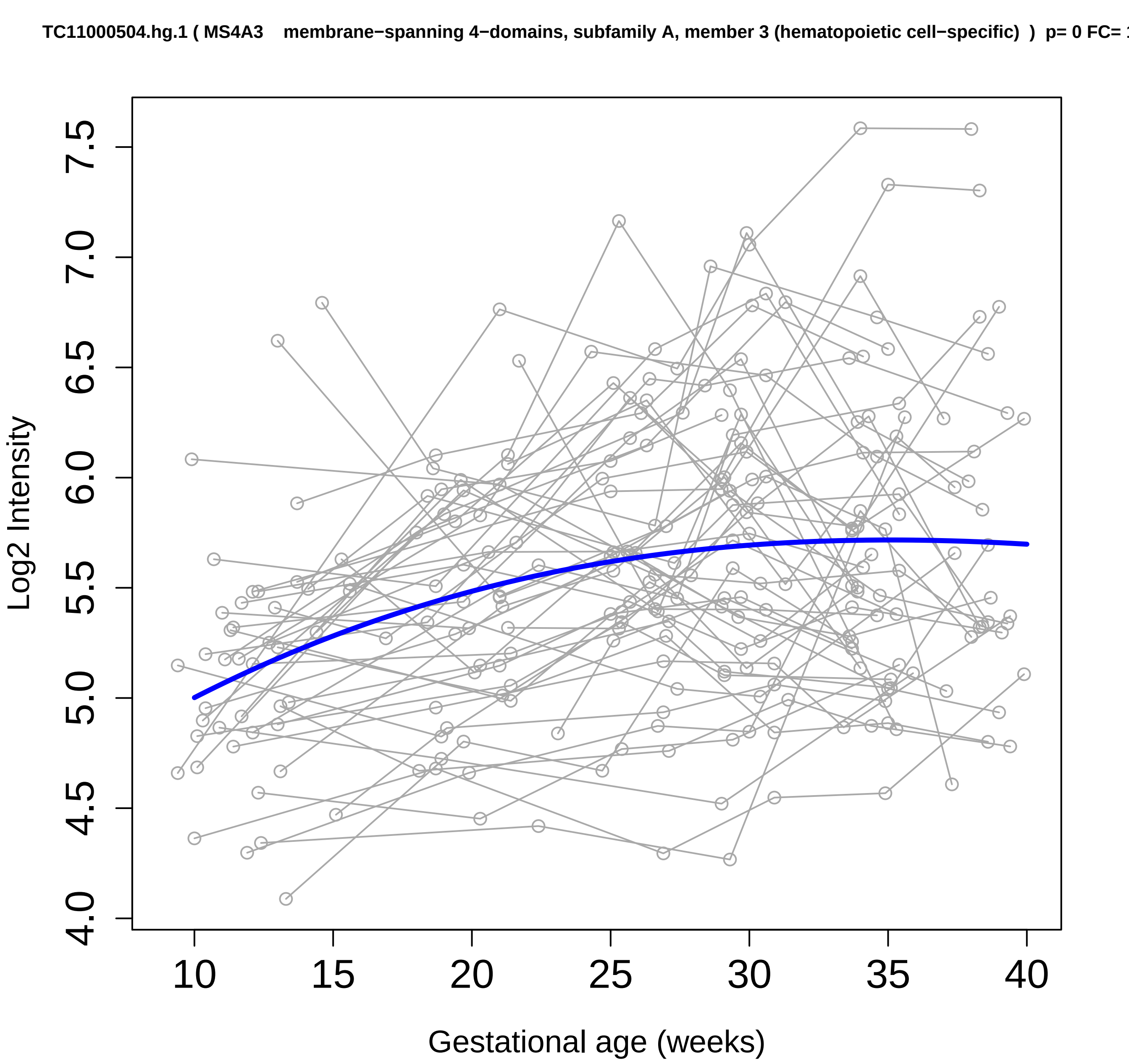
TC11000504.hg.1 (MS4A3 membrane−spanning 4−domains, subfamily A, member 3 (hematopoietic cell−specific)) p= 0 FC=
Log2 Intensity
7.5
7.0
6.5
6.0
5.5
5.0
4.5
4.0
10
15
20
25
30
35
40
Gestational age (weeks)

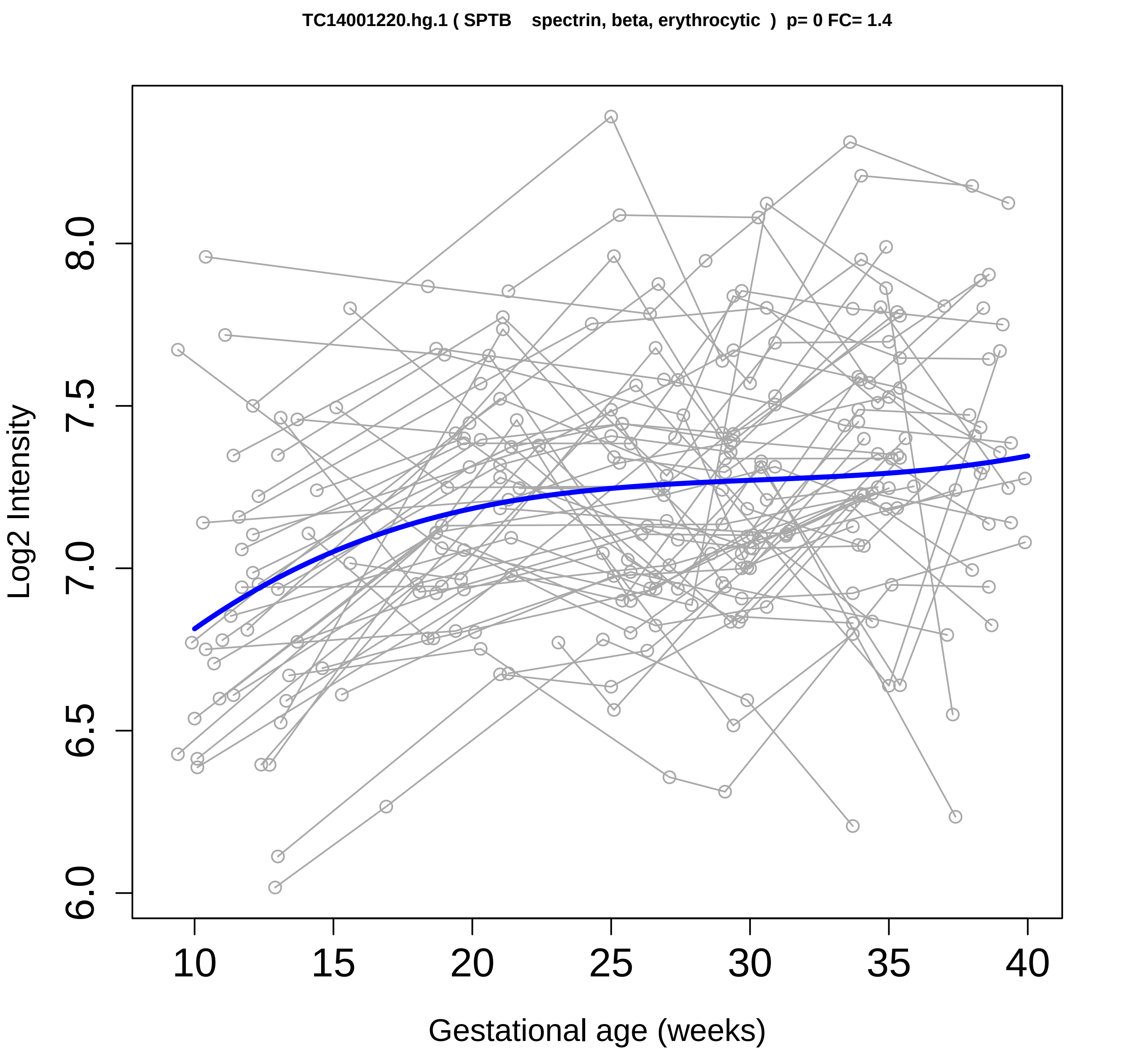

TC14001220.hg.1 (SPTB spectrin, beta, erythrocytic) p= 0 FC= 1.4
Log2 Intensity
6.0
6.5
7.0
7.5
8.0
10
15
20
25
30
35
40
Gestational age (weeks)

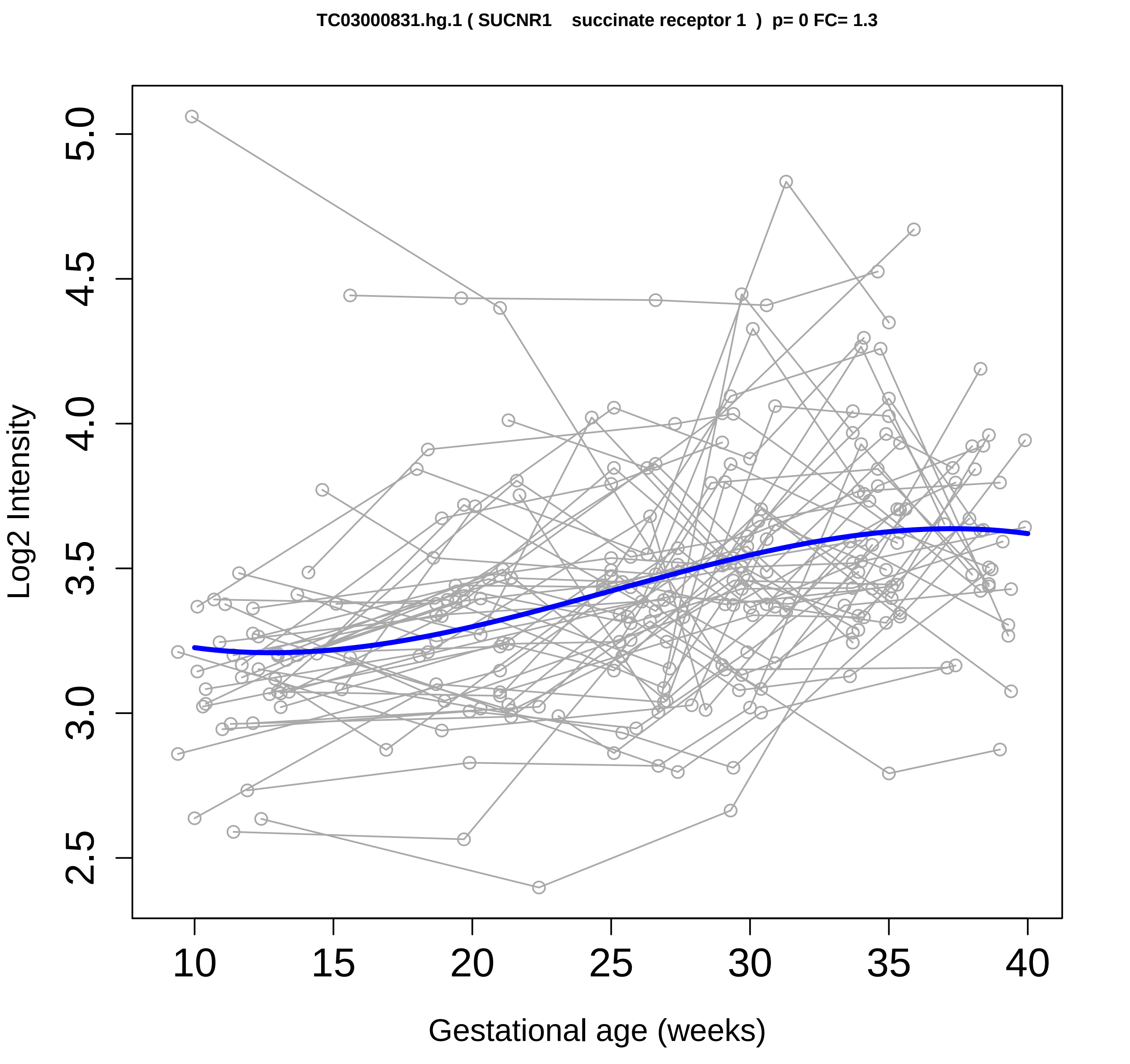

TC03000831.hg.1 (SUCNR1 succinate receptor 1) p= 0 FC= 1.3
Log2 Intensity
5.0
4.5
4.0
3.5
3.0
2.5
10
15
20
25
30
35
40
Gestational age (weeks)

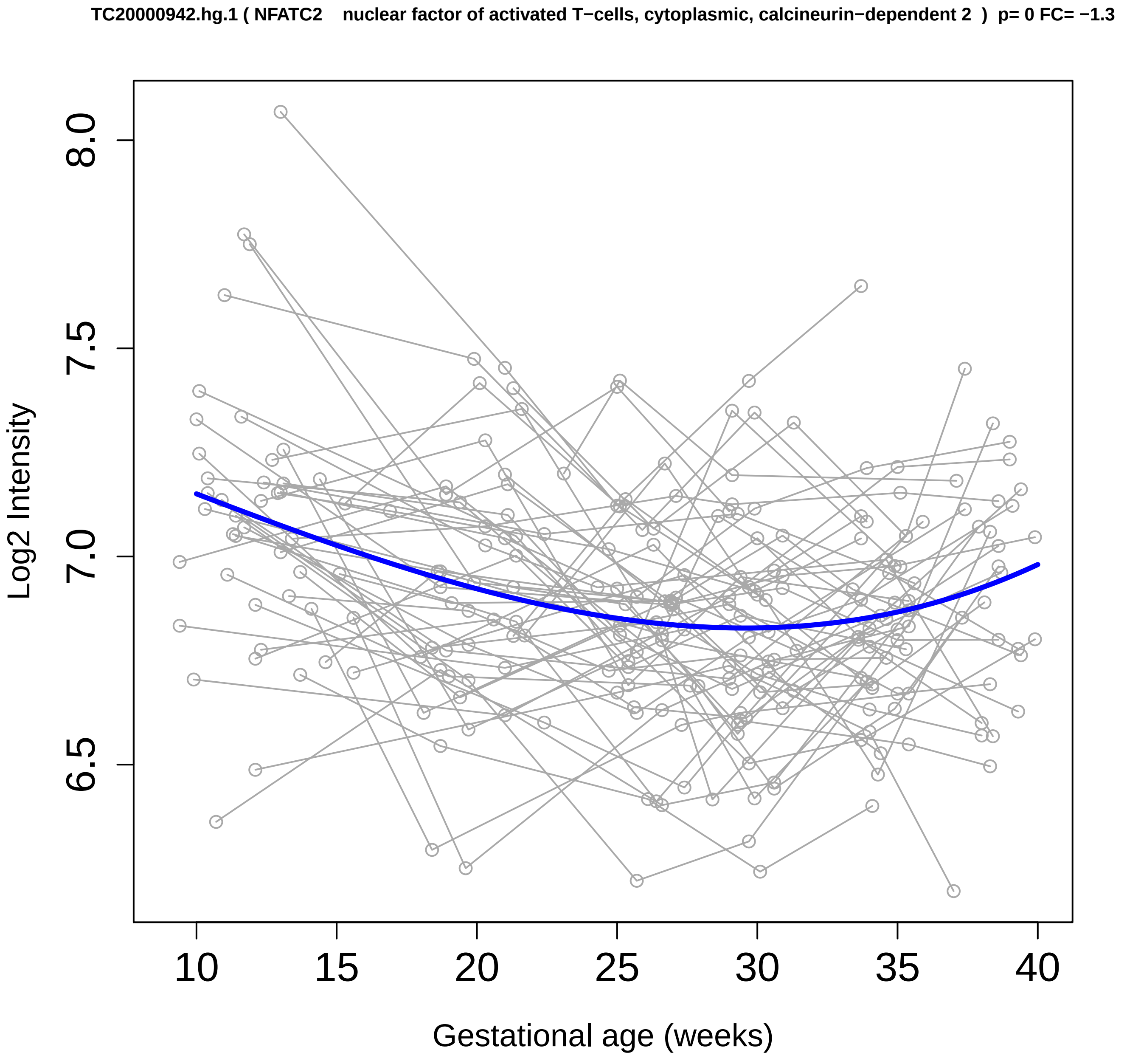

TC20000942.hg.1 (NFATC2 nuclear factor of activated T−cells, cytoplasmic, calcineurin−dependent 2) p= 0 FC= −1.3
Log2 Intensity
8.0
7.5
7.0
6.5
10
15
20
25
30
35
40
Gestational age (weeks)

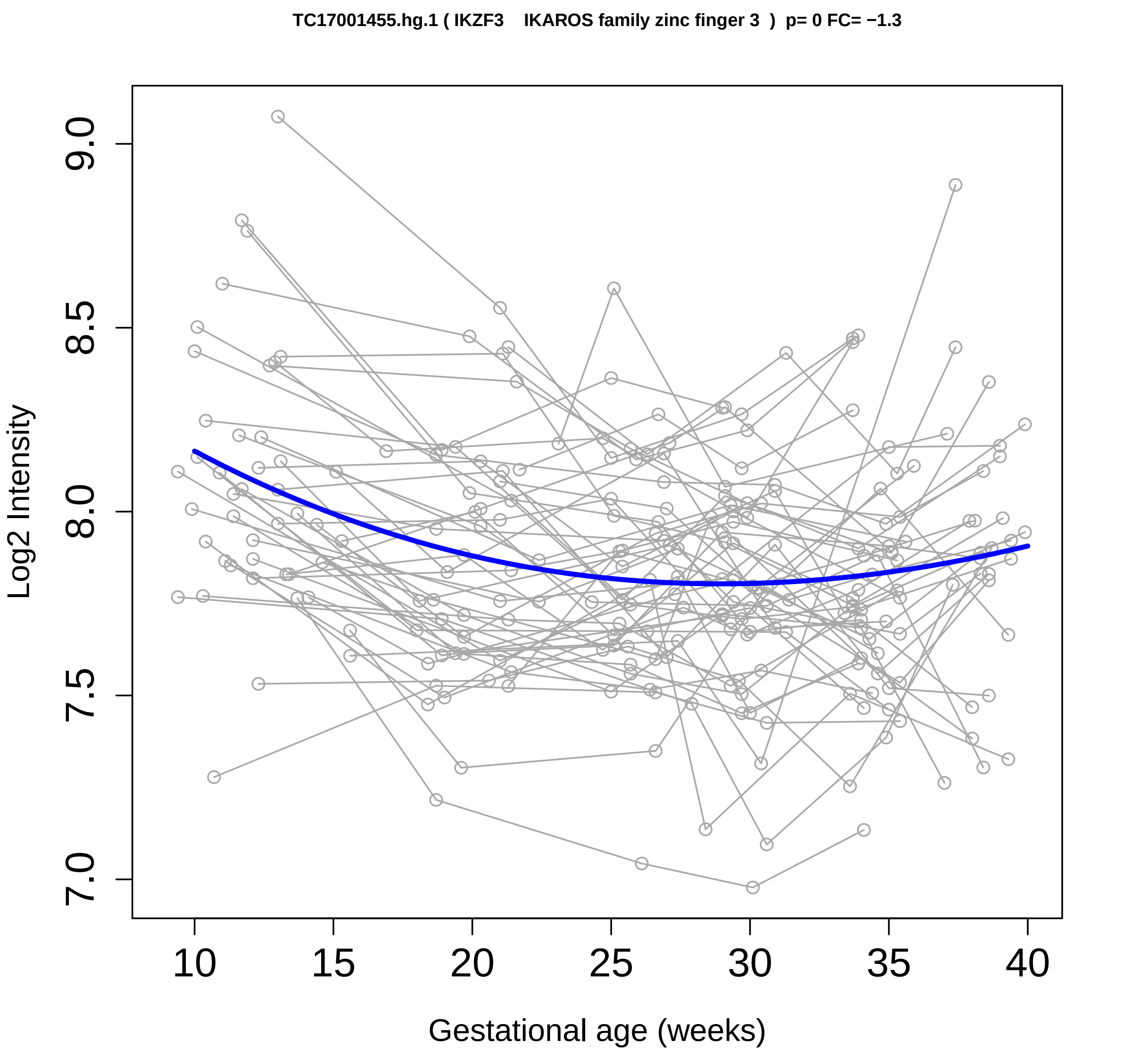

TC17001455.hg.1 (IKZF3 IKAROS family zinc finger 3) p= 0 FC= −1.3
Log2 Intensity
7.0
7.5
8.0
8.5
9.0
10
15
20
25
30
35
40
Gestational age (weeks)

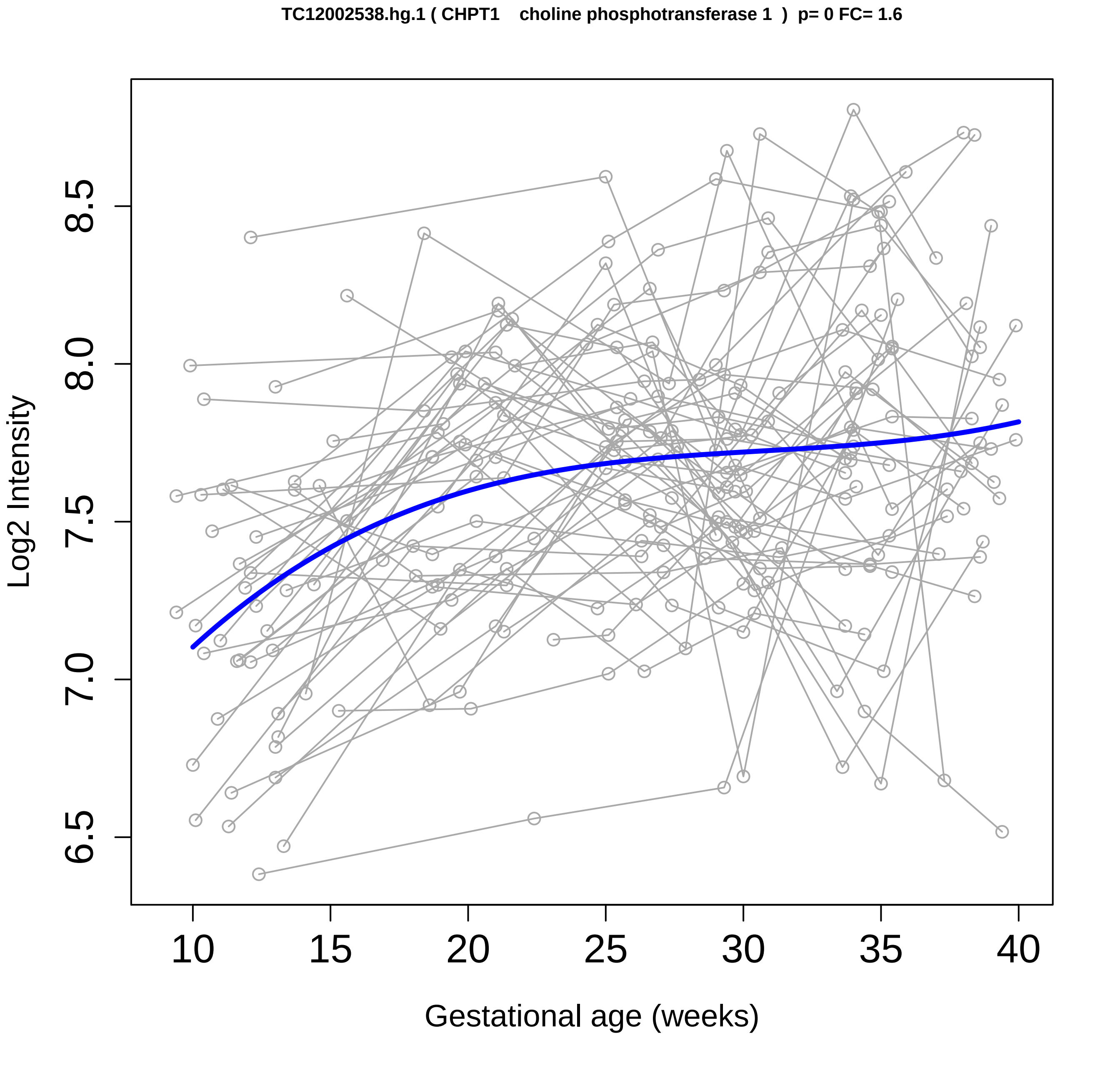

TC12002538.hg.1 (CHPT1 choline phosphotransferase 1) p= 0 FC= 1.6
Log2 Intensity
6.5
7.0
7.5
8.0
8.5
10
15
20
25
30
35
40
Gestational age (weeks)

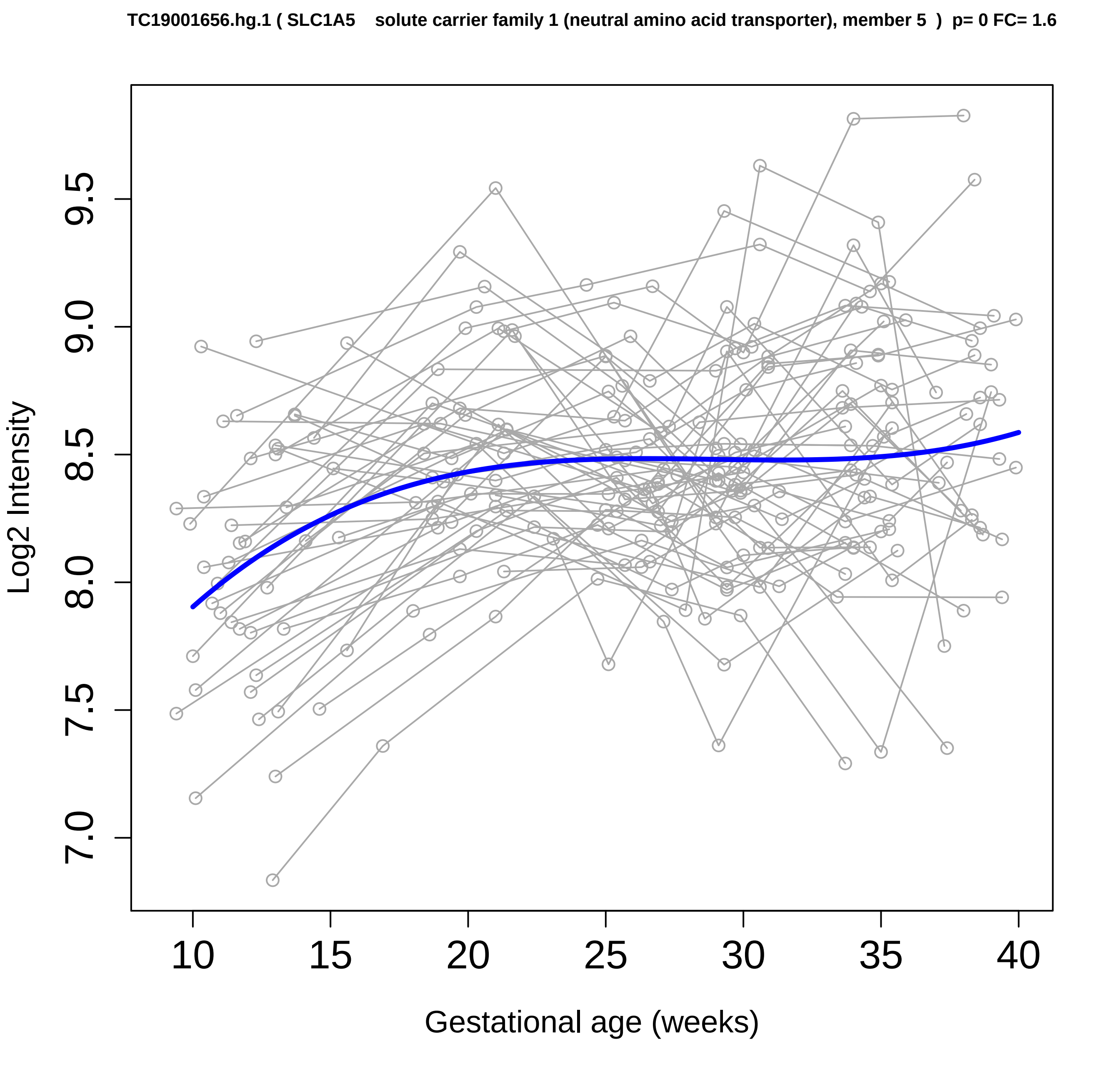

TC19001656.hg.1 (SLC1A5 solute carrier family 1 (neutral amino acid transporter), member 5) p= 0 FC= 1.6
Log2 Intensity
7.0
7.5
8.0
8.5
9.0
9.5
10
15
20
25
30
35
40
Gestational age (weeks)

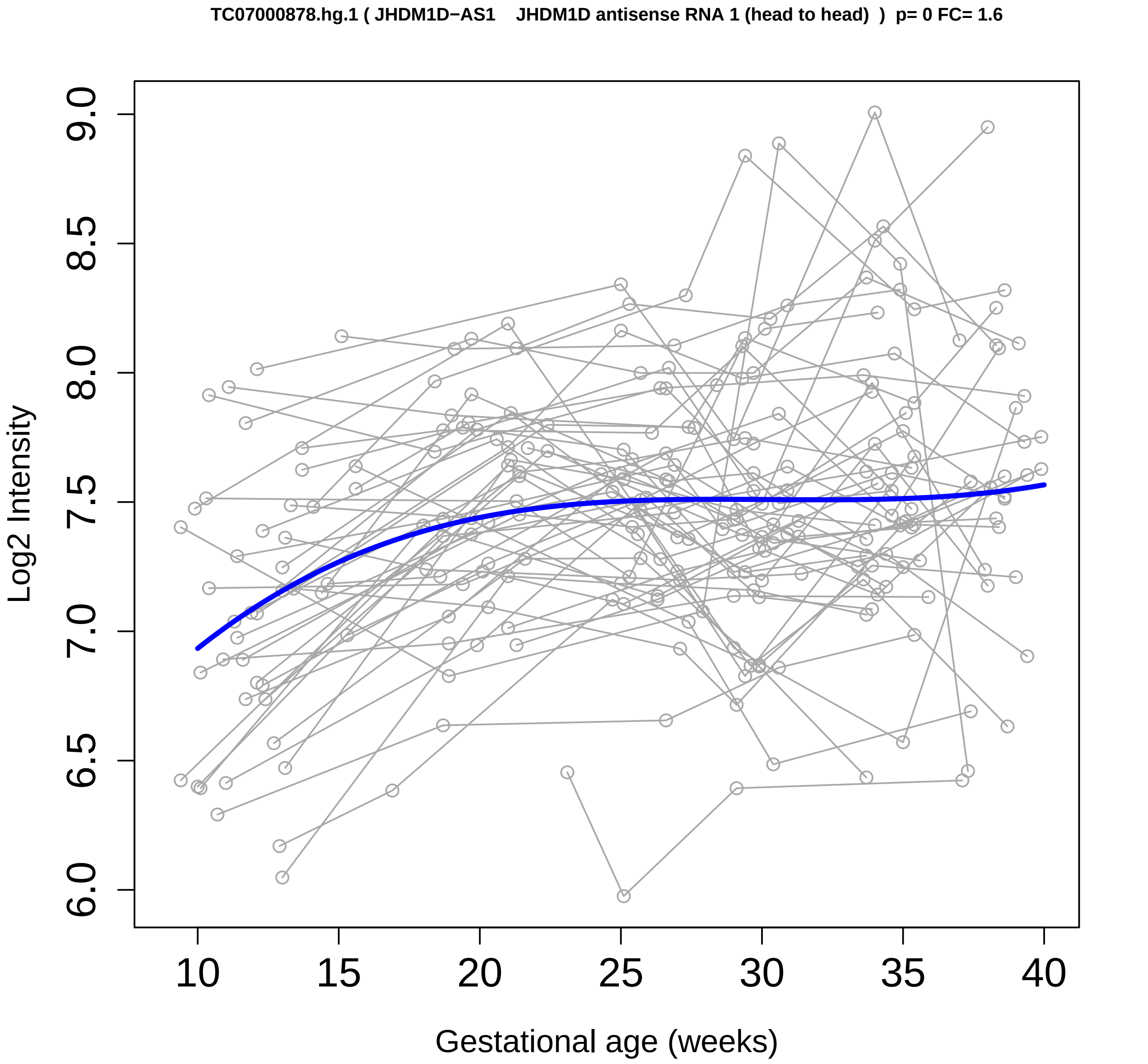
TC07000878.hg.1 (JHDM1D−AS1   JHDM1D antisense RNA 1 (head to head)) p= 0 FC= 1.6
Log2 Intensity
9.0
8.5
8.0
7.5
7.0
6.5
6.0
10
15
20
25
30
35
40
Gestational age (weeks)

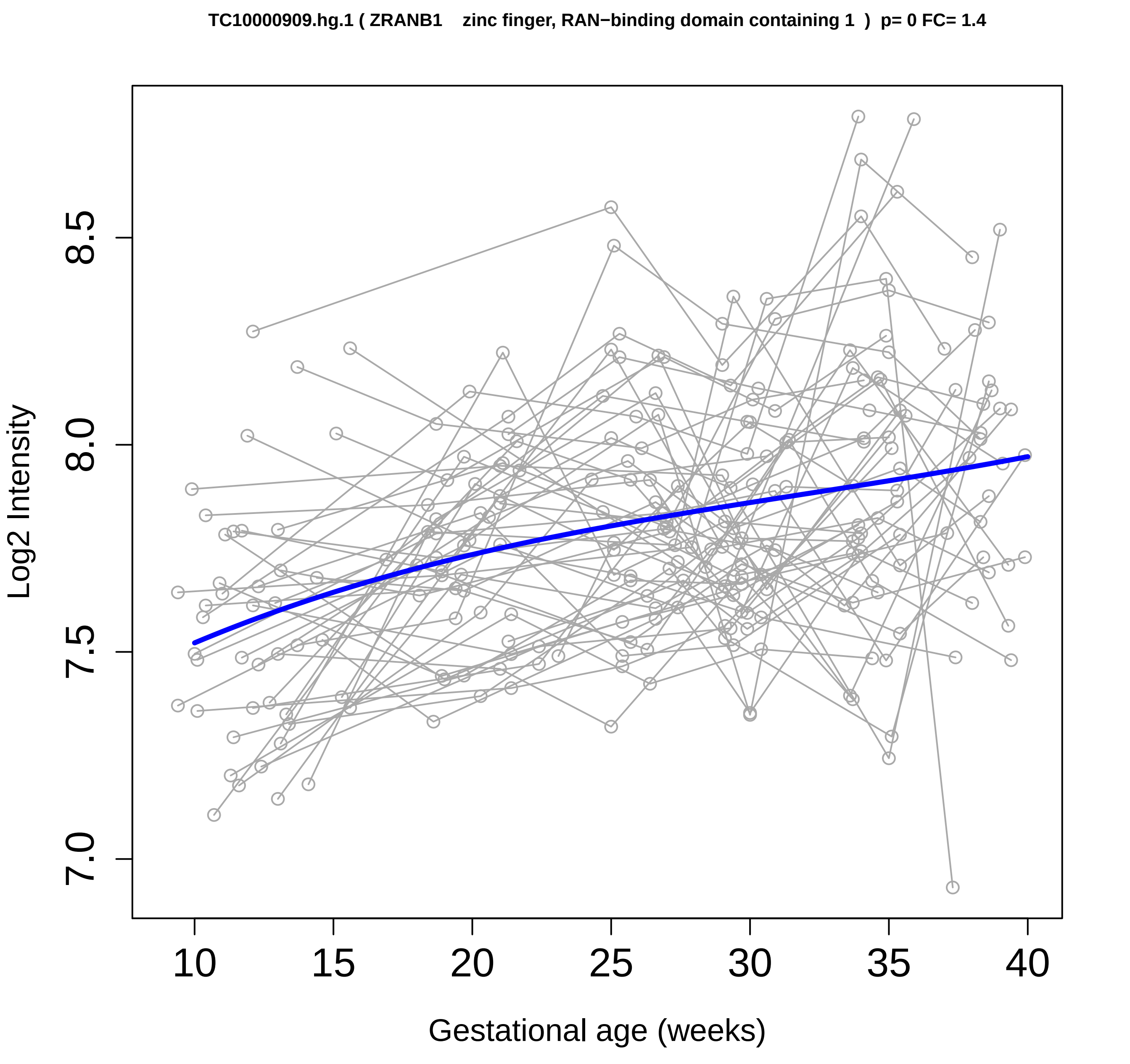
TC10000909.hg.1 (ZRANB1 zinc finger, RAN−binding domain containing 1) p= 0 FC= 1.4
Log2 Intensity
7.0
7.5
8.0
8.5
10
15
20
25
30
35
40
Gestational age (weeks)

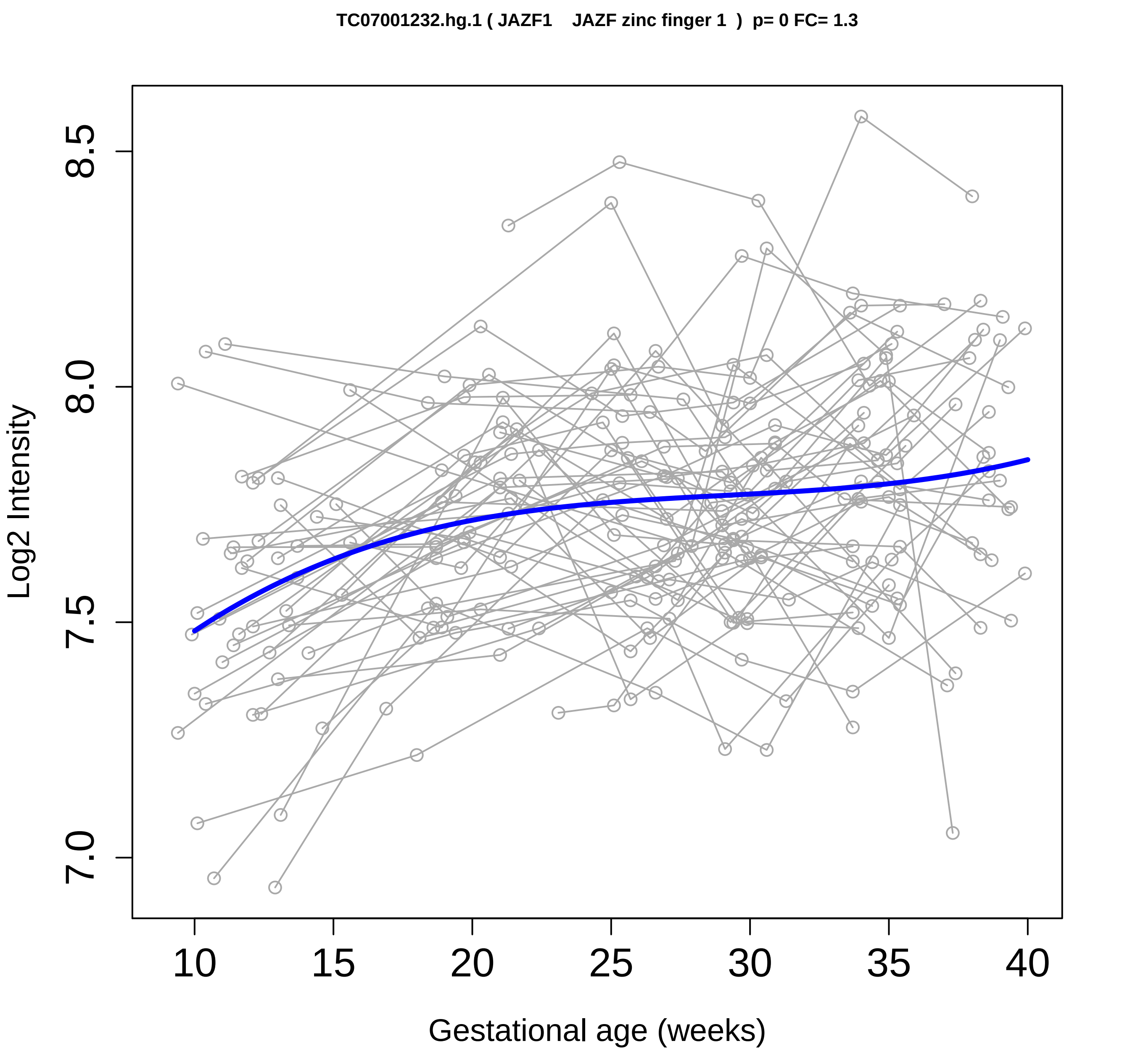

TC07001232.hg.1 (JAZF1 JAZF zinc finger 1) p= 0 FC= 1.3
Log2 Intensity
7.0
7.5
8.0
8.5
10
15
20
25
30
35
40
Gestational age (weeks)

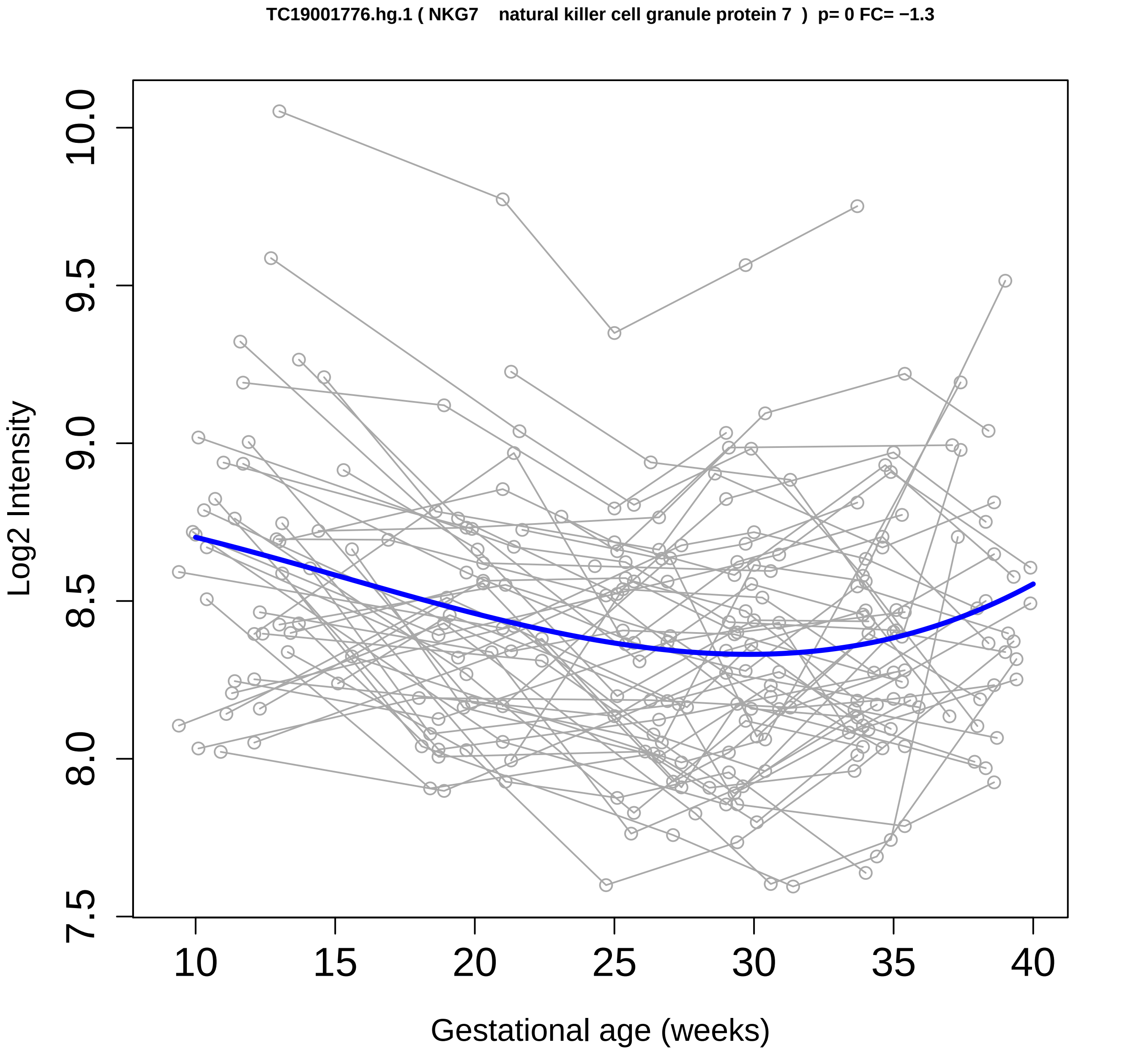
TC19001776.hg.1 (NKG7 natural killer cell granule protein 7) p= 0 FC= −1.3
Log2 Intensity
10.0
9.5
9.0
8.5
8.0
7.5
10
15
20
25
30
35
40
Gestational age (weeks)

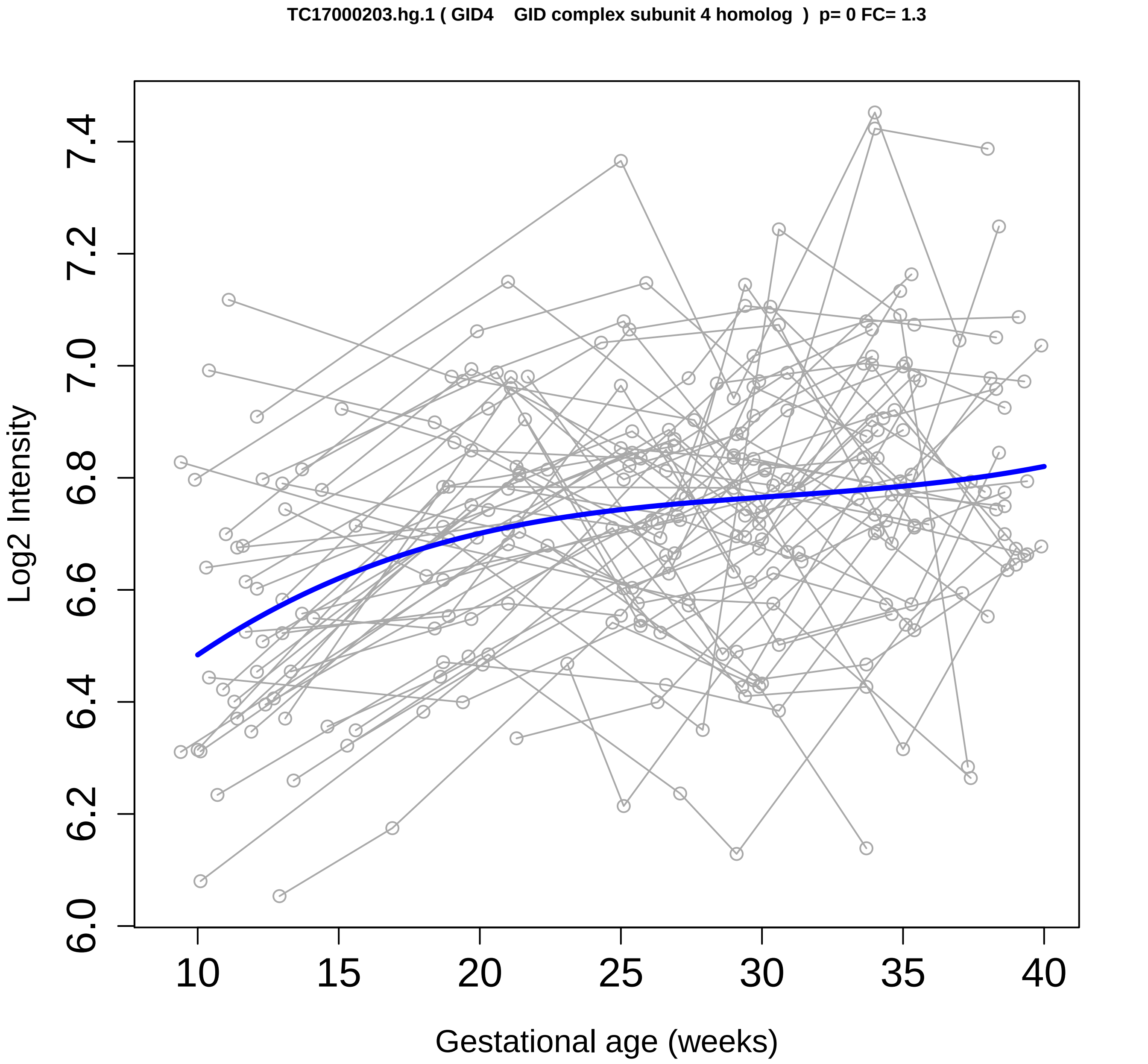
TC17000203.hg.1 (GID4 GID complex subunit 4 homolog) p= 0 FC= 1.3
Log2 Intensity
6.0
6.2
6.4
6.6
6.8
7.0
7.2
7.4
10
15
20
25
30
35
40
Gestational age (weeks)

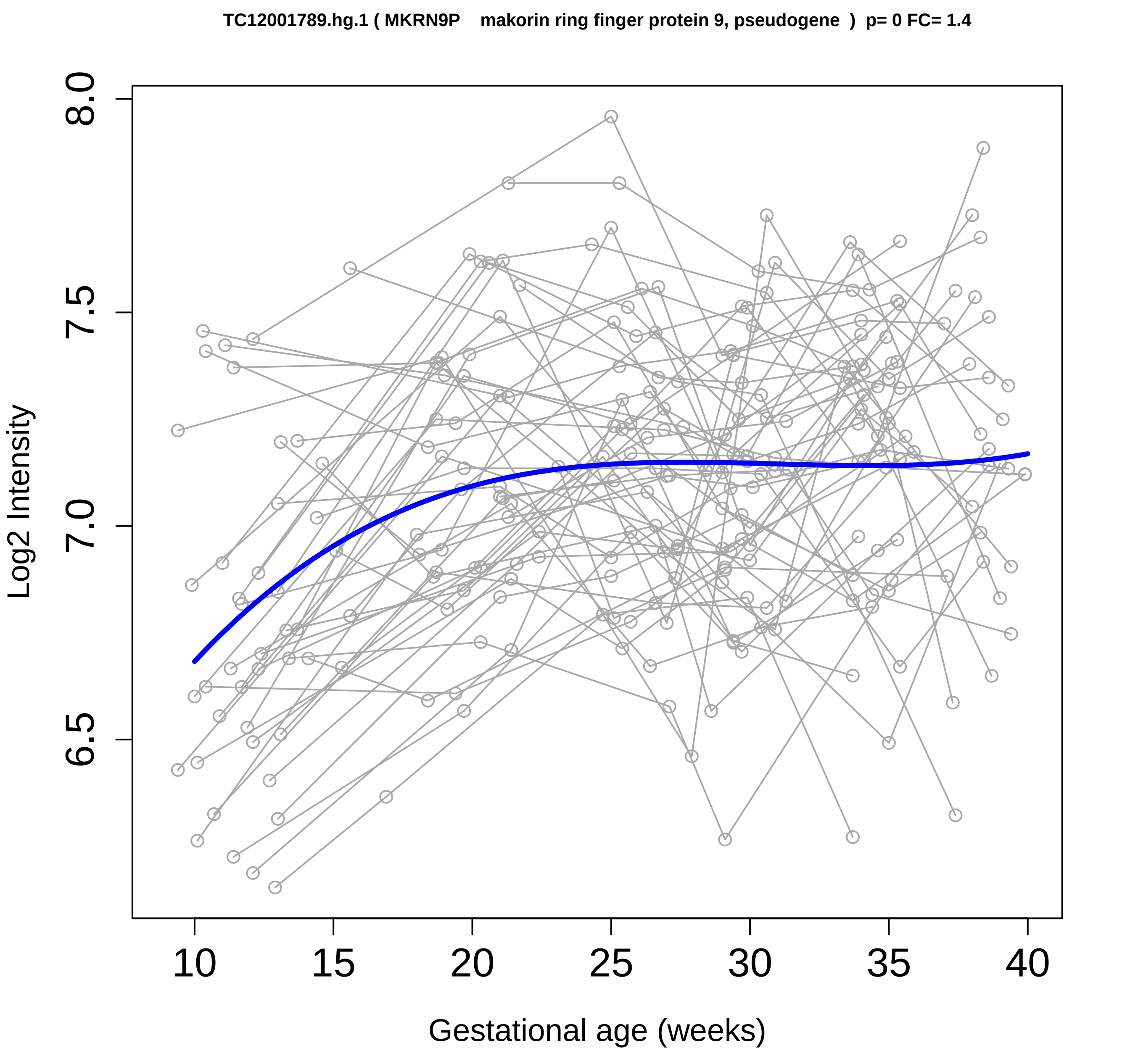

TC12001789.hg.1 (MKRN9P   makorin ring finger protein 9, pseudogene) p= 0 FC= 1.4
Log2 Intensity
8.0
7.5
7.0
6.5
10
15
20
25
30
35
40
Gestational age (weeks)

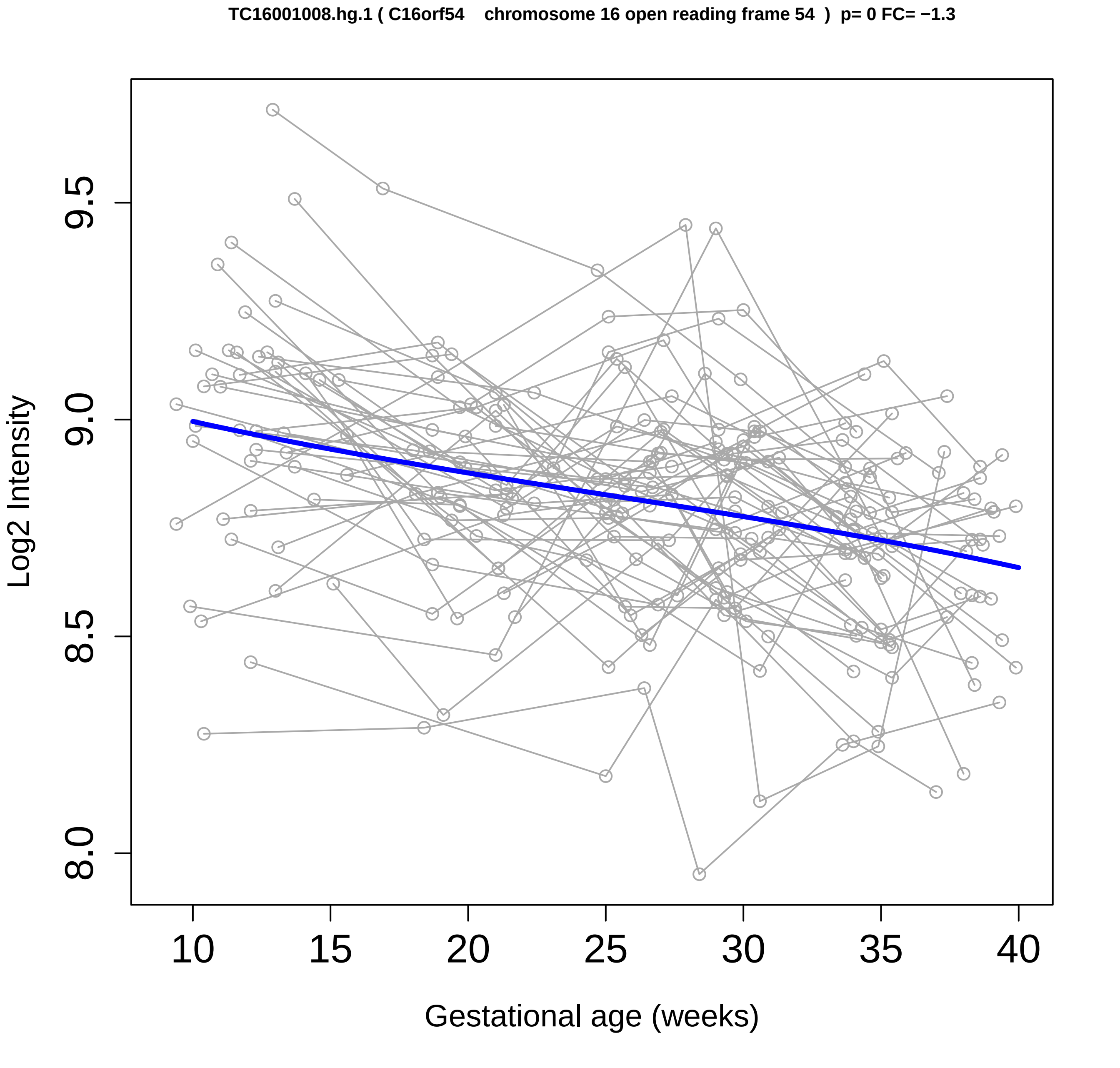
TC16001008.hg.1 (C16orf54 chromosome 16 open reading frame 54) p= 0 FC= −1.3
Log2 Intensity
9.5
9.0
8.5
8.0
10
15
20
25
30
35
40
Gestational age (weeks)

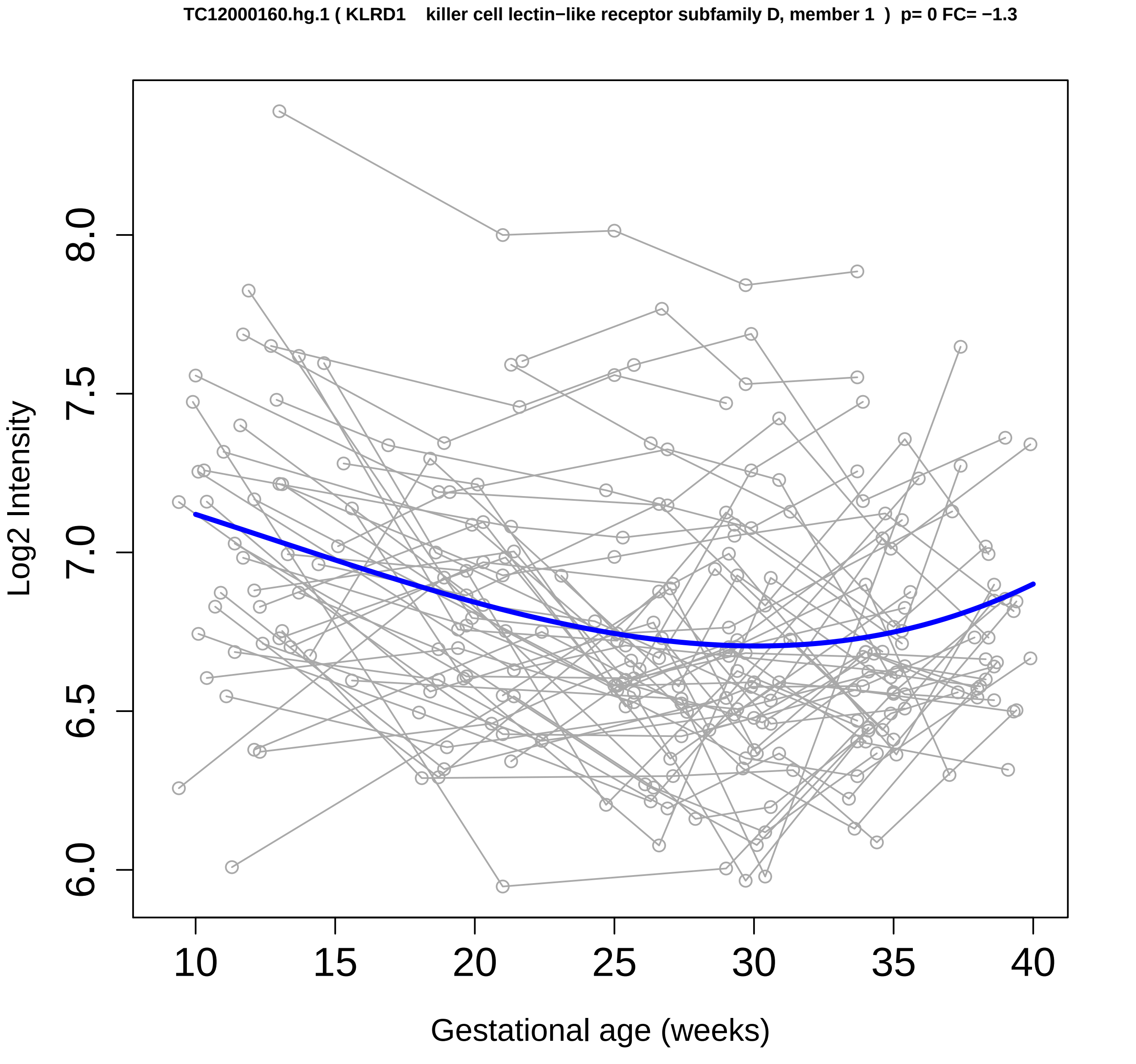
TC12000160.hg.1 (KLRD1   killer cell lectin−like receptor subfamily D, member 1) p= 0 FC= −1.3
Log2 Intensity
6.0
6.5
7.0
7.5
8.0
10
15
20
25
30
35
40
Gestational age (weeks)

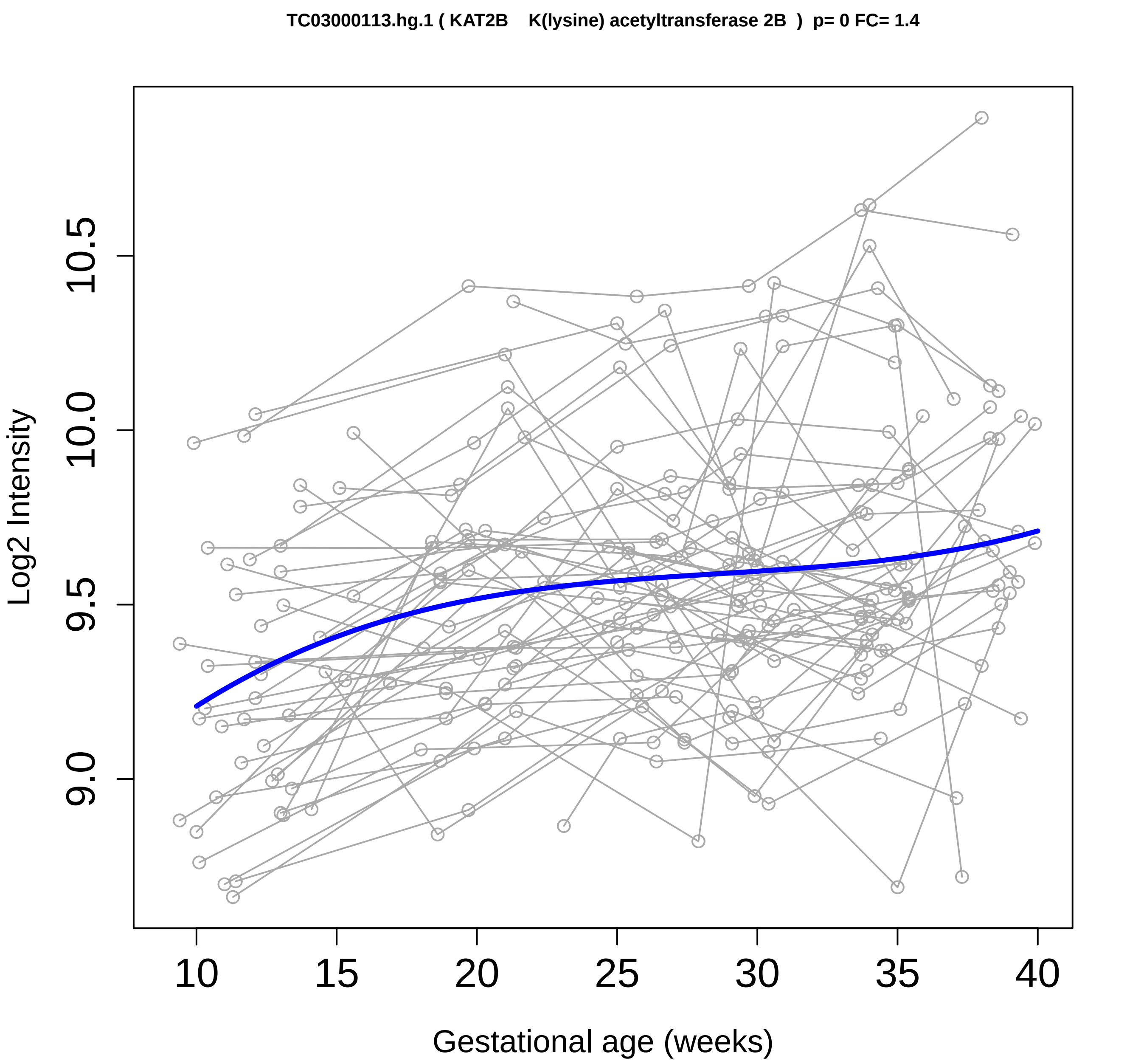

TC03000113.hg.1 (KAT2B K(lysine) acetyltransferase 2B) p= 0 FC= 1.4
Log2 Intensity
9.0
9.5
10.0
10.5
10
15
20
25
30
35
40
Gestational age (weeks)

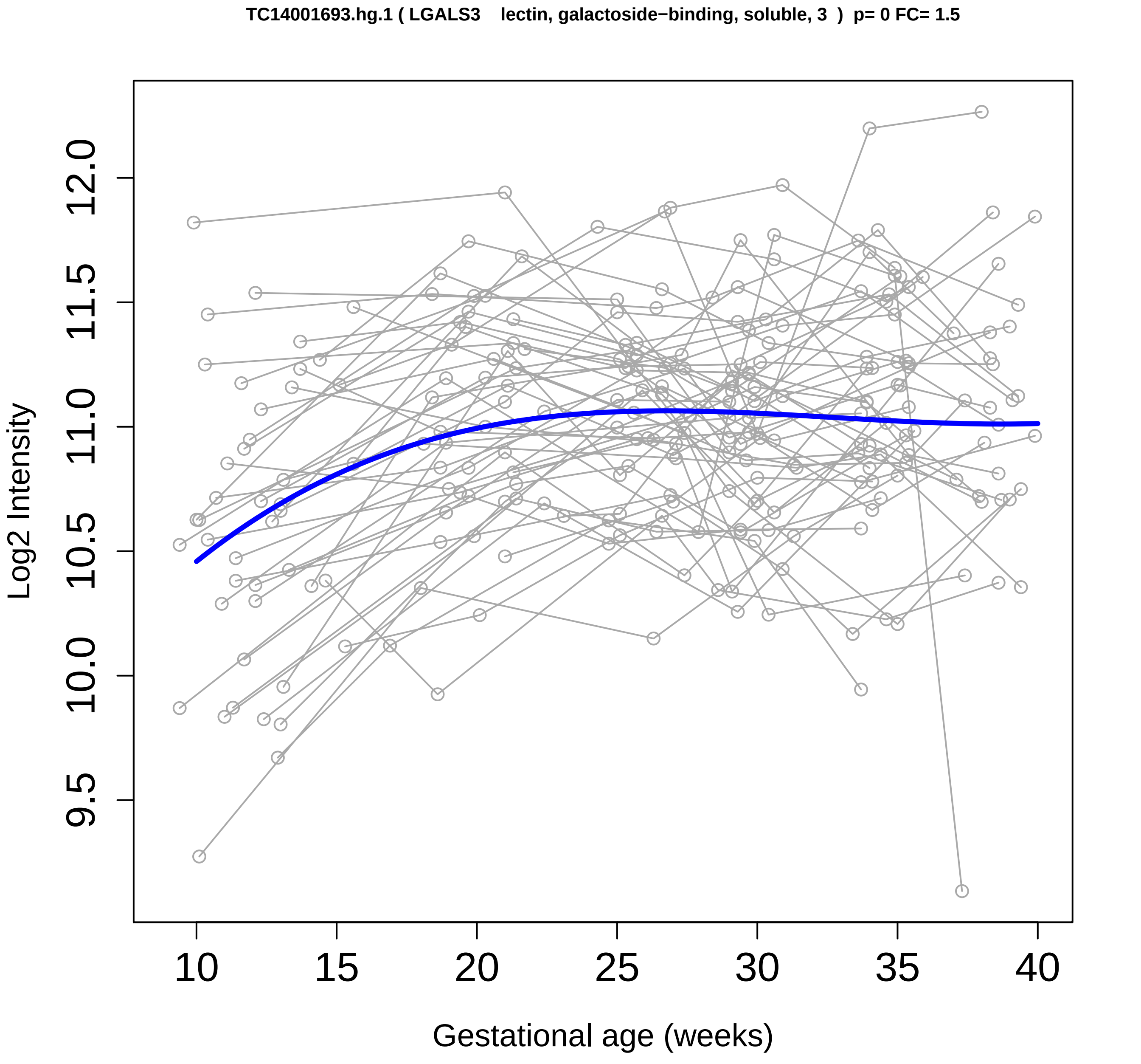

TC14001693.hg.1 (LGALS3 lectin, galactoside−binding, soluble, 3) p= 0 FC= 1.5
Log2 Intensity
9.5
10.0
10.5
11.0
11.5
12.0
10
15
20
25
30
35
40
Gestational age (weeks)

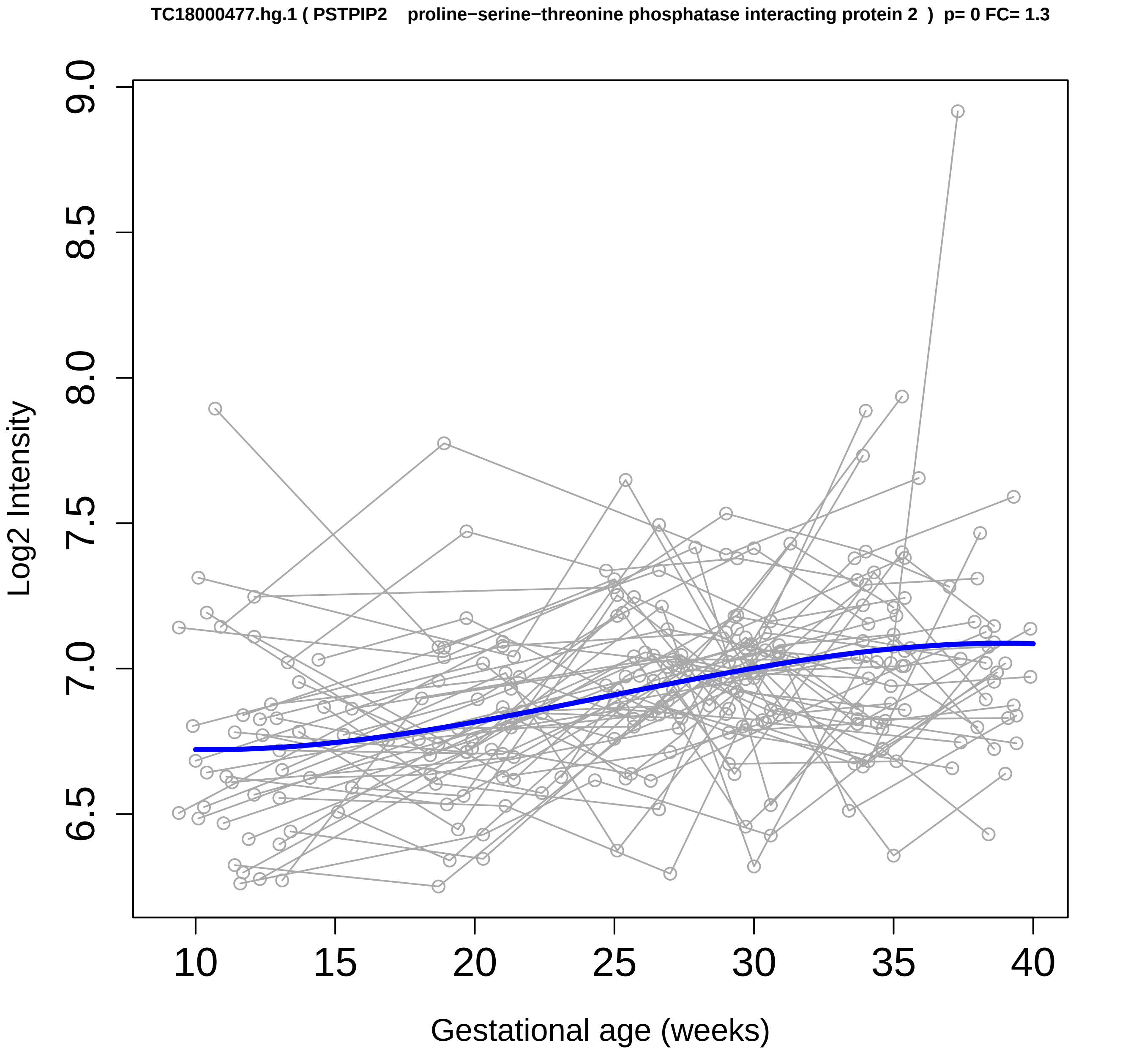

TC18000477.hg.1 (PSTPIP2   proline−serine−threonine phosphatase interacting protein 2) p= 0 FC= 1.3
Log2 Intensity
9.0
8.5
8.0
7.5
7.0
6.5
10
15
20
25
30
35
40
Gestational age (weeks)

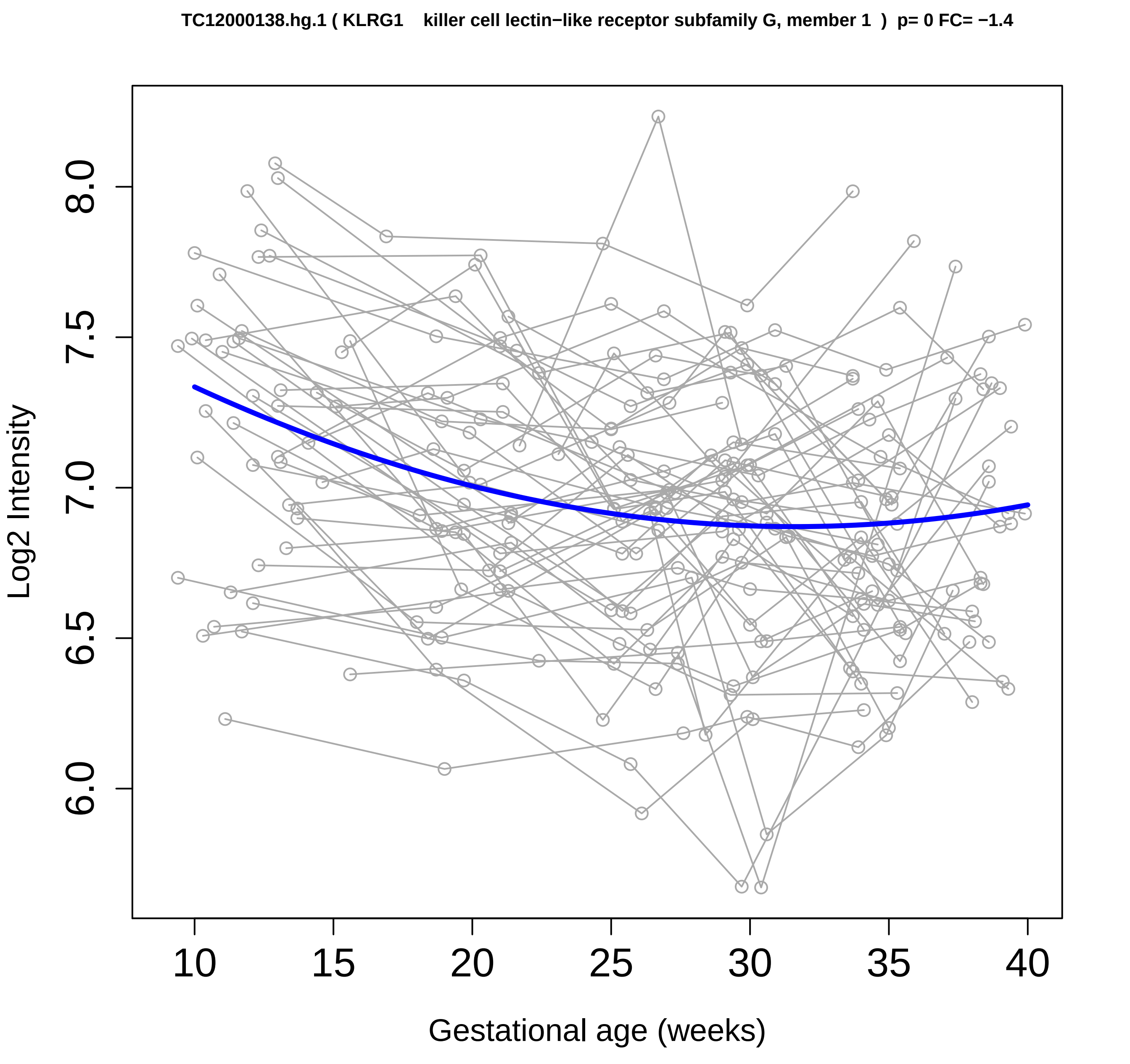

TC12000138.hg.1 (KLRG1 killer cell lectin−like receptor subfamily G, member 1) p= 0 FC= −1.4
Log2 Intensity
6.0
6.5
7.0
7.5
8.0
10
15
20
25
30
35
40
Gestational age (weeks)

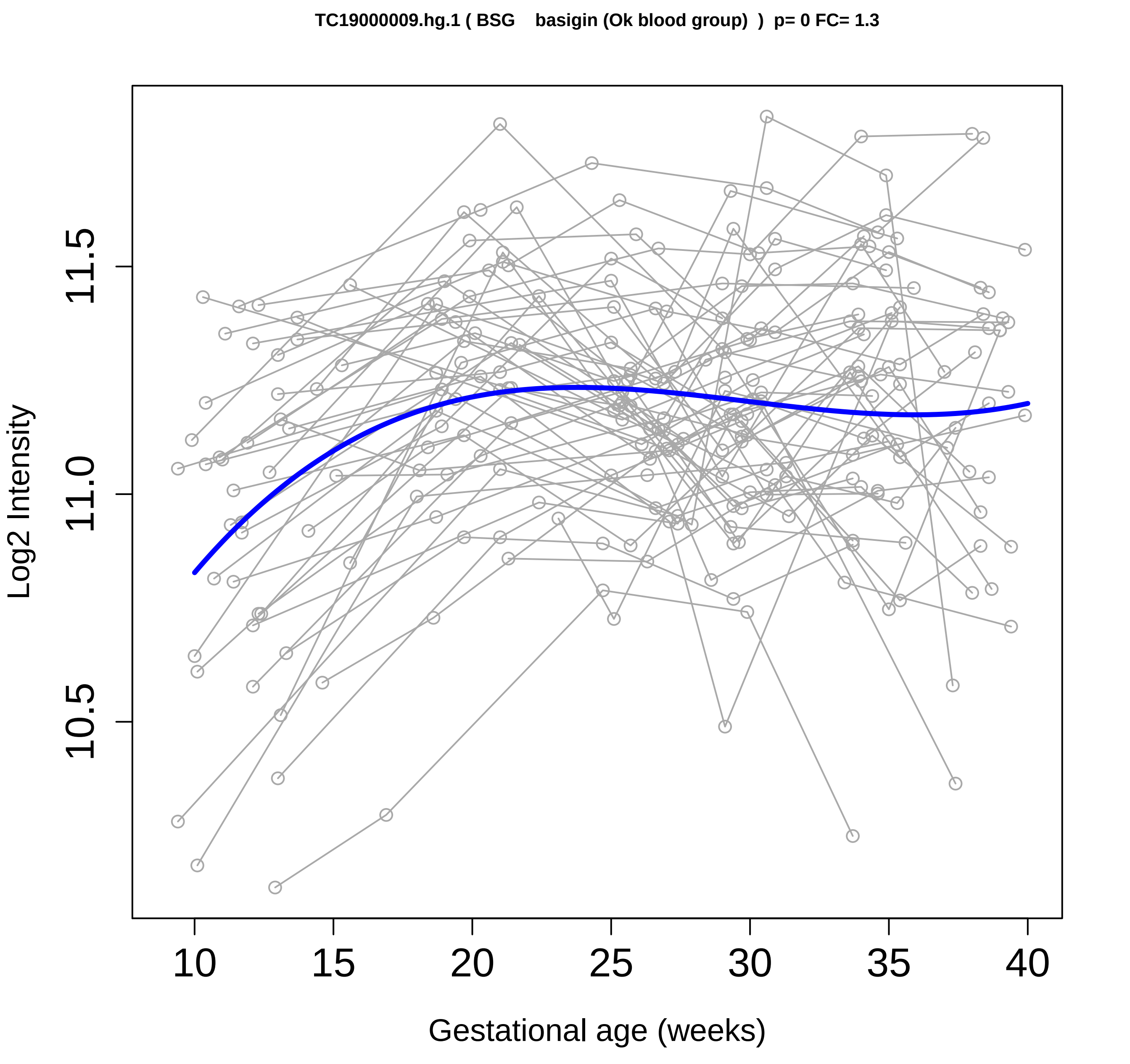
TC19000009.hg.1 (BSG   basigin (Ok blood group)) p= 0 FC= 1.3
Log2 Intensity
10.5
11.0
11.5
10
15
20
25
30
35
40
Gestational age (weeks)

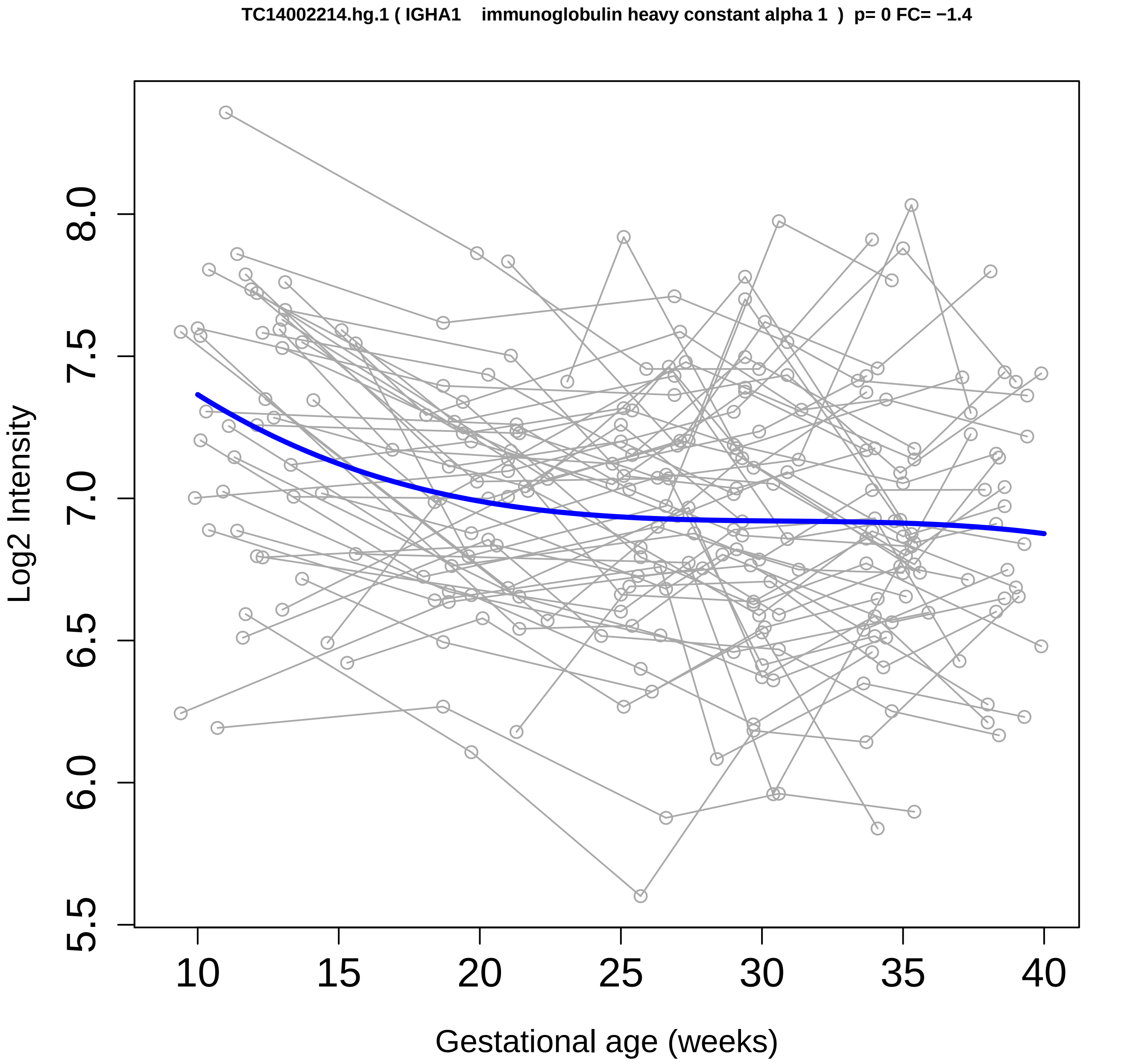

TC14002214.hg.1 (IGHA1 immunoglobulin heavy constant alpha 1) p= 0 FC= −1.4
Log2 Intensity
5.5
6.0
6.5
7.0
7.5
8.0
10
15
20
25
30
35
40
Gestational age (weeks)

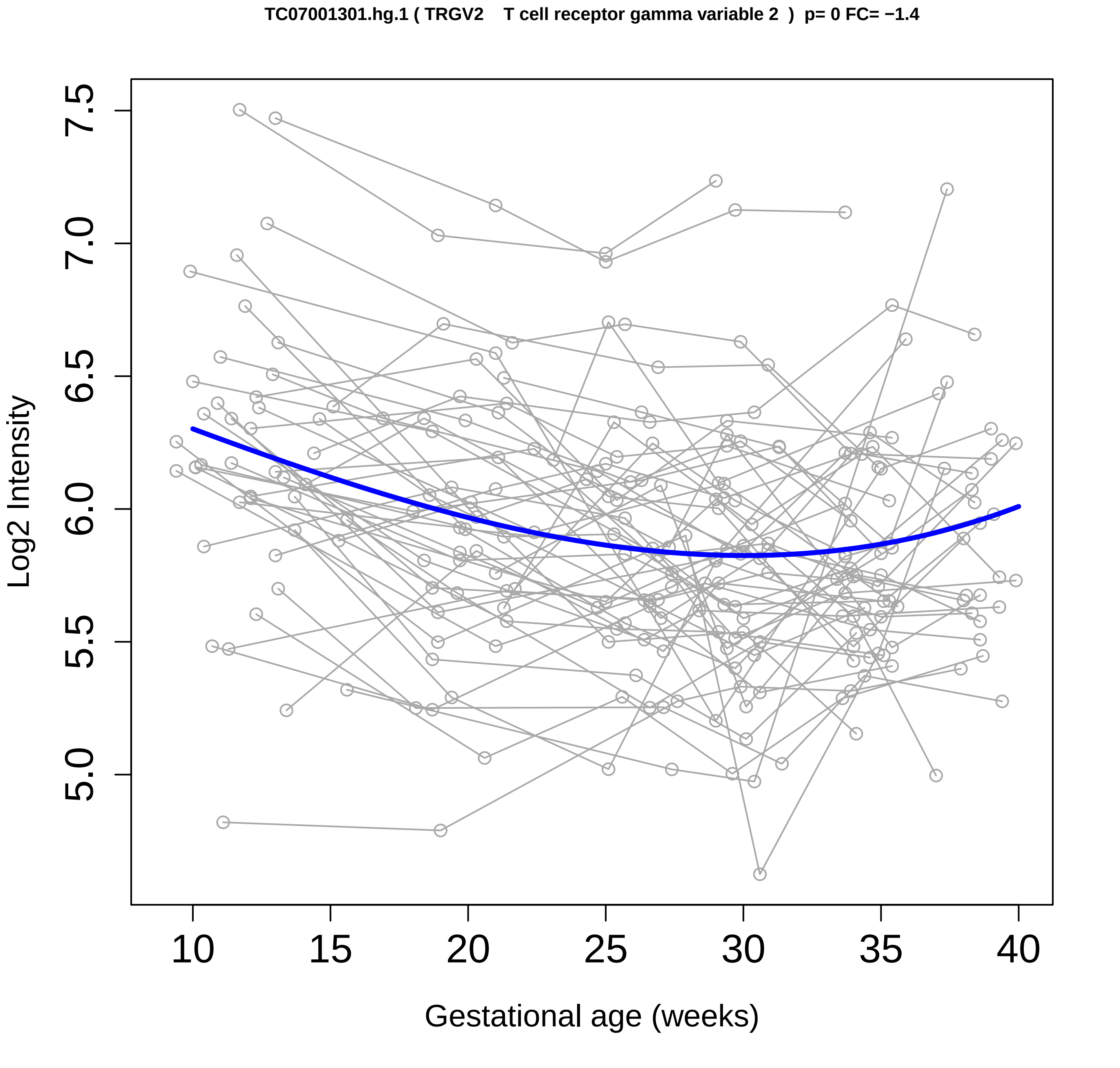
TC07001301.hg.1 (TRGV2 T cell receptor gamma variable 2) p= 0 FC= −1.4
Log2 Intensity
7.5
7.0
6.5
6.0
5.5
5.0
10
15
20
25
30
35
40
Gestational age (weeks)

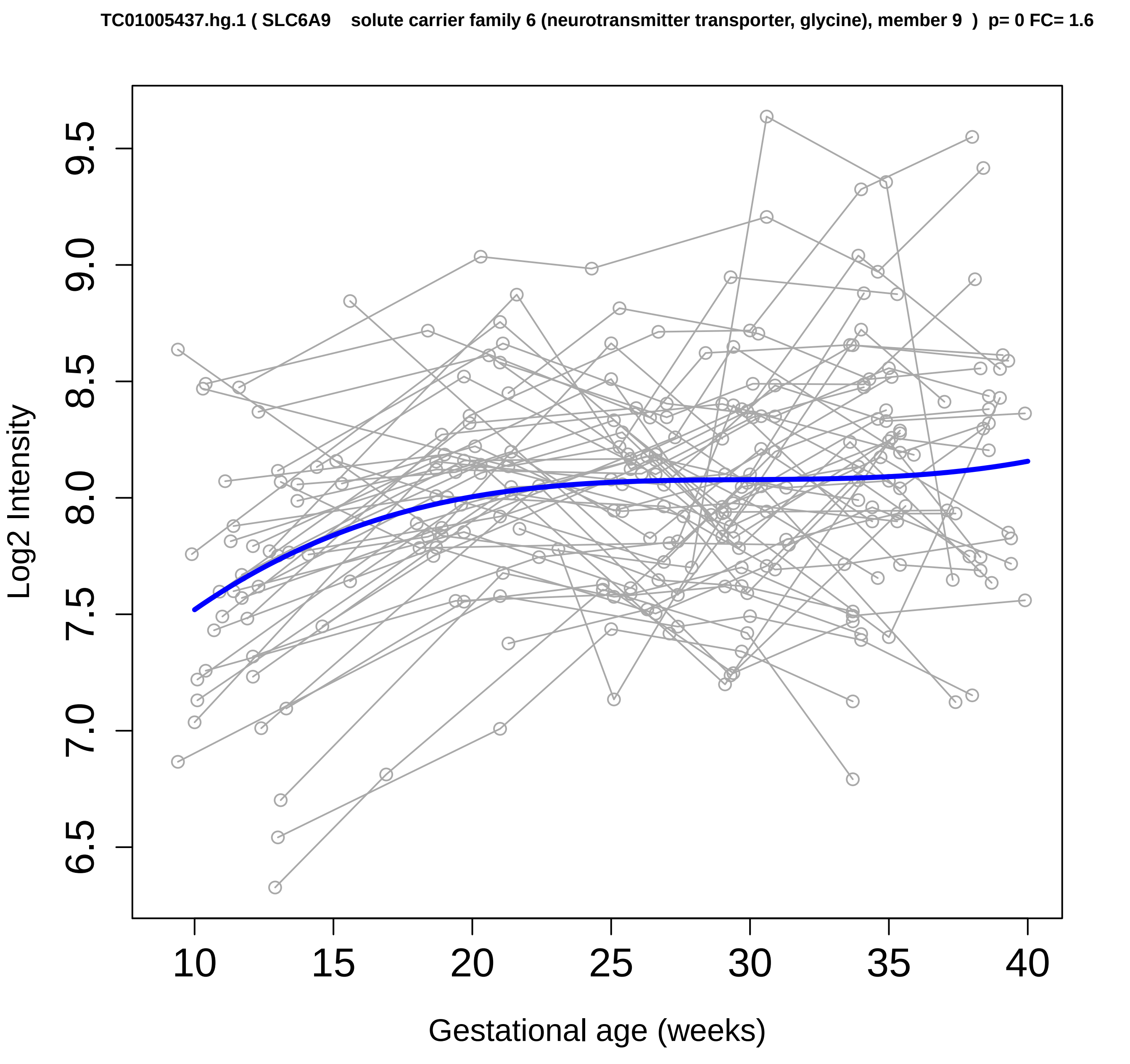
TC01005437.hg.1 (SLC6A9 solute carrier family 6 (neurotransmitter transporter, glycine), member 9) p= 0 FC= 1.6
Log2 Intensity
6.5
7.0
7.5
8.0
8.5
9.0
9.5
10
15
20
25
30
35
40
Gestational age (weeks)

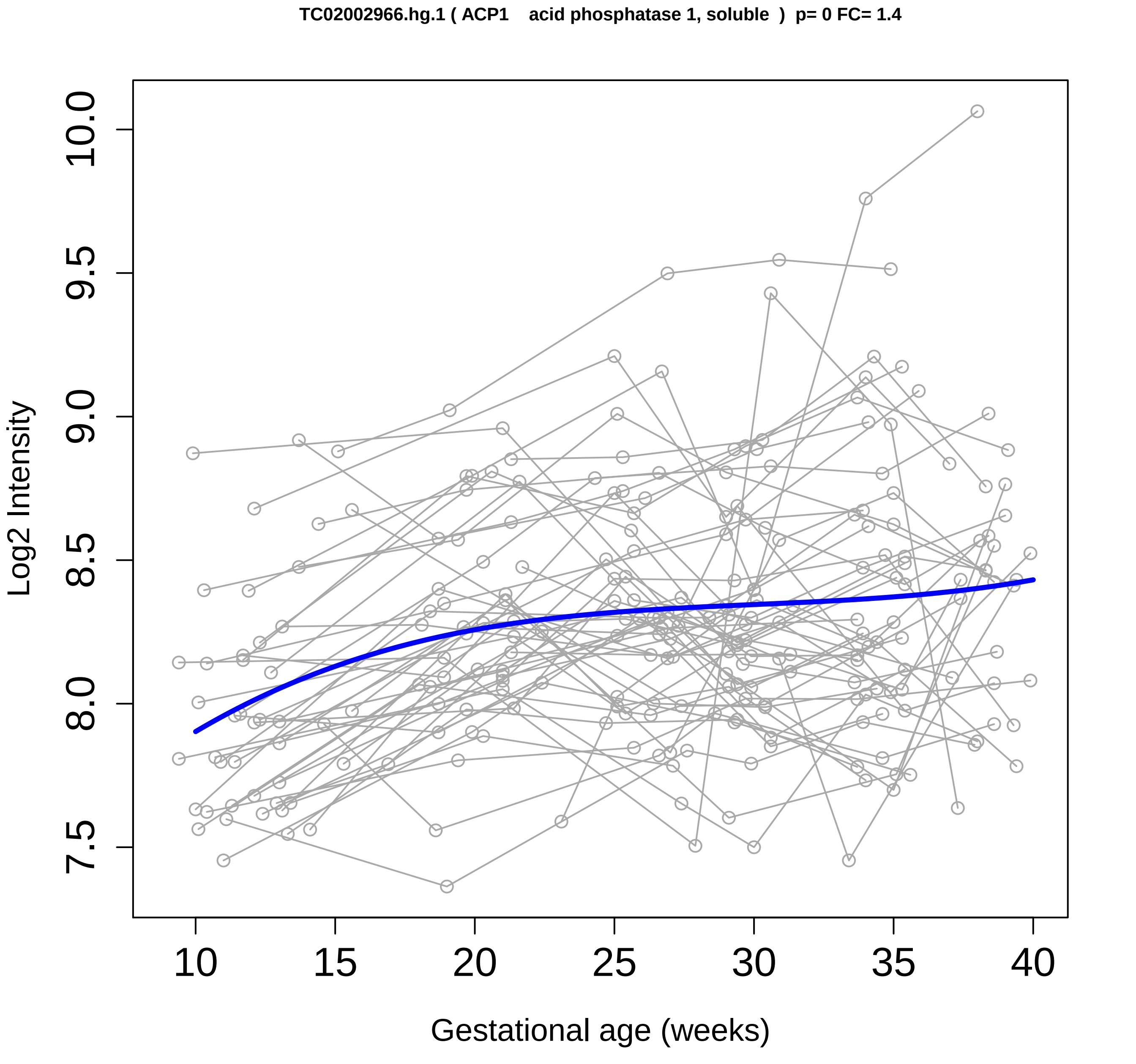

TC02002966.hg.1 (ACP1 acid phosphatase 1, soluble) p= 0 FC= 1.4
Log2 Intensity
10.0
9.5
9.0
8.5
8.0
7.5
10
15
20
25
30
35
40
Gestational age (weeks)

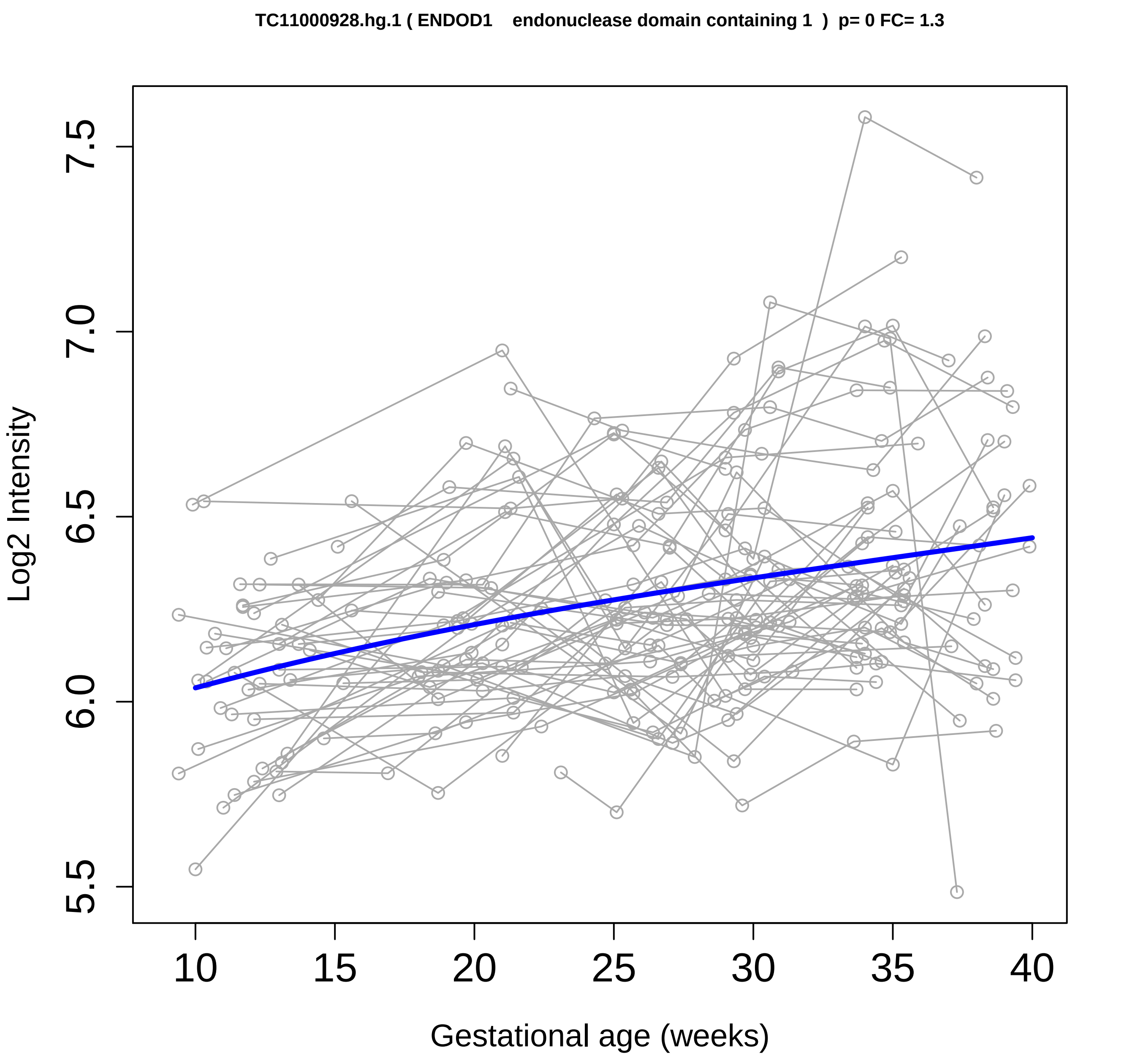

TC11000928.hg.1 (ENDOD1 endonuclease domain containing 1) p= 0 FC= 1.3
Log2 Intensity
5.5
6.0
6.5
7.0
7.5
10
15
20
25
30
35
40
Gestational age (weeks)

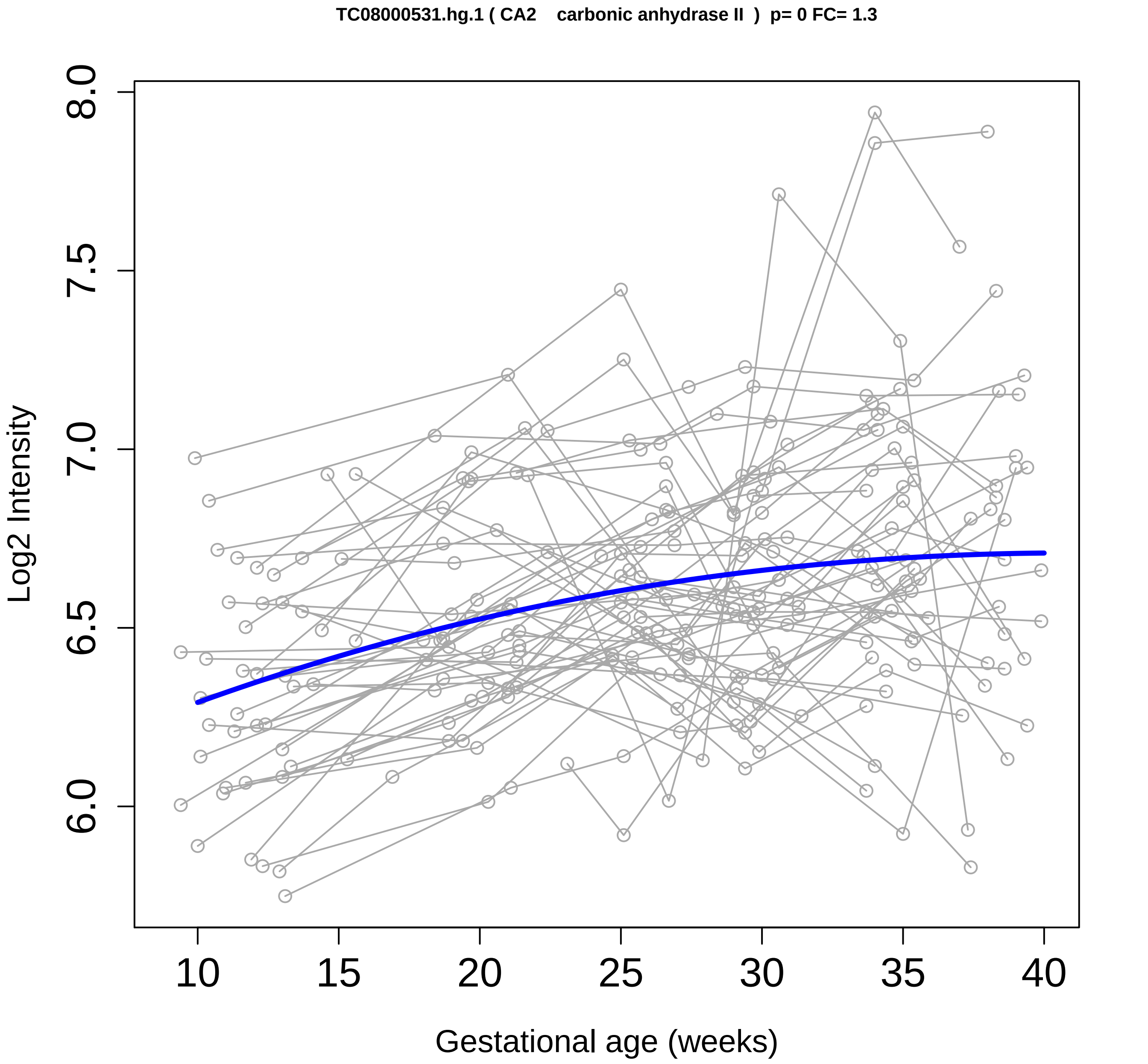

TC08000531.hg.1 (CA2 carbonic anhydrase II) p= 0 FC= 1.3
Log2 Intensity
6.0
6.5
7.0
7.5
8.0
10
15
20
25
30
35
40
Gestational age (weeks)

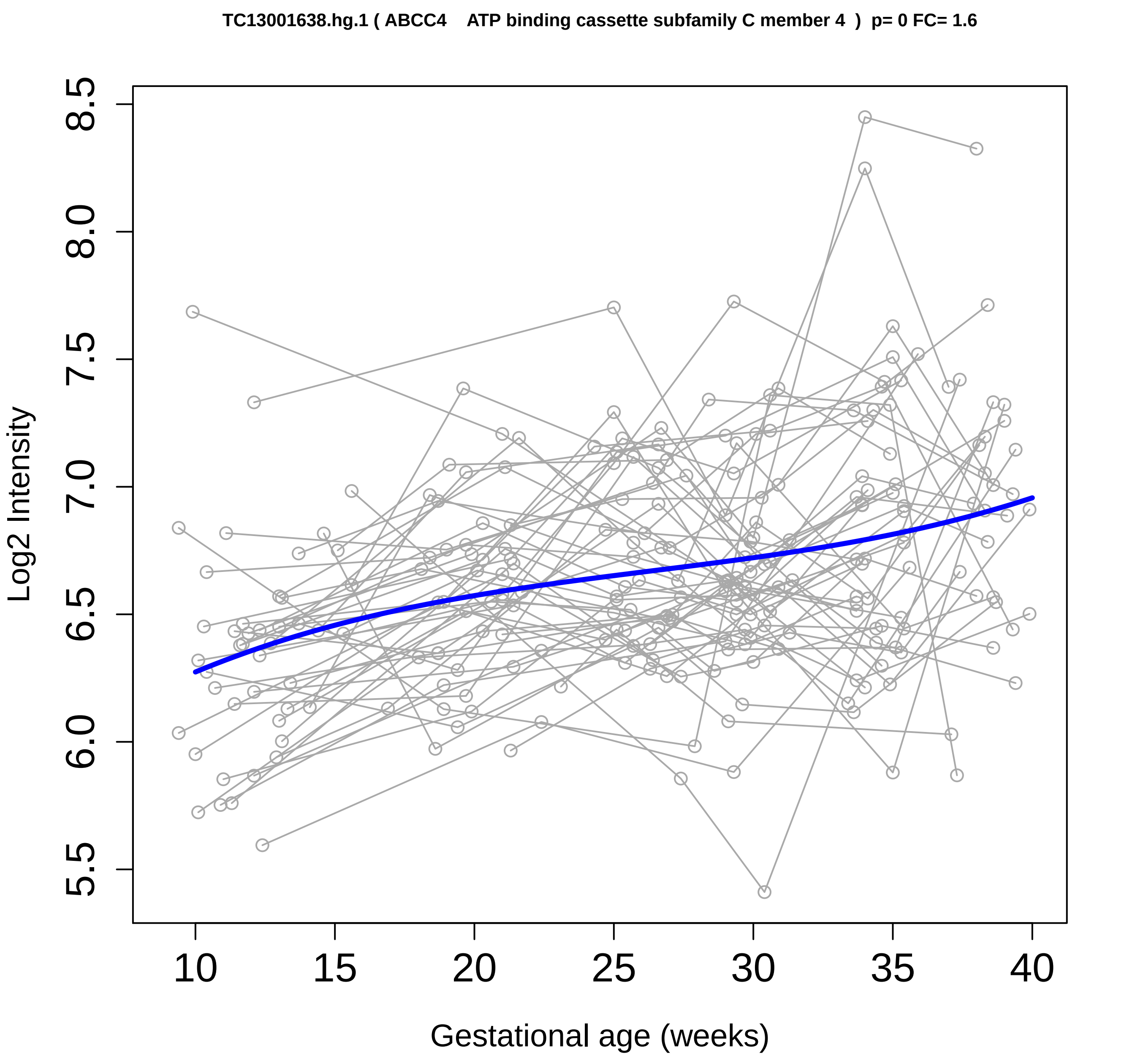
TC13001638.hg.1 (ABCC4 ATP binding cassette subfamily C member 4) p= 0 FC= 1.6
Log2 Intensity
5.5
6.0
6.5
7.0
7.5
8.0
8.5
10
15
20
25
30
35
40
Gestational age (weeks)

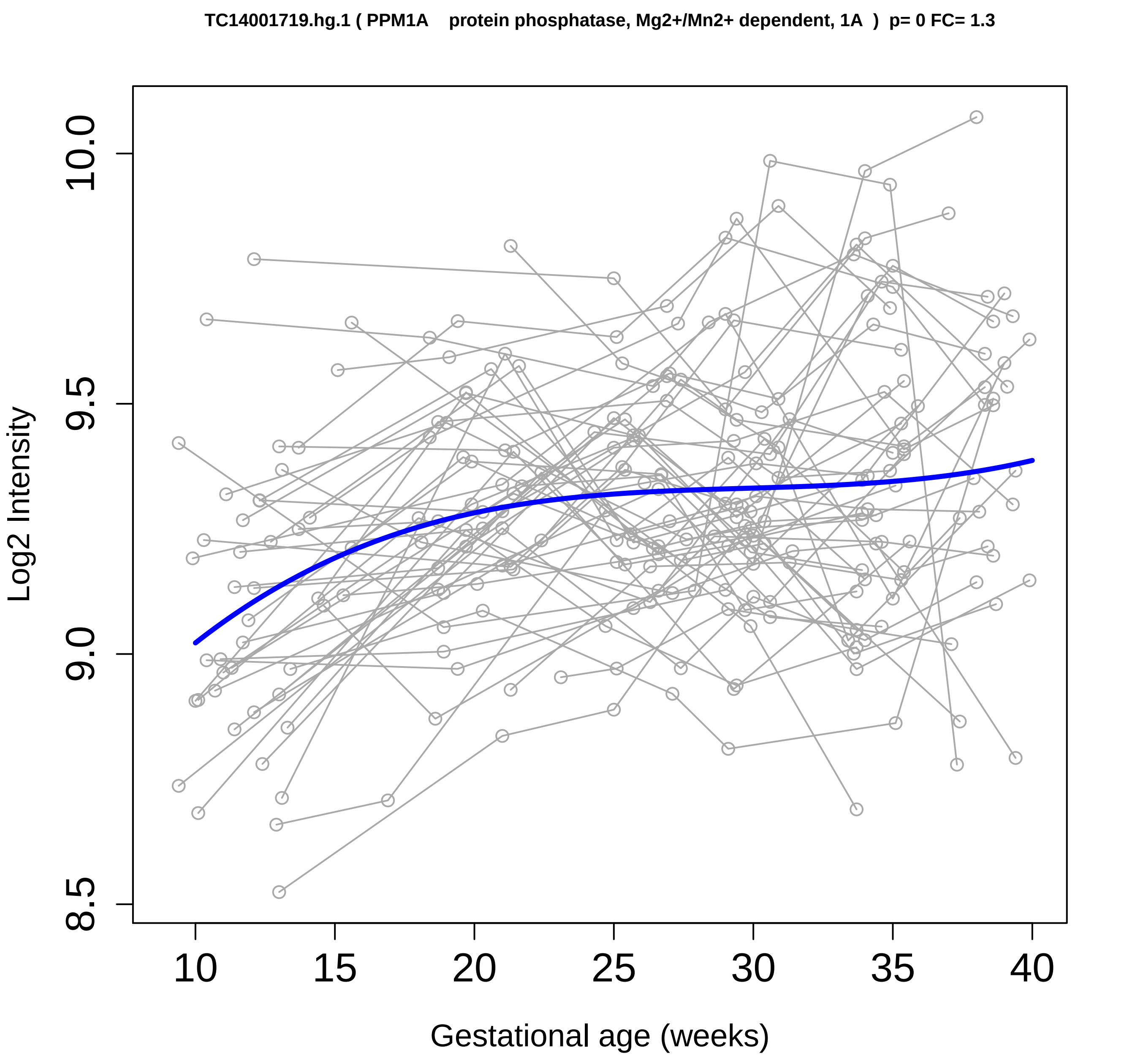

TC14001719.hg.1 (PPM1A protein phosphatase, Mg2+/Mn2+ dependent, 1A) p= 0 FC= 1.3
Log2 Intensity
8.5
9.0
9.5
10.0
10
15
20
25
30
35
40
Gestational age (weeks)

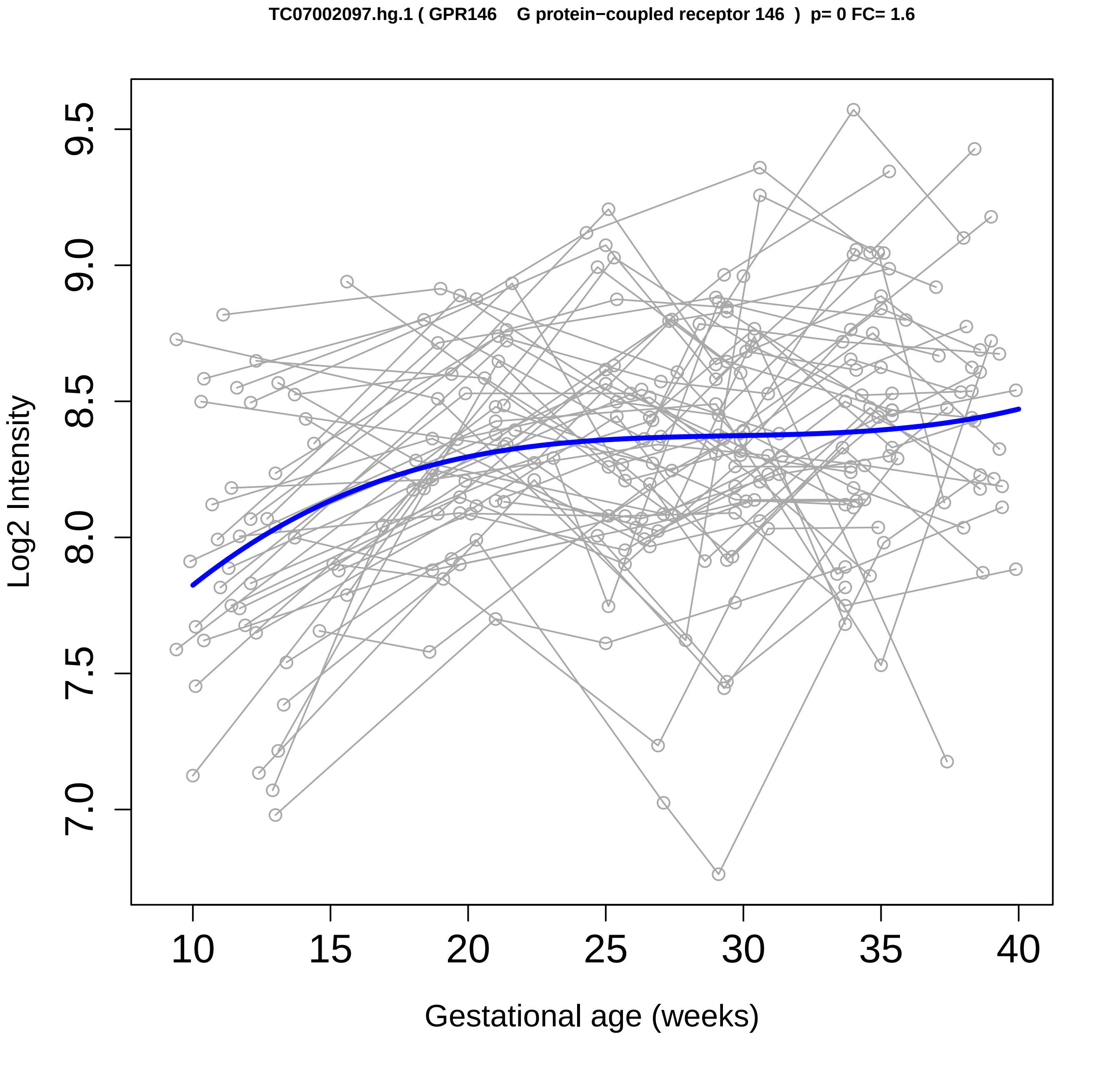

TC07002097.hg.1 (GPR146   G protein−coupled receptor 146) p= 0 FC= 1.6
Log2 Intensity
7.0
7.5
8.0
8.5
9.0
9.5
10
15
20
25
30
35
40
Gestational age (weeks)

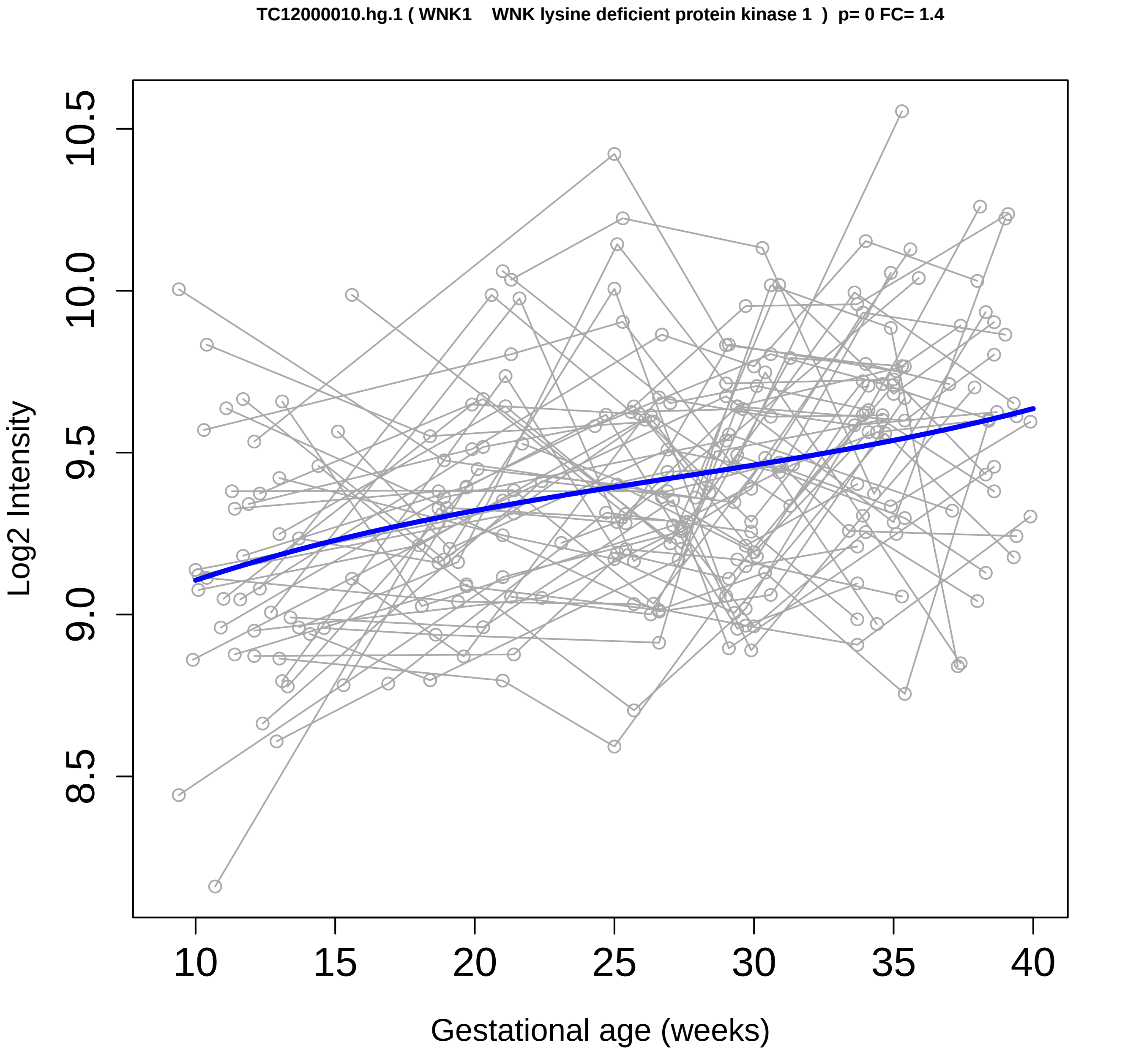

TC12000010.hg.1 (WNK1   WNK lysine deficient protein kinase 1) p= 0 FC= 1.4
Log2 Intensity
10.5
10.0
9.5
9.0
8.5
10
15
20
25
30
35
40
Gestational age (weeks)

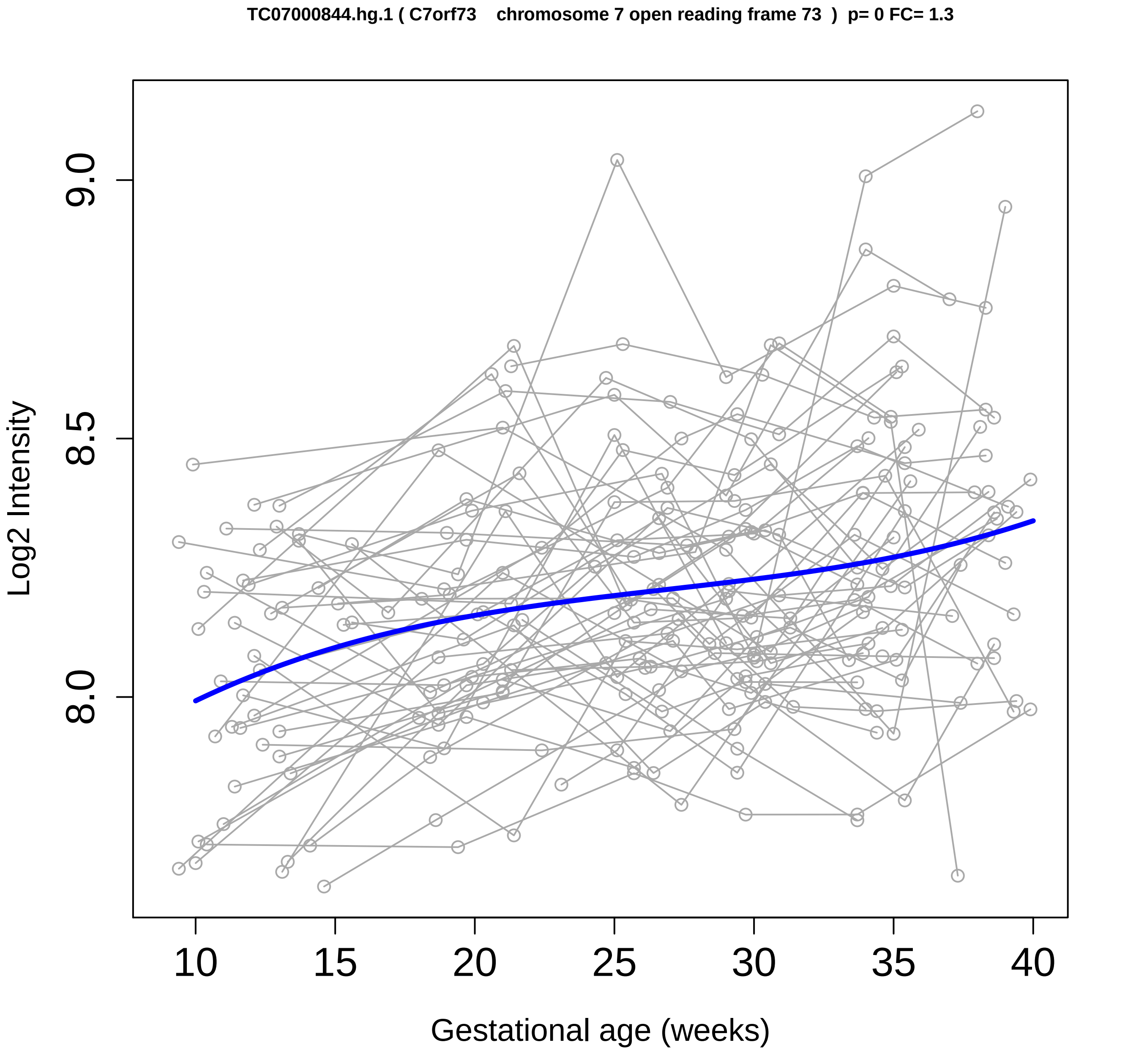
TC07000844.hg.1 (C7orf73 chromosome 7 open reading frame 73) p= 0 FC= 1.3
Log2 Intensity
9.0
8.5
8.0
10
15
20
25
30
35
40
Gestational age (weeks)

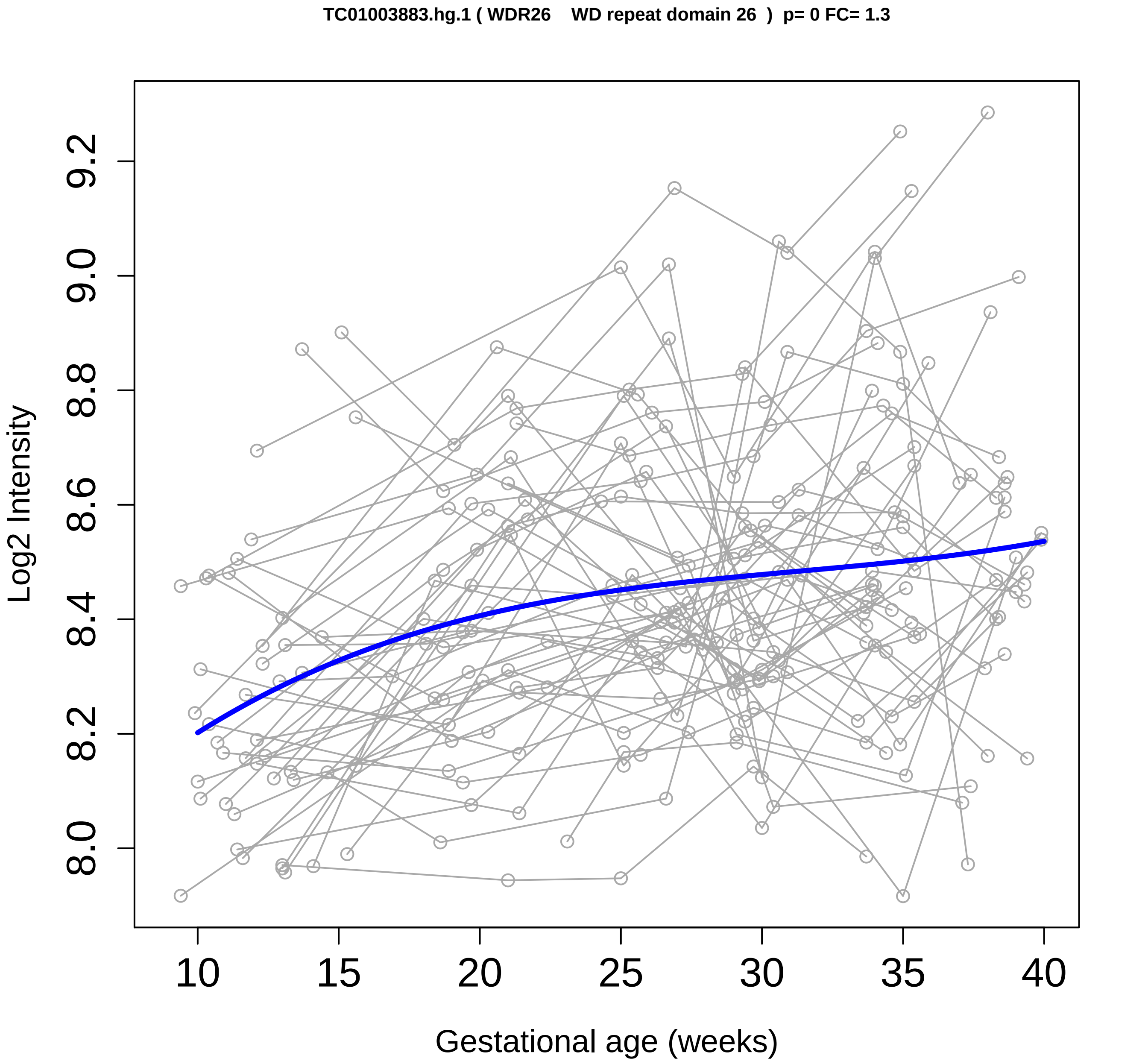
TC01003883.hg.1 (WDR26 WD repeat domain 26) p= 0 FC= 1.3
Log2 Intensity
8.0
8.2
8.4
8.6
8.8
9.0
9.2
10
15
20
25
30
35
40
Gestational age (weeks)

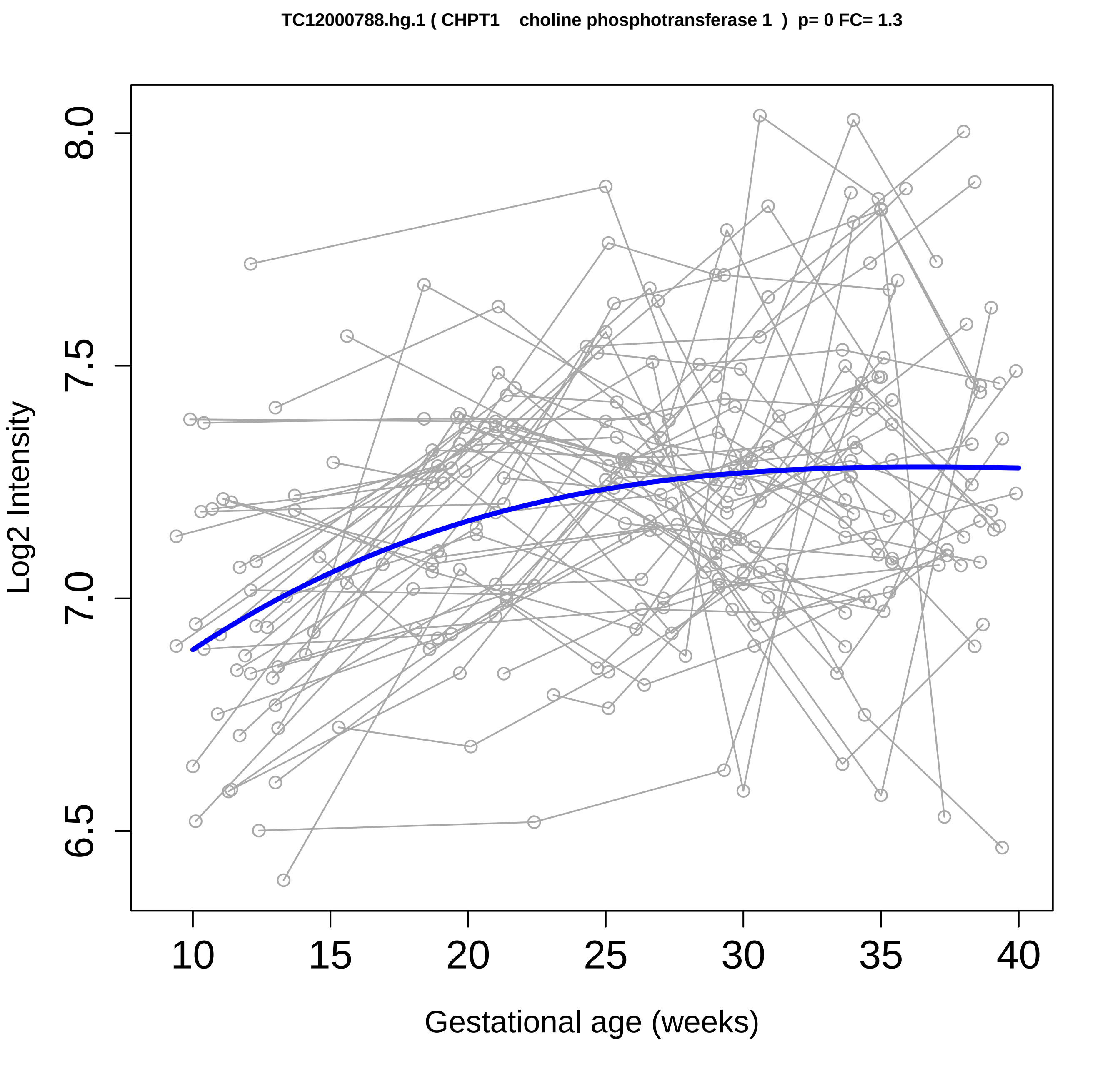

TC12000788.hg.1 (CHPT1 choline phosphotransferase 1) p= 0 FC= 1.3
Log2 Intensity
8.0
7.5
7.0
6.5
10
15
20
25
30
35
40
Gestational age (weeks)

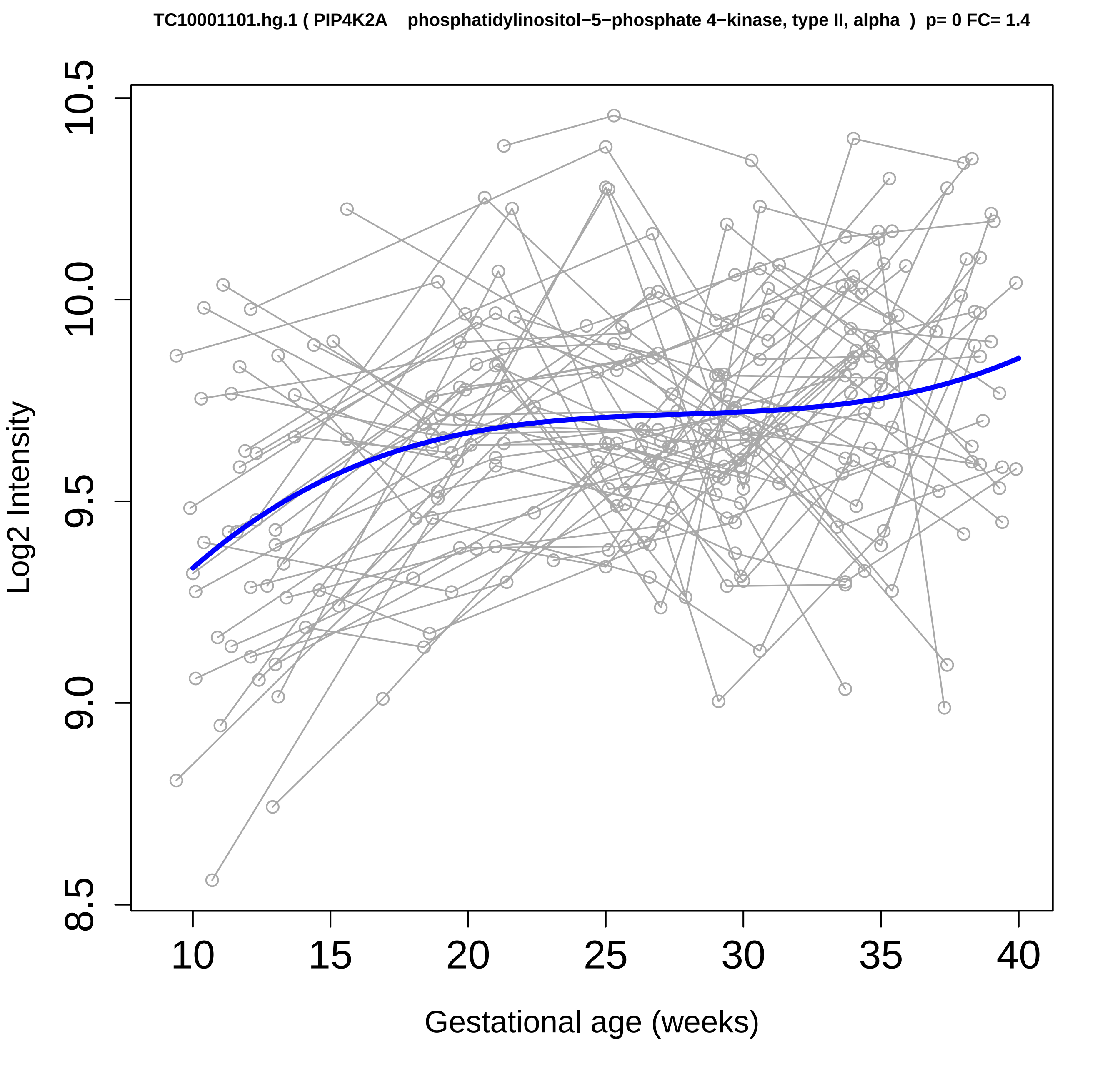

TC10001101.hg.1 (PIP4K2A phosphatidylinositol−5−phosphate 4−kinase, type II, alpha) p= 0 FC= 1.4
Log2 Intensity
8.5
9.0
9.5
10.0
10.5
10
15
20
25
30
35
40
Gestational age (weeks)

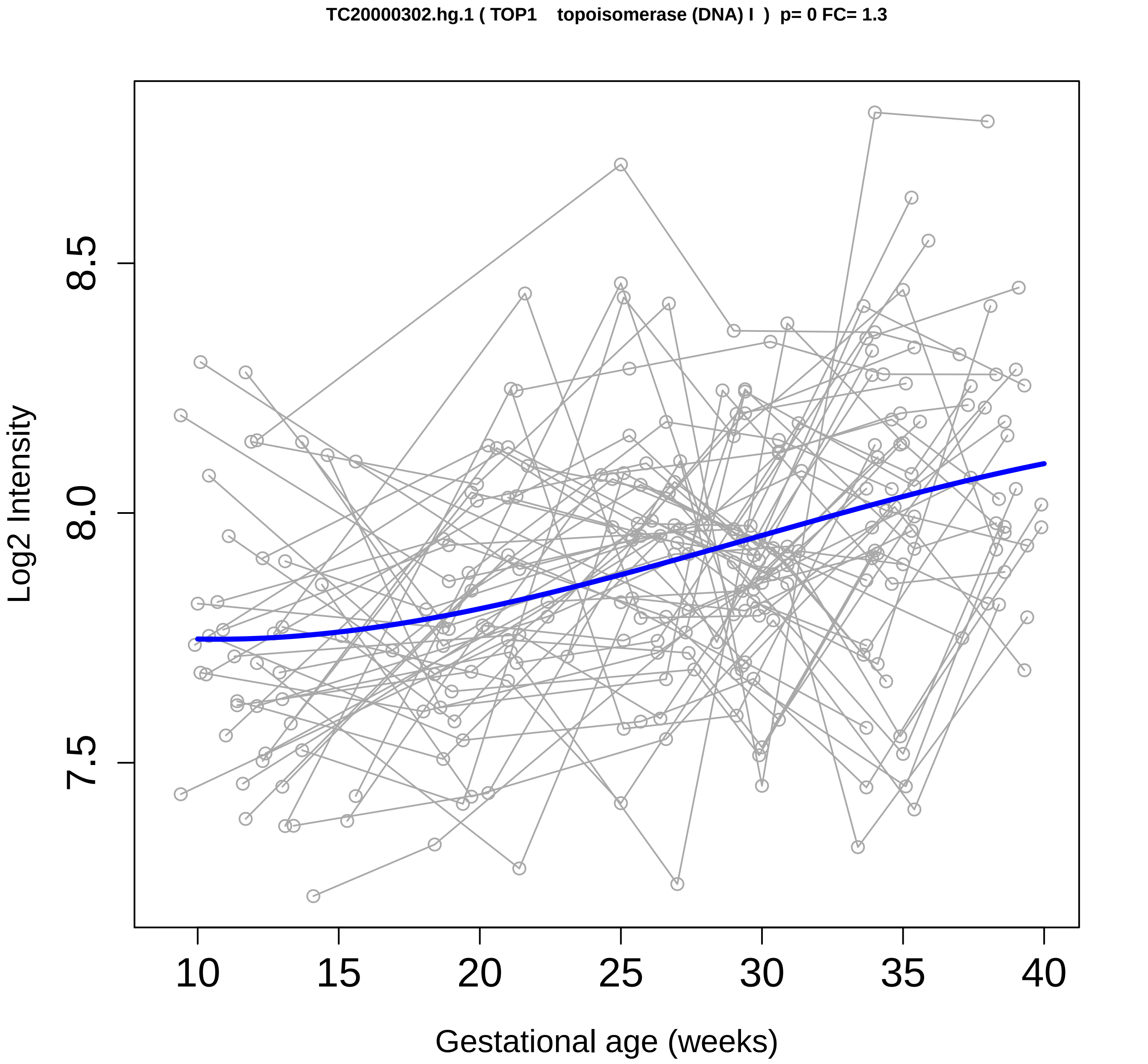
TC20000302.hg.1 (TOP1   topoisomerase (DNA) I) p= 0 FC= 1.3
Log2 Intensity
8.5
8.0
7.5
10
15
20
25
30
35
40
Gestational age (weeks)

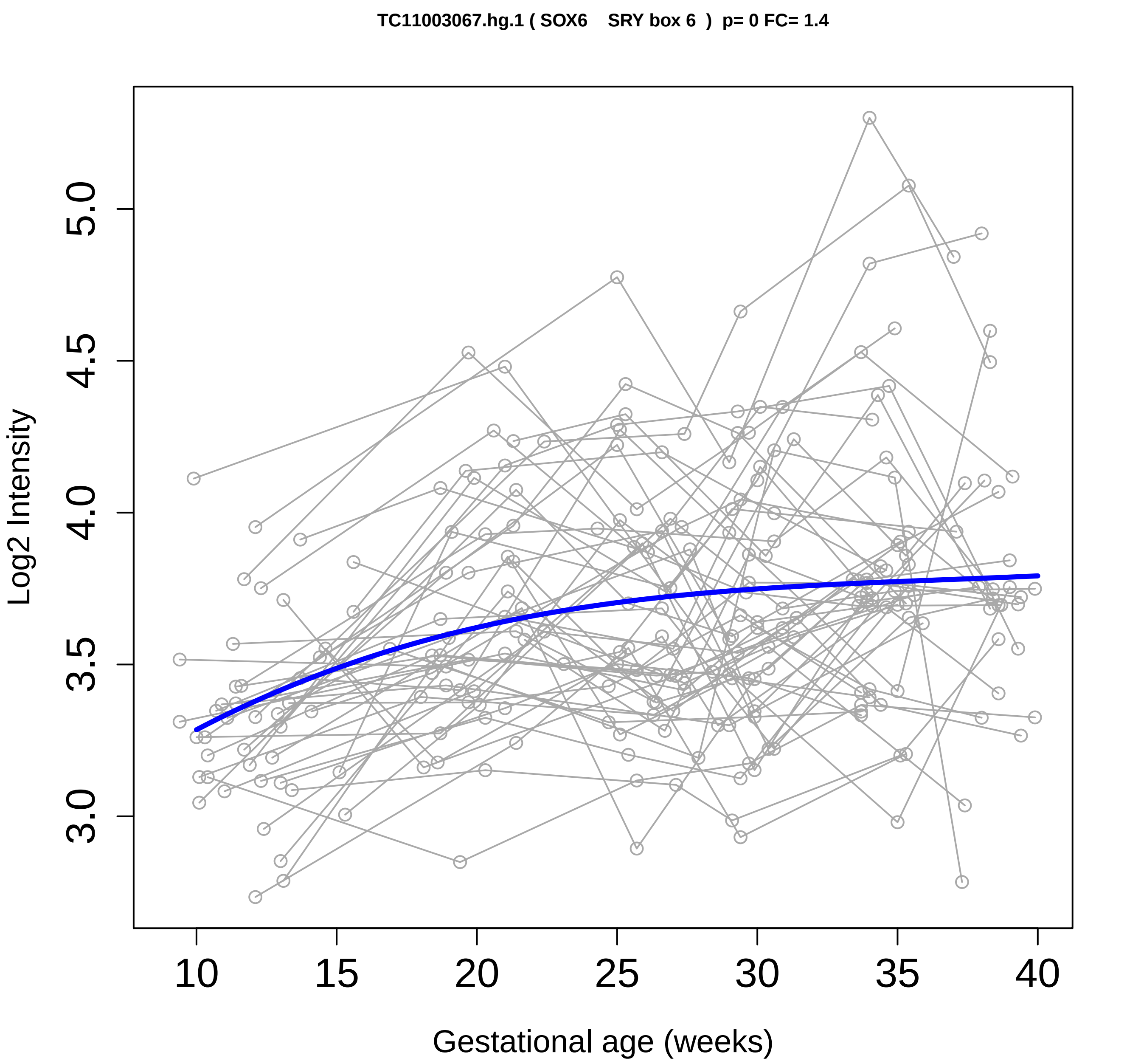

TC11003067.hg.1 (SOX6 SRY box 6) p= 0 FC= 1.4
Log2 Intensity
3.0
3.5
4.0
4.5
5.0
10
15
20
25
30
35
40
Gestational age (weeks)

TC16001854.hg.1 (SLC6A10P solute carrier family 6 (neurotransmitter transporter), member 10, pseudogene) p= 0 FC= 1.5

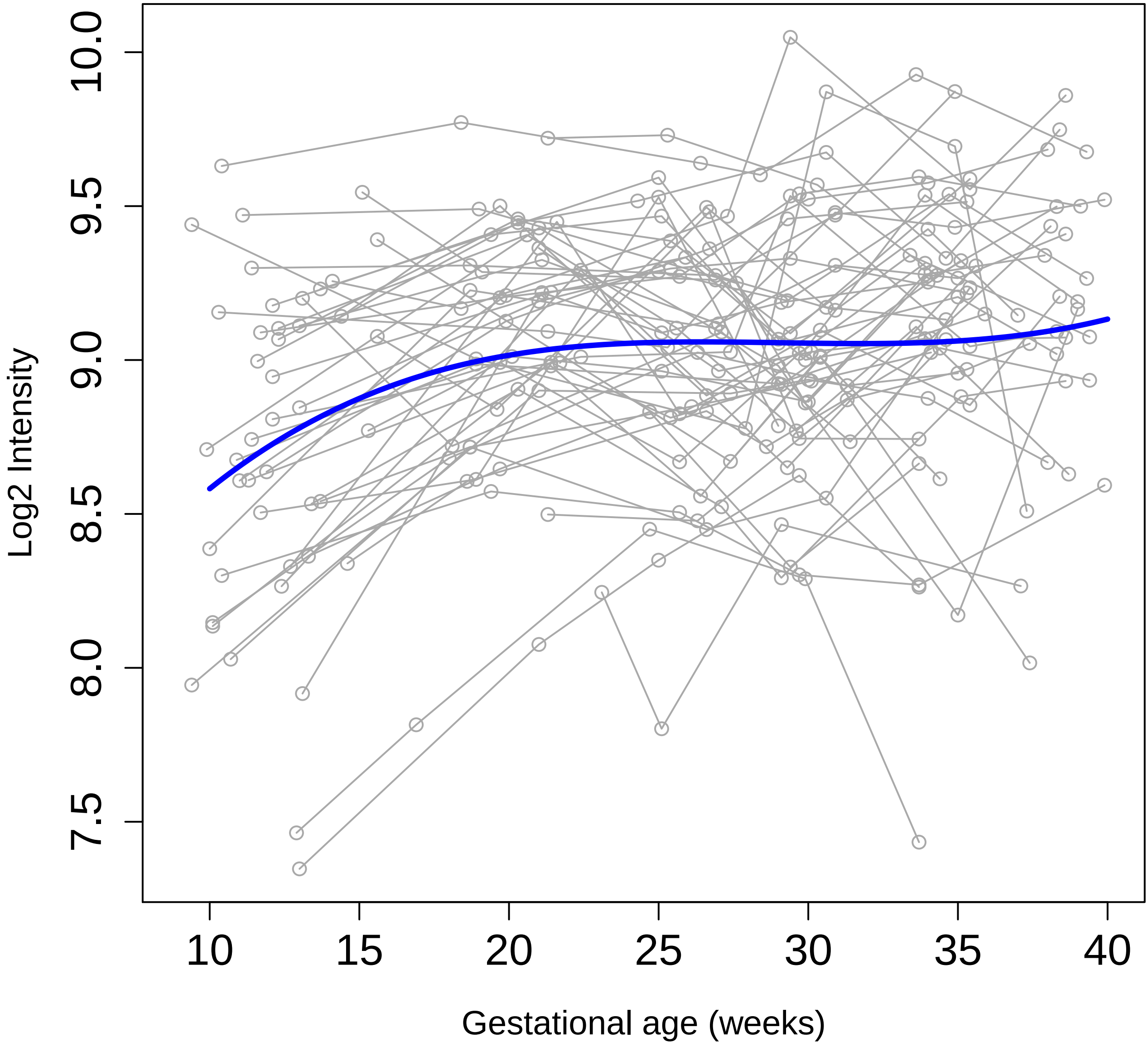

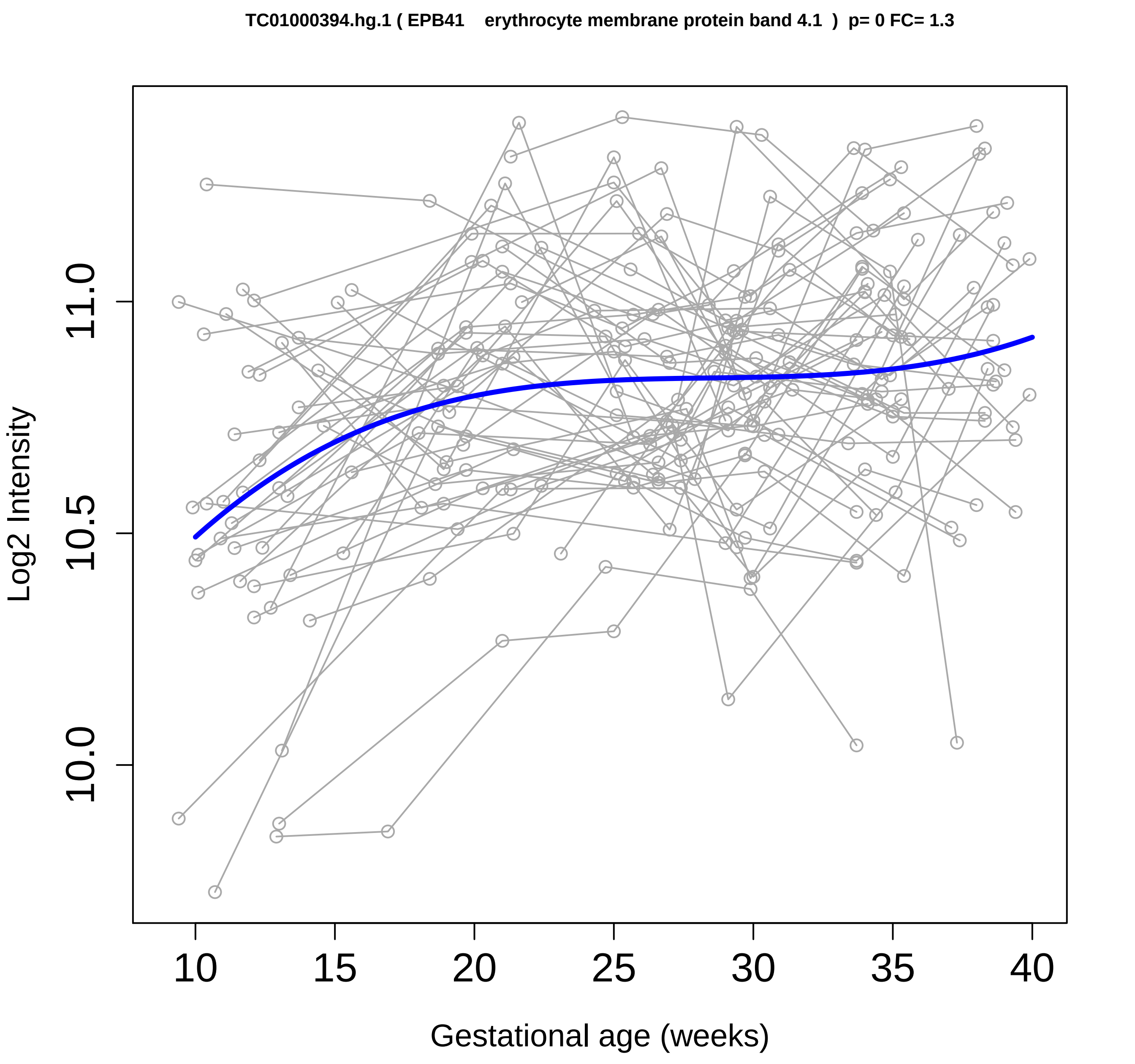

TC01000394.hg.1 (EPB41 erythrocyte membrane protein band 4.1) p= 0 FC= 1.3
Log2 Intensity
10.0
10.5
11.0
10
15
20
25
30
35
40
Gestational age (weeks)

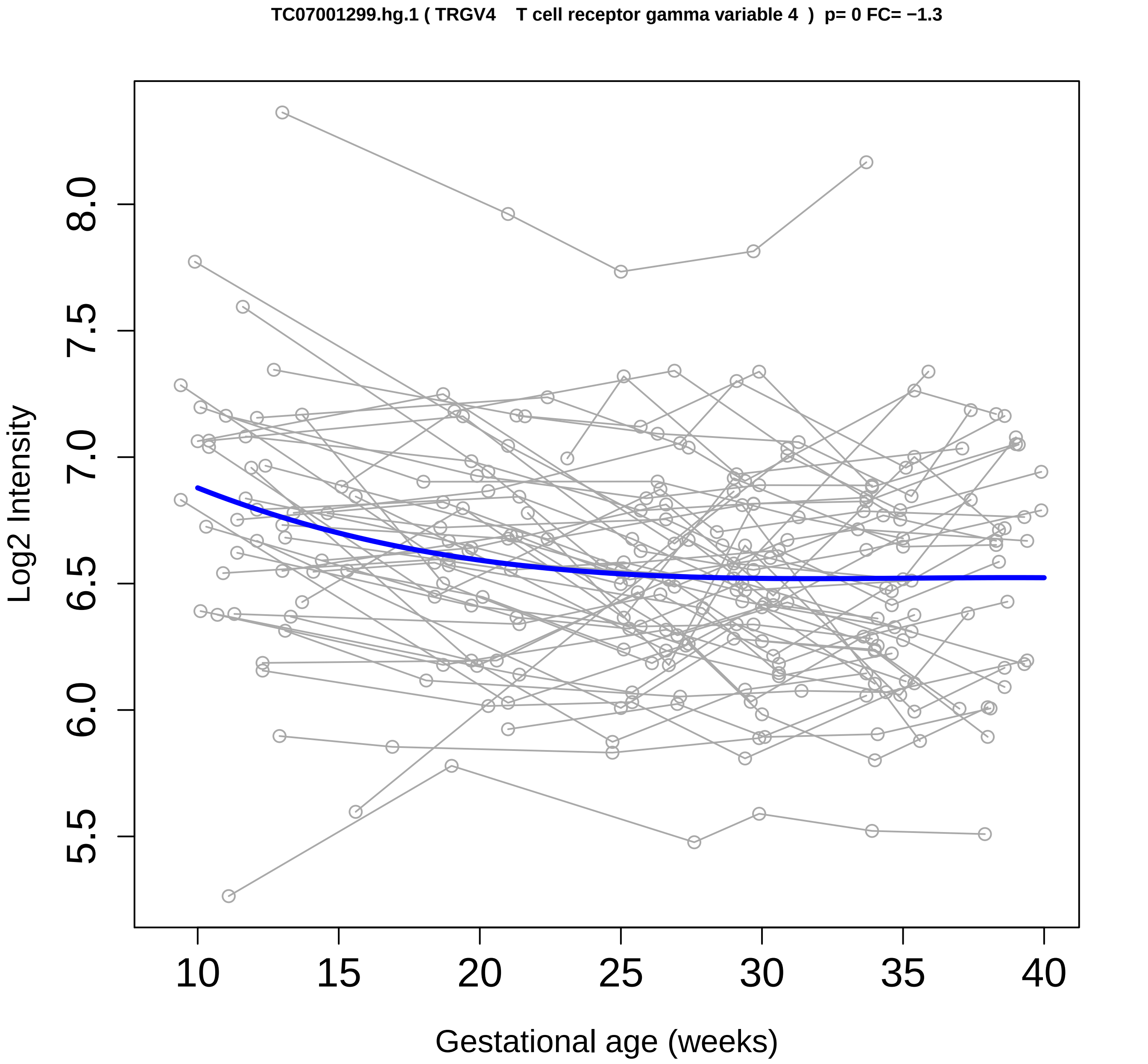

TC07001299.hg.1 (TRGV4 T cell receptor gamma variable 4) p= 0 FC= −1.3
Log2 Intensity
Gestational age (weeks)
5.5
6.0
6.5
7.0
7.5
8.0
10
15
20
25
30
35
40

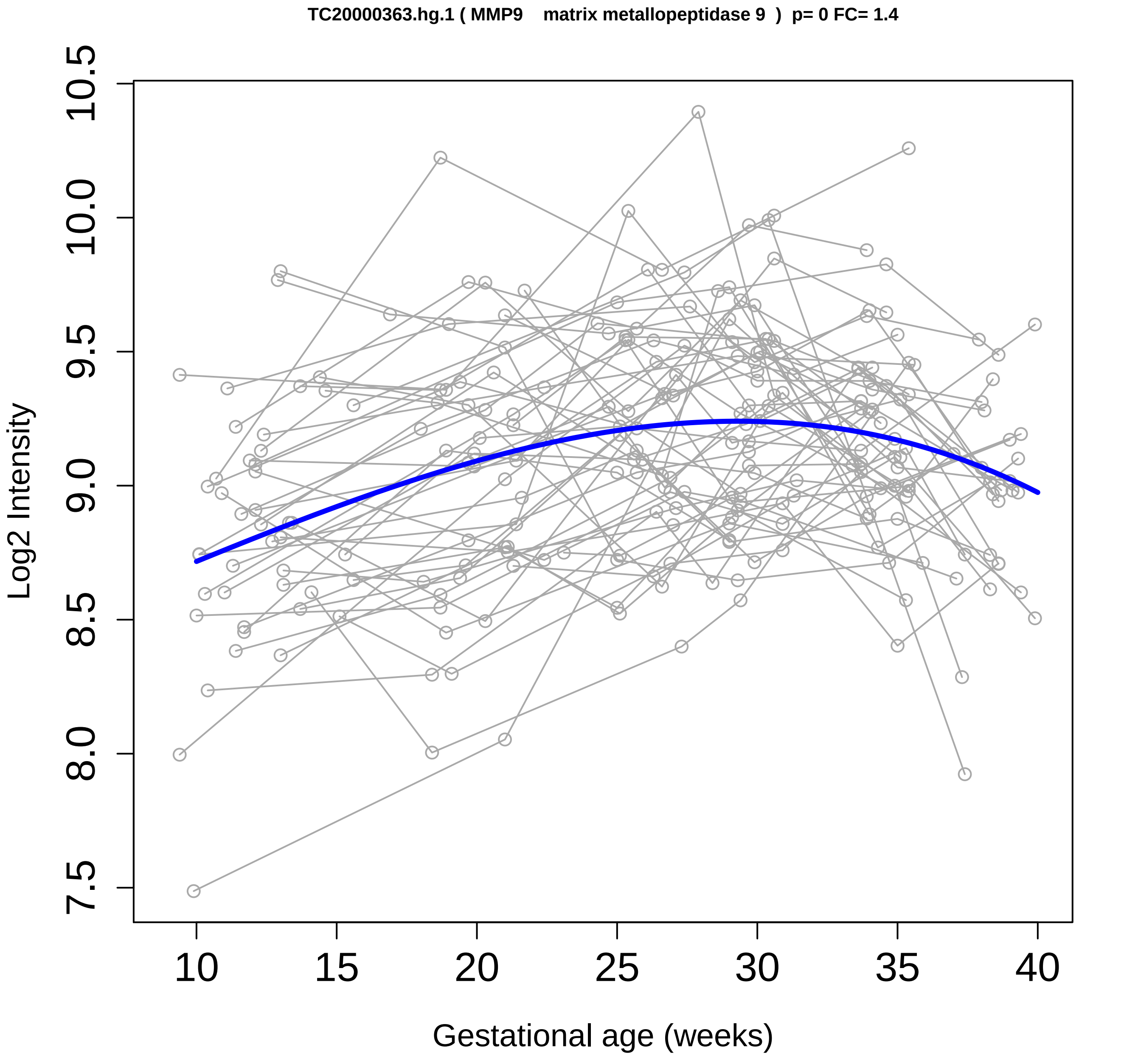

TC20000363.hg.1 (MMP9 matrix metallopeptidase 9) p= 0 FC= 1.4
Log2 Intensity
7.5
8.0
8.5
9.0
9.5
10.0
10.5
10
15
20
25
30
35
40
Gestational age (weeks)

TC16001066.hg.1 (SLC6A10P solute carrier family 6 (neurotransmitter transporter), member 10, pseudogene) p= 0 FC= 1.4

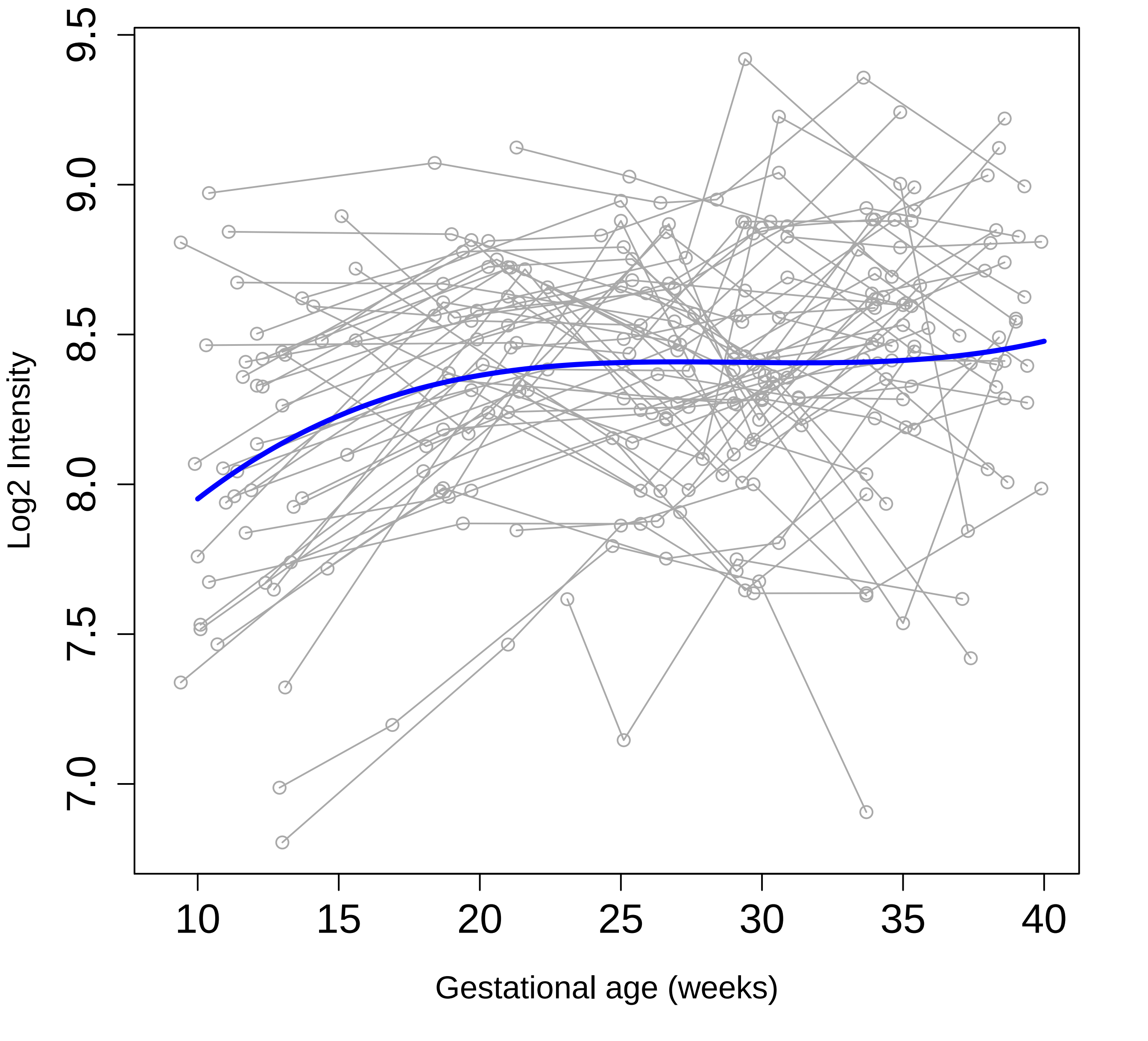

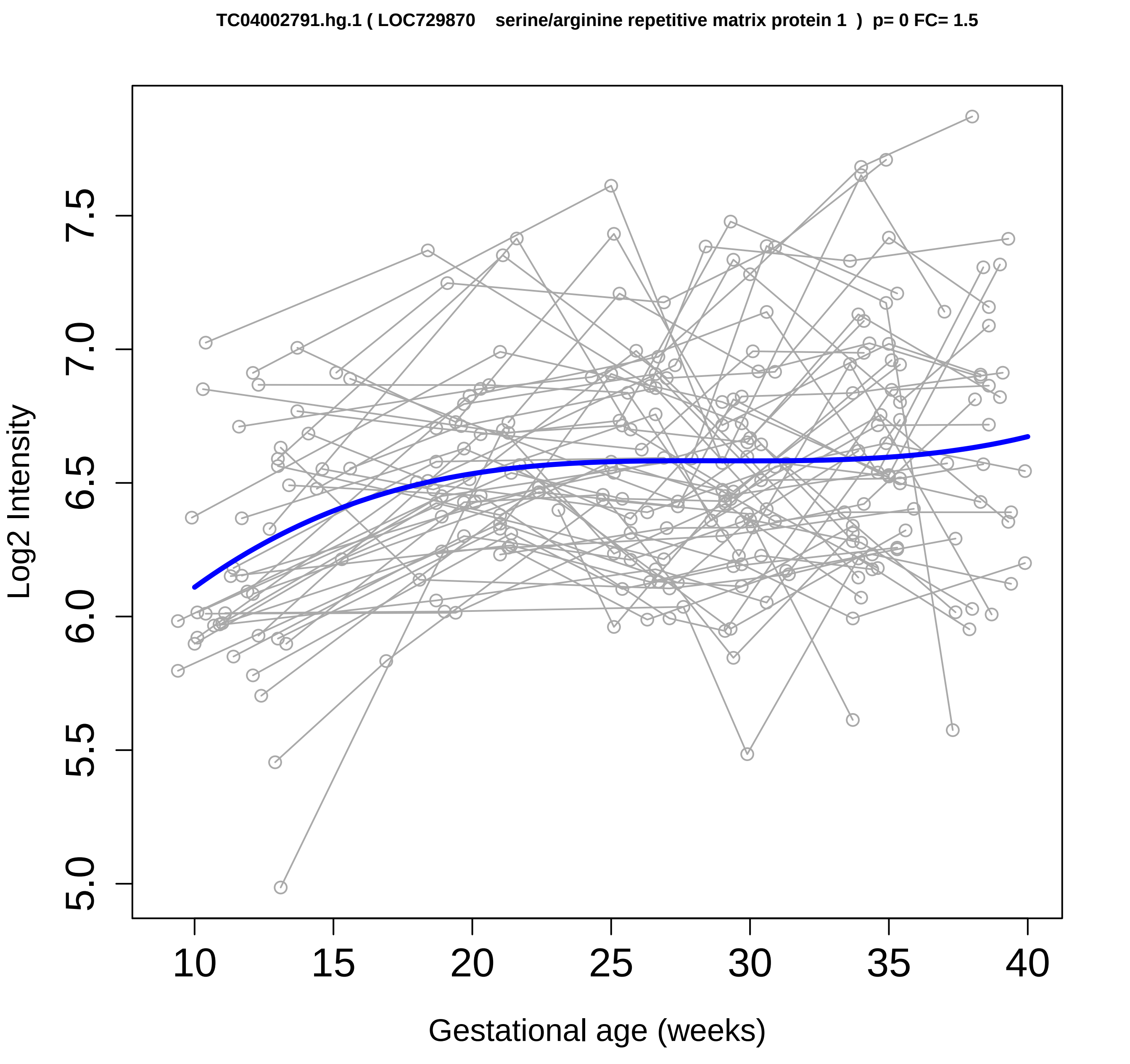
TC04002791.hg.1 (LOC729870   serine/arginine repetitive matrix protein 1) p= 0 FC= 1.5
Log2 Intensity
5.0
5.5
6.0
6.5
7.0
7.5
10
15
20
25
30
35
40
Gestational age (weeks)

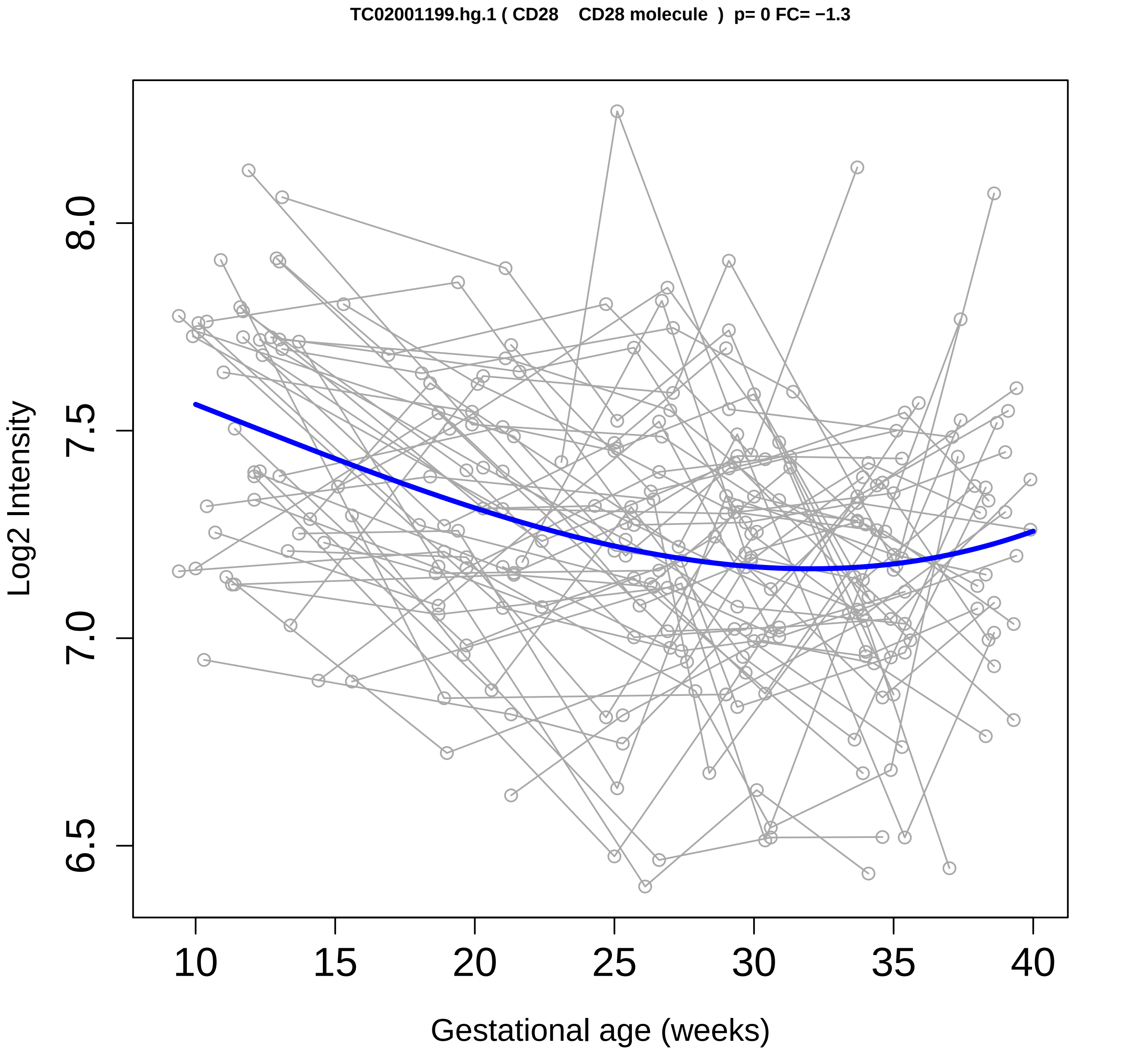
TC02001199.hg.1 (CD28 CD28 molecule) p= 0 FC= −1.3
Log2 Intensity
6.5
7.0
7.5
8.0
10
15
20
25
30
35
40
Gestational age (weeks)

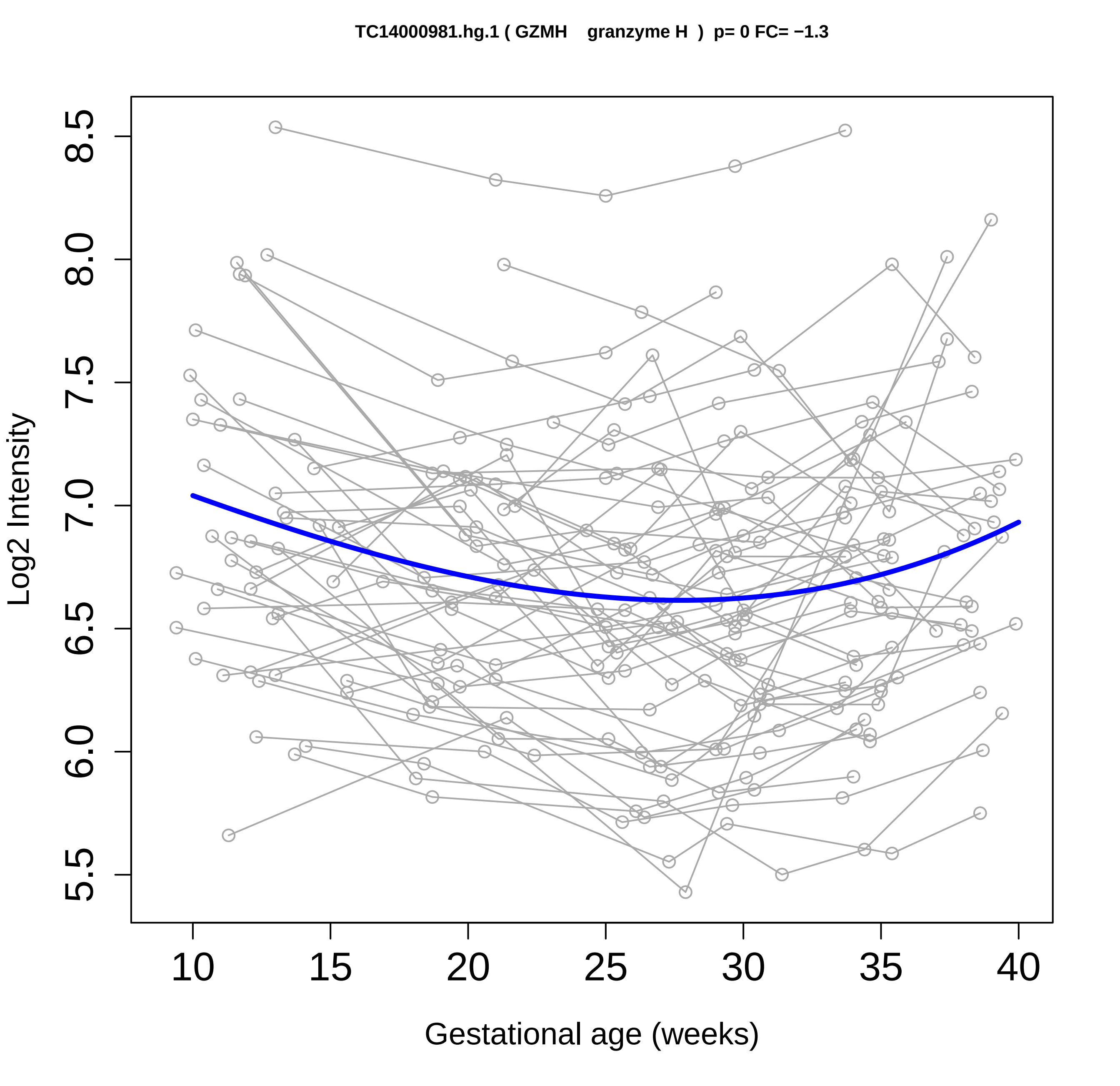

TC14000981.hg.1 (GZMH granzyme H) p= 0 FC= −1.3
Log2 Intensity
5.5
6.0
6.5
7.0
7.5
8.0
8.5
10
15
20
25
30
35
40
Gestational age (weeks)

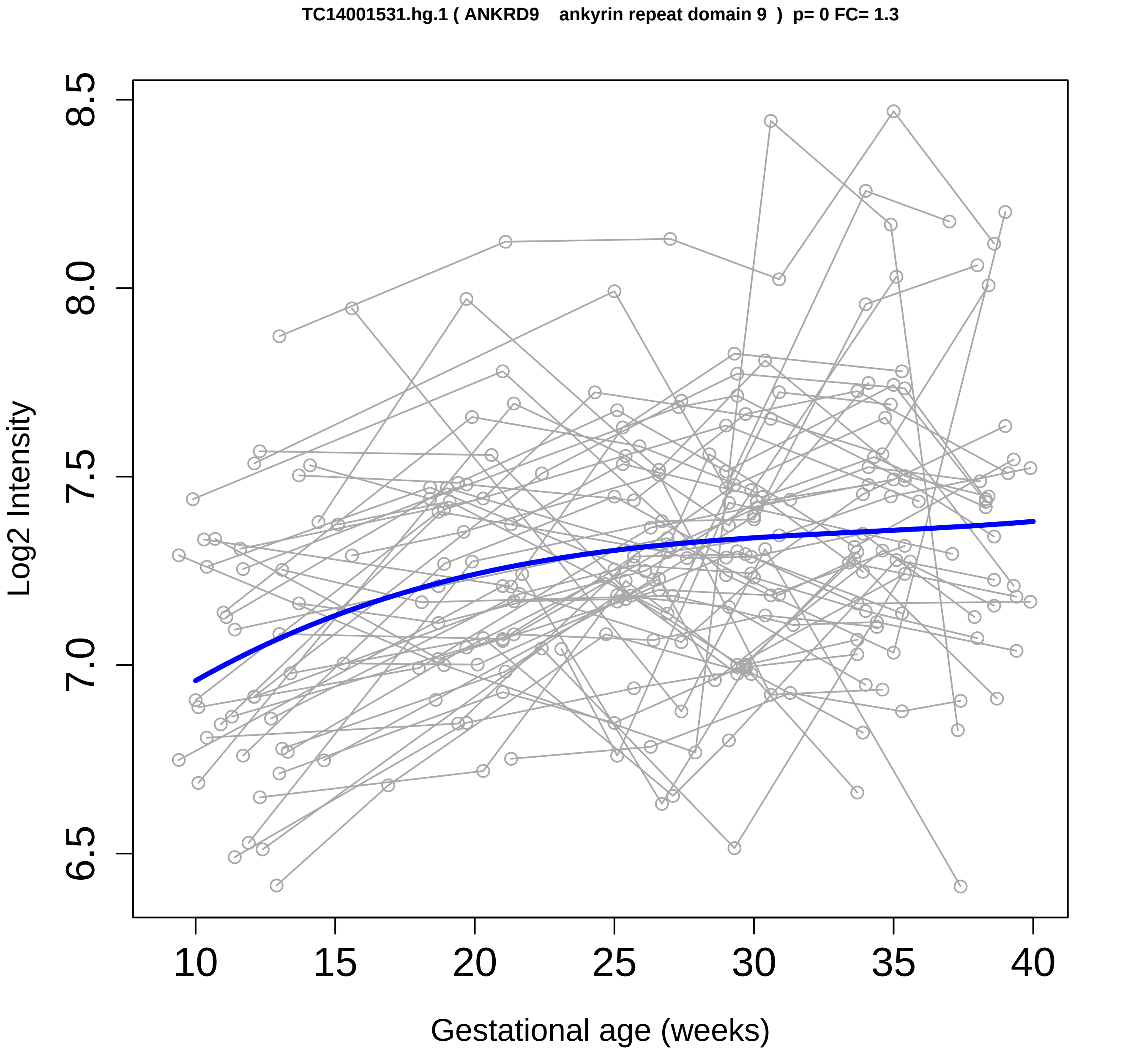
TC14001531.hg.1 (ANKRD9 ankyrin repeat domain 9) p= 0 FC= 1.3
Log2 Intensity
8.5
8.0
7.5
7.0
6.5
10
15
20
25
30
35
40
Gestational age (weeks)

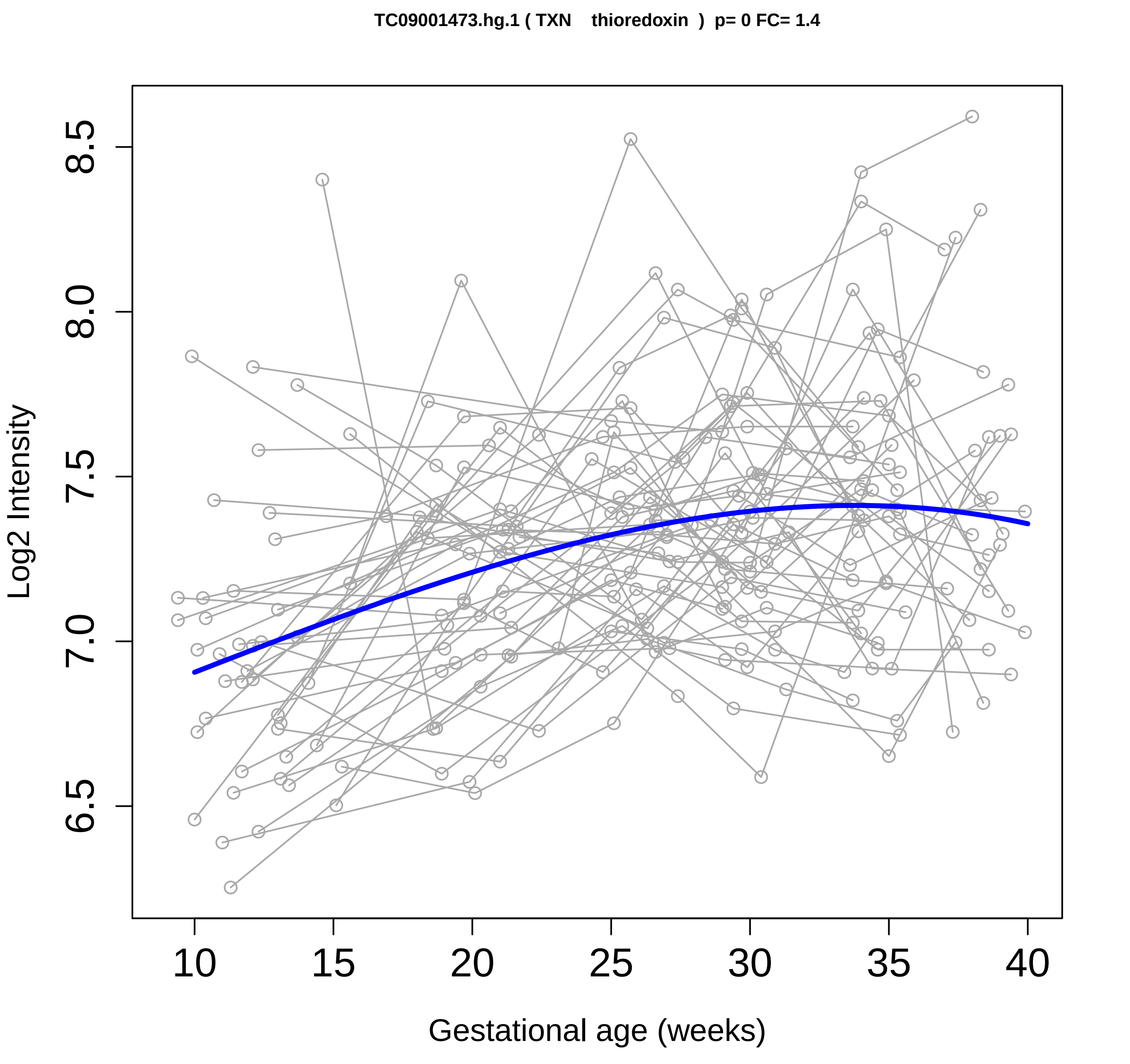
TC09001473.hg.1 (TXN thioredoxin) p= 0 FC= 1.4
Log2 Intensity
6.5
7.0
7.5
8.0
8.5
10
15
20
25
30
35
40
Gestational age (weeks)

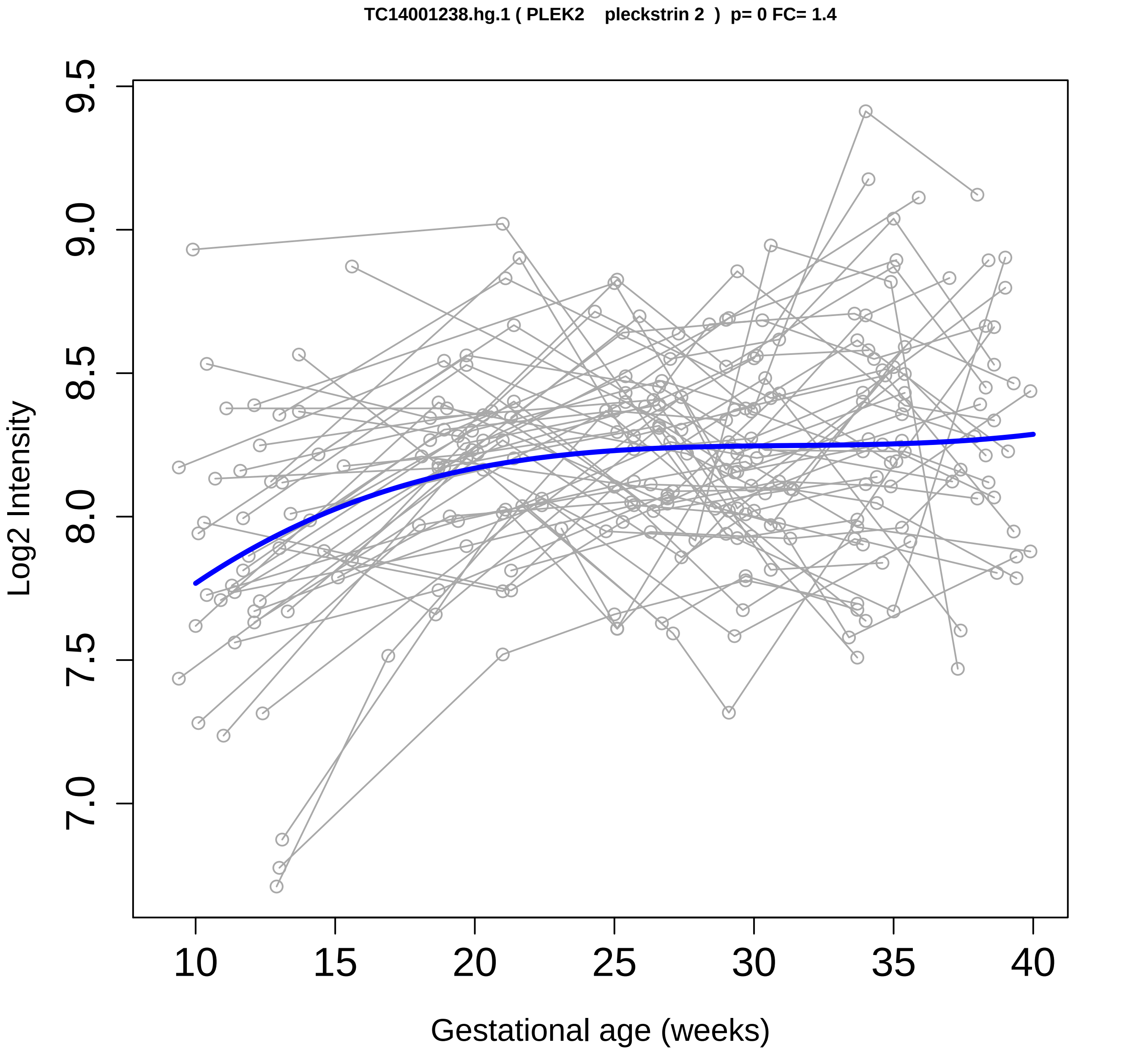
TC14001238.hg.1 (PLEK2 pleckstrin 2) p= 0 FC= 1.4
Log2 Intensity
7.0
7.5
8.0
8.5
9.0
9.5
10
15
20
25
30
35
40
Gestational age (weeks)

TC16000400.hg.1 (SLC6A10PB   solute carrier family 6 (neurotransmitter transporter), member 8 pseudogene) p= 0 FC= 1.5

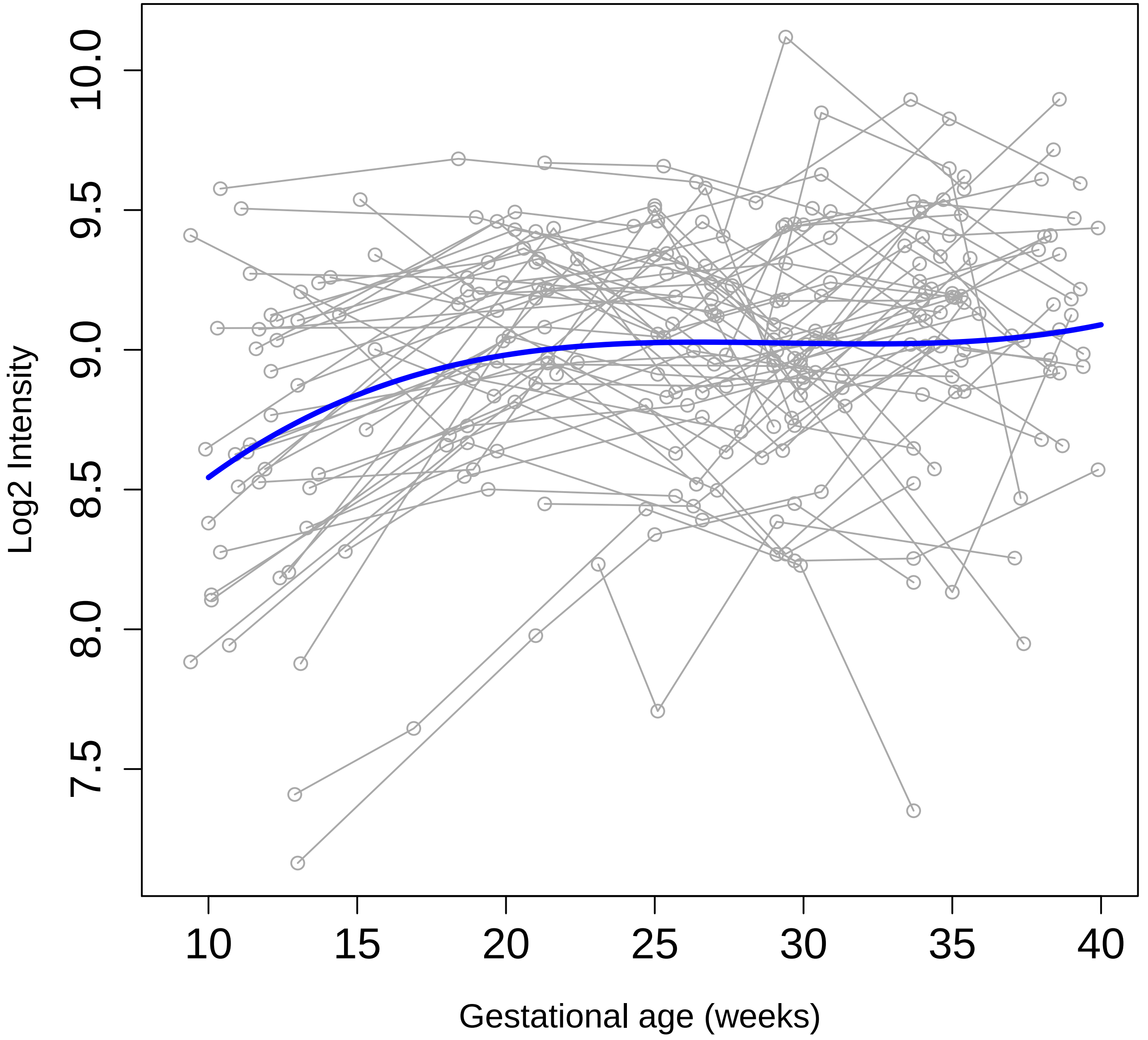

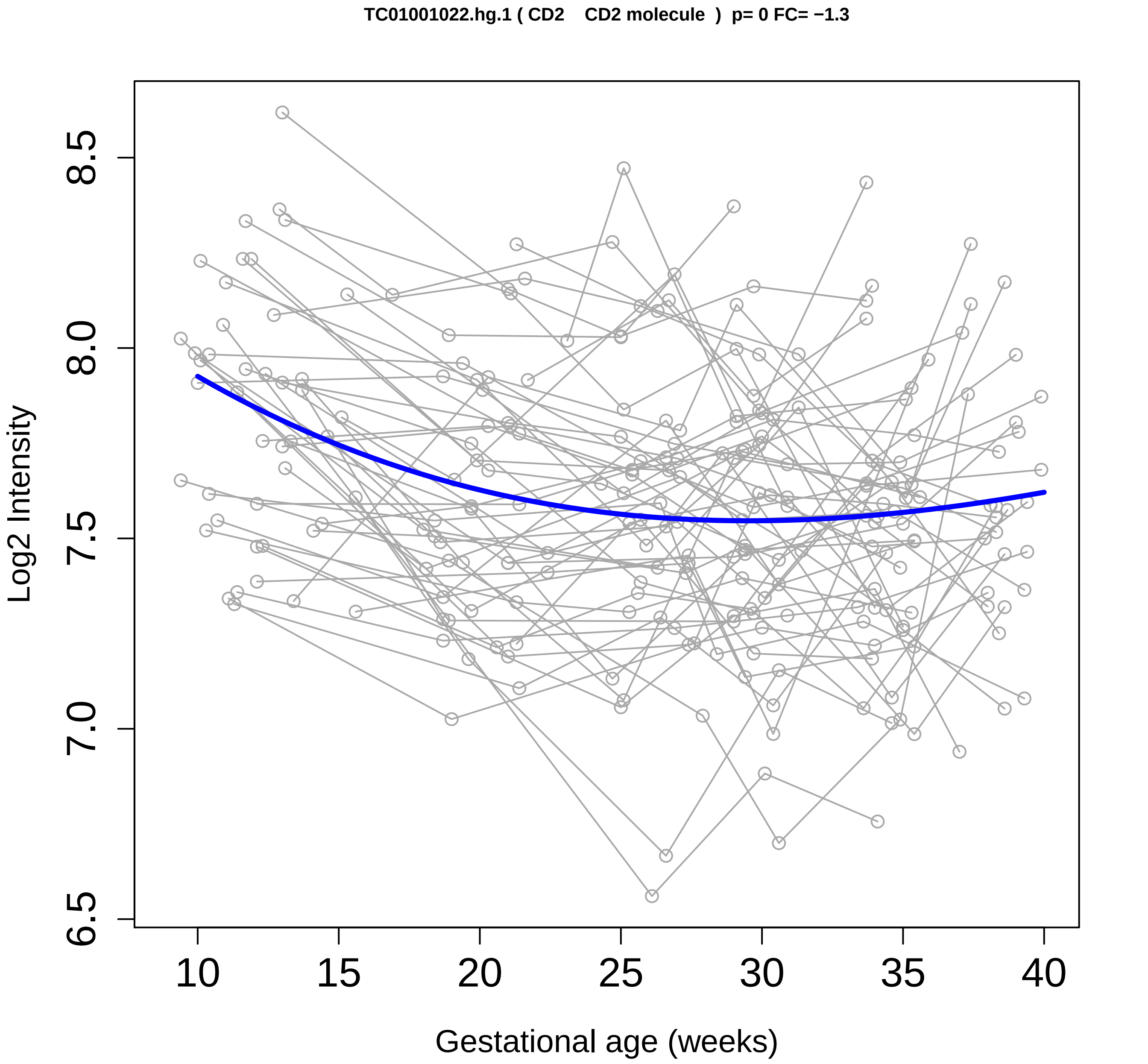

TC01001022.hg.1 (CD2   CD2 molecule) p= 0 FC= −1.3
Log2 Intensity
6.5
7.0
7.5
8.0
8.5
10
15
20
25
30
35
40
Gestational age (weeks)

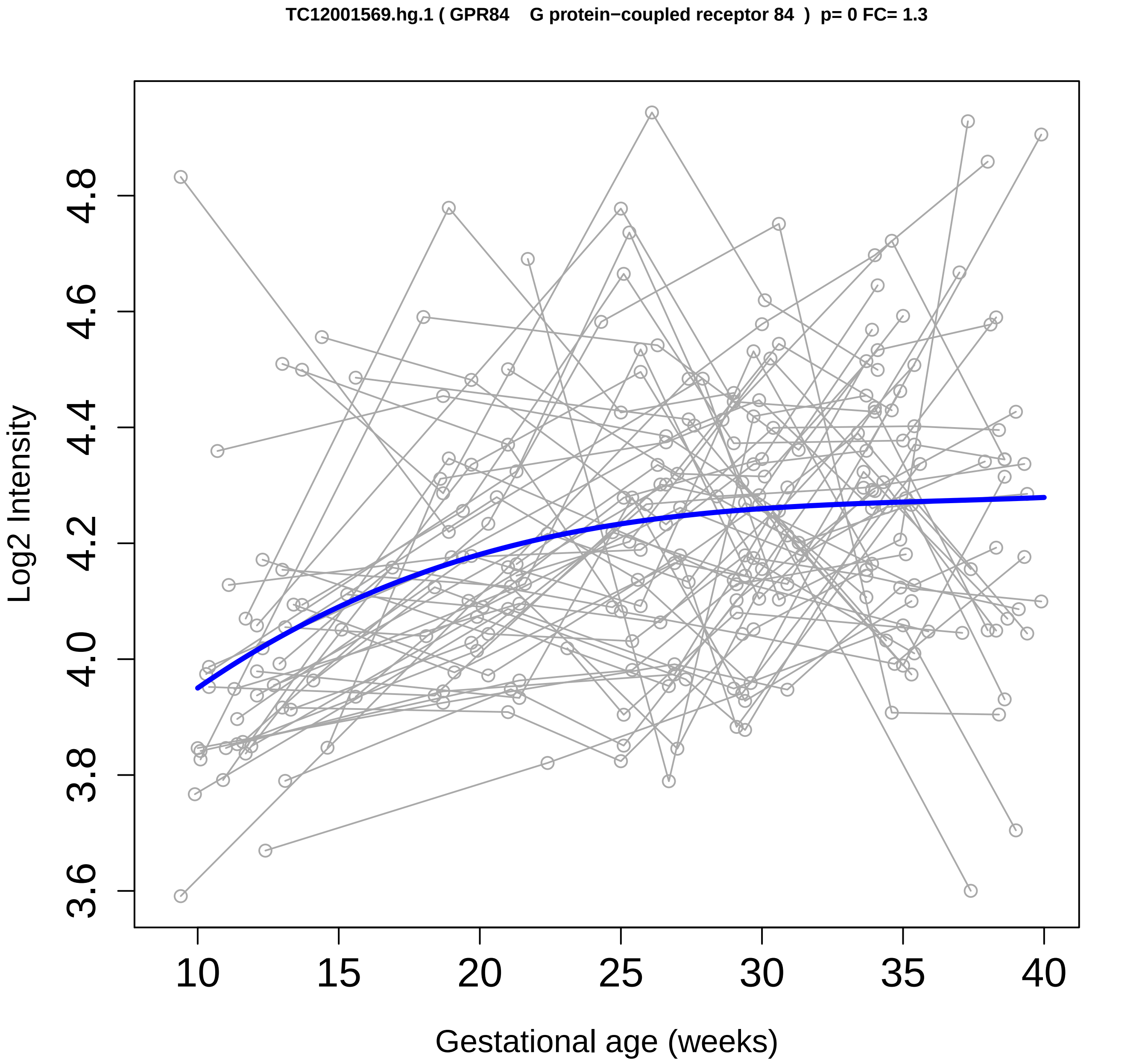
TC12001569.hg.1 (GPR84 G protein−coupled receptor 84) p= 0 FC= 1.3
Log2 Intensity
3.6
3.8
4.0
4.2
4.4
4.6
4.8
10
15
20
25
30
35
40
Gestational age (weeks)

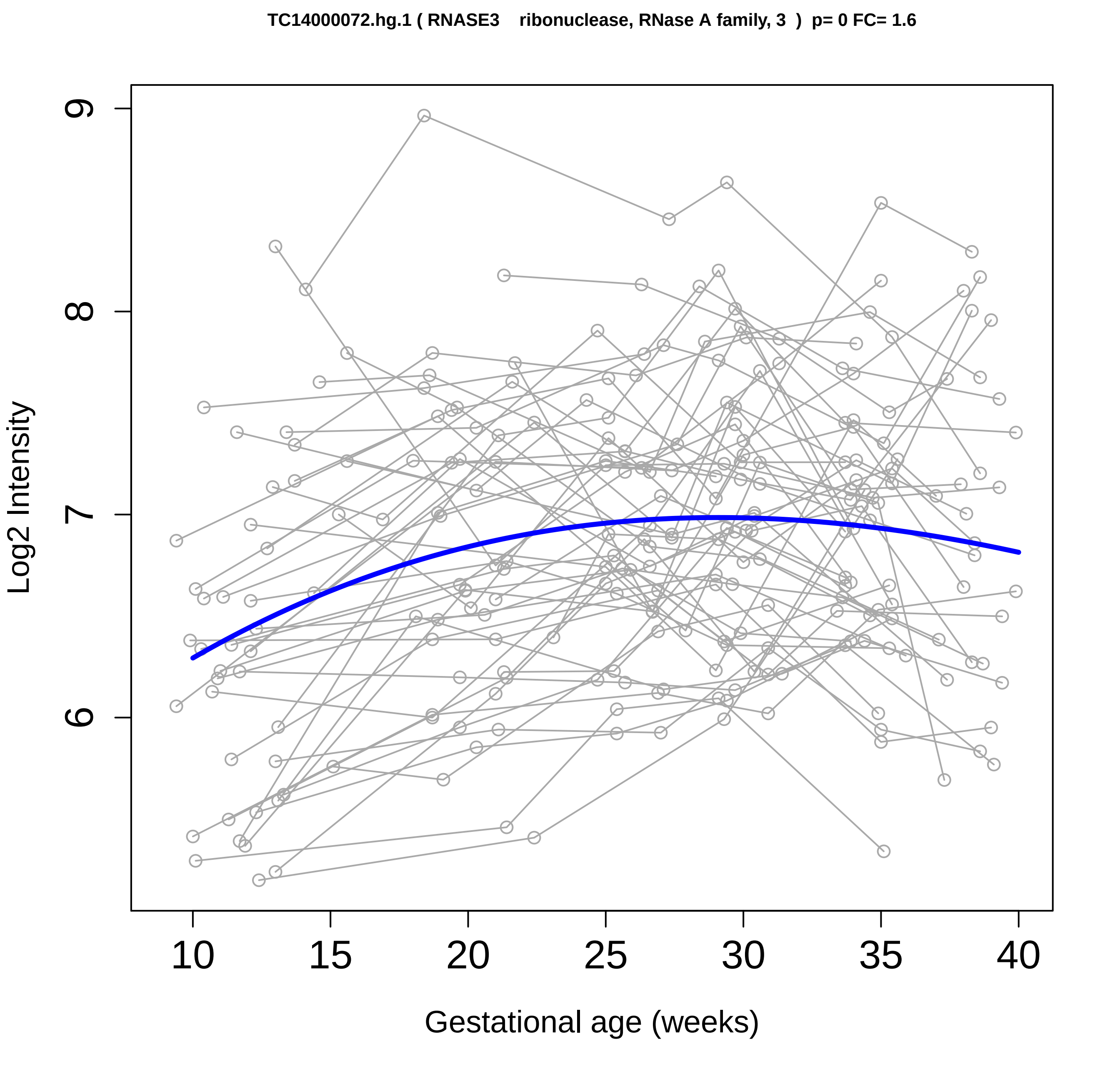
TC14000072.hg.1 (RNASE3 ribonuclease, RNase A family, 3) p= 0 FC= 1.6
Log2 Intensity
6
7
8
9
10
15
20
25
30
35
40
Gestational age (weeks)

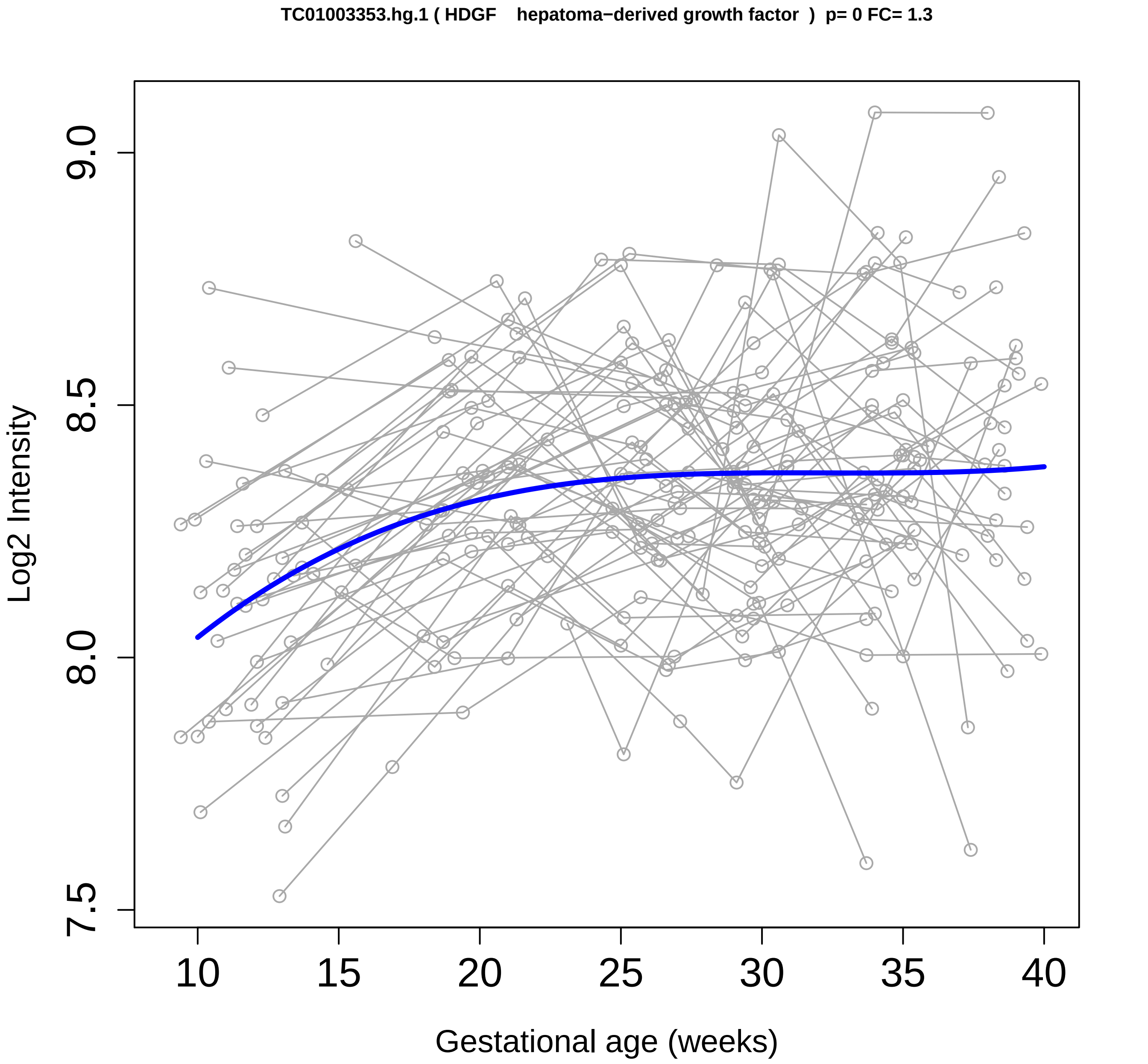
TC01003353.hg.1 (HDGF hepatoma−derived growth factor) p= 0 FC= 1.3
Log2 Intensity
9.0
8.5
8.0
7.5
10
15
20
25
30
35
40
Gestational age (weeks)

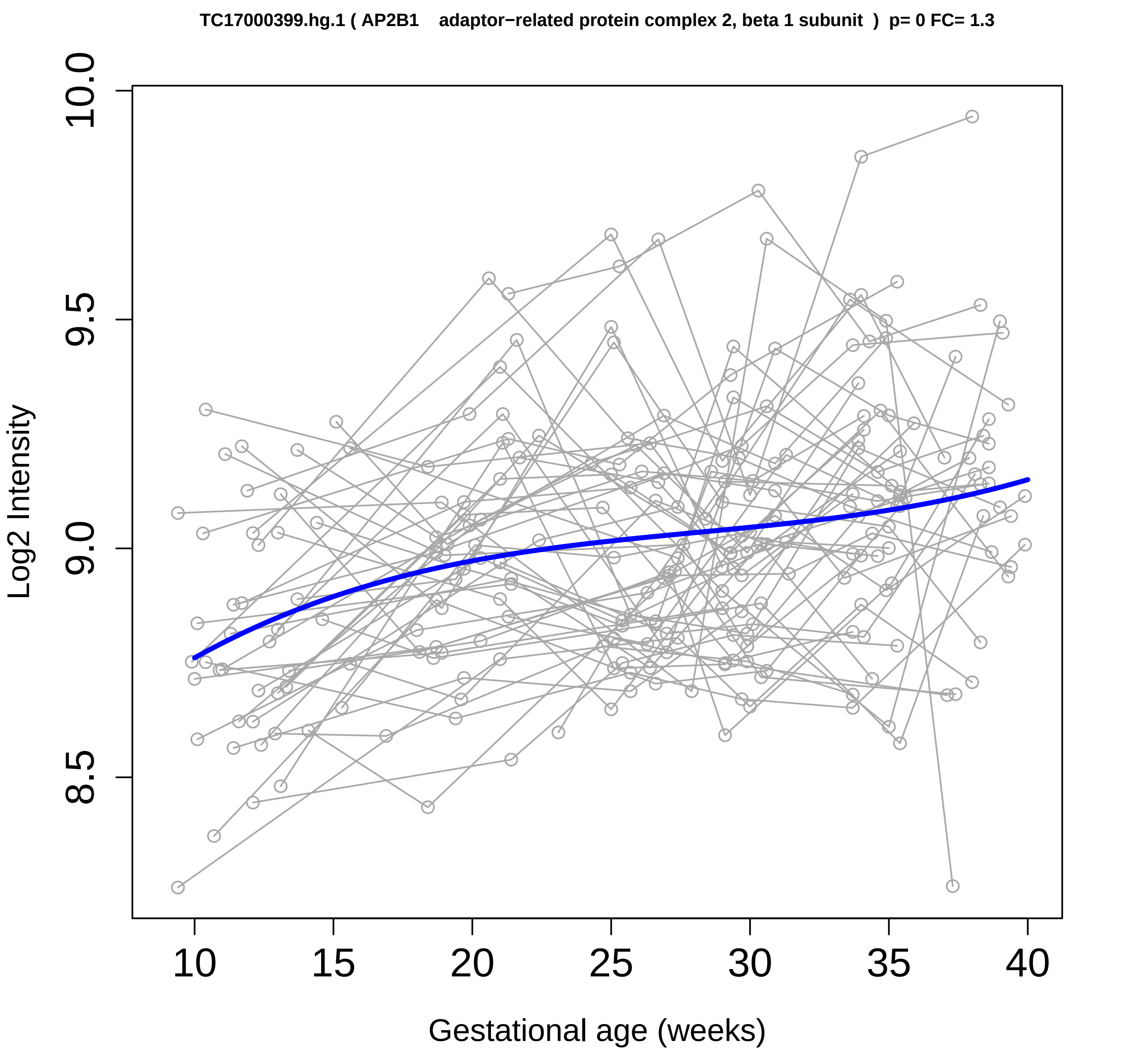
TC17000399.hg.1 (AP2B1 adaptor−related protein complex 2, beta 1 subunit) p= 0 FC= 1.3
Log2 Intensity
10.0
9.5
9.0
8.5
10
15
20
25
30
35
40
Gestational age (weeks)

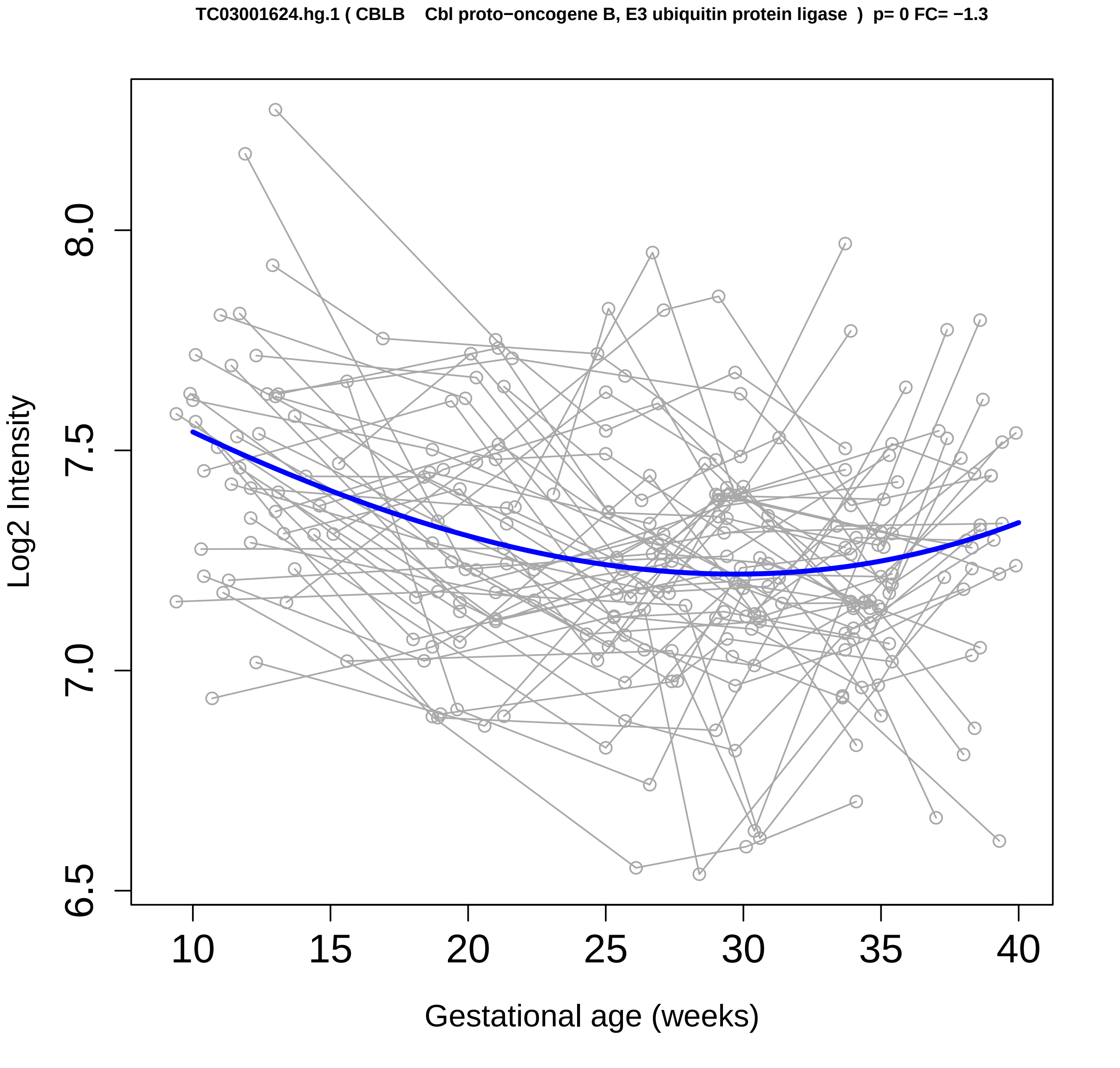

TC03001624.hg.1 (CBLB Cbl proto−oncogene B, E3 ubiquitin protein ligase) p= 0 FC= −1.3
Log2 Intensity
6.5
7.0
7.5
8.0
10
15
20
25
30
35
40
Gestational age (weeks)

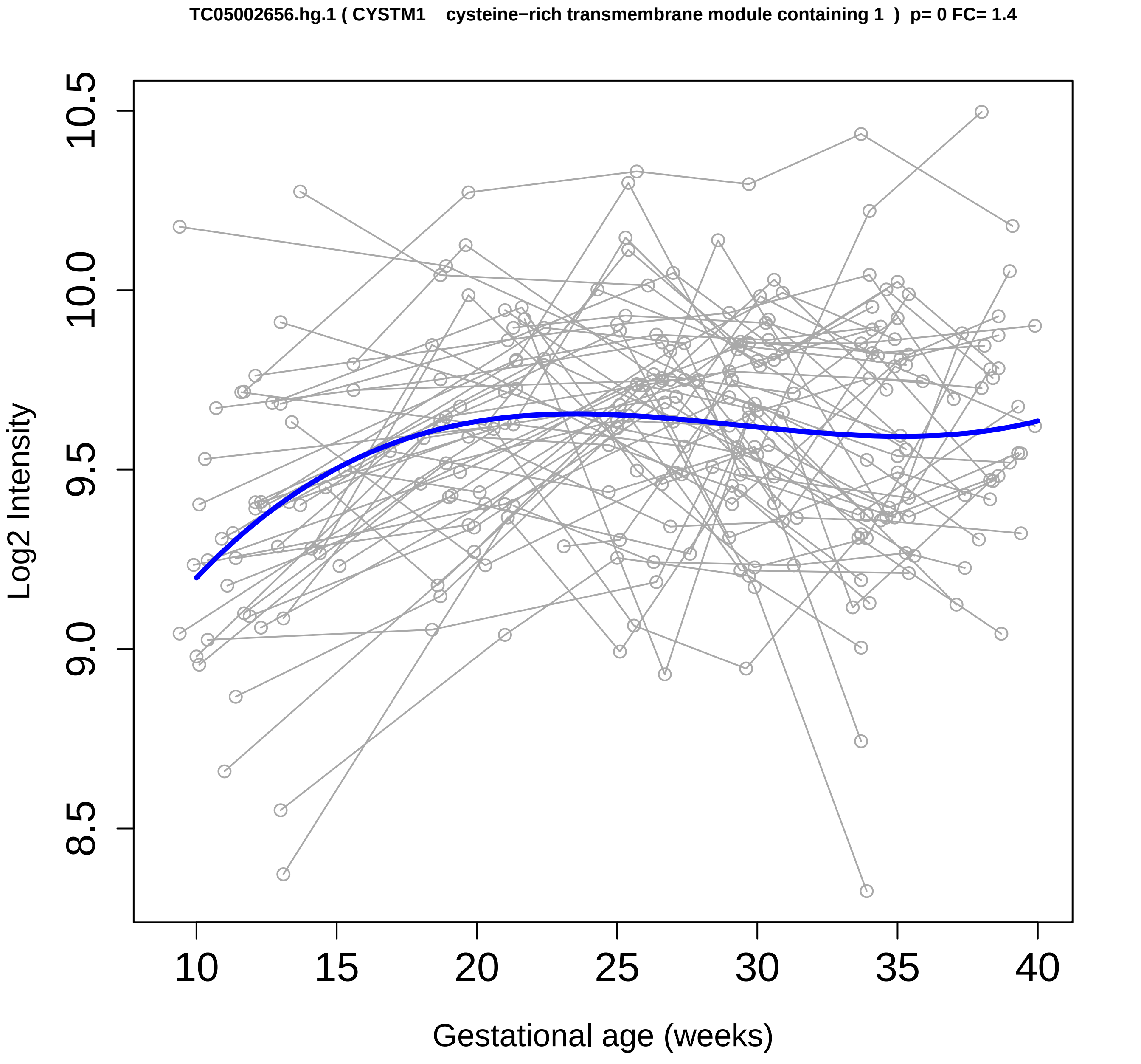

TC05002656.hg.1 (CYSTM1 cysteine−rich transmembrane module containing 1) p= 0 FC= 1.4
Log2 Intensity
8.5
9.0
9.5
10.0
10.5
10
15
20
25
30
35
40
Gestational age (weeks)

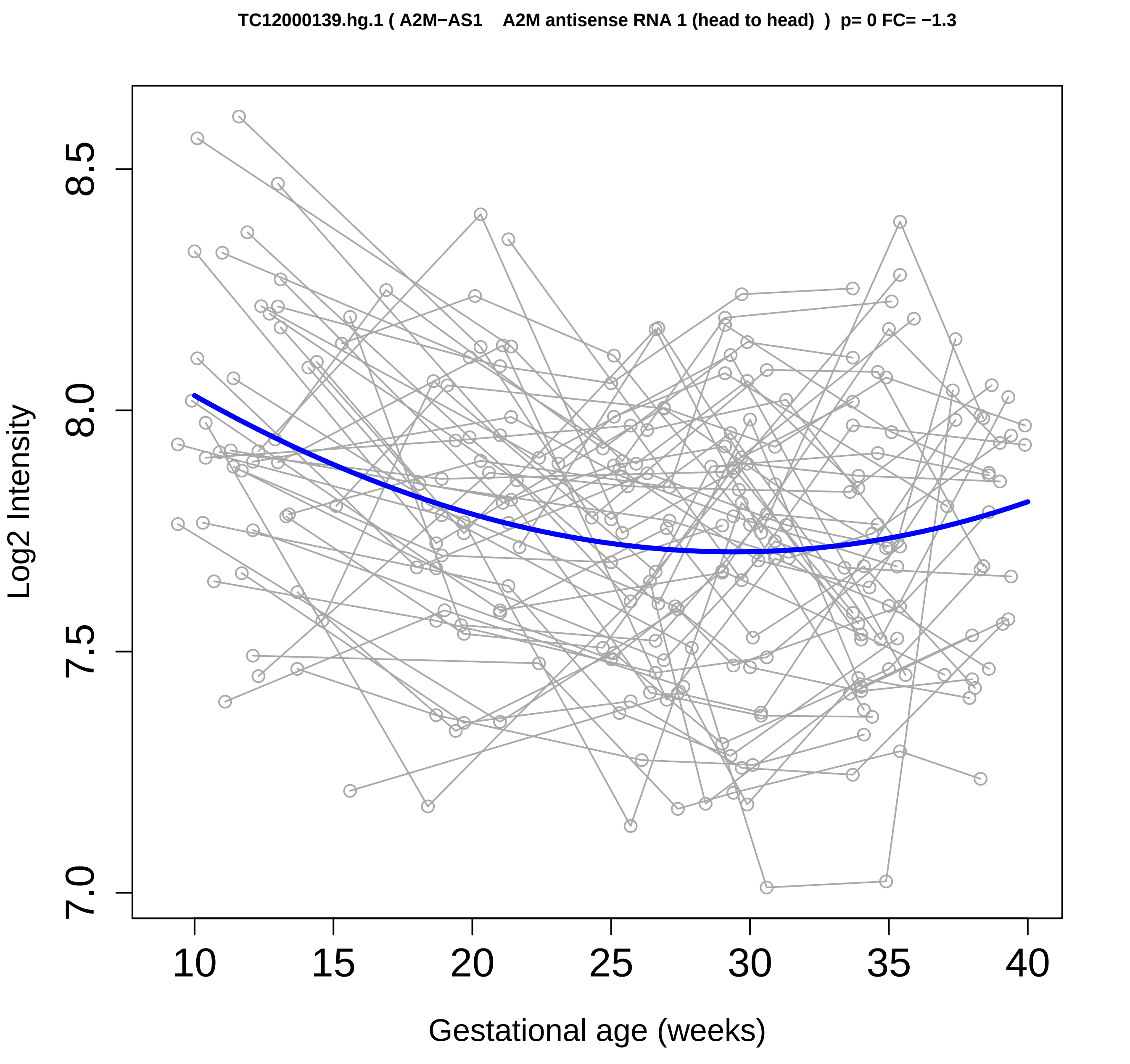

TC12000139.hg.1 (A2M−AS1 A2M antisense RNA 1 (head to head)) p= 0 FC= −1.3
Log2 Intensity
8.5
8.0
7.5
7.0
10
15
20
25
30
35
40
Gestational age (weeks)

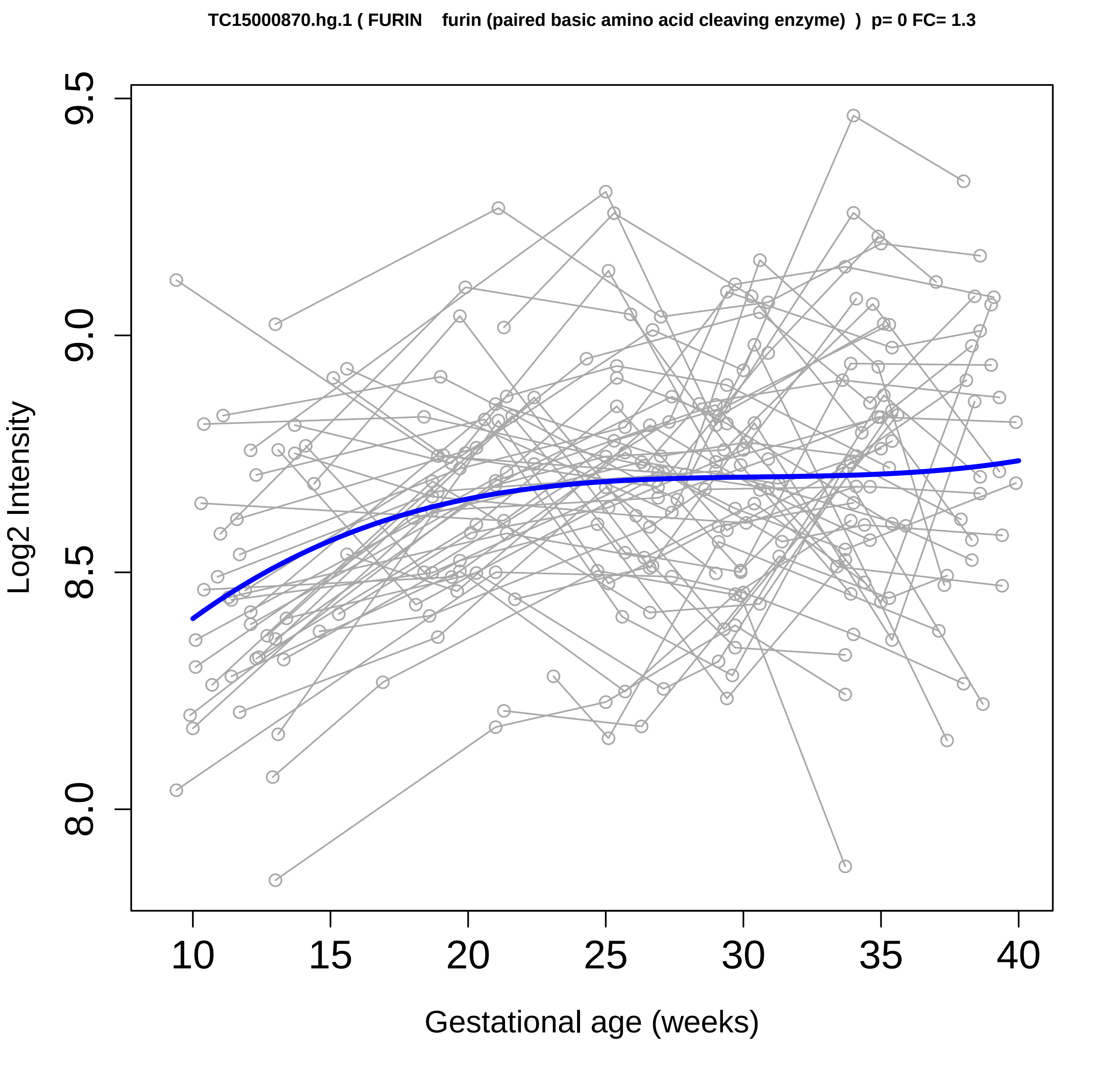
TC15000870.hg.1 (FURIN furin (paired basic amino acid cleaving enzyme)) p= 0 FC= 1.3
Log2 Intensity
9.5
9.0
8.5
8.0
10
15
20
25
30
35
40
Gestational age (weeks)

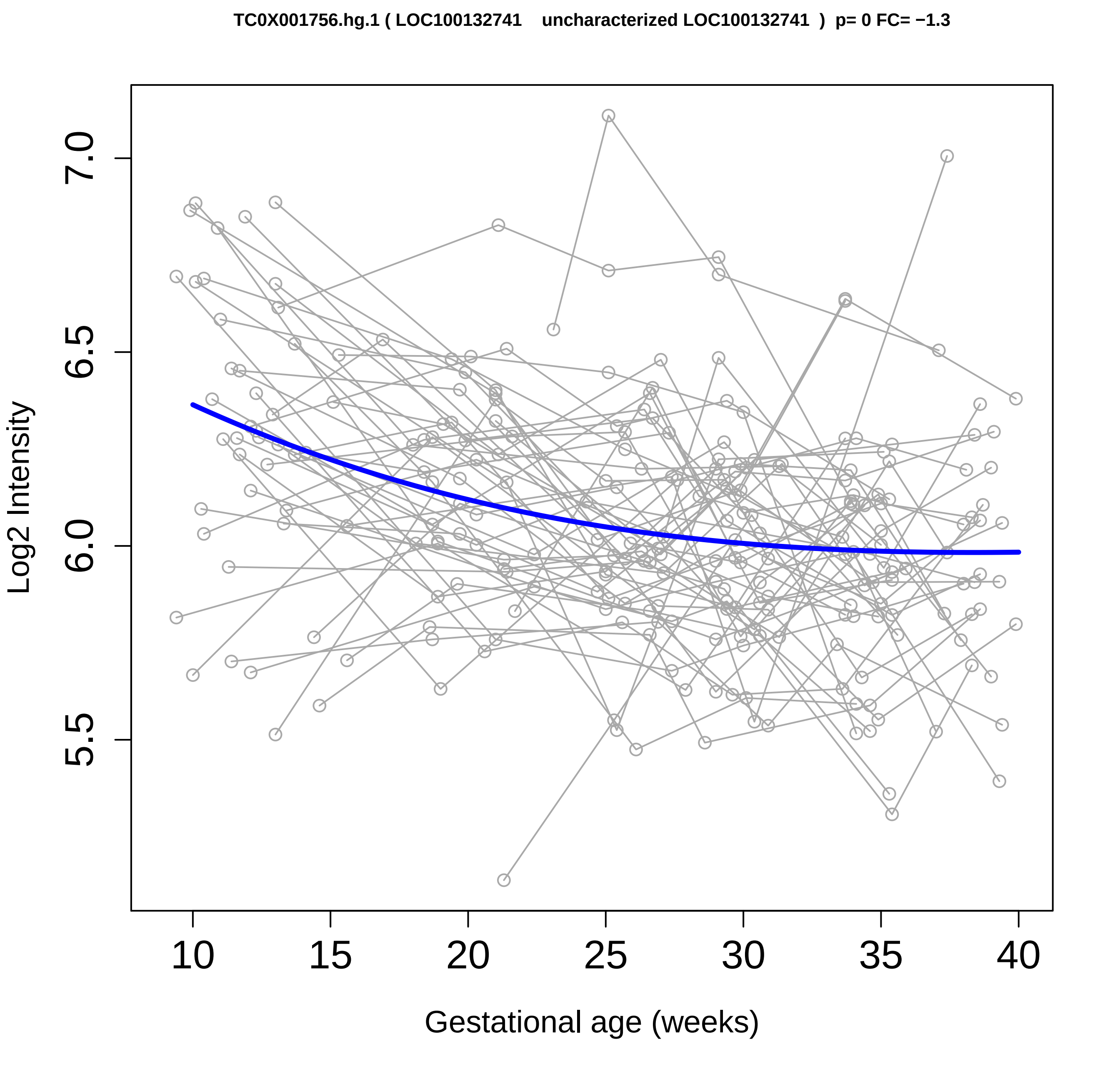

TC0X001756.hg.1 (LOC100132741 uncharacterized LOC100132741) p= 0 FC= −1.3
Log2 Intensity
7.0
6.5
6.0
5.5
10
15
20
25
30
35
40
Gestational age (weeks)

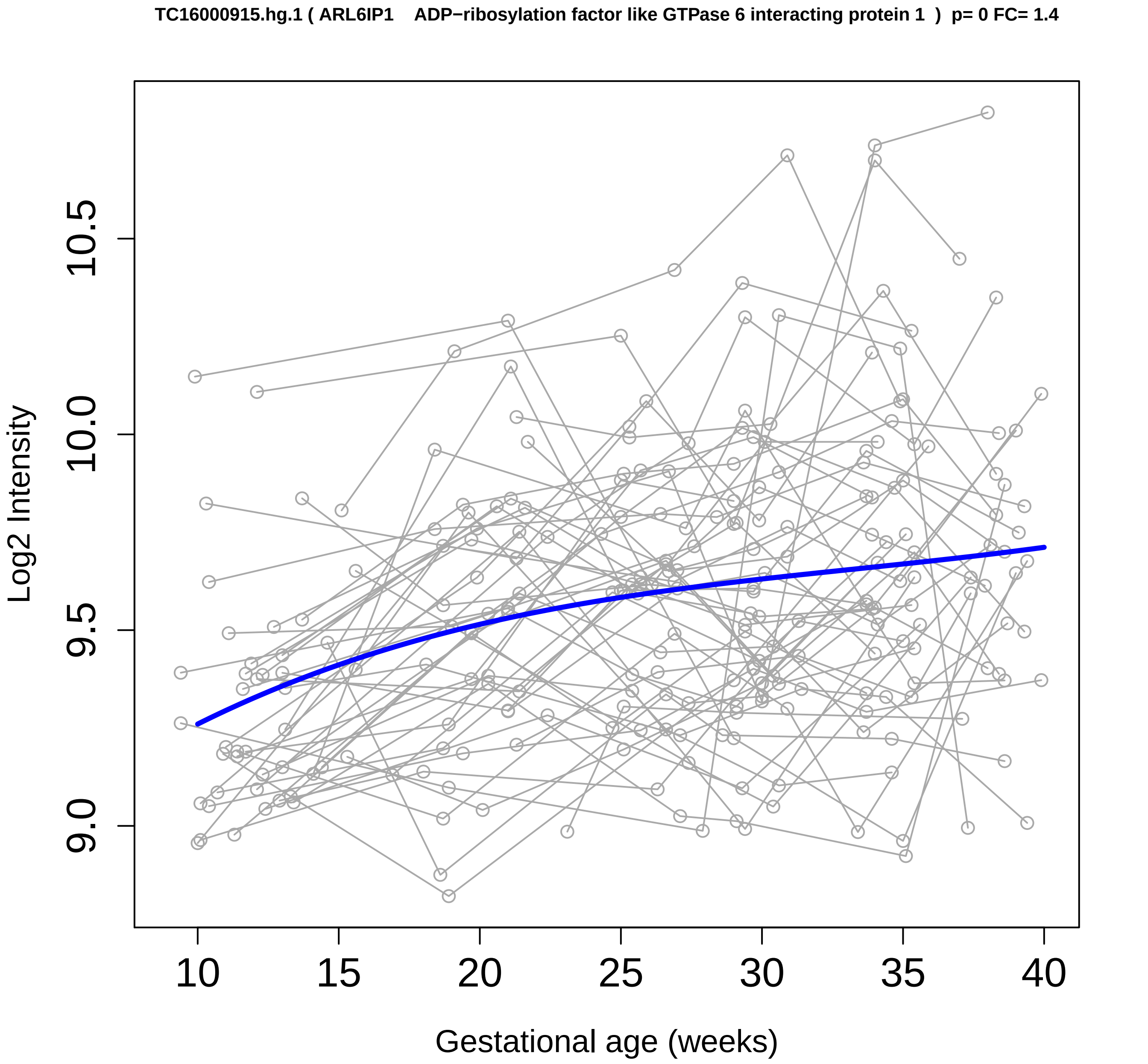

TC16000915.hg.1 (ARL6IP1   ADP−ribosylation factor like GTPase 6 interacting protein 1) p= 0 FC= 1.4
Log2 Intensity
9.0
9.5
10.0
10.5
10
15
20
25
30
35
40
Gestational age (weeks)

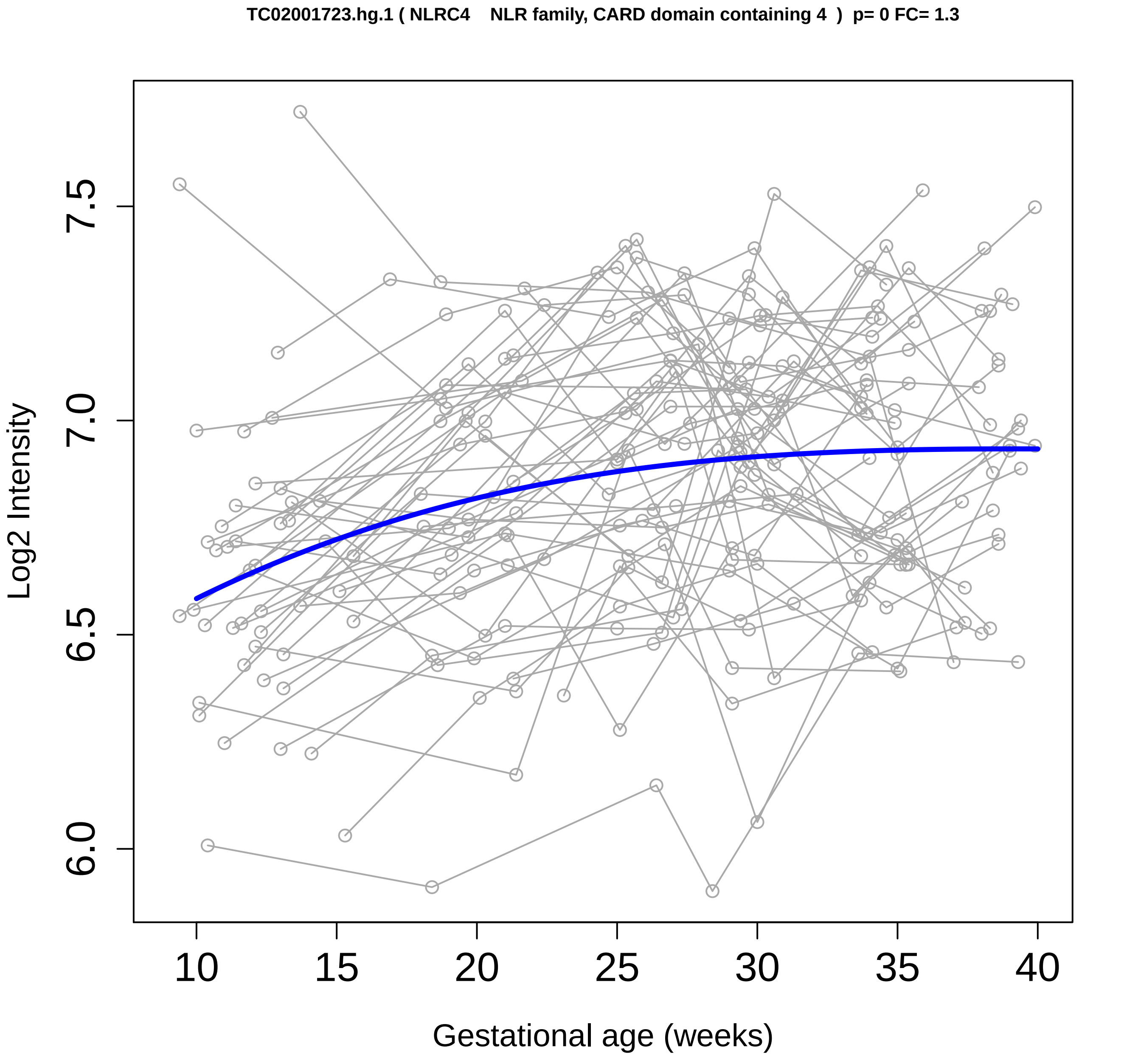
TC02001723.hg.1 (NLRC4 NLR family, CARD domain containing 4) p= 0 FC= 1.3
Log2 Intensity
6.0
6.5
7.0
7.5
10
15
20
25
30
35
40
Gestational age (weeks)

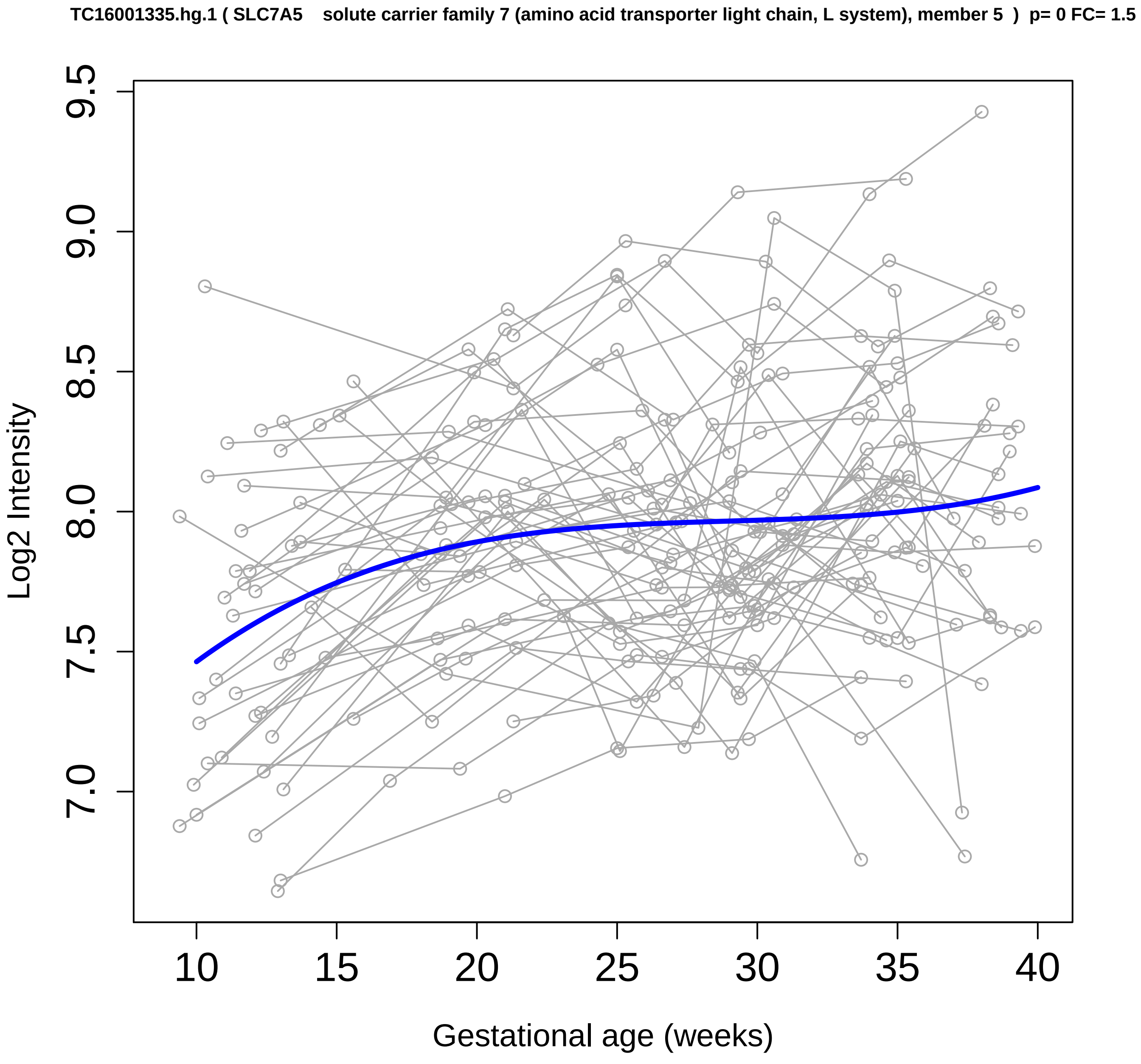

TC16001335.hg.1 (SLC7A5 solute carrier family 7 (amino acid transporter light chain, L system), member 5) p= 0 FC= 1.5
Log2 Intensity
9.5
9.0
8.5
8.0
7.5
7.0
10
15
20
25
30
35
40
Gestational age (weeks)

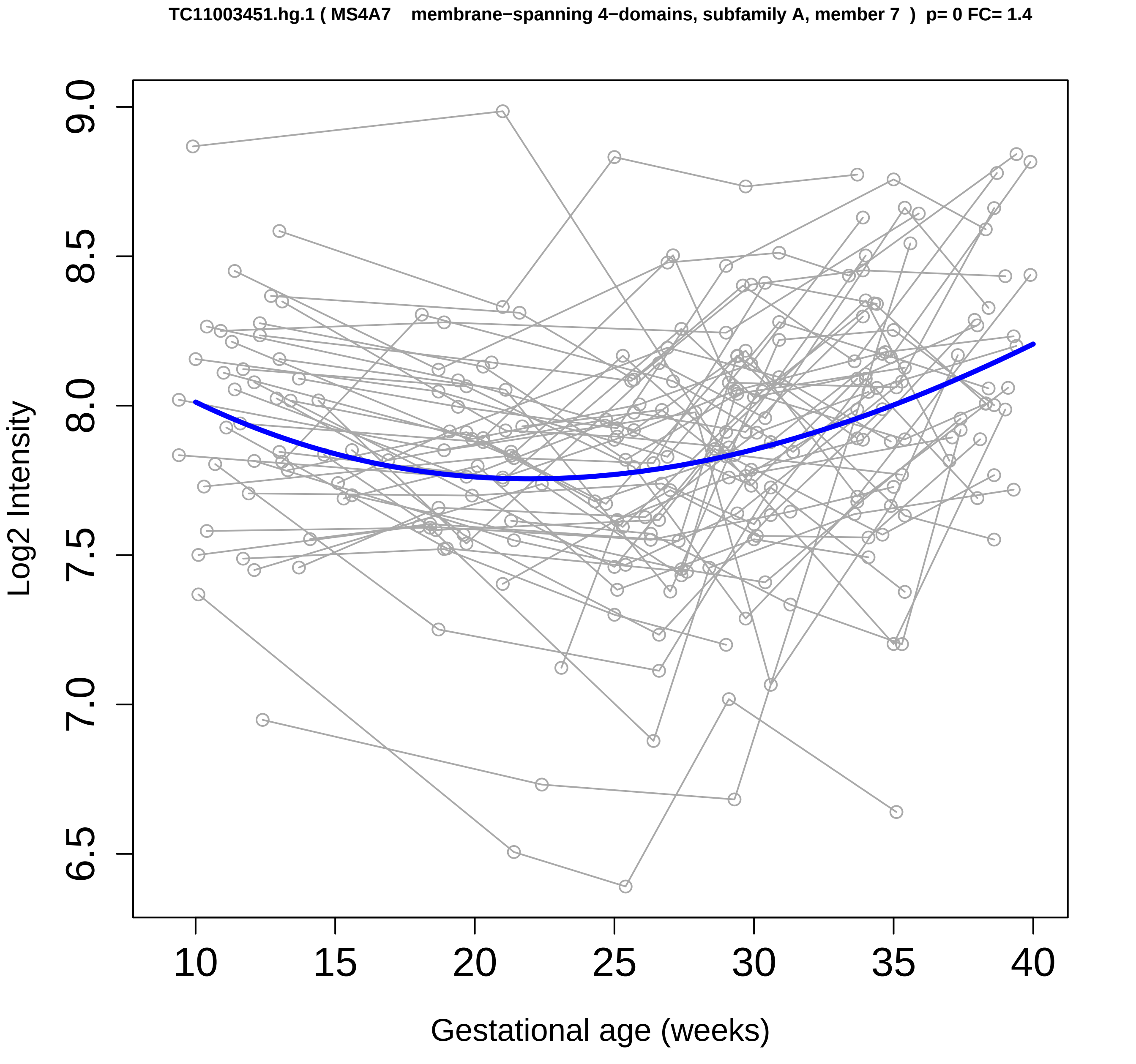

TC11003451.hg.1 (MS4A7 membrane−spanning 4−domains, subfamily A, member 7) p= 0 FC= 1.4
Log2 Intensity
9.0
8.5
8.0
7.5
7.0
6.5
10
15
20
25
30
35
40
Gestational age (weeks)

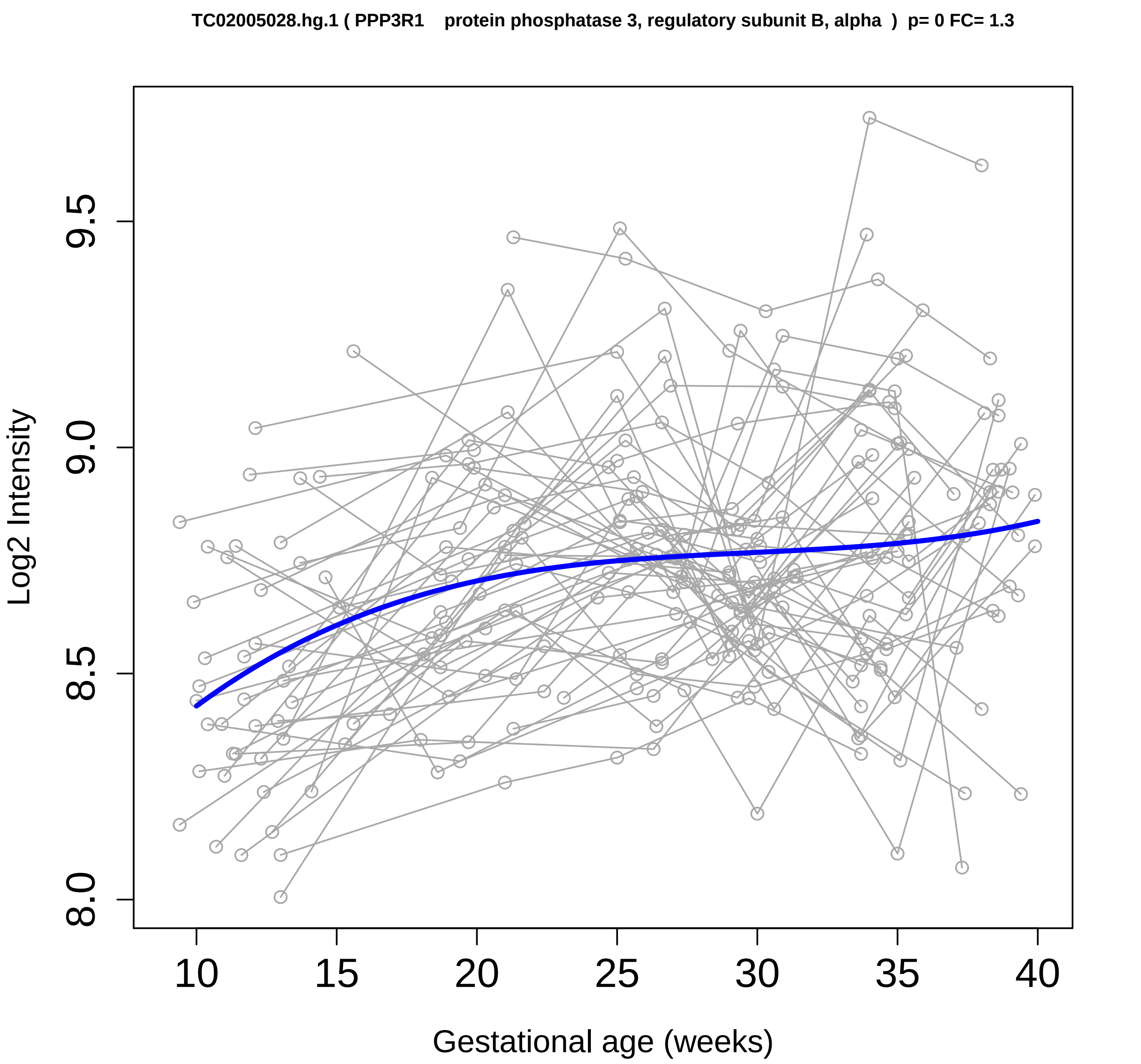
TC02005028.hg.1 (PPP3R1 protein phosphatase 3, regulatory subunit B, alpha) p= 0 FC= 1.3
Log2 Intensity
8.0
8.5
9.0
9.5
10
15
20
25
30
35
40
Gestational age (weeks)

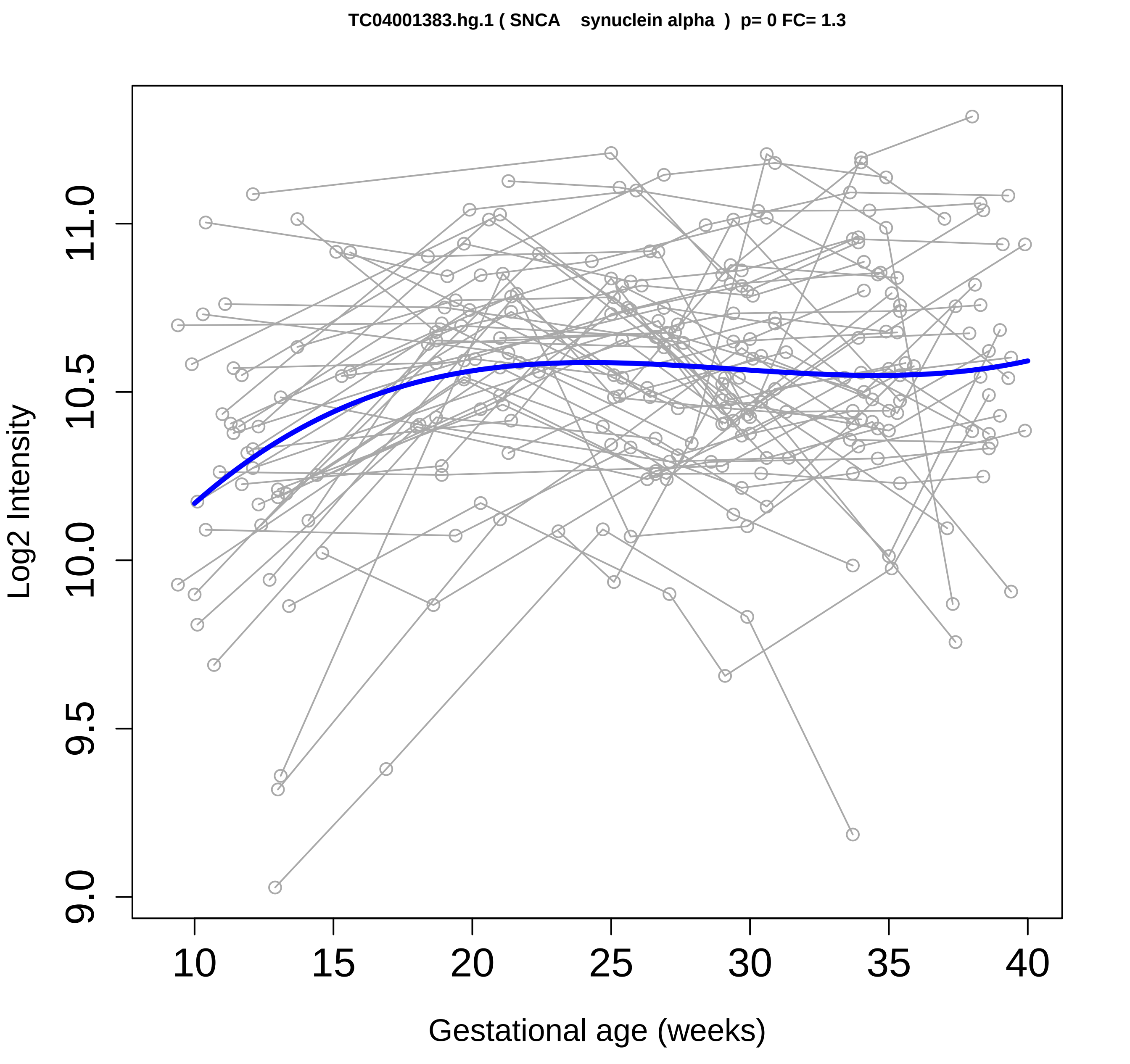
TC04001383.hg.1 (SNCA synuclein alpha) p= 0 FC= 1.3
Log2 Intensity
9.0
9.5
10.0
10.5
11.0
10
15
20
25
30
35
40
Gestational age (weeks)

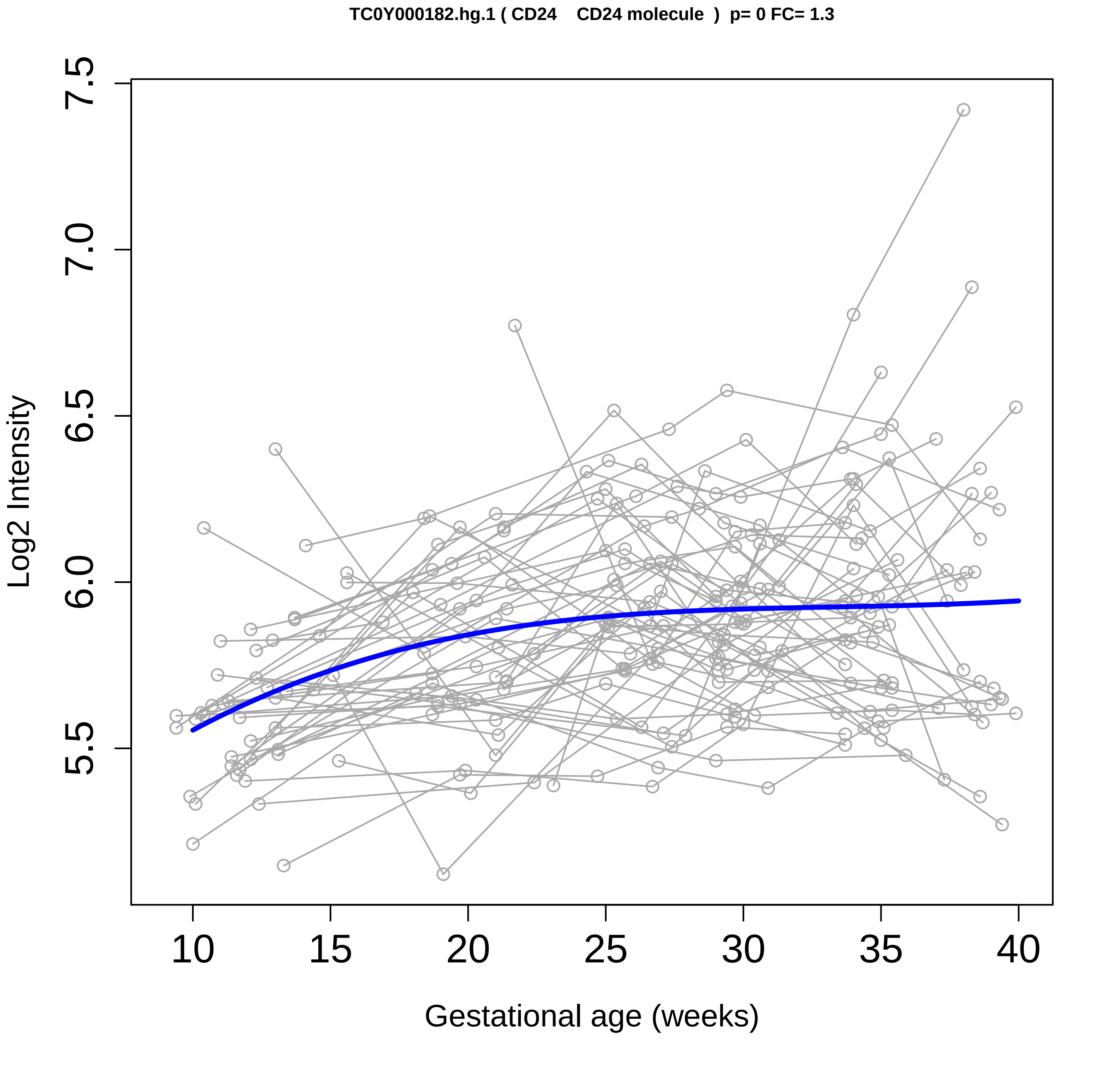
TC0Y000182.hg.1 (CD24   CD24 molecule) p= 0 FC= 1.3
Log2 Intensity
7.5
7.0
6.5
6.0
5.5
10
15
20
25
30
35
40
Gestational age (weeks)

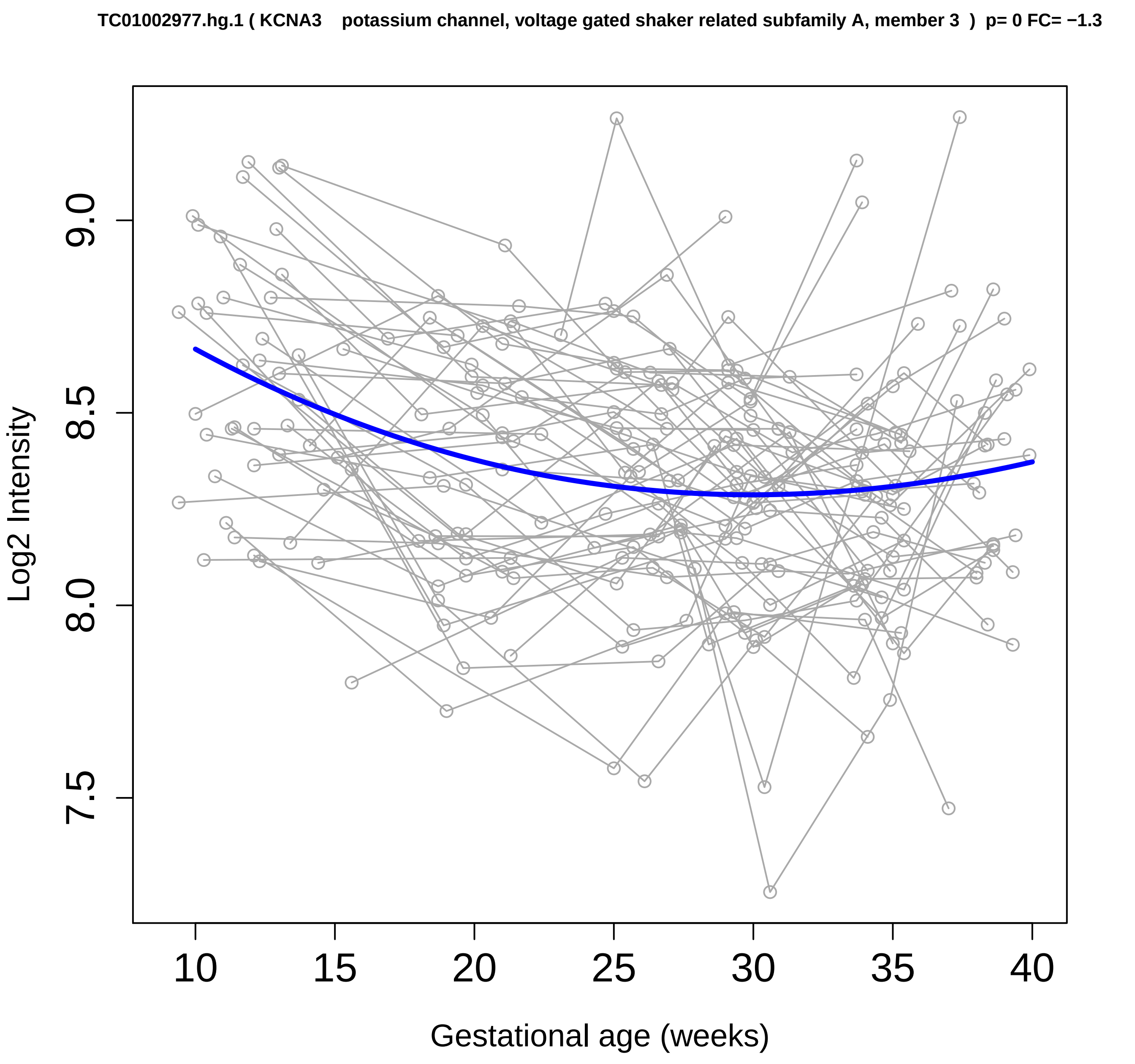

TC01002977.hg.1 (KCNA3 potassium channel, voltage gated shaker related subfamily A, member 3) p= 0 FC= −1.3
Log2 Intensity
7.5
8.0
8.5
9.0
10
15
20
25
30
35
40
Gestational age (weeks)

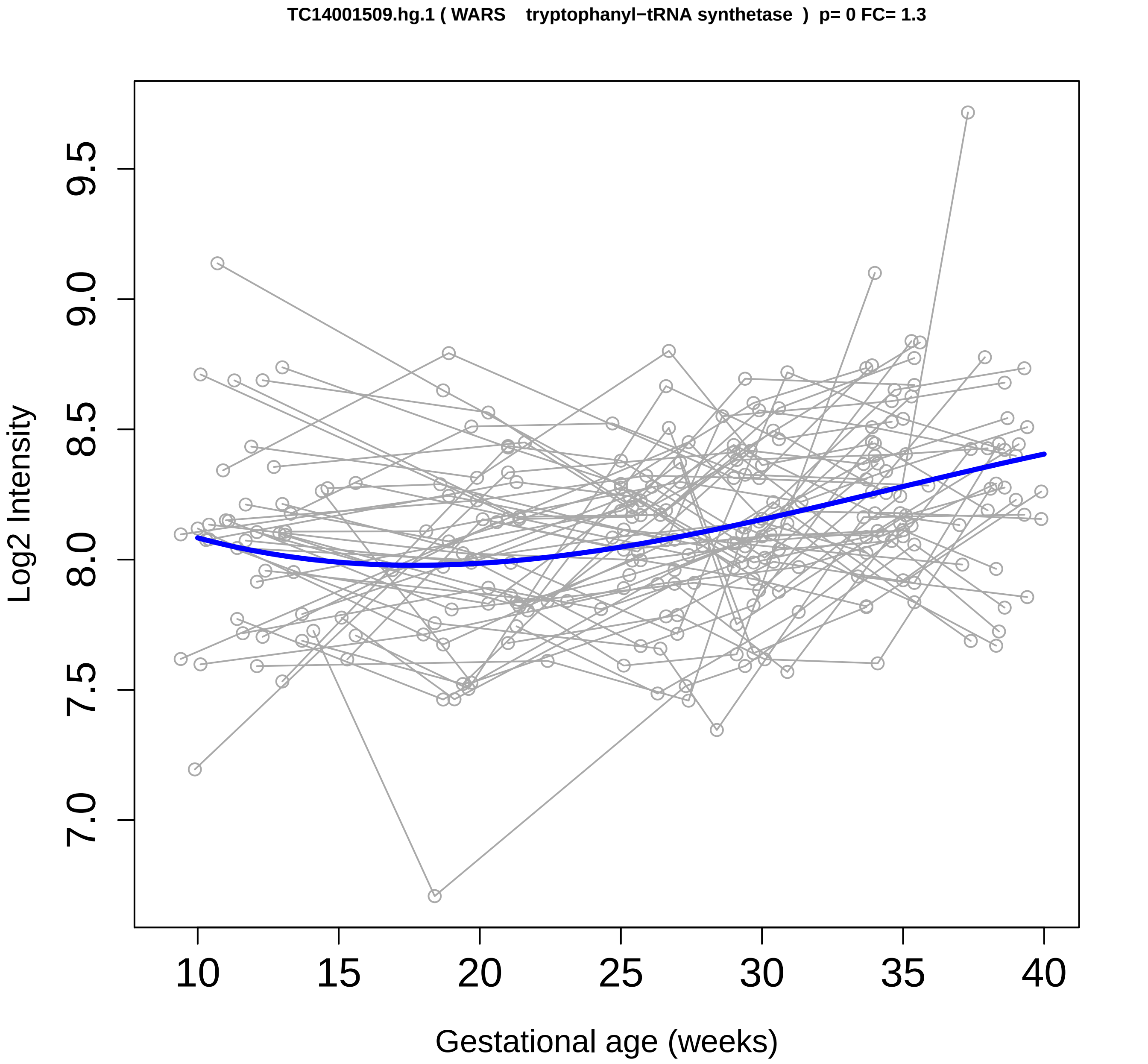

TC14001509.hg.1 (WARS tryptophanyl−tRNA synthetase) p= 0 FC= 1.3
Log2 Intensity
9.5
9.0
8.5
8.0
7.5
7.0
10
15
20
25
30
35
40
Gestational age (weeks)

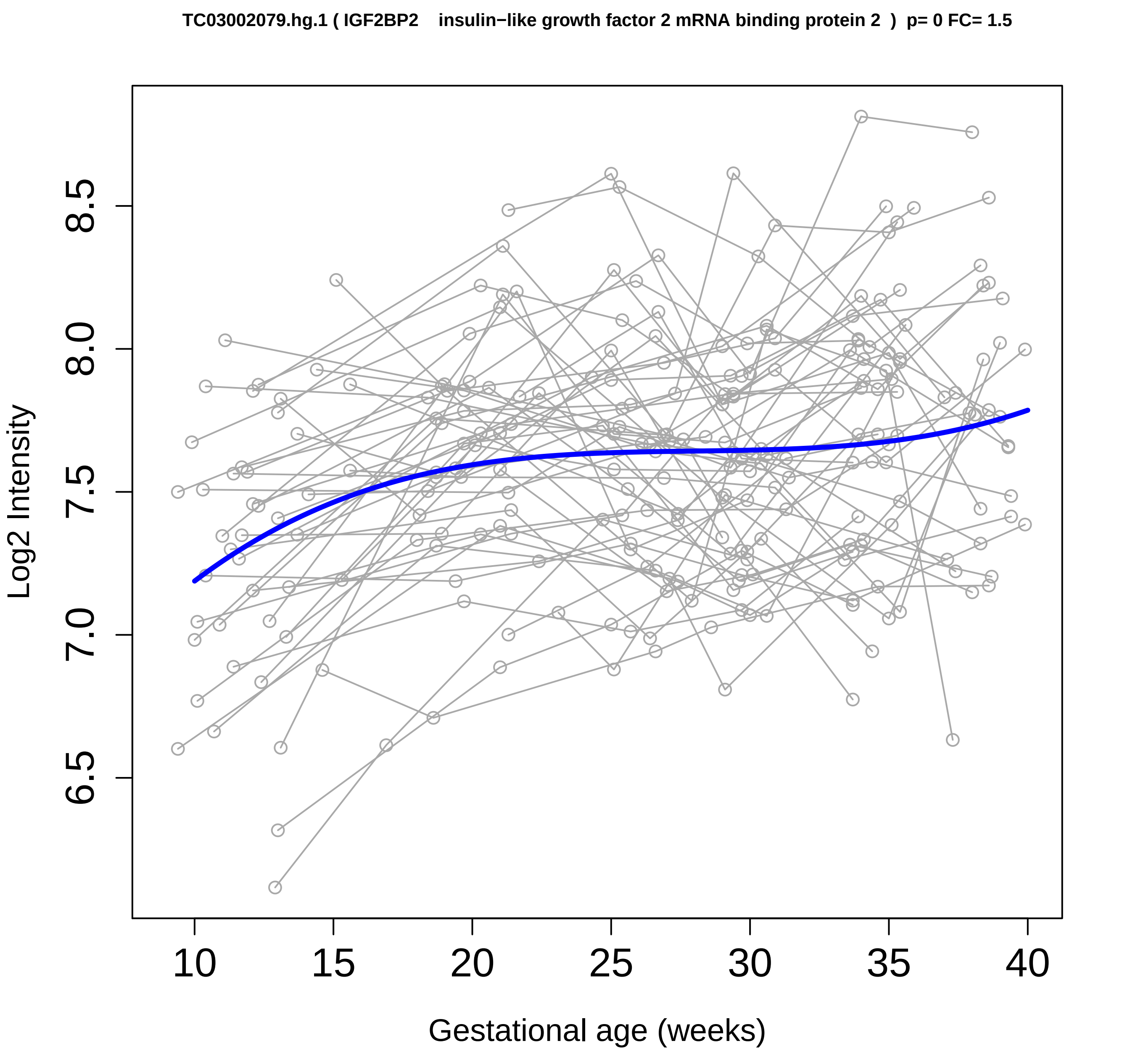

TC03002079.hg.1 (IGF2BP2   insulin−like growth factor 2 mRNA binding protein 2) p= 0 FC= 1.5
Log2 Intensity
6.5
7.0
7.5
8.0
8.5
10
15
20
25
30
35
40
Gestational age (weeks)

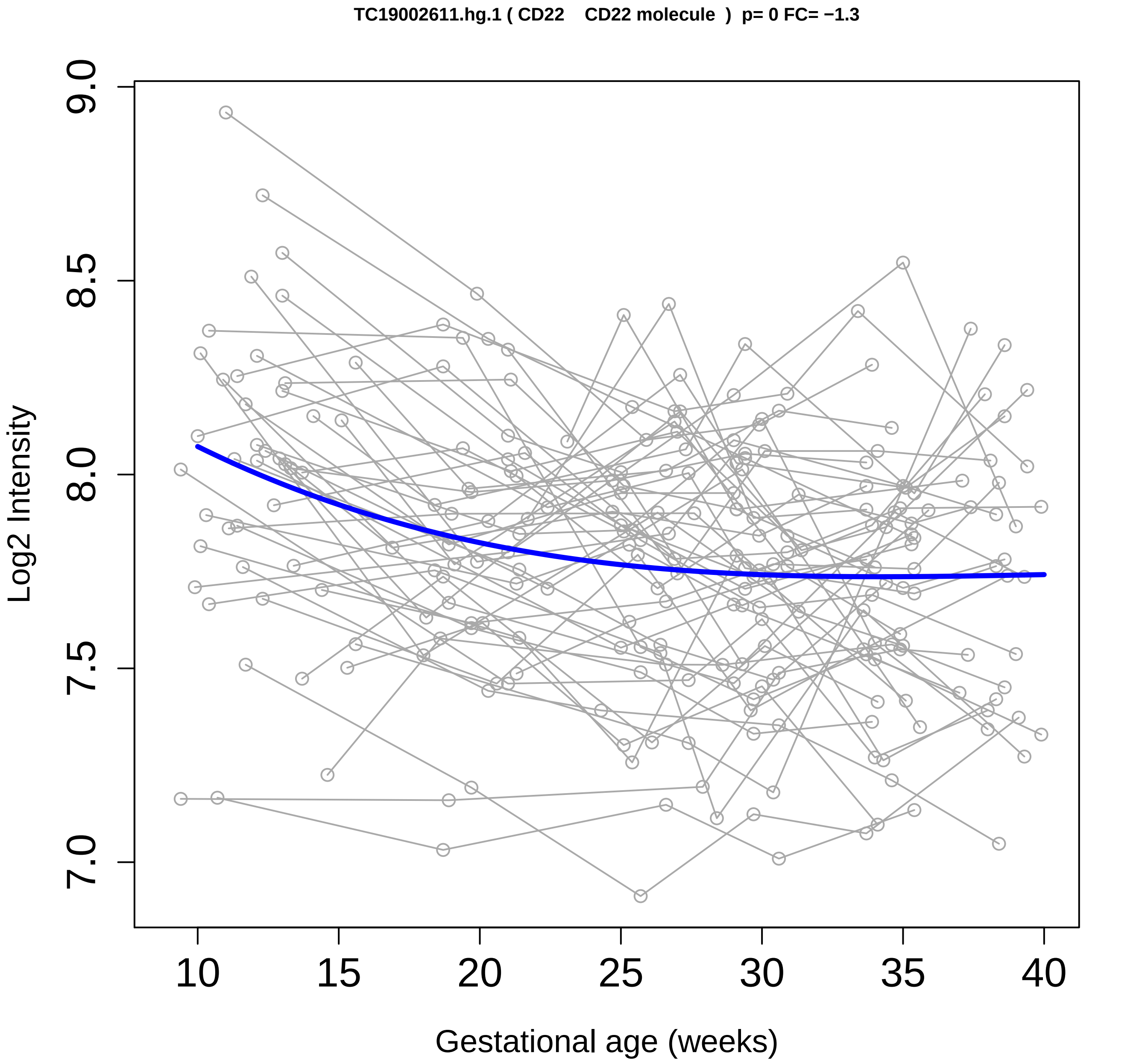
TC19002611.hg.1 (CD22 CD22 molecule) p= 0 FC= −1.3
Log2 Intensity
9.0
8.5
8.0
7.5
7.0
10
15
20
25
30
35
40
Gestational age (weeks)

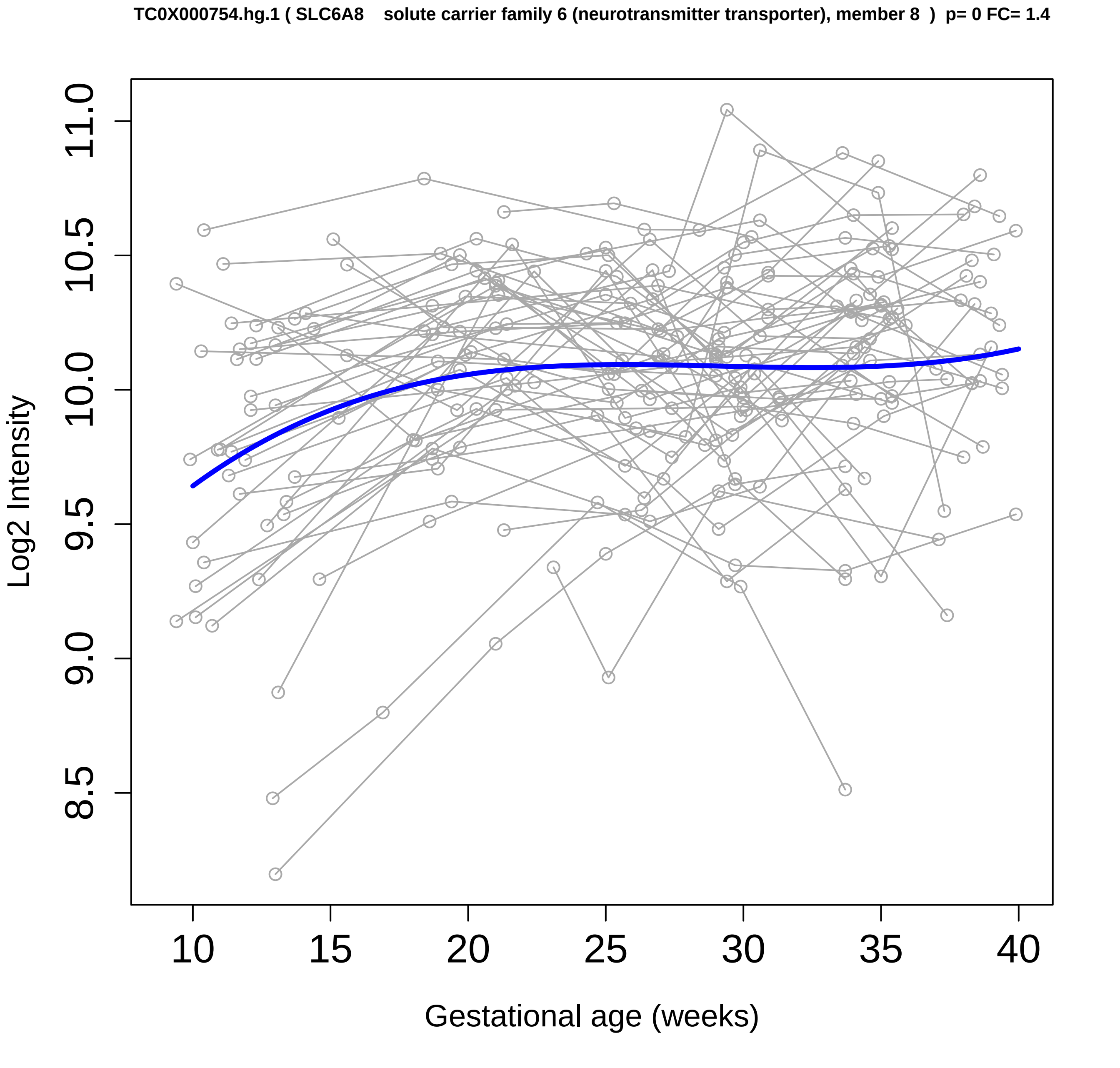

TC0X000754.hg.1 (SLC6A8 solute carrier family 6 (neurotransmitter transporter), member 8) p= 0 FC= 1.4
Log2 Intensity
11.0
10.5
10.0
9.5
9.0
8.5
10
15
20
25
30
35
40
Gestational age (weeks)

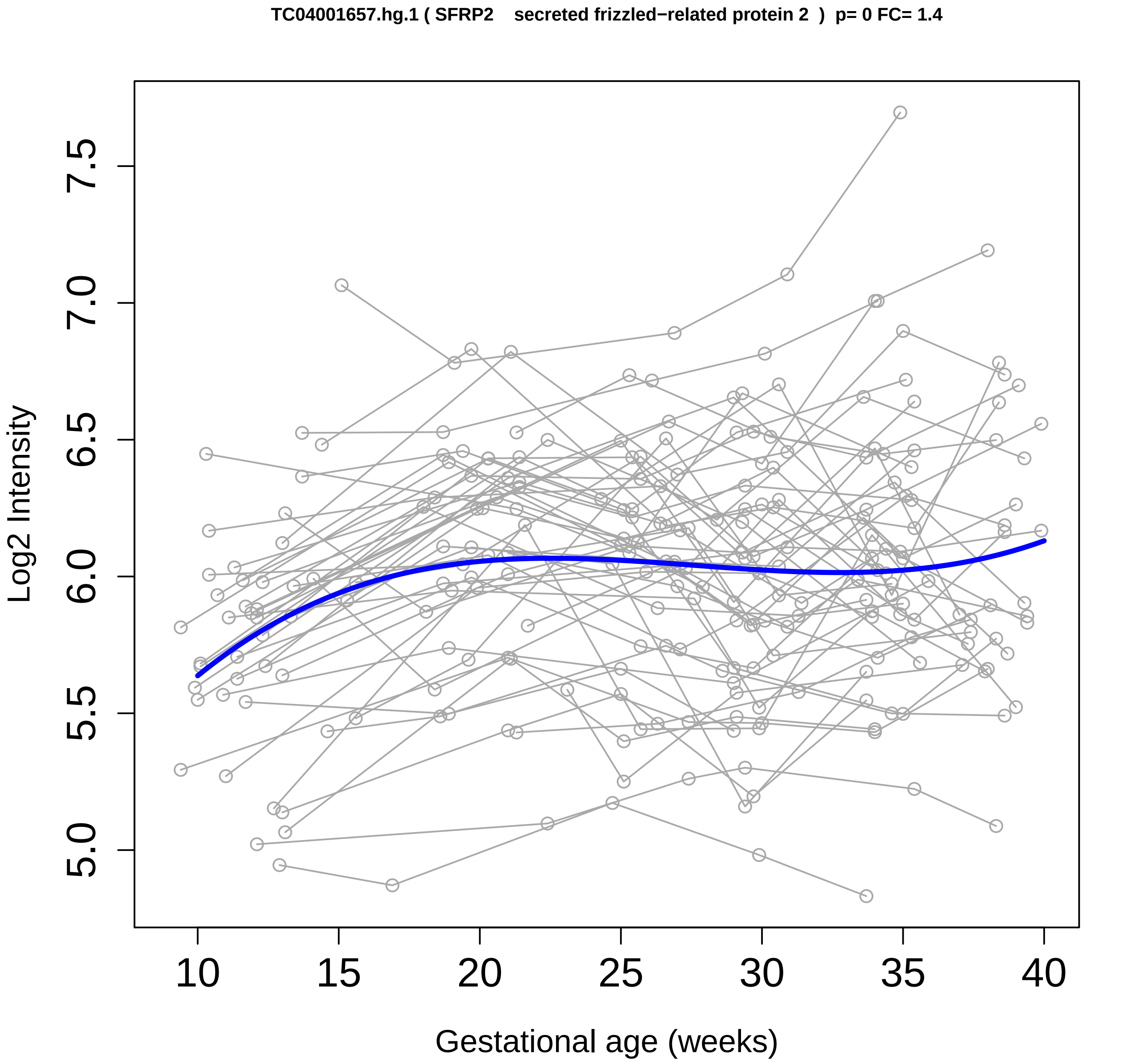

TC04001657.hg.1 (SFRP2 secreted frizzled−related protein 2) p= 0 FC= 1.4
Log2 Intensity
5.0
5.5
6.0
6.5
7.0
7.5
10
15
20
25
30
35
40
Gestational age (weeks)

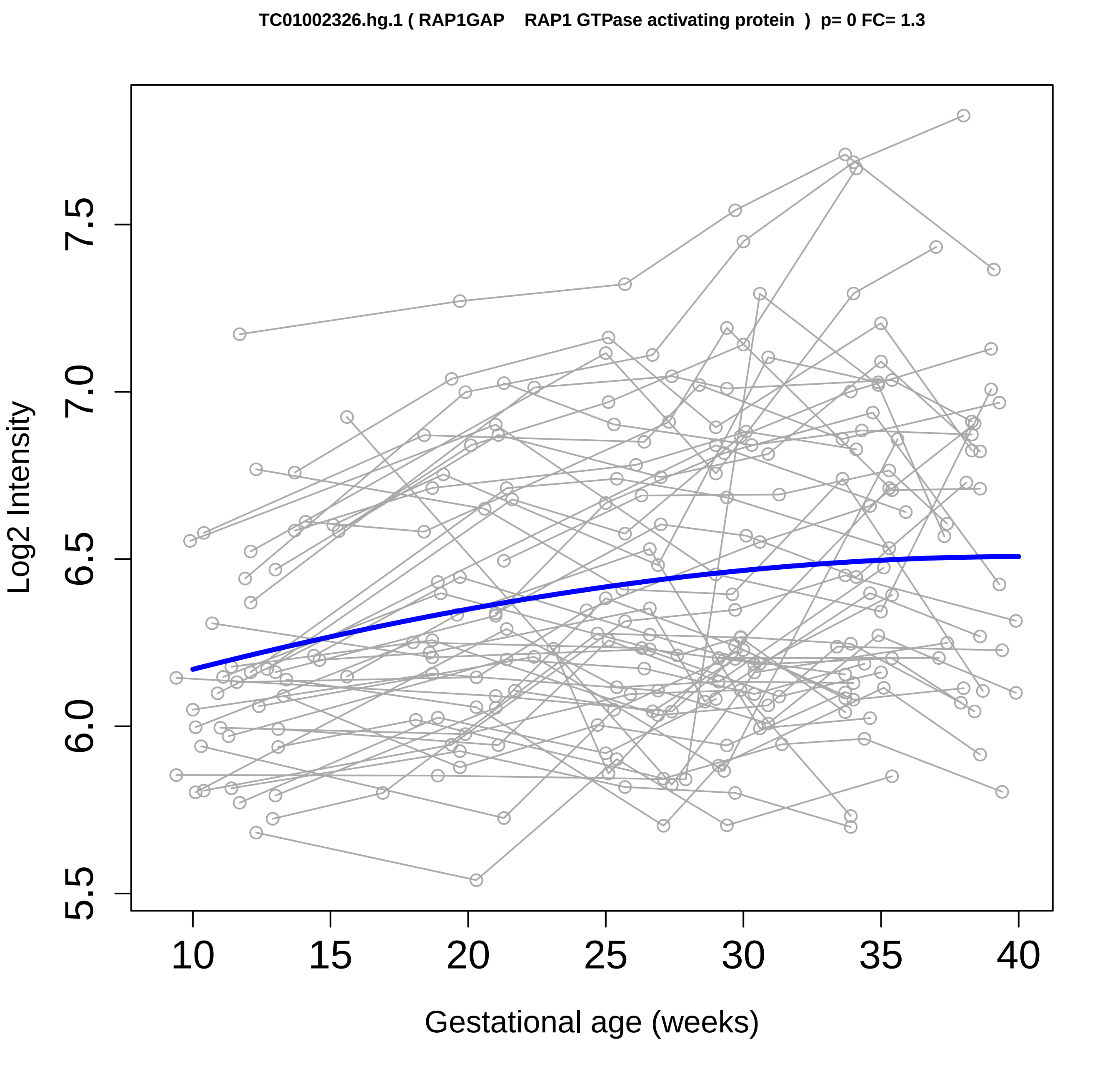

TC01002326.hg.1 (RAP1GAP RAP1 GTPase activating protein) p= 0 FC= 1.3
Log2 Intensity
5.5
6.0
6.5
7.0
7.5
10
15
20
25
30
35
40
Gestational age (weeks)

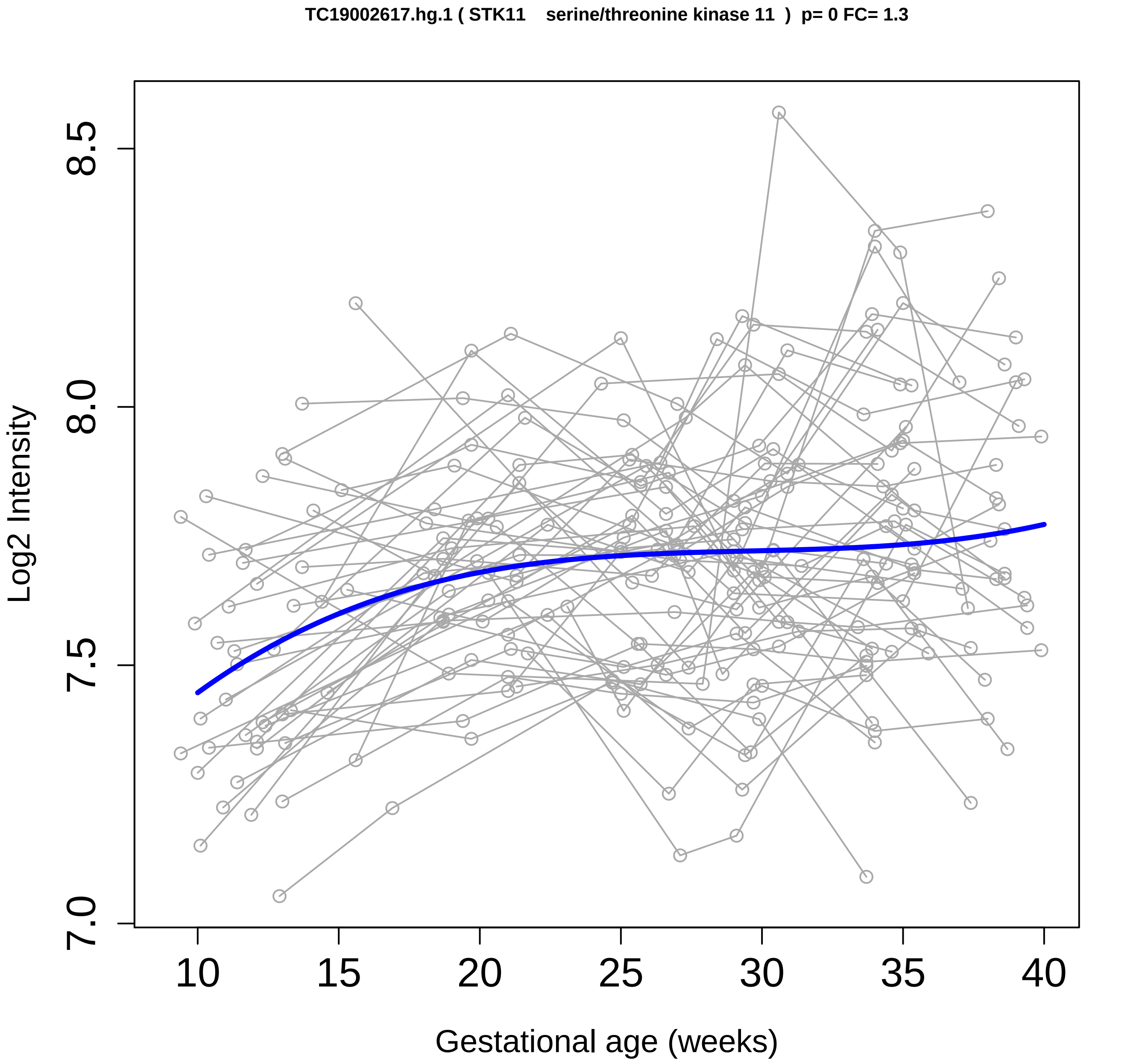

TC19002617.hg.1 (STK11 serine/threonine kinase 11) p= 0 FC= 1.3
Log2 Intensity
7.0
7.5
8.0
8.5
10
15
20
25
30
35
40
Gestational age (weeks)

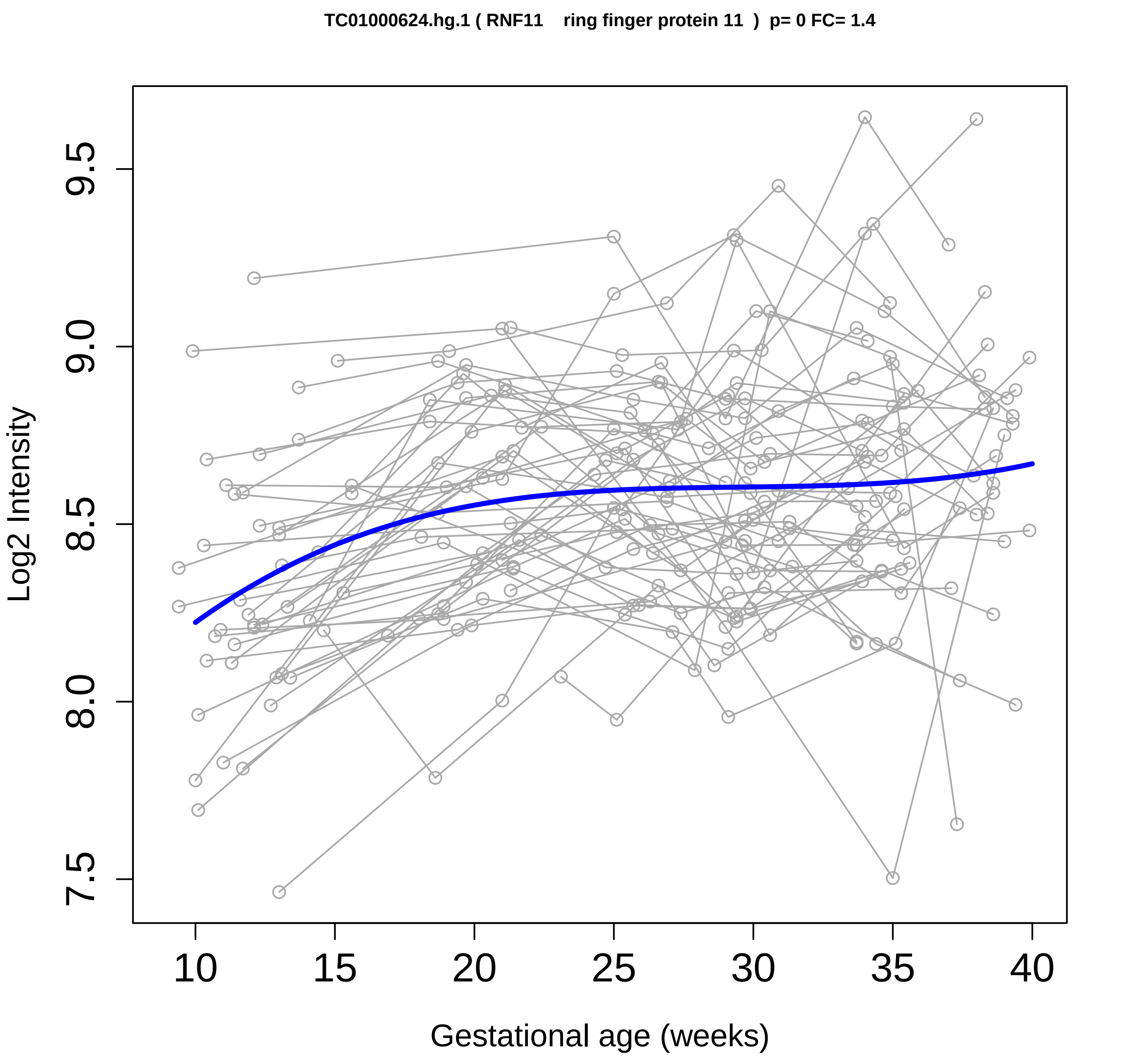
TC01000624.hg.1 (RNF11 ring finger protein 11) p= 0 FC= 1.4
Log2 Intensity
9.5
9.0
8.5
8.0
7.5
10
15
20
25
30
35
40
Gestational age (weeks)

TC6_apd_hap1000069.hg.1 (TRIM10 tripartite motif containing 10) p= 0 FC= 1.4

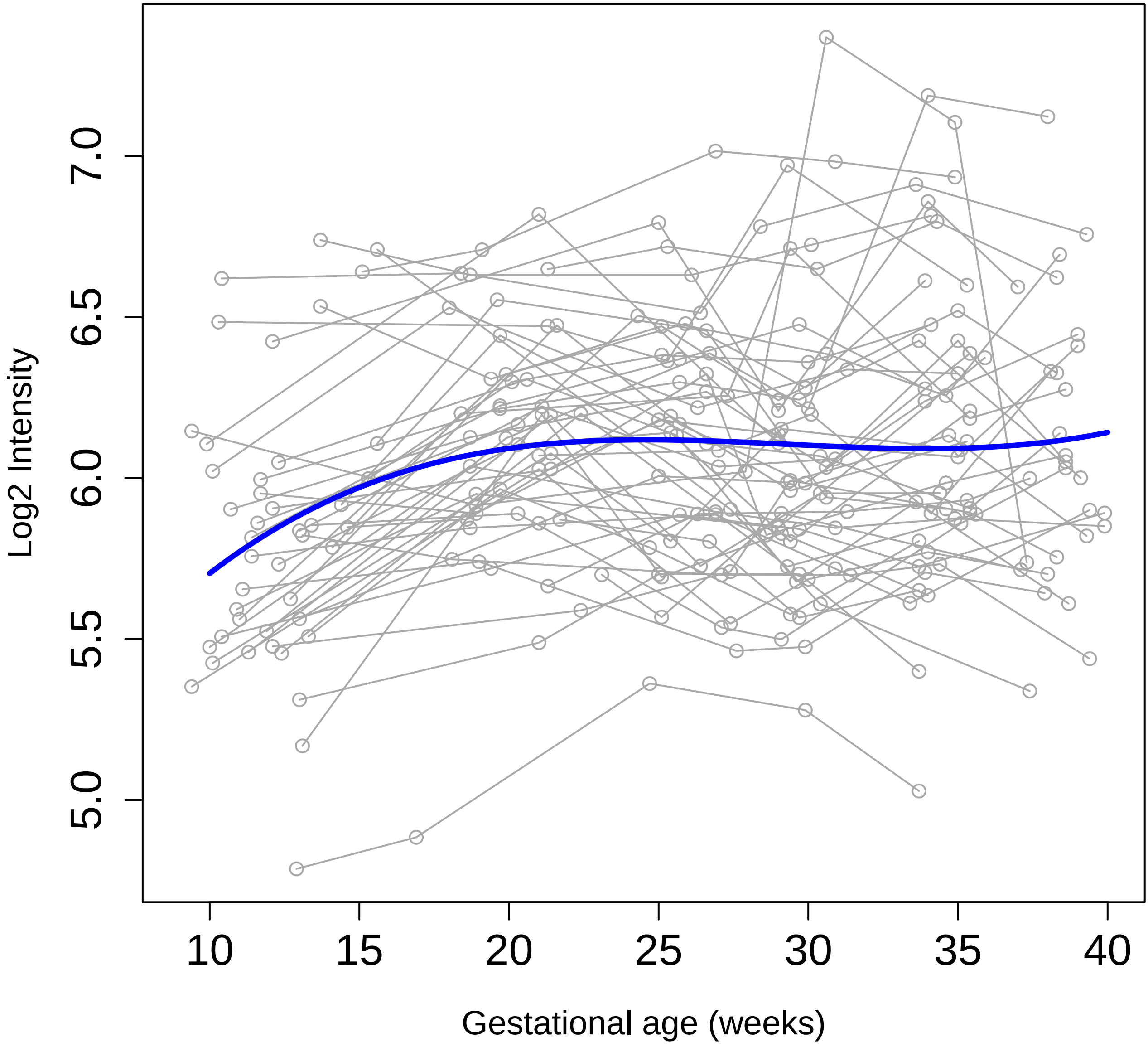

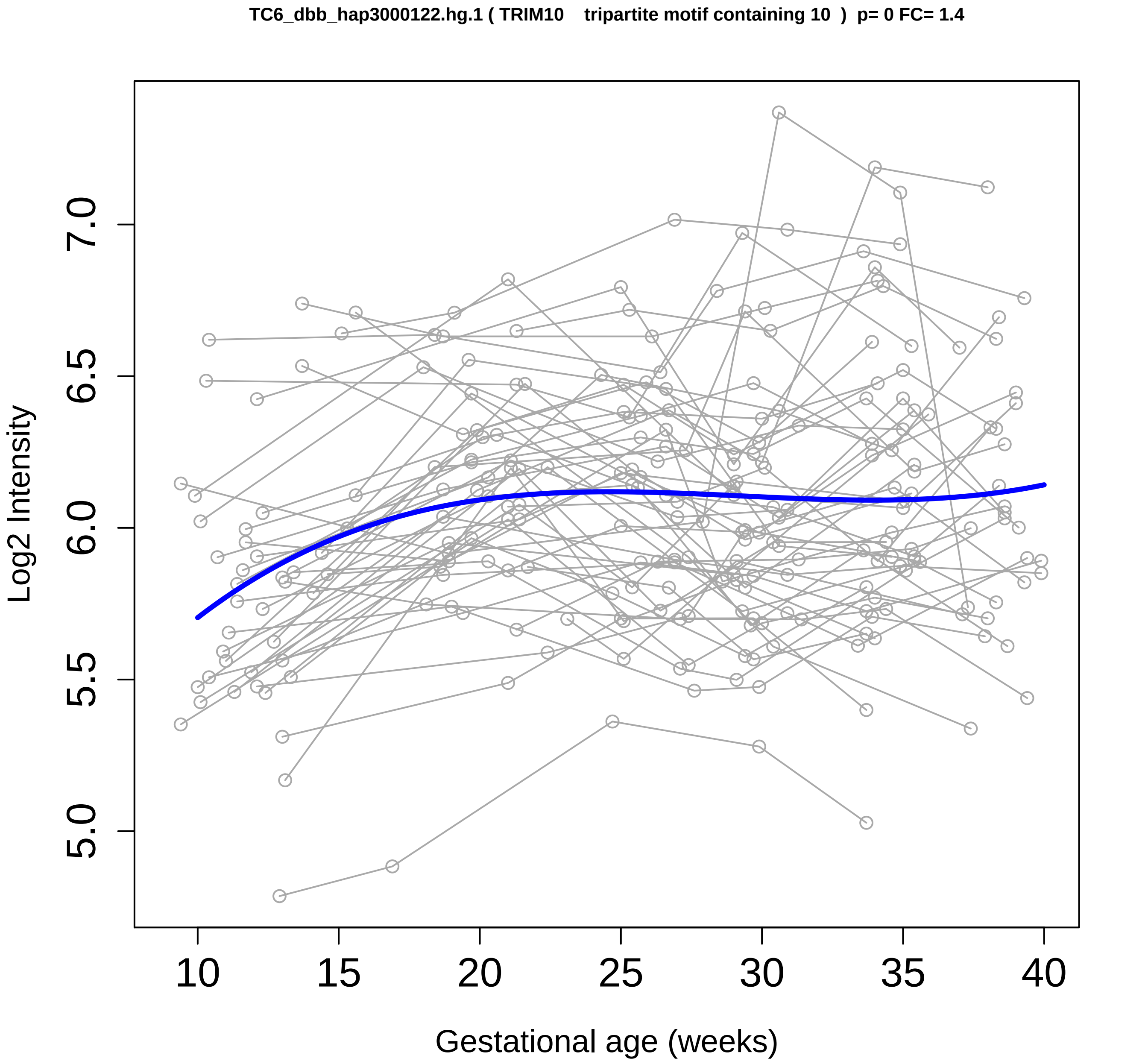

TC6_dbb_hap3000122.hg.1 (TRIM10 tripartite motif containing 10) p= 0 FC= 1.4
Log2 Intensity
5.0
5.5
6.0
6.5
7.0
10
15
20
25
30
35
40
Gestational age (weeks)

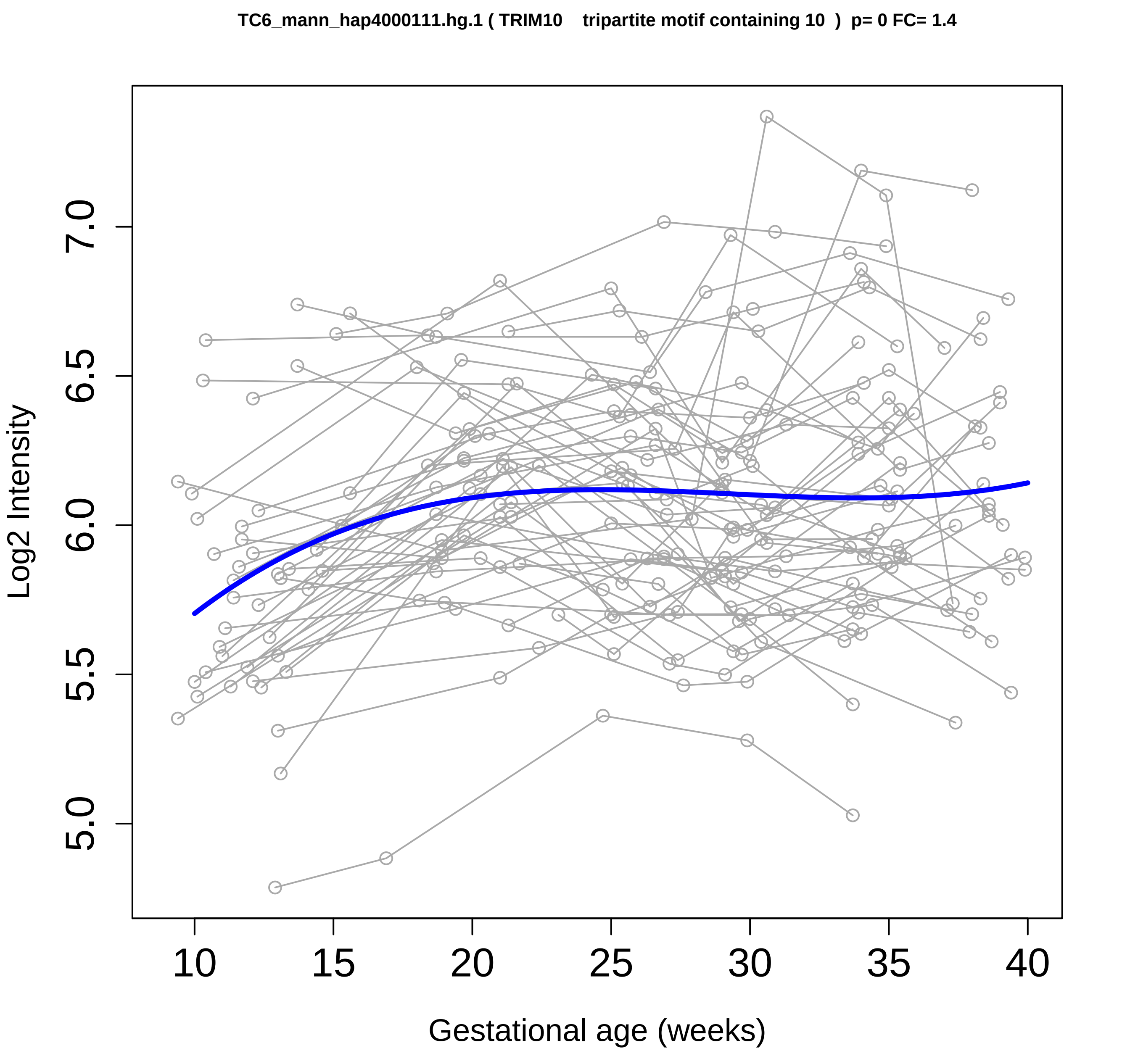
TC6_mann_hap4000111.hg.1 (TRIM10   tripartite motif containing 10) p= 0 FC= 1.4
Log2 Intensity
5.0
5.5
6.0
6.5
7.0
10
15
20
25
30
35
40
Gestational age (weeks)

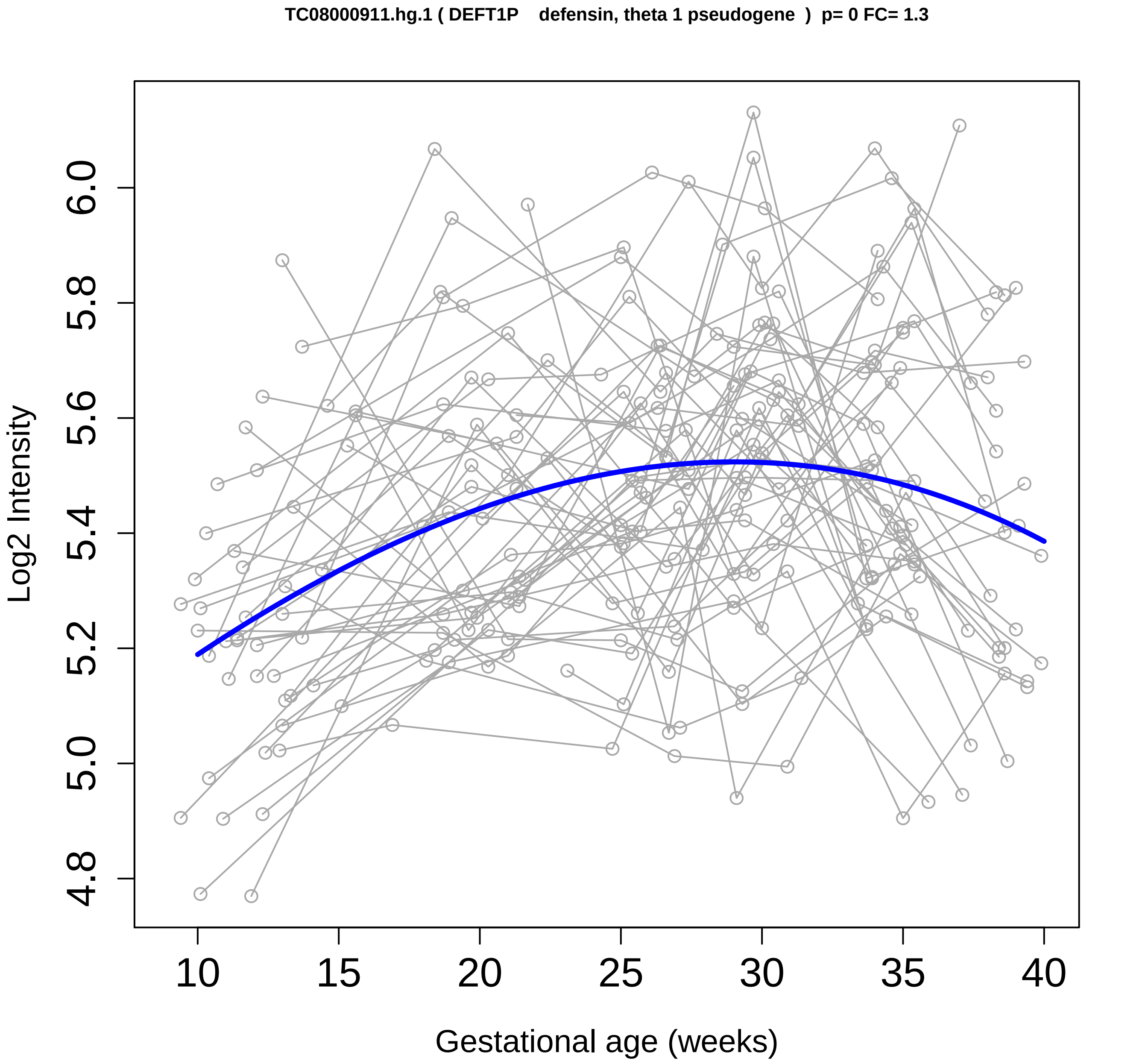
TC08000911.hg.1 (DEFT1P defensin, theta 1 pseudogene) p= 0 FC= 1.3
Log2 Intensity
4.8
5.0
5.2
5.4
5.6
5.8
6.0
10
15
20
25
30
35
40
Gestational age (weeks)

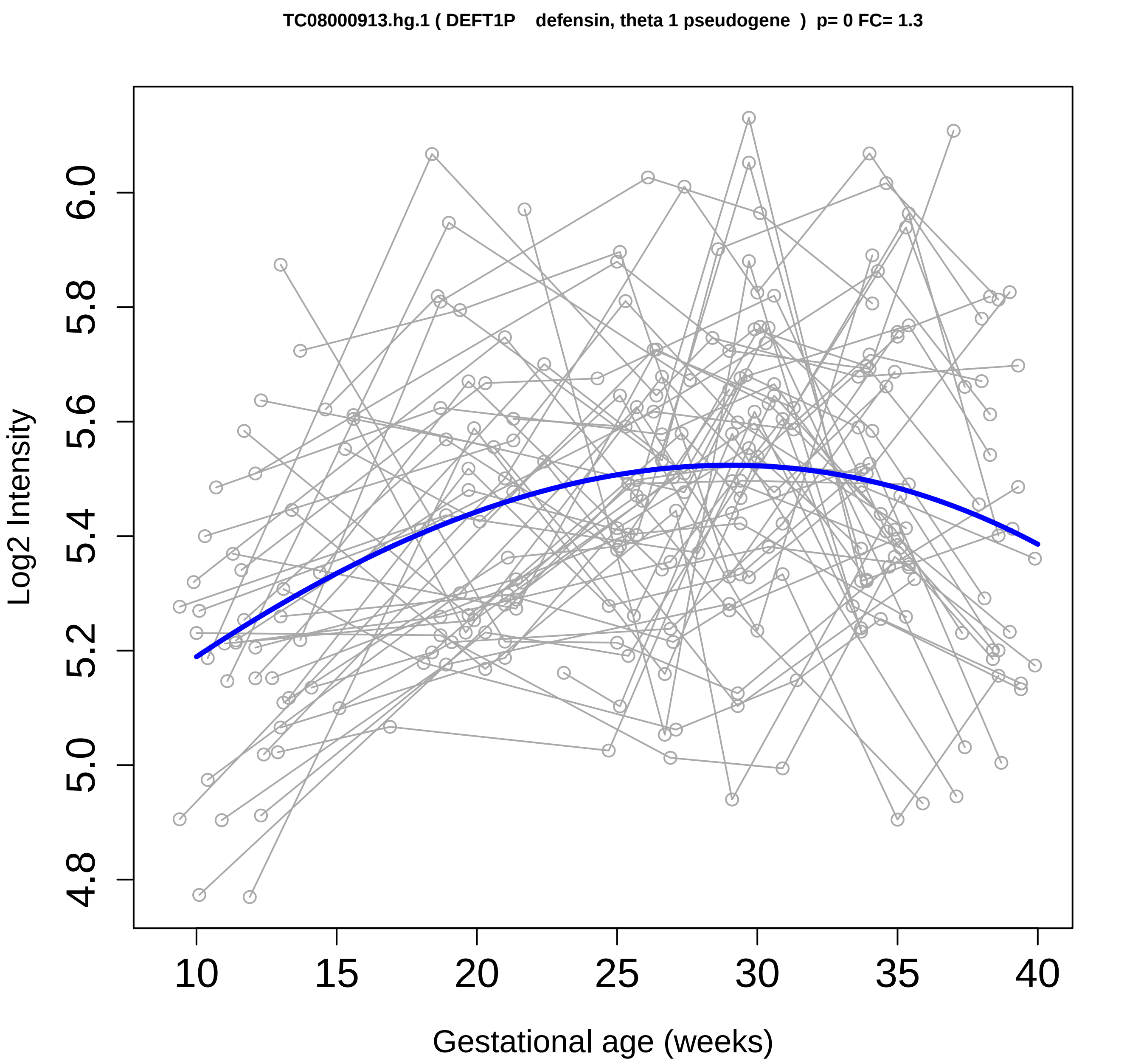

TC08000913.hg.1 (DEFT1P defensin, theta 1 pseudogene) p= 0 FC= 1.3
Log2 Intensity
4.8
5.0
5.2
5.4
5.6
5.8
6.0
10
15
20
25
30
35
40
Gestational age (weeks)

TC6_cox_hap2000133.hg.1 (TRIM10   tripartite motif containing 10) p= 0 FC= 1.4

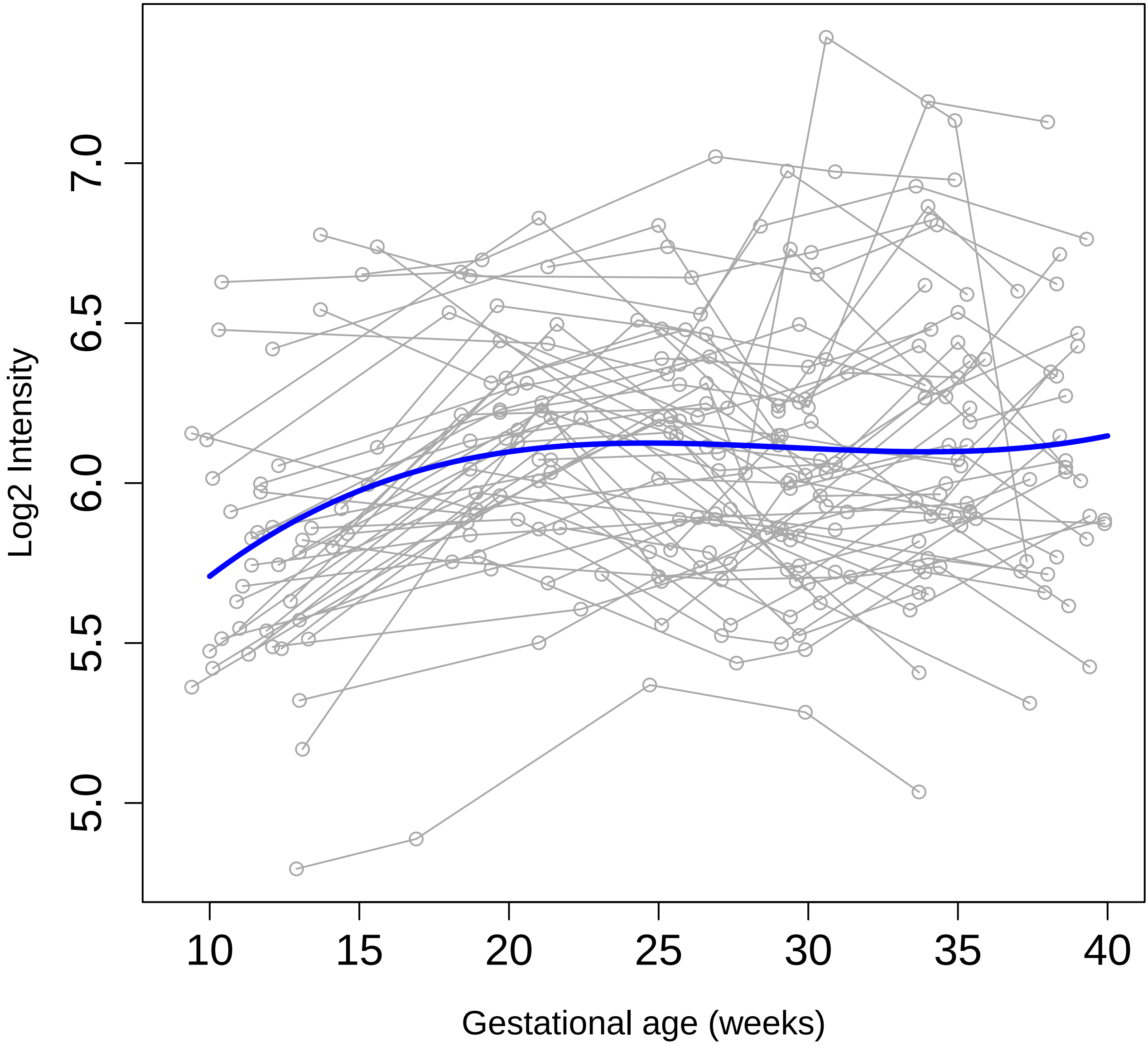

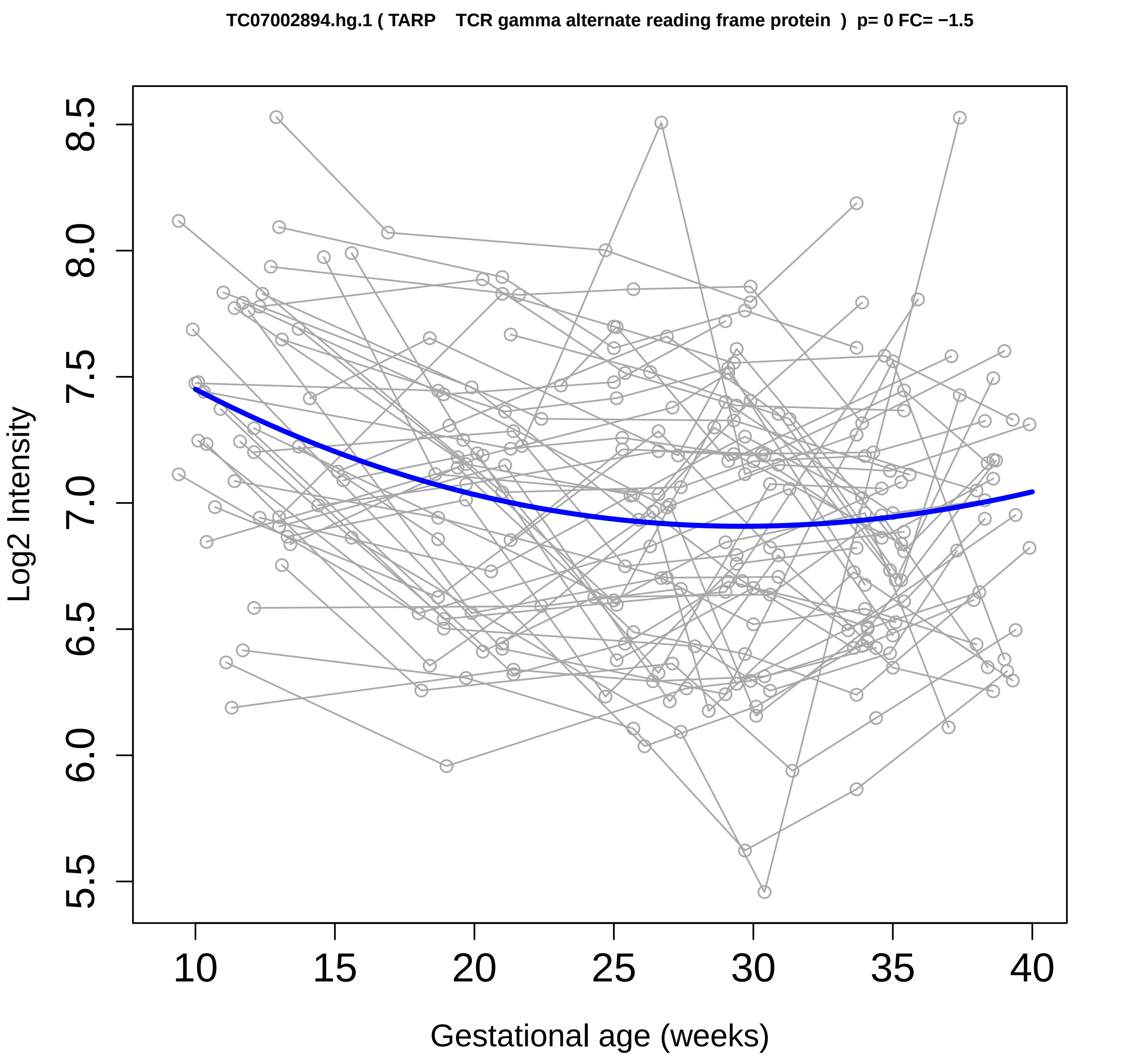
TC07002894.hg.1 (TARP TCR gamma alternate reading frame protein) p= 0 FC= −1.5
Log2 Intensity
8.5
8.0
7.5
7.0
6.5
6.0
5.5
10
15
20
25
30
35
40
Gestational age (weeks)

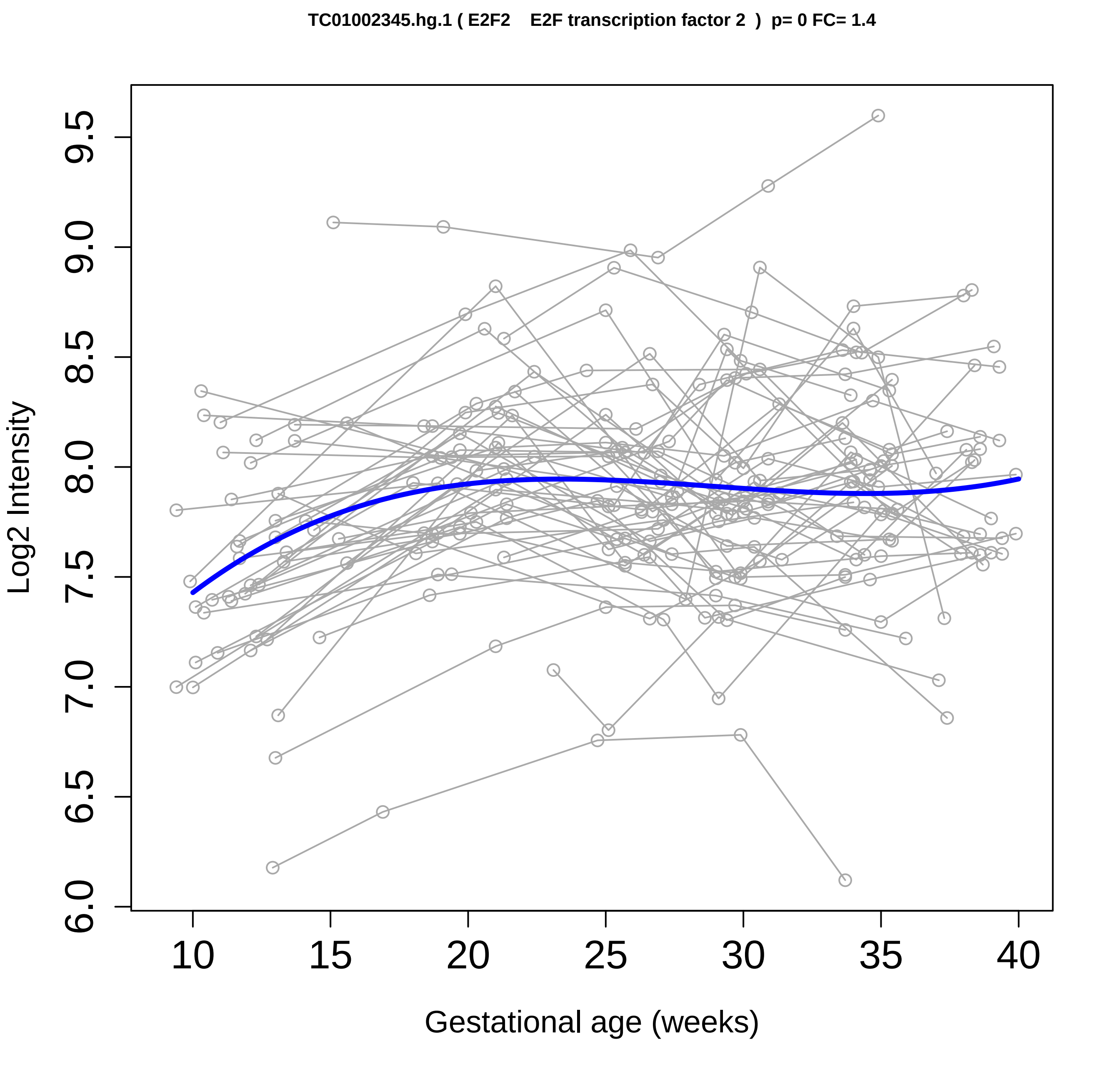

TC01002345.hg.1 (E2F2 E2F transcription factor 2) p= 0 FC= 1.4
Log2 Intensity
6.0
6.5
7.0
7.5
8.0
8.5
9.0
9.5
10
15
20
25
30
35
40
Gestational age (weeks)

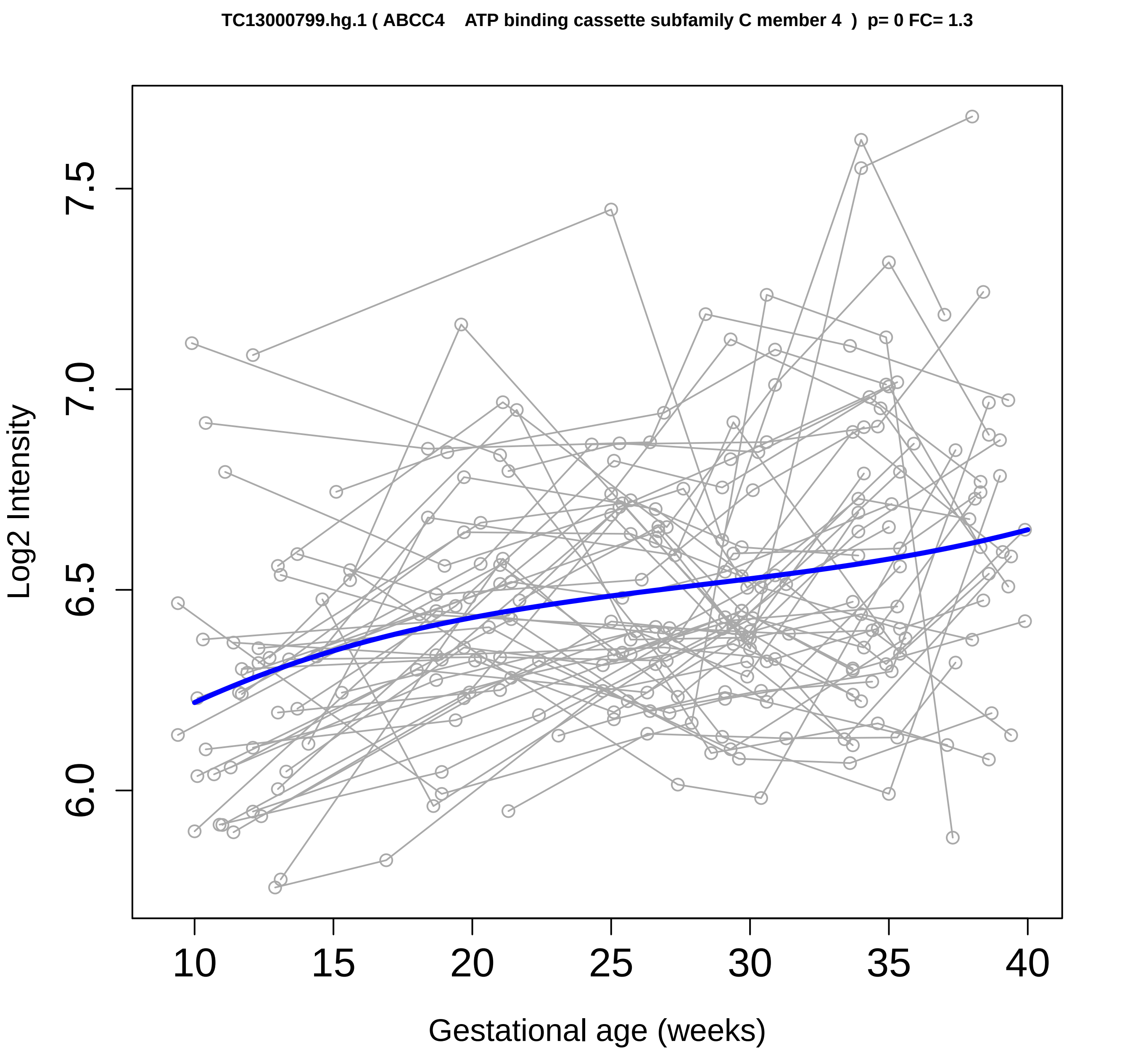

TC13000799.hg.1 (ABCC4 ATP binding cassette subfamily C member 4) p= 0 FC= 1.3
Log2 Intensity
7.5
7.0
6.5
6.0
10
15
20
25
30
35
40
Gestational age (weeks)

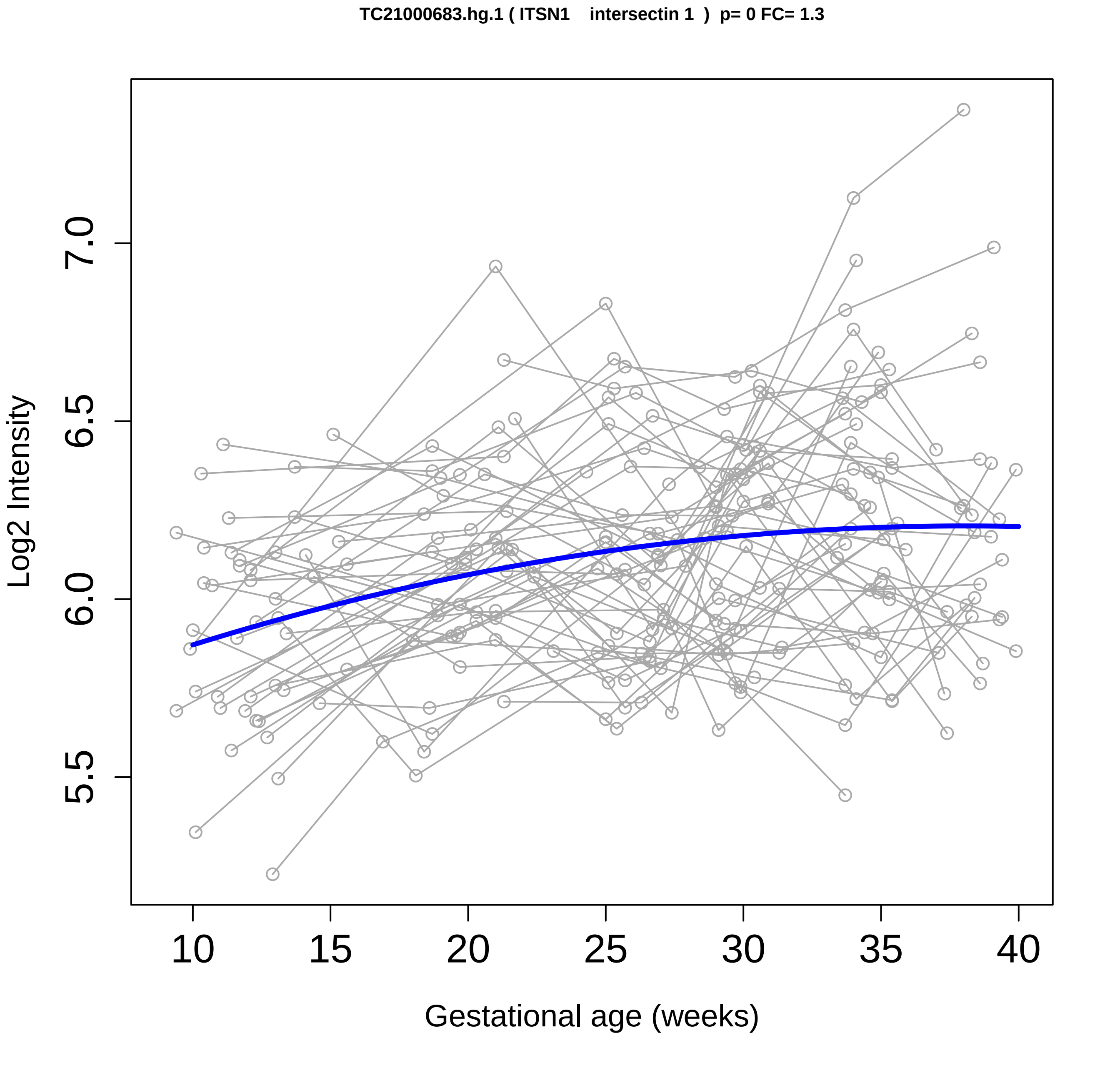
TC21000683.hg.1 (ITSN1   intersectin 1) p= 0 FC= 1.3
Log2 Intensity
5.5
6.0
6.5
7.0
10
15
20
25
30
35
40
Gestational age (weeks)

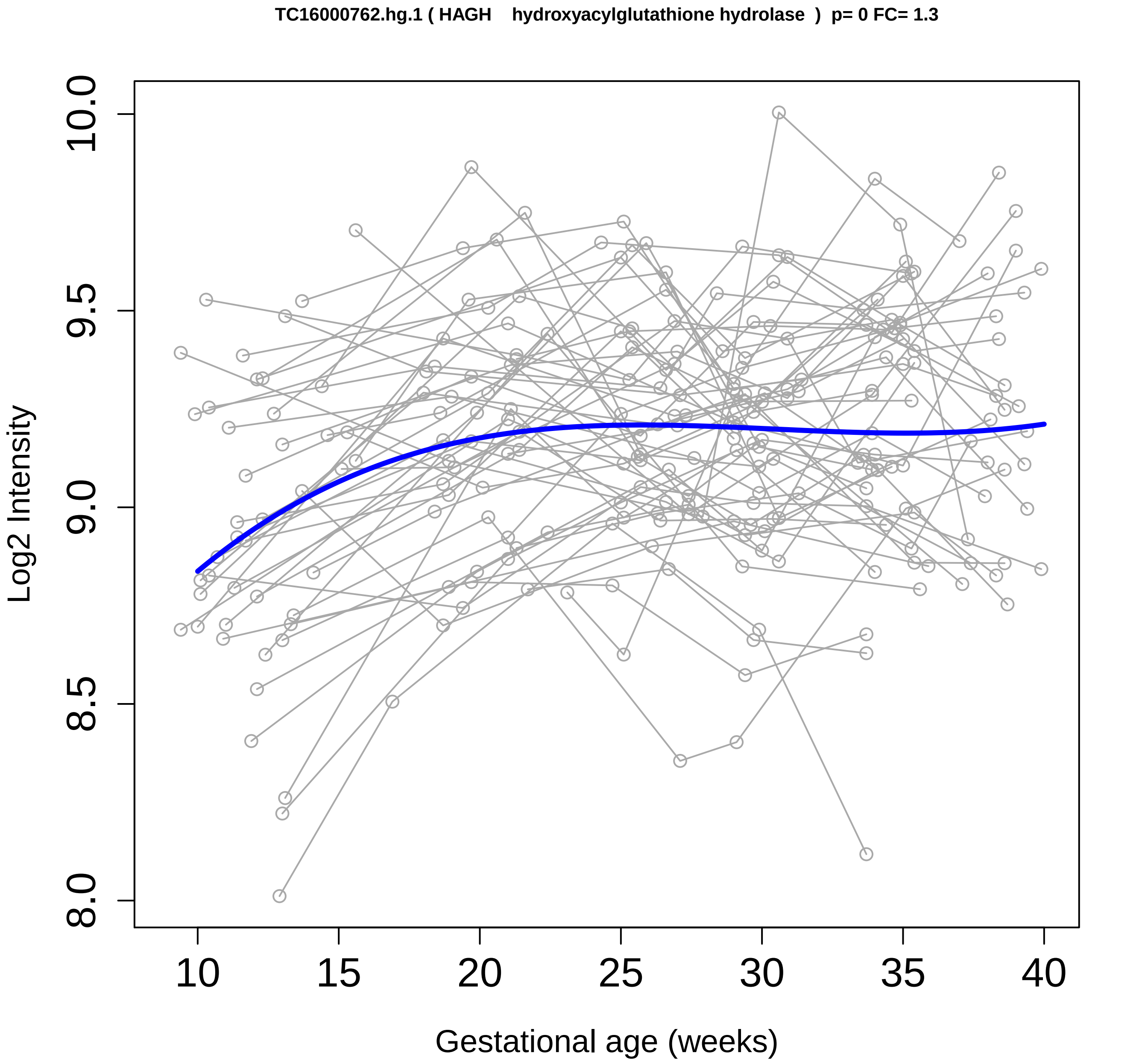
TC16000762.hg.1 (HAGH hydroxyacylglutathione hydrolase) p= 0 FC= 1.3
Log2 Intensity
8.0
8.5
9.0
9.5
10.0
10
15
20
25
30
35
40
Gestational age (weeks)

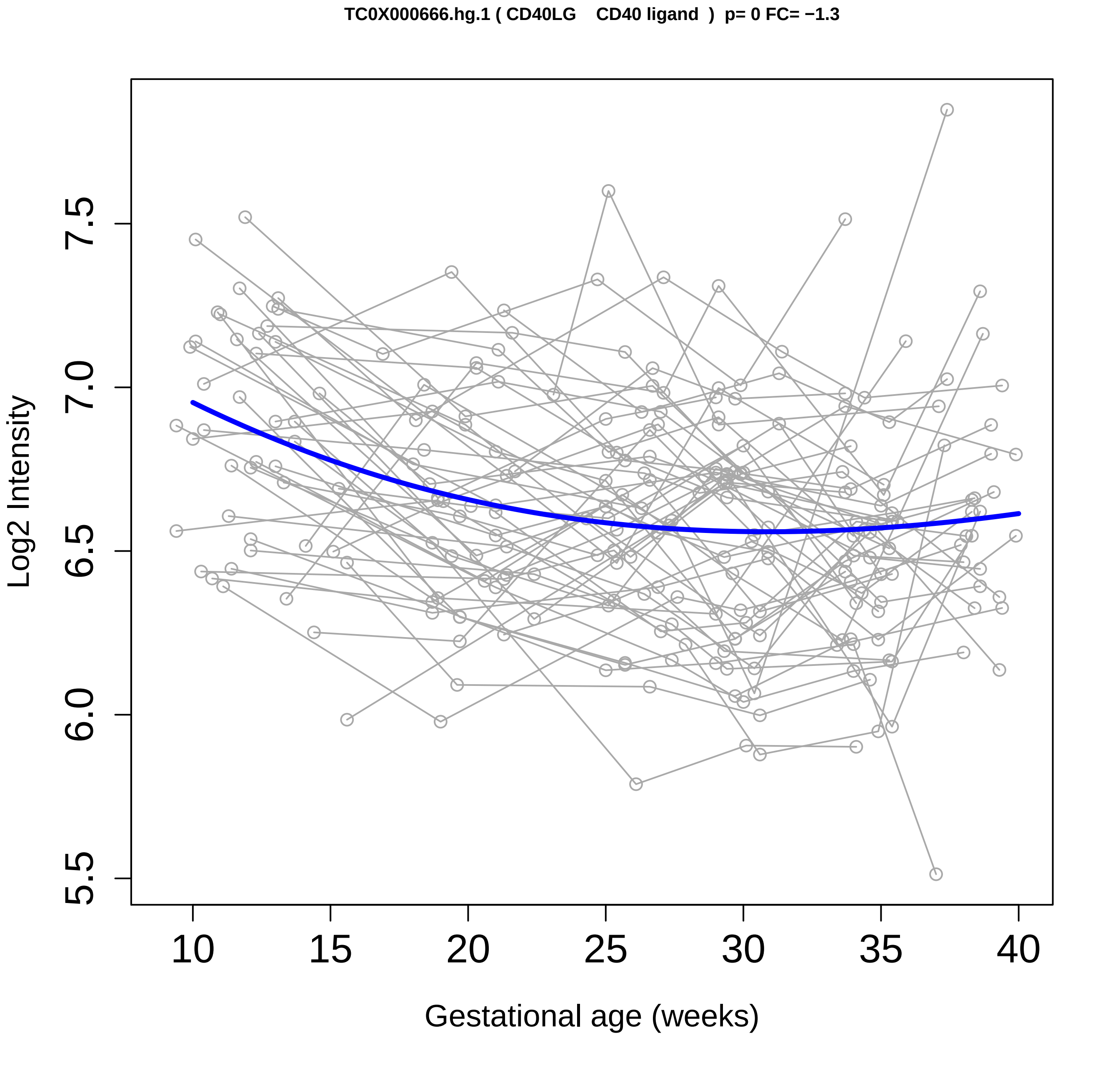
TC0X000666.hg.1 (CD40LG CD40 ligand) p= 0 FC= −1.3
Log2 Intensity
5.5
6.0
6.5
7.0
7.5
10
15
20
25
30
35
40
Gestational age (weeks)

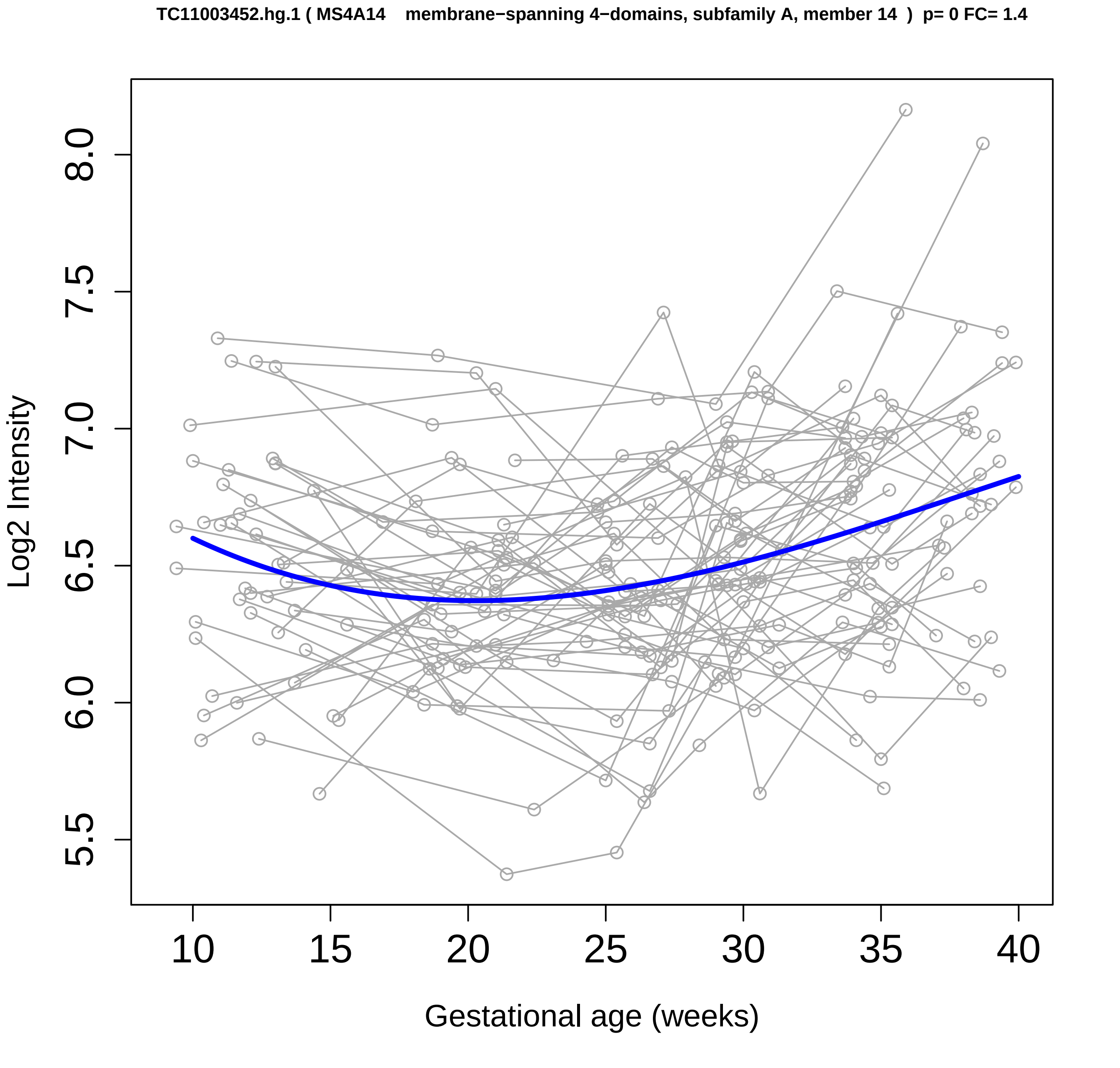
TC11003452.hg.1 (MS4A14   membrane−spanning 4−domains, subfamily A, member 14) p= 0 FC= 1.4
Log2 Intensity
5.5
6.0
6.5
7.0
7.5
8.0
10
15
20
25
30
35
40
Gestational age (weeks)

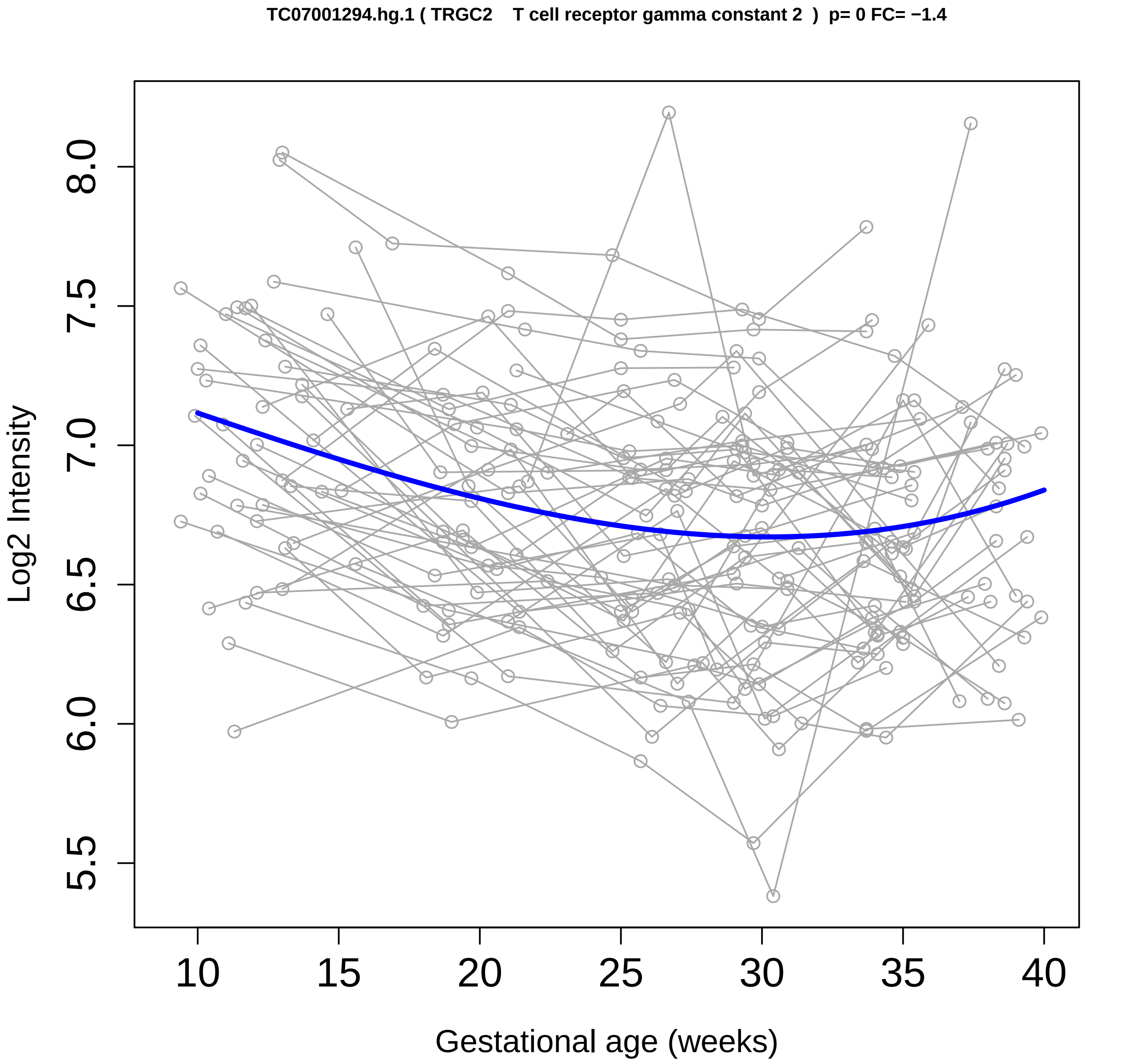
TC07001294.hg.1 (TRGC2 T cell receptor gamma constant 2) p= 0 FC= −1.4
Log2 Intensity
5.5
6.0
6.5
7.0
7.5
8.0
10
15
20
25
30
35
40
Gestational age (weeks)

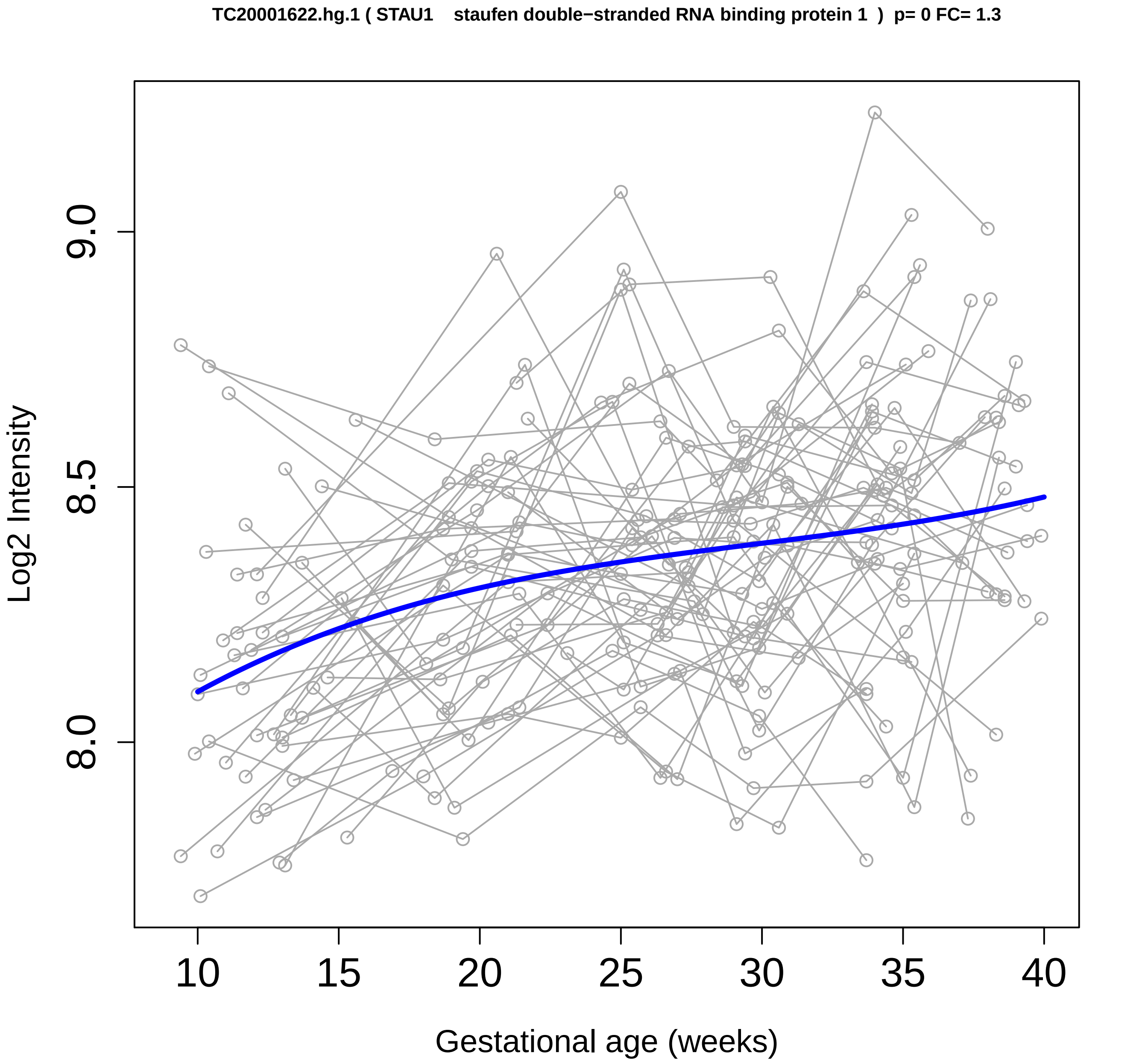
TC20001622.hg.1 (STAU1   staufen double−stranded RNA binding protein 1) p= 0 FC= 1.3
Log2 Intensity
9.0
8.5
8.0
10
15
20
25
30
35
40
Gestational age (weeks)

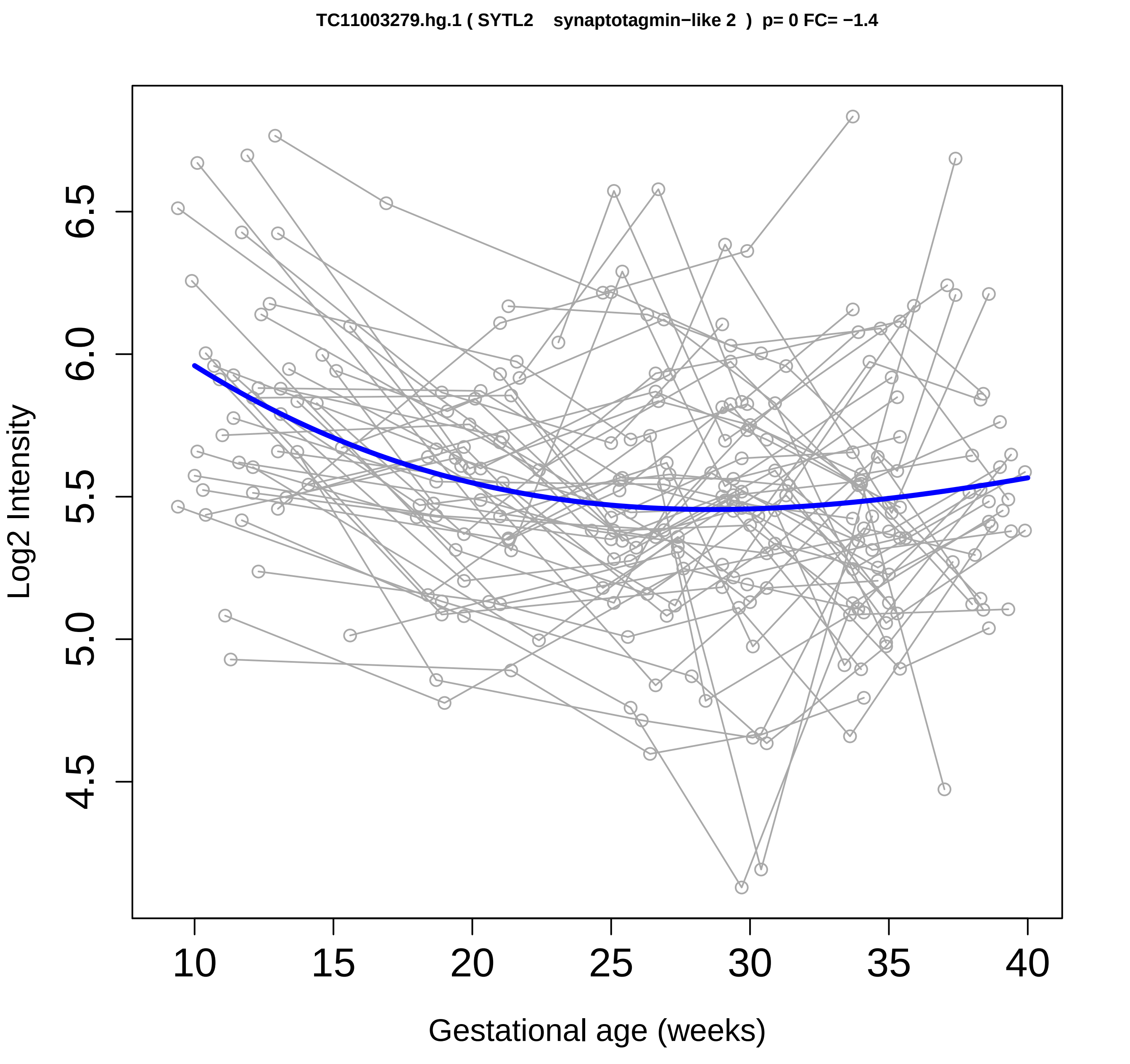
TC11003279.hg.1 (SYTL2 synaptotagmin−like 2) p= 0 FC= −1.4
Log2 Intensity
4.5
5.0
5.5
6.0
6.5
10
15
20
25
30
35
40
Gestational age (weeks)

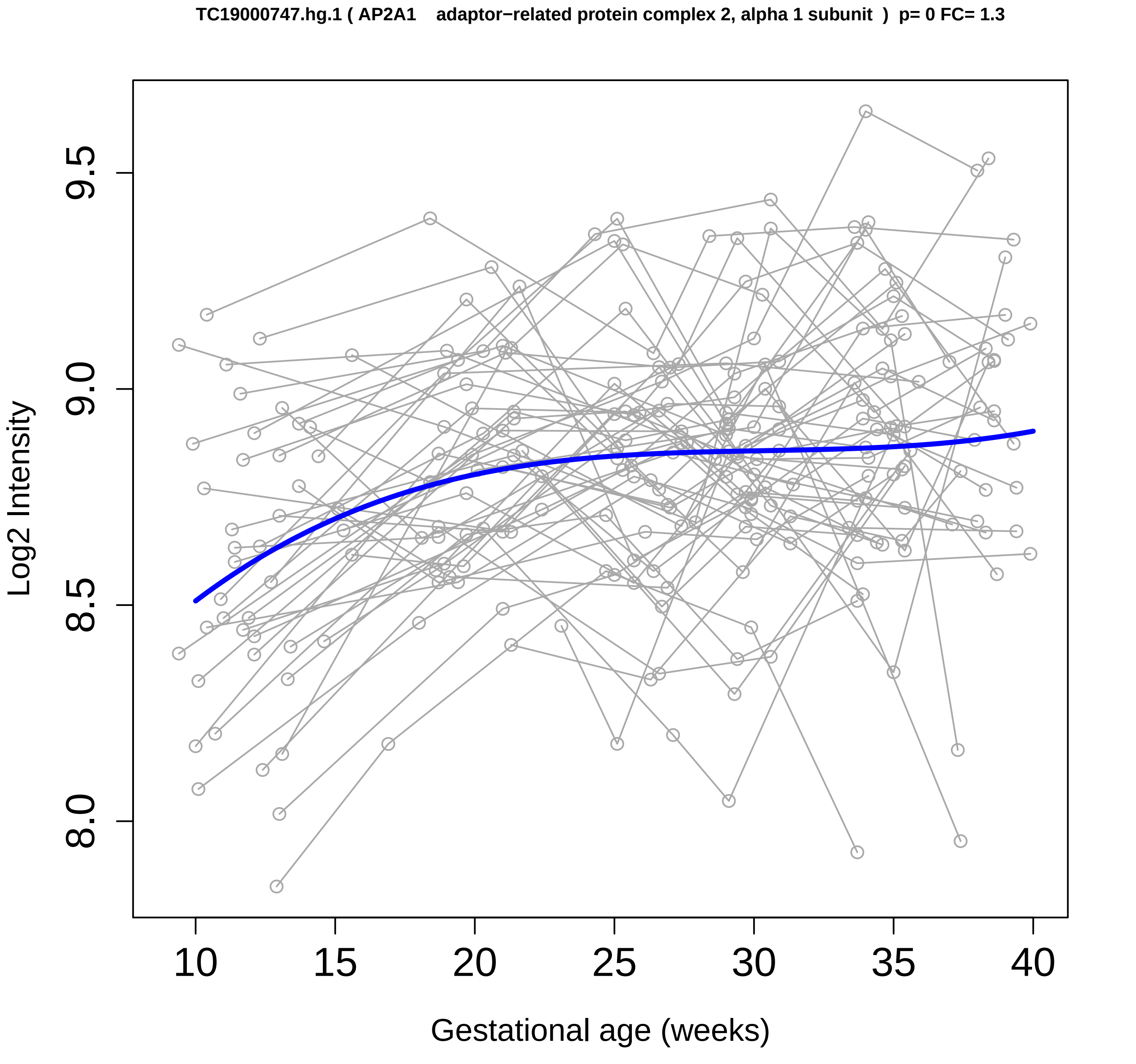
TC19000747.hg.1 (AP2A1 adaptor−related protein complex 2, alpha 1 subunit) p= 0 FC= 1.3
Log2 Intensity
8.0
8.5
9.0
9.5
10
15
20
25
30
35
40
Gestational age (weeks)

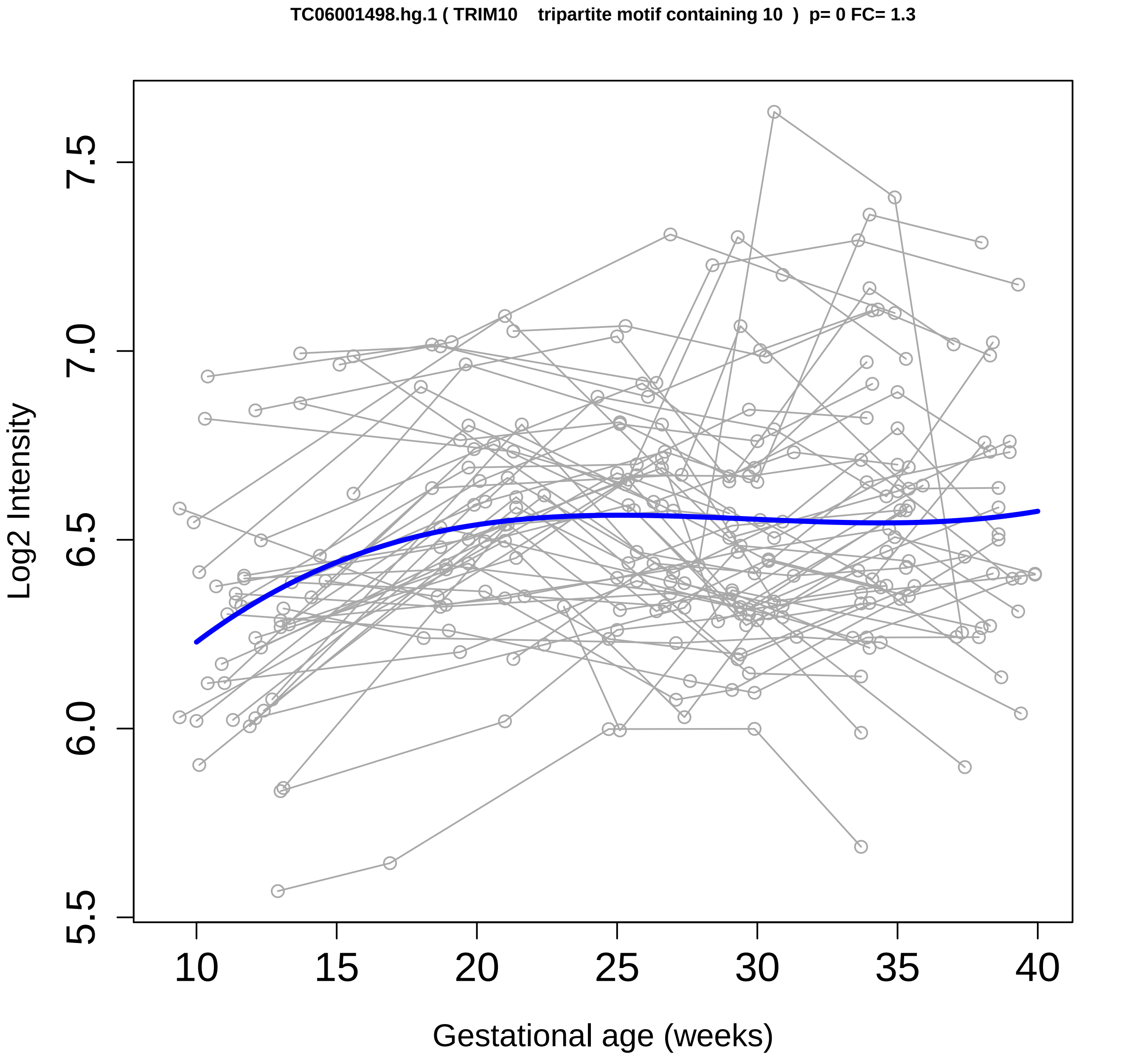

TC06001498.hg.1 (TRIM10 tripartite motif containing 10) p= 0 FC= 1.3
Log2 Intensity
5.5
6.0
6.5
7.0
7.5
10
15
20
25
30
35
40
Gestational age (weeks)

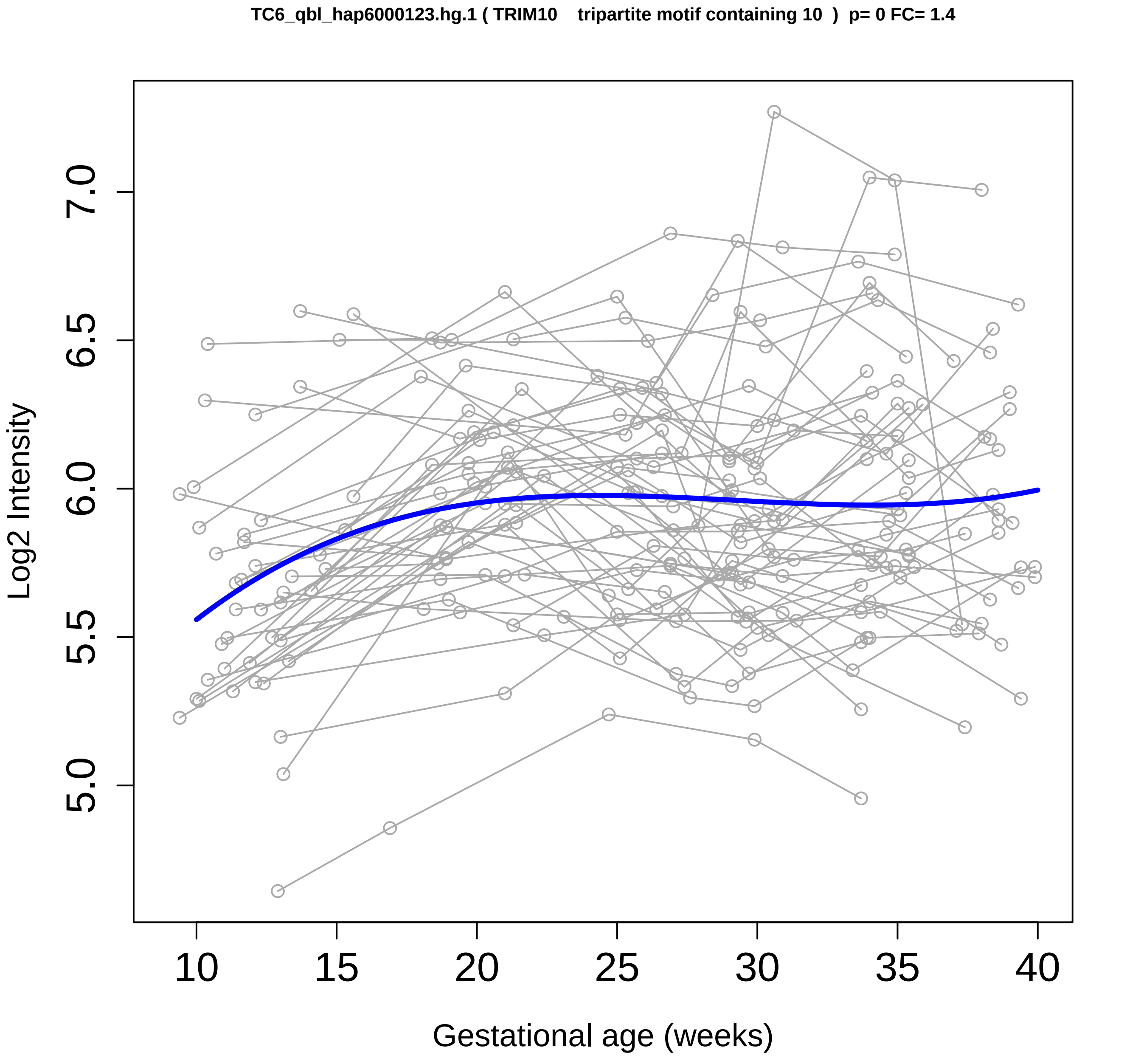
TC6_qbl_hap6000123.hg.1 (TRIM10 tripartite motif containing 10) p= 0 FC= 1.4
Log2 Intensity
5.0
5.5
6.0
6.5
7.0
10
15
20
25
30
35
40
Gestational age (weeks)

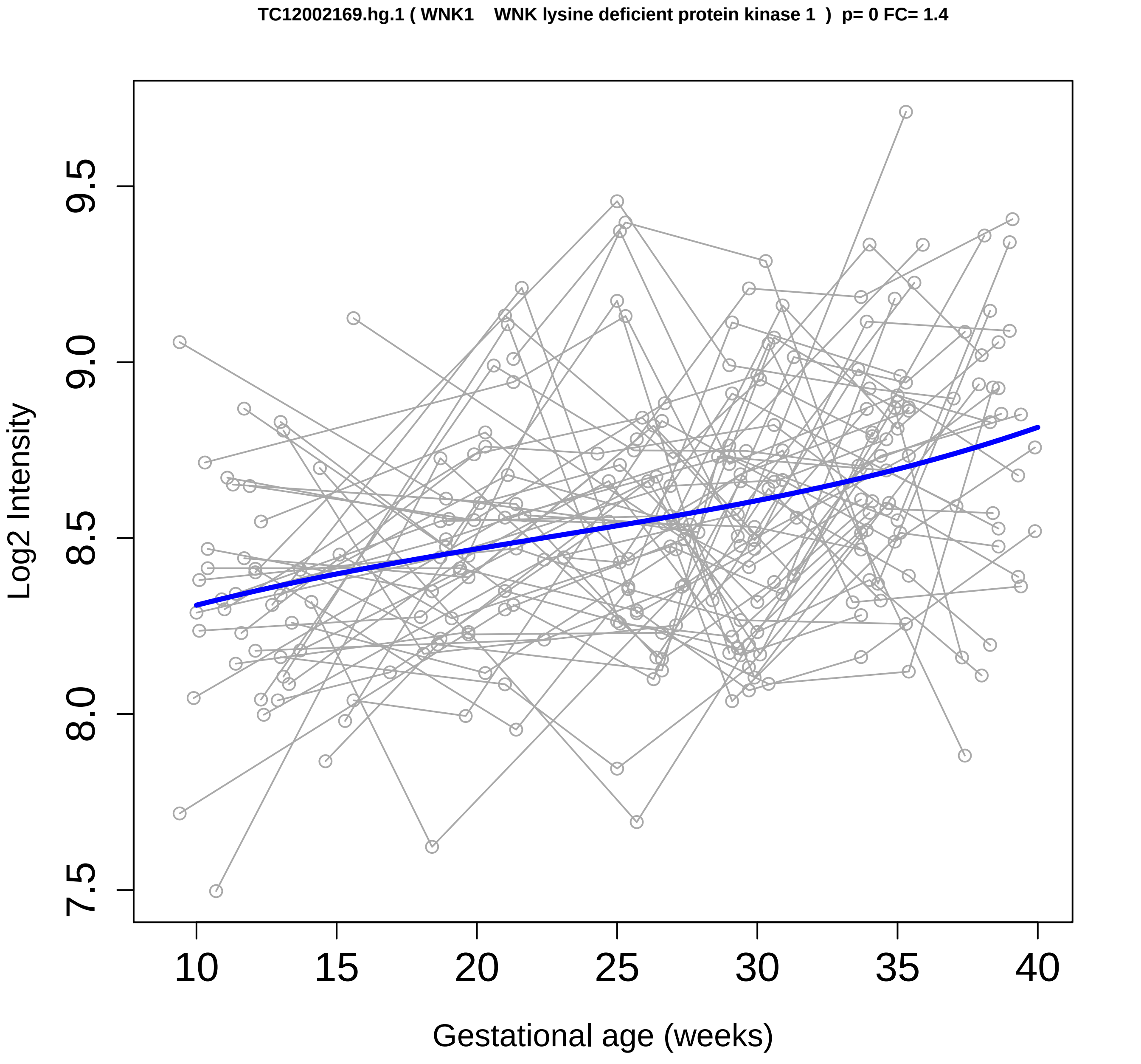

TC12002169.hg.1 (WNK1   WNK lysine deficient protein kinase 1) p= 0 FC= 1.4
Log2 Intensity
9.5
9.0
8.5
8.0
7.5
10
15
20
25
30
35
40
Gestational age (weeks)

TC04001974.hg.1 (DTHD1 death domain containing 1) p= 0 FC= −1.3

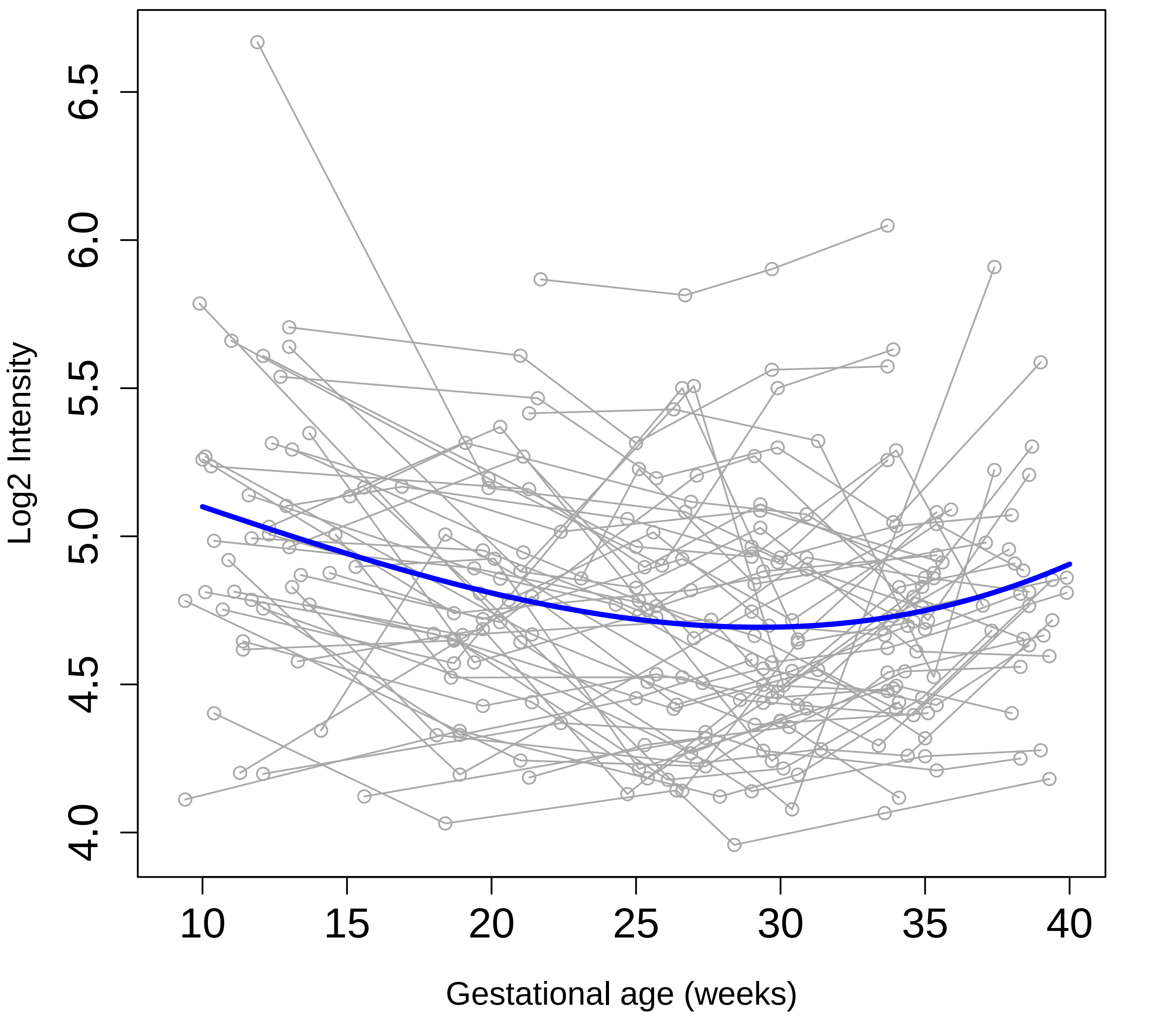

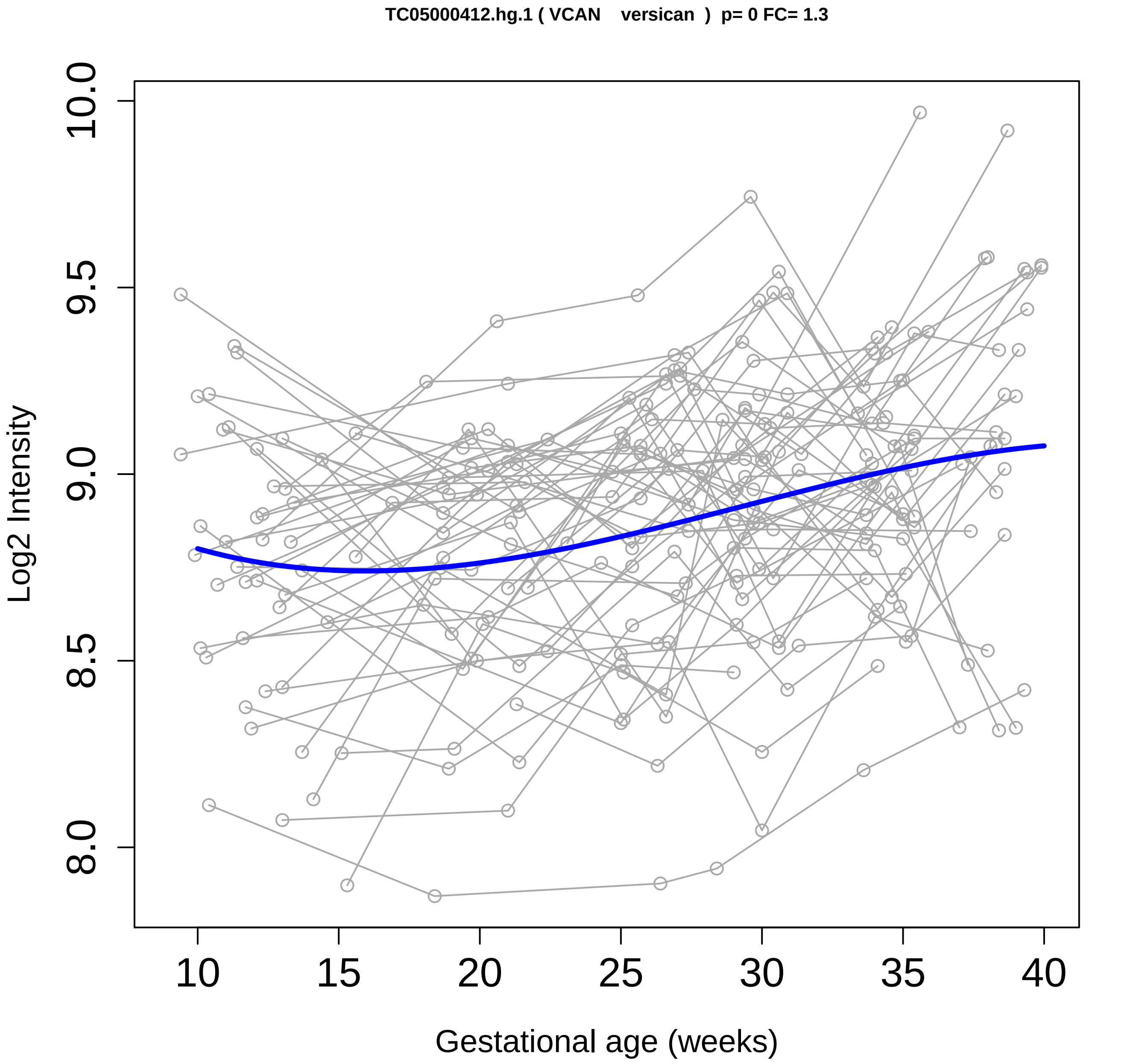
TC05000412.hg.1 (VCAN versican) p= 0 FC= 1.3
Log2 Intensity
10.0
9.5
9.0
8.5
8.0
10
15
20
25
30
35
40
Gestational age (weeks)

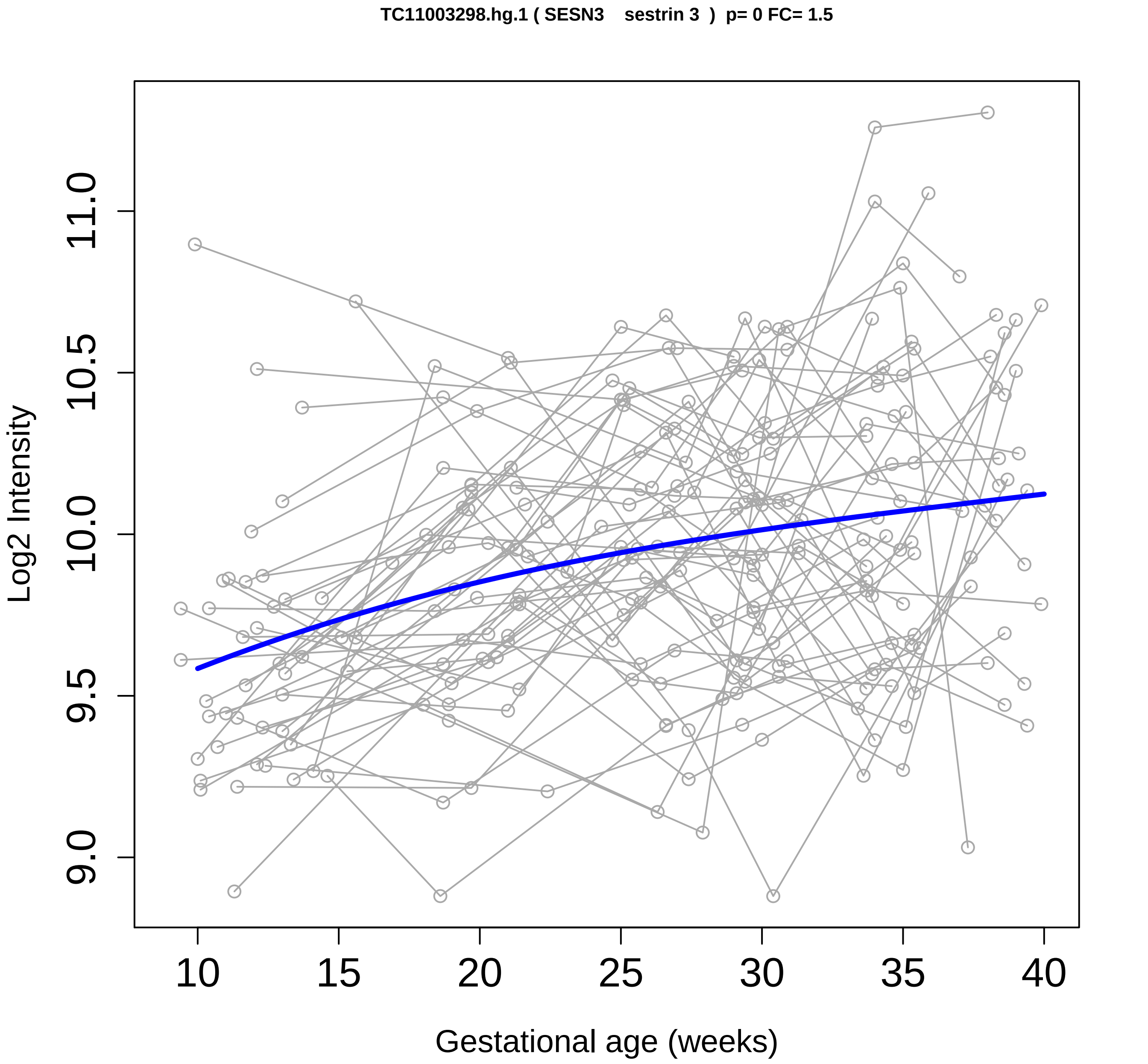

TC11003298.hg.1 (SESN3 sestrin 3) p= 0 FC= 1.5
Log2 Intensity
9.0
9.5
10.0
10.5
11.0
10
15
20
25
30
35
40
Gestational age (weeks)

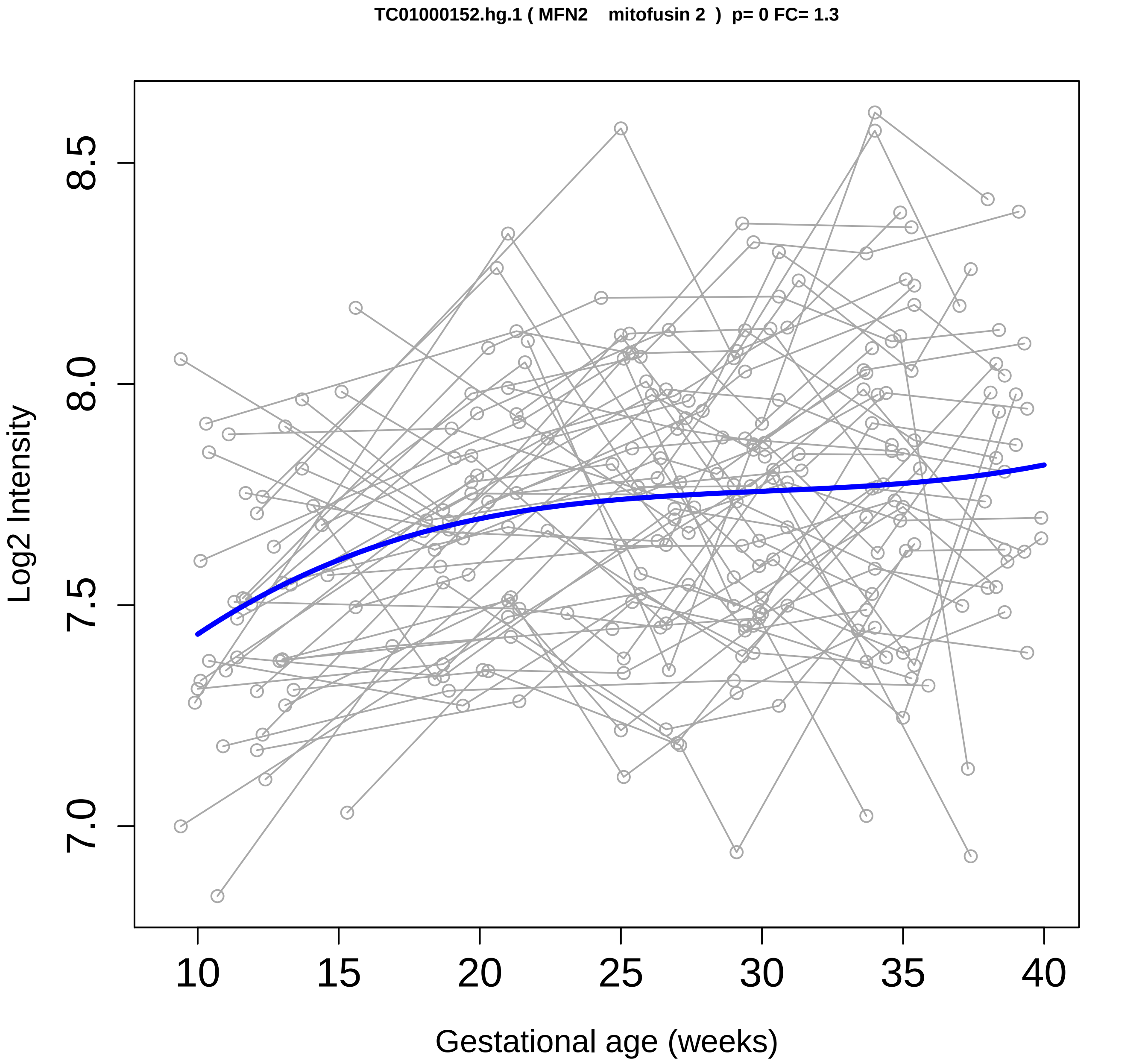

TC01000152.hg.1 (MFN2 mitofusin 2) p= 0 FC= 1.3
Log2 Intensity
7.0
7.5
8.0
8.5
10
15
20
25
30
35
40
Gestational age (weeks)

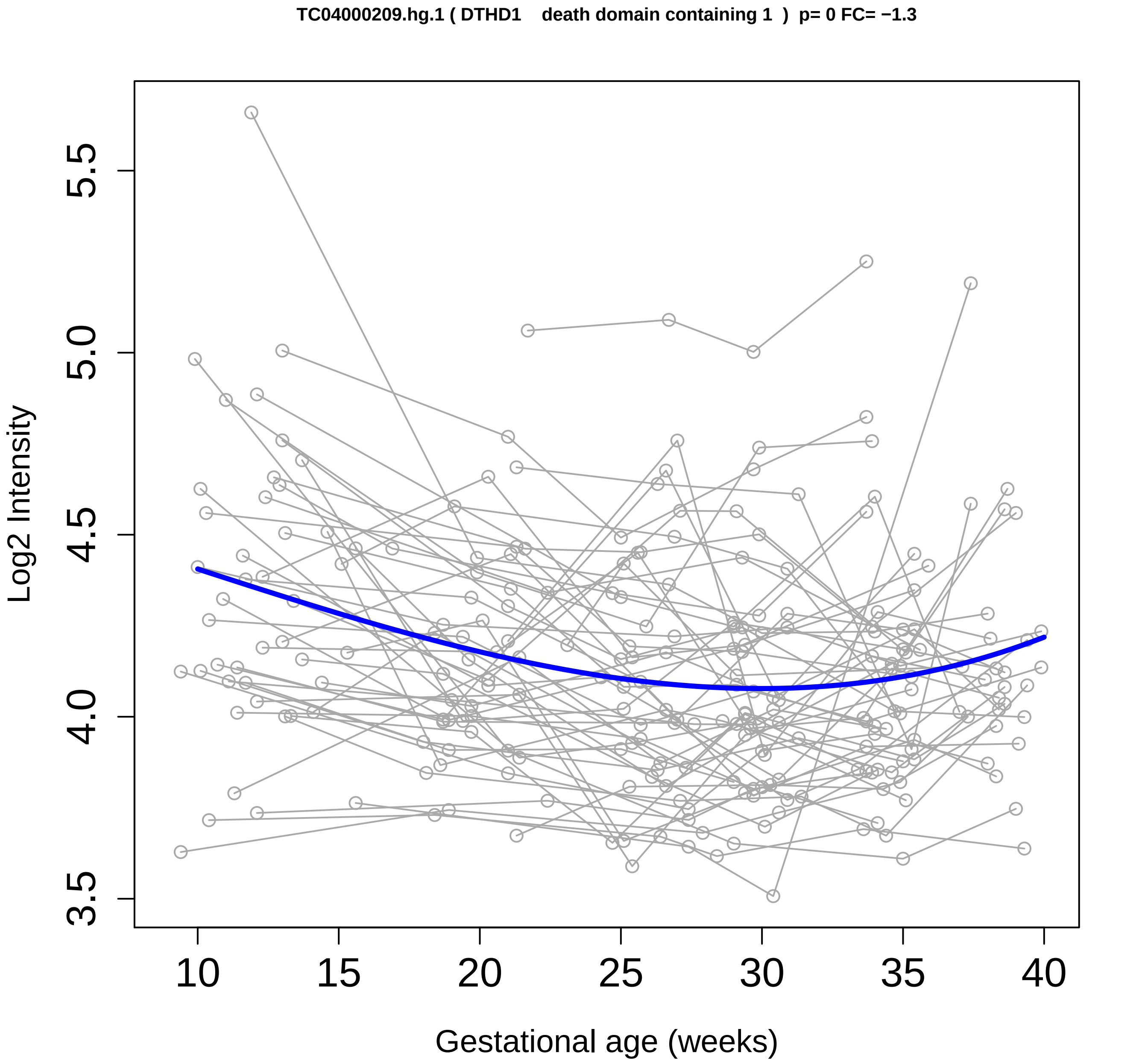
TC04000209.hg.1 (DTHD1 death domain containing 1) p= 0 FC= −1.3
Log2 Intensity
5.5
5.0
4.5
4.0
3.5
10
15
20
25
30
35
40
Gestational age (weeks)

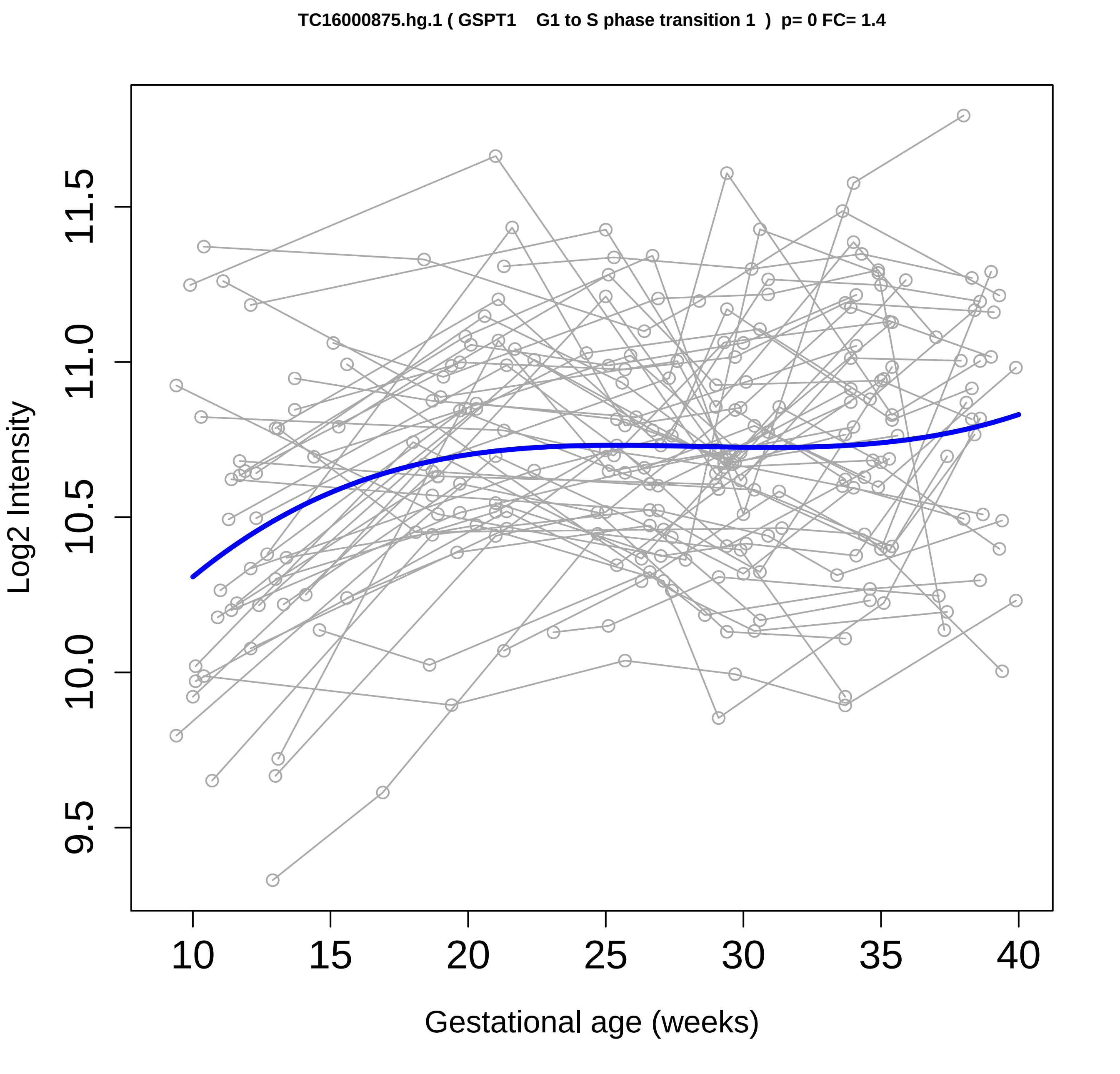

TC16000875.hg.1 (GSPT1 G1 to S phase transition 1) p= 0 FC= 1.4
Log2 Intensity
9.5
10.0
10.5
11.0
11.5
10
15
20
25
30
35
40
Gestational age (weeks)

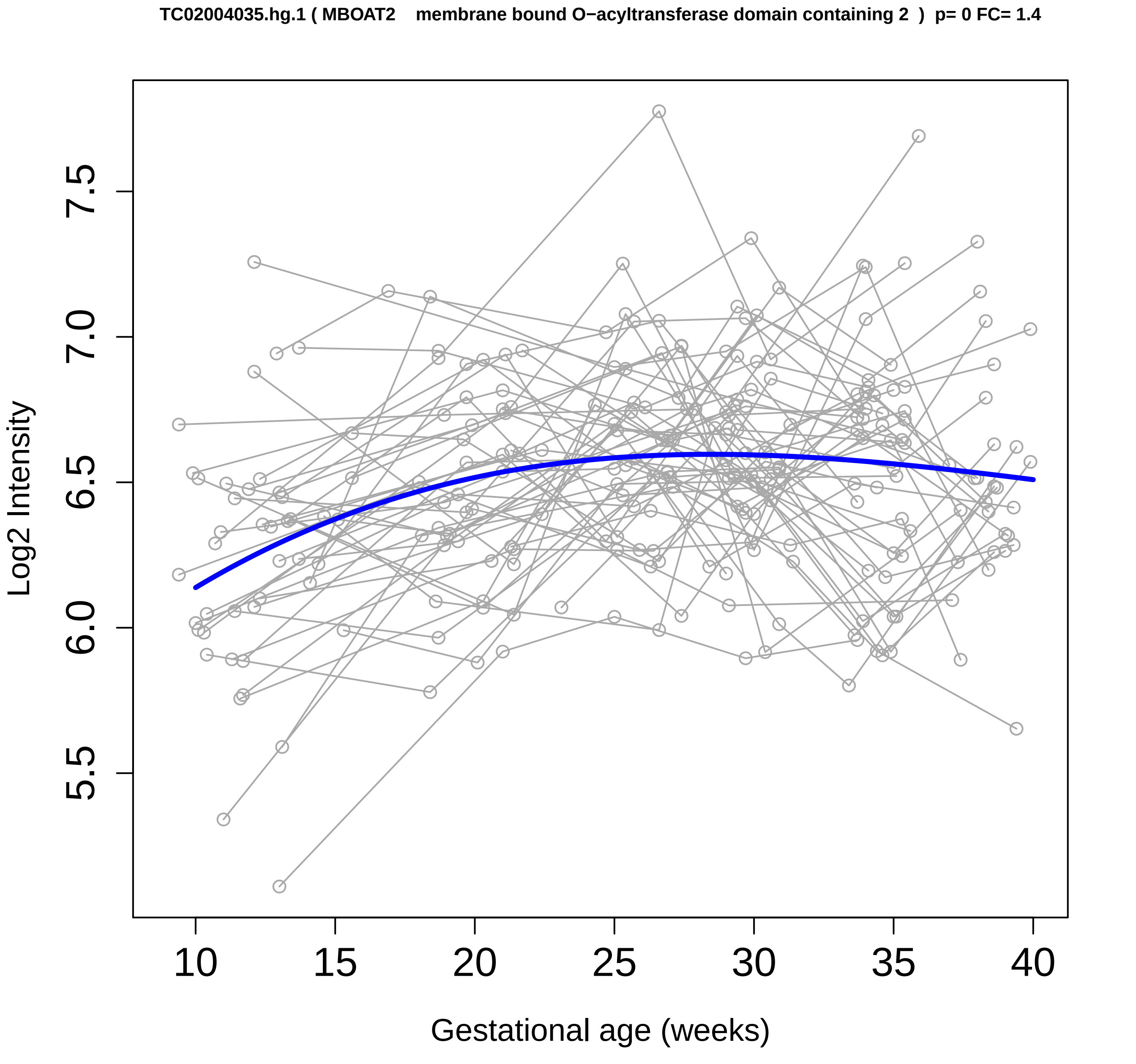
TC02004035.hg.1 (MBOAT2 membrane bound O−acyltransferase domain containing 2) p= 0 FC= 1.4
Log2 Intensity
7.5
7.0
6.5
6.0
5.5
10
15
20
25
30
35
40
Gestational age (weeks)

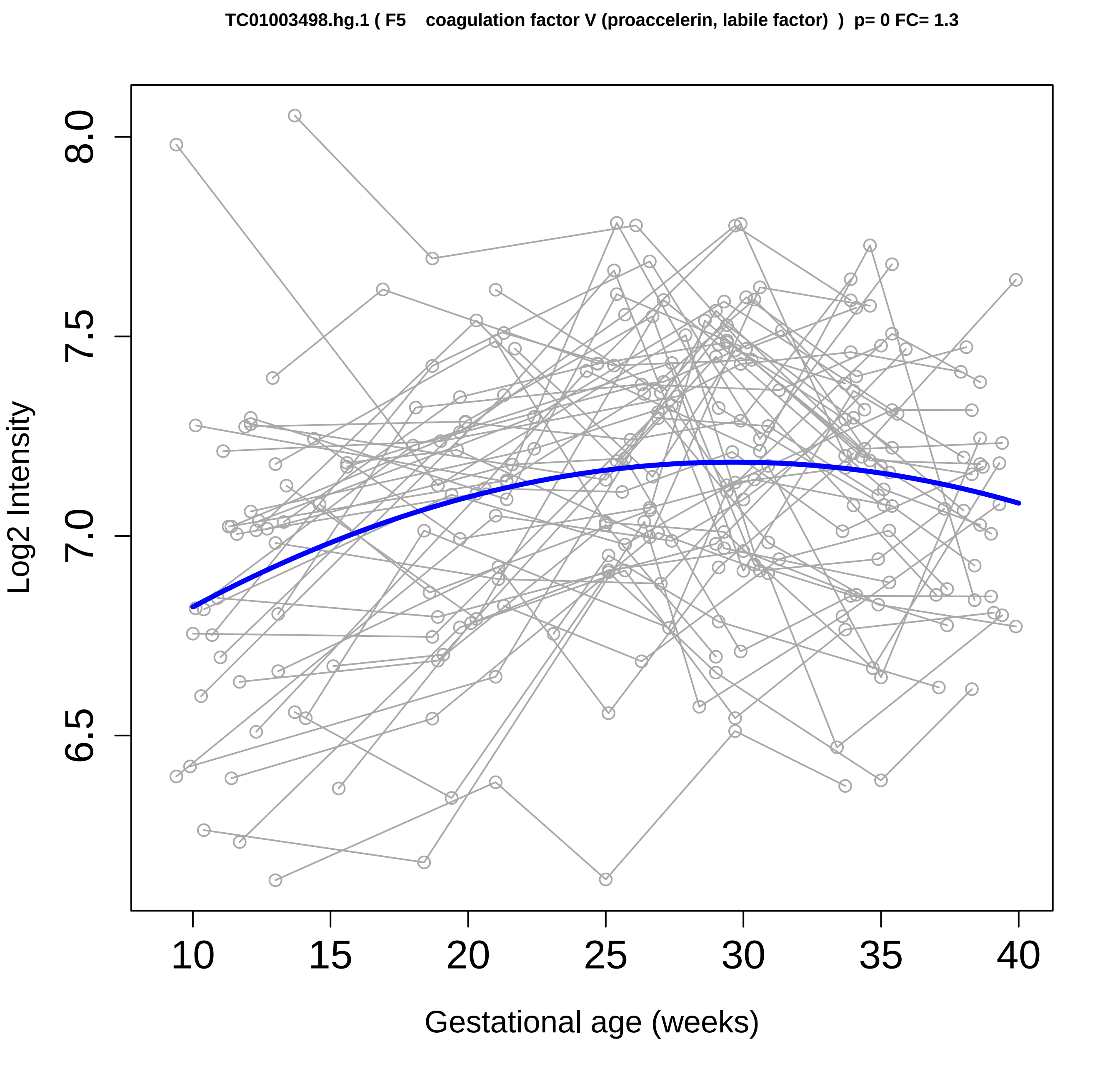

TC01003498.hg.1 (F5   coagulation factor V (proaccelerin, labile factor)) p= 0 FC= 1.3
Log2 Intensity
8.0
7.5
7.0
6.5
10
15
20
25
30
35
40
Gestational age (weeks)

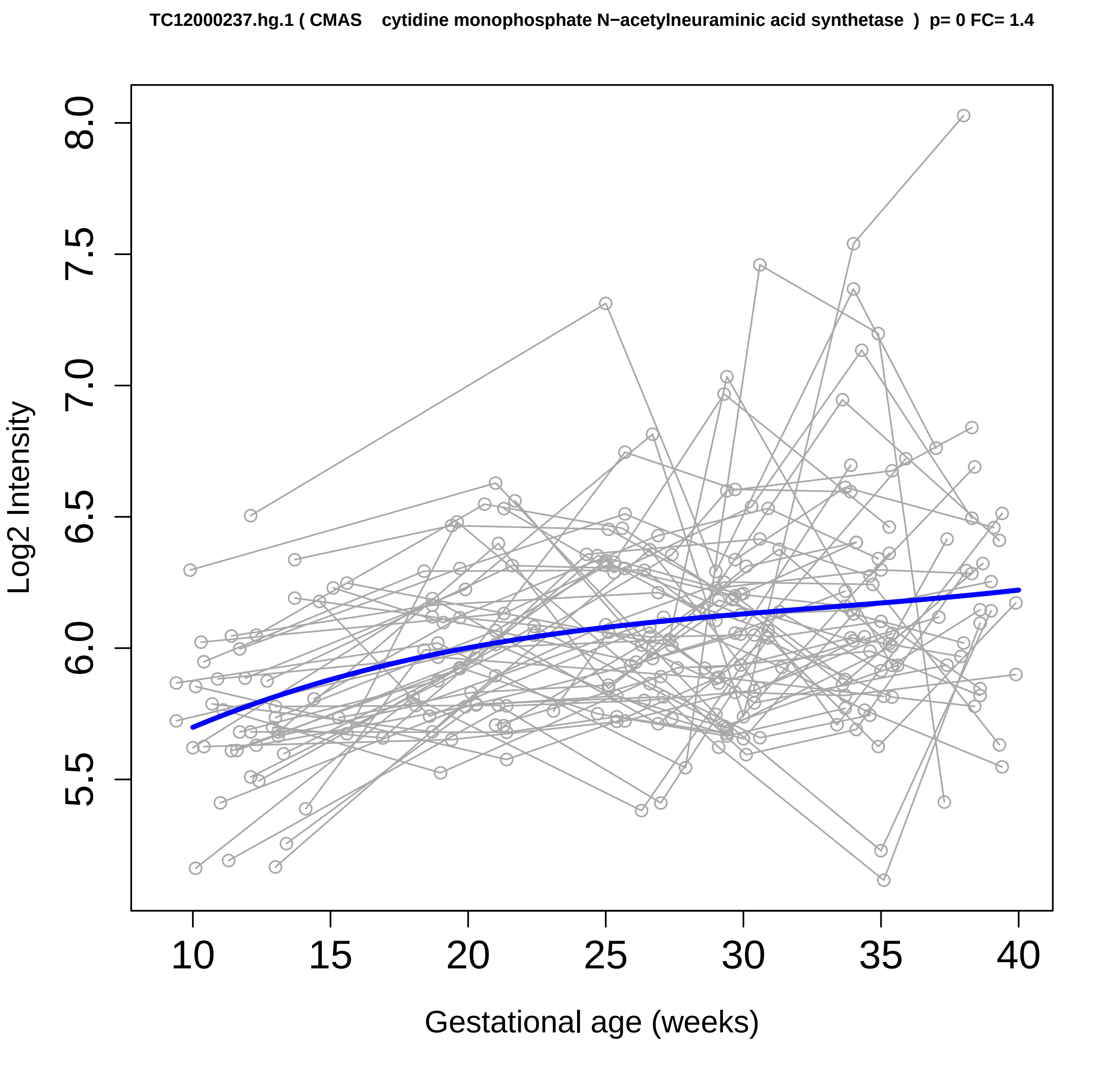
TC12000237.hg.1 (CMAS   cytidine monophosphate N−acetylneuraminic acid synthetase) p= 0 FC= 1.4
Log2 Intensity
5.5
6.0
6.5
7.0
7.5
8.0
10
15
20
25
30
35
40
Gestational age (weeks)

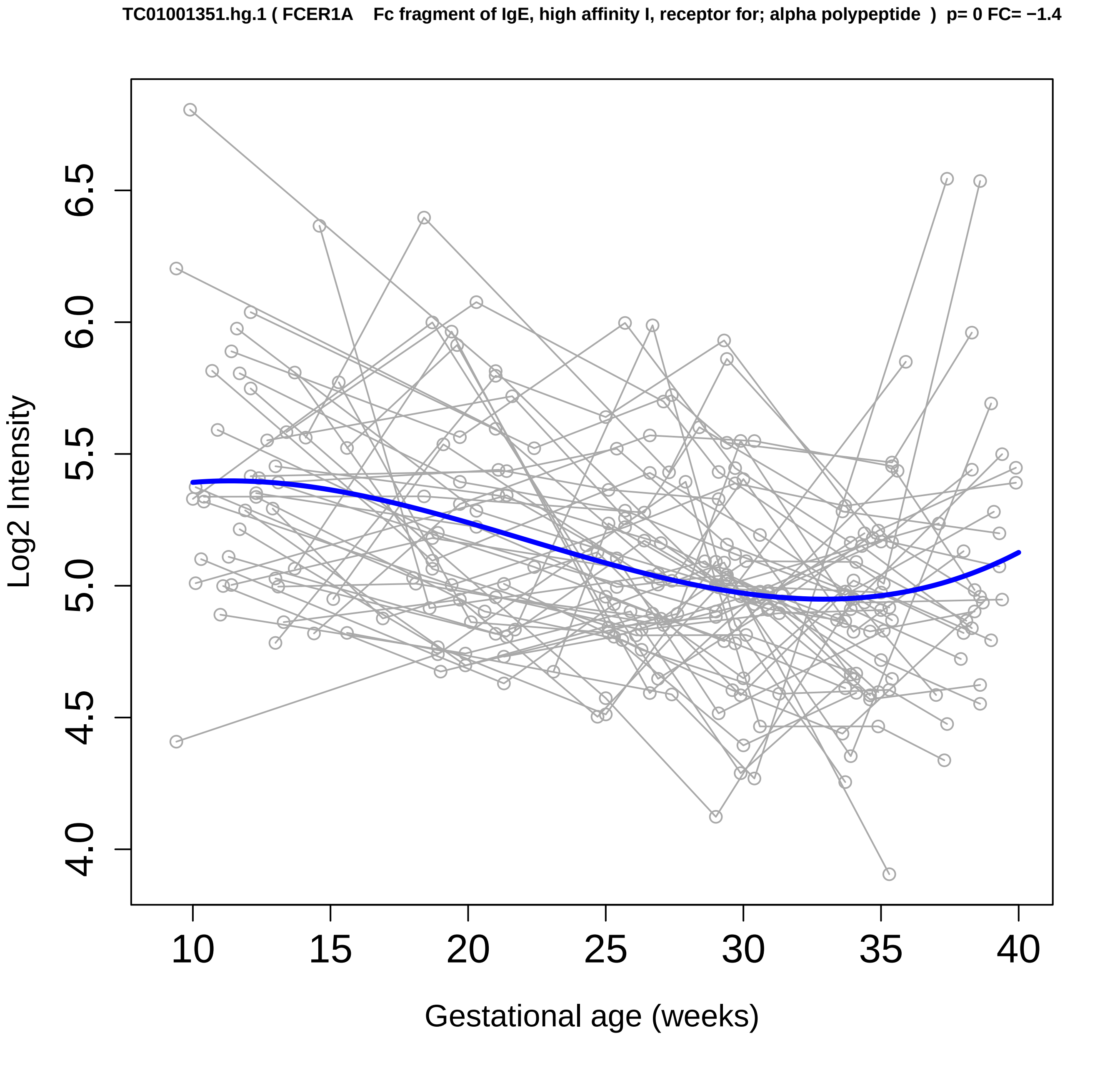

TC01001351.hg.1 (FCER1A Fc fragment of IgE, high affinity I, receptor for; alpha polypeptide) p= 0 FC= −1.4
Log2 Intensity
4.0
4.5
5.0
5.5
6.0
6.5
10
15
20
25
30
35
40
Gestational age (weeks)

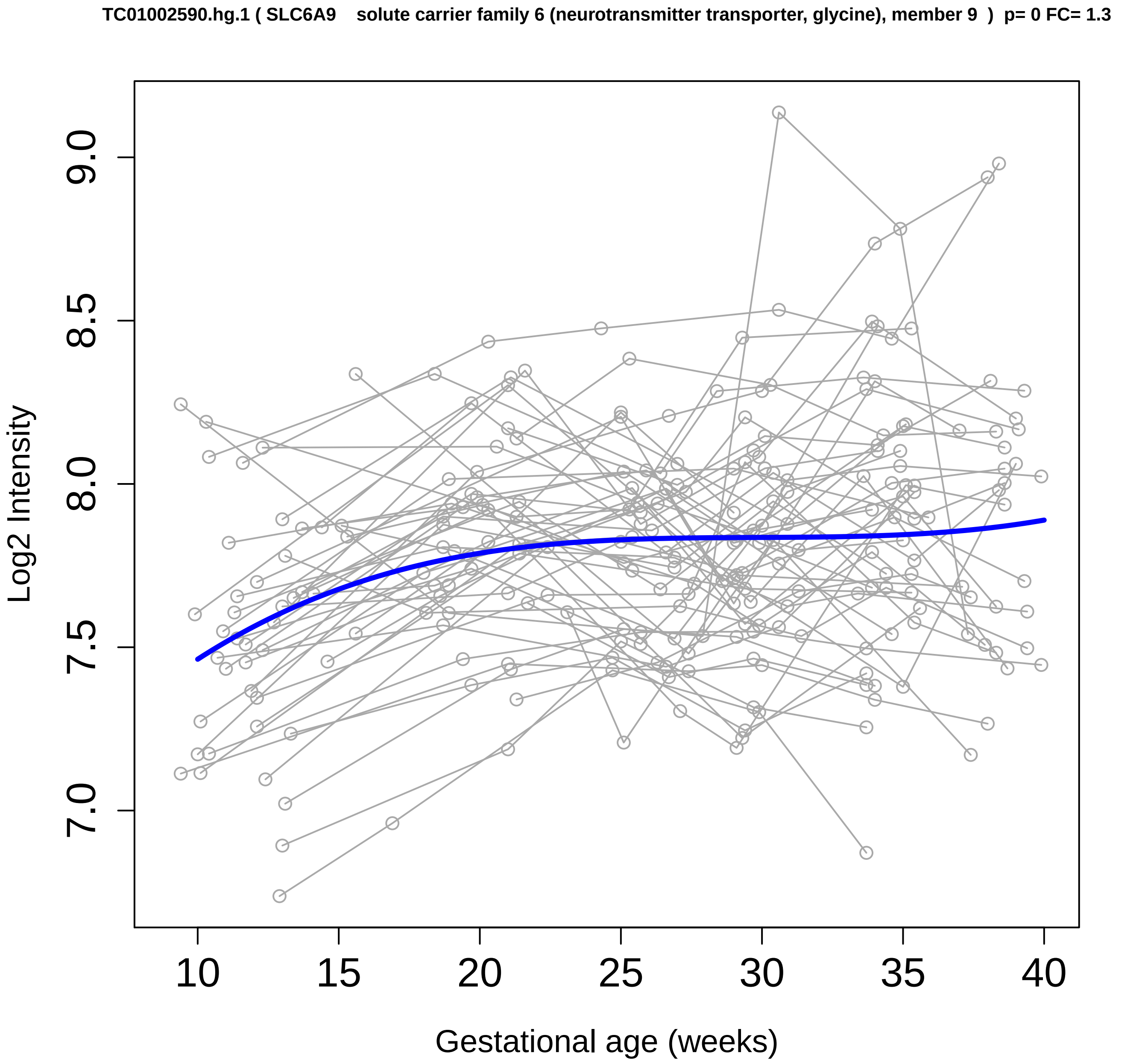
TC01002590.hg.1 (SLC6A9 solute carrier family 6 (neurotransmitter transporter, glycine), member 9) p= 0 FC= 1.3
Log2 Intensity
9.0
8.5
8.0
7.5
7.0
10
15
20
25
30
35
40
Gestational age (weeks)

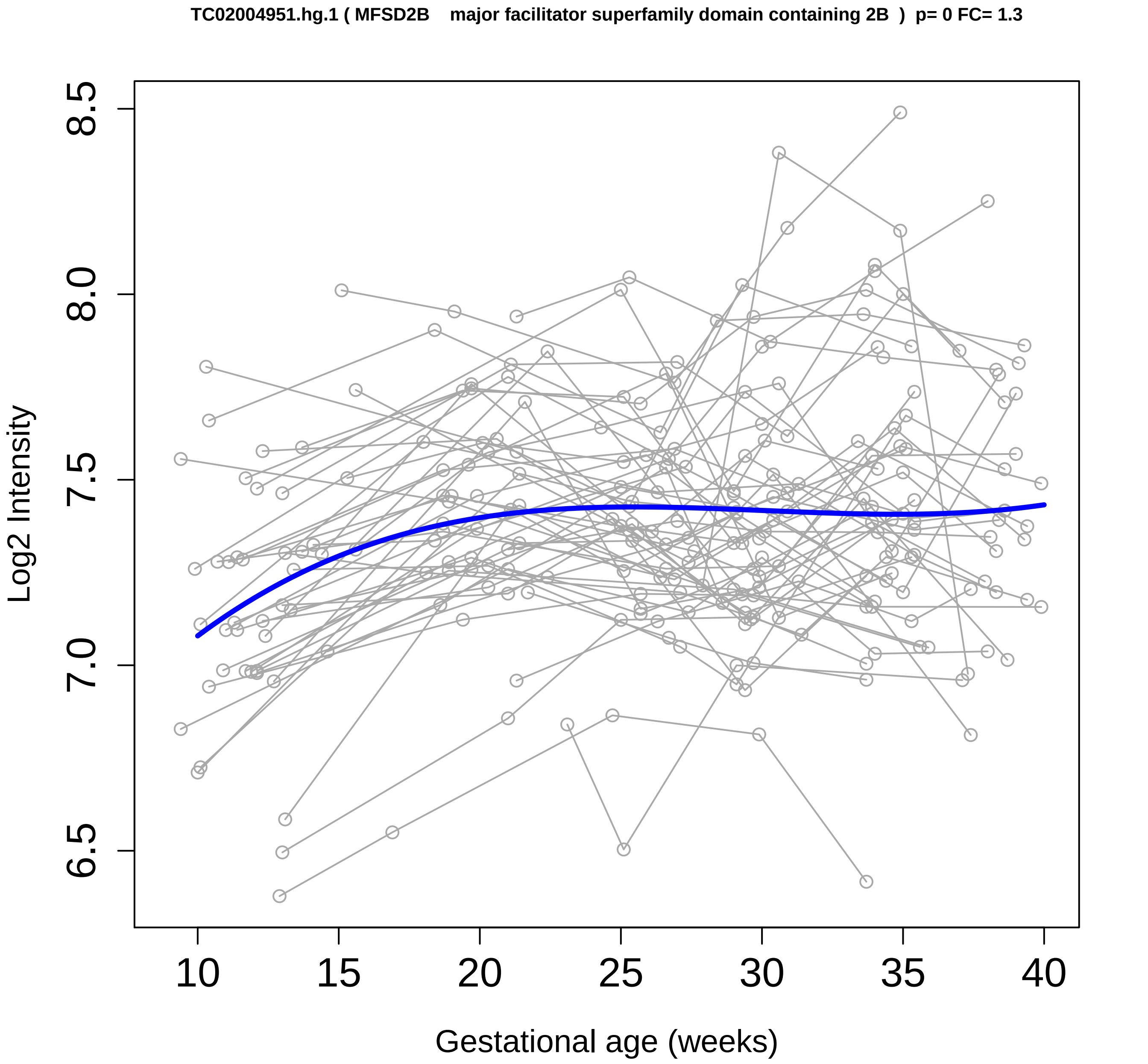

TC02004951.hg.1 (MFSD2B major facilitator superfamily domain containing 2B) p= 0 FC= 1.3
Log2 Intensity
6.5
7.0
7.5
8.0
8.5
10
15
20
25
30
35
40
Gestational age (weeks)

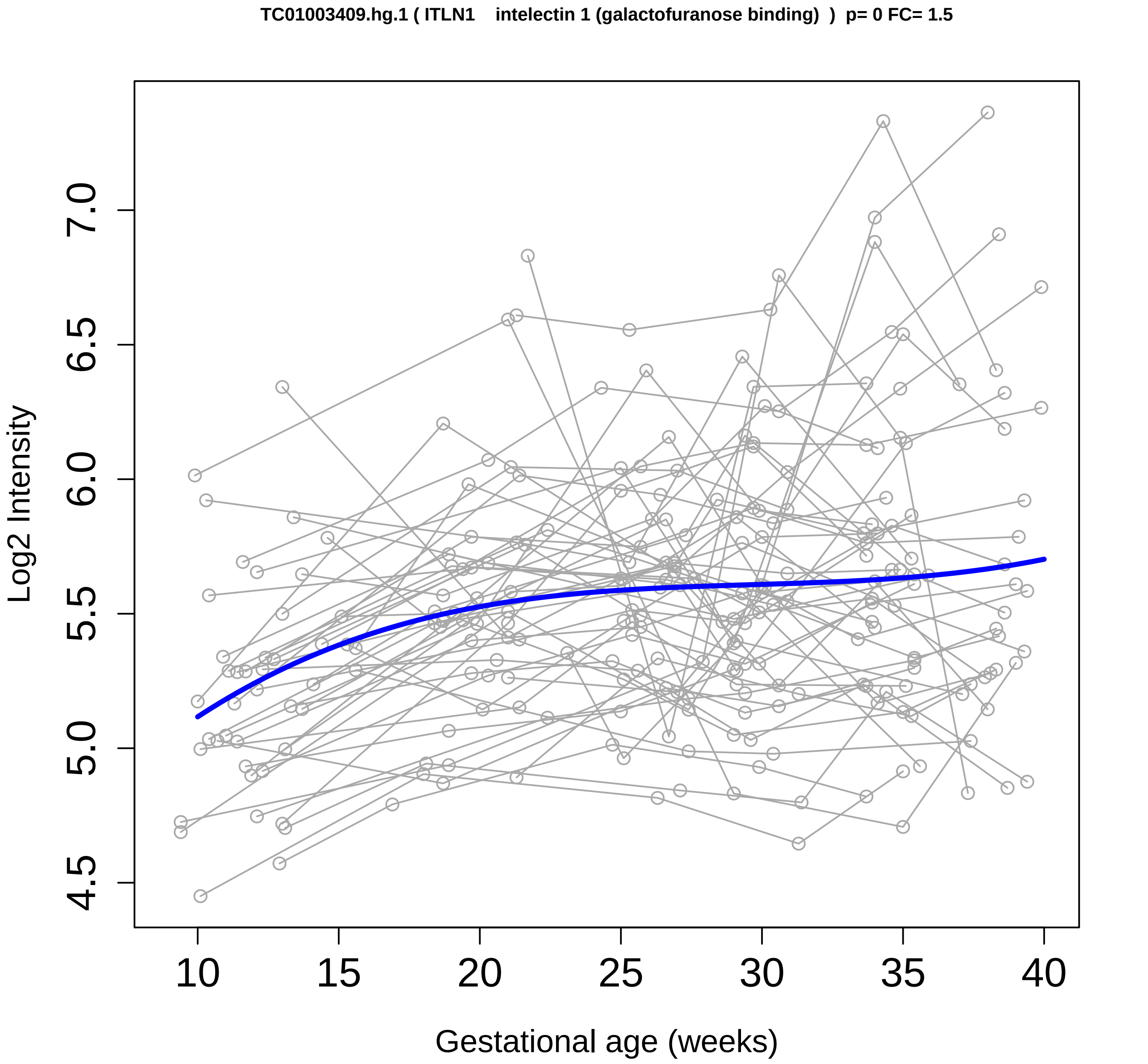
TC01003409.hg.1 (ITLN1 intelectin 1 (galactofuranose binding)) p= 0 FC= 1.5
Log2 Intensity
4.5
5.0
5.5
6.0
6.5
7.0
10
15
20
25
30
35
40
Gestational age (weeks)

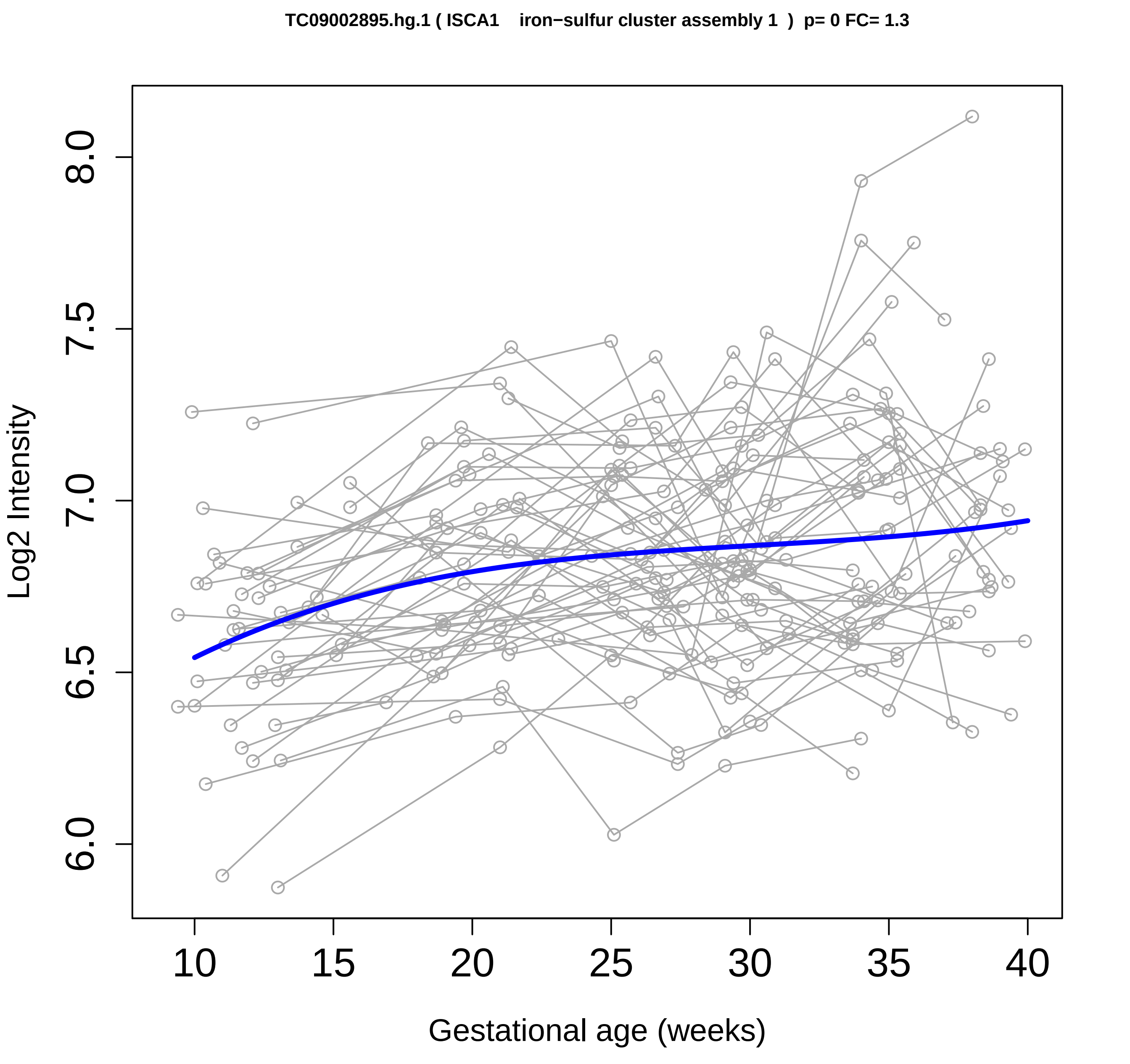

TC09002895.hg.1 (ISCA1 iron−sulfur cluster assembly 1) p= 0 FC= 1.3
Log2 Intensity
6.0
6.5
7.0
7.5
8.0
10
15
20
25
30
35
40
Gestational age (weeks)

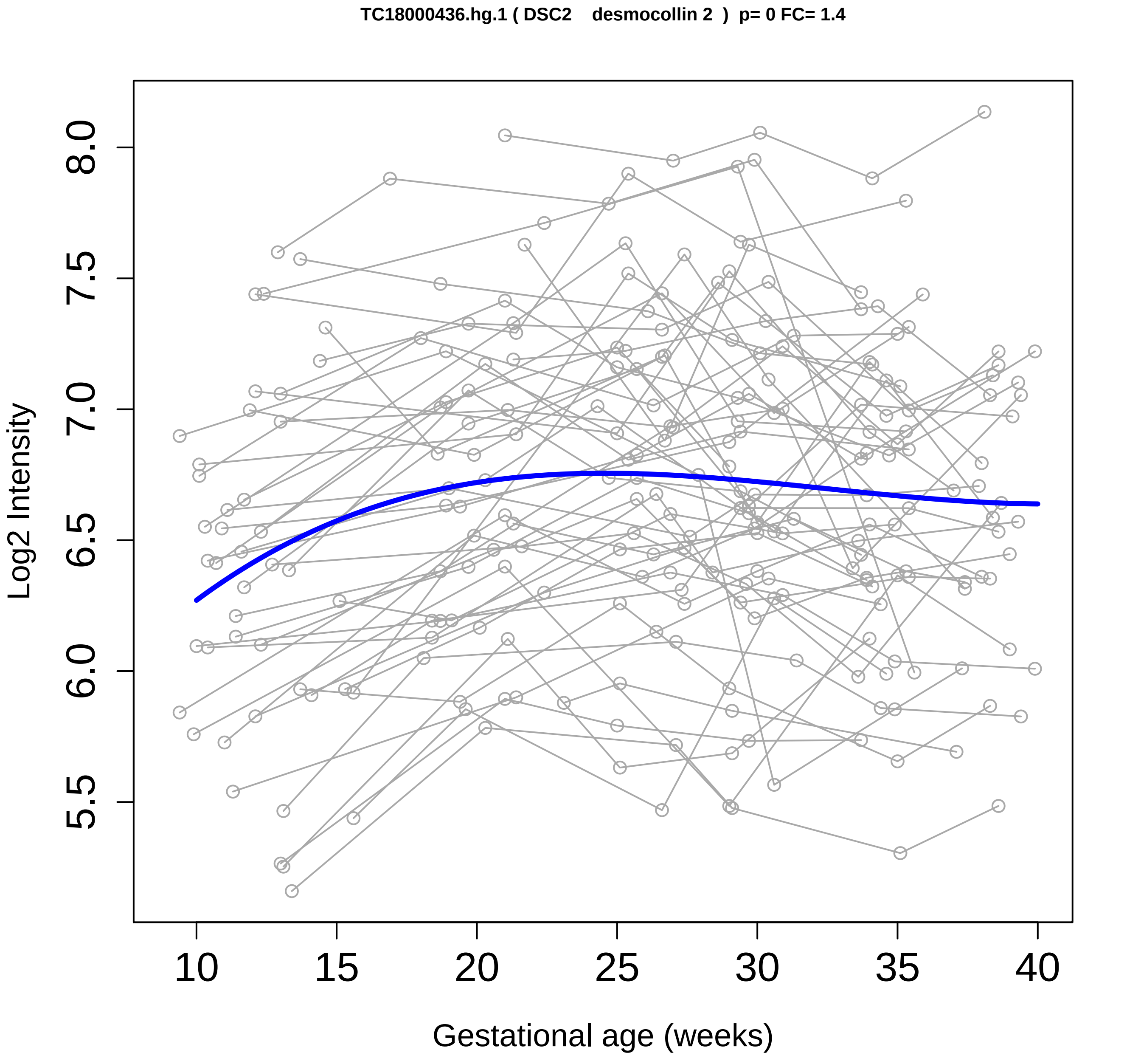
TC18000436.hg.1 (DSC2 desmocollin 2) p= 0 FC= 1.4
Log2 Intensity
8.0
7.5
7.0
6.5
6.0
5.5
10
15
20
25
30
35
40
Gestational age (weeks)

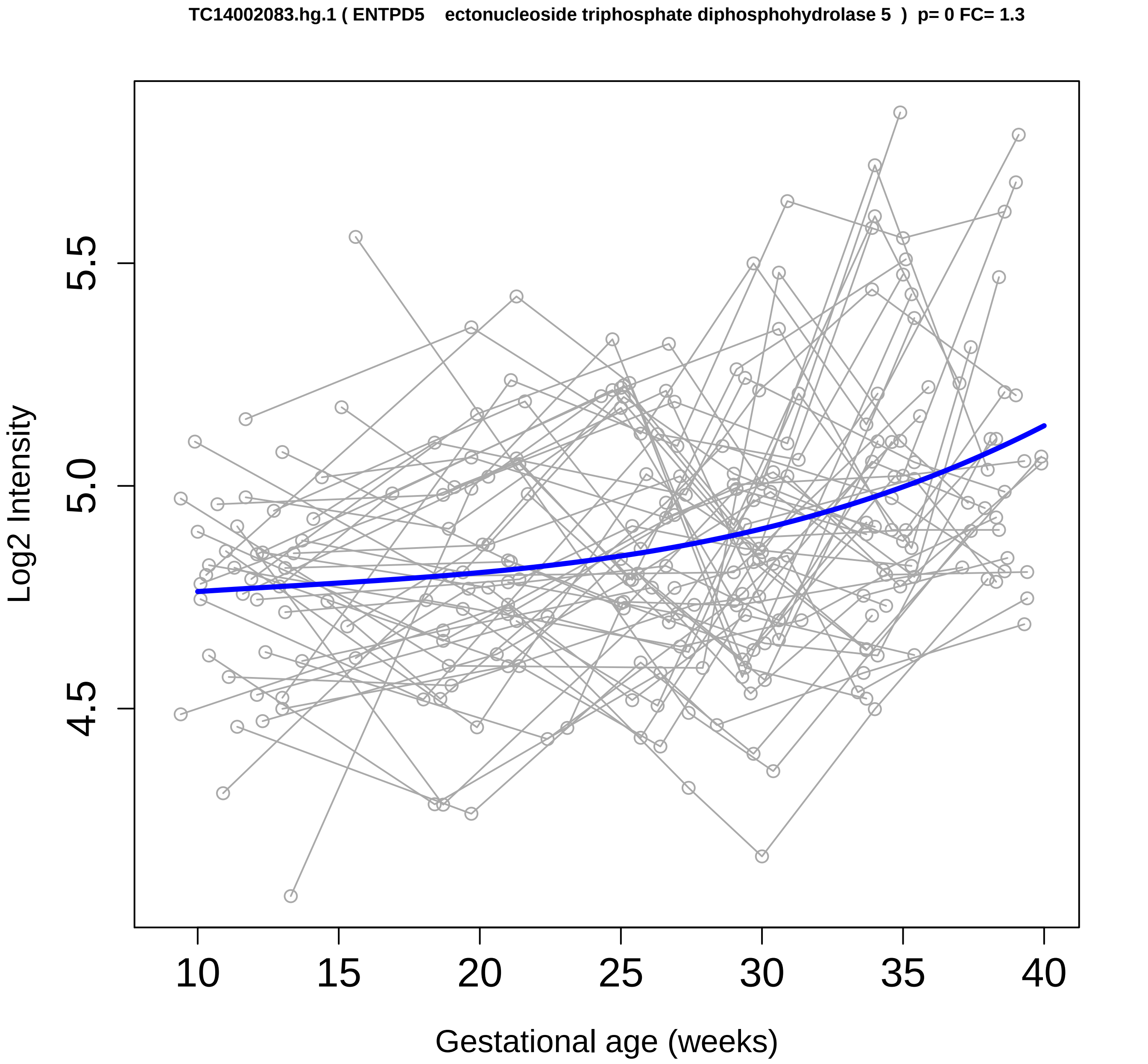
TC14002083.hg.1 (ENTPD5 ectonucleoside triphosphate diphosphohydrolase 5) p= 0 FC= 1.3
Log2 Intensity
5.5
5.0
4.5
10
15
20
25
30
35
40
Gestational age (weeks)

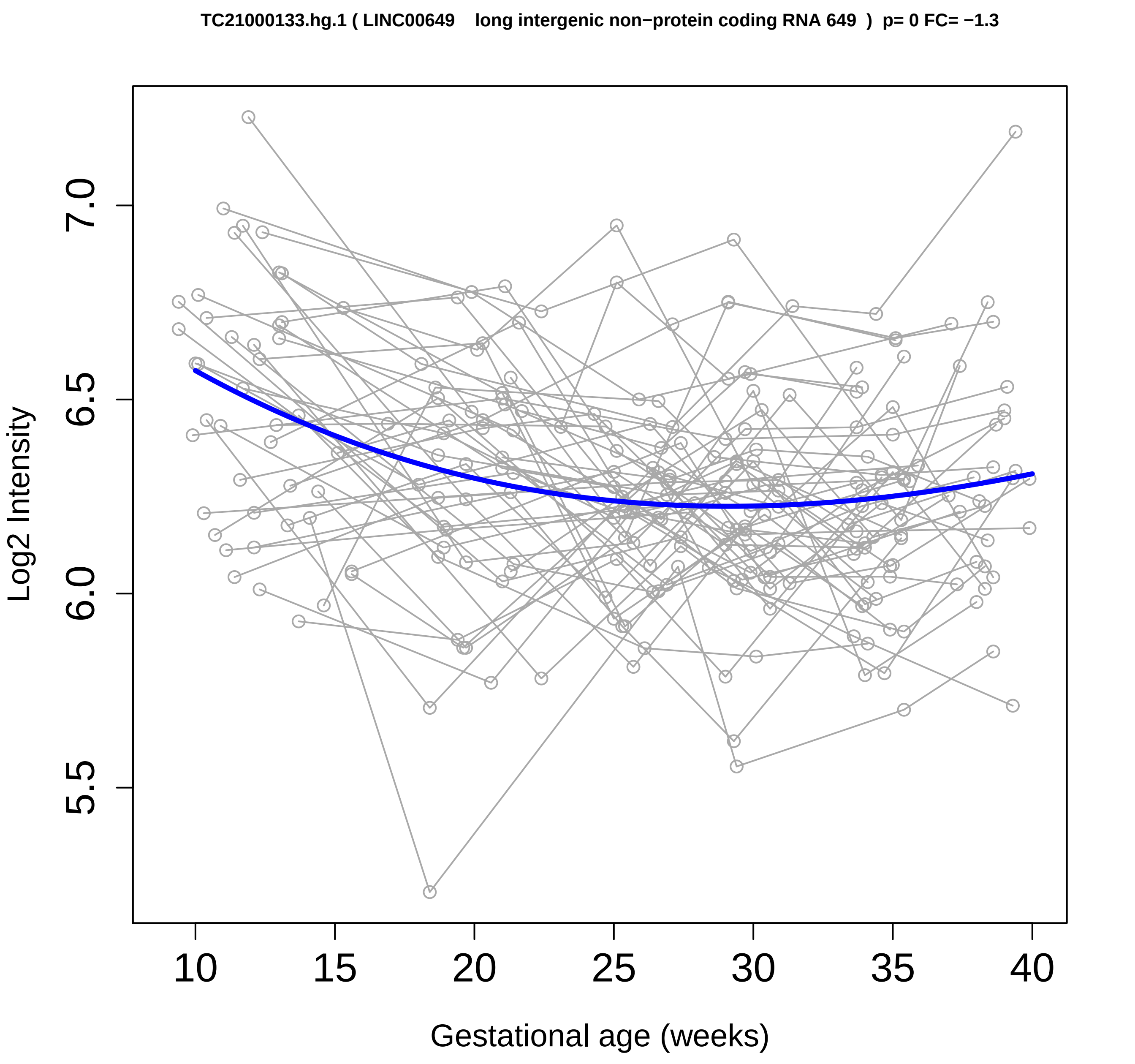

TC21000133.hg.1 (LINC00649 long intergenic non−protein coding RNA 649) p= 0 FC= −1.3
Log2 Intensity
7.0
6.5
6.0
5.5
10
15
20
25
30
35
40
Gestational age (weeks)

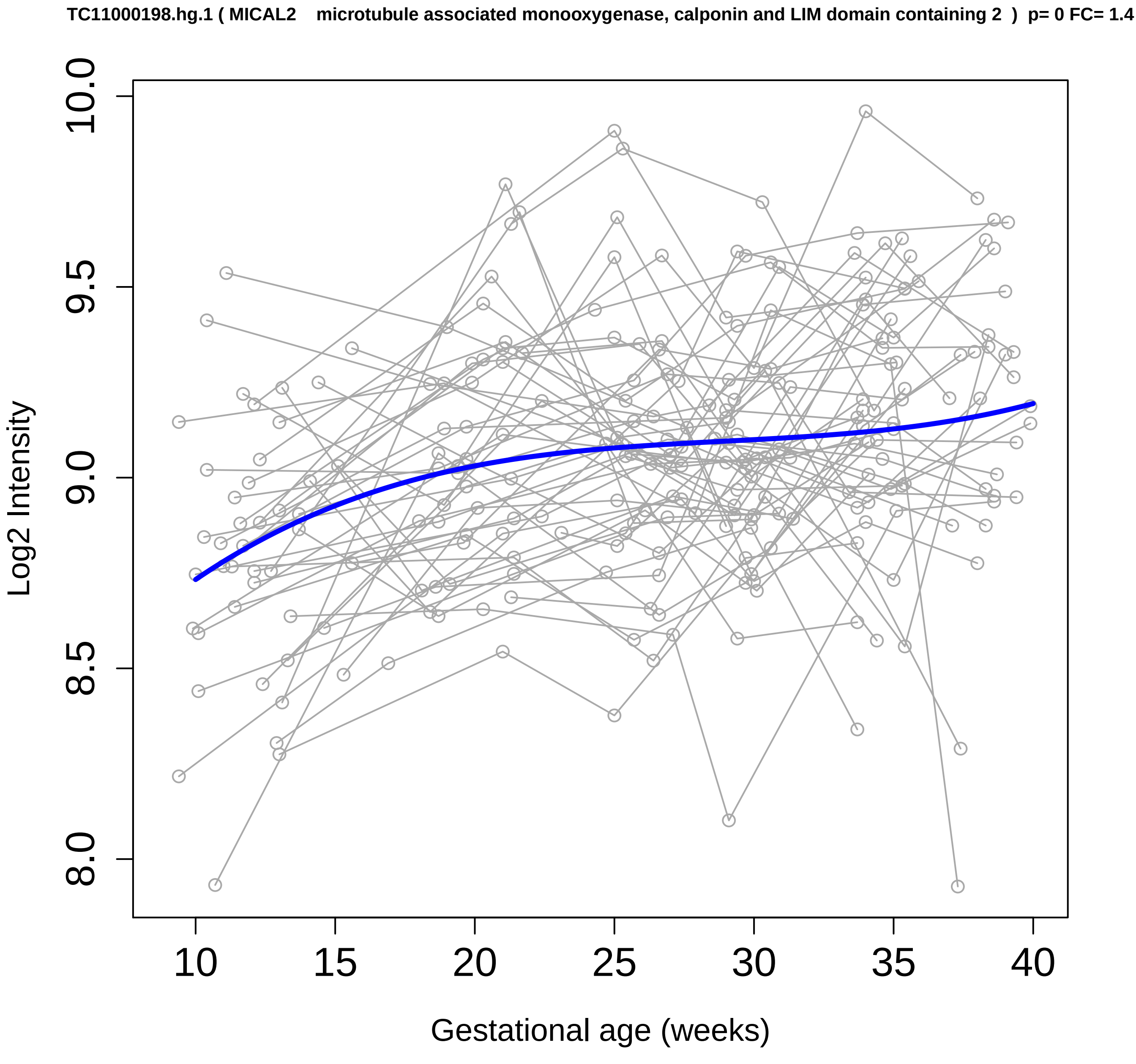

TC11000198.hg.1 (MICAL2 microtubule associated monooxygenase, calponin and LIM domain containing 2) p= 0 FC= 1.4
Log2 Intensity
8.0
8.5
9.0
9.5
10.0
10
15
20
25
30
35
40
Gestational age (weeks)

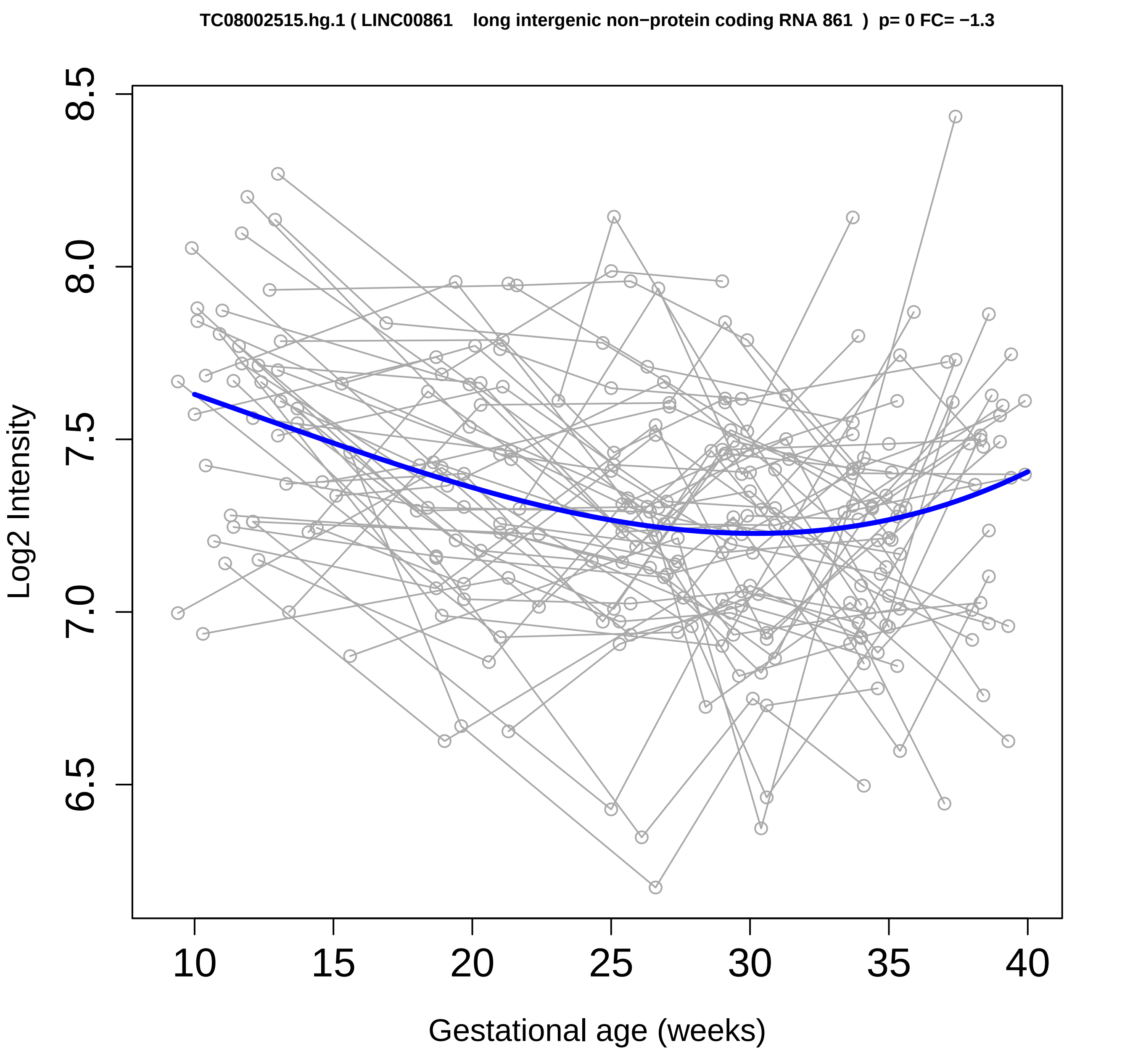

TC08002515.hg.1 (LINC00861 long intergenic non−protein coding RNA 861) p= 0 FC= −1.3
Log2 Intensity
6.5
7.0
7.5
8.0
8.5
10
15
20
25
30
35
40
Gestational age (weeks)

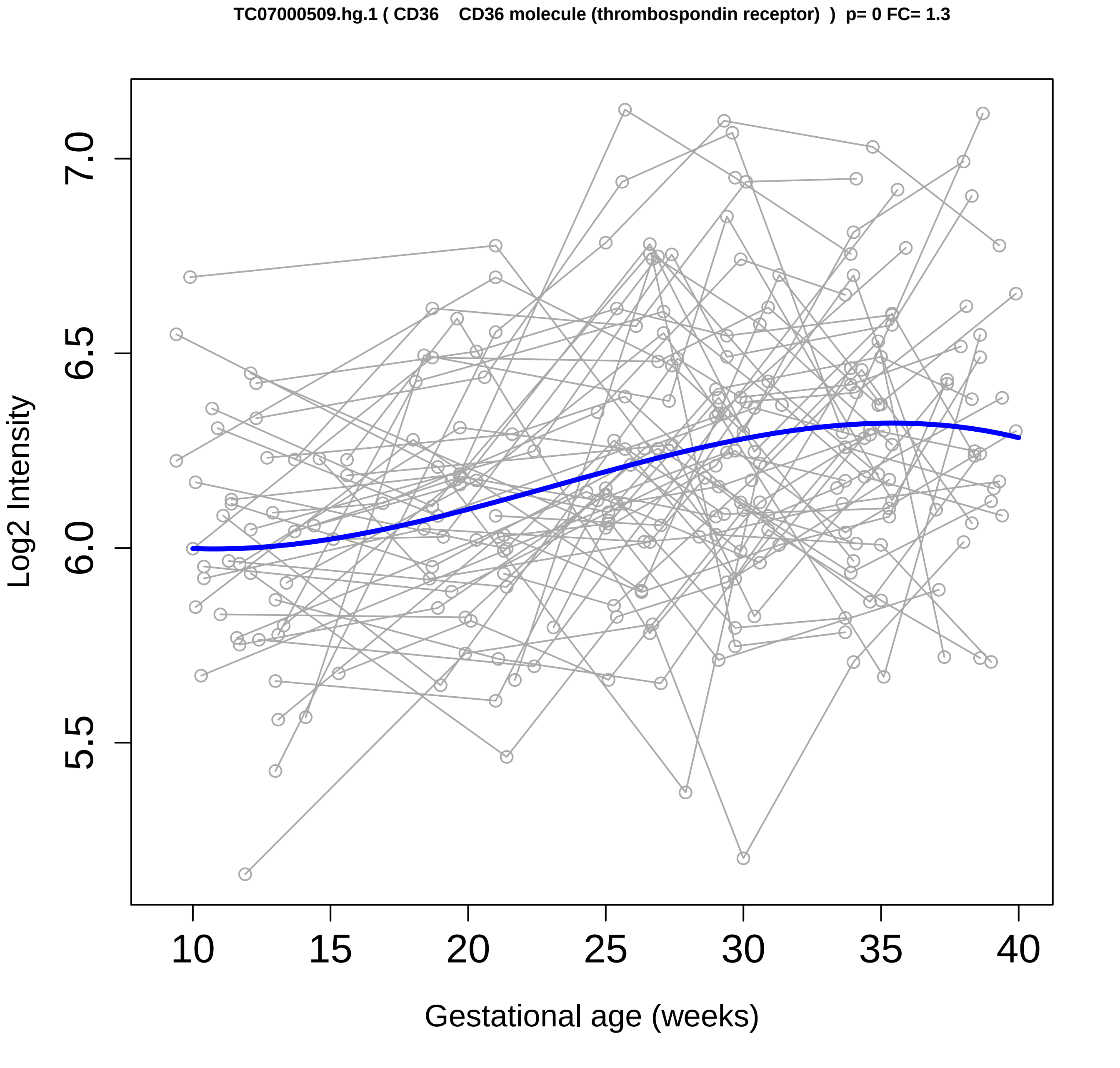
TC07000509.hg.1 (CD36   CD36 molecule (thrombospondin receptor)) p= 0 FC= 1.3
Log2 Intensity
5.5
6.0
6.5
7.0
10
15
20
25
30
35
40
Gestational age (weeks)

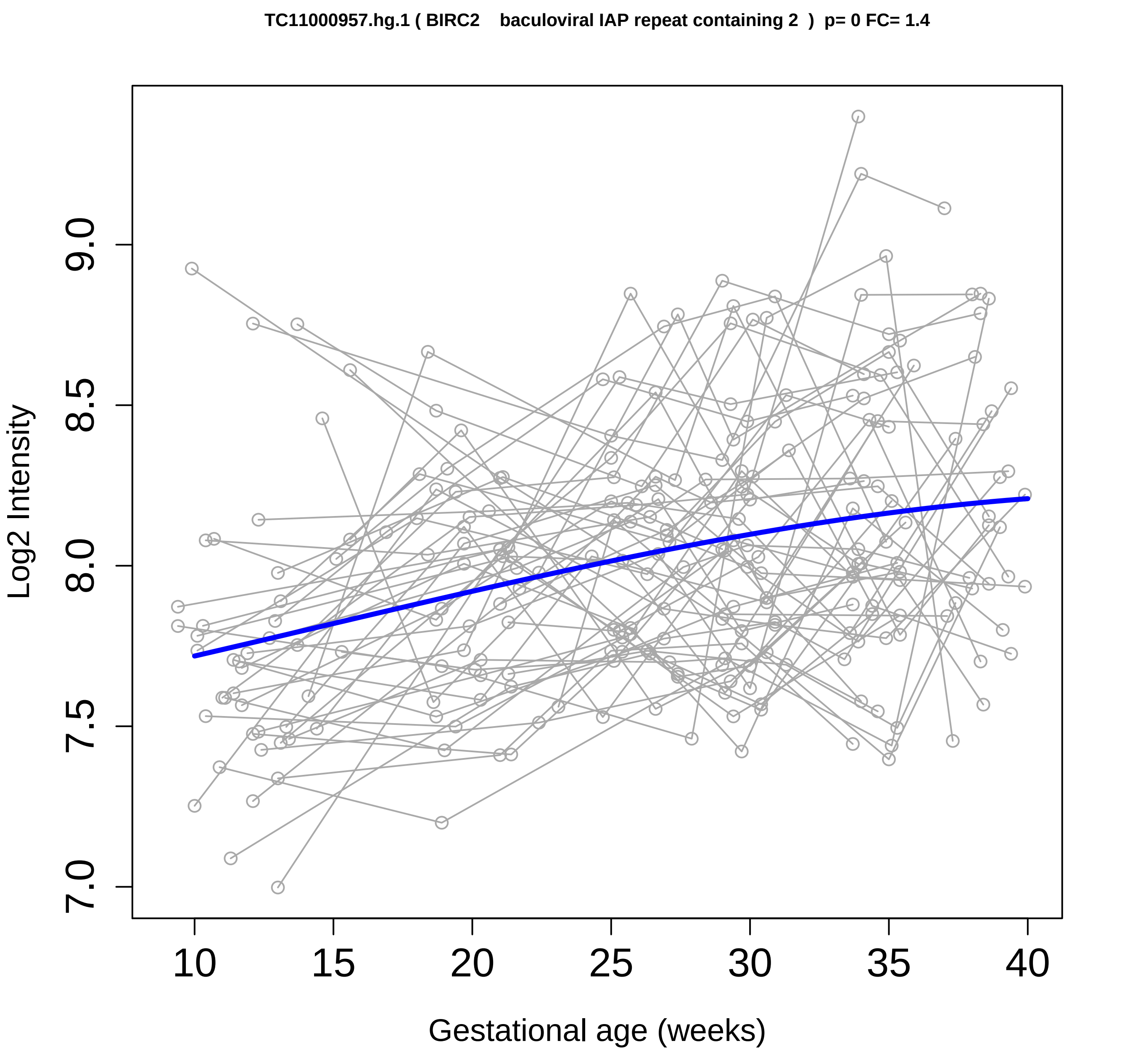

TC11000957.hg.1 (BIRC2 baculoviral IAP repeat containing 2) p= 0 FC= 1.4
Log2 Intensity
7.0
7.5
8.0
8.5
9.0
10
15
20
25
30
35
40
Gestational age (weeks)

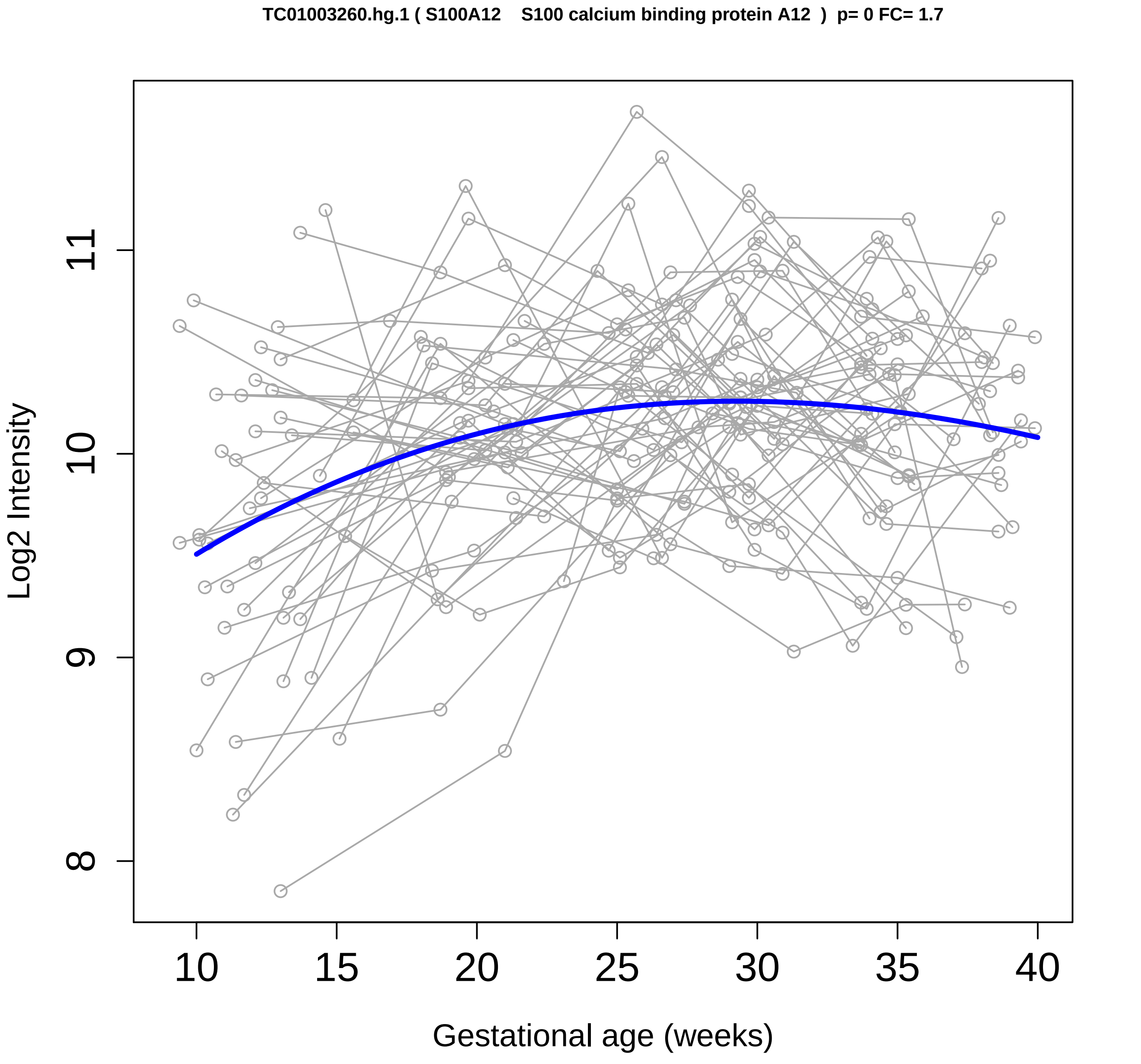

TC01003260.hg.1 (S100A12   S100 calcium binding protein A12) p= 0 FC= 1.7
Log2 Intensity
8
9
10
11
10
15
20
25
30
35
40
Gestational age (weeks)

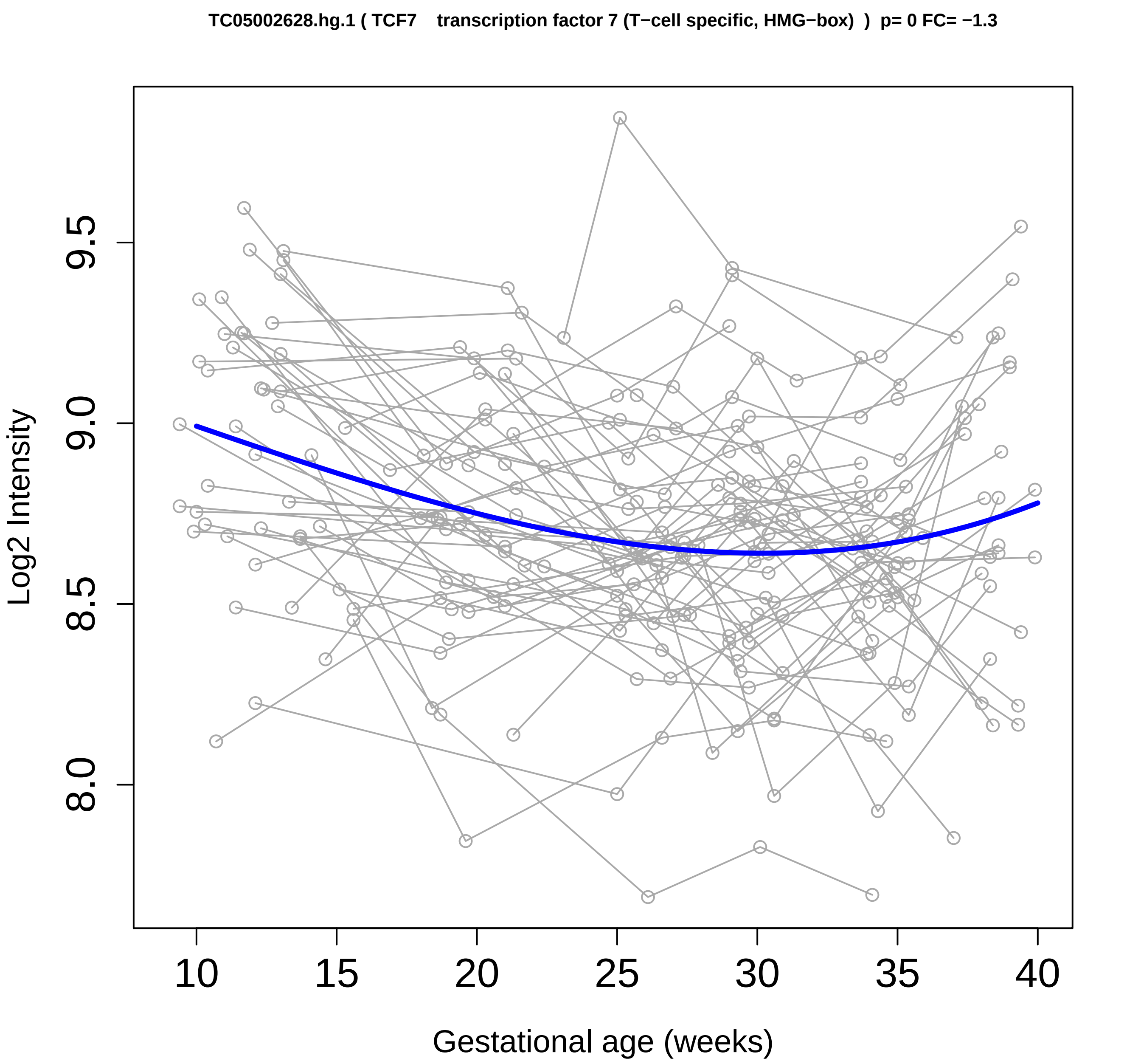
TC05002628.hg.1 (TCF7 transcription factor 7 (T−cell specific, HMG−box)) p= 0 FC= −1.3
Log2 Intensity
8.0
8.5
9.0
9.5
10
15
20
25
30
35
40
Gestational age (weeks)

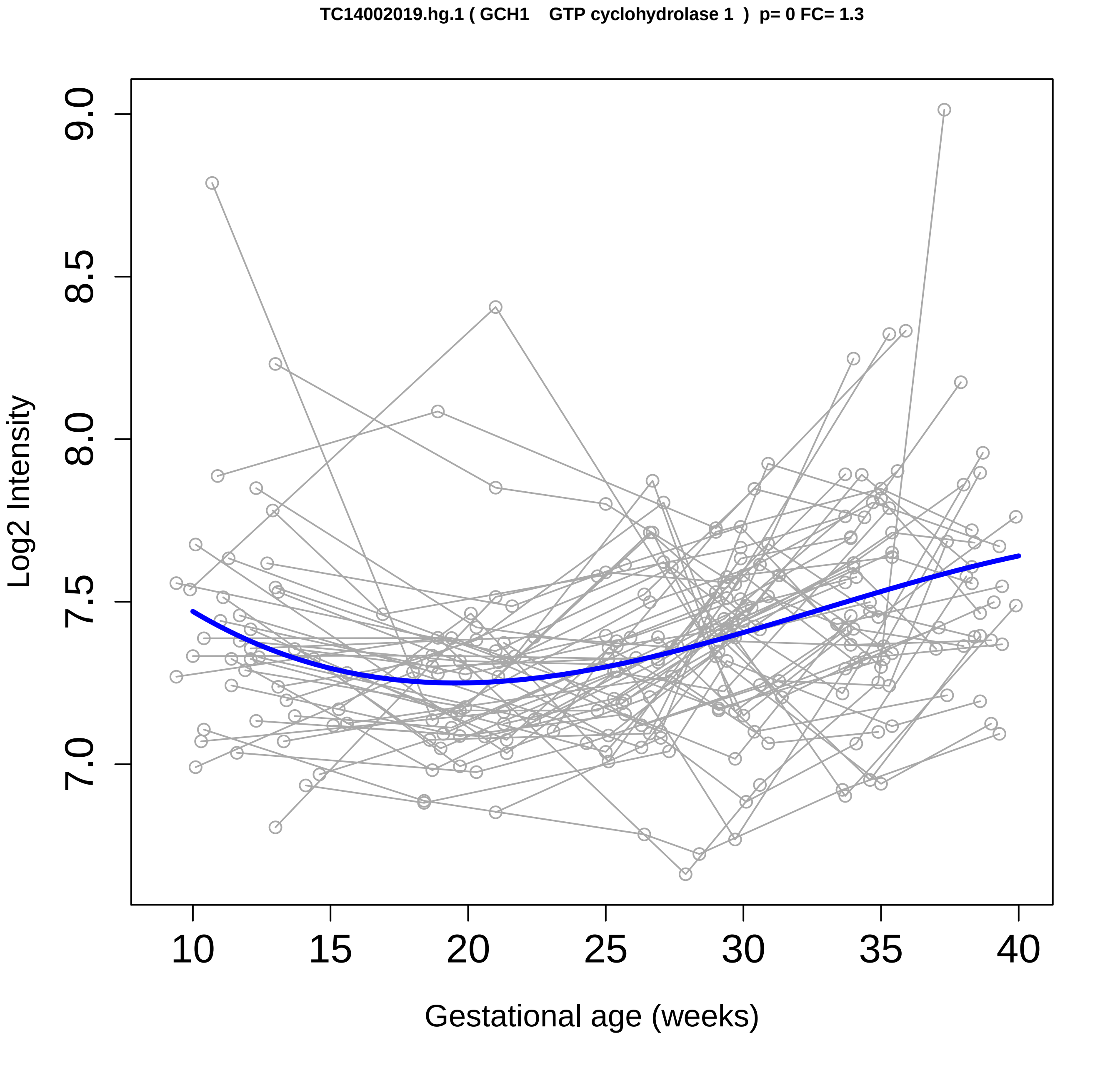

TC14002019.hg.1 (GCH1 GTP cyclohydrolase 1) p= 0 FC= 1.3
Log2 Intensity
9.0
8.5
8.0
7.5
7.0
10
15
20
25
30
35
40
Gestational age (weeks)

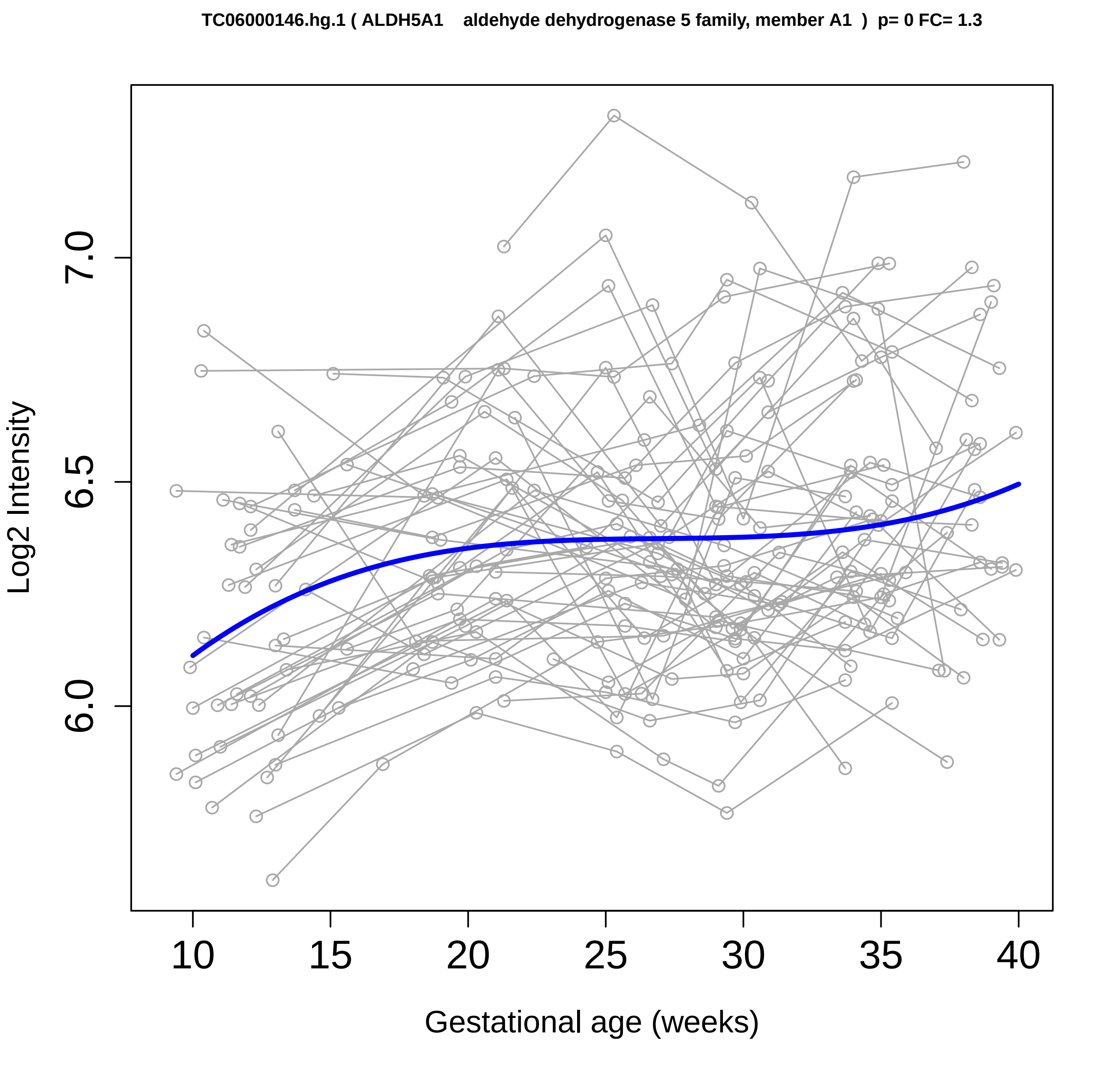

TC06000146.hg.1 (ALDH5A1 aldehyde dehydrogenase 5 family, member A1) p= 0 FC= 1.3
Log2 Intensity
Gestational age (weeks)
6.0
6.5
7.0
10
15
20
25
30
35
40

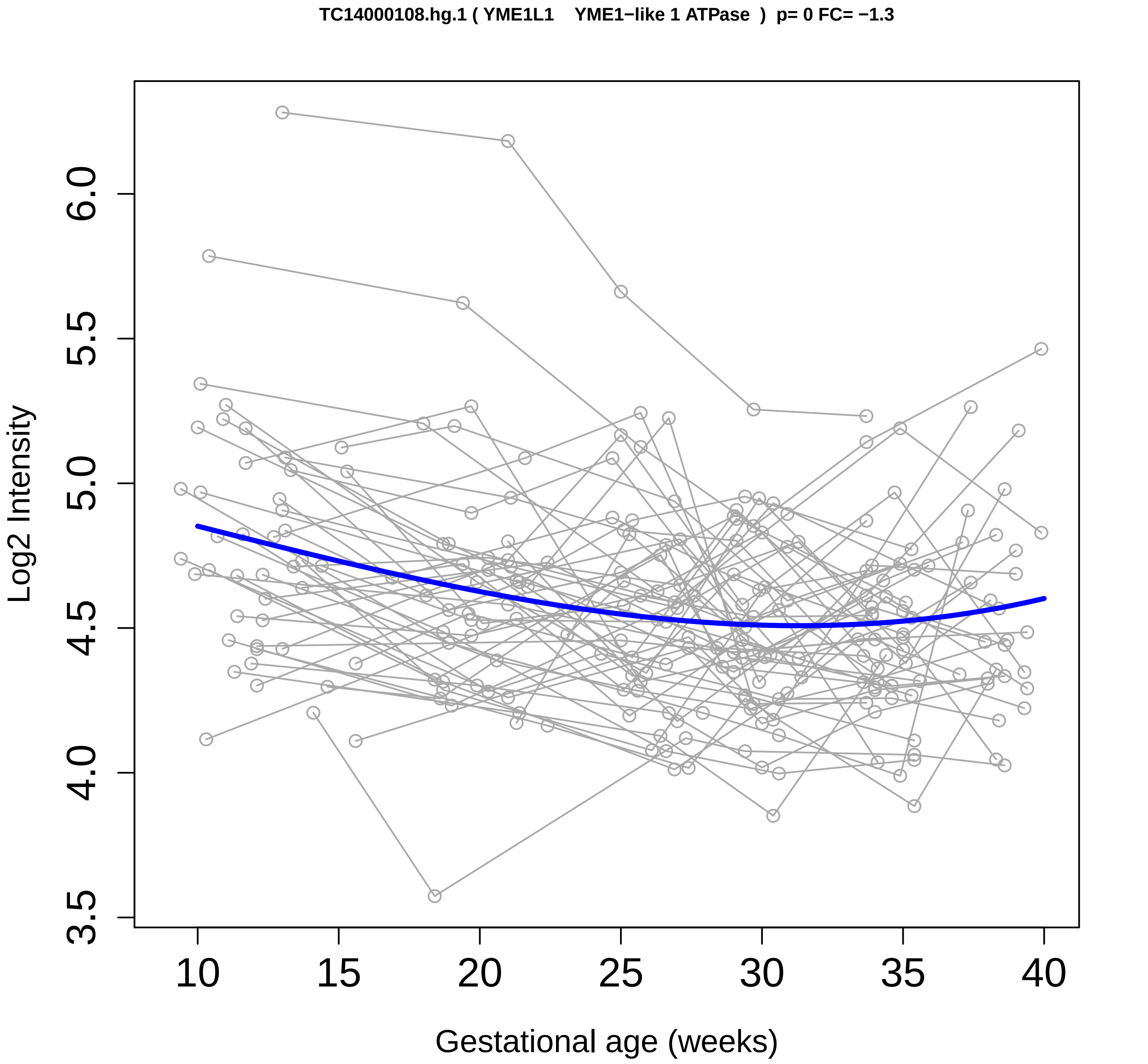

TC14000108.hg.1 (YME1L1   YME1−like 1 ATPase) p= 0 FC= −1.3
Log2 Intensity
3.5
4.0
4.5
5.0
5.5
6.0
10
15
20
25
30
35
40
Gestational age (weeks)

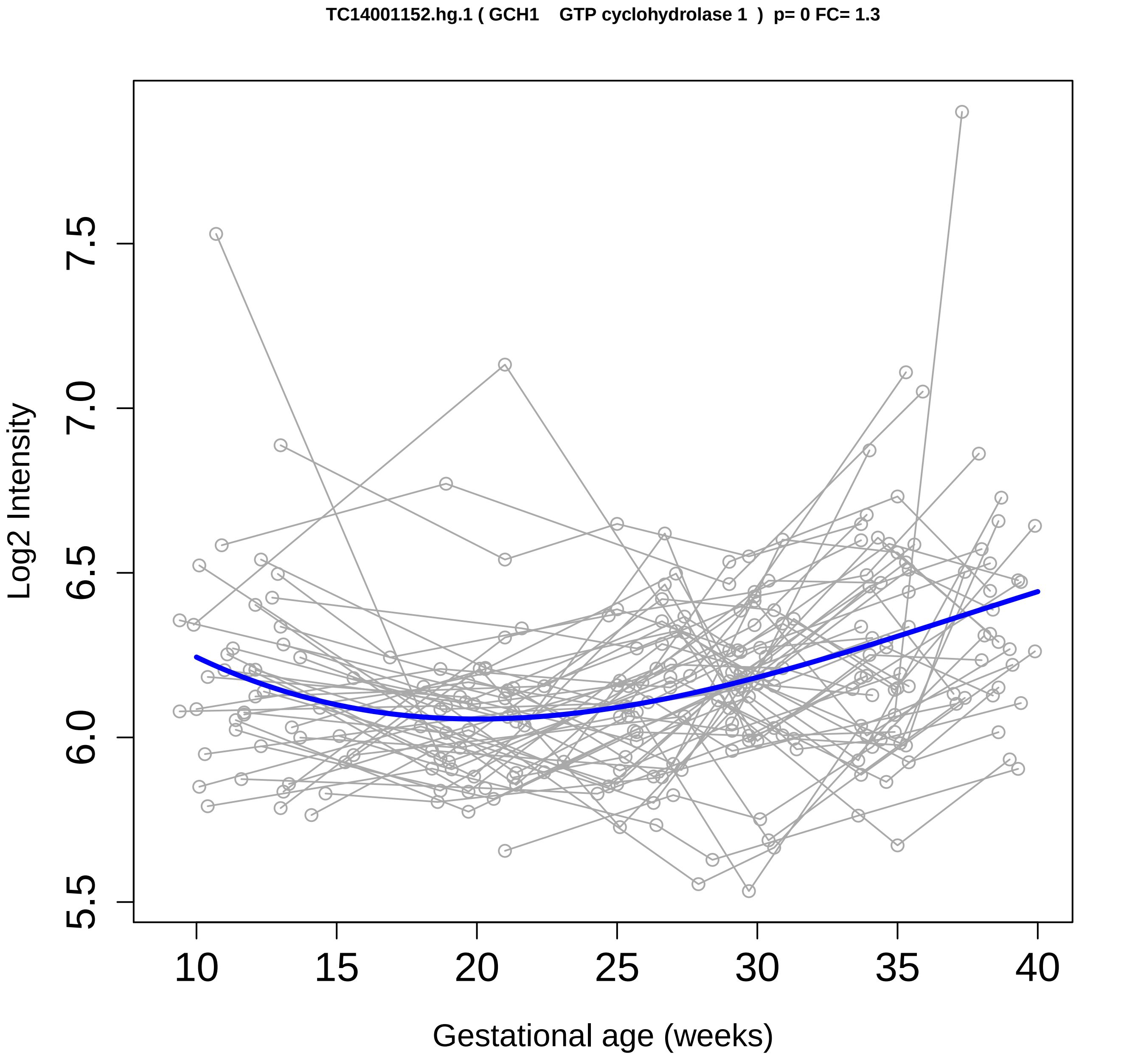
TC14001152.hg.1 (GCH1 GTP cyclohydrolase 1) p= 0 FC= 1.3
Log2 Intensity
5.5
6.0
6.5
7.0
7.5
10
15
20
25
30
35
40
Gestational age (weeks)

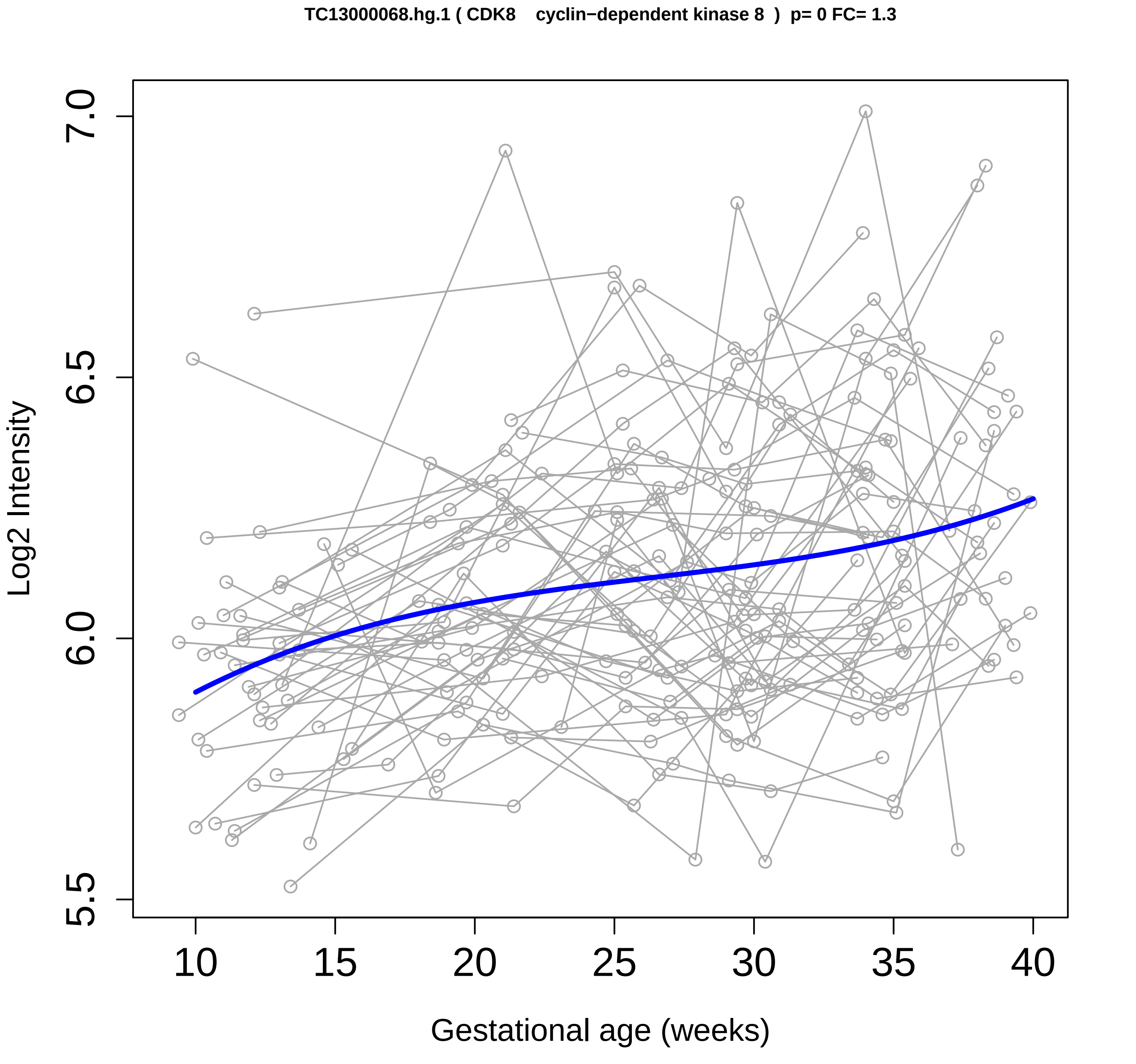

TC13000068.hg.1 (CDK8 cyclin−dependent kinase 8) p= 0 FC= 1.3
Log2 Intensity
7.0
6.5
6.0
5.5
10
15
20
25
30
35
40
Gestational age (weeks)

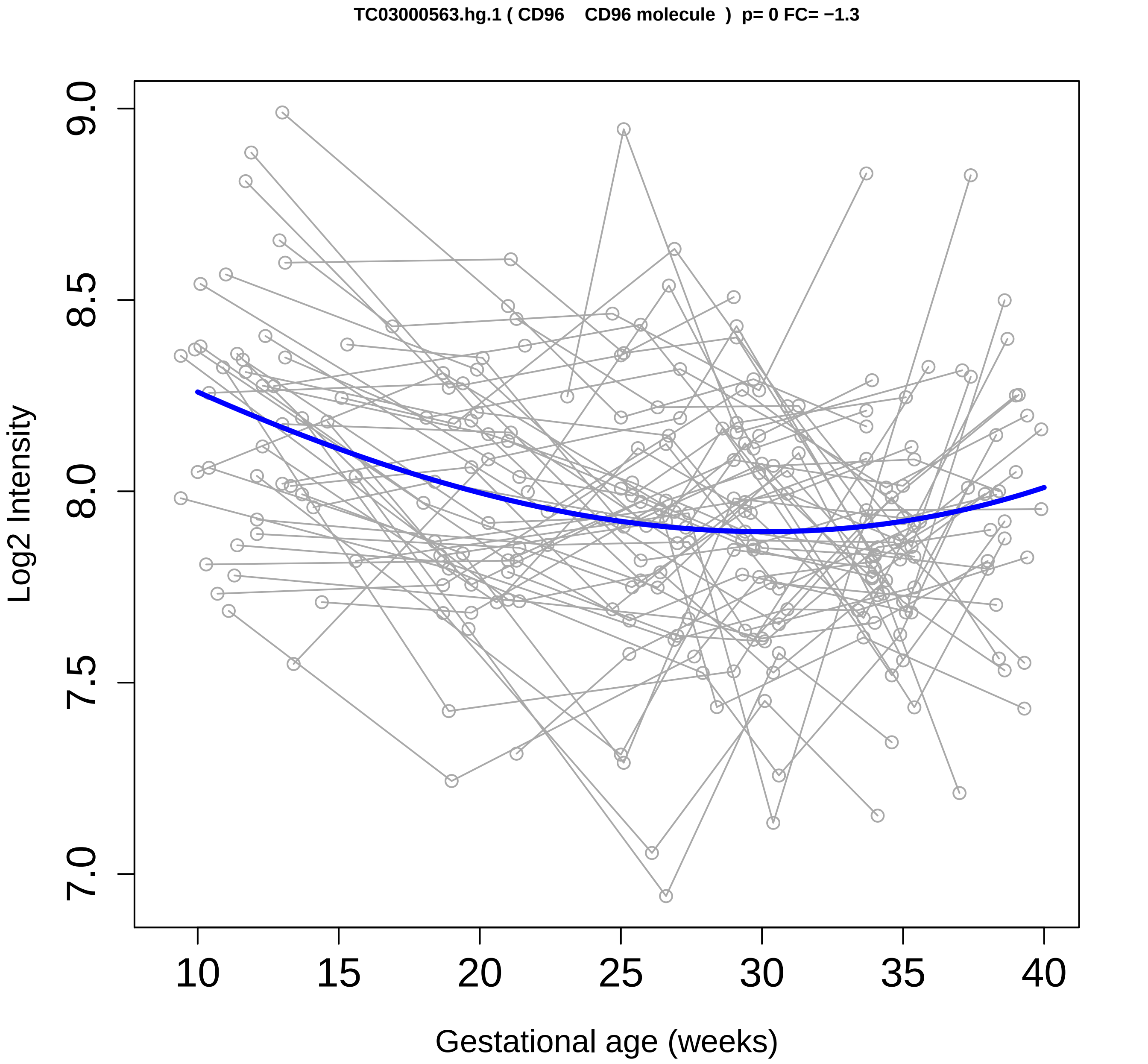
TC03000563.hg.1 (CD96   CD96 molecule) p= 0 FC= −1.3
Log2 Intensity
9.0
8.5
8.0
7.5
7.0
10
15
20
25
30
35
40
Gestational age (weeks)

TC12001797.hg.1 (POC1B   POC1 centriolar protein B) p= 0 FC= 1.3

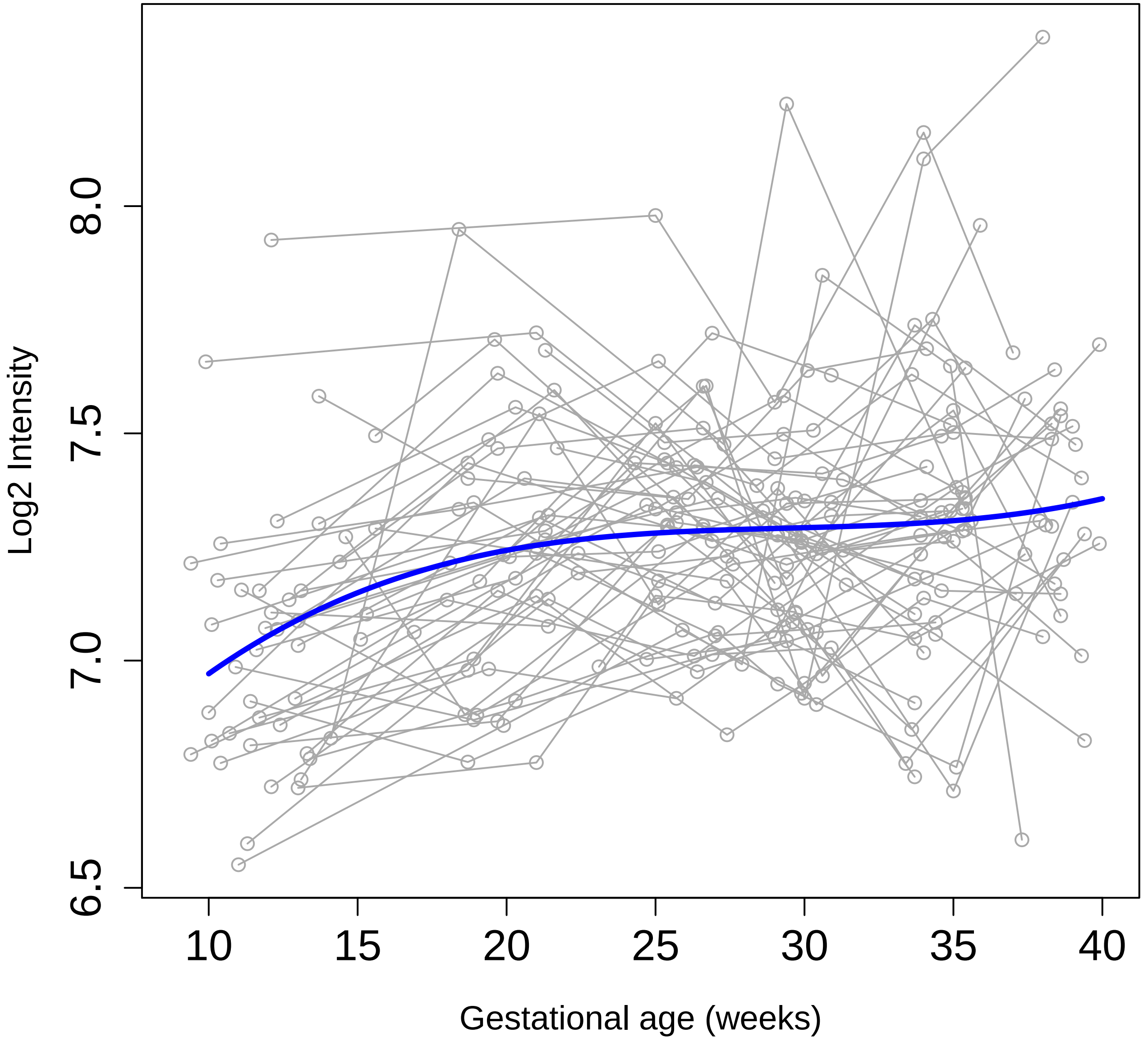

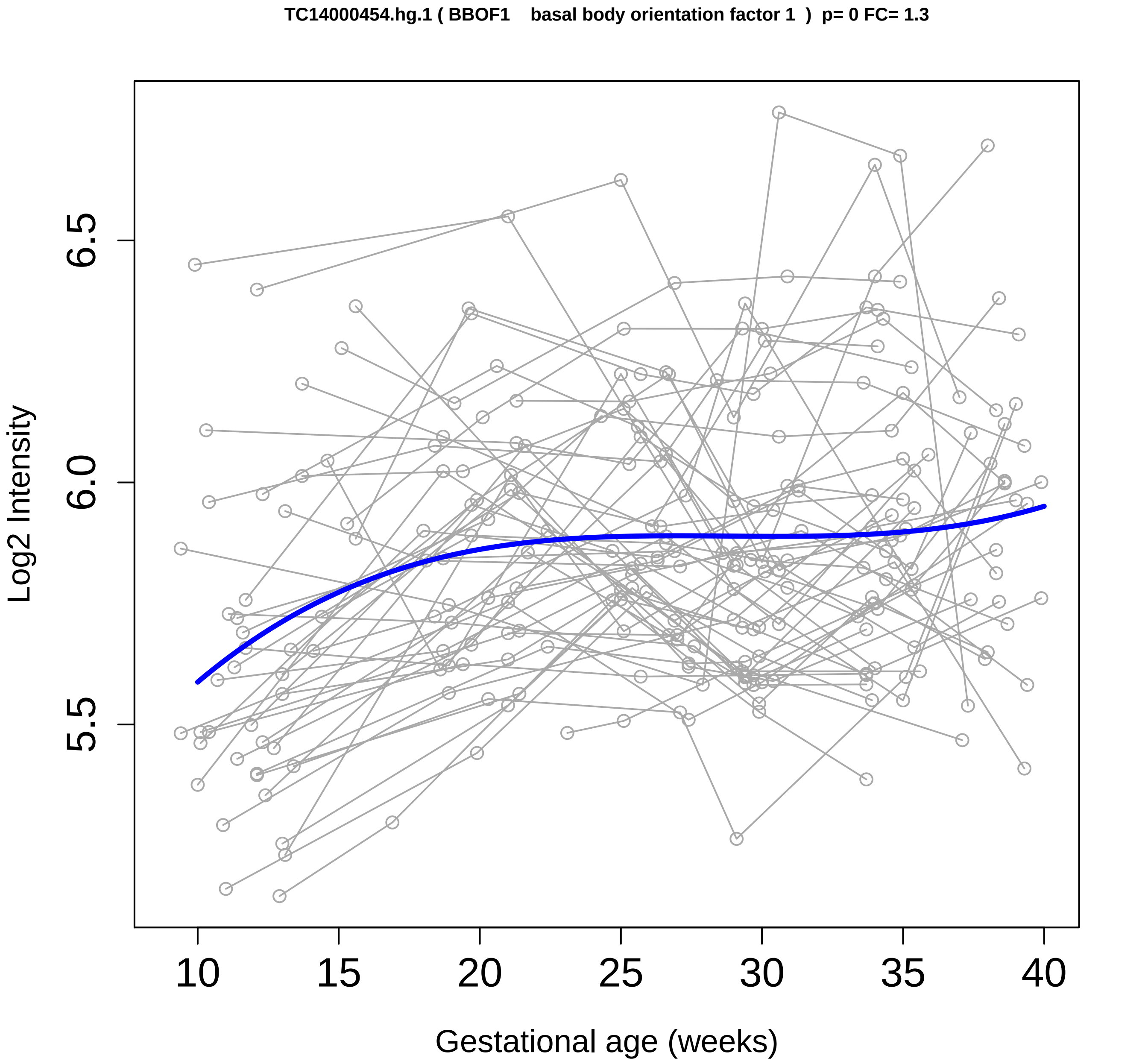

TC14000454.hg.1 (BBOF1 basal body orientation factor 1) p= 0 FC= 1.3
Log2 Intensity
5.5
6.0
6.5
10
15
20
25
30
35
40
Gestational age (weeks)

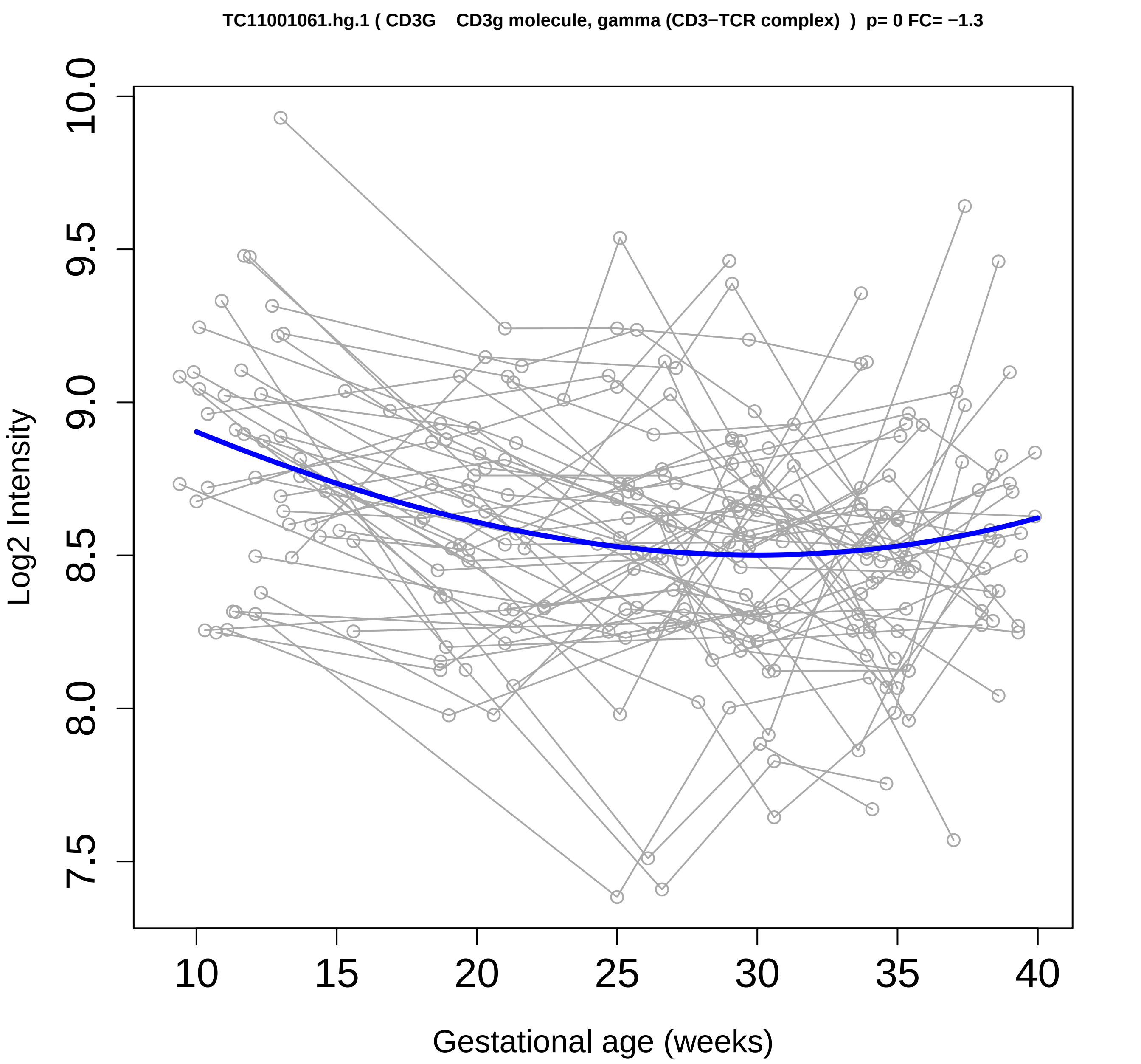

TC11001061.hg.1 (CD3G CD3g molecule, gamma (CD3−TCR complex)) p= 0 FC= −1.3
Log2 Intensity
10.0
9.5
9.0
8.5
8.0
7.5
10
15
20
25
30
35
40
Gestational age (weeks)

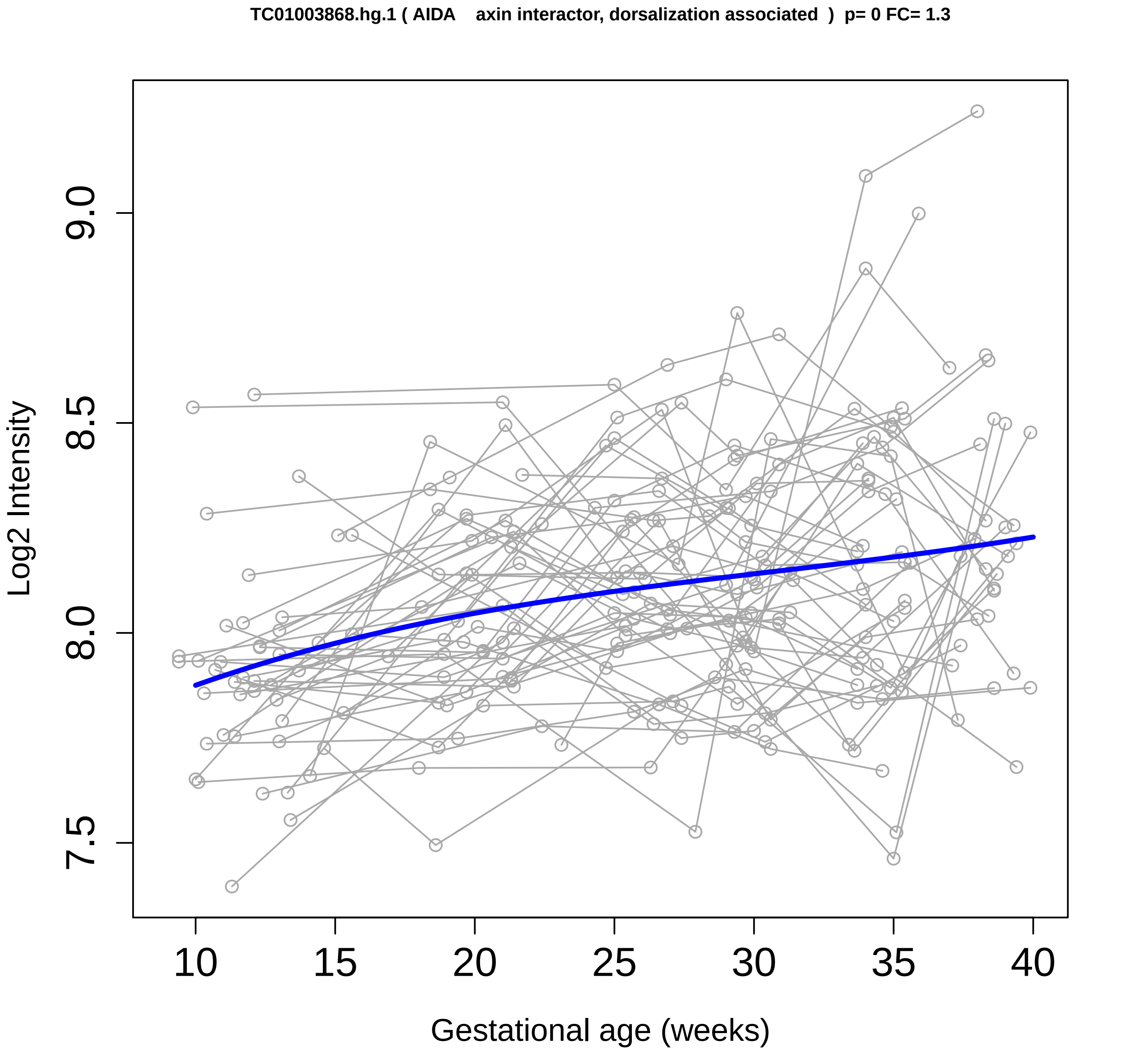
TC01003868.hg.1 (AIDA axin interactor, dorsalization associated) p= 0 FC= 1.3
Log2 Intensity
7.5
8.0
8.5
9.0
10
15
20
25
30
35
40
Gestational age (weeks)

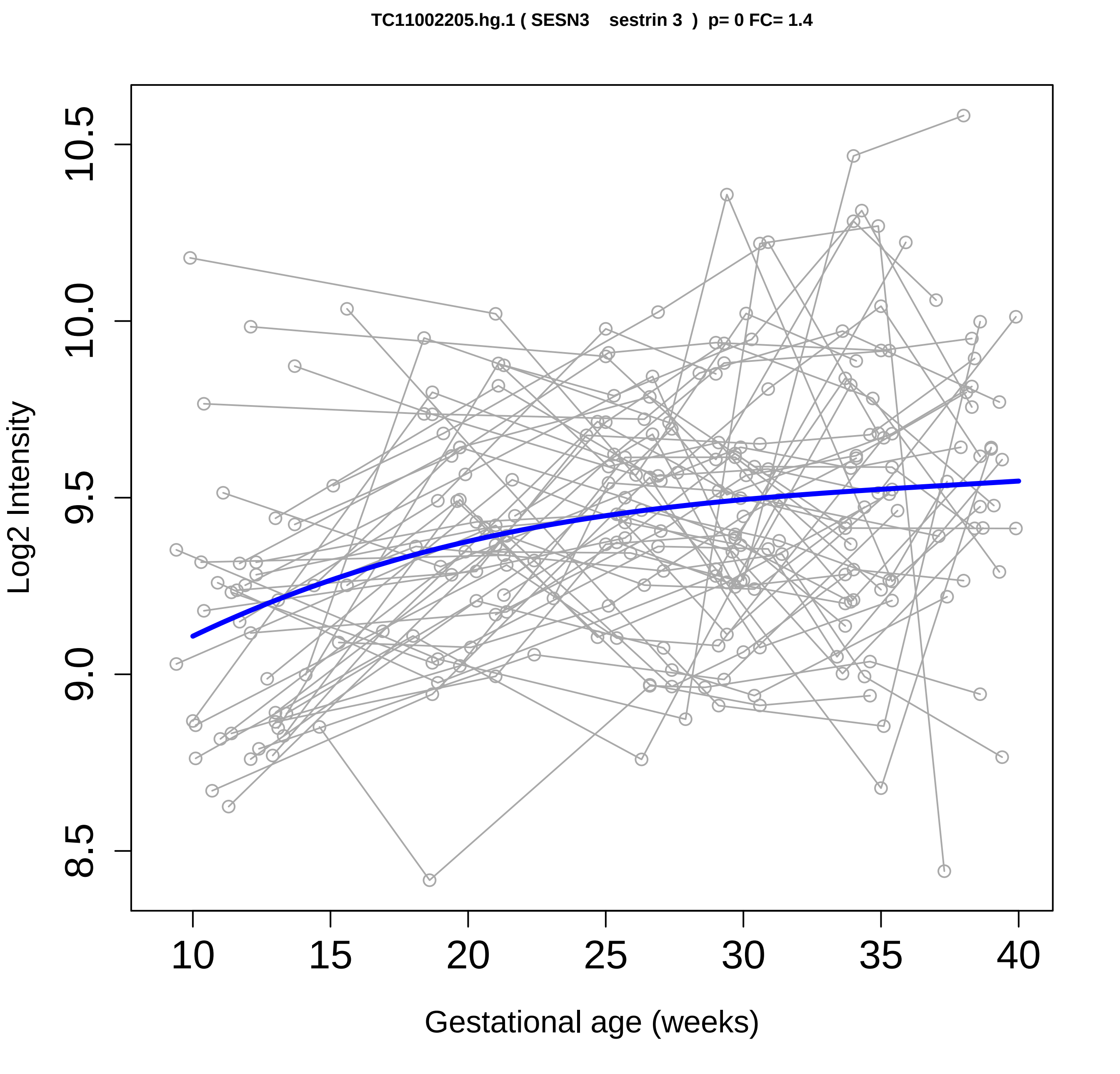
TC11002205.hg.1 (SESN3 sestrin 3) p= 0 FC= 1.4
Log2 Intensity
8.5
9.0
9.5
10.0
10.5
10
15
20
25
30
35
40
Gestational age (weeks)

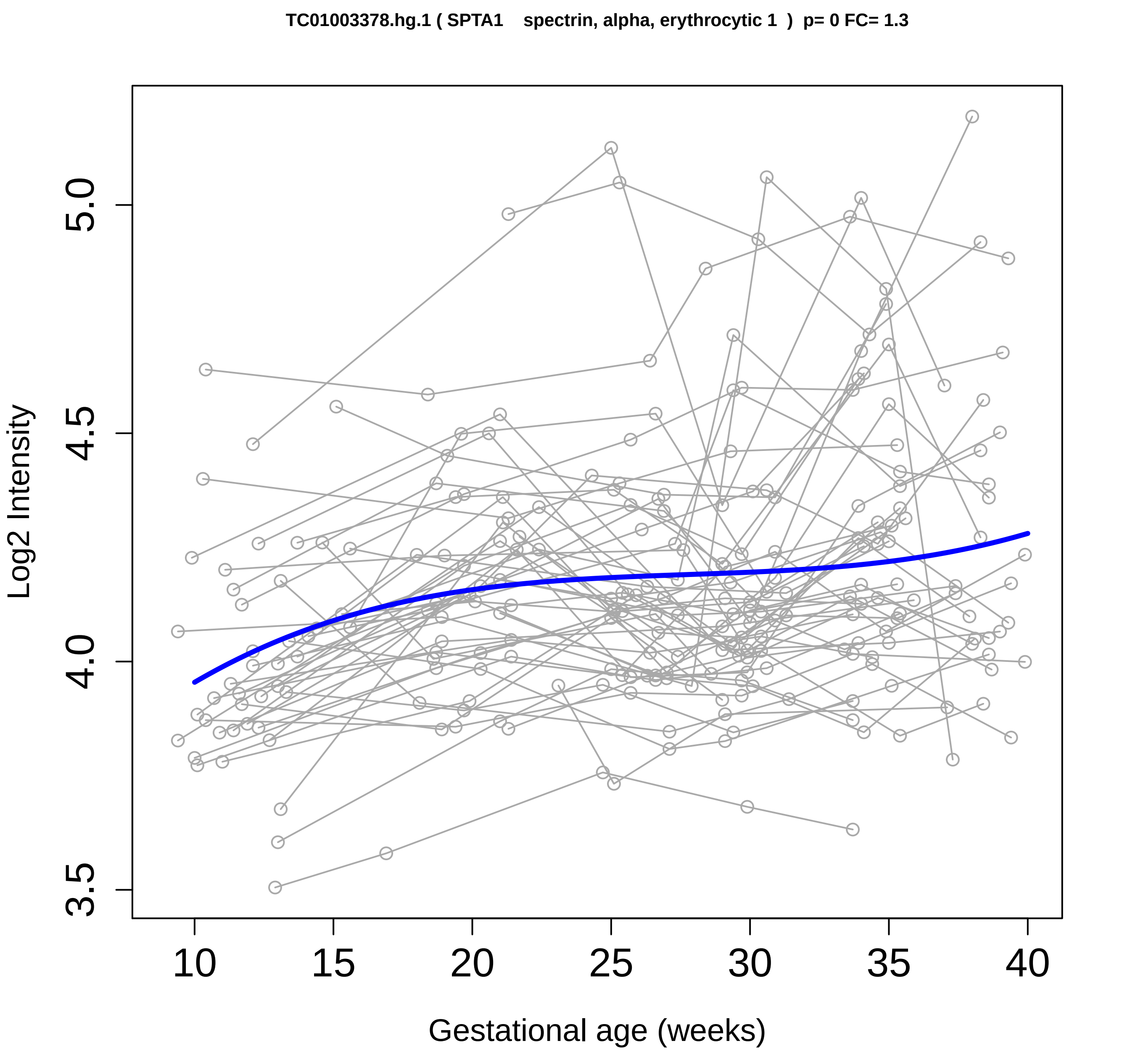
TC01003378.hg.1 (SPTA1 spectrin, alpha, erythrocytic 1) p= 0 FC= 1.3
Log2 Intensity
3.5
4.0
4.5
5.0
10
15
20
25
30
35
40
Gestational age (weeks)

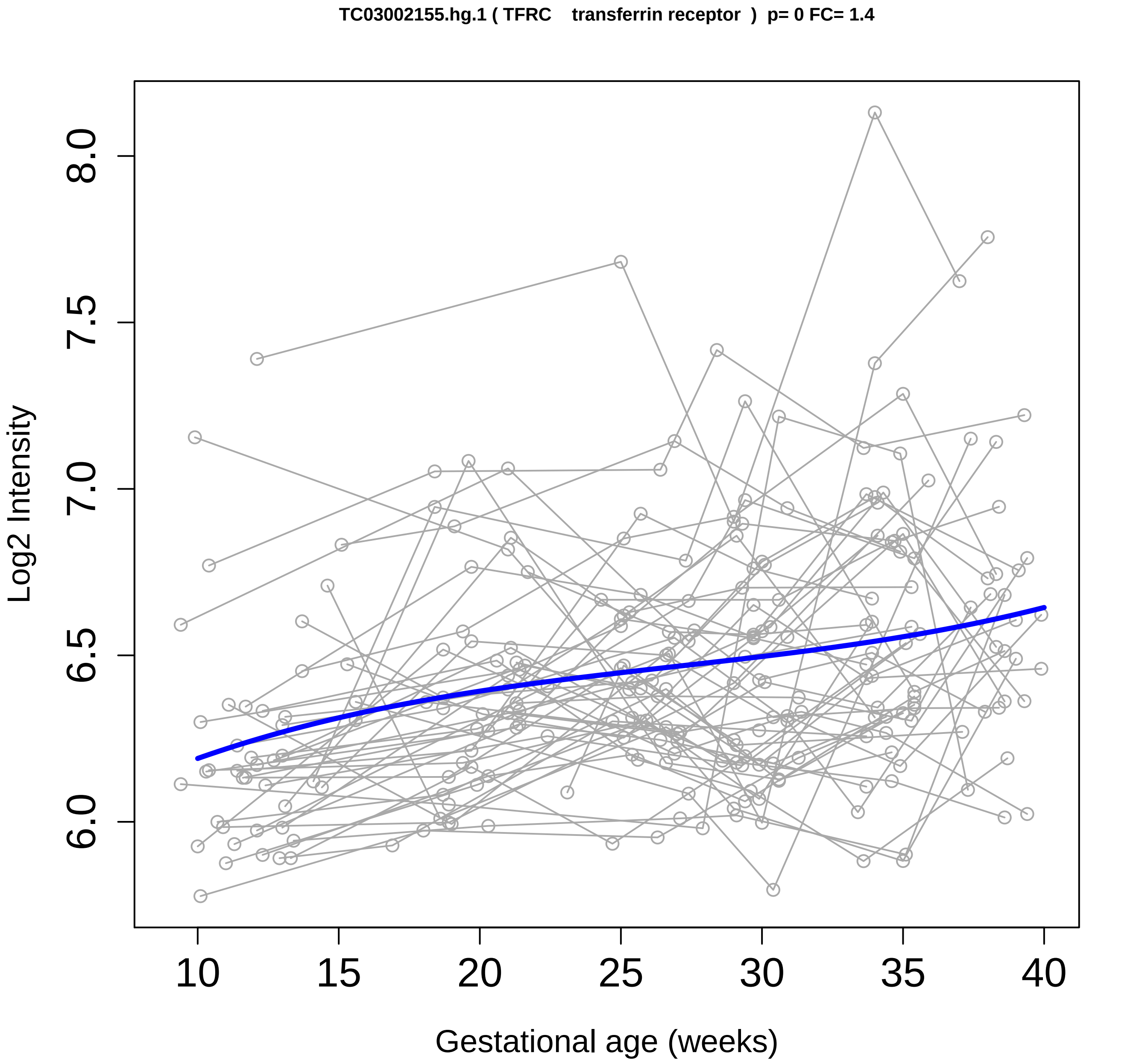

TC03002155.hg.1 (TFRC transferrin receptor) p= 0 FC= 1.4
Log2 Intensity
6.0
6.5
7.0
7.5
8.0
10
15
20
25
30
35
40
Gestational age (weeks)

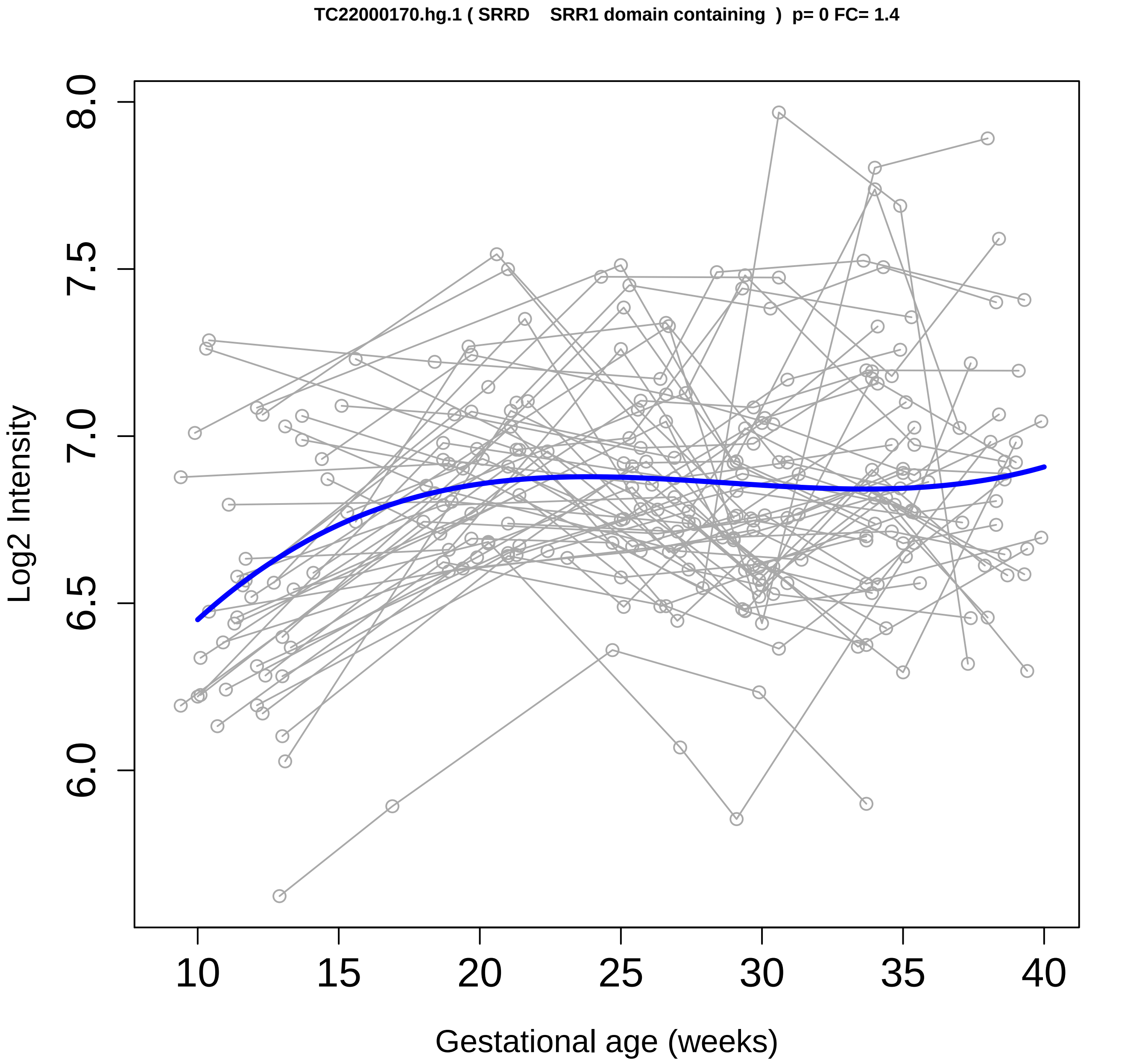
TC22000170.hg.1 (SRRD SRR1 domain containing) p= 0 FC= 1.4
Log2 Intensity
8.0
7.5
7.0
6.5
6.0
10
15
20
25
30
35
40
Gestational age (weeks)

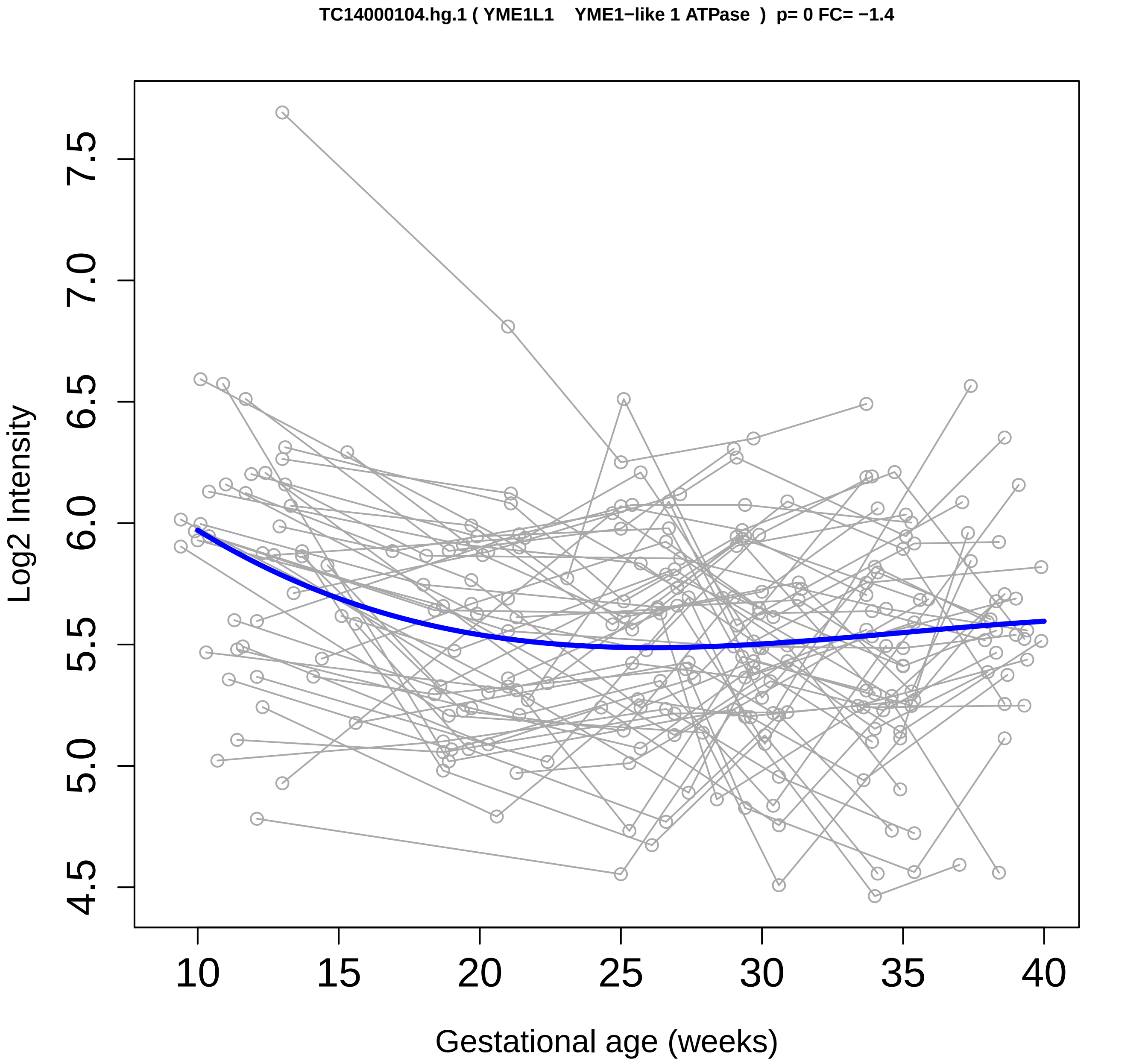

TC14000104.hg.1 (YME1L1 YME1−like 1 ATPase) p= 0 FC= −1.4
Log2 Intensity
4.5
5.0
5.5
6.0
6.5
7.0
7.5
10
15
20
25
30
35
40
Gestational age (weeks)

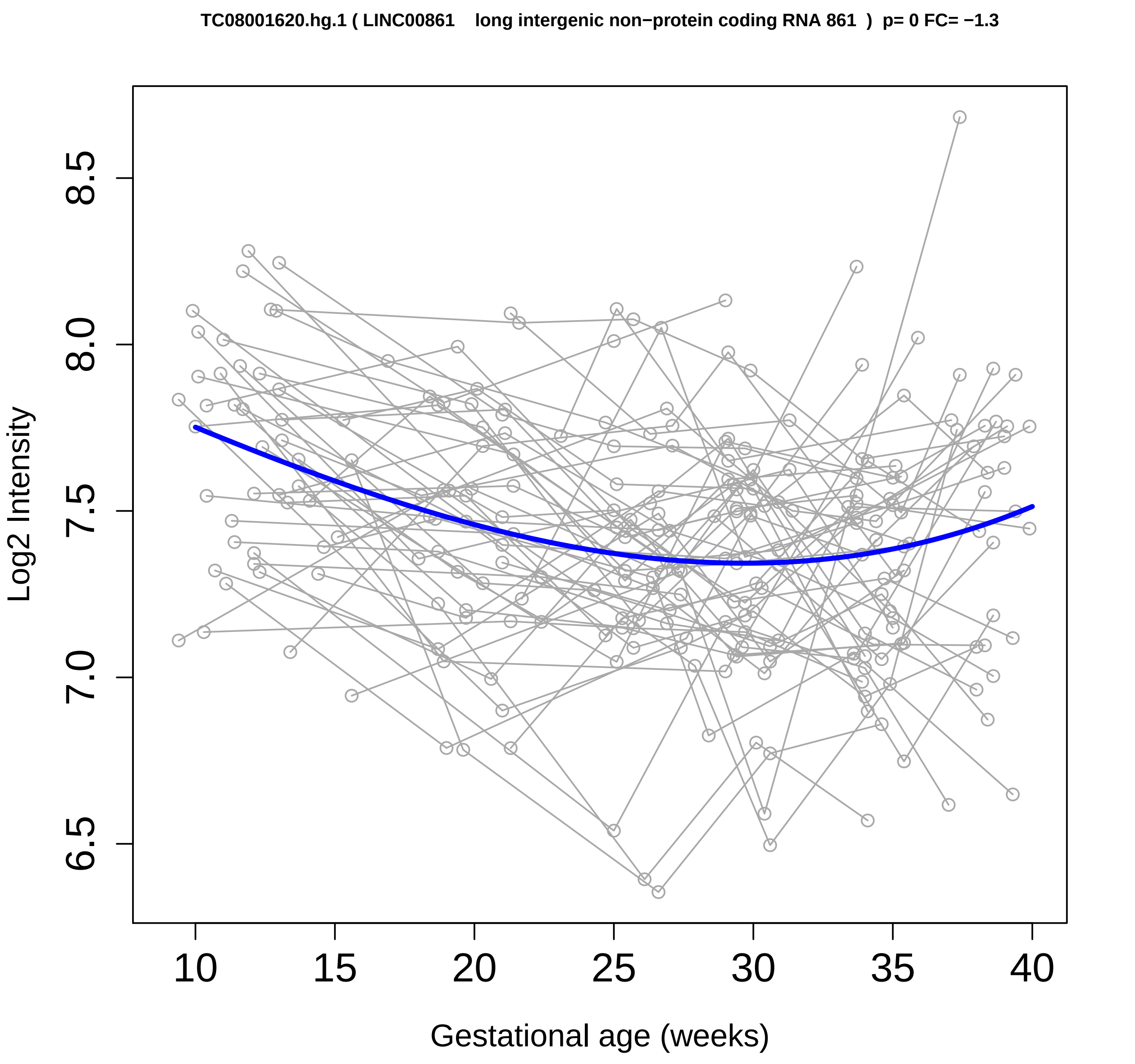

TC08001620.hg.1 (LINC00861 long intergenic non−protein coding RNA 861) p= 0 FC= −1.3
Log2 Intensity
8.5
8.0
7.5
7.0
6.5
10
15
20
25
30
35
40
Gestational age (weeks)

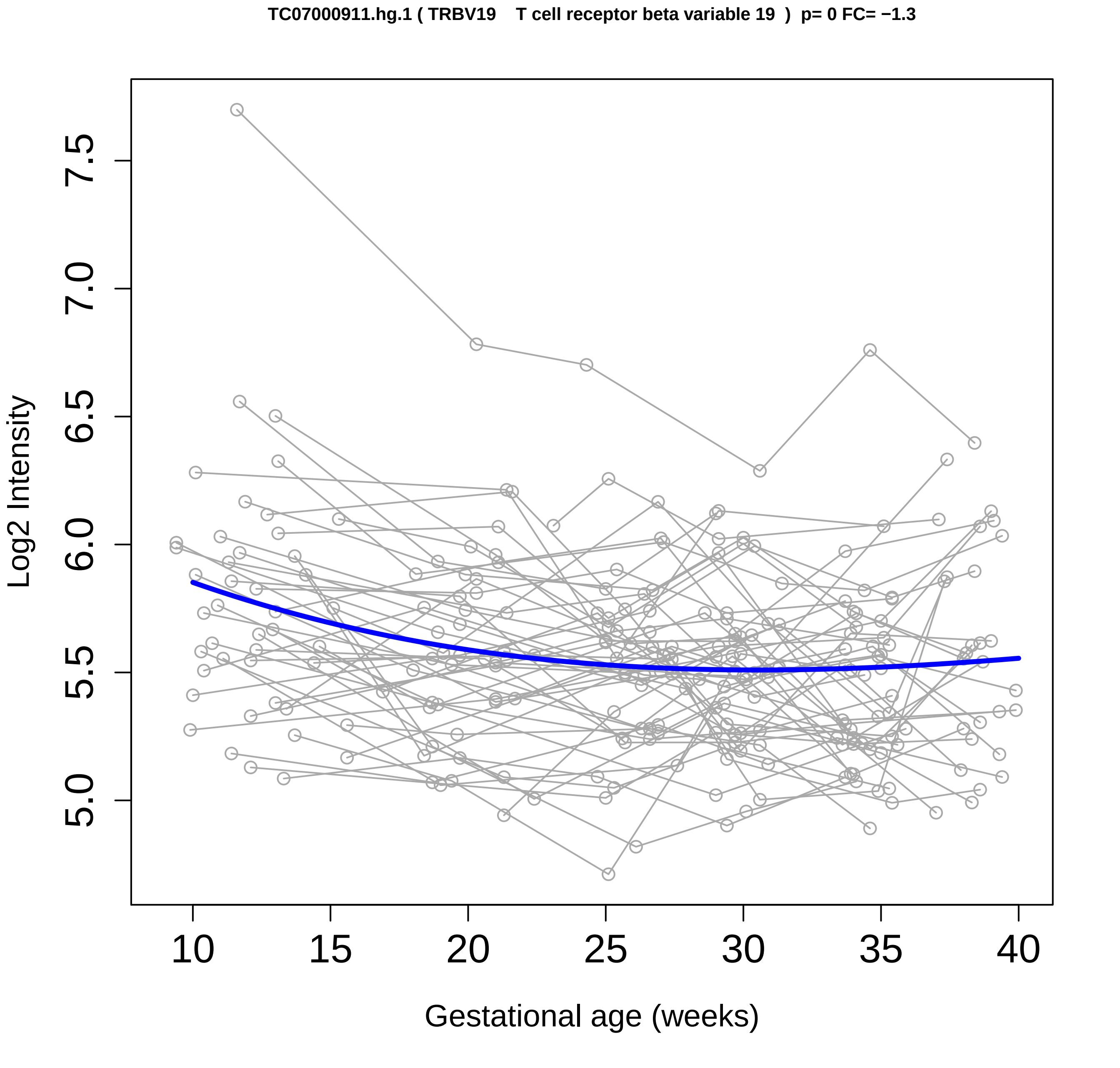
TC07000911.hg.1 (TRBV19 T cell receptor beta variable 19) p= 0 FC= −1.3
Log2 Intensity
7.5
7.0
6.5
6.0
5.5
5.0
10
15
20
25
30
35
40
Gestational age (weeks)

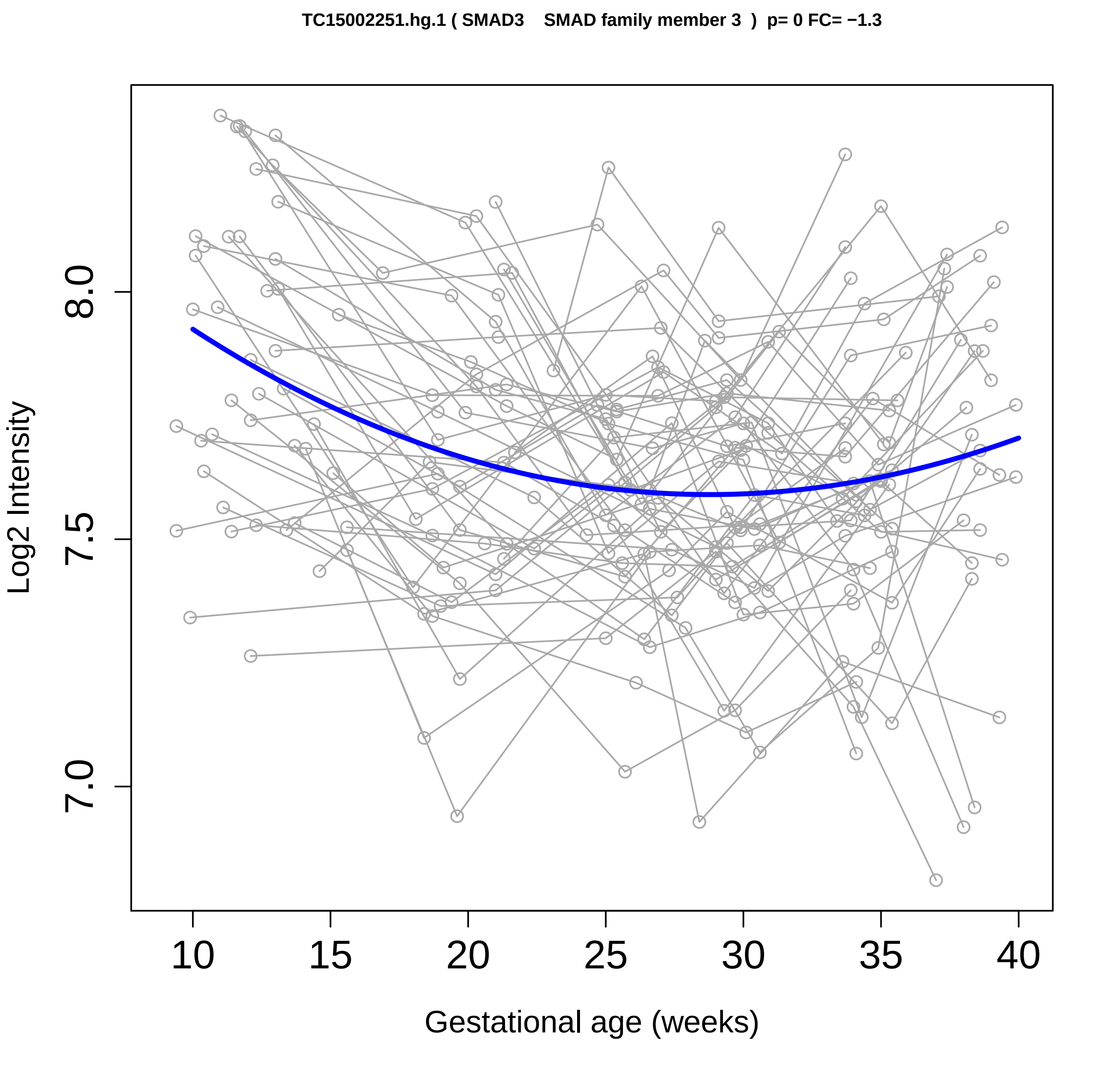

TC15002251.hg.1 (SMAD3 SMAD family member 3) p= 0 FC= −1.3
Log2 Intensity
7.0
7.5
8.0
10
15
20
25
30
35
40
Gestational age (weeks)

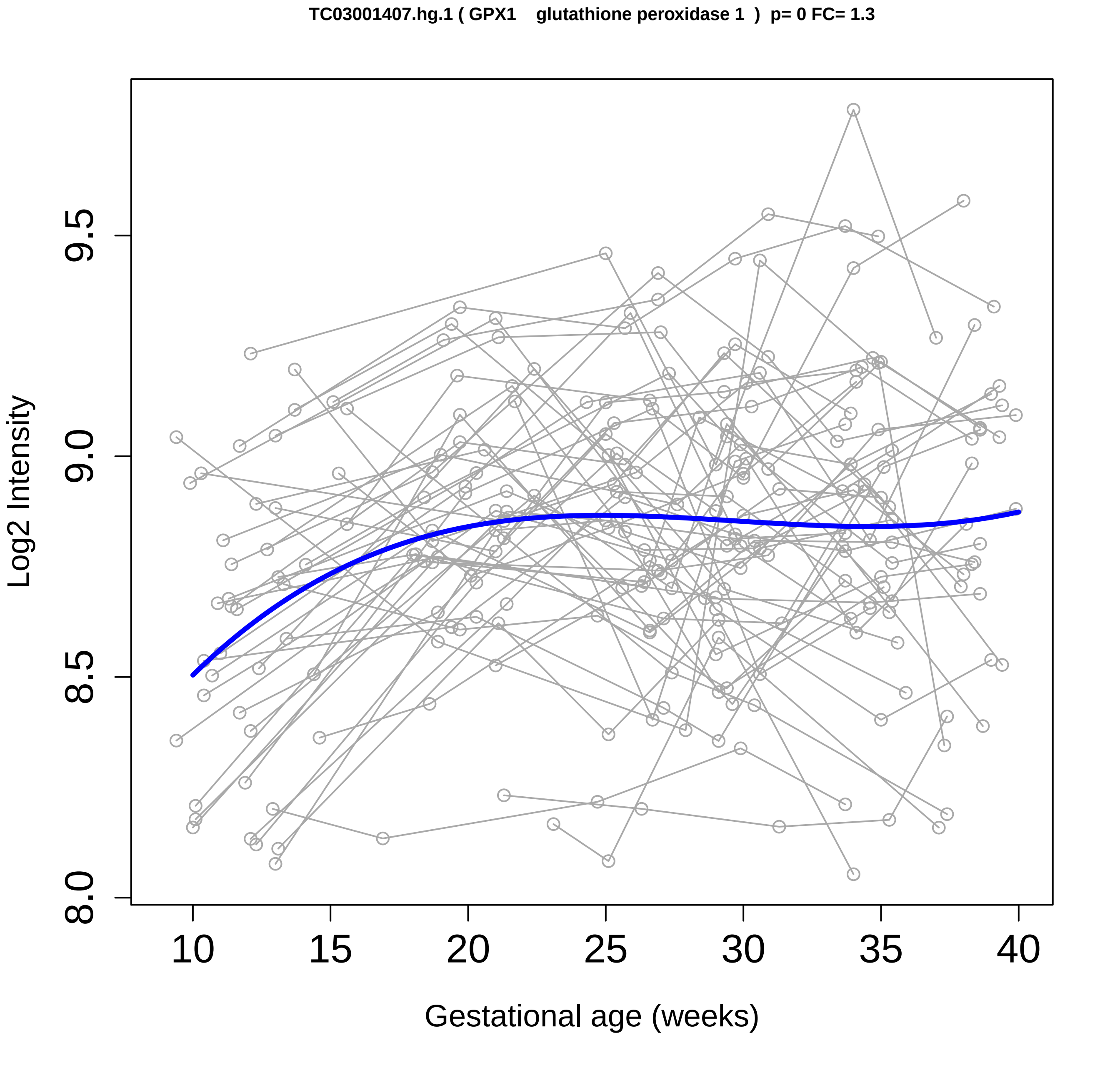

TC03001407.hg.1 (GPX1 glutathione peroxidase 1) p= 0 FC= 1.3
Log2 Intensity
8.0
8.5
9.0
9.5
10
15
20
25
30
35
40
Gestational age (weeks)

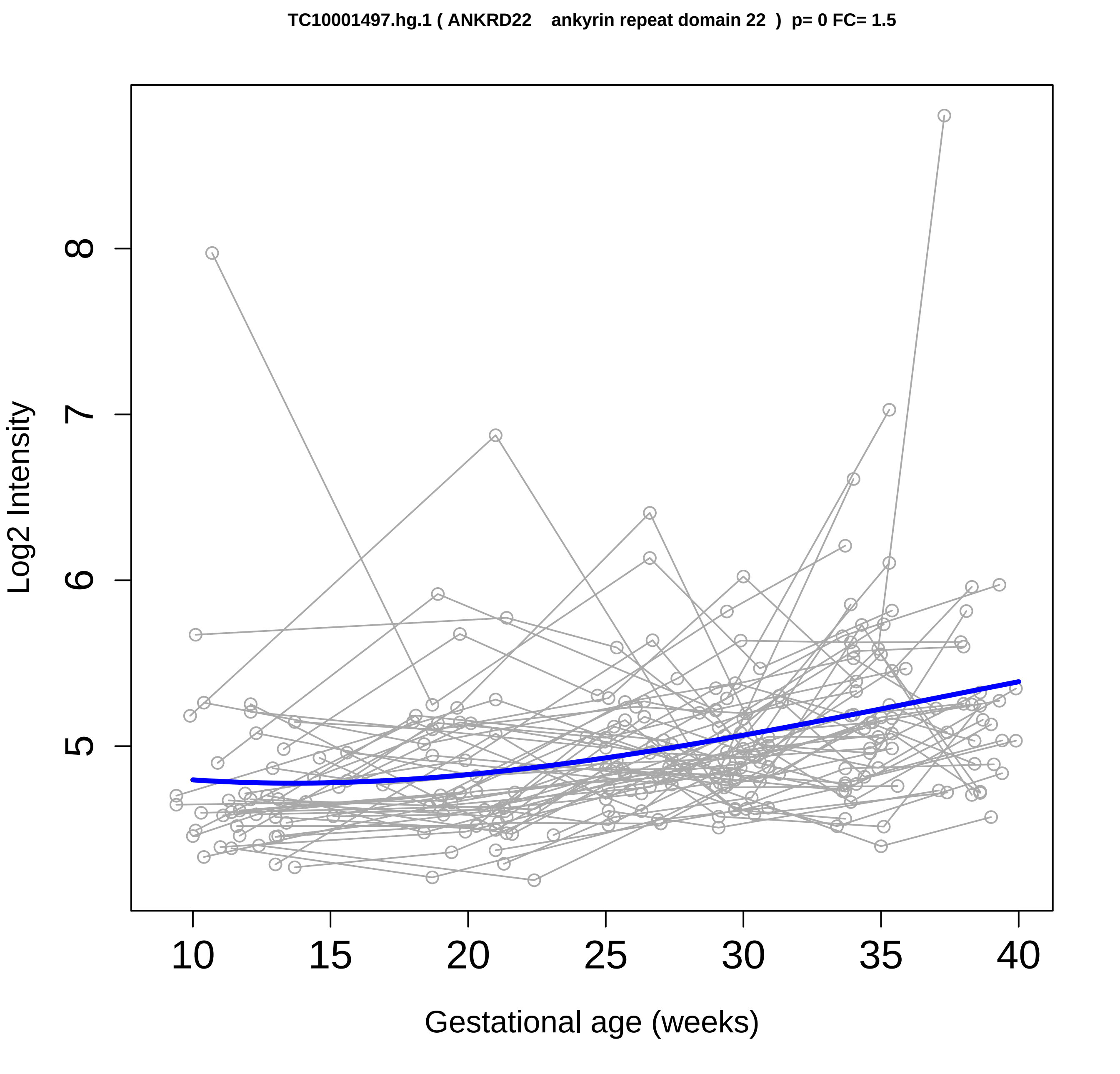
TC10001497.hg.1 (ANKRD22 ankyrin repeat domain 22) p= 0 FC= 1.5
Log2 Intensity
5
6
7
8
10
15
20
25
30
35
40
Gestational age (weeks)

TC17000299.hg.1 (UBBP4   ubiquitin B pseudogene 4) p= 0 FC= 1.3

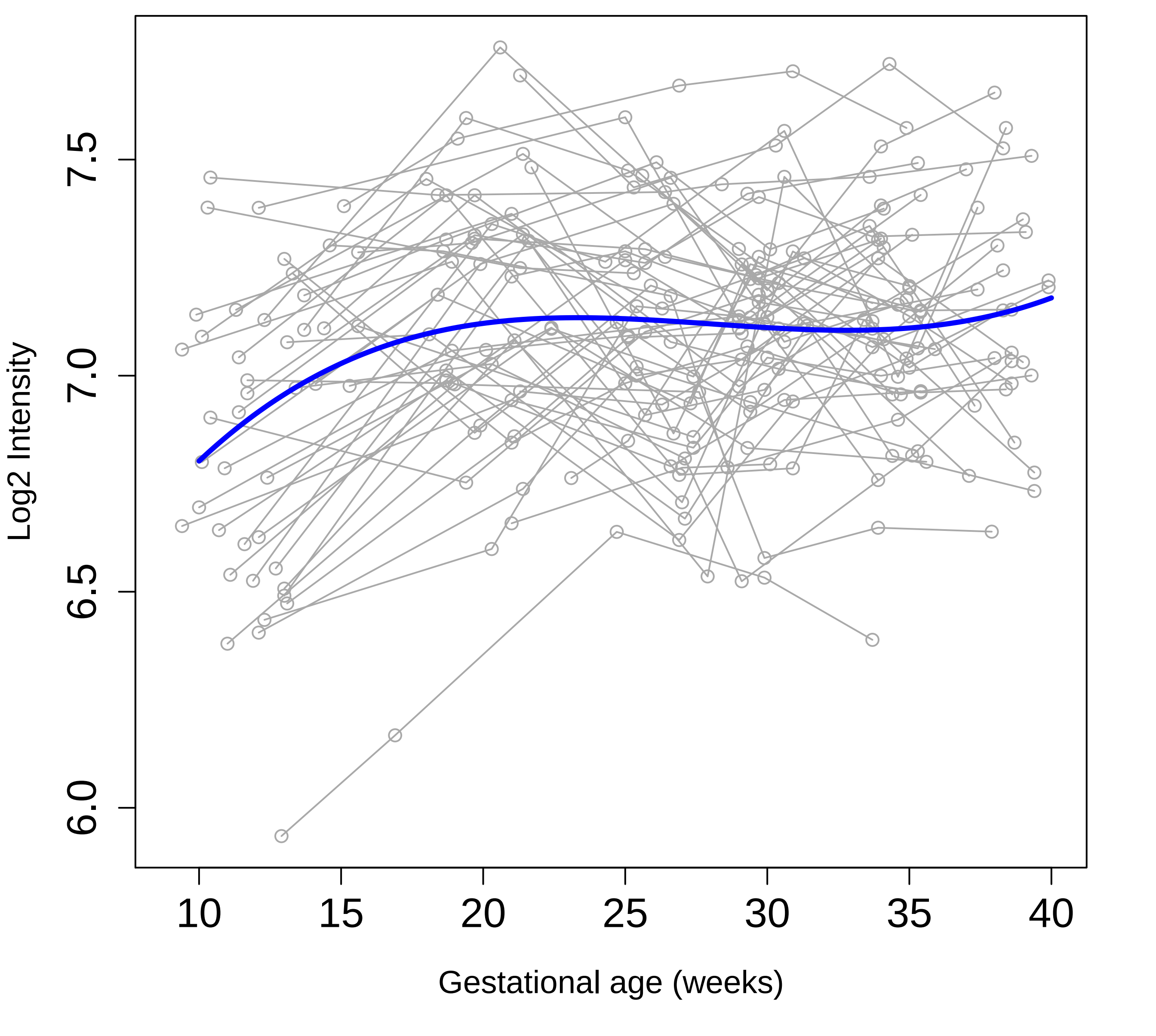

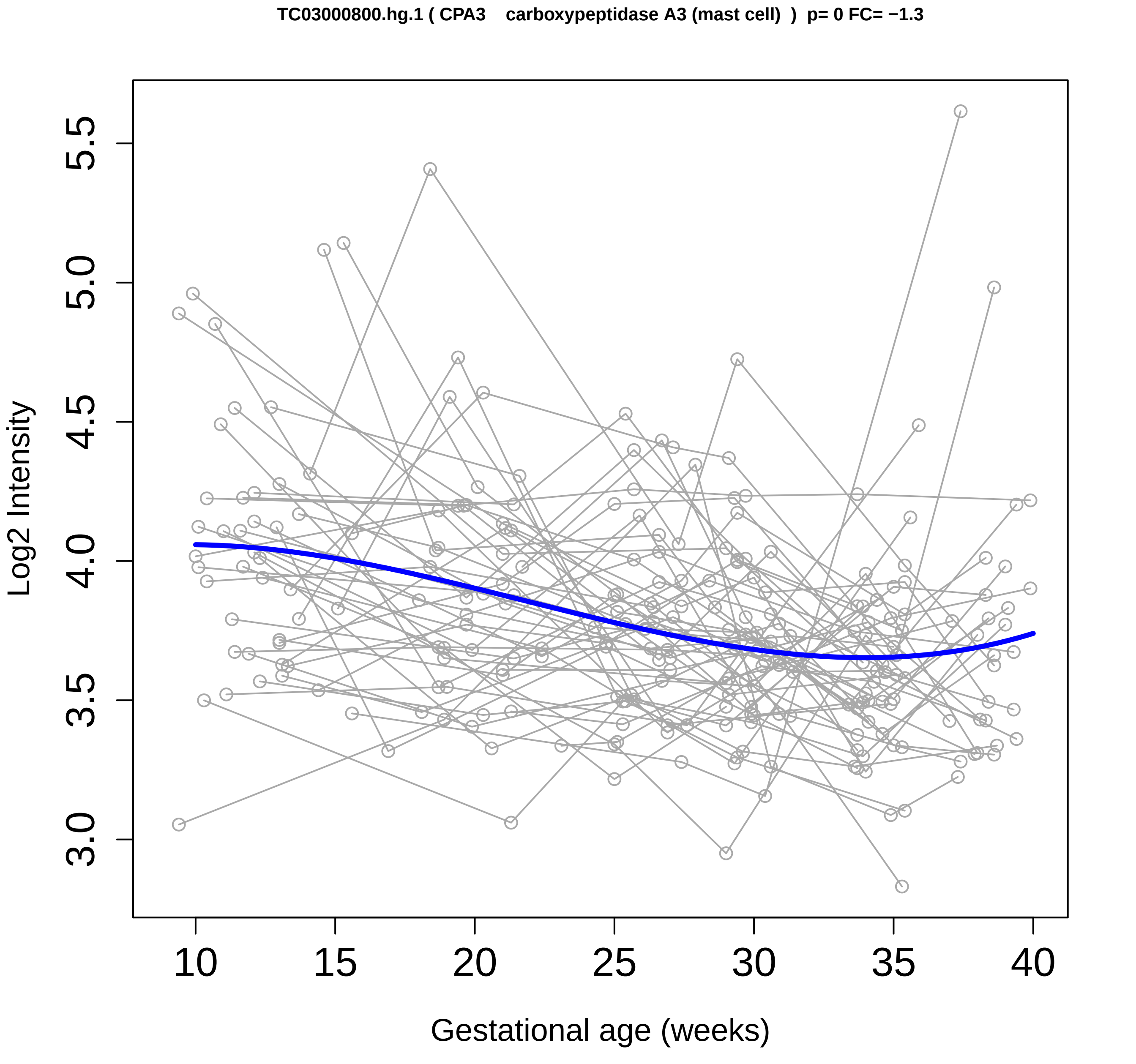

TC03000800.hg.1 (CPA3   carboxypeptidase A3 (mast cell)) p= 0 FC= −1.3
Log2 Intensity
3.0
3.5
4.0
4.5
5.0
5.5
10
15
20
25
30
35
40
Gestational age (weeks)

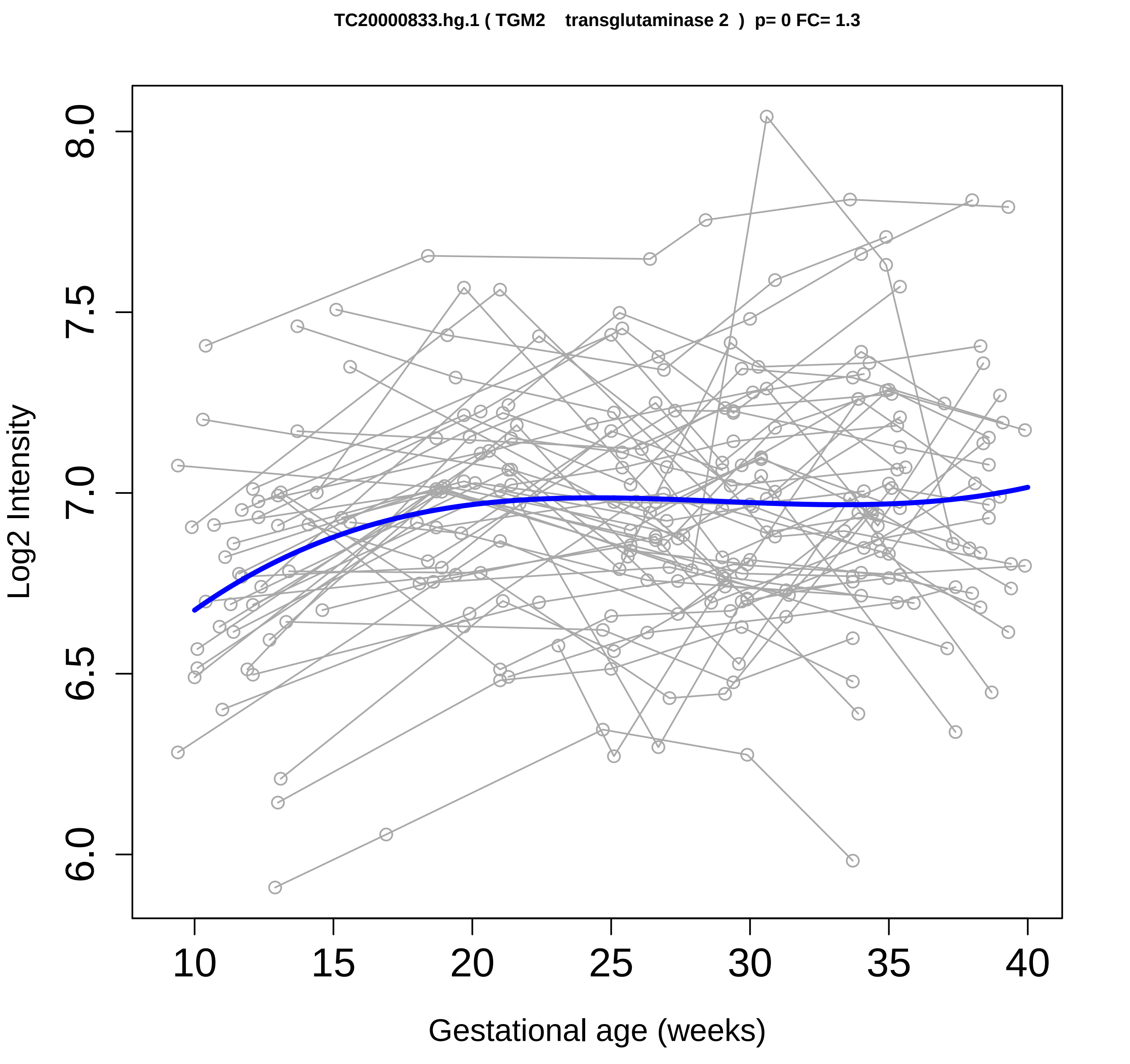

TC20000833.hg.1 (TGM2 transglutaminase 2) p= 0 FC= 1.3
Log2 Intensity
8.0
7.5
7.0
6.5
6.0
10
15
20
25
30
35
40
Gestational age (weeks)

TC14000323.hg.1 (LGALS3   lectin, galactoside−binding, soluble, 3) p= 0 FC= 1.3

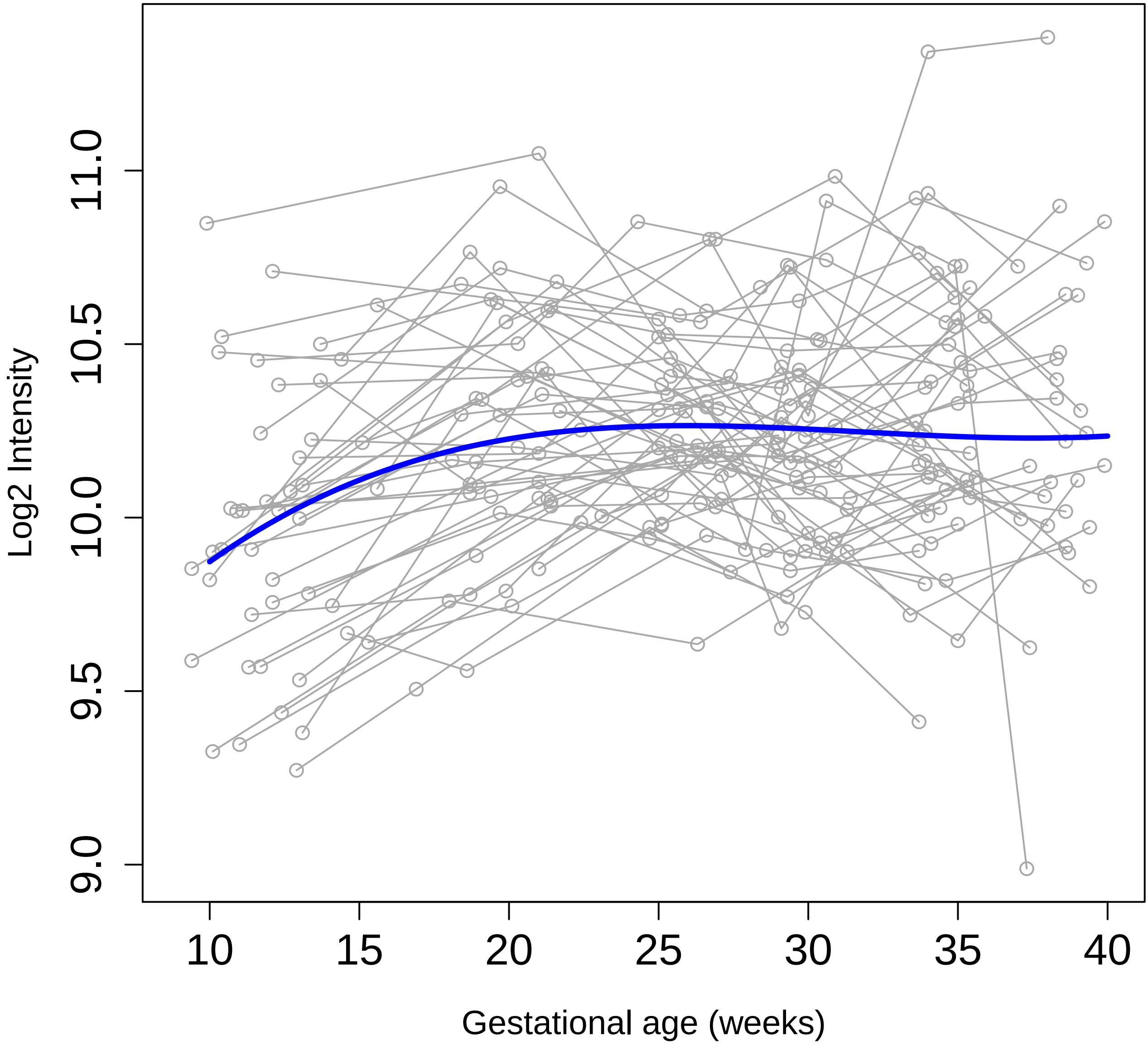

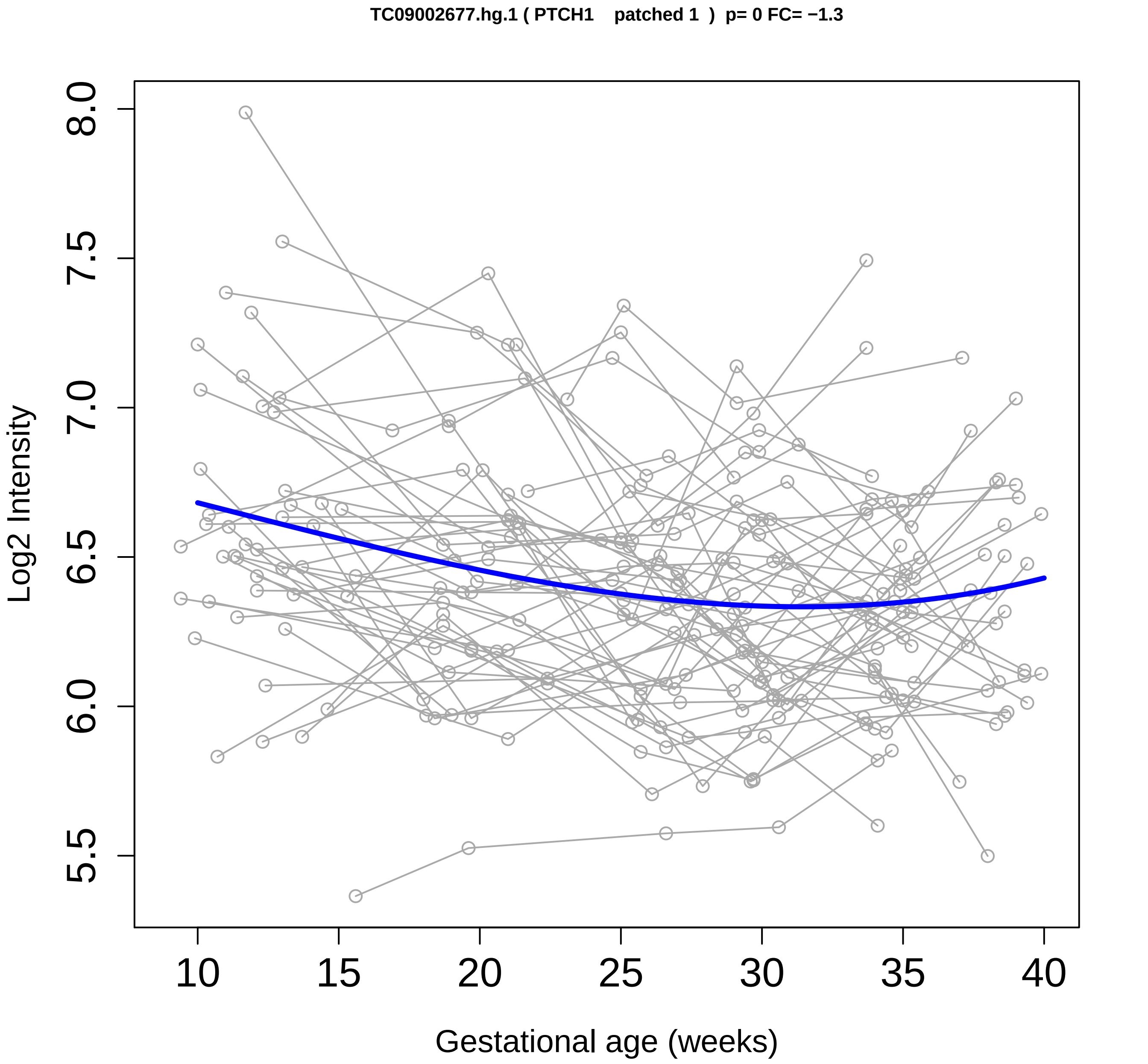
TC09002677.hg.1 (PTCH1 patched 1) p= 0 FC= −1.3
Log2 Intensity
8.0
7.5
7.0
6.5
6.0
5.5
10
15
20
25
30
35
40
Gestational age (weeks)

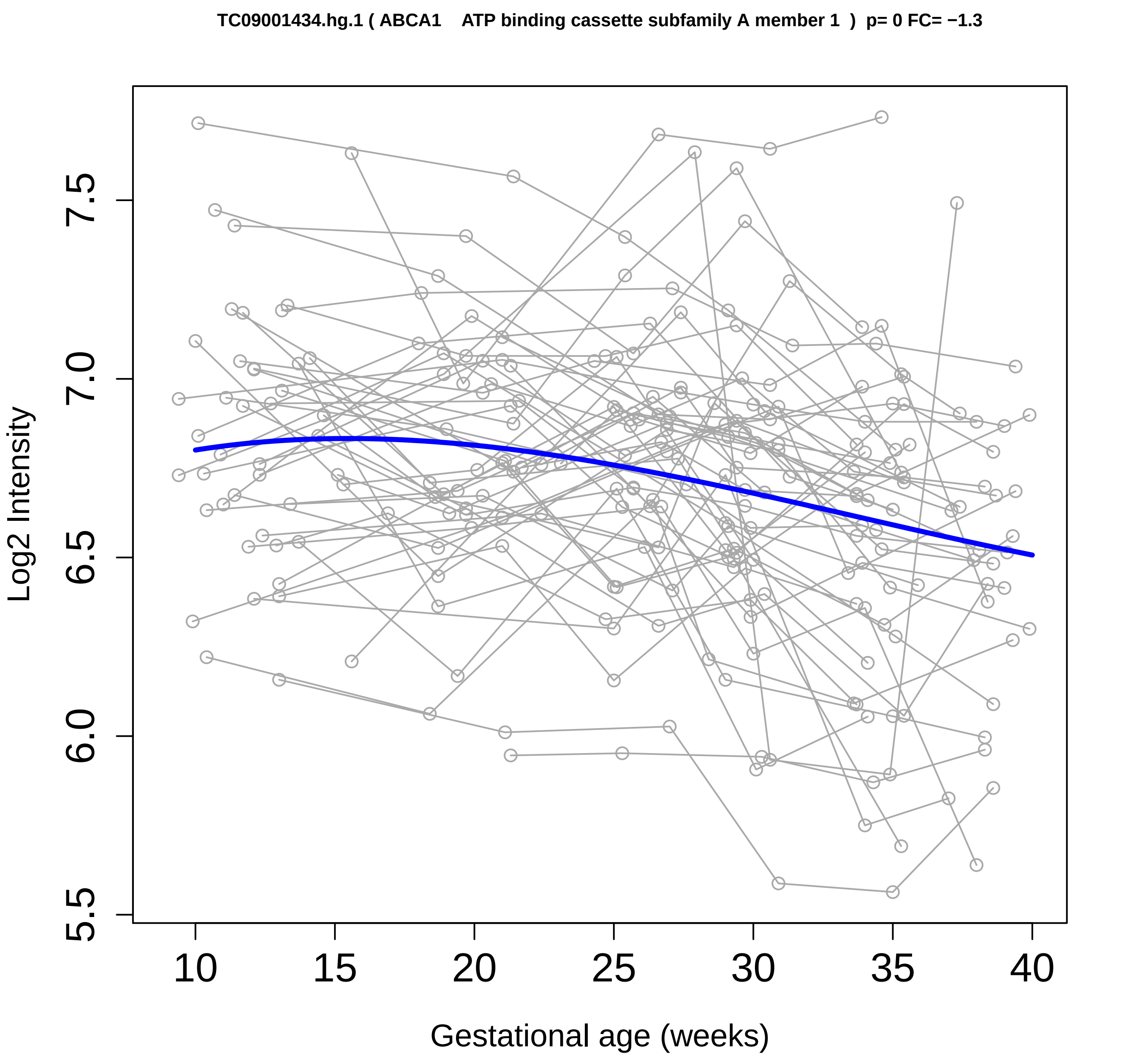

TC09001434.hg.1 (ABCA1 ATP binding cassette subfamily A member 1) p= 0 FC= −1.3
Log2 Intensity
7.5
7.0
6.5
6.0
5.5
10
15
20
25
30
35
40
Gestational age (weeks)

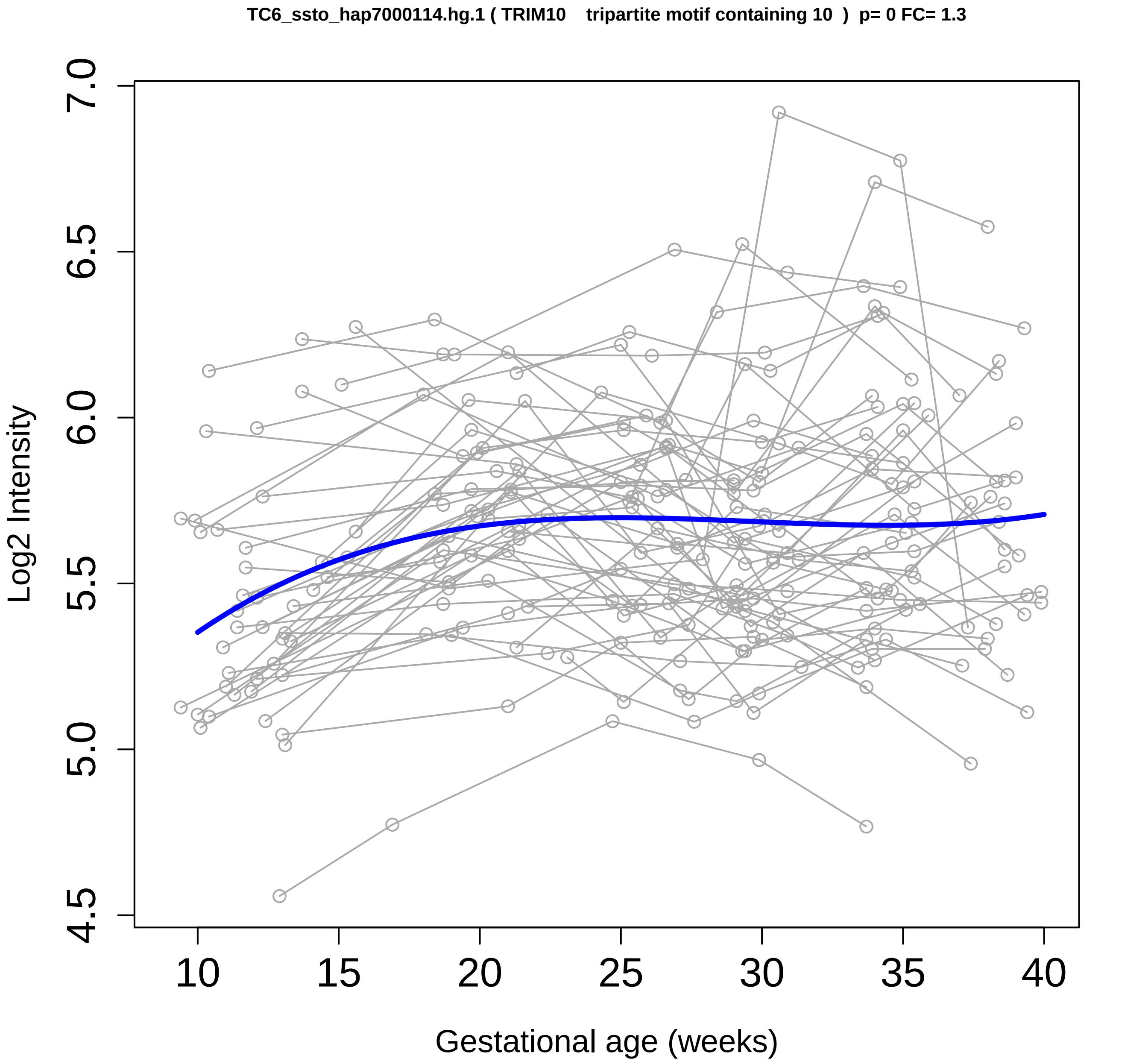

TC6_ssto_hap7000114.hg.1 (TRIM10 tripartite motif containing 10) p= 0 FC= 1.3
Log2 Intensity
4.5
5.0
5.5
6.0
6.5
7.0
10
15
20
25
30
35
40
Gestational age (weeks)

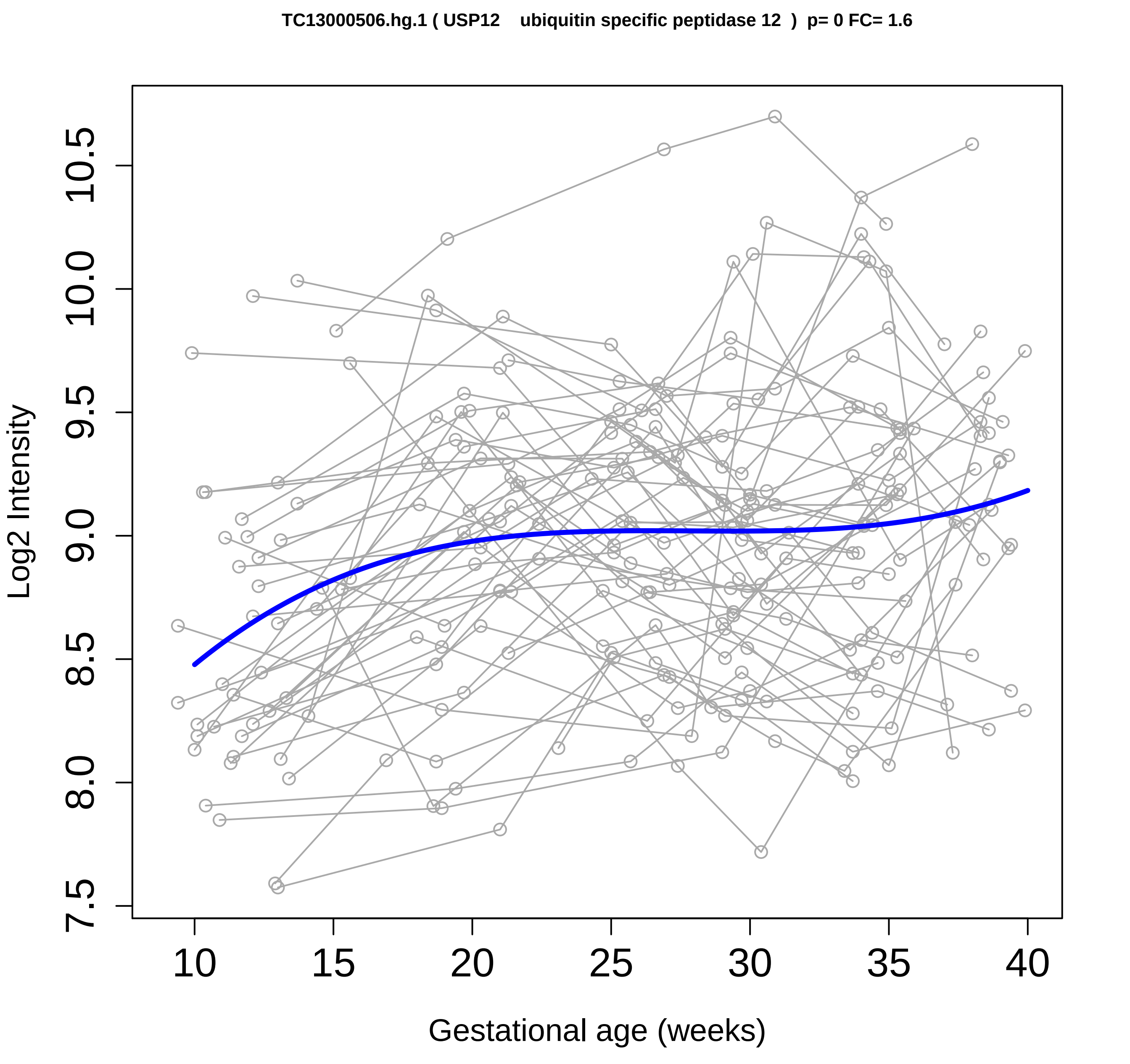

TC13000506.hg.1 (USP12 ubiquitin specific peptidase 12) p= 0 FC= 1.6
Log2 Intensity
7.5
8.0
8.5
9.0
9.5
10.0
10.5
10
15
20
25
30
35
40
Gestational age (weeks)

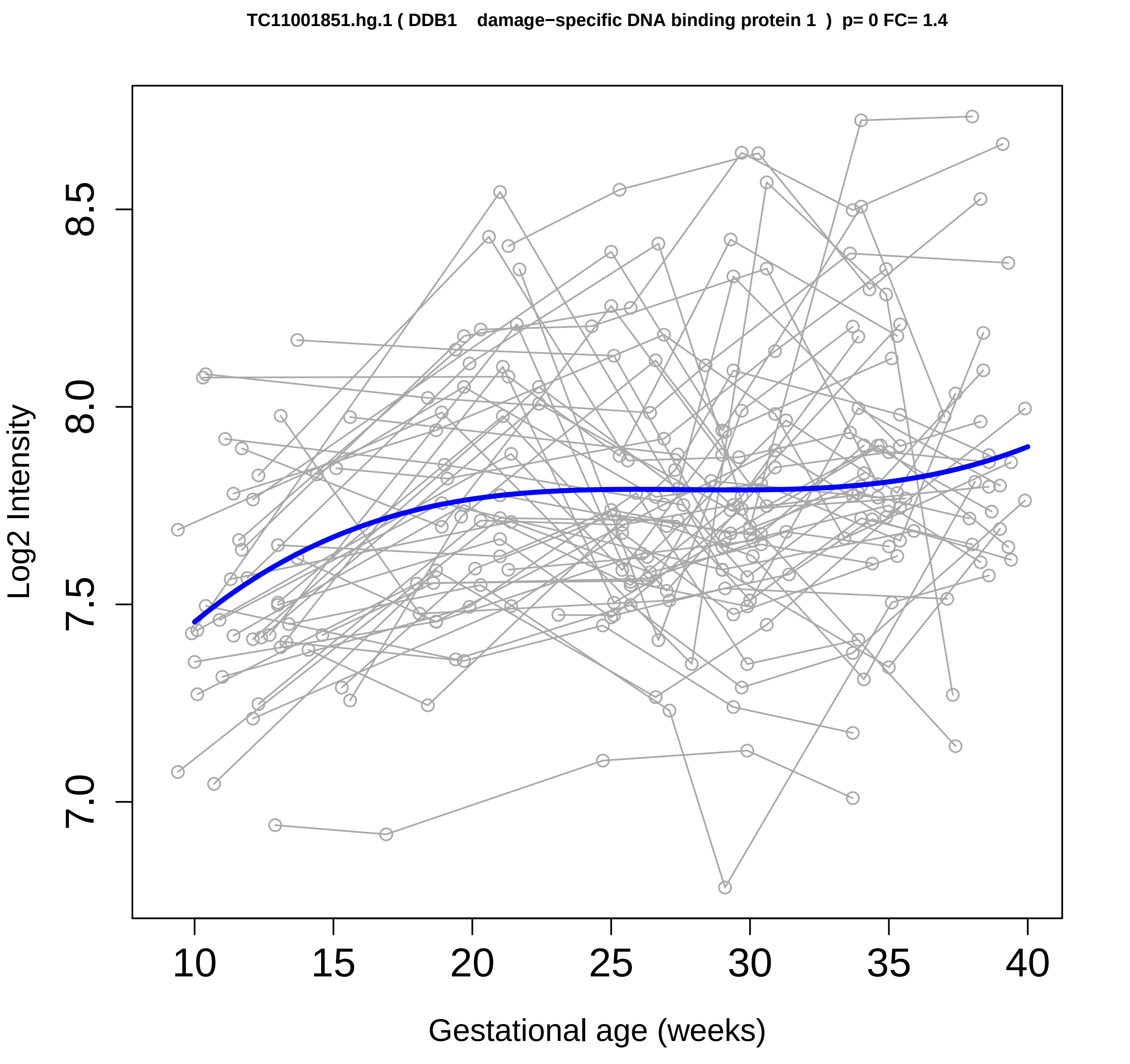

TC11001851.hg.1 (DDB1 damage−specific DNA binding protein 1) p= 0 FC= 1.4
Log2 Intensity
7.0
7.5
8.0
8.5
10
15
20
25
30
35
40
Gestational age (weeks)

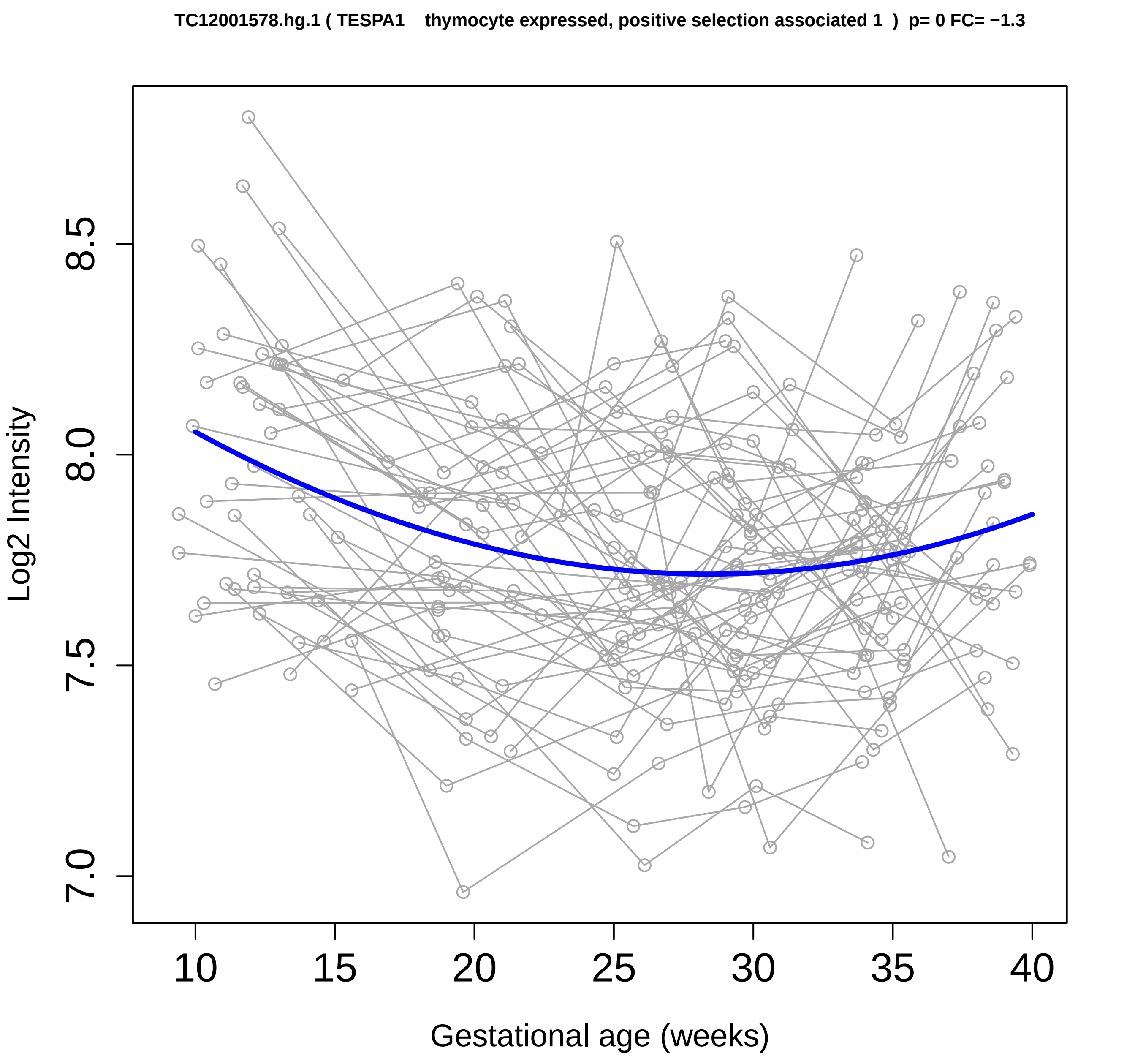
TC12001578.hg.1 (TESPA1 thymocyte expressed, positive selection associated 1) p= 0 FC= −1.3
Log2 Intensity
8.5
8.0
7.5
7.0
10
15
20
25
30
35
40
Gestational age (weeks)

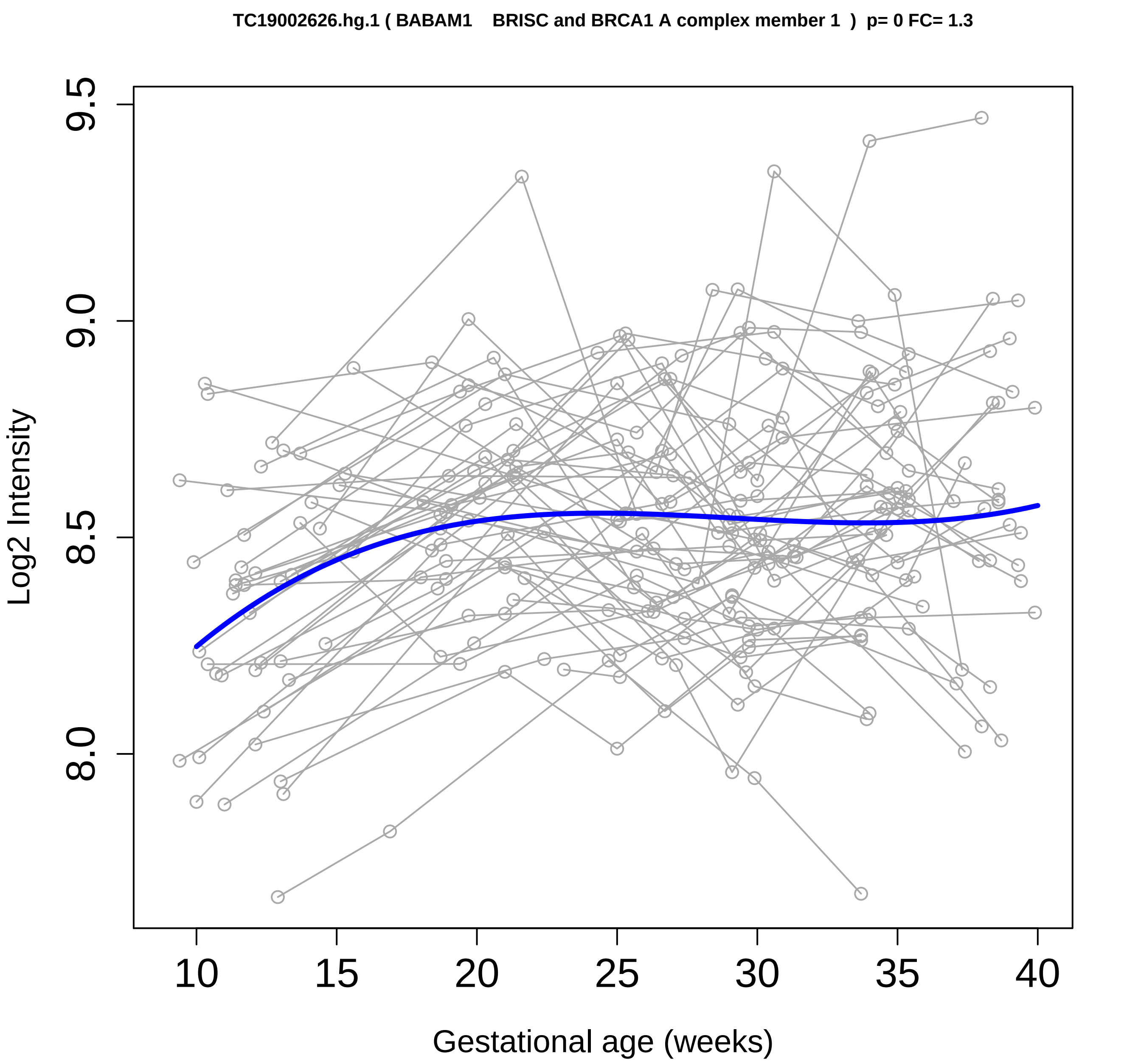

TC19002626.hg.1 (BABAM1 BRISC and BRCA1 A complex member 1) p= 0 FC= 1.3
Log2 Intensity
9.5
9.0
8.5
8.0
10
15
20
25
30
35
40
Gestational age (weeks)

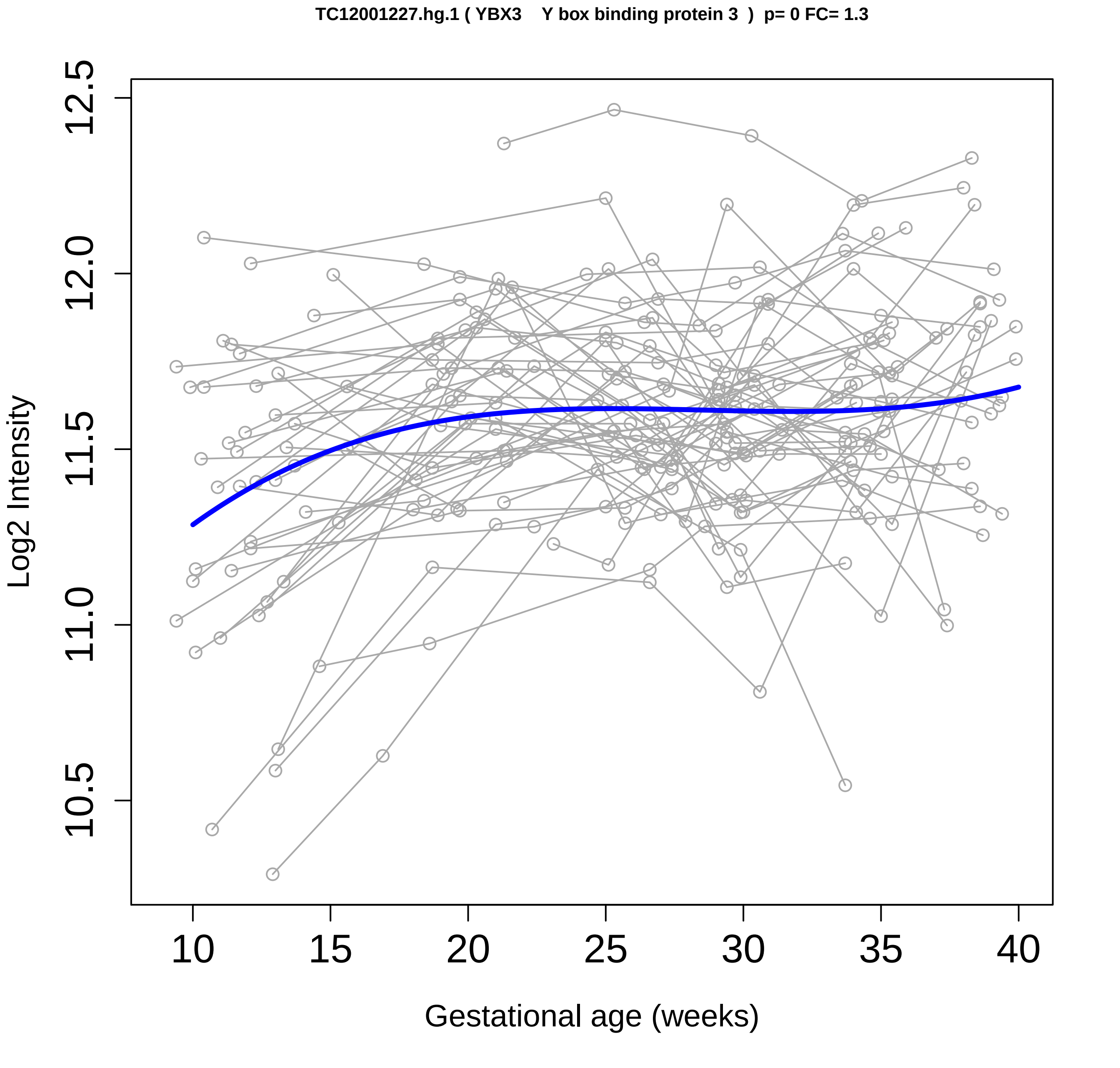
TC12001227.hg.1 (YBX3 Y box binding protein 3) p= 0 FC= 1.3
Log2 Intensity
10.5
11.0
11.5
12.0
12.5
10
15
20
25
30
35
40
Gestational age (weeks)

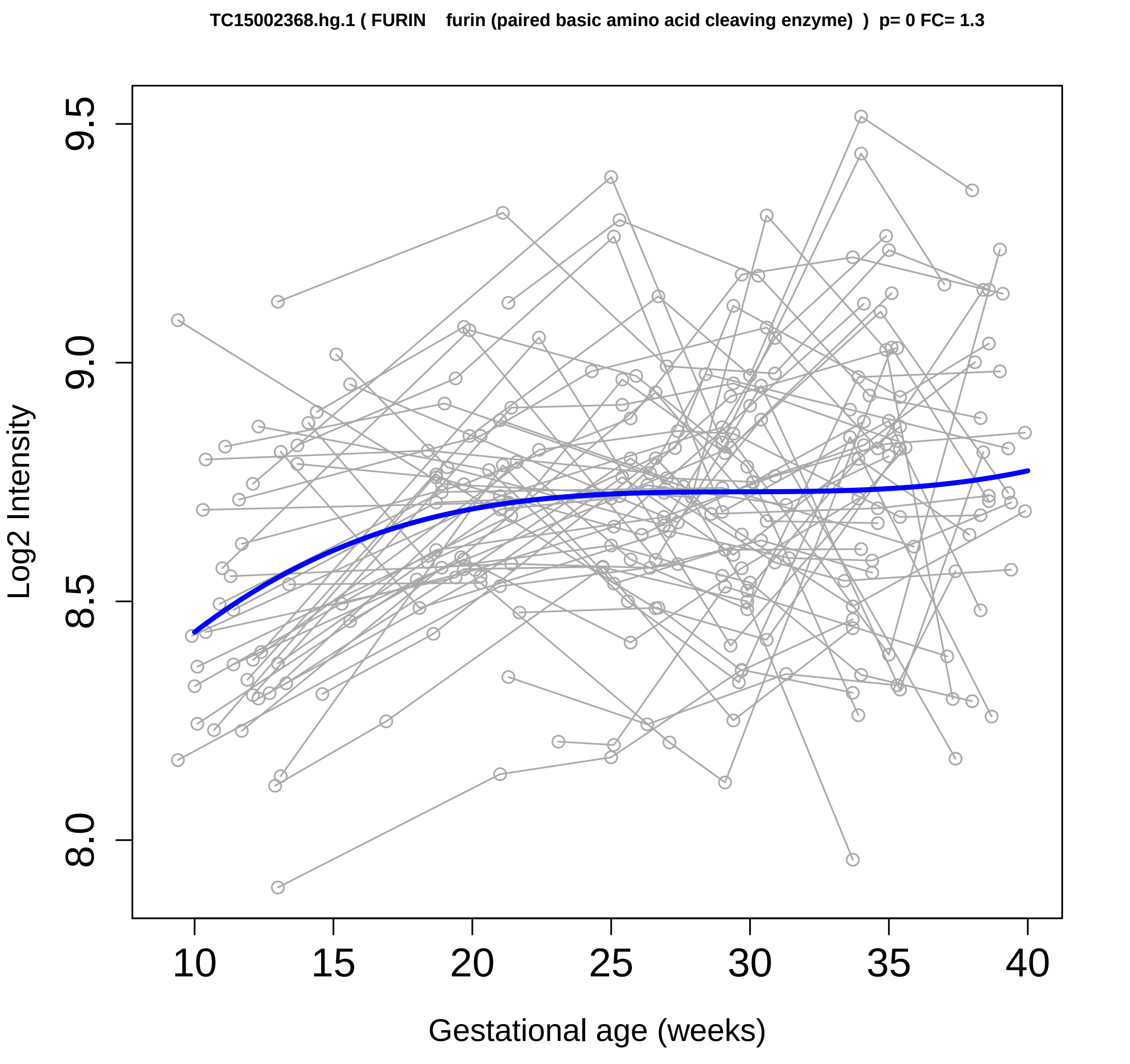

TC15002368.hg.1 (FURIN furin (paired basic amino acid cleaving enzyme)) p= 0 FC= 1.3
Log2 Intensity
9.5
9.0
8.5
8.0
10
15
20
25
30
35
40
Gestational age (weeks)

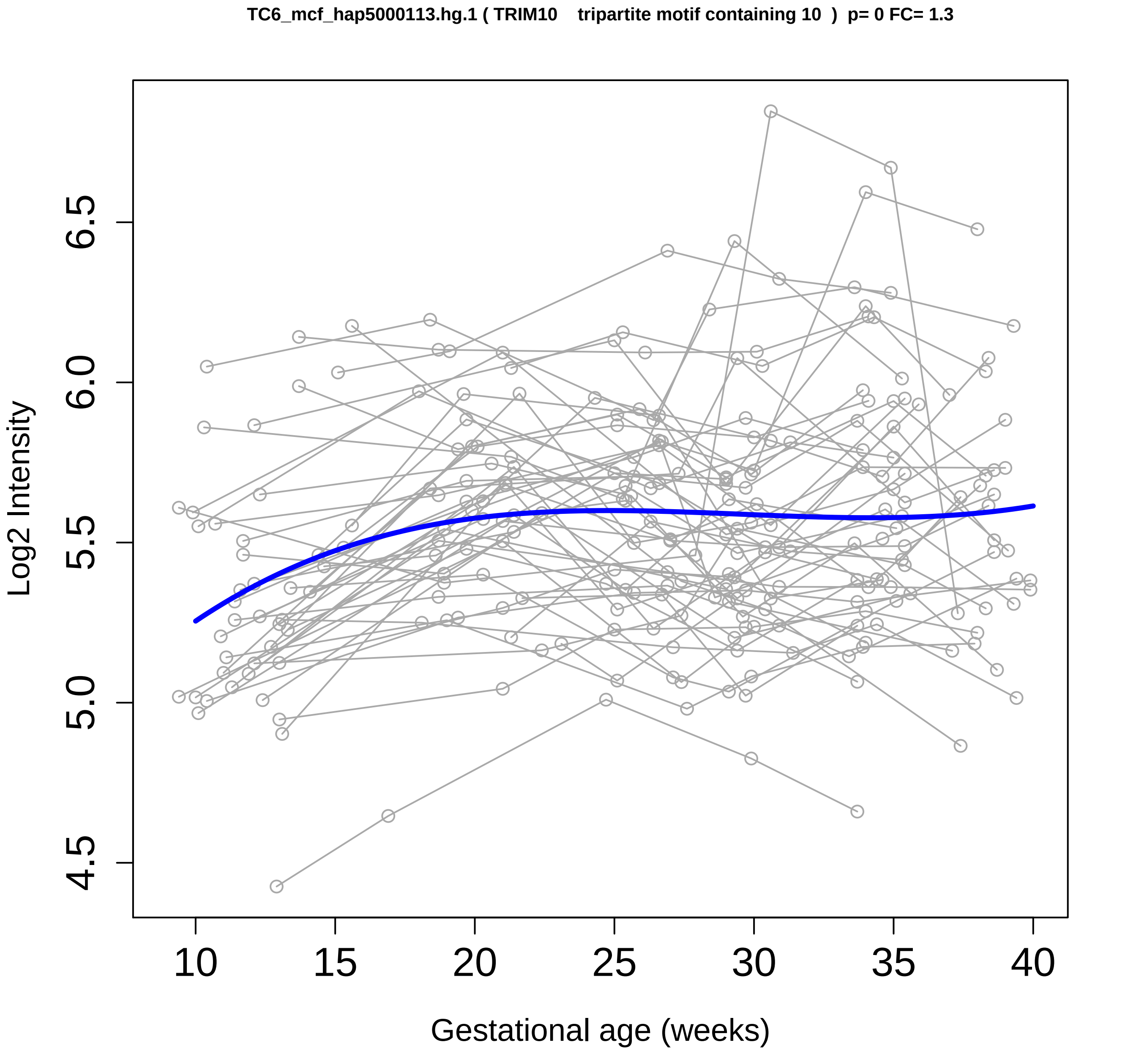
TC6_mcf_hap5000113.hg.1 (TRIM10 tripartite motif containing 10) p= 0 FC= 1.3
Log2 Intensity
4.5
5.0
5.5
6.0
6.5
10
15
20
25
30
35
40
Gestational age (weeks)

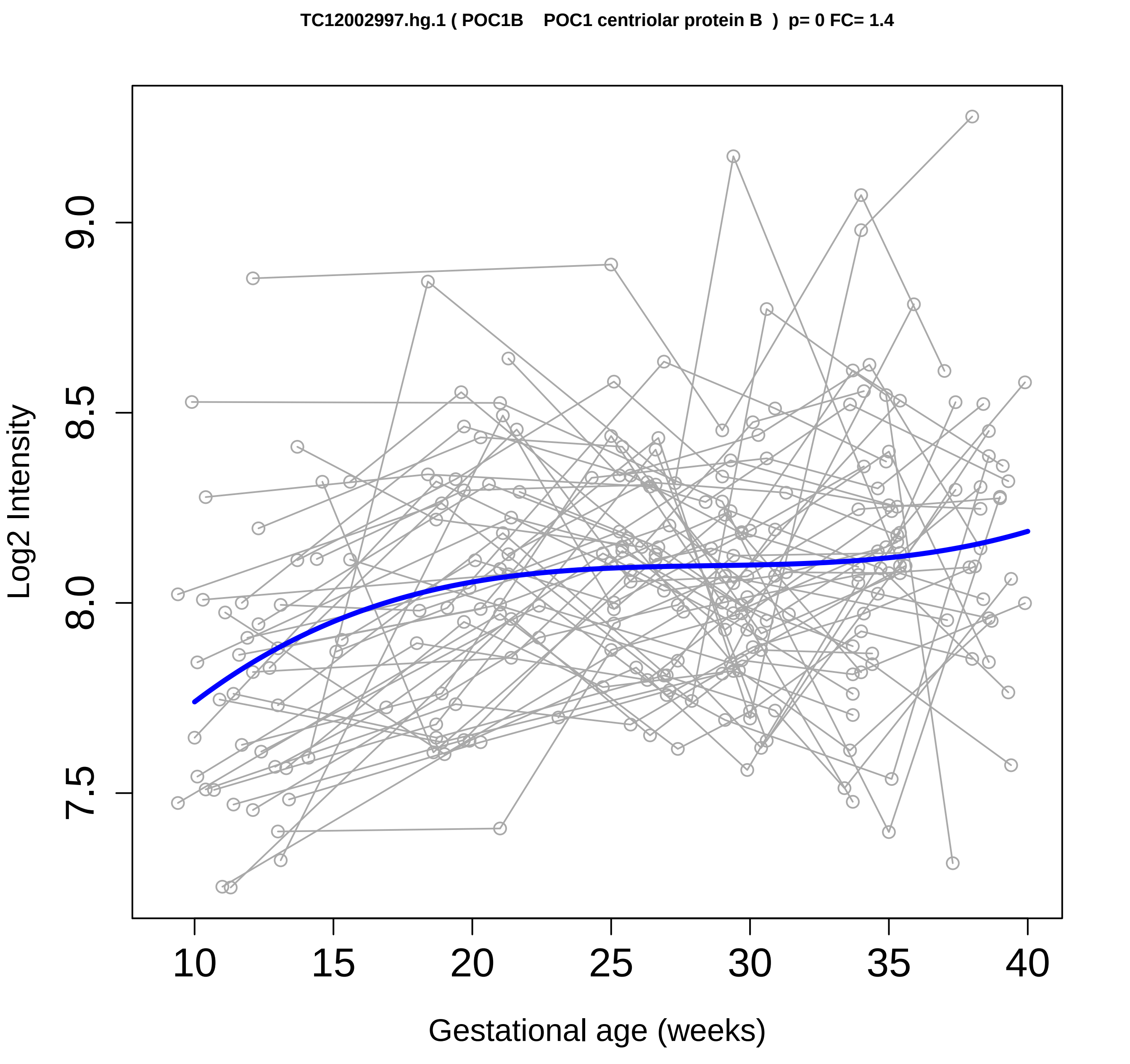

TC12002997.hg.1 (POC1B POC1 centriolar protein B) p= 0 FC= 1.4
Log2 Intensity
9.0
8.5
8.0
7.5
10
15
20
25
30
35
40
Gestational age (weeks)

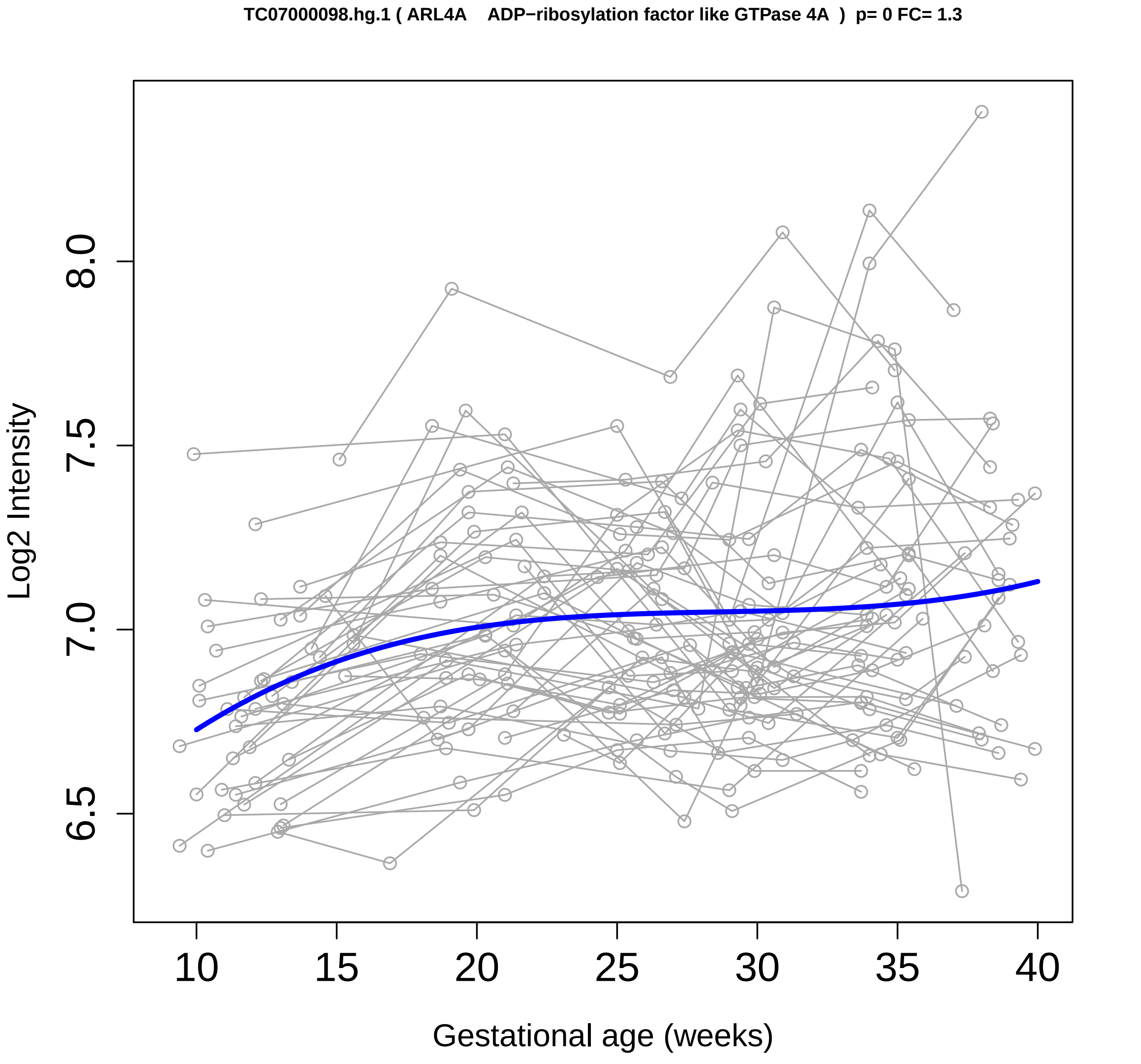
TC07000098.hg.1 (ARL4A ADP−ribosylation factor like GTPase 4A) p= 0 FC= 1.3
Log2 Intensity
6.5
7.0
7.5
8.0
10
15
20
25
30
35
40
Gestational age (weeks)

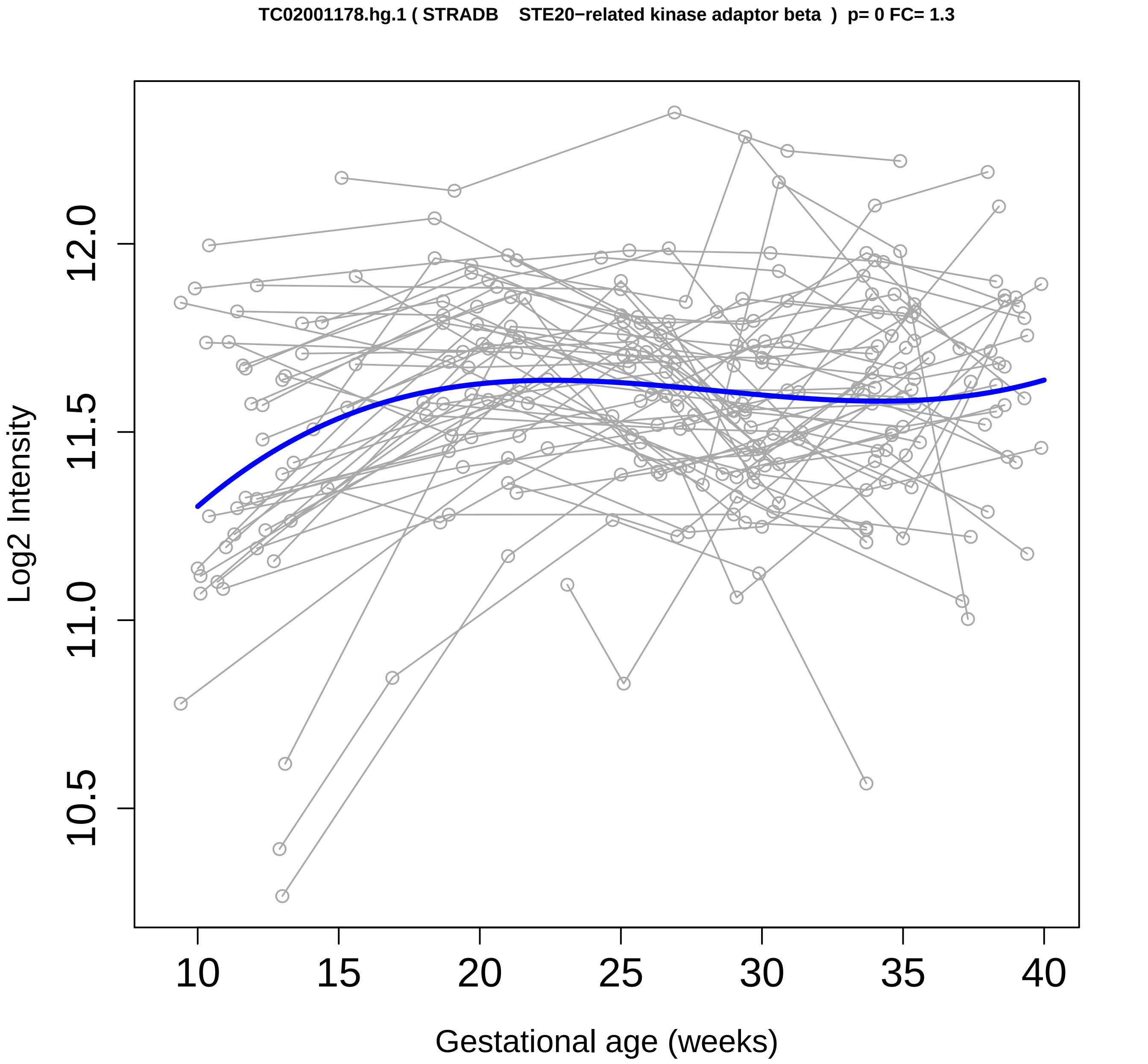
TC02001178.hg.1 (STRADB STE20−related kinase adaptor beta) p= 0 FC= 1.3
Log2 Intensity
10.5
11.0
11.5
12.0
10
15
20
25
30
35
40
Gestational age (weeks)

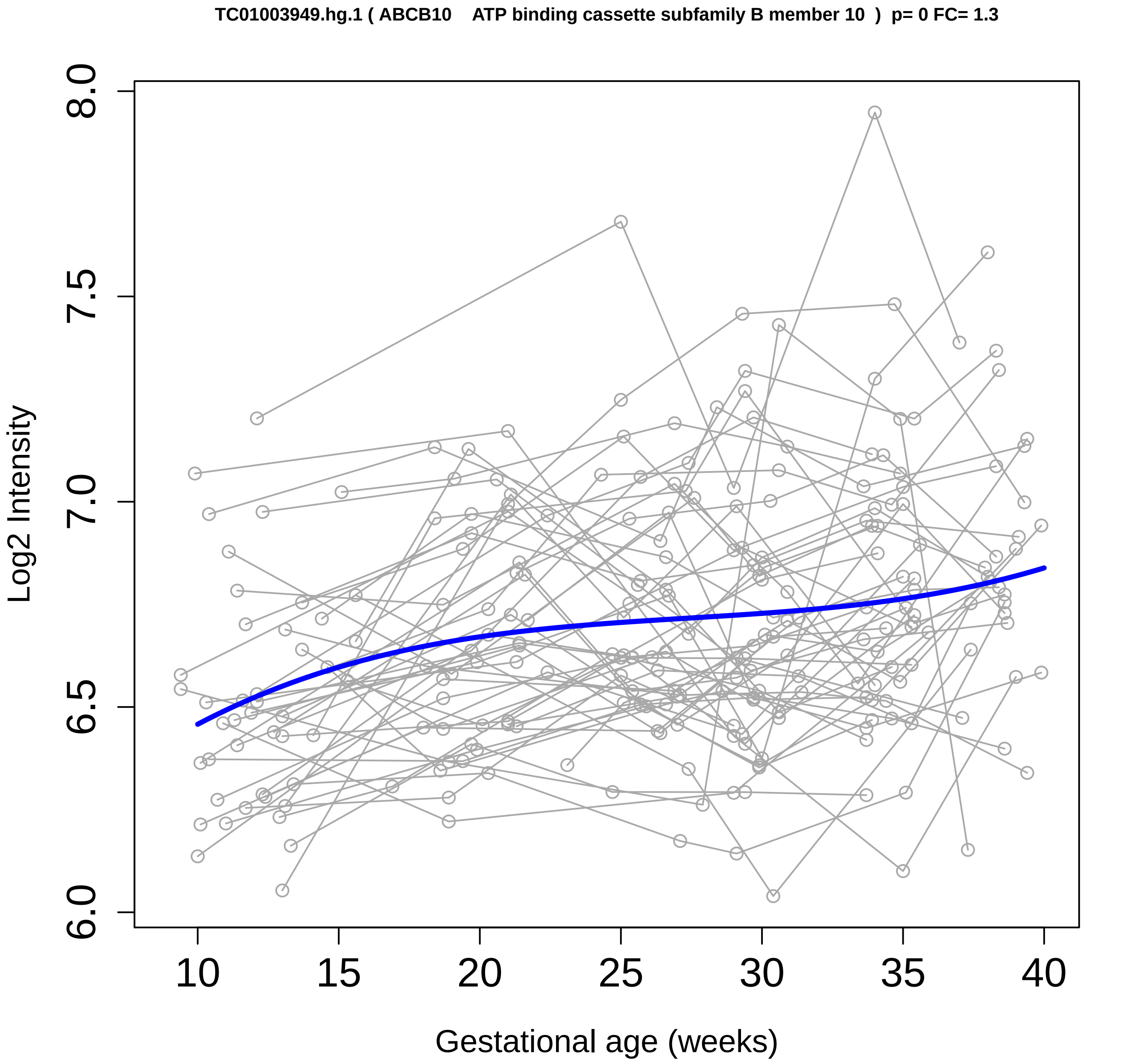
TC01003949.hg.1 (ABCB10 ATP binding cassette subfamily B member 10) p= 0 FC= 1.3
Log2 Intensity
6.0
6.5
7.0
7.5
8.0
10
15
20
25
30
35
40
Gestational age (weeks)

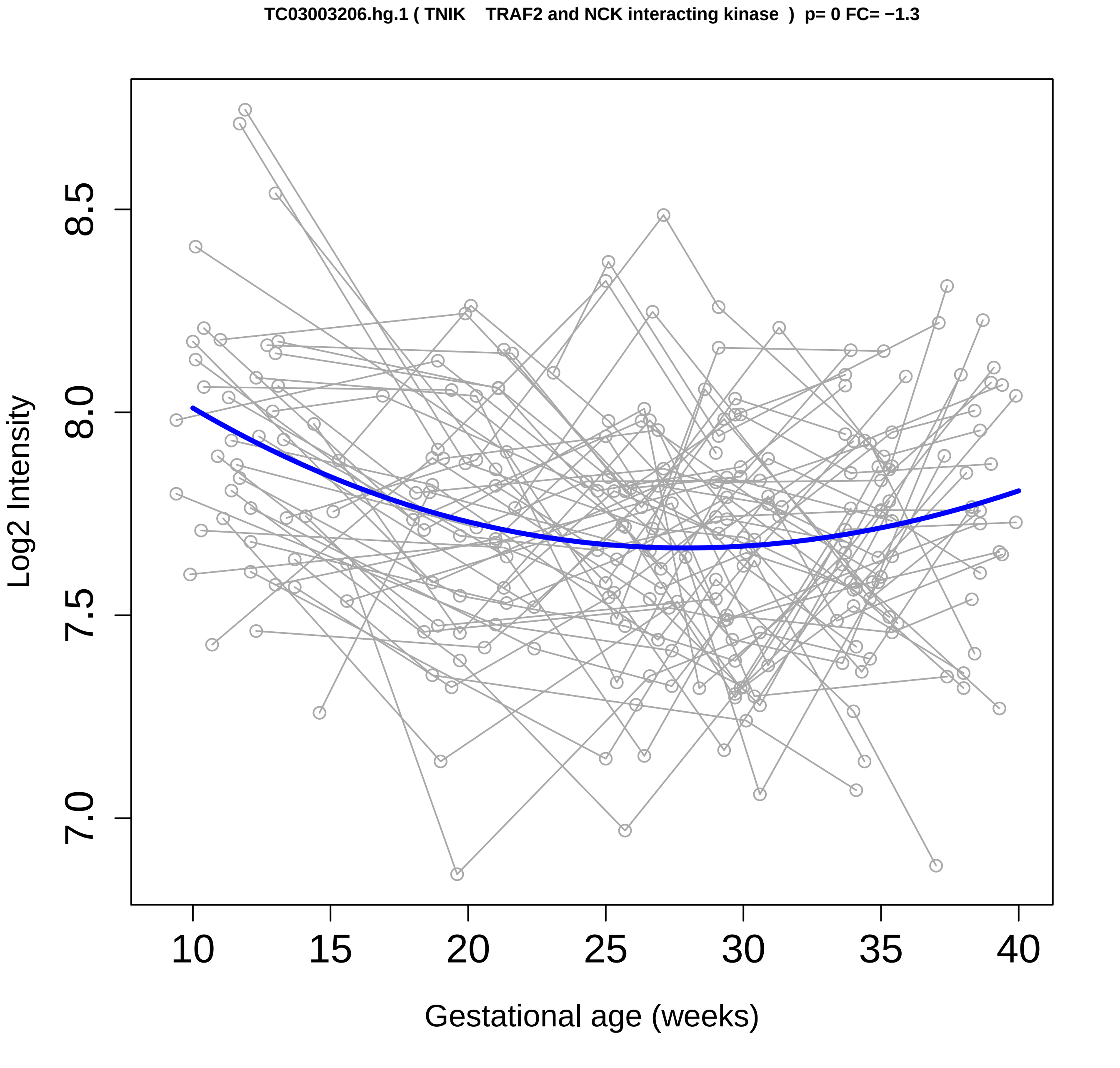

TC03003206.hg.1 (TNIK TRAF2 and NCK interacting kinase) p= 0 FC= −1.3
Log2 Intensity
8.5
8.0
7.5
7.0
10
15
20
25
30
35
40
Gestational age (weeks)

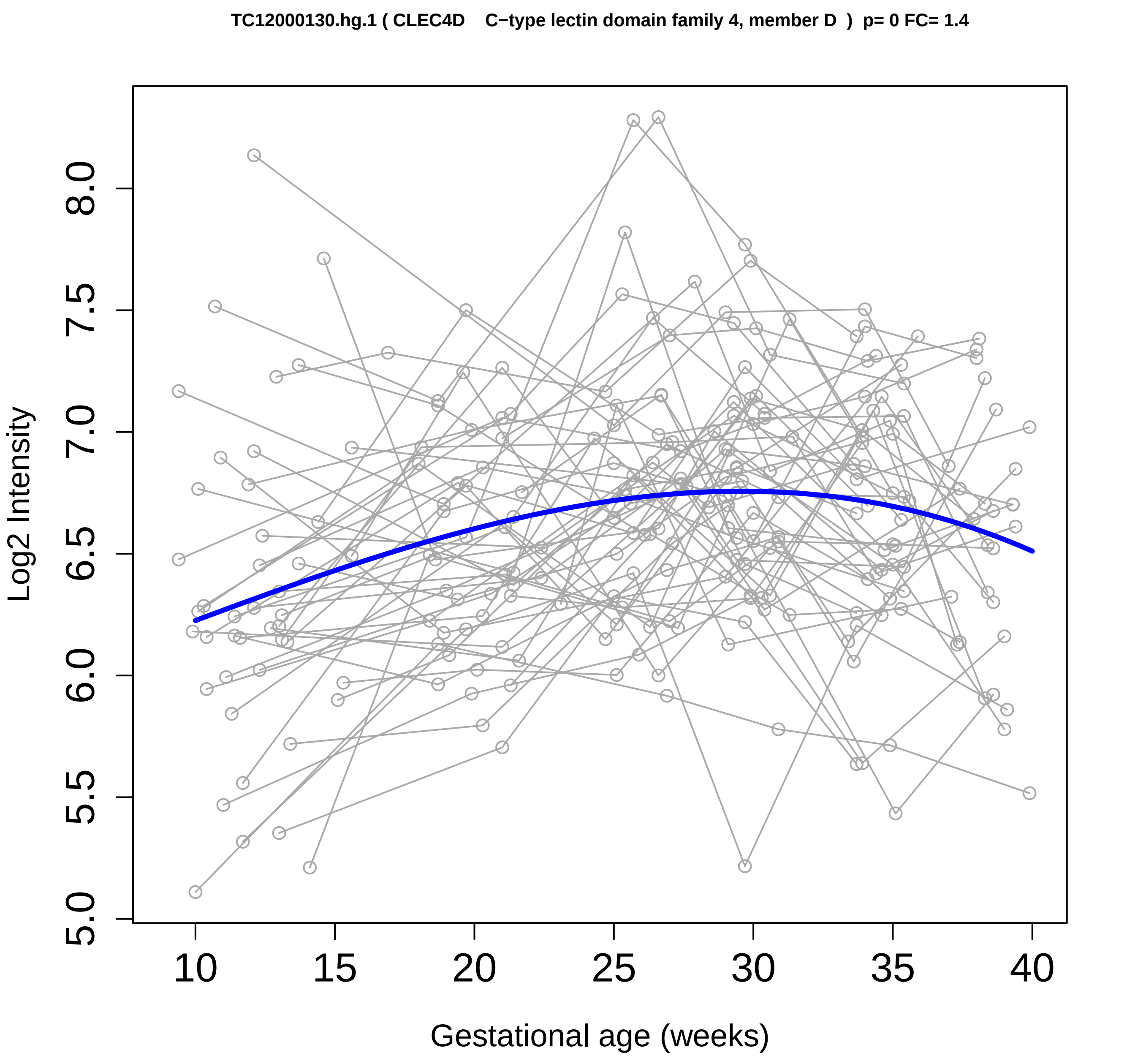
TC12000130.hg.1 (CLEC4D C−type lectin domain family 4, member D) p= 0 FC= 1.4
Log2 Intensity
5.0
5.5
6.0
6.5
7.0
7.5
8.0
10
15
20
25
30
35
40
Gestational age (weeks)

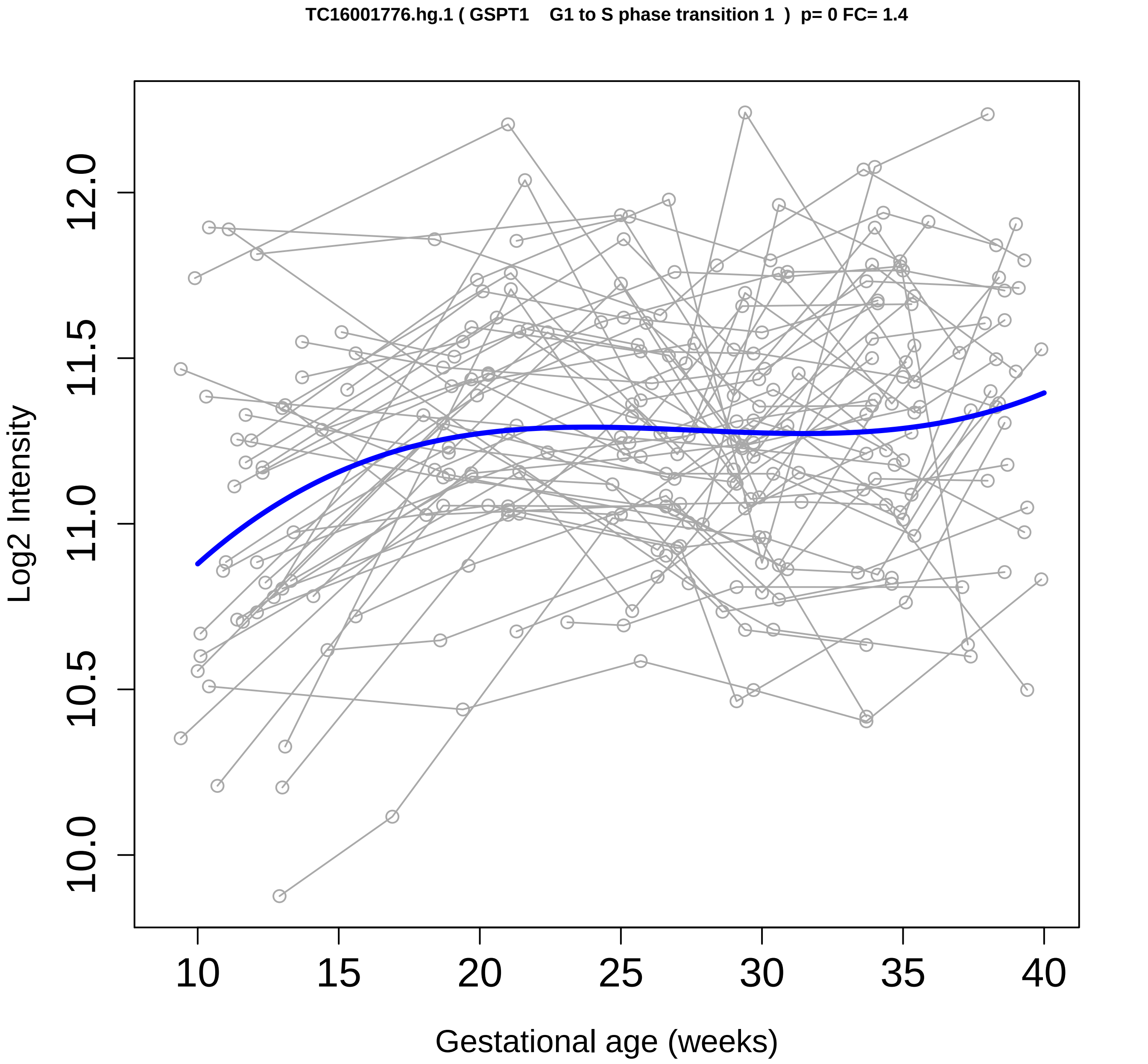
TC16001776.hg.1 (GSPT1 G1 to S phase transition 1) p= 0 FC= 1.4
Log2 Intensity
10.0
10.5
11.0
11.5
12.0
10
15
20
25
30
35
40
Gestational age (weeks)

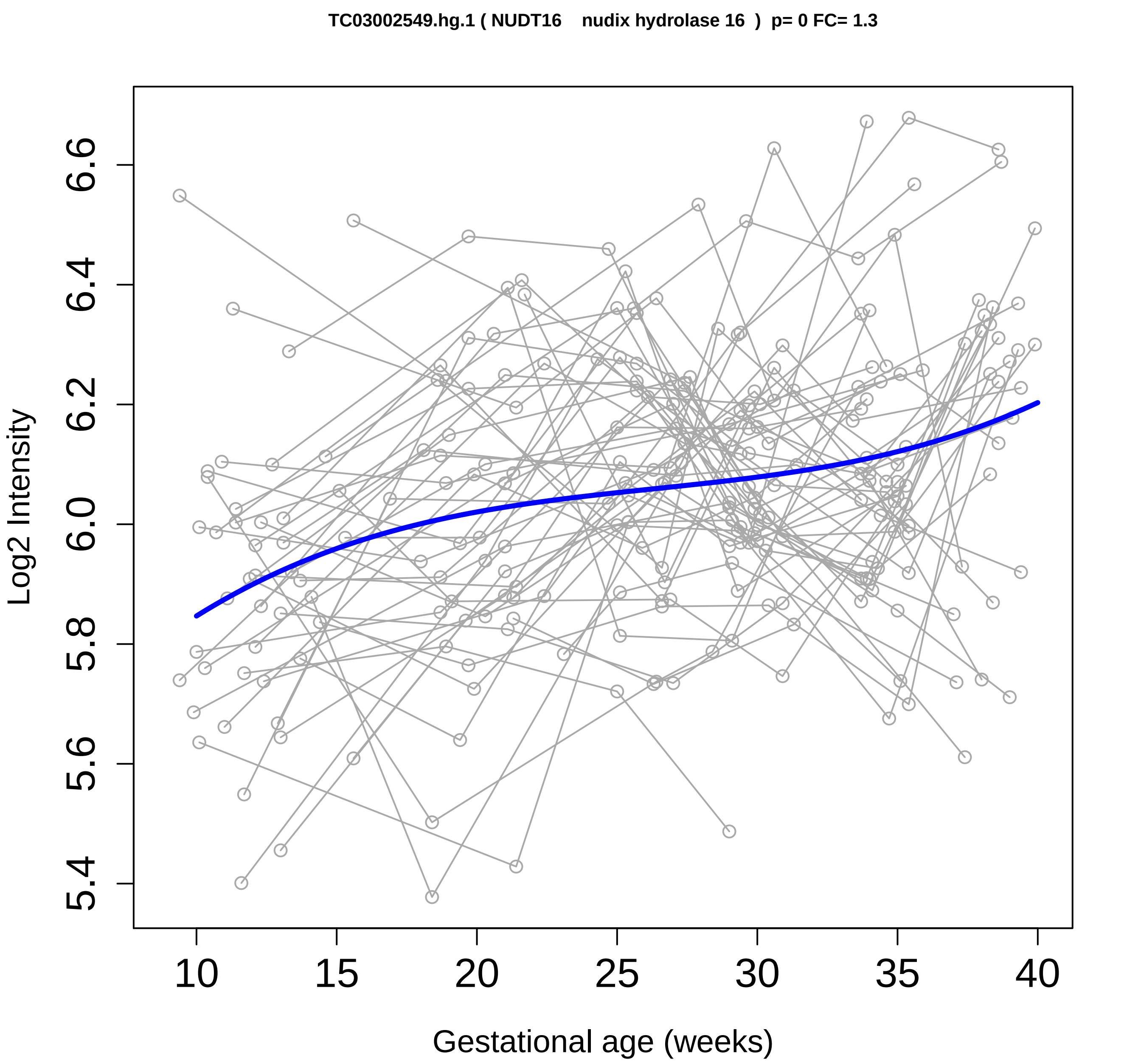

TC03002549.hg.1 (NUDT16 nudix hydrolase 16) p= 0 FC= 1.3
Log2 Intensity
5.4
5.6
5.8
6.0
6.2
6.4
6.6
10
15
20
25
30
35
40
Gestational age (weeks)

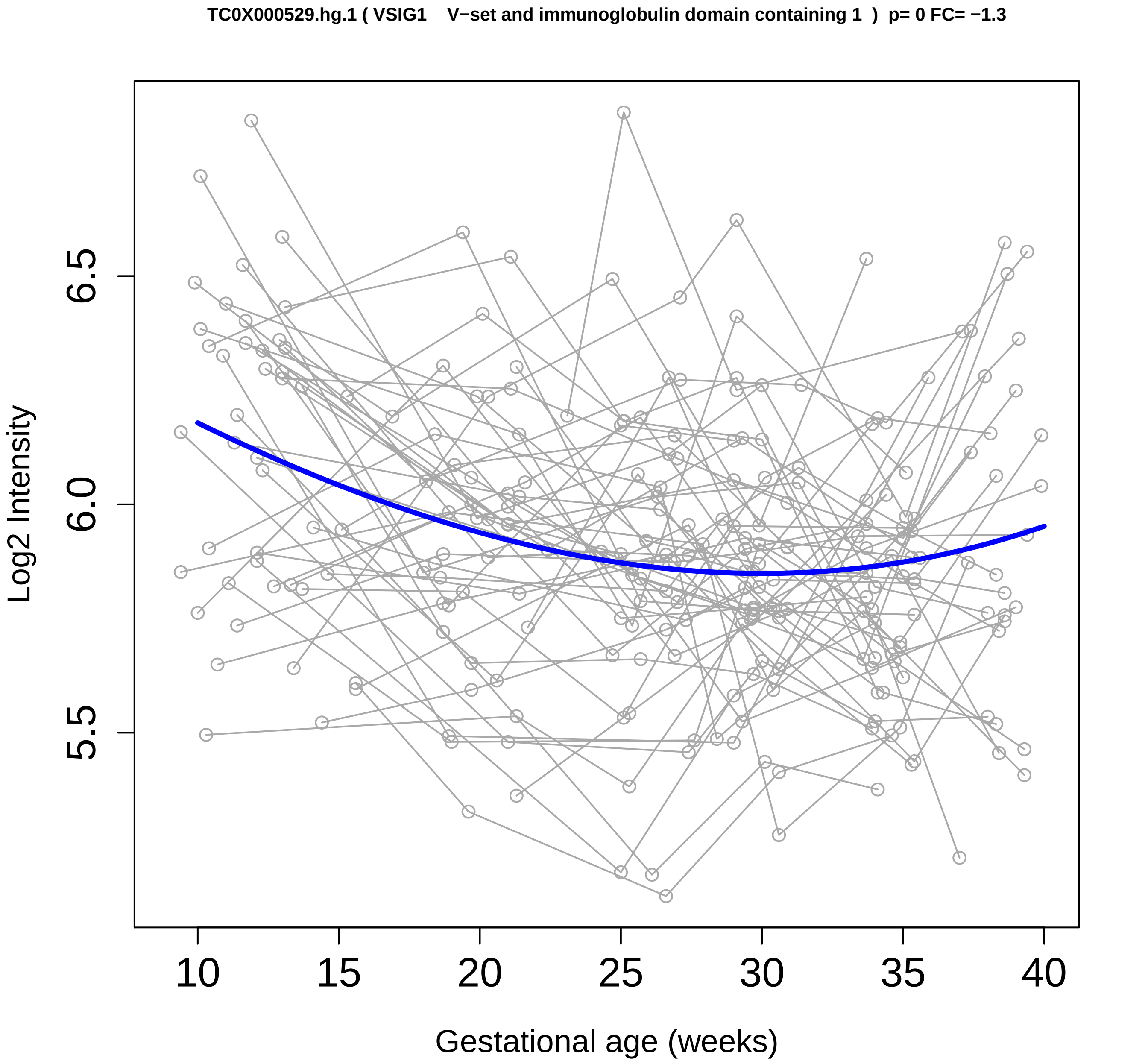
TC0X000529.hg.1 (VSIG1 V−set and immunoglobulin domain containing 1) p= 0 FC= −1.3
Log2 Intensity
5.5
6.0
6.5
10
15
20
25
30
35
40
Gestational age (weeks)

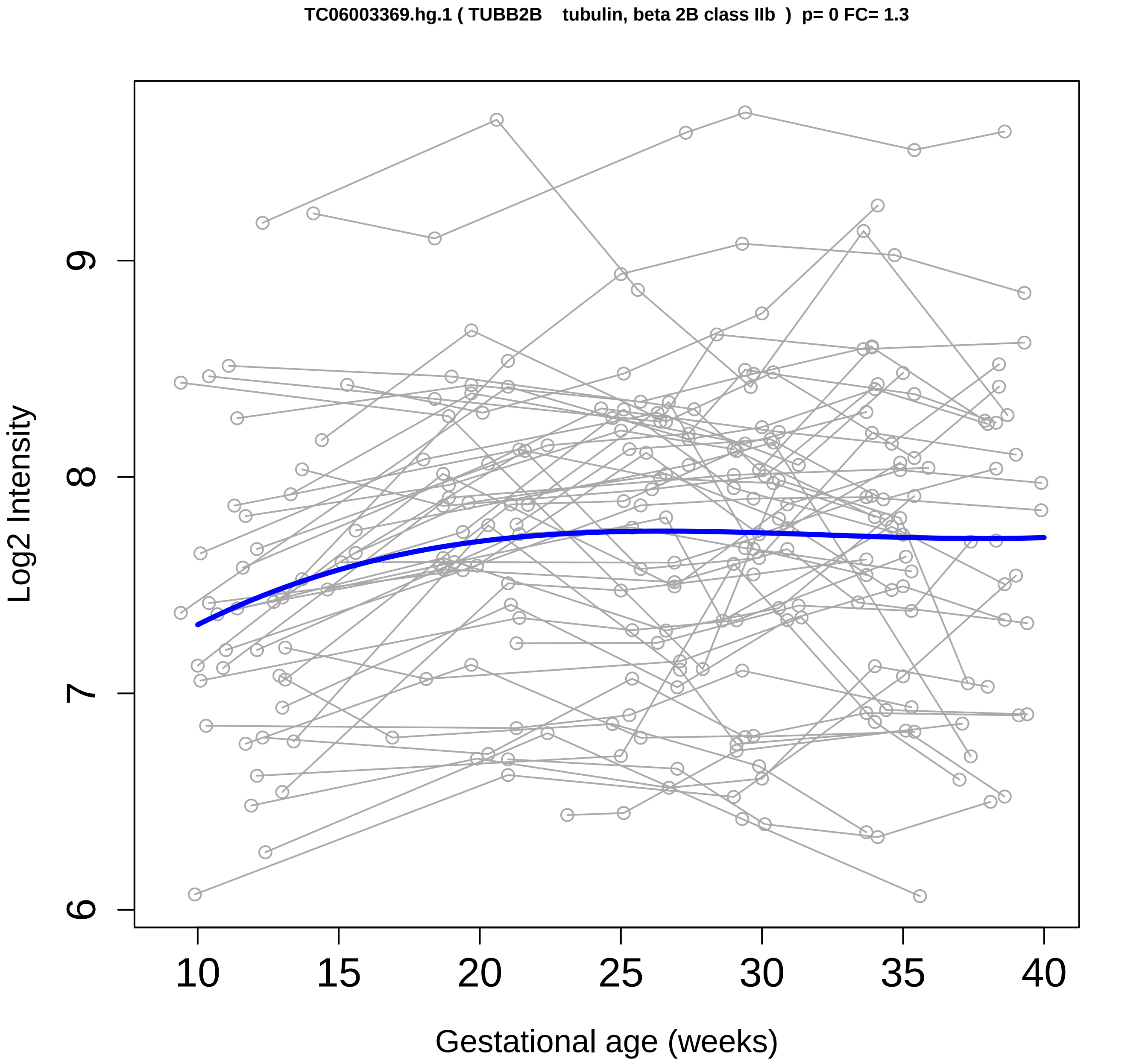

TC06003369.hg.1 (TUBB2B tubulin, beta 2B class IIb) p= 0 FC= 1.3
Log2 Intensity
6
7
8
9
10
15
20
25
30
35
40
Gestational age (weeks)

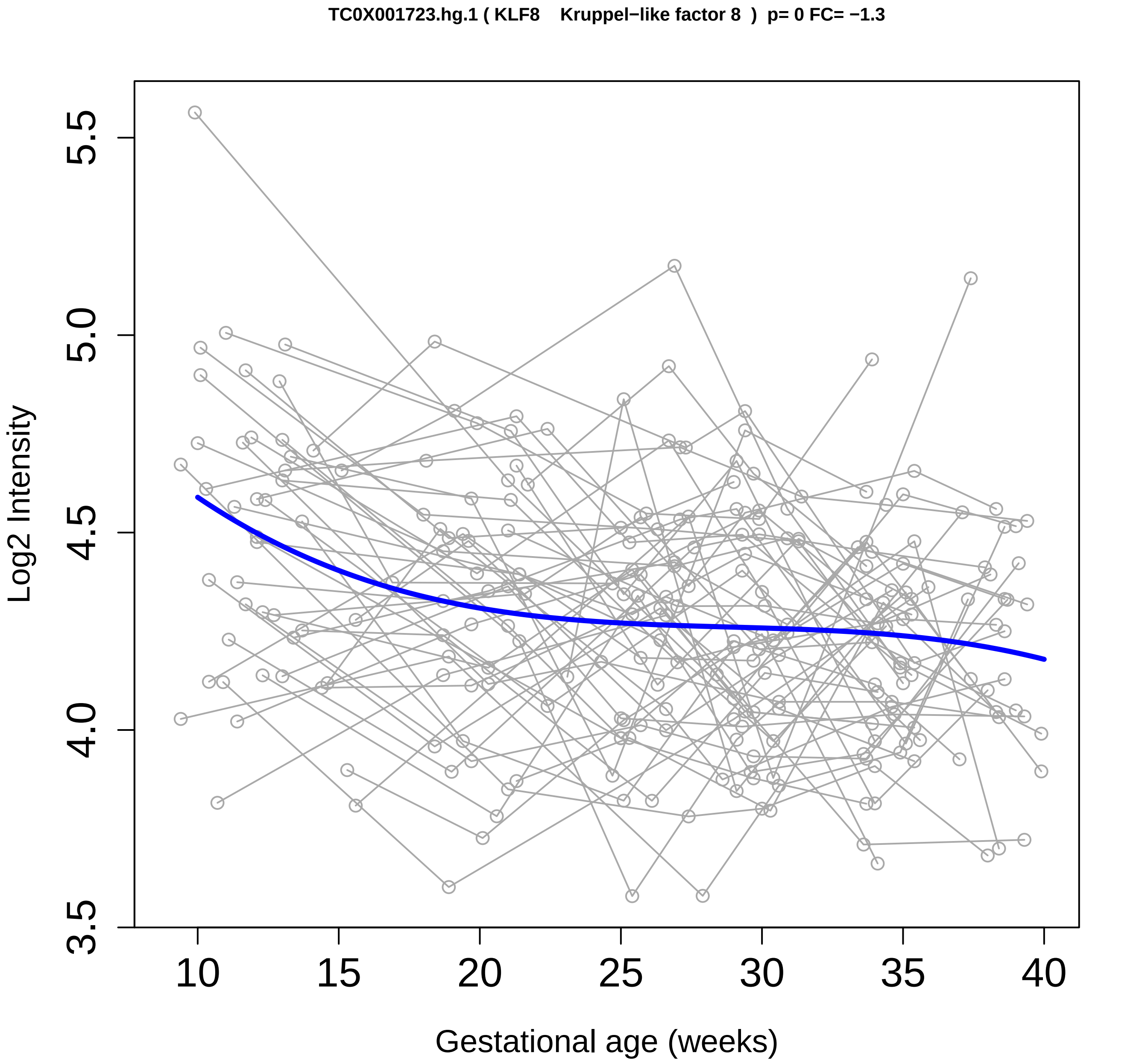

TC0X001723.hg.1 (KLF8 Kruppel−like factor 8) p= 0 FC= −1.3
Log2 Intensity
3.5
4.0
4.5
5.0
5.5
10
15
20
25
30
35
40
Gestational age (weeks)

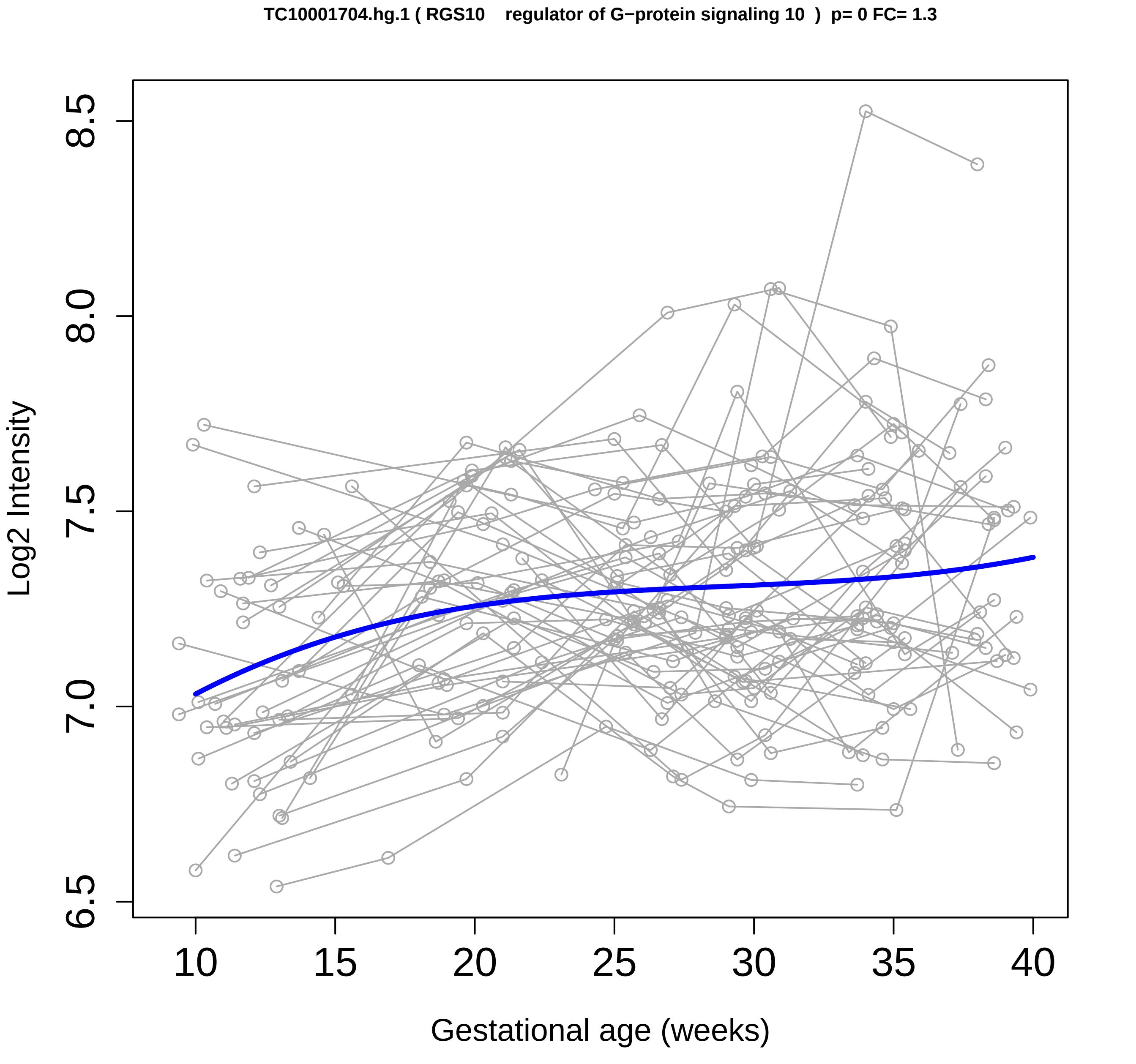

TC10001704.hg.1 (RGS10 regulator of G−protein signaling 10) p= 0 FC= 1.3
Log2 Intensity
6.5
7.0
7.5
8.0
8.5
10
15
20
25
30
35
40
Gestational age (weeks)

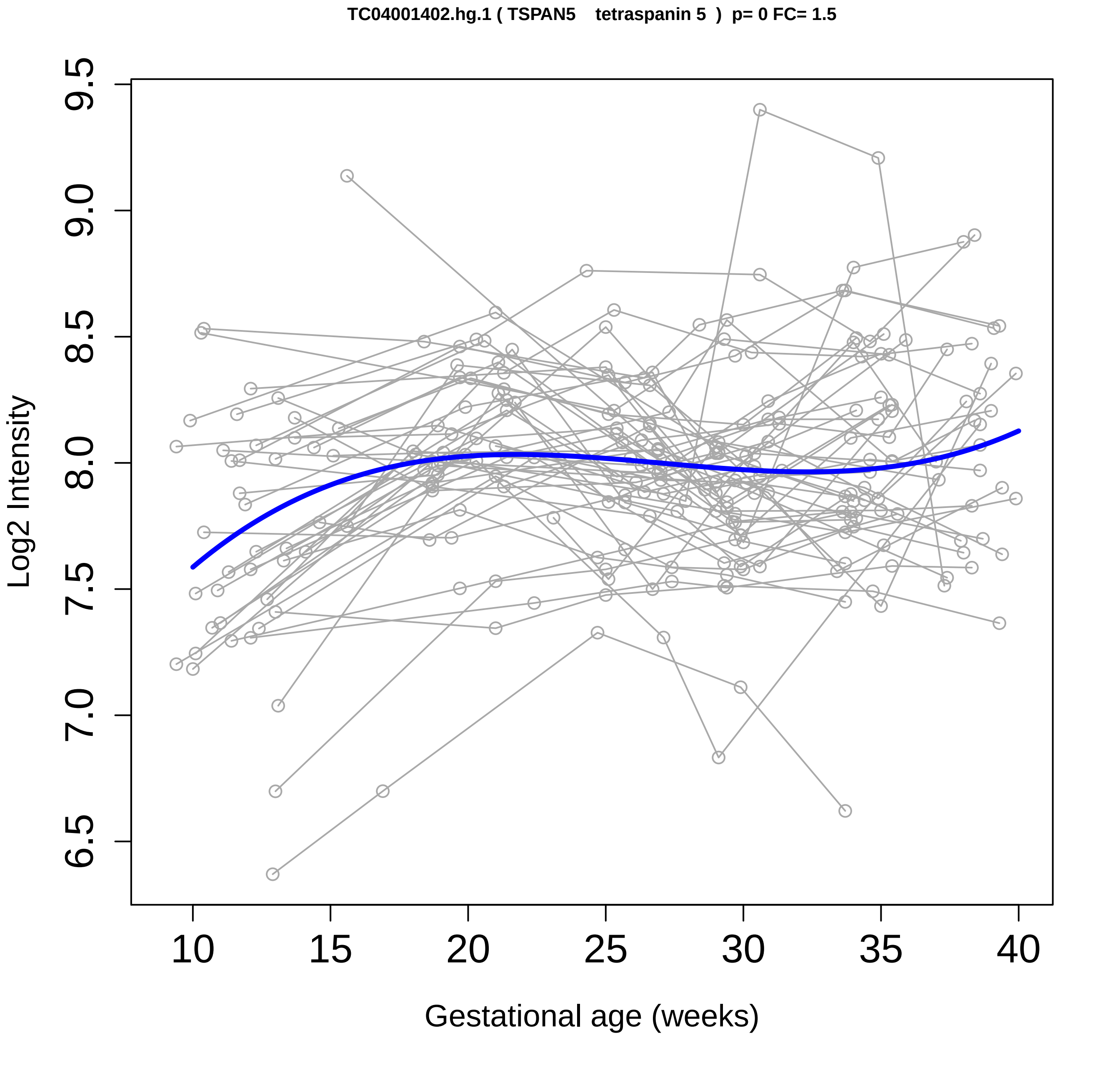

TC04001402.hg.1 (TSPAN5 tetraspanin 5) p= 0 FC= 1.5
Log2 Intensity
6.5
7.0
7.5
8.0
8.5
9.0
9.5
10
15
20
25
30
35
40
Gestational age (weeks)

TC16002075.hg.1 (MT1L metallothionein 1L (gene/pseudogene)) p= 0 FC= 1.3

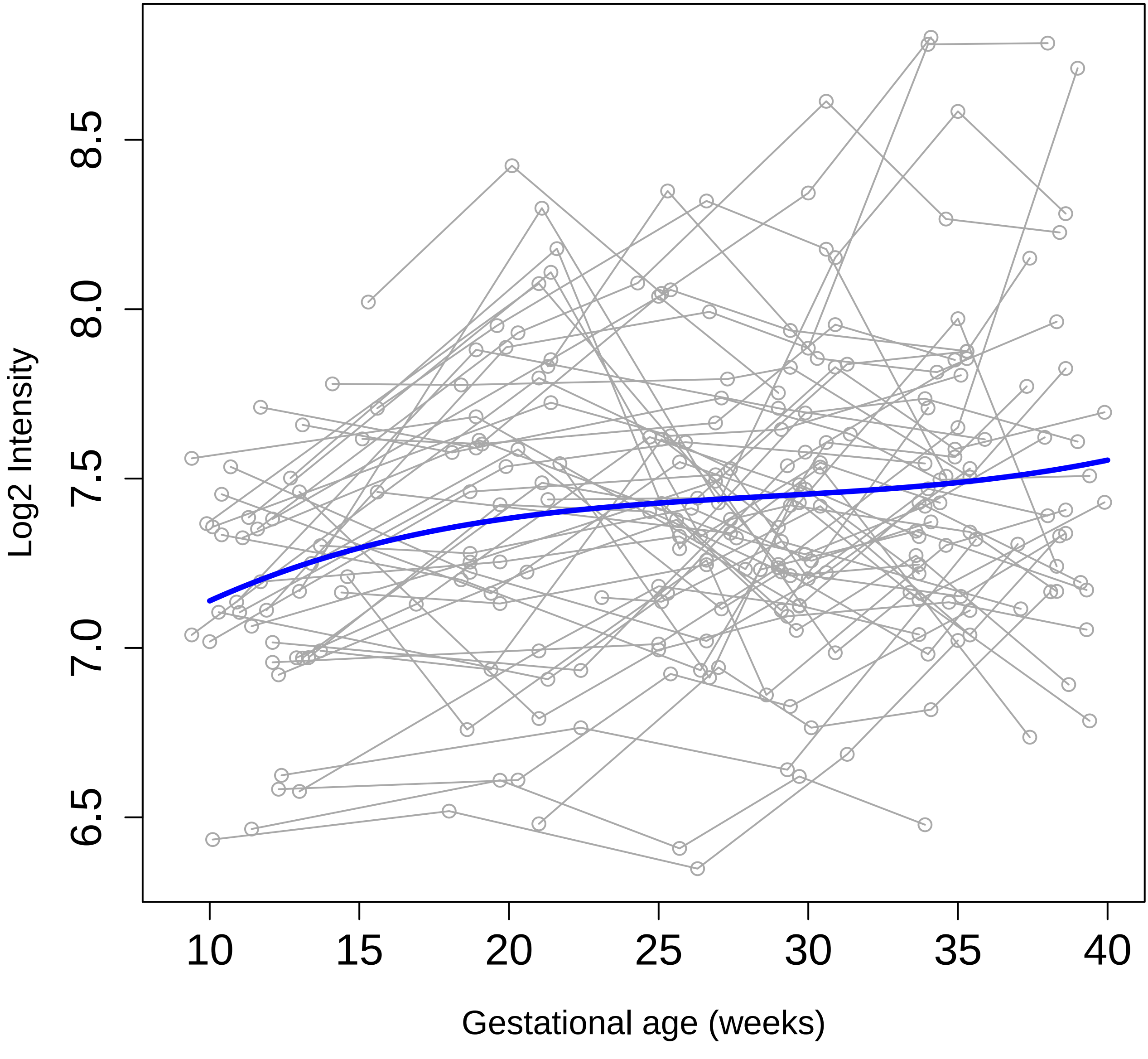

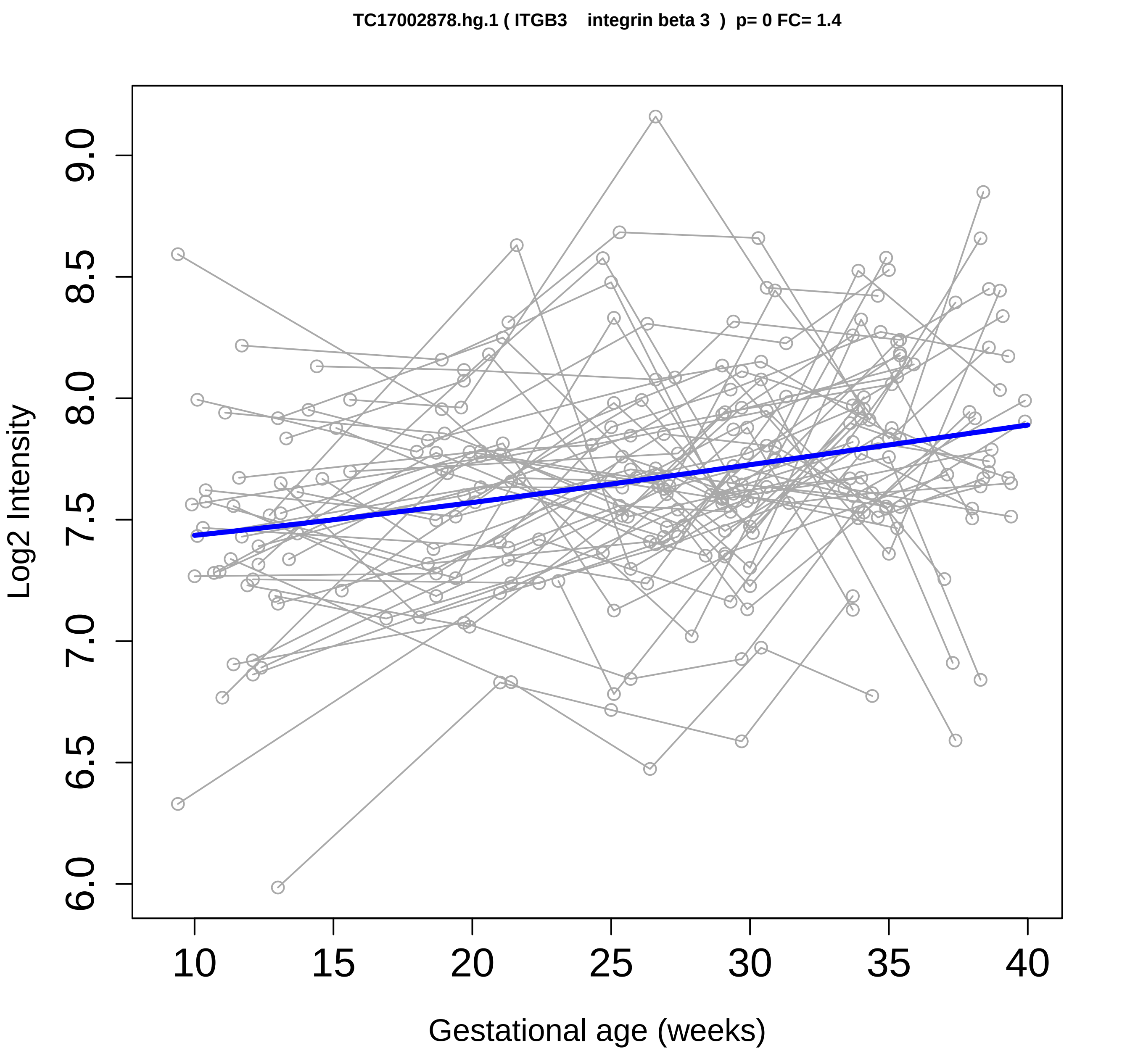
TC17002878.hg.1 (ITGB3 integrin beta 3) p= 0 FC= 1.4
Log2 Intensity
6.0
6.5
7.0
7.5
8.0
8.5
9.0
10
15
20
25
30
35
40
Gestational age (weeks)

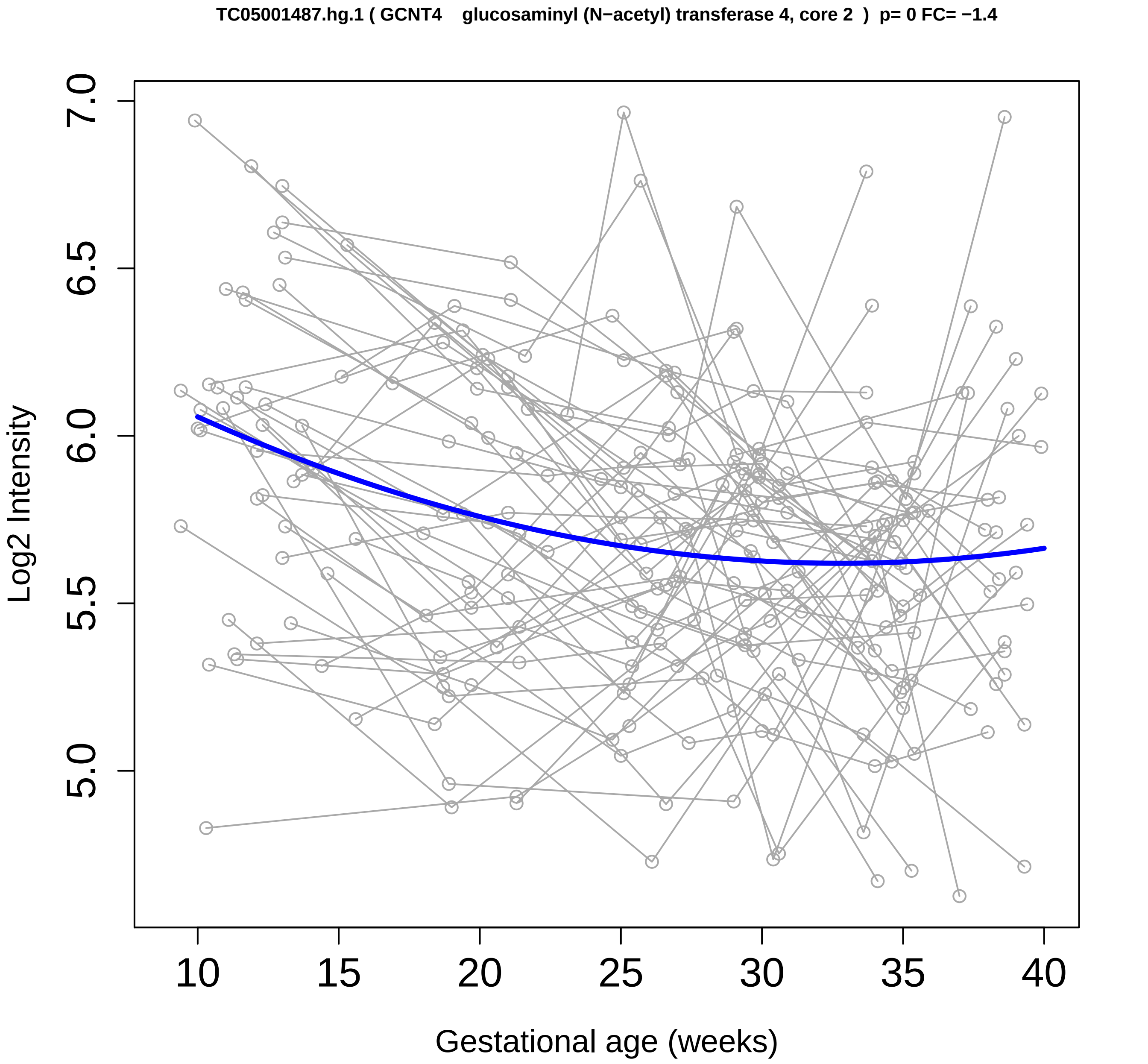
TC05001487.hg.1 (GCNT4 glucosaminyl (N−acetyl) transferase 4, core 2) p= 0 FC= −1.4
Log2 Intensity
7.0
6.5
6.0
5.5
5.0
10
15
20
25
30
35
40
Gestational age (weeks)

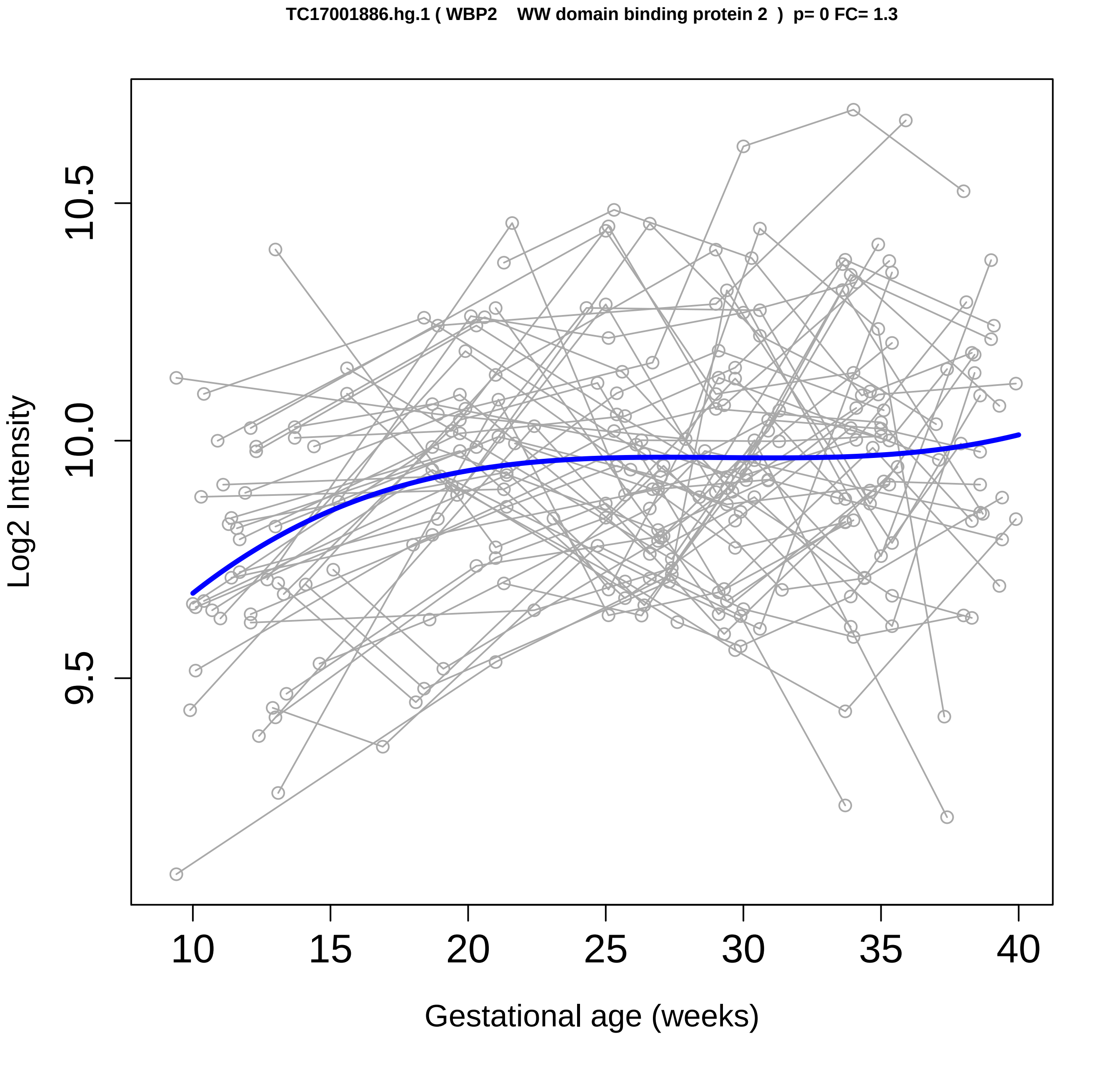

TC17001886.hg.1 (WBP2 WW domain binding protein 2) p= 0 FC= 1.3
Log2 Intensity
10.5
10.0
9.5
10
15
20
25
30
35
40
Gestational age (weeks)

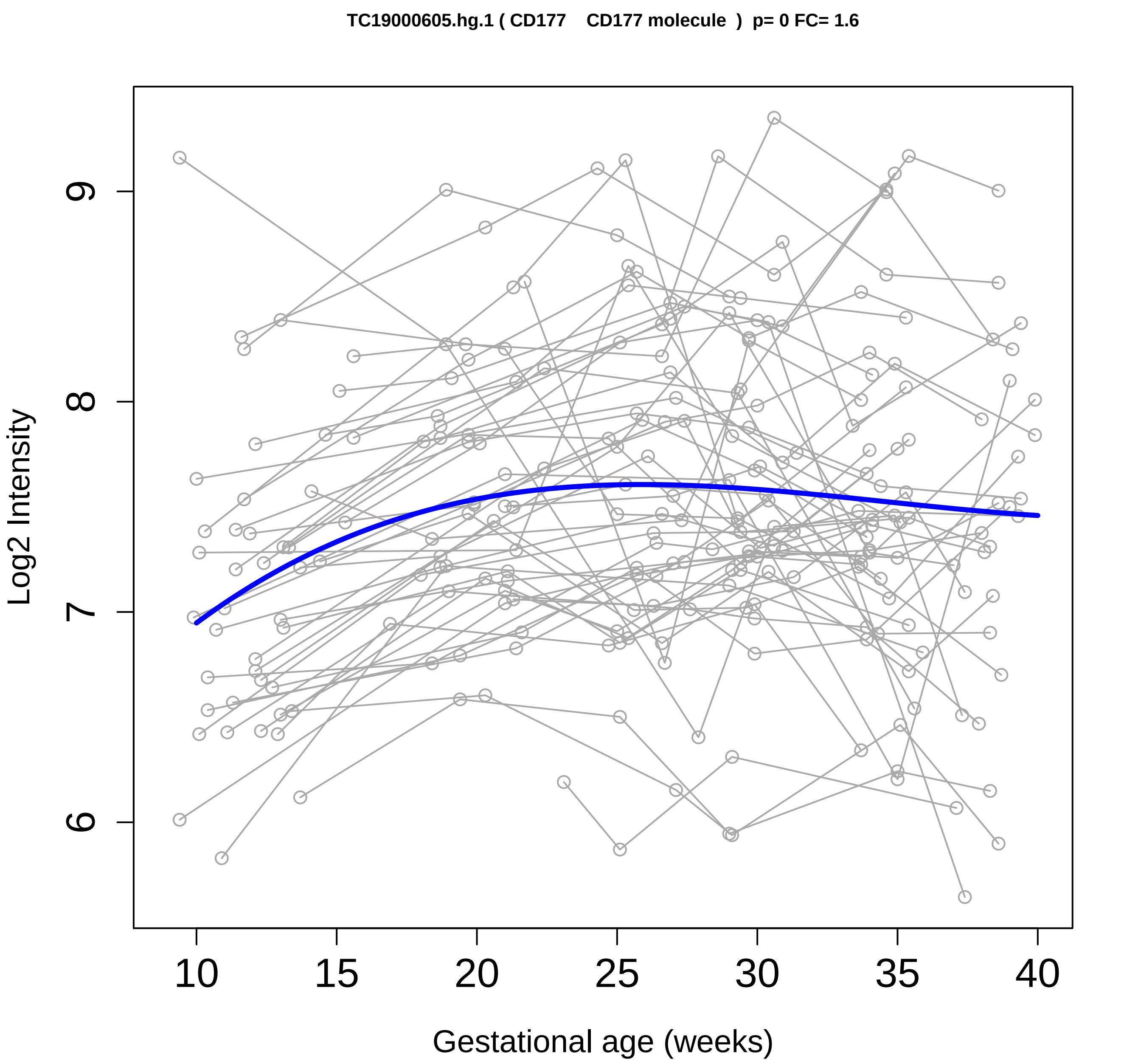
TC19000605.hg.1 (CD177 CD177 molecule) p= 0 FC= 1.6
Log2 Intensity
Gestational age (weeks)
6
7
8
9
10
15
20
25
30
35
40

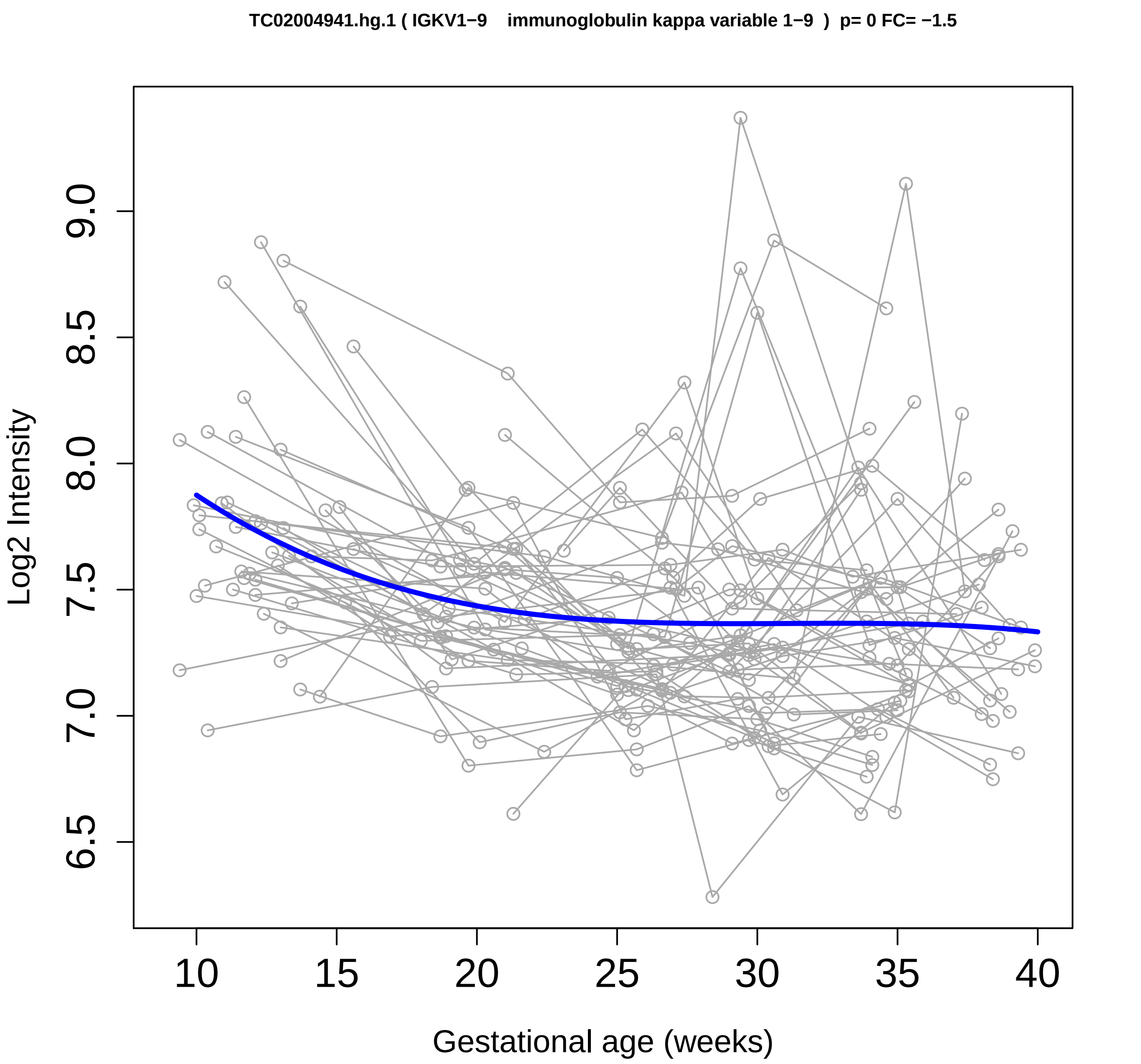

TC02004941.hg.1 (IGKV1−9 immunoglobulin kappa variable 1−9) p= 0 FC= −1.5
Log2 Intensity
6.5
7.0
7.5
8.0
8.5
9.0
10
15
20
25
30
35
40
Gestational age (weeks)

TC22001450.hg.1 (IGLL5 immunoglobulin lambda−like polypeptide 5) p= 0 FC= −1.3

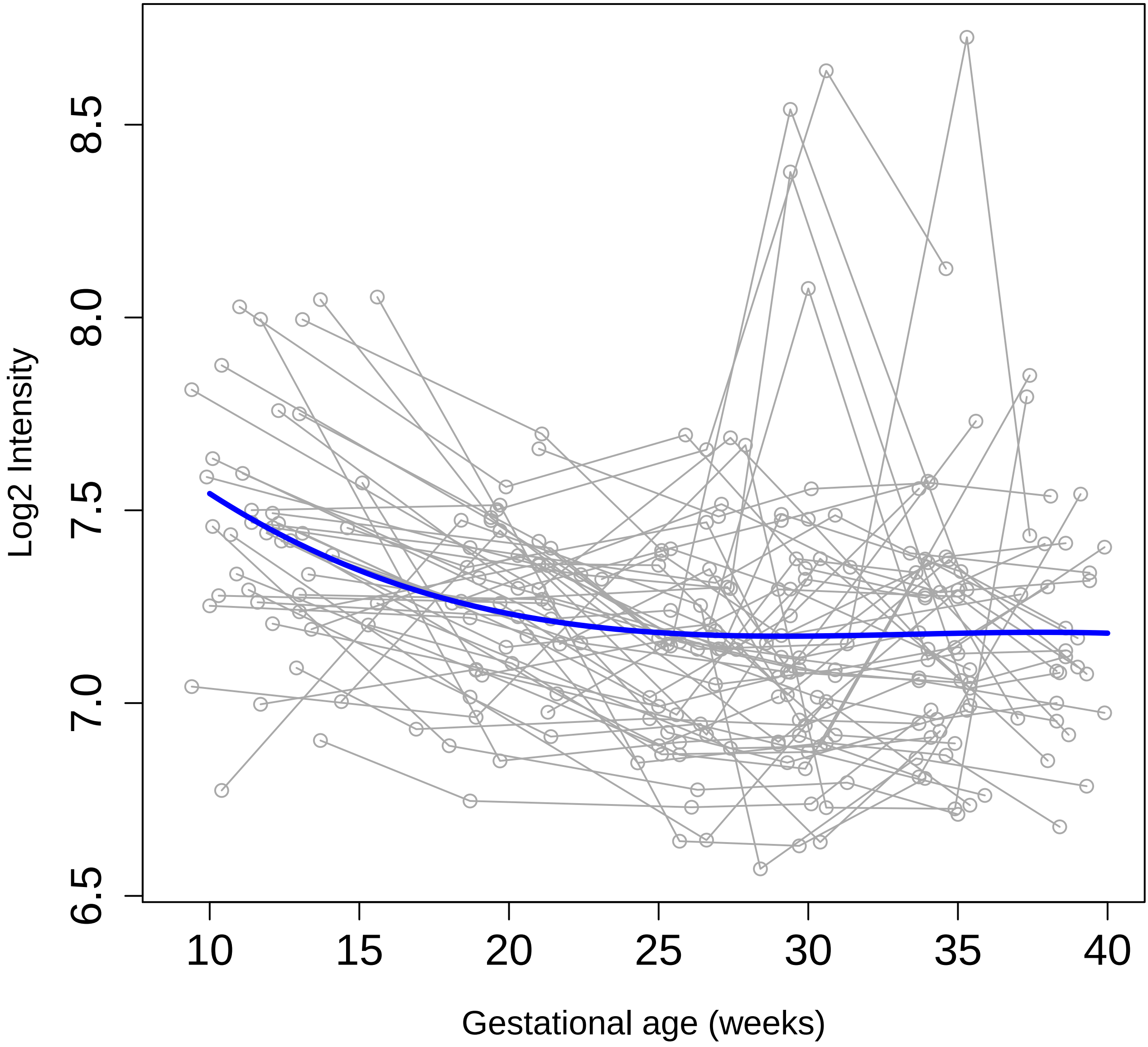

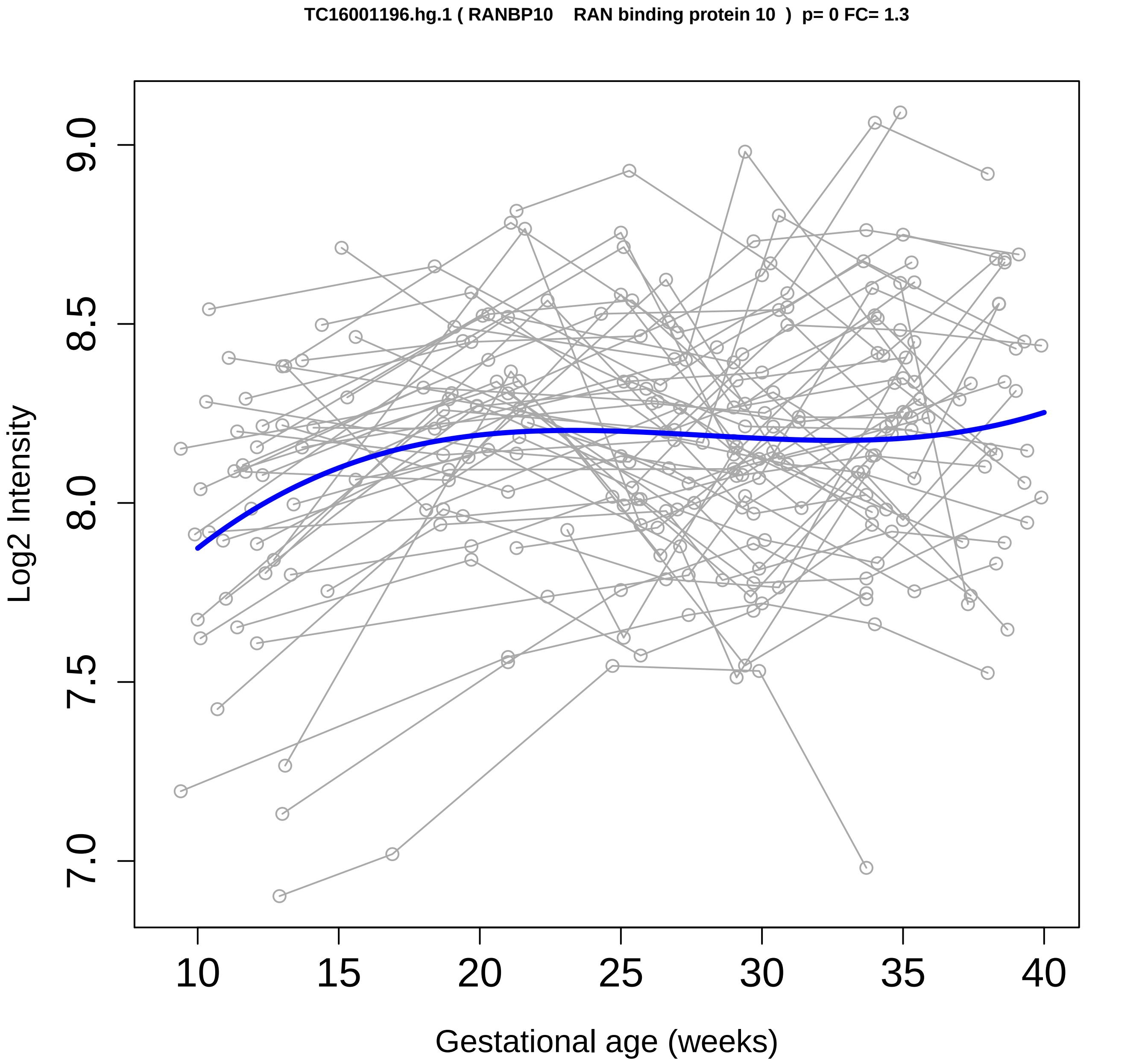
TC16001196.hg.1 (RANBP10 RAN binding protein 10) p= 0 FC= 1.3
Log2 Intensity
9.0
8.5
8.0
7.5
7.0
10
15
20
25
30
35
40
Gestational age (weeks)

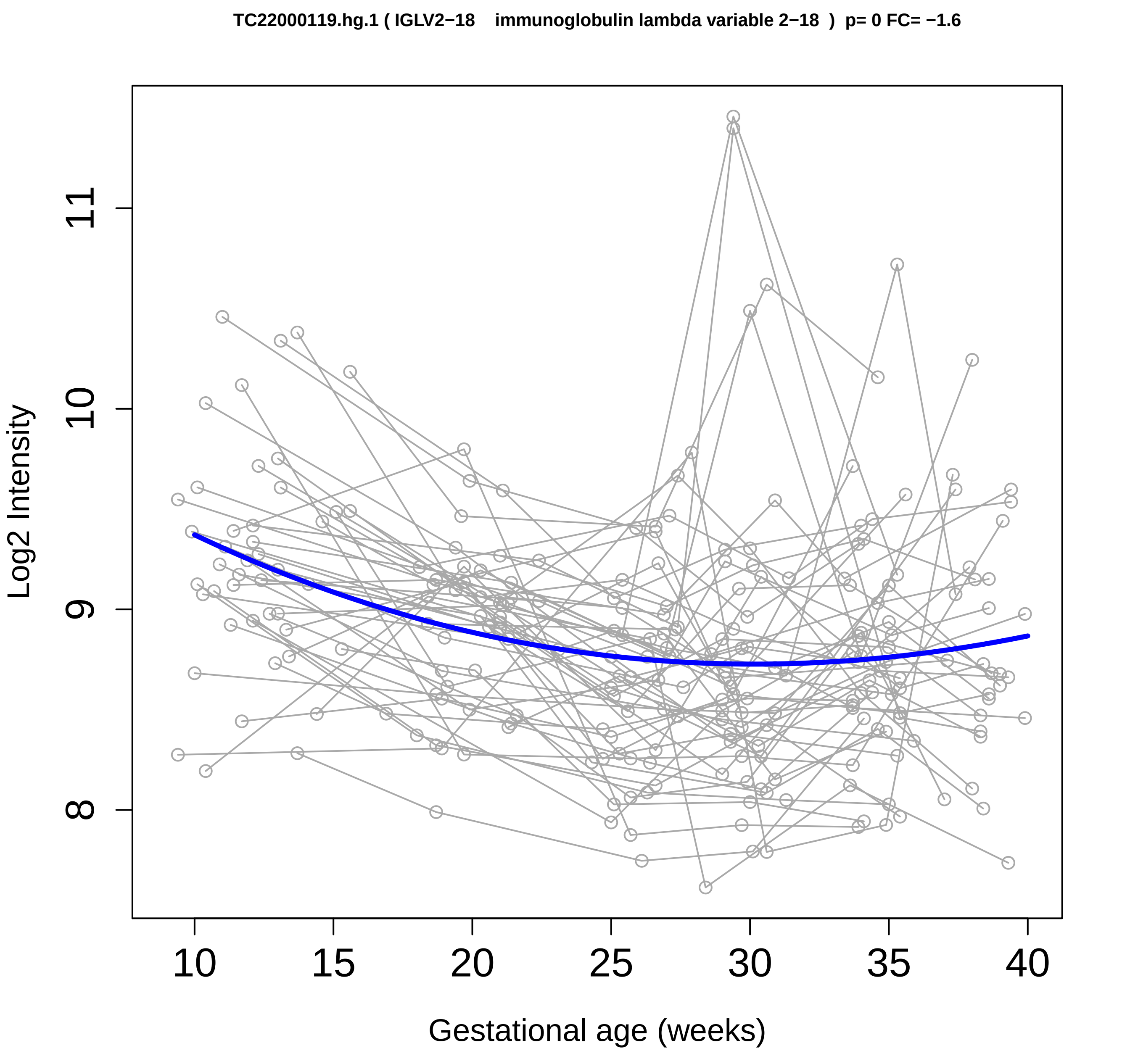

TC22000119.hg.1 (IGLV2−18 immunoglobulin lambda variable 2−18) p= 0 FC= −1.6
Log2 Intensity
8
9
10
11
10
15
20
25
30
35
40
Gestational age (weeks)

TC01002847.hg.1 (GBP1 guanylate binding protein 1, interferon−inducible) p= 0 FC= 1.6

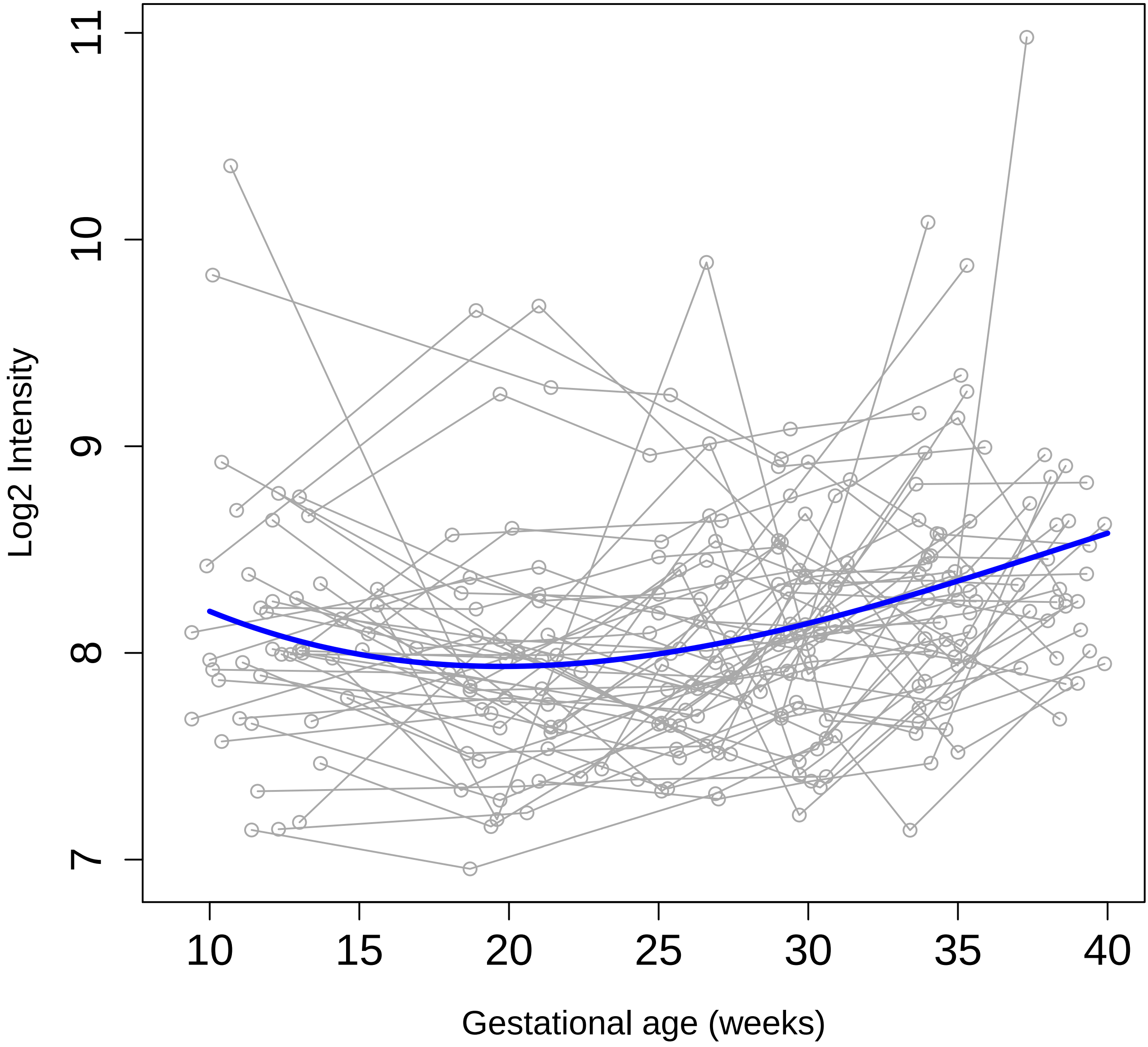

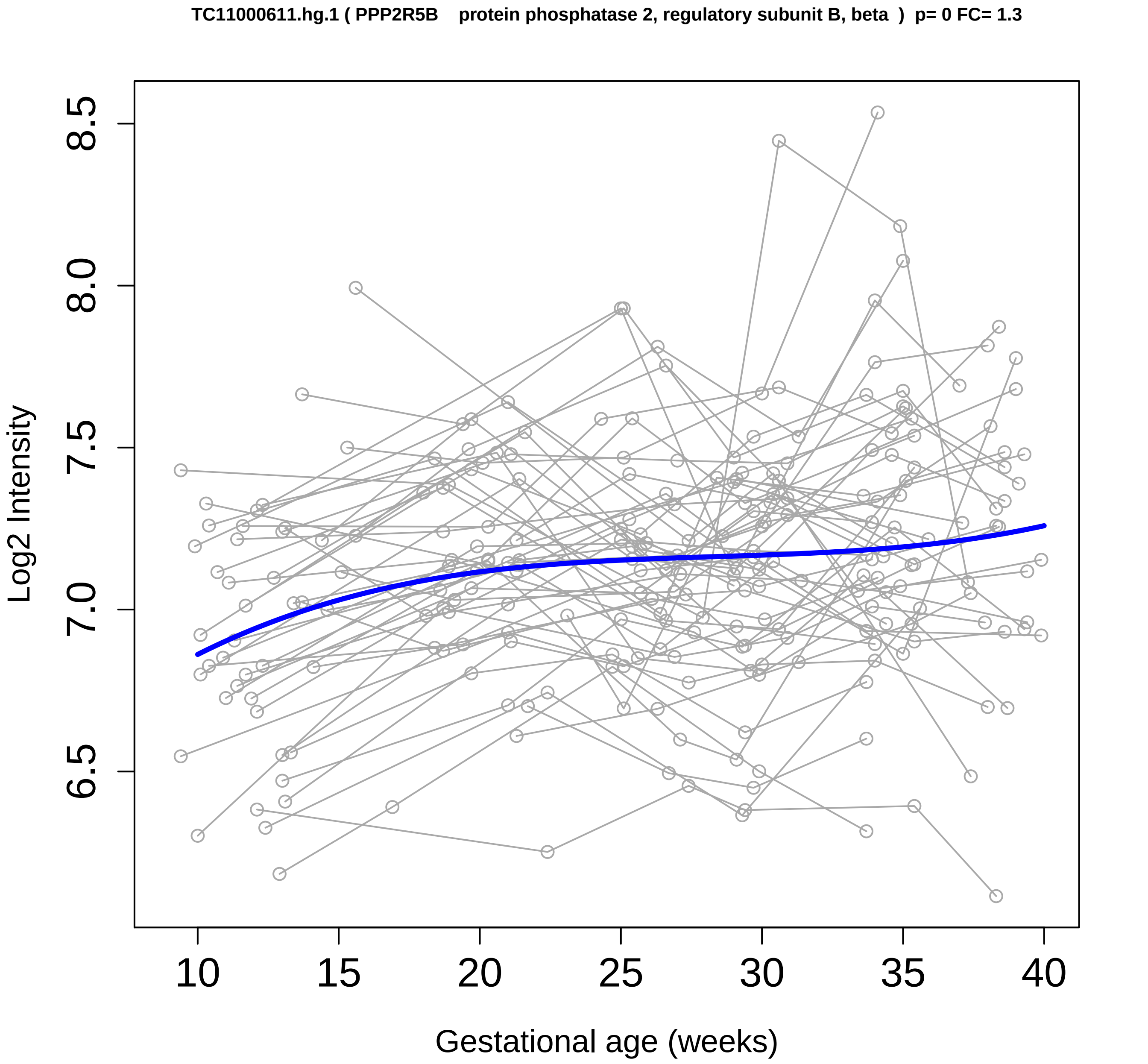
TC11000611.hg.1 (PPP2R5B protein phosphatase 2, regulatory subunit B, beta) p= 0 FC= 1.3
Log2 Intensity
8.5
8.0
7.5
7.0
6.5
10
15
20
25
30
35
40
Gestational age (weeks)

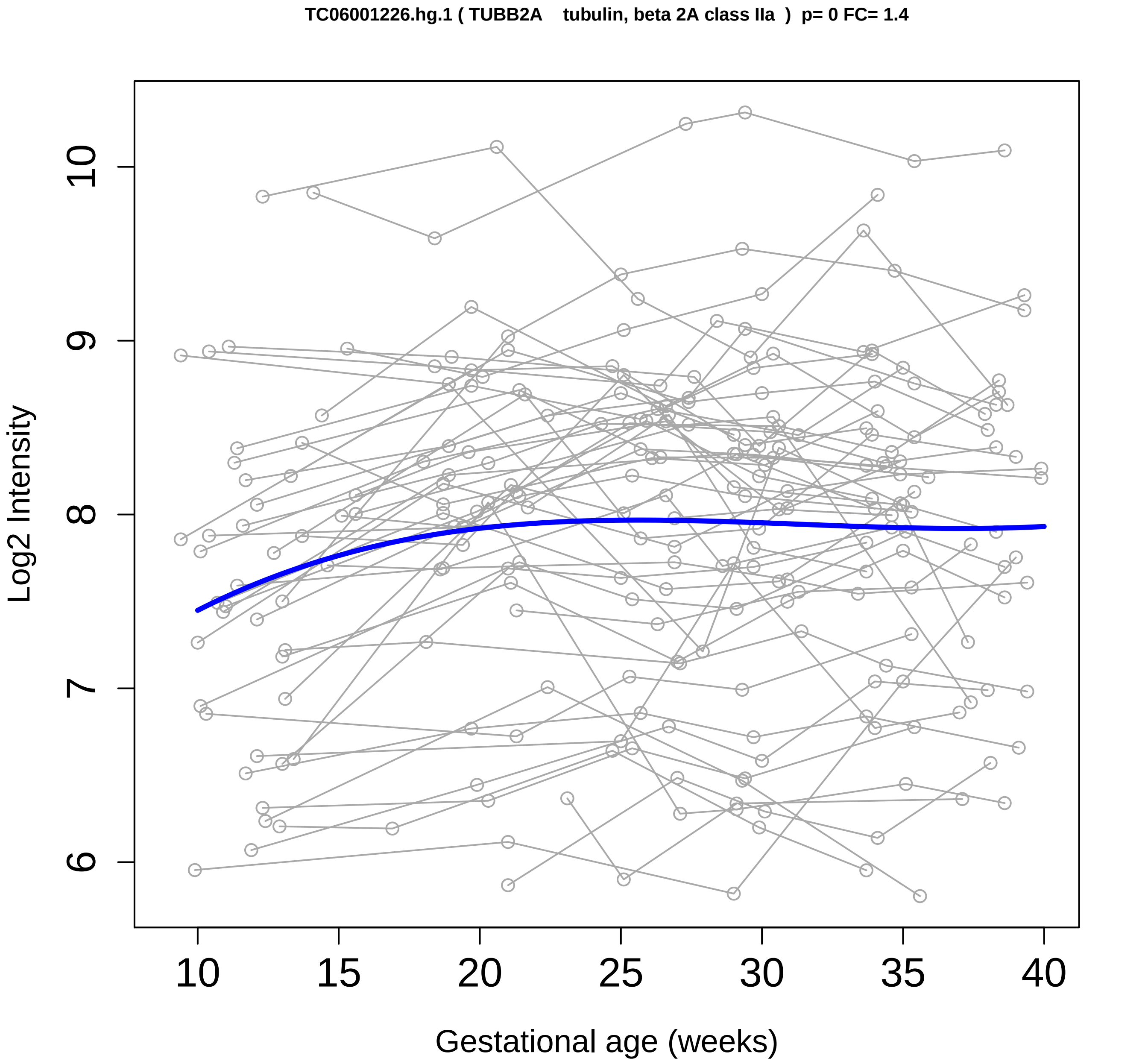

TC06001226.hg.1 (TUBB2A tubulin, beta 2A class IIa) p= 0 FC= 1.4
Log2 Intensity
6
7
8
9
10
10
15
20
25
30
35
40
Gestational age (weeks)

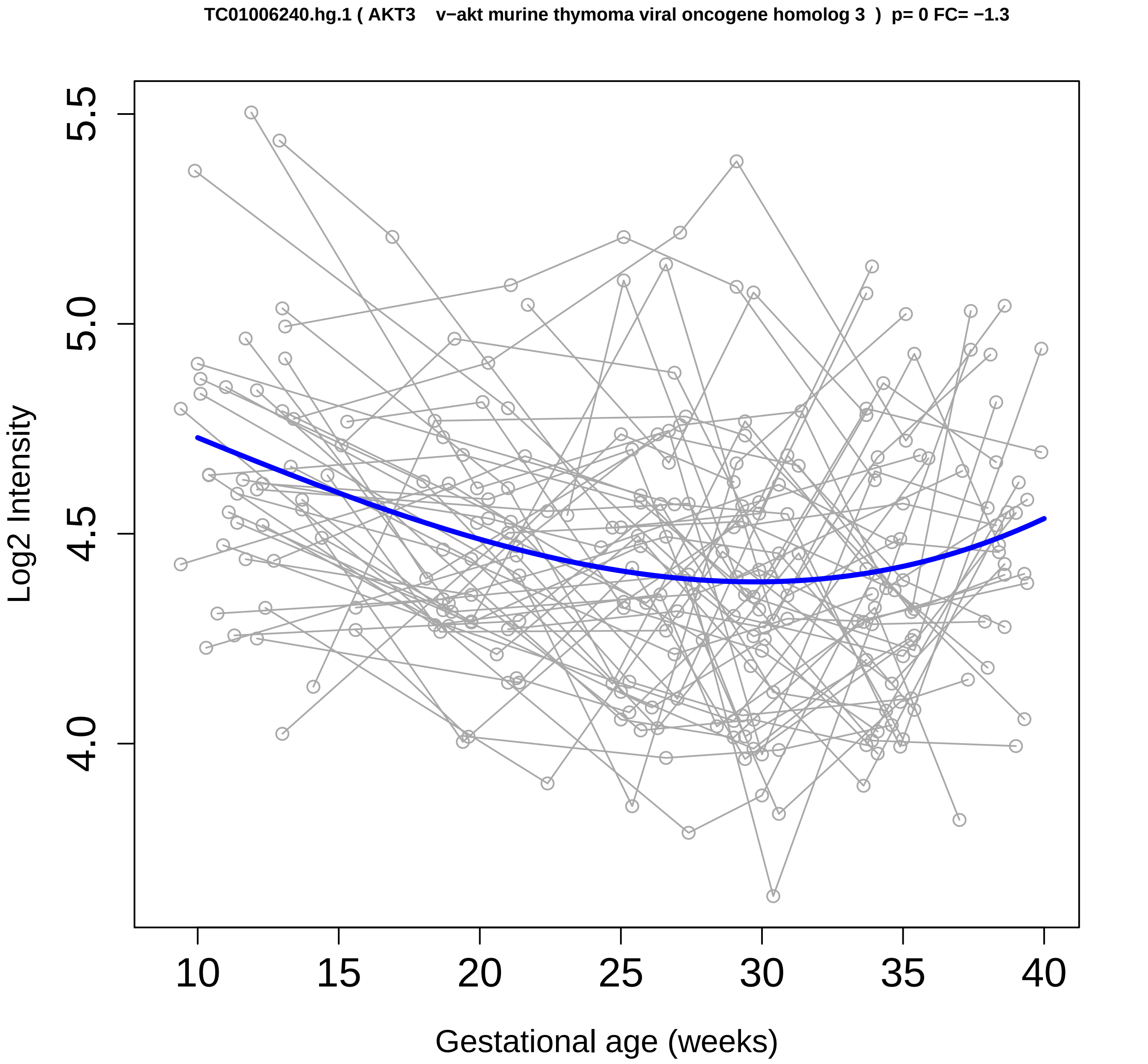
TC01006240.hg.1 (AKT3 v−akt murine thymoma viral oncogene homolog 3) p= 0 FC= −1.3
Log2 Intensity
5.5
5.0
4.5
4.0
10
15
20
25
30
35
40
Gestational age (weeks)

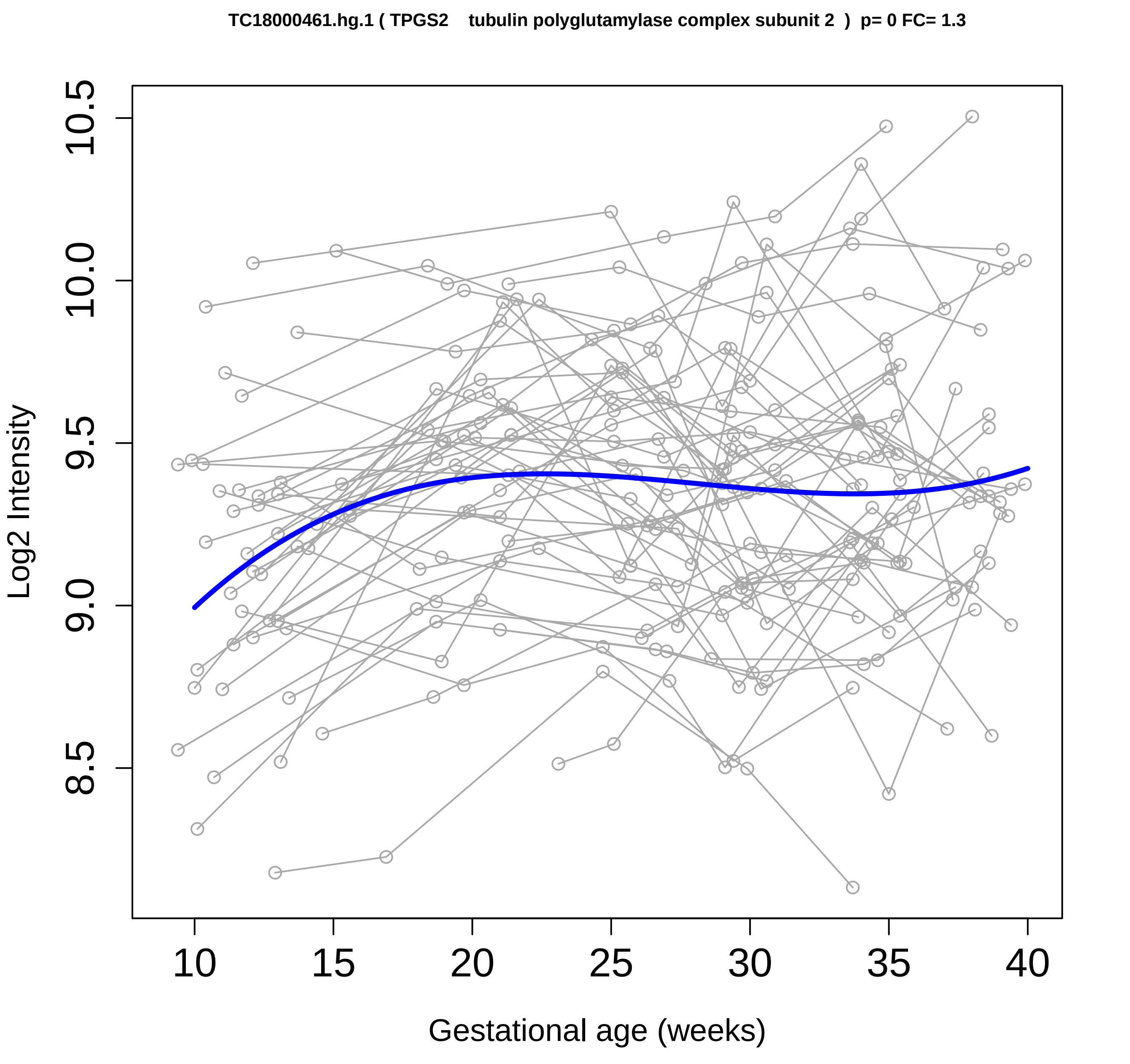

TC18000461.hg.1 (TPGS2 tubulin polyglutamylase complex subunit 2) p= 0 FC= 1.3
Log2 Intensity
8.5
9.0
9.5
10.0
10.5
10
15
20
25
30
35
40
Gestational age (weeks)

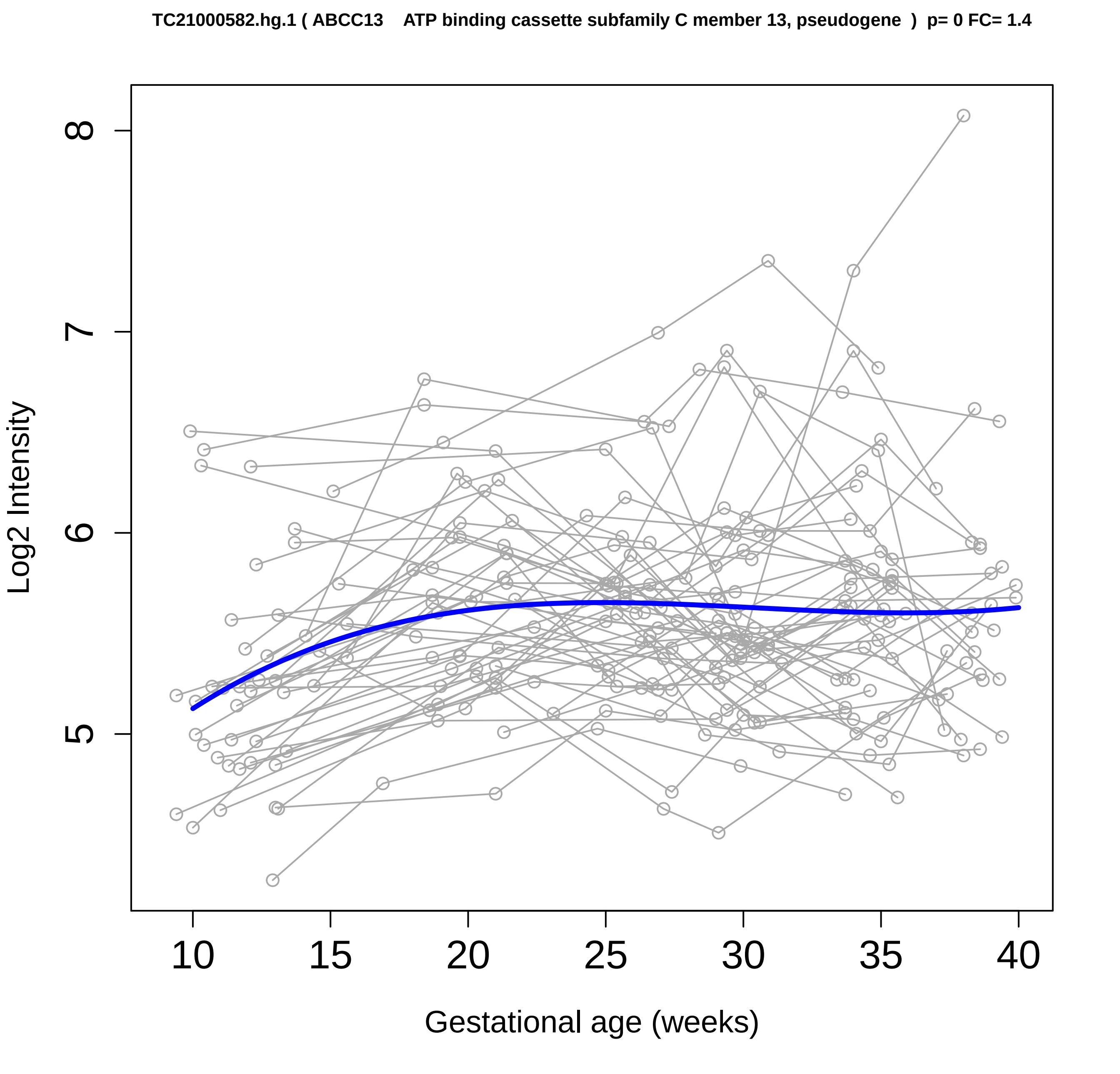
TC21000582.hg.1 (ABCC13 ATP binding cassette subfamily C member 13, pseudogene) p= 0 FC= 1.4
Log2 Intensity
8
7
6
5
10
15
20
25
30
35
40
Gestational age (weeks)

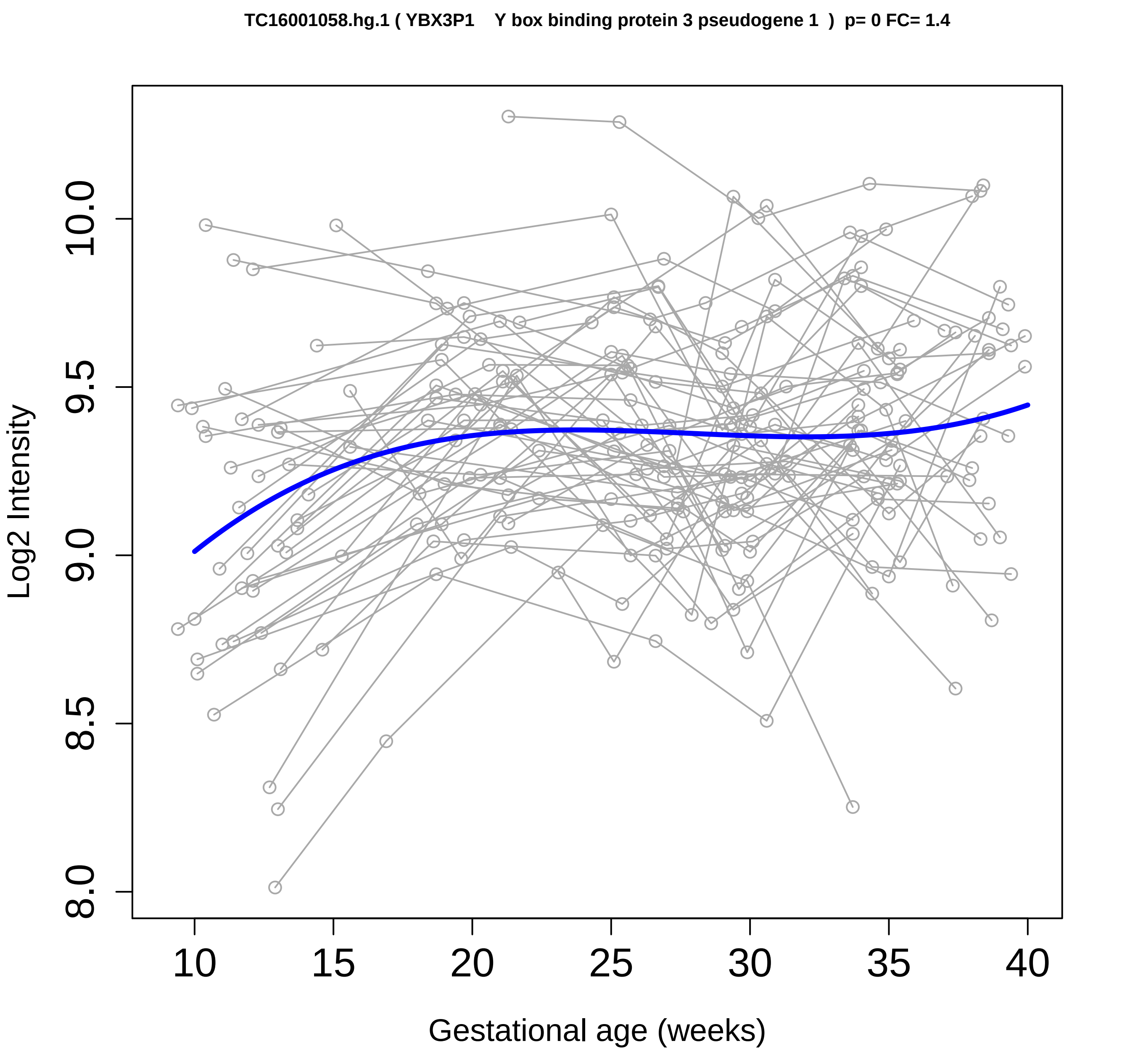
TC16001058.hg.1 (YBX3P1 Y box binding protein 3 pseudogene 1) p= 0 FC= 1.4
Log2 Intensity
8.0
8.5
9.0
9.5
10.0
10
15
20
25
30
35
40
Gestational age (weeks)

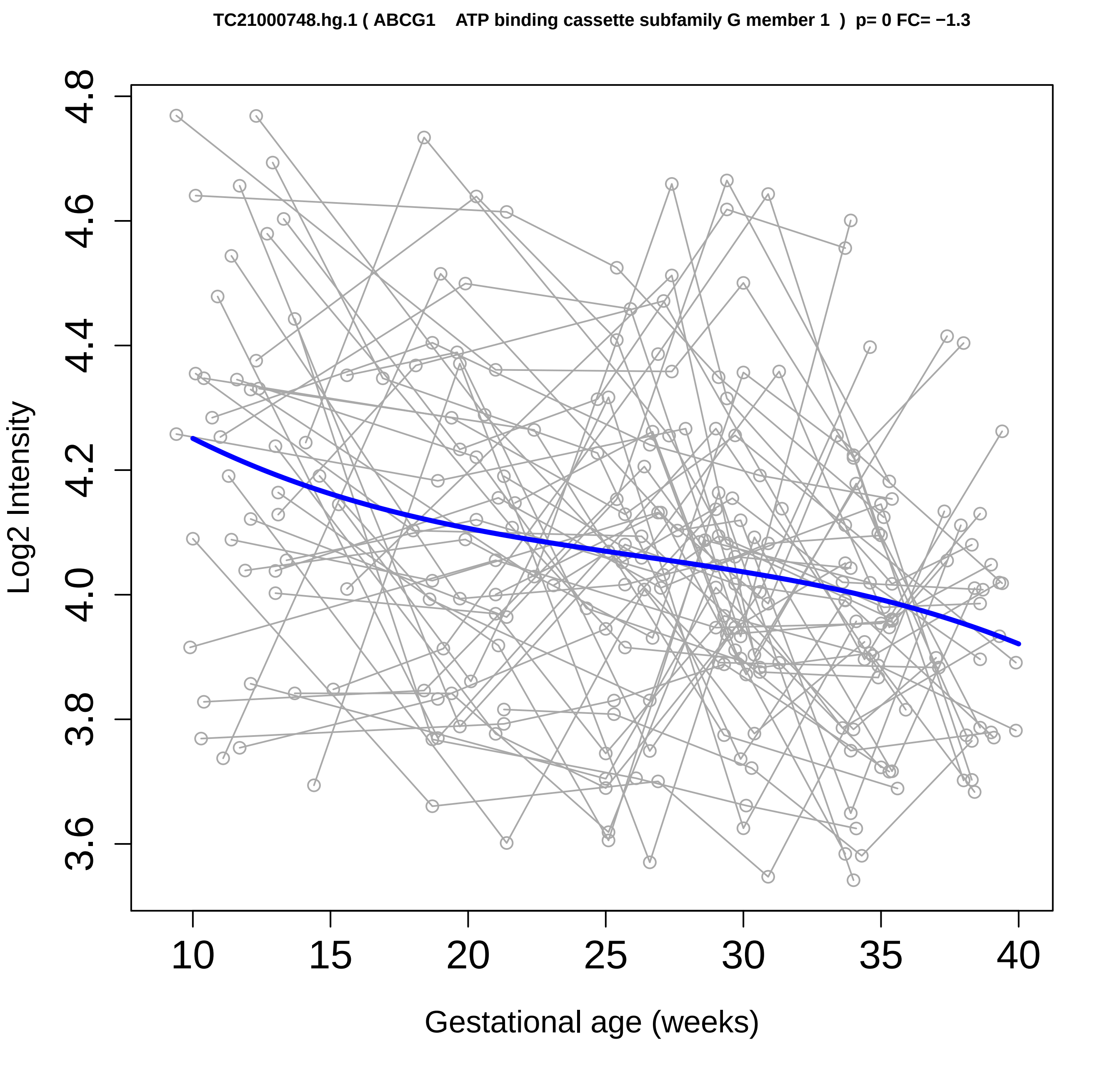
TC21000748.hg.1 (ABCG1   ATP binding cassette subfamily G member 1) p= 0 FC= −1.3
Log2 Intensity
4.8
4.6
4.4
4.2
4.0
3.8
3.6
10
15
20
25
30
35
40
Gestational age (weeks)

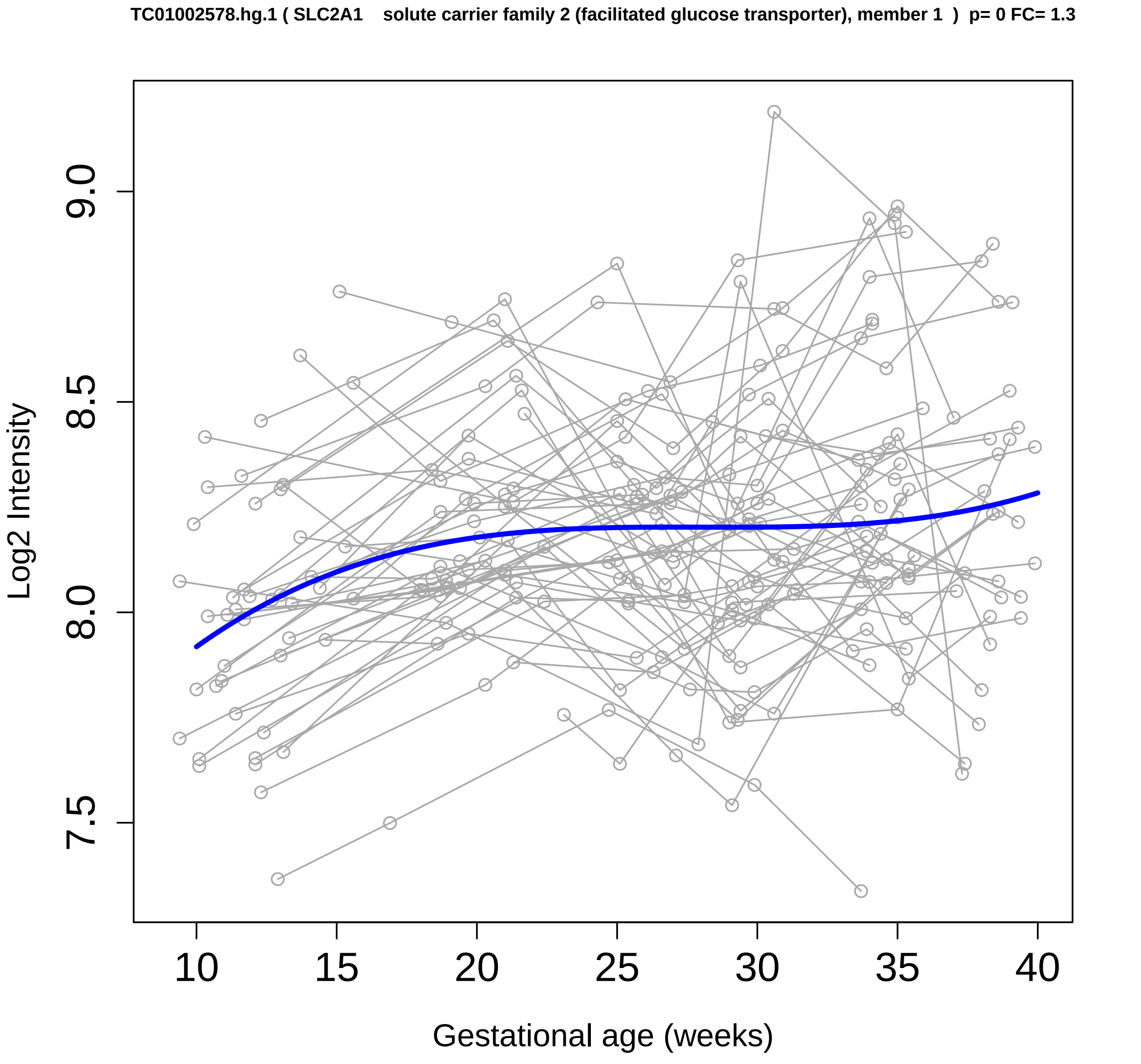

TC01002578.hg.1 (SLC2A1 solute carrier family 2 (facilitated glucose transporter), member 1) p= 0 FC= 1.3
Log2 Intensity
9.0
8.5
8.0
7.5
10
15
20
25
30
35
40
Gestational age (weeks)

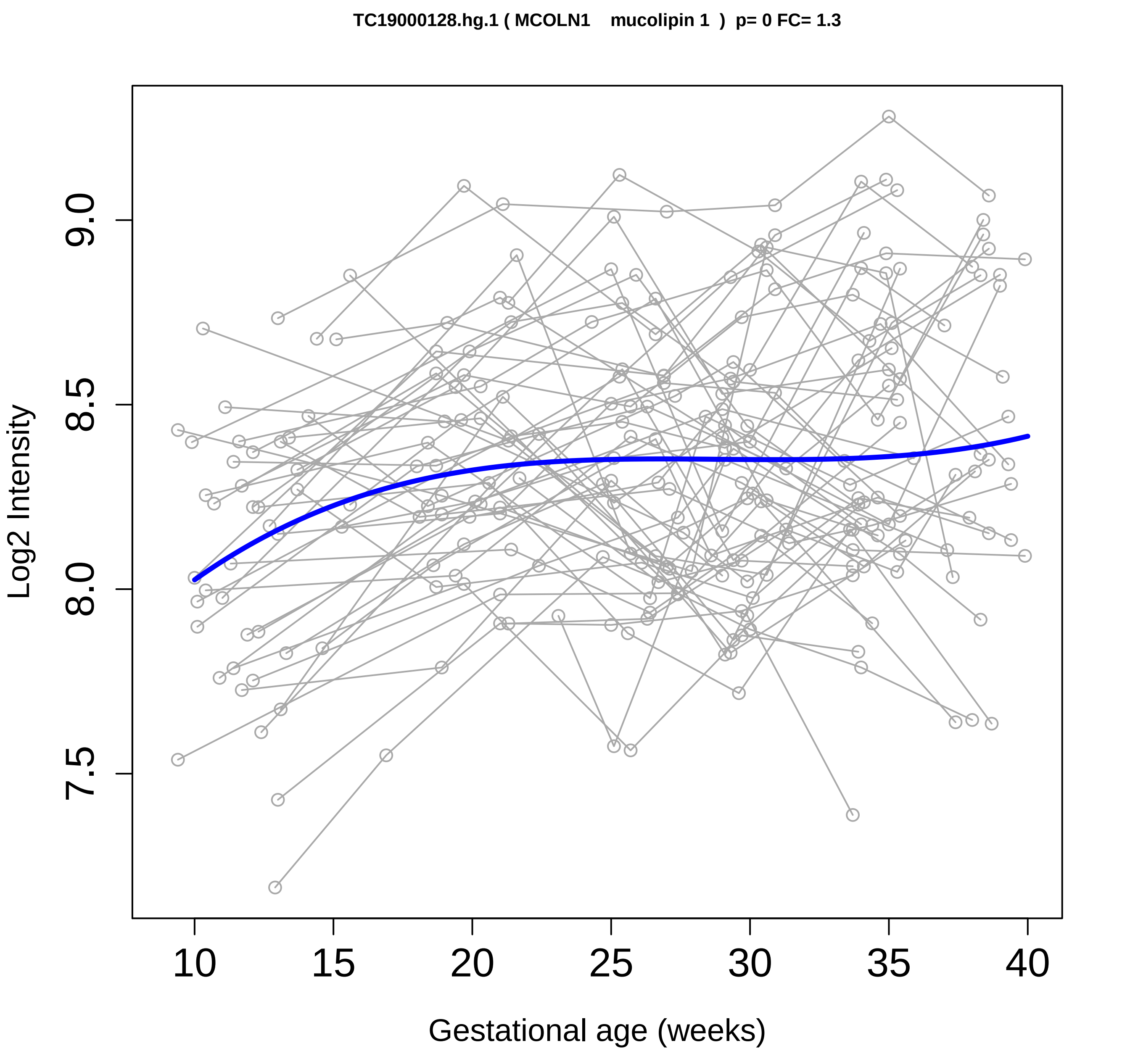
TC19000128.hg.1 (MCOLN1 mucolipin 1) p= 0 FC= 1.3
Log2 Intensity
9.0
8.5
8.0
7.5
10
15
20
25
30
35
40
Gestational age (weeks)

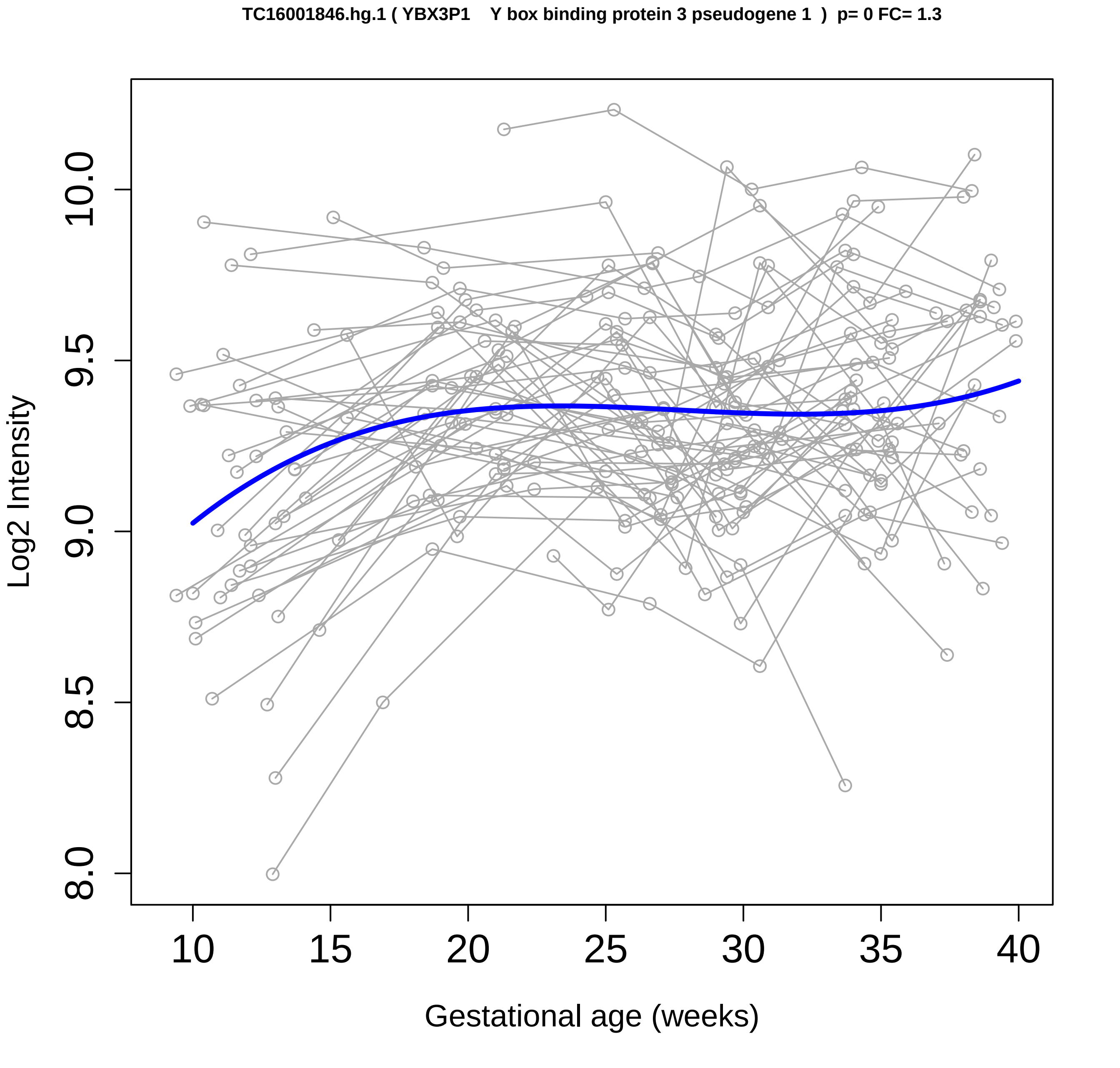

TC16001846.hg.1 (YBX3P1 Y box binding protein 3 pseudogene 1) p= 0 FC= 1.3
Log2 Intensity
8.0
8.5
9.0
9.5
10.0
10
15
20
25
30
35
40
Gestational age (weeks)

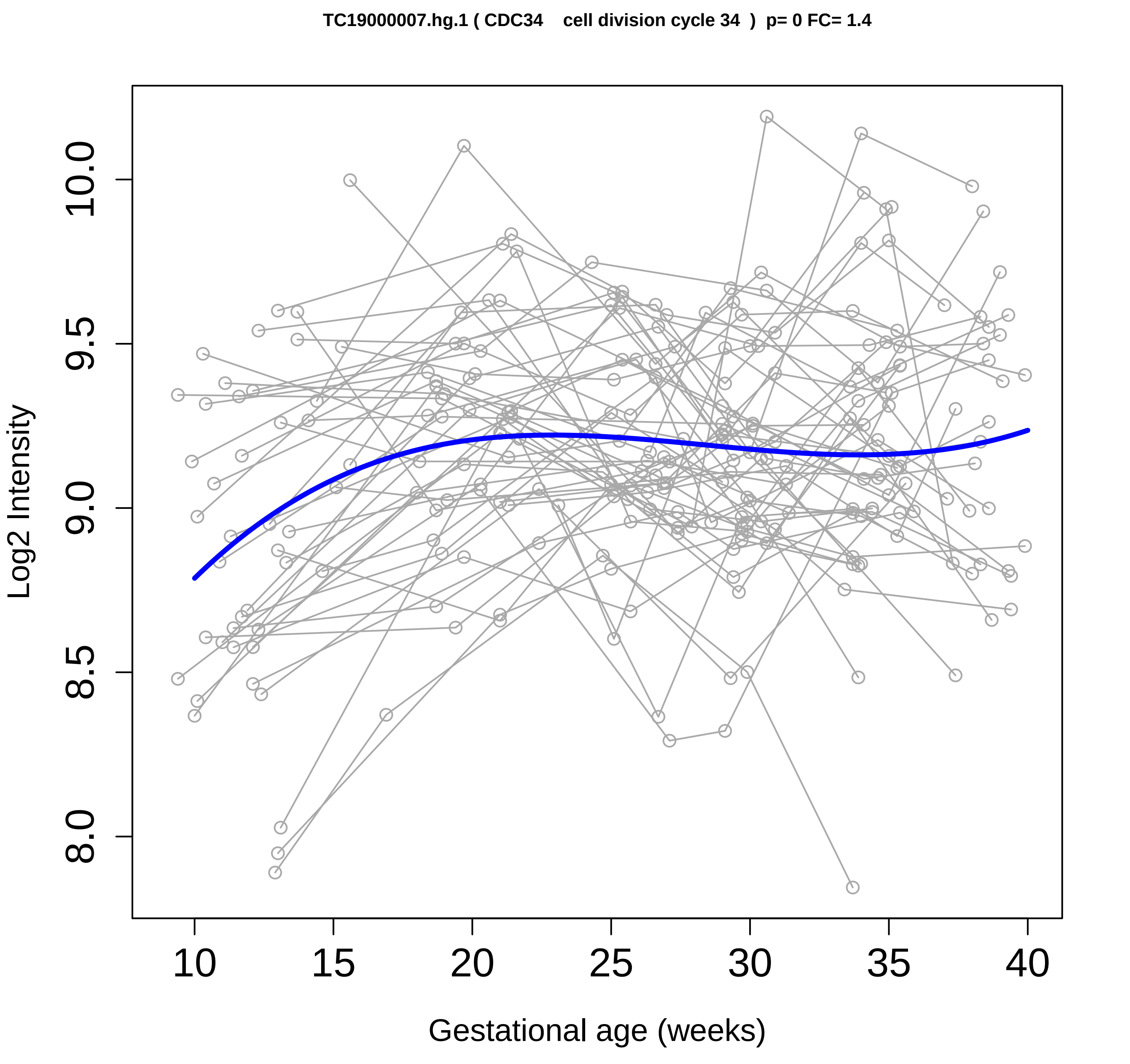

TC19000007.hg.1 (CDC34 cell division cycle 34) p= 0 FC= 1.4
Log2 Intensity
8.0
8.5
9.0
9.5
10.0
10
15
20
25
30
35
40
Gestational age (weeks)

TC02004974.hg.1 (IGKV1D−39 immunoglobulin kappa variable 1D−39) p= 0 FC= −1.5

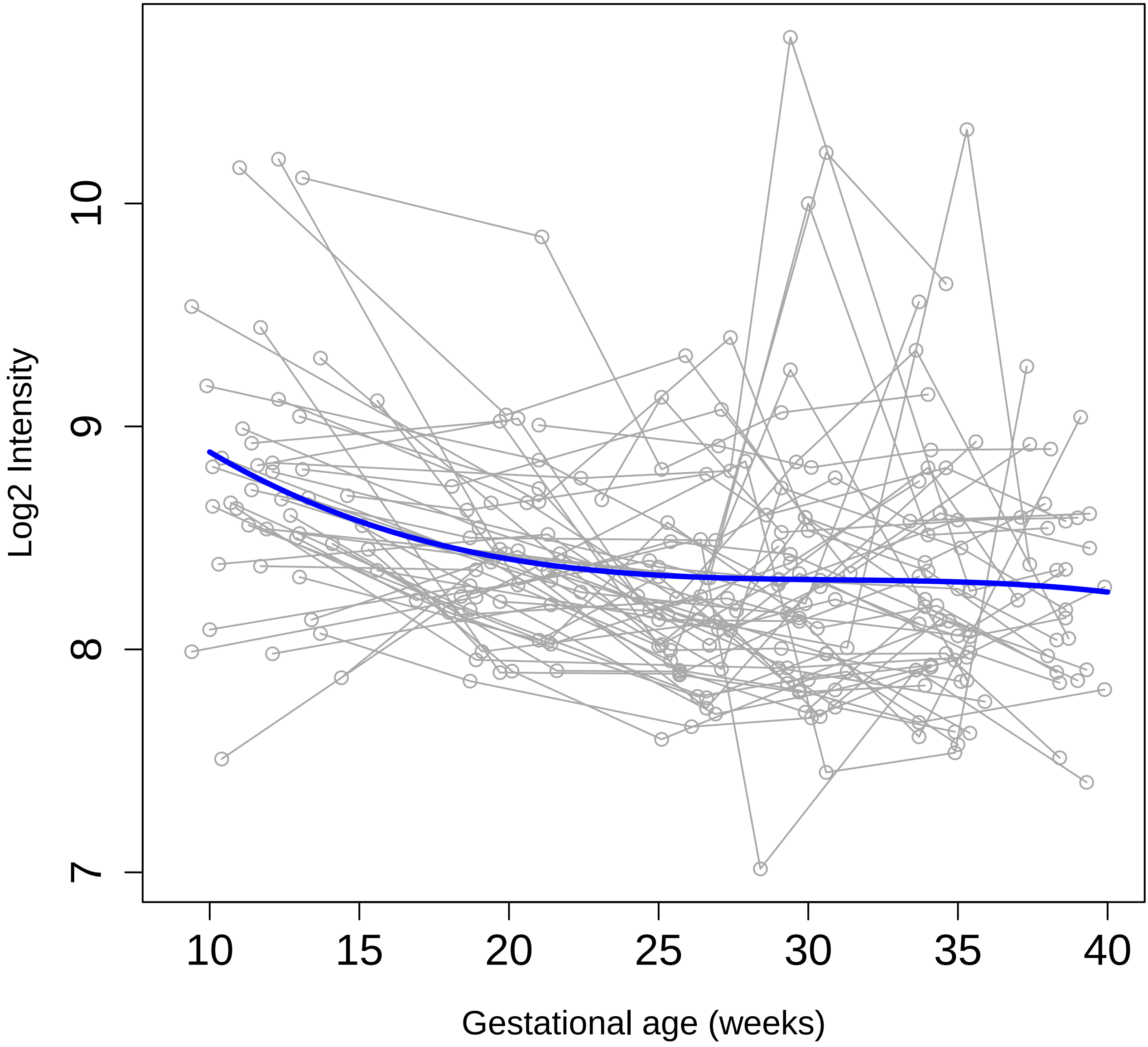

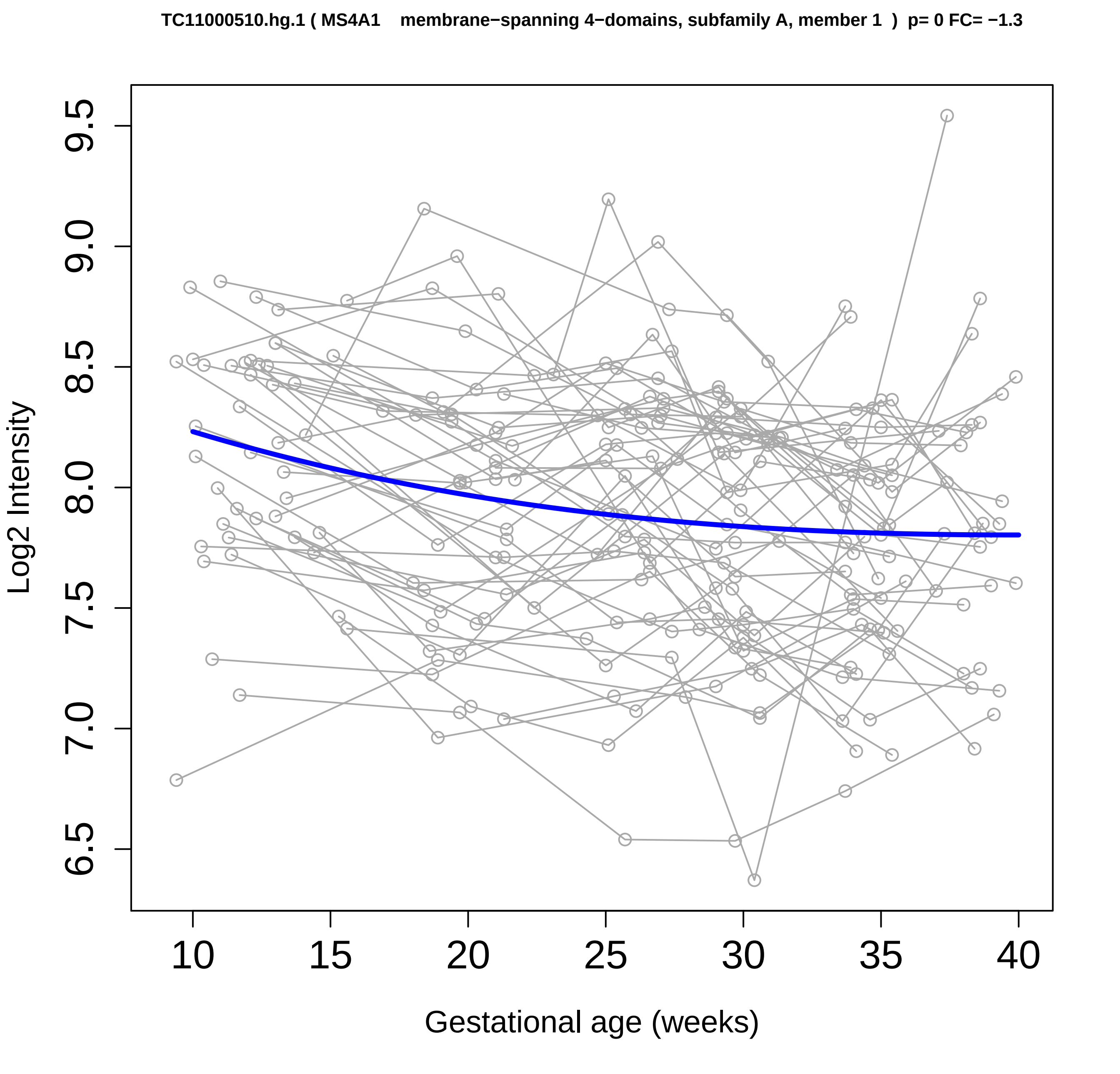

TC11000510.hg.1 (MS4A1 membrane−spanning 4−domains, subfamily A, member 1) p= 0 FC= −1.3
Log2 Intensity
6.5
7.0
7.5
8.0
8.5
9.0
9.5
10
15
20
25
30
35
40
Gestational age (weeks)

TC15000606.hg.1 (HACD3   3−hydroxyacyl−CoA dehydratase 3) p= 0 FC= −1.3

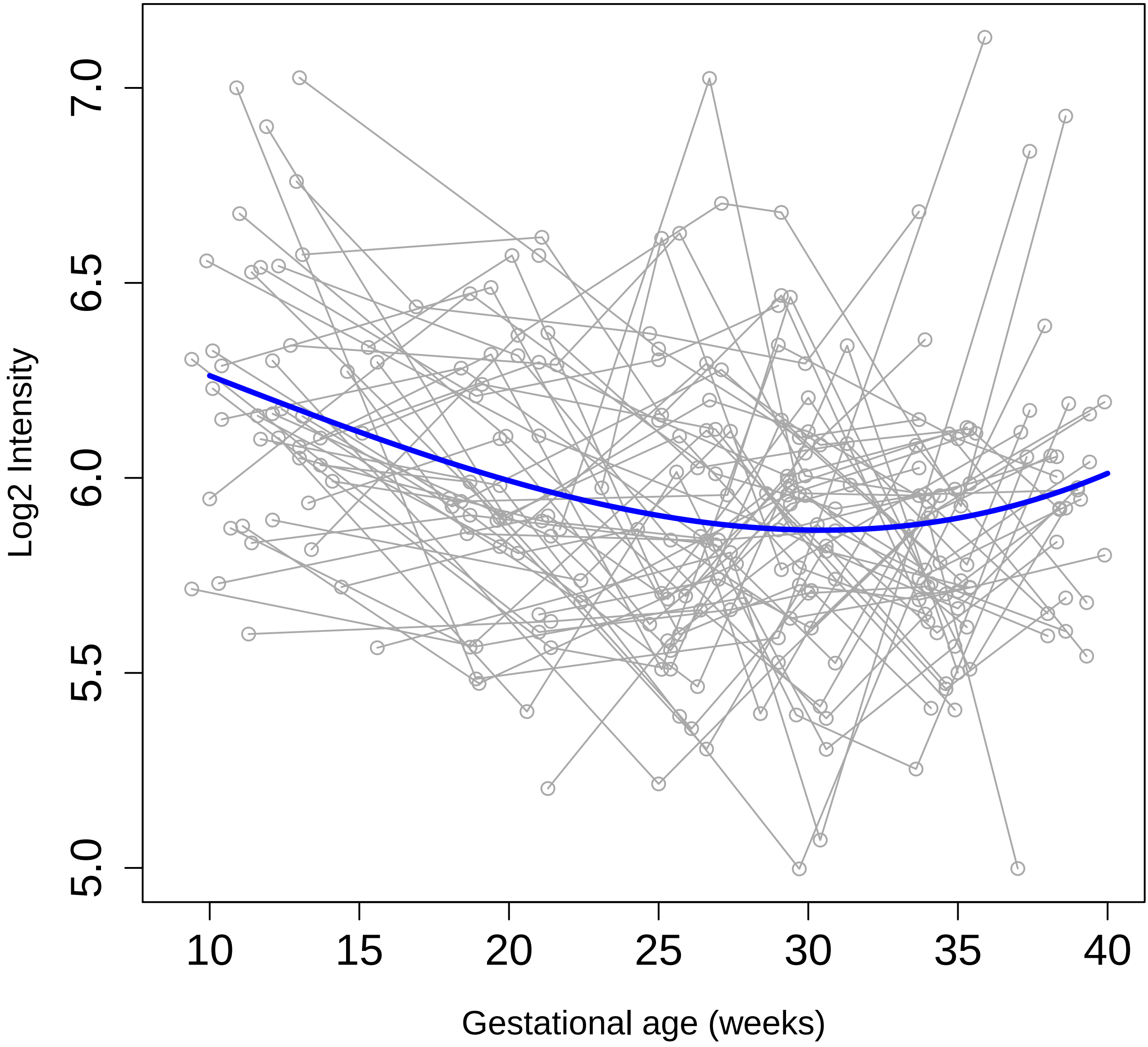

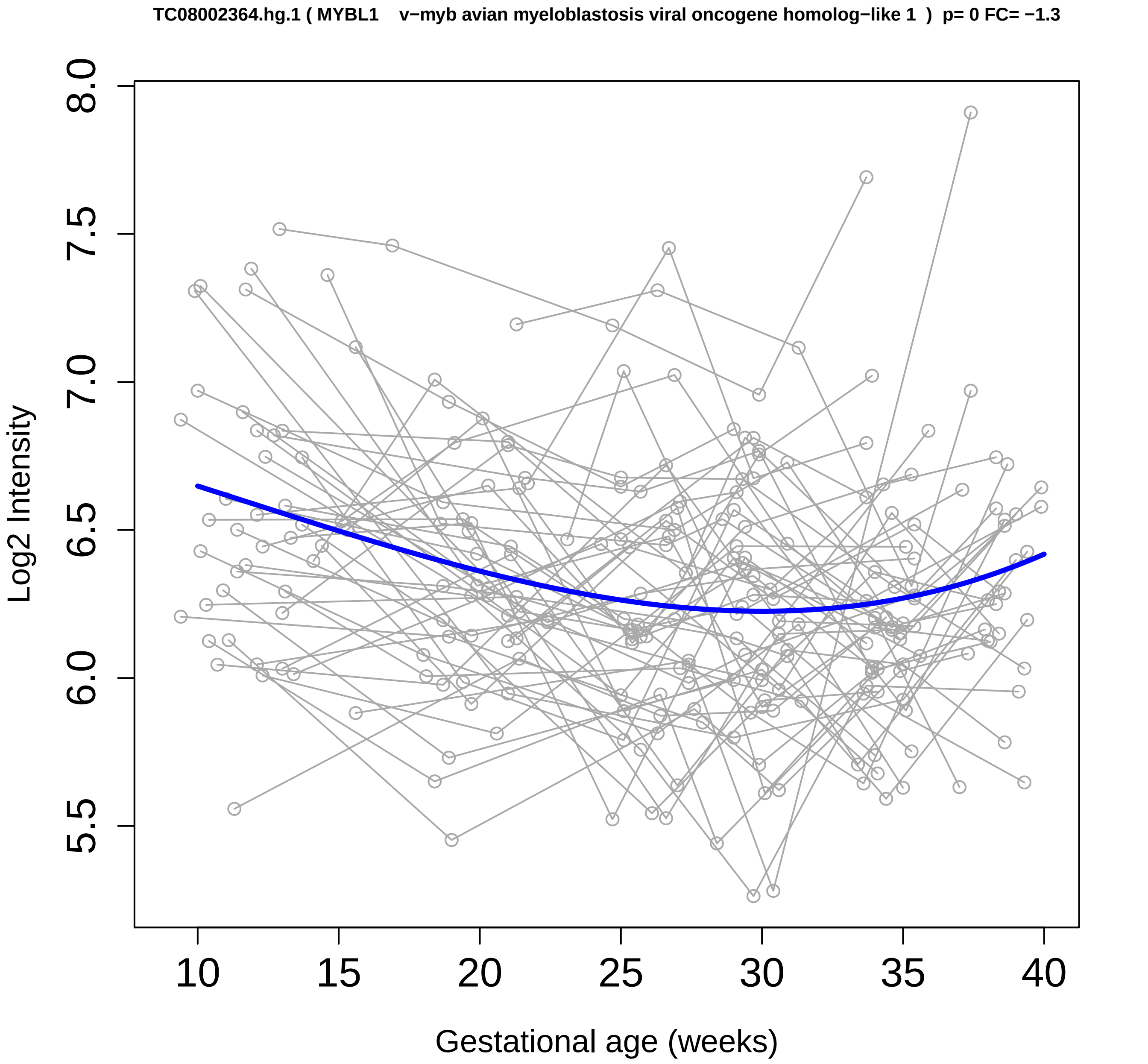

TC08002364.hg.1 (MYBL1 v−myb avian myeloblastosis viral oncogene homolog−like 1) p= 0 FC= −1.3
Log2 Intensity
8.0
7.5
7.0
6.5
6.0
5.5
10
15
20
25
30
35
40
Gestational age (weeks)

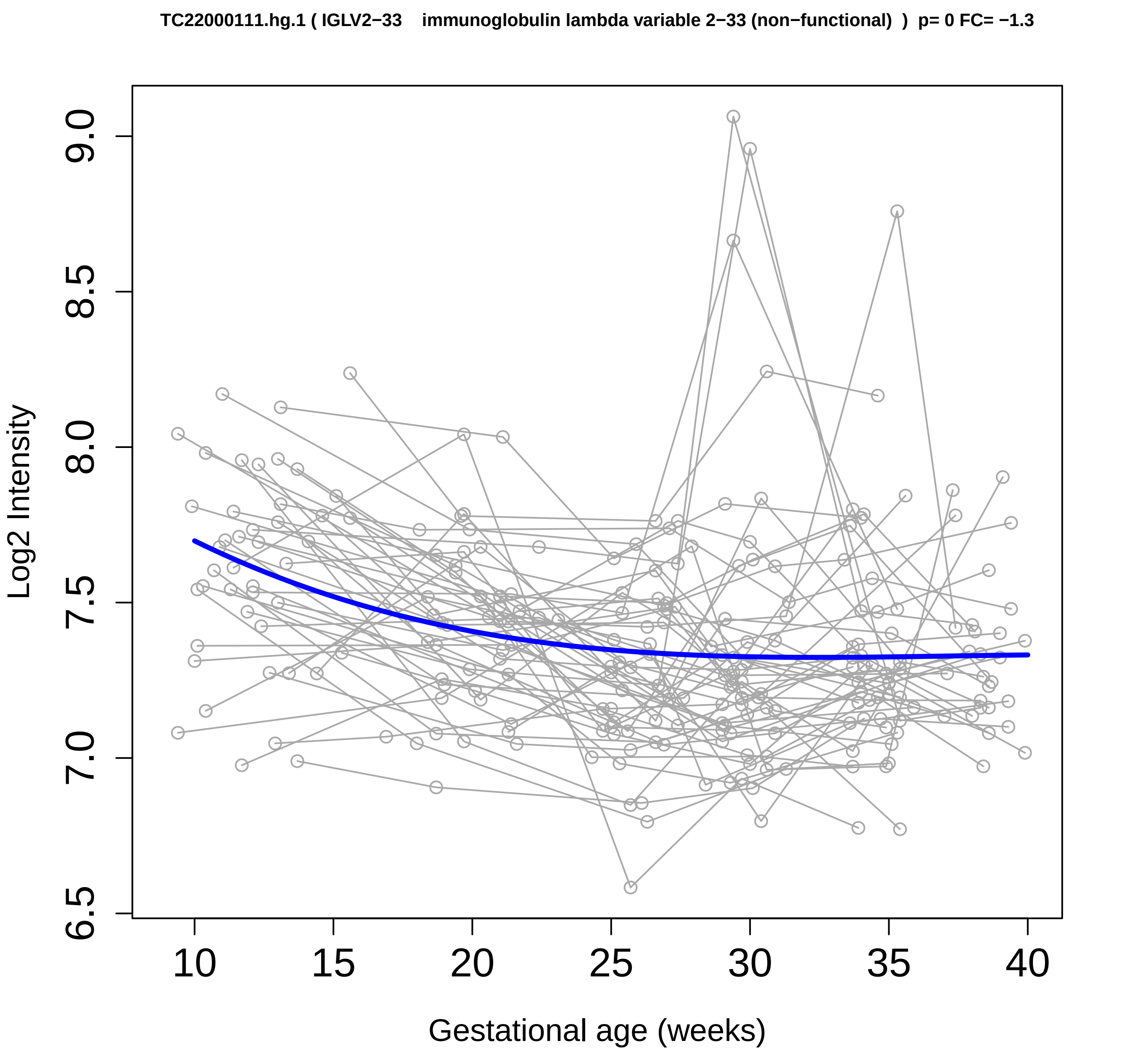

TC22000111.hg.1 (IGLV2−33   immunoglobulin lambda variable 2−33 (non−functional)) p= 0 FC= −1.3
Log2 Intensity
6.5
7.0
7.5
8.0
8.5
9.0
10
15
20
25
30
35
40
Gestational age (weeks)

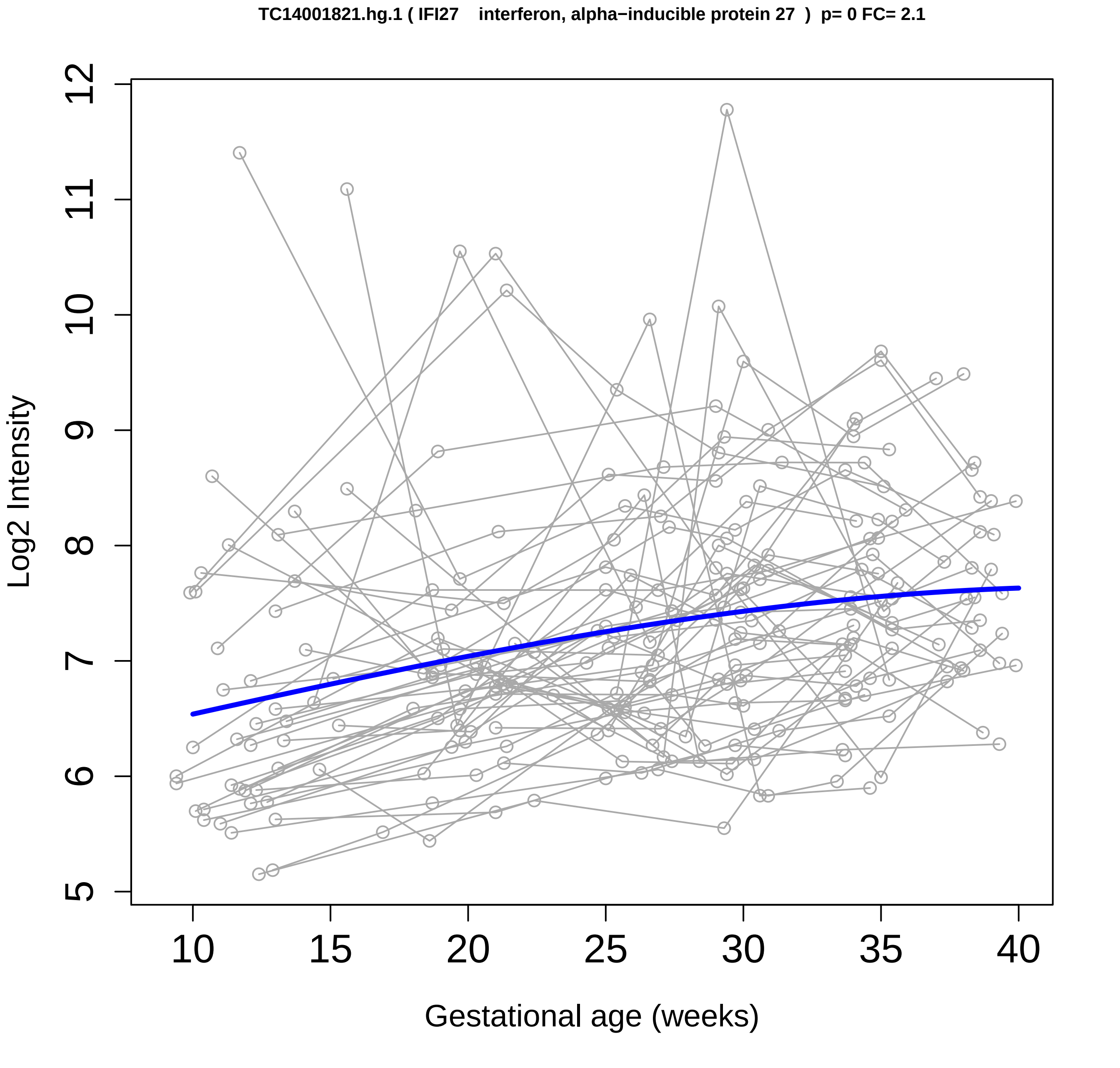

TC14001821.hg.1 (IFI27 interferon, alpha−inducible protein 27) p= 0 FC= 2.1
Log2 Intensity
5
6
7
8
9
10
11
12
10
15
20
25
30
35
40
Gestational age (weeks)

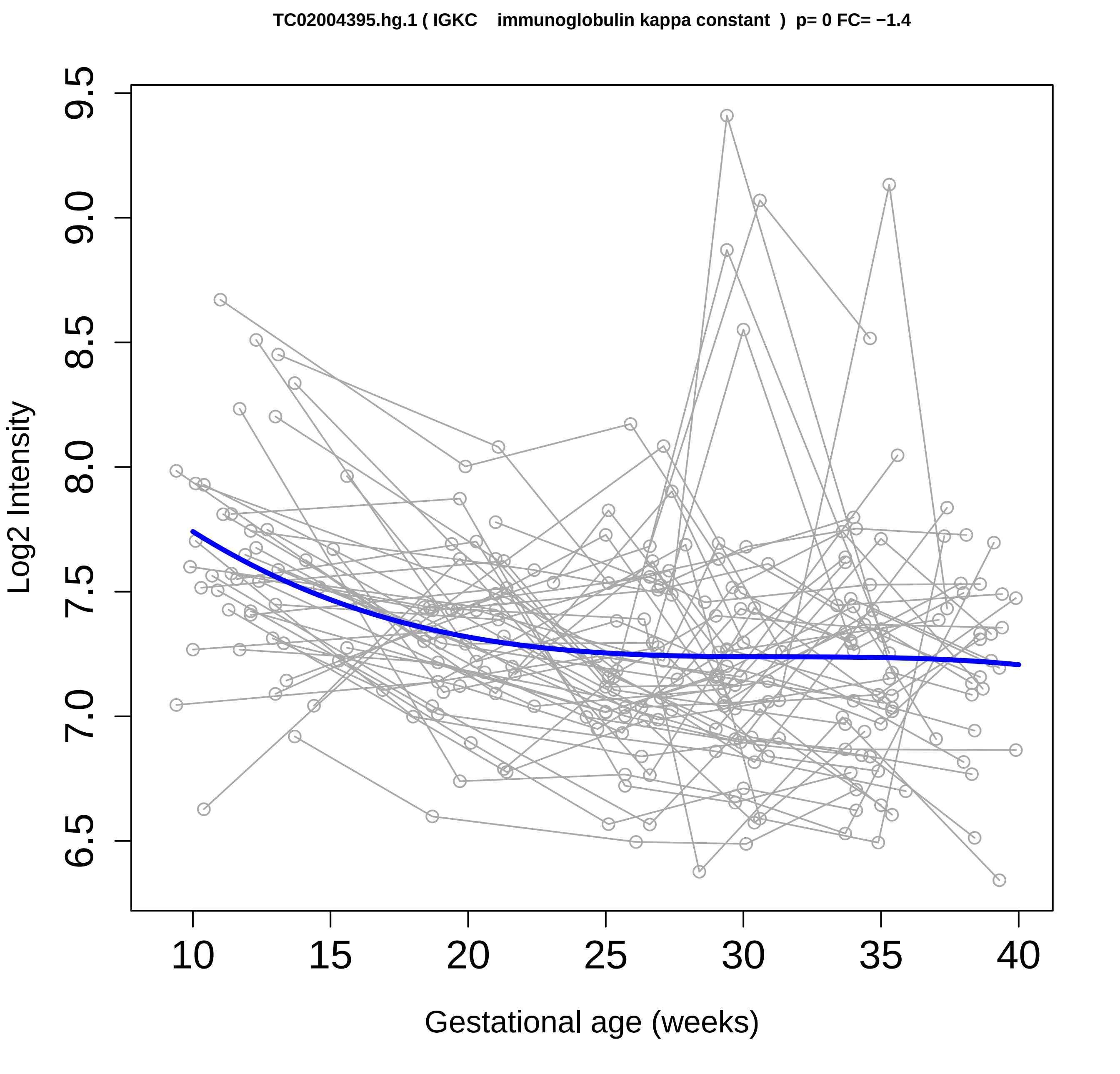

TC02004395.hg.1 (IGKC   immunoglobulin kappa constant) p= 0 FC= −1.4
Log2 Intensity
9.5
9.0
8.5
8.0
7.5
7.0
6.5
10
15
20
25
30
35
40
Gestational age (weeks)

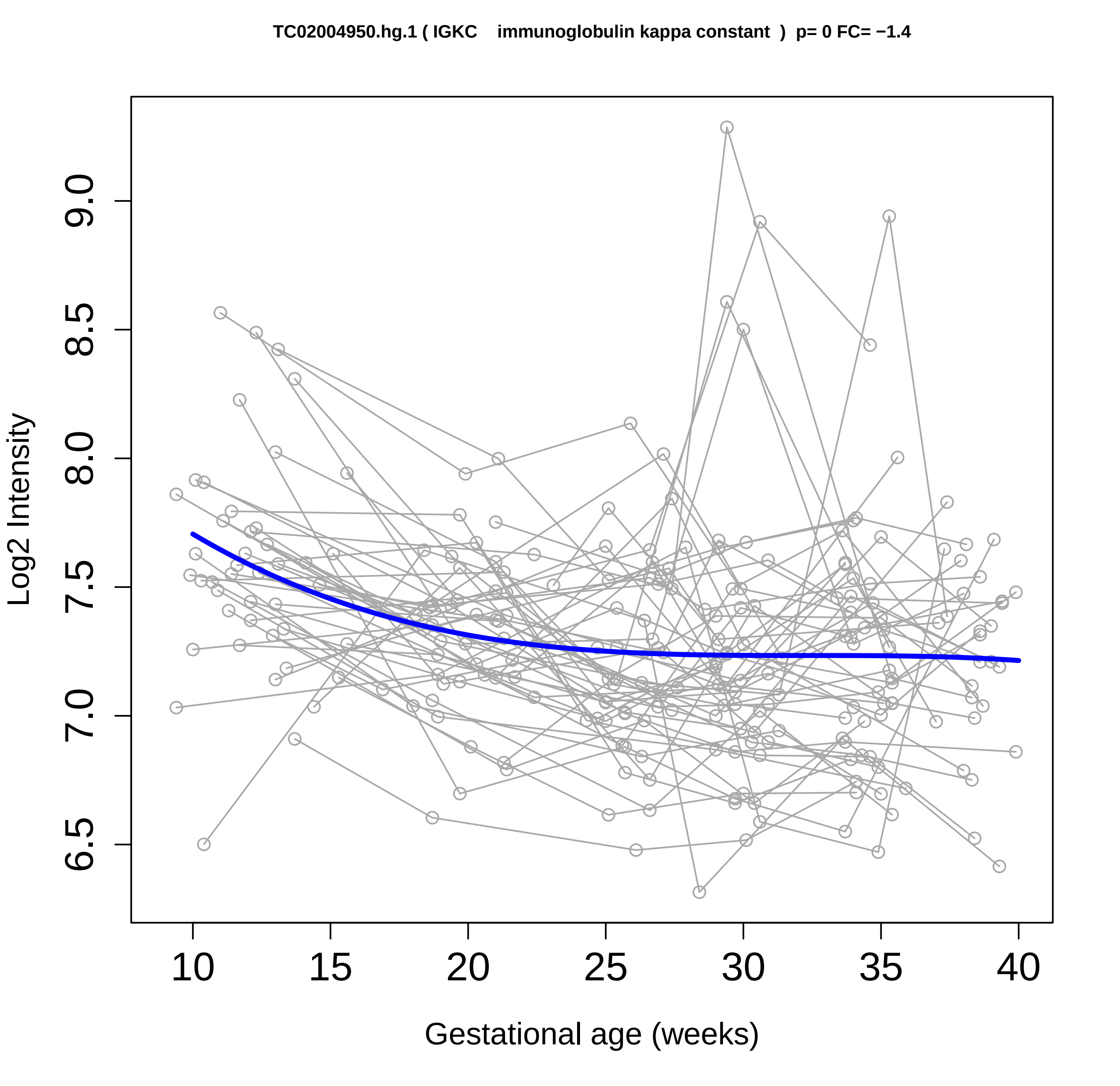

TC02004950.hg.1 (IGKC immunoglobulin kappa constant) p= 0 FC= −1.4
Log2 Intensity
6.5
7.0
7.5
8.0
8.5
9.0
10
15
20
25
30
35
40
Gestational age (weeks)

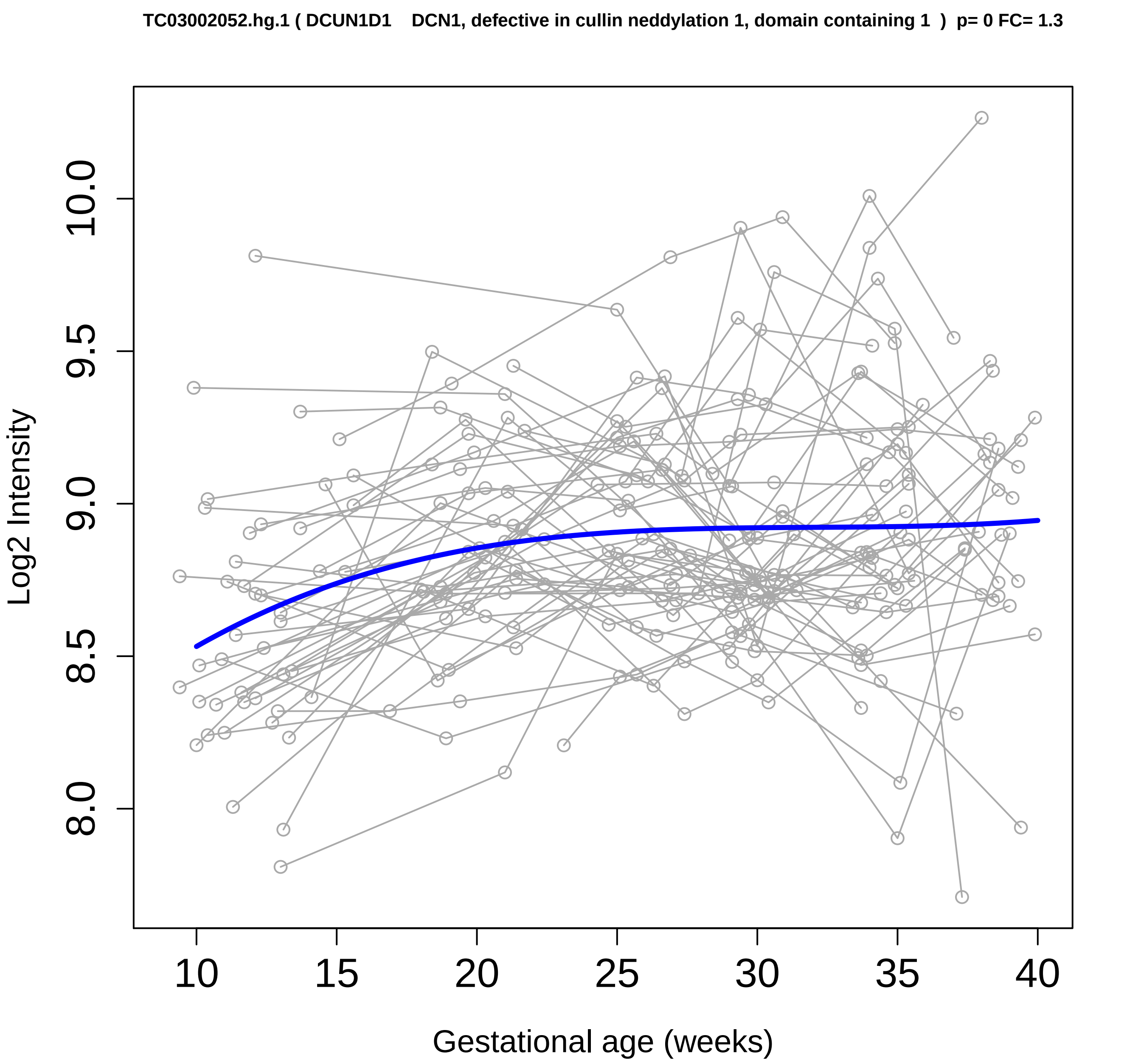

TC03002052.hg.1 (DCUN1D1 DCN1, defective in cullin neddylation 1, domain containing 1) p= 0 FC= 1.3
Log2 Intensity
8.0
8.5
9.0
9.5
10.0
10
15
20
25
30
35
40
Gestational age (weeks)

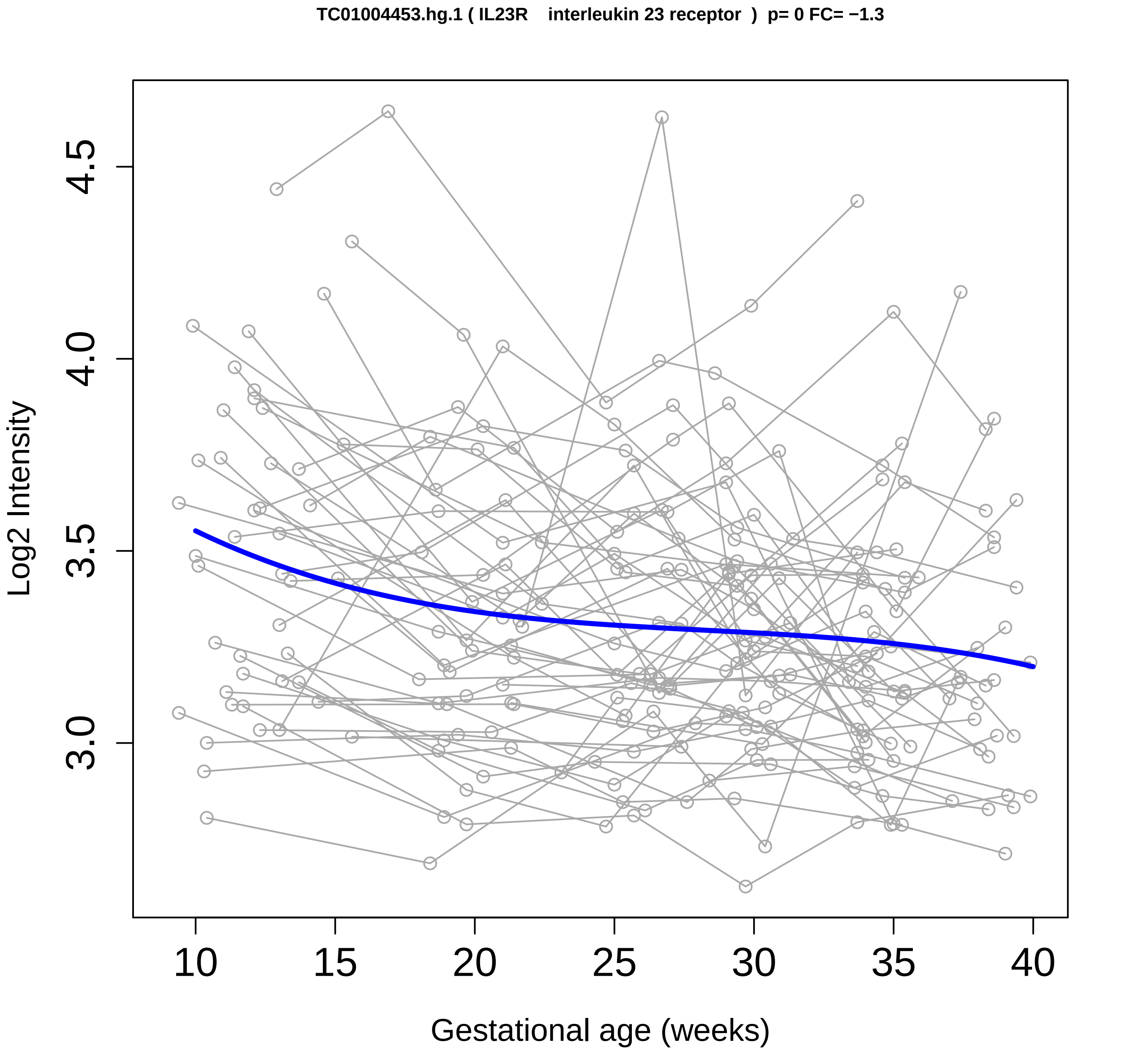
TC01004453.hg.1 (IL23R interleukin 23 receptor) p= 0 FC= −1.3
Log2 Intensity
3.0
3.5
4.0
4.5
10
15
20
25
30
35
40
Gestational age (weeks)

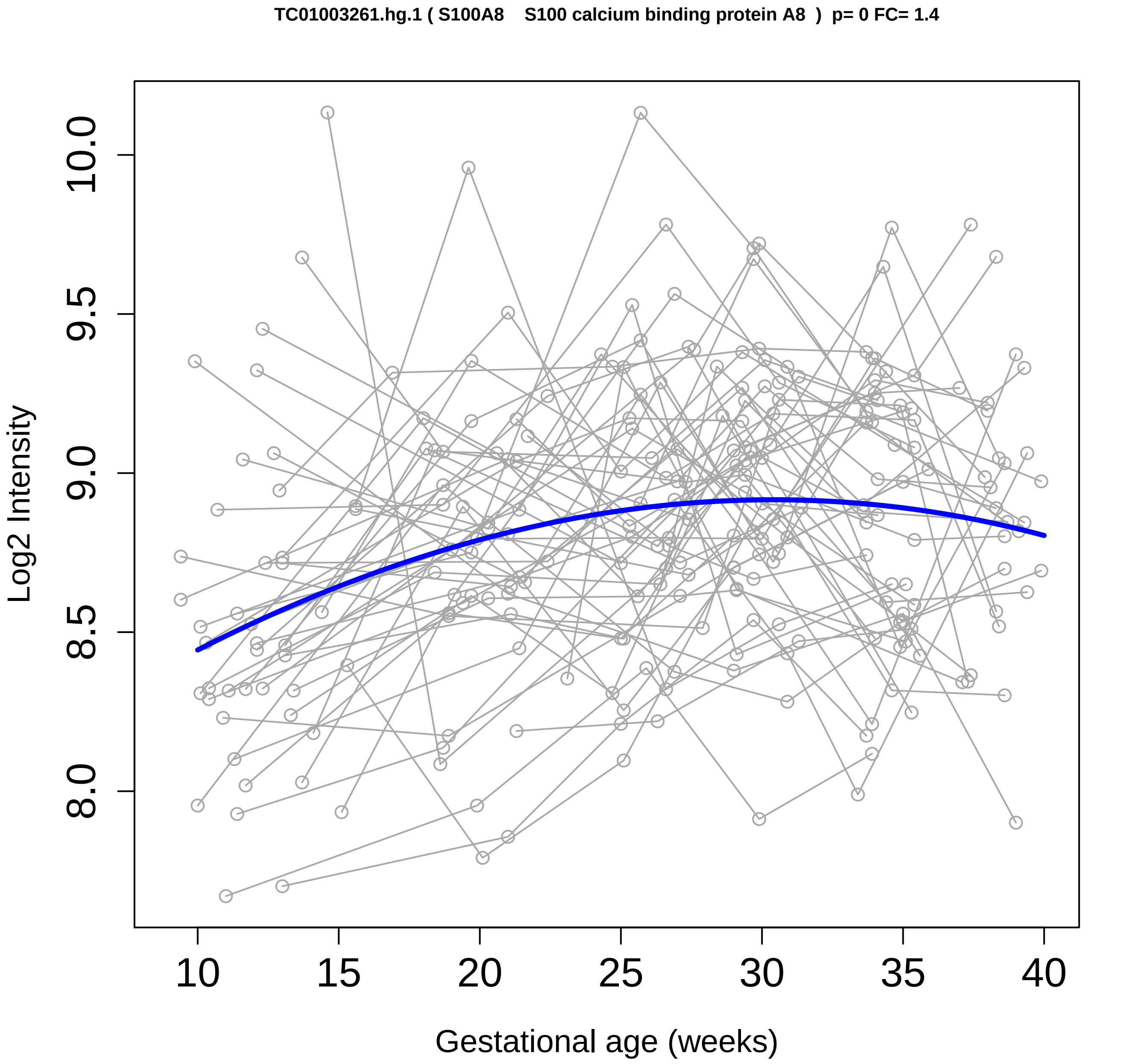
TC01003261.hg.1 (S100A8 S100 calcium binding protein A8) p= 0 FC= 1.4
Log2 Intensity
8.0
8.5
9.0
9.5
10.0
10
15
20
25
30
35
40
Gestational age (weeks)

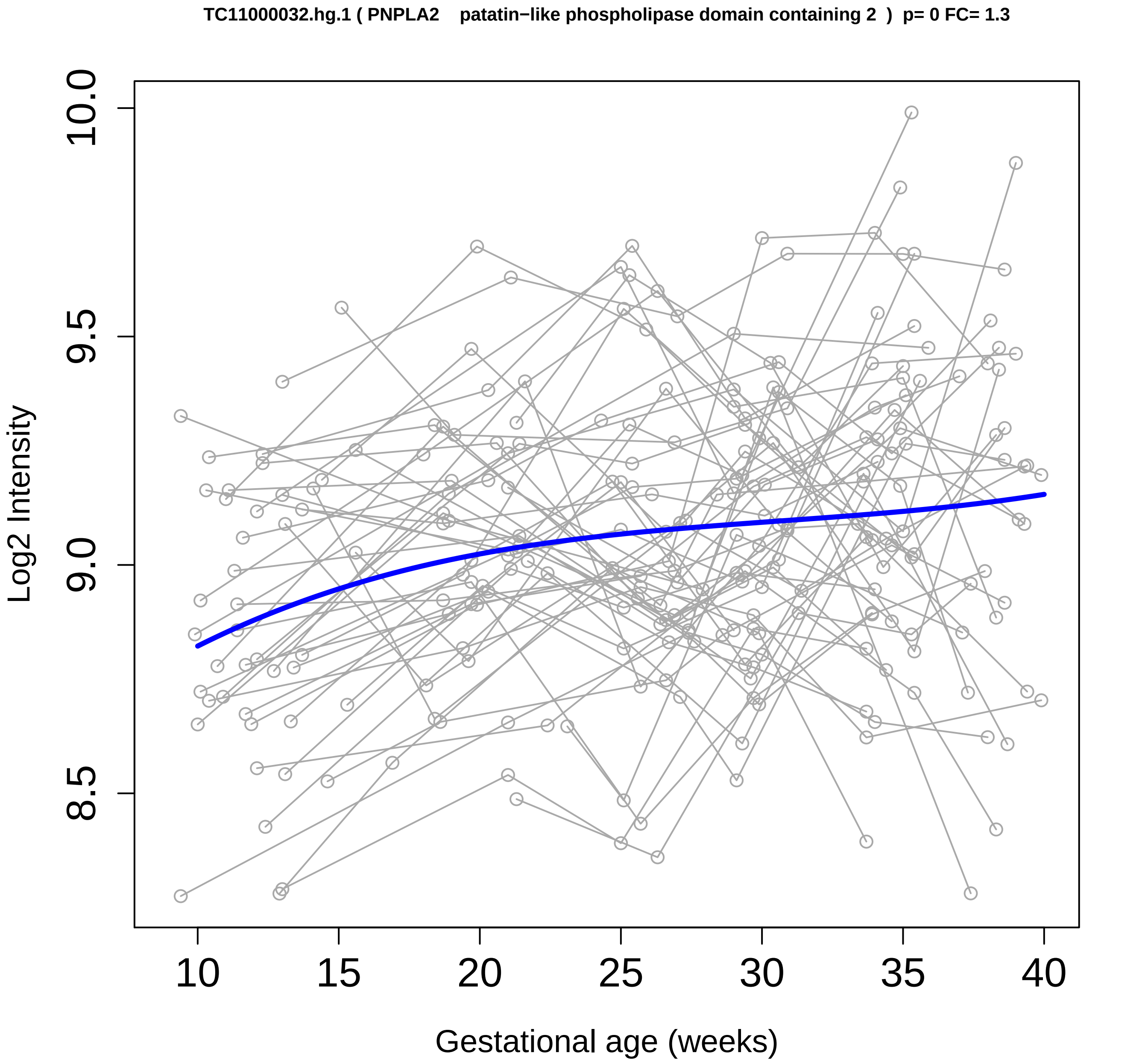

TC11000032.hg.1 (PNPLA2 patatin−like phospholipase domain containing 2) p= 0 FC= 1.3
Log2 Intensity
10.0
9.5
9.0
8.5
10
15
20
25
30
35
40
Gestational age (weeks)

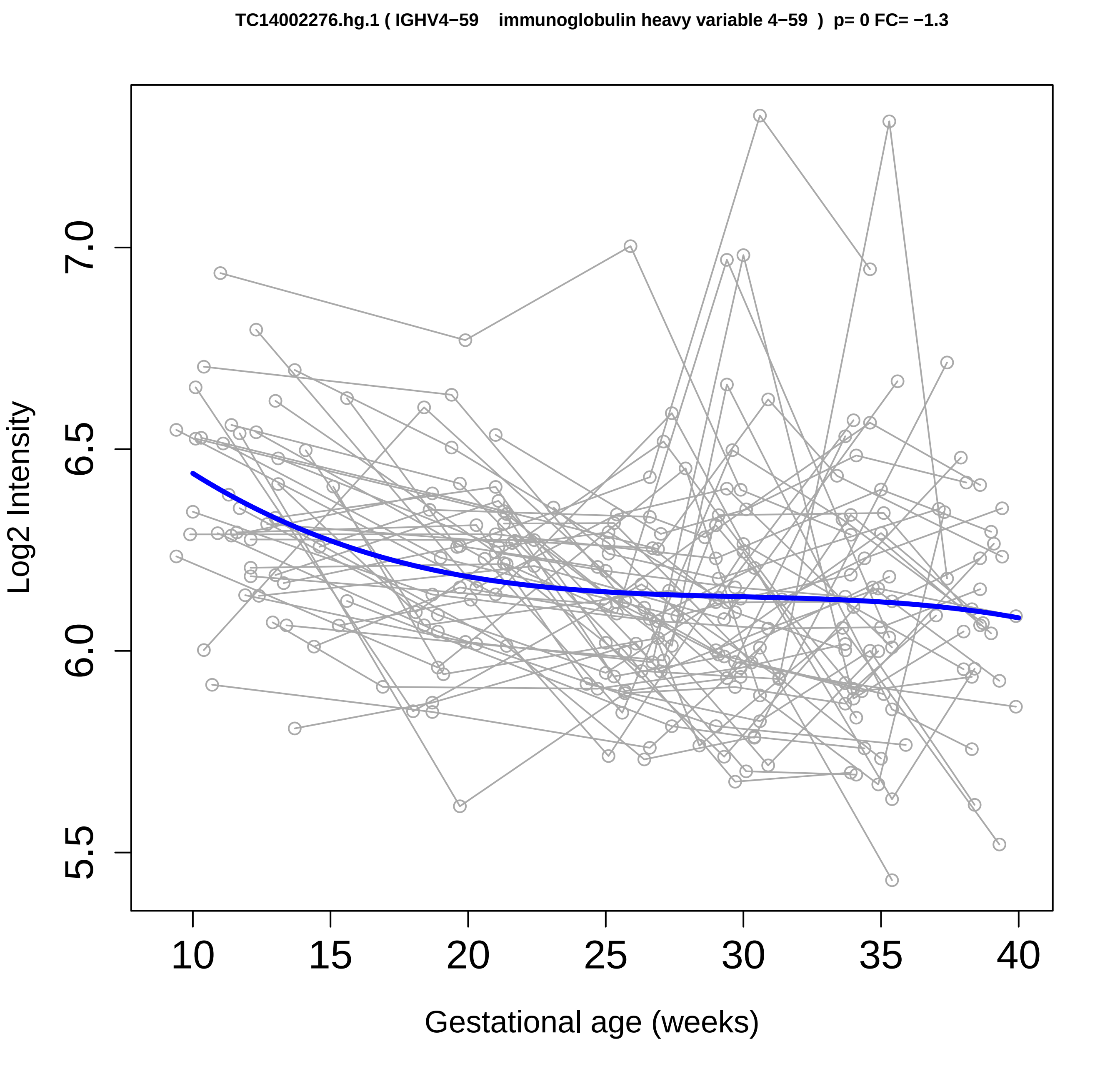
TC14002276.hg.1 (IGHV4−59   immunoglobulin heavy variable 4−59) p= 0 FC= −1.3
Log2 Intensity
5.5
6.0
6.5
7.0
10
15
20
25
30
35
40
Gestational age (weeks)

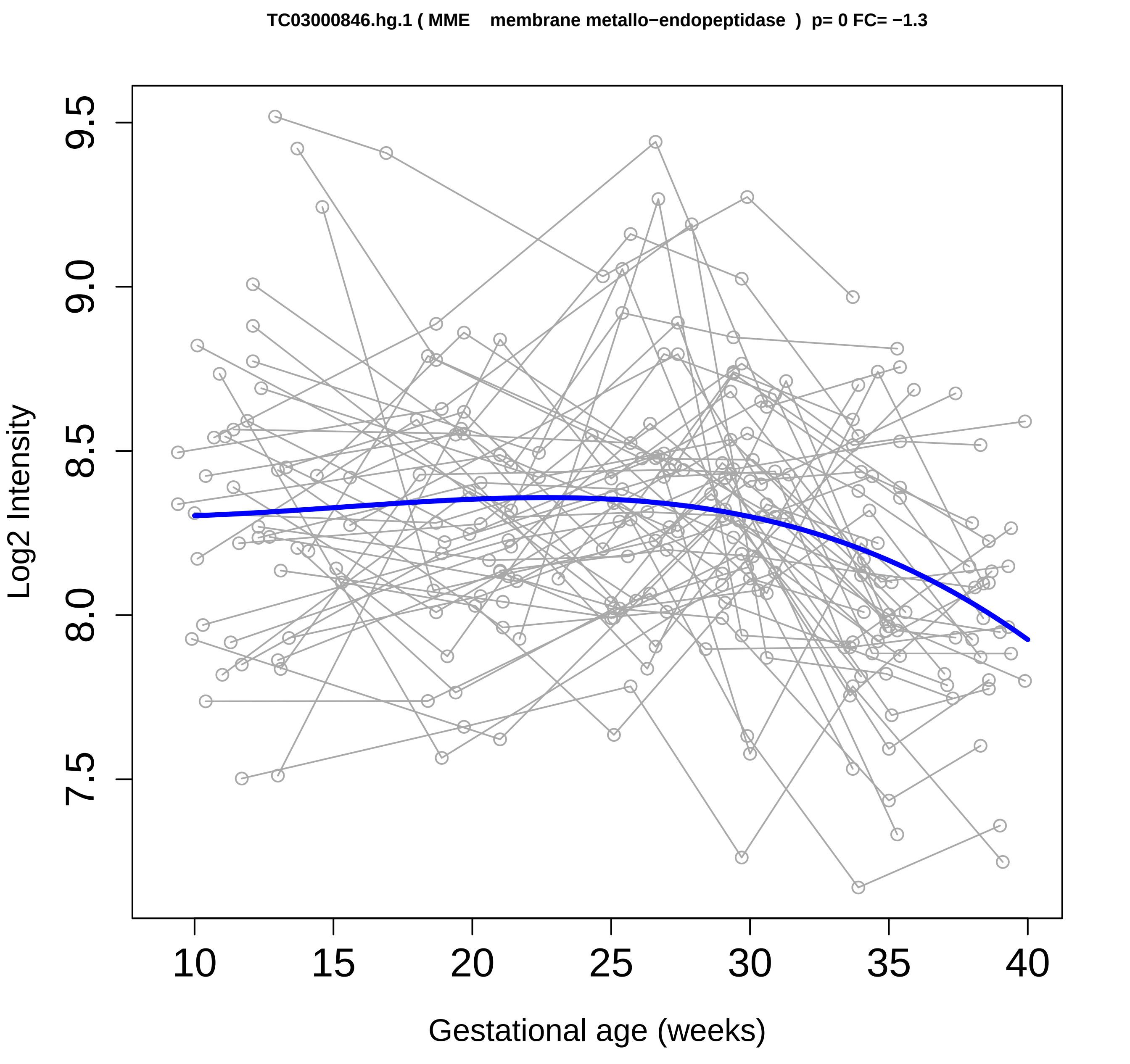
TC03000846.hg.1 (MME   membrane metallo−endopeptidase) p= 0 FC= −1.3
Log2 Intensity
9.5
9.0
8.5
8.0
7.5
10
15
20
25
30
35
40
Gestational age (weeks)

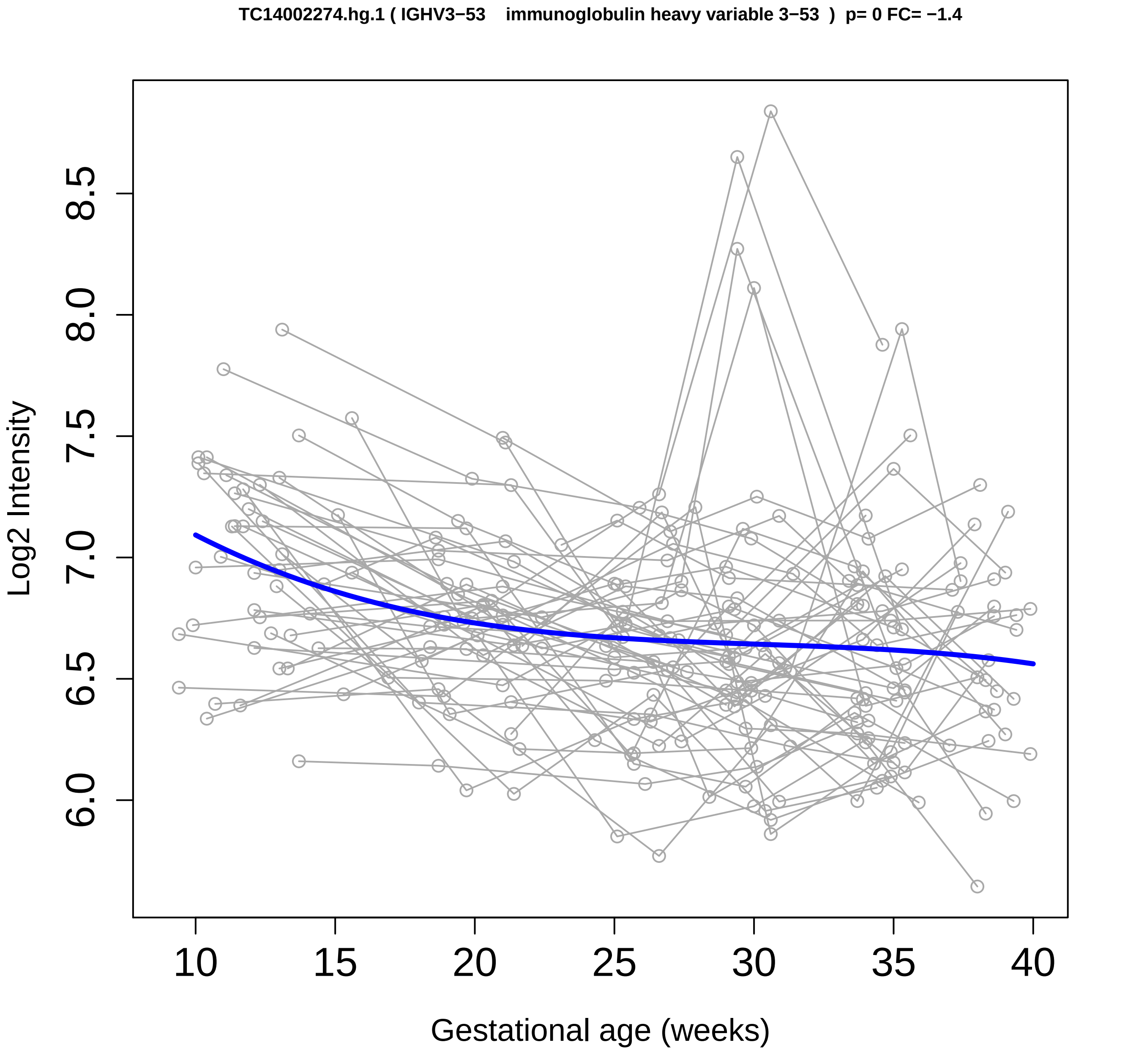

TC14002274.hg.1 (IGHV3−53 immunoglobulin heavy variable 3−53) p= 0 FC= −1.4
Log2 Intensity
6.0
6.5
7.0
7.5
8.0
8.5
10
15
20
25
30
35
40
Gestational age (weeks)

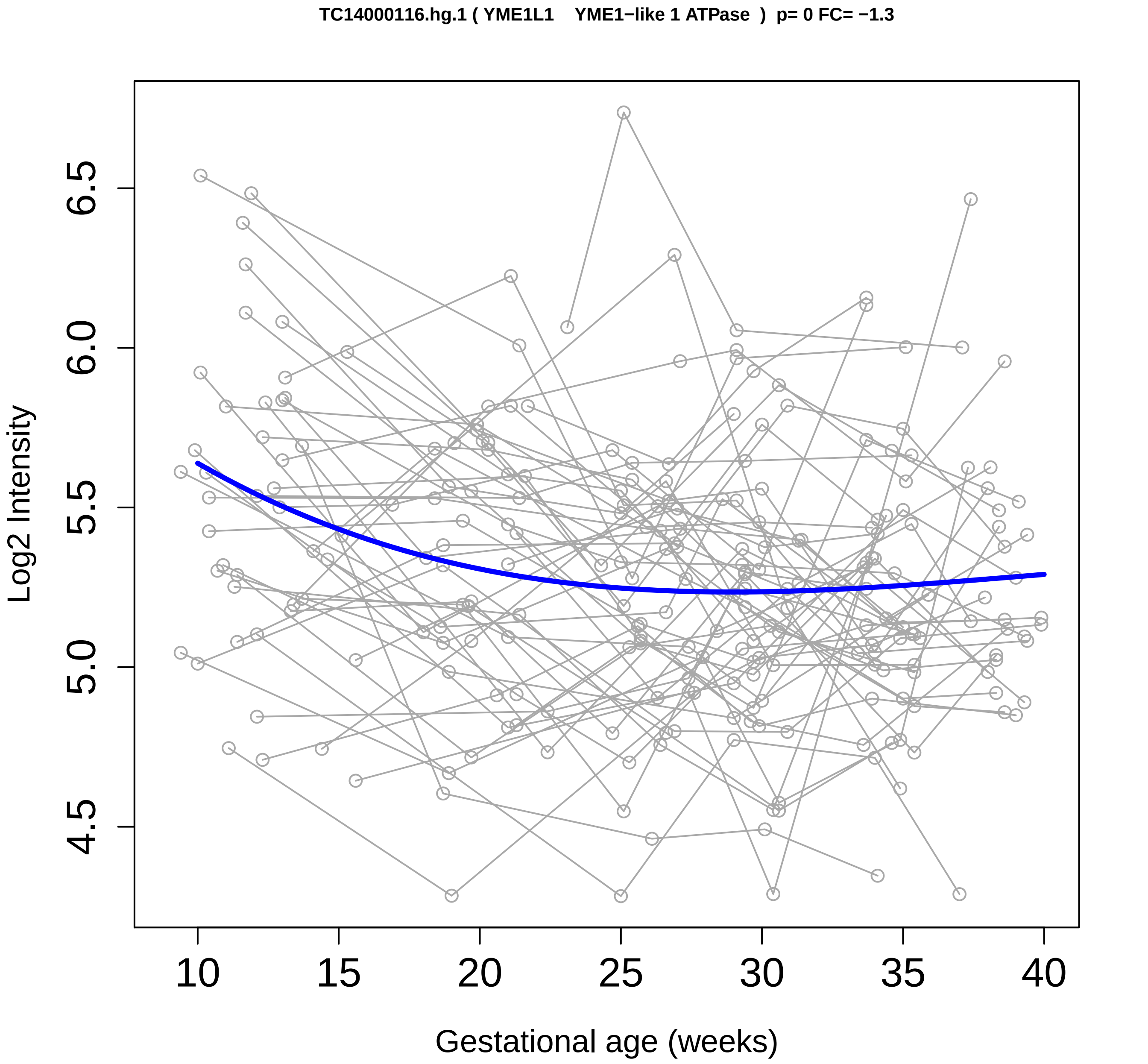
TC14000116.hg.1 (YME1L1 YME1−like 1 ATPase) p= 0 FC= −1.3
Log2 Intensity
6.5
6.0
5.5
5.0
4.5
10
15
20
25
30
35
40
Gestational age (weeks)

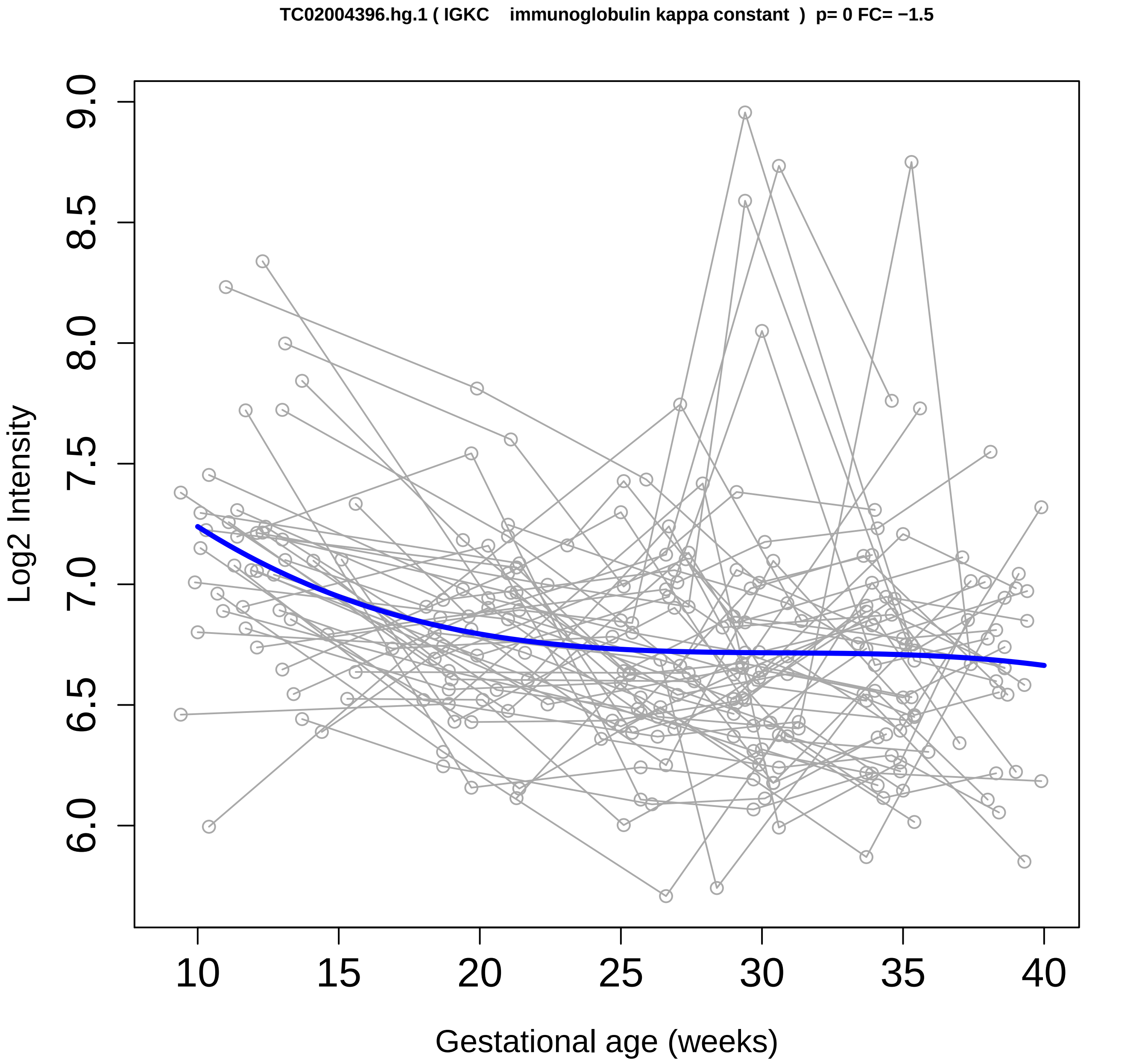

TC02004396.hg.1 (IGKC immunoglobulin kappa constant) p= 0 FC= −1.5
Log2 Intensity
6.0
6.5
7.0
7.5
8.0
8.5
9.0
10
15
20
25
30
35
40
Gestational age (weeks)

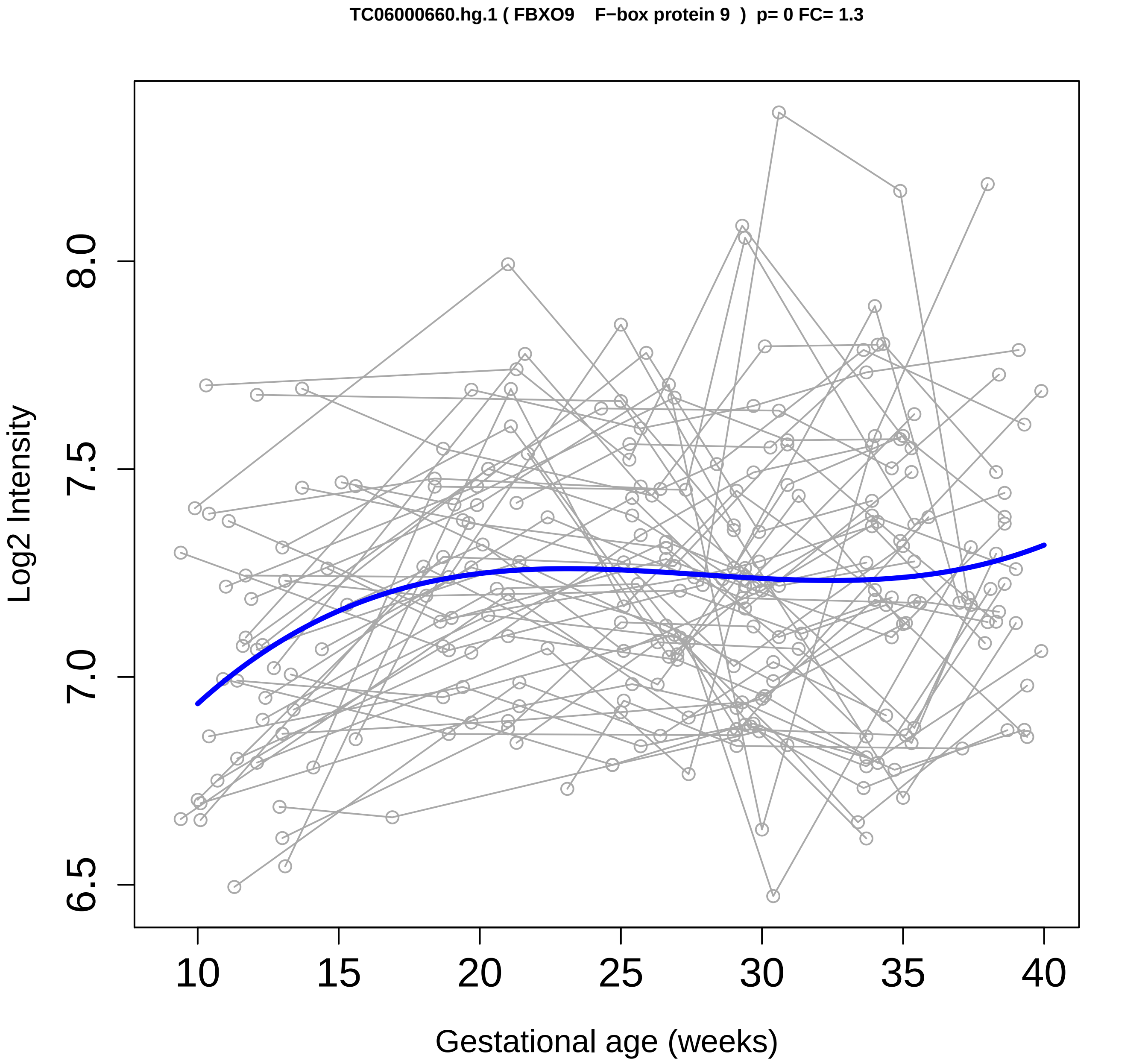
TC06000660.hg.1 (FBXO9 F−box protein 9) p= 0 FC= 1.3
Log2 Intensity
8.0
7.5
7.0
6.5
10
15
20
25
30
35
40
Gestational age (weeks)

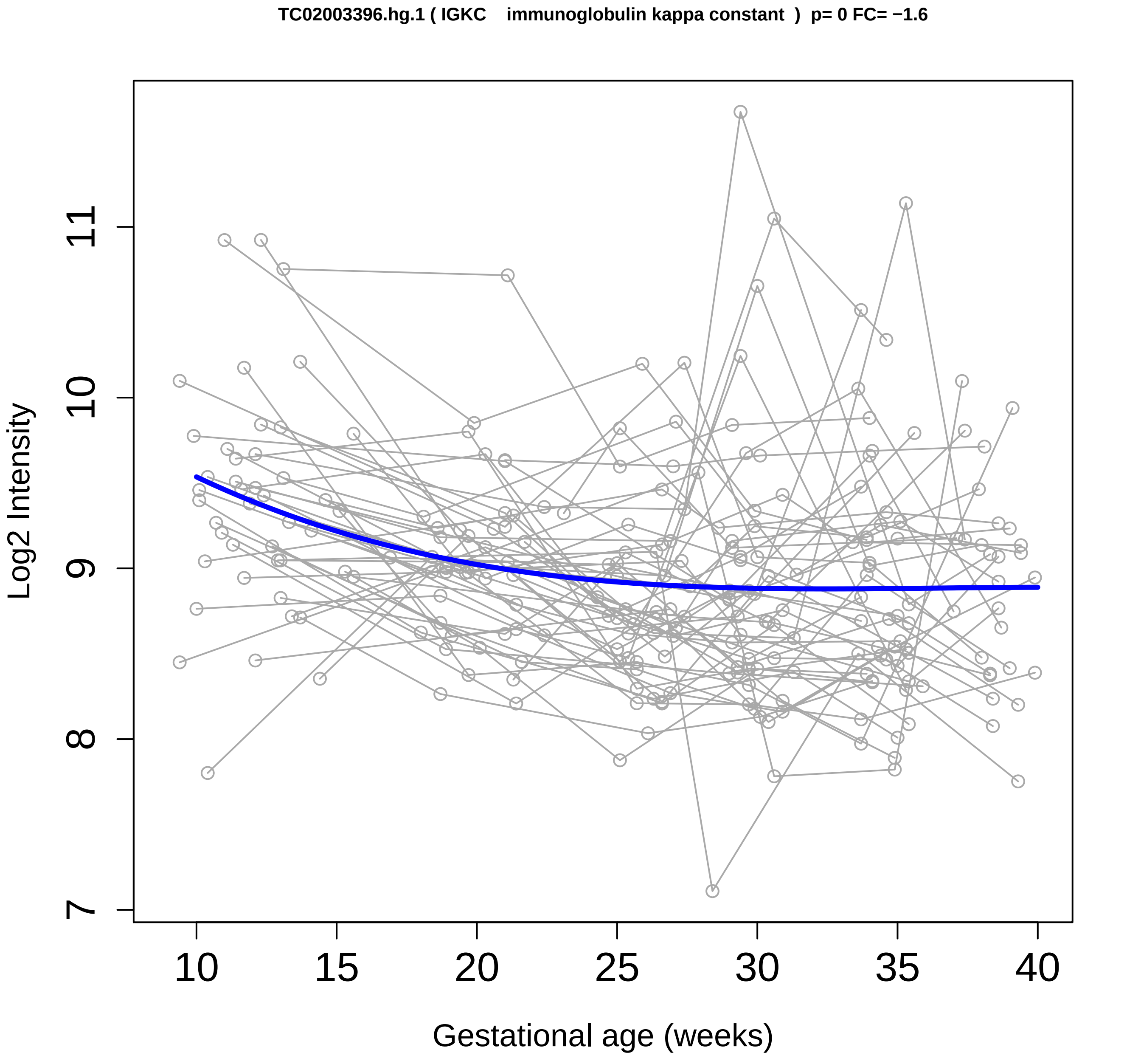

TC02003396.hg.1 (IGKC immunoglobulin kappa constant) p= 0 FC= −1.6
Log2 Intensity
7
8
9
10
11
10
15
20
25
30
35
40
Gestational age (weeks)

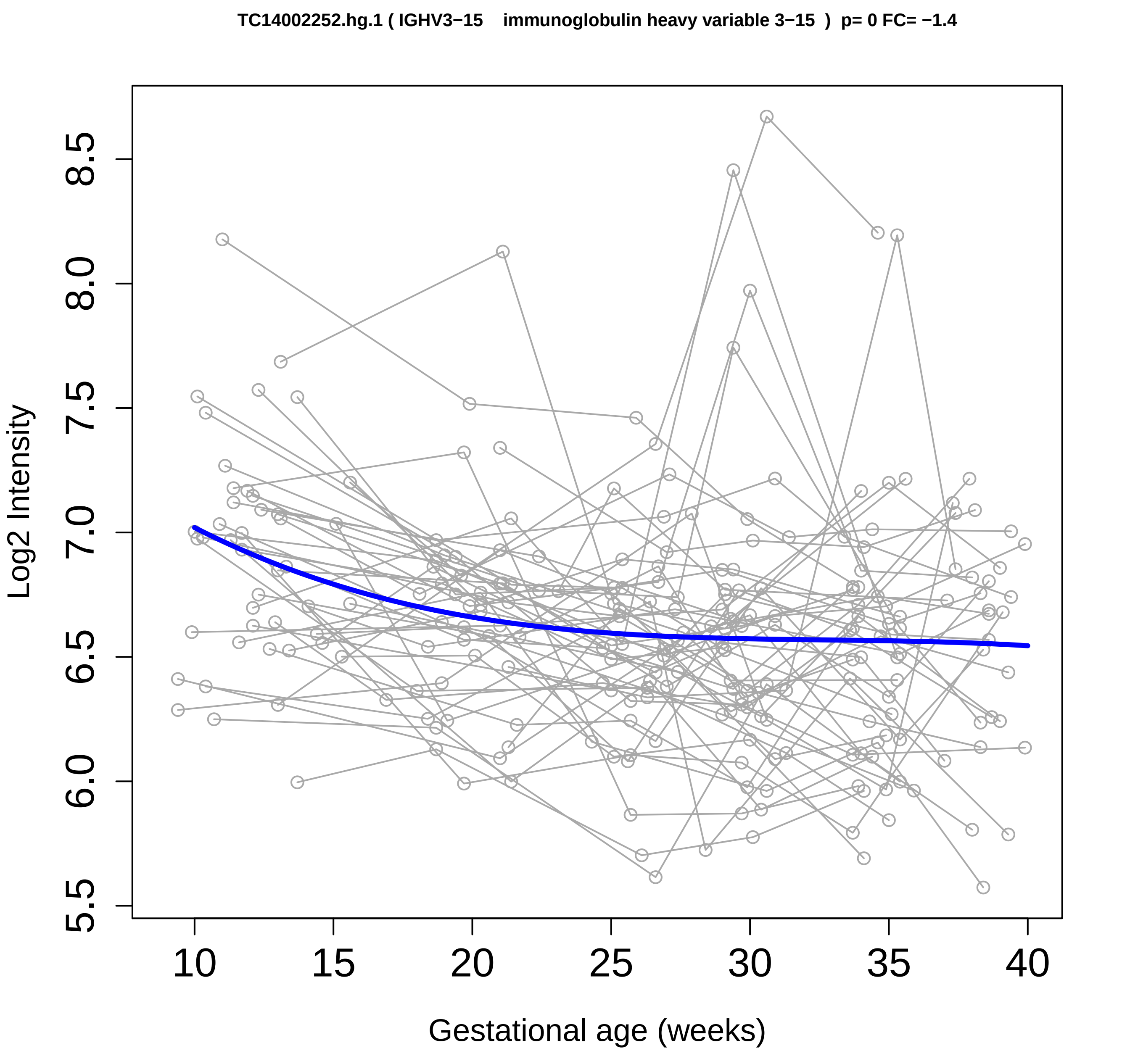
TC14002252.hg.1 (IGHV3−15 immunoglobulin heavy variable 3−15) p= 0 FC= −1.4
Log2 Intensity
5.5
6.0
6.5
7.0
7.5
8.0
8.5
10
15
20
25
30
35
40
Gestational age (weeks)

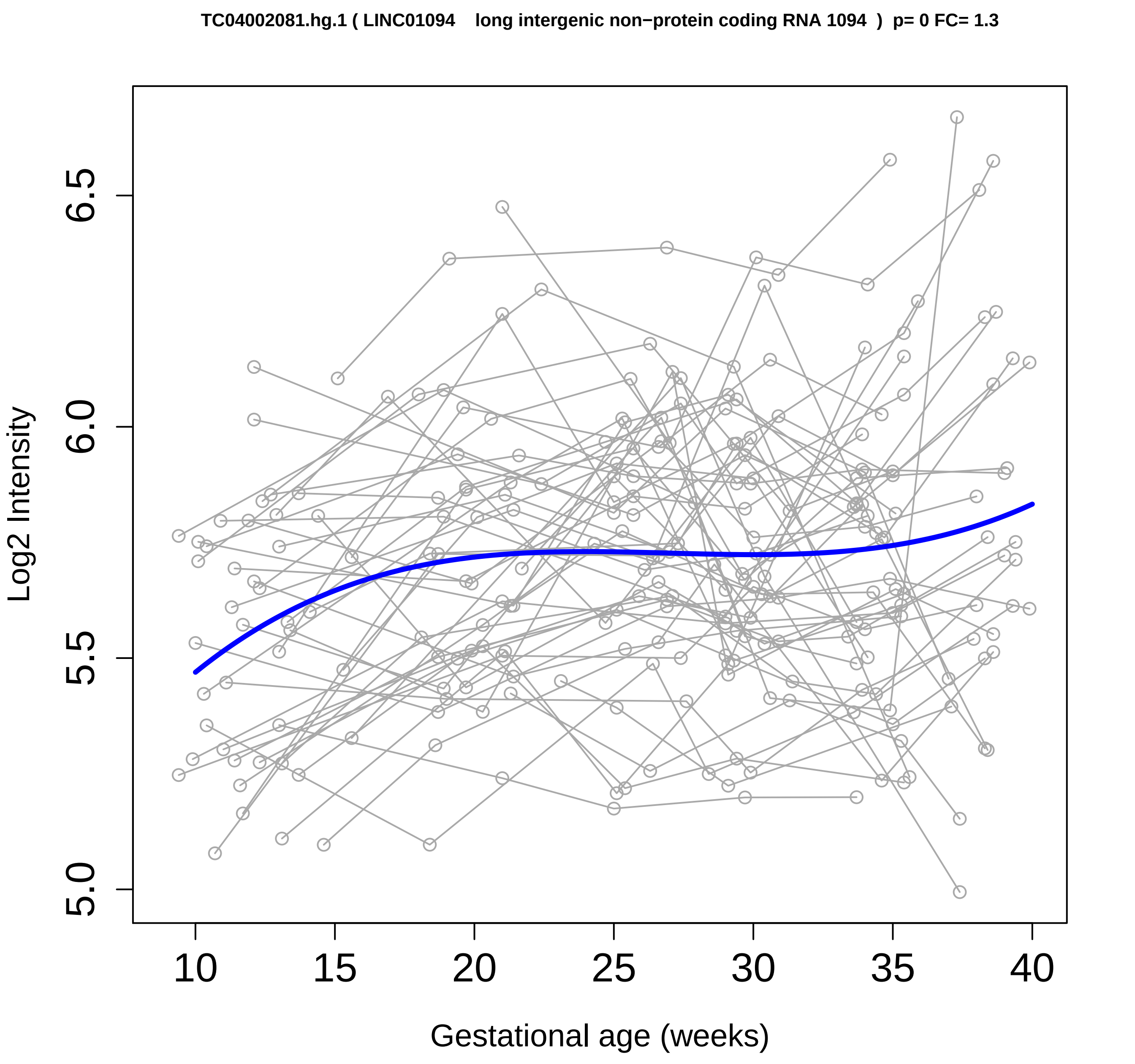

TC04002081.hg.1 (LINC01094 long intergenic non−protein coding RNA 1094) p= 0 FC= 1.3
Log2 Intensity
5.0
5.5
6.0
6.5
10
15
20
25
30
35
40
Gestational age (weeks)

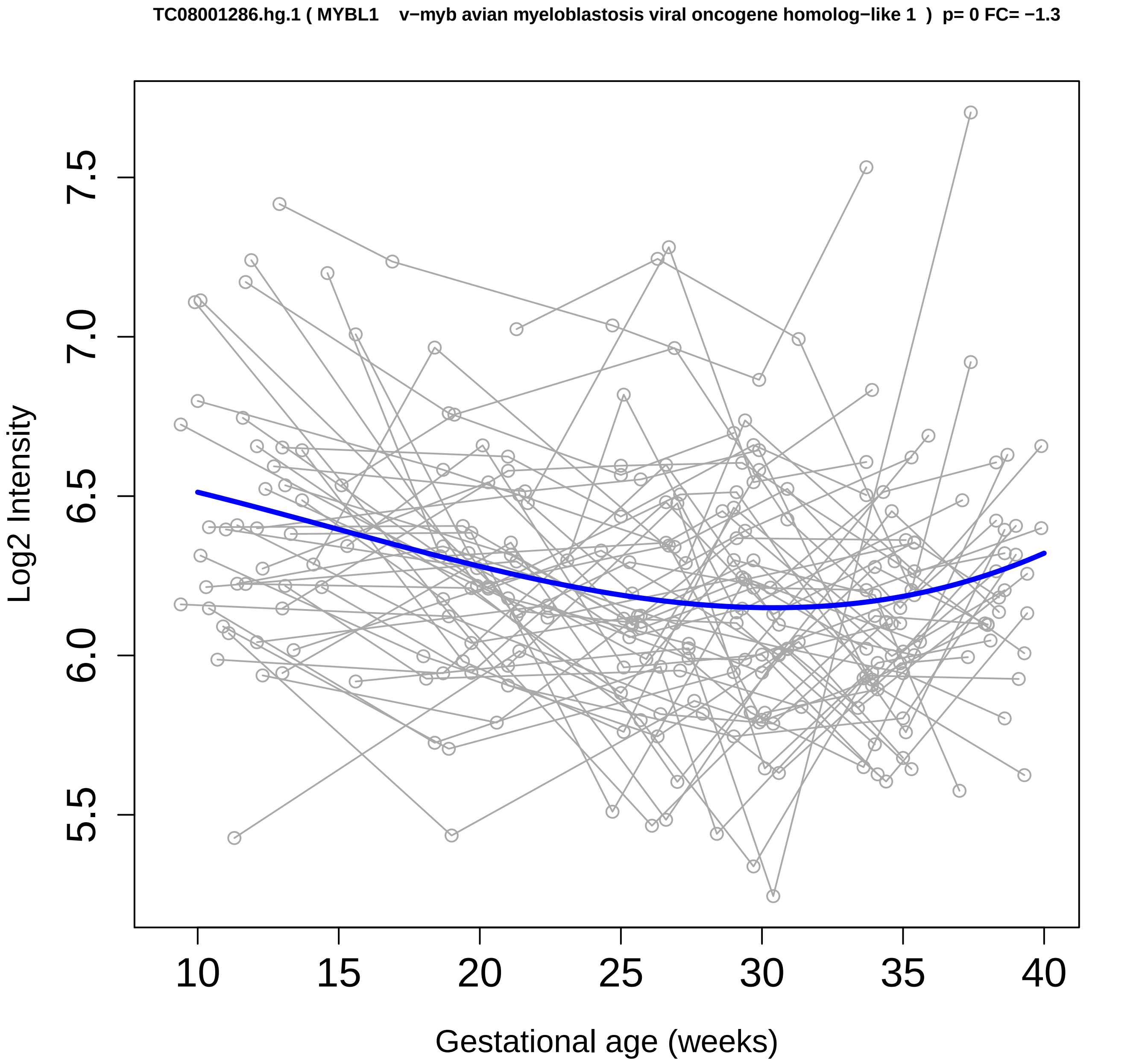

TC08001286.hg.1 (MYBL1 v−myb avian myeloblastosis viral oncogene homolog−like 1) p= 0 FC= −1.3
Log2 Intensity
5.5
6.0
6.5
7.0
7.5
10
15
20
25
30
35
40
Gestational age (weeks)

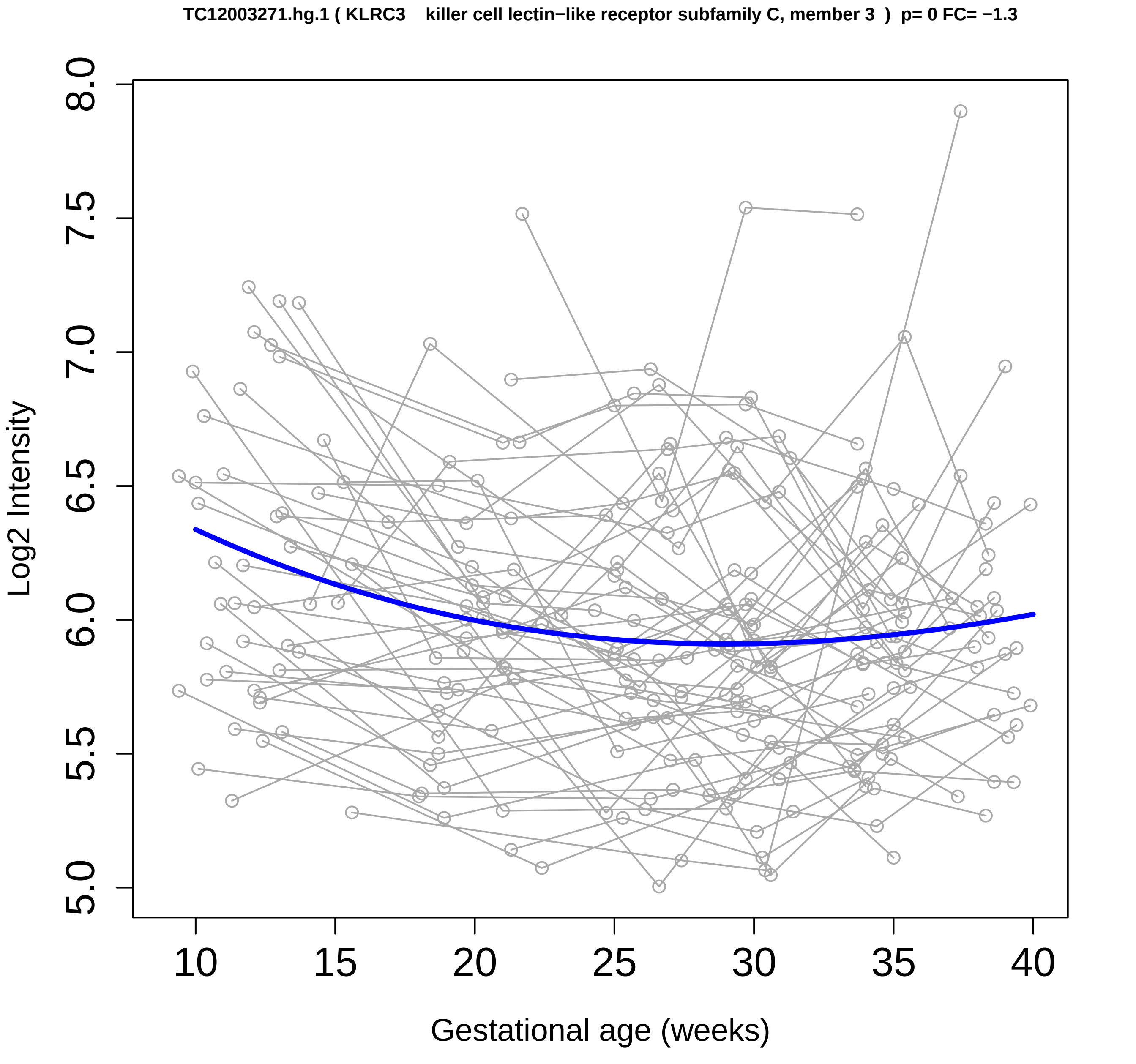
TC12003271.hg.1 (KLRC3 killer cell lectin−like receptor subfamily C, member 3) p= 0 FC= −1.3
Log2 Intensity
5.0
5.5
6.0
6.5
7.0
7.5
8.0
10
15
20
25
30
35
40
Gestational age (weeks)

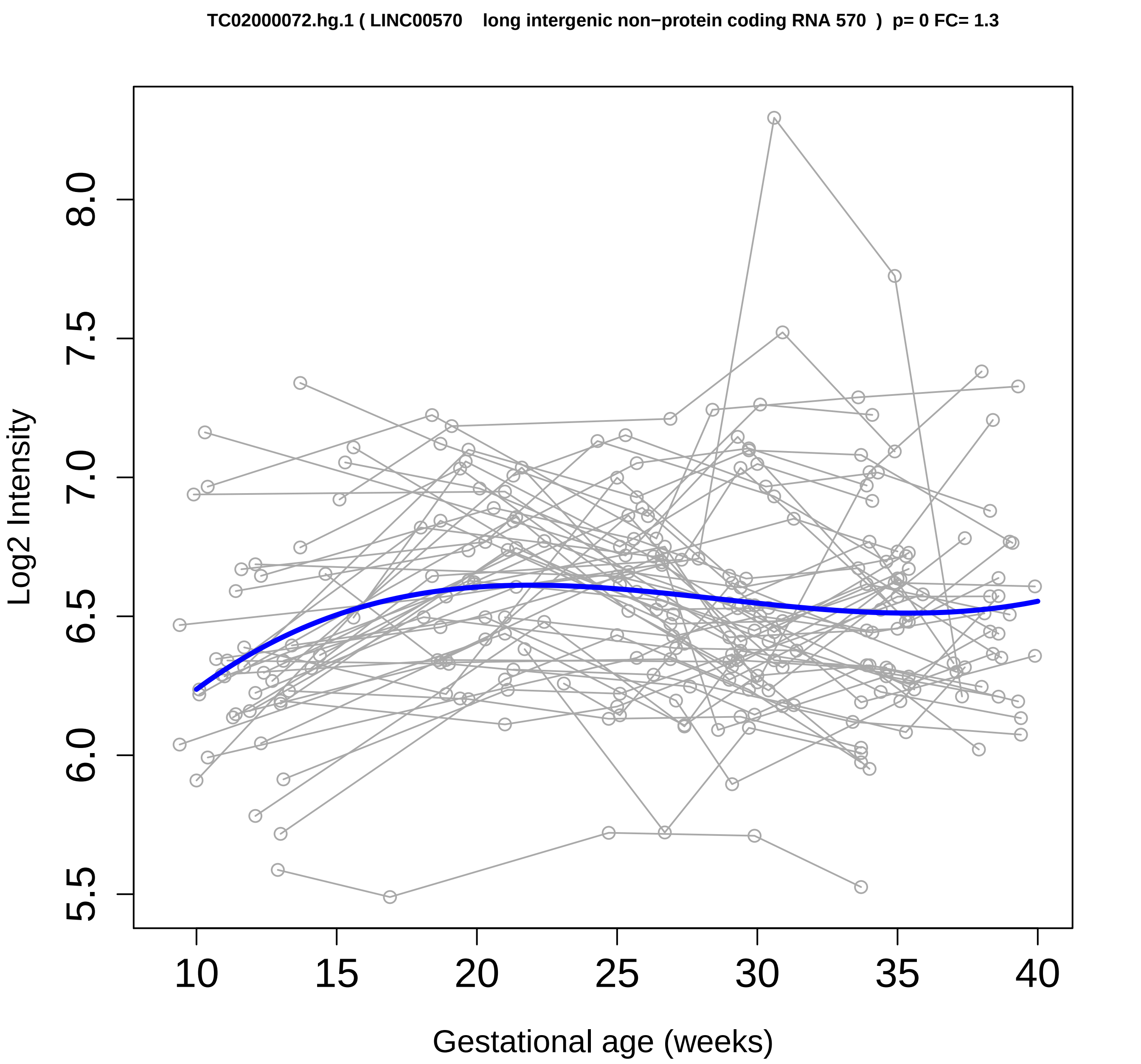

TC02000072.hg.1 (LINC00570   long intergenic non−protein coding RNA 570) p= 0 FC= 1.3
Log2 Intensity
5.5
6.0
6.5
7.0
7.5
8.0
10
15
20
25
30
35
40
Gestational age (weeks)

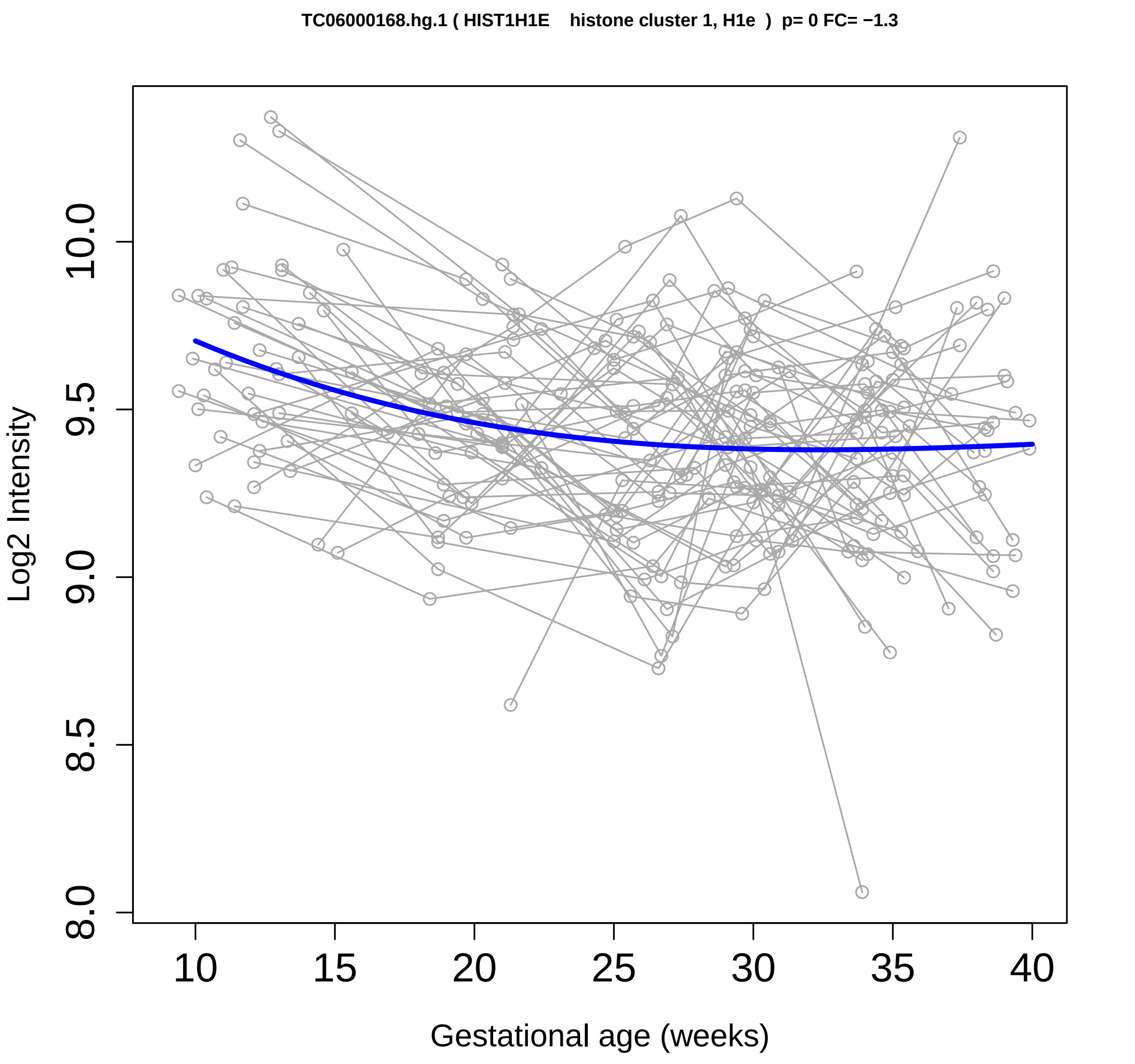
TC06000168.hg.1 (HIST1H1E histone cluster 1, H1e) p= 0 FC= −1.3
Log2 Intensity
8.0
8.5
9.0
9.5
10.0
10
15
20
25
30
35
40
Gestational age (weeks)

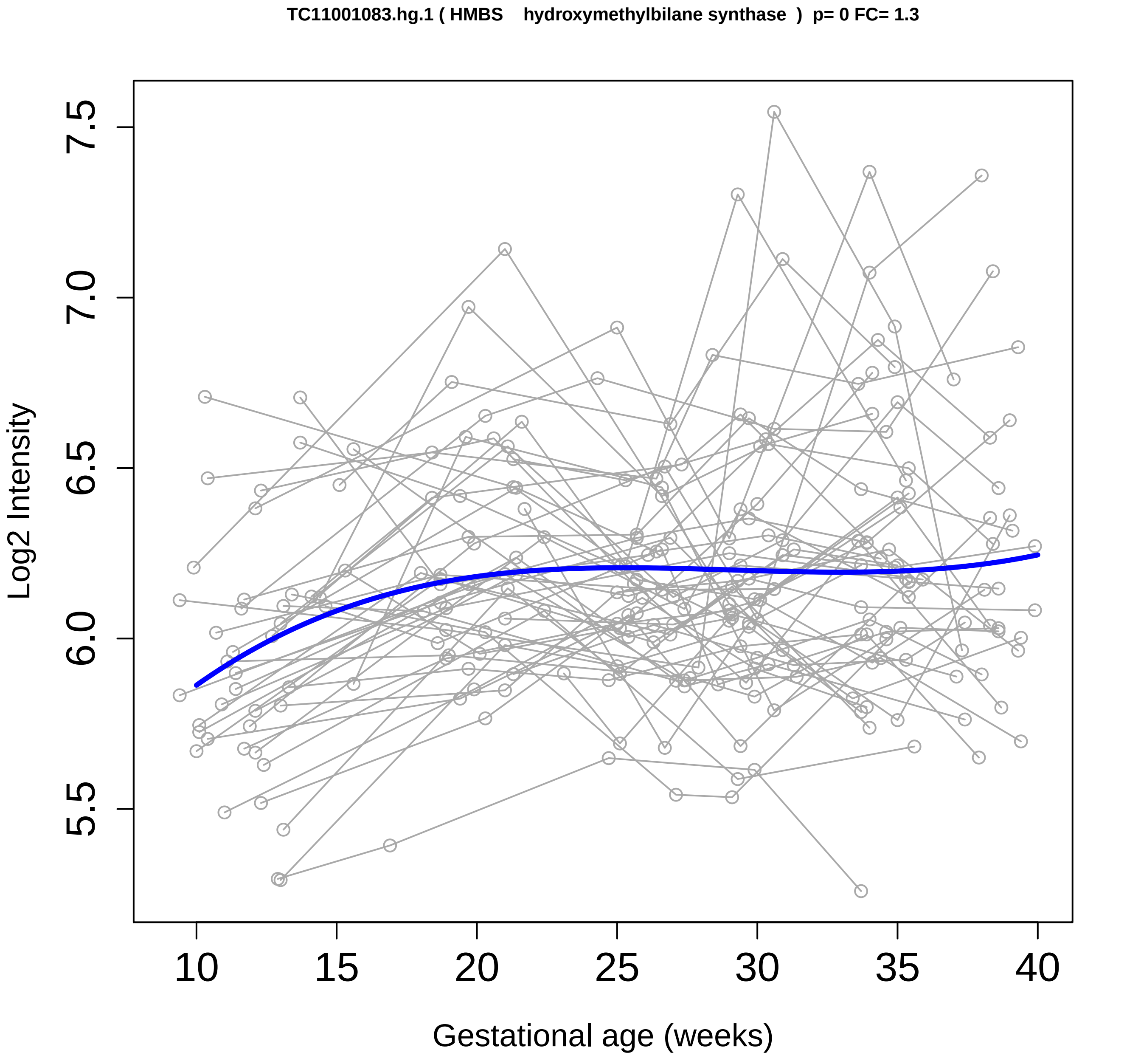

TC11001083.hg.1 (HMBS hydroxymethylbilane synthase) p= 0 FC= 1.3
Log2 Intensity
7.5
7.0
6.5
6.0
5.5
10
15
20
25
30
35
40
Gestational age (weeks)

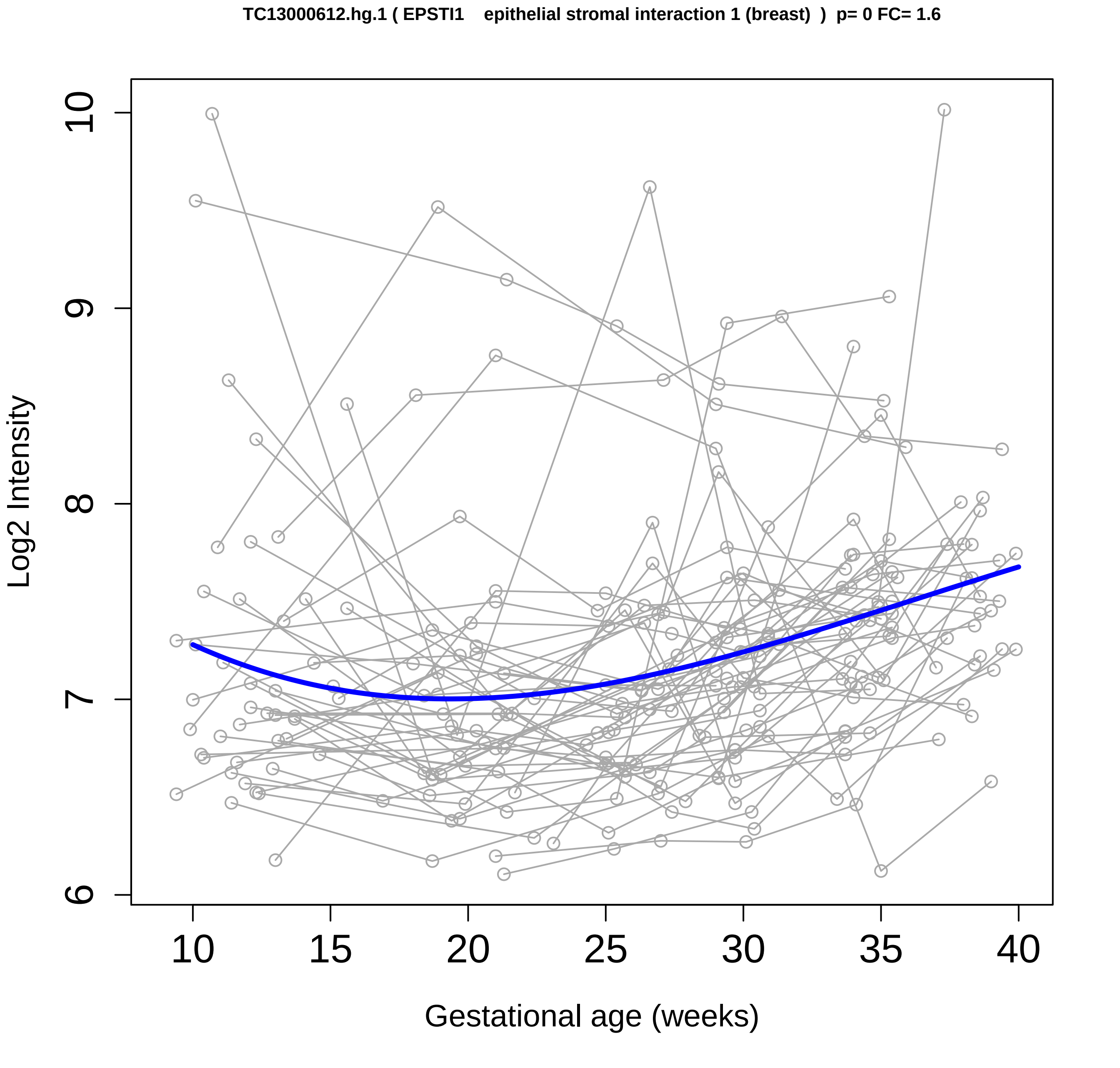
TC13000612.hg.1 (EPSTI1 epithelial stromal interaction 1 (breast)) p= 0 FC= 1.6
Log2 Intensity
6
7
8
9
10
10
15
20
25
30
35
40
Gestational age (weeks)

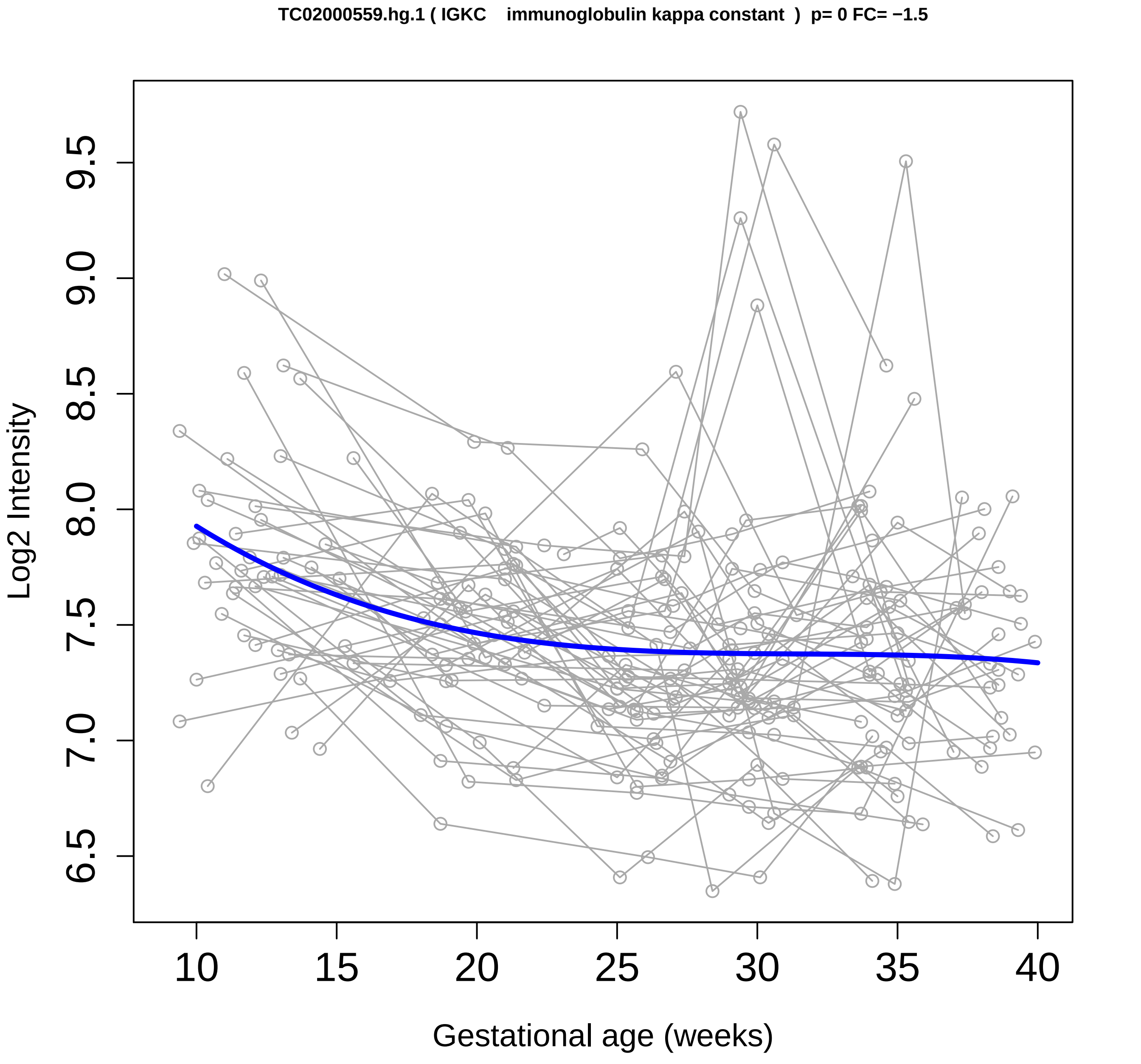
TC02000559.hg.1 (IGKC immunoglobulin kappa constant) p= 0 FC= −1.5
Log2 Intensity
6.5
7.0
7.5
8.0
8.5
9.0
9.5
10
15
20
25
30
35
40
Gestational age (weeks)

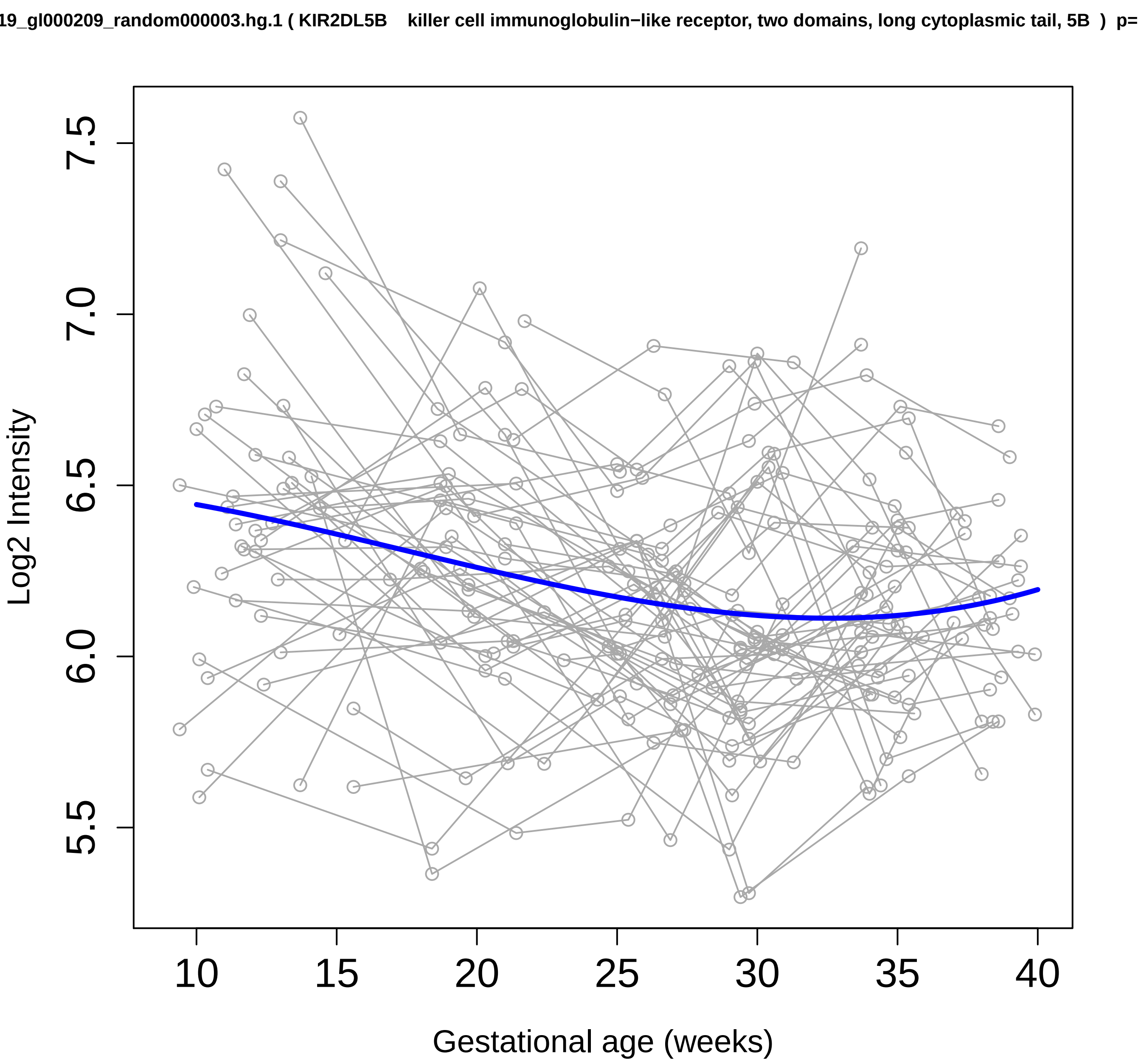
19_gl000209_random000003.hg.1 (KIR2DL5B killer cell immunoglobulin−like receptor, two domains, long cytoplasmic tail, 5B) p=
Log2 Intensity
7.5
7.0
6.5
6.0
5.5
10
15
20
25
30
35
40
Gestational age (weeks)

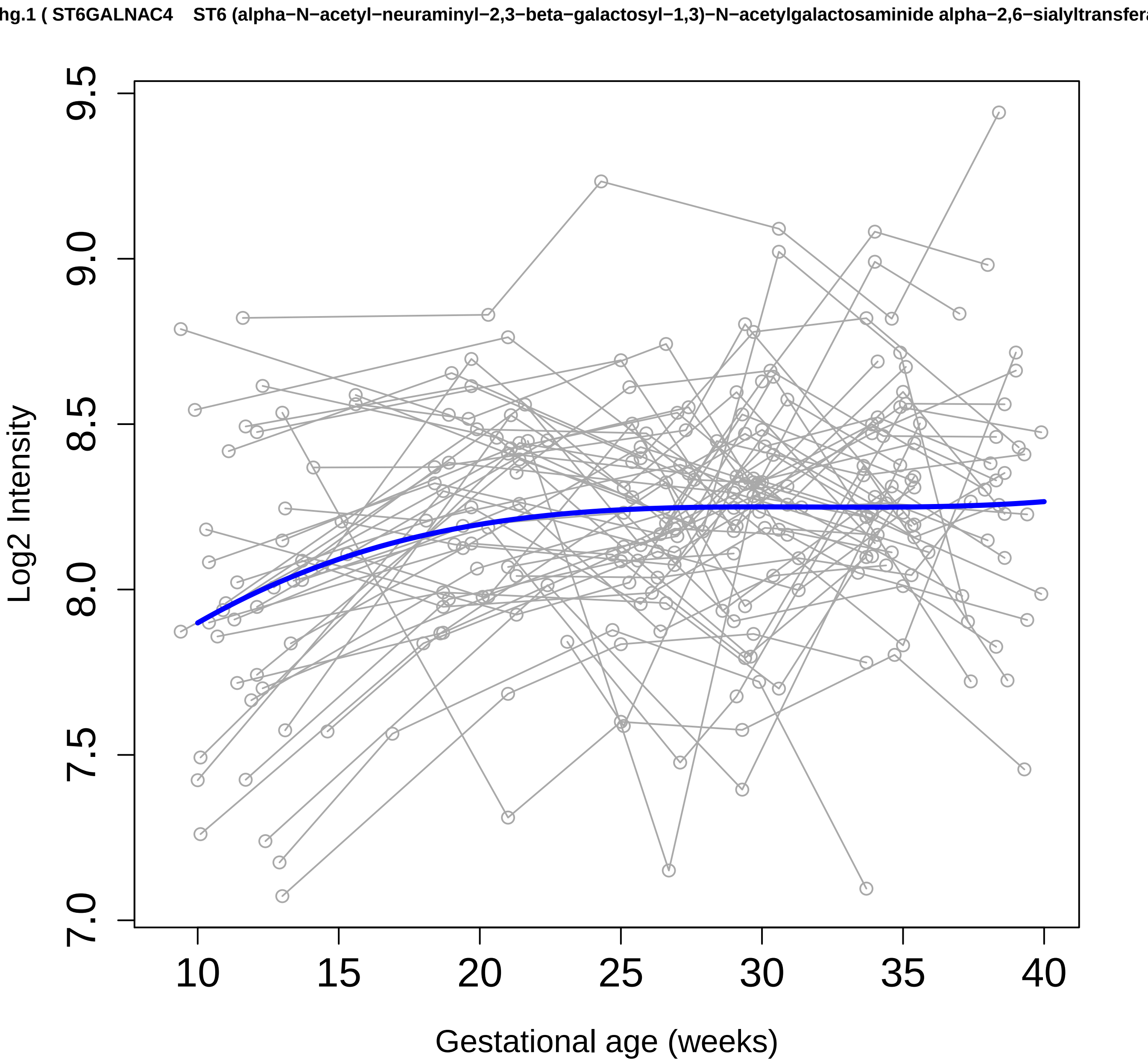
hg.1 (ST6GALNAC4   ST6 (alpha−N−acetyl−neuraminyl−2,3−beta−galactosyl−1,3)−N−acetylgalactosaminide alpha−2,6−sialyltransfera
Log2 Intensity
7.0
7.5
8.0
8.5
9.0
9.5
10
15
20
25
30
35
40
Gestational age (weeks)

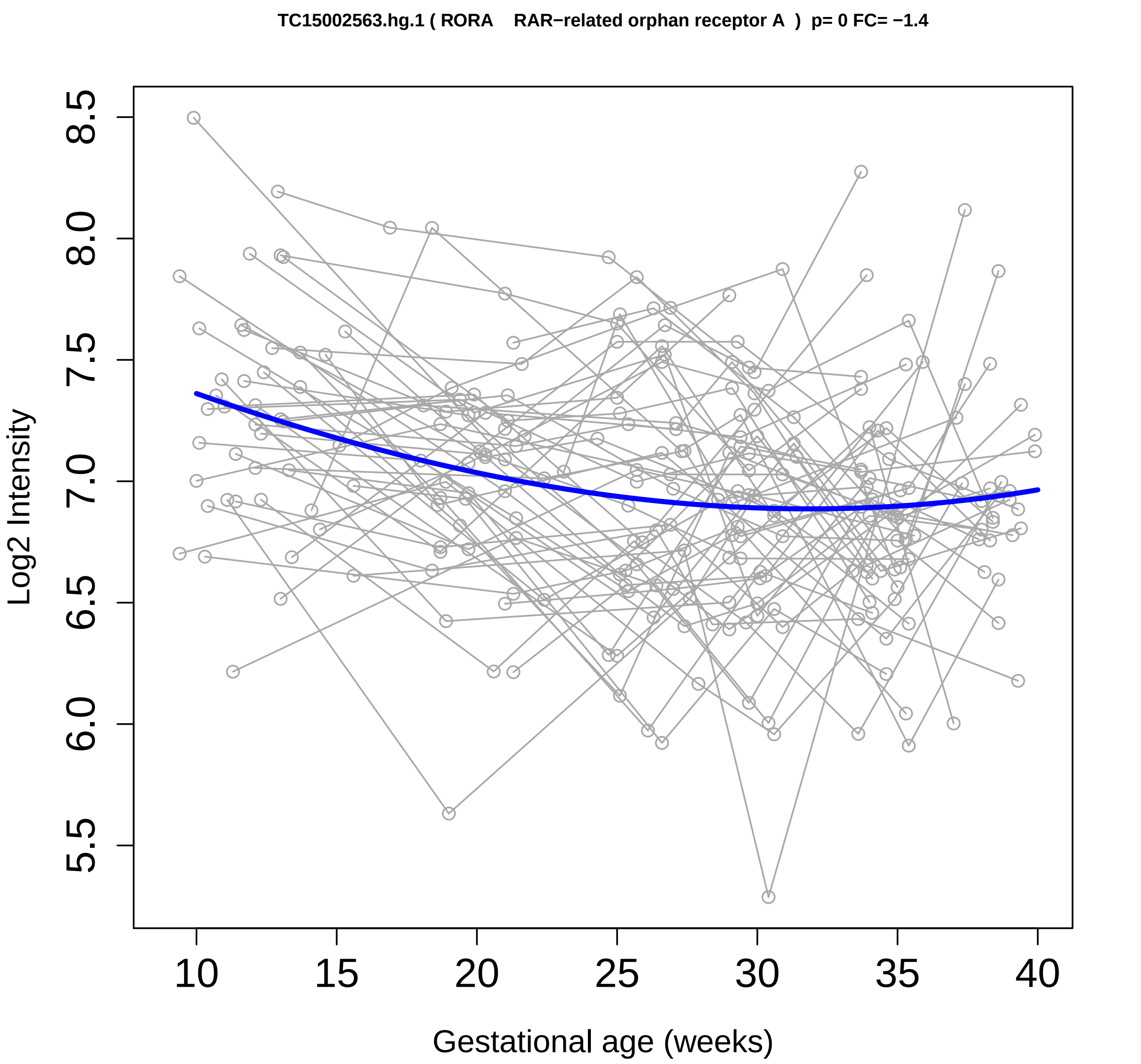
TC15002563.hg.1 (RORA RAR−related orphan receptor A) p= 0 FC= −1.4
Log2 Intensity
8.5
8.0
7.5
7.0
6.5
6.0
5.5
10
15
20
25
30
35
40
Gestational age (weeks)

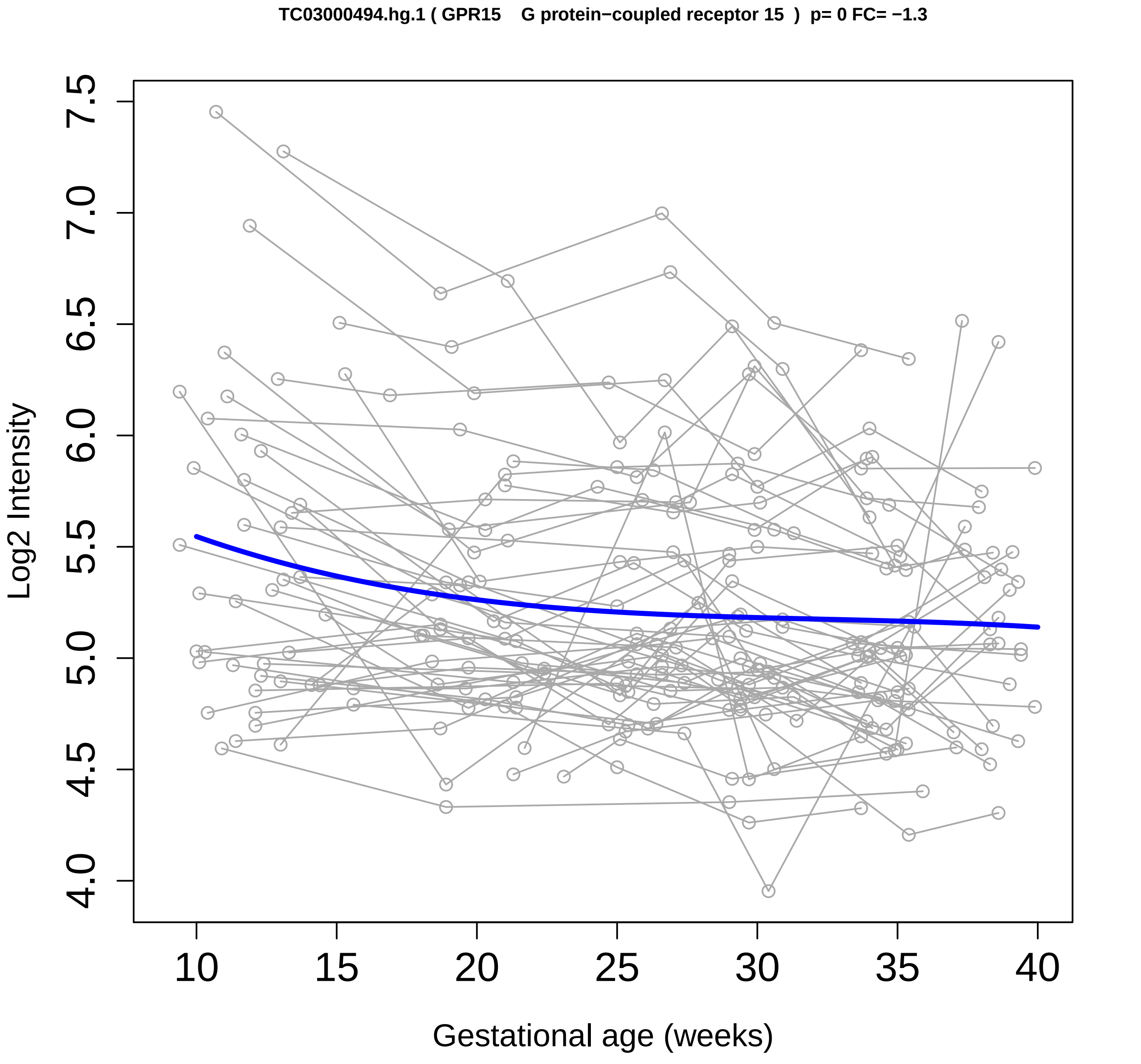

TC03000494.hg.1 (GPR15 G protein−coupled receptor 15) p= 0 FC= −1.3
Log2 Intensity
Gestational age (weeks)
4.0
4.5
5.0
5.5
6.0
6.5
7.0
7.5
10
15
20
25
30
35
40

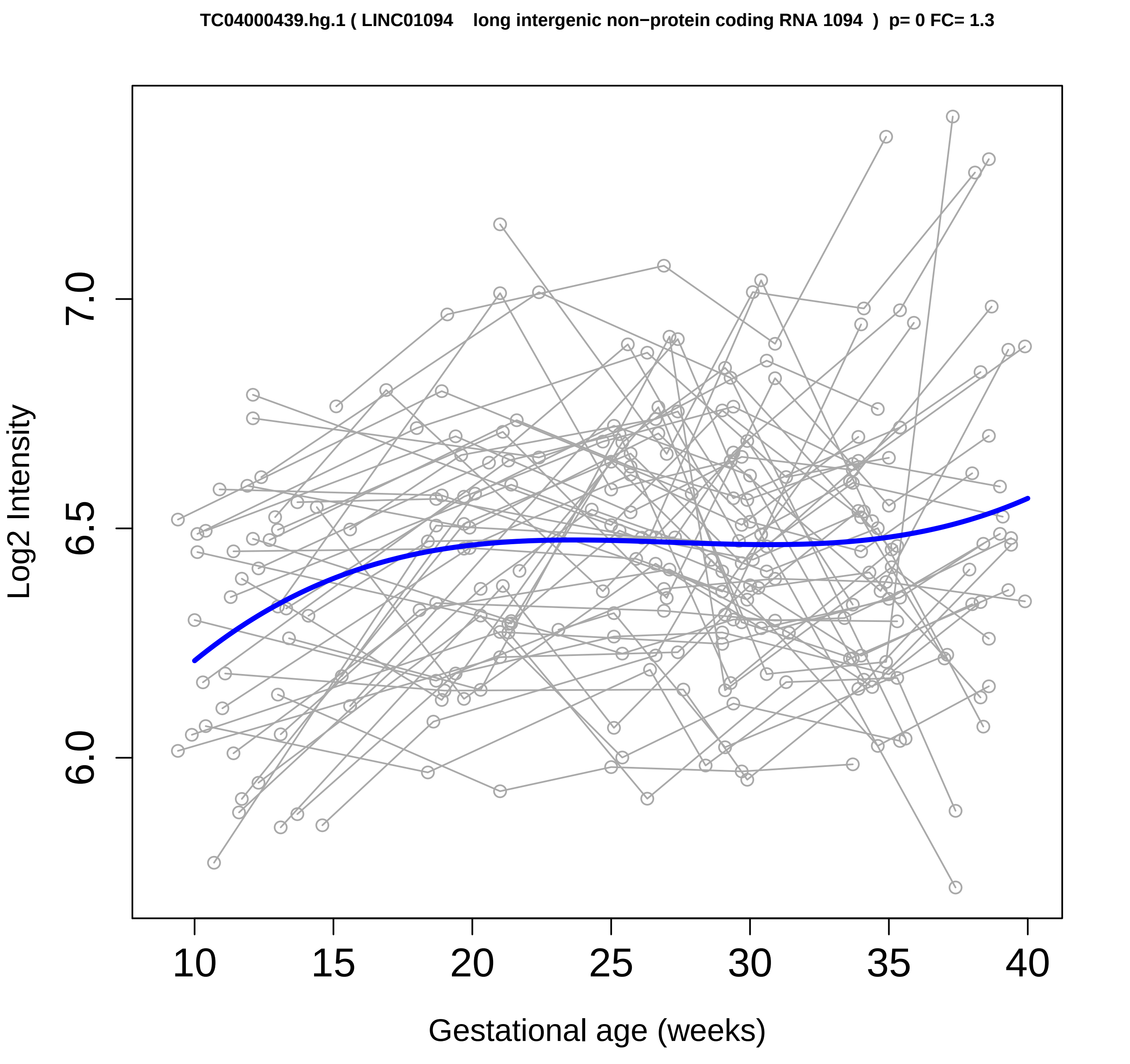
TC04000439.hg.1 (LINC01094 long intergenic non−protein coding RNA 1094) p= 0 FC= 1.3
Log2 Intensity
6.0
6.5
7.0
10
15
20
25
30
35
40
Gestational age (weeks)

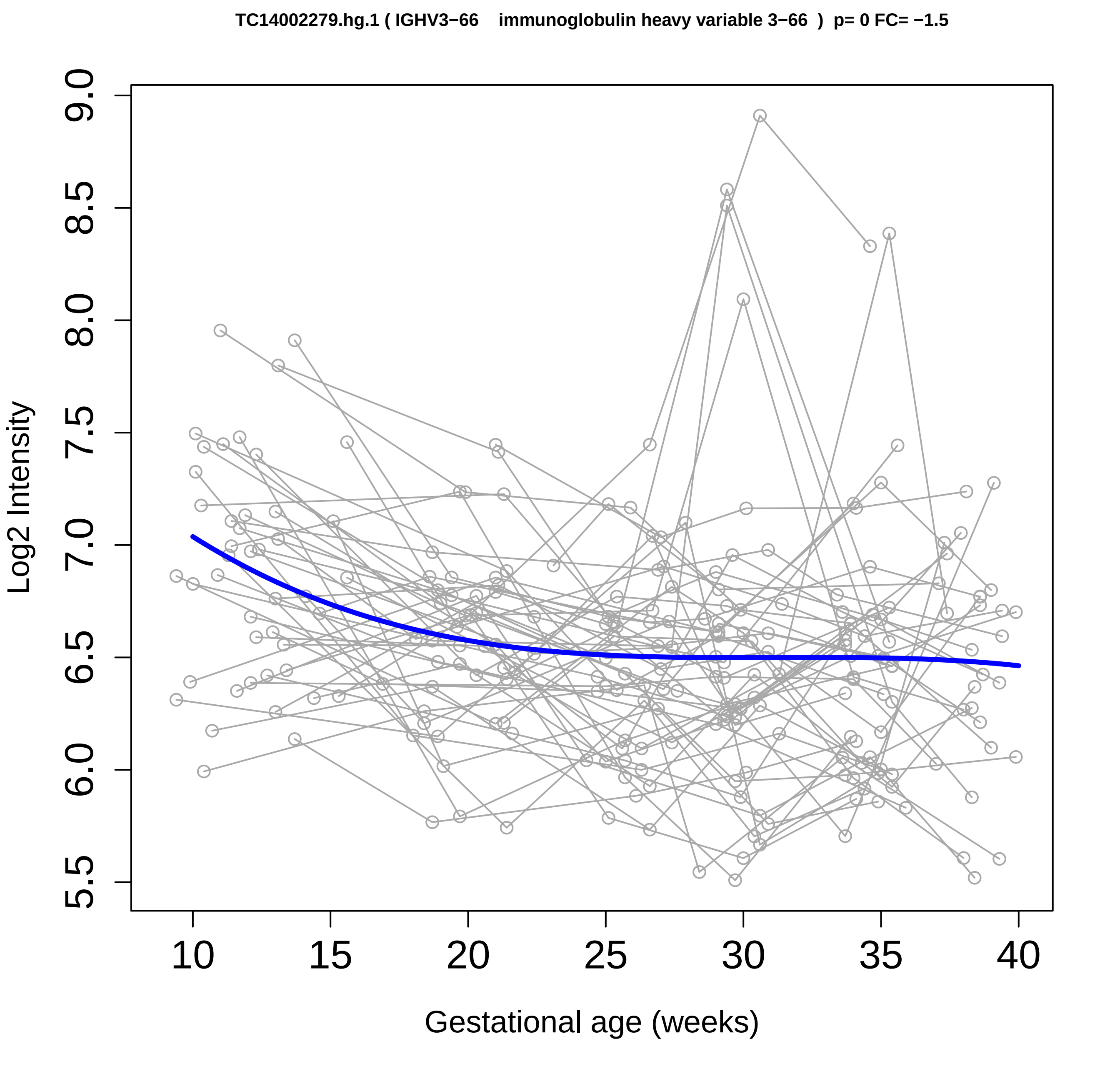

TC14002279.hg.1 (IGHV3−66 immunoglobulin heavy variable 3−66) p= 0 FC= −1.5
Log2 Intensity
5.5
6.0
6.5
7.0
7.5
8.0
8.5
9.0
10
15
20
25
30
35
40
Gestational age (weeks)

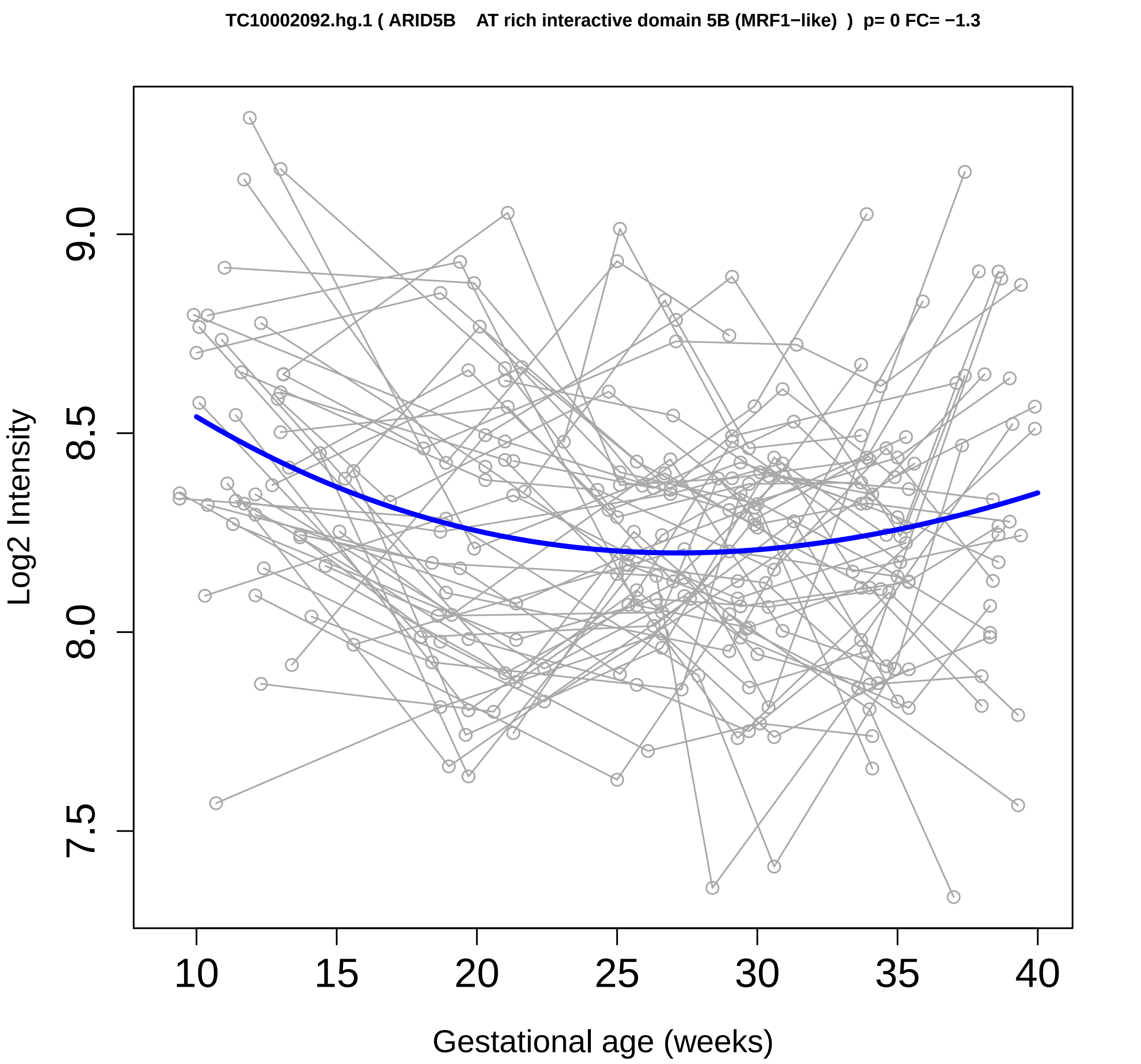
TC10002092.hg.1 (ARID5B AT rich interactive domain 5B (MRF1−like)) p= 0 FC= −1.3
Log2 Intensity
9.0
8.5
8.0
7.5
10
15
20
25
30
35
40
Gestational age (weeks)

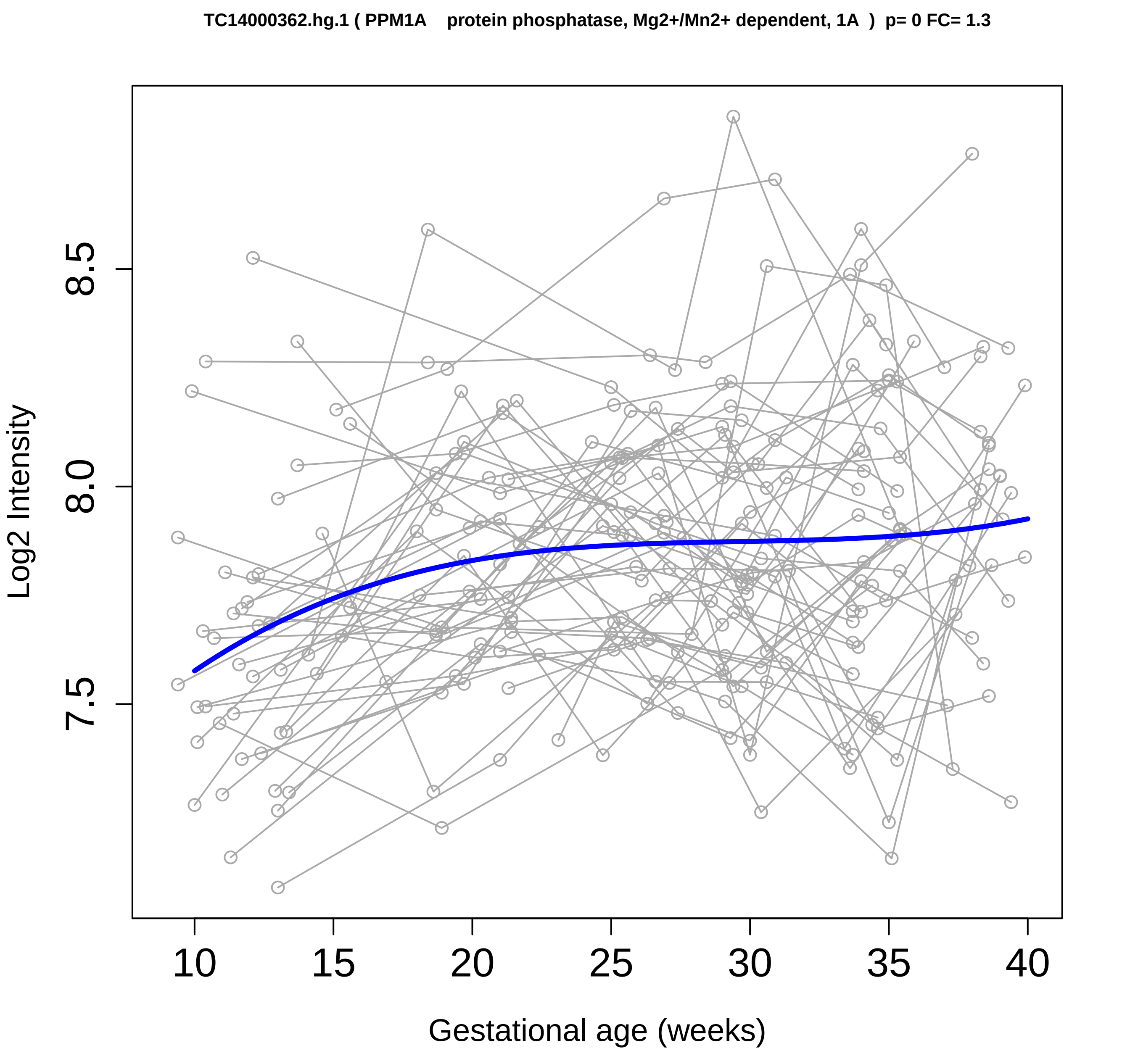

TC14000362.hg.1 (PPM1A protein phosphatase, Mg2+/Mn2+ dependent, 1A) p= 0 FC= 1.3
Log2 Intensity
7.5
8.0
8.5
10
15
20
25
30
35
40
Gestational age (weeks)

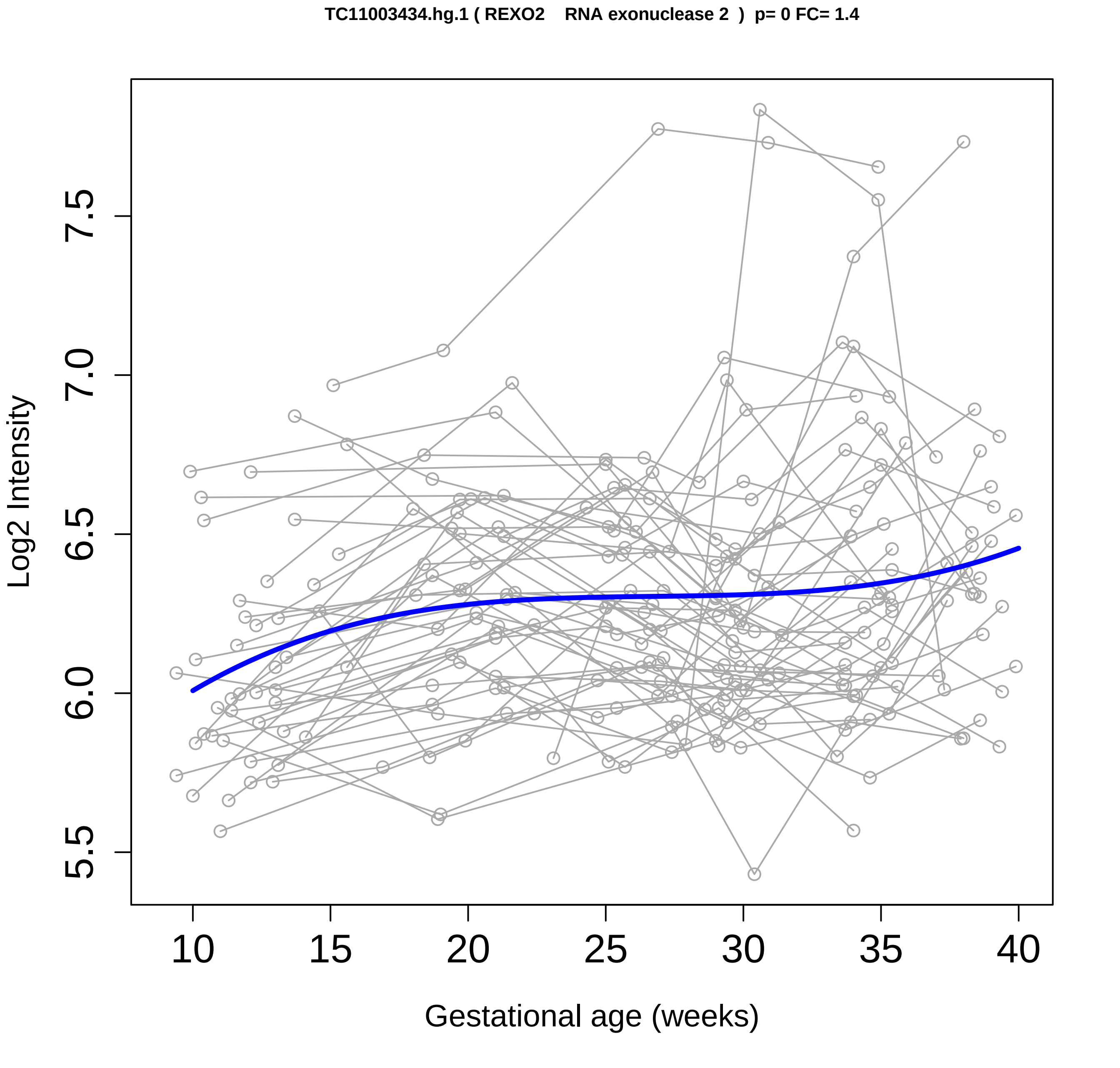
TC11003434.hg.1 (REXO2 RNA exonuclease 2) p= 0 FC= 1.4
Log2 Intensity
5.5
6.0
6.5
7.0
7.5
10
15
20
25
30
35
40
Gestational age (weeks)

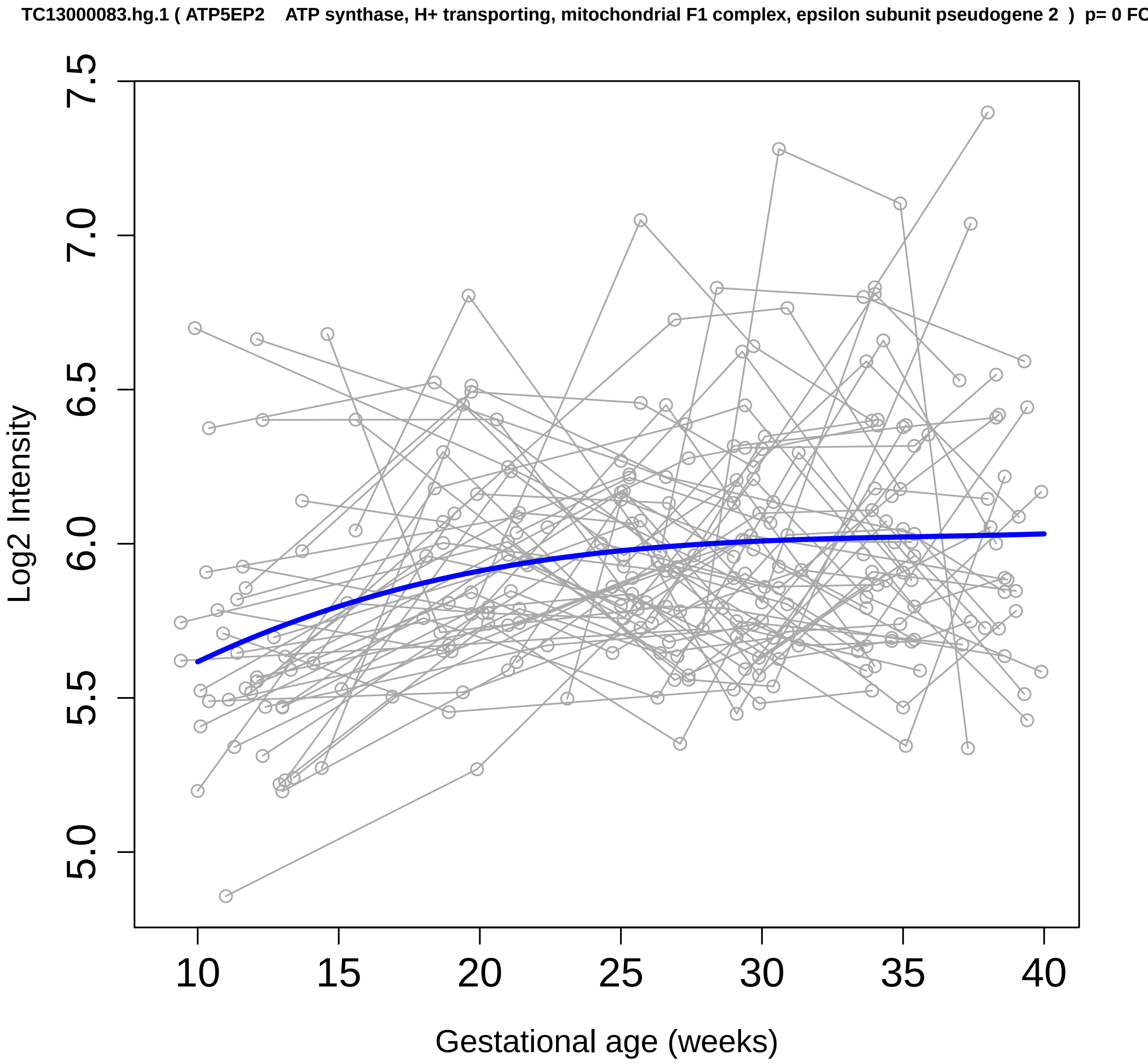
TC13000083.hg.1 (ATP5EP2 ATP synthase, H+ transporting, mitochondrial F1 complex, epsilon subunit pseudogene 2) p= 0 FC
Log2 Intensity
7.5
7.0
6.5
6.0
5.5
5.0
10
15
20
25
30
35
40
Gestational age (weeks)

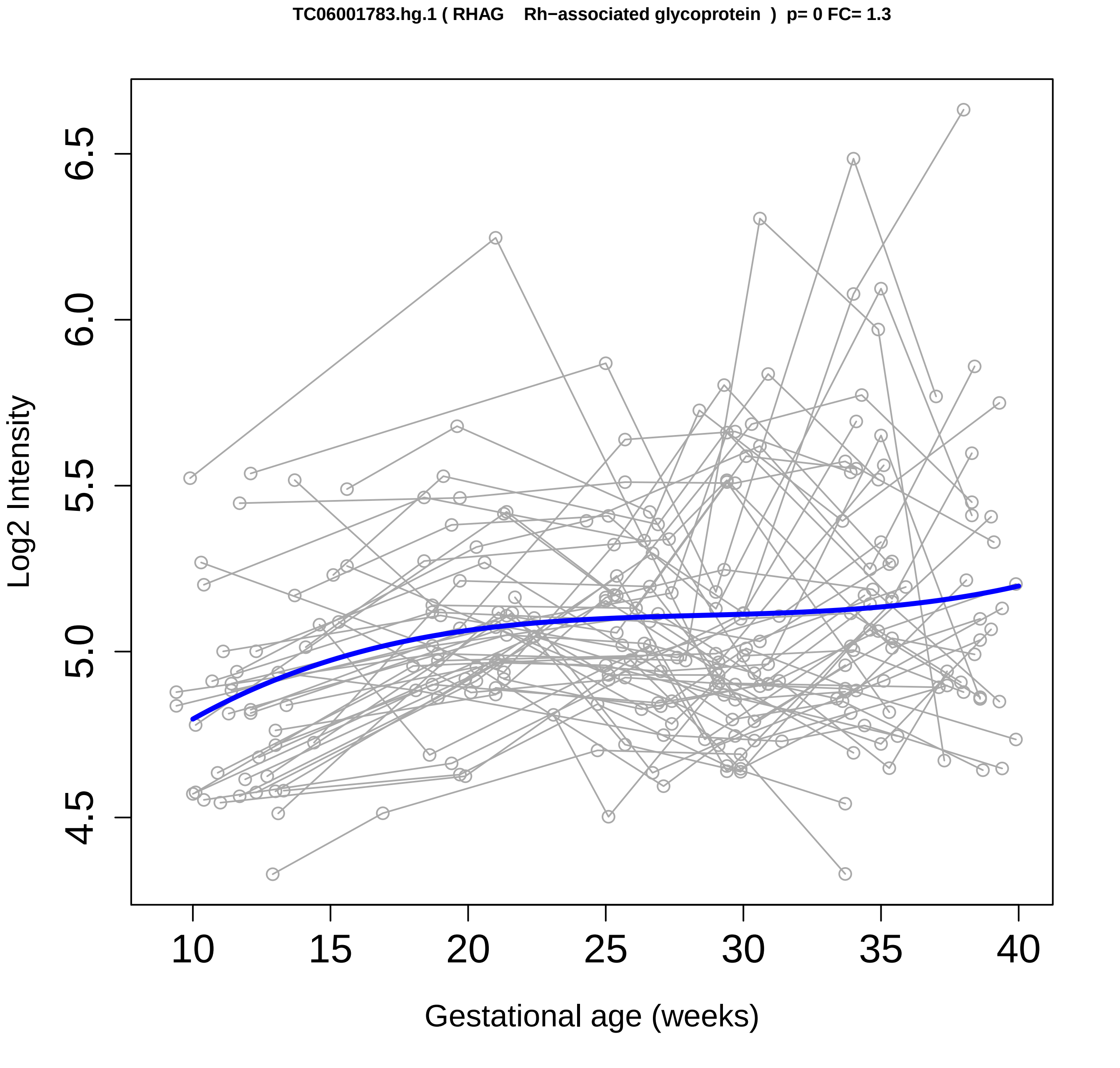
TC06001783.hg.1 (RHAG Rh−associated glycoprotein) p= 0 FC= 1.3
Log2 Intensity
4.5
5.0
5.5
6.0
6.5
10
15
20
25
30
35
40
Gestational age (weeks)

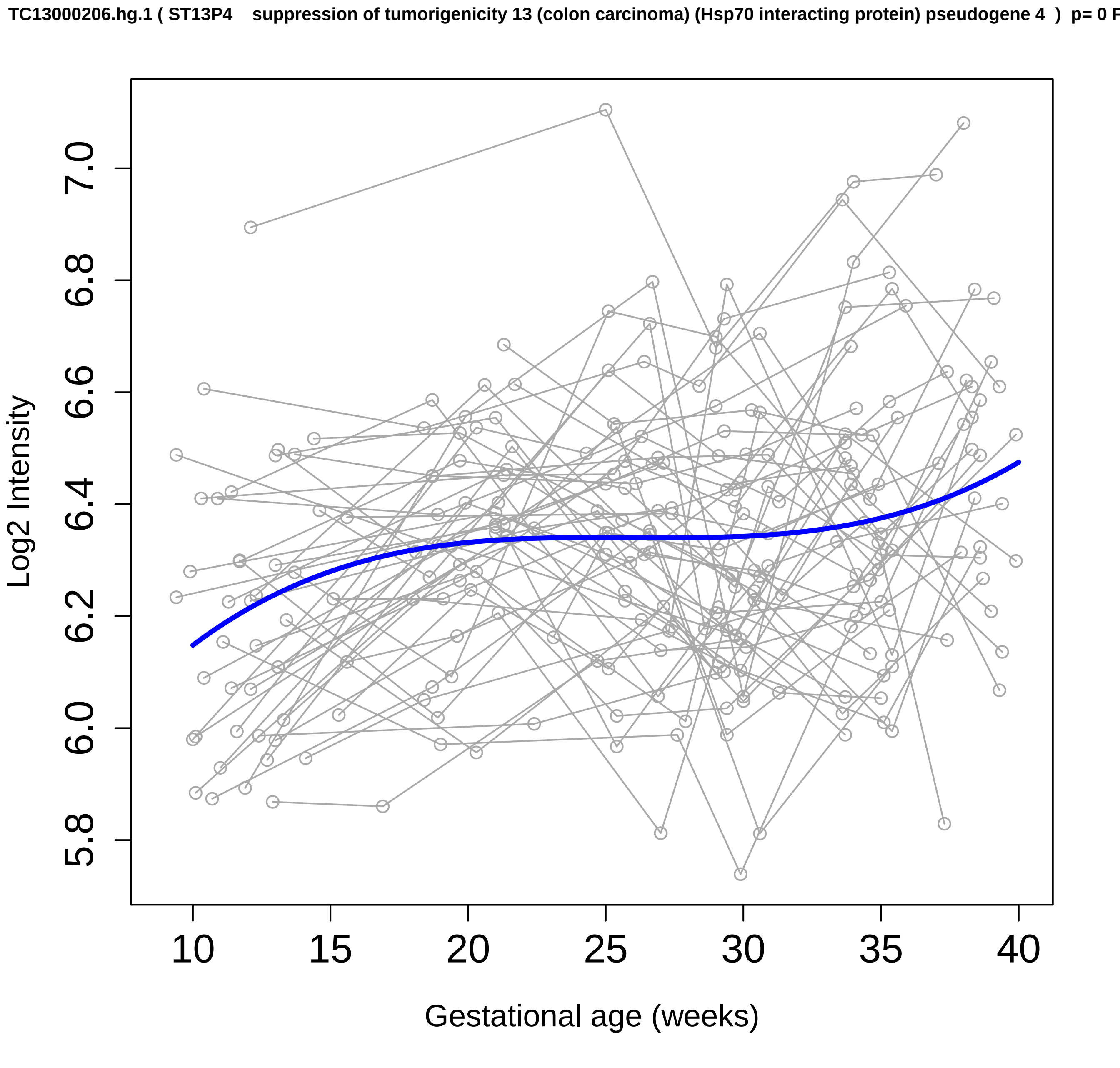
TC13000206.hg.1 (ST13P4 suppression of tumorigenicity 13 (colon carcinoma) (Hsp70 interacting protein) pseudogene 4) p= 0 F
Log2 Intensity
5.8
6.0
6.2
6.4
6.6
6.8
7.0
10
15
20
25
30
35
40
Gestational age (weeks)

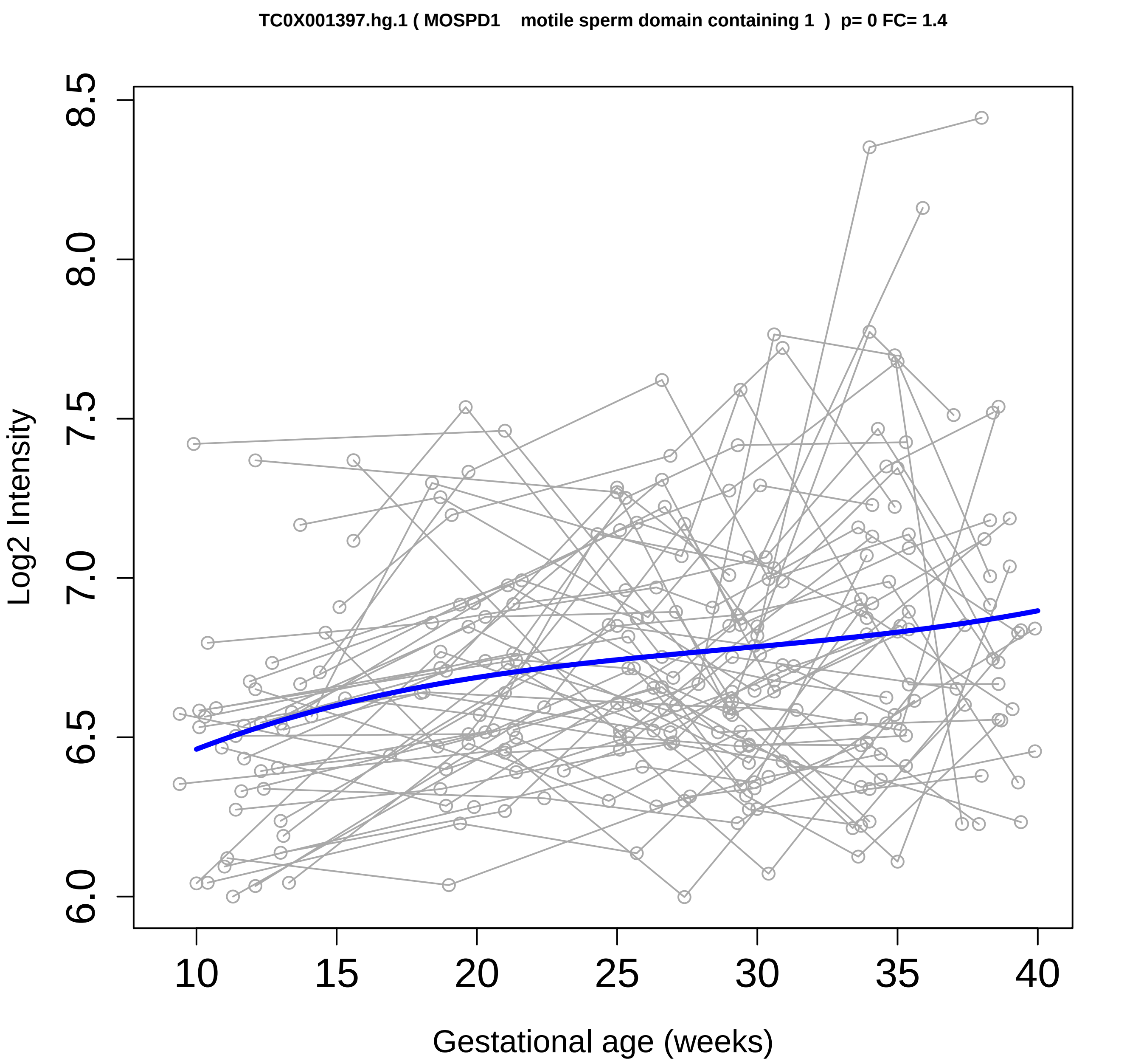

TC0X001397.hg.1 (MOSPD1 motile sperm domain containing 1) p= 0 FC= 1.4
Log2 Intensity
6.0
6.5
7.0
7.5
8.0
8.5
10
15
20
25
30
35
40
Gestational age (weeks)

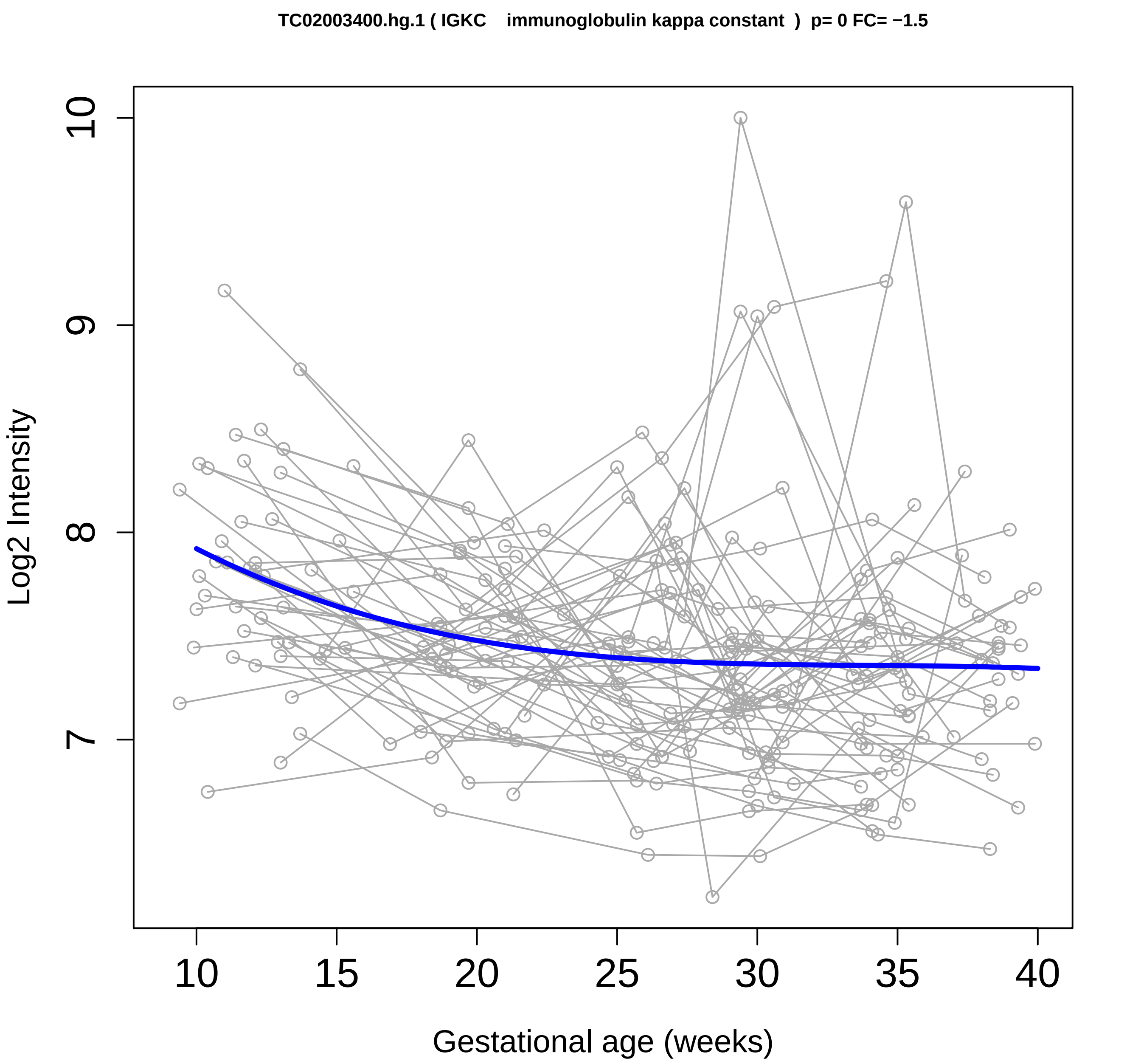

TC02003400.hg.1 (IGKC immunoglobulin kappa constant) p= 0 FC= −1.5
Log2 Intensity
10
9
8
7
10
15
20
25
30
35
40
Gestational age (weeks)

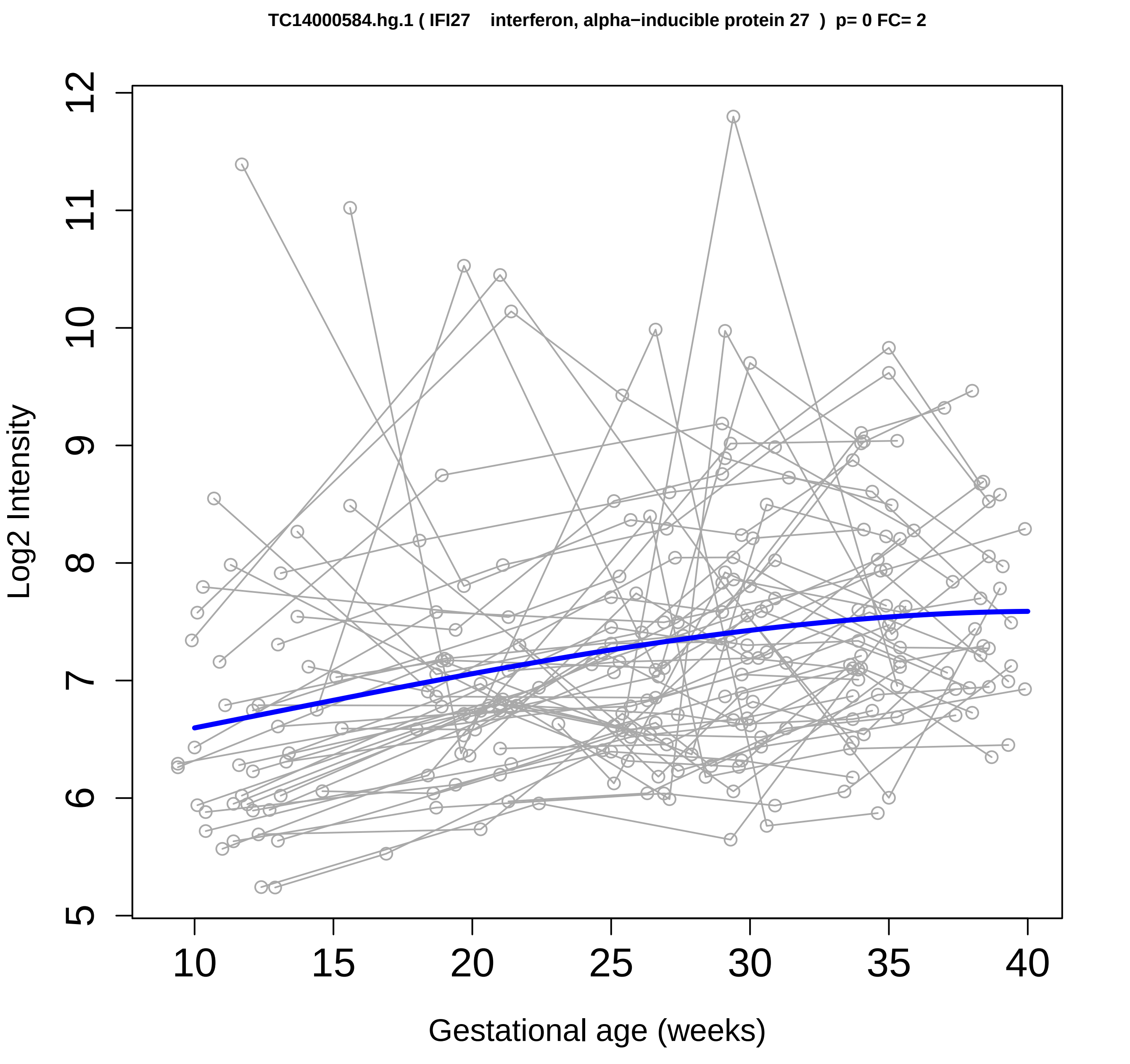

TC14000584.hg.1 (IFI27   interferon, alpha−inducible protein 27) p= 0 FC= 2
Log2 Intensity
5
6
7
8
9
10
11
12
10
15
20
25
30
35
40
Gestational age (weeks)

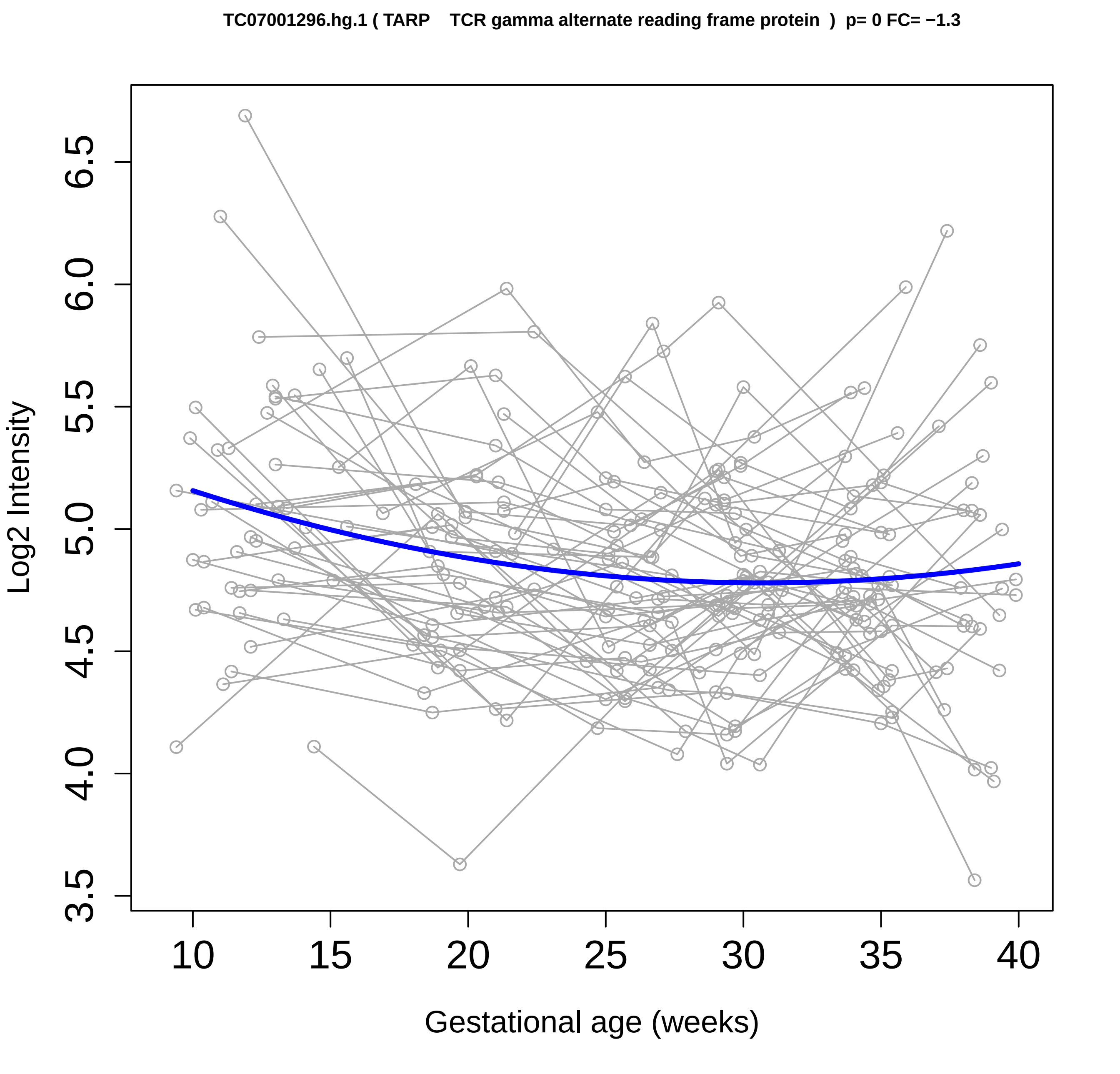
TC07001296.hg.1 (TARP TCR gamma alternate reading frame protein) p= 0 FC= −1.3
Log2 Intensity
3.5
4.0
4.5
5.0
5.5
6.0
6.5
10
15
20
25
30
35
40
Gestational age (weeks)

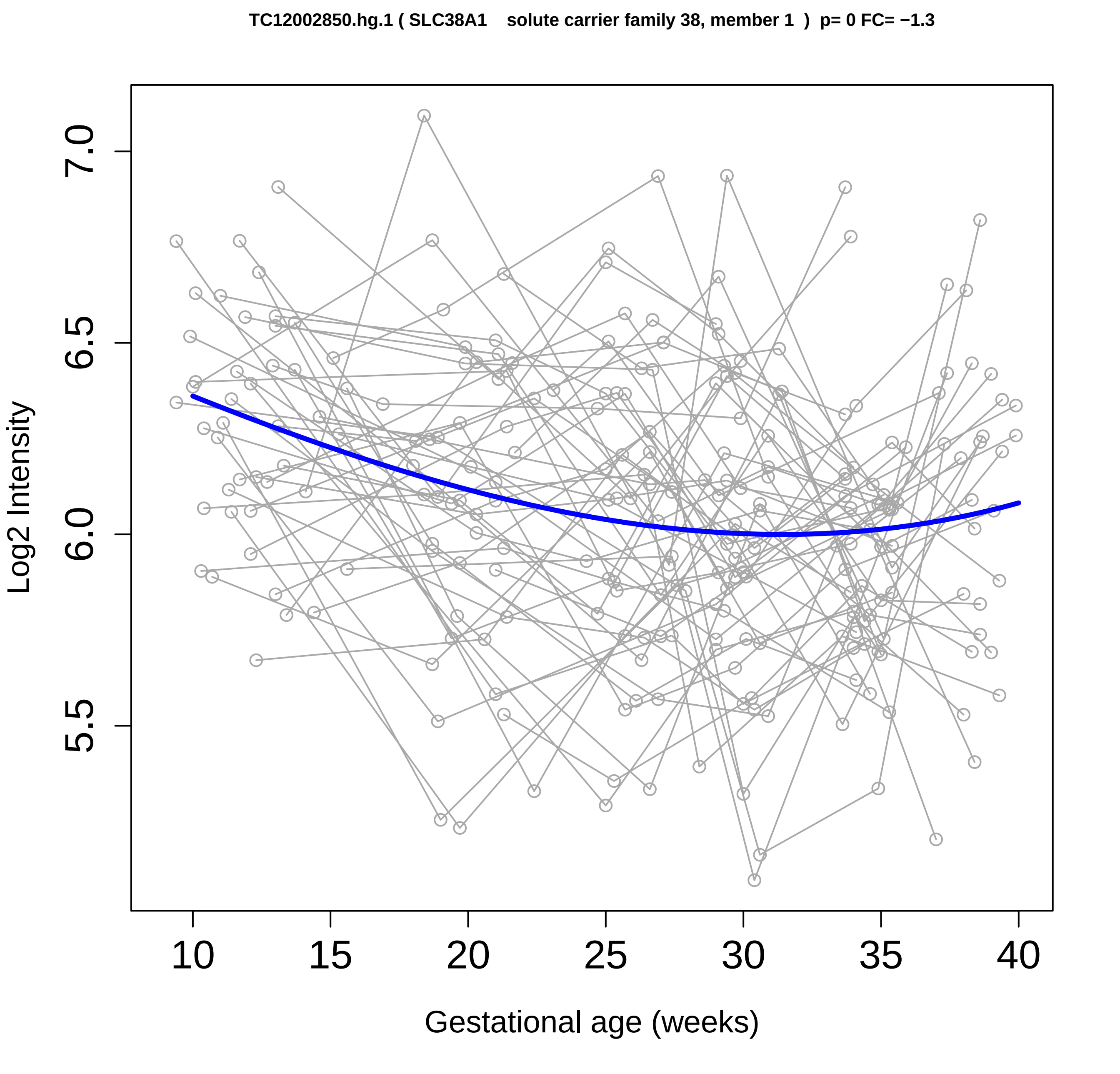

TC12002850.hg.1 (SLC38A1 solute carrier family 38, member 1) p= 0 FC= −1.3
Log2 Intensity
7.0
6.5
6.0
5.5
10
15
20
25
30
35
40
Gestational age (weeks)

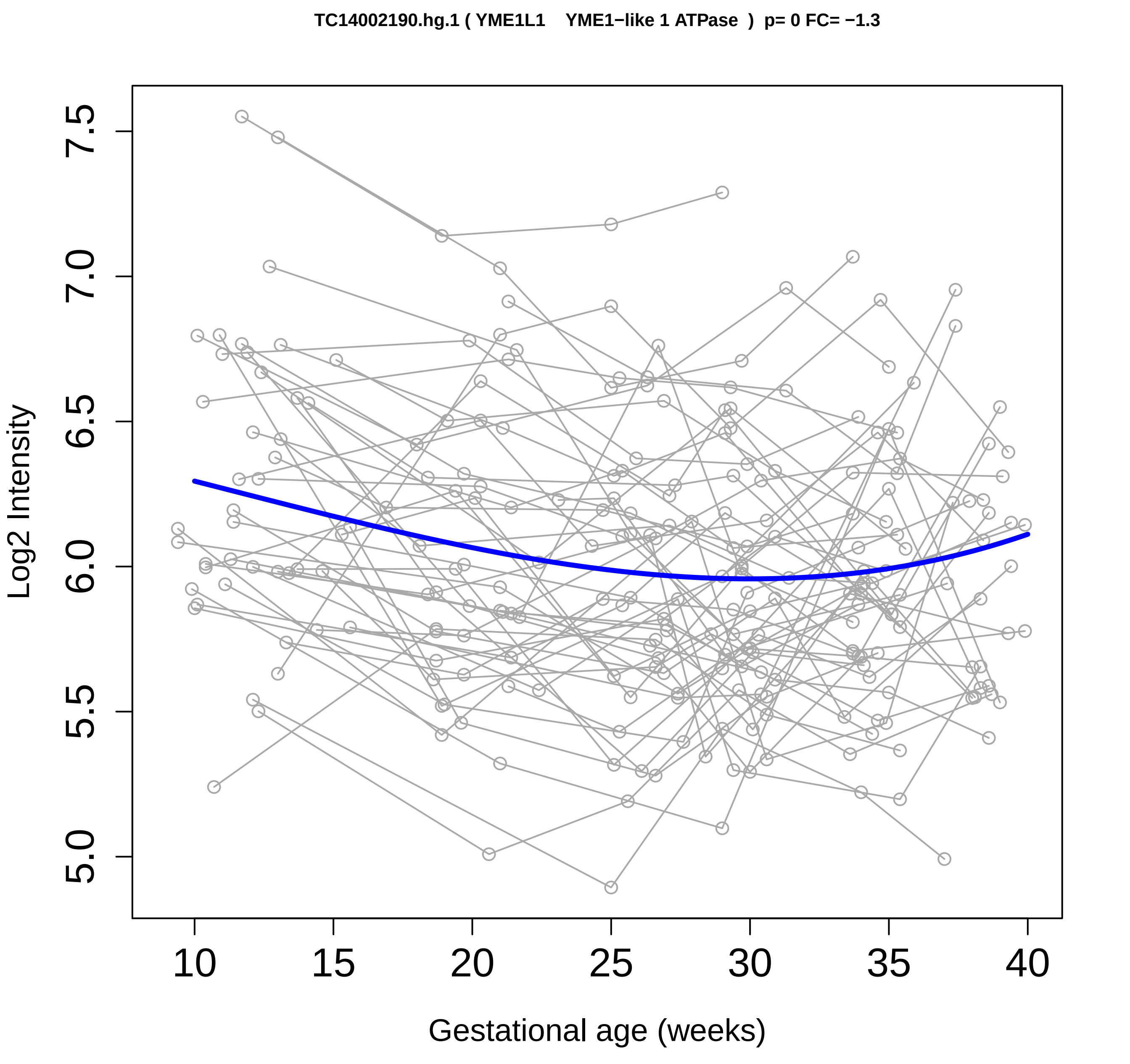

TC14002190.hg.1 (YME1L1 YME1−like 1 ATPase) p= 0 FC= −1.3
Log2 Intensity
5.0
5.5
6.0
6.5
7.0
7.5
10
15
20
25
30
35
40
Gestational age (weeks)

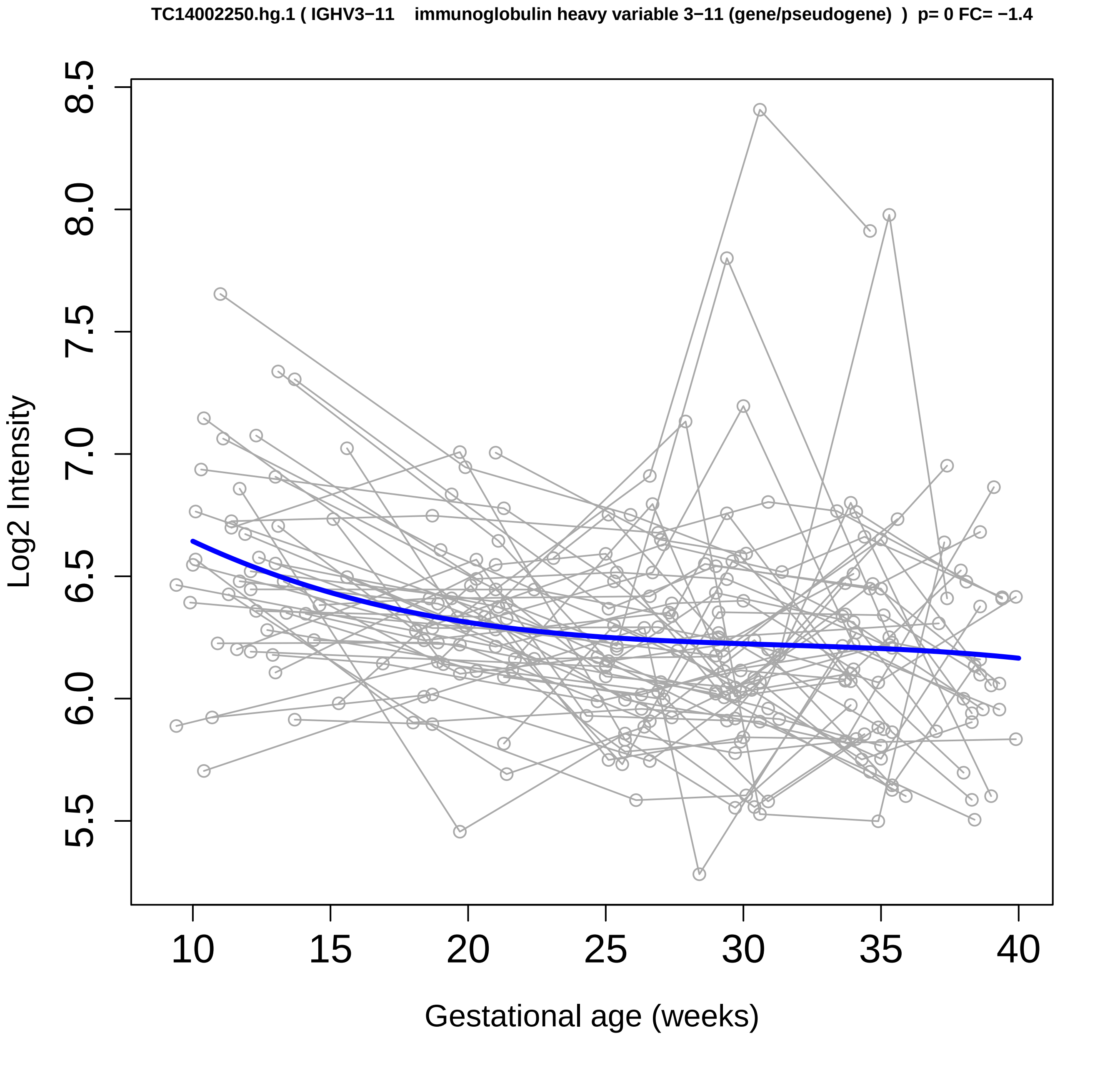
TC14002250.hg.1 (IGHV3−11 immunoglobulin heavy variable 3−11 (gene/pseudogene)) p= 0 FC= −1.4
Log2 Intensity
8.5
8.0
7.5
7.0
6.5
6.0
5.5
10
15
20
25
30
35
40
Gestational age (weeks)

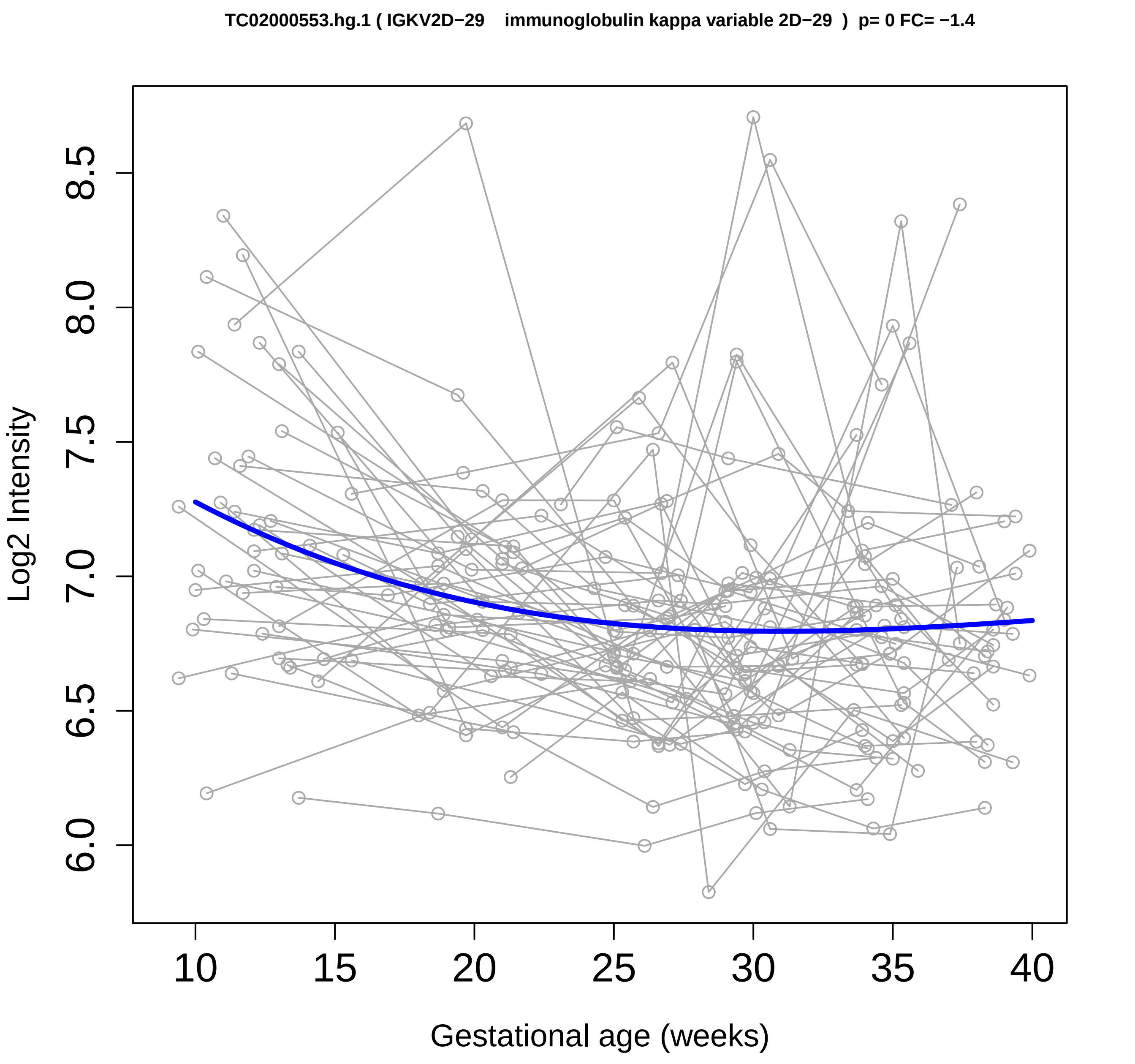
TC02000553.hg.1 (IGKV2D−29   immunoglobulin kappa variable 2D−29) p= 0 FC= −1.4
Log2 Intensity
6.0
6.5
7.0
7.5
8.0
8.5
10
15
20
25
30
35
40
Gestational age (weeks)

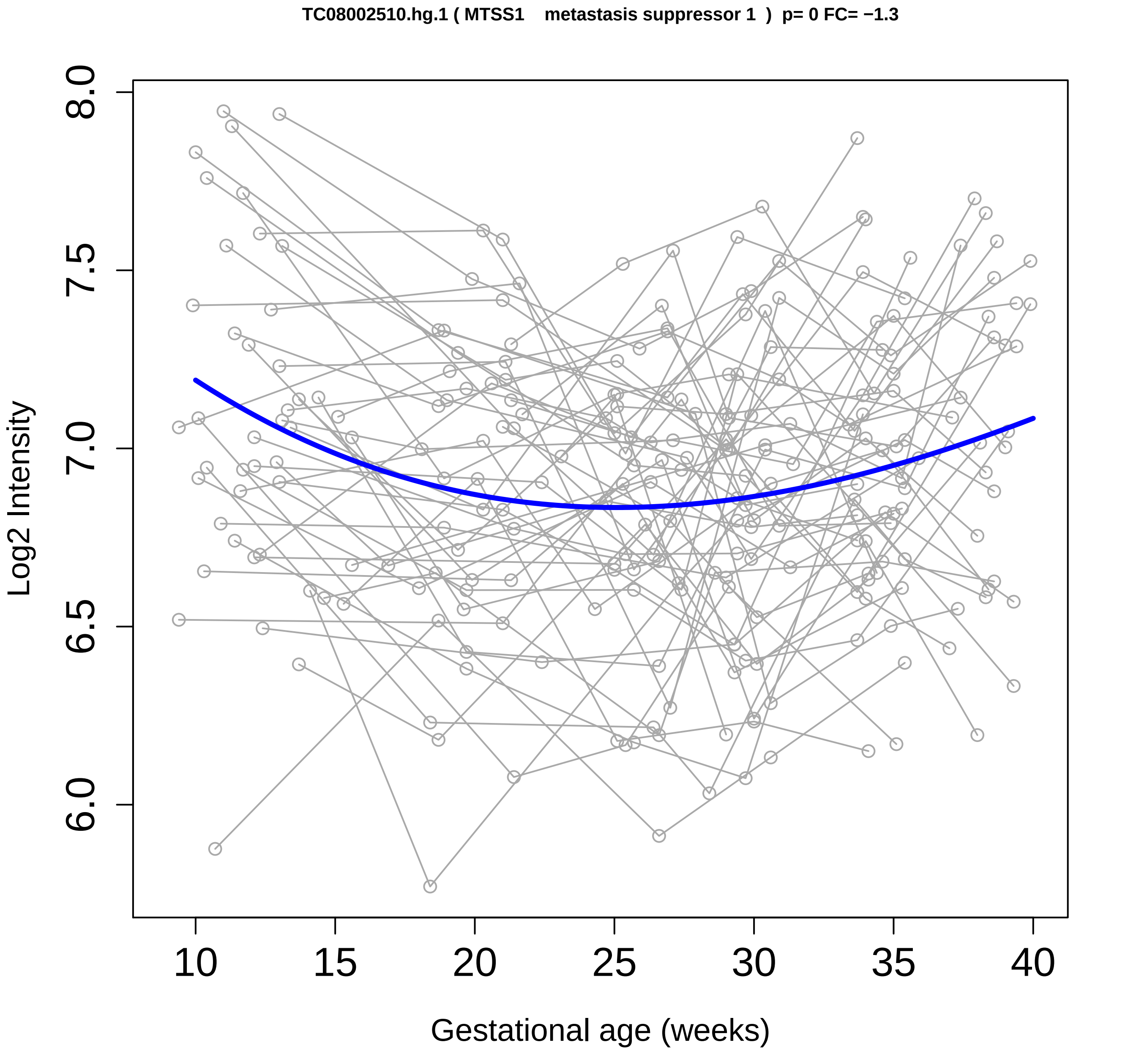

TC08002510.hg.1 (MTSS1 metastasis suppressor 1) p= 0 FC= −1.3
8.0
7.5
7.0
6.5
6.0
Log2 Intensity
10
15
20
25
30
35
40
Gestational age (weeks)

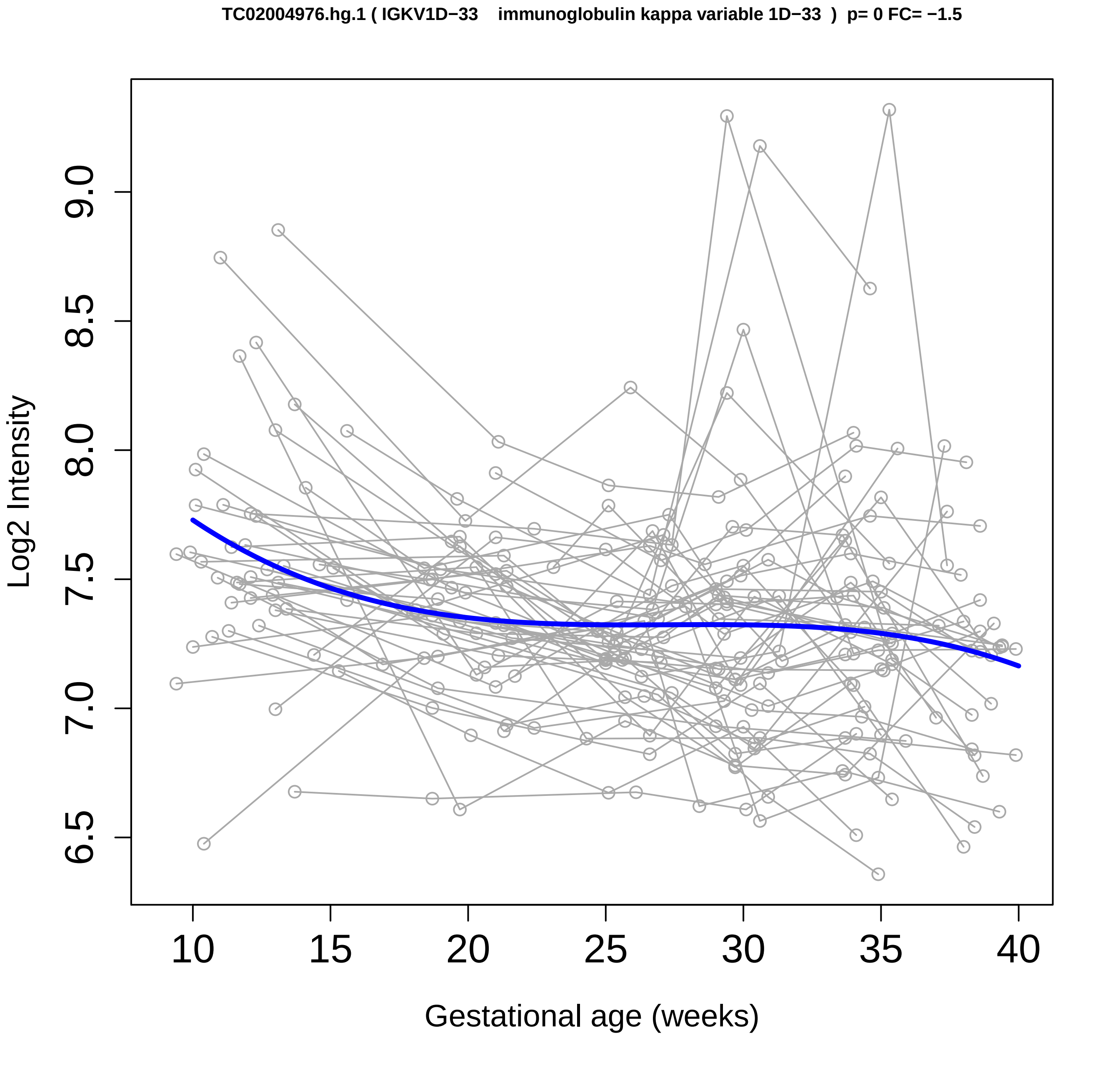

TC02004976.hg.1 (IGKV1D−33 immunoglobulin kappa variable 1D−33) p= 0 FC= −1.5
Log2 Intensity
9.0
8.5
8.0
7.5
7.0
6.5
10
15
20
25
30
35
40
Gestational age (weeks)

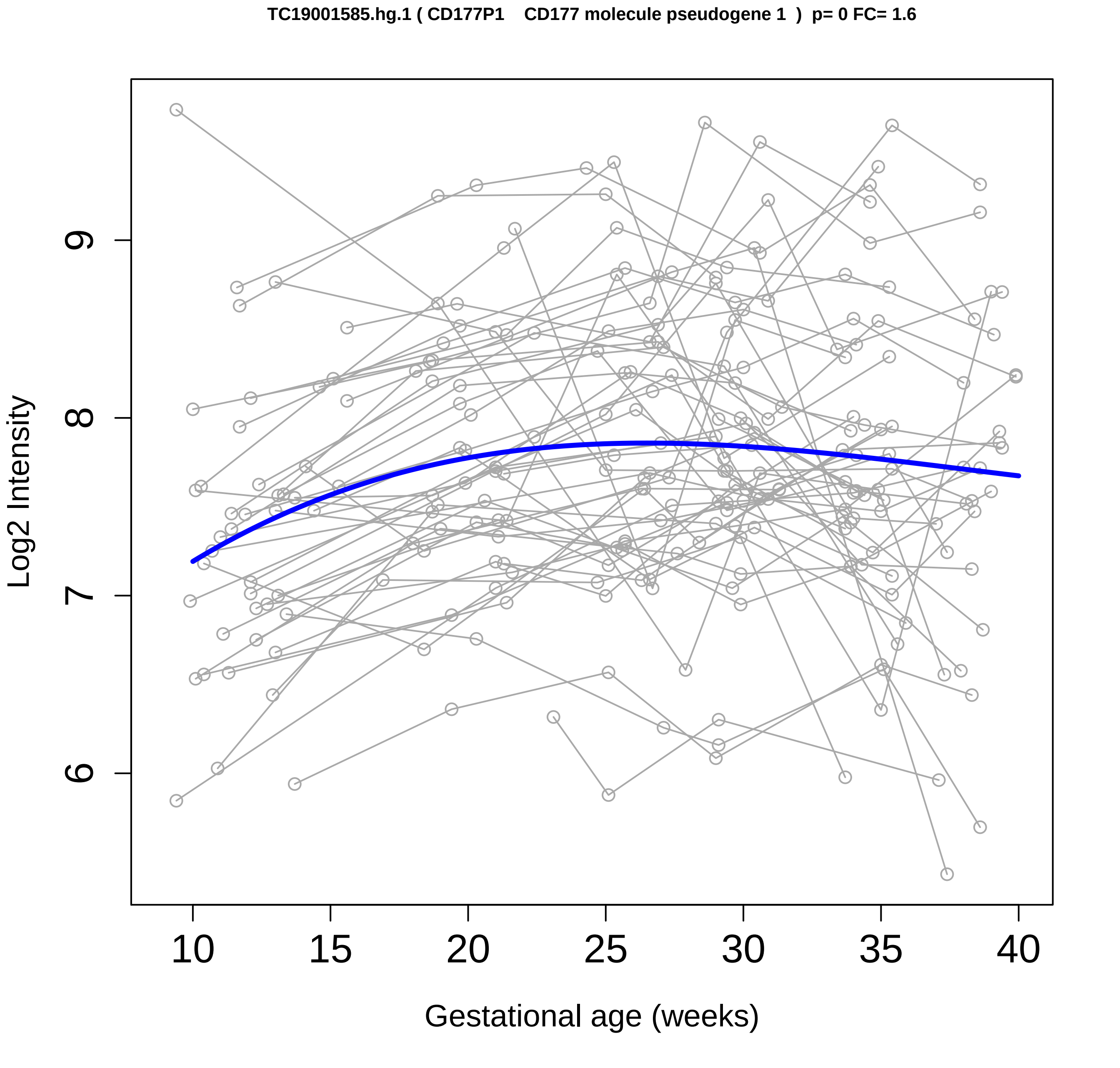

TC19001585.hg.1 (CD177P1   CD177 molecule pseudogene 1) p= 0 FC= 1.6
Log2 Intensity
6
7
8
9
10
15
20
25
30
35
40
Gestational age (weeks)

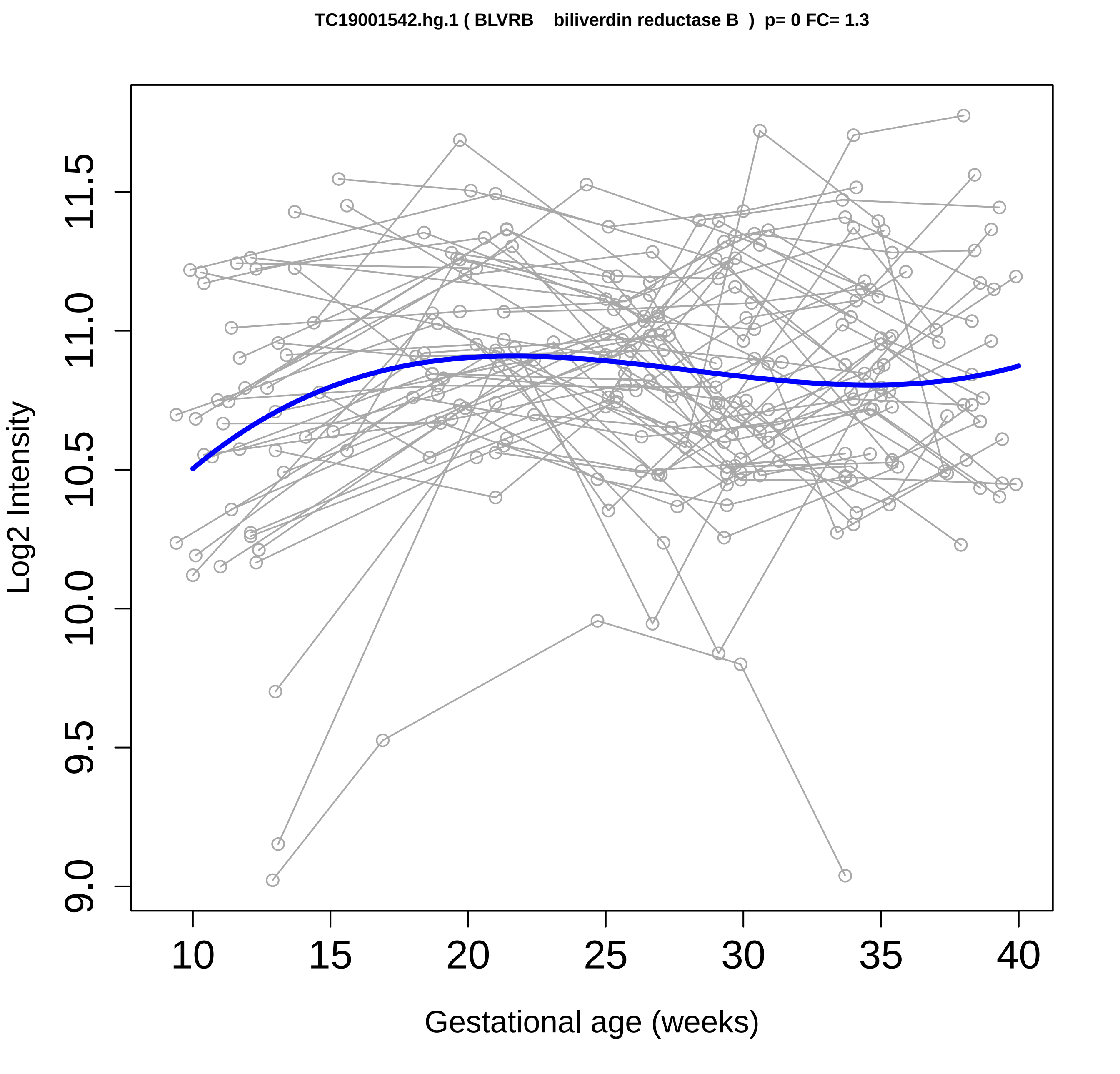

TC19001542.hg.1 (BLVRB biliverdin reductase B) p= 0 FC= 1.3
Log2 Intensity
9.0
9.5
10.0
10.5
11.0
11.5
10
15
20
25
30
35
40
Gestational age (weeks)

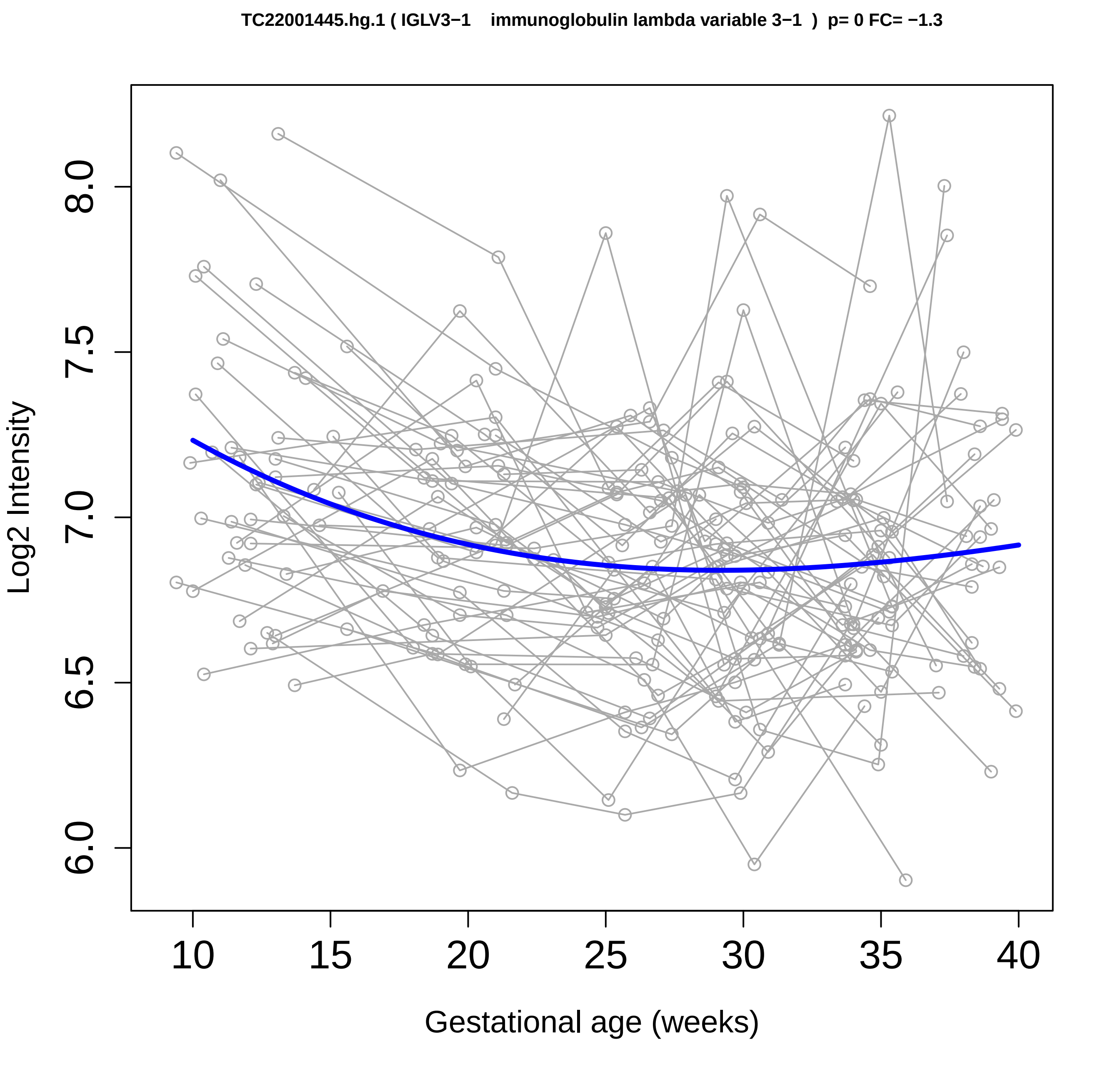
TC22001445.hg.1 (IGLV3−1 immunoglobulin lambda variable 3−1) p= 0 FC= −1.3
Log2 Intensity
8.0
7.5
7.0
6.5
6.0
10
15
20
25
30
35
40
Gestational age (weeks)

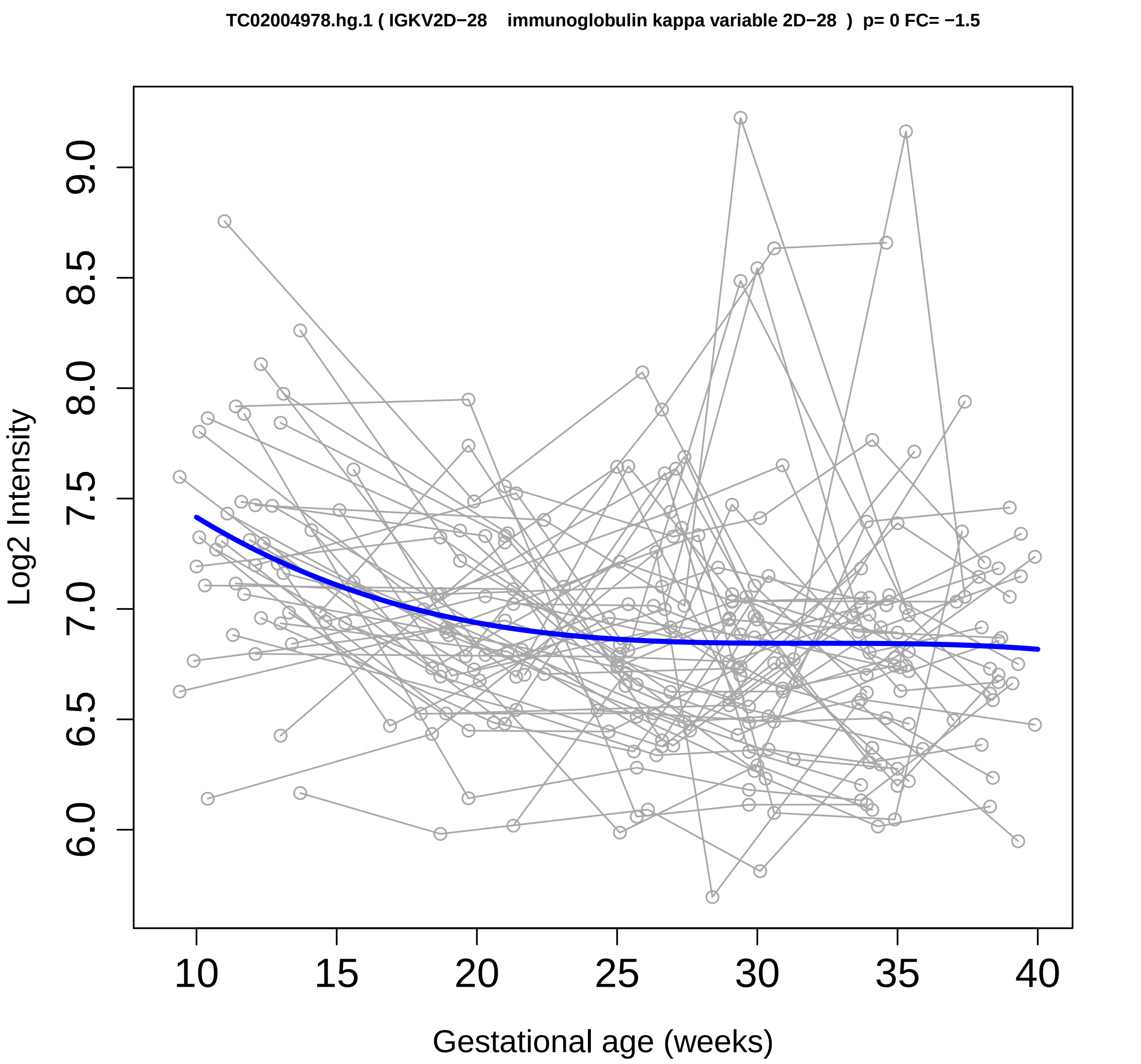

TC02004978.hg.1 (IGKV2D−28   immunoglobulin kappa variable 2D−28) p= 0 FC= −1.5
Log2 Intensity
6.0
6.5
7.0
7.5
8.0
8.5
9.0
10
15
20
25
30
35
40
Gestational age (weeks)

TC17000606.hg.1 (MYL4   myosin light chain 4) p= 0 FC= 1.4

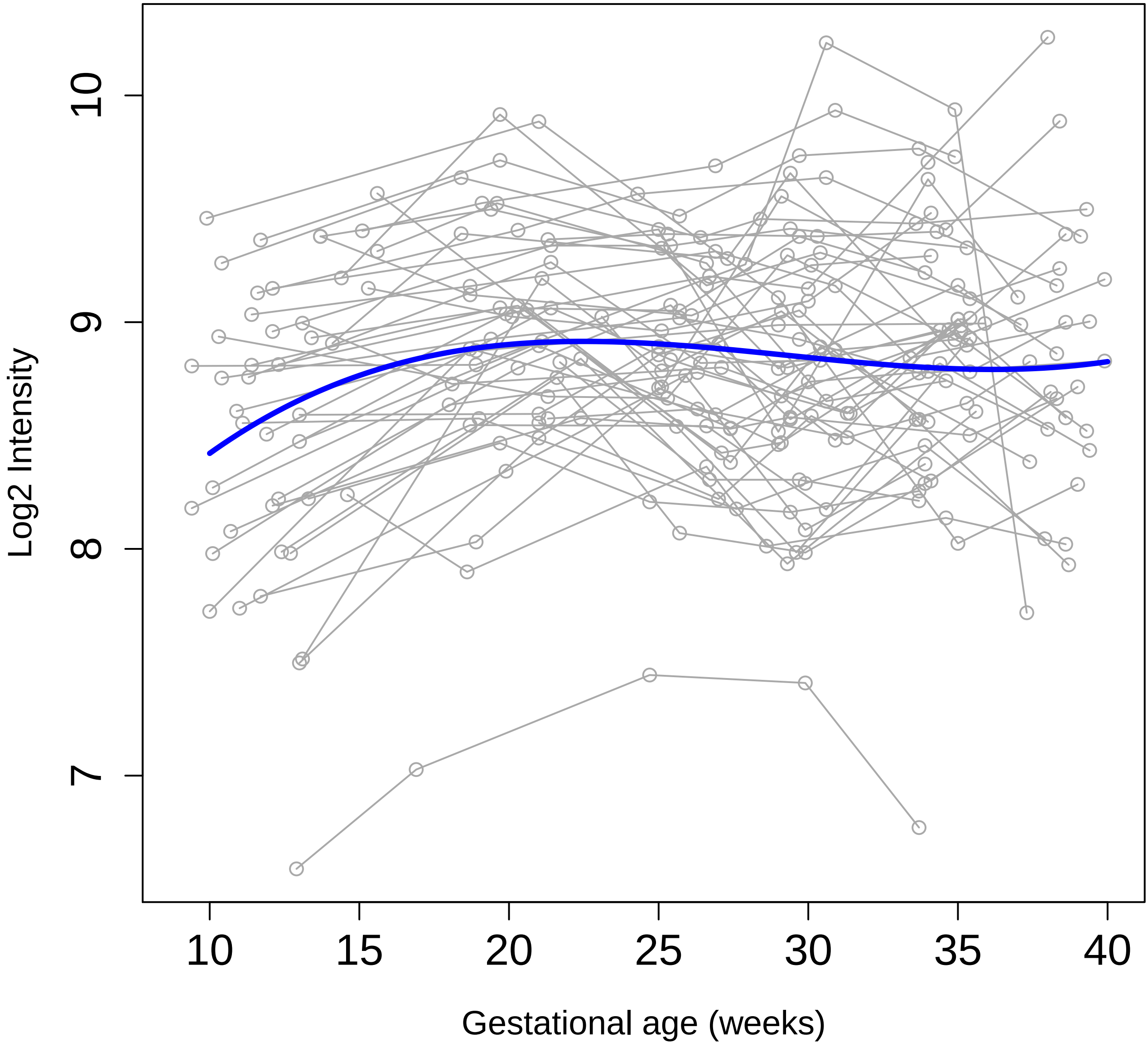

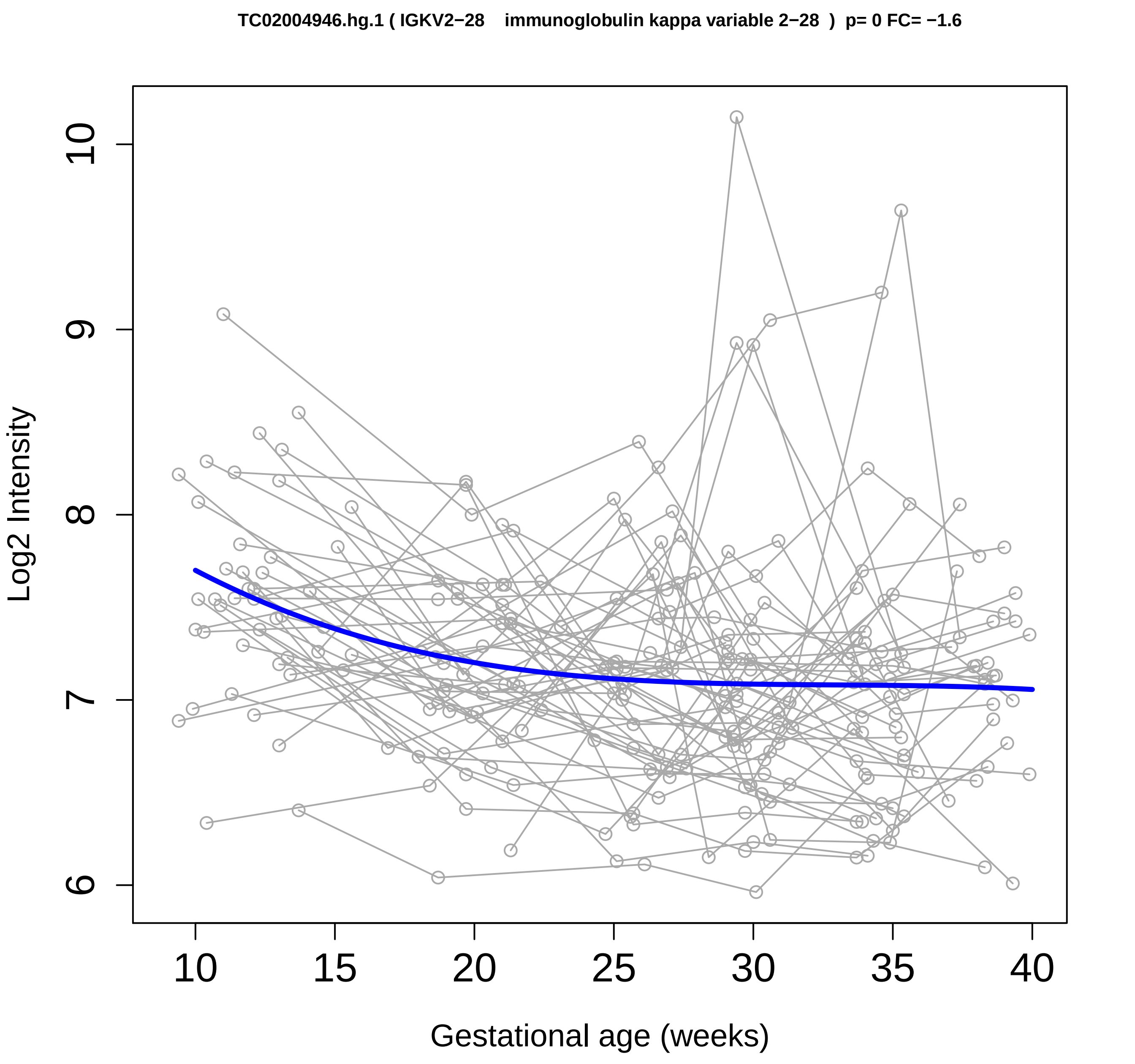
TC02004946.hg.1 (IGKV2−28   immunoglobulin kappa variable 2−28) p= 0 FC= −1.6
Log2 Intensity
10
9
8
7
6
10
15
20
25
30
35
40
Gestational age (weeks)

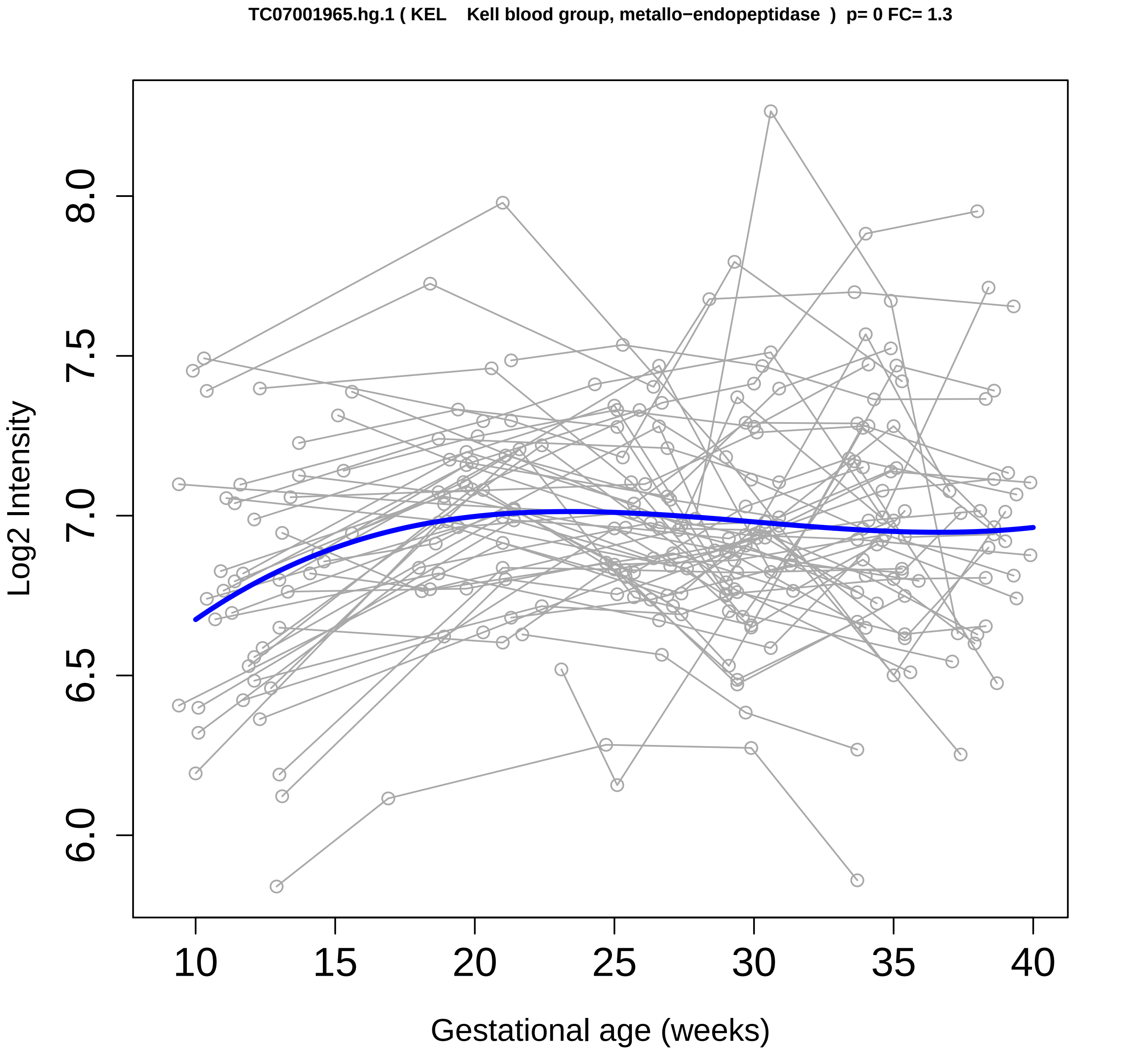

TC07001965.hg.1 (KEL Kell blood group, metallo−endopeptidase) p= 0 FC= 1.3
Log2 Intensity
8.0
7.5
7.0
6.5
6.0
10
15
20
25
30
35
40
Gestational age (weeks)

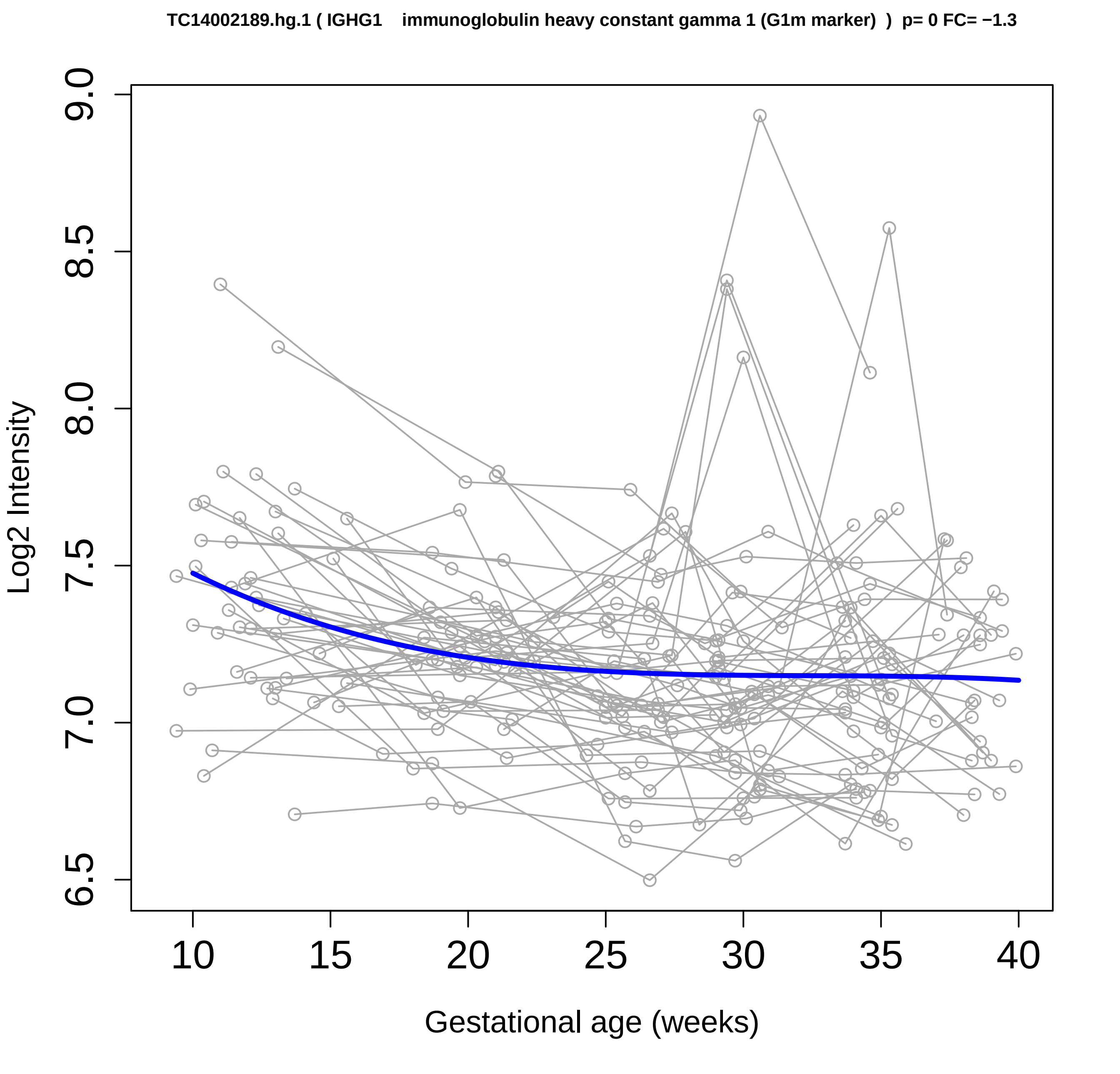

TC14002189.hg.1 (IGHG1 immunoglobulin heavy constant gamma 1 (G1m marker)) p= 0 FC= −1.3
Log2 Intensity
9.0
8.5
8.0
7.5
7.0
6.5
10
15
20
25
30
35
40
Gestational age (weeks)

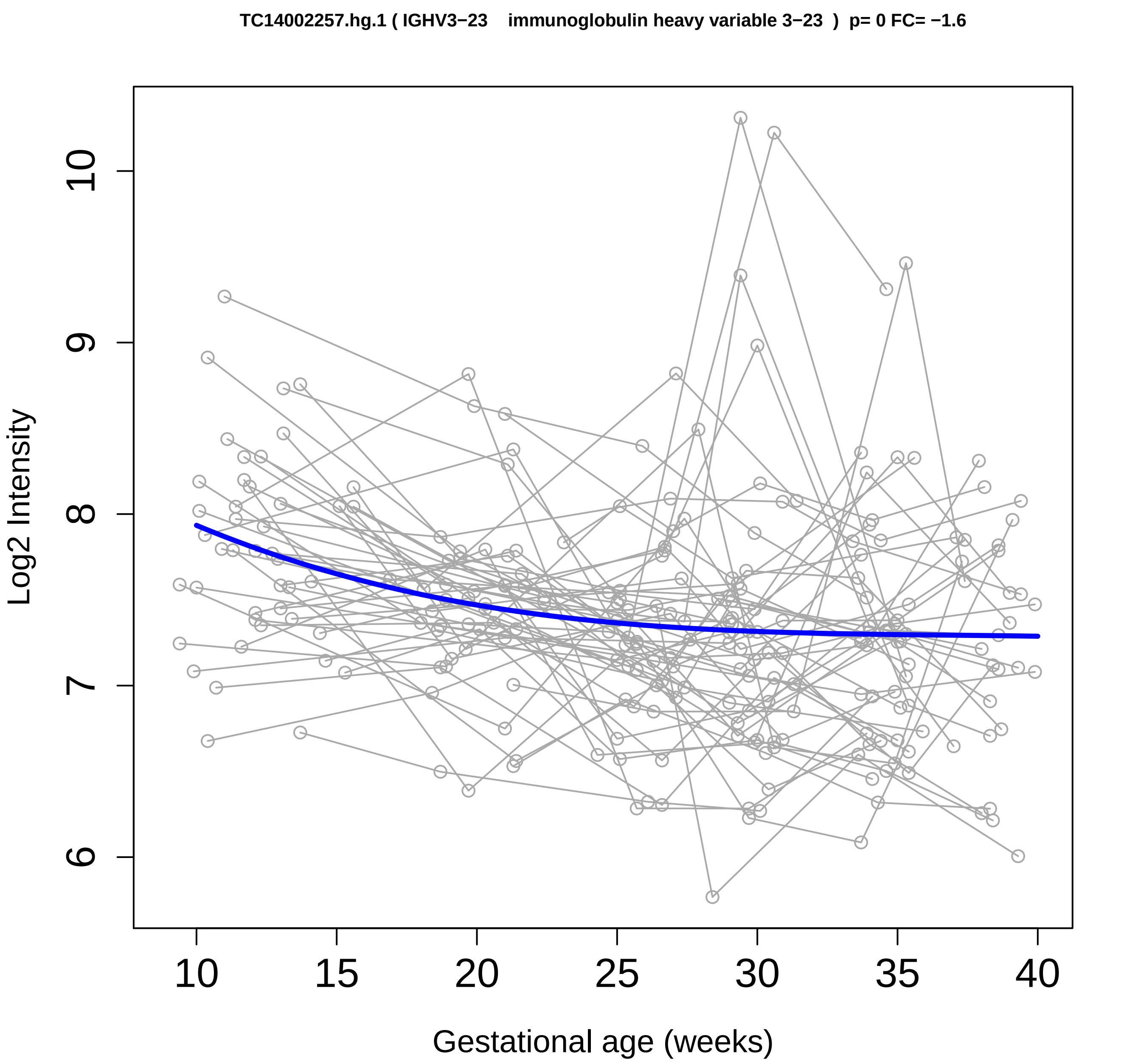
TC14002257.hg.1 (IGHV3−23 immunoglobulin heavy variable 3−23) p= 0 FC= −1.6
Log2 Intensity
6
7
8
9
10
10
15
20
25
30
35
40
Gestational age (weeks)

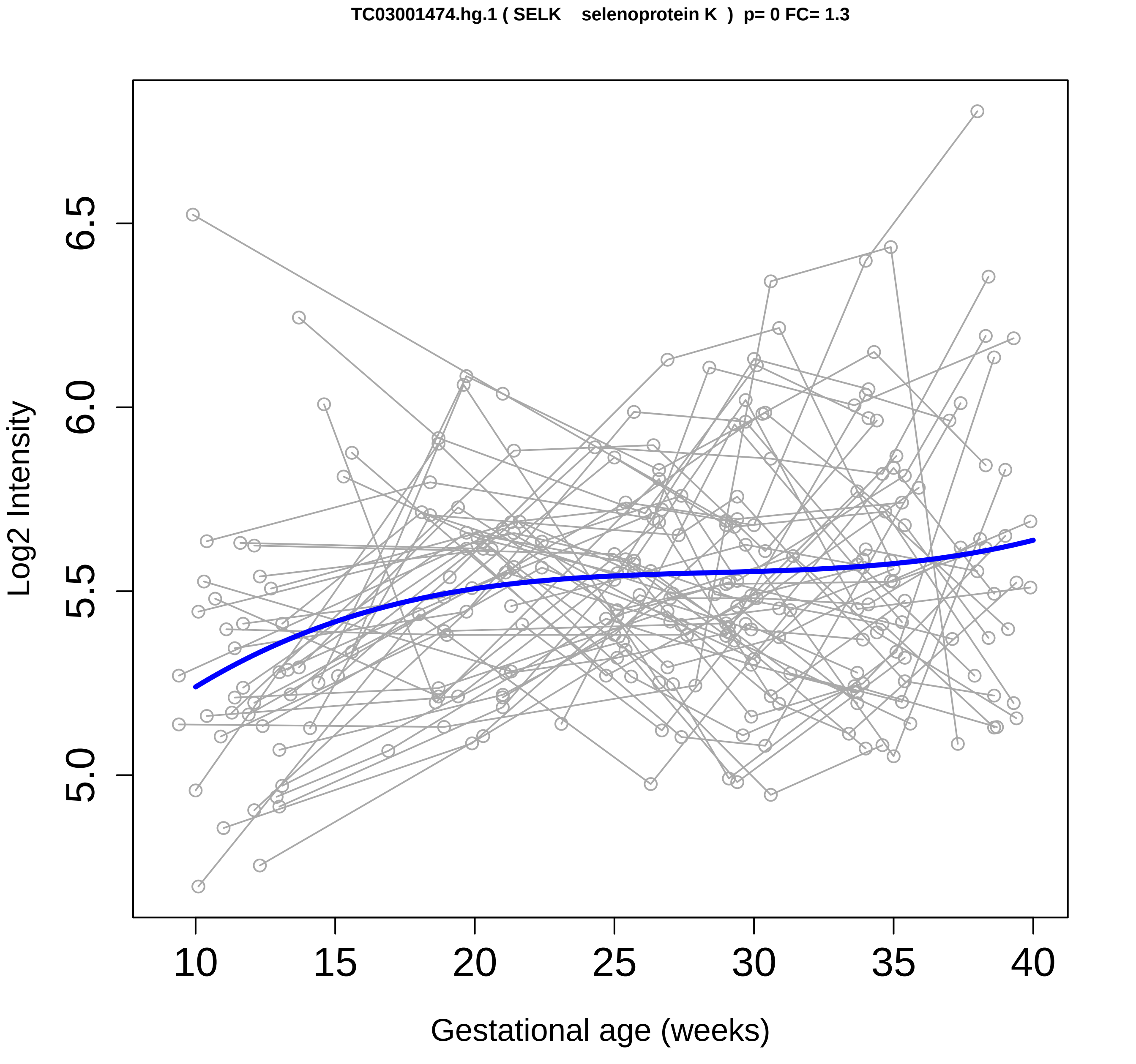
TC03001474.hg.1 (SELK   selenoprotein K) p= 0 FC= 1.3
Log2 Intensity
6.5
6.0
5.5
5.0
10
15
20
25
30
35
40
Gestational age (weeks)

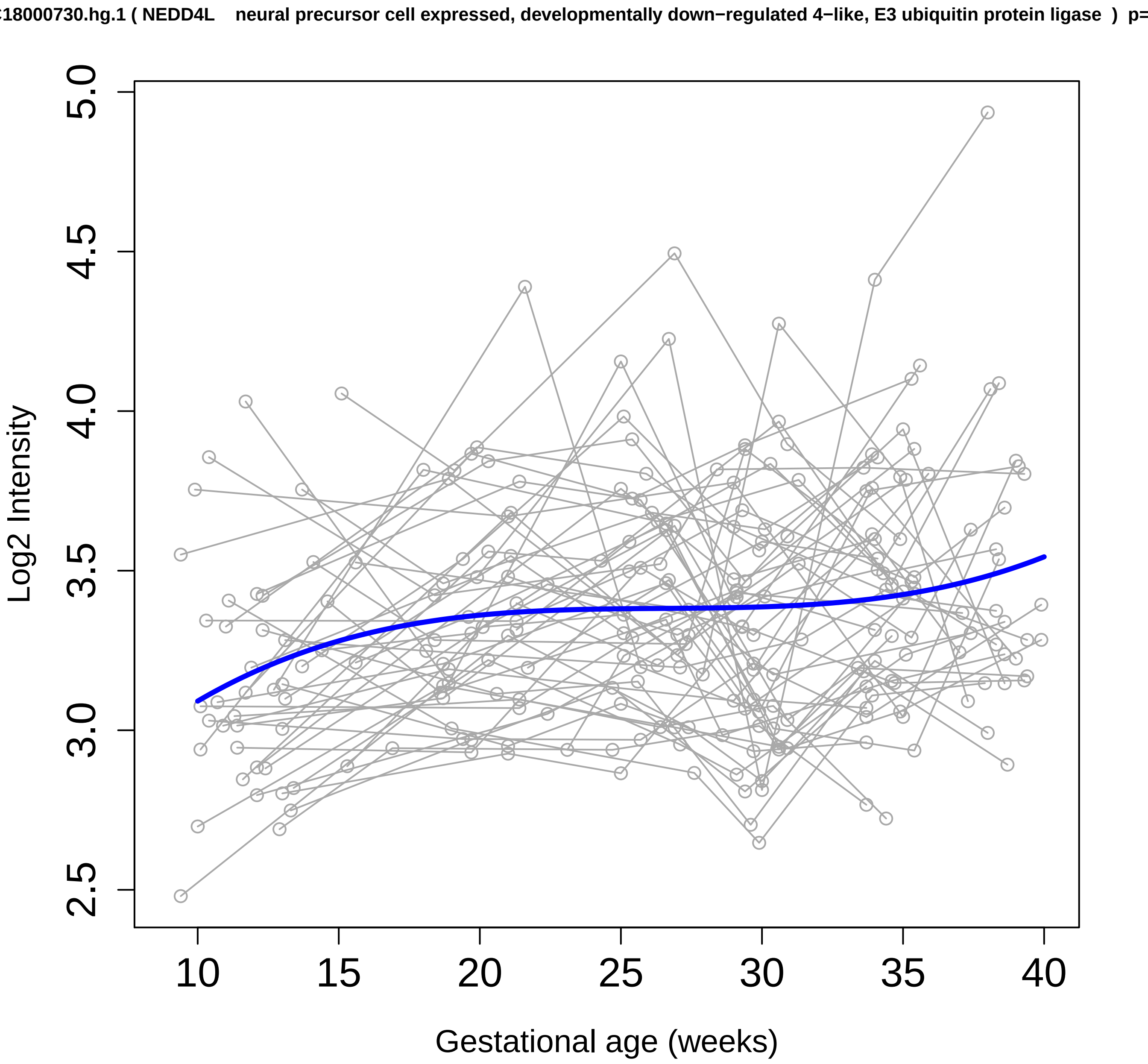

:18000730.hg.1 (NEDD4L neural precursor cell expressed, developmentally down−regulated 4−like, E3 ubiquitin protein ligase) p=
Log2 Intensity
2.5
3.0
3.5
4.0
4.5
5.0
10
15
20
25
30
35
40
Gestational age (weeks)

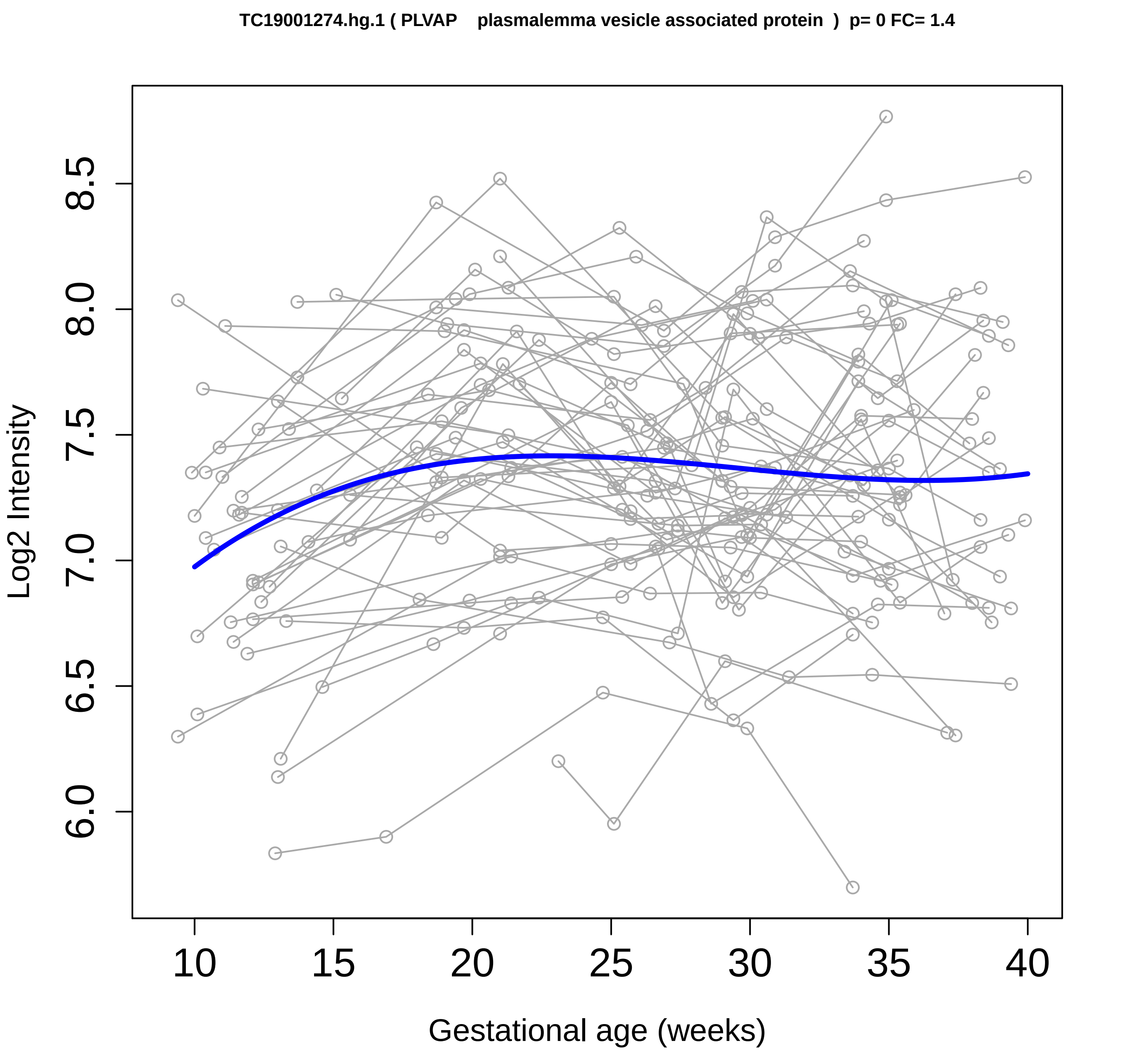
TC19001274.hg.1 (PLVAP plasmalemma vesicle associated protein) p= 0 FC= 1.4
Log2 Intensity
6.0
6.5
7.0
7.5
8.0
8.5
10
15
20
25
30
35
40
Gestational age (weeks)

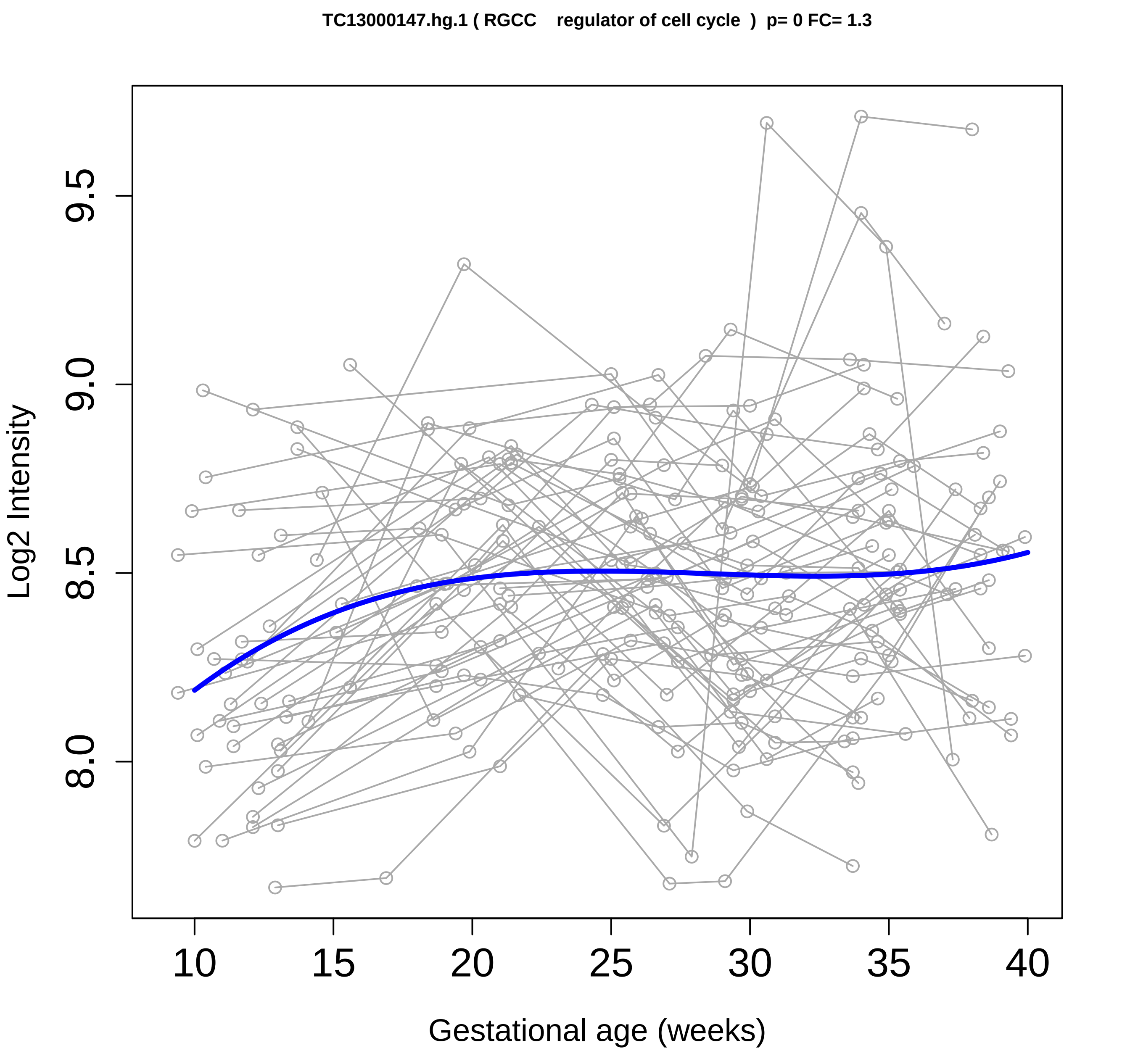
TC13000147.hg.1 (RGCC regulator of cell cycle) p= 0 FC= 1.3
Log2 Intensity
8.0
8.5
9.0
9.5
10
15
20
25
30
35
40
Gestational age (weeks)

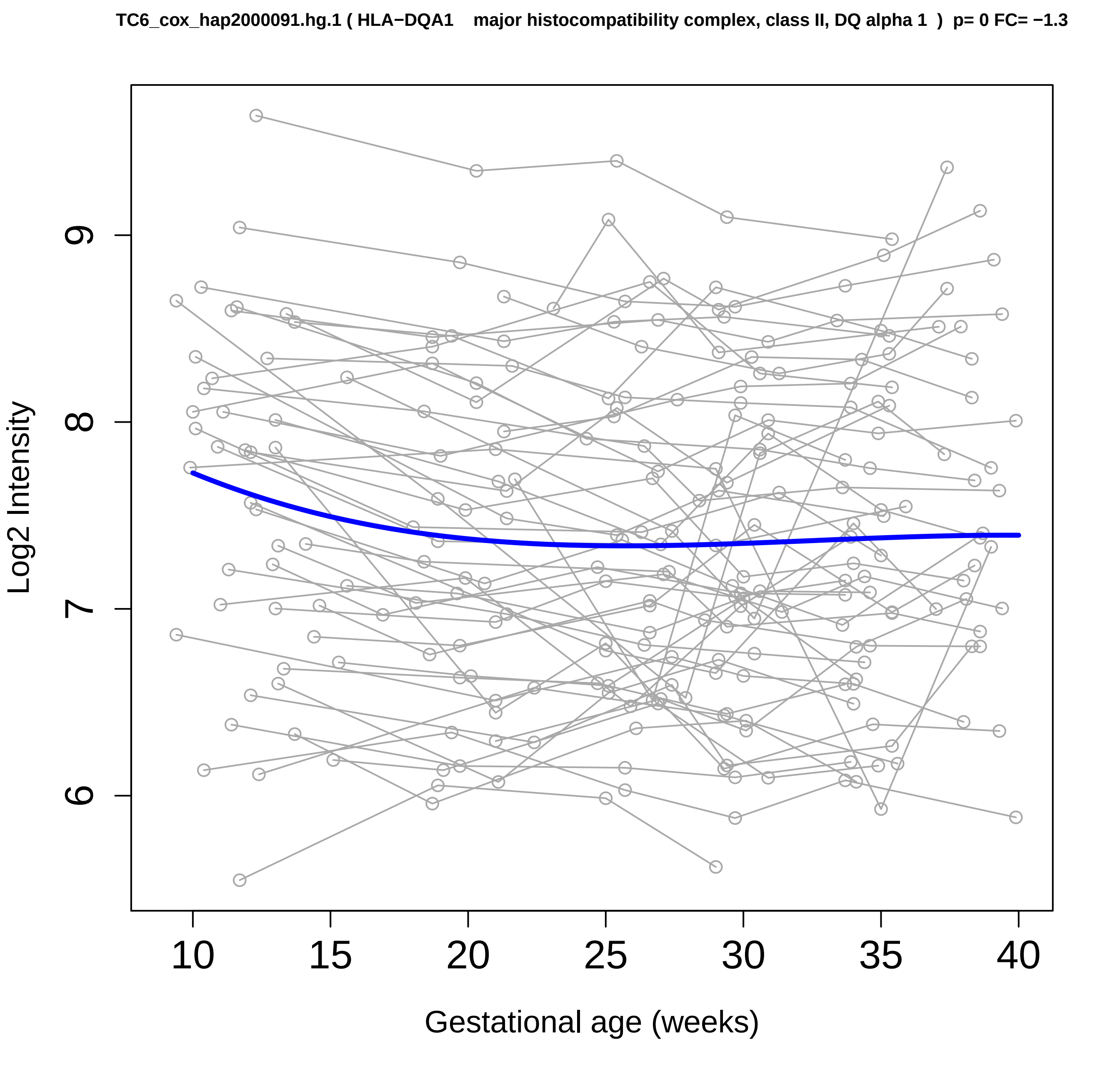

TC6_cox_hap2000091.hg.1 (HLA−DQA1 major histocompatibility complex, class II, DQ alpha 1) p= 0 FC= −1.3
Log2 Intensity
6
7
8
9
10
15
20
25
30
35
40
Gestational age (weeks)

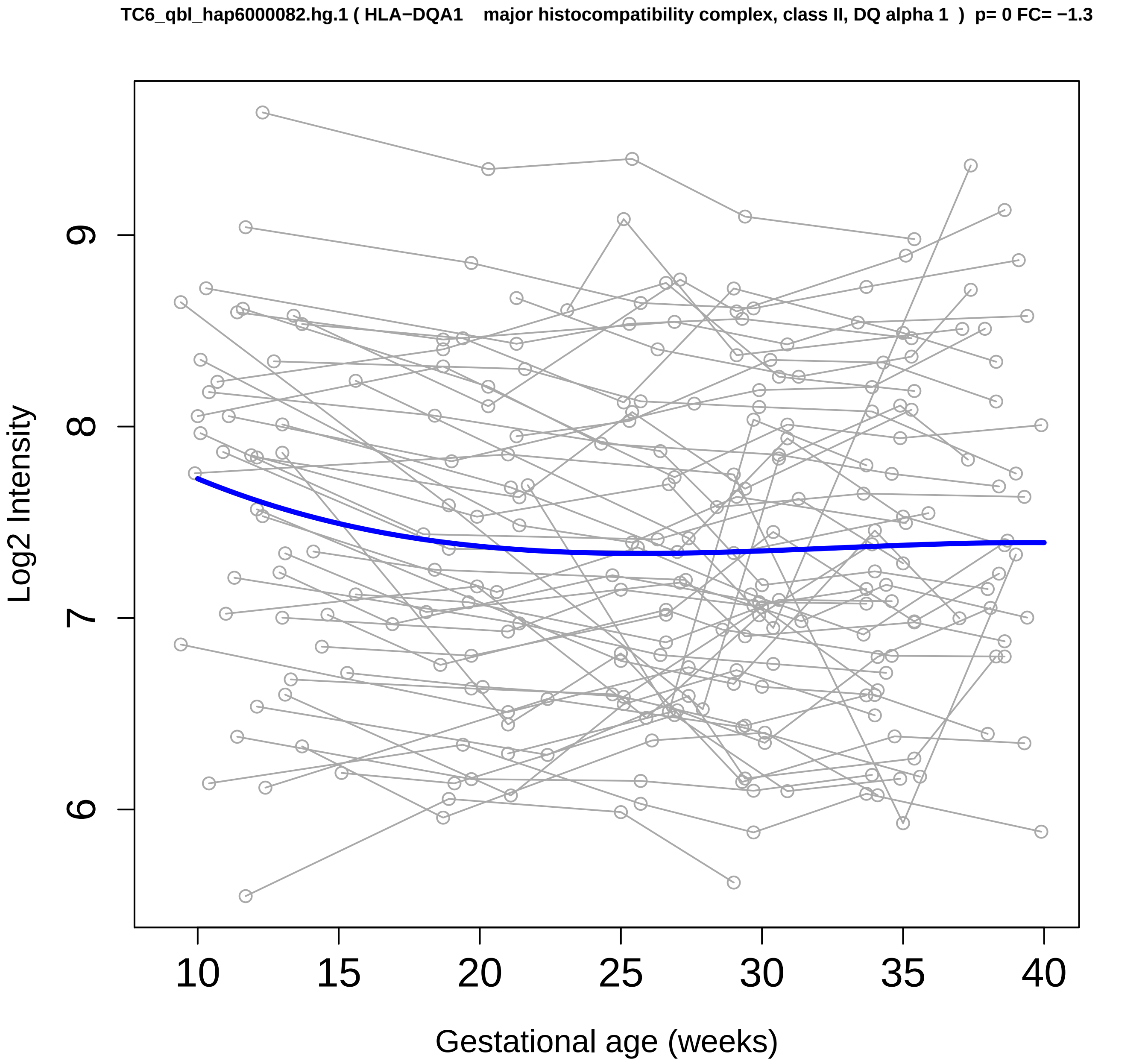

TC6_qbl_hap6000082.hg.1 (HLA−DQA1 major histocompatibility complex, class II, DQ alpha 1) p= 0 FC= −1.3
Log2 Intensity
6
7
8
9
10
15
20
25
30
35
40
Gestational age (weeks)

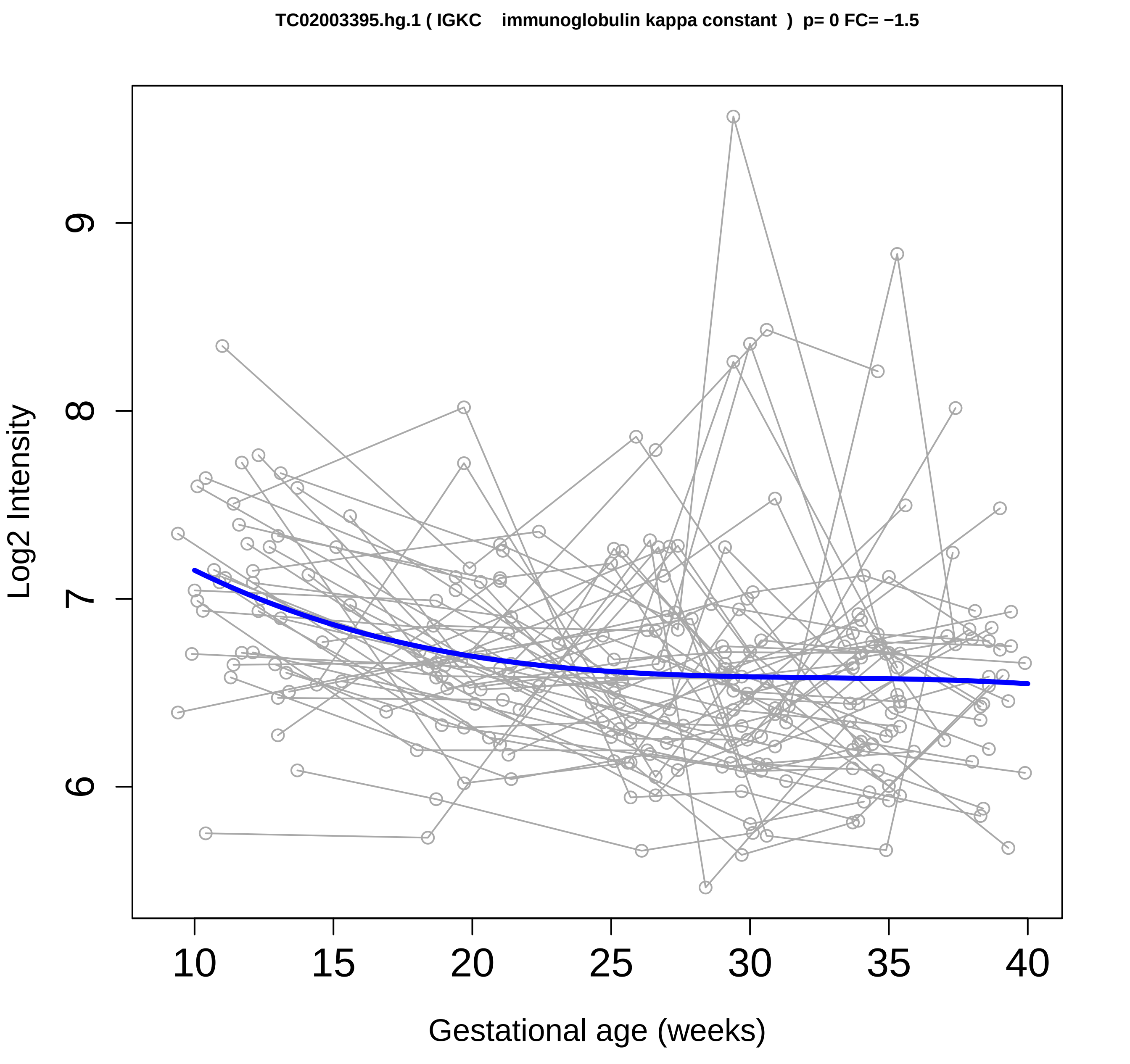

TC02003395.hg.1 (IGKC immunoglobulin kappa constant) p= 0 FC= −1.5
Log2 Intensity
6
7
8
9
10
15
20
25
30
35
40
Gestational age (weeks)

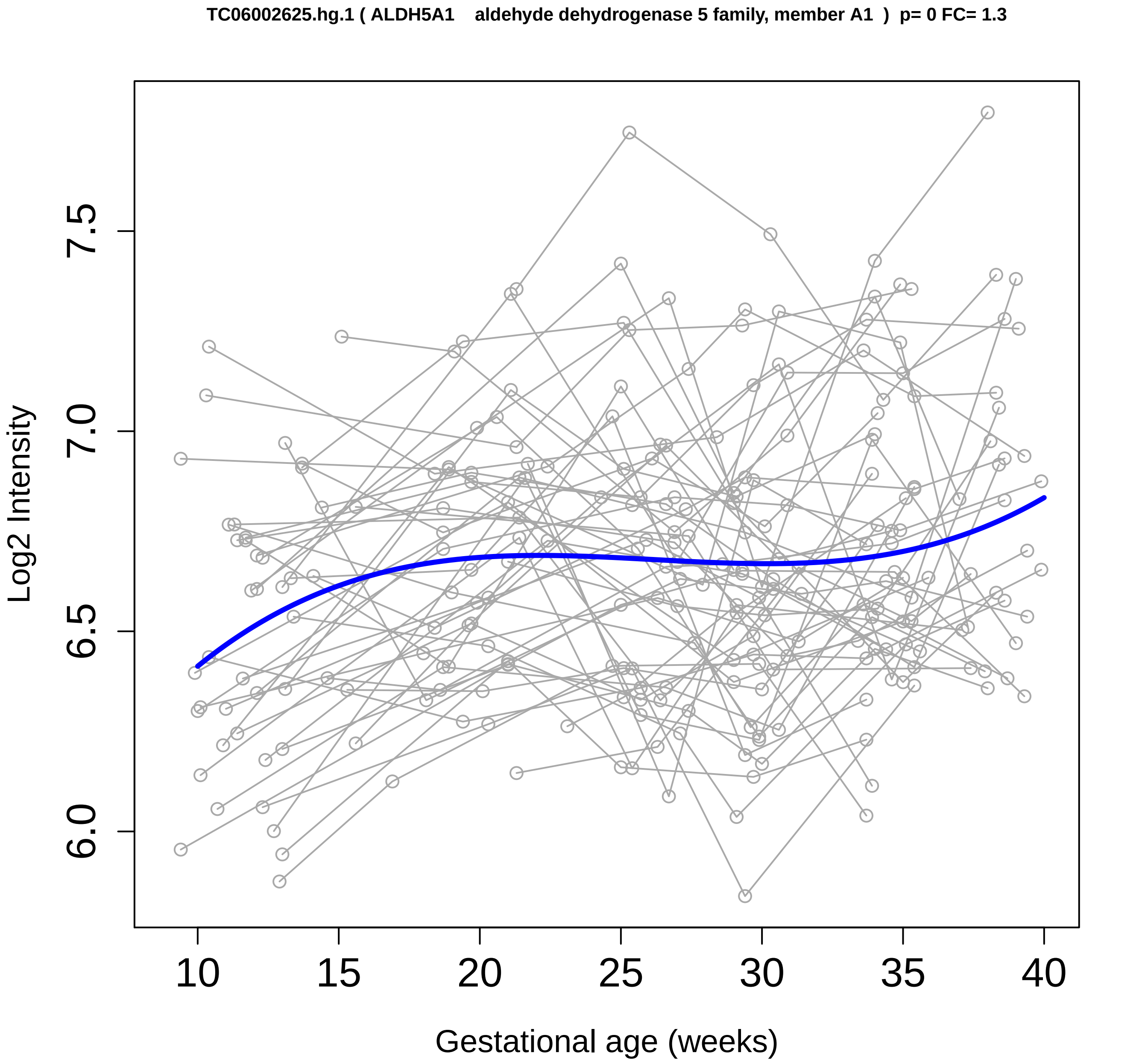

TC06002625.hg.1 (ALDH5A1 aldehyde dehydrogenase 5 family, member A1) p= 0 FC= 1.3
Log2 Intensity
6.0
6.5
7.0
7.5
10
15
20
25
30
35
40
Gestational age (weeks)

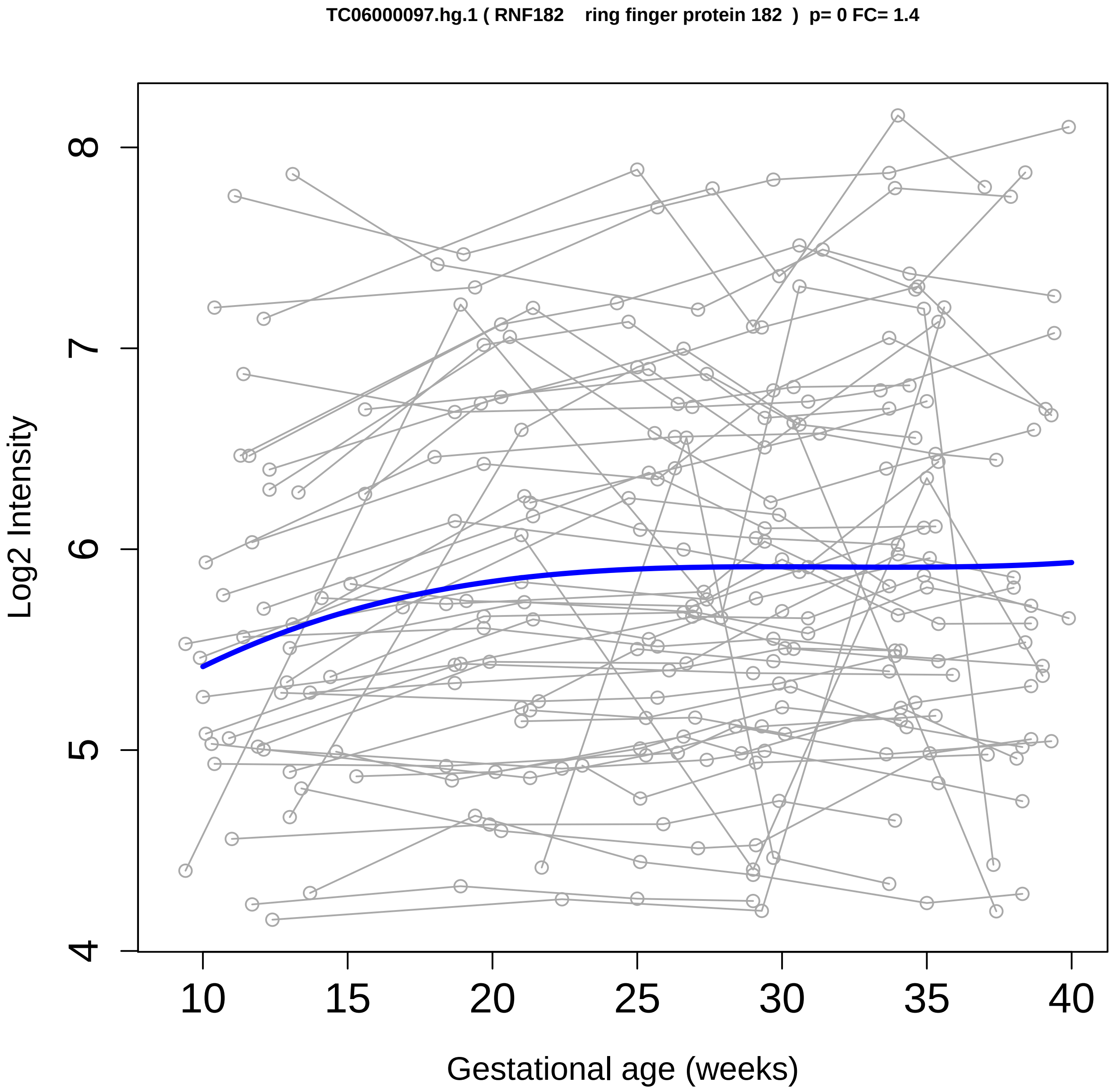
TC06000097.hg.1 (RNF182 ring finger protein 182) p= 0 FC= 1.4
Log2 Intensity
4
5
6
7
8
10
15
20
25
30
35
40
Gestational age (weeks)

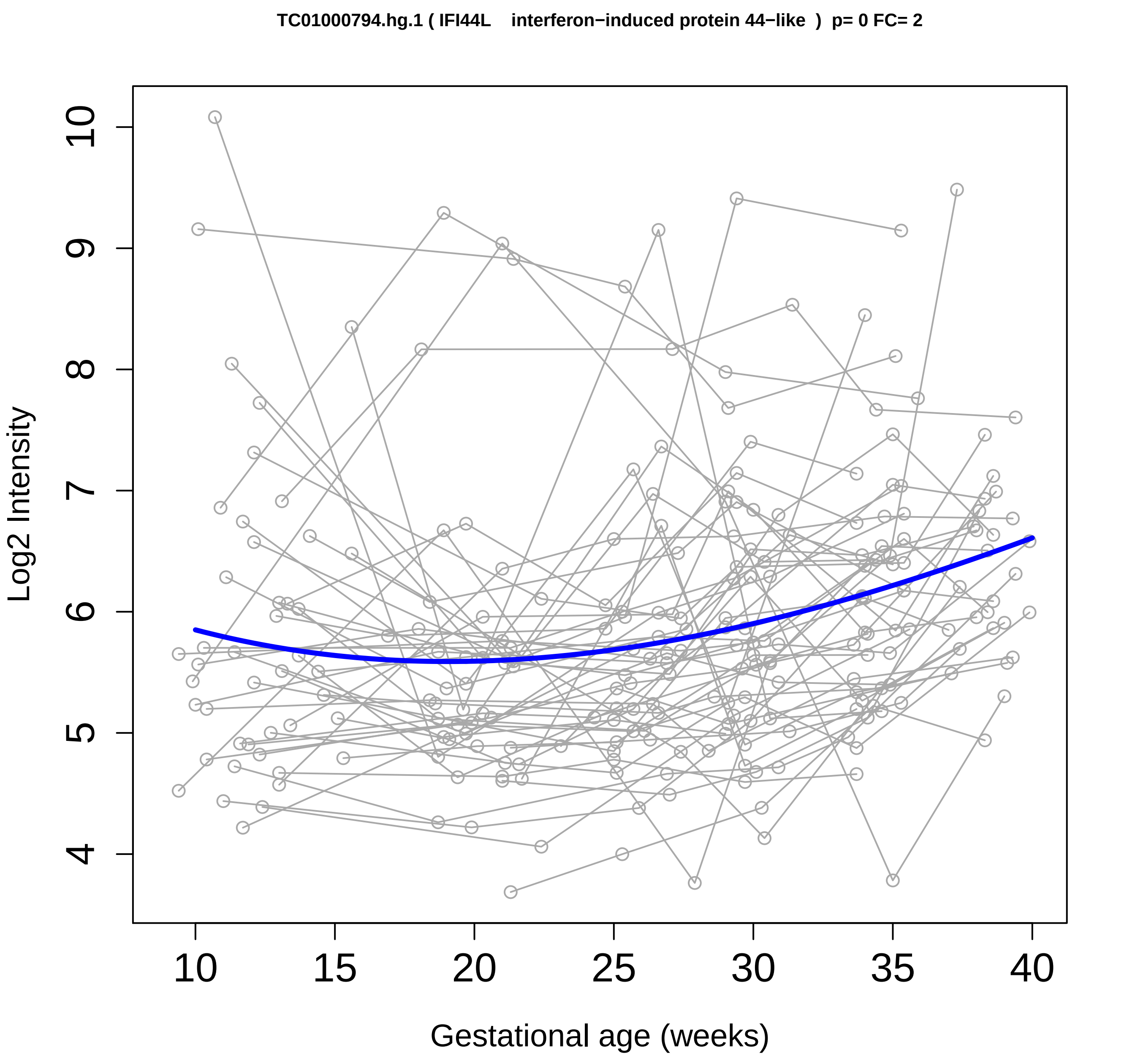
TC01000794.hg.1 (IFI44L interferon−induced protein 44−like) p= 0 FC= 2
Log2 Intensity
4
5
6
7
8
9
10
10
15
20
25
30
35
40
Gestational age (weeks)

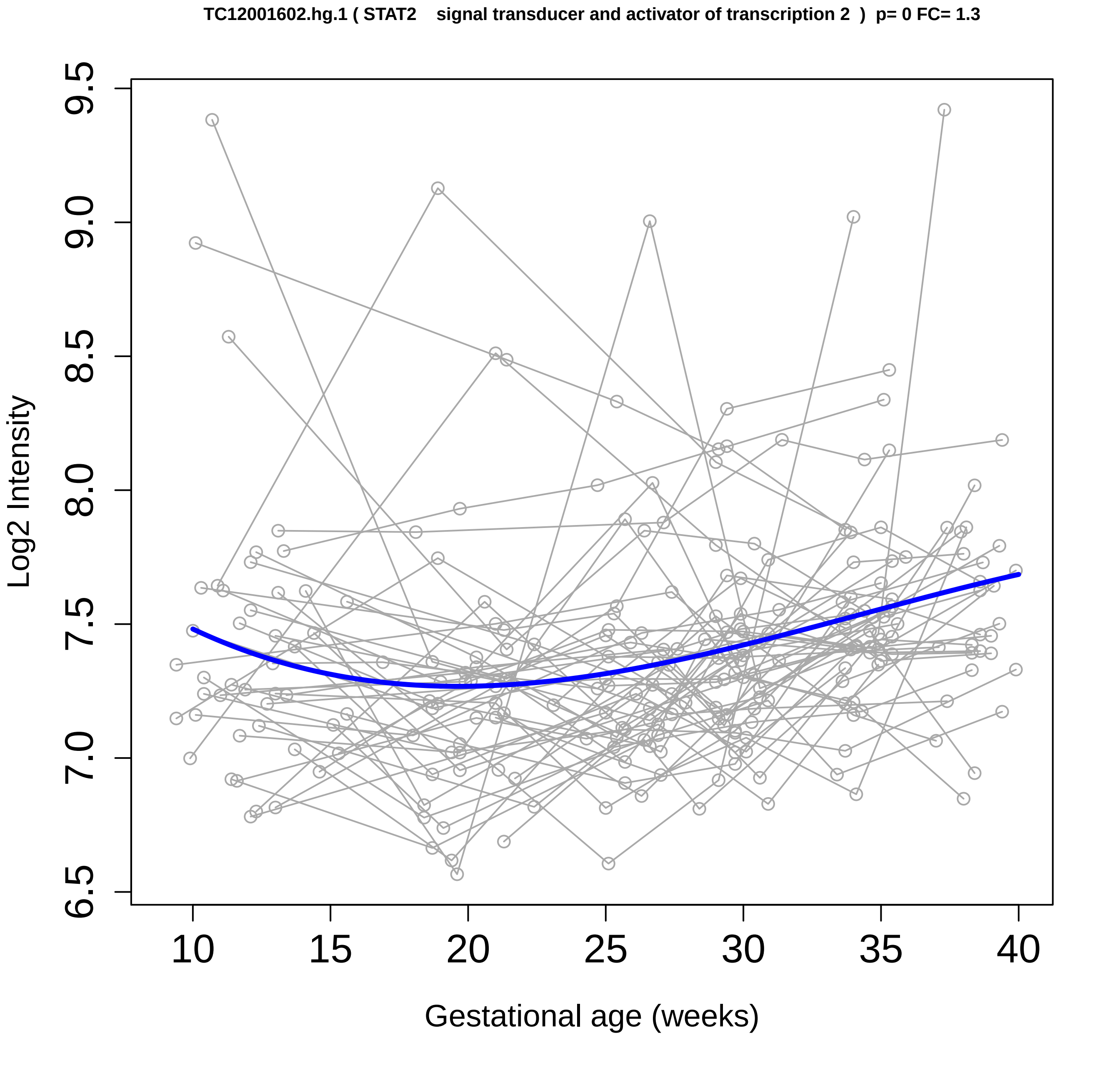

TC12001602.hg.1 (STAT2 signal transducer and activator of transcription 2) p= 0 FC= 1.3
Log2 Intensity
6.5
7.0
7.5
8.0
8.5
9.0
9.5
10
15
20
25
30
35
40
Gestational age (weeks)

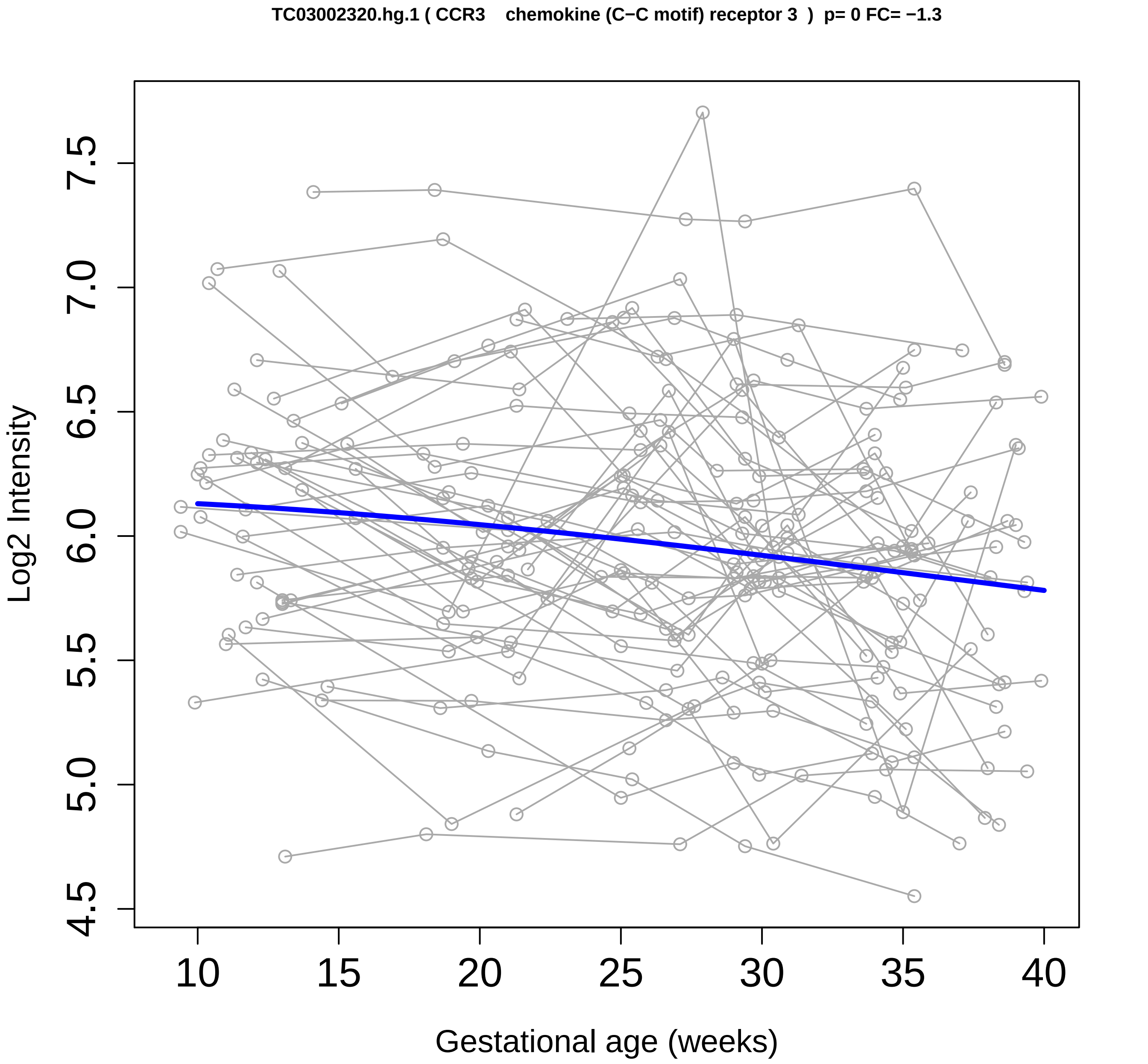
TC03002320.hg.1 (CCR3   chemokine (C−C motif) receptor 3) p= 0 FC= −1.3
Log2 Intensity
4.5
5.0
5.5
6.0
6.5
7.0
7.5
10
15
20
25
30
35
40
Gestational age (weeks)

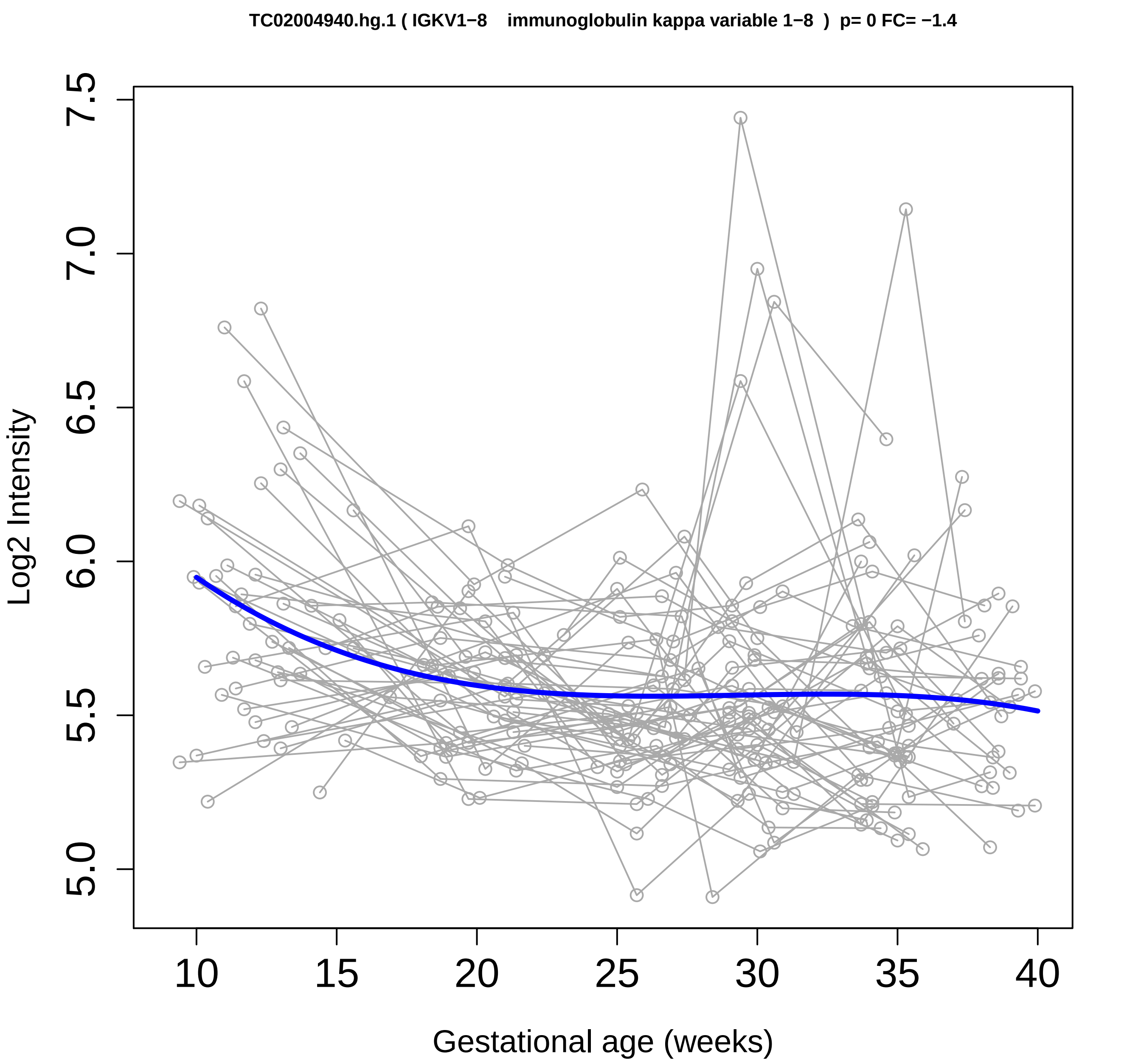
TC02004940.hg.1 (IGKV1−8 immunoglobulin kappa variable 1−8) p= 0 FC= −1.4
Log2 Intensity
7.5
7.0
6.5
6.0
5.5
5.0
10
15
20
25
30
35
40
Gestational age (weeks)

TC02004942.hg.1 (IGKV3−11   immunoglobulin kappa variable 3−11) p= 0 FC= −1.4

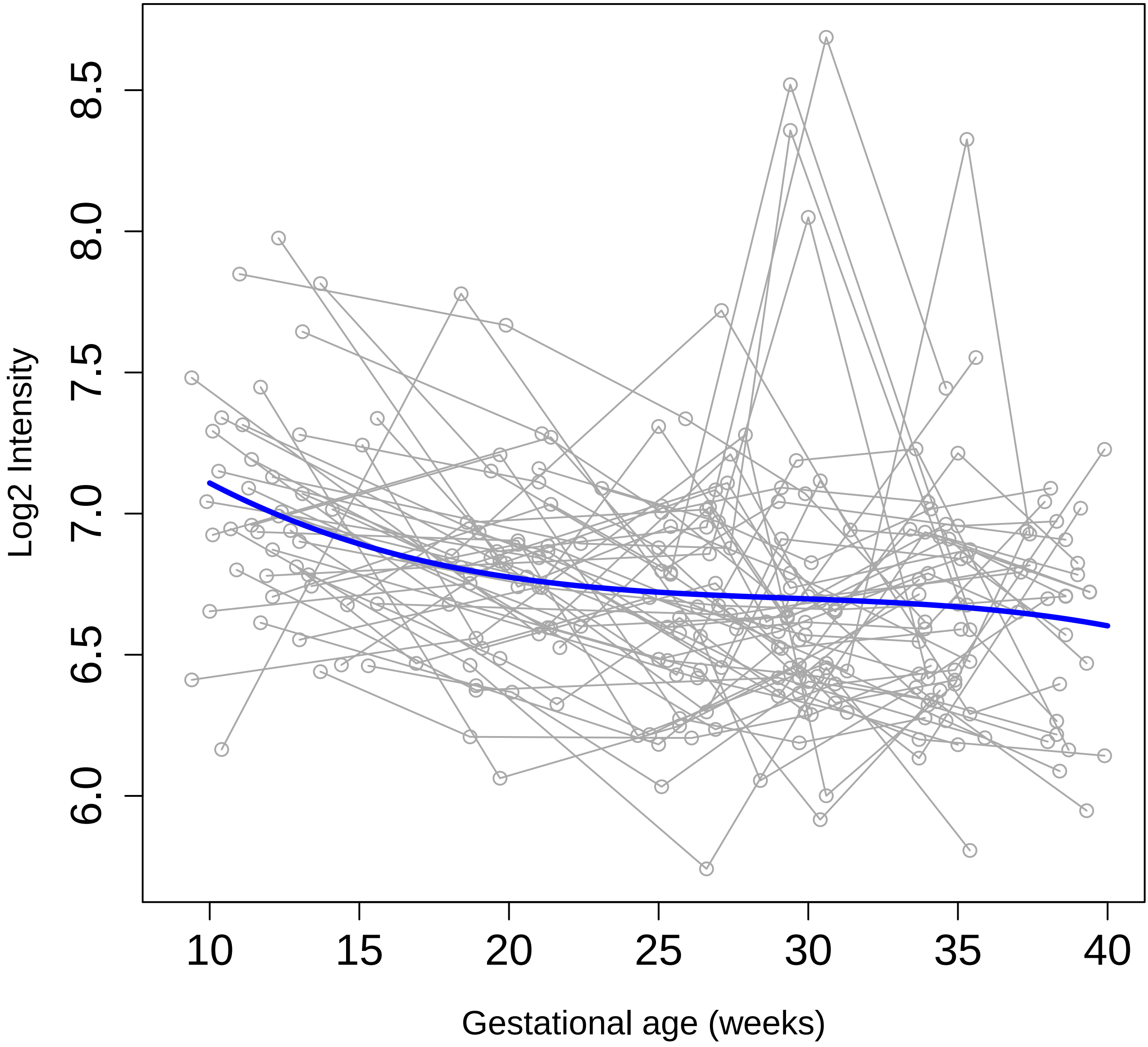

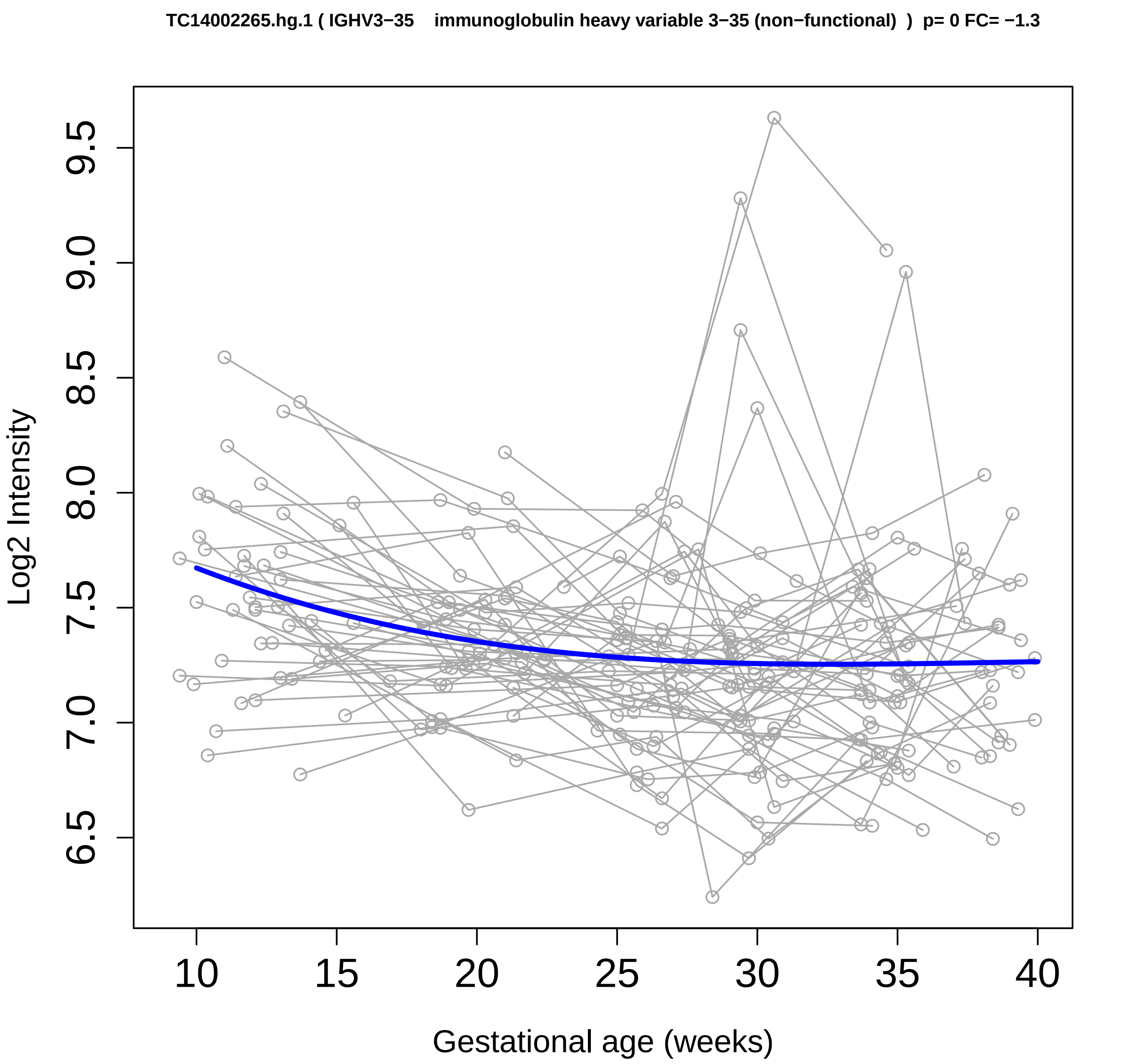

TC14002265.hg.1 (IGHV3−35 immunoglobulin heavy variable 3−35 (non−functional)) p= 0 FC= −1.3
Log2 Intensity
6.5
7.0
7.5
8.0
8.5
9.0
9.5
10
15
20
25
30
35
40
Gestational age (weeks)

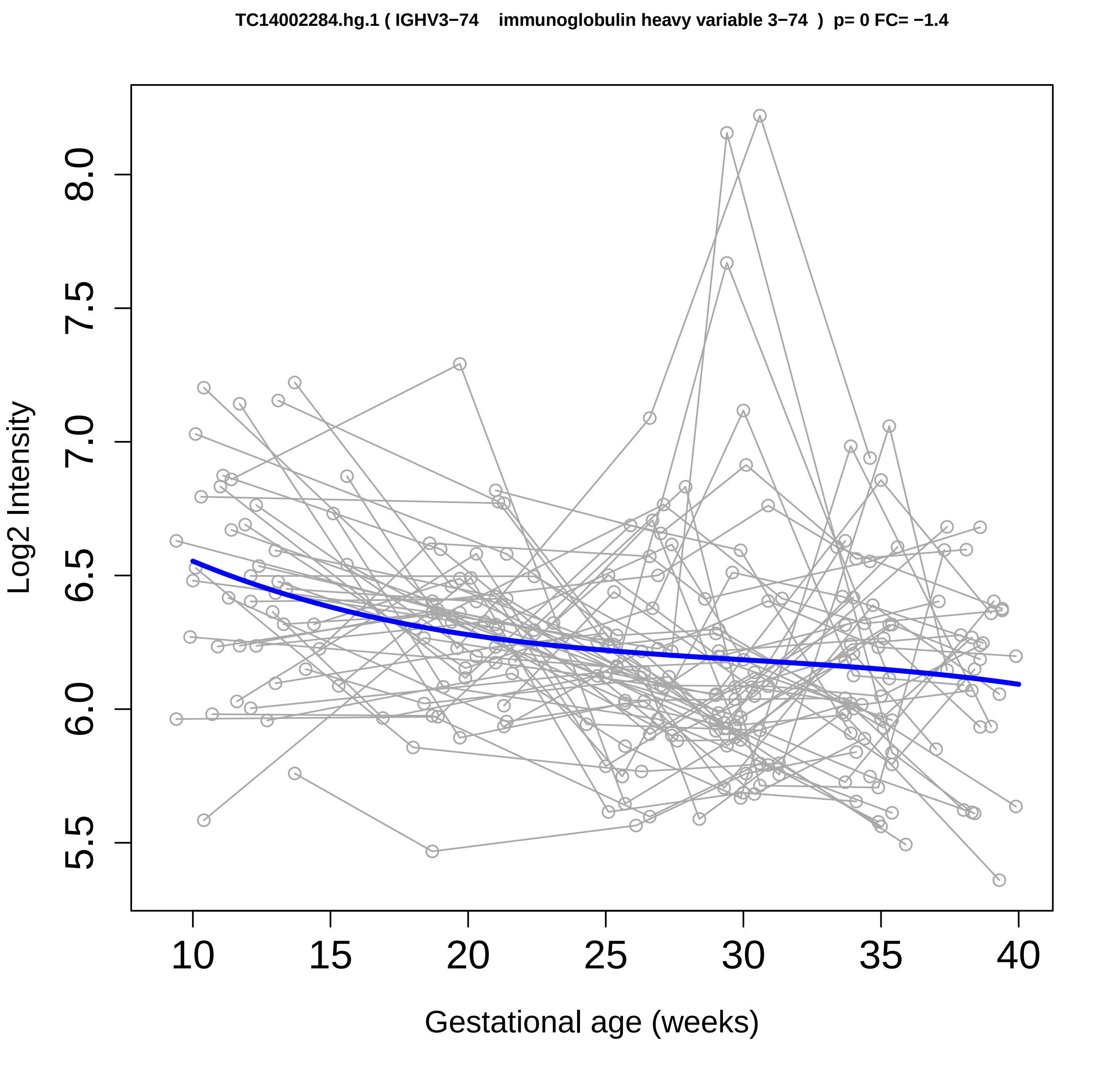

TC14002284.hg.1 (IGHV3−74 immunoglobulin heavy variable 3−74) p= 0 FC= −1.4
Log2 Intensity
5.5
6.0
6.5
7.0
7.5
8.0
10
15
20
25
30
35
40
Gestational age (weeks)

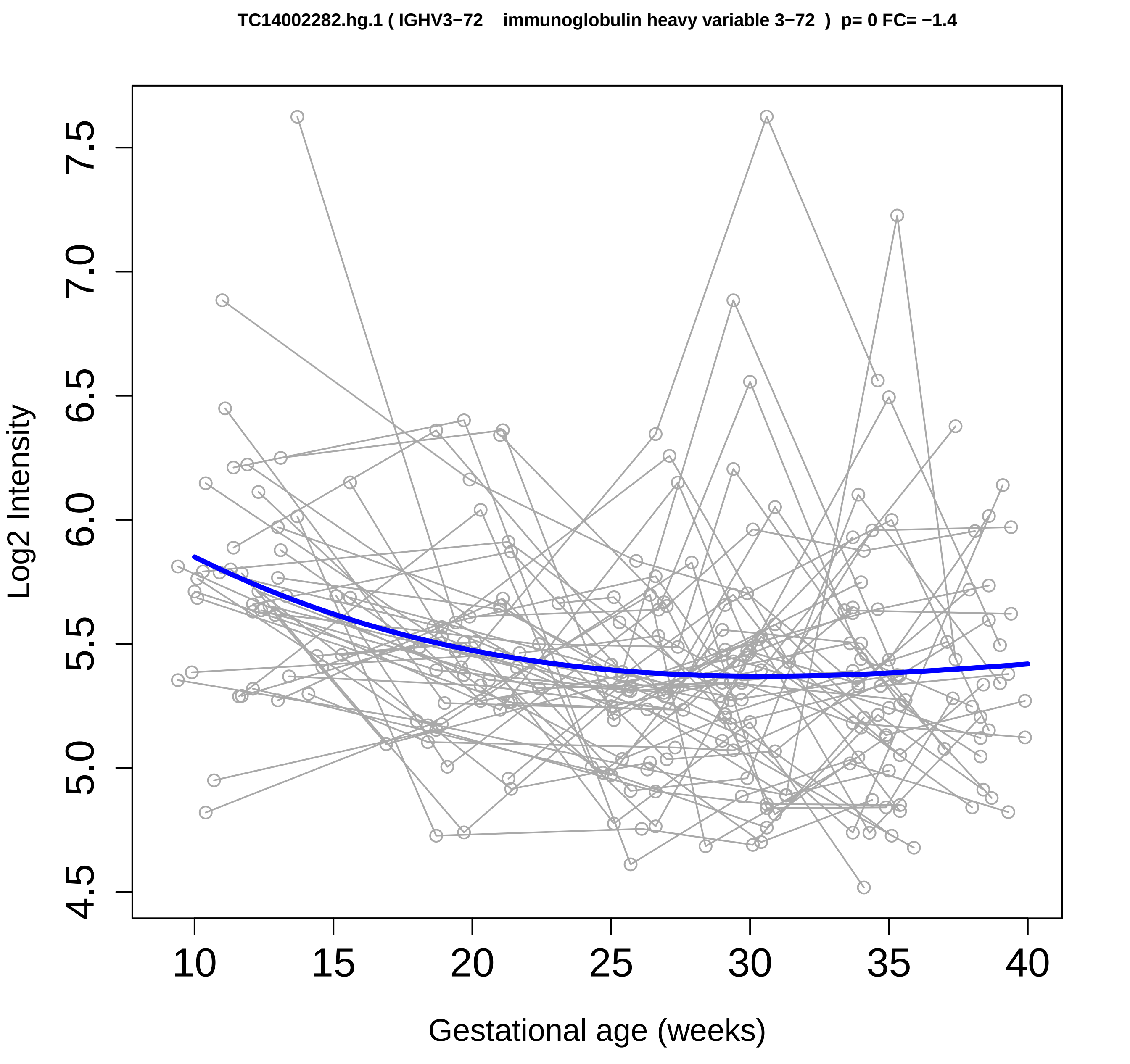

TC14002282.hg.1 (IGHV3−72 immunoglobulin heavy variable 3−72) p= 0 FC= −1.4
Log2 Intensity
7.5
7.0
6.5
6.0
5.5
5.0
4.5
10
15
20
25
30
35
40
Gestational age (weeks)

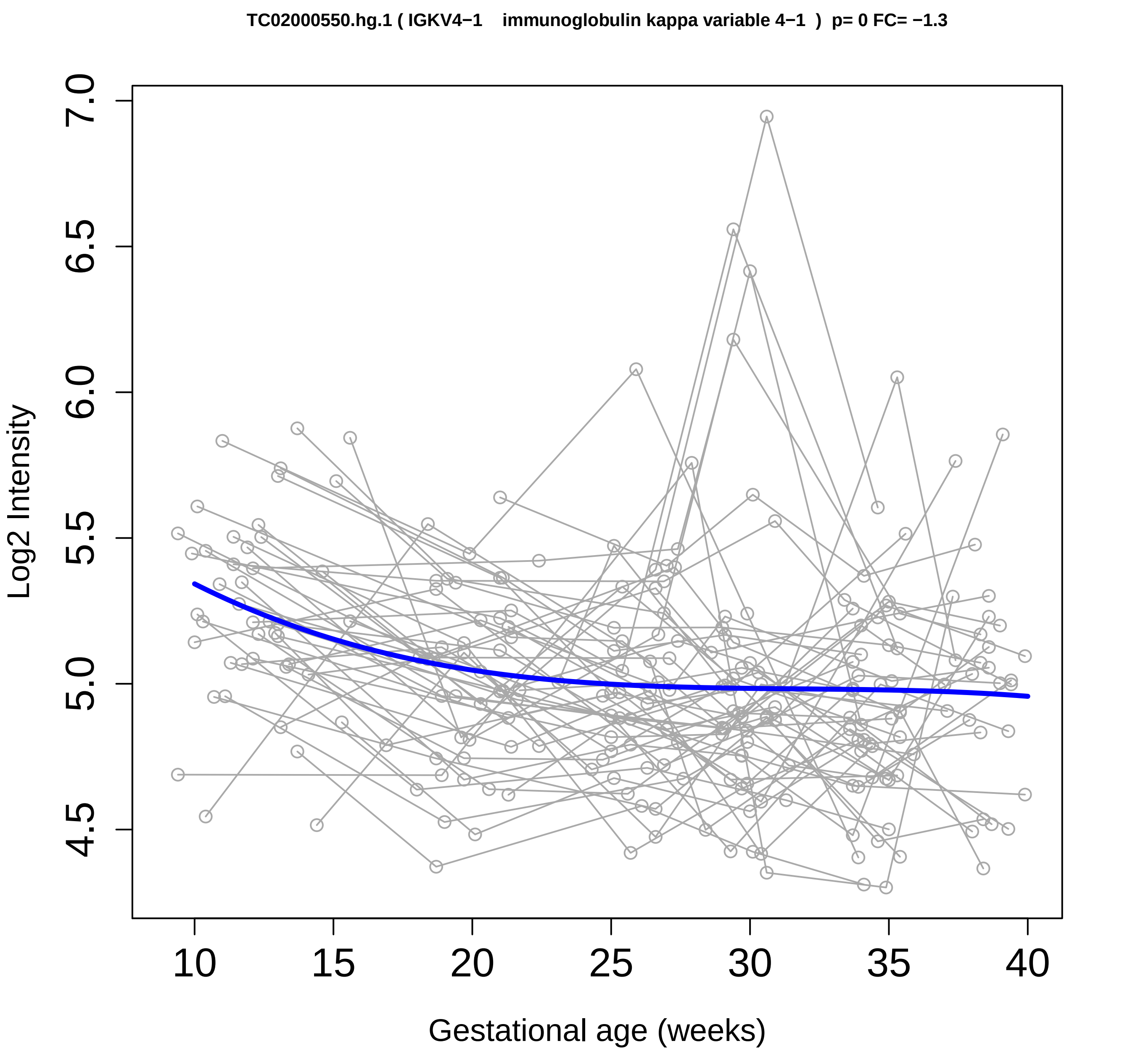
TC02000550.hg.1 (IGKV4−1 immunoglobulin kappa variable 4−1) p= 0 FC= −1.3
Log2 Intensity
7.0
6.5
6.0
5.5
5.0
4.5
10
15
20
25
30
35
40
Gestational age (weeks)

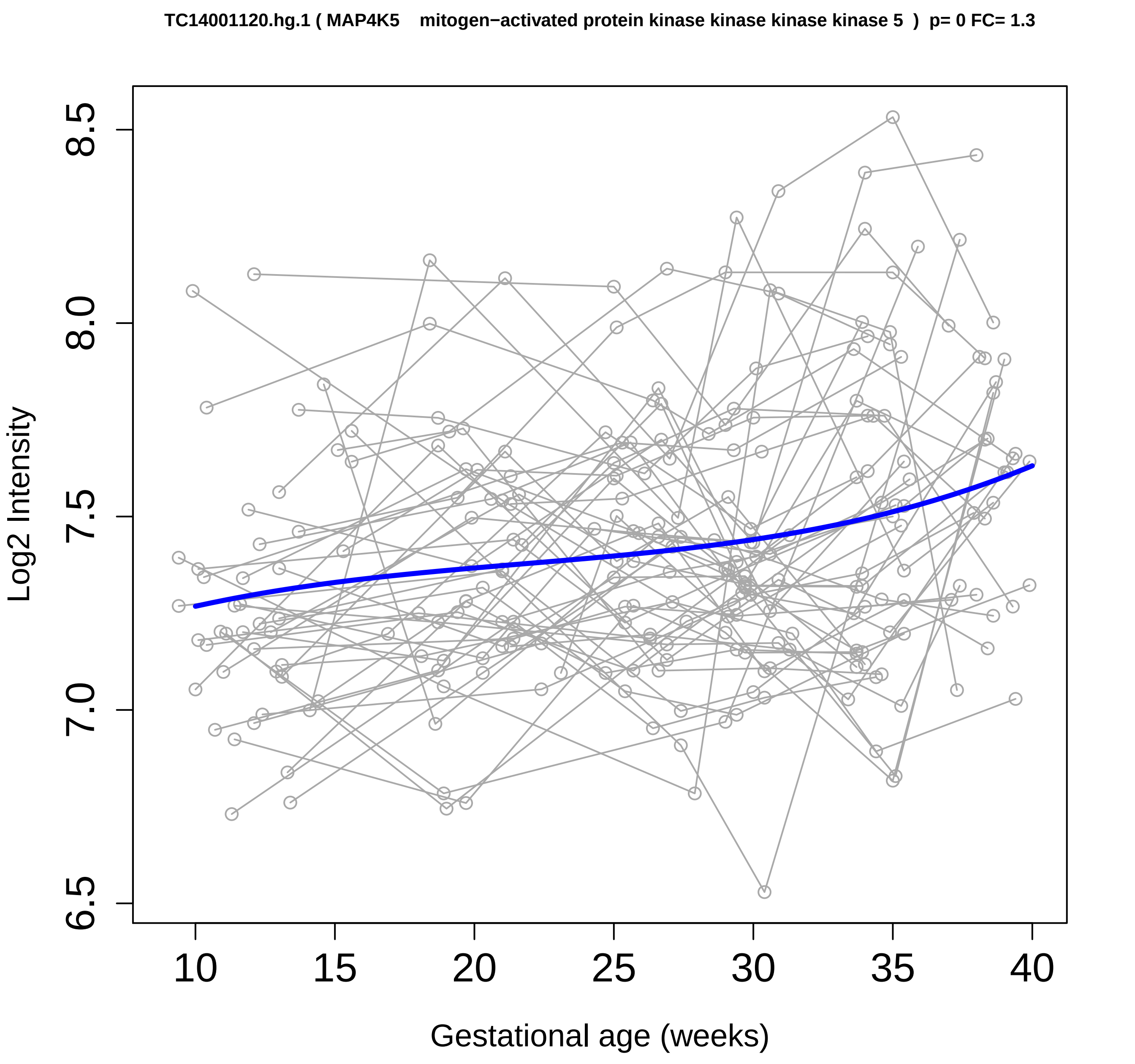

TC14001120.hg.1 (MAP4K5 mitogen−activated protein kinase kinase kinase kinase 5) p= 0 FC= 1.3
Log2 Intensity
6.5
7.0
7.5
8.0
8.5
10
15
20
25
30
35
40
Gestational age (weeks)

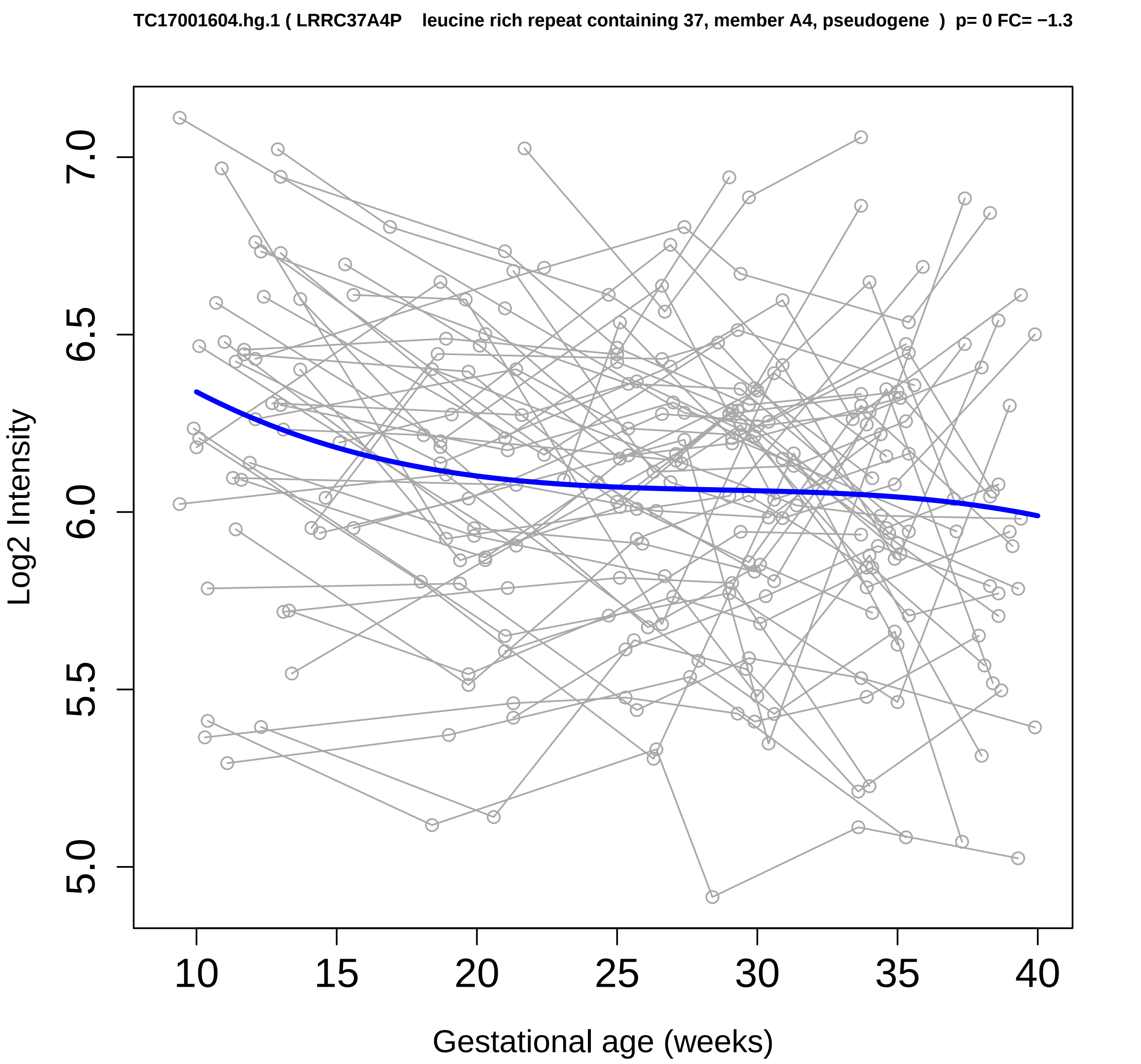

TC17001604.hg.1 (LRRC37A4P leucine rich repeat containing 37, member A4, pseudogene) p= 0 FC= −1.3
Log2 Intensity
7.0
6.5
6.0
5.5
5.0
10
15
20
25
30
35
40
Gestational age (weeks)

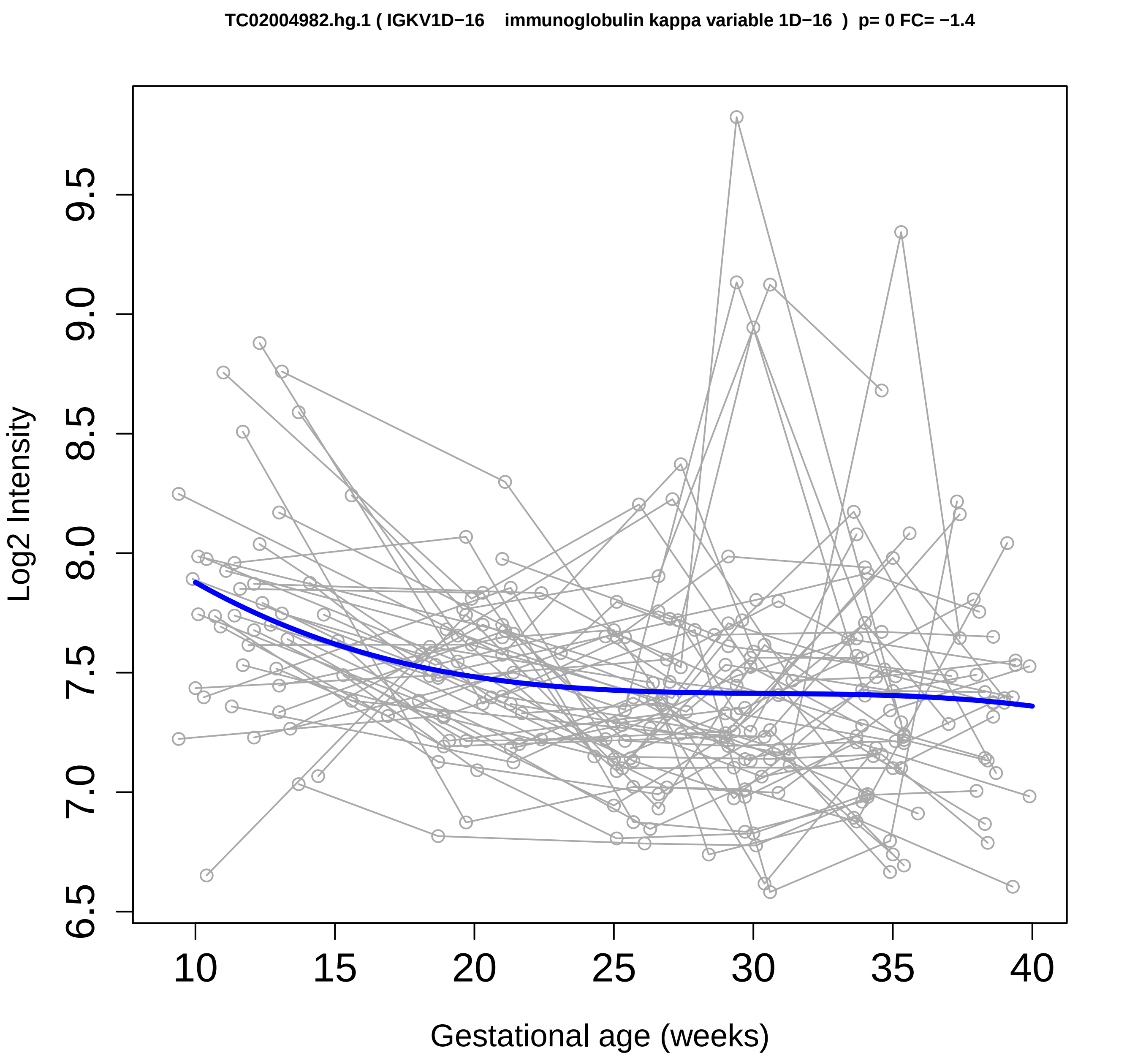

TC02004982.hg.1 (IGKV1D−16 immunoglobulin kappa variable 1D−16) p= 0 FC= −1.4
Log2 Intensity
6.5
7.0
7.5
8.0
8.5
9.0
9.5
10
15
20
25
30
35
40
Gestational age (weeks)

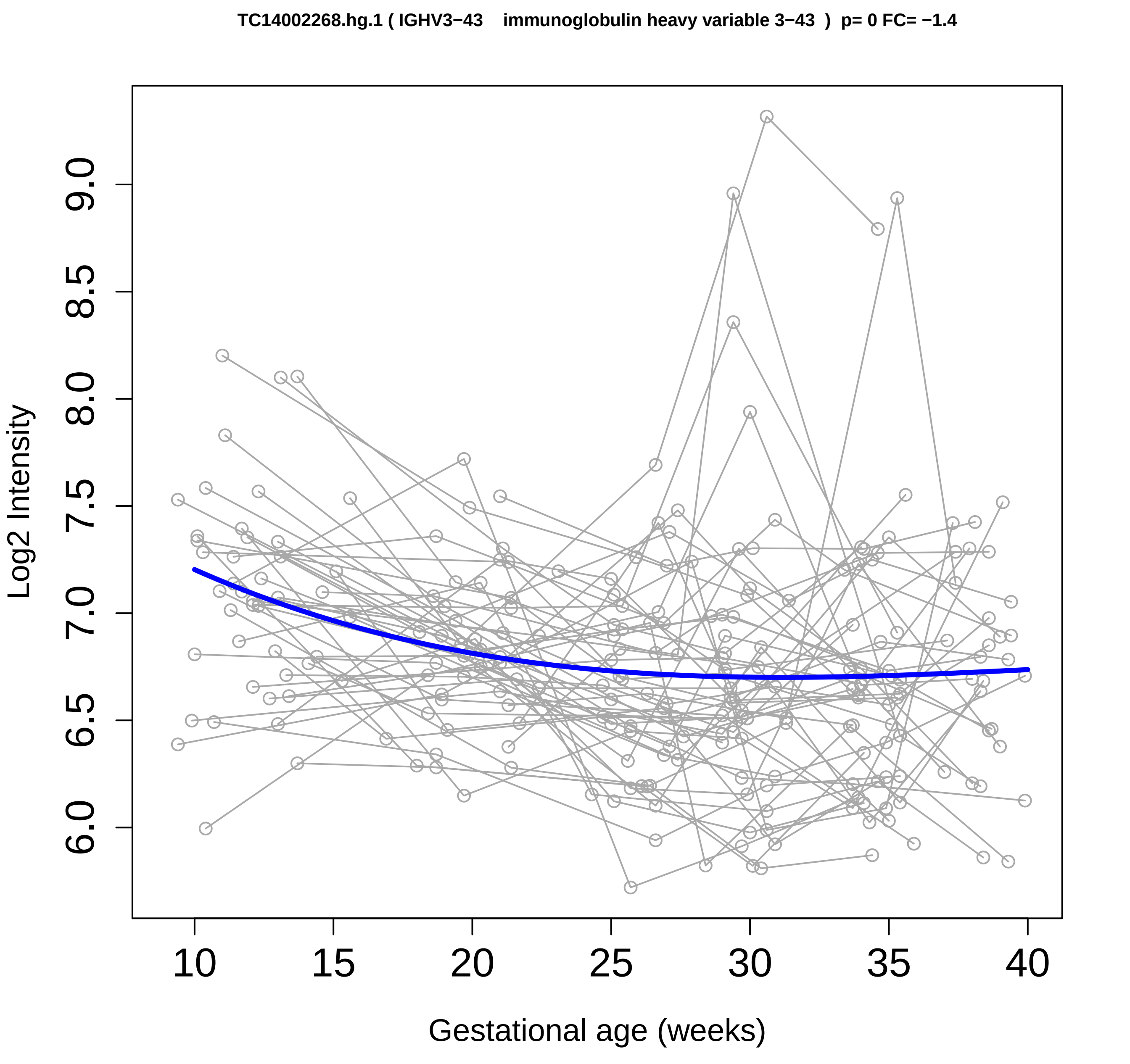

TC14002268.hg.1 (IGHV3−43 immunoglobulin heavy variable 3−43) p= 0 FC= −1.4
Log2 Intensity
6.0
6.5
7.0
7.5
8.0
8.5
9.0
10
15
20
25
30
35
40
Gestational age (weeks)

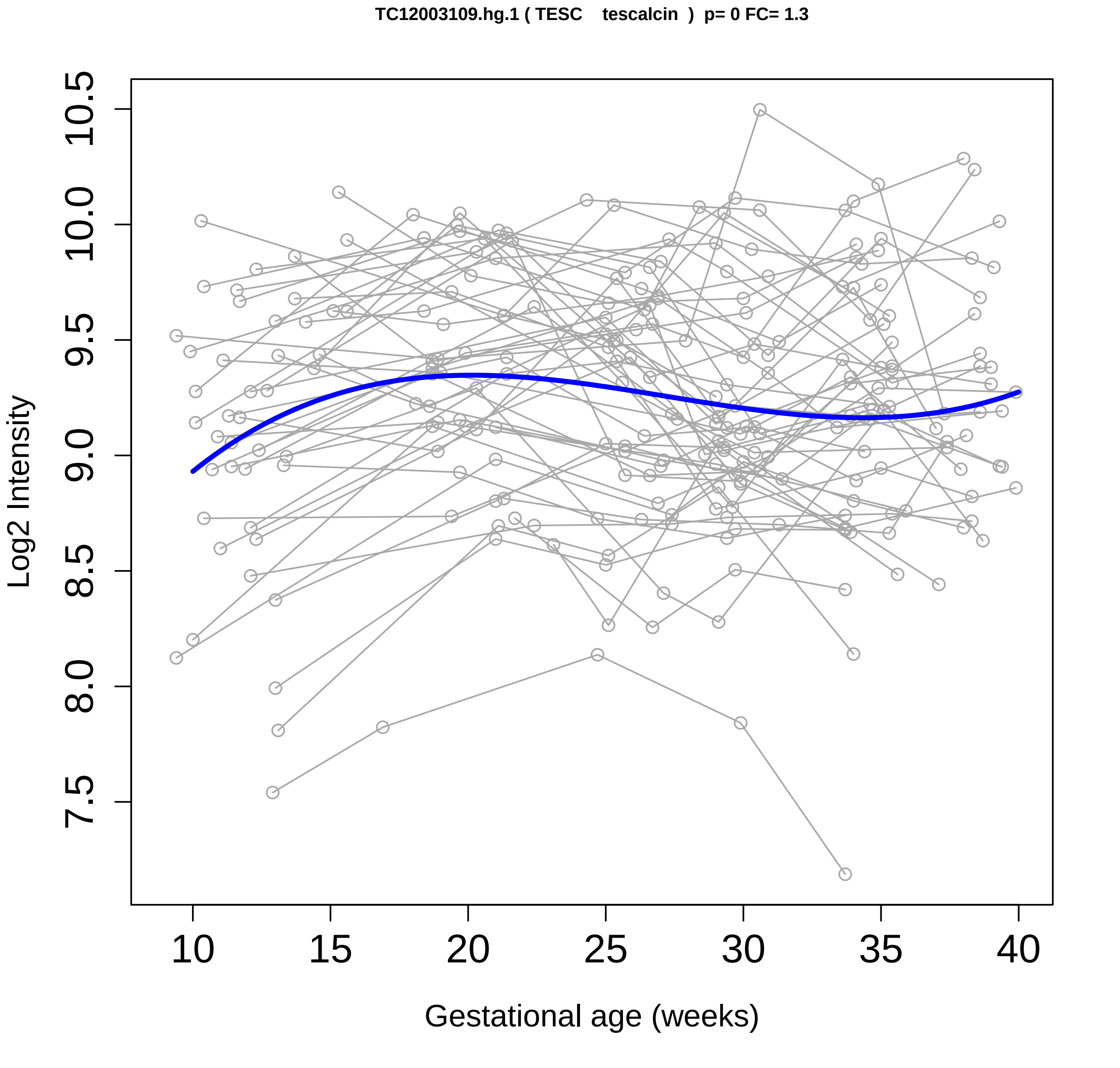

TC12003109.hg.1 (TESC tescalcin) p= 0 FC= 1.3
Log2 Intensity
7.5
8.0
8.5
9.0
9.5
10.0
10.5
10
15
20
25
30
35
40
Gestational age (weeks)

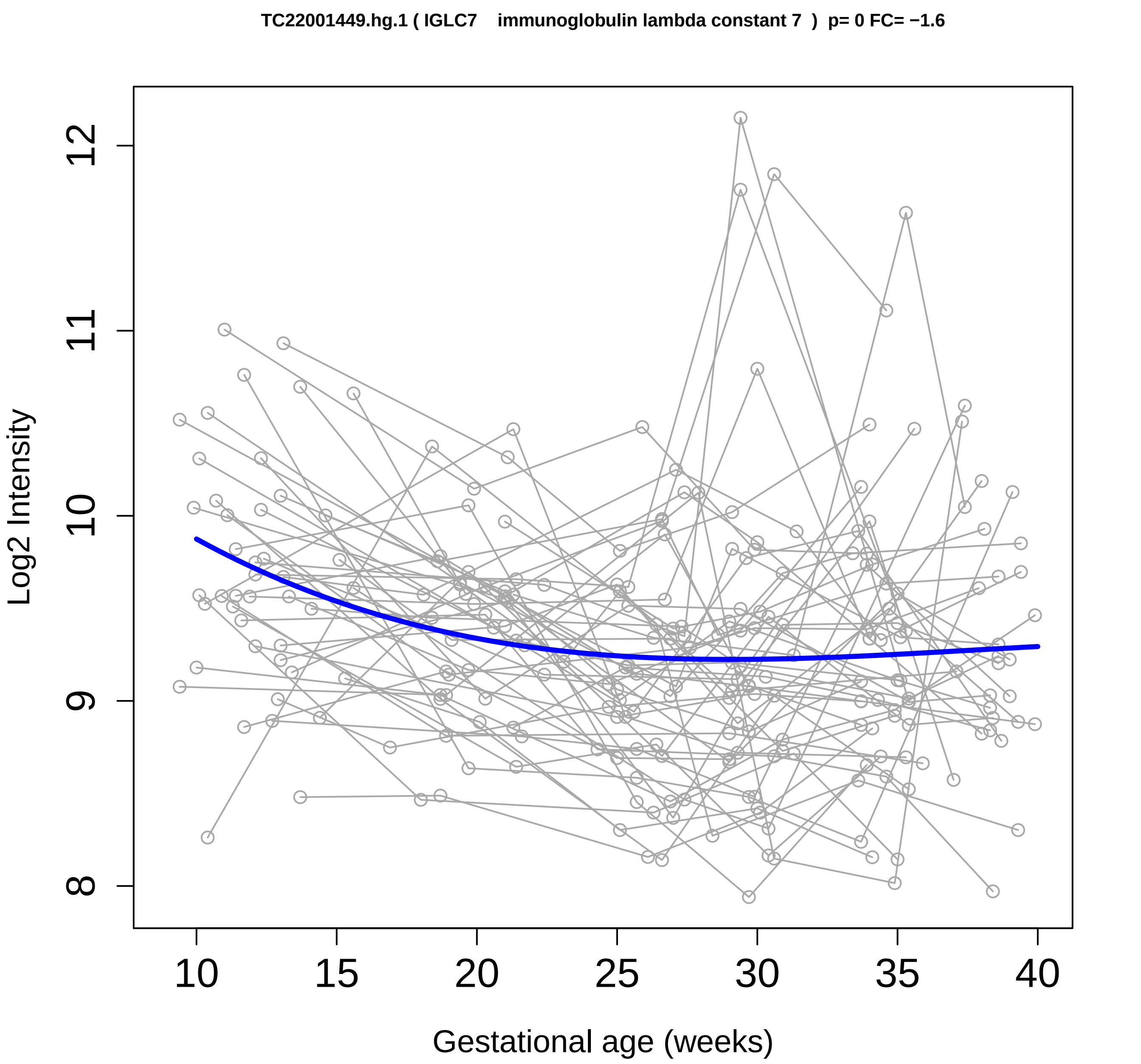

TC22001449.hg.1 (IGLC7 immunoglobulin lambda constant 7) p= 0 FC= −1.6
Log2 Intensity
8
9
10
11
12
10
15
20
25
30
35
40
Gestational age (weeks)

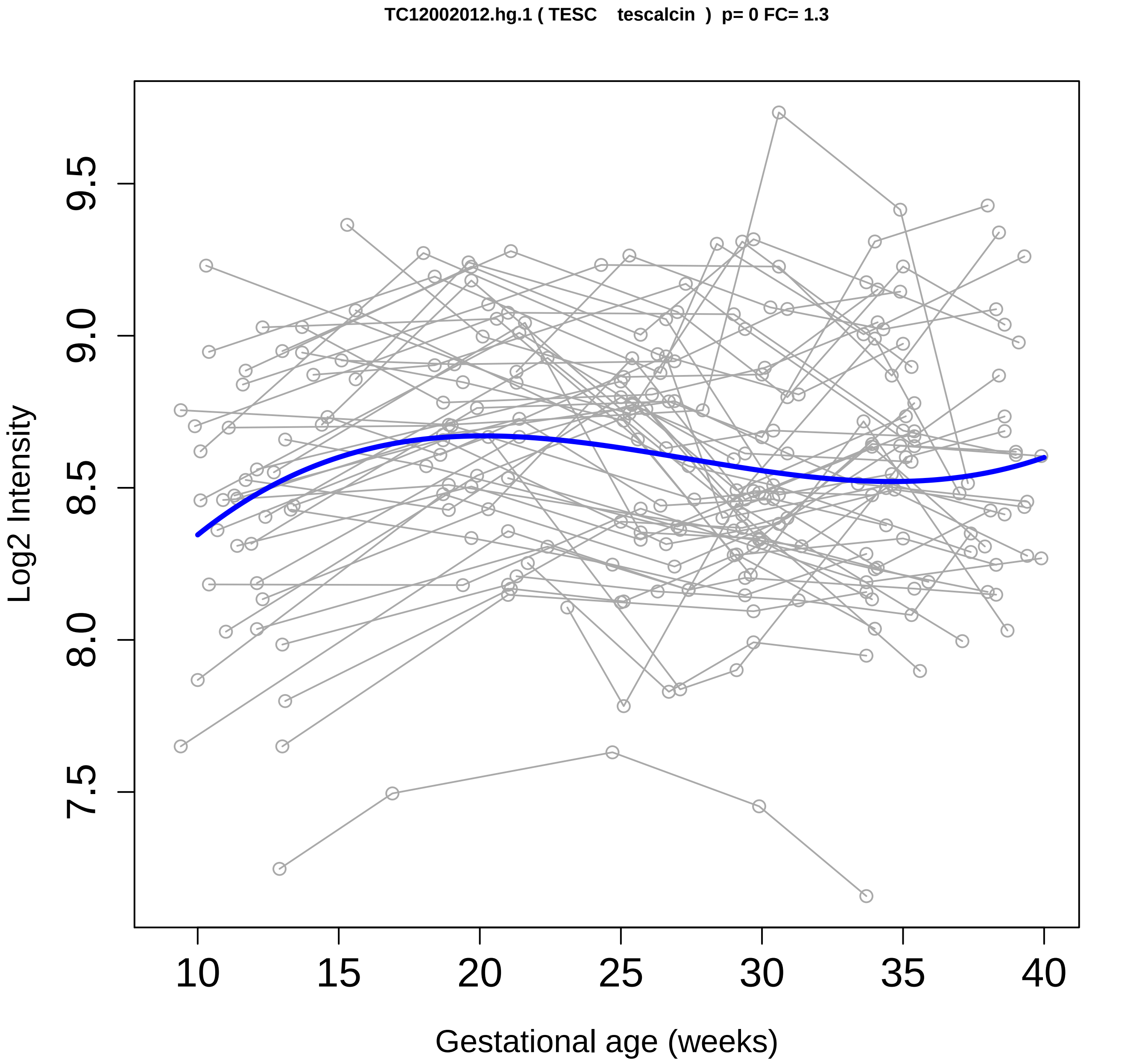

TC12002012.hg.1 (TESC tescalcin) p= 0 FC= 1.3
Log2 Intensity
7.5
8.0
8.5
9.0
9.5
10
15
20
25
30
35
40
Gestational age (weeks)

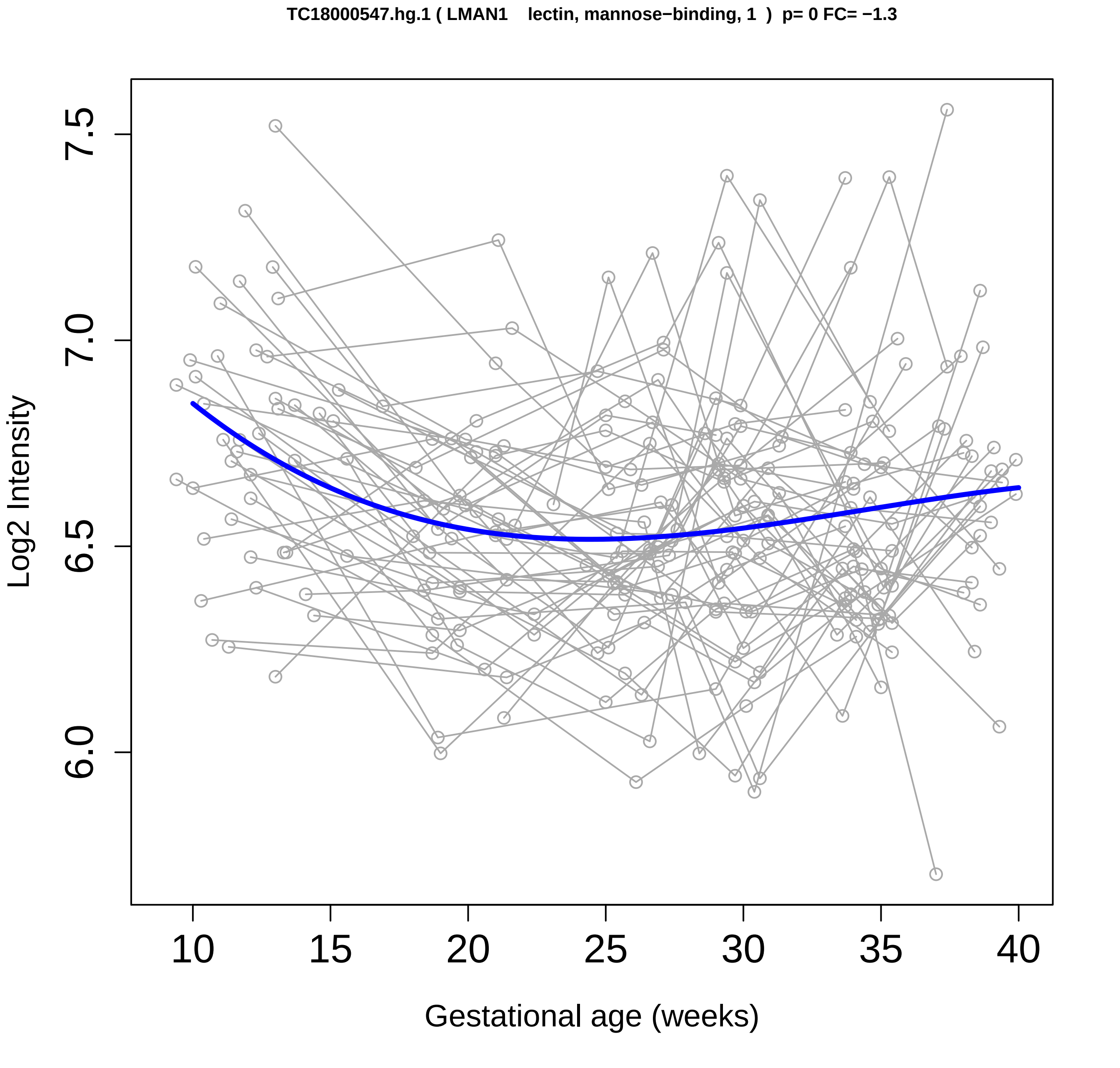

TC18000547.hg.1 (LMAN1 lectin, mannose−binding, 1) p= 0 FC= −1.3
Log2 Intensity
6.0
6.5
7.0
7.5
10
15
20
25
30
35
40
Gestational age (weeks)

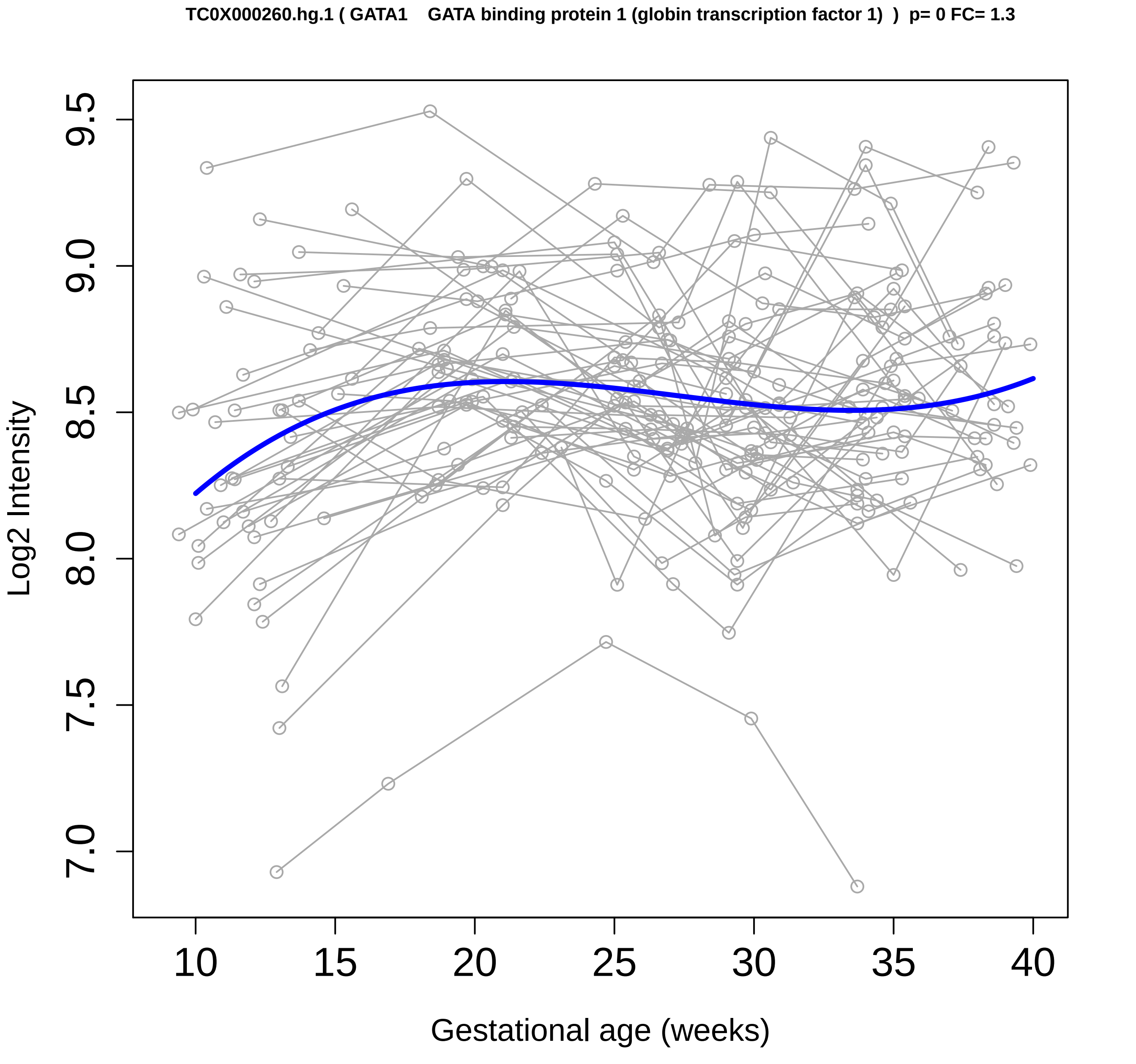
TC0X000260.hg.1 (GATA1 GATA binding protein 1 (globin transcription factor 1)) p= 0 FC= 1.3
Log2 Intensity
9.5
9.0
8.5
8.0
7.5
7.0
10
15
20
25
30
35
40
Gestational age (weeks)

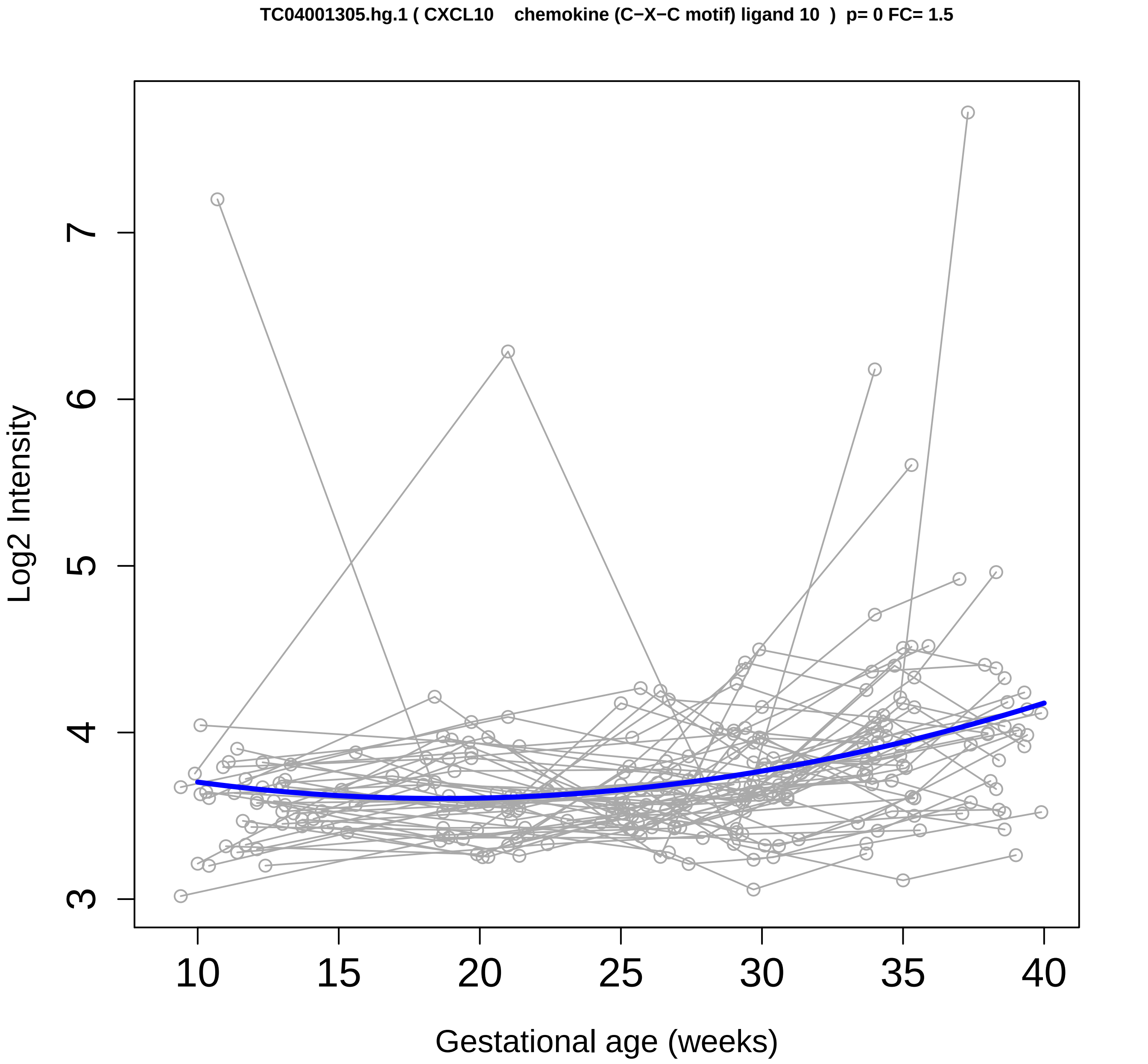

TC04001305.hg.1 (CXCL10 chemokine (C−X−C motif) ligand 10) p= 0 FC= 1.5
Log2 Intensity
3
4
5
6
7
10
15
20
25
30
35
40
Gestational age (weeks)

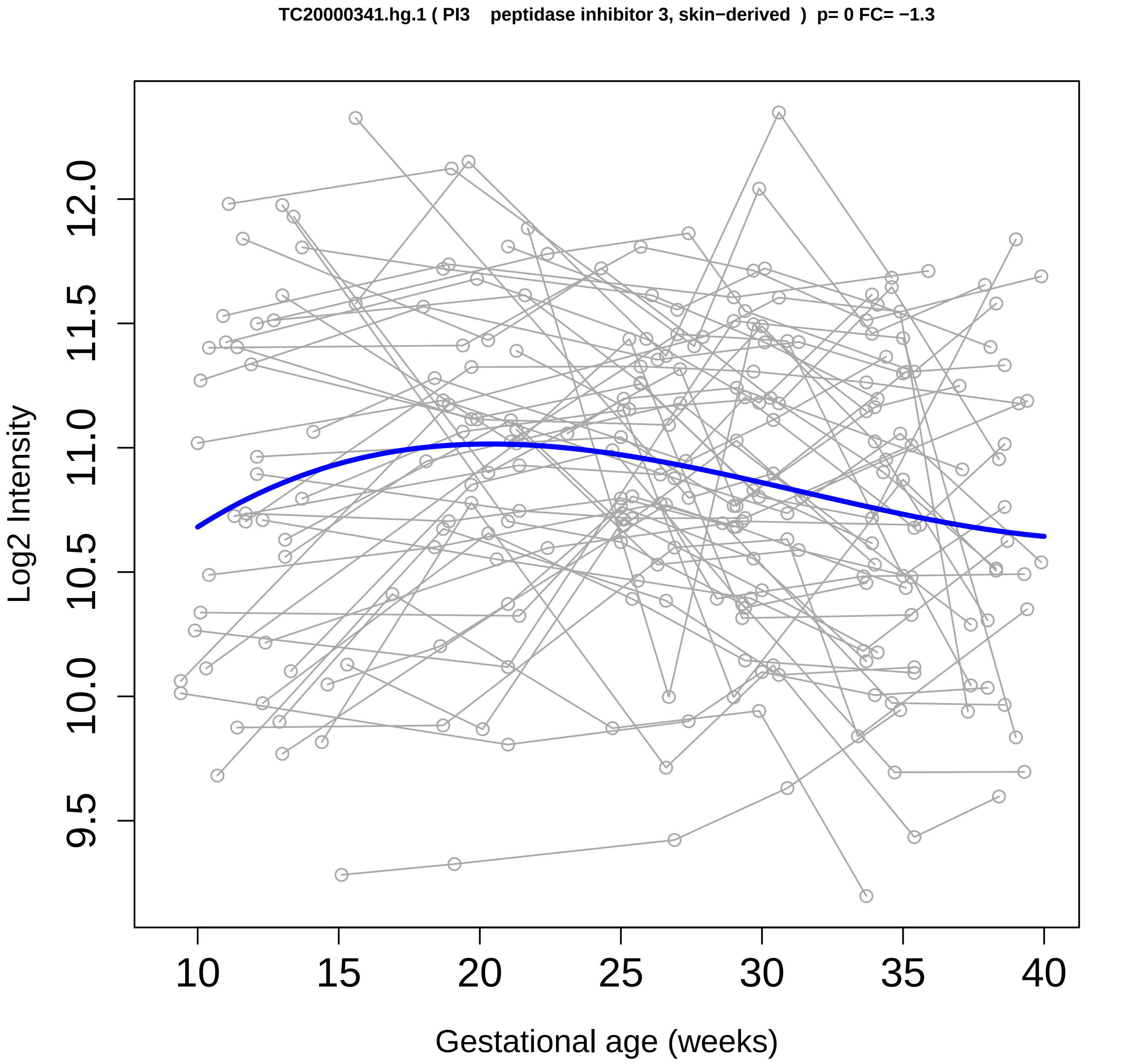
TC20000341.hg.1 (PI3 peptidase inhibitor 3, skin−derived) p= 0 FC= −1.3
Log2 Intensity
9.5
10.0
10.5
11.0
11.5
12.0
10
15
20
25
30
35
40
Gestational age (weeks)

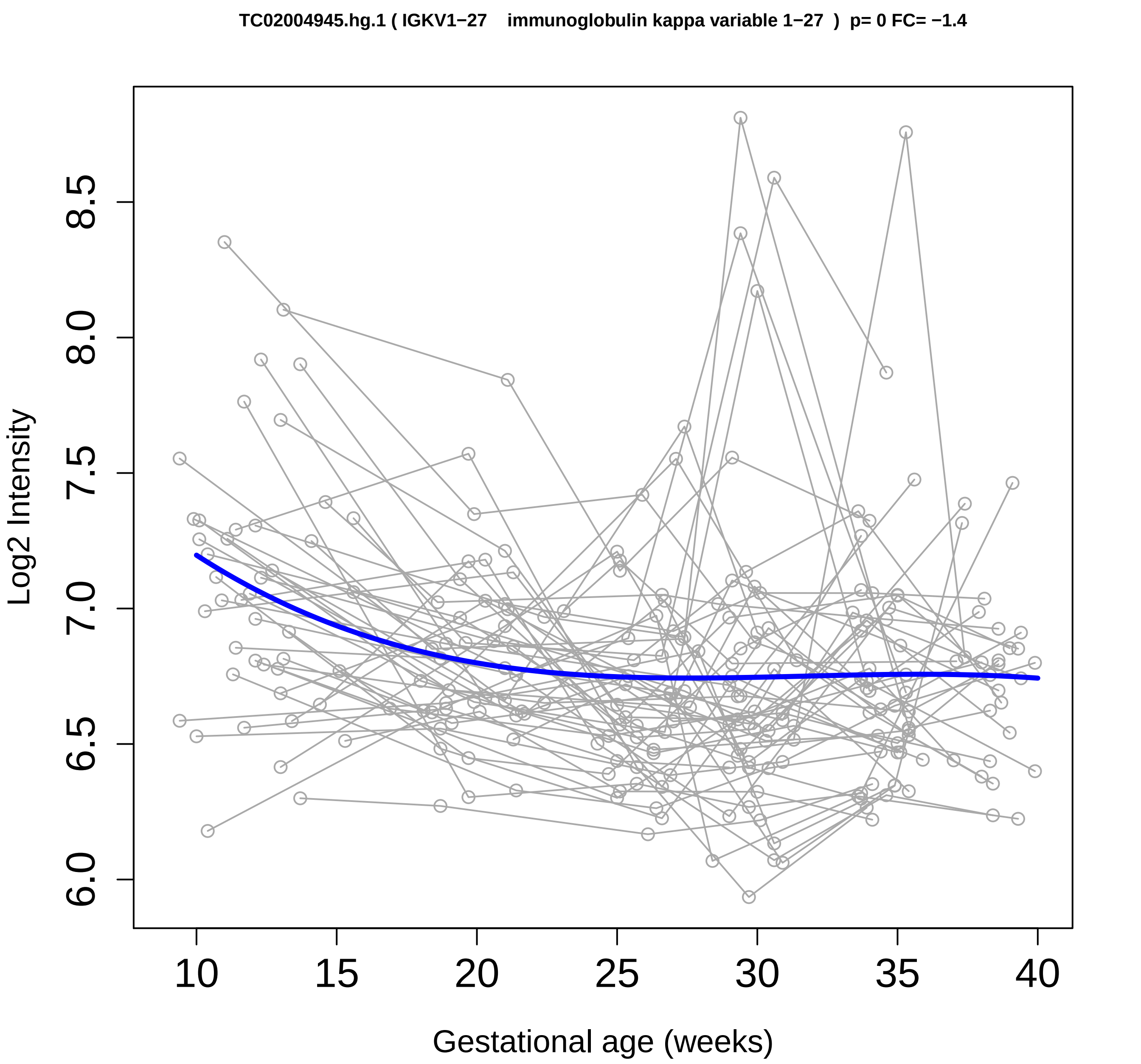

TC02004945.hg.1 (IGKV1−27 immunoglobulin kappa variable 1−27) p= 0 FC= −1.4
Log2 Intensity
6.0
6.5
7.0
7.5
8.0
8.5
10
15
20
25
30
35
40
Gestational age (weeks)

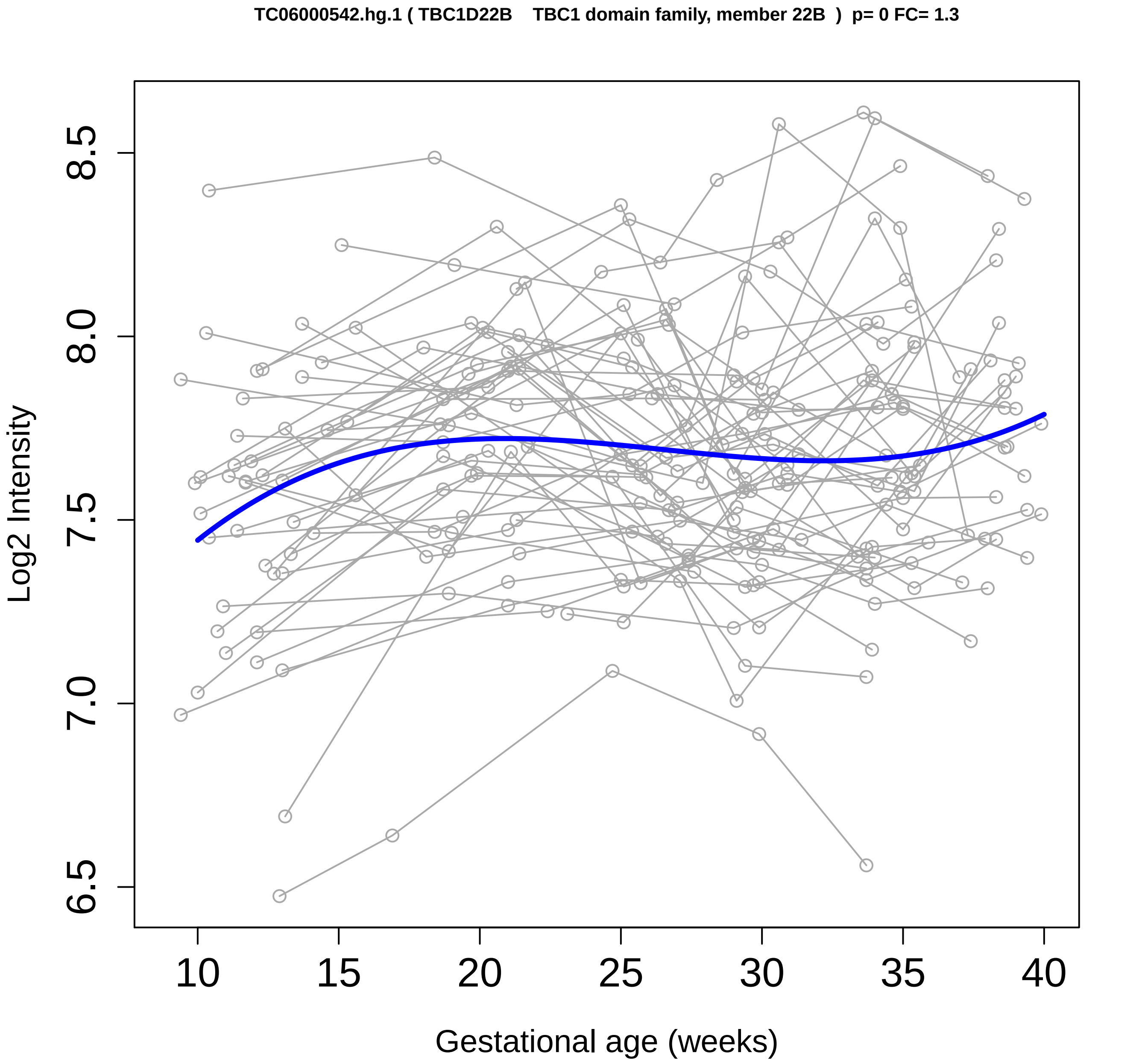

TC06000542.hg.1 (TBC1D22B TBC1 domain family, member 22B) p= 0 FC= 1.3
Log2 Intensity
6.5
7.0
7.5
8.0
8.5
10
15
20
25
30
35
40
Gestational age (weeks)

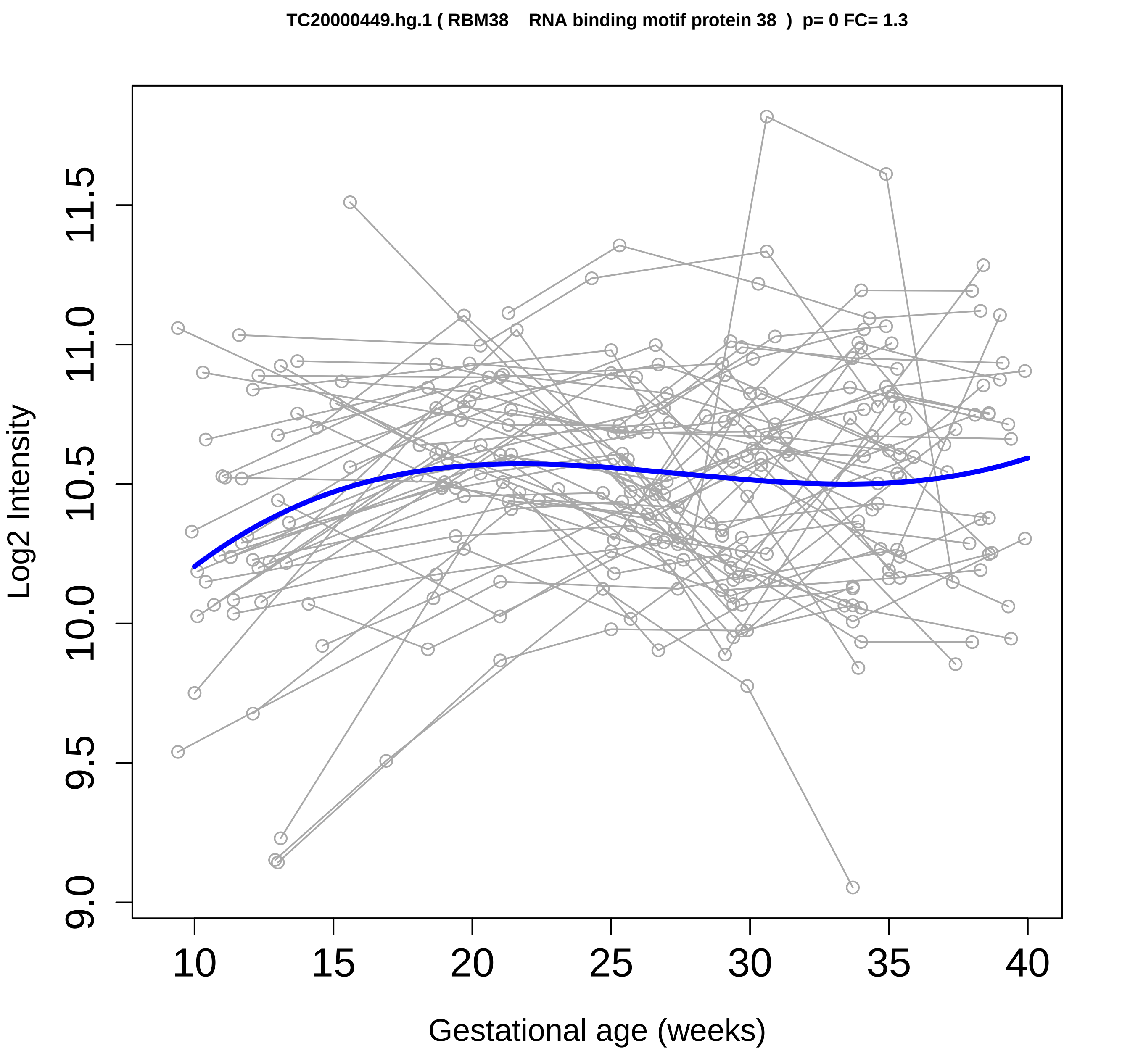
TC20000449.hg.1 (RBM38   RNA binding motif protein 38) p= 0 FC= 1.3
Log2 Intensity
9.0
9.5
10.0
10.5
11.0
11.5
10
15
20
25
30
35
40
Gestational age (weeks)

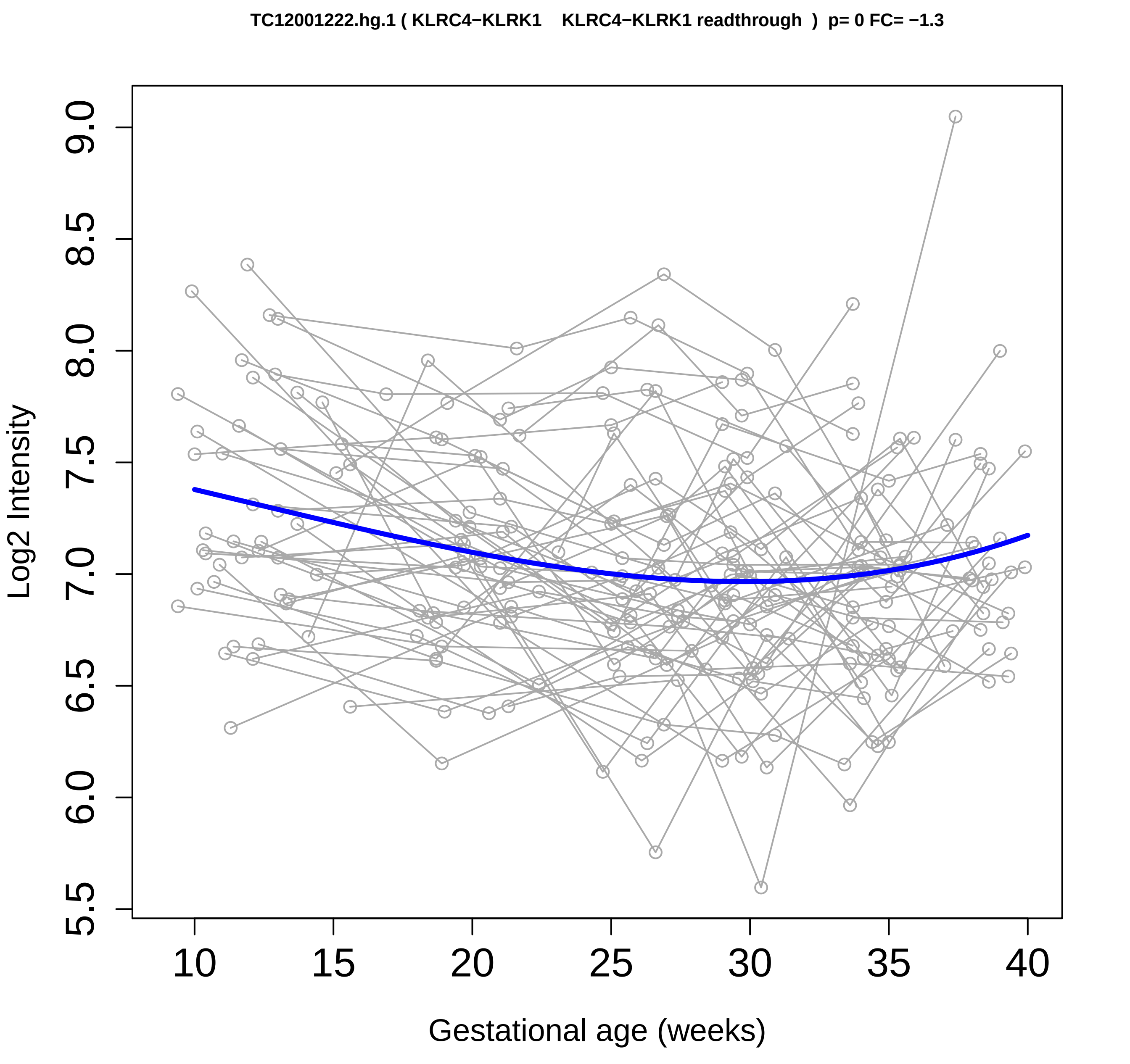

TC12001222.hg.1 (KLRC4−KLRK1 KLRC4−KLRK1 readthrough) p= 0 FC= −1.3
Log2 Intensity
9.0
8.5
8.0
7.5
7.0
6.5
6.0
5.5
10
15
20
25
30
35
40
Gestational age (weeks)

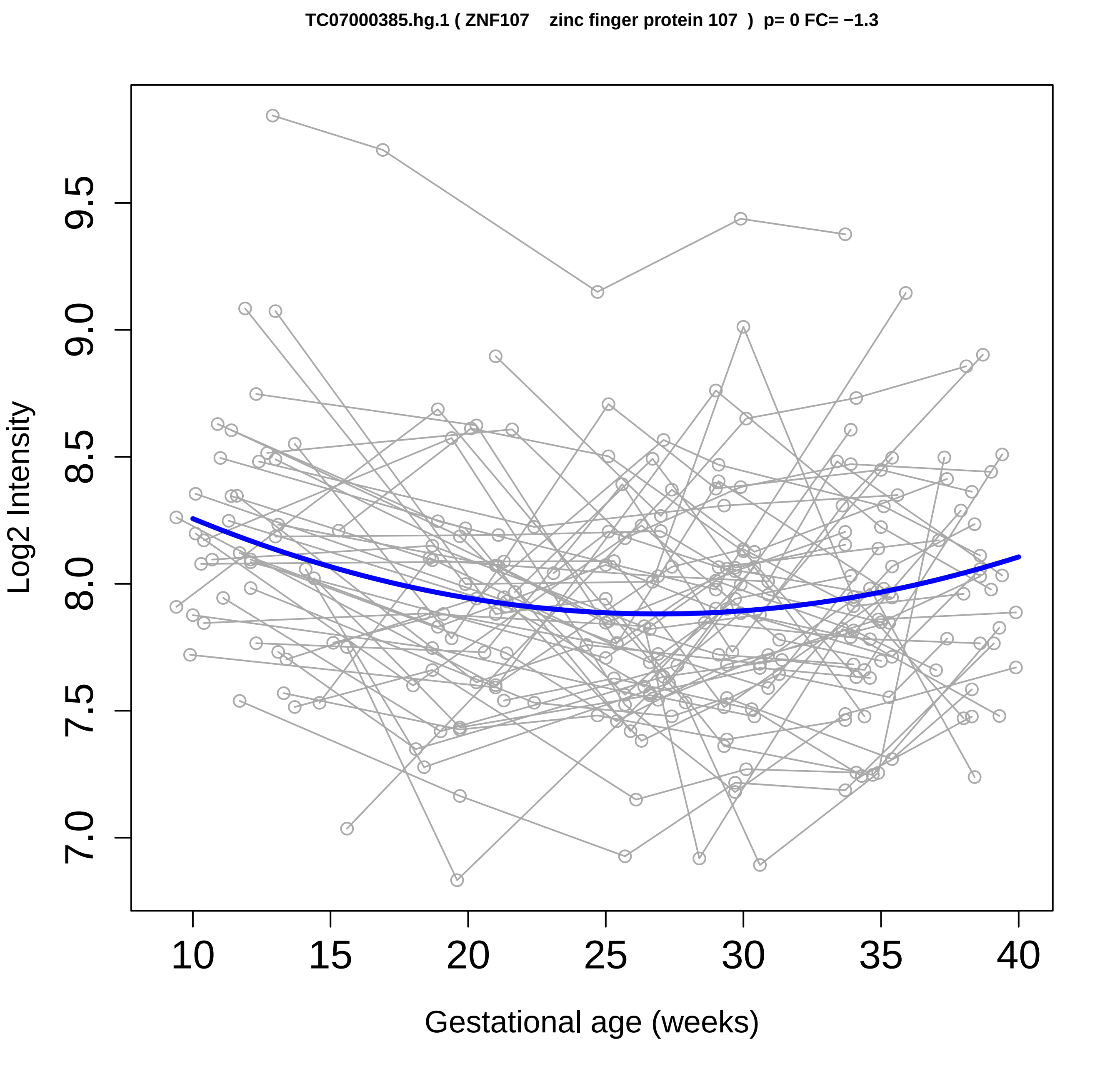

TC07000385.hg.1 (ZNF107 zinc finger protein 107) p= 0 FC= −1.3
Log2 Intensity
7.0
7.5
8.0
8.5
9.0
9.5
10
15
20
25
30
35
40
Gestational age (weeks)

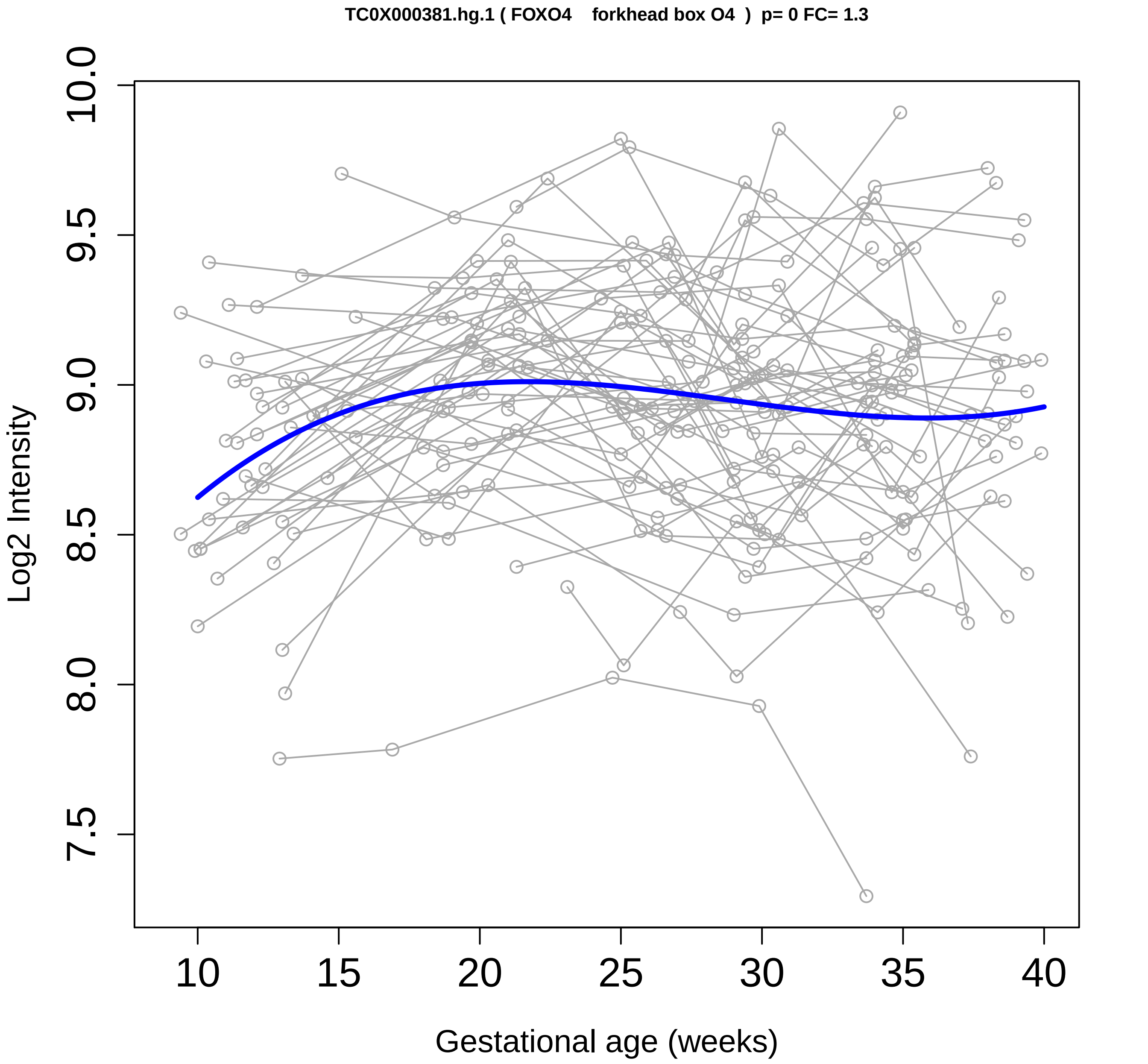

TC0X000381.hg.1 (FOXO4 forkhead box O4) p= 0 FC= 1.3
Log2 Intensity
10.0
9.5
9.0
8.5
8.0
7.5
10
15
20
25
30
35
40
Gestational age (weeks)

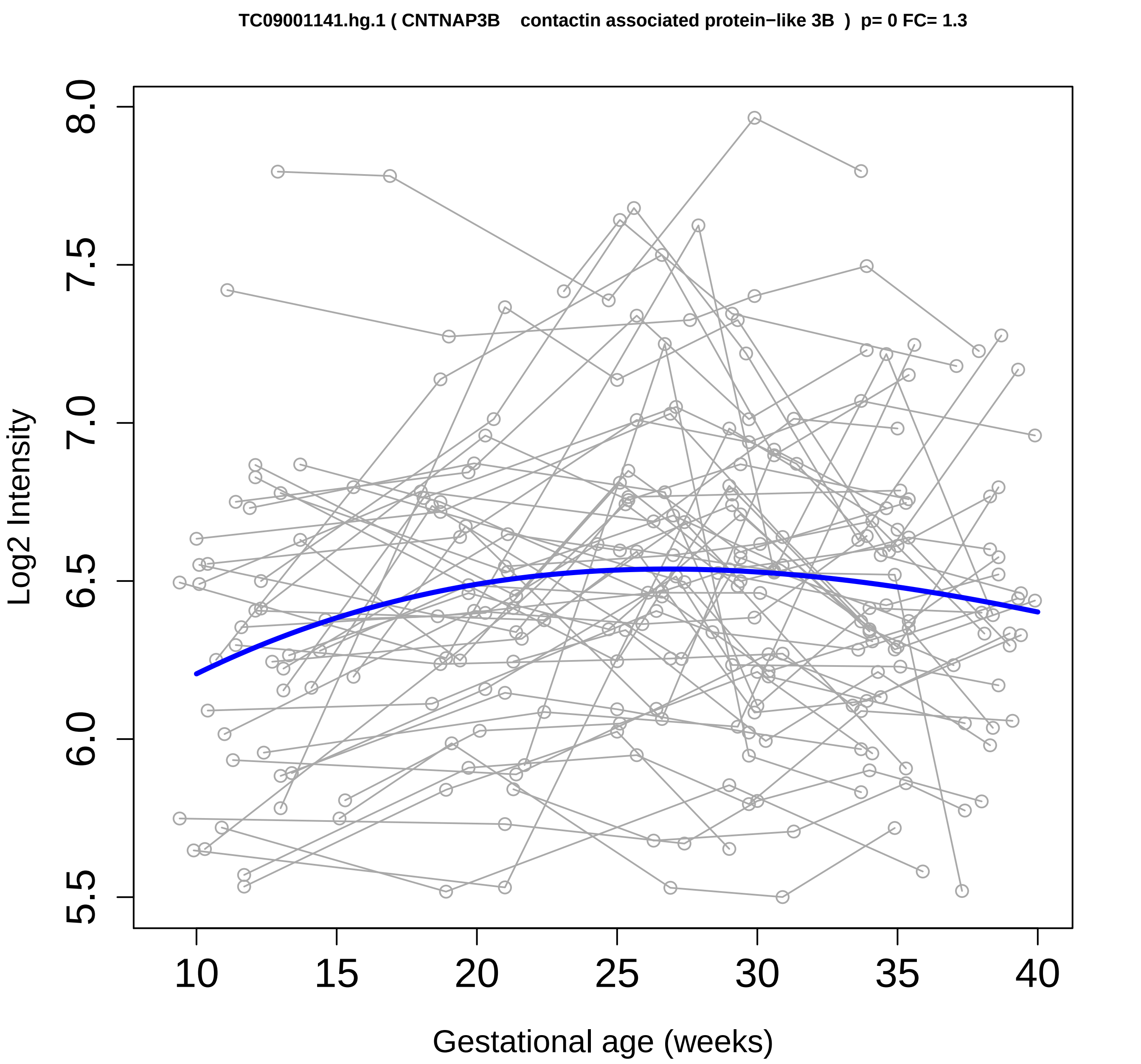

TC09001141.hg.1 (CNTNAP3B contactin associated protein−like 3B) p= 0 FC= 1.3
Log2 Intensity
5.5
6.0
6.5
7.0
7.5
8.0
10
15
20
25
30
35
40
Gestational age (weeks)

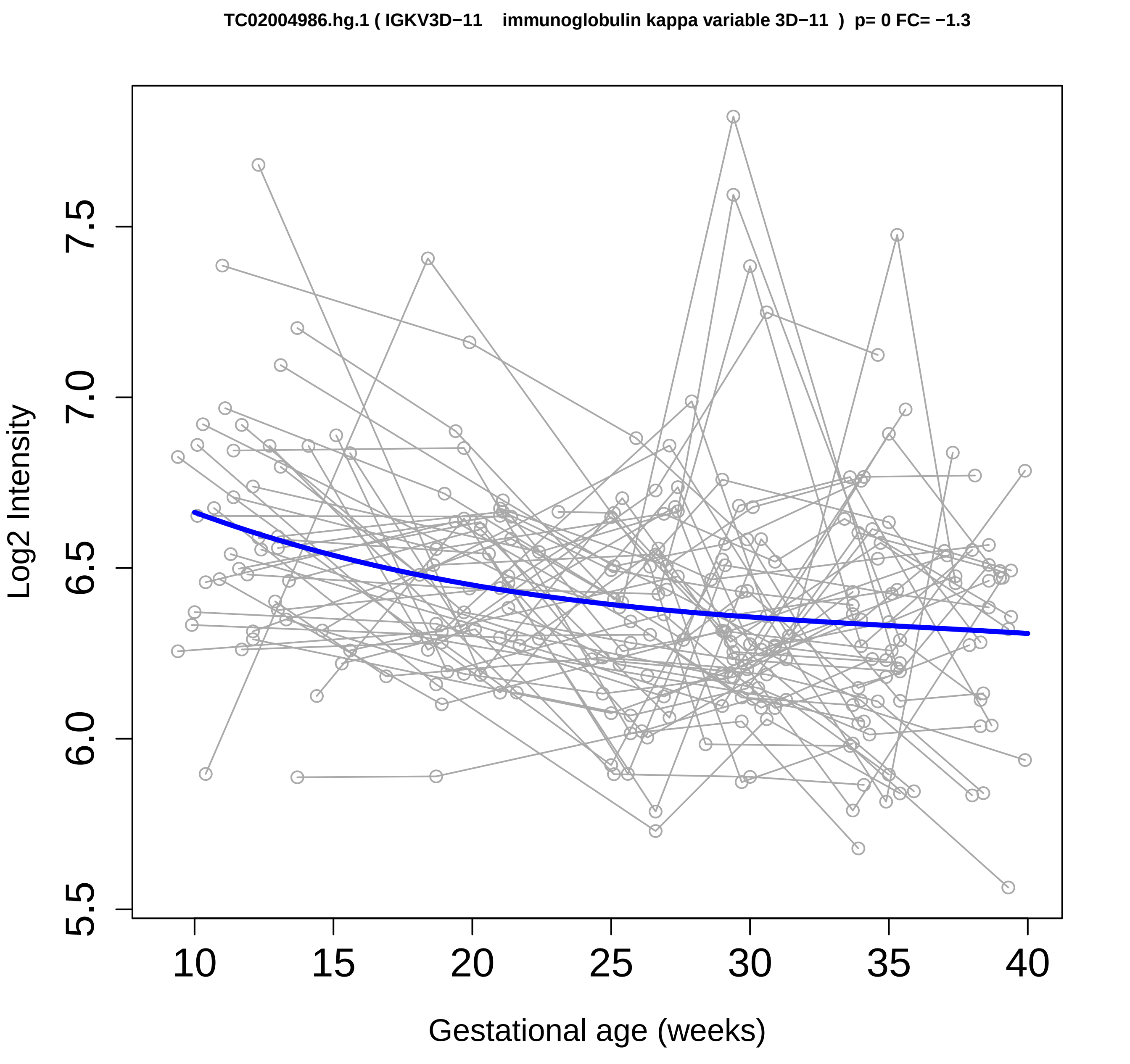

TC02004986.hg.1 (IGKV3D−11   immunoglobulin kappa variable 3D−11) p= 0 FC= −1.3
Log2 Intensity
5.5
6.0
6.5
7.0
7.5
10
15
20
25
30
35
40
Gestational age (weeks)

TC14002261.hg.1 (IGHV3−30   immunoglobulin heavy variable 3−30) p= 0 FC= −1.5

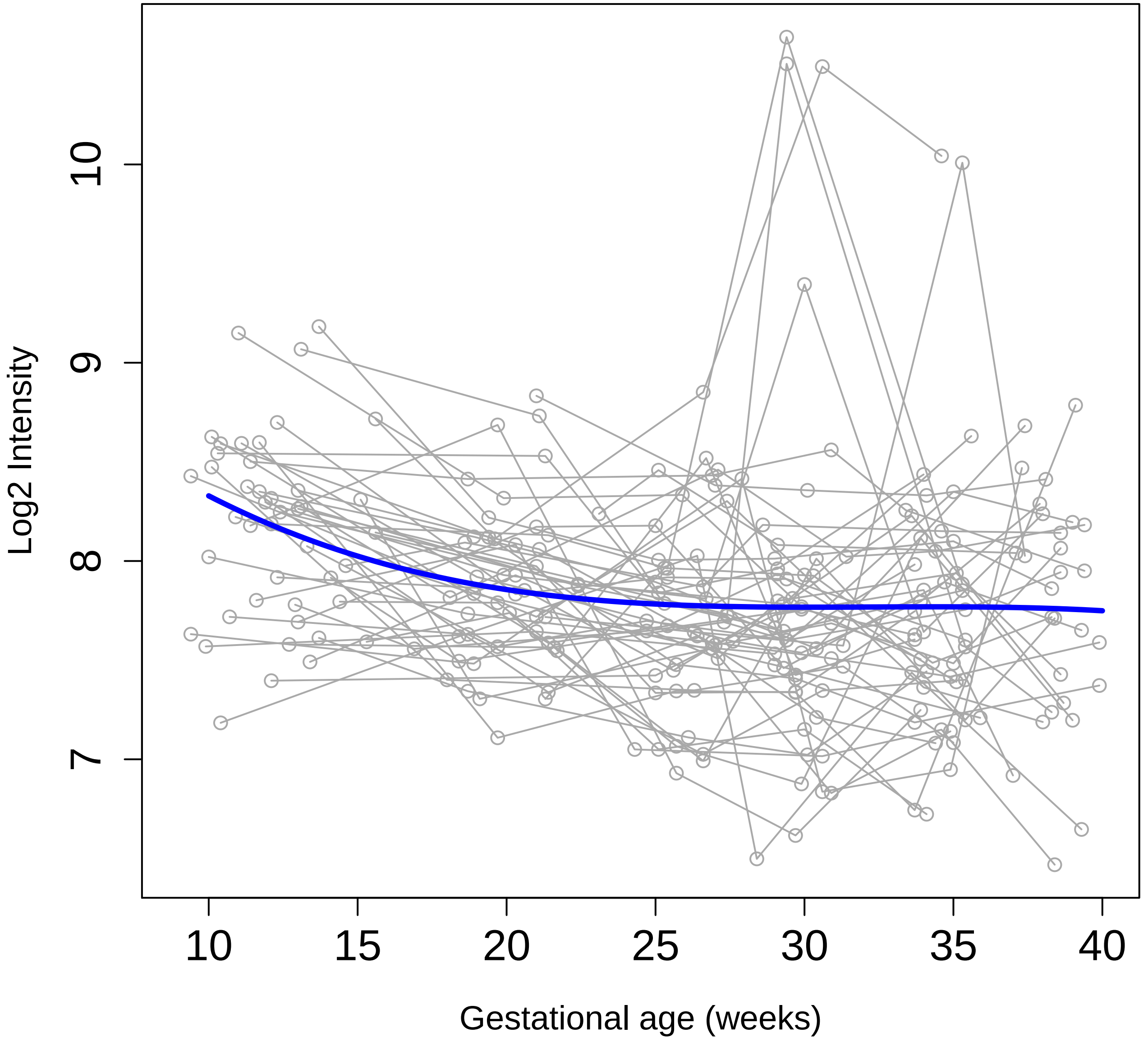

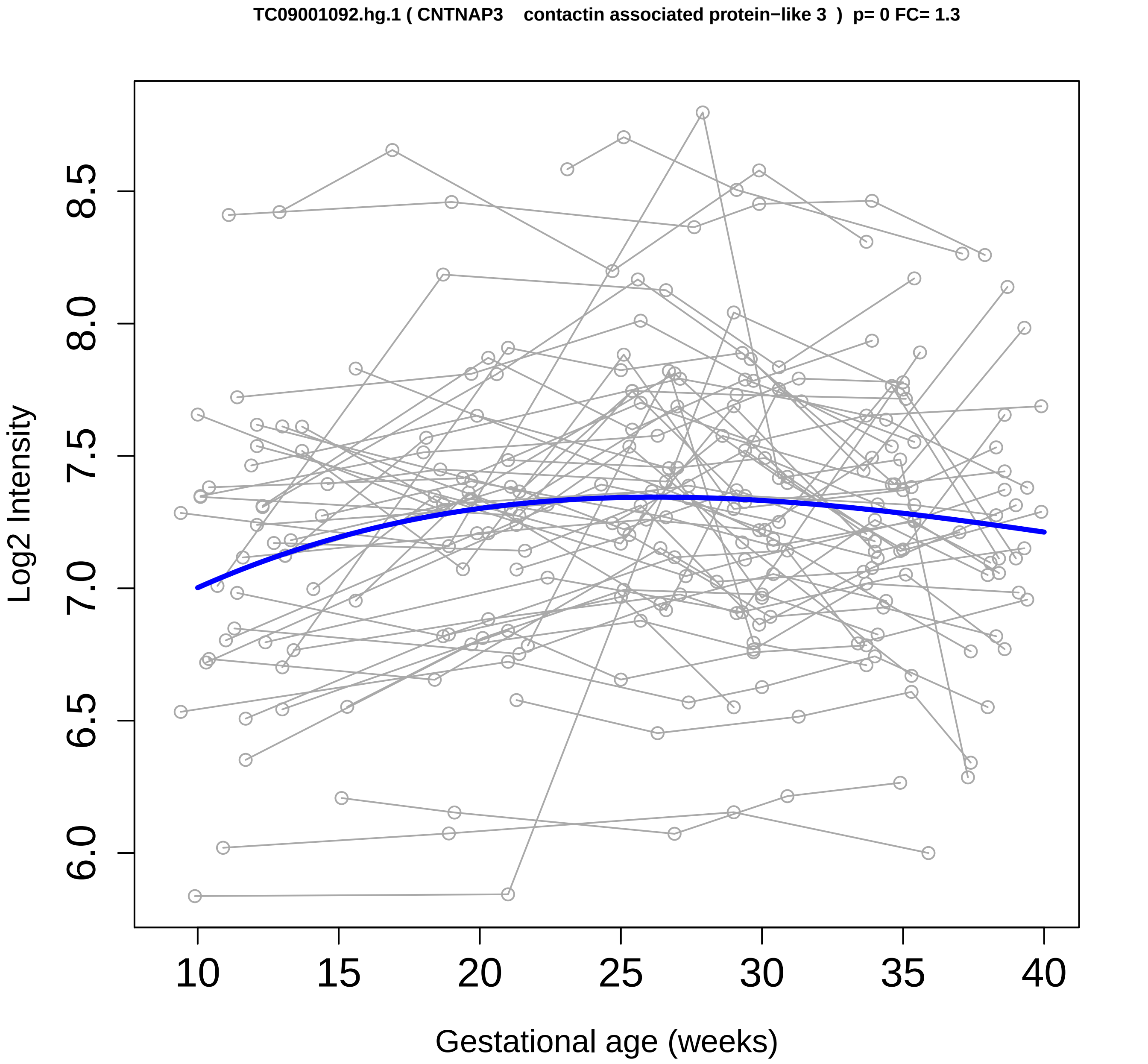
TC09001092.hg.1 (CNTNAP3   contactin associated protein−like 3) p= 0 FC= 1.3
Log2 Intensity
6.0
6.5
7.0
7.5
8.0
8.5
10
15
20
25
30
35
40
Gestational age (weeks)

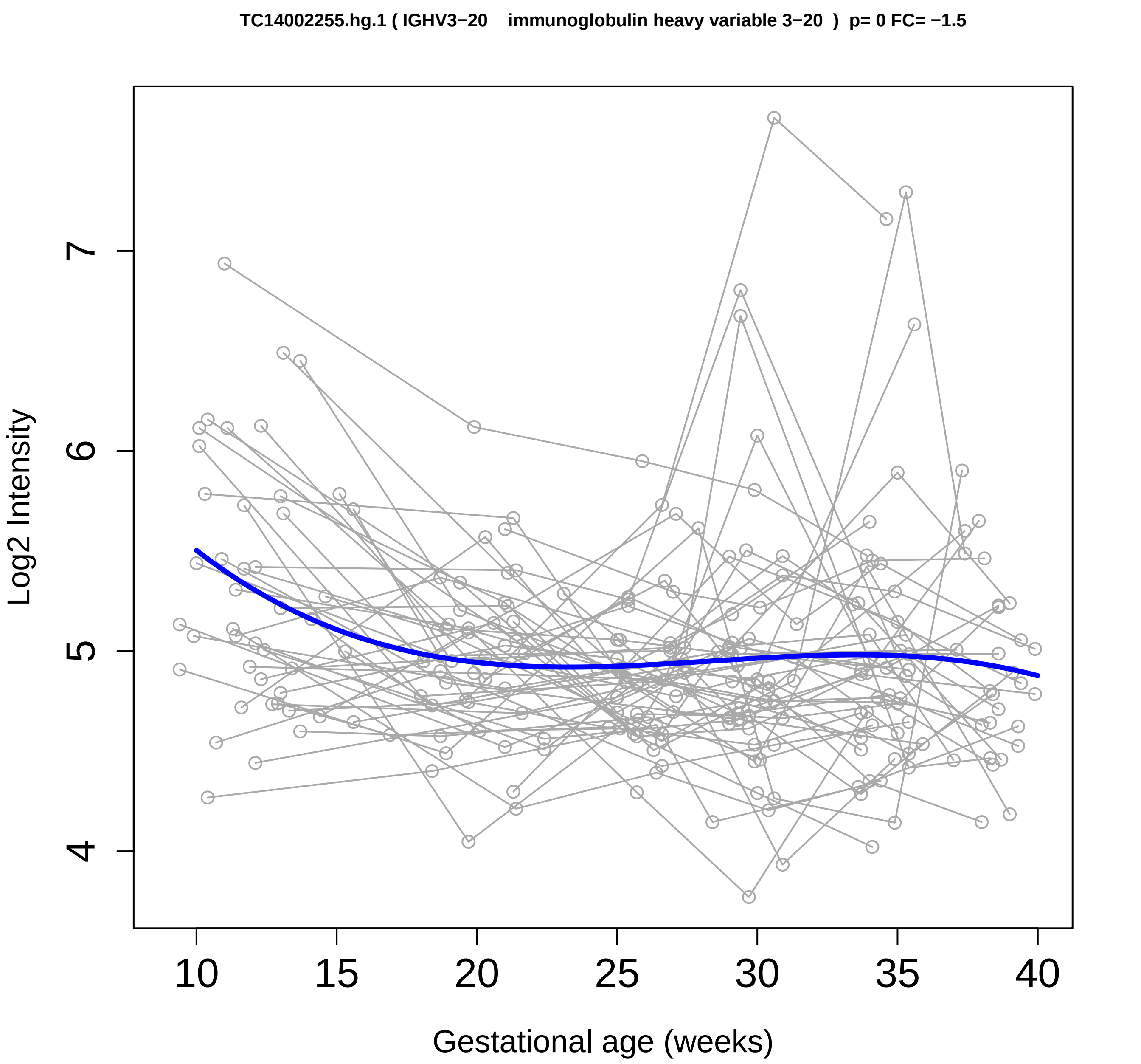
TC14002255.hg.1 (IGHV3−20 immunoglobulin heavy variable 3−20) p= 0 FC= −1.5
Log2 Intensity
4
5
6
7
10
15
20
25
30
35
40
Gestational age (weeks)

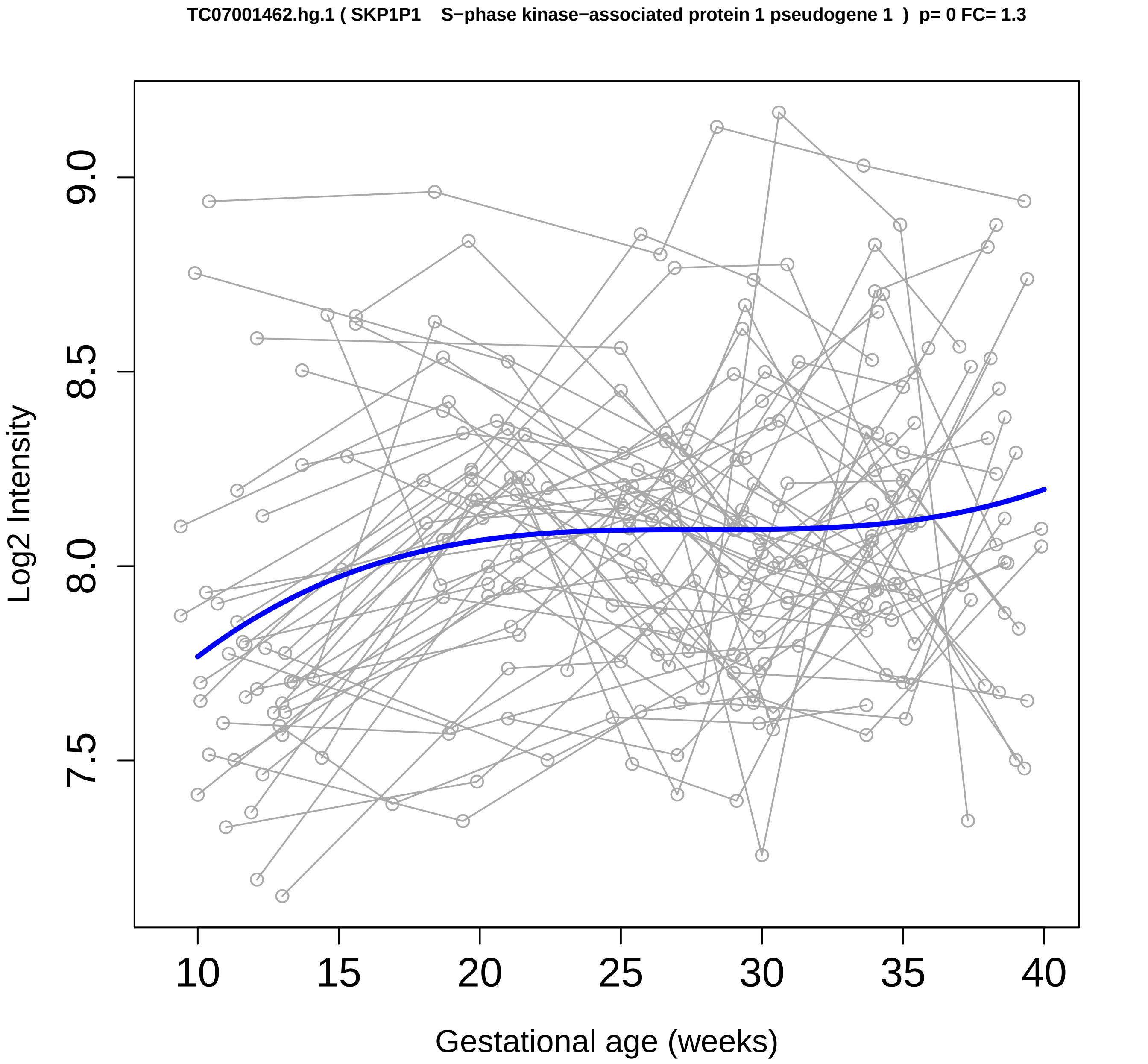
TC07001462.hg.1 (SKP1P1   S−phase kinase−associated protein 1 pseudogene 1) p= 0 FC= 1.3
Log2 Intensity
9.0
8.5
8.0
7.5
10
15
20
25
30
35
40
Gestational age (weeks)

TC12003272.hg.1 (KLRC2 killer cell lectin−like receptor subfamily C, member 2) p= 0 FC= −1.3

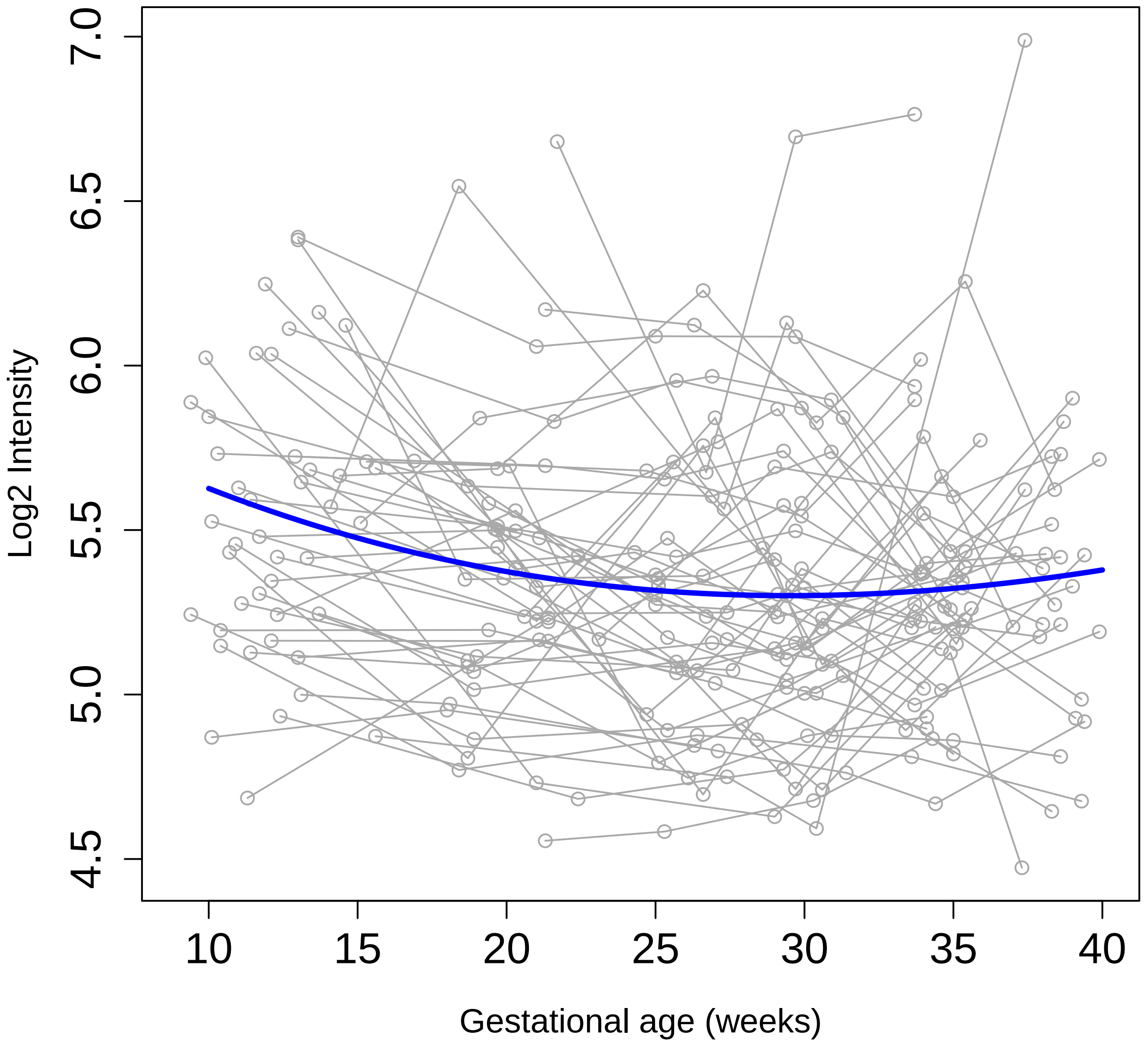

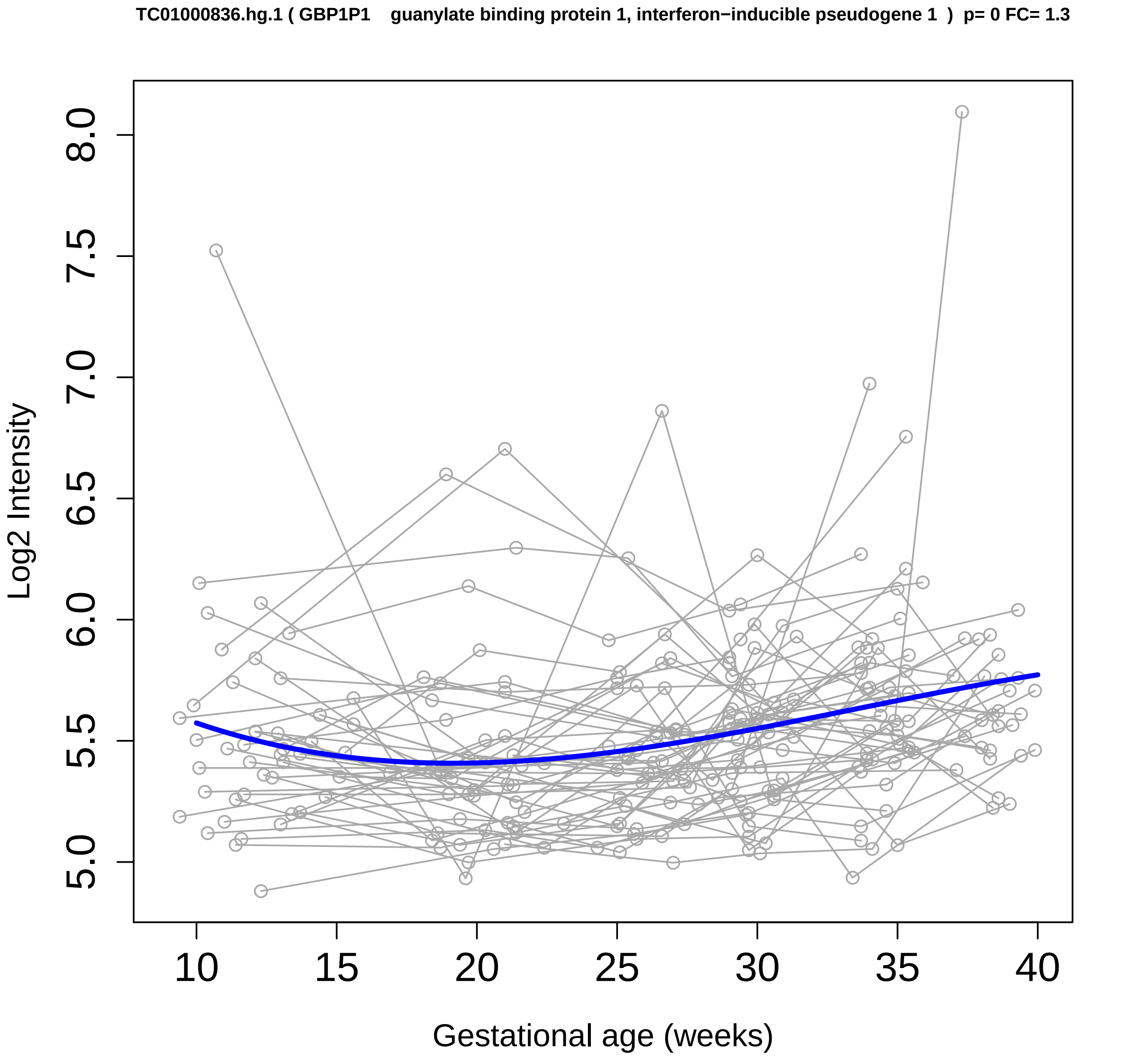
TC01000836.hg.1 (GBP1P1   guanylate binding protein 1, interferon−inducible pseudogene 1) p= 0 FC= 1.3
Log2 Intensity
5.0
5.5
6.0
6.5
7.0
7.5
8.0
10
15
20
25
30
35
40
Gestational age (weeks)

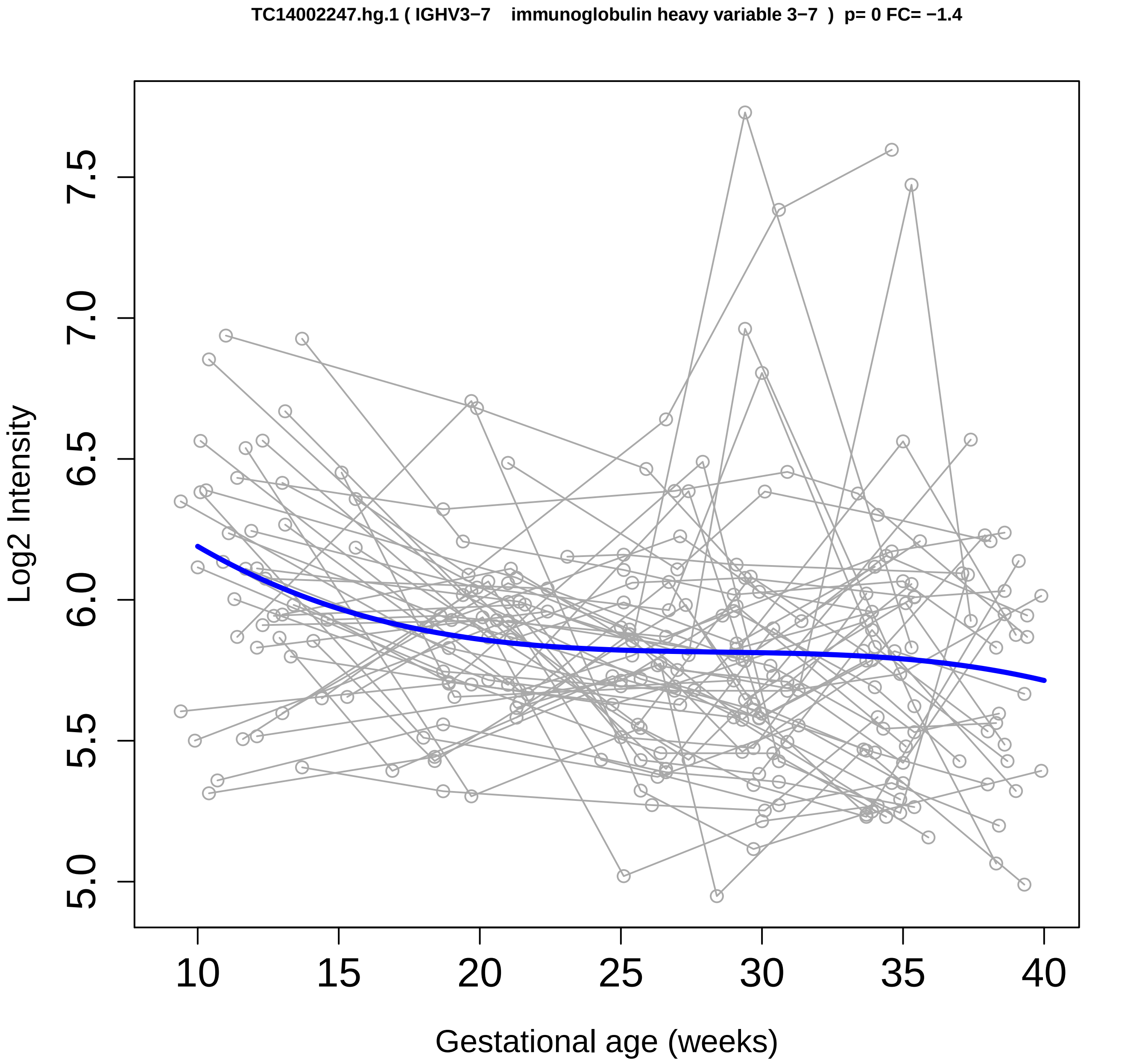

TC14002247.hg.1 (IGHV3−7   immunoglobulin heavy variable 3−7) p= 0 FC= −1.4
Log2 Intensity
5.0
5.5
6.0
6.5
7.0
7.5
10
15
20
25
30
35
40
Gestational age (weeks)

TC01002845.hg.1 (GBP3 guanylate binding protein 3) p= 0 FC= 1.3

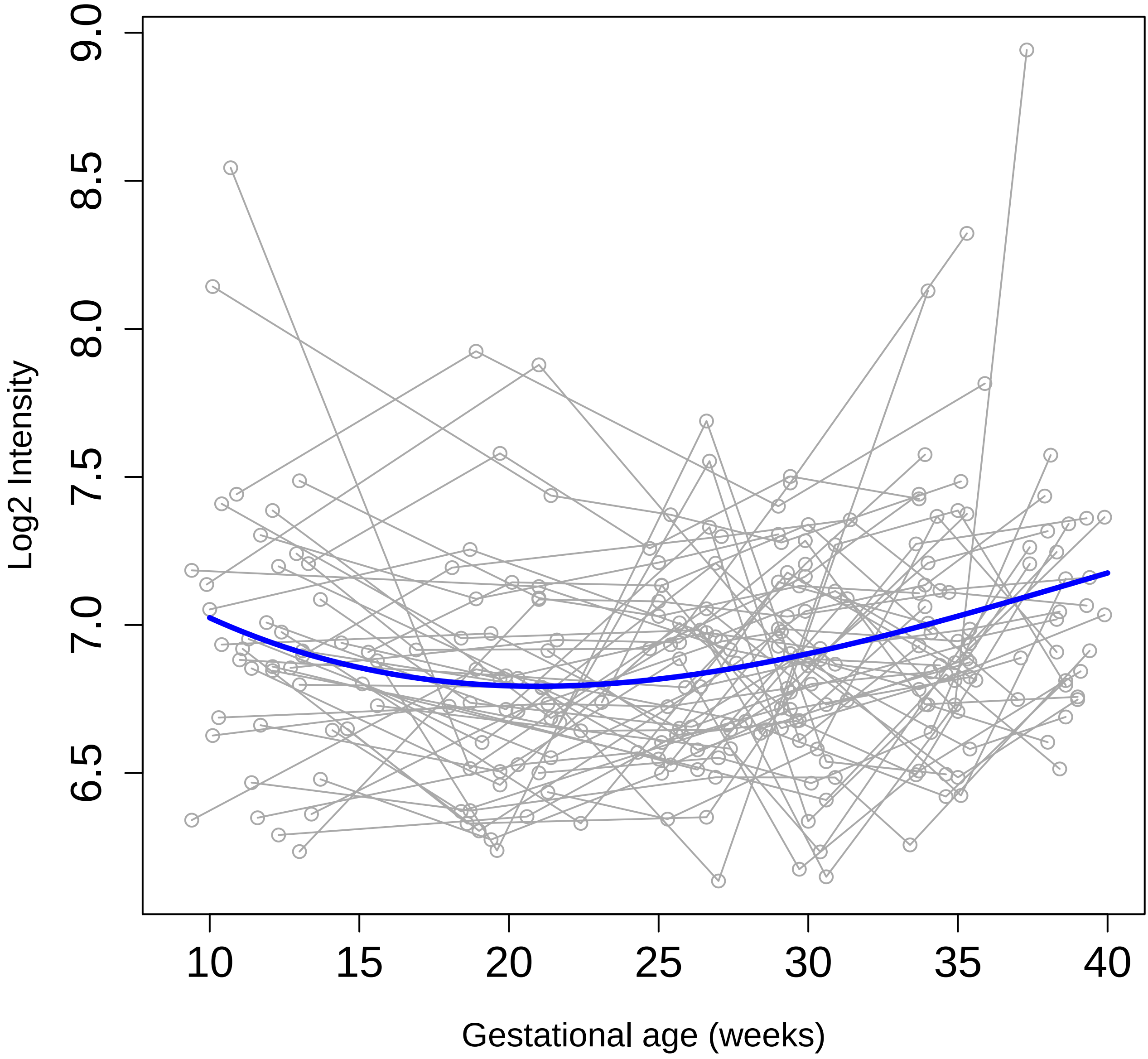

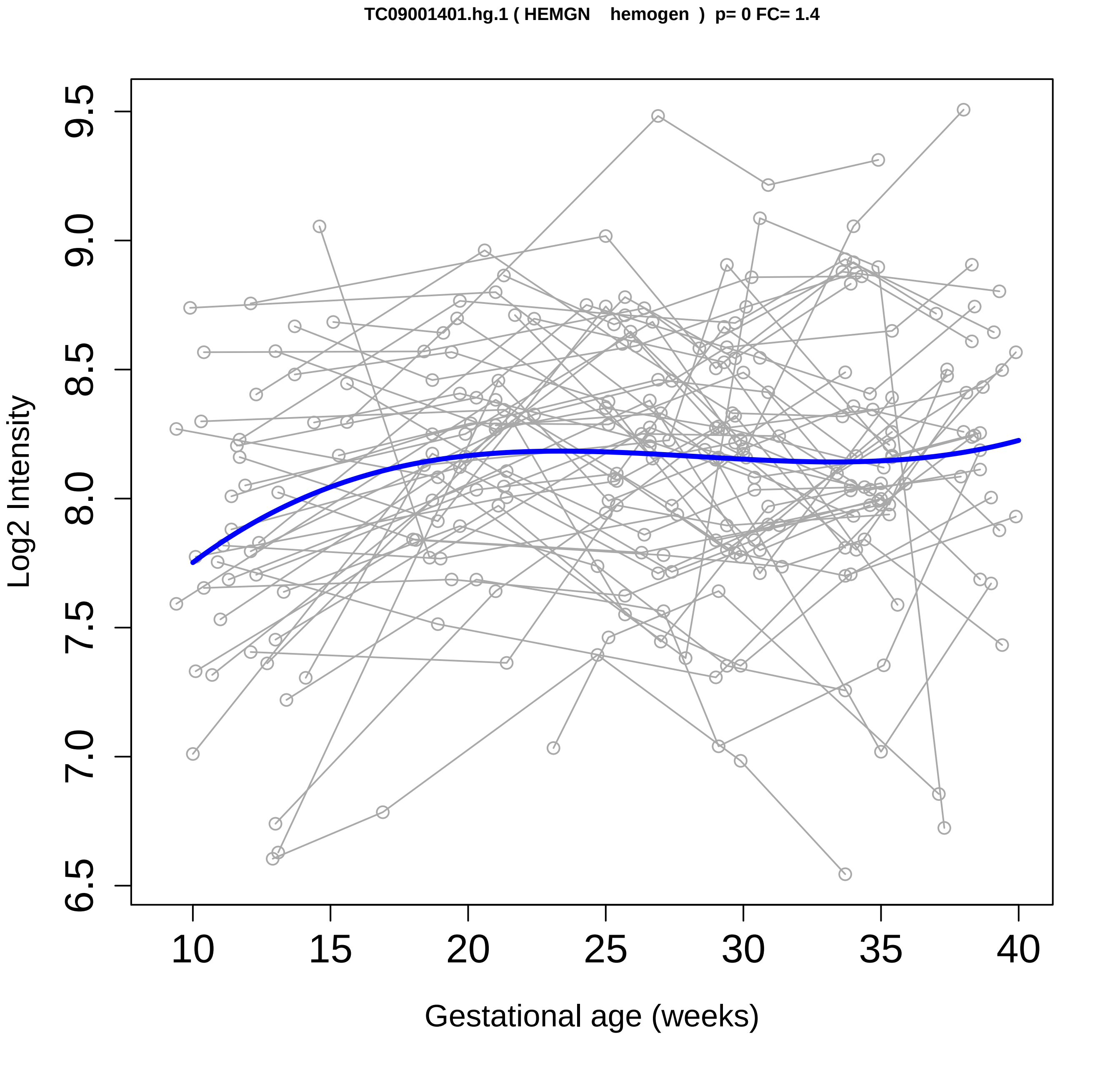

TC09001401.hg.1 (HEMGN hemogen) p= 0 FC= 1.4
Log2 Intensity
9.5
9.0
8.5
8.0
7.5
7.0
6.5
10
15
20
25
30
35
40
Gestational age (weeks)

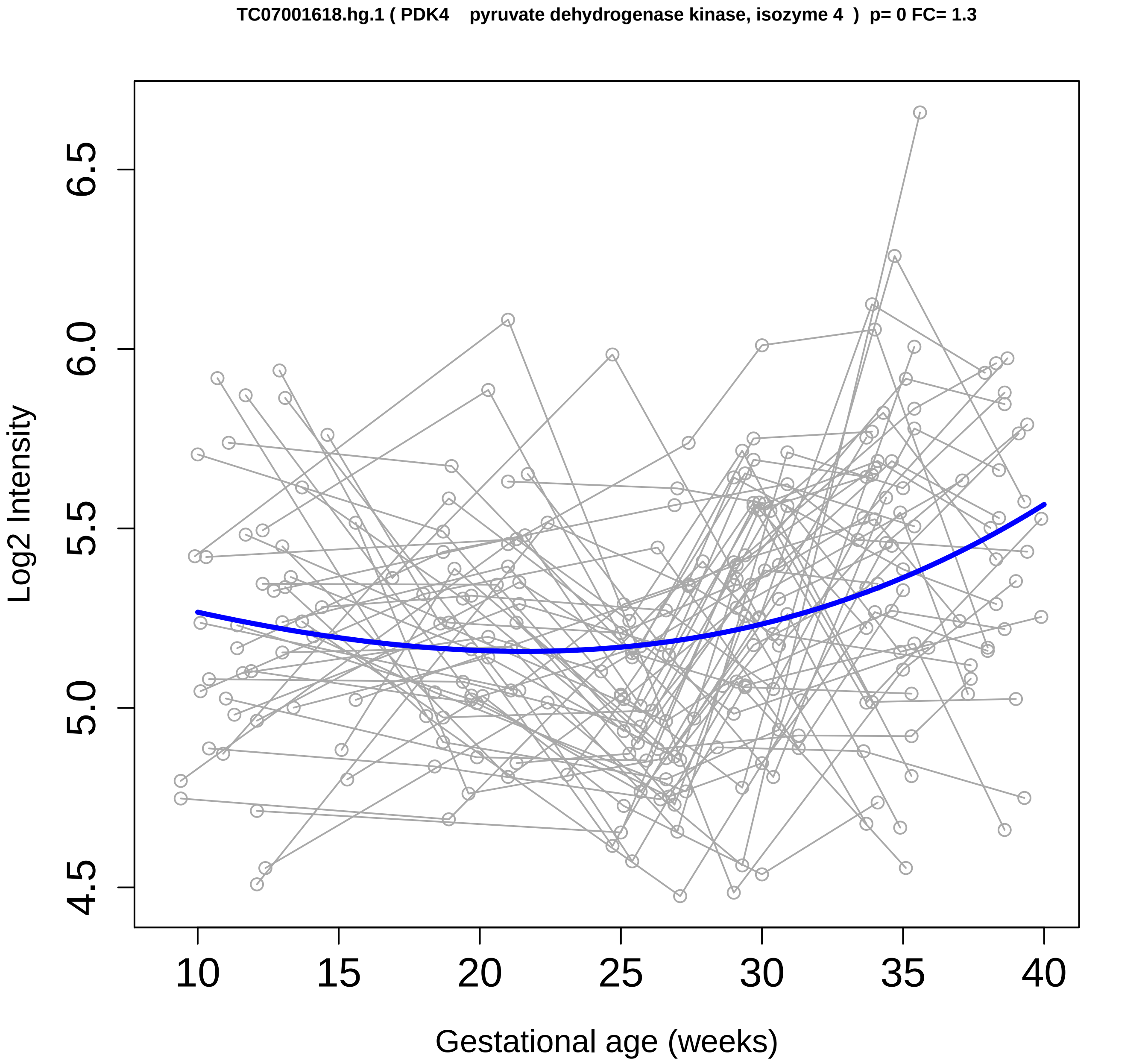
TC07001618.hg.1 (PDK4 pyruvate dehydrogenase kinase, isozyme 4) p= 0 FC= 1.3
Log2 Intensity
4.5
5.0
5.5
6.0
6.5
10
15
20
25
30
35
40
Gestational age (weeks)

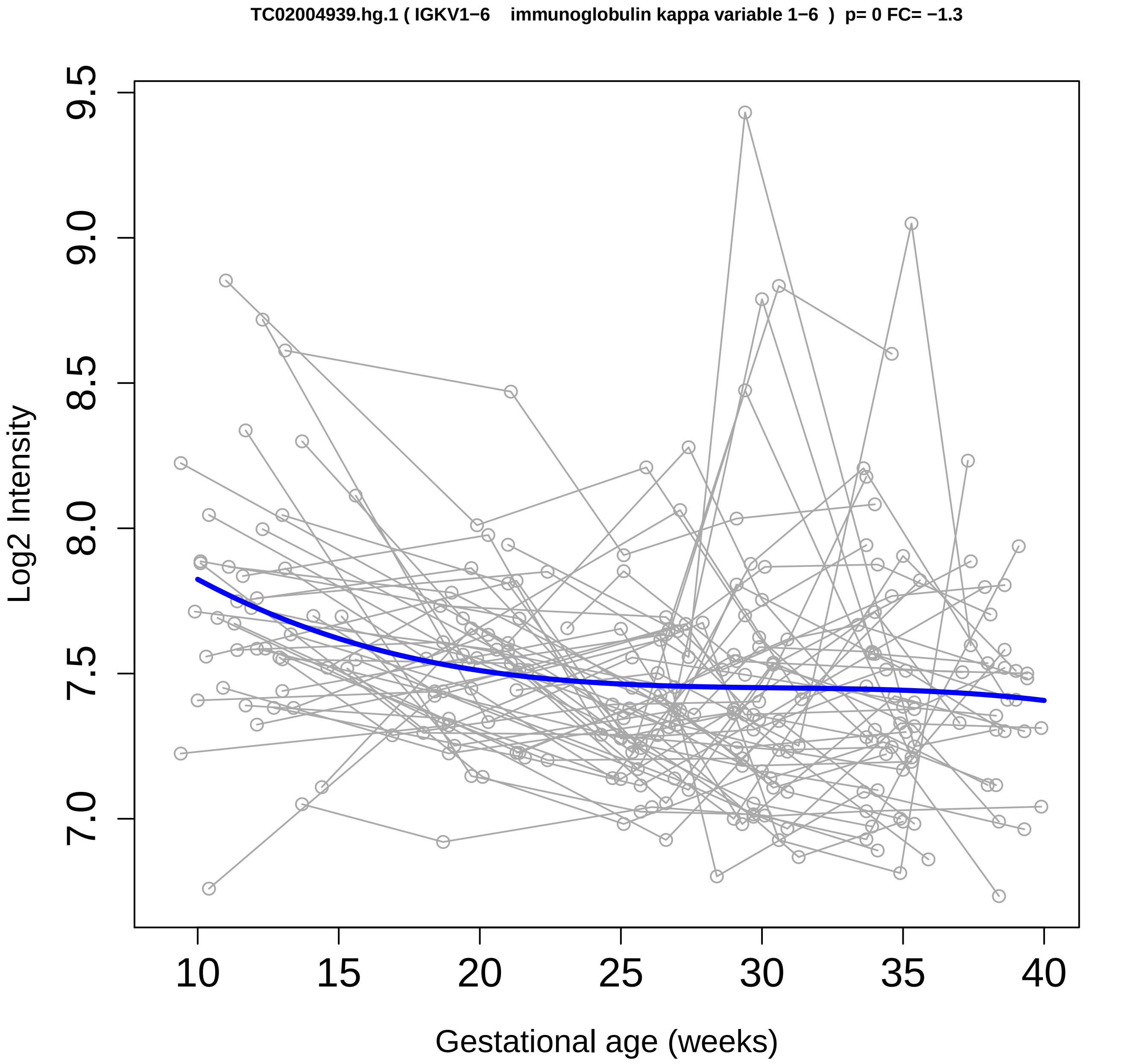
TC02004939.hg.1 (IGKV1−6 immunoglobulin kappa variable 1−6) p= 0 FC= −1.3
Log2 Intensity
9.5
9.0
8.5
8.0
7.5
7.0
10
15
20
25
30
35
40
Gestational age (weeks)

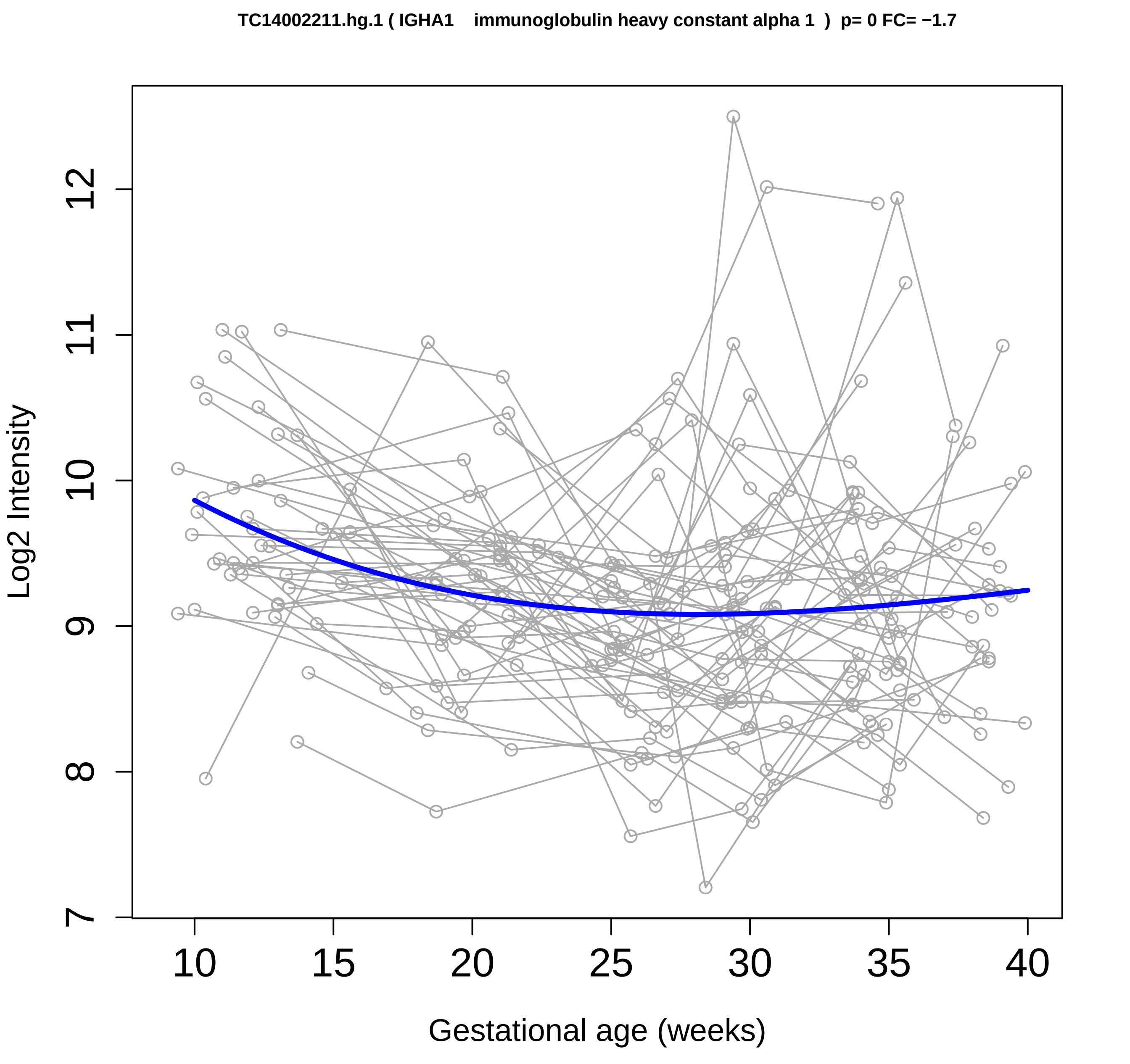

TC14002211.hg.1 (IGHA1 immunoglobulin heavy constant alpha 1) p= 0 FC= −1.7
Log2 Intensity
7
8
9
10
11
12
10
15
20
25
30
35
40
Gestational age (weeks)

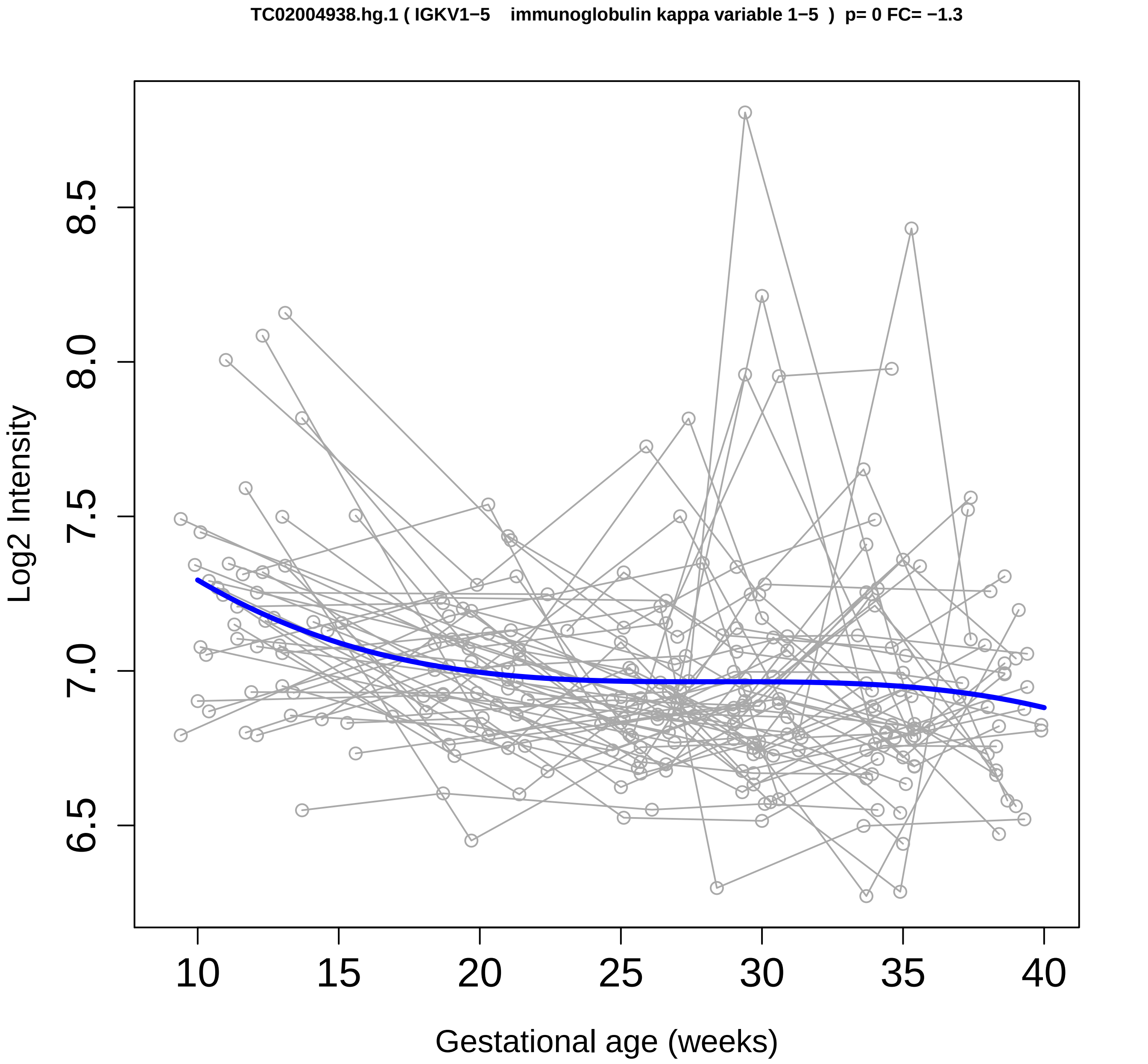

TC02004938.hg.1 (IGKV1−5 immunoglobulin kappa variable 1−5) p= 0 FC= −1.3
Log2 Intensity
8.5
8.0
7.5
7.0
6.5
10
15
20
25
30
35
40
Gestational age (weeks)

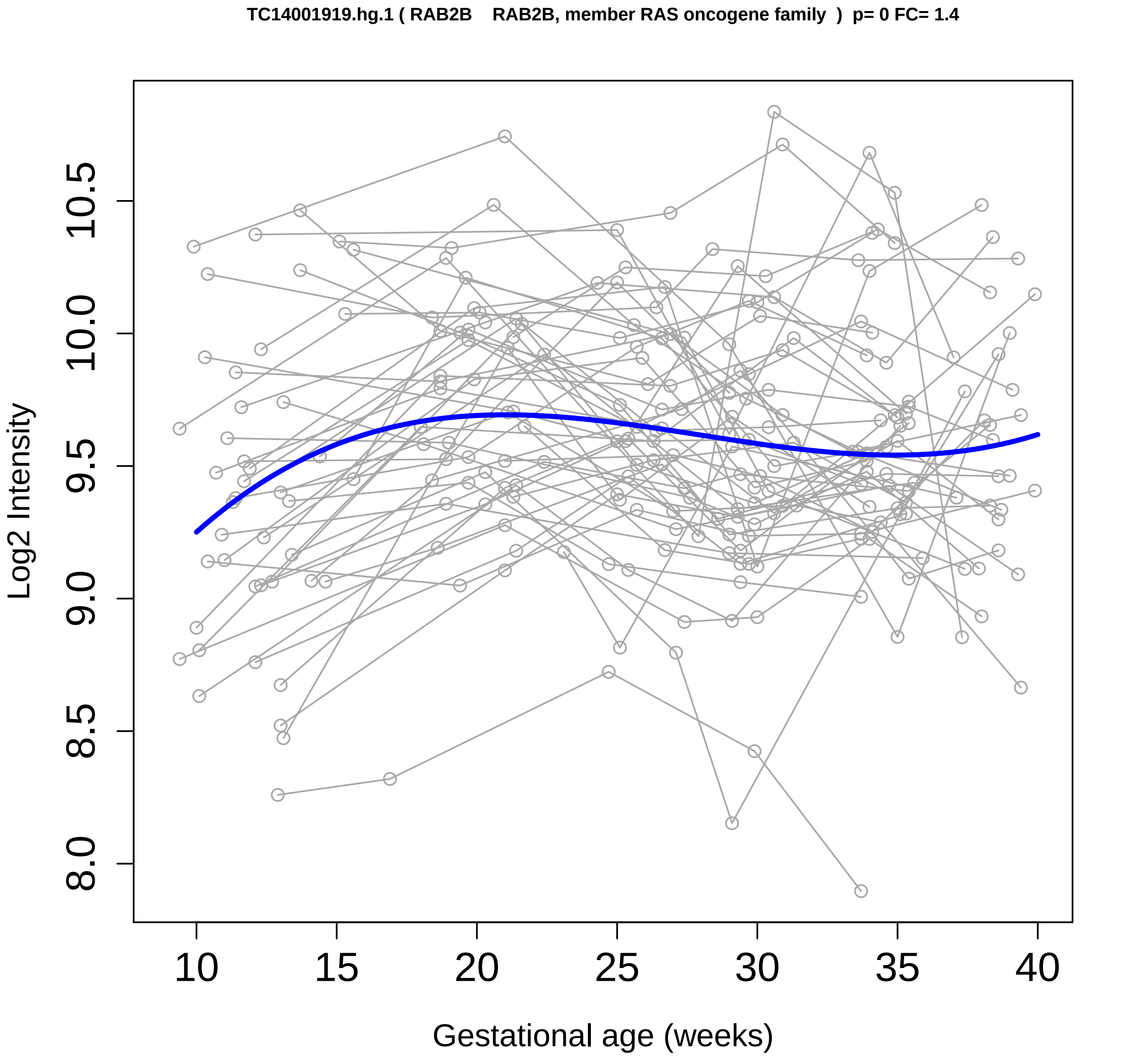
TC14001919.hg.1 (RAB2B RAB2B, member RAS oncogene family) p= 0 FC= 1.4
Log2 Intensity
8.0
8.5
9.0
9.5
10.0
10.5
10
15
20
25
30
35
40
Gestational age (weeks)

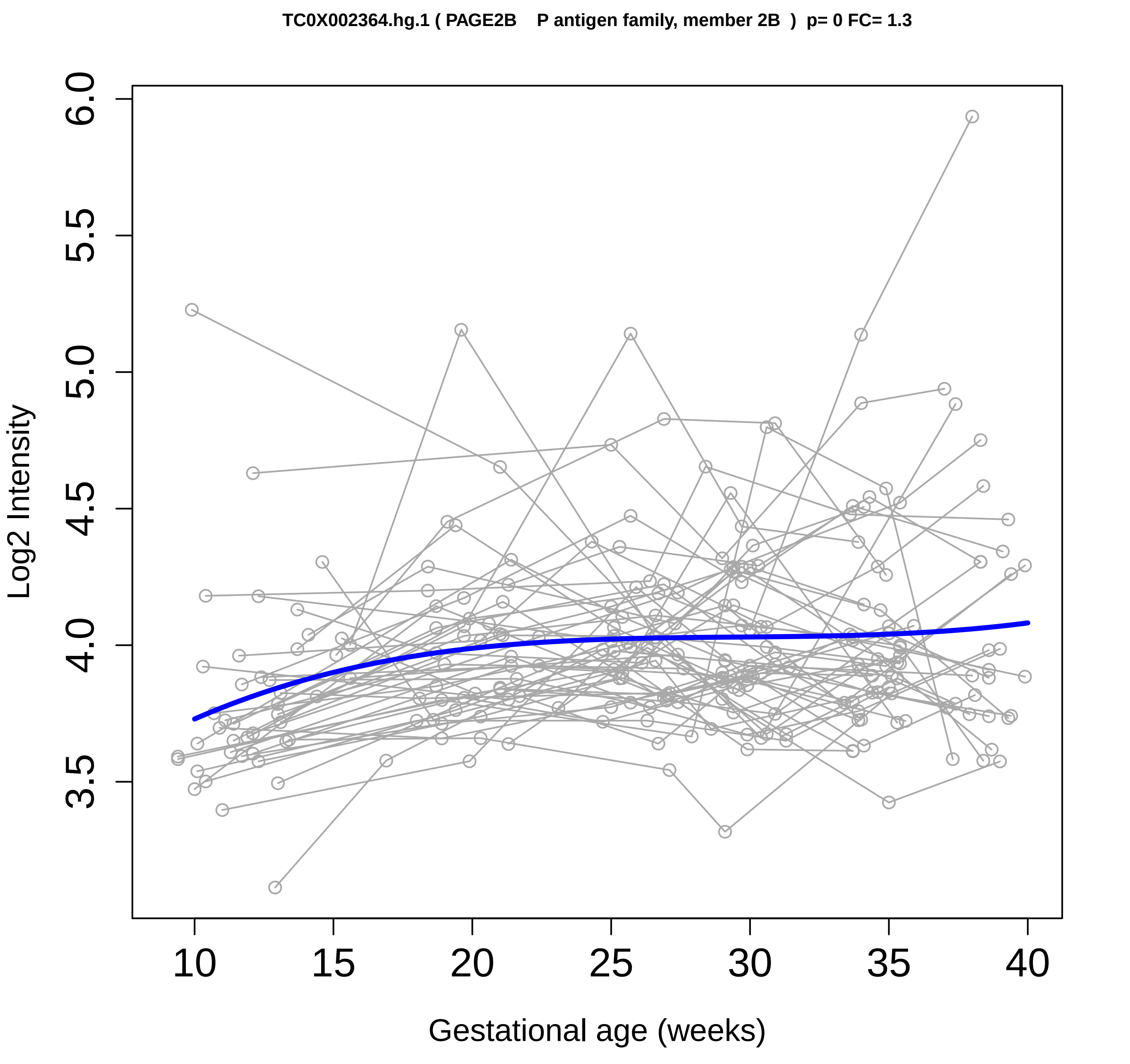
TC0X002364.hg.1 (PAGE2B P antigen family, member 2B) p= 0 FC= 1.3
Log2 Intensity
3.5
4.0
4.5
5.0
5.5
6.0
10
15
20
25
30
35
40
Gestational age (weeks)

TC04002763.hg.1 (GYPB   glycophorin B (MNS blood group)) p= 0 FC= 1.4

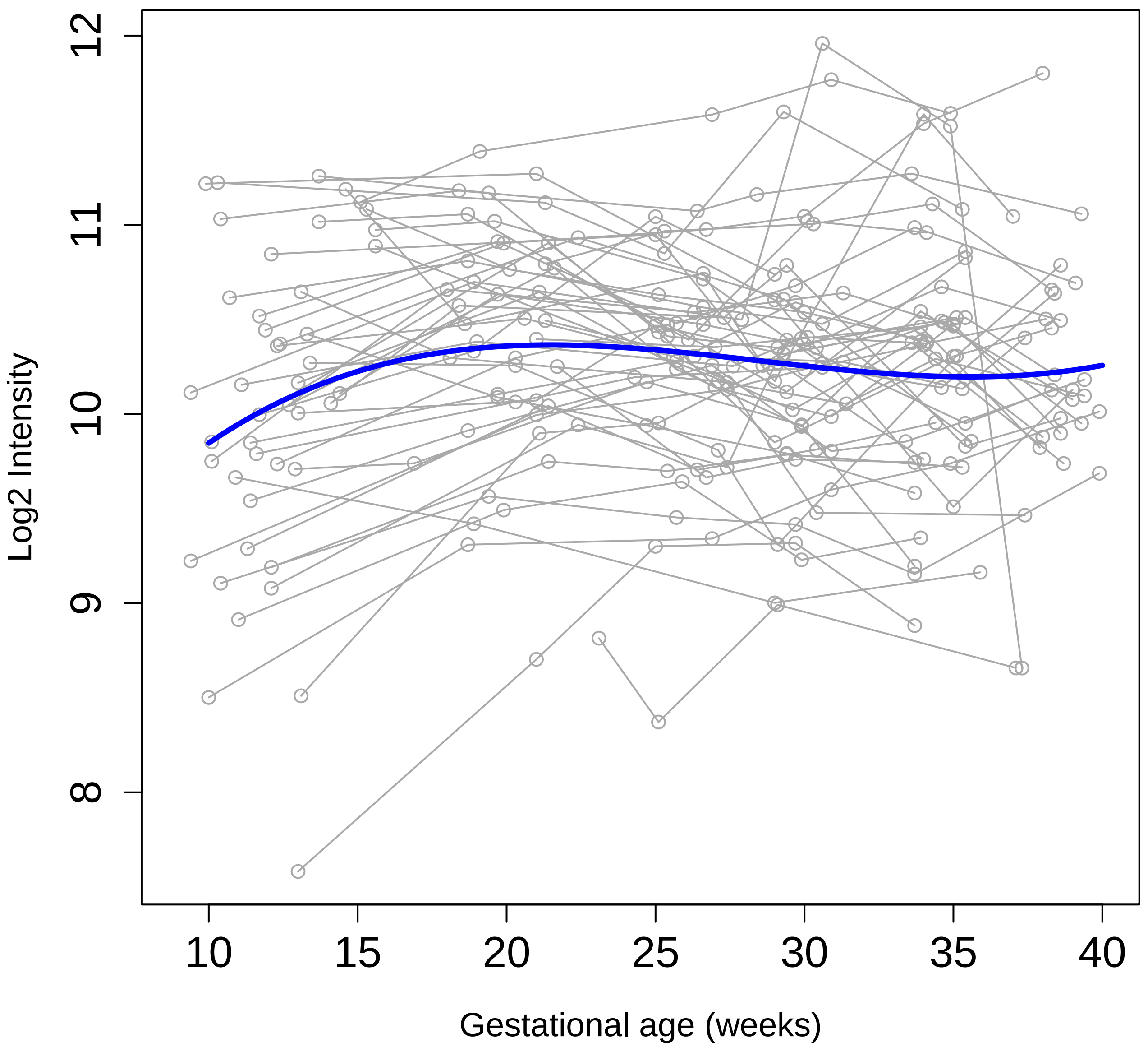

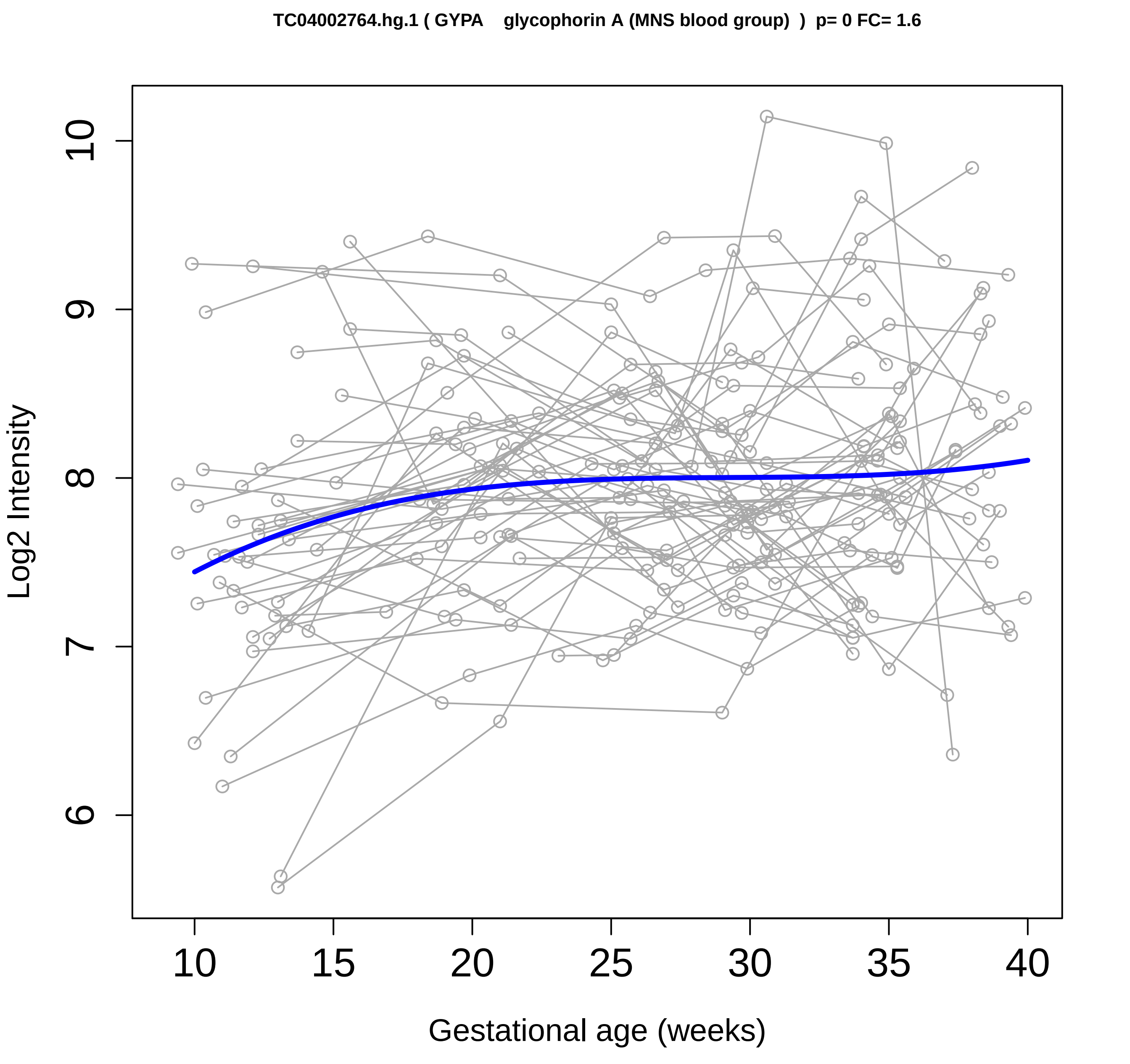

TC04002764.hg.1 (GYPA   glycophorin A (MNS blood group)) p= 0 FC= 1.6
Log2 Intensity
10
9
8
7
6
10
15
20
25
30
35
40
Gestational age (weeks)

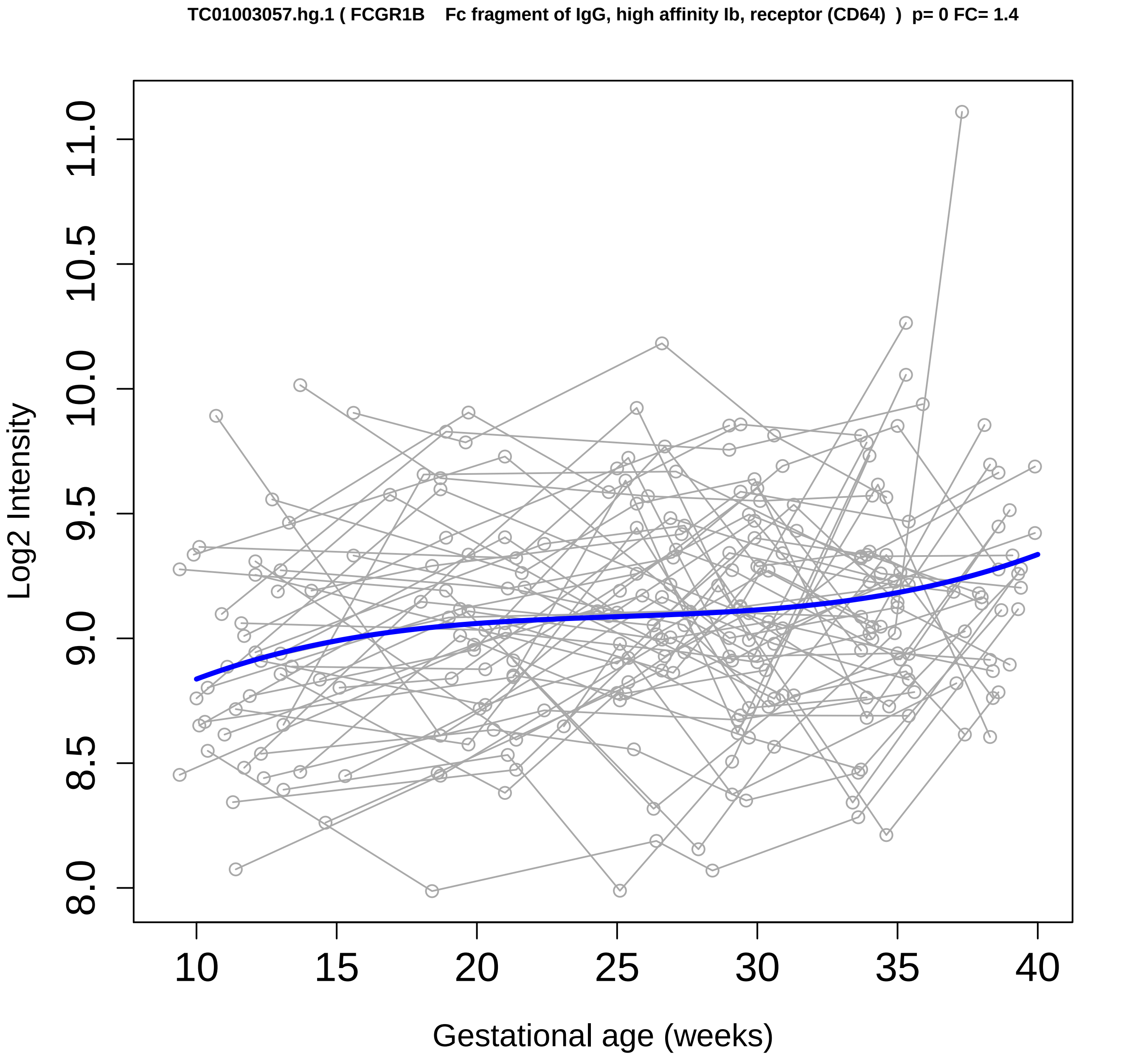

TC01003057.hg.1 (FCGR1B Fc fragment of IgG, high affinity Ib, receptor (CD64)) p= 0 FC= 1.4
Log2 Intensity
8.0
8.5
9.0
9.5
10.0
10.5
11.0
10
15
20
25
30
35
40
Gestational age (weeks)

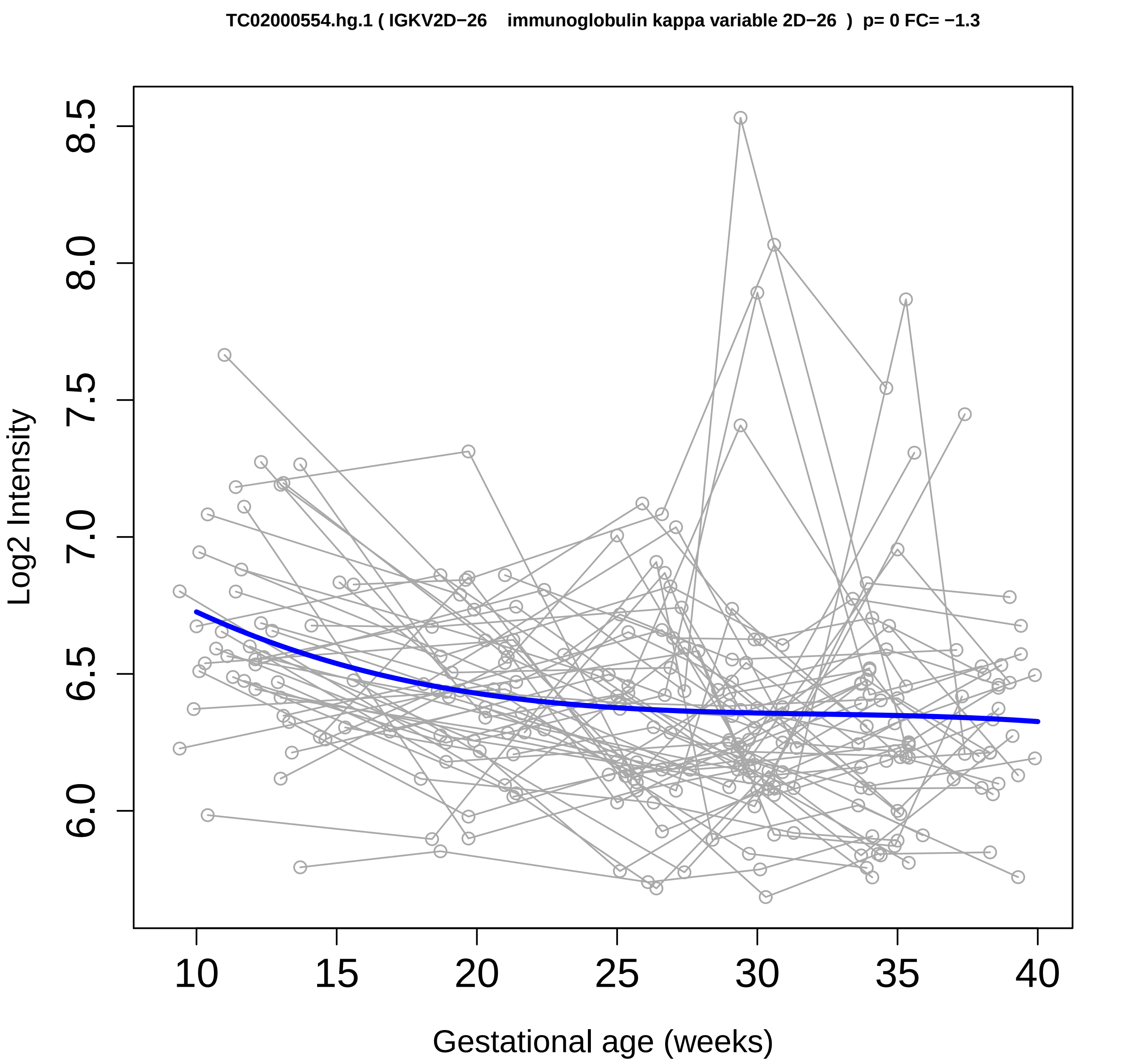
TC02000554.hg.1 (IGKV2D−26 immunoglobulin kappa variable 2D−26) p= 0 FC= −1.3
Log2 Intensity
6.0
6.5
7.0
7.5
8.0
8.5
10
15
20
25
30
35
40
Gestational age (weeks)

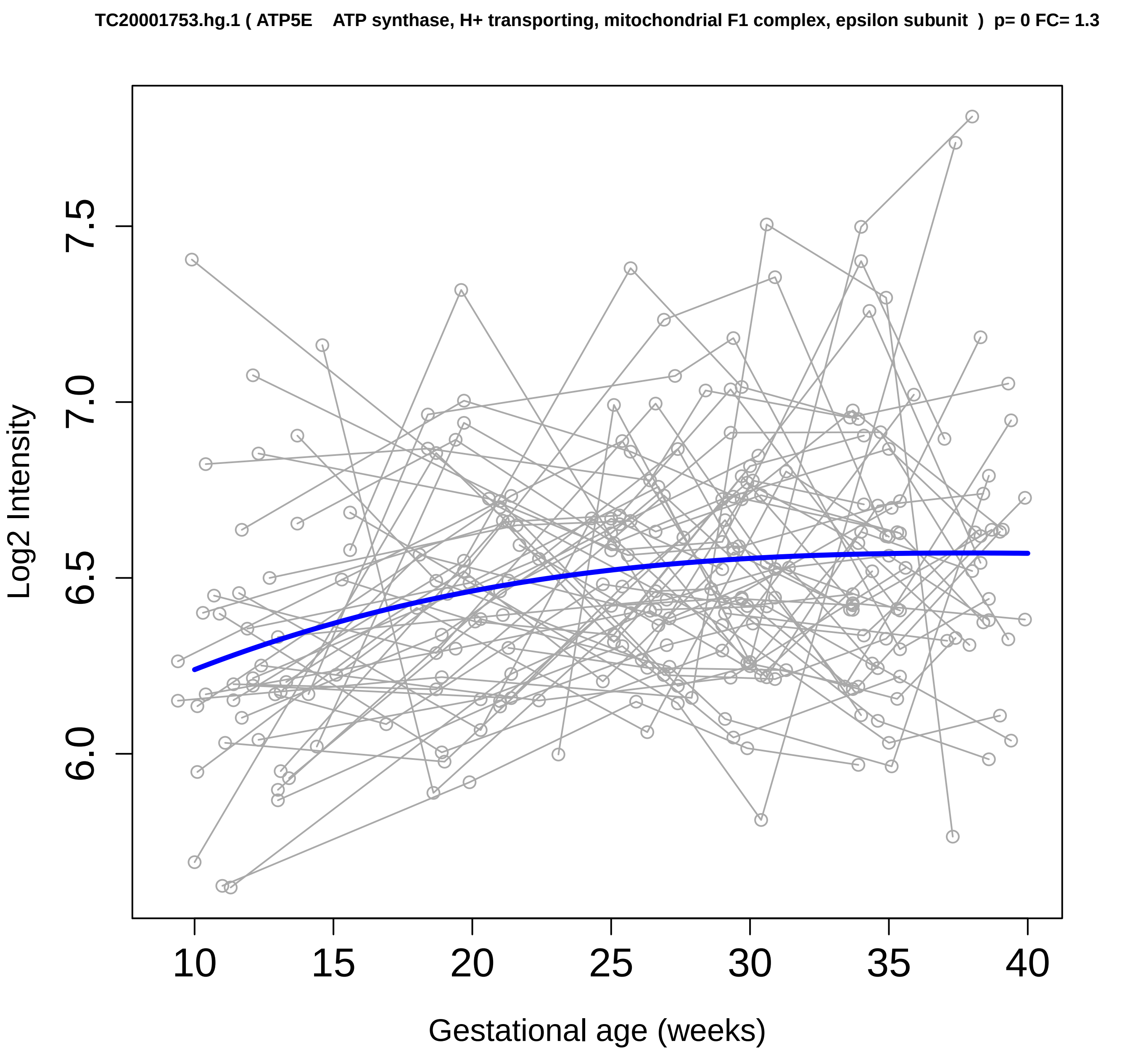
TC20001753.hg.1 (ATP5E   ATP synthase, H+ transporting, mitochondrial F1 complex, epsilon subunit) p= 0 FC= 1.3
Log2 Intensity
6.0
6.5
7.0
7.5
10
15
20
25
30
35
40
Gestational age (weeks)

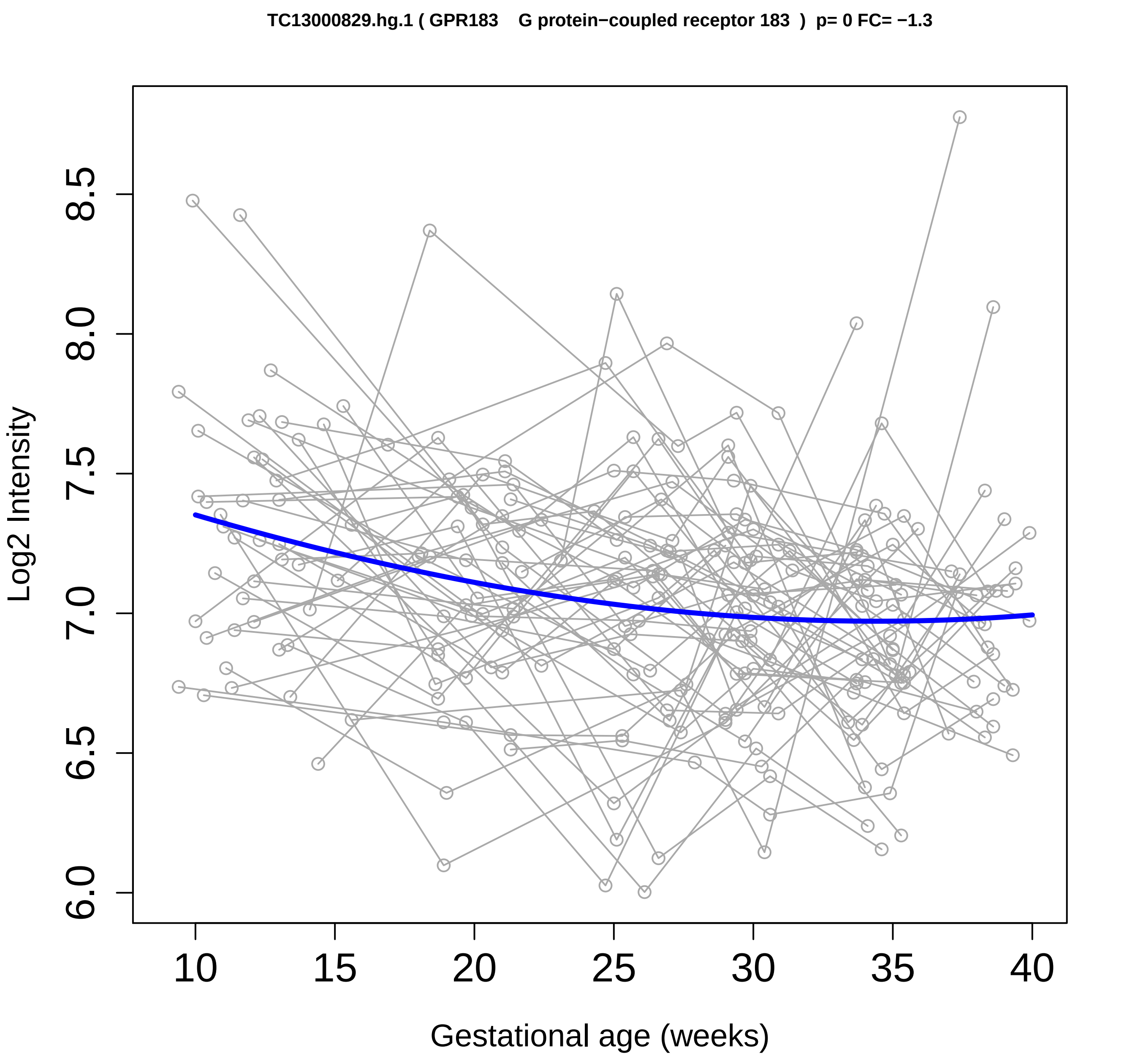
TC13000829.hg.1 (GPR183   G protein−coupled receptor 183) p= 0 FC= −1.3
Log2 Intensity
6.0
6.5
7.0
7.5
8.0
8.5
10
15
20
25
30
35
40
Gestational age (weeks)

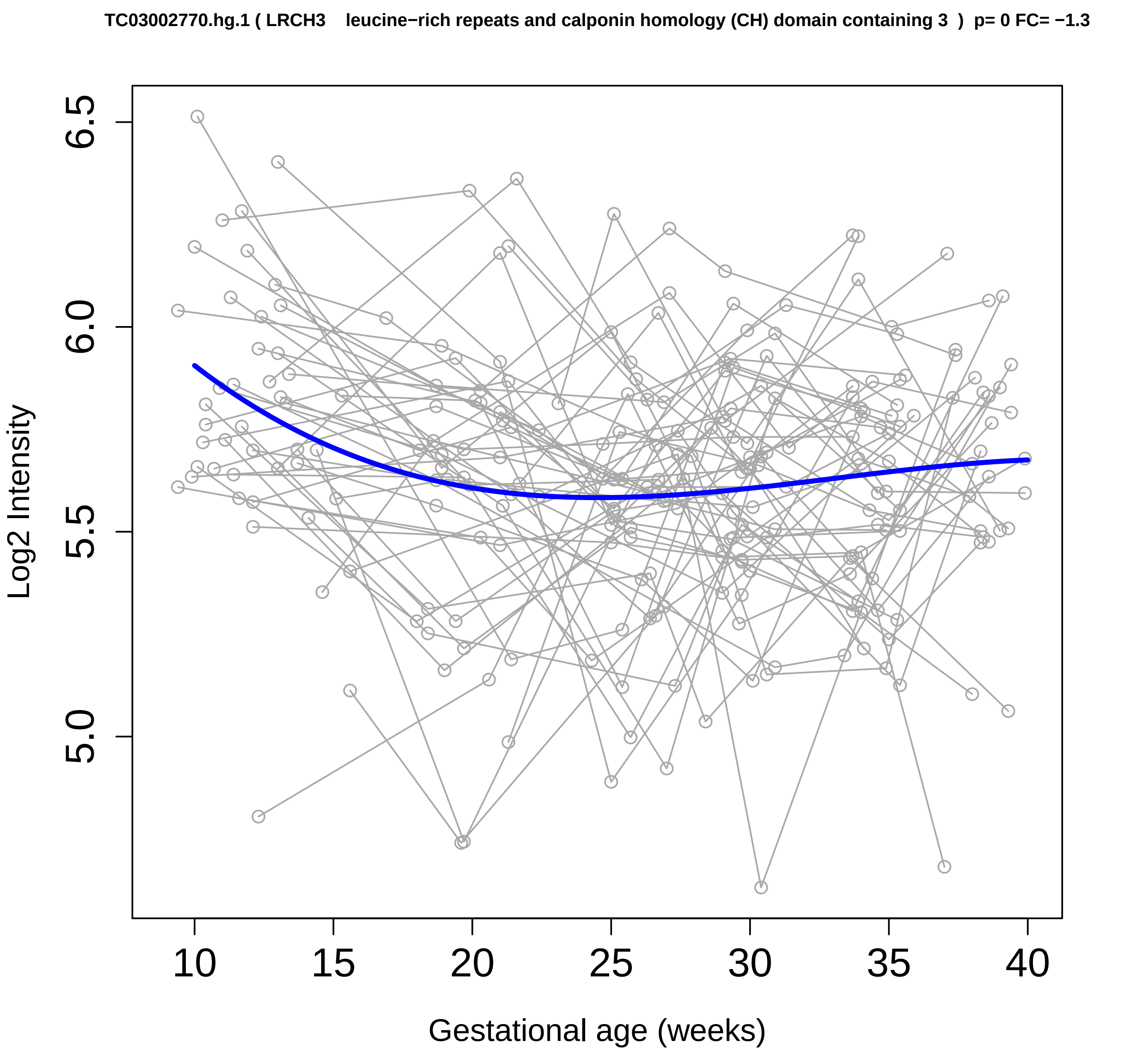
TC03002770.hg.1 (LRCH3   leucine−rich repeats and calponin homology (CH) domain containing 3) p= 0 FC= −1.3
Log2 Intensity
6.5
6.0
5.5
5.0
10
15
20
25
30
35
40
Gestational age (weeks)

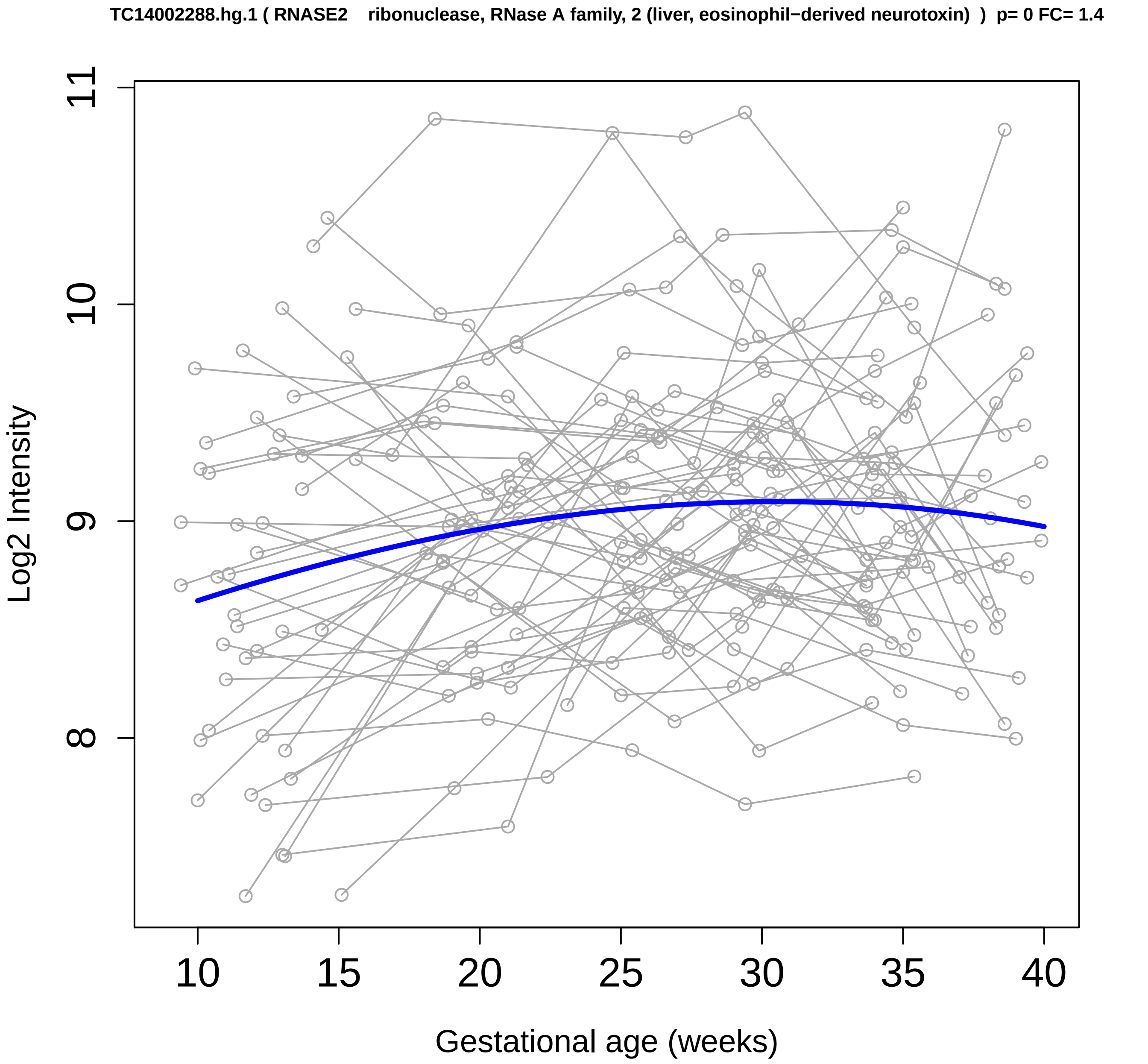
TC14002288.hg.1 (RNASE2 ribonuclease, RNase A family, 2 (liver, eosinophil−derived neurotoxin)) p= 0 FC= 1.4
Log2 Intensity
8
9
10
11
10
15
20
25
30
35
40
Gestational age (weeks)

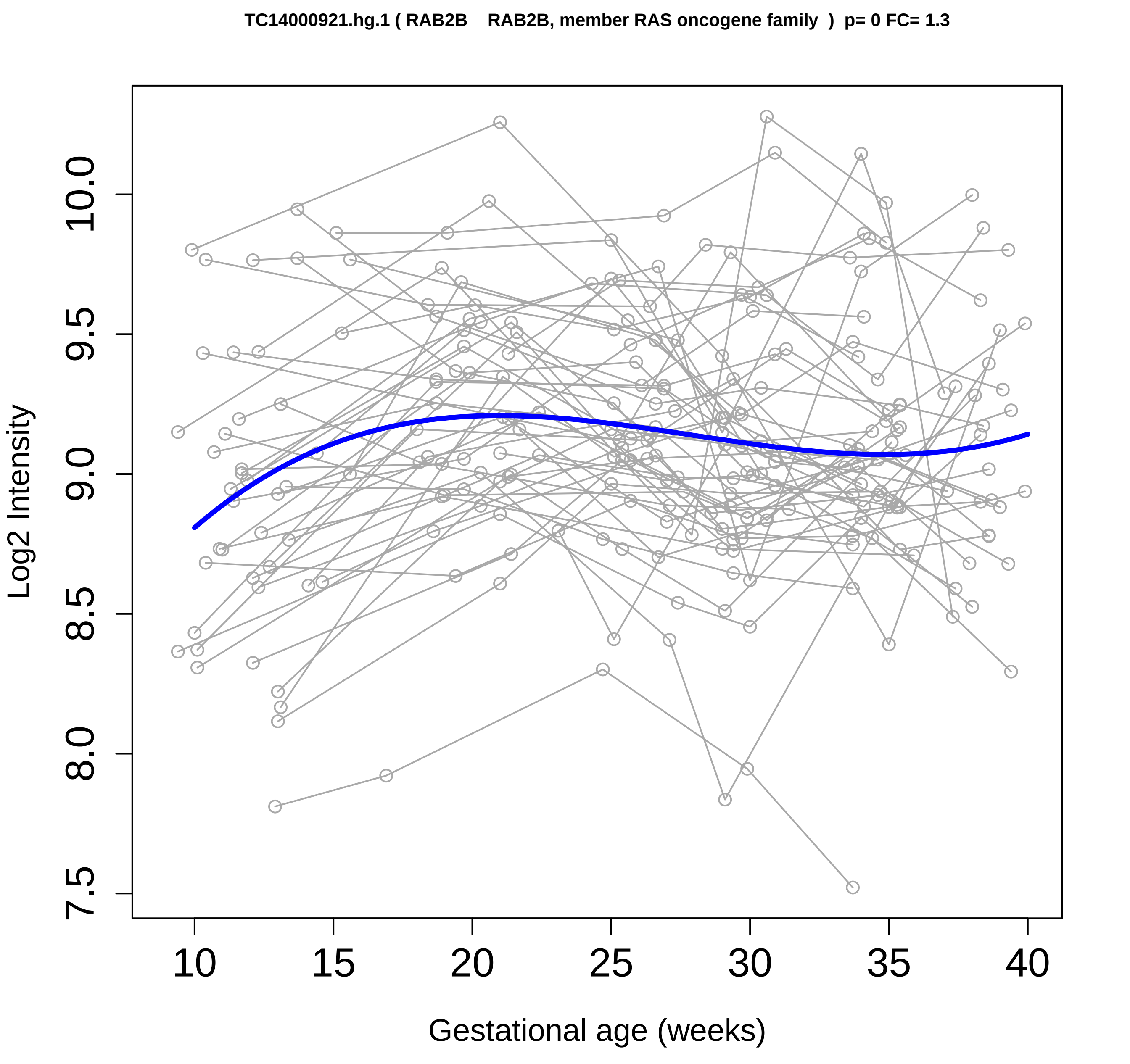
TC14000921.hg.1 (RAB2B   RAB2B, member RAS oncogene family) p= 0 FC= 1.3
Log2 Intensity
7.5
8.0
8.5
9.0
9.5
10.0
10
15
20
25
30
35
40
Gestational age (weeks)

TC12000260.hg.1 (FGFR1OP2   FGFR1 oncogene partner 2) p= 0 FC= 1.3

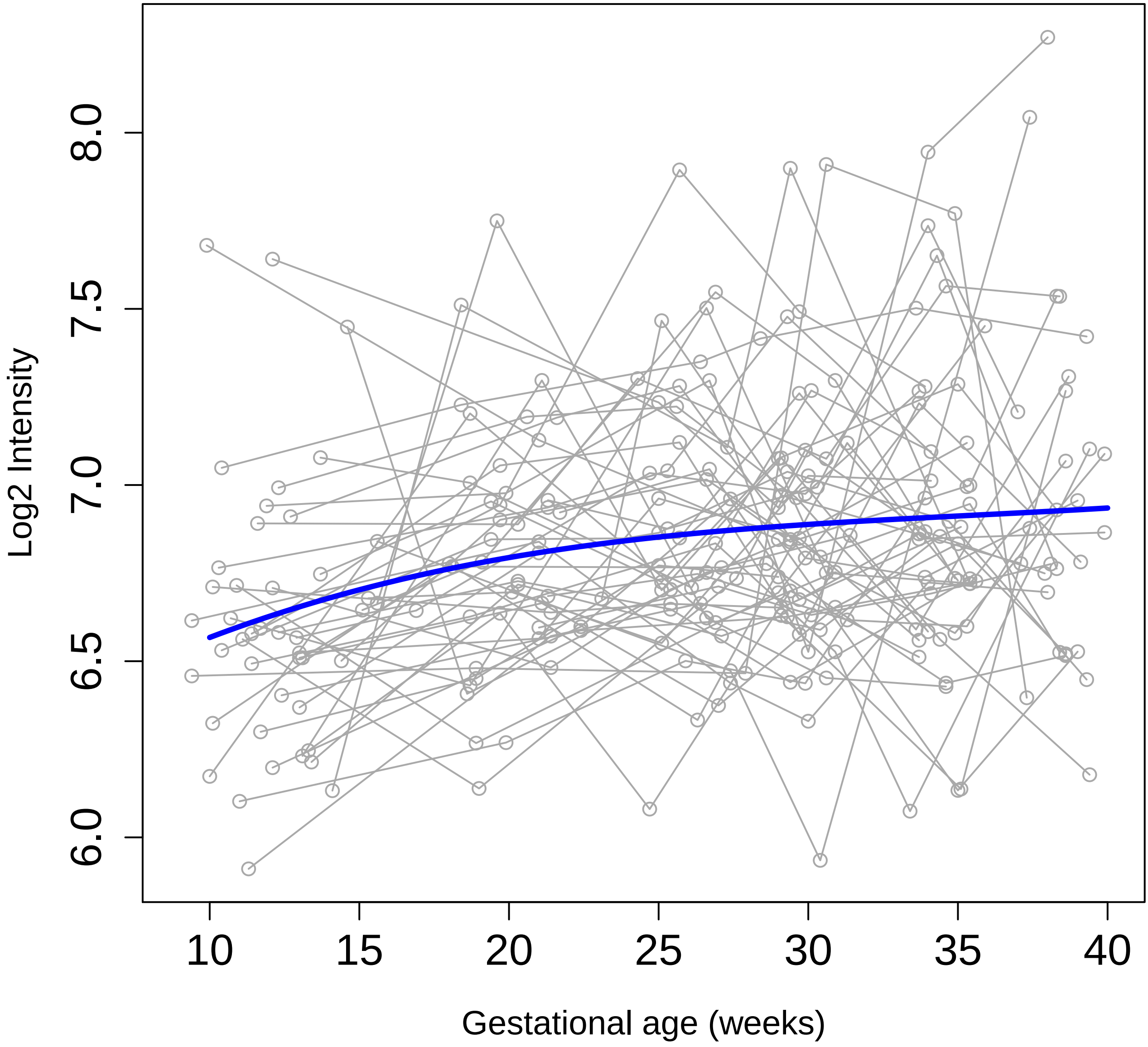

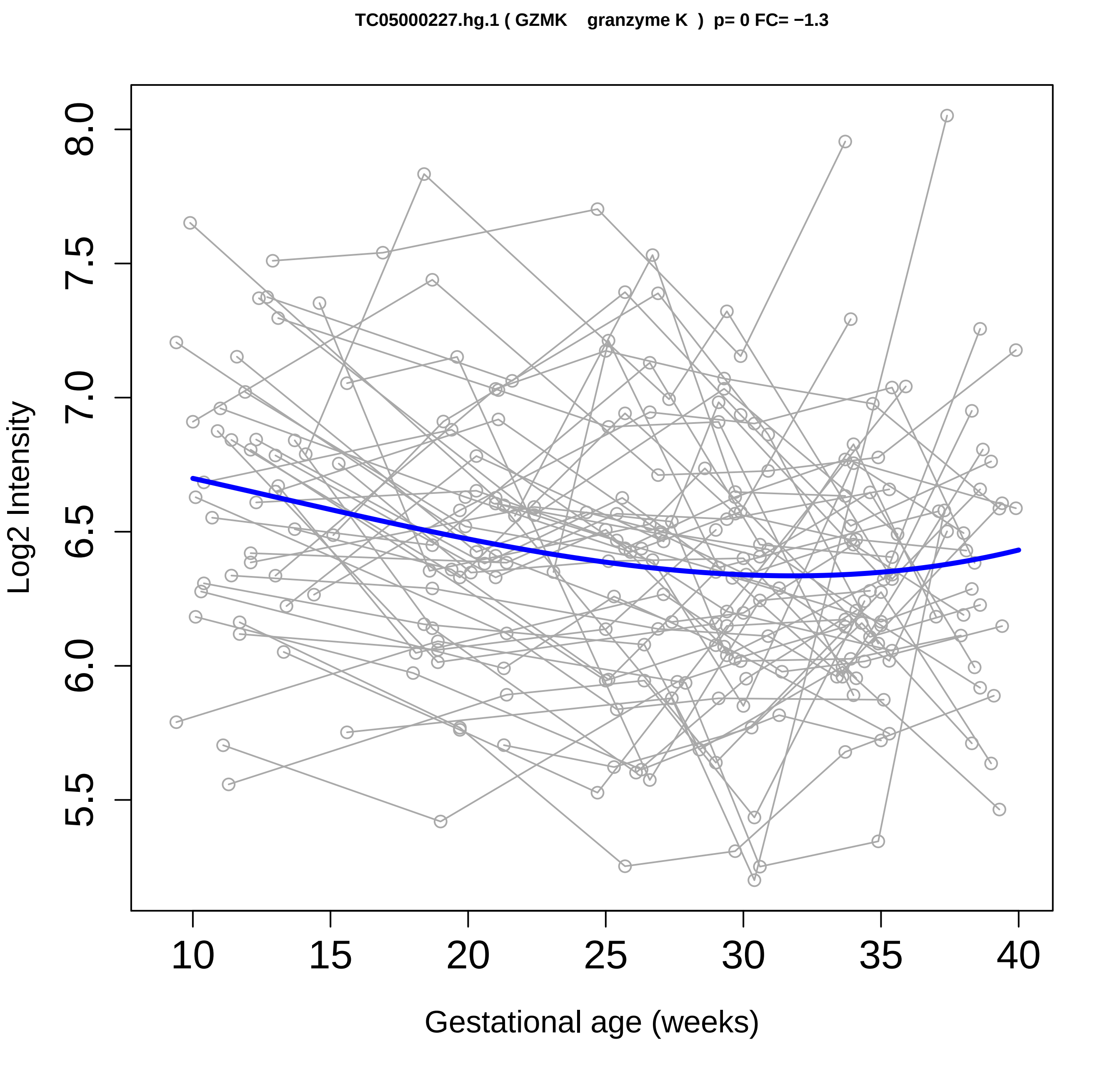
TC05000227.hg.1 (GZMK granzyme K) p= 0 FC= −1.3
Log2 Intensity
5.5
6.0
6.5
7.0
7.5
8.0
10
15
20
25
30
35
40
Gestational age (weeks)

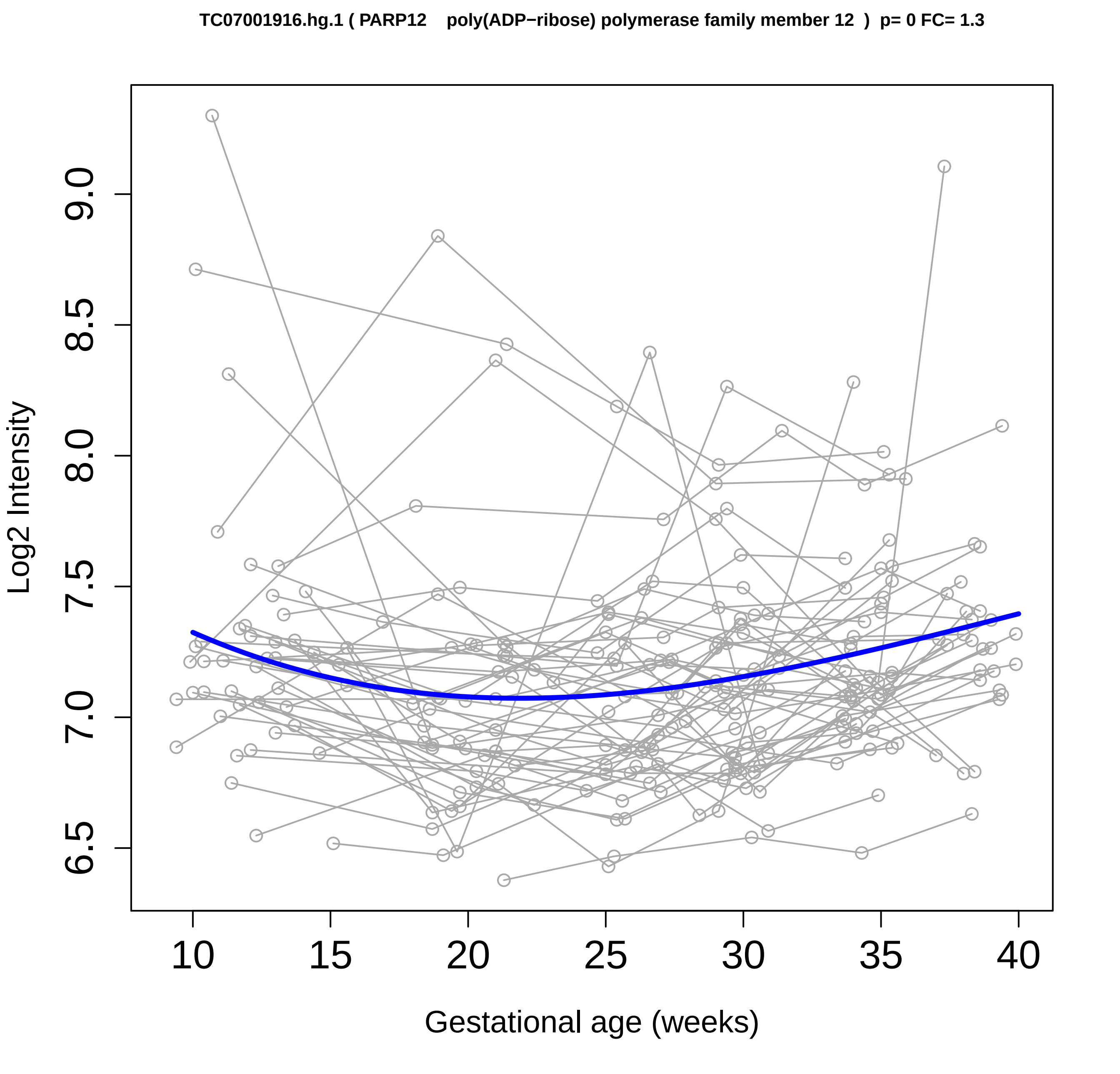
TC07001916.hg.1 (PARP12 poly(ADP−ribose) polymerase family member 12) p= 0 FC= 1.3
Log2 Intensity
6.5
7.0
7.5
8.0
8.5
9.0
10
15
20
25
30
35
40
Gestational age (weeks)

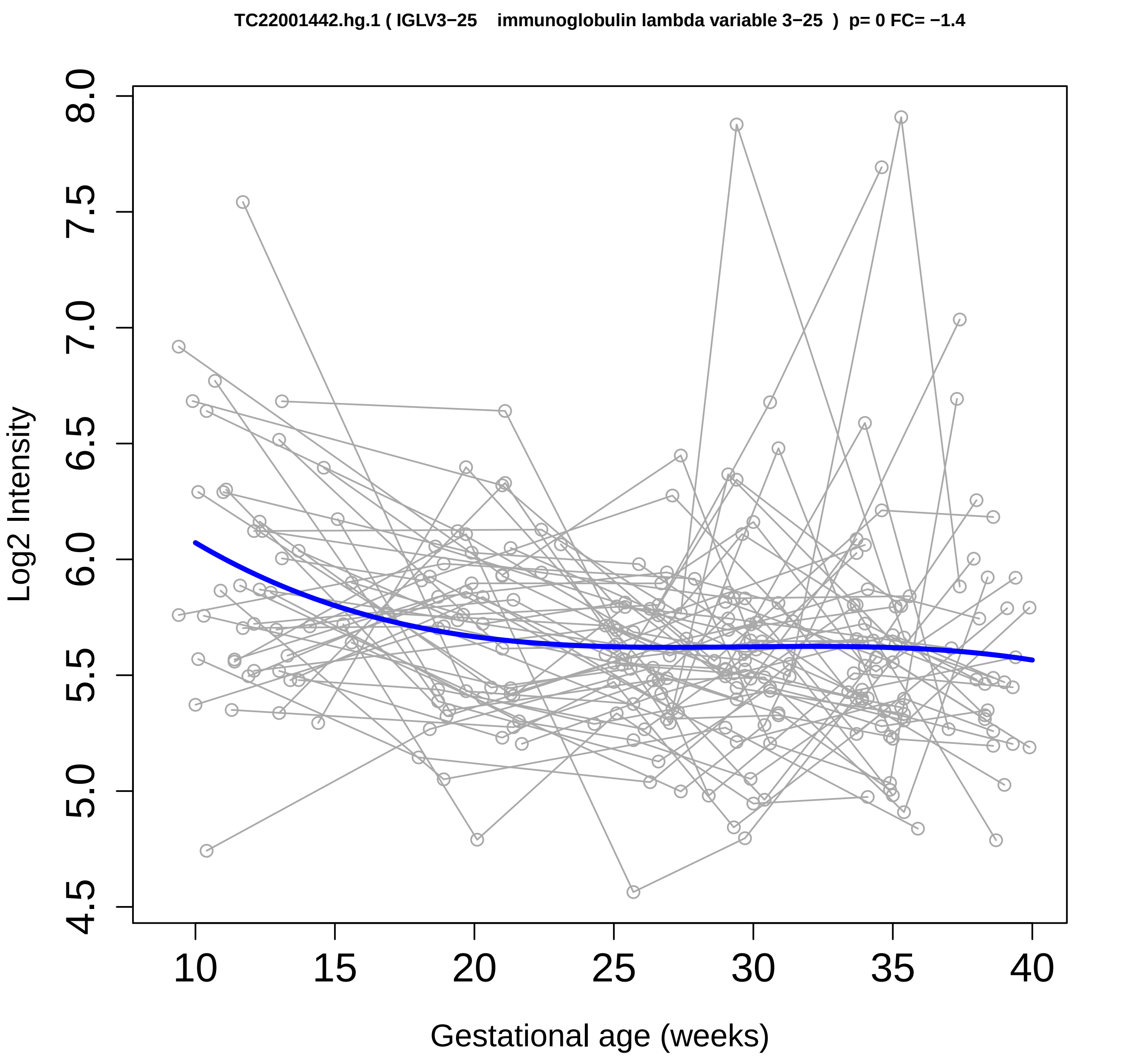
TC22001442.hg.1 (IGLV3−25 immunoglobulin lambda variable 3−25) p= 0 FC= −1.4
Log2 Intensity
4.5
5.0
5.5
6.0
6.5
7.0
7.5
8.0
10
15
20
25
30
35
40
Gestational age (weeks)

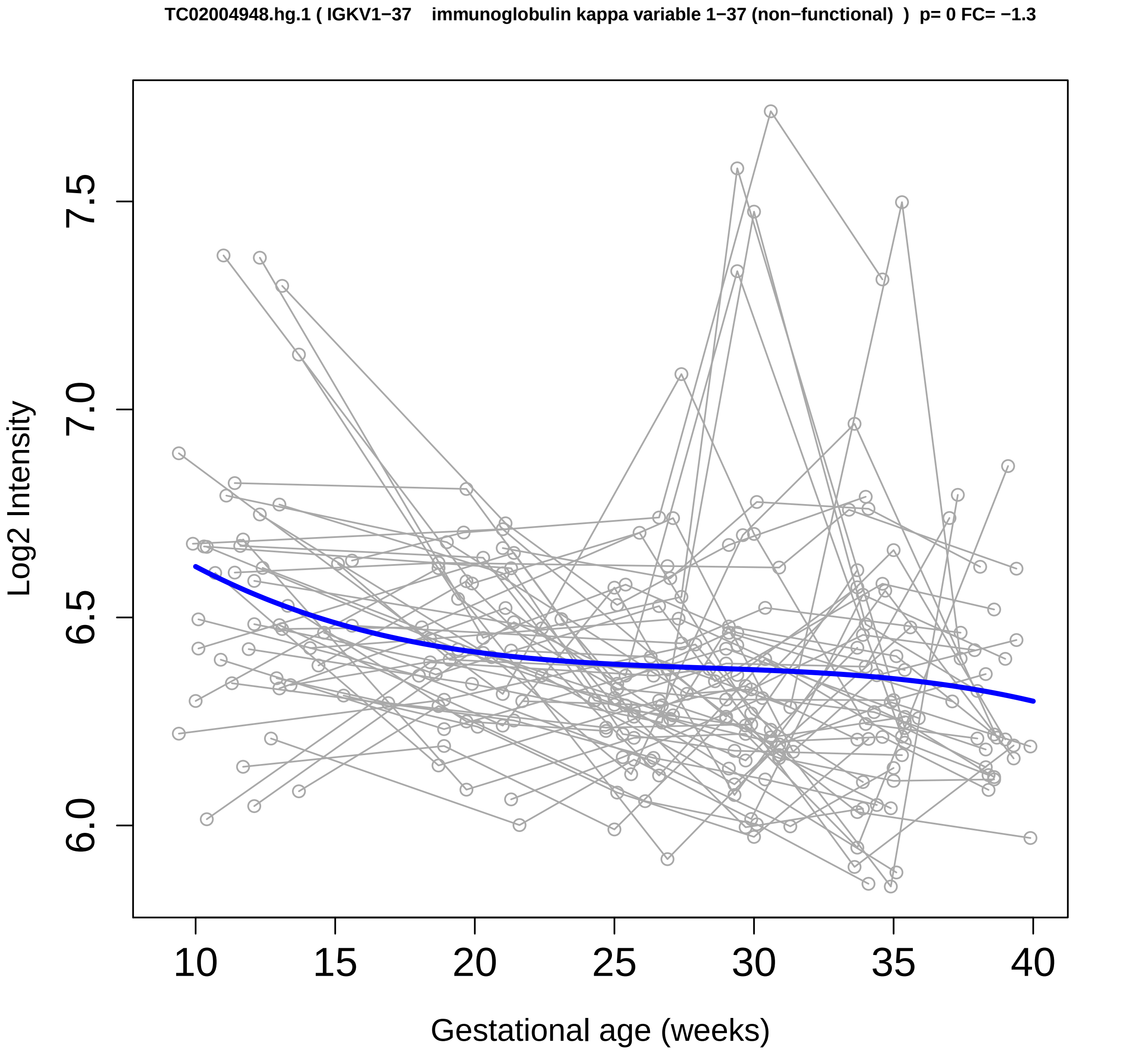

TC02004948.hg.1 (IGKV1−37 immunoglobulin kappa variable 1−37 (non−functional)) p= 0 FC= −1.3
Log2 Intensity
6.0
6.5
7.0
7.5
10
15
20
25
30
35
40
Gestational age (weeks)

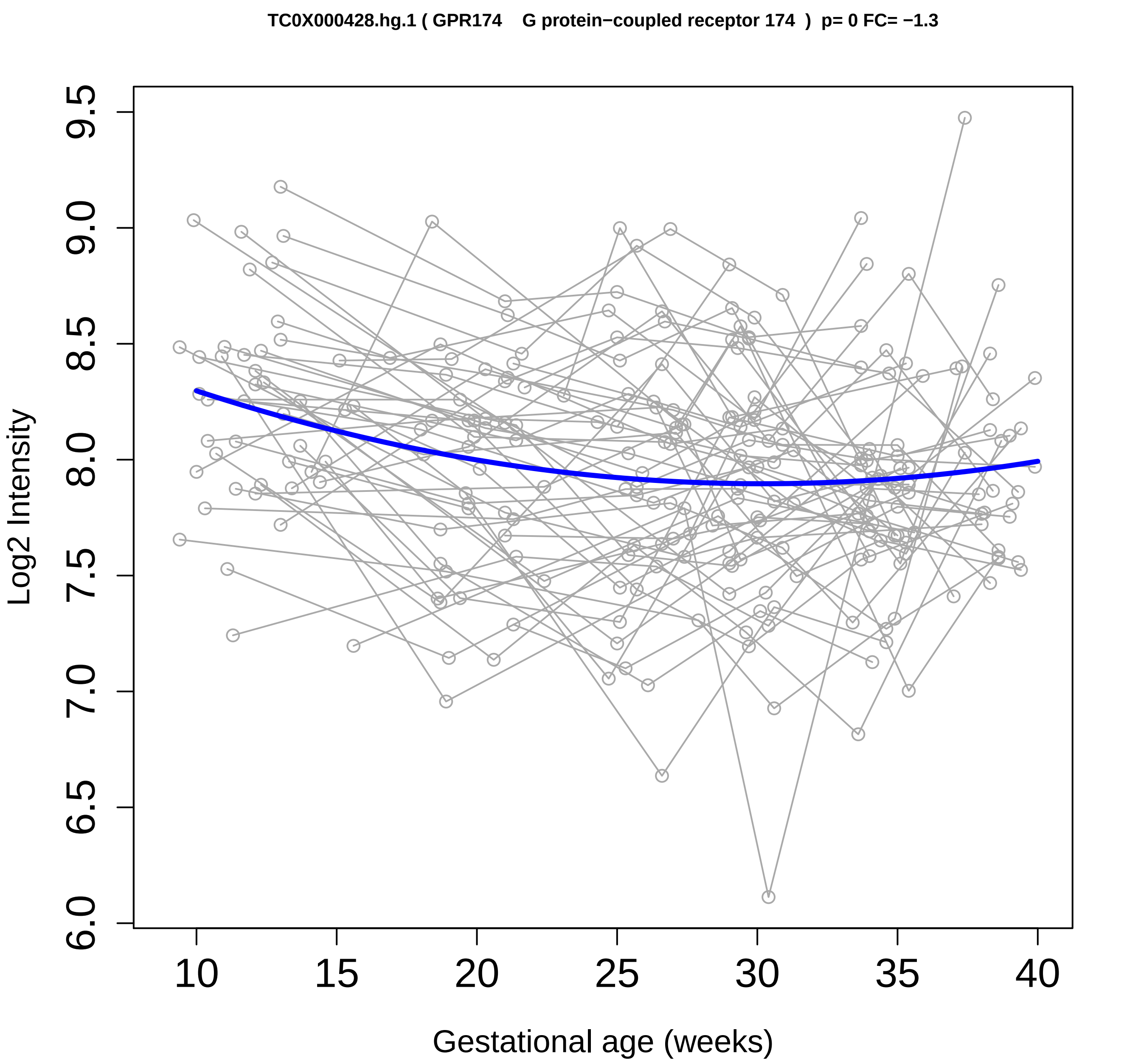
TC0X000428.hg.1 (GPR174 G protein−coupled receptor 174) p= 0 FC= −1.3
Log2 Intensity
9.5
9.0
8.5
8.0
7.5
7.0
6.5
6.0
10
15
20
25
30
35
40
Gestational age (weeks)

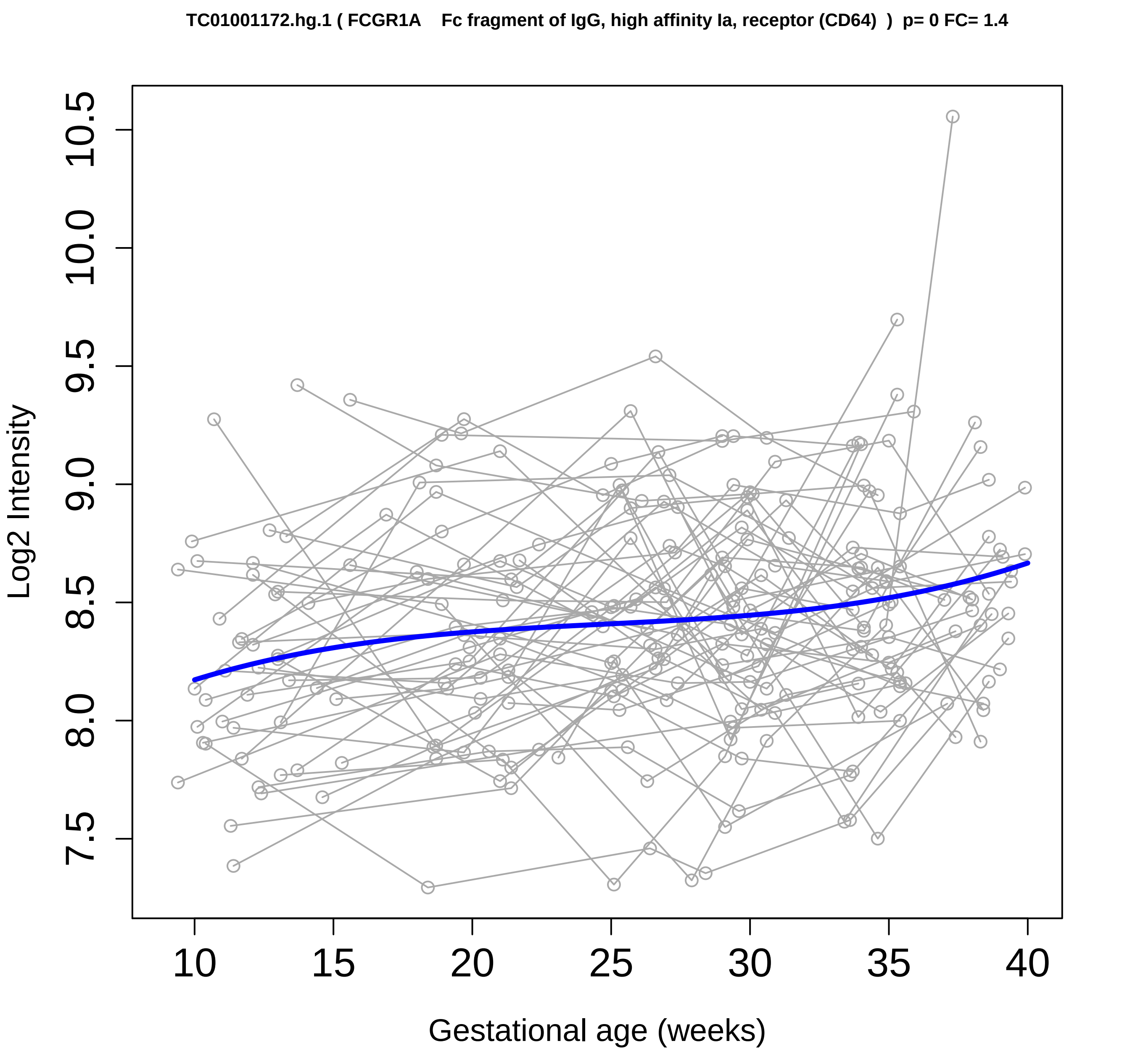
TC01001172.hg.1 (FCGR1A Fc fragment of IgG, high affinity Ia, receptor (CD64)) p= 0 FC= 1.4
Log2 Intensity
10.5
10.0
9.5
9.0
8.5
8.0
7.5
10
15
20
25
30
35
40
Gestational age (weeks)

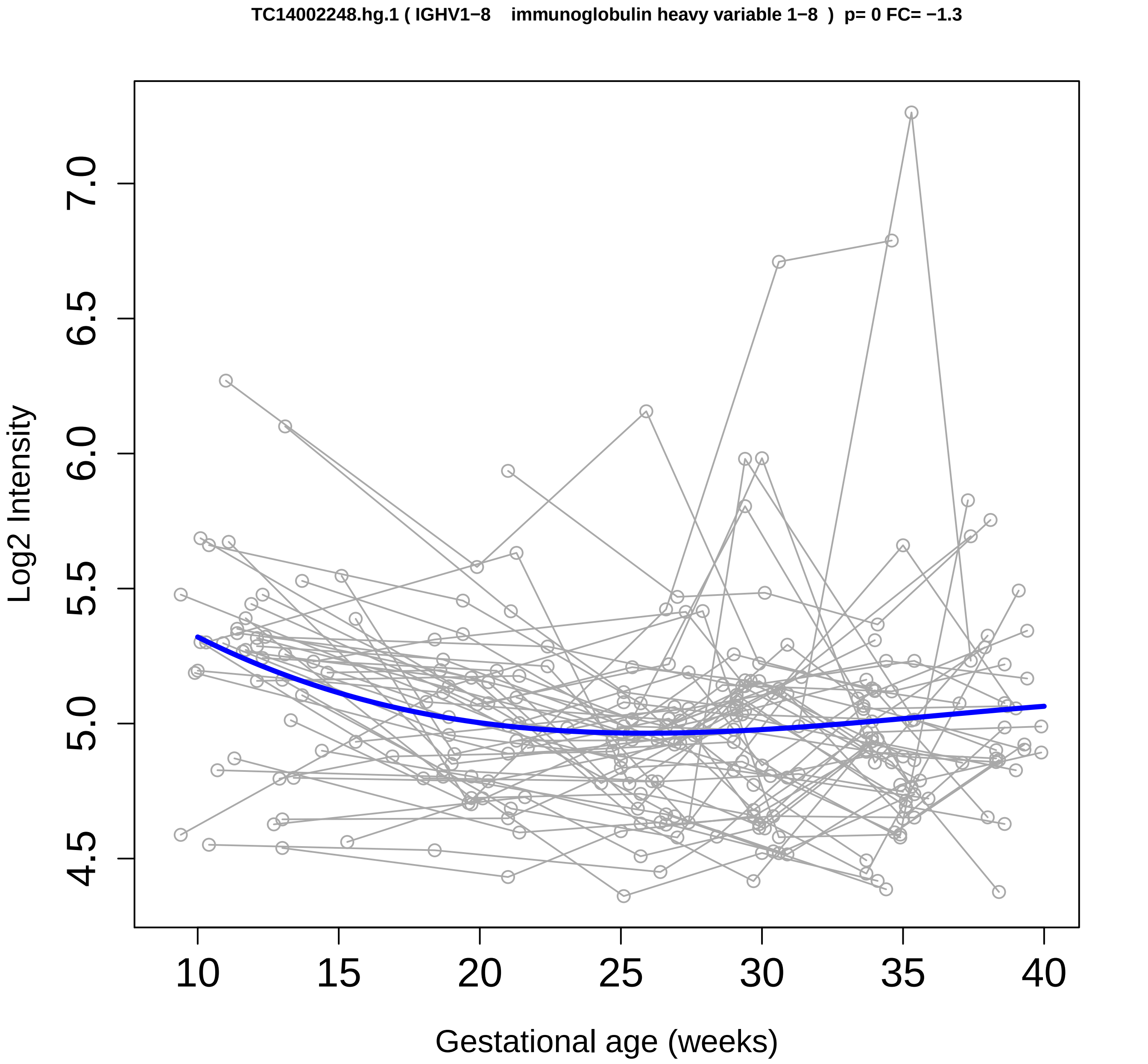
TC14002248.hg.1 (IGHV1−8 immunoglobulin heavy variable 1−8) p= 0 FC= −1.3
Log2 Intensity
7.0
6.5
6.0
5.5
5.0
4.5
10
15
20
25
30
35
40
Gestational age (weeks)

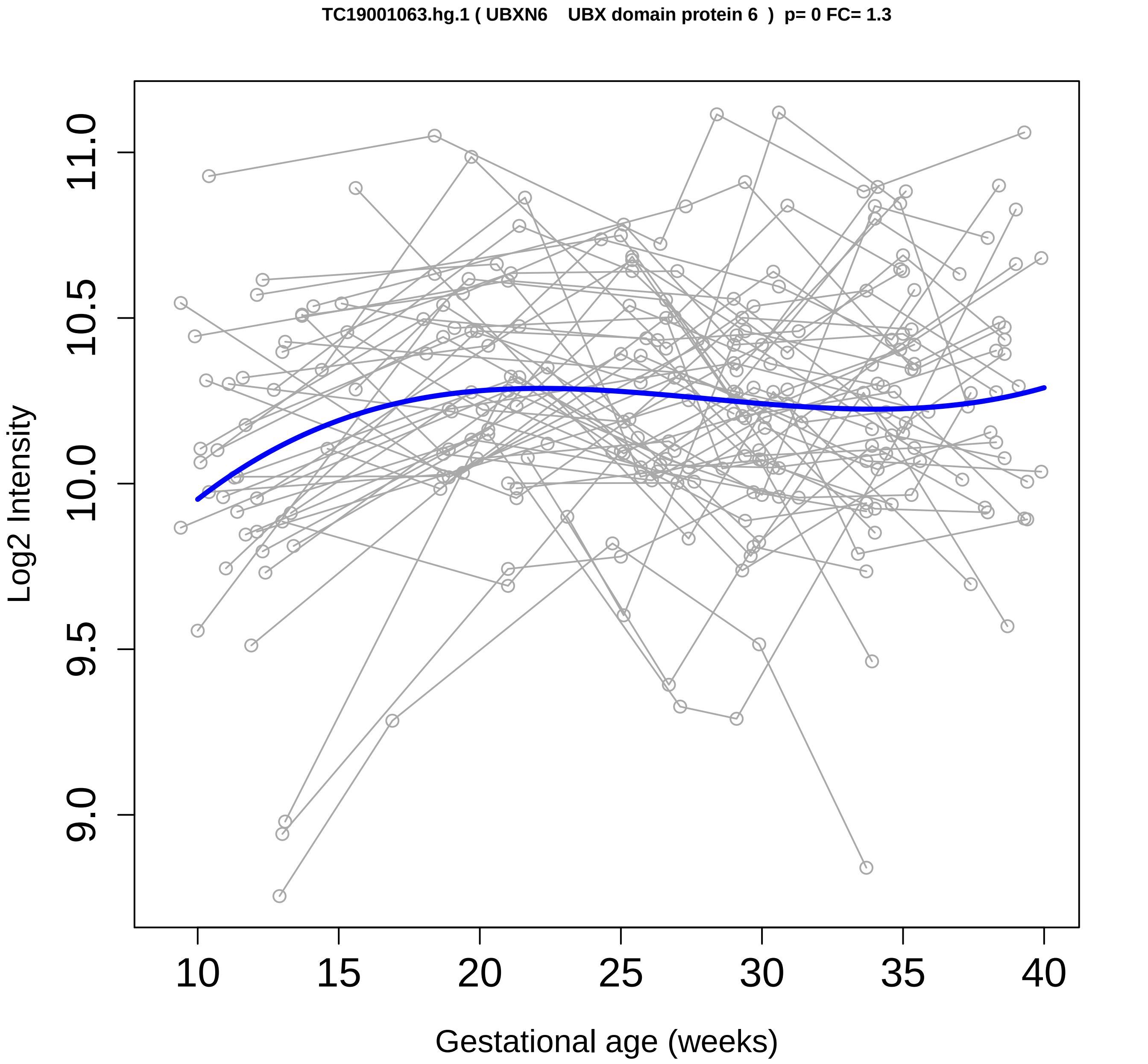

TC19001063.hg.1 (UBXN6 UBX domain protein 6) p= 0 FC= 1.3
Log2 Intensity
11.0
10.5
10.0
9.5
9.0
10
15
20
25
30
35
40
Gestational age (weeks)

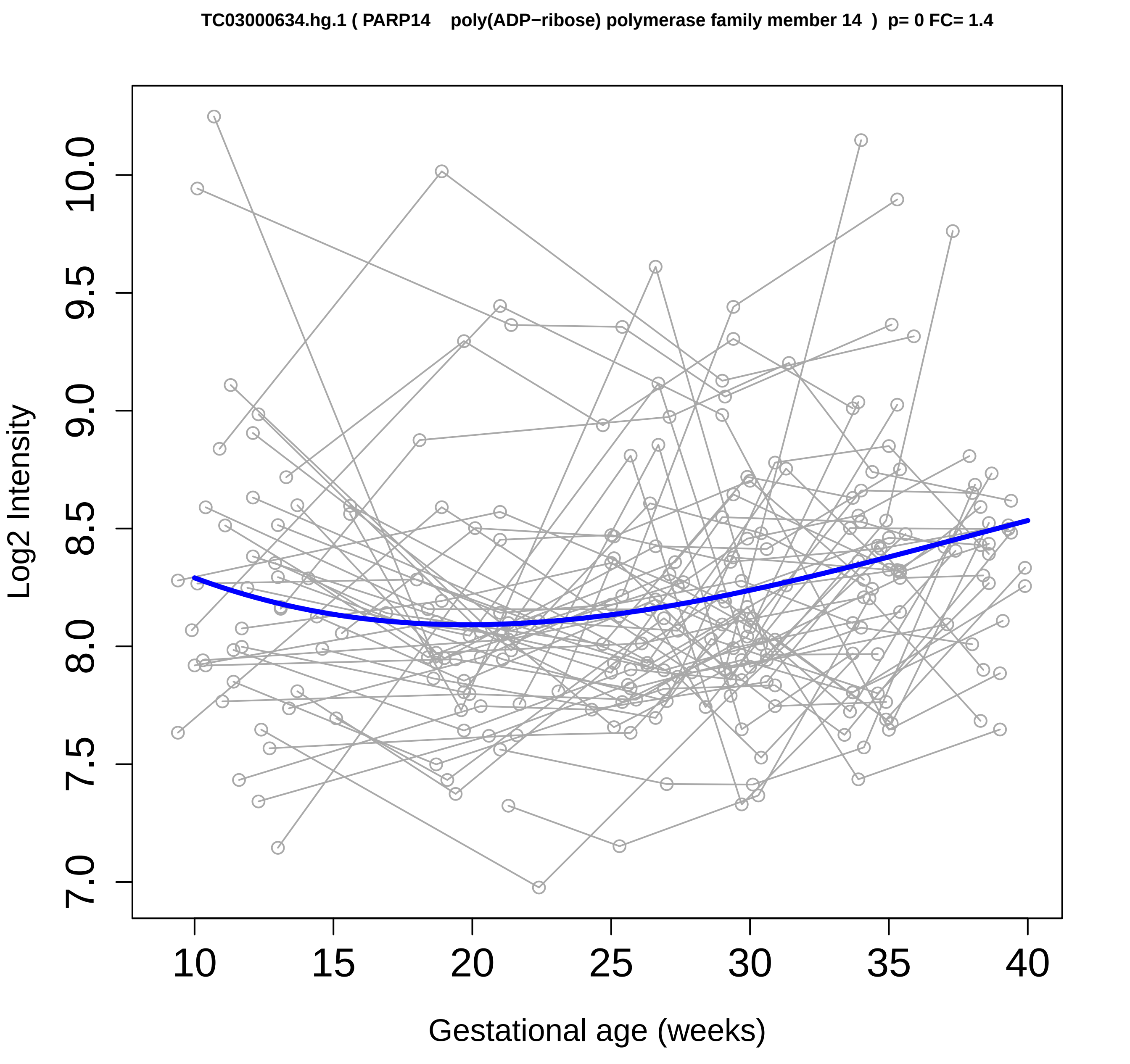
TC03000634.hg.1 (PARP14 poly(ADP−ribose) polymerase family member 14) p= 0 FC= 1.4
Log2 Intensity
7.0
7.5
8.0
8.5
9.0
9.5
10.0
10
15
20
25
30
35
40
Gestational age (weeks)

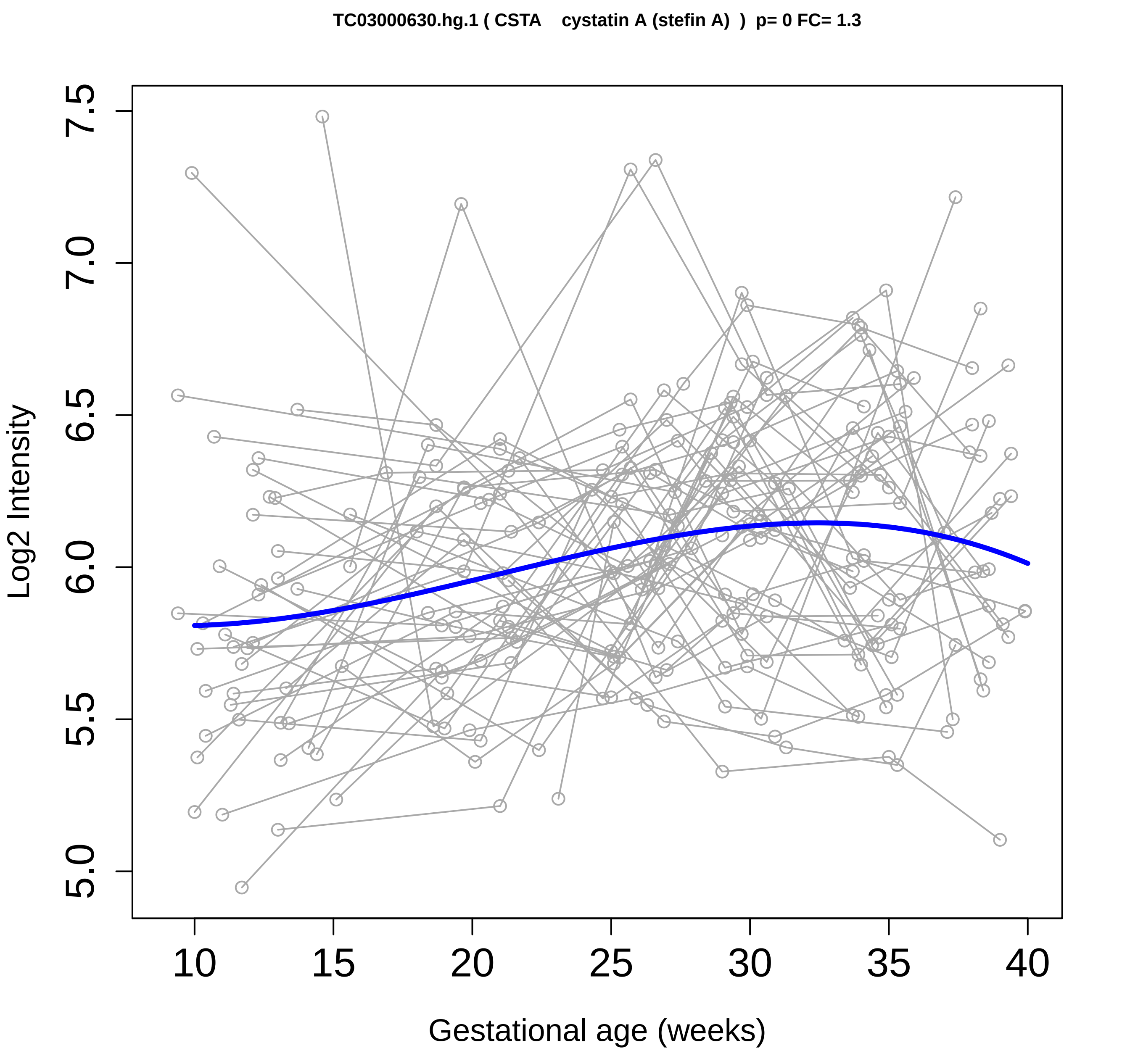

TC03000630.hg.1 (CSTA cystatin A (stefin A)) p= 0 FC= 1.3
Log2 Intensity
7.5
7.0
6.5
6.0
5.5
5.0
10
15
20
25
30
35
40
Gestational age (weeks)

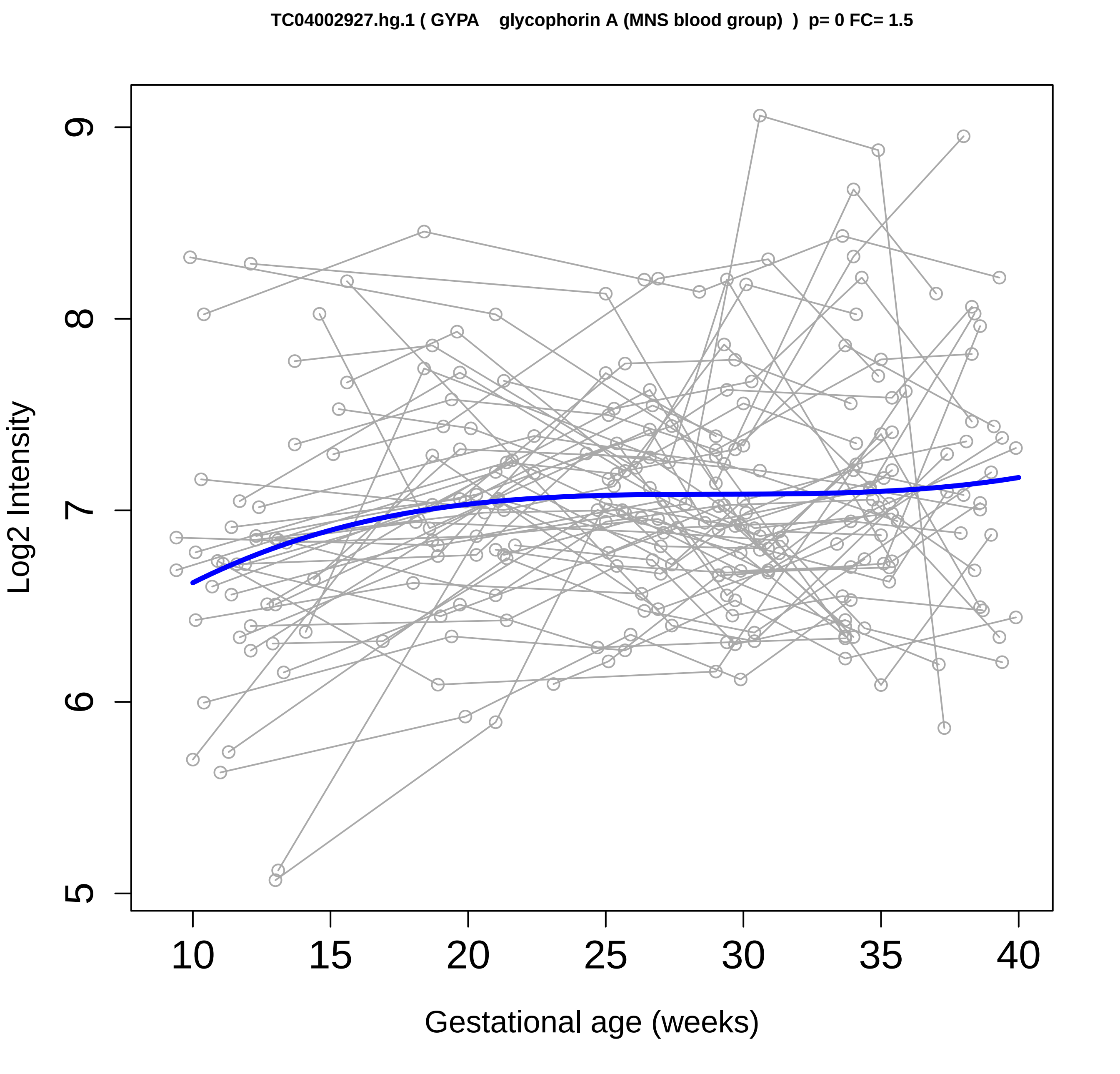

TC04002927.hg.1 (GYPA glycophorin A (MNS blood group)) p= 0 FC= 1.5
Log2 Intensity
5
6
7
8
9
10
15
20
25
30
35
40
Gestational age (weeks)

TC14002277.hg.1 (IGHV4−61   immunoglobulin heavy variable 4−61) p= 0 FC= −1.3

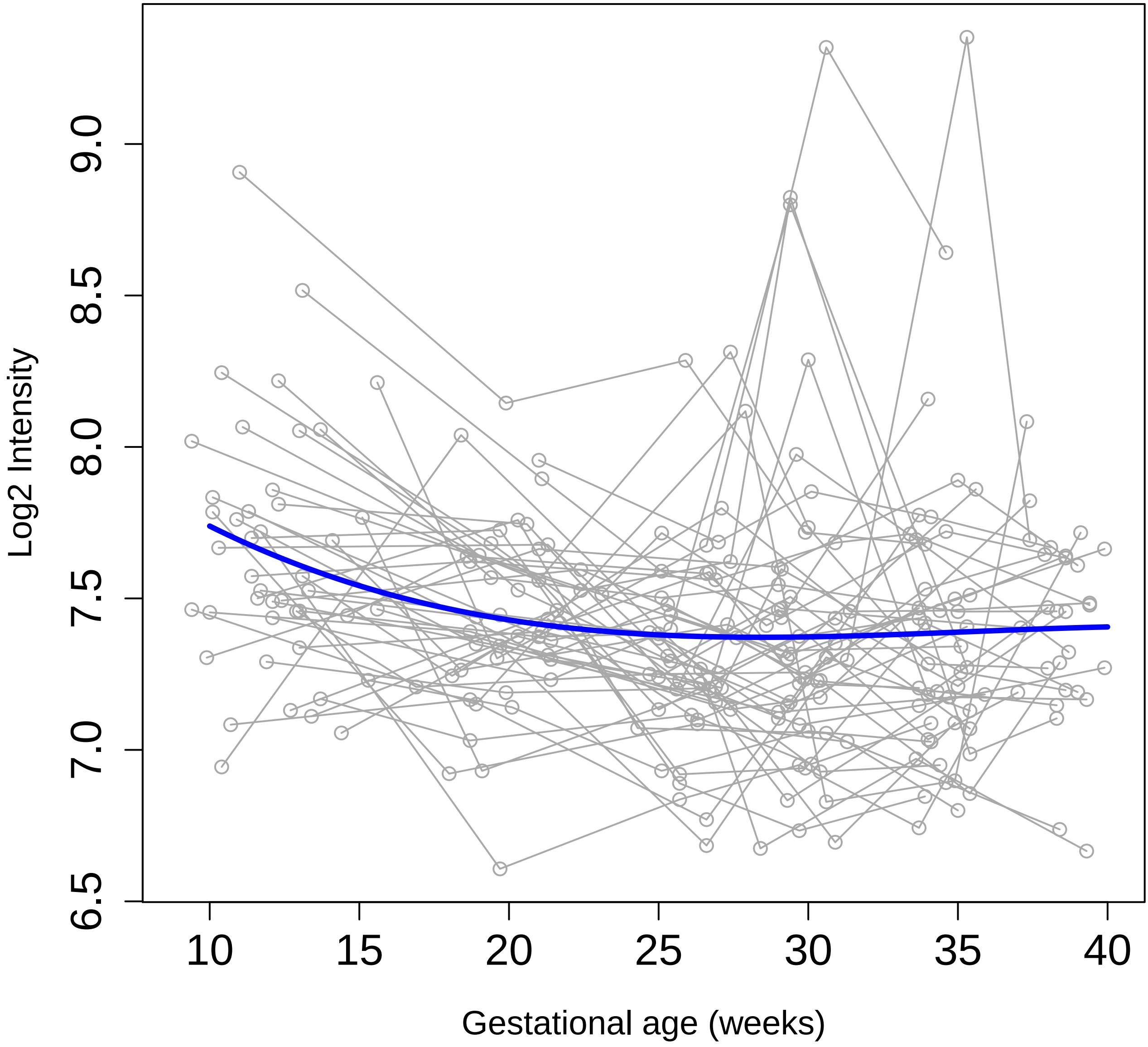

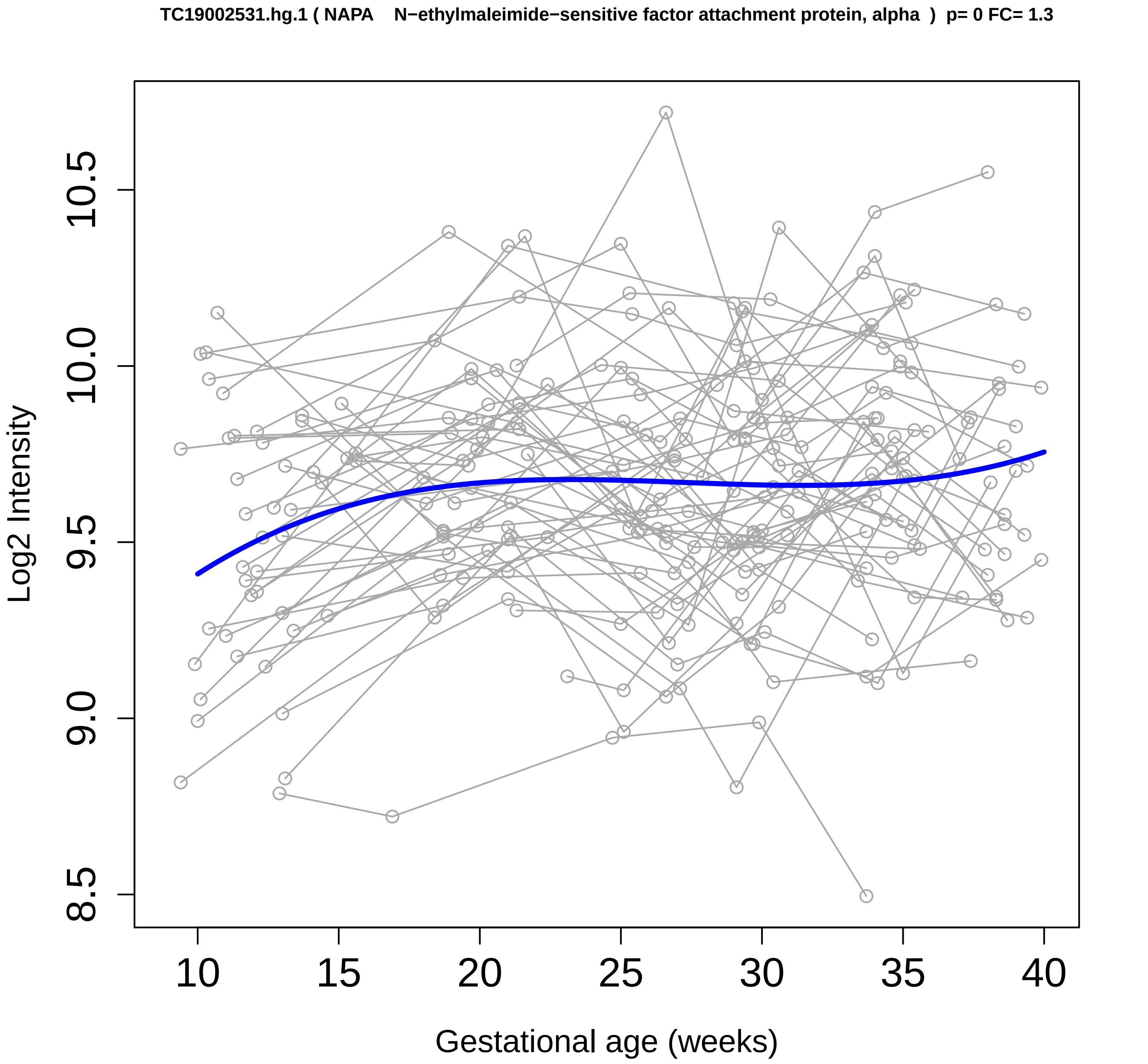

TC19002531.hg.1 (NAPA N−ethylmaleimide−sensitive factor attachment protein, alpha) p= 0 FC= 1.3
Log2 Intensity
8.5
9.0
9.5
10.0
10.5
10
15
20
25
30
35
40
Gestational age (weeks)

TC06002707.hg.1 (HLA−B   major histocompatibility complex, class I, B) p= 0 FC= −1.3

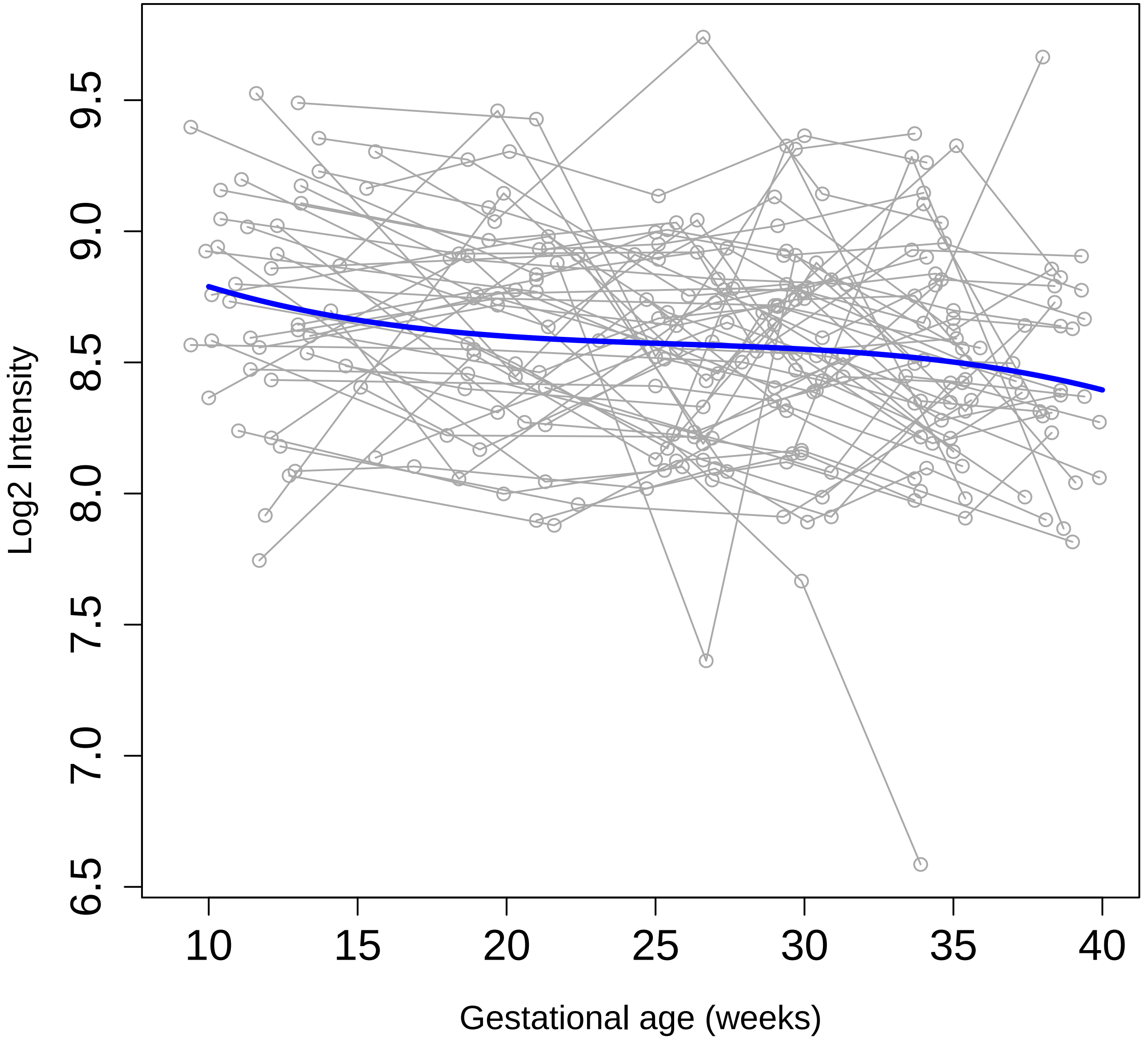

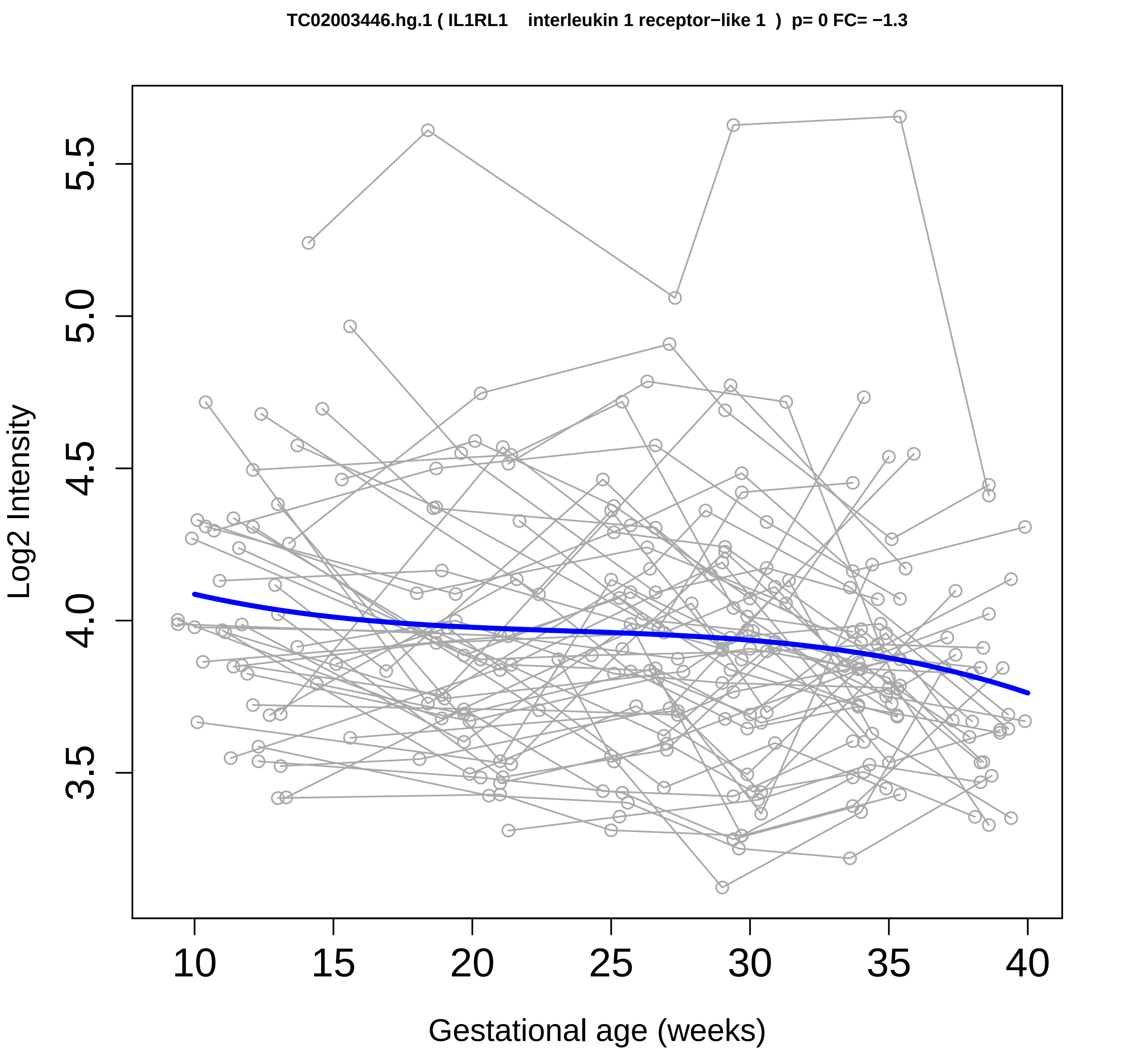
TC02003446.hg.1 (IL1RL1 interleukin 1 receptor−like 1) p= 0 FC= −1.3
Log2 Intensity
3.5
4.0
4.5
5.0
5.5
10
15
20
25
30
35
40
Gestational age (weeks)

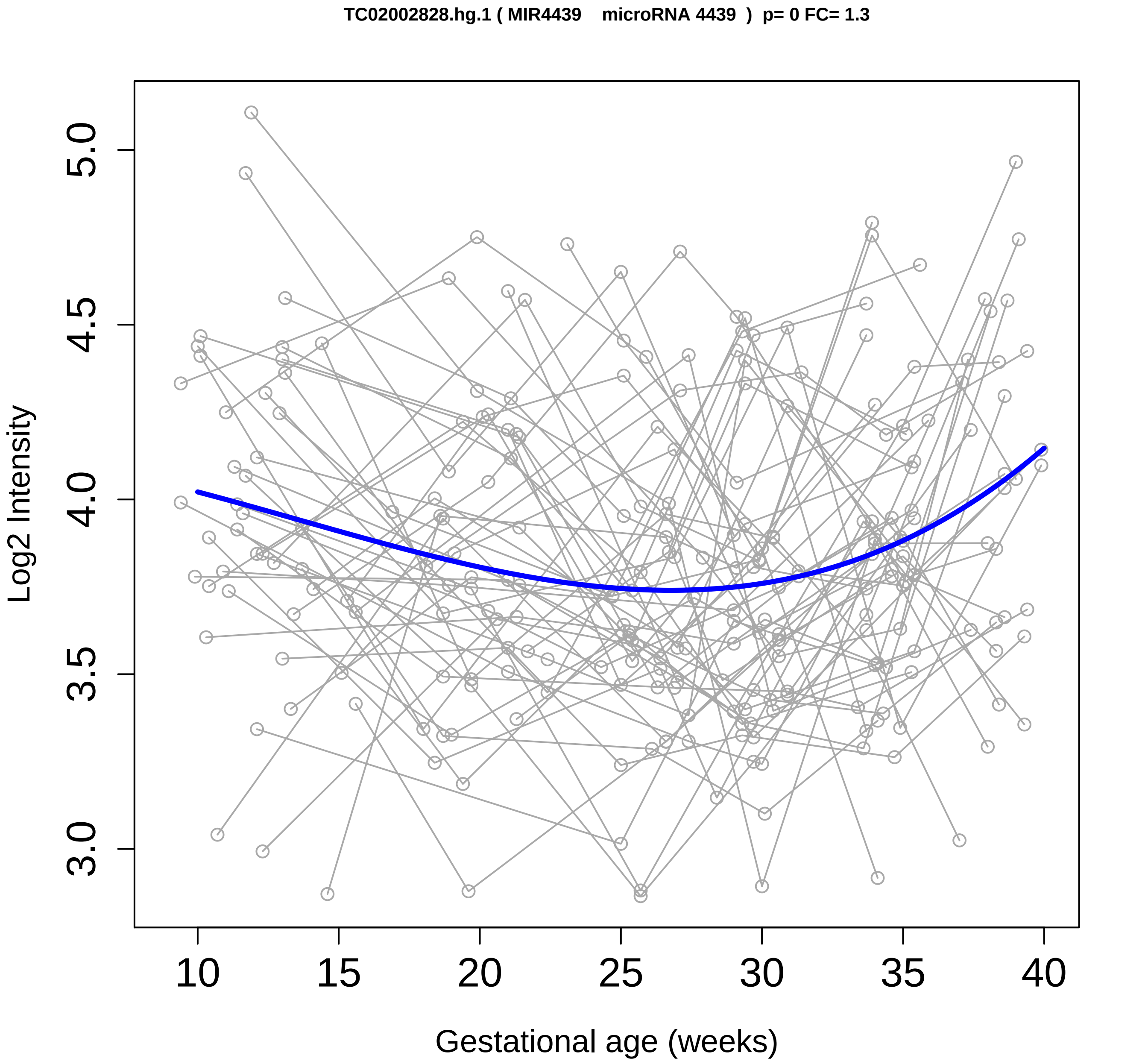

TC02002828.hg.1 (MIR4439 microRNA 4439) p= 0 FC= 1.3
Log2 Intensity
3.0
3.5
4.0
4.5
5.0
10
15
20
25
30
35
40
Gestational age (weeks)

TC01001161.hg.1 (FCGR1C   Fc fragment of IgG, high affinity Ic, receptor (CD64), pseudogene) p= 0 FC= 1.4

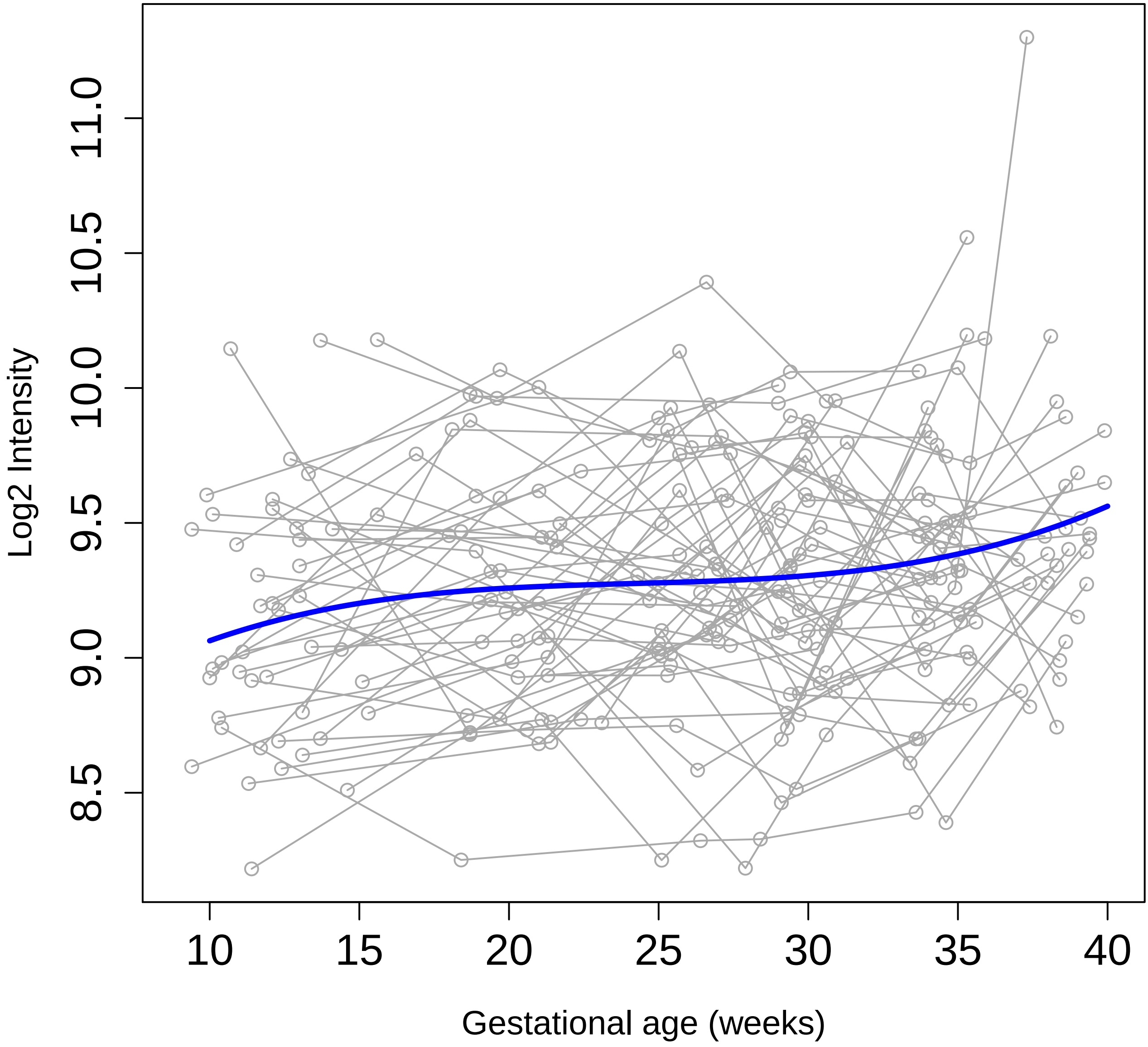

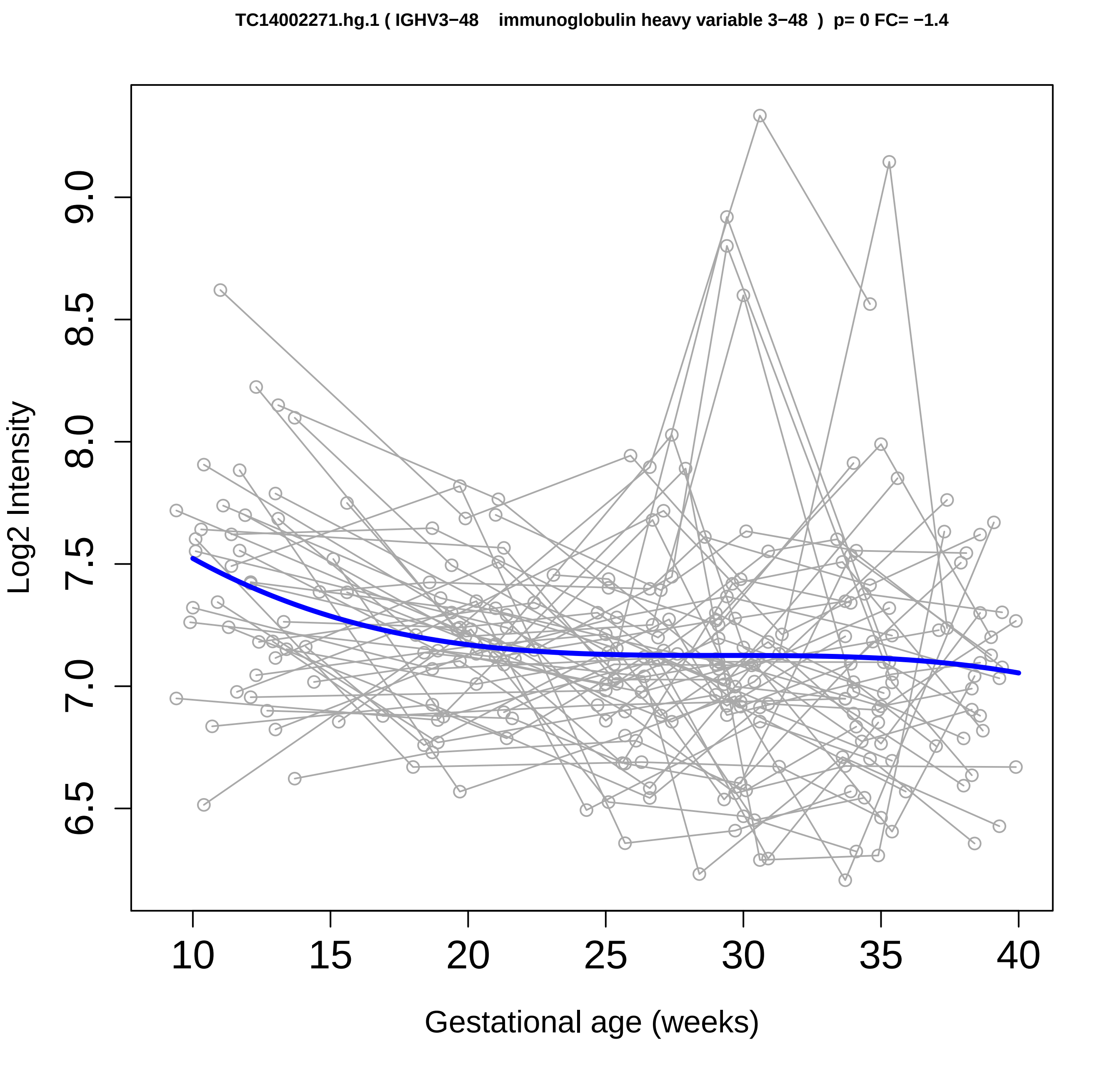
TC14002271.hg.1 (IGHV3−48 immunoglobulin heavy variable 3−48) p= 0 FC= −1.4
Log2 Intensity
6.5
7.0
7.5
8.0
8.5
9.0
10
15
20
25
30
35
40
Gestational age (weeks)

TC14002272.hg.1 (IGHV3−49   immunoglobulin heavy variable 3−49) p= 0 FC= −1.4

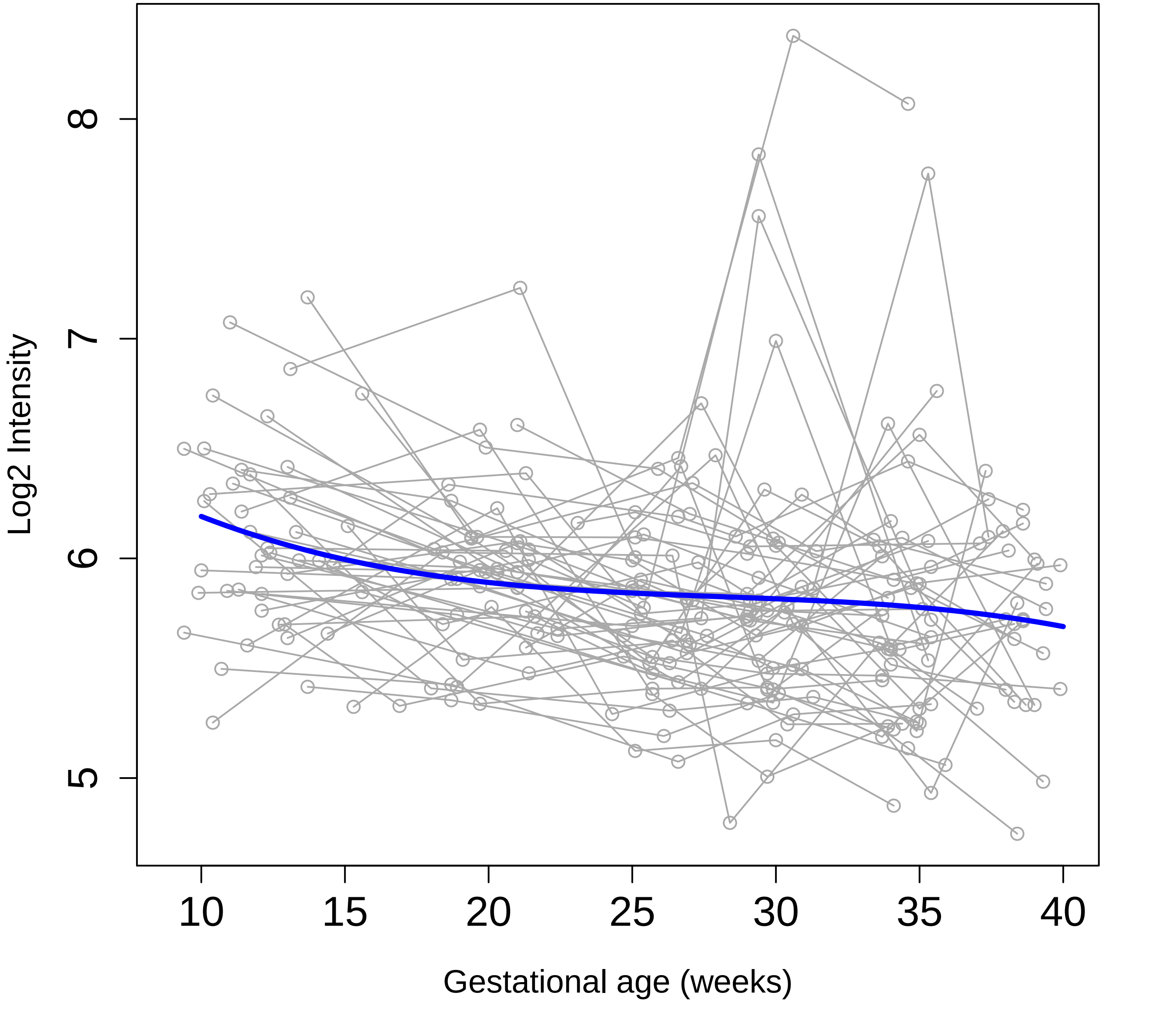

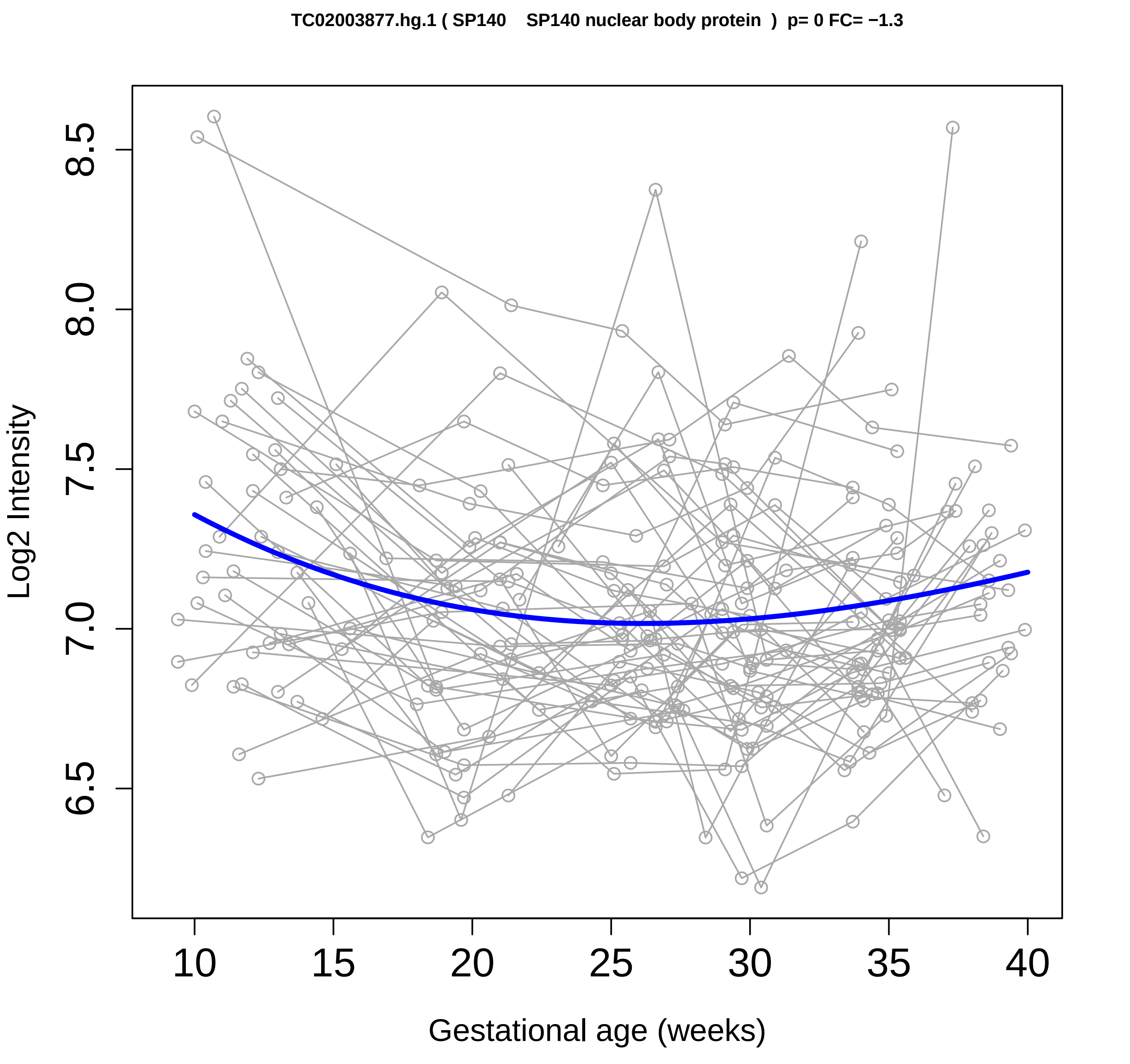

TC02003877.hg.1 (SP140 SP140 nuclear body protein) p= 0 FC= −1.3
Log2 Intensity
6.5
7.0
7.5
8.0
8.5
10
15
20
25
30
35
40
Gestational age (weeks)

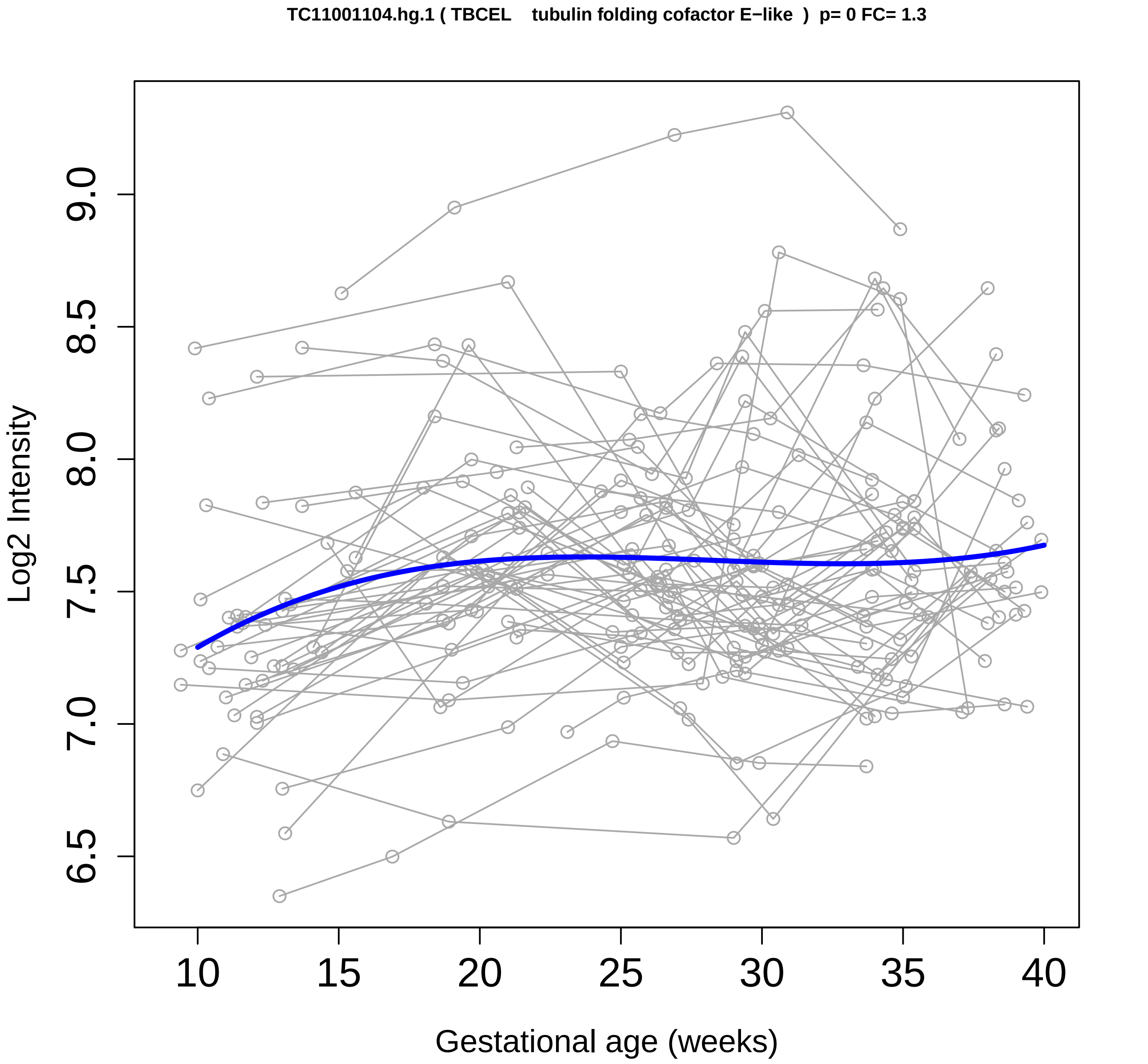
TC11001104.hg.1 (TBCEL tubulin folding cofactor E−like) p= 0 FC= 1.3
Log2 Intensity
9.0
8.5
8.0
7.5
7.0
6.5
10
15
20
25
30
35
40
Gestational age (weeks)

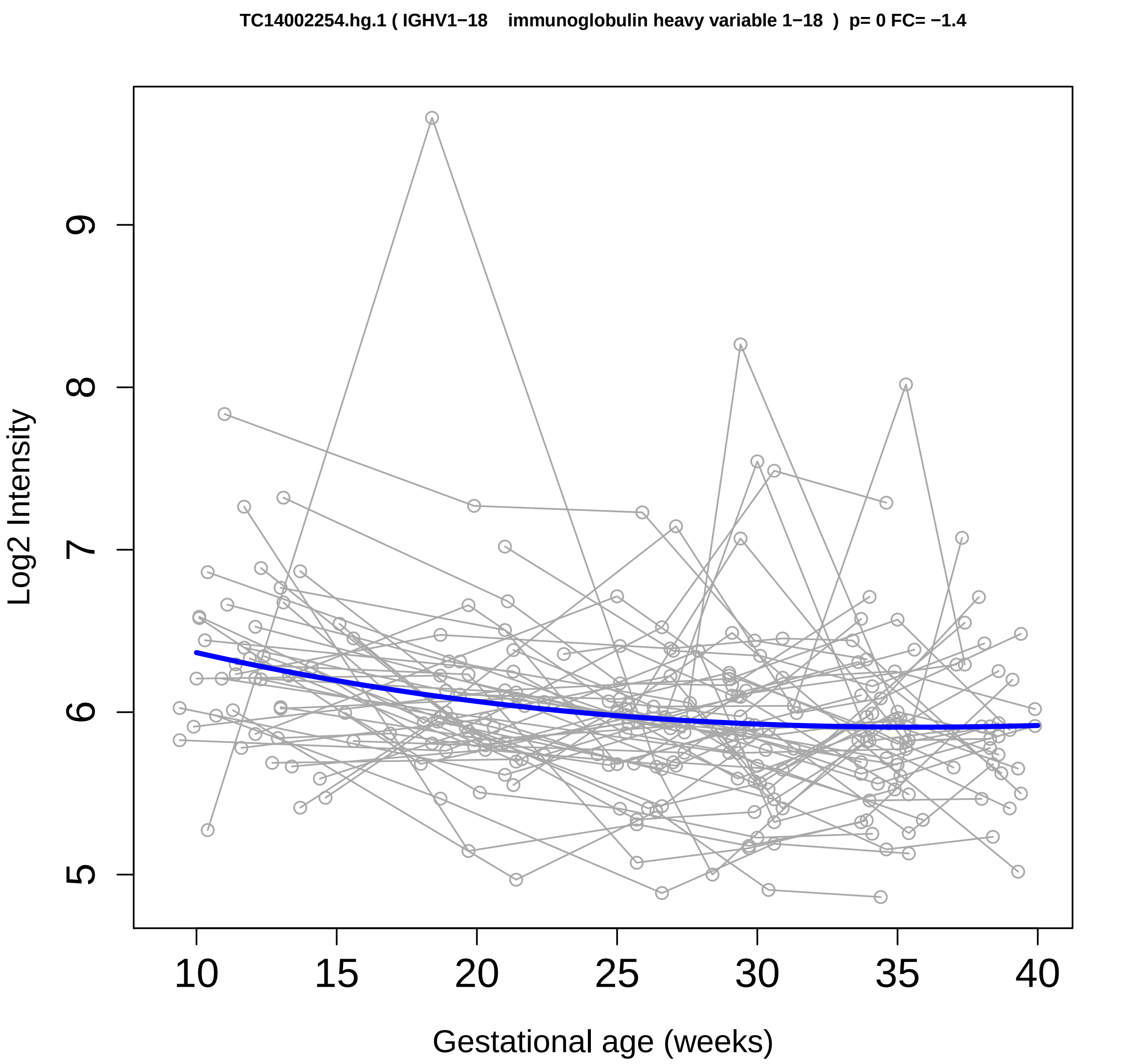

TC14002254.hg.1 (IGHV1−18 immunoglobulin heavy variable 1−18) p= 0 FC= −1.4
Log2 Intensity
5
6
7
8
9
10
15
20
25
30
35
40
Gestational age (weeks)

TC02004975.hg.1 (IGKV1D−37   immunoglobulin kappa variable 1D−37 (non−functional)) p= 0 FC= −1.3

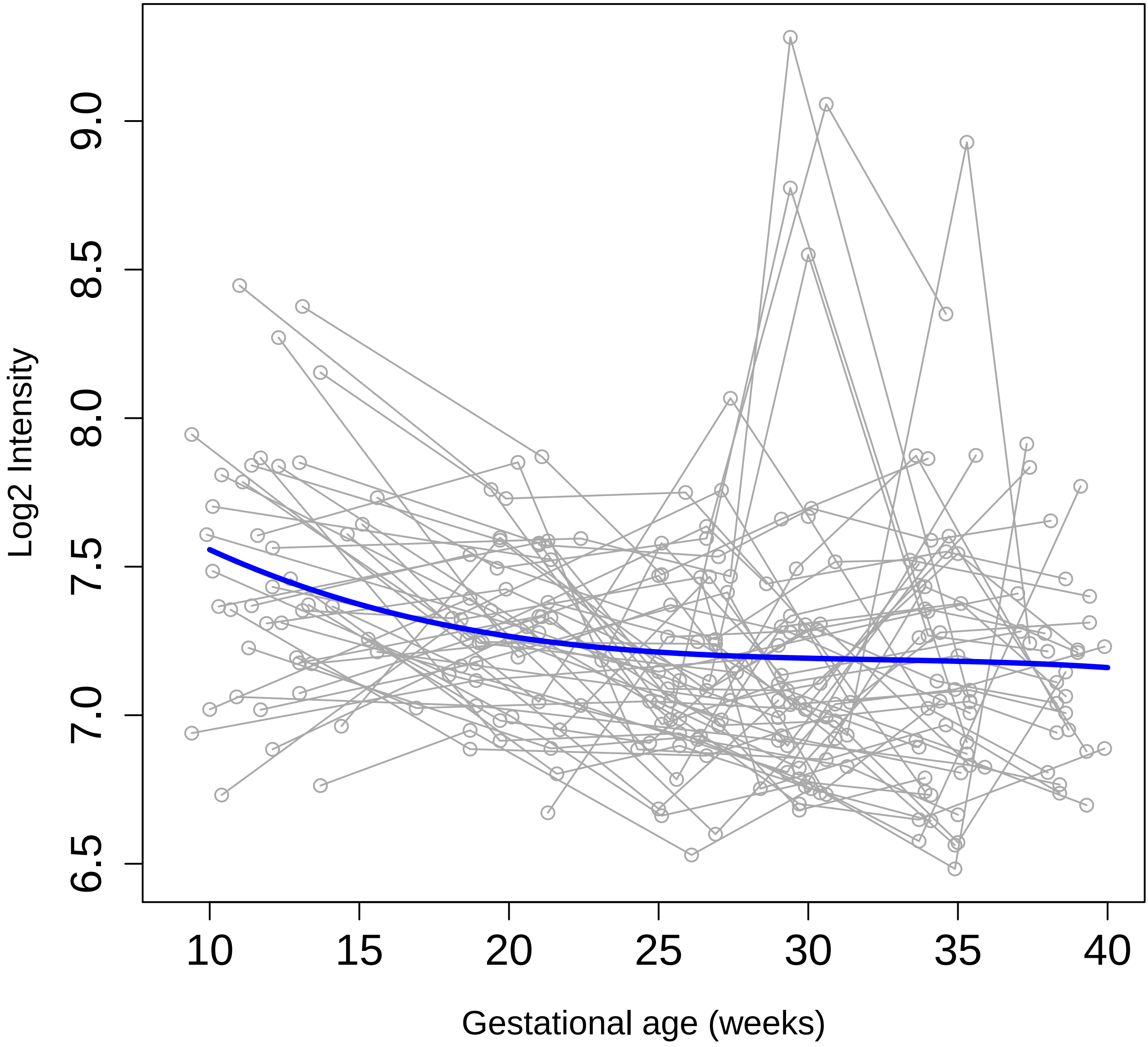

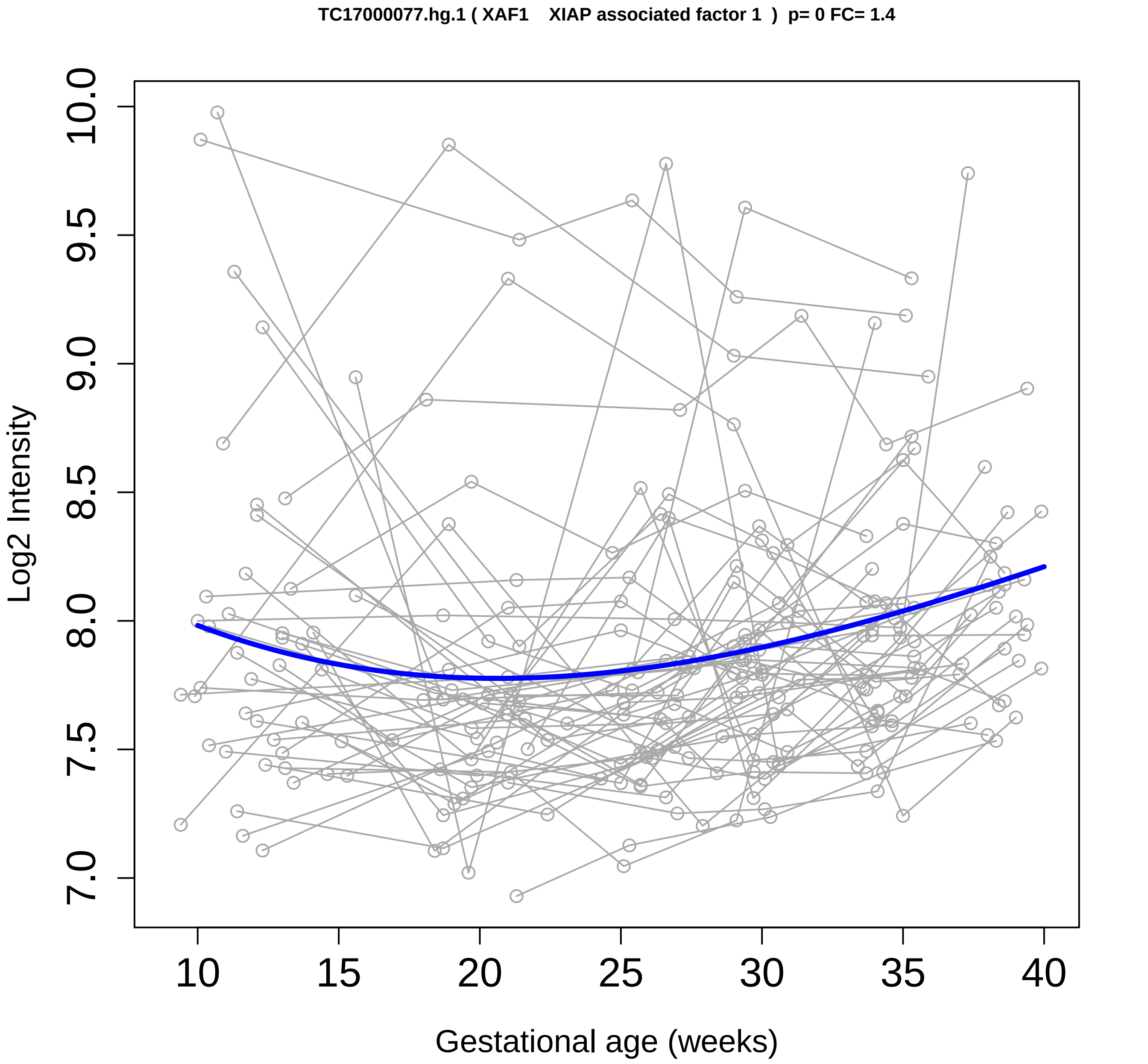

TC17000077.hg.1 (XAF1 XIAP associated factor 1) p= 0 FC= 1.4
Log2 Intensity
7.0
7.5
8.0
8.5
9.0
9.5
10.0
10
15
20
25
30
35
40
Gestational age (weeks)

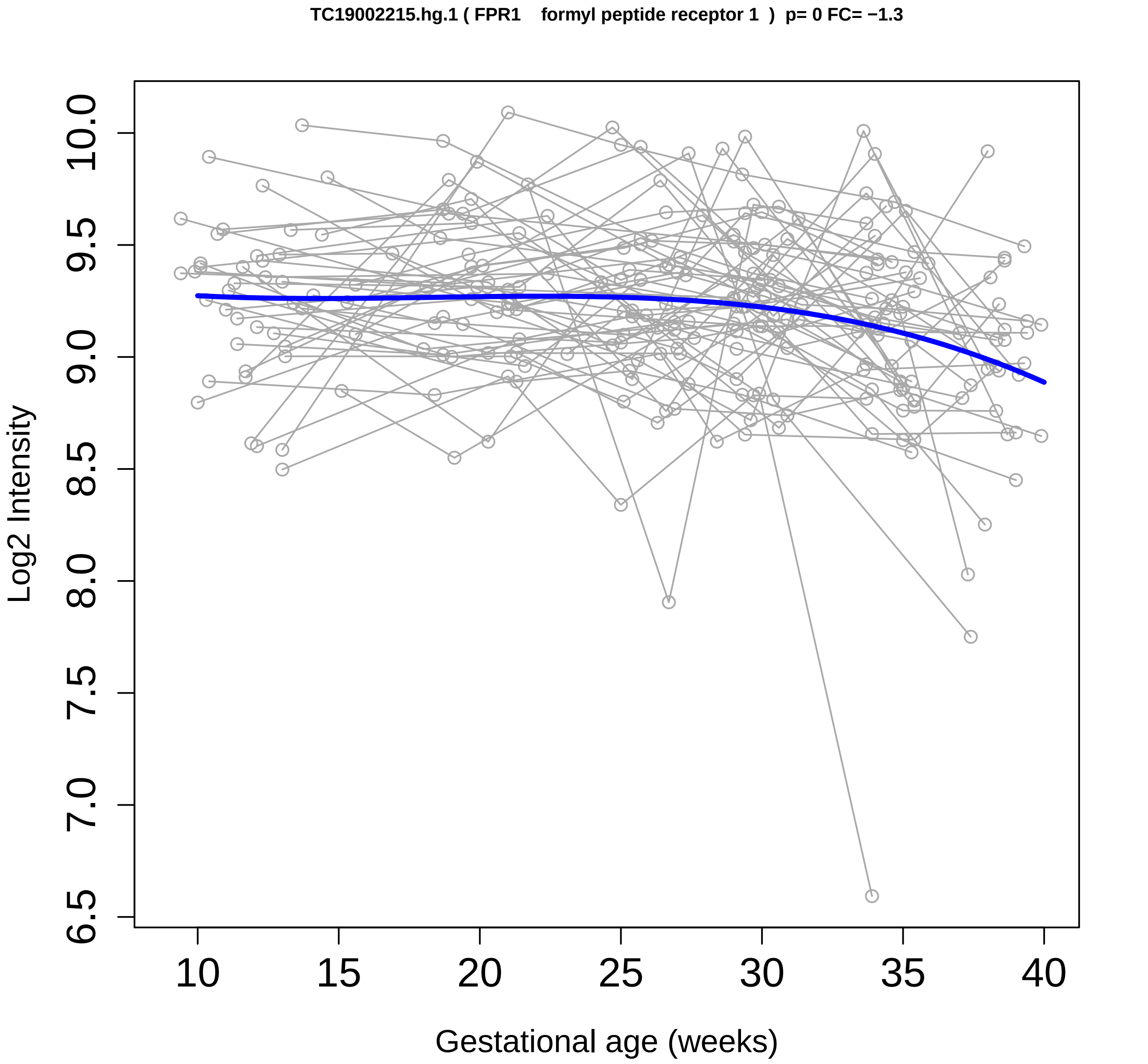

TC19002215.hg.1 (FPR1 formyl peptide receptor 1) p= 0 FC= −1.3
Log2 Intensity
6.5
7.0
7.5
8.0
8.5
9.0
9.5
10.0
10
15
20
25
30
35
40
Gestational age (weeks)

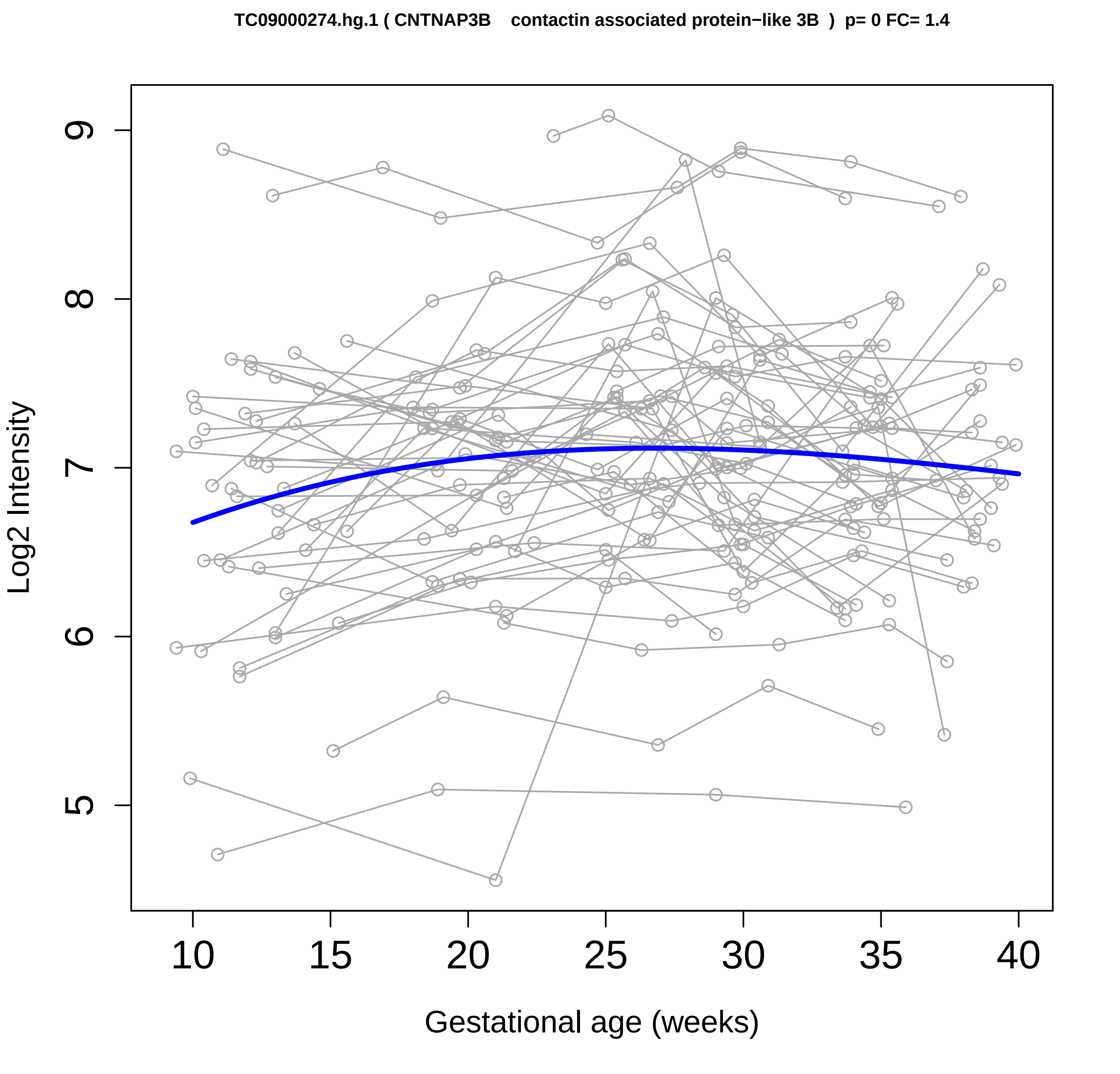
TC09000274.hg.1 (CNTNAP3B contactin associated protein−like 3B) p= 0 FC= 1.4
Log2 Intensity
9
8
7
6
5
10
15
20
25
30
35
40
Gestational age (weeks)

TC05000309.hg.1 (POM121L9P   POM121 transmembrane nucleoporin−like 9, pseudogene) p= 0 FC= 1.4

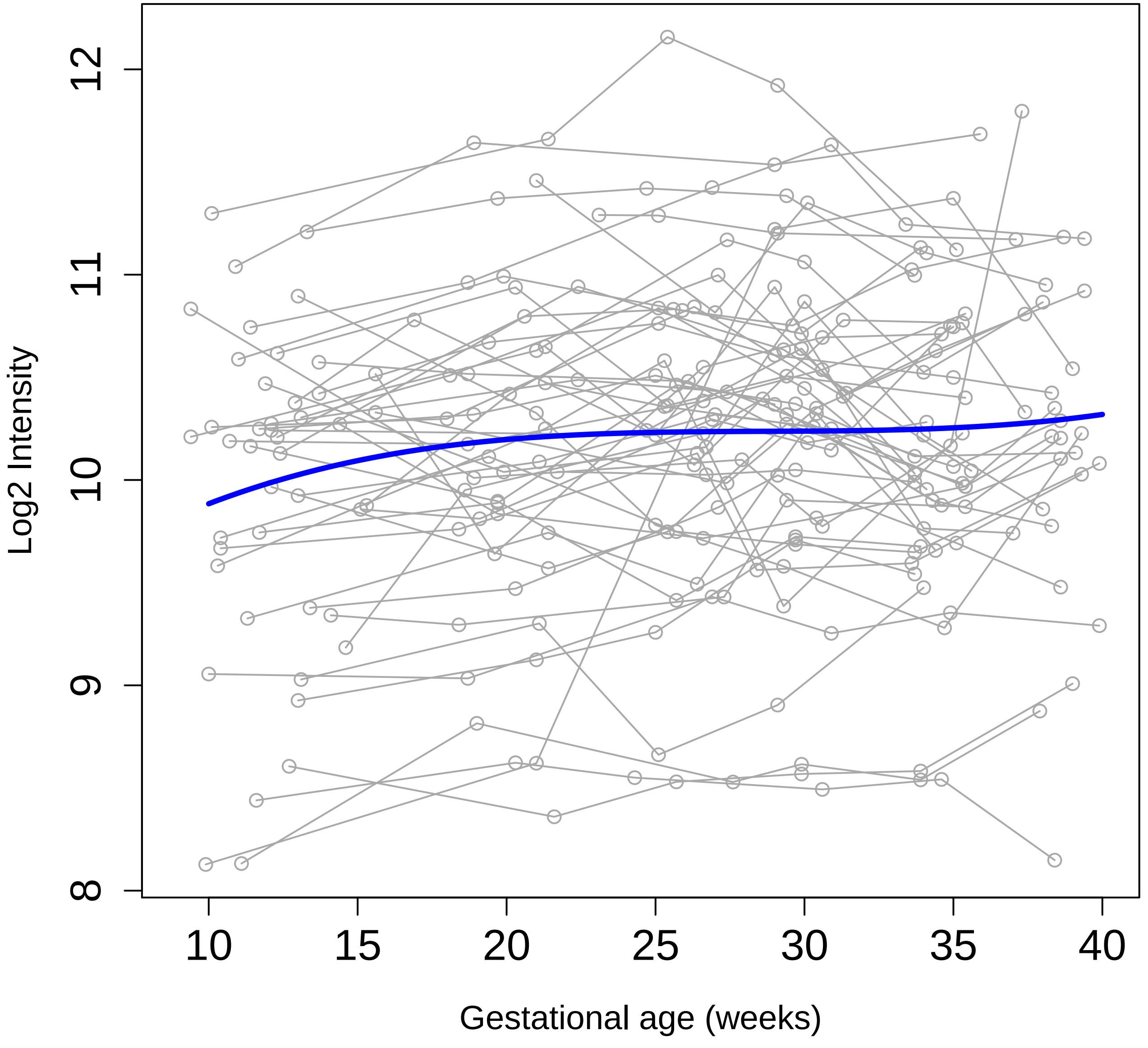

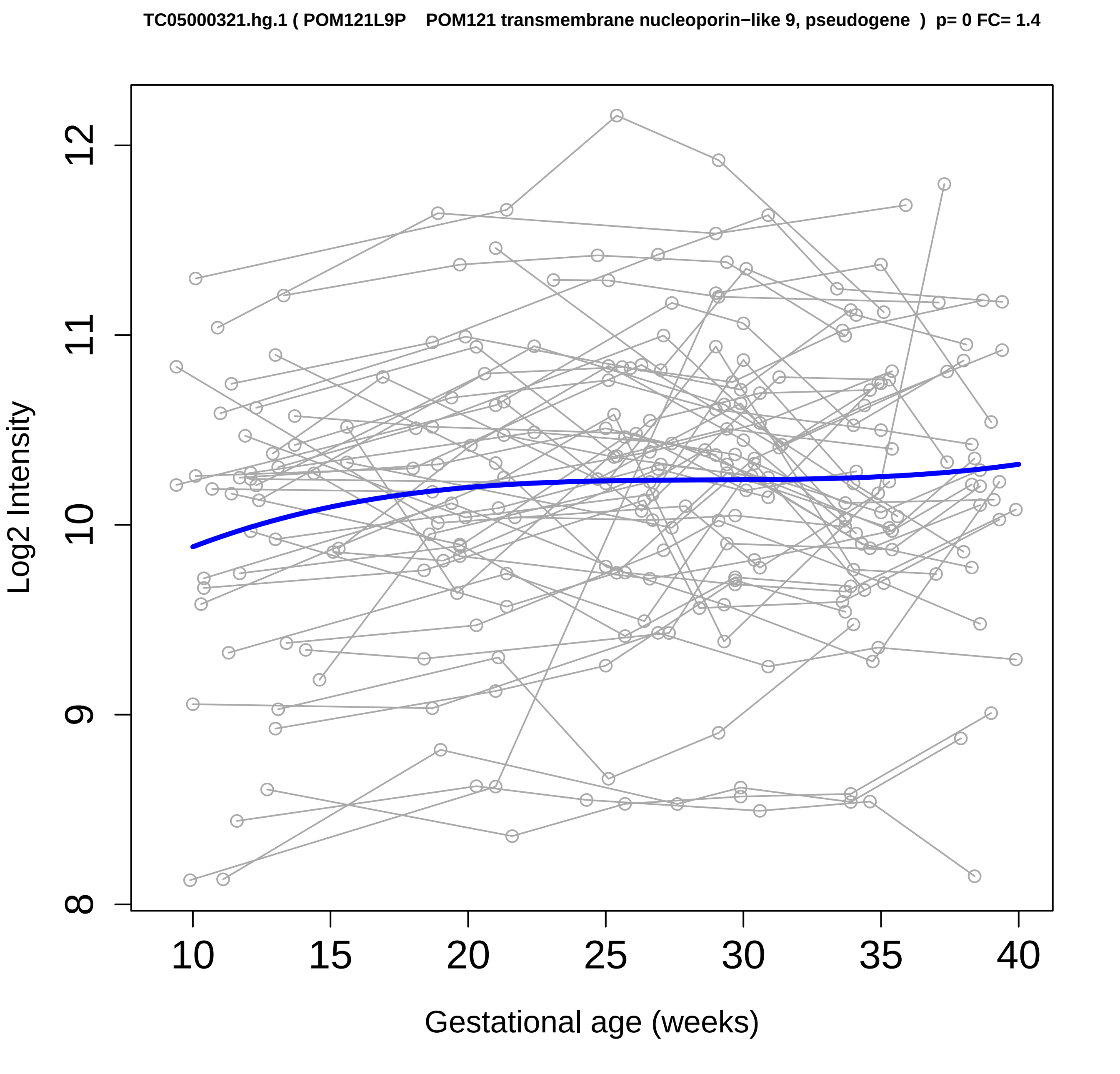
TC05000321.hg.1 (POM121L9P POM121 transmembrane nucleoporin−like 9, pseudogene) p= 0 FC= 1.4
Log2 Intensity
8
9
10
11
12
10
15
20
25
30
35
40
Gestational age (weeks)

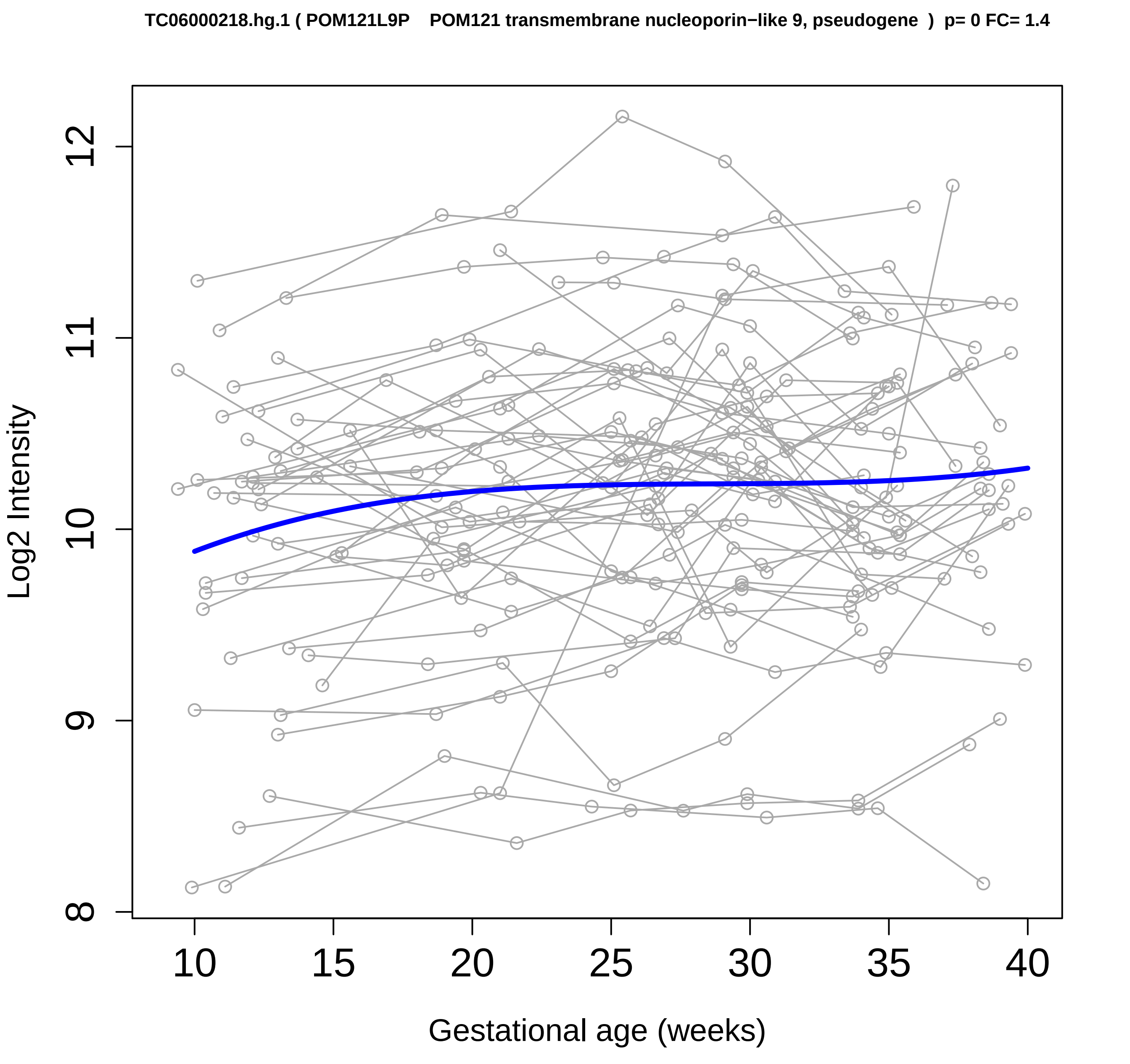
TC06000218.hg.1 (POM121L9P POM121 transmembrane nucleoporin−like 9, pseudogene) p= 0 FC= 1.4
Log2 Intensity
8
9
10
11
12
10
15
20
25
30
35
40
Gestational age (weeks)

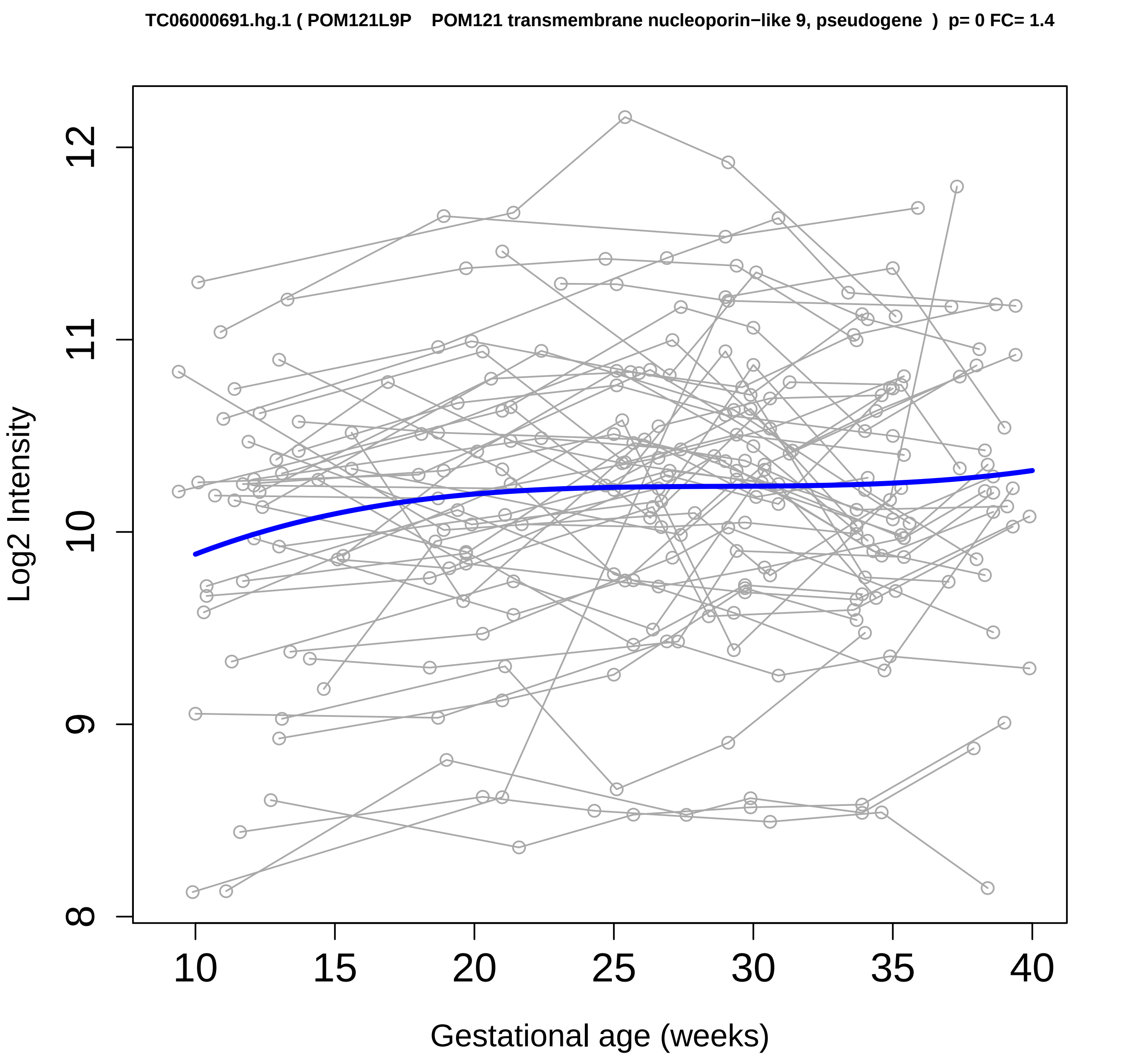

TC06000691.hg.1 (POM121L9P POM121 transmembrane nucleoporin−like 9, pseudogene) p= 0 FC= 1.4
Log2 Intensity
8
9
10
11
12
10
15
20
25
30
35
40
Gestational age (weeks)

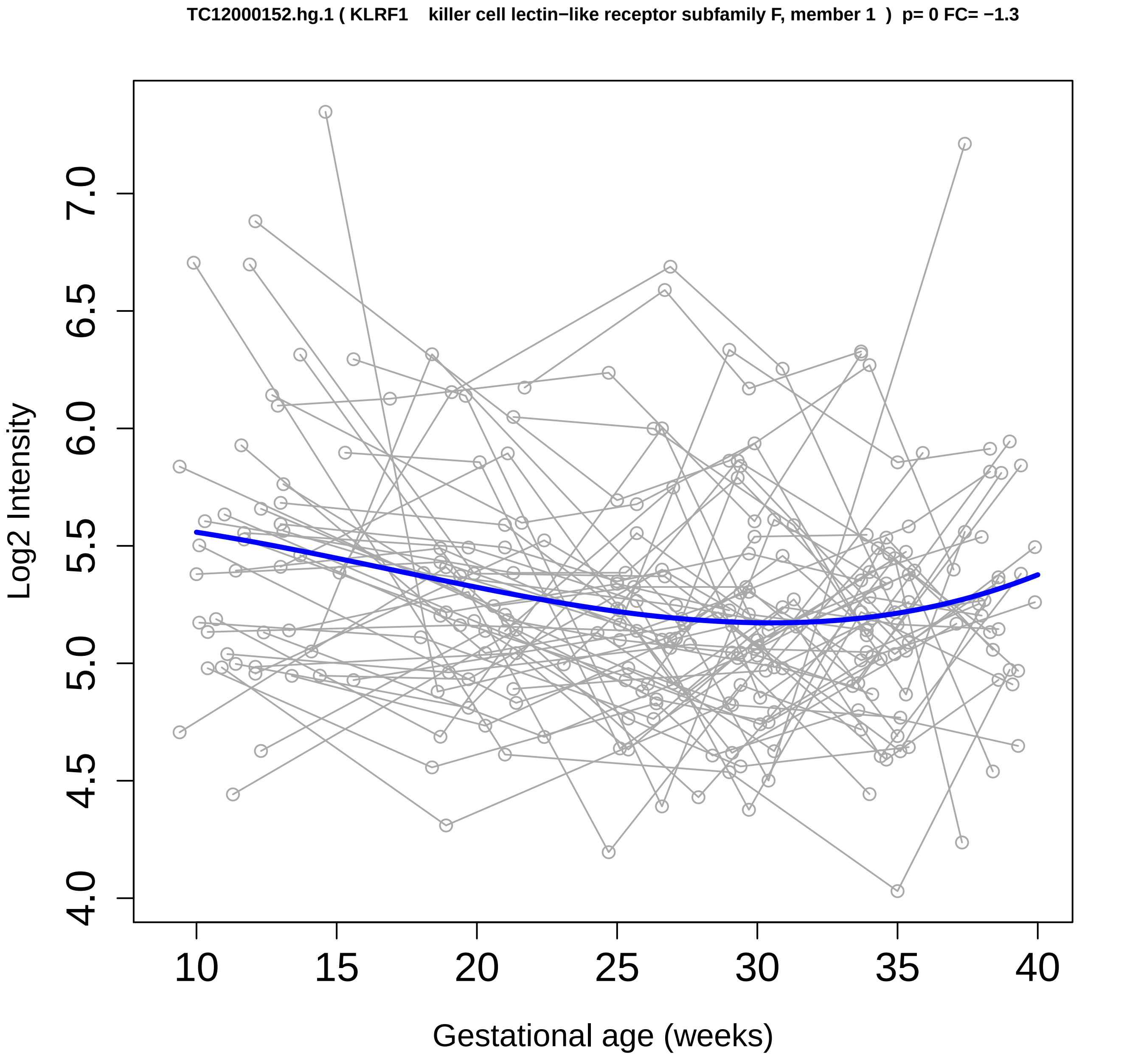
TC12000152.hg.1 (KLRF1 killer cell lectin−like receptor subfamily F, member 1) p= 0 FC= −1.3
Log2 Intensity
4.0
4.5
5.0
5.5
6.0
6.5
7.0
10
15
20
25
30
35
40
Gestational age (weeks)

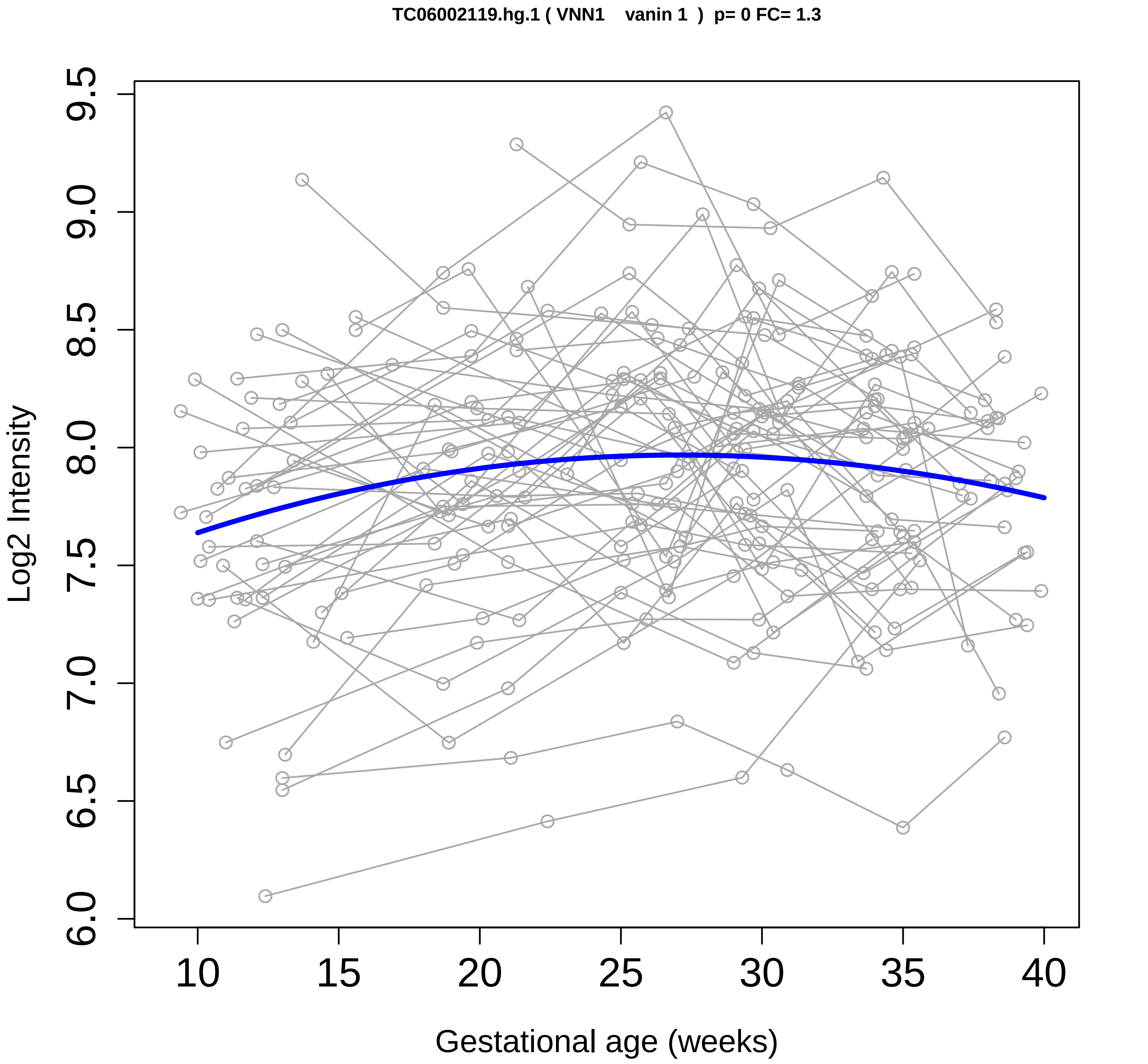
TC06002119.hg.1 (VNN1 vanin 1) p= 0 FC= 1.3
Log2 Intensity
6.0
6.5
7.0
7.5
8.0
8.5
9.0
9.5
10
15
20
25
30
35
40
Gestational age (weeks)

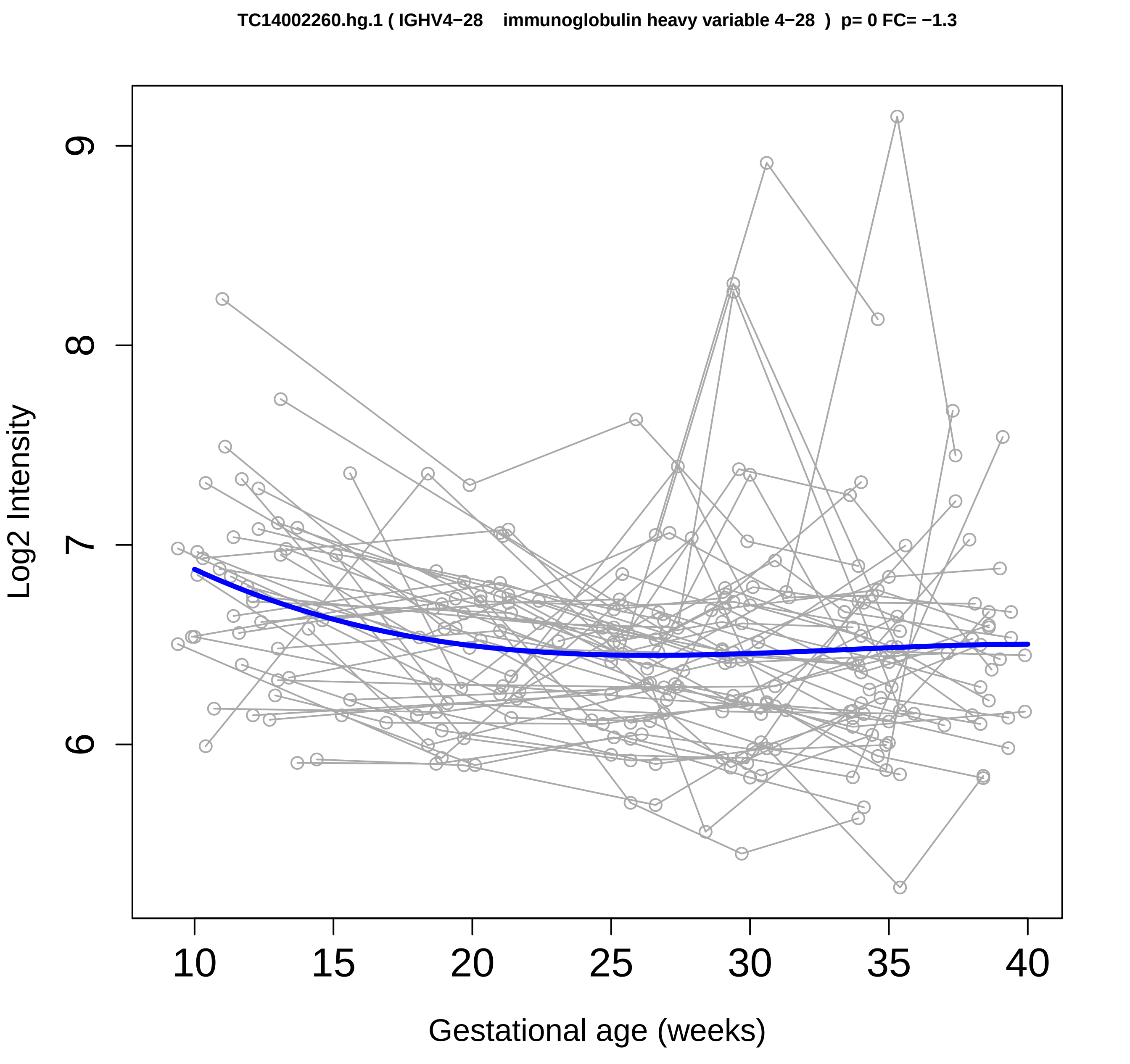

TC14002260.hg.1 (IGHV4−28   immunoglobulin heavy variable 4−28) p= 0 FC= −1.3
Log2 Intensity
9
8
7
6
10
15
20
25
30
35
40
Gestational age (weeks)

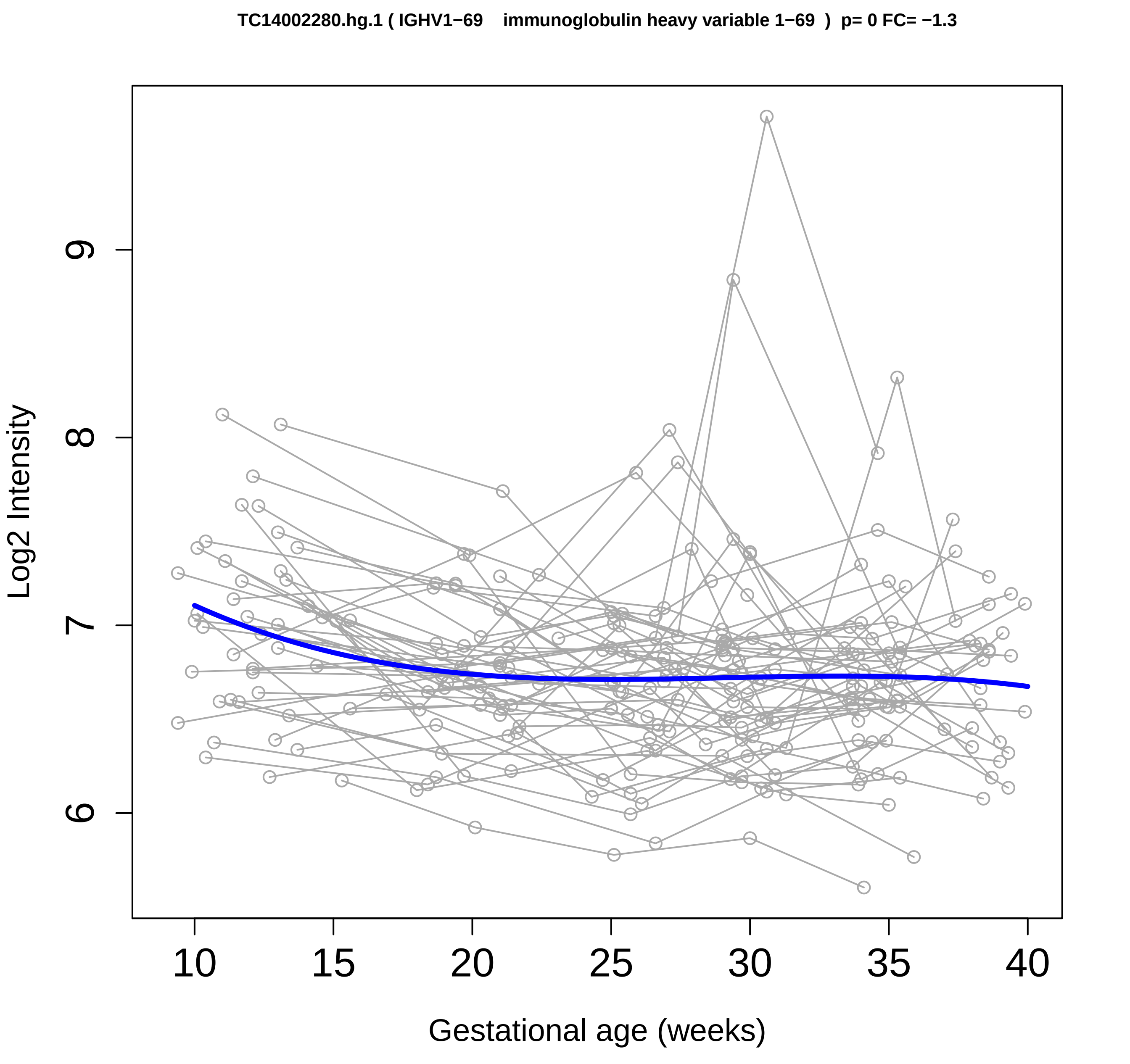
TC14002280.hg.1 (IGHV1−69 immunoglobulin heavy variable 1−69) p= 0 FC= −1.3
Log2 Intensity
6
7
8
9
10
15
20
25
30
35
40
Gestational age (weeks)

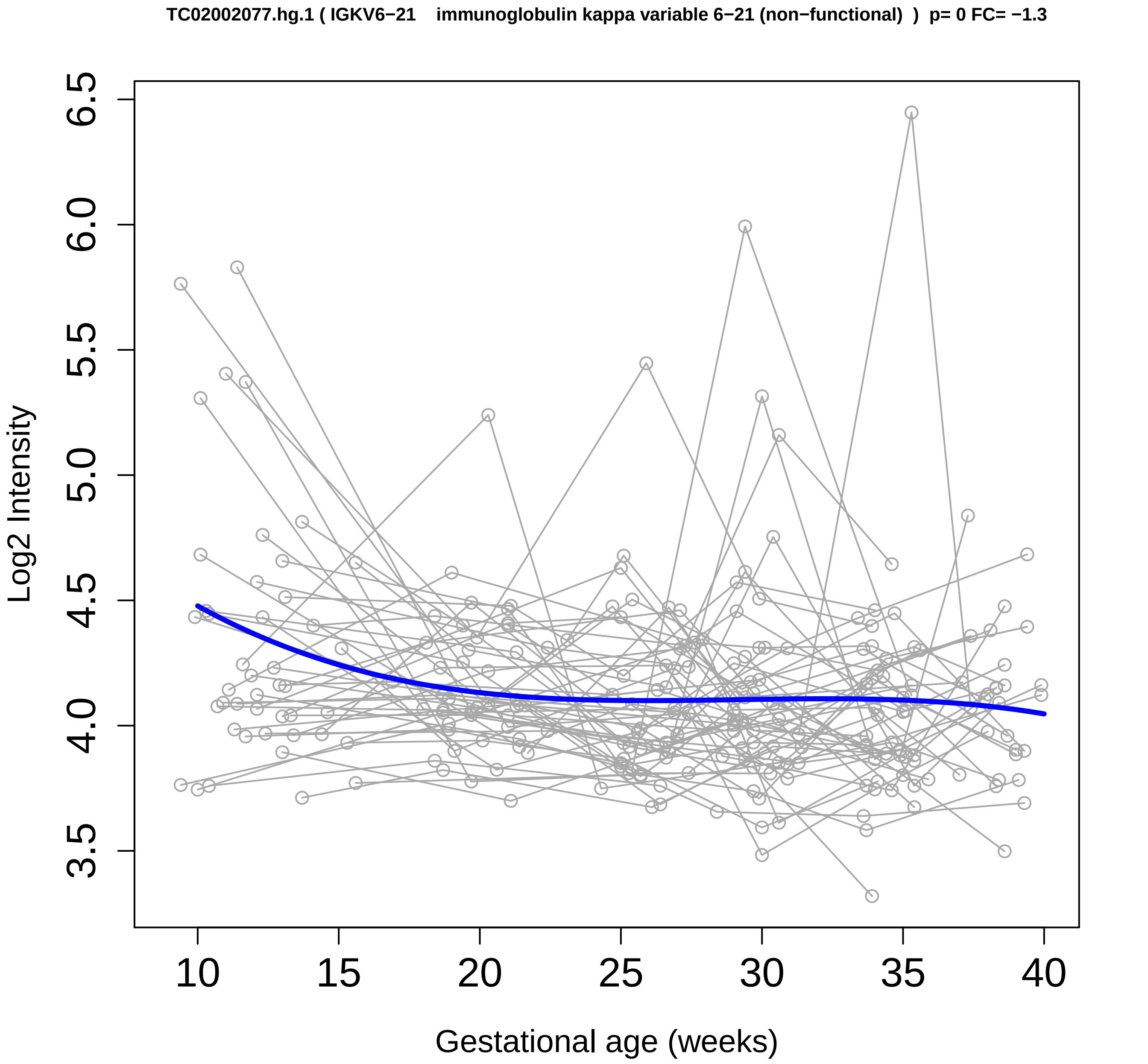
TC02002077.hg.1 (IGKV6−21 immunoglobulin kappa variable 6−21 (non−functional)) p= 0 FC= −1.3
Log2 Intensity
3.5
4.0
4.5
5.0
5.5
6.0
6.5
10
15
20
25
30
35
40
Gestational age (weeks)

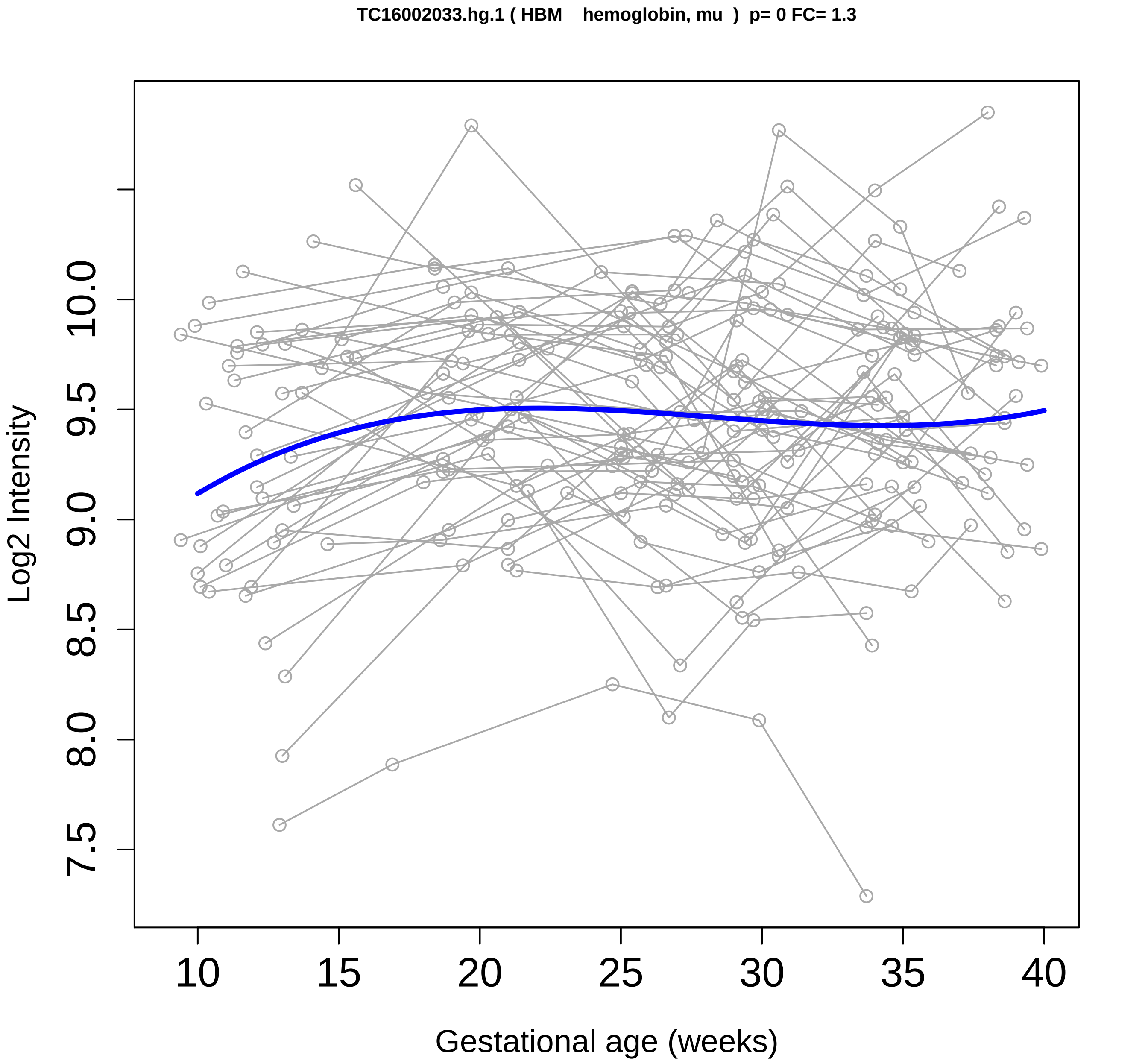
TC16002033.hg.1 (HBM hemoglobin, mu) p= 0 FC= 1.3
Log2 Intensity
7.5
8.0
8.5
9.0
9.5
10.0
10
15
20
25
30
35
40
Gestational age (weeks)

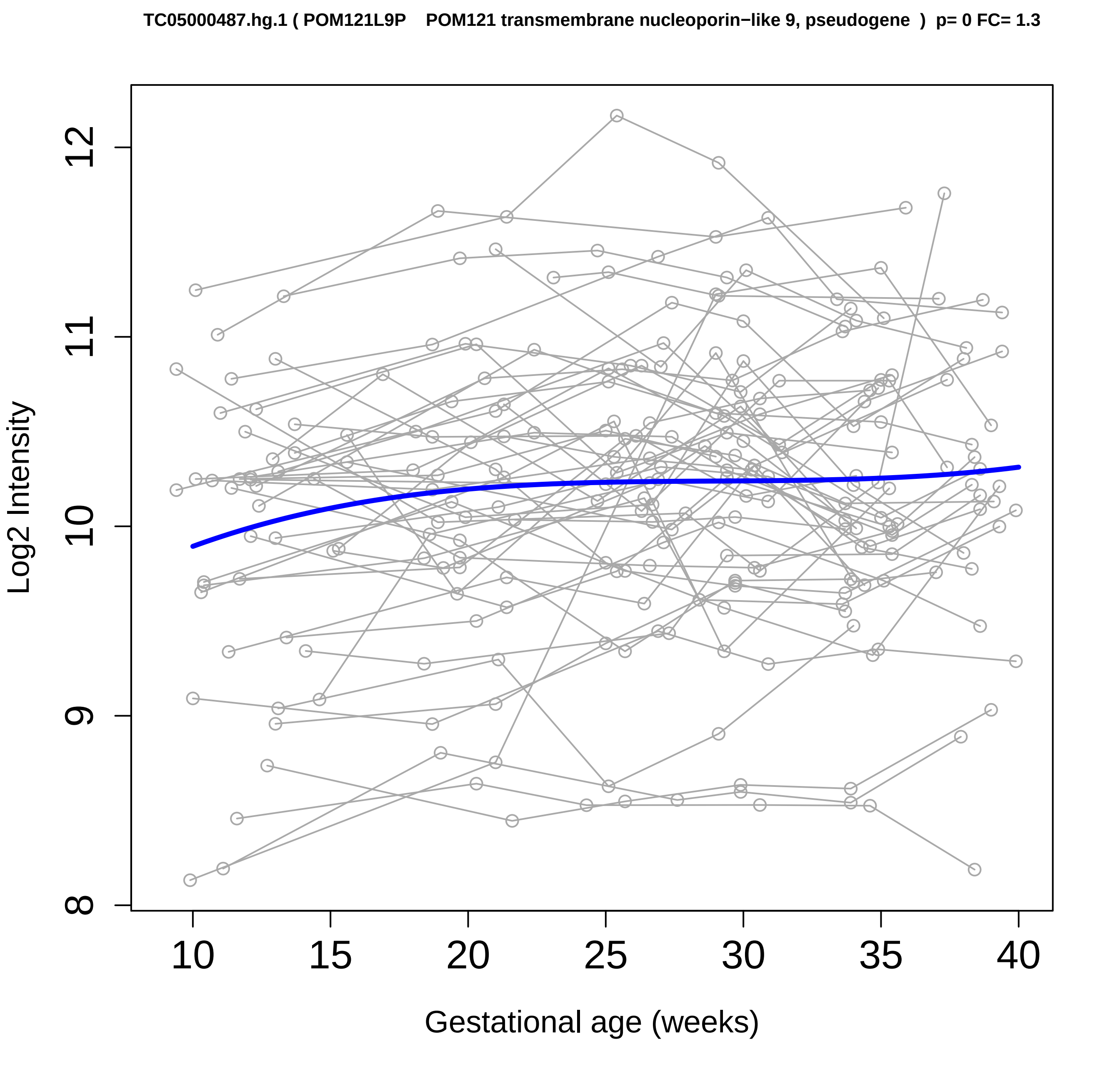
TC05000487.hg.1 (POM121L9P POM121 transmembrane nucleoporin−like 9, pseudogene) p= 0 FC= 1.3
Log2 Intensity
8
9
10
11
12
10
15
20
25
30
35
40
Gestational age (weeks)

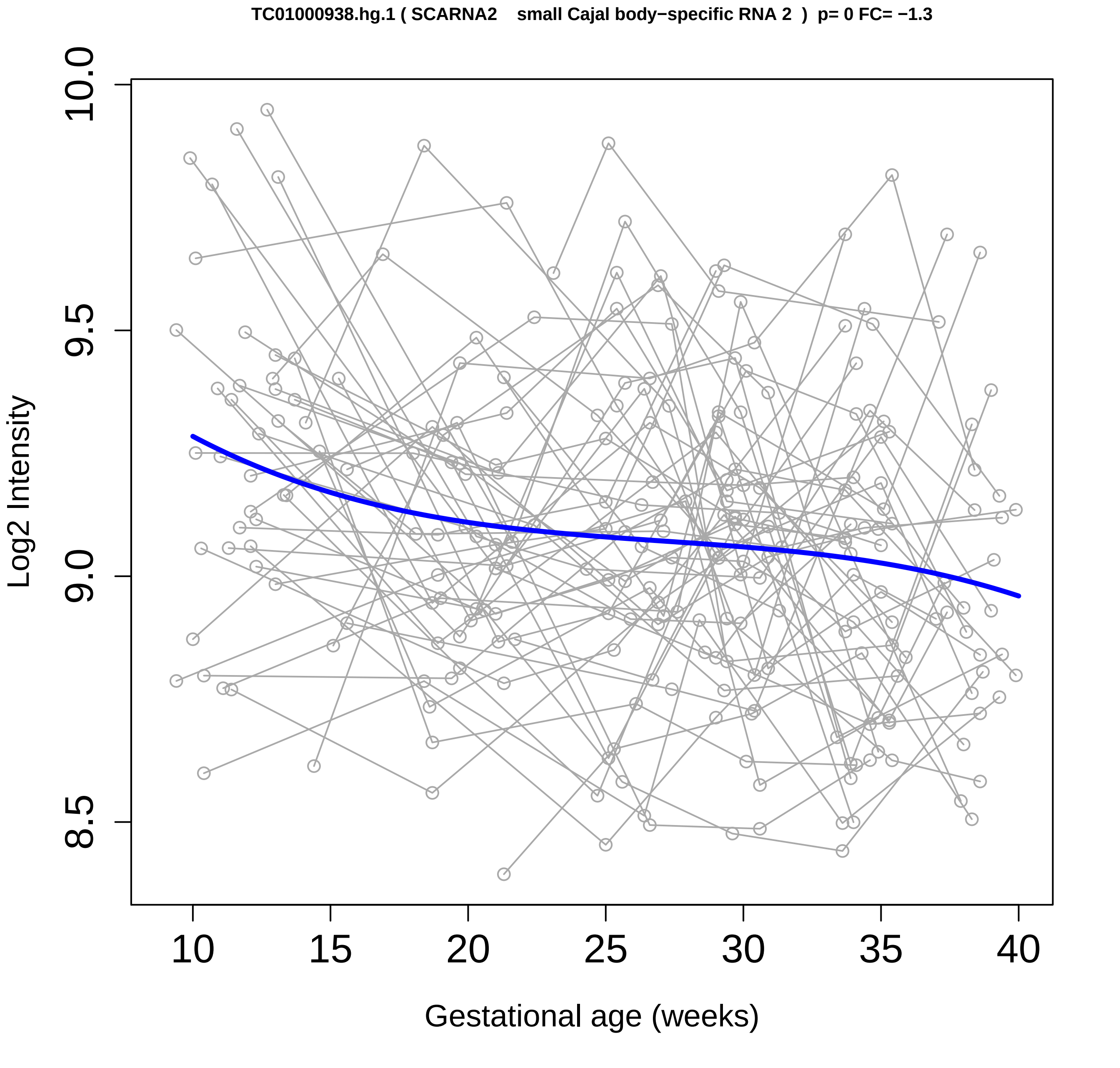

TC01000938.hg.1 (SCARNA2 small Cajal body−specific RNA 2) p= 0 FC= −1.3
Log2 Intensity
10.0
9.5
9.0
8.5
10
15
20
25
30
35
40
Gestational age (weeks)

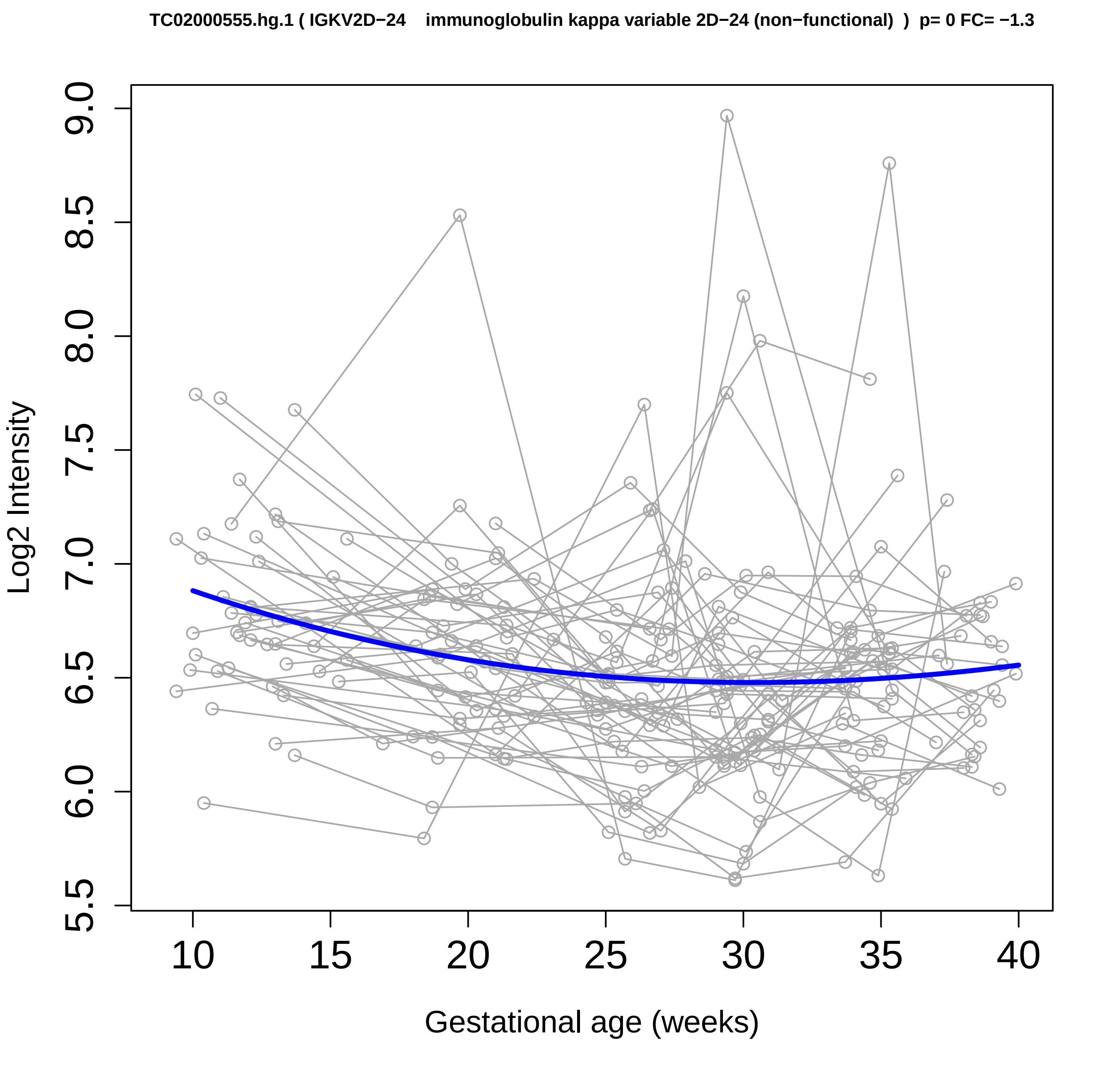

TC02000555.hg.1 (IGKV2D−24   immunoglobulin kappa variable 2D−24 (non−functional)) p= 0 FC= −1.3
Log2 Intensity
5.5
6.0
6.5
7.0
7.5
8.0
8.5
9.0
10
15
20
25
30
35
40
Gestational age (weeks)

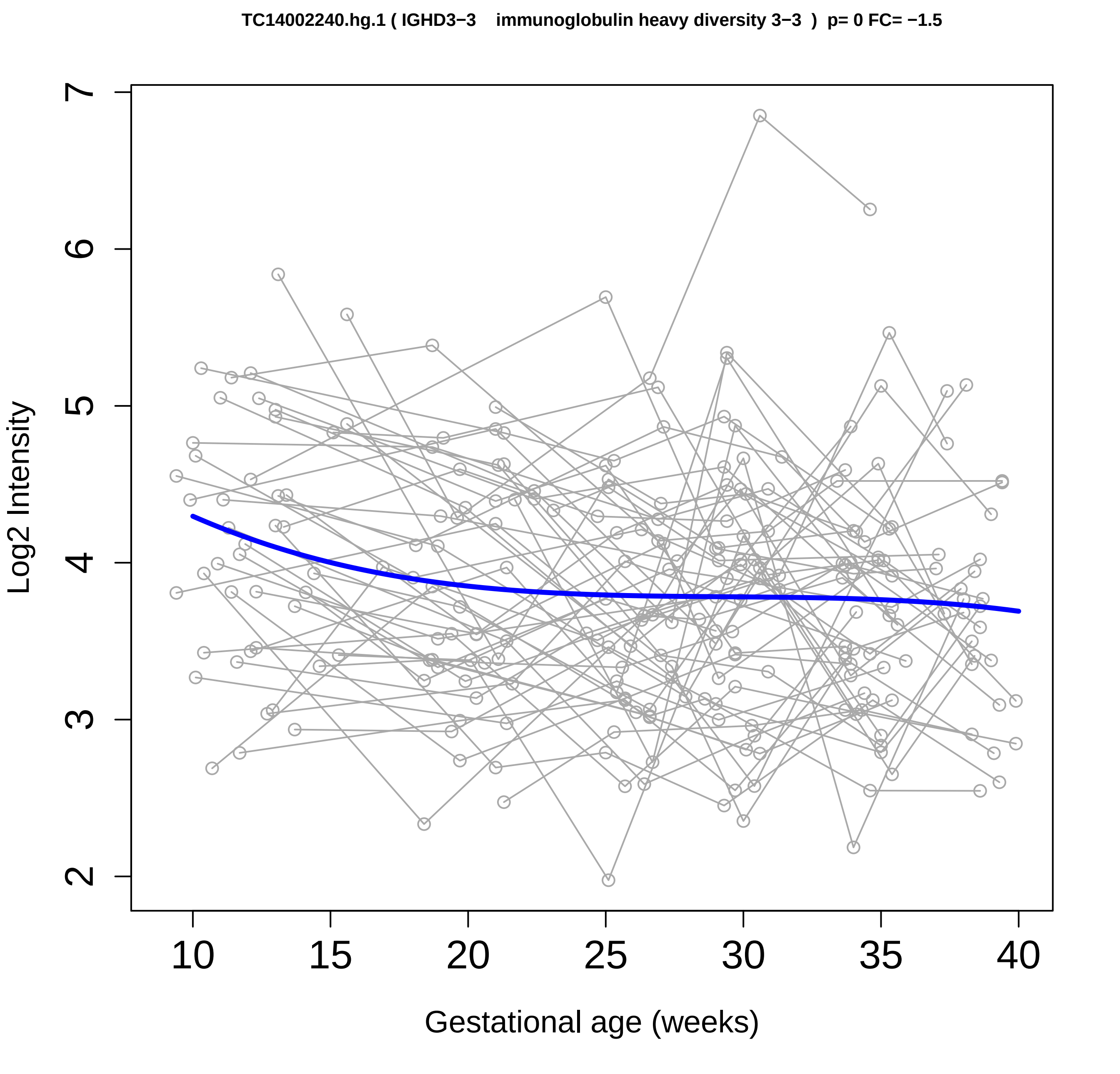

TC14002240.hg.1 (IGHD3−3   immunoglobulin heavy diversity 3−3) p= 0 FC= −1.5
Log2 Intensity
2
3
4
5
6
7
10
15
20
25
30
35
40
Gestational age (weeks)

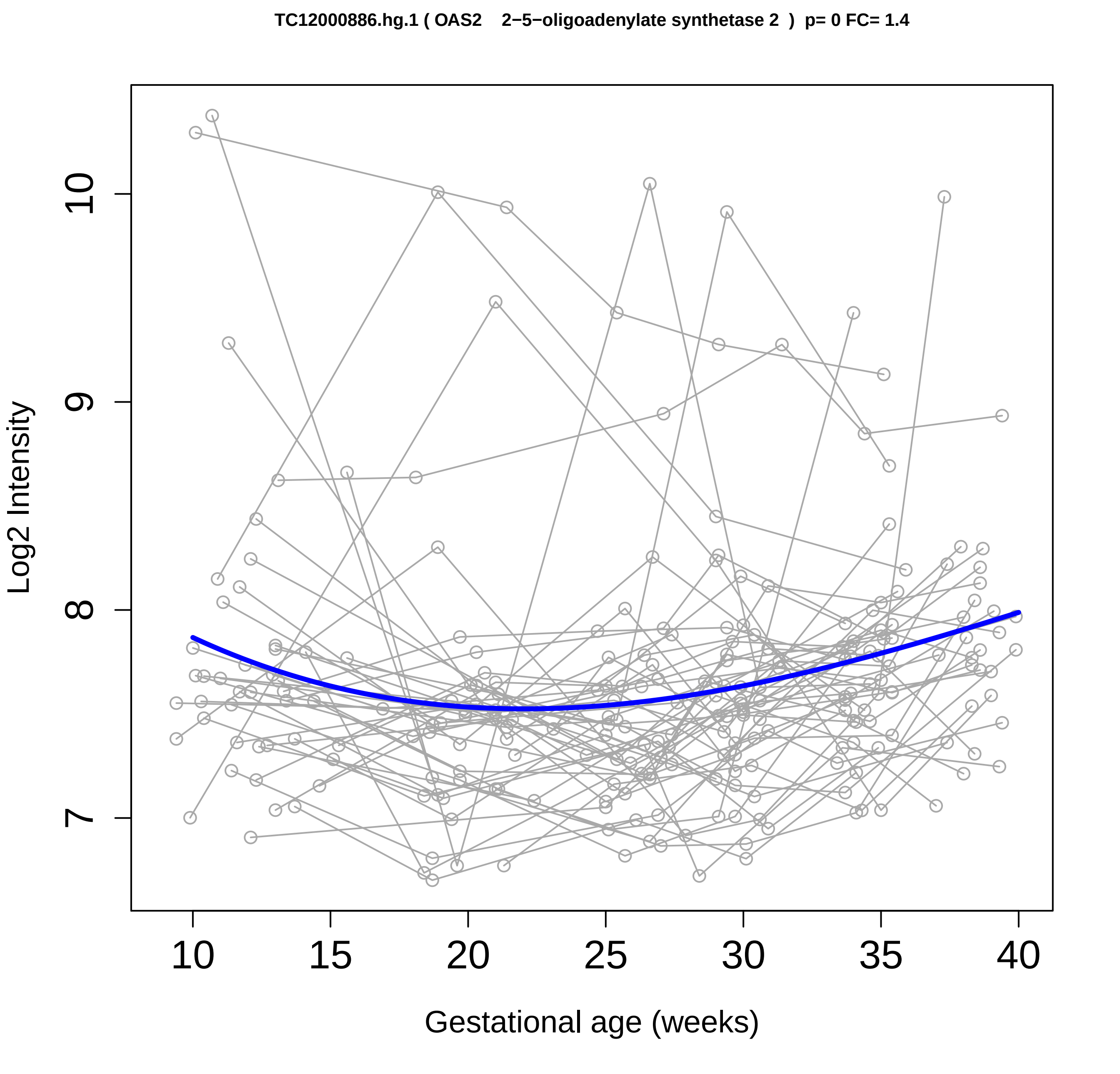
TC12000886.hg.1 (OAS2 2−5−oligoadenylate synthetase 2) p= 0 FC= 1.4
Log2 Intensity
7
8
9
10
10
15
20
25
30
35
40
Gestational age (weeks)

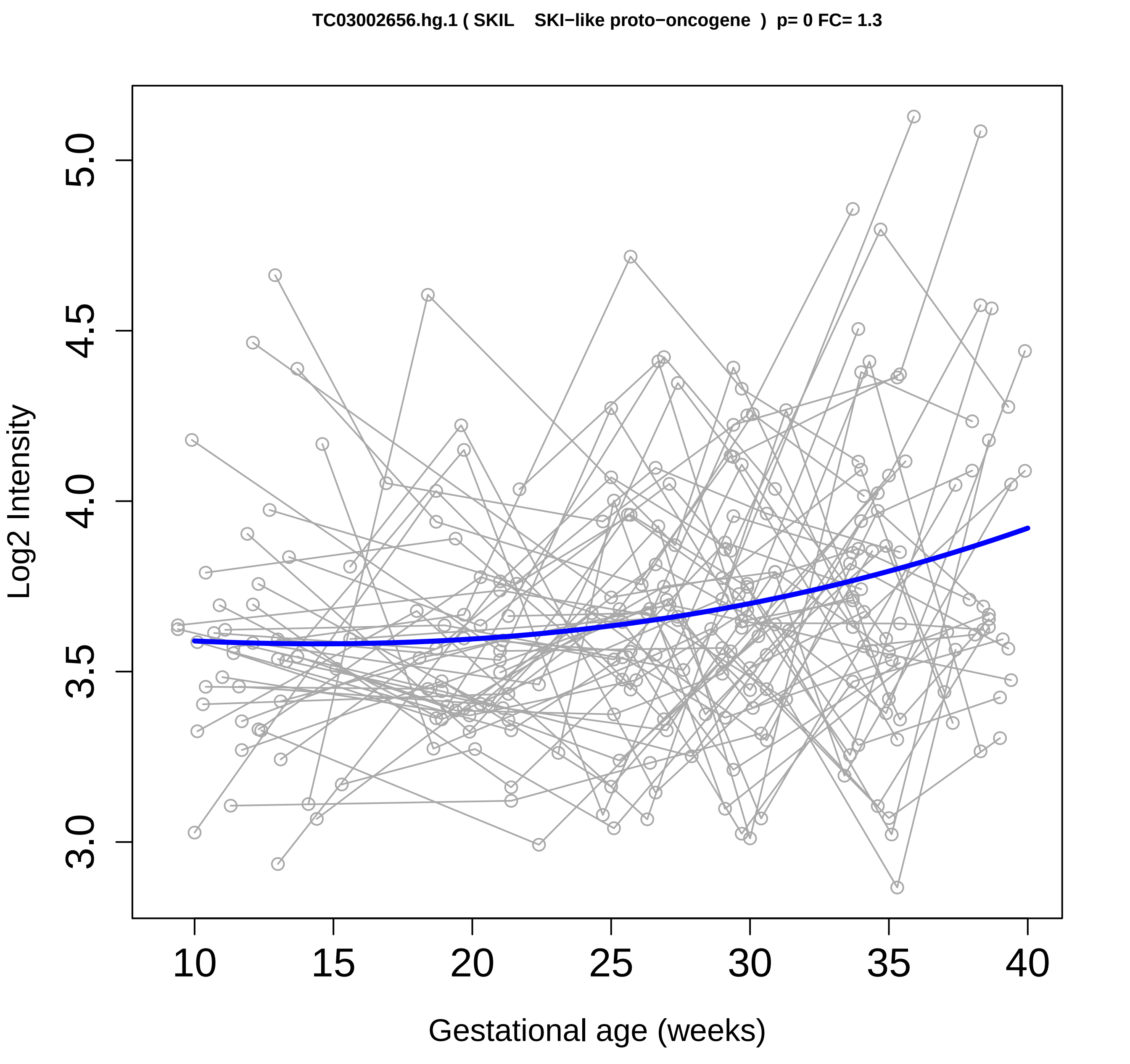
TC03002656.hg.1 (SKIL   SKI−like proto−oncogene) p= 0 FC= 1.3
Log2 Intensity
3.0
3.5
4.0
4.5
5.0
10
15
20
25
30
35
40
Gestational age (weeks)

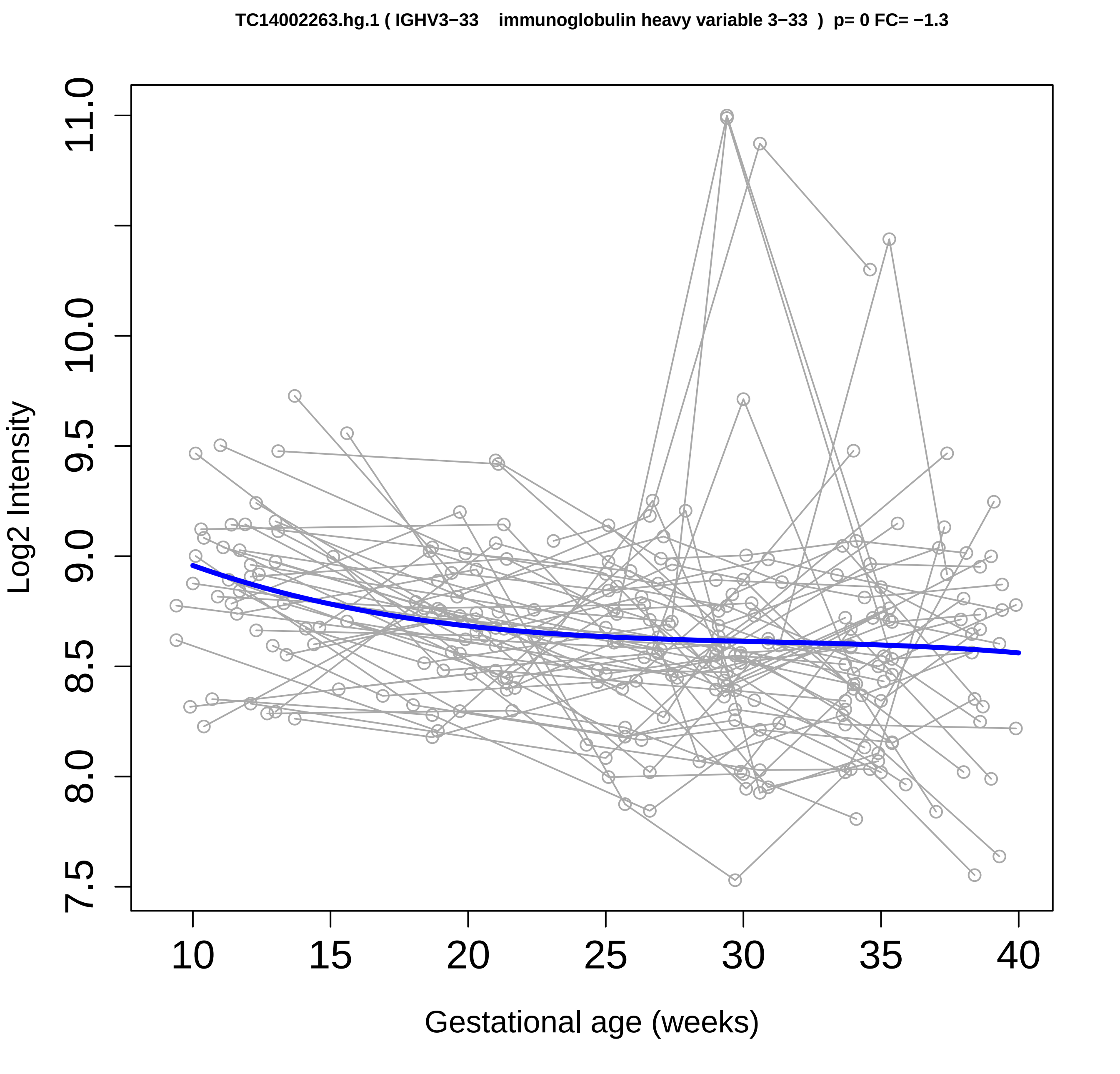

TC14002263.hg.1 (IGHV3−33 immunoglobulin heavy variable 3−33) p= 0 FC= −1.3
Log2 Intensity
7.5
8.0
8.5
9.0
9.5
10.0
11.0
10
15
20
25
30
35
40
Gestational age (weeks)

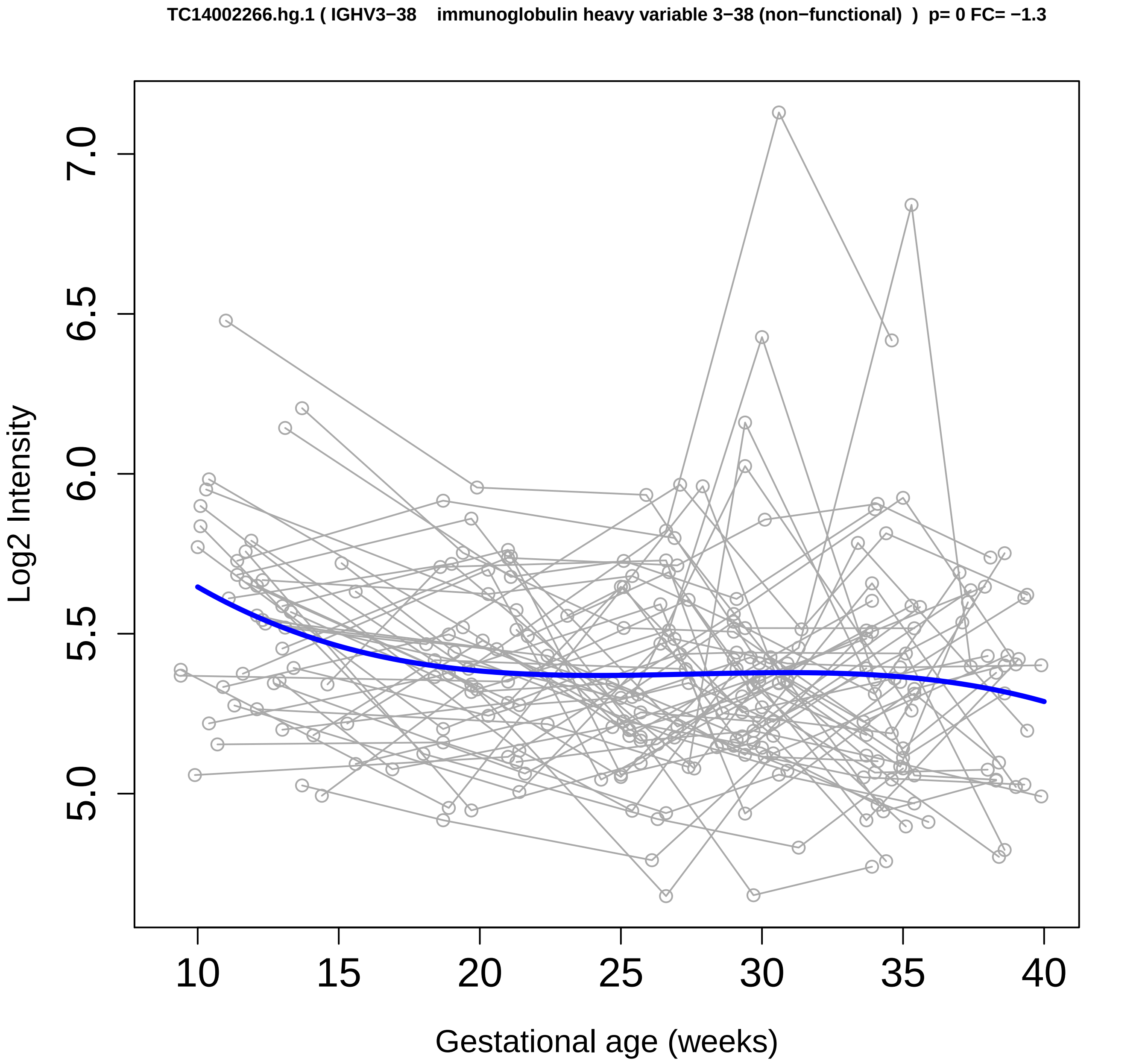

TC14002266.hg.1 (IGHV3−38 immunoglobulin heavy variable 3−38 (non−functional)) p= 0 FC= −1.3
Log2 Intensity
5.0
5.5
6.0
6.5
7.0
10
15
20
25
30
35
40
Gestational age (weeks)

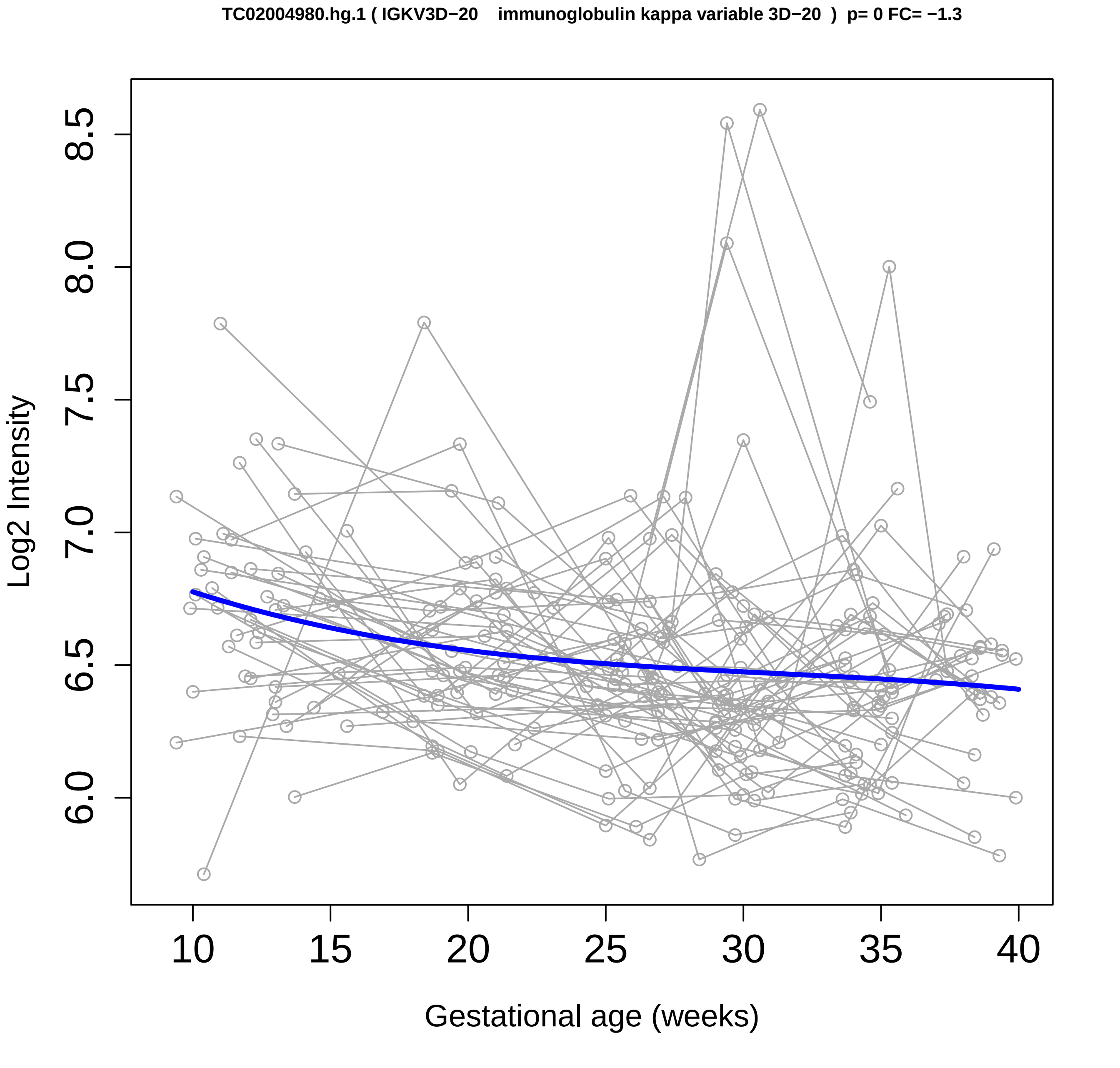
TC02004980.hg.1 (IGKV3D−20   immunoglobulin kappa variable 3D−20) p= 0 FC= −1.3
Log2 Intensity
6.0
6.5
7.0
7.5
8.0
8.5
10
15
20
25
30
35
40
Gestational age (weeks)

TC01000795.hg.1 (IFI44 interferon−induced protein 44) p= 0 FC= 1.6

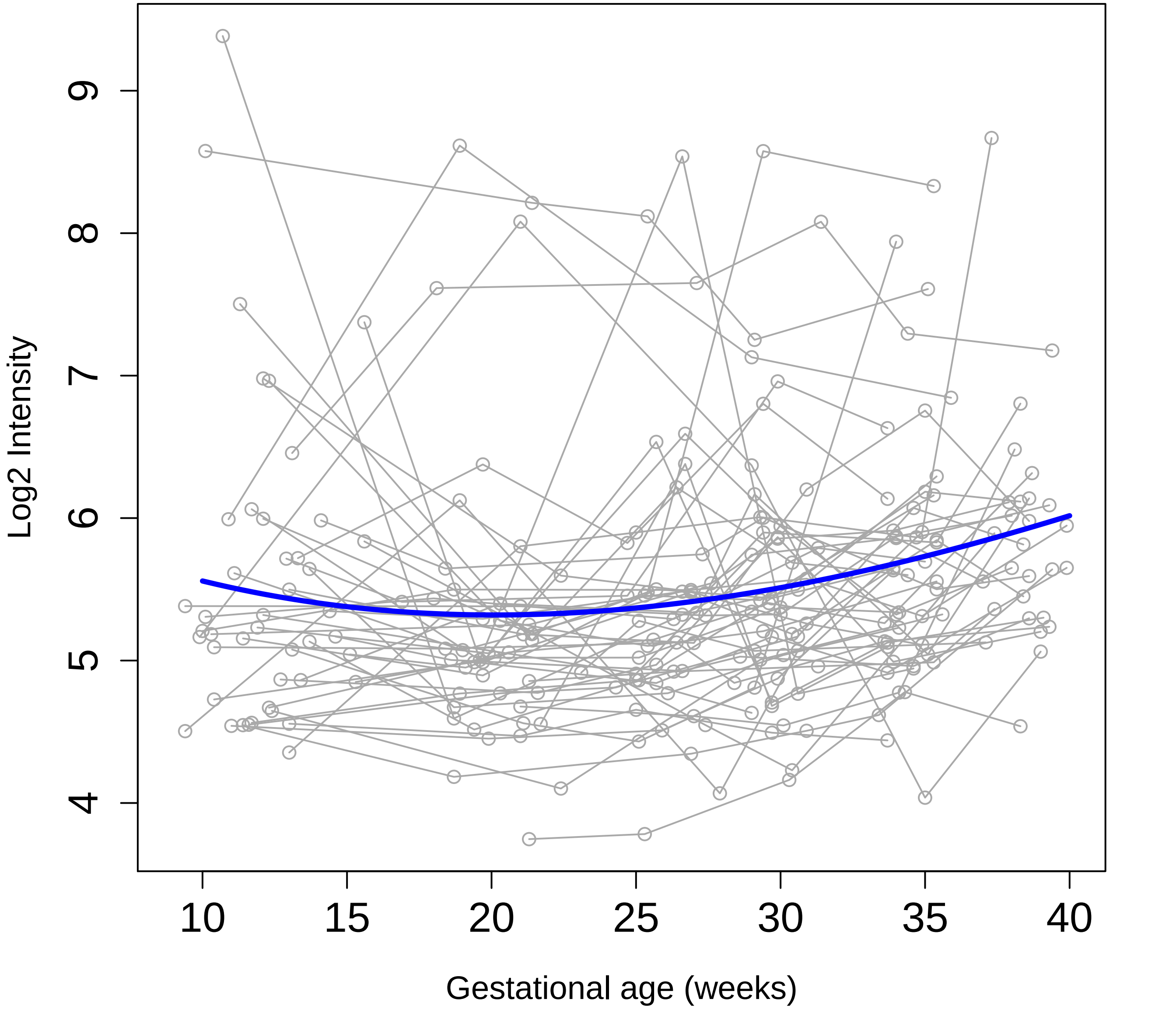

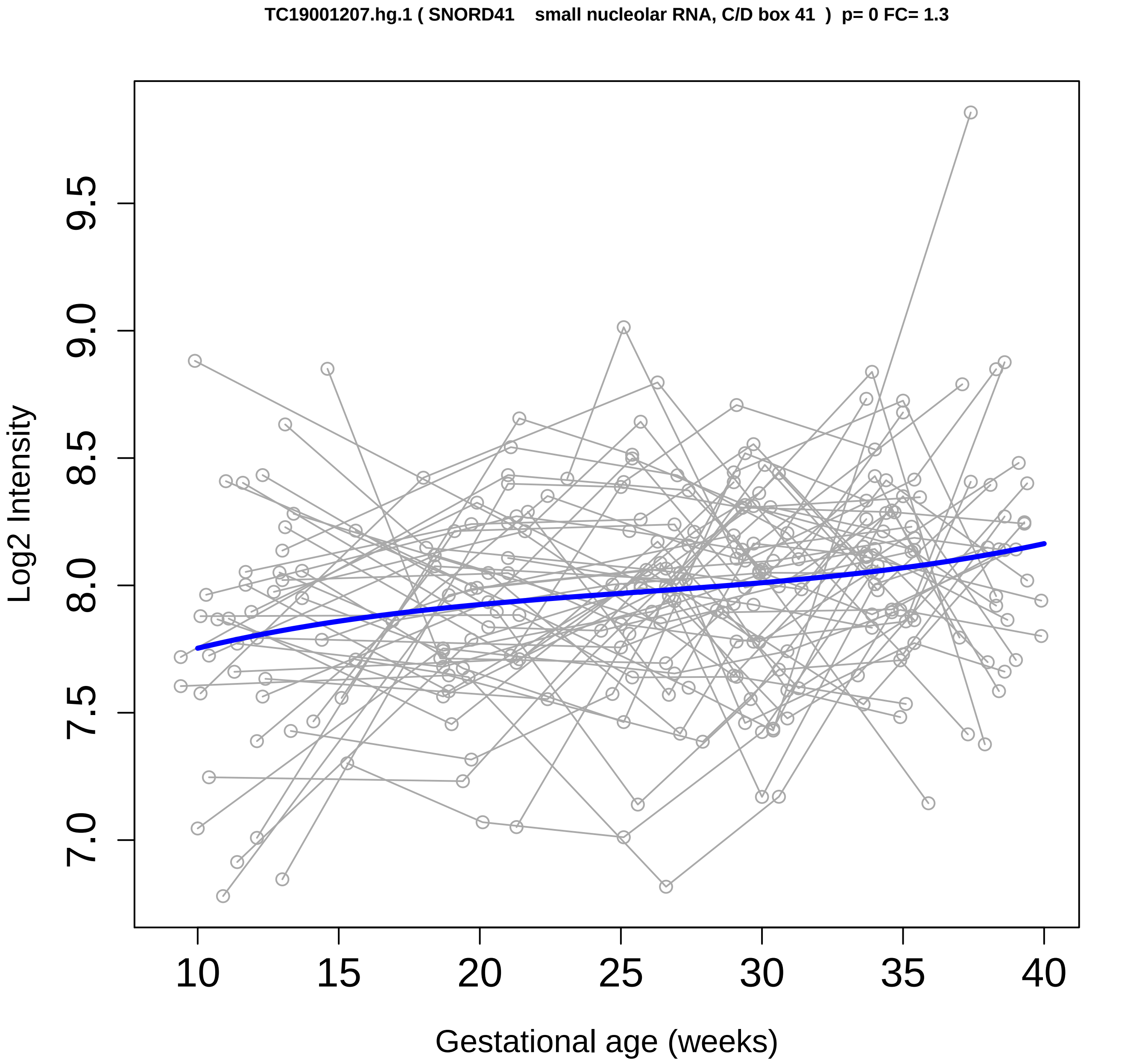

TC19001207.hg.1 (SNORD41 small nucleolar RNA, C/D box 41) p= 0 FC= 1.3
Log2 Intensity
7.0
7.5
8.0
8.5
9.0
9.5
10
15
20
25
30
35
40
Gestational age (weeks)

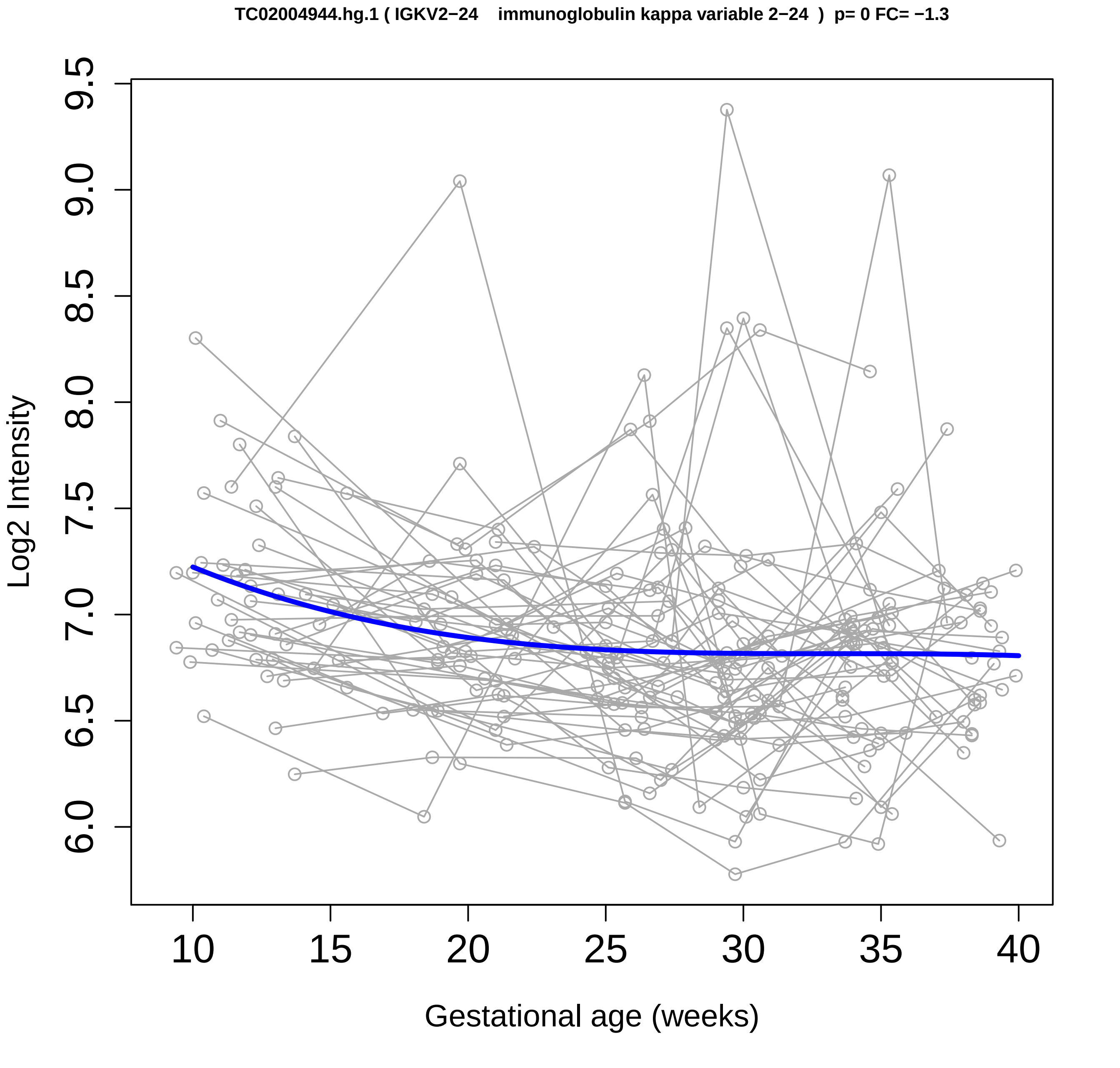
TC02004944.hg.1 (IGKV2−24 immunoglobulin kappa variable 2−24) p= 0 FC= −1.3
Log2 Intensity
9.5
9.0
8.5
8.0
7.5
7.0
6.5
6.0
10
15
20
25
30
35
40
Gestational age (weeks)

TC14002215.hg.1 (IGHJ5 immunoglobulin heavy joining 5) p= 0 FC= −1.3

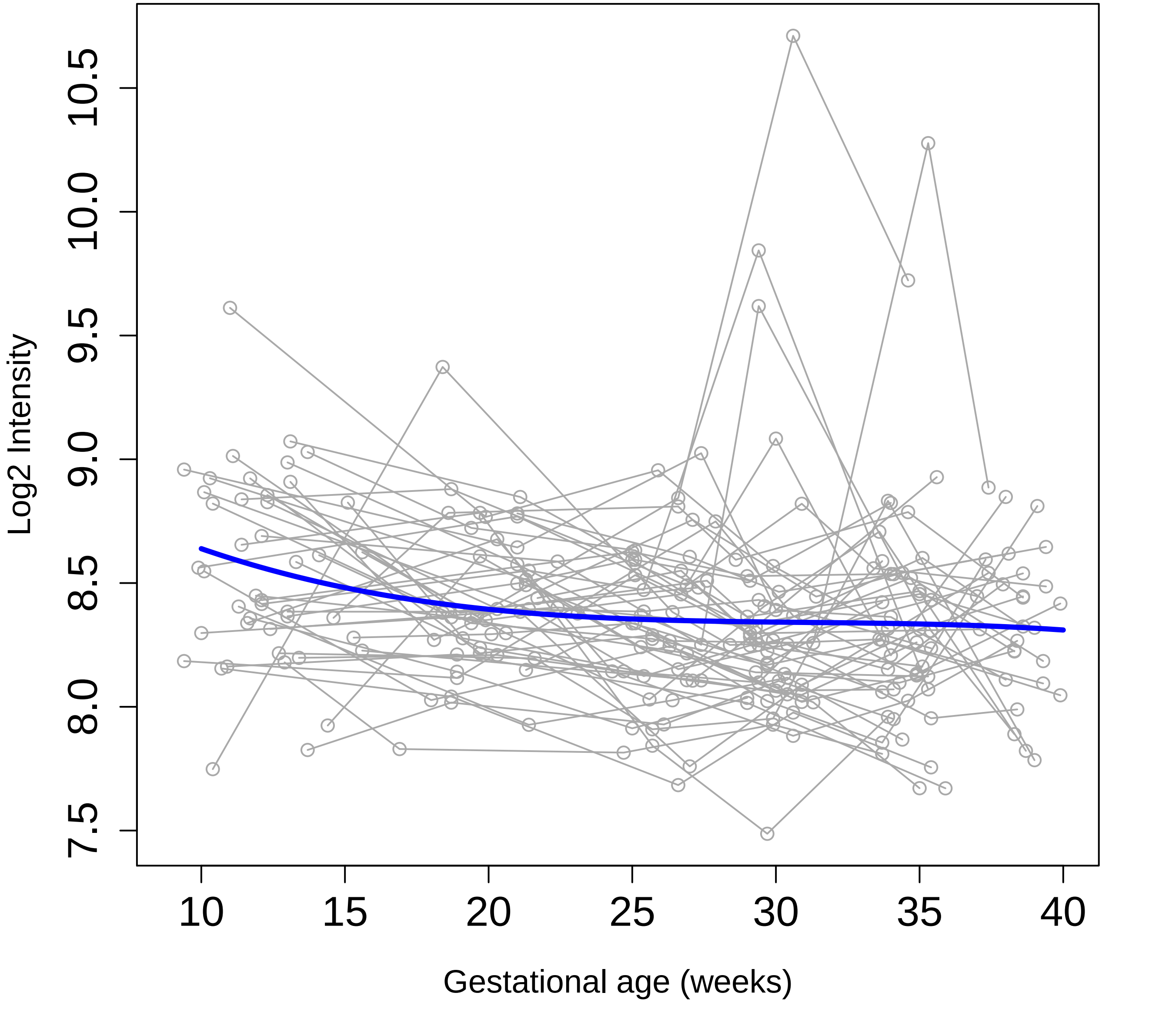

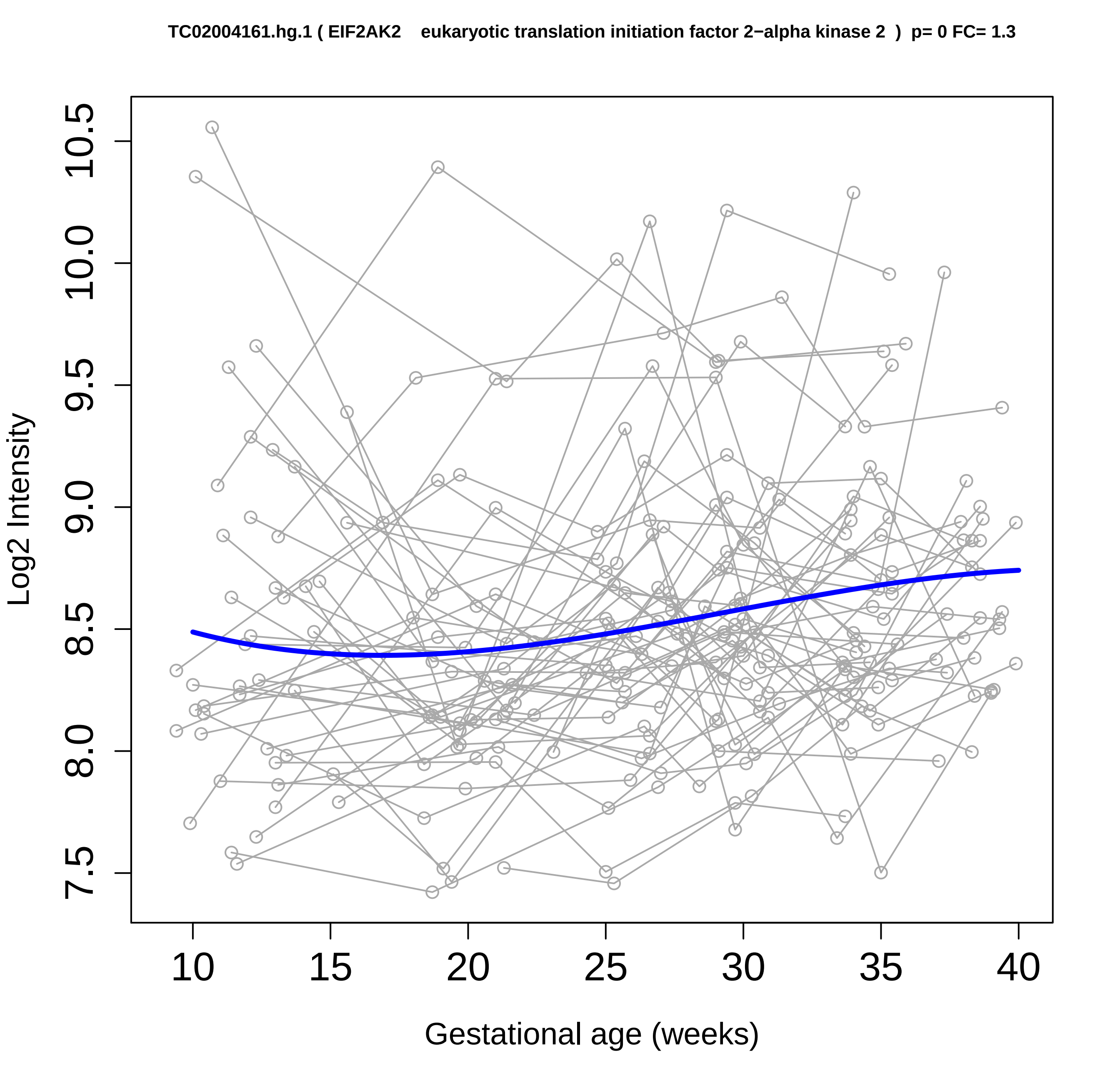
TC02004161.hg.1 (EIF2AK2 eukaryotic translation initiation factor 2−alpha kinase 2) p= 0 FC= 1.3
Log2 Intensity
10.5
10.0
9.5
9.0
8.5
8.0
7.5
10
15
20
25
30
35
40
Gestational age (weeks)

TC04002926.hg.1 (GYPB glycophorin B (MNS blood group)) p= 0 FC= 1.3

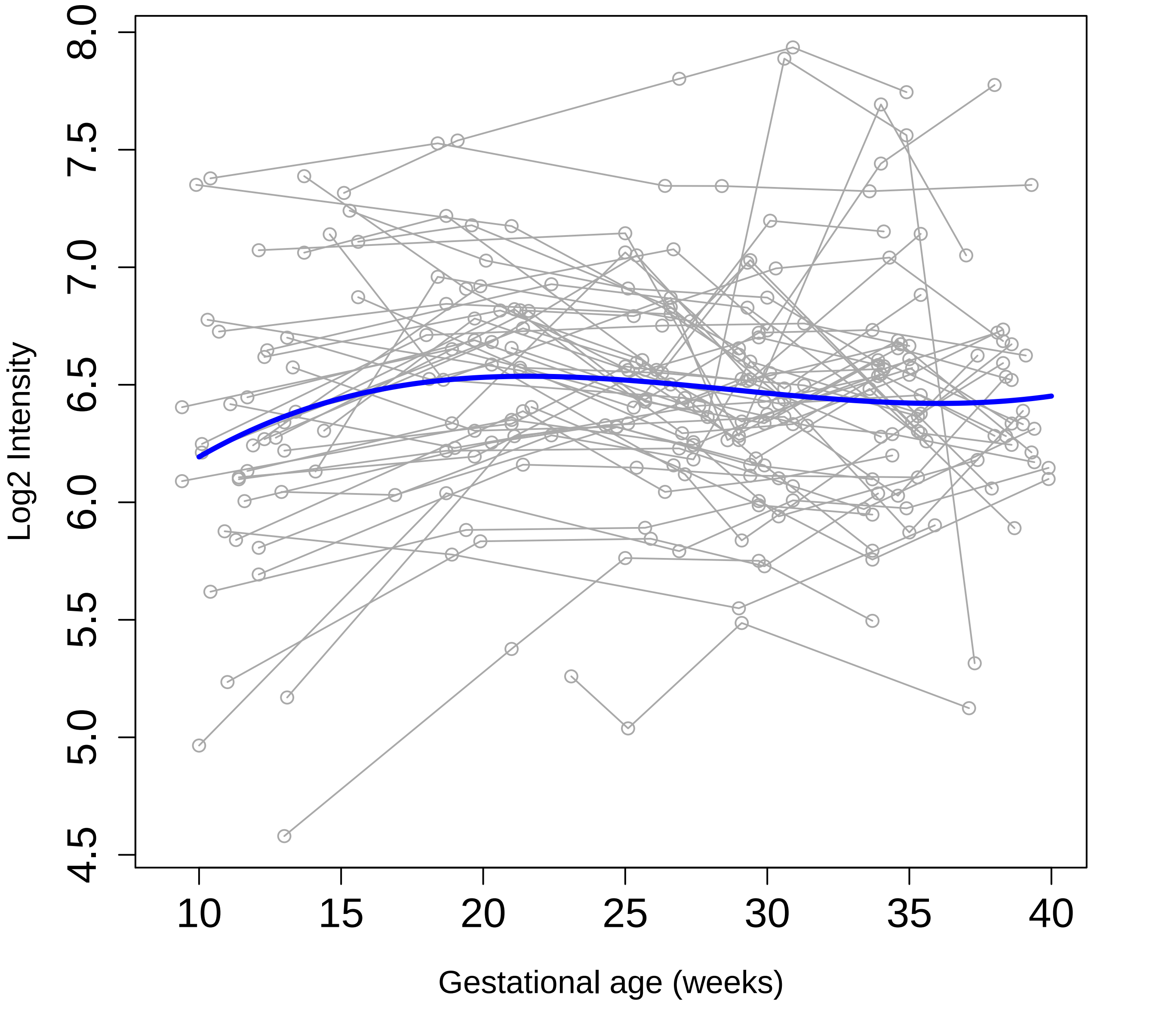

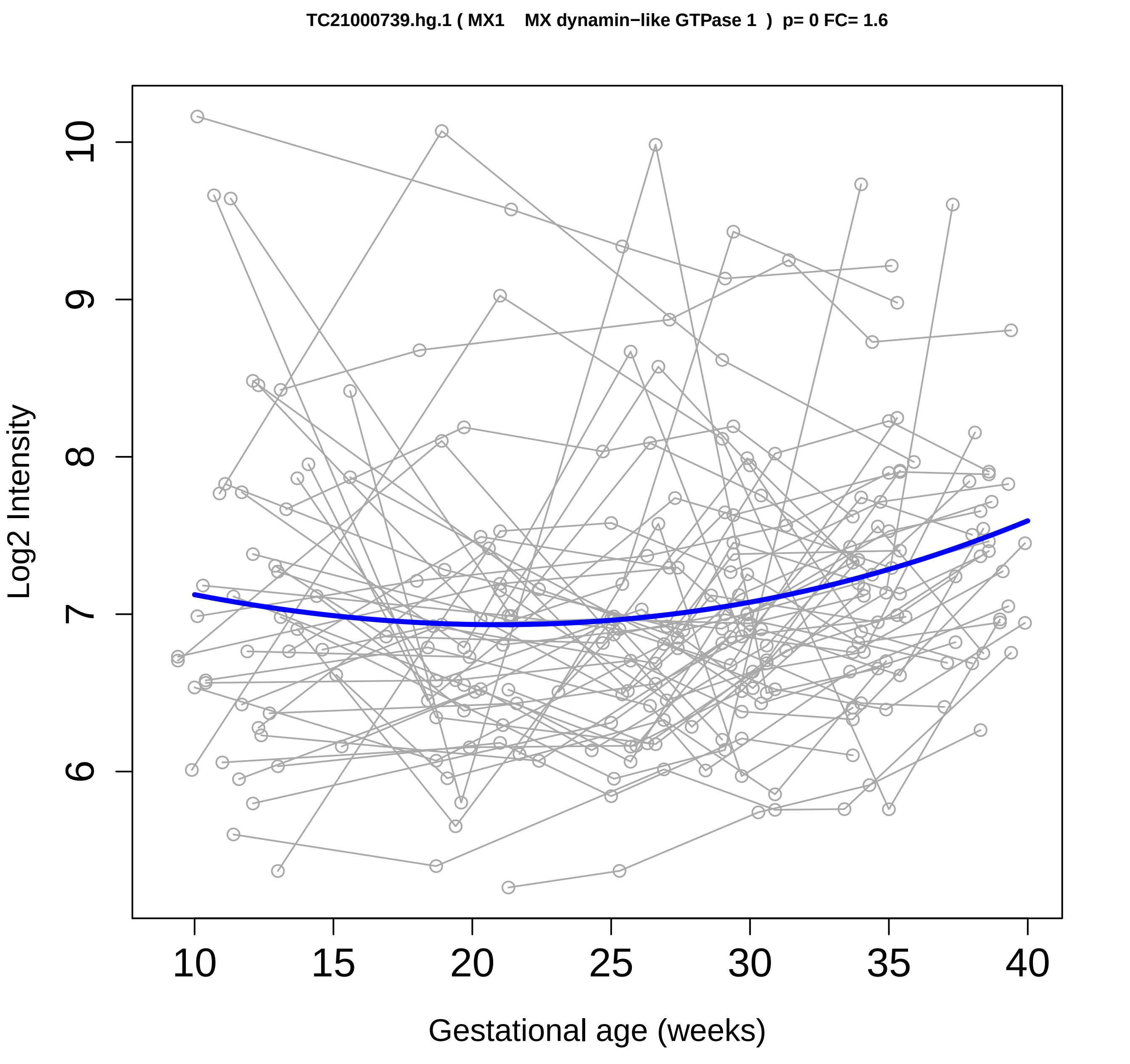
TC21000739.hg.1 (MX1   MX dynamin−like GTPase 1) p= 0 FC= 1.6
Log2 Intensity
6
7
8
9
10
10
15
20
25
30
35
40
Gestational age (weeks)

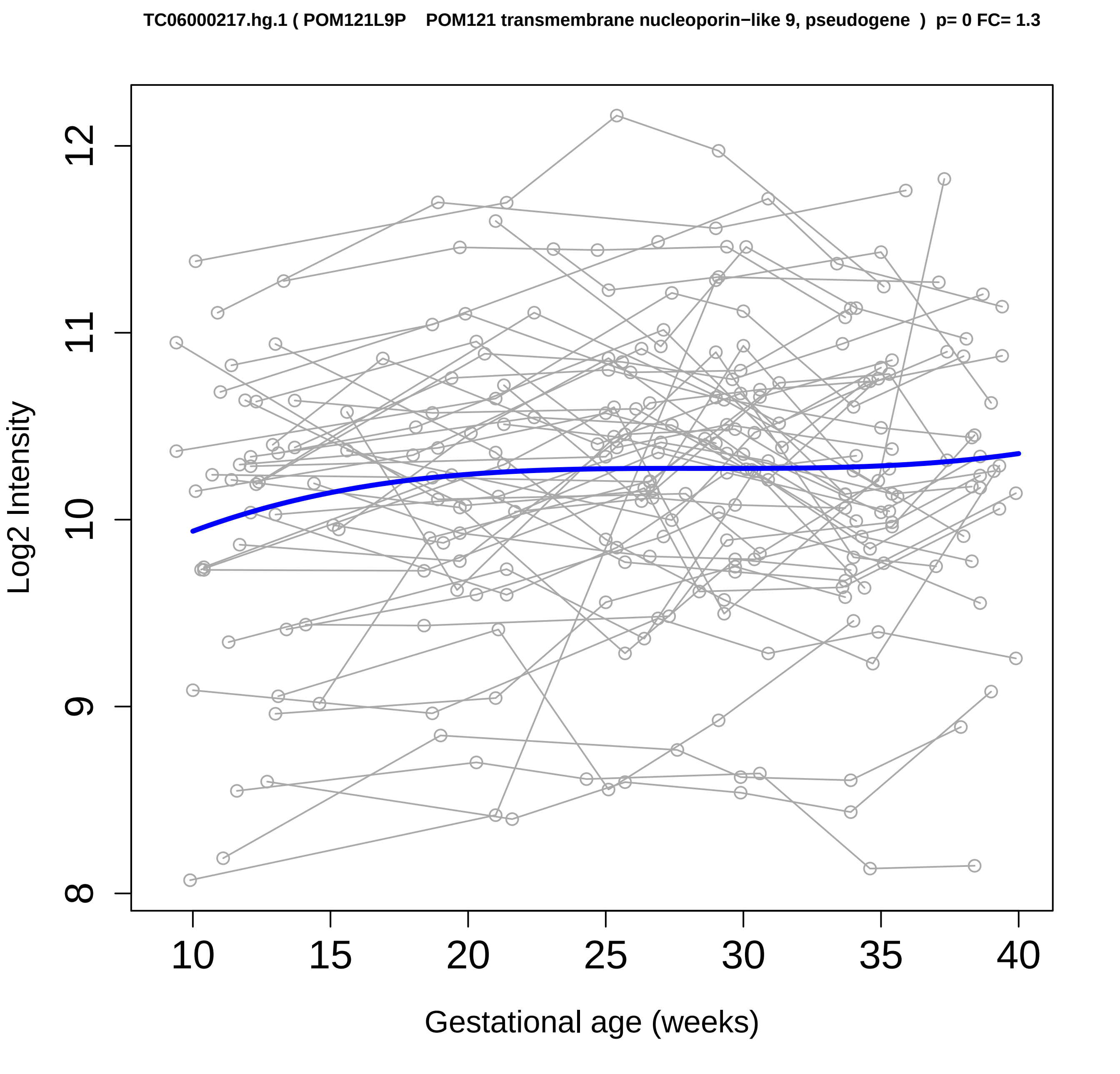
TC06000217.hg.1 (POM121L9P POM121 transmembrane nucleoporin−like 9, pseudogene) p= 0 FC= 1.3
Log2 Intensity
Gestational age (weeks)
8
9
10
11
12
10
15
20
25
30
35
40

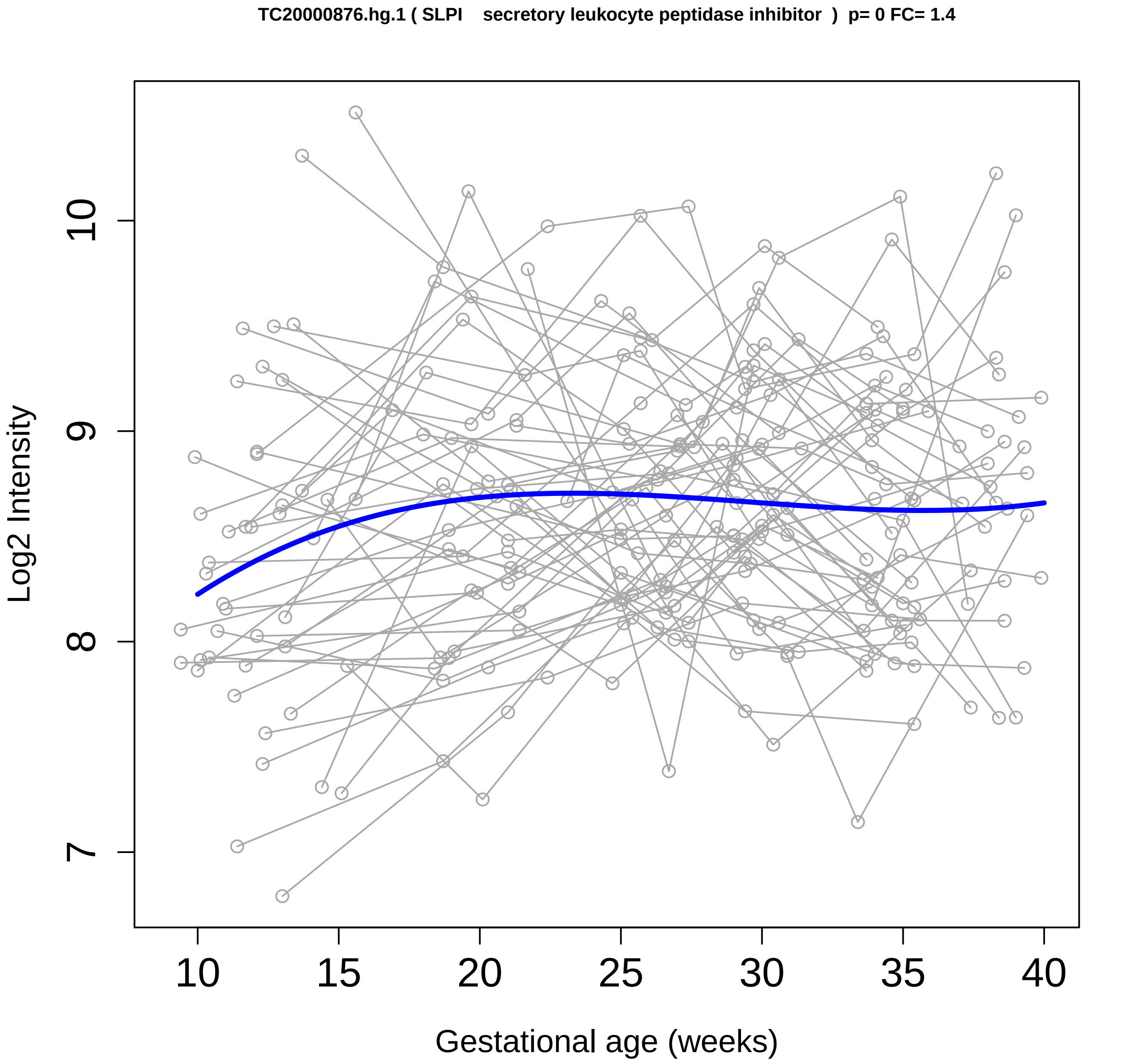
TC20000876.hg.1 (SLPI secretory leukocyte peptidase inhibitor) p= 0 FC= 1.4
Log2 Intensity
7
8
9
10
10
15
20
25
30
35
40
Gestational age (weeks)

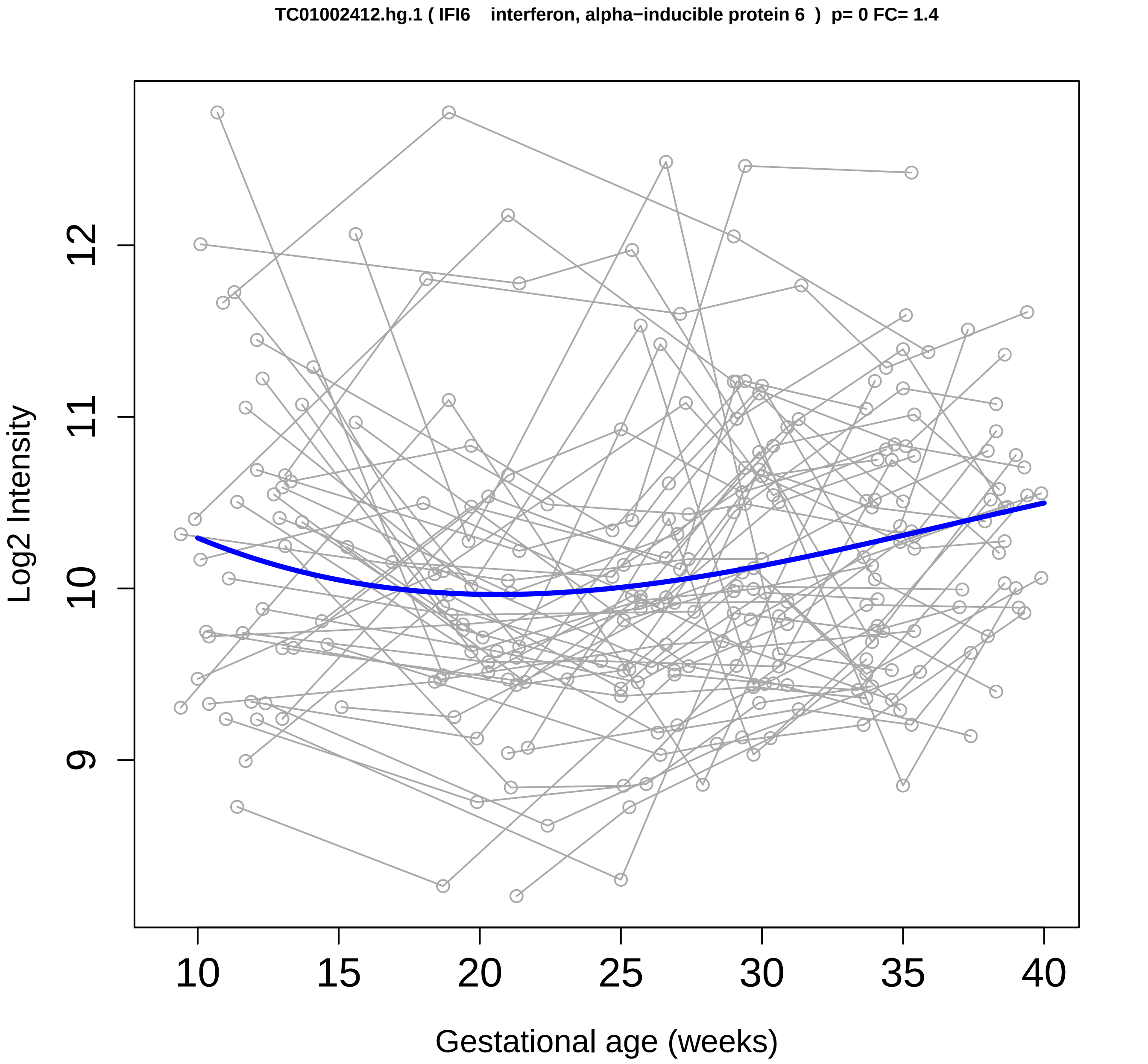

TC01002412.hg.1 (IFI6 interferon, alpha−inducible protein 6) p= 0 FC= 1.4
Log2 Intensity
9
10
11
12
10
15
20
25
30
35
40
Gestational age (weeks)

TC01004503.hg.1 (PRKACB   protein kinase, cAMP−dependent, catalytic, beta) p= 0 FC= −1.3

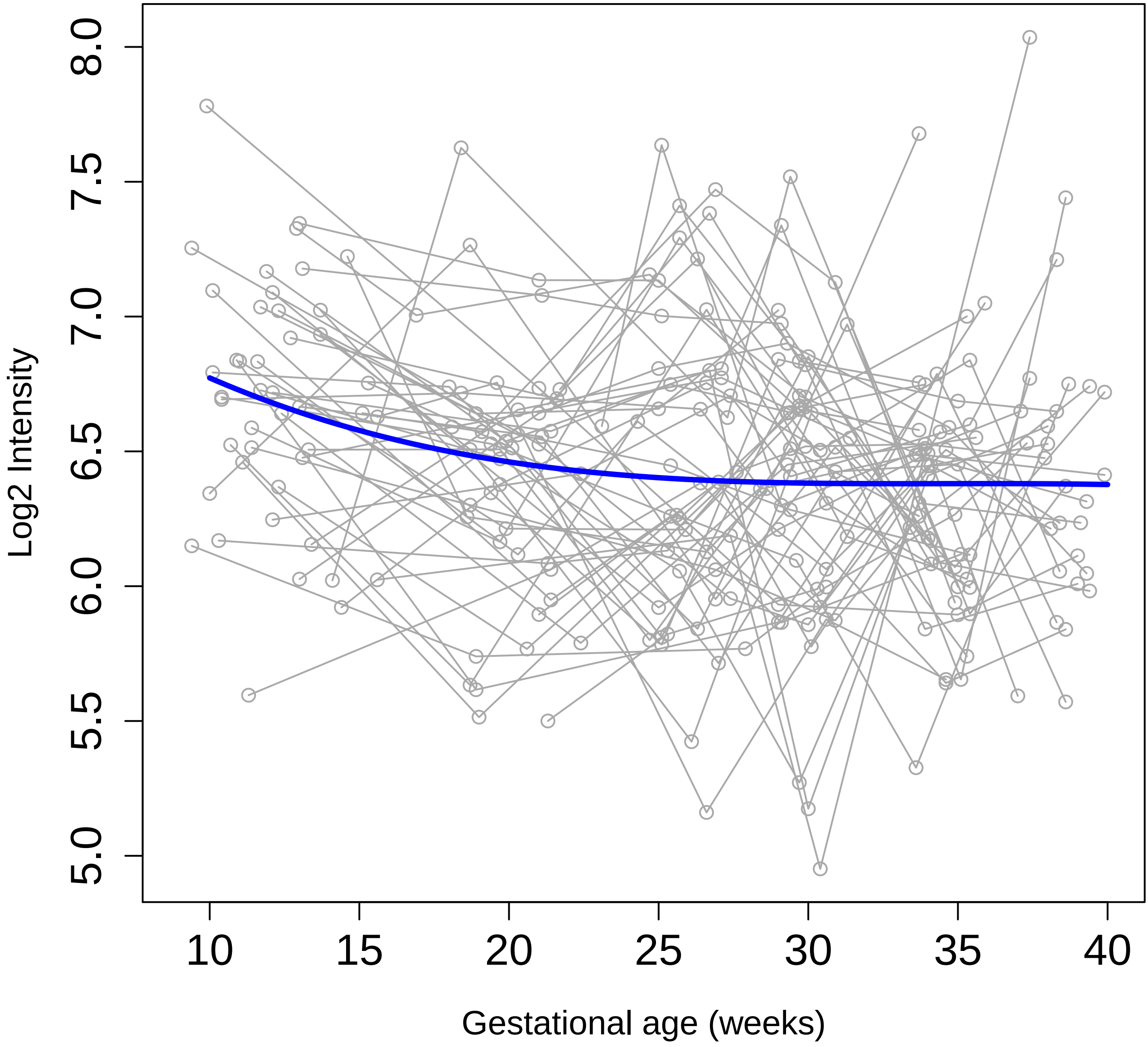

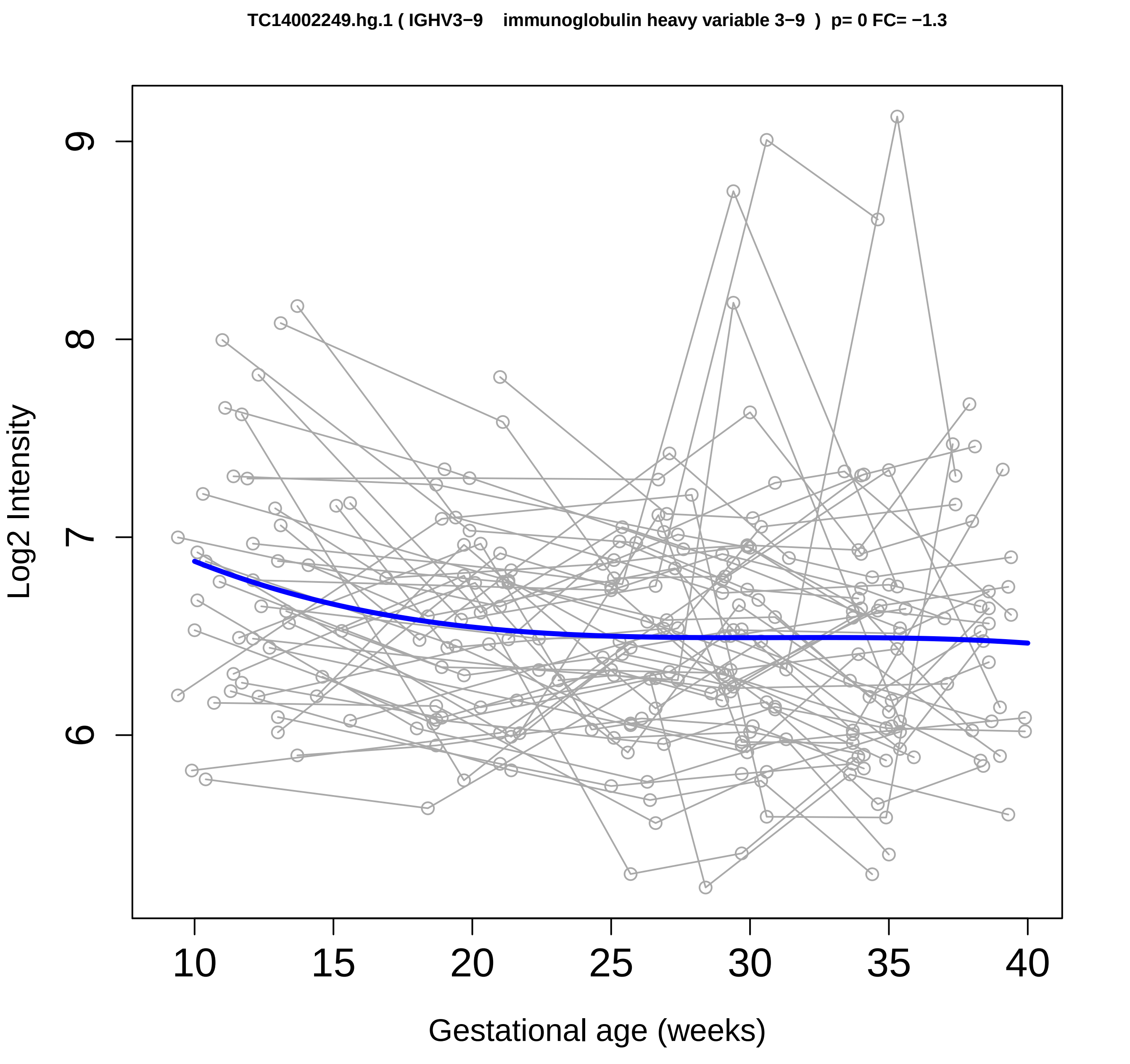
TC14002249.hg.1 (IGHV3−9 immunoglobulin heavy variable 3−9) p= 0 FC= −1.3
Log2 Intensity
9
8
7
6
10
15
20
25
30
35
40
Gestational age (weeks)

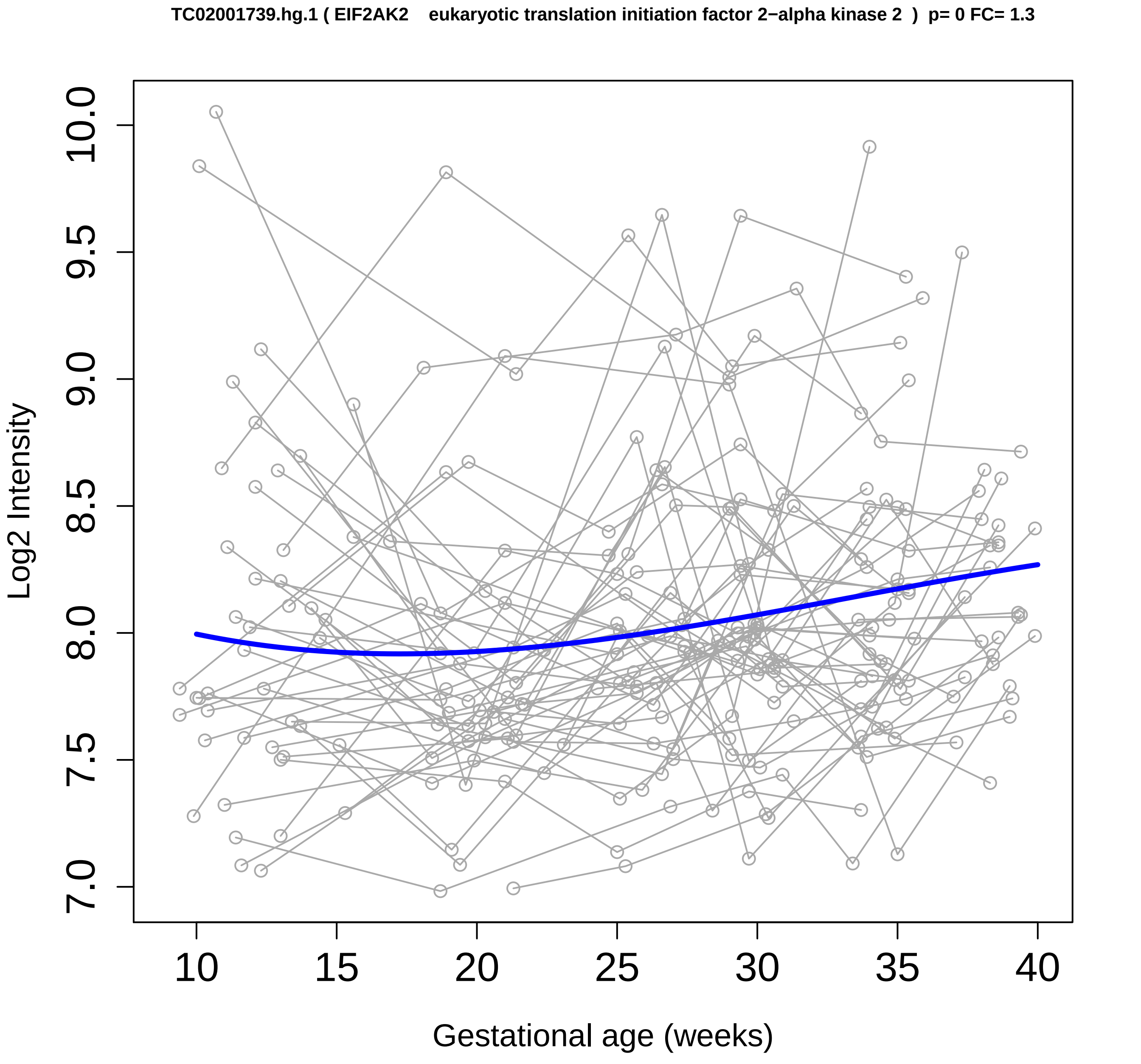
TC02001739.hg.1 (EIF2AK2 eukaryotic translation initiation factor 2−alpha kinase 2) p= 0 FC= 1.3
Log2 Intensity
7.0
7.5
8.0
8.5
9.0
9.5
10.0
10
15
20
25
30
35
40
Gestational age (weeks)

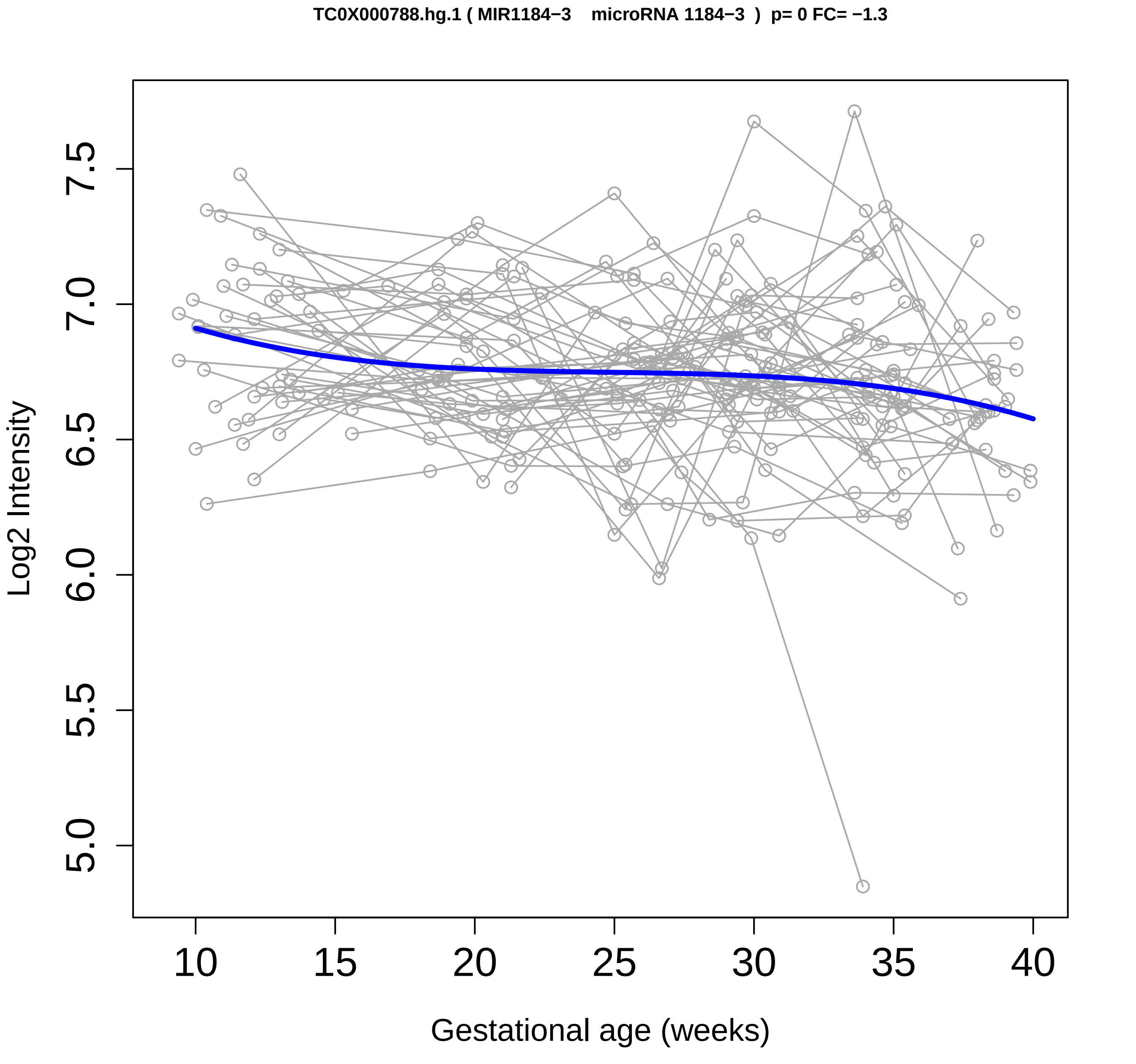

TC0X000788.hg.1 (MIR1184−3 microRNA 1184−3) p= 0 FC= −1.3
Log2 Intensity
7.5
7.0
6.5
6.0
5.5
5.0
10
15
20
25
30
35
40
Gestational age (weeks)

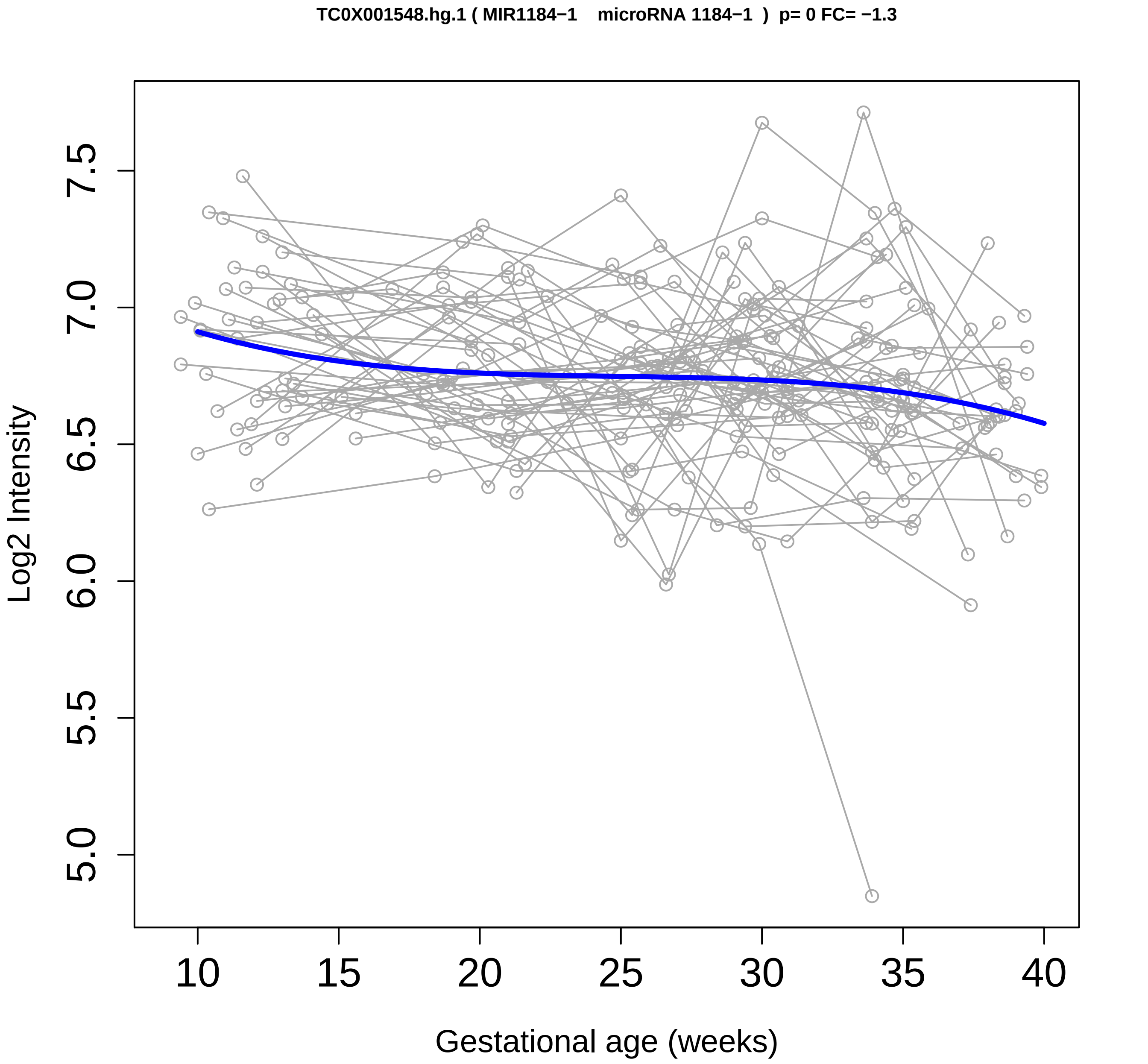

TC0X001548.hg.1 (MIR1184−1 microRNA 1184−1) p= 0 FC= −1.3
Log2 Intensity
7.5
7.0
6.5
6.0
5.5
5.0
10
15
20
25
30
35
40
Gestational age (weeks)

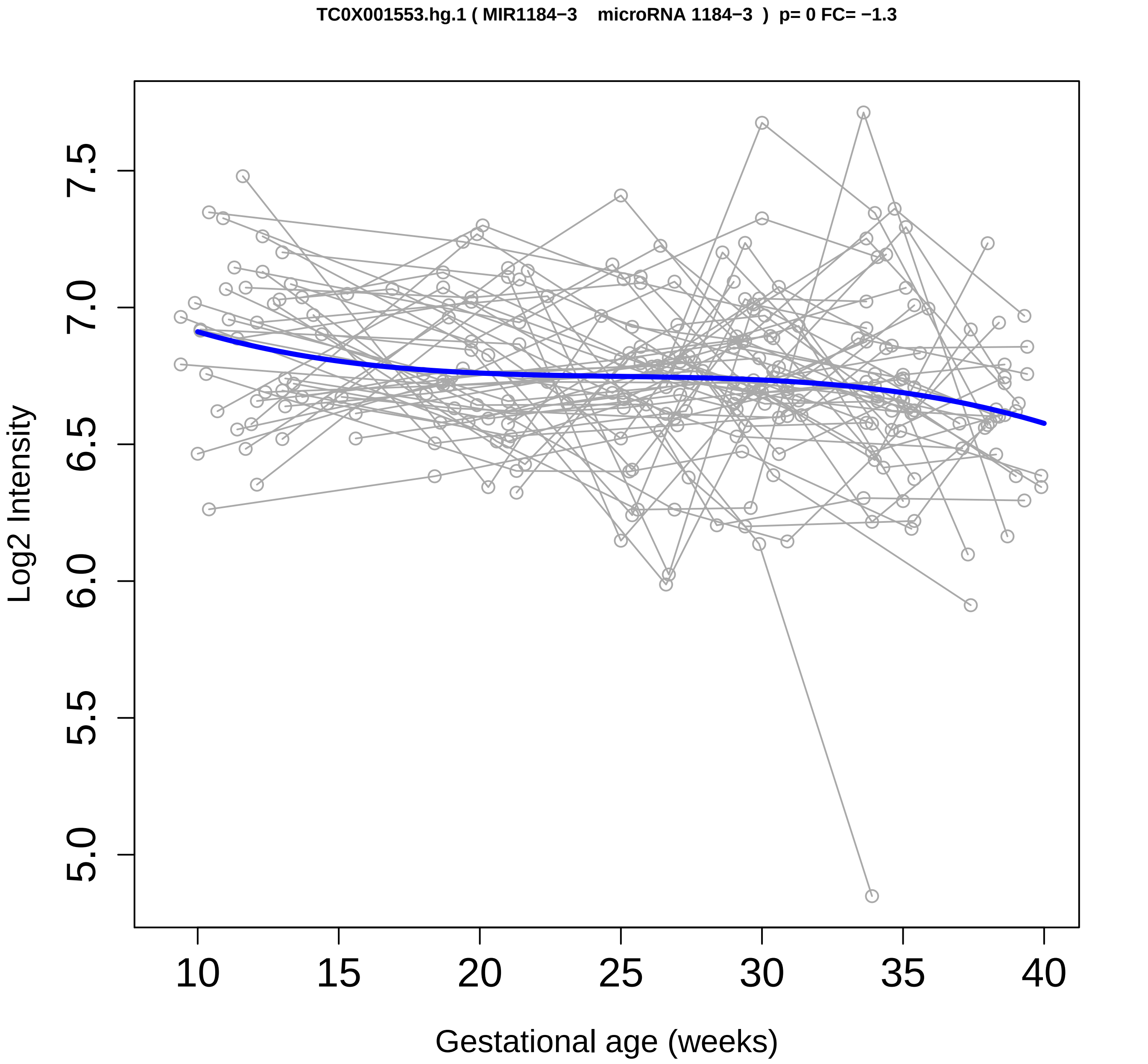
TC0X001553.hg.1 (MIR1184−3 microRNA 1184−3) p= 0 FC= −1.3
Log2 Intensity
7.5
7.0
6.5
6.0
5.5
5.0
10
15
20
25
30
35
40
Gestational age (weeks)

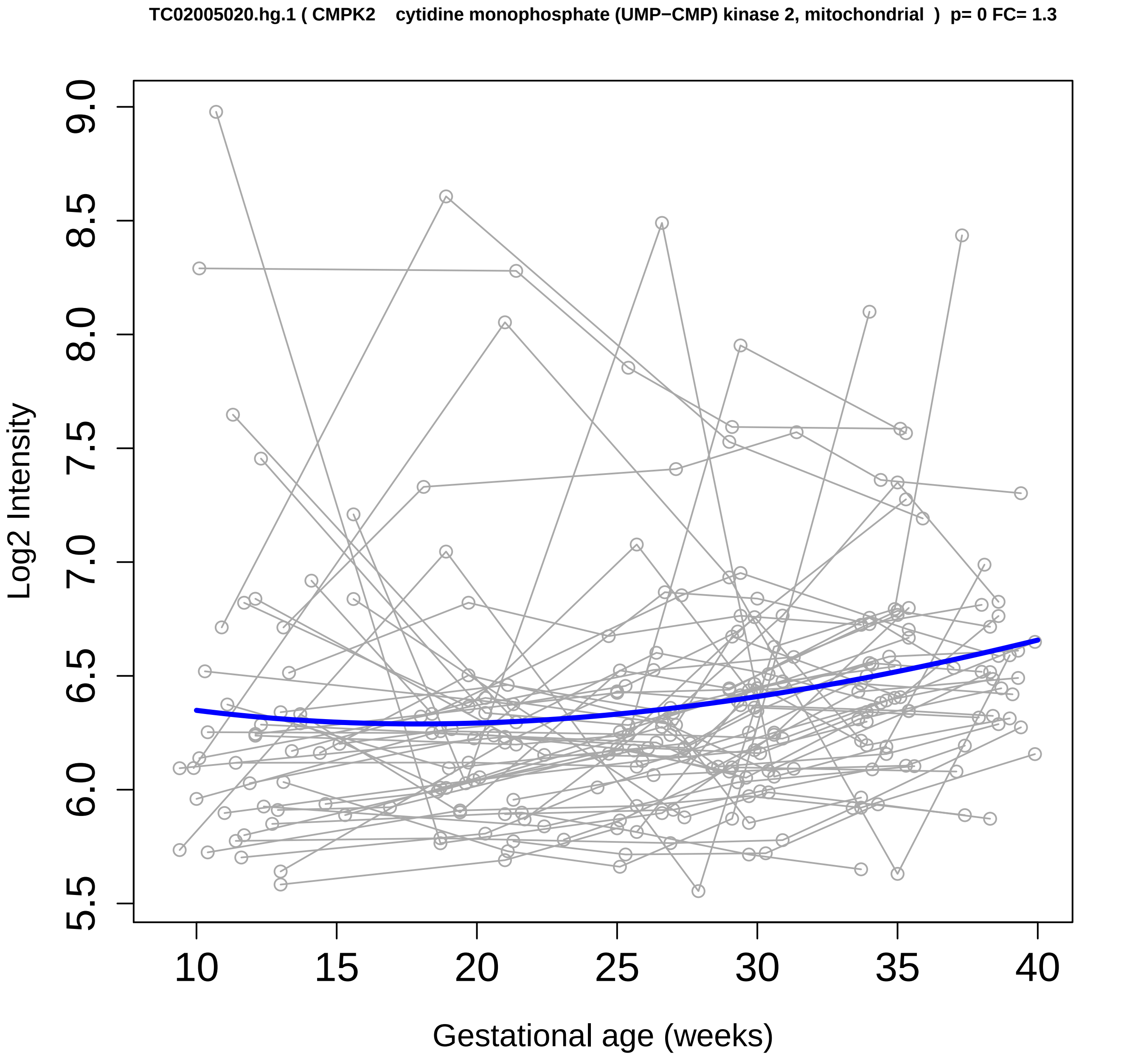

TC02005020.hg.1 (CMPK2 cytidine monophosphate (UMP−CMP) kinase 2, mitochondrial) p= 0 FC= 1.3
Log2 Intensity
5.5
6.0
6.5
7.0
7.5
8.0
8.5
9.0
10
15
20
25
30
35
40
Gestational age (weeks)

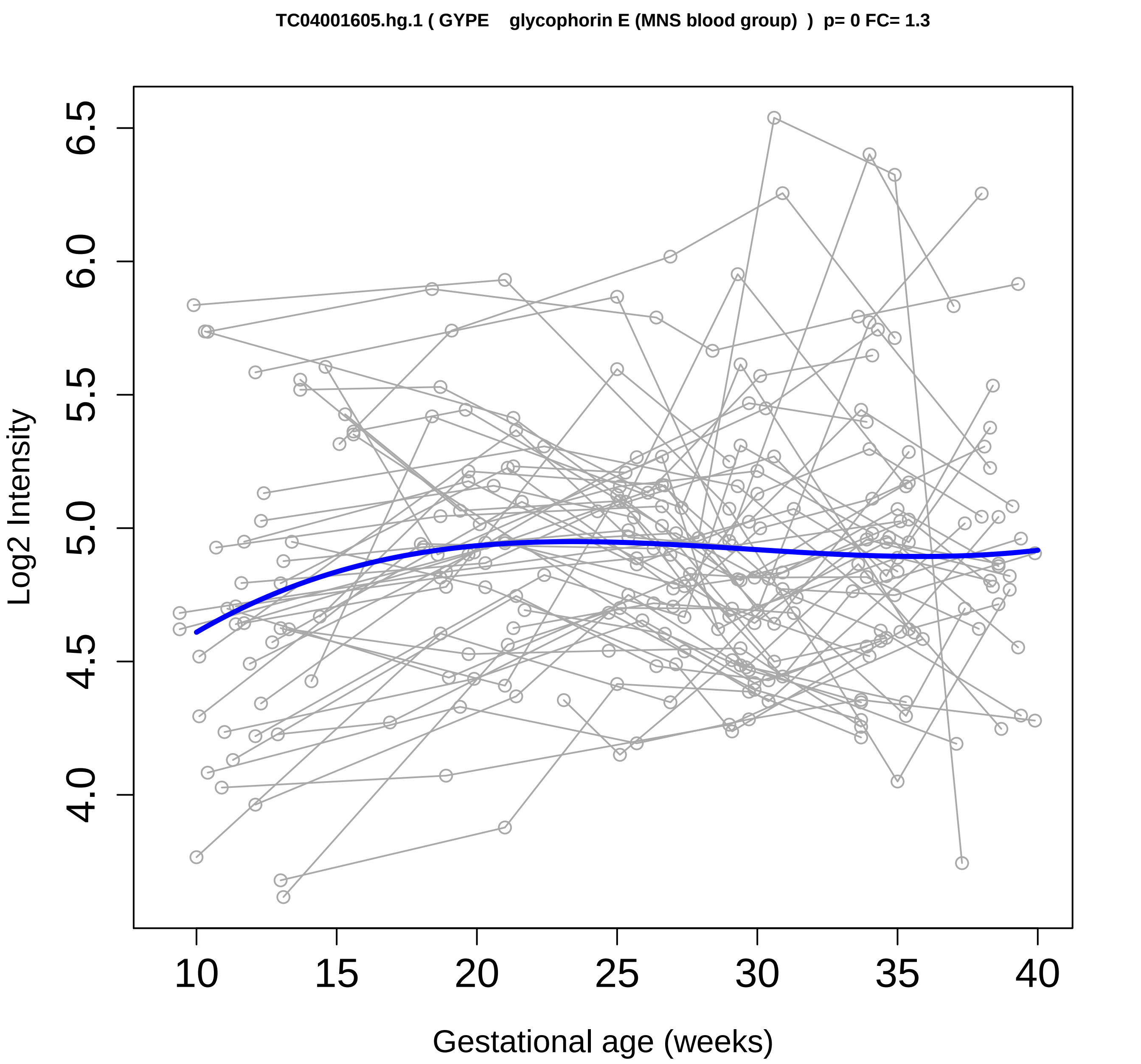

TC04001605.hg.1 (GYPE glycophorin E (MNS blood group)) p= 0 FC= 1.3
Log2 Intensity
4.0
4.5
5.0
5.5
6.0
6.5
10
15
20
25
30
35
40
Gestational age (weeks)

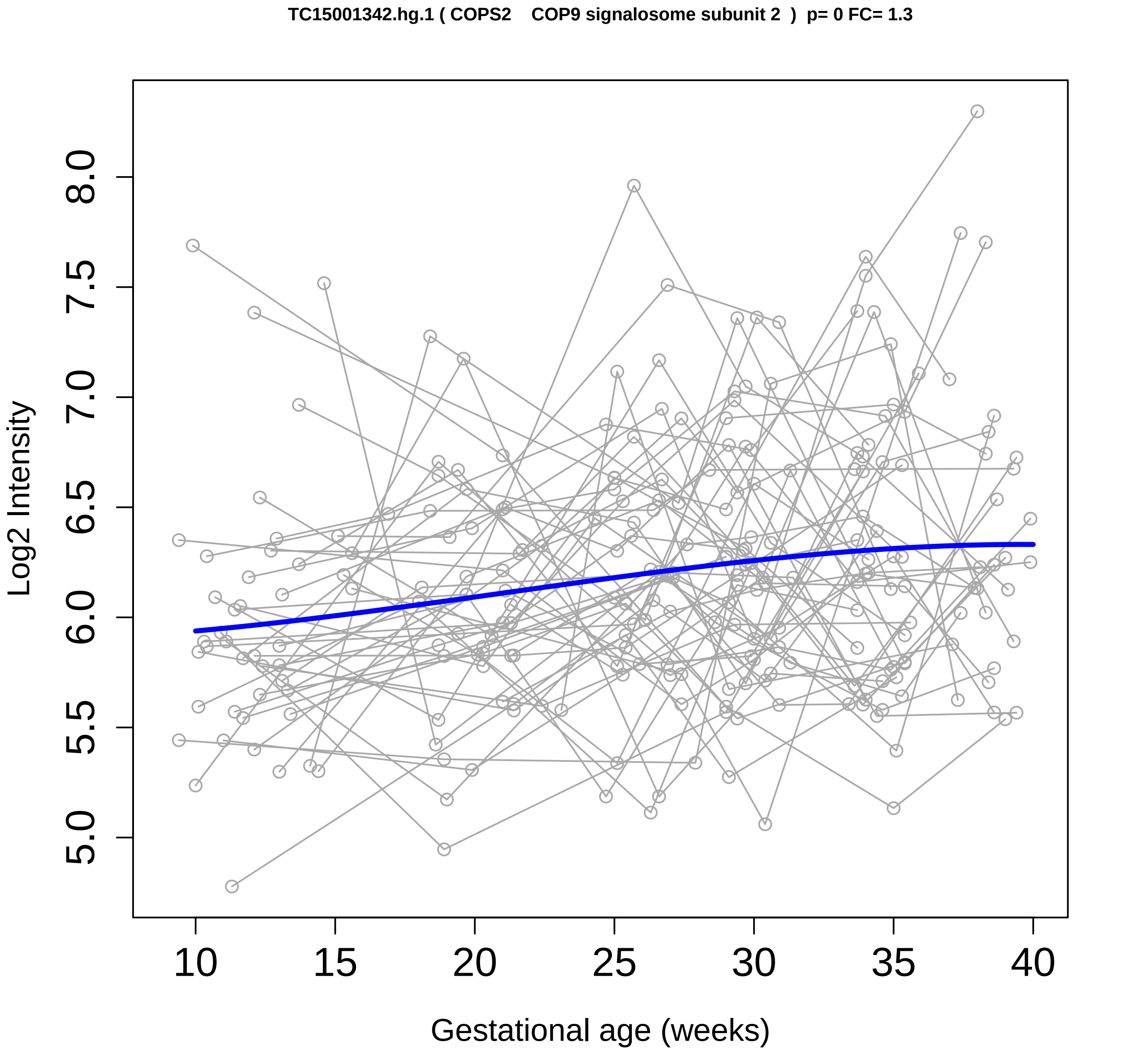
TC15001342.hg.1 (COPS2   COP9 signalosome subunit 2) p= 0 FC= 1.3
Log2 Intensity
5.0
5.5
6.0
6.5
7.0
7.5
8.0
10
15
20
25
30
35
40
Gestational age (weeks)

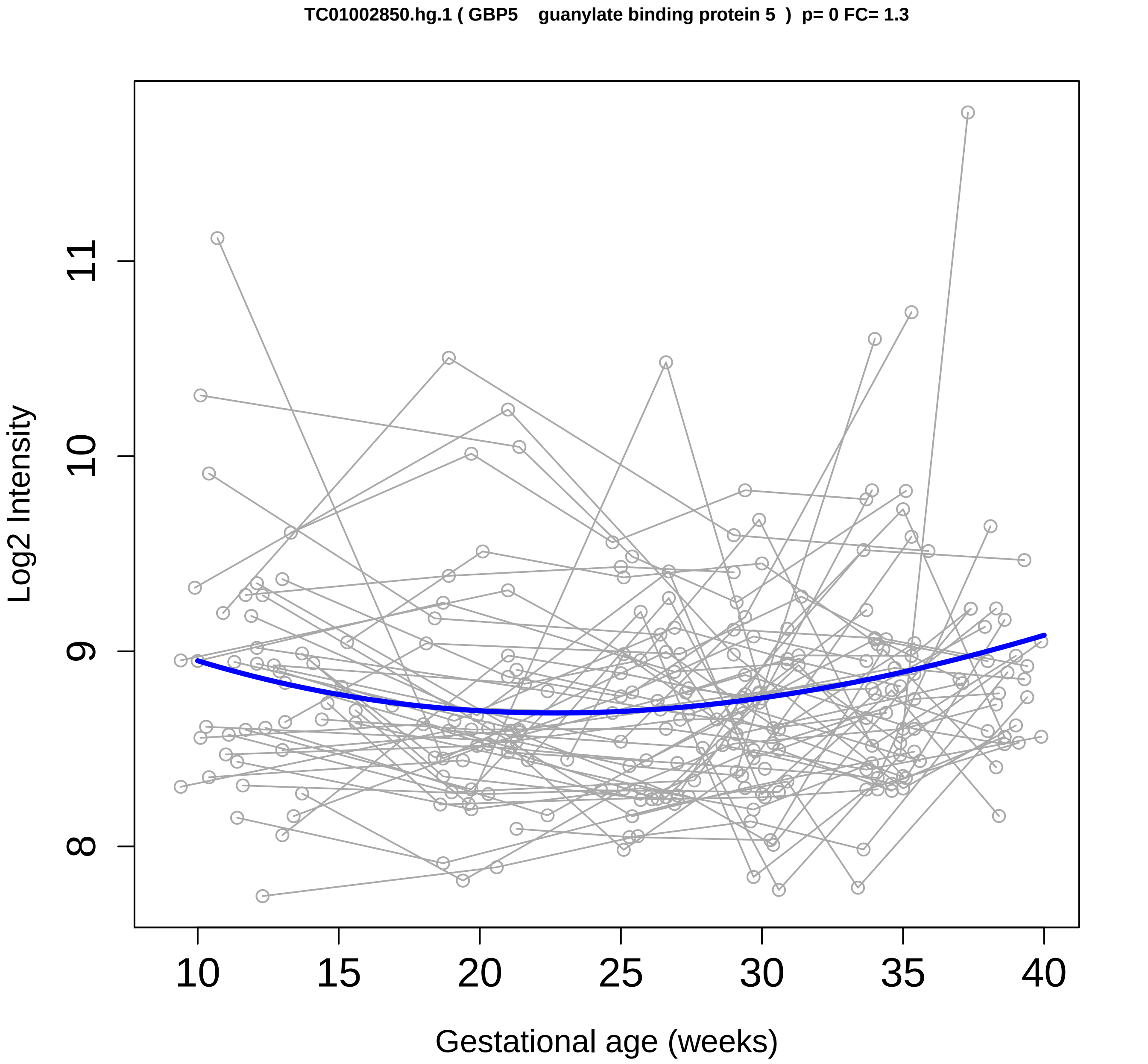
TC01002850.hg.1 (GBP5 guanylate binding protein 5) p= 0 FC= 1.3
Log2 Intensity
8
9
10
11
10
15
20
25
30
35
40
Gestational age (weeks)

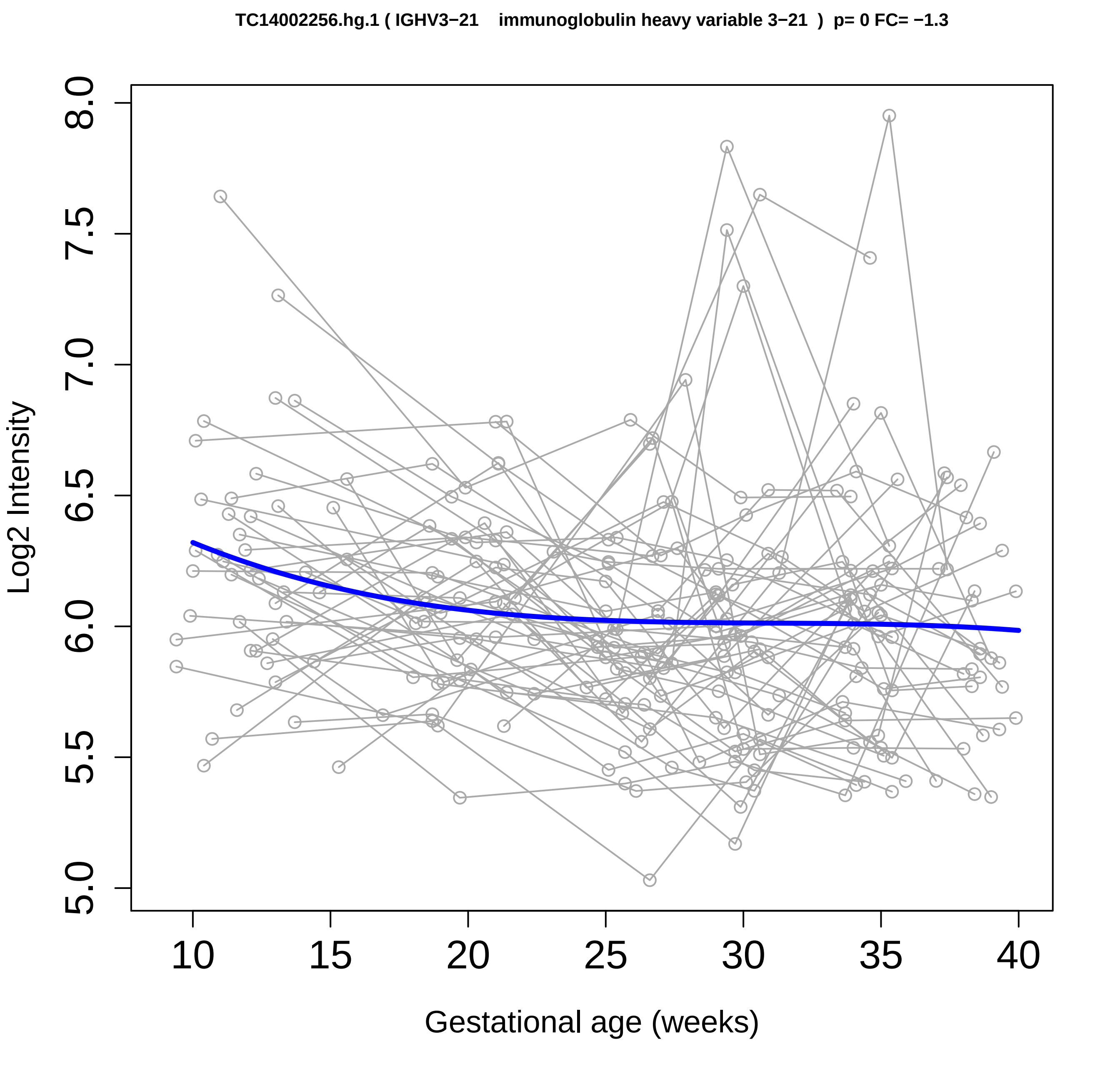
TC14002256.hg.1 (IGHV3−21 immunoglobulin heavy variable 3−21) p= 0 FC= −1.3
Log2 Intensity
5.0
5.5
6.0
6.5
7.0
7.5
8.0
10
15
20
25
30
35
40
Gestational age (weeks)

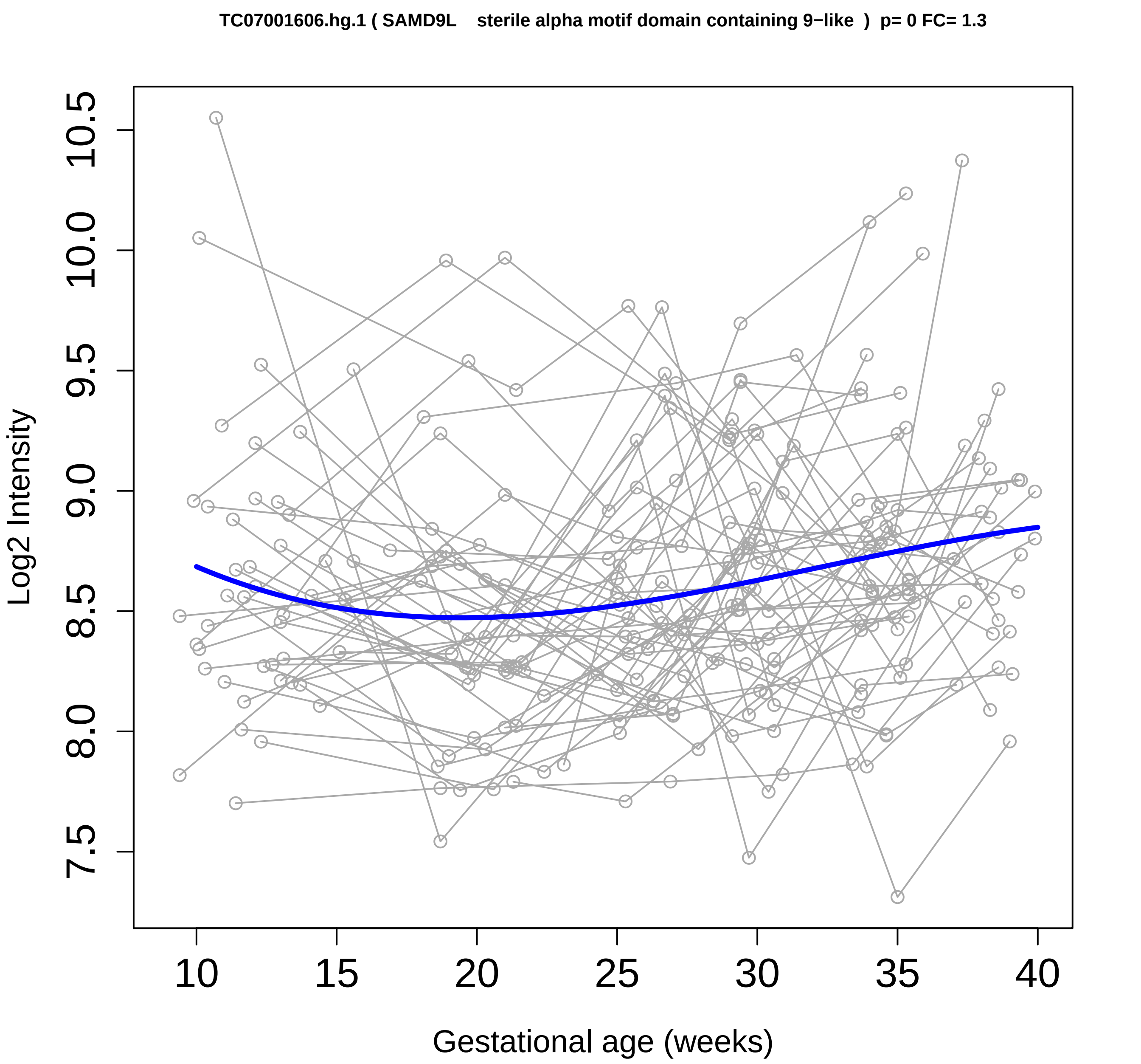
TC07001606.hg.1 (SAMD9L sterile alpha motif domain containing 9−like) p= 0 FC= 1.3
Log2 Intensity
10.5
10.0
9.5
9.0
8.5
8.0
7.5
10
15
20
25
30
35
40
Gestational age (weeks)

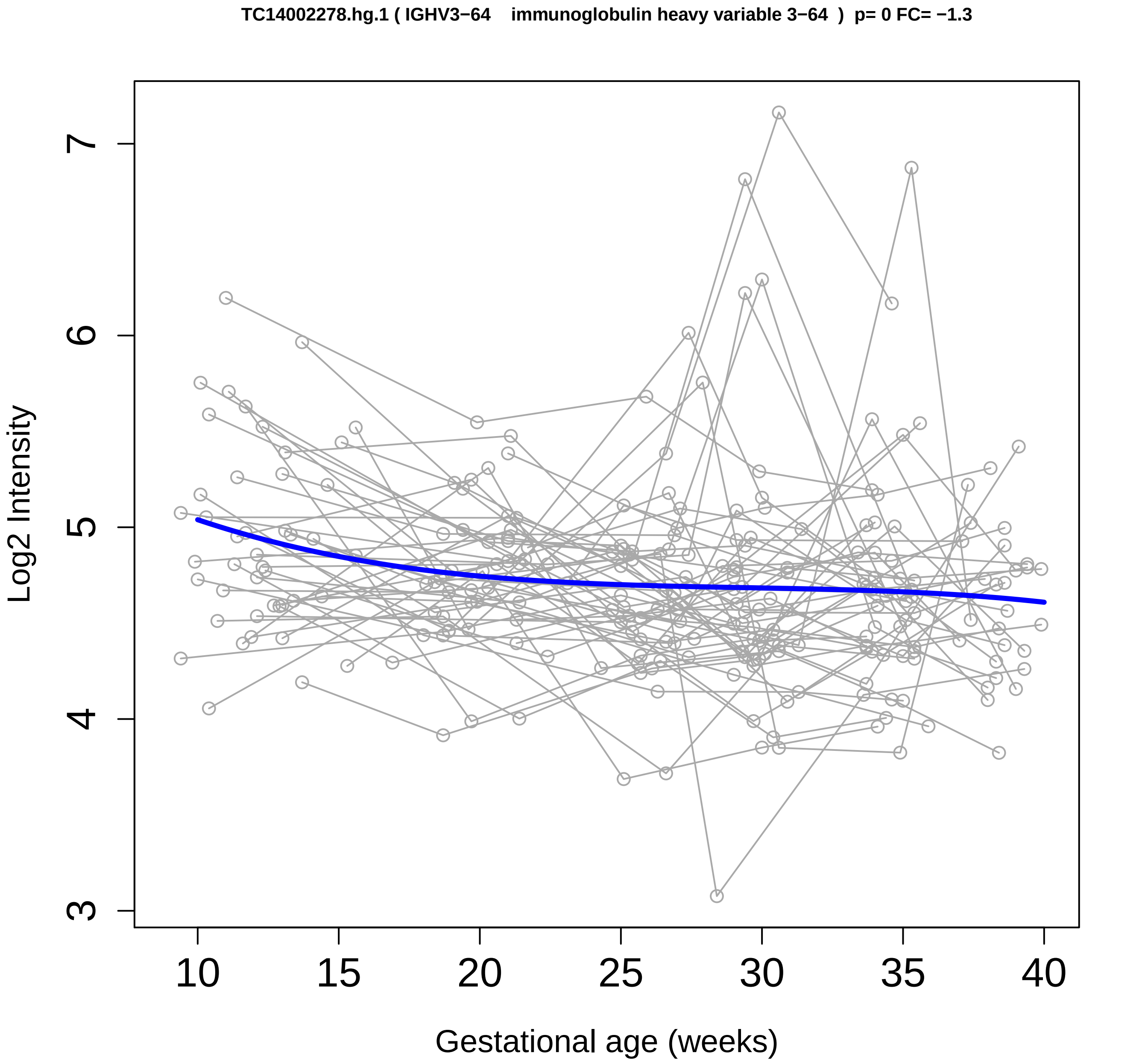

TC14002278.hg.1 (IGHV3−64 immunoglobulin heavy variable 3−64) p= 0 FC= −1.3
Log2 Intensity
3
4
5
6
7
10
15
20
25
30
35
40
Gestational age (weeks)

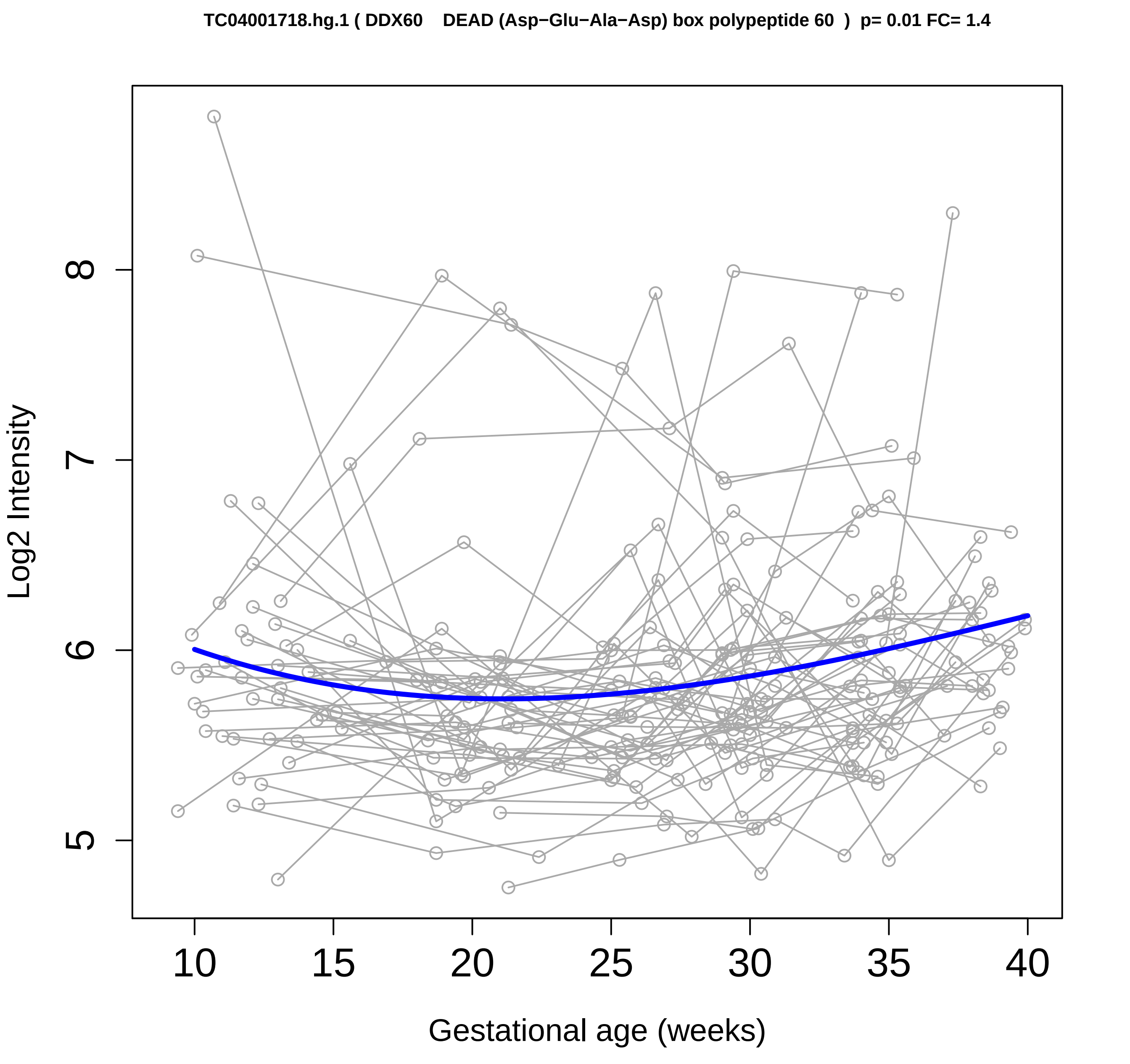
TC04001718.hg.1 (DDX60   DEAD (Asp−Glu−Ala−Asp) box polypeptide 60) p= 0.01 FC= 1.4
Log2 Intensity
5
6
7
8
10
15
20
25
30
35
40
Gestational age (weeks)

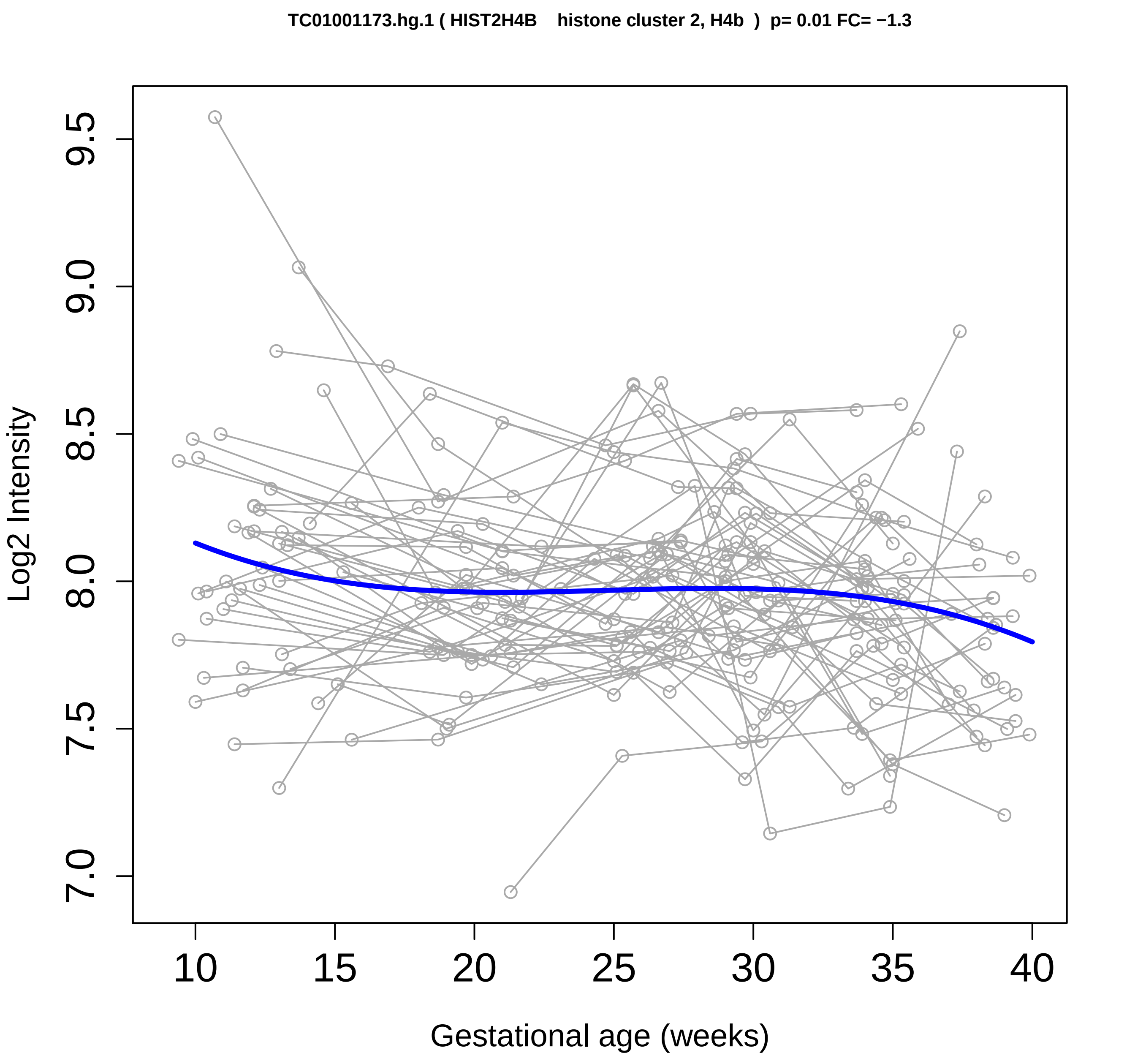
TC01001173.hg.1 (HIST2H4B histone cluster 2, H4b) p= 0.01 FC= −1.3
Log2 Intensity
9.5
9.0
8.5
8.0
7.5
7.0
10
15
20
25
30
35
40
Gestational age (weeks)

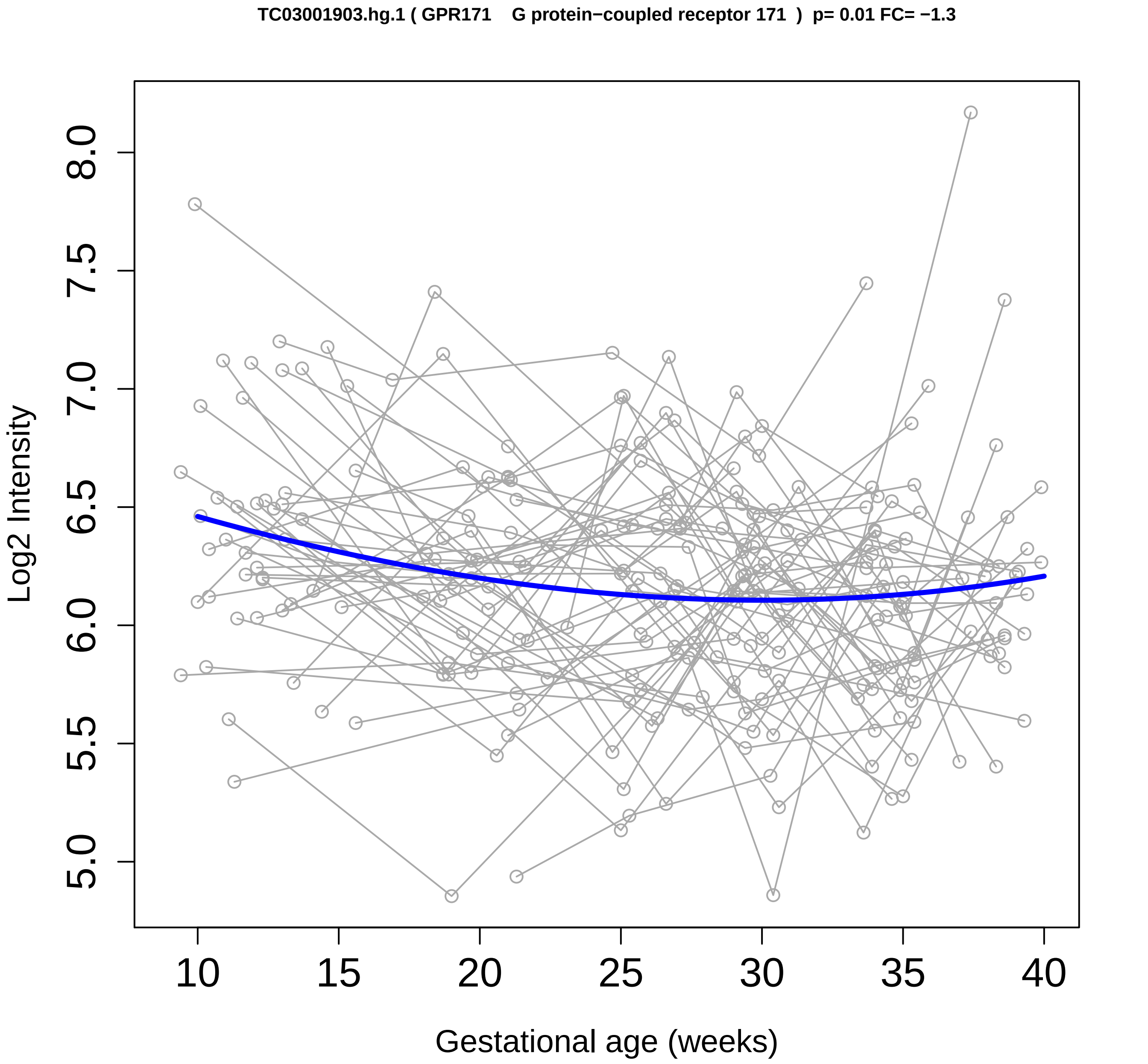

TC03001903.hg.1 (GPR171 G protein−coupled receptor 171) p= 0.01 FC= −1.3
Log2 Intensity
5.0
5.5
6.0
6.5
7.0
7.5
8.0
10
15
20
25
30
35
40
Gestational age (weeks)

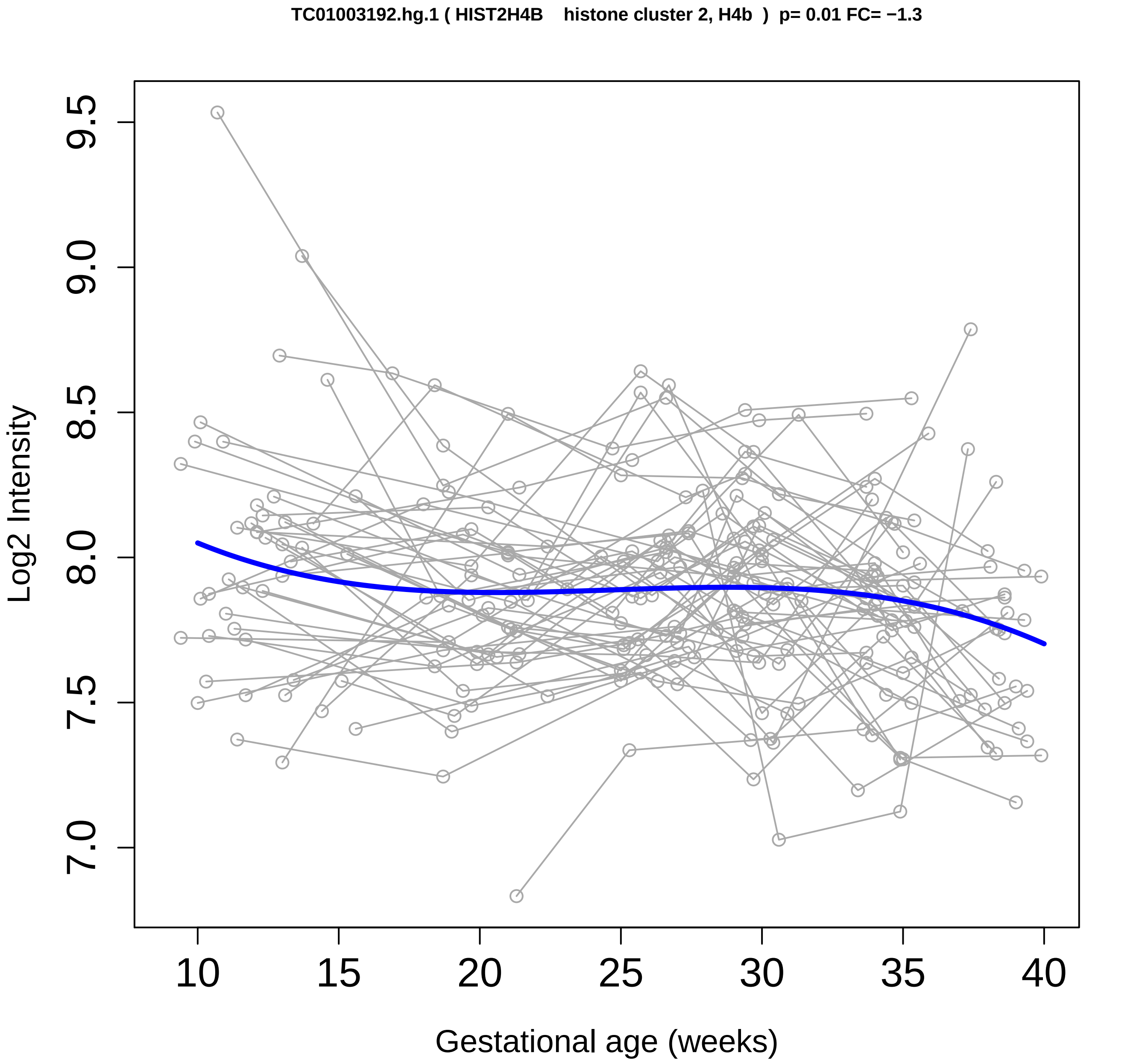

TC01003192.hg.1 (HIST2H4B histone cluster 2, H4b) p= 0.01 FC= −1.3
Log2 Intensity
9.5
9.0
8.5
8.0
7.5
7.0
10
15
20
25
30
35
40
Gestational age (weeks)

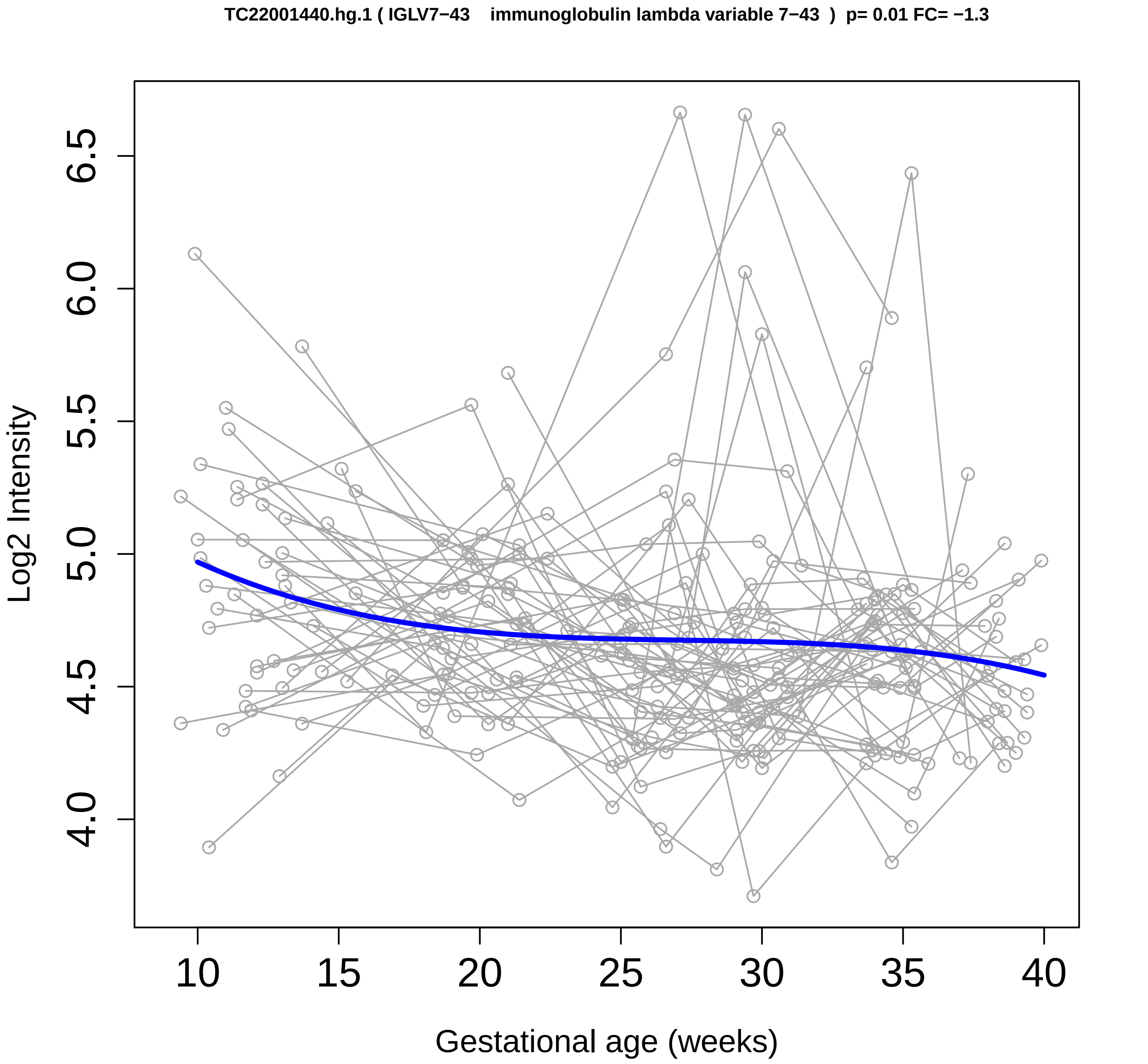
TC22001440.hg.1 (IGLV7−43   immunoglobulin lambda variable 7−43) p= 0.01 FC= −1.3
Log2 Intensity
4.0
4.5
5.0
5.5
6.0
6.5
10
15
20
25
30
35
40
Gestational age (weeks)

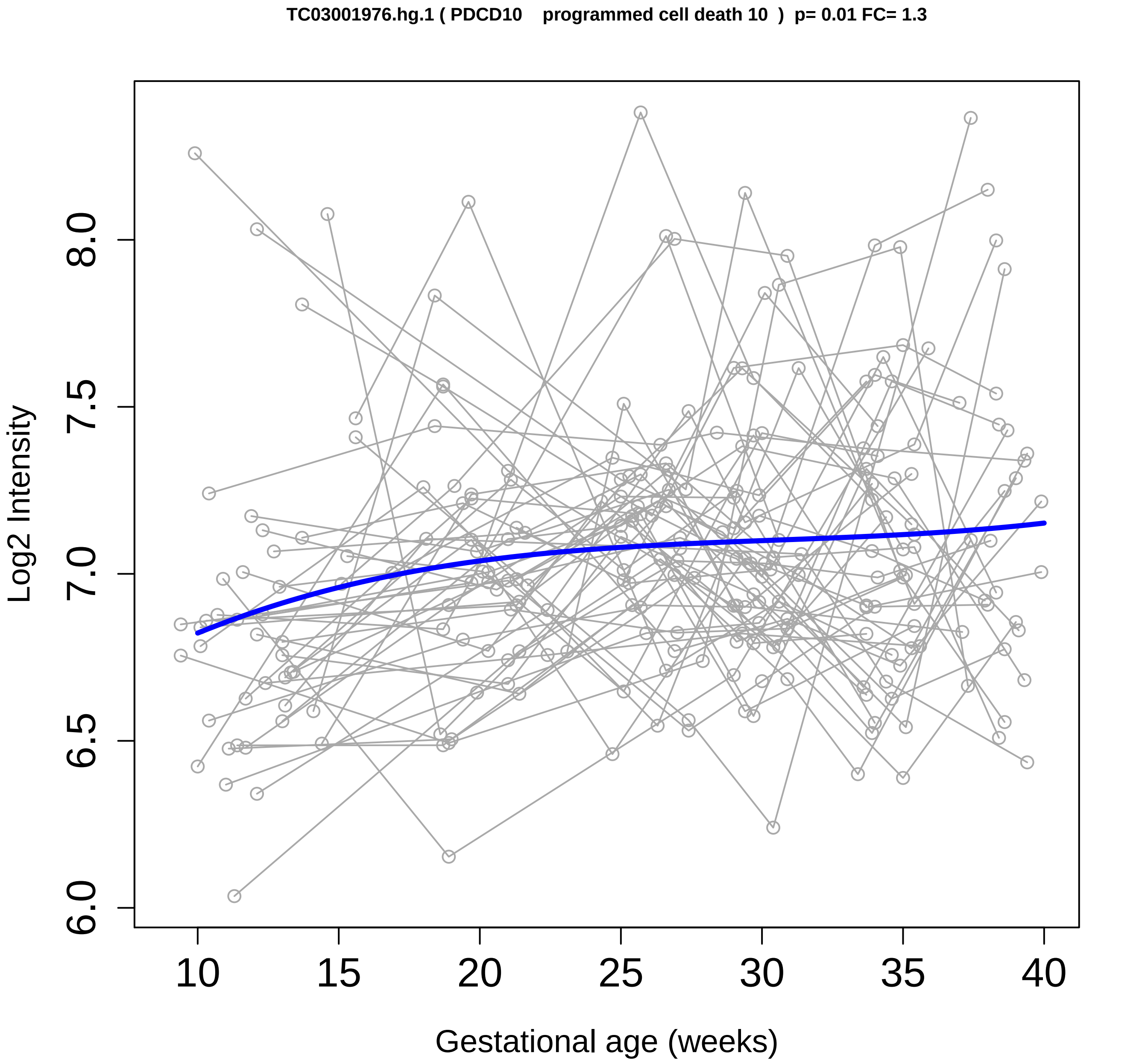

TC03001976.hg.1 (PDCD10 programmed cell death 10) p= 0.01 FC= 1.3
Log2 Intensity
6.0
6.5
7.0
7.5
8.0
10
15
20
25
30
35
40
Gestational age (weeks)

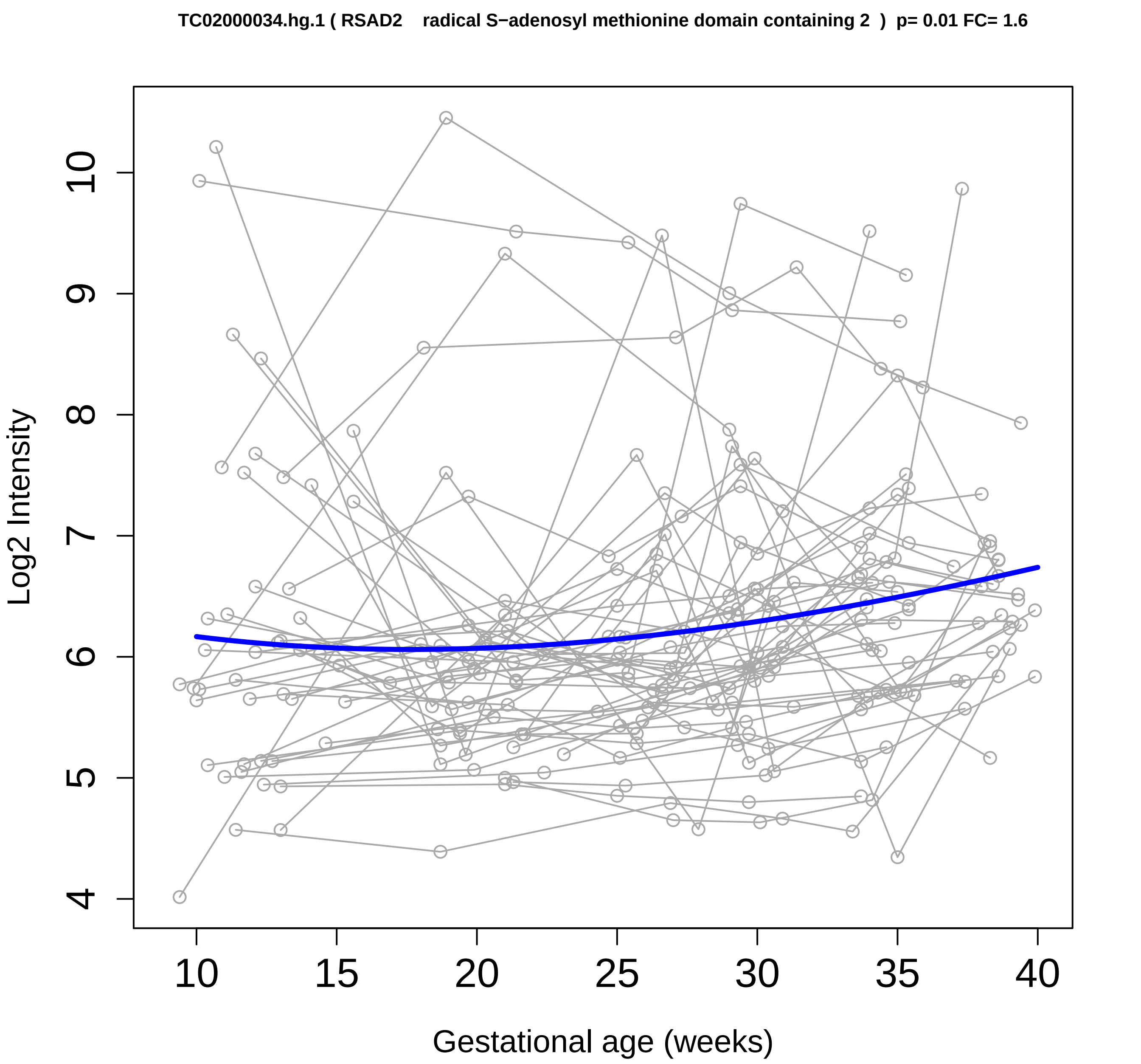
TC02000034.hg.1 (RSAD2 radical S−adenosyl methionine domain containing 2) p= 0.01 FC= 1.6
Log2 Intensity
4
5
6
7
8
9
10
10
15
20
25
30
35
40
Gestational age (weeks)

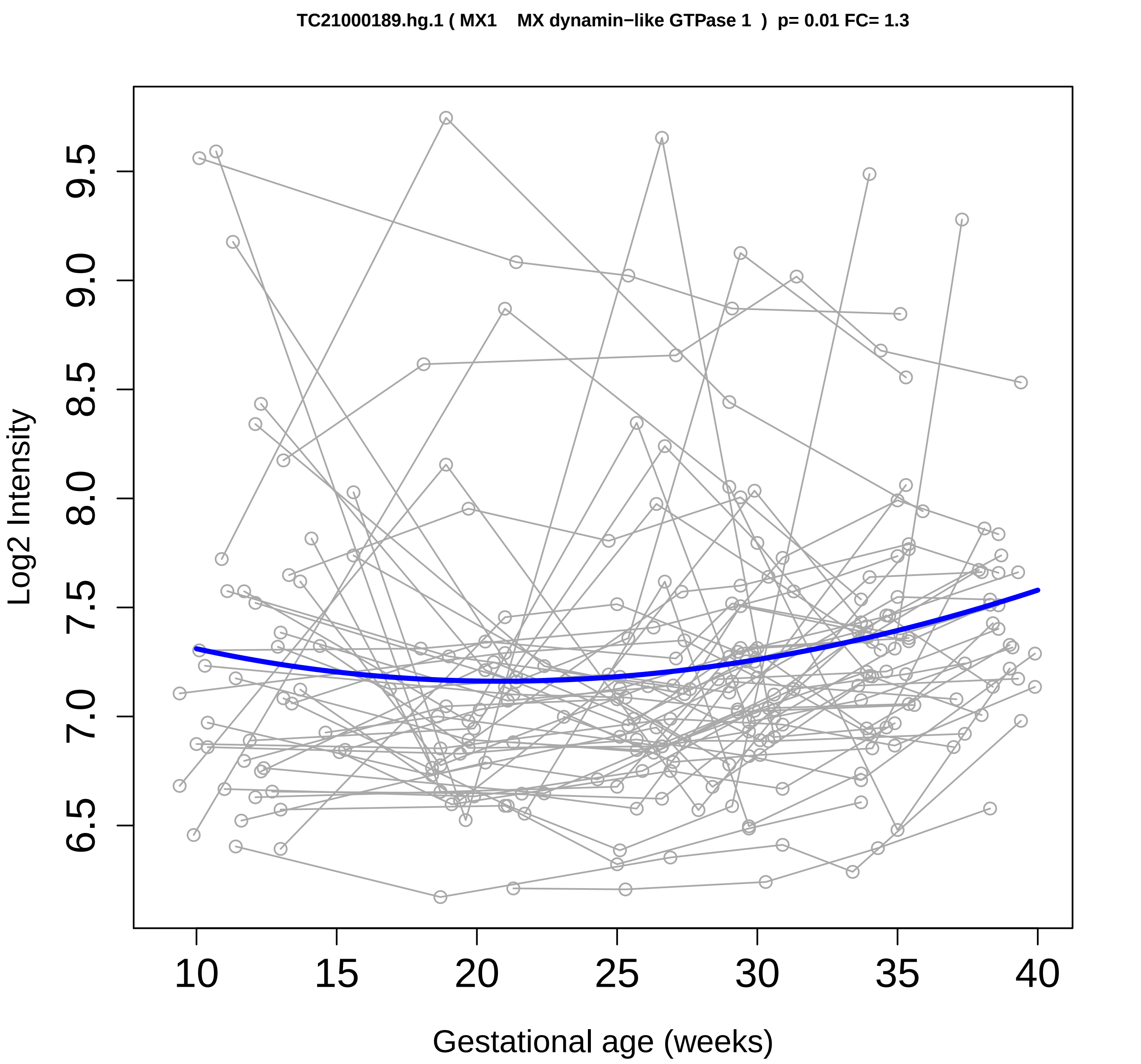

TC21000189.hg.1 (MX1   MX dynamin−like GTPase 1) p= 0.01 FC= 1.3
Log2 Intensity
6.5
7.0
7.5
8.0
8.5
9.0
9.5
10
15
20
25
30
35
40
Gestational age (weeks)

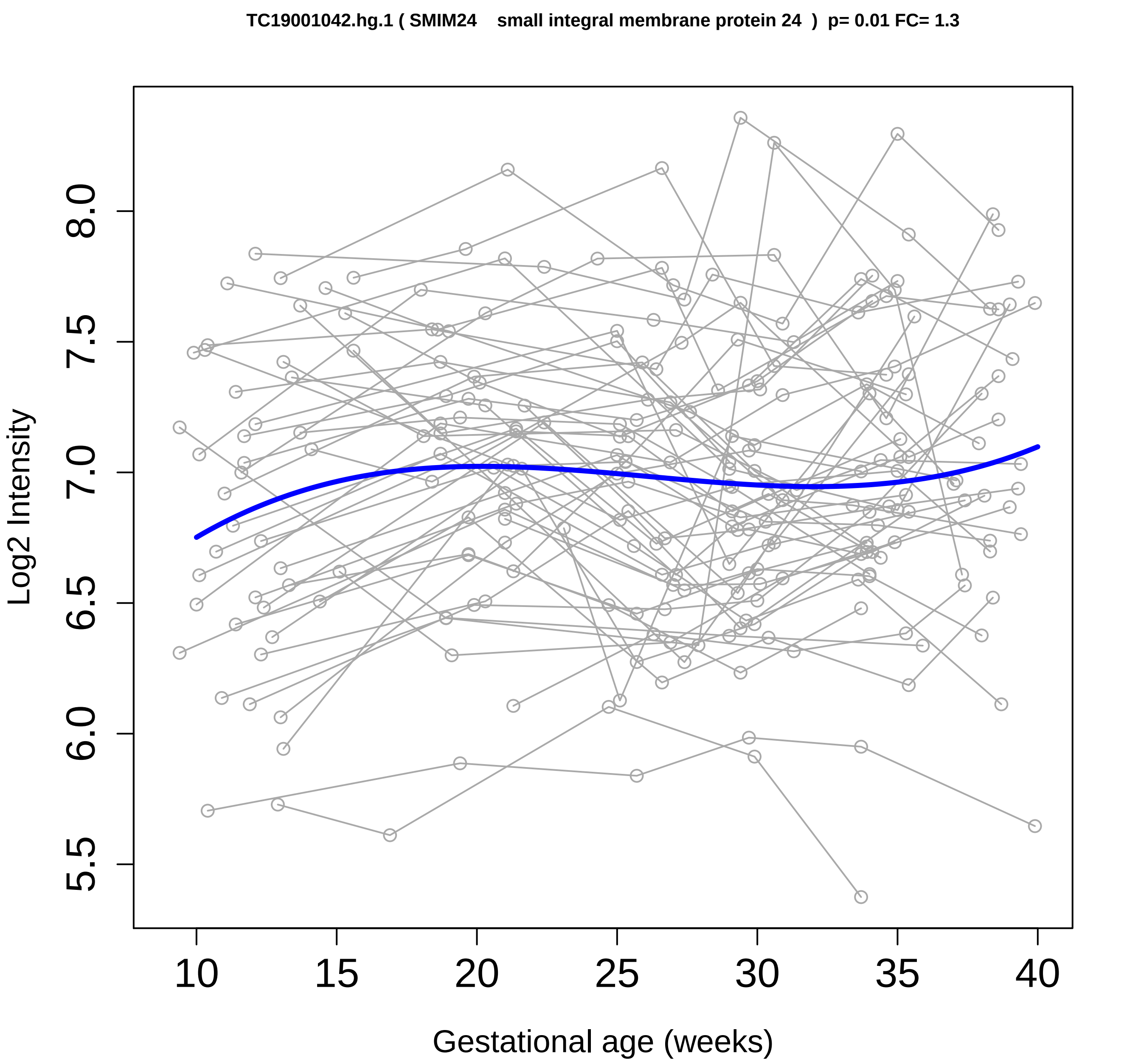
TC19001042.hg.1 (SMIM24   small integral membrane protein 24) p= 0.01 FC= 1.3
Log2 Intensity
5.5
6.0
6.5
7.0
7.5
8.0
10
15
20
25
30
35
40
Gestational age (weeks)

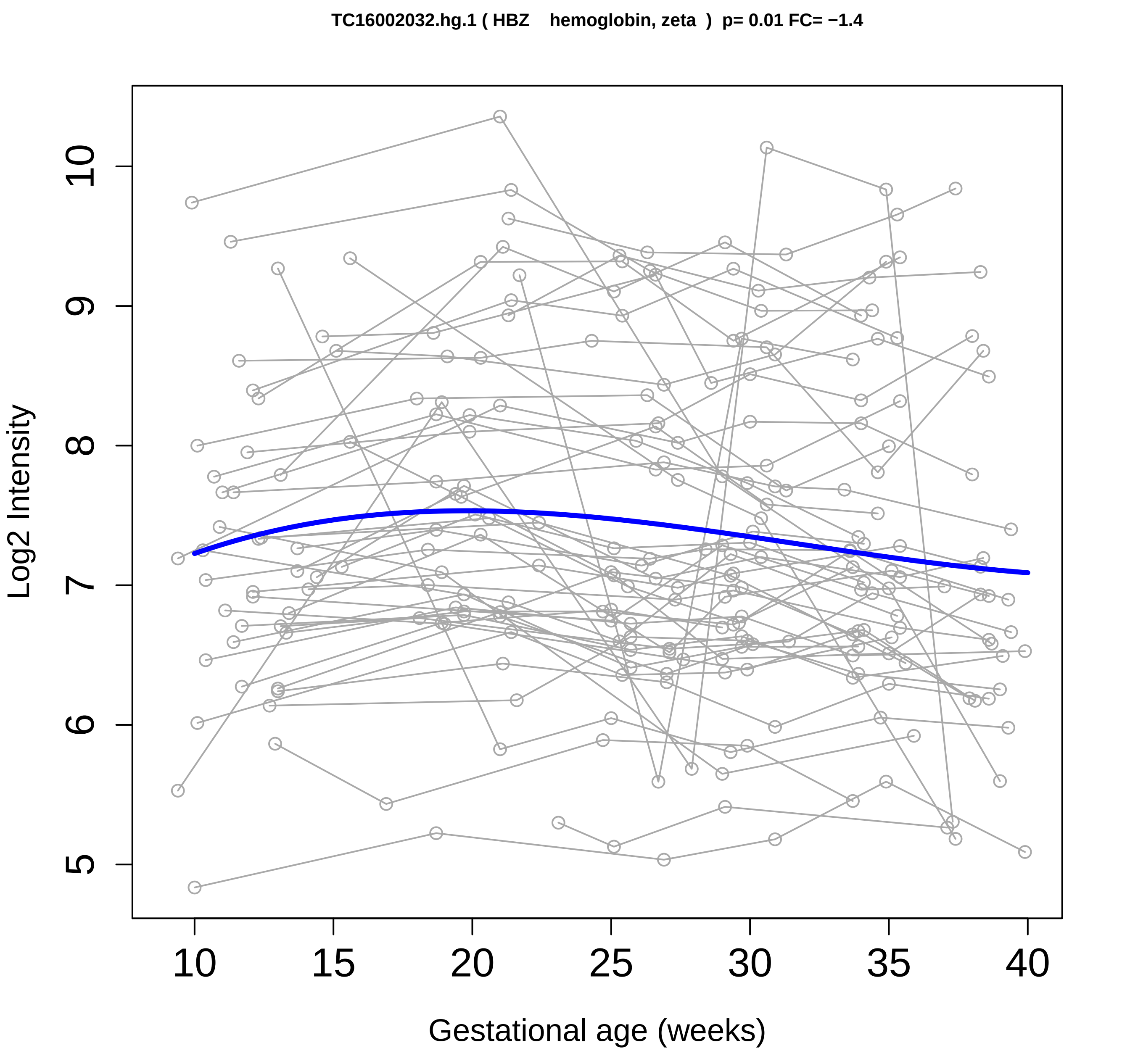

TC16002032.hg.1 (HBZ hemoglobin, zeta) p= 0.01 FC= −1.4
Log2 Intensity
Gestational age (weeks)
5
6
7
8
9
10
10
15
20
25
30
35
40

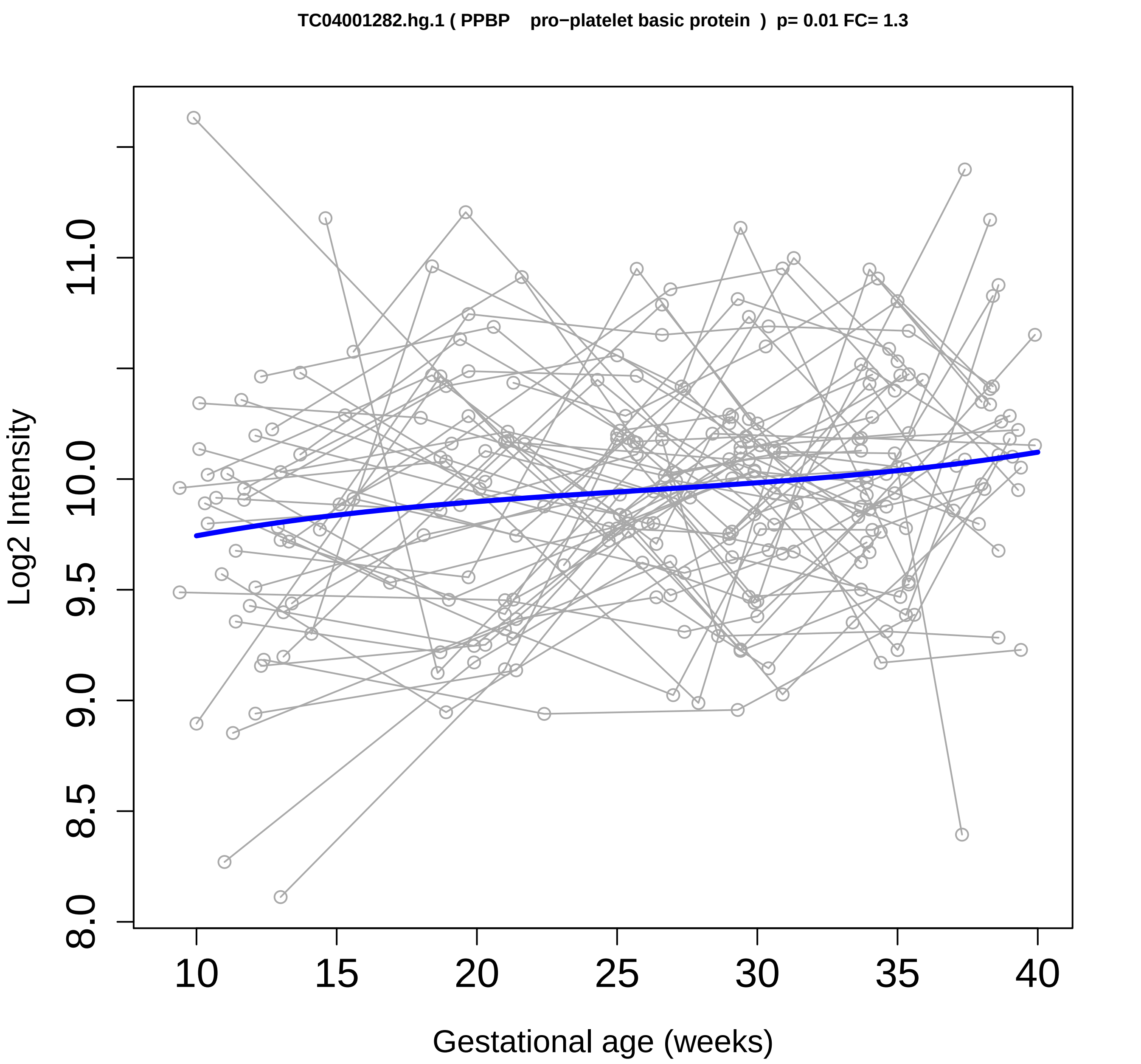
TC04001282.hg.1 (PPBP pro−platelet basic protein) p= 0.01 FC= 1.3
Log2 Intensity
8.0
8.5
9.0
9.5
10.0
11.0
10
15
20
25
30
35
40
Gestational age (weeks)

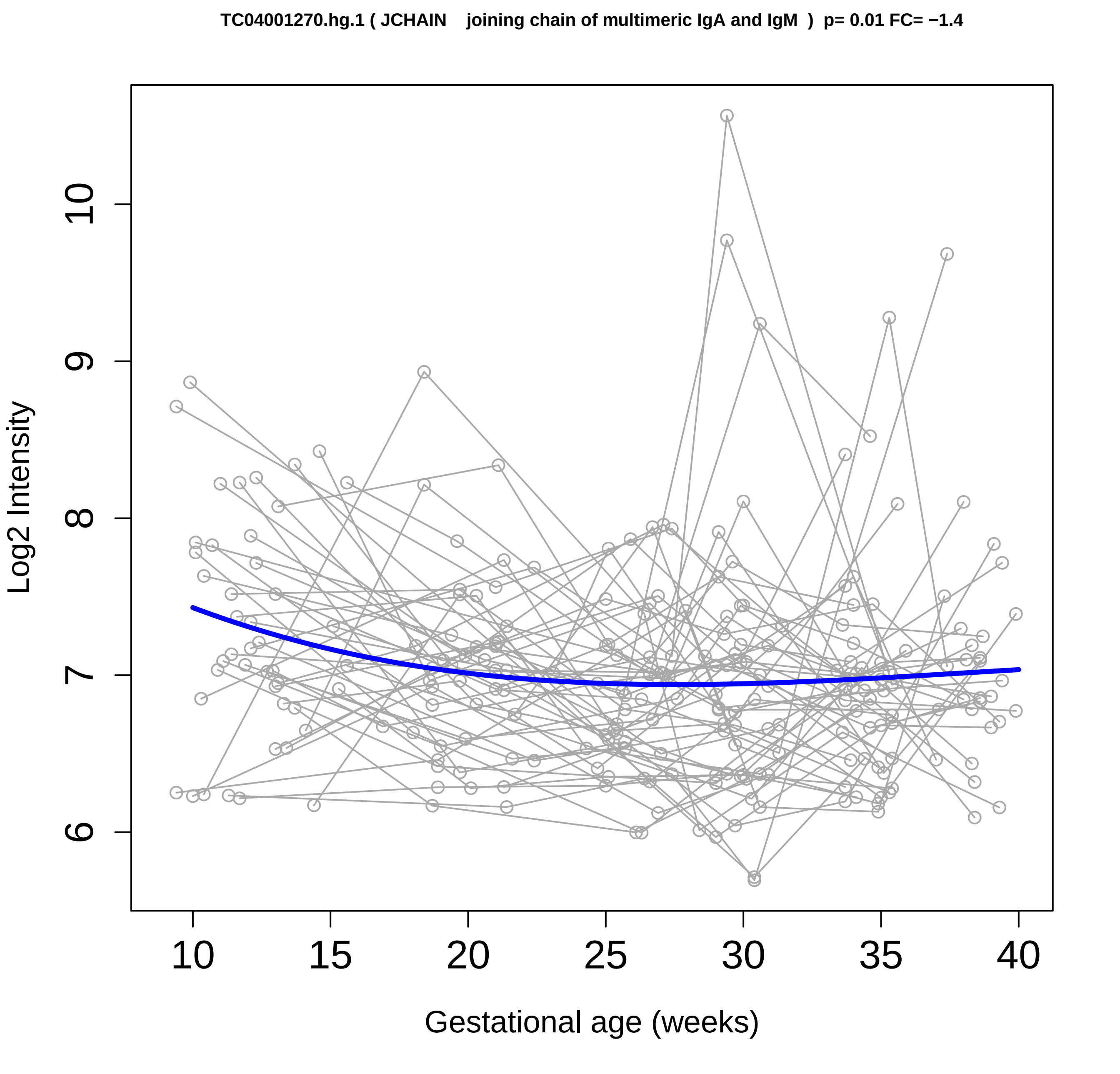
TC04001270.hg.1 (JCHAIN joining chain of multimeric IgA and IgM) p= 0.01 FC= −1.4
Log2 Intensity
6
7
8
9
10
10
15
20
25
30
35
40
Gestational age (weeks)

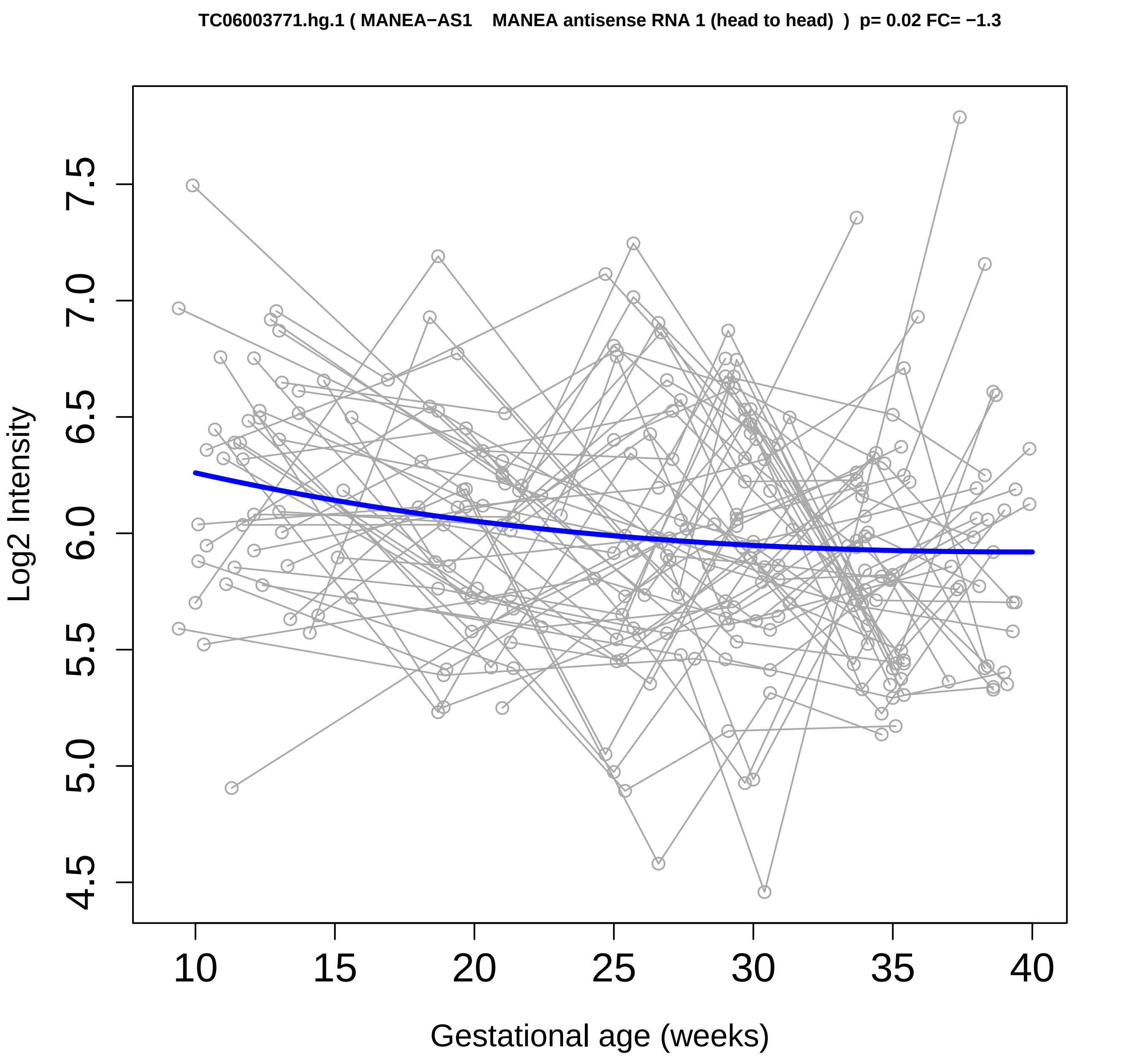
TC06003771.hg.1 (MANEA−AS1   MANEA antisense RNA 1 (head to head)) p= 0.02 FC= −1.3
Log2 Intensity
4.5
5.0
5.5
6.0
6.5
7.0
7.5
10
15
20
25
30
35
40
Gestational age (weeks)

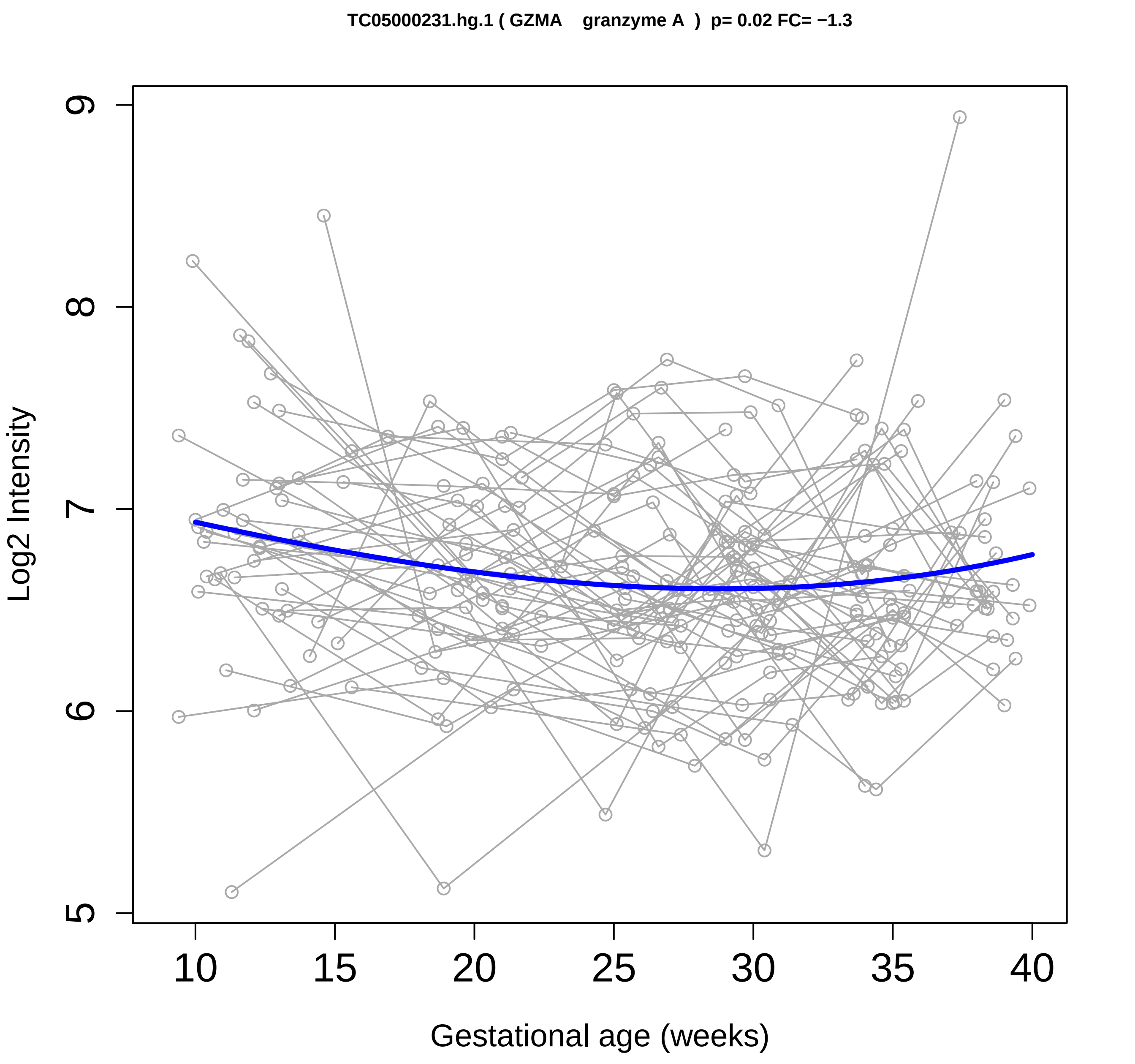

TC05000231.hg.1 (GZMA granzyme A) p= 0.02 FC= −1.3
Log2 Intensity
5
6
7
8
9
10
15
20
25
30
35
40
Gestational age (weeks)

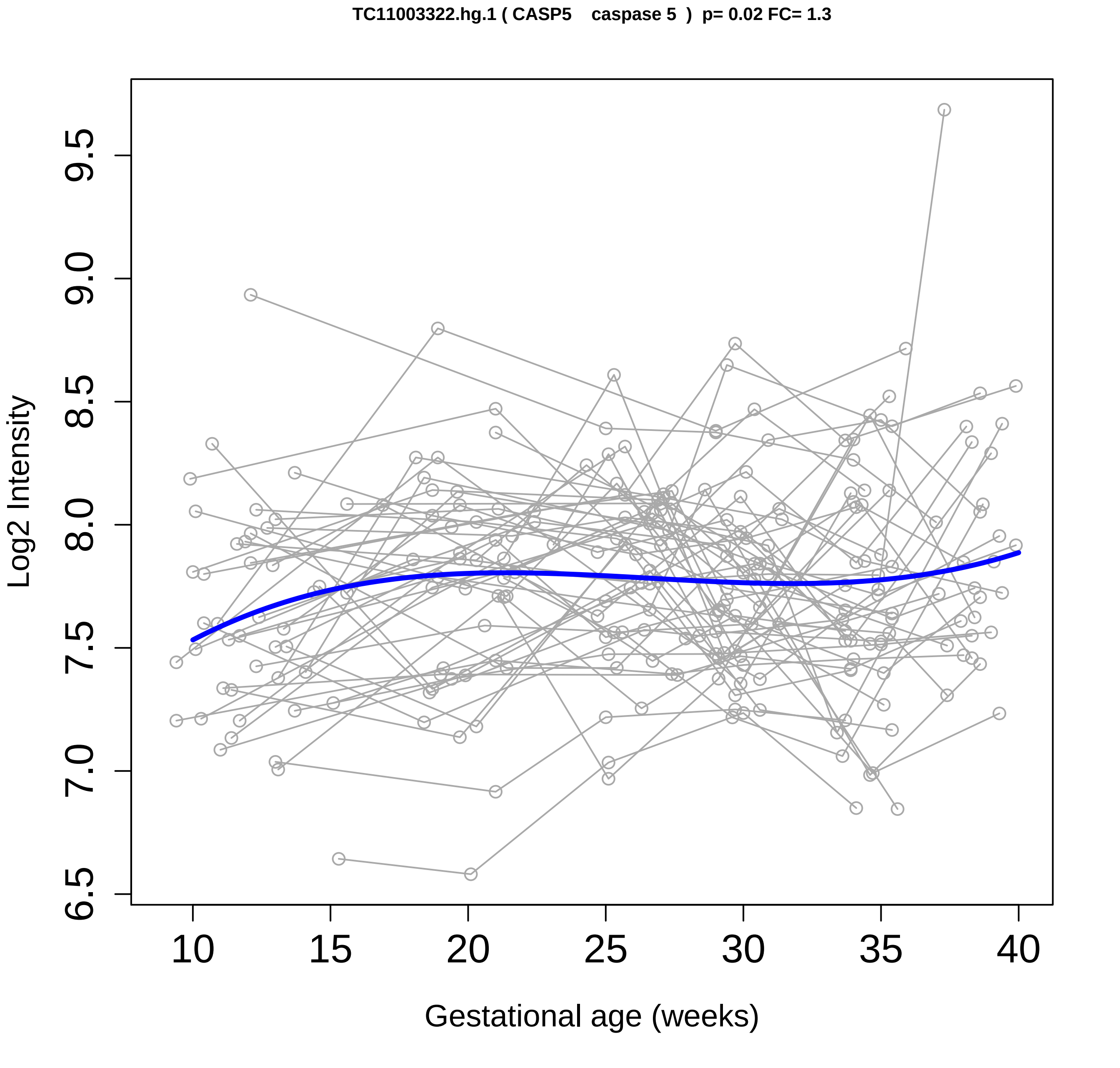
TC11003322.hg.1 (CASP5 caspase 5) p= 0.02 FC= 1.3
Log2 Intensity
6.5
7.0
7.5
8.0
8.5
9.0
9.5
10
15
20
25
30
35
40
Gestational age (weeks)

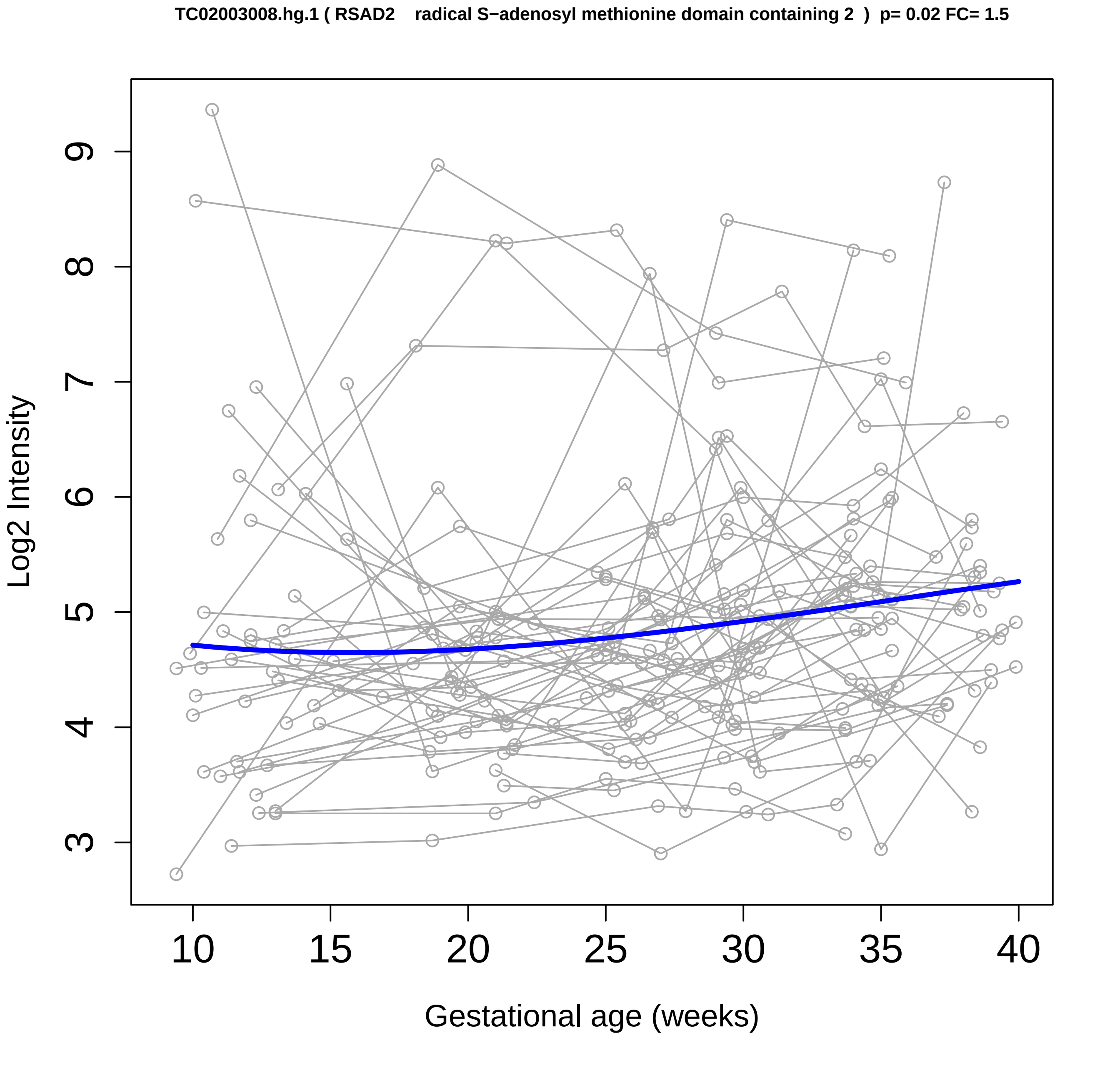
TC02003008.hg.1 (RSAD2 radical S−adenosyl methionine domain containing 2) p= 0.02 FC= 1.5
Log2 Intensity
3
4
5
6
7
8
9
10
15
20
25
30
35
40
Gestational age (weeks)

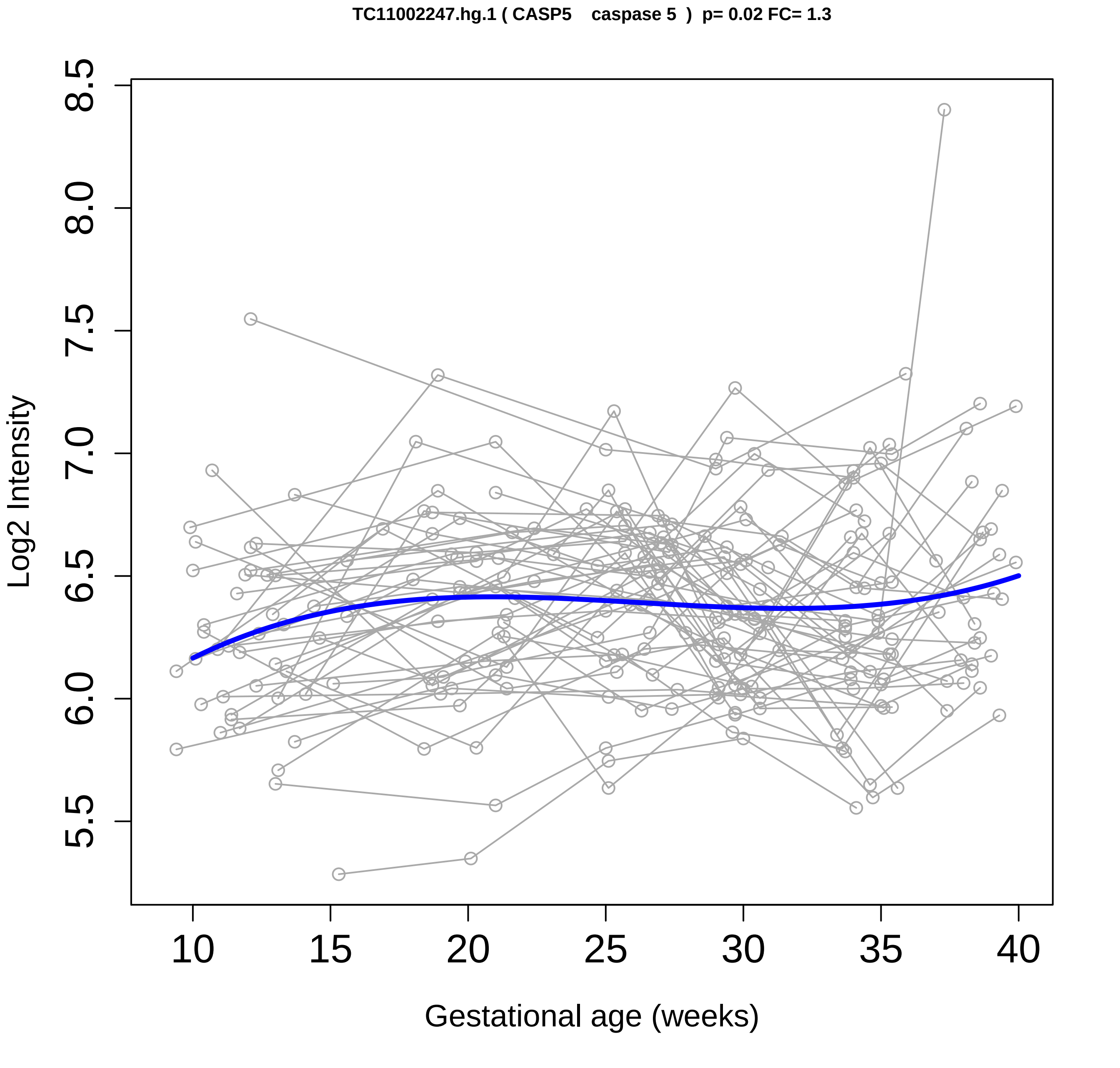
TC11002247.hg.1 (CASP5 caspase 5) p= 0.02 FC= 1.3
Log2 Intensity
8.5
8.0
7.5
7.0
6.5
6.0
5.5
10
15
20
25
30
35
40
Gestational age (weeks)

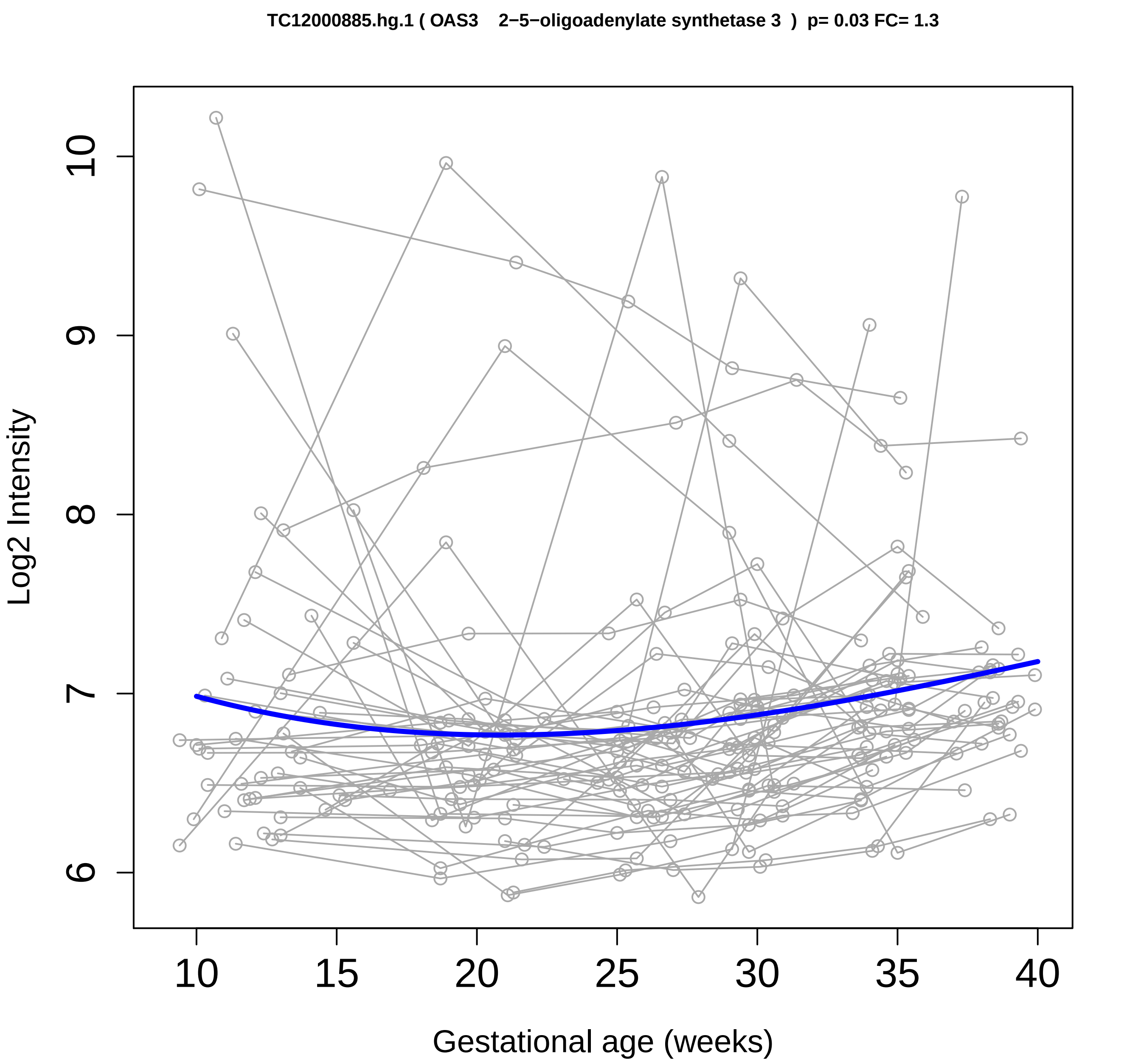

TC12000885.hg.1 (OAS3   2−5−oligoadenylate synthetase 3) p= 0.03 FC= 1.3
Log2 Intensity
6
7
8
9
10
10
15
20
25
30
35
40
Gestational age (weeks)
